# Supplementary figures and images for: Glycine decarboxylase advances IgA nephropathy by boosting mesangial cell proliferation through the pyrimidine pathway (part 2 of 7)
Source: EMBO Mol Med. 2025 Oct 13;17(11):3039–63. doi: 10.1038/s44321-025-00315-2 (PMC12603144; doi:10.1038/s44321-025-00315-2)

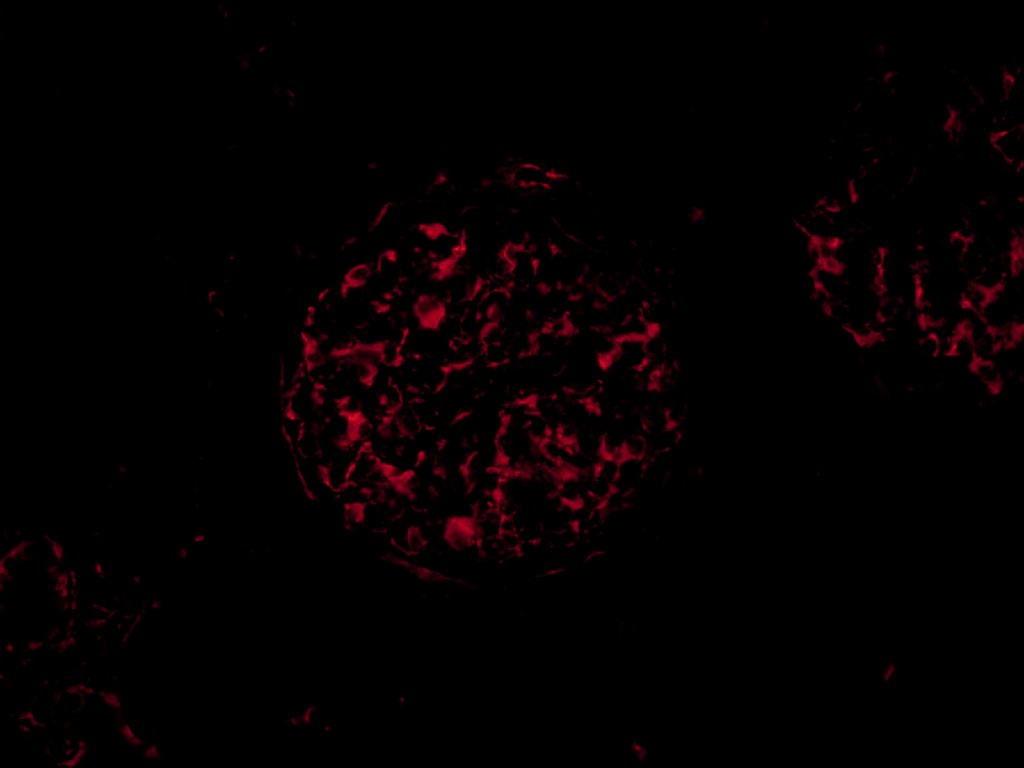

Supplement: Supplementary file 2 — Source data Fig. 1 [file 44321_2025_315_MOESM2_ESM.zip › Figure 1/F1A/1-GLDC-PDGFRbeta/Lee III/9 (2).jpg]

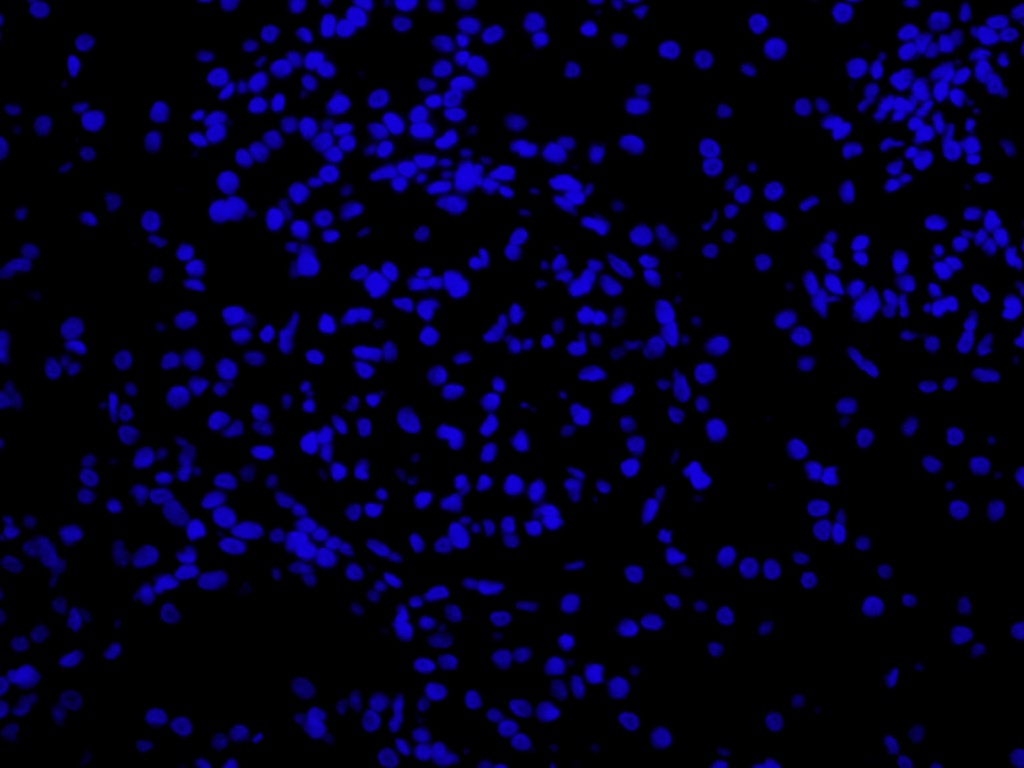

Supplement: Supplementary file 2 — Source data Fig. 1 [file 44321_2025_315_MOESM2_ESM.zip › Figure 1/F1A/1-GLDC-PDGFRbeta/Lee III/9 (3).jpg]

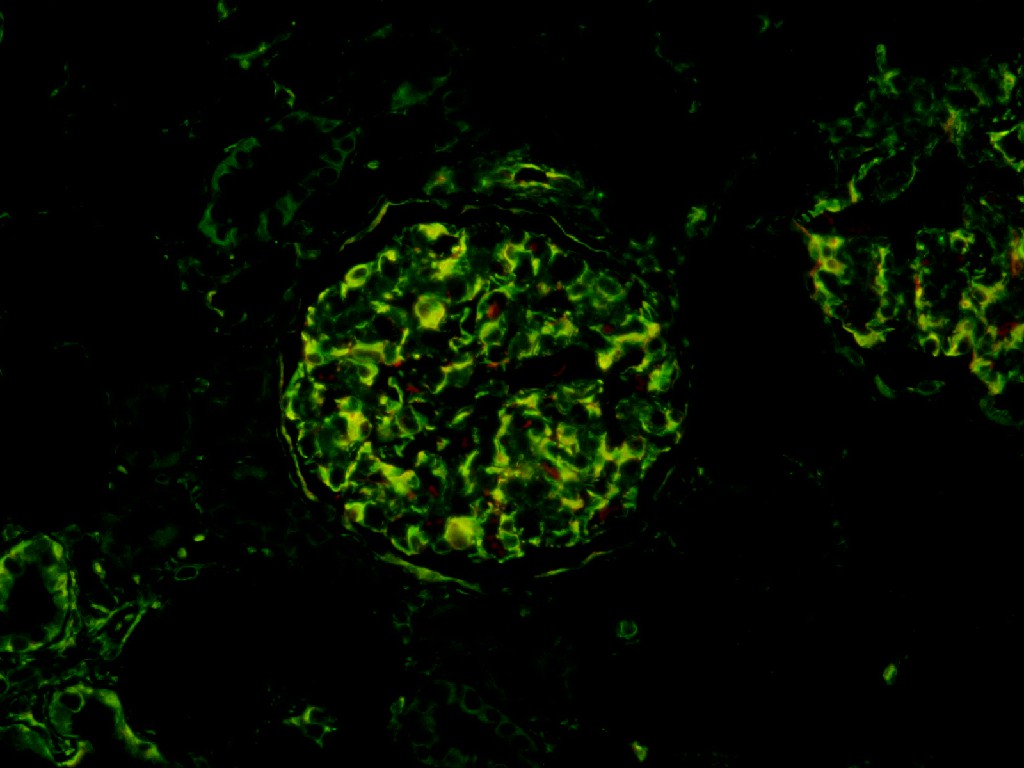

Supplement: Supplementary file 2 — Source data Fig. 1 [file 44321_2025_315_MOESM2_ESM.zip › Figure 1/F1A/1-GLDC-PDGFRbeta/Lee III/9 (4).jpg]

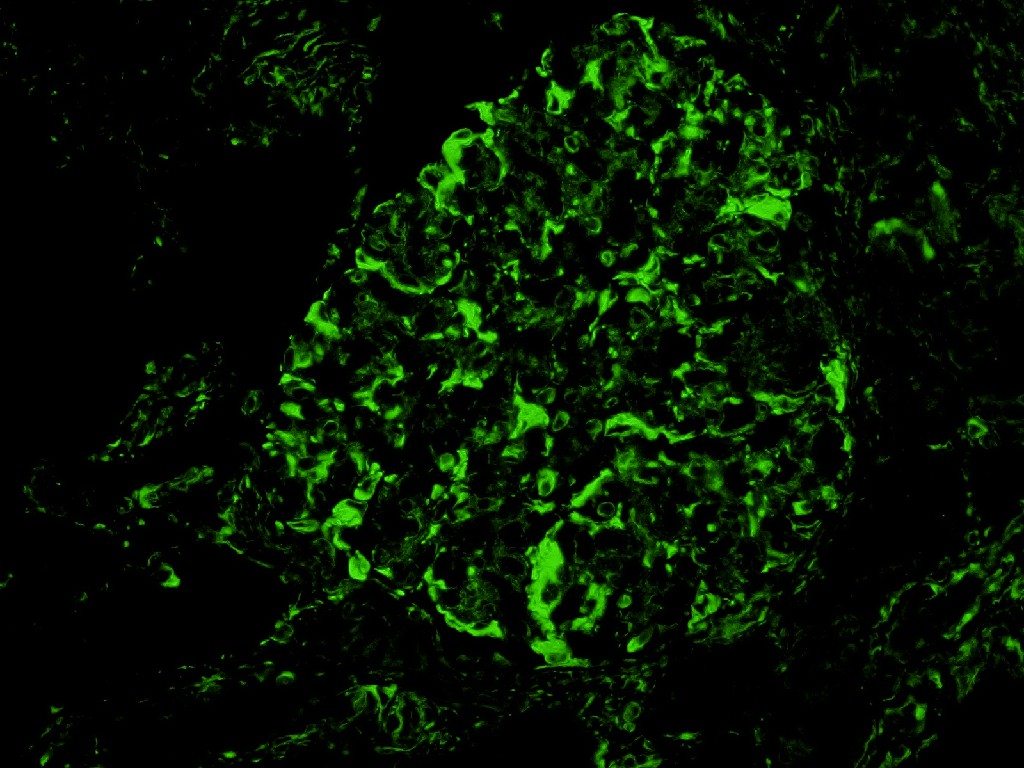

Supplement: Supplementary file 2 — Source data Fig. 1 [file 44321_2025_315_MOESM2_ESM.zip › Figure 1/F1A/1-GLDC-PDGFRbeta/Lee IV/1 (1).jpg]

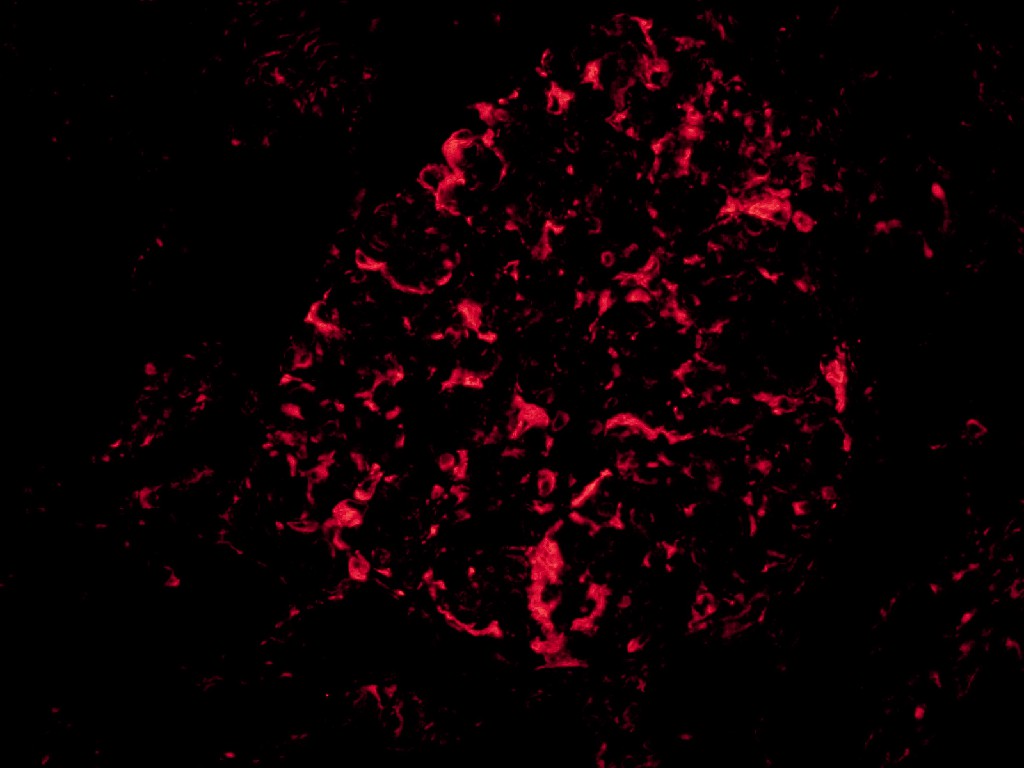

Supplement: Supplementary file 2 — Source data Fig. 1 [file 44321_2025_315_MOESM2_ESM.zip › Figure 1/F1A/1-GLDC-PDGFRbeta/Lee IV/1 (2).jpg]

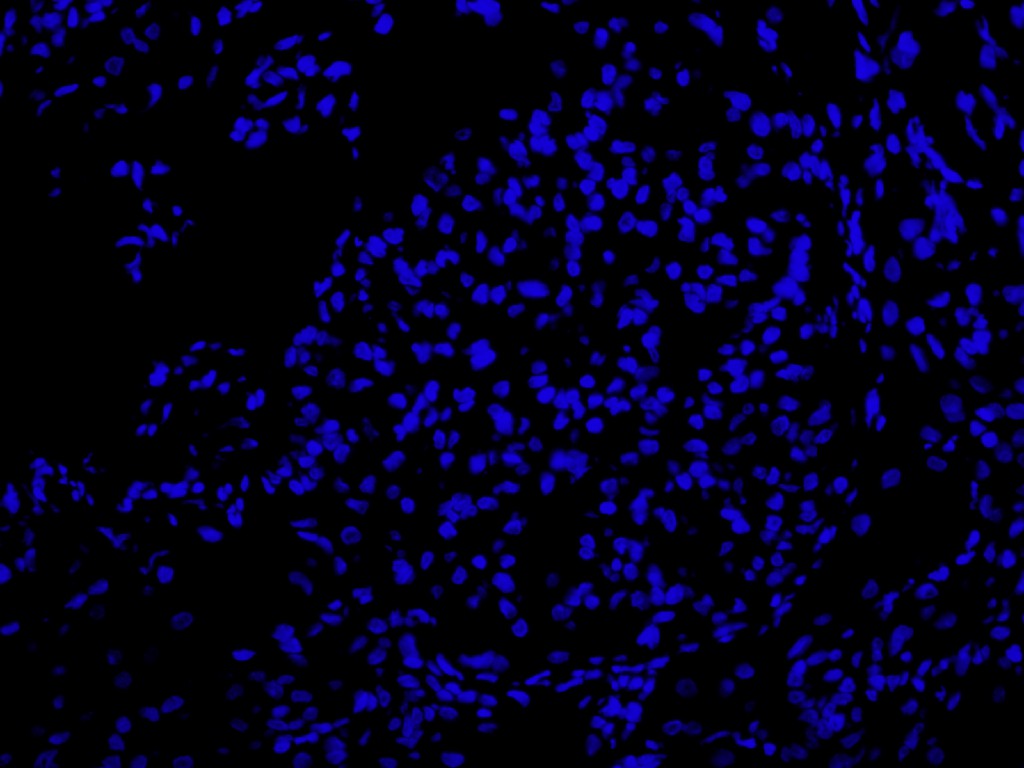

Supplement: Supplementary file 2 — Source data Fig. 1 [file 44321_2025_315_MOESM2_ESM.zip › Figure 1/F1A/1-GLDC-PDGFRbeta/Lee IV/1 (3).jpg]

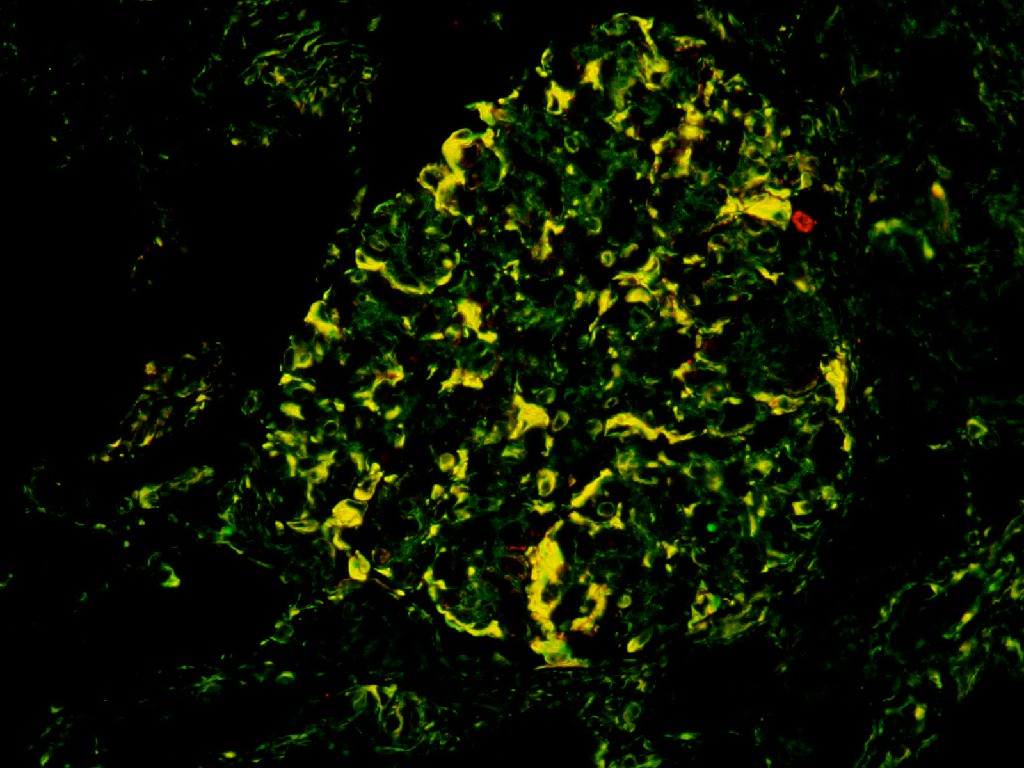

Supplement: Supplementary file 2 — Source data Fig. 1 [file 44321_2025_315_MOESM2_ESM.zip › Figure 1/F1A/1-GLDC-PDGFRbeta/Lee IV/1 (4).jpg]

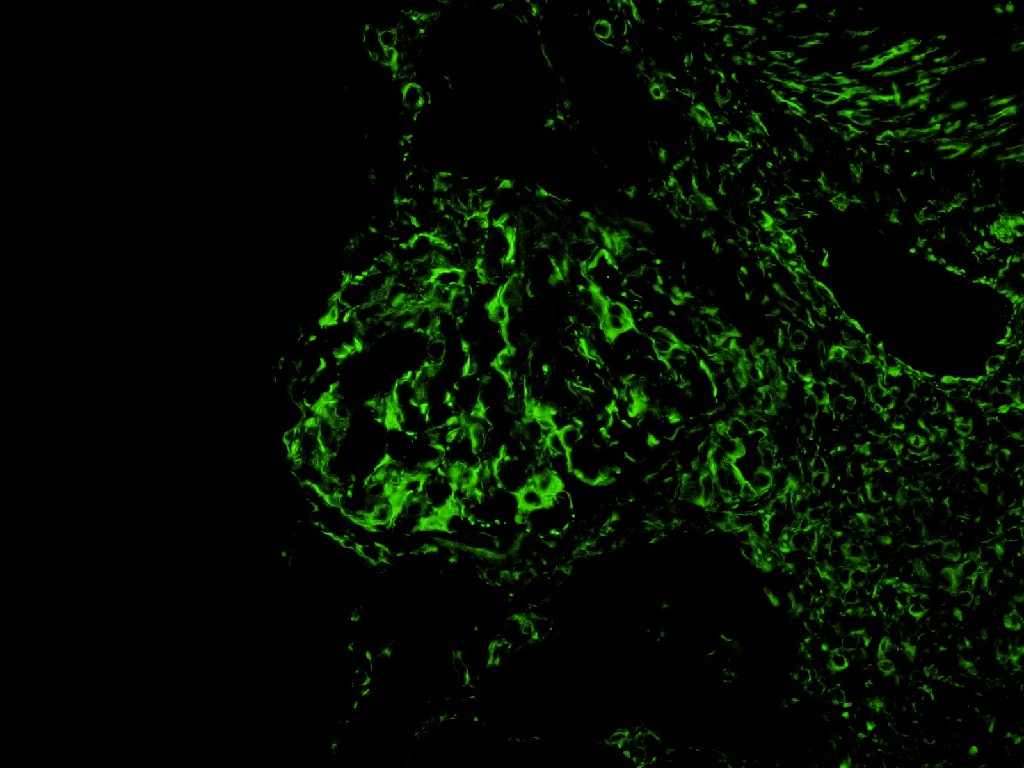

Supplement: Supplementary file 2 — Source data Fig. 1 [file 44321_2025_315_MOESM2_ESM.zip › Figure 1/F1A/1-GLDC-PDGFRbeta/Lee IV/10 (1).jpg]

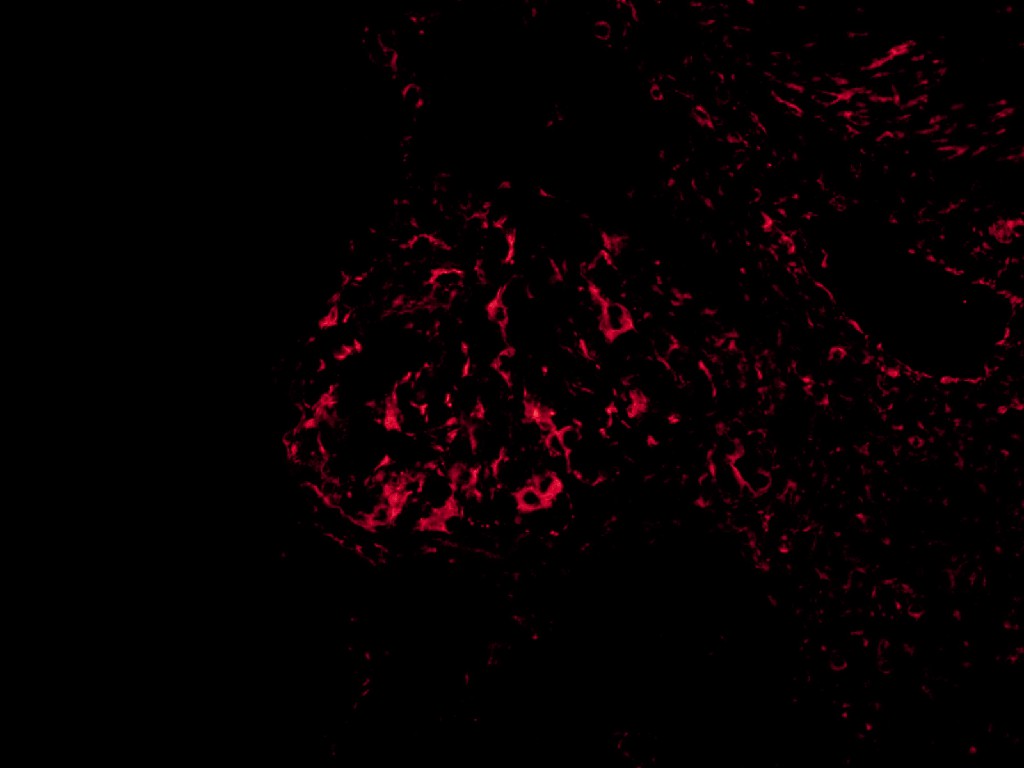

Supplement: Supplementary file 2 — Source data Fig. 1 [file 44321_2025_315_MOESM2_ESM.zip › Figure 1/F1A/1-GLDC-PDGFRbeta/Lee IV/10 (2).jpg]

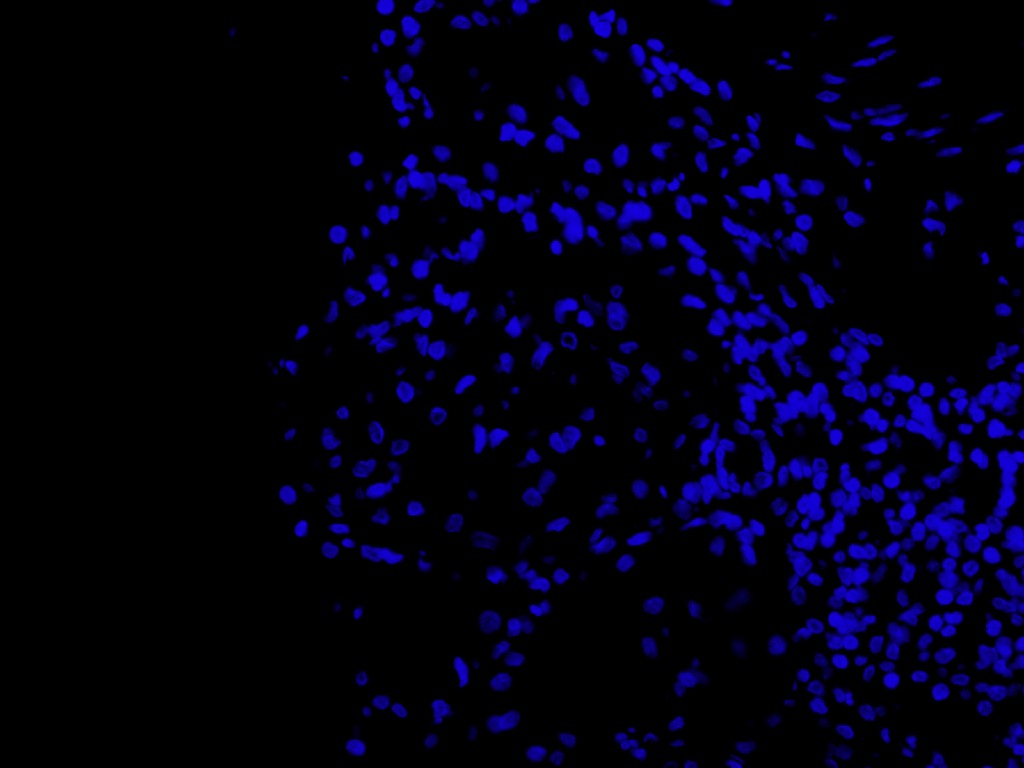

Supplement: Supplementary file 2 — Source data Fig. 1 [file 44321_2025_315_MOESM2_ESM.zip › Figure 1/F1A/1-GLDC-PDGFRbeta/Lee IV/10 (3).jpg]

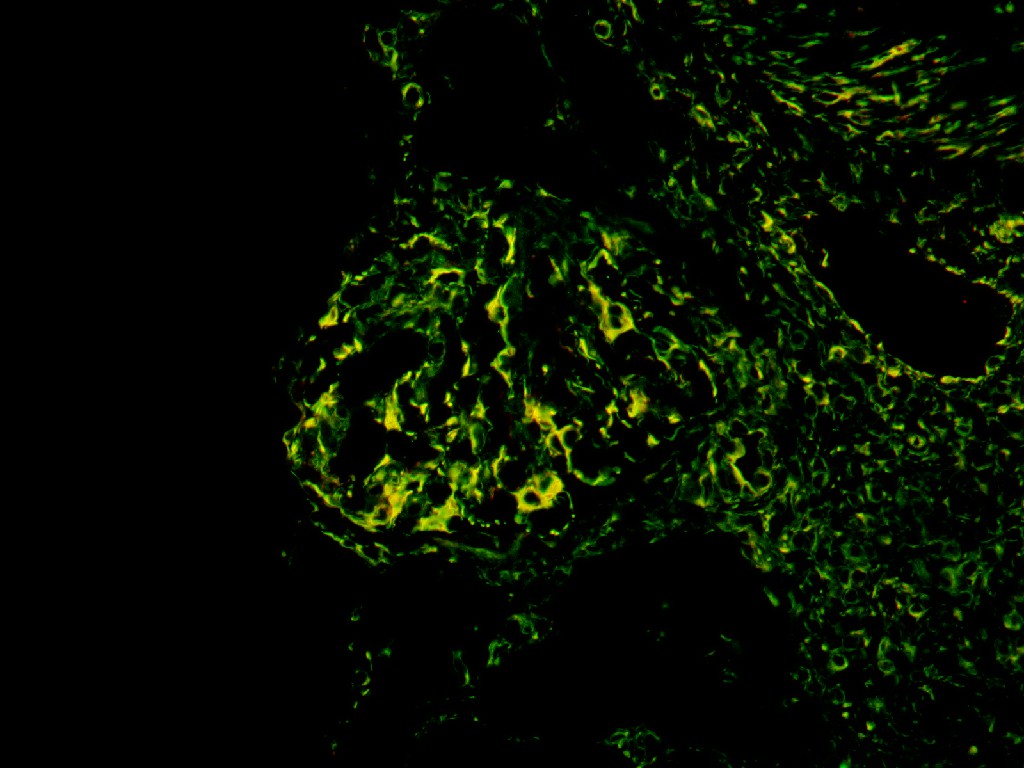

Supplement: Supplementary file 2 — Source data Fig. 1 [file 44321_2025_315_MOESM2_ESM.zip › Figure 1/F1A/1-GLDC-PDGFRbeta/Lee IV/10 (4).jpg]

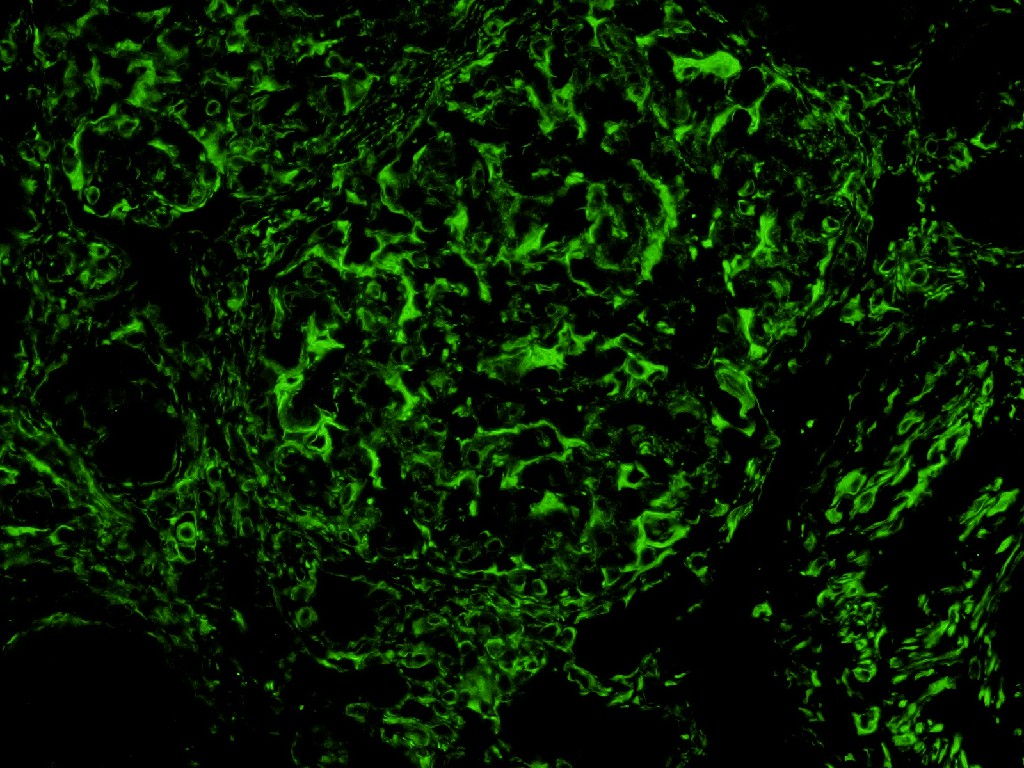

Supplement: Supplementary file 2 — Source data Fig. 1 [file 44321_2025_315_MOESM2_ESM.zip › Figure 1/F1A/1-GLDC-PDGFRbeta/Lee IV/11 (1).jpg]

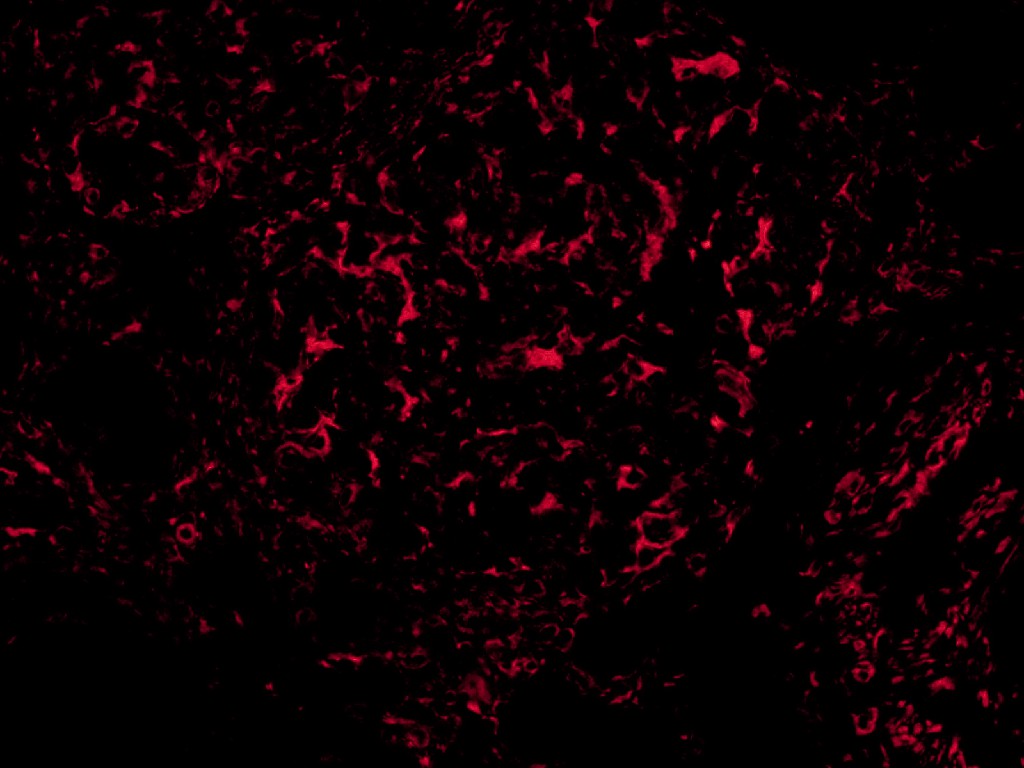

Supplement: Supplementary file 2 — Source data Fig. 1 [file 44321_2025_315_MOESM2_ESM.zip › Figure 1/F1A/1-GLDC-PDGFRbeta/Lee IV/11 (2).jpg]

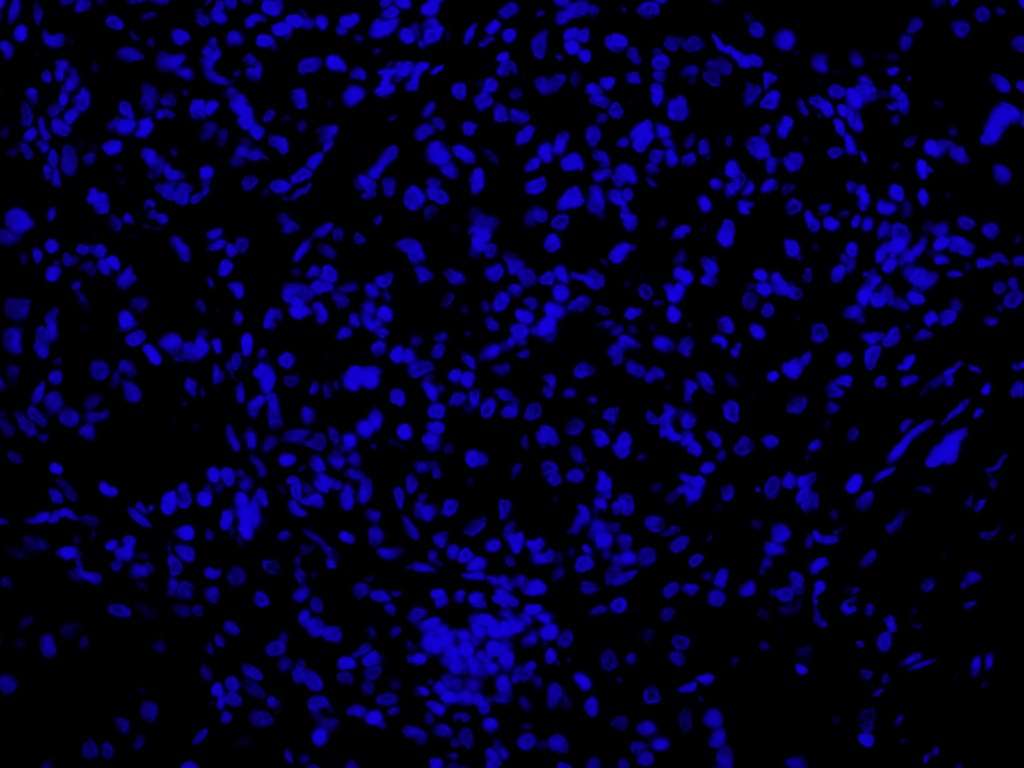

Supplement: Supplementary file 2 — Source data Fig. 1 [file 44321_2025_315_MOESM2_ESM.zip › Figure 1/F1A/1-GLDC-PDGFRbeta/Lee IV/11 (3).jpg]

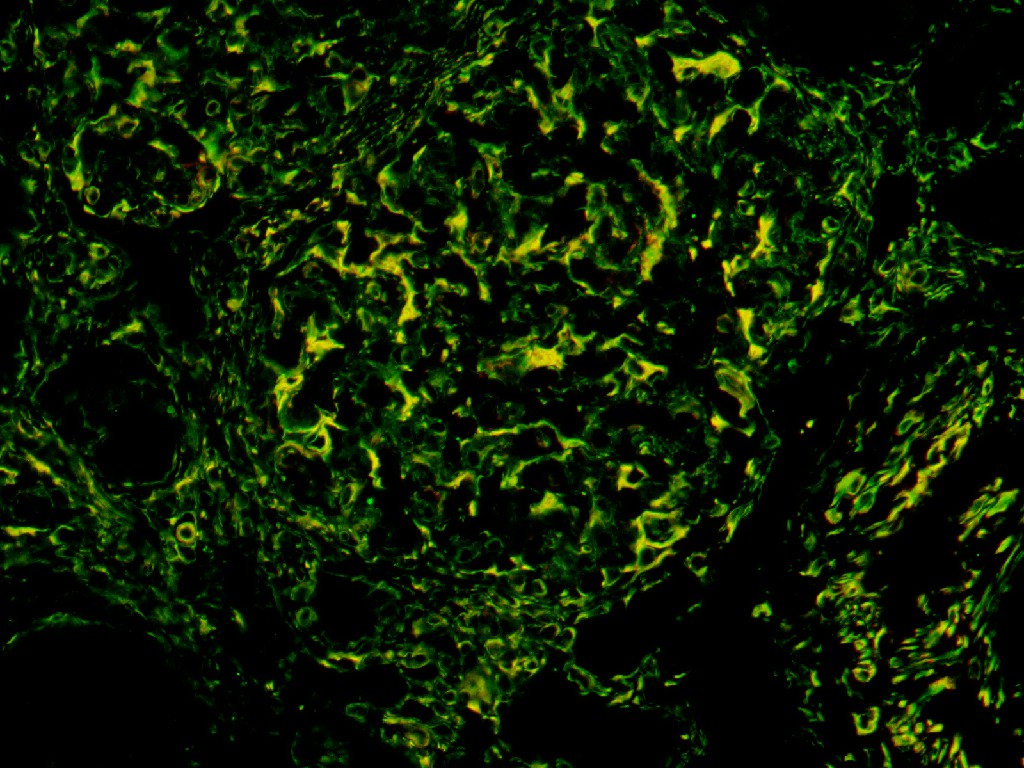

Supplement: Supplementary file 2 — Source data Fig. 1 [file 44321_2025_315_MOESM2_ESM.zip › Figure 1/F1A/1-GLDC-PDGFRbeta/Lee IV/11 (4).jpg]

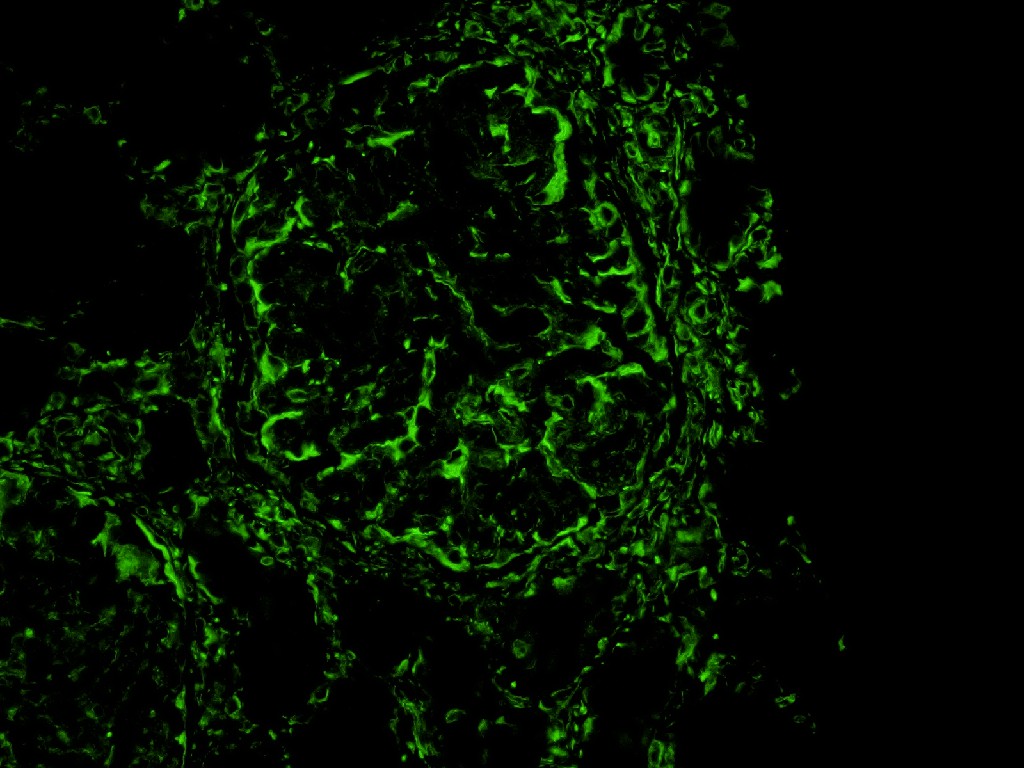

Supplement: Supplementary file 2 — Source data Fig. 1 [file 44321_2025_315_MOESM2_ESM.zip › Figure 1/F1A/1-GLDC-PDGFRbeta/Lee IV/12 (1).jpg]

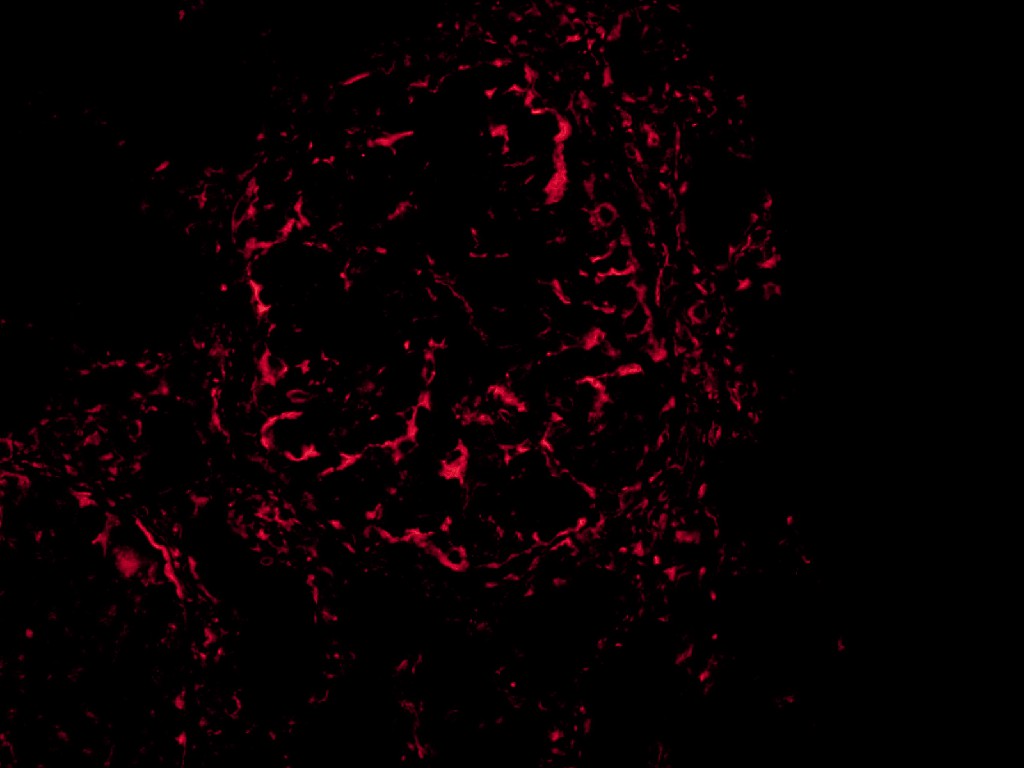

Supplement: Supplementary file 2 — Source data Fig. 1 [file 44321_2025_315_MOESM2_ESM.zip › Figure 1/F1A/1-GLDC-PDGFRbeta/Lee IV/12 (2).jpg]

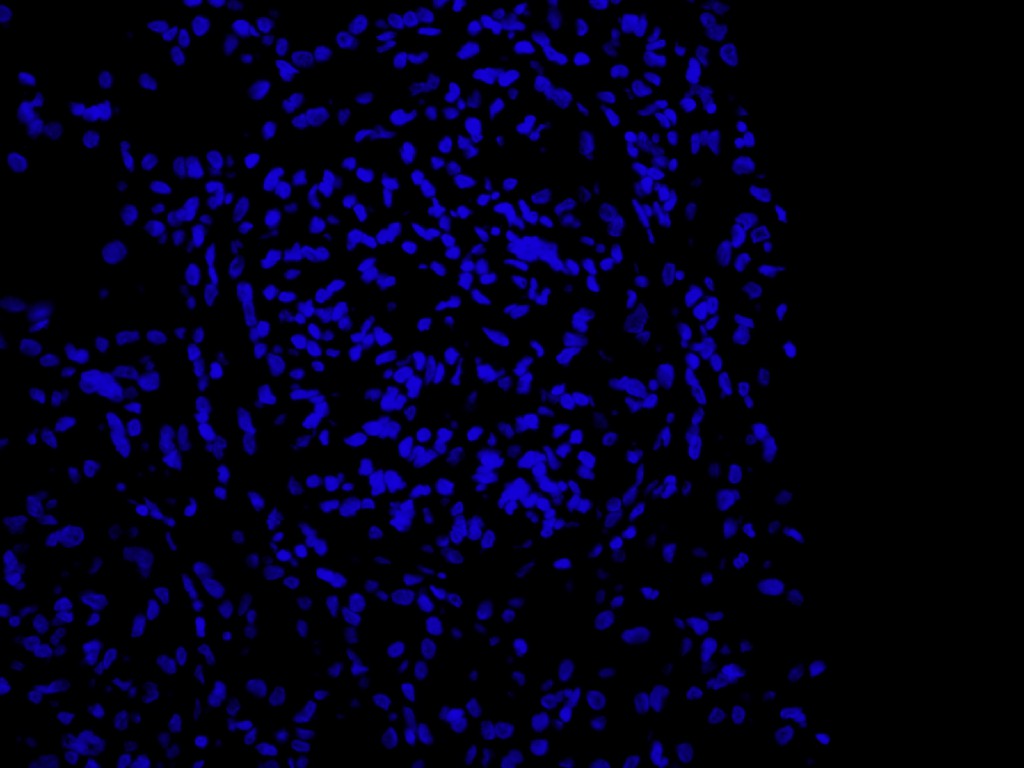

Supplement: Supplementary file 2 — Source data Fig. 1 [file 44321_2025_315_MOESM2_ESM.zip › Figure 1/F1A/1-GLDC-PDGFRbeta/Lee IV/12 (3).jpg]

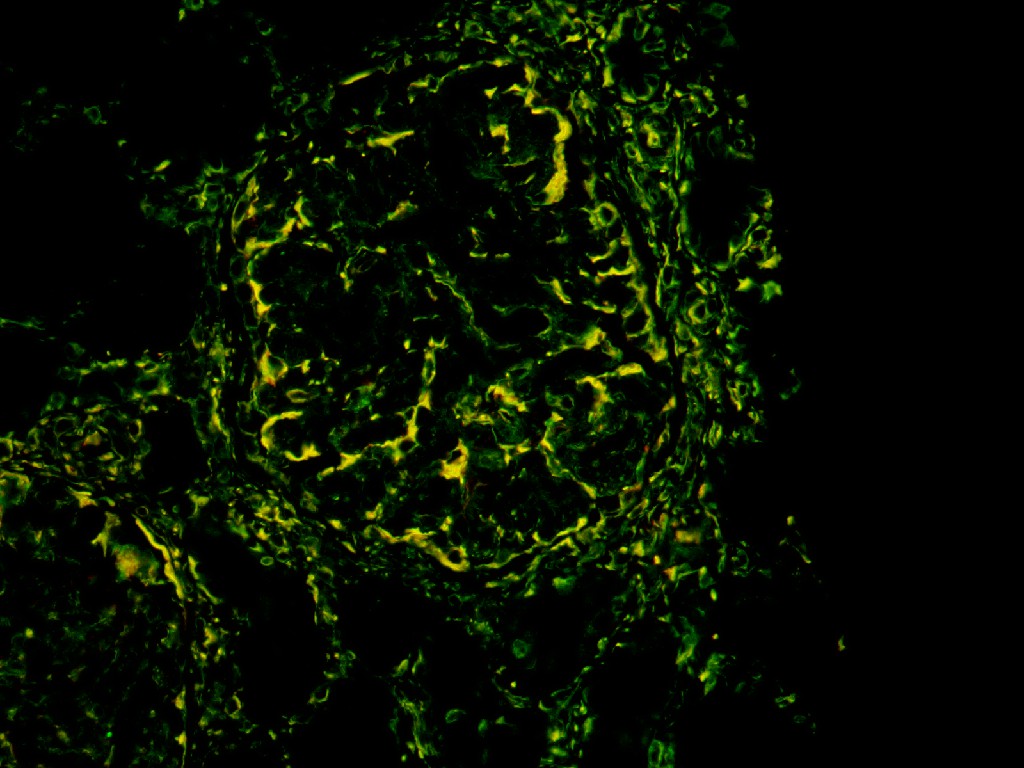

Supplement: Supplementary file 2 — Source data Fig. 1 [file 44321_2025_315_MOESM2_ESM.zip › Figure 1/F1A/1-GLDC-PDGFRbeta/Lee IV/12 (4).jpg]

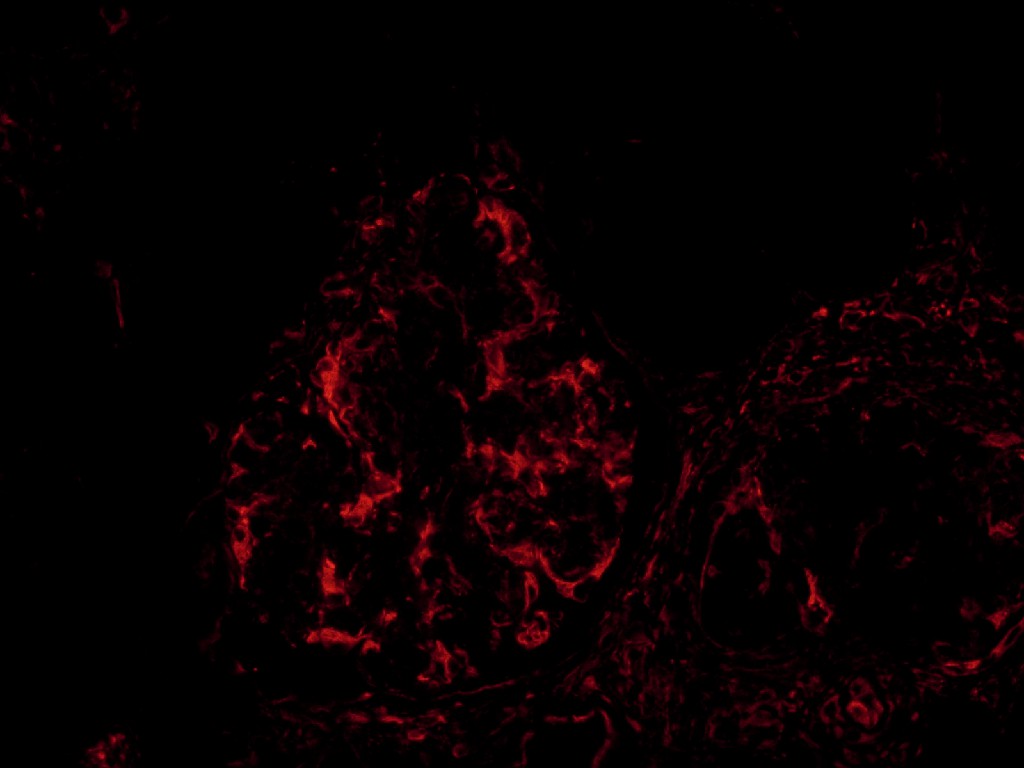

Supplement: Supplementary file 2 — Source data Fig. 1 [file 44321_2025_315_MOESM2_ESM.zip › Figure 1/F1A/1-GLDC-PDGFRbeta/Lee IV/2 (1).jpg]

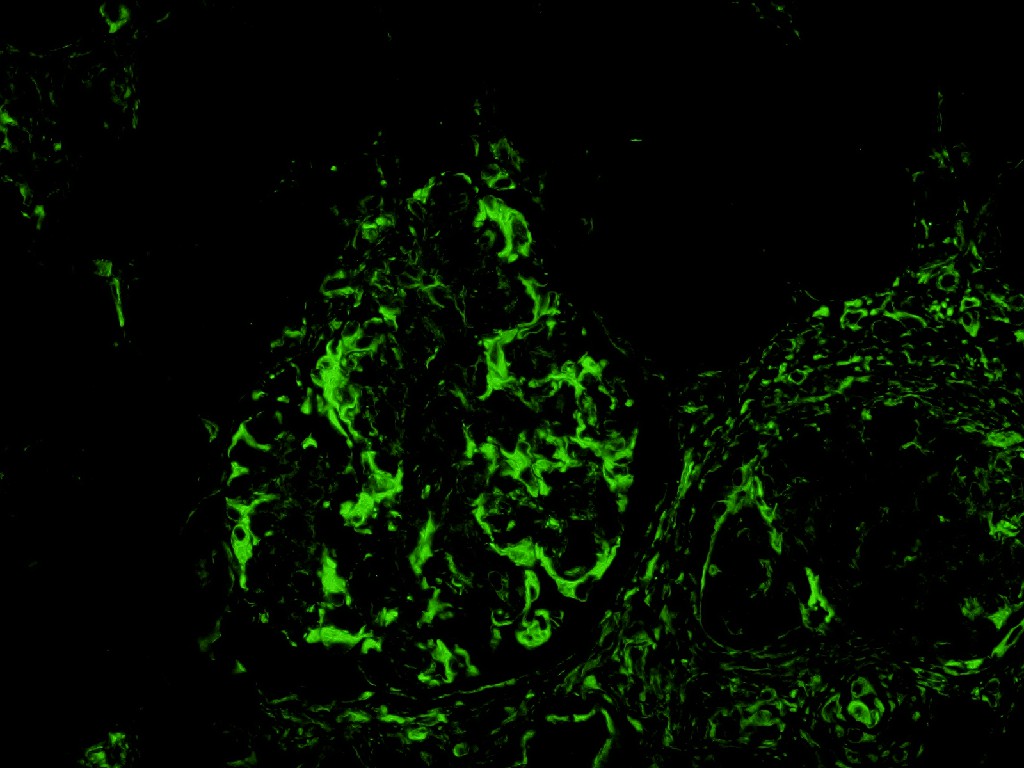

Supplement: Supplementary file 2 — Source data Fig. 1 [file 44321_2025_315_MOESM2_ESM.zip › Figure 1/F1A/1-GLDC-PDGFRbeta/Lee IV/2 (2).jpg]

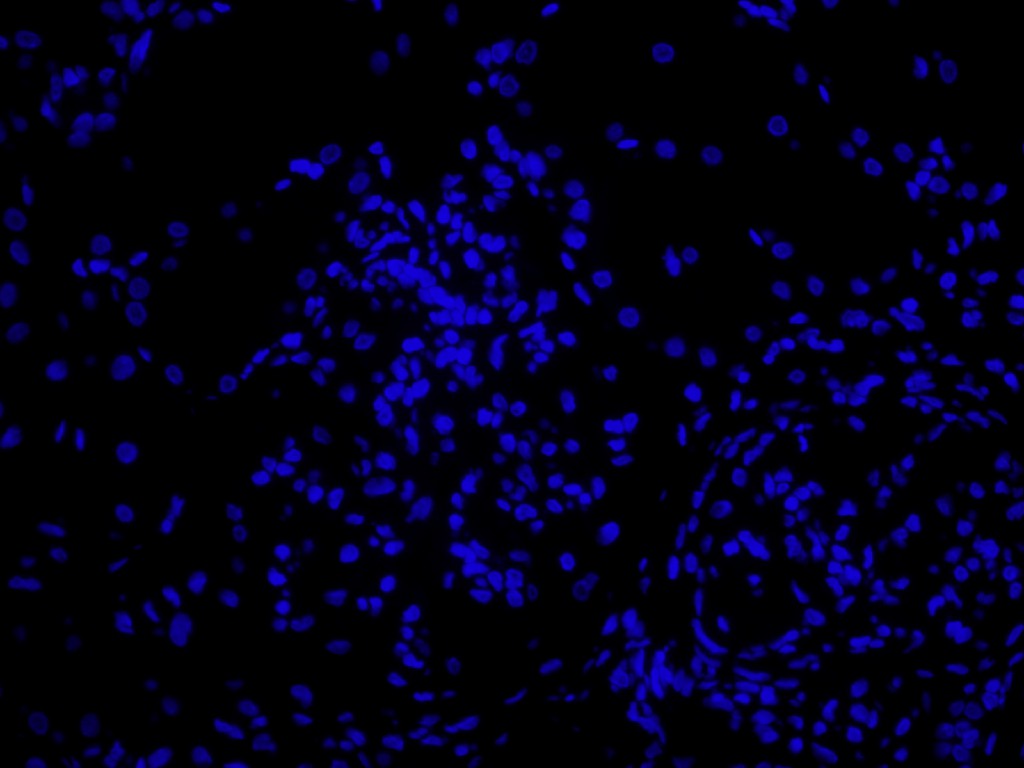

Supplement: Supplementary file 2 — Source data Fig. 1 [file 44321_2025_315_MOESM2_ESM.zip › Figure 1/F1A/1-GLDC-PDGFRbeta/Lee IV/2 (3).jpg]

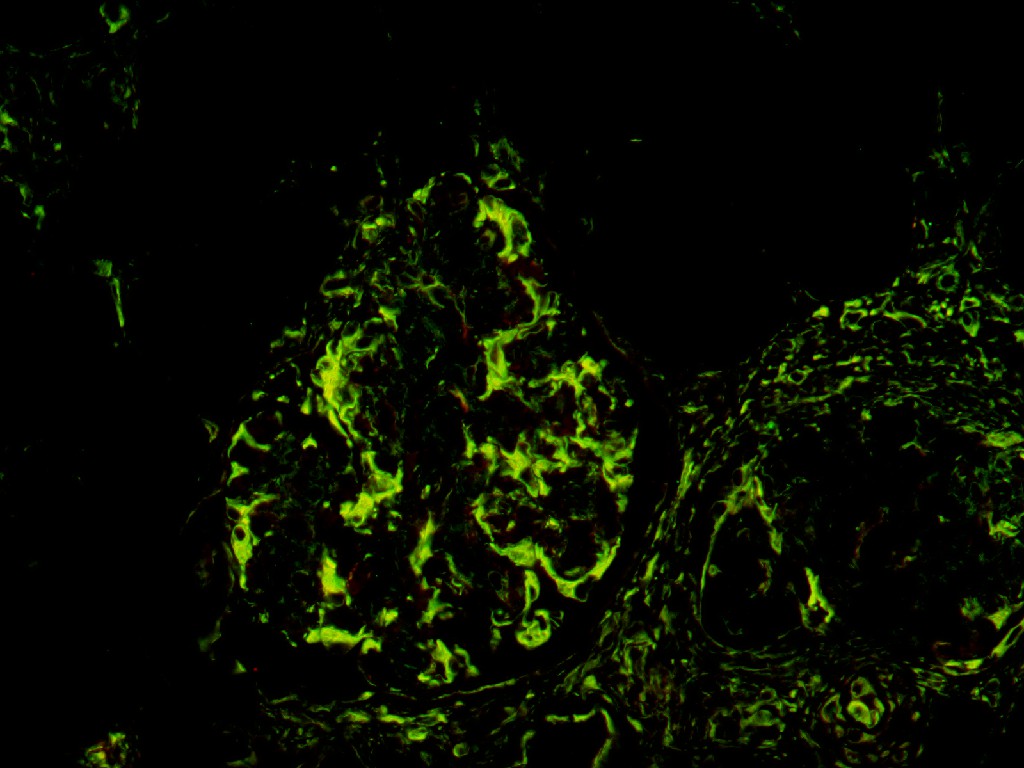

Supplement: Supplementary file 2 — Source data Fig. 1 [file 44321_2025_315_MOESM2_ESM.zip › Figure 1/F1A/1-GLDC-PDGFRbeta/Lee IV/2 (4).jpg]

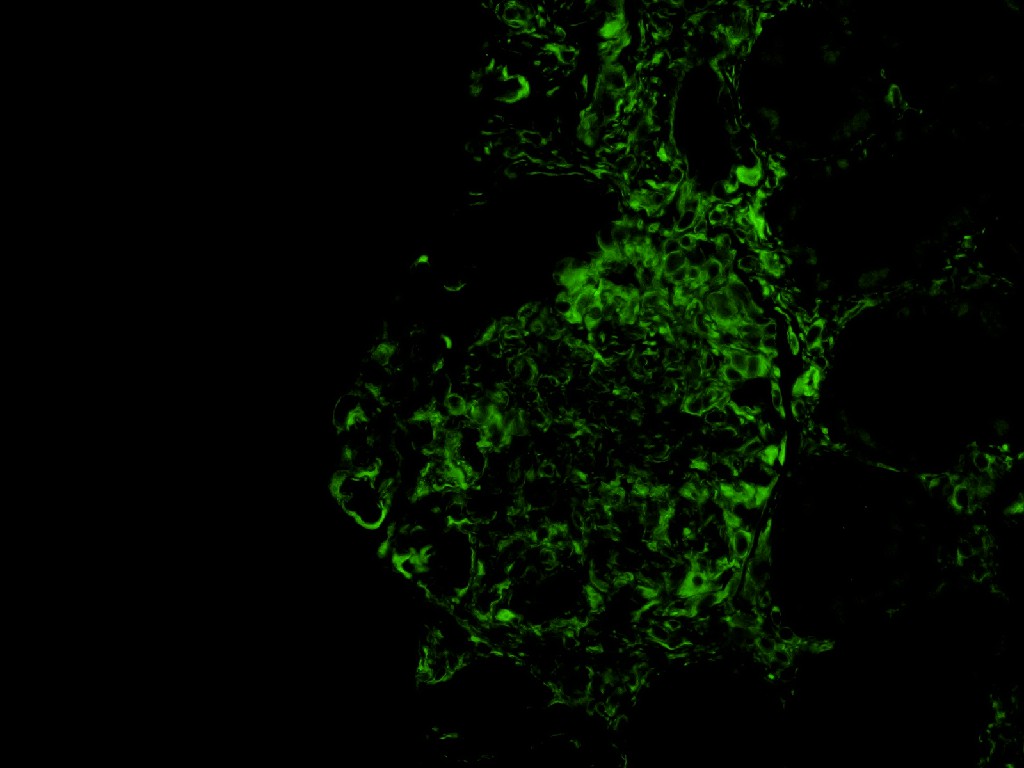

Supplement: Supplementary file 2 — Source data Fig. 1 [file 44321_2025_315_MOESM2_ESM.zip › Figure 1/F1A/1-GLDC-PDGFRbeta/Lee IV/3 (1).jpg]

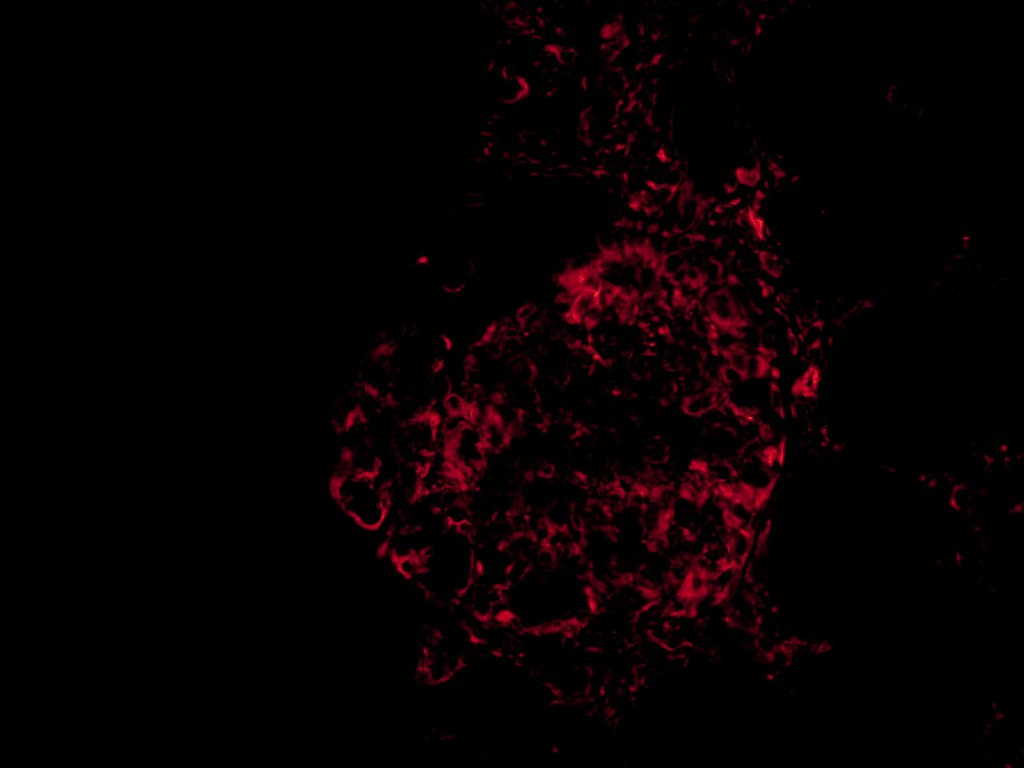

Supplement: Supplementary file 2 — Source data Fig. 1 [file 44321_2025_315_MOESM2_ESM.zip › Figure 1/F1A/1-GLDC-PDGFRbeta/Lee IV/3 (2).jpg]

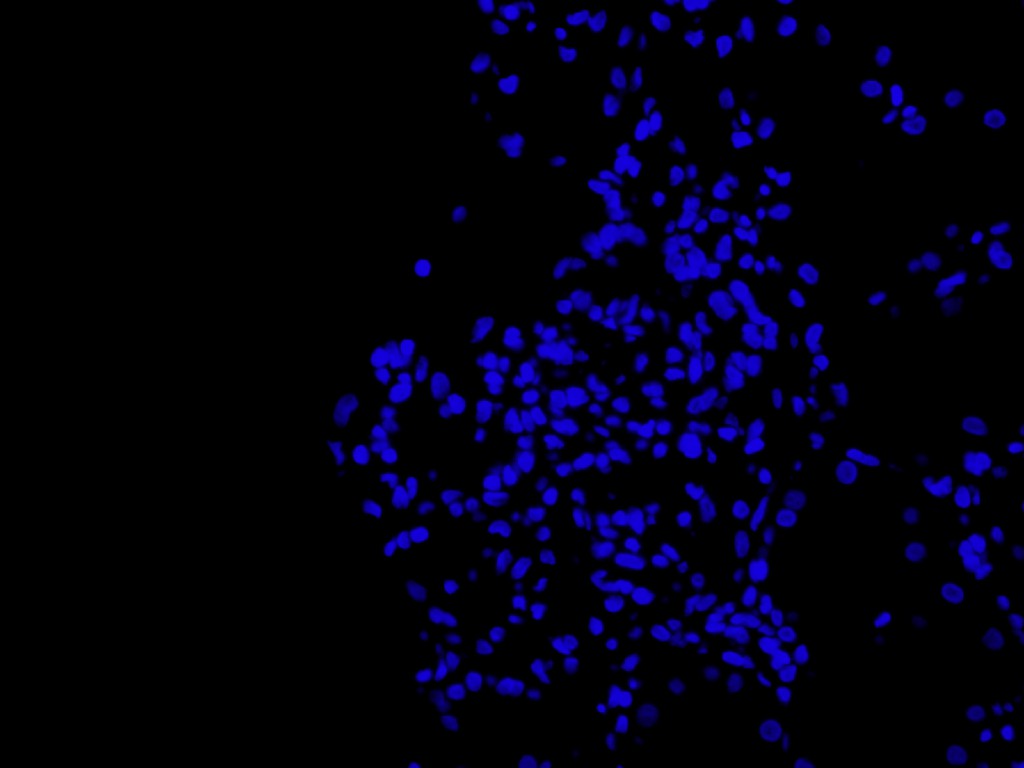

Supplement: Supplementary file 2 — Source data Fig. 1 [file 44321_2025_315_MOESM2_ESM.zip › Figure 1/F1A/1-GLDC-PDGFRbeta/Lee IV/3 (3).jpg]

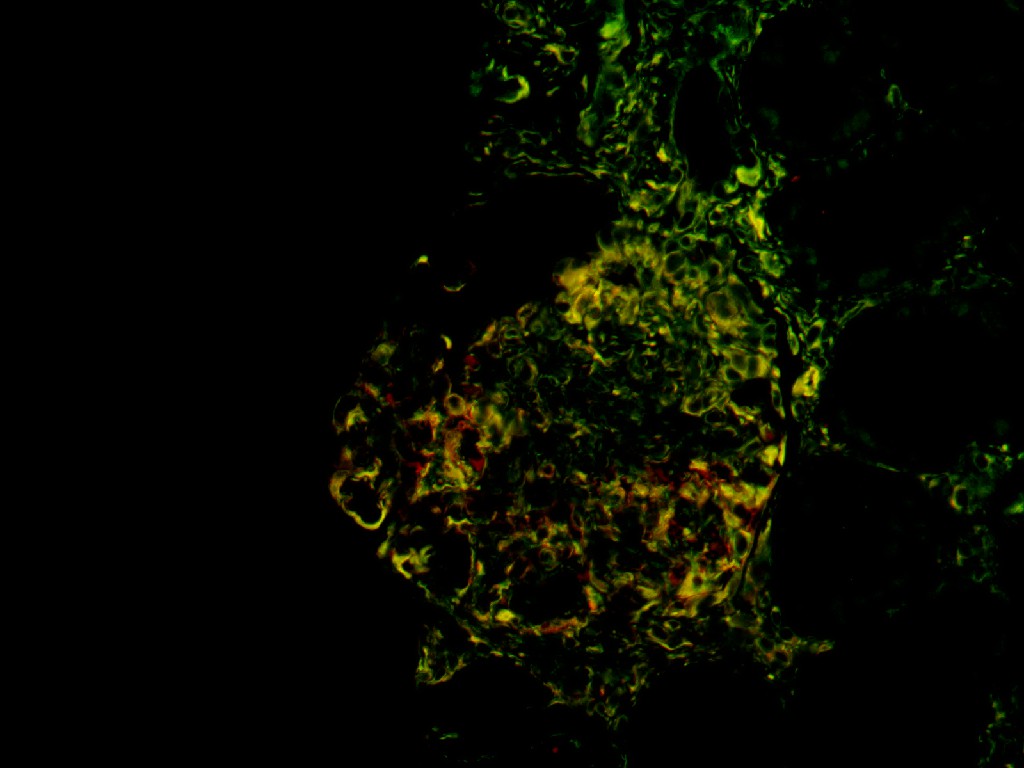

Supplement: Supplementary file 2 — Source data Fig. 1 [file 44321_2025_315_MOESM2_ESM.zip › Figure 1/F1A/1-GLDC-PDGFRbeta/Lee IV/3 (4).jpg]

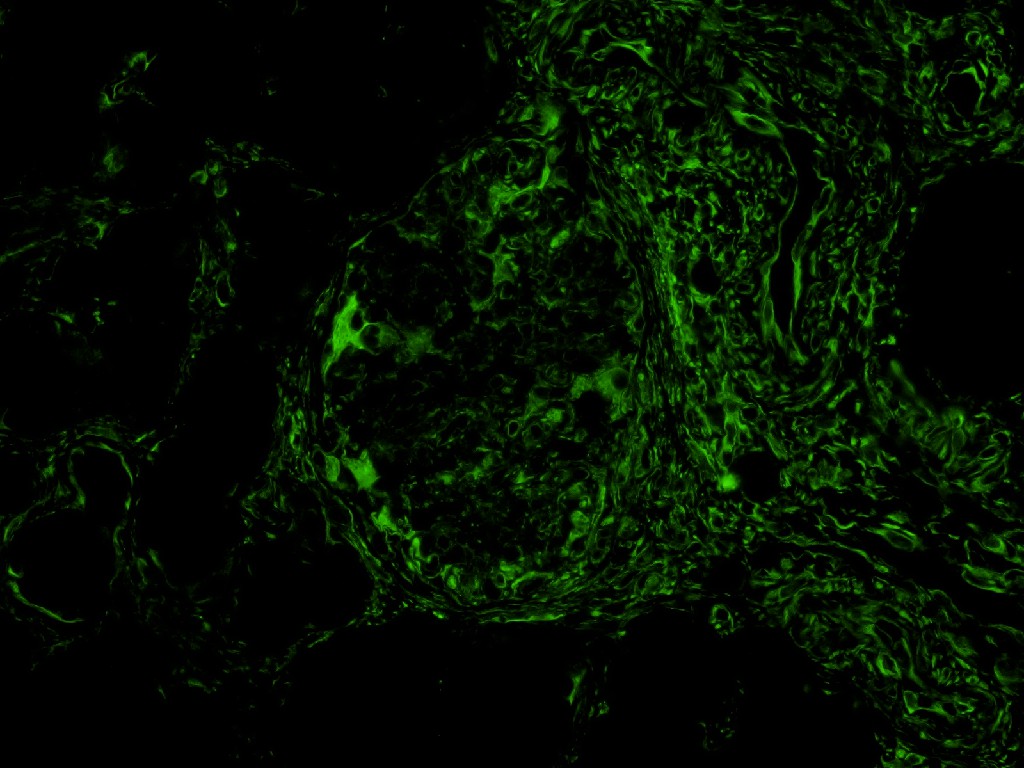

Supplement: Supplementary file 2 — Source data Fig. 1 [file 44321_2025_315_MOESM2_ESM.zip › Figure 1/F1A/1-GLDC-PDGFRbeta/Lee IV/4 (1).jpg]

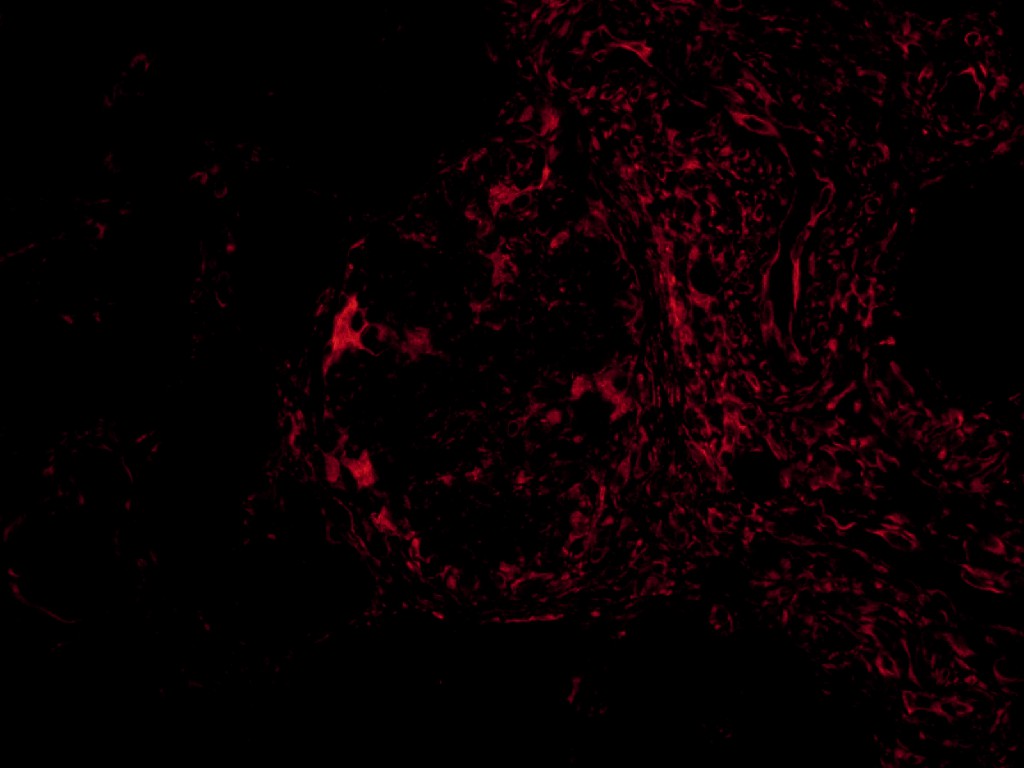

Supplement: Supplementary file 2 — Source data Fig. 1 [file 44321_2025_315_MOESM2_ESM.zip › Figure 1/F1A/1-GLDC-PDGFRbeta/Lee IV/4 (2).jpg]

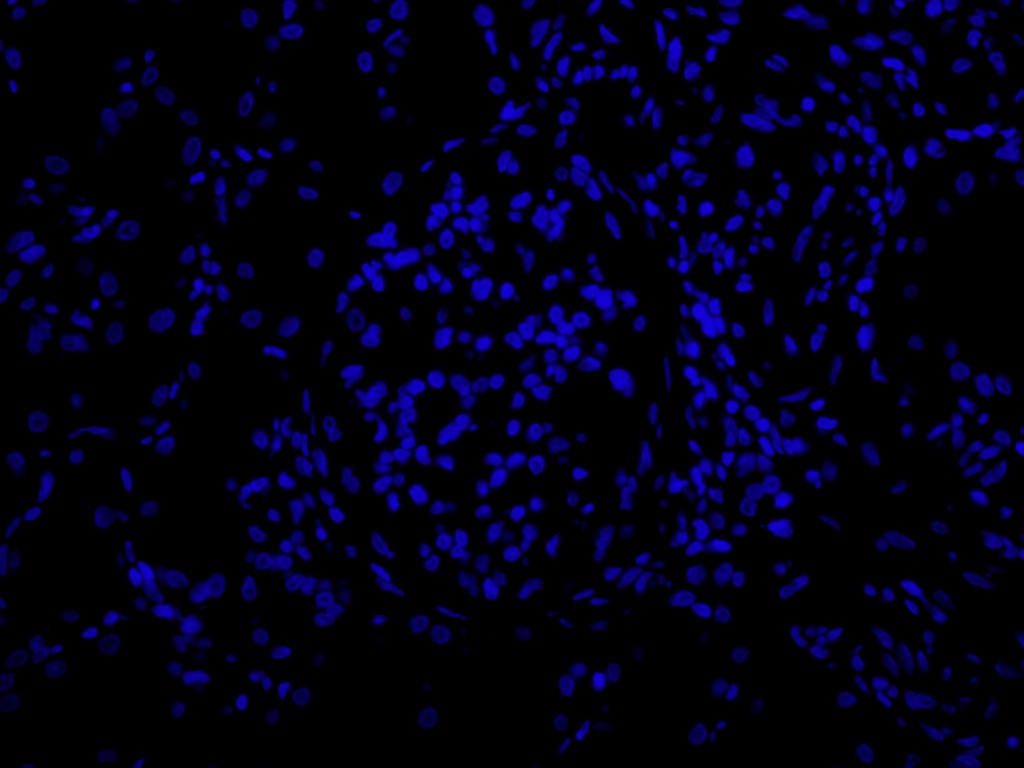

Supplement: Supplementary file 2 — Source data Fig. 1 [file 44321_2025_315_MOESM2_ESM.zip › Figure 1/F1A/1-GLDC-PDGFRbeta/Lee IV/4 (3).jpg]

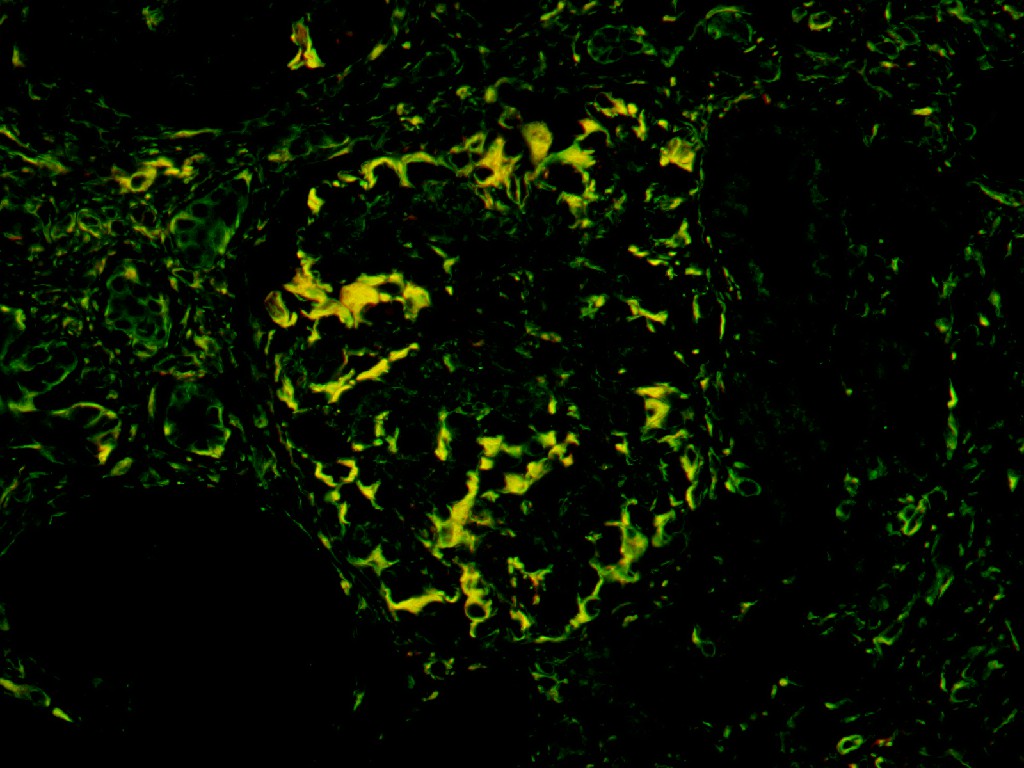

Supplement: Supplementary file 2 — Source data Fig. 1 [file 44321_2025_315_MOESM2_ESM.zip › Figure 1/F1A/1-GLDC-PDGFRbeta/Lee IV/4 (4).jpg]

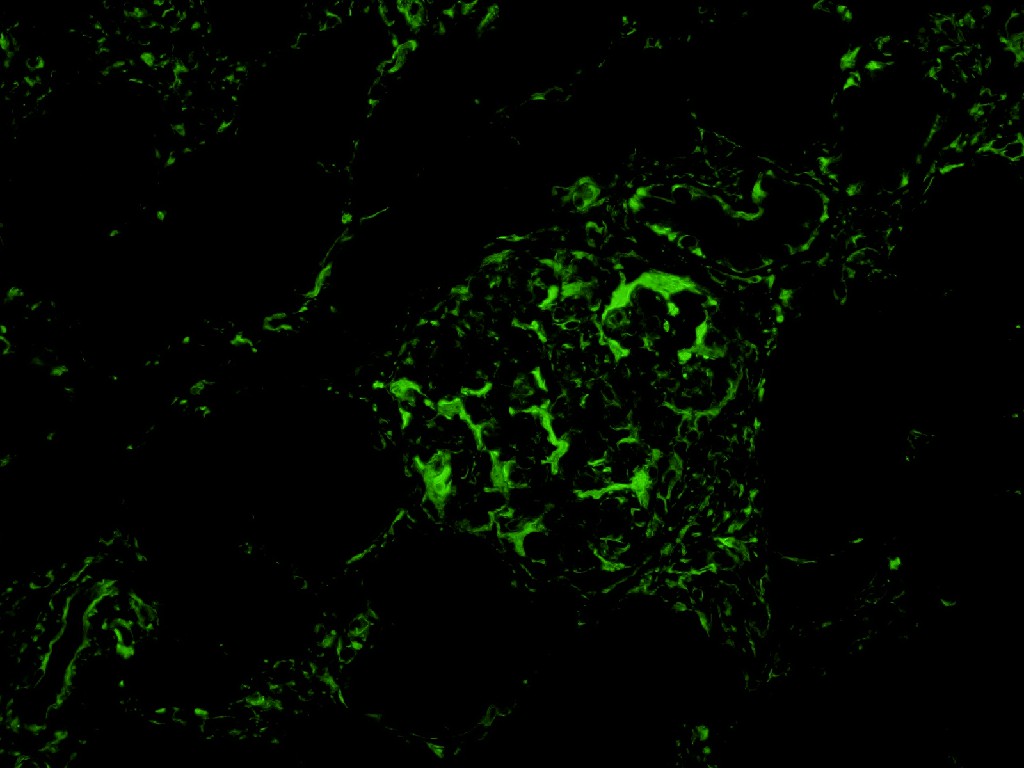

Supplement: Supplementary file 2 — Source data Fig. 1 [file 44321_2025_315_MOESM2_ESM.zip › Figure 1/F1A/1-GLDC-PDGFRbeta/Lee IV/5 (1).jpg]

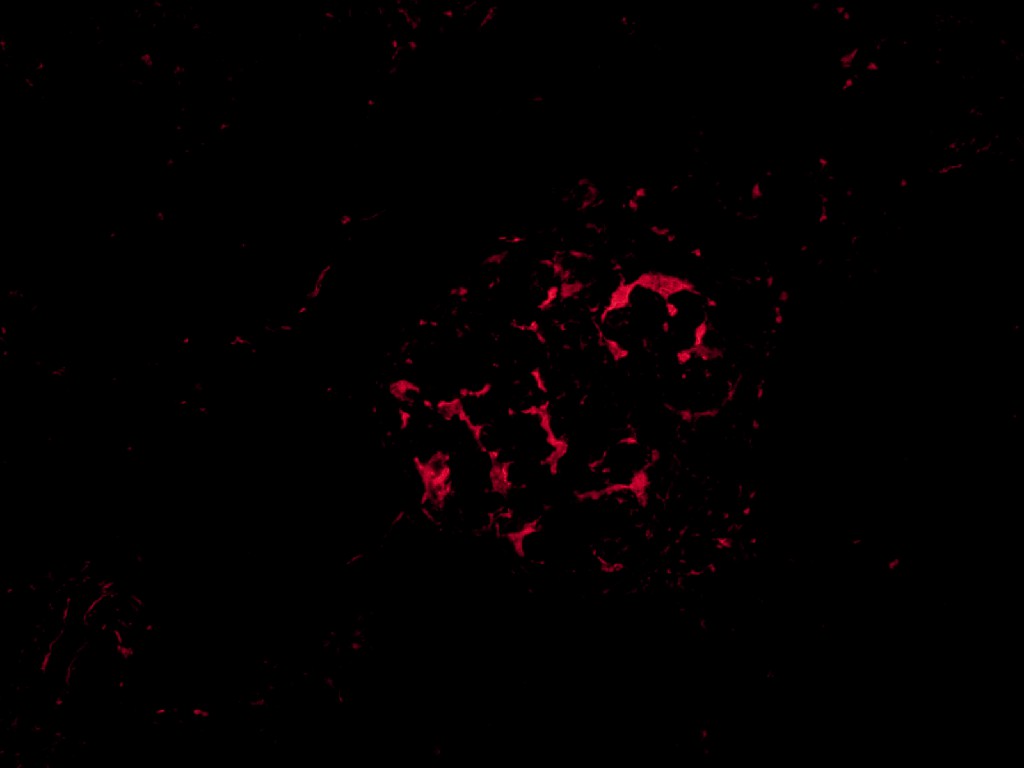

Supplement: Supplementary file 2 — Source data Fig. 1 [file 44321_2025_315_MOESM2_ESM.zip › Figure 1/F1A/1-GLDC-PDGFRbeta/Lee IV/5 (2).jpg]

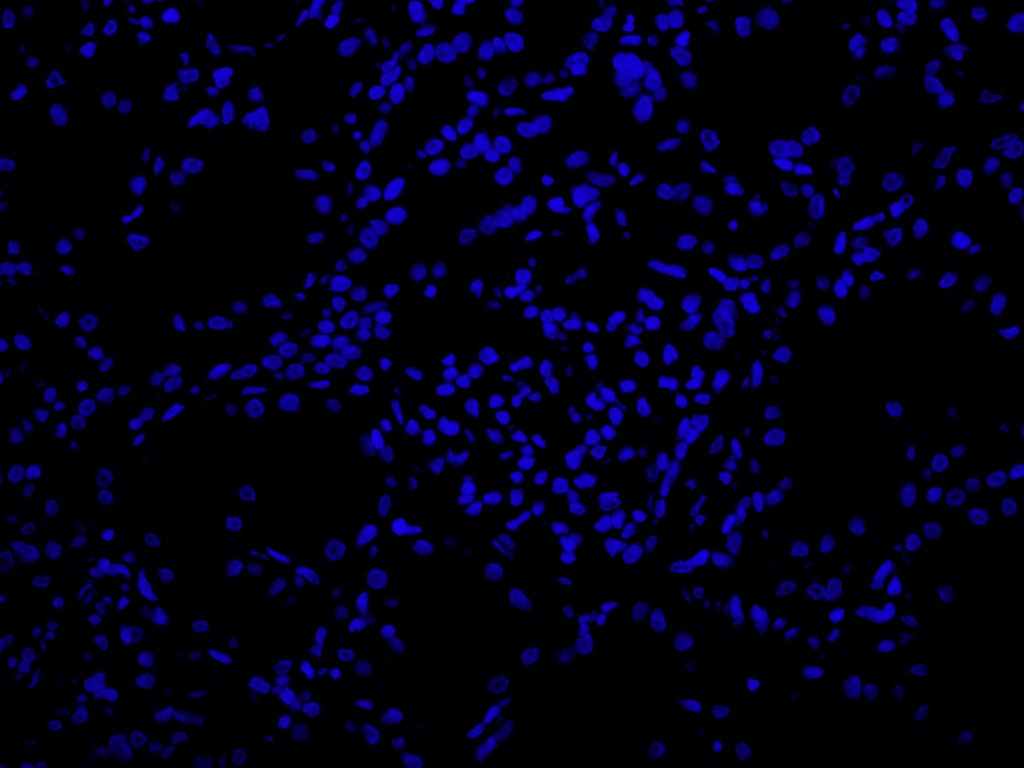

Supplement: Supplementary file 2 — Source data Fig. 1 [file 44321_2025_315_MOESM2_ESM.zip › Figure 1/F1A/1-GLDC-PDGFRbeta/Lee IV/5 (3).jpg]

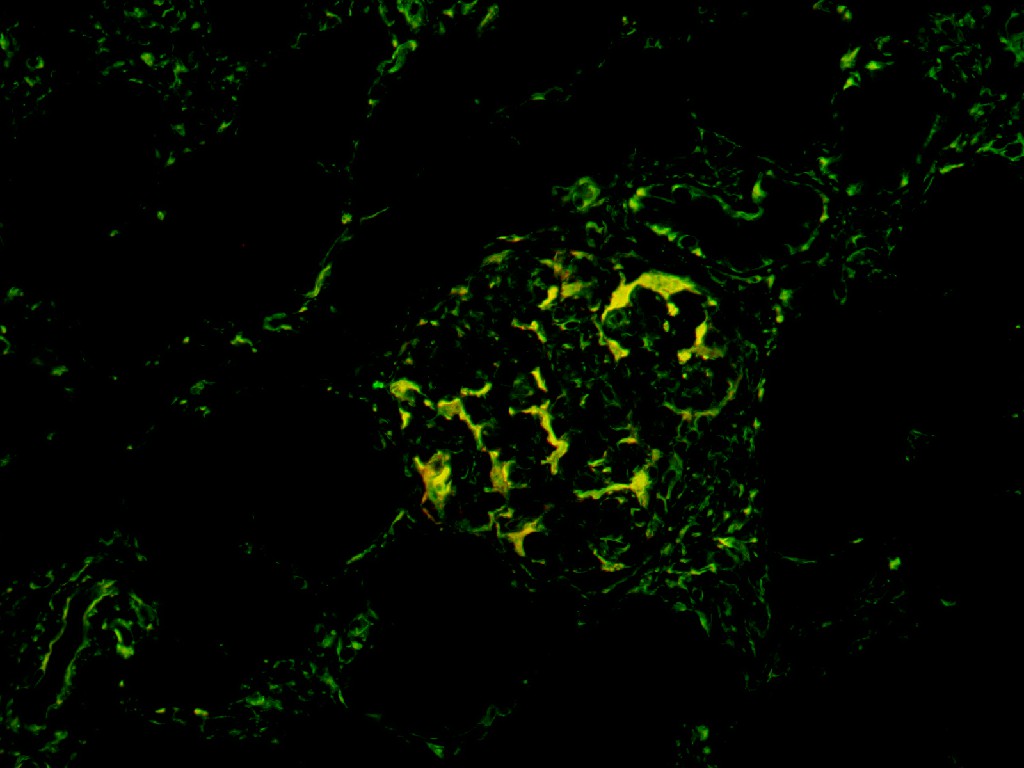

Supplement: Supplementary file 2 — Source data Fig. 1 [file 44321_2025_315_MOESM2_ESM.zip › Figure 1/F1A/1-GLDC-PDGFRbeta/Lee IV/5 (4).jpg]

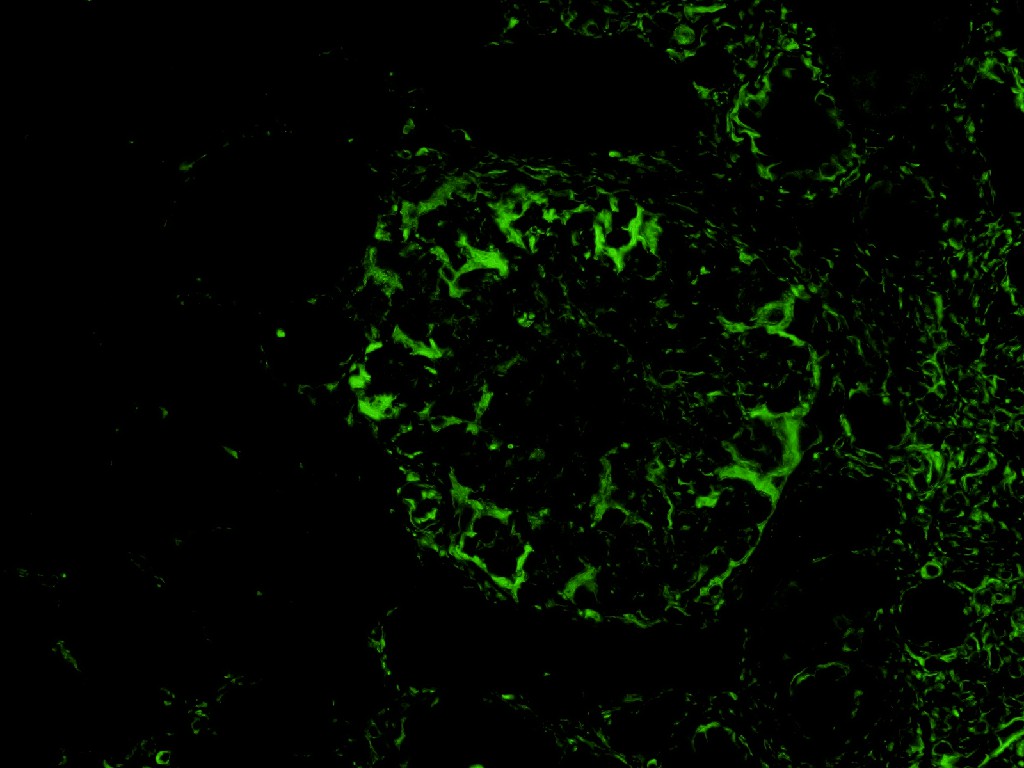

Supplement: Supplementary file 2 — Source data Fig. 1 [file 44321_2025_315_MOESM2_ESM.zip › Figure 1/F1A/1-GLDC-PDGFRbeta/Lee IV/6 (1).jpg]

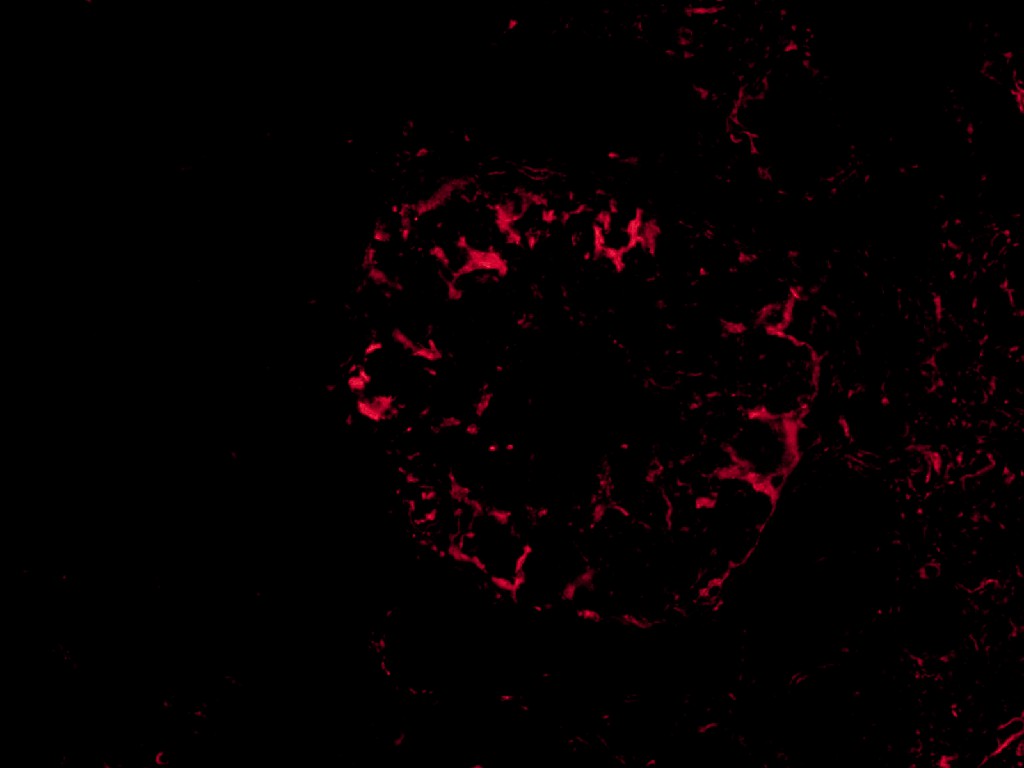

Supplement: Supplementary file 2 — Source data Fig. 1 [file 44321_2025_315_MOESM2_ESM.zip › Figure 1/F1A/1-GLDC-PDGFRbeta/Lee IV/6 (2).jpg]

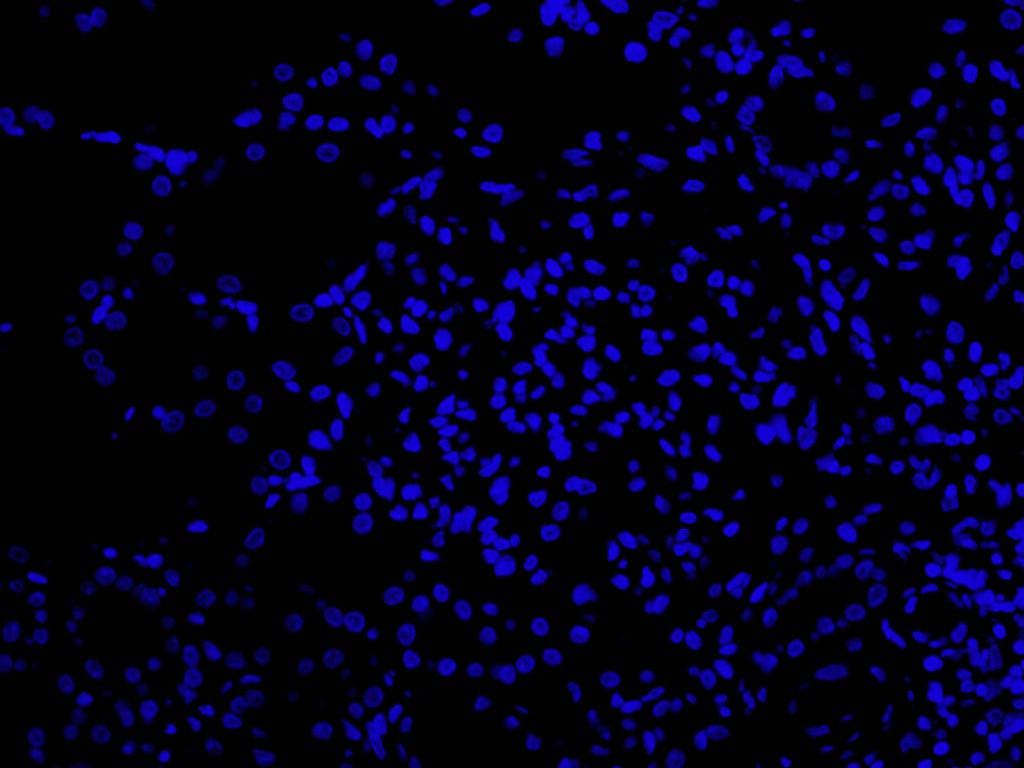

Supplement: Supplementary file 2 — Source data Fig. 1 [file 44321_2025_315_MOESM2_ESM.zip › Figure 1/F1A/1-GLDC-PDGFRbeta/Lee IV/6 (3).jpg]

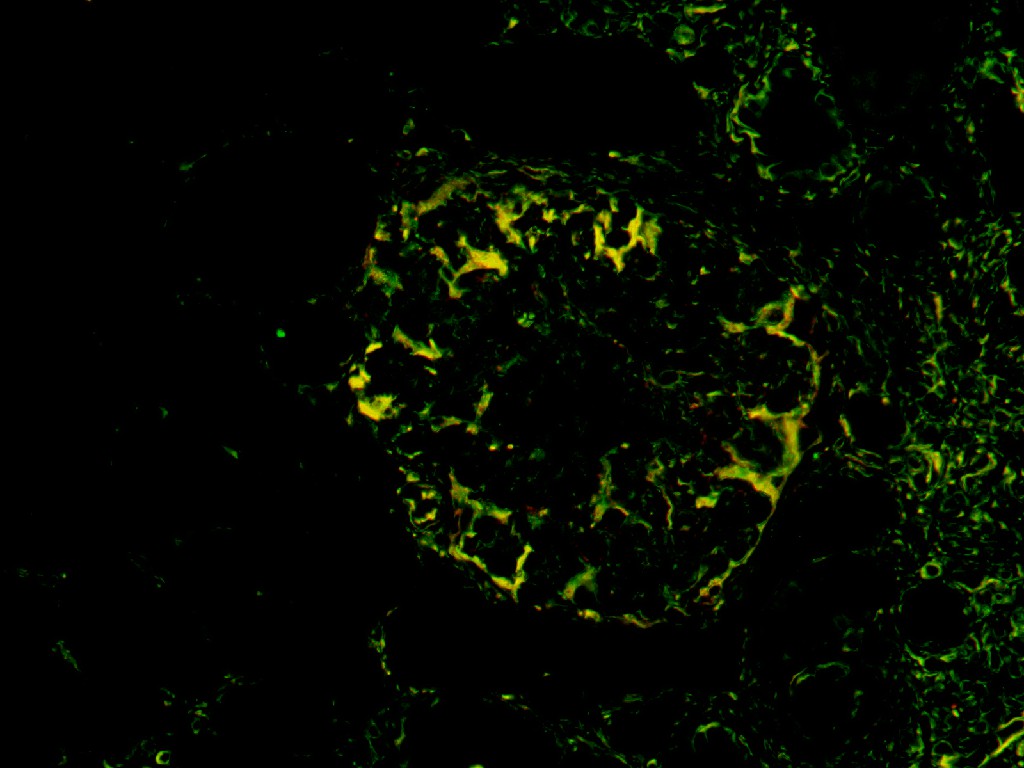

Supplement: Supplementary file 2 — Source data Fig. 1 [file 44321_2025_315_MOESM2_ESM.zip › Figure 1/F1A/1-GLDC-PDGFRbeta/Lee IV/6 (4).jpg]

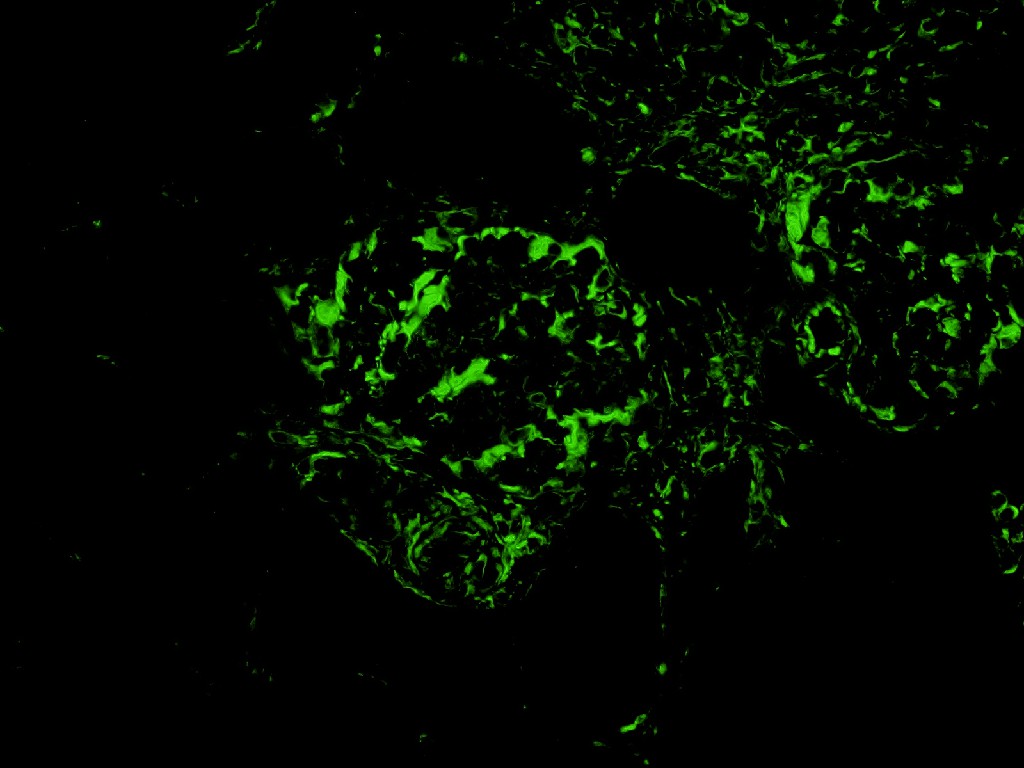

Supplement: Supplementary file 2 — Source data Fig. 1 [file 44321_2025_315_MOESM2_ESM.zip › Figure 1/F1A/1-GLDC-PDGFRbeta/Lee IV/7 (1).jpg]

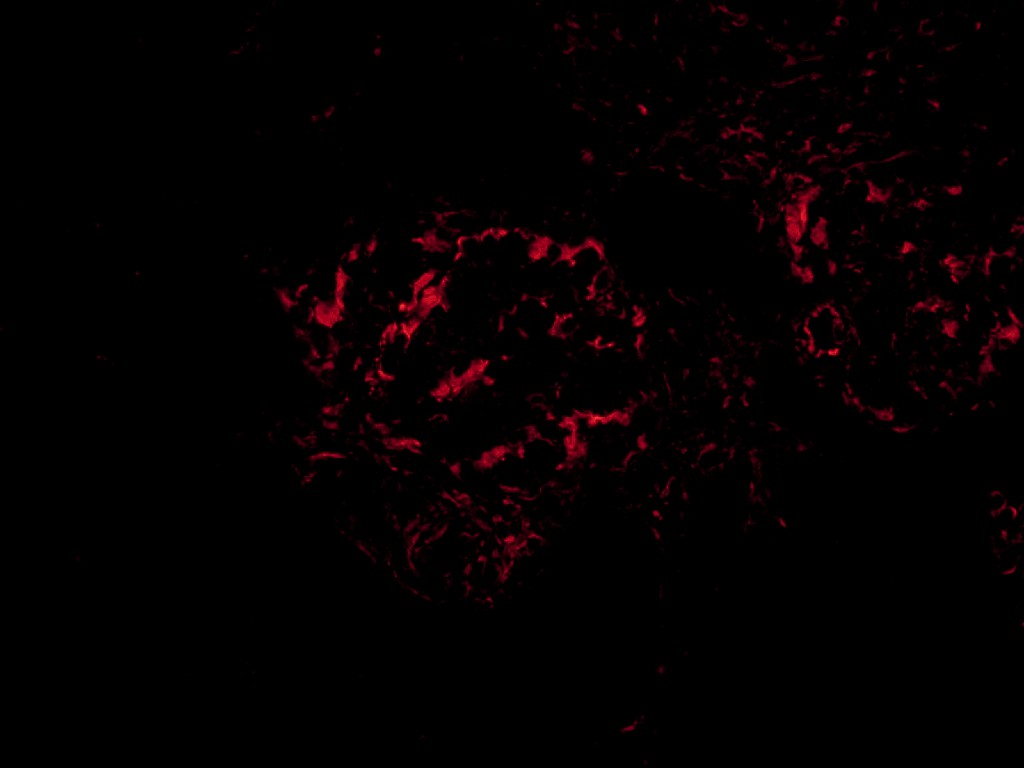

Supplement: Supplementary file 2 — Source data Fig. 1 [file 44321_2025_315_MOESM2_ESM.zip › Figure 1/F1A/1-GLDC-PDGFRbeta/Lee IV/7 (2).jpg]

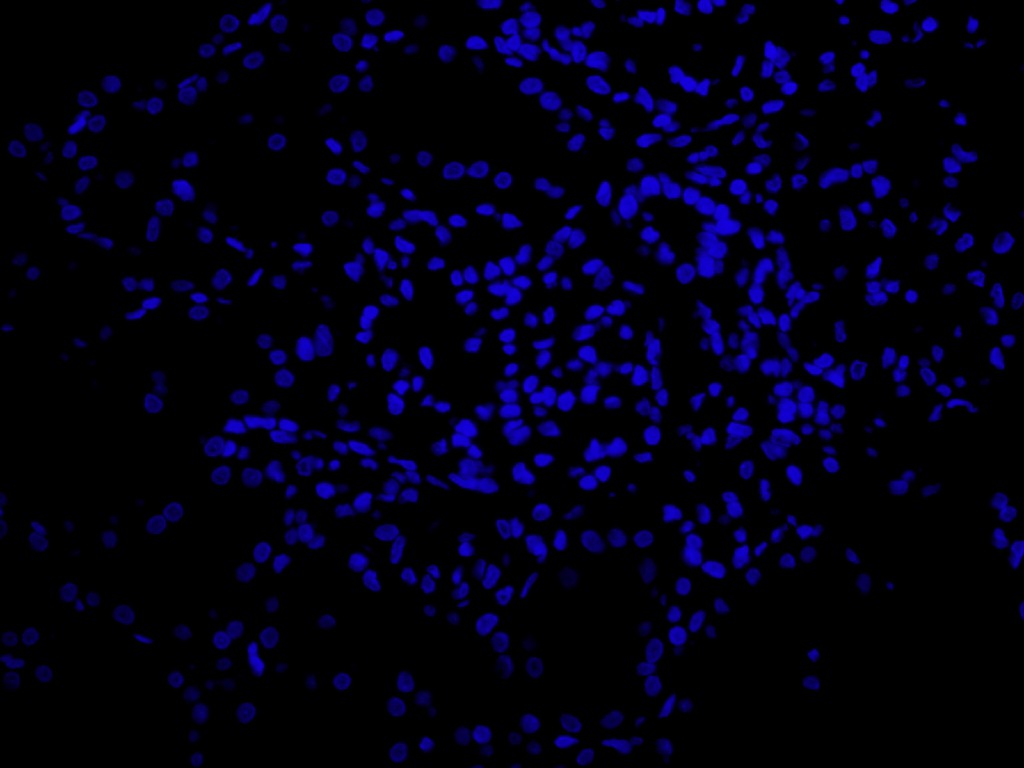

Supplement: Supplementary file 2 — Source data Fig. 1 [file 44321_2025_315_MOESM2_ESM.zip › Figure 1/F1A/1-GLDC-PDGFRbeta/Lee IV/7 (3).jpg]

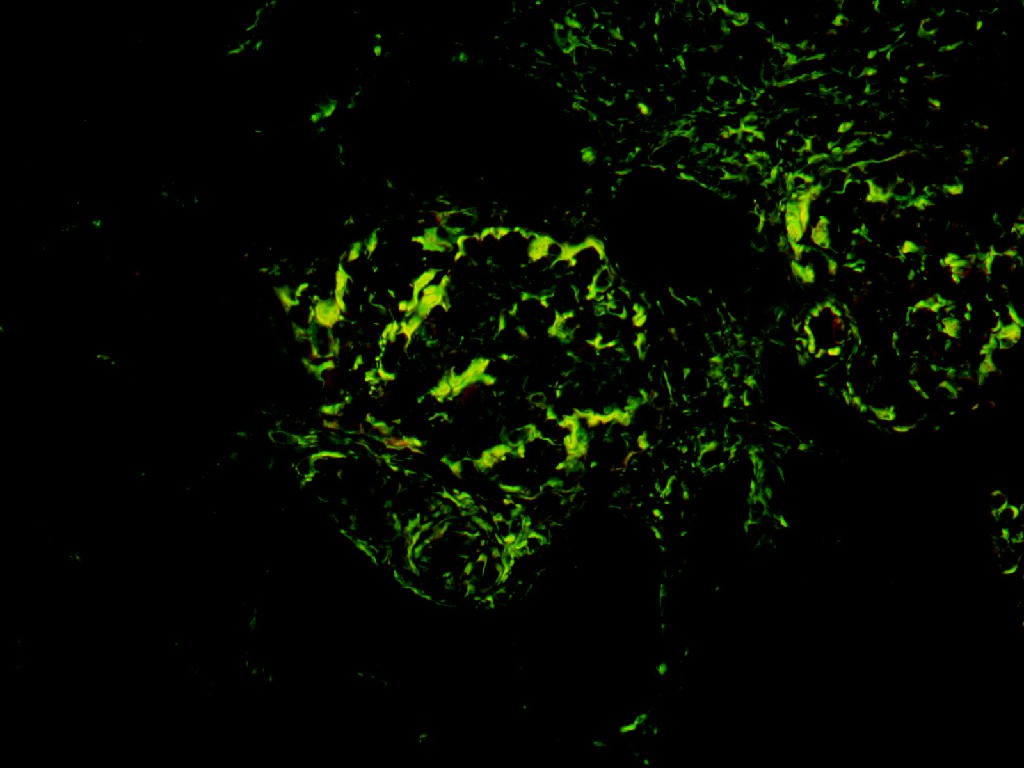

Supplement: Supplementary file 2 — Source data Fig. 1 [file 44321_2025_315_MOESM2_ESM.zip › Figure 1/F1A/1-GLDC-PDGFRbeta/Lee IV/7 (4).jpg]

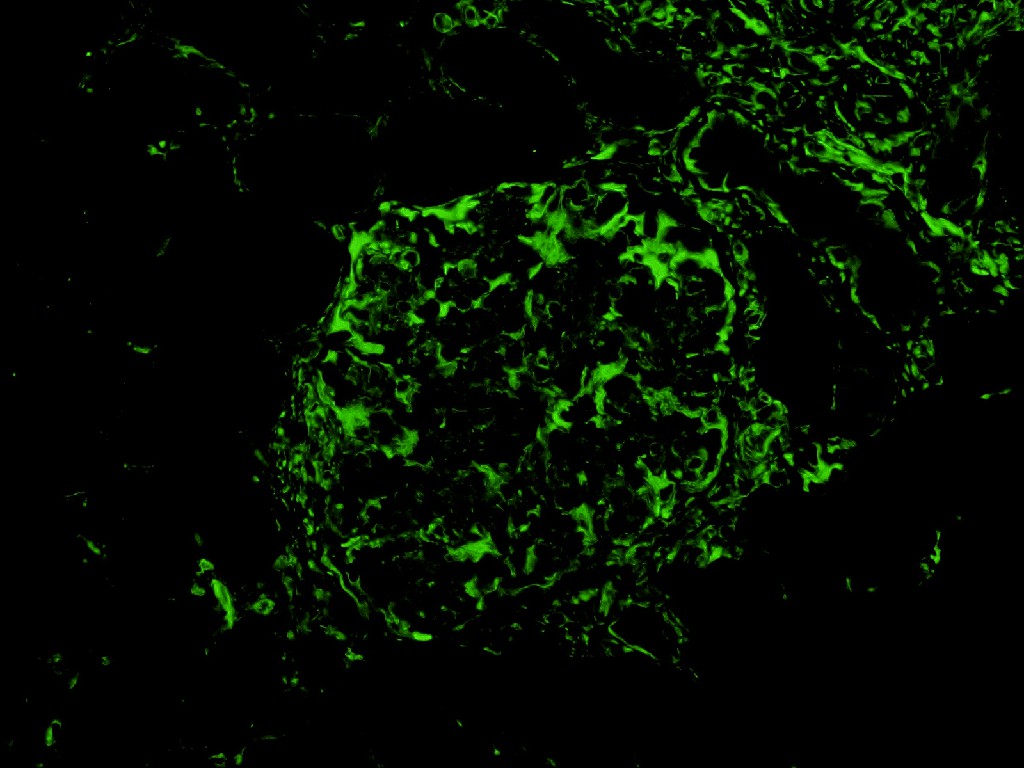

Supplement: Supplementary file 2 — Source data Fig. 1 [file 44321_2025_315_MOESM2_ESM.zip › Figure 1/F1A/1-GLDC-PDGFRbeta/Lee IV/8 (1).jpg]

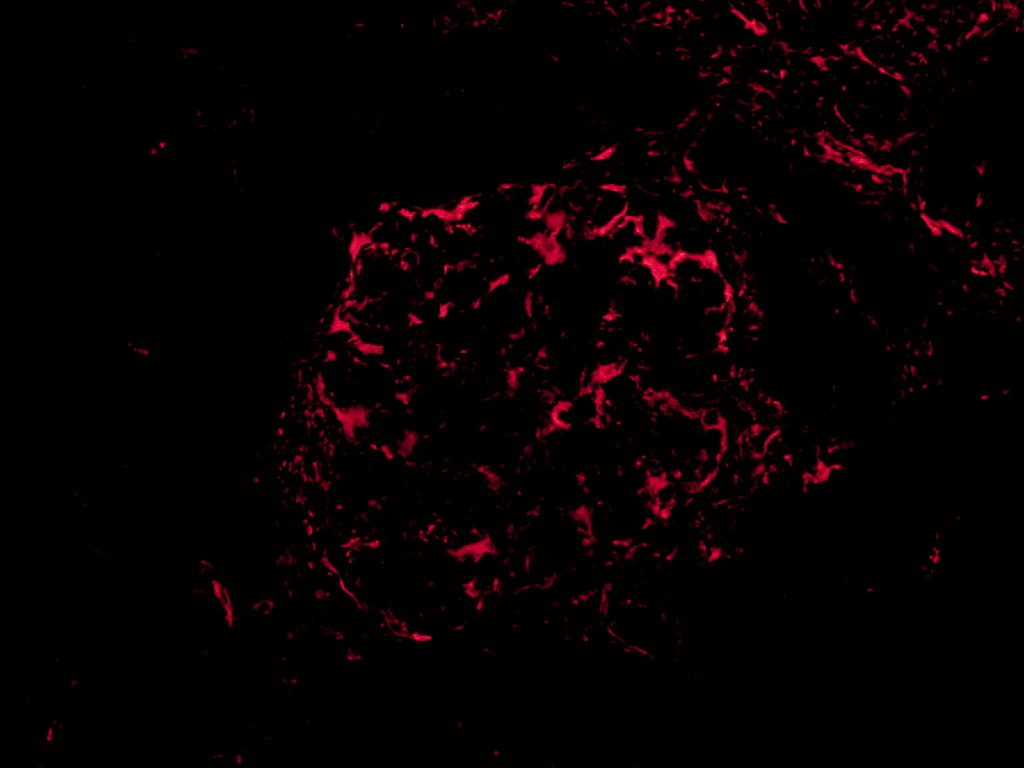

Supplement: Supplementary file 2 — Source data Fig. 1 [file 44321_2025_315_MOESM2_ESM.zip › Figure 1/F1A/1-GLDC-PDGFRbeta/Lee IV/8 (2).jpg]

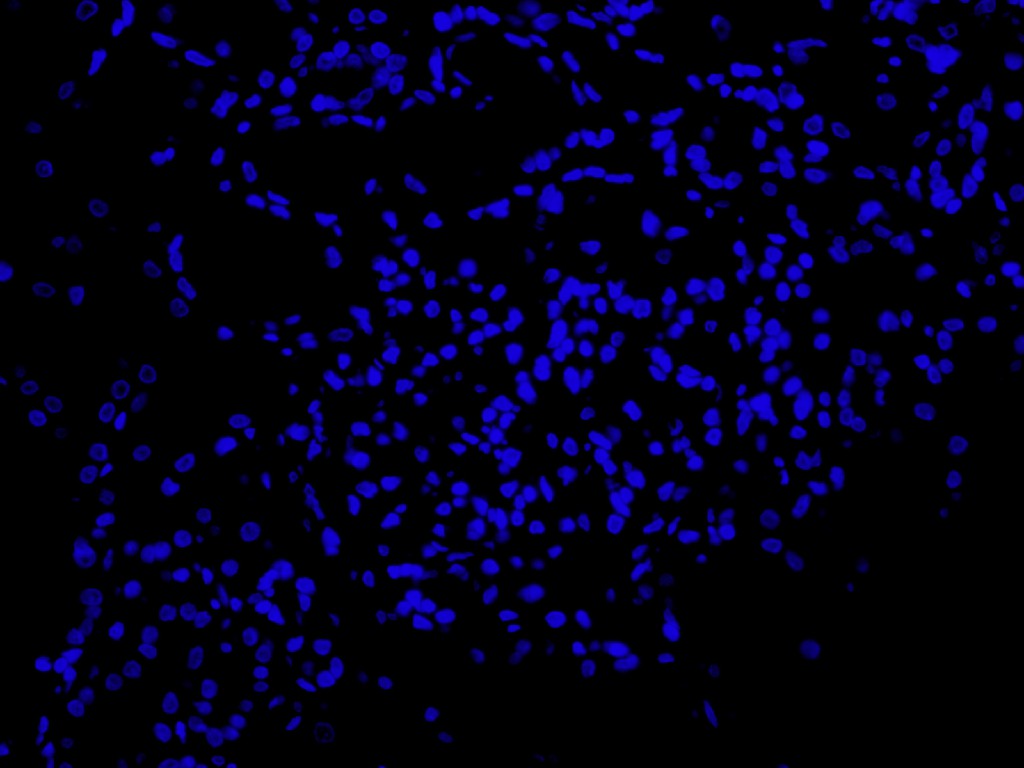

Supplement: Supplementary file 2 — Source data Fig. 1 [file 44321_2025_315_MOESM2_ESM.zip › Figure 1/F1A/1-GLDC-PDGFRbeta/Lee IV/8 (3).jpg]

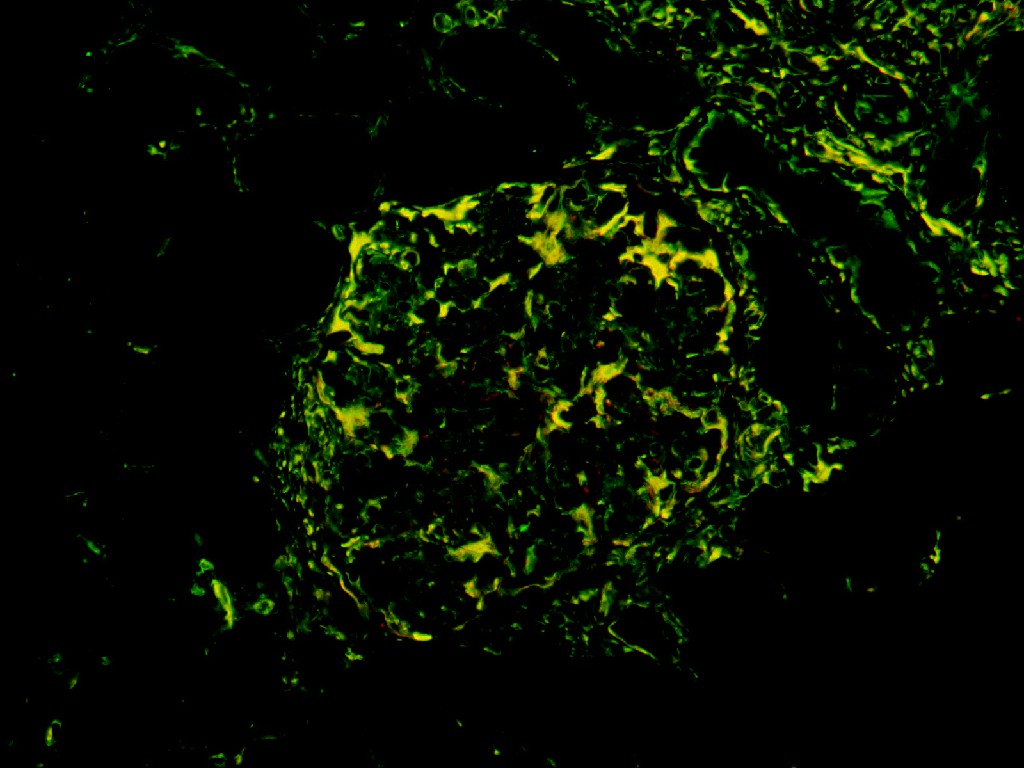

Supplement: Supplementary file 2 — Source data Fig. 1 [file 44321_2025_315_MOESM2_ESM.zip › Figure 1/F1A/1-GLDC-PDGFRbeta/Lee IV/8 (4).jpg]

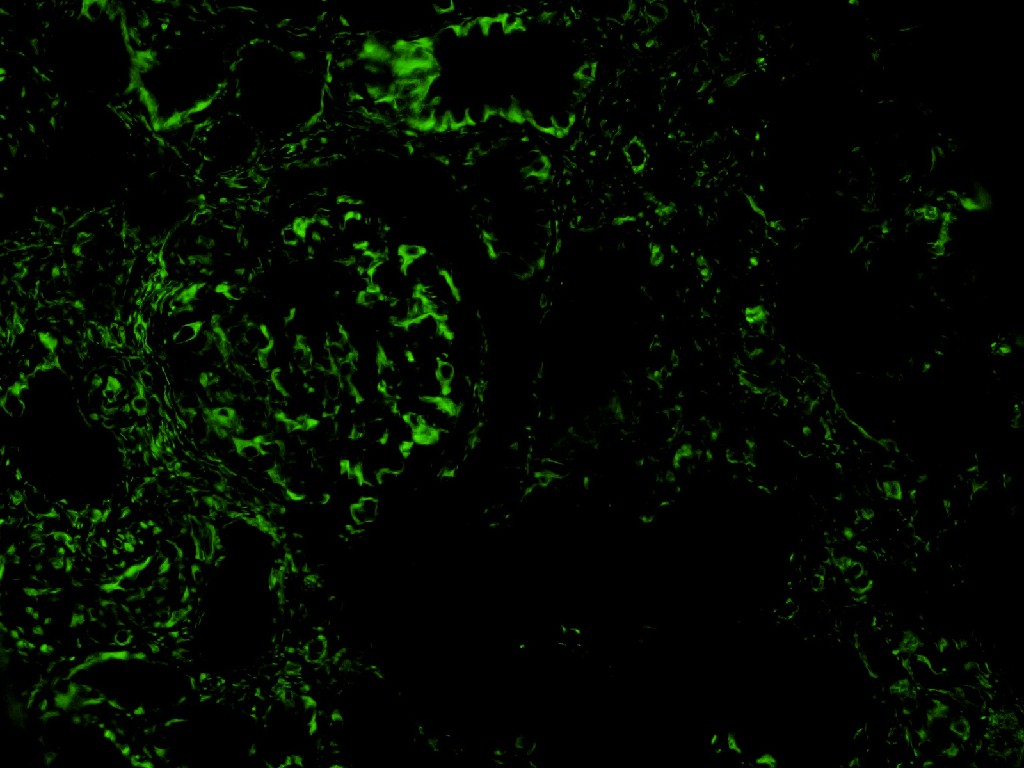

Supplement: Supplementary file 2 — Source data Fig. 1 [file 44321_2025_315_MOESM2_ESM.zip › Figure 1/F1A/1-GLDC-PDGFRbeta/Lee IV/9 (1).jpg]

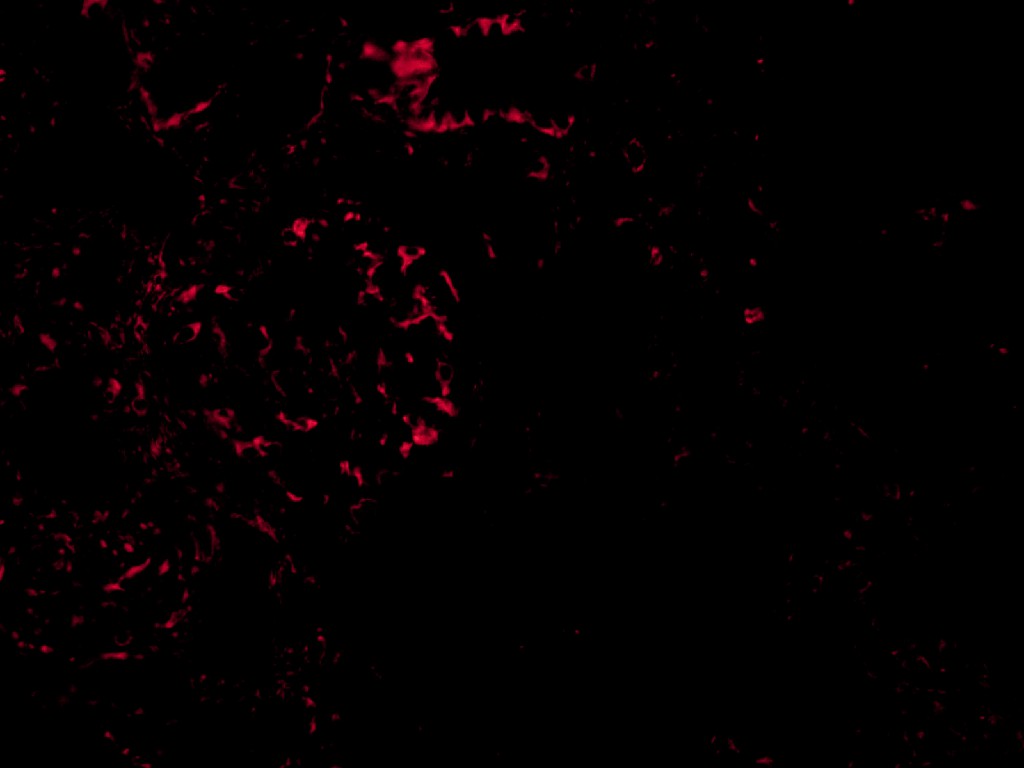

Supplement: Supplementary file 2 — Source data Fig. 1 [file 44321_2025_315_MOESM2_ESM.zip › Figure 1/F1A/1-GLDC-PDGFRbeta/Lee IV/9 (2).jpg]

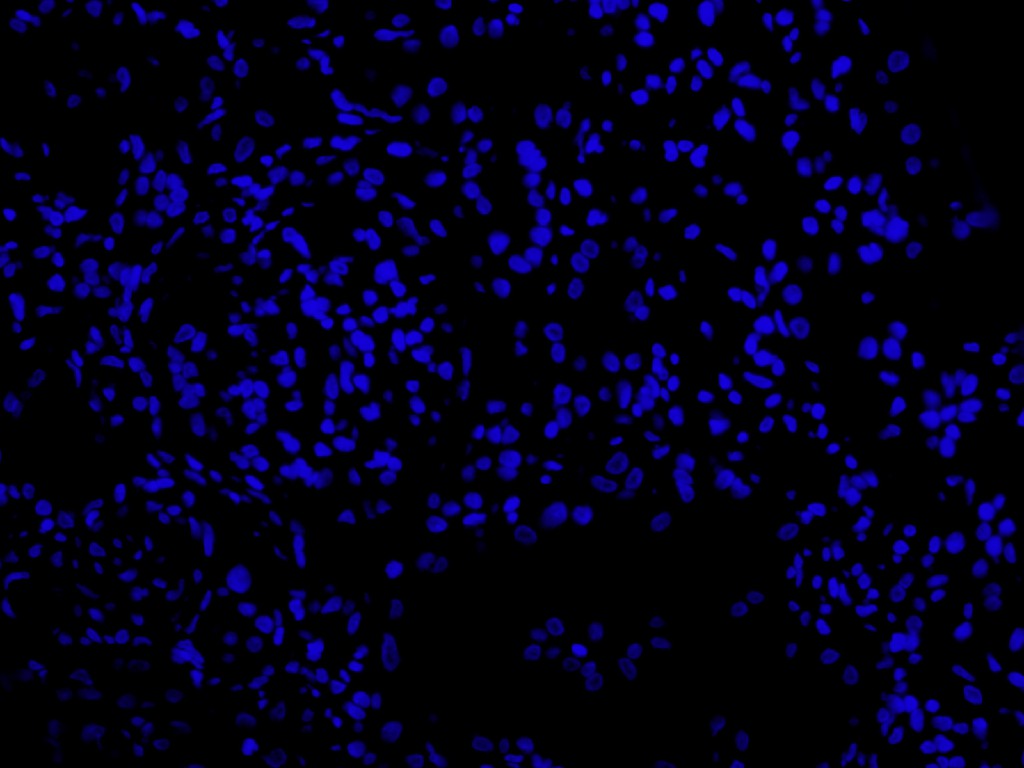

Supplement: Supplementary file 2 — Source data Fig. 1 [file 44321_2025_315_MOESM2_ESM.zip › Figure 1/F1A/1-GLDC-PDGFRbeta/Lee IV/9 (3).jpg]

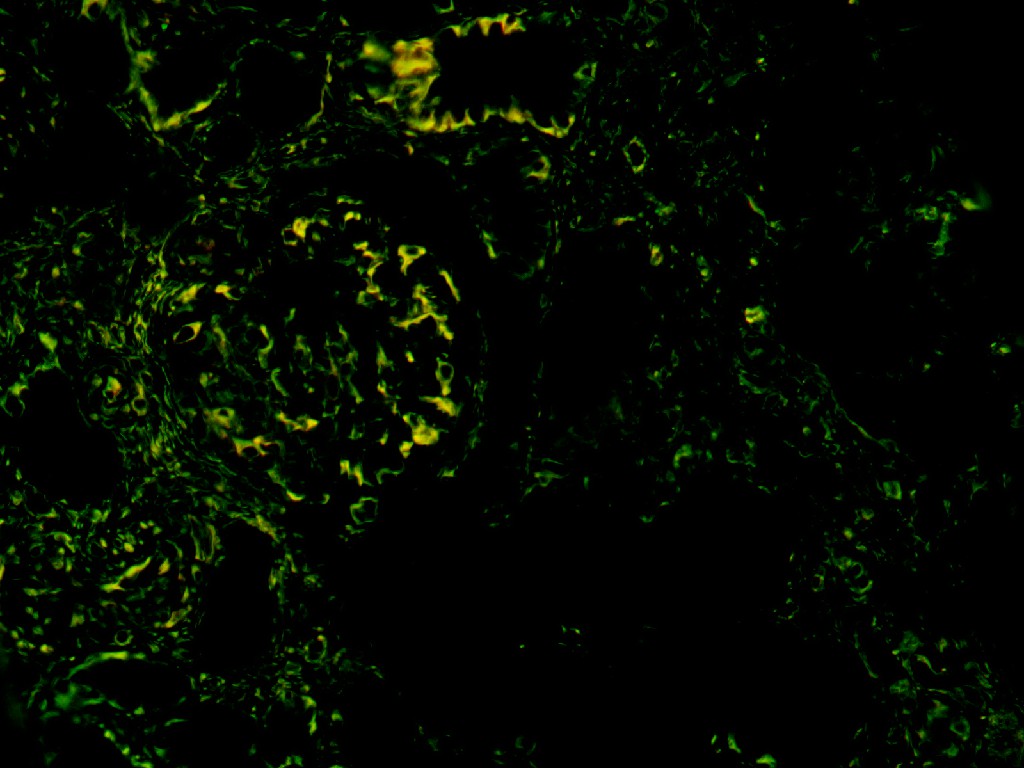

Supplement: Supplementary file 2 — Source data Fig. 1 [file 44321_2025_315_MOESM2_ESM.zip › Figure 1/F1A/1-GLDC-PDGFRbeta/Lee IV/9 (4).jpg]

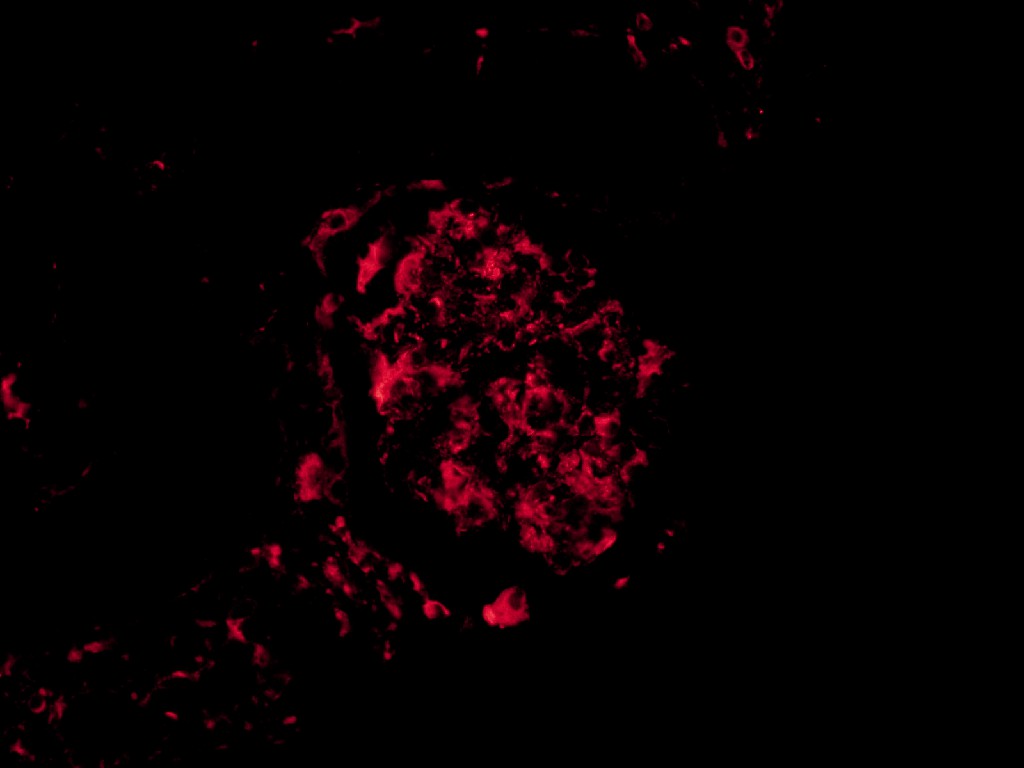

Supplement: Supplementary file 2 — Source data Fig. 1 [file 44321_2025_315_MOESM2_ESM.zip › Figure 1/F1A/1-GLDC-PDGFRbeta/Lee V/1 (1).jpg]

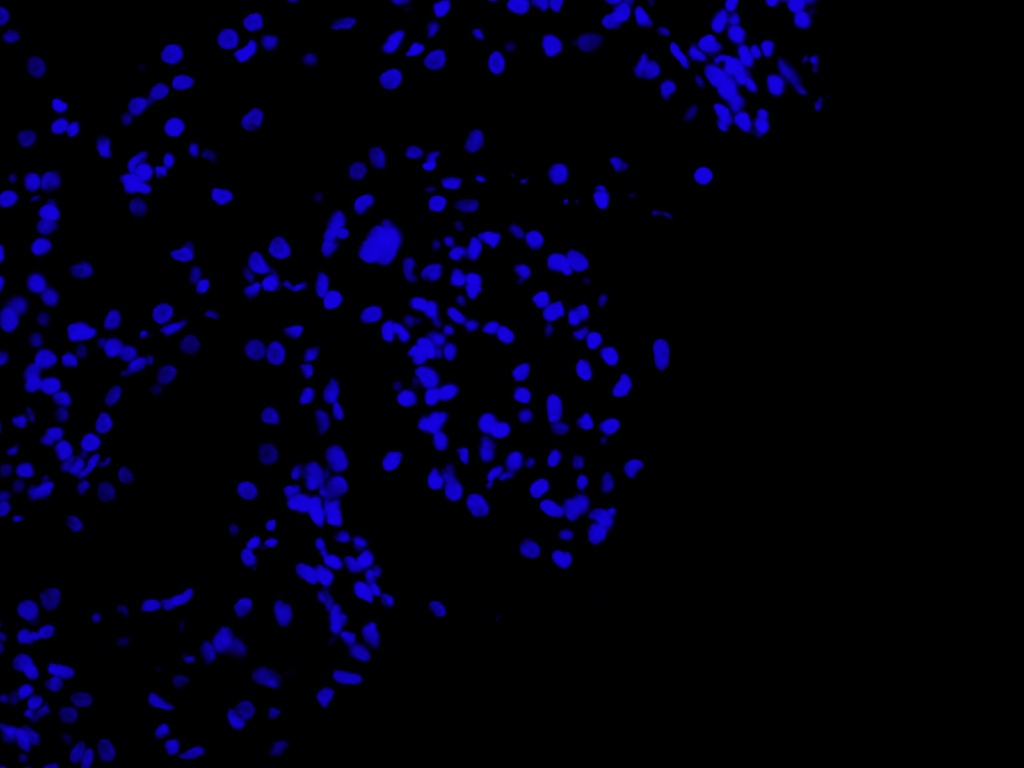

Supplement: Supplementary file 2 — Source data Fig. 1 [file 44321_2025_315_MOESM2_ESM.zip › Figure 1/F1A/1-GLDC-PDGFRbeta/Lee V/1 (2).jpg]

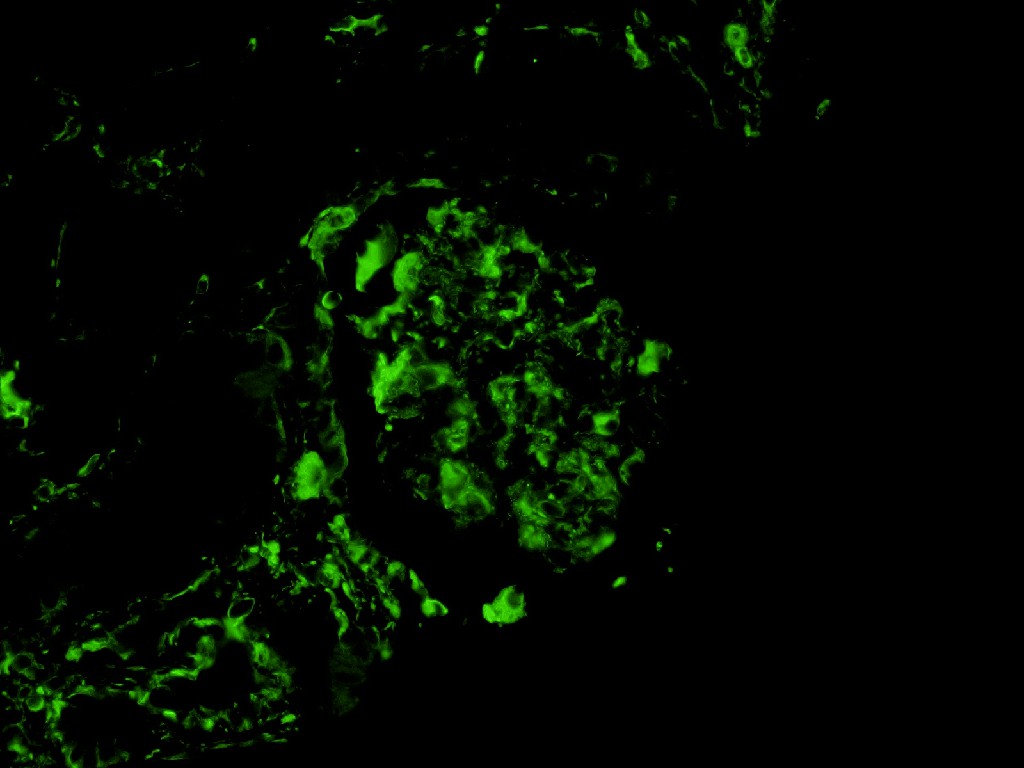

Supplement: Supplementary file 2 — Source data Fig. 1 [file 44321_2025_315_MOESM2_ESM.zip › Figure 1/F1A/1-GLDC-PDGFRbeta/Lee V/1 (3).jpg]

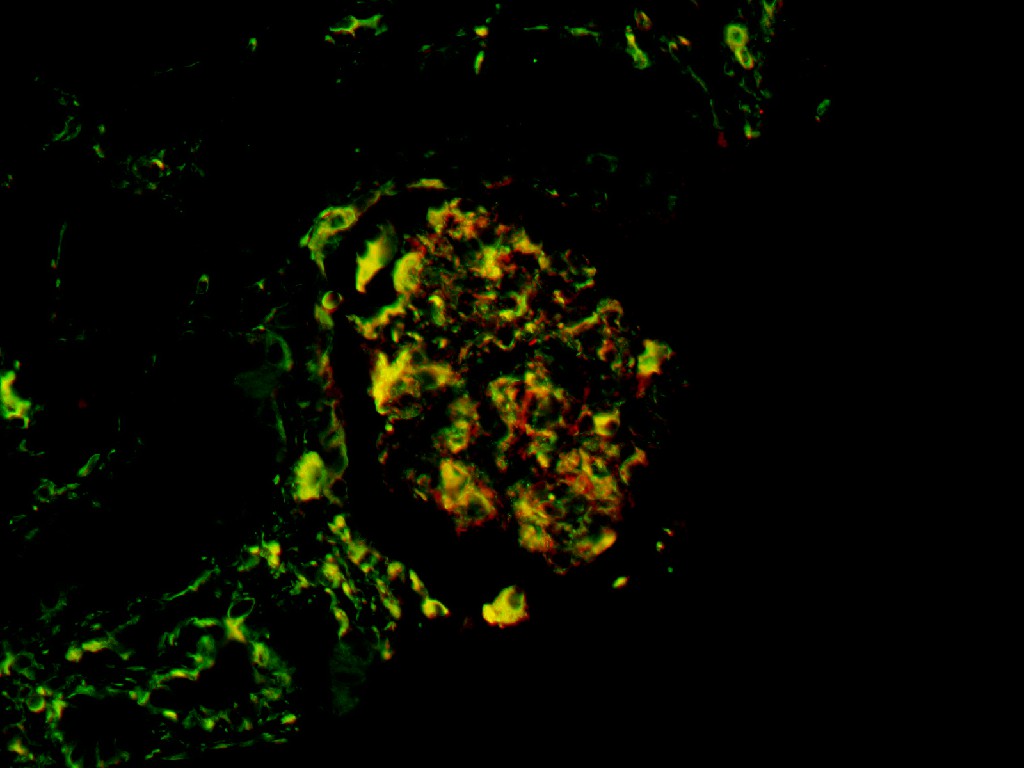

Supplement: Supplementary file 2 — Source data Fig. 1 [file 44321_2025_315_MOESM2_ESM.zip › Figure 1/F1A/1-GLDC-PDGFRbeta/Lee V/1 (4).jpg]

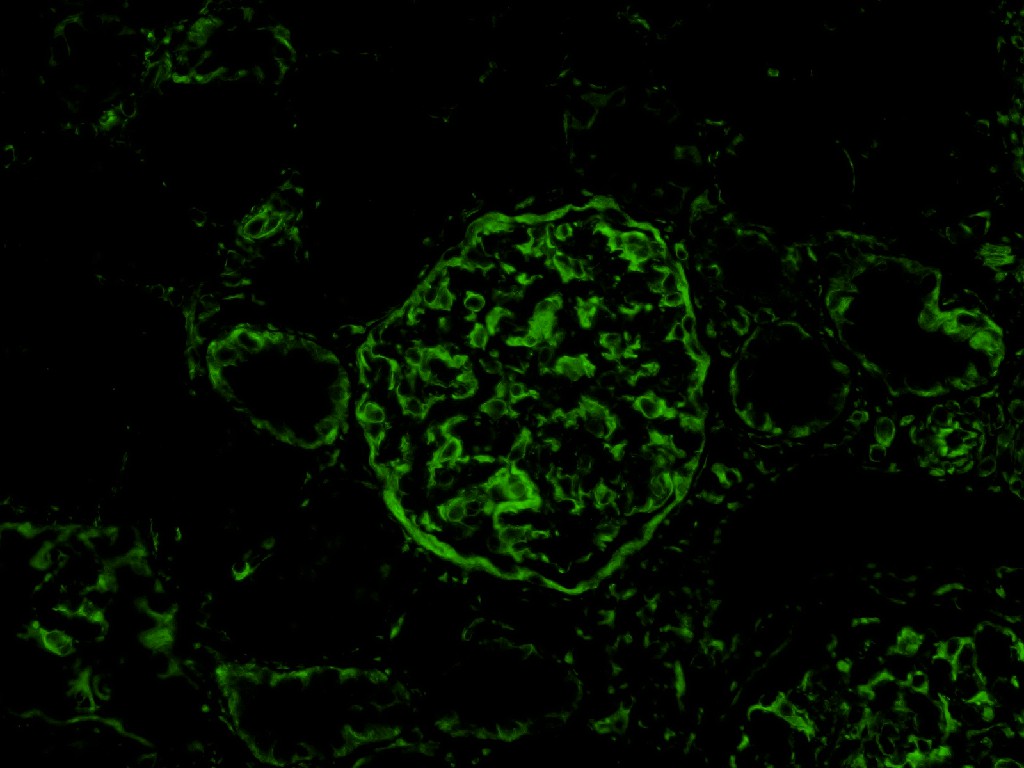

Supplement: Supplementary file 2 — Source data Fig. 1 [file 44321_2025_315_MOESM2_ESM.zip › Figure 1/F1A/1-GLDC-PDGFRbeta/Lee V/10 (1).jpg]

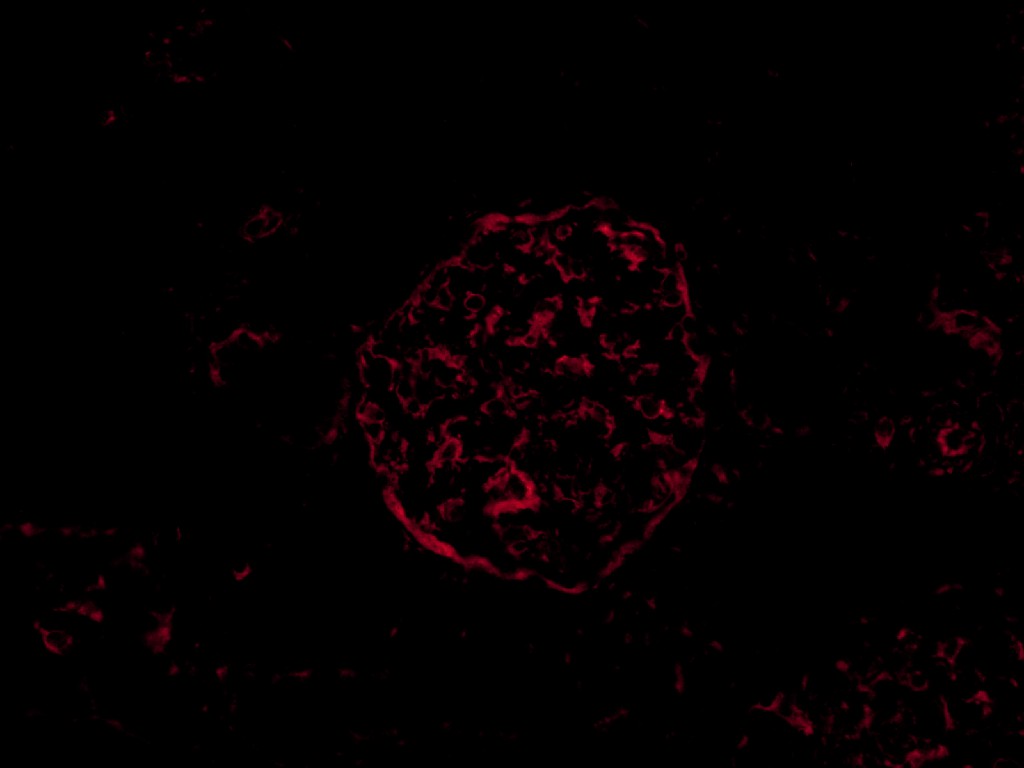

Supplement: Supplementary file 2 — Source data Fig. 1 [file 44321_2025_315_MOESM2_ESM.zip › Figure 1/F1A/1-GLDC-PDGFRbeta/Lee V/10 (2).jpg]

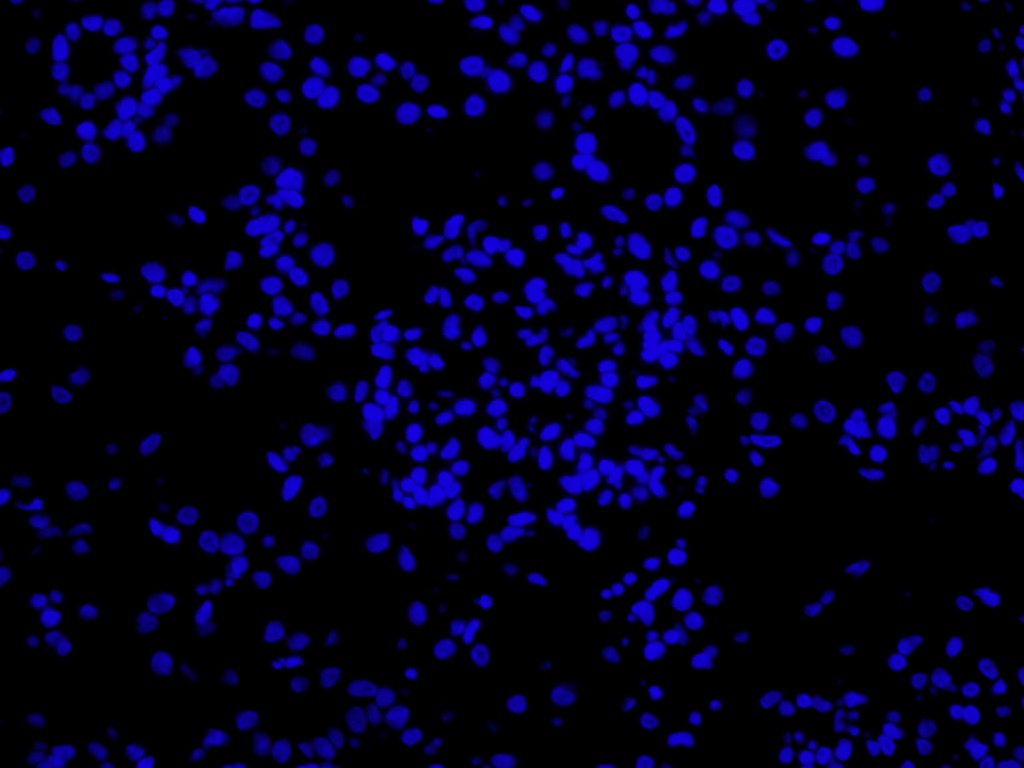

Supplement: Supplementary file 2 — Source data Fig. 1 [file 44321_2025_315_MOESM2_ESM.zip › Figure 1/F1A/1-GLDC-PDGFRbeta/Lee V/10 (3).jpg]

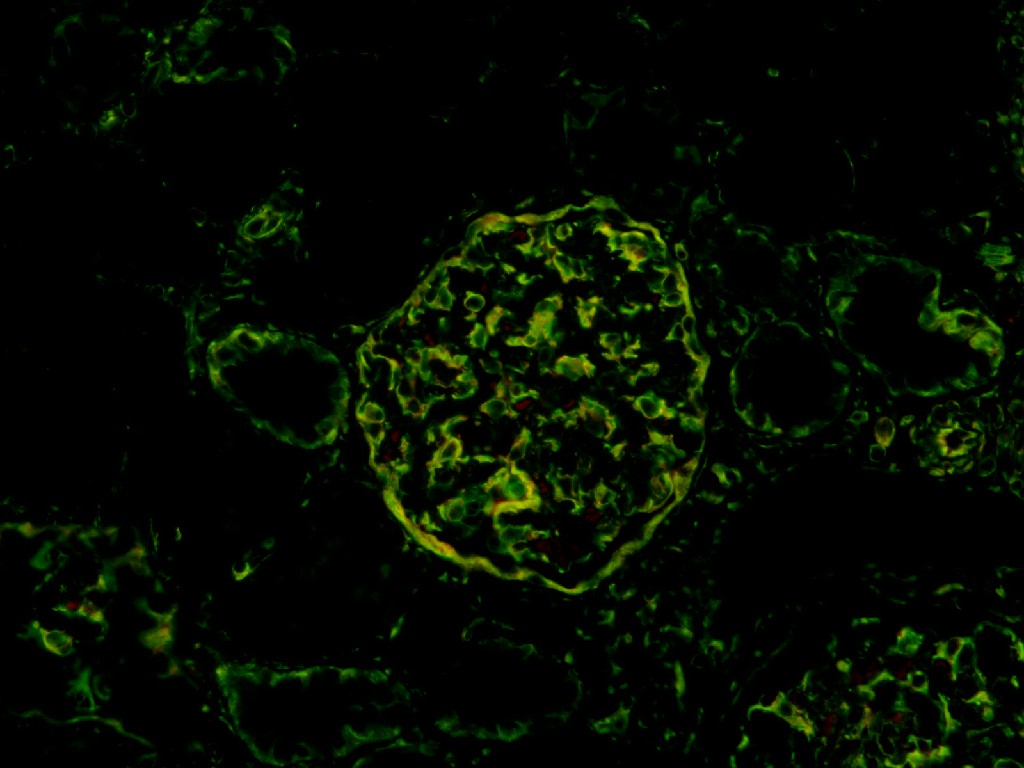

Supplement: Supplementary file 2 — Source data Fig. 1 [file 44321_2025_315_MOESM2_ESM.zip › Figure 1/F1A/1-GLDC-PDGFRbeta/Lee V/10 (4).jpg]

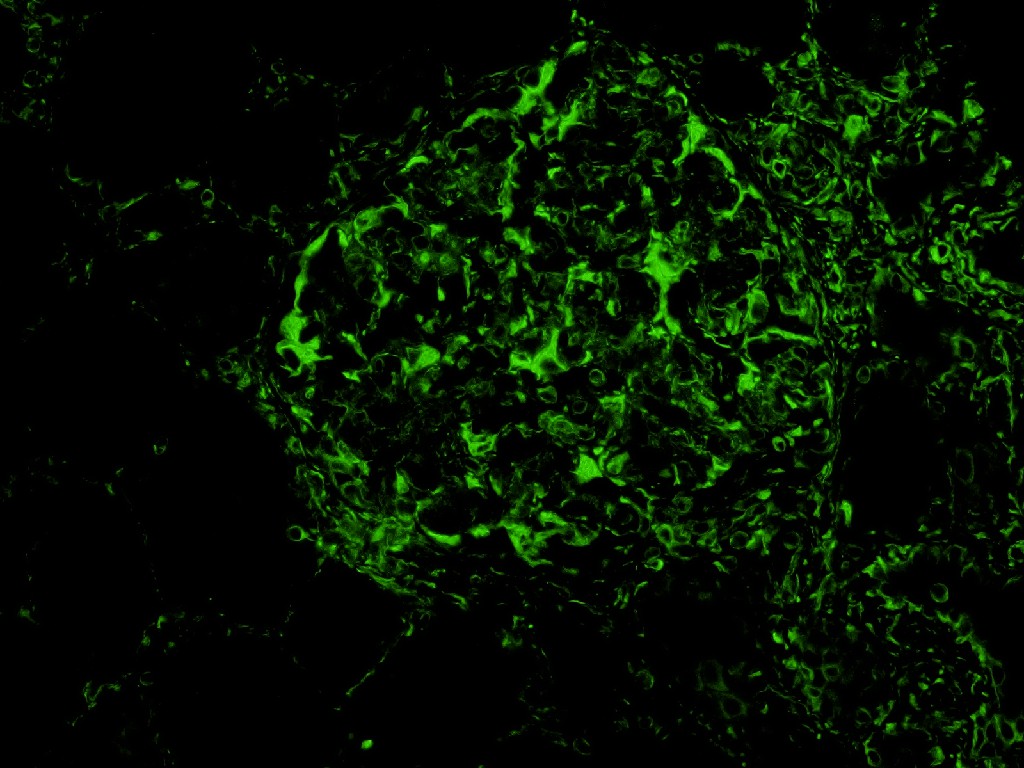

Supplement: Supplementary file 2 — Source data Fig. 1 [file 44321_2025_315_MOESM2_ESM.zip › Figure 1/F1A/1-GLDC-PDGFRbeta/Lee V/11 (1).jpg]

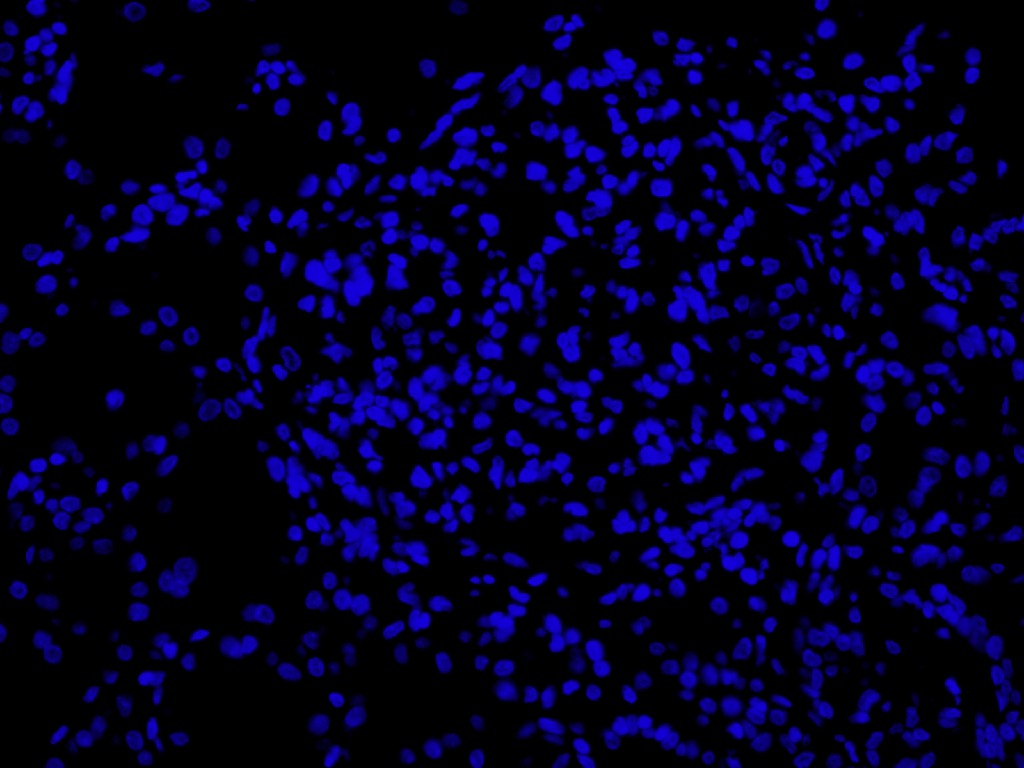

Supplement: Supplementary file 2 — Source data Fig. 1 [file 44321_2025_315_MOESM2_ESM.zip › Figure 1/F1A/1-GLDC-PDGFRbeta/Lee V/11 (2).jpg]

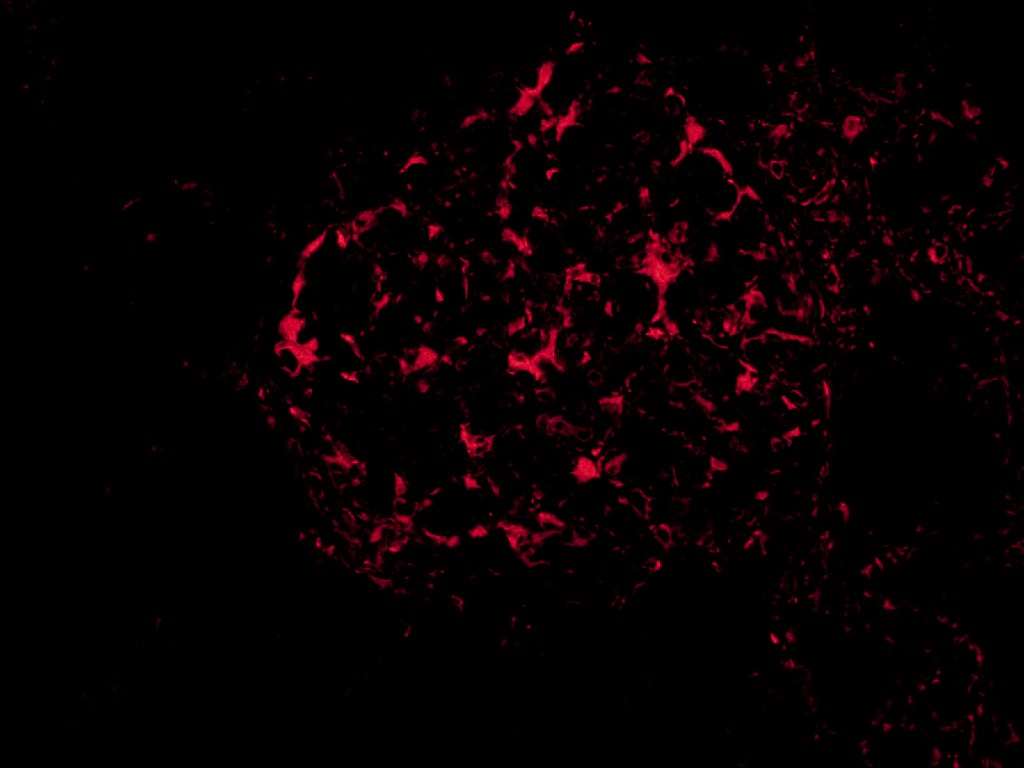

Supplement: Supplementary file 2 — Source data Fig. 1 [file 44321_2025_315_MOESM2_ESM.zip › Figure 1/F1A/1-GLDC-PDGFRbeta/Lee V/11 (3).jpg]

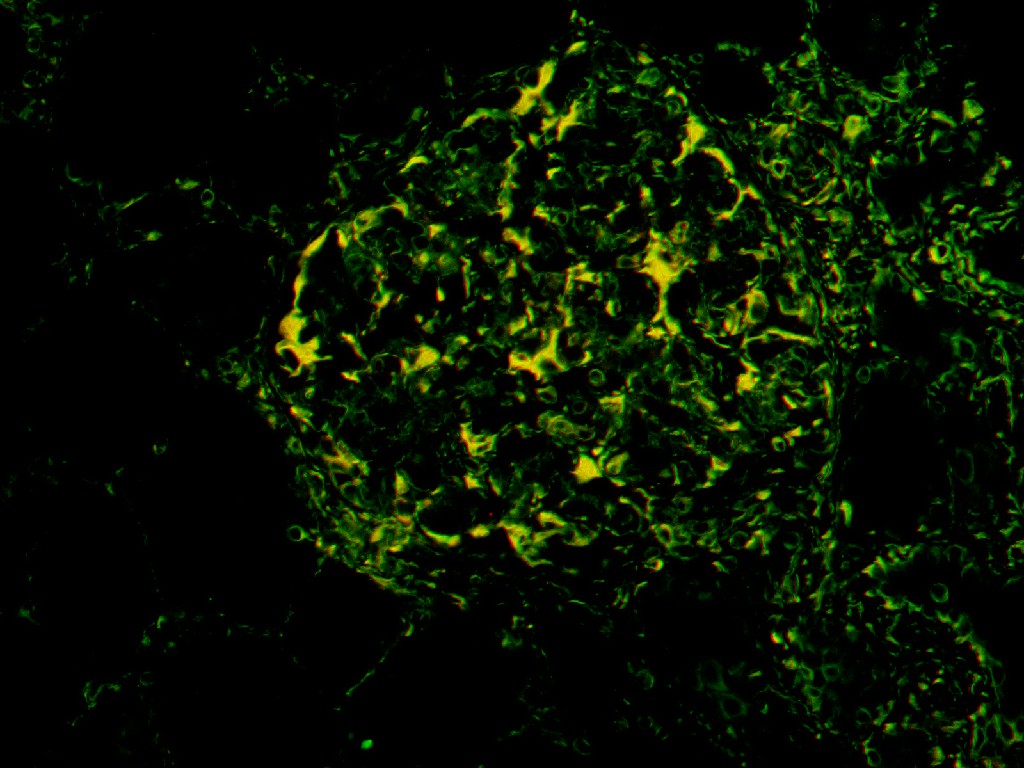

Supplement: Supplementary file 2 — Source data Fig. 1 [file 44321_2025_315_MOESM2_ESM.zip › Figure 1/F1A/1-GLDC-PDGFRbeta/Lee V/11 (4).jpg]

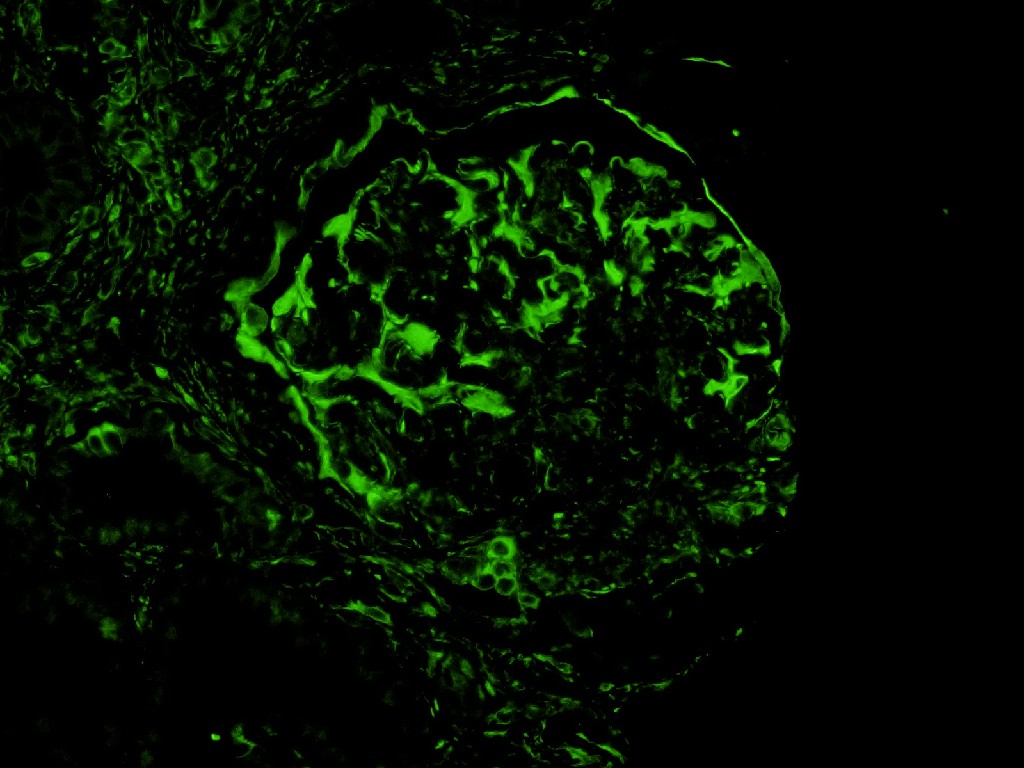

Supplement: Supplementary file 2 — Source data Fig. 1 [file 44321_2025_315_MOESM2_ESM.zip › Figure 1/F1A/1-GLDC-PDGFRbeta/Lee V/2 (1).jpg]

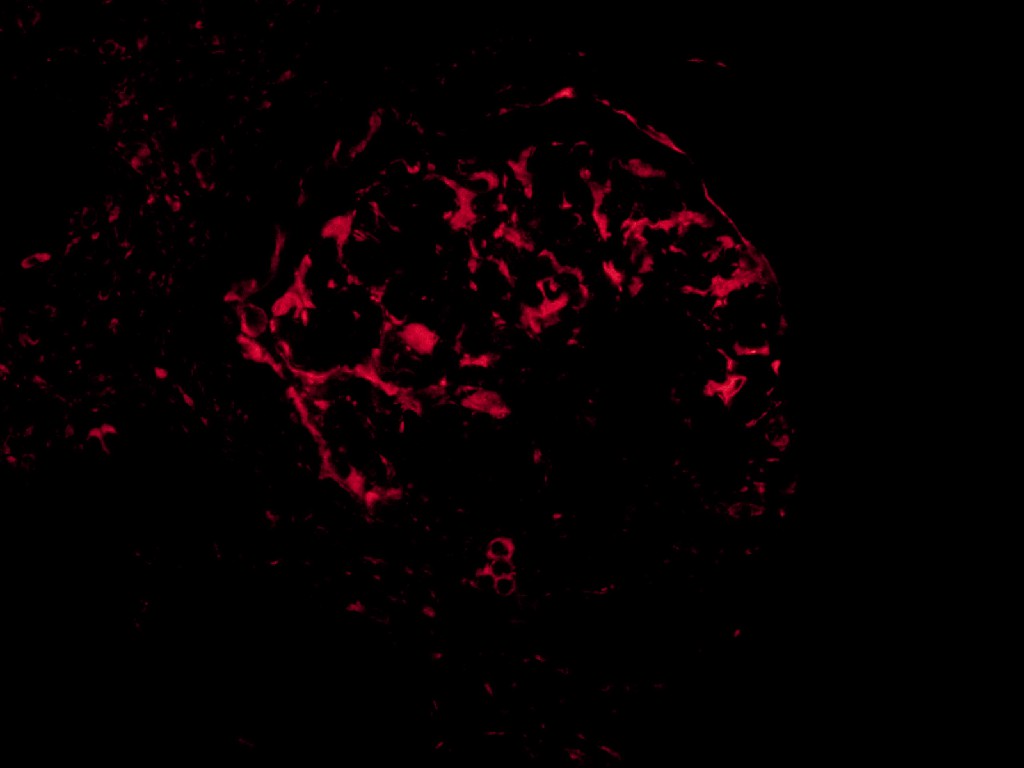

Supplement: Supplementary file 2 — Source data Fig. 1 [file 44321_2025_315_MOESM2_ESM.zip › Figure 1/F1A/1-GLDC-PDGFRbeta/Lee V/2 (2).jpg]

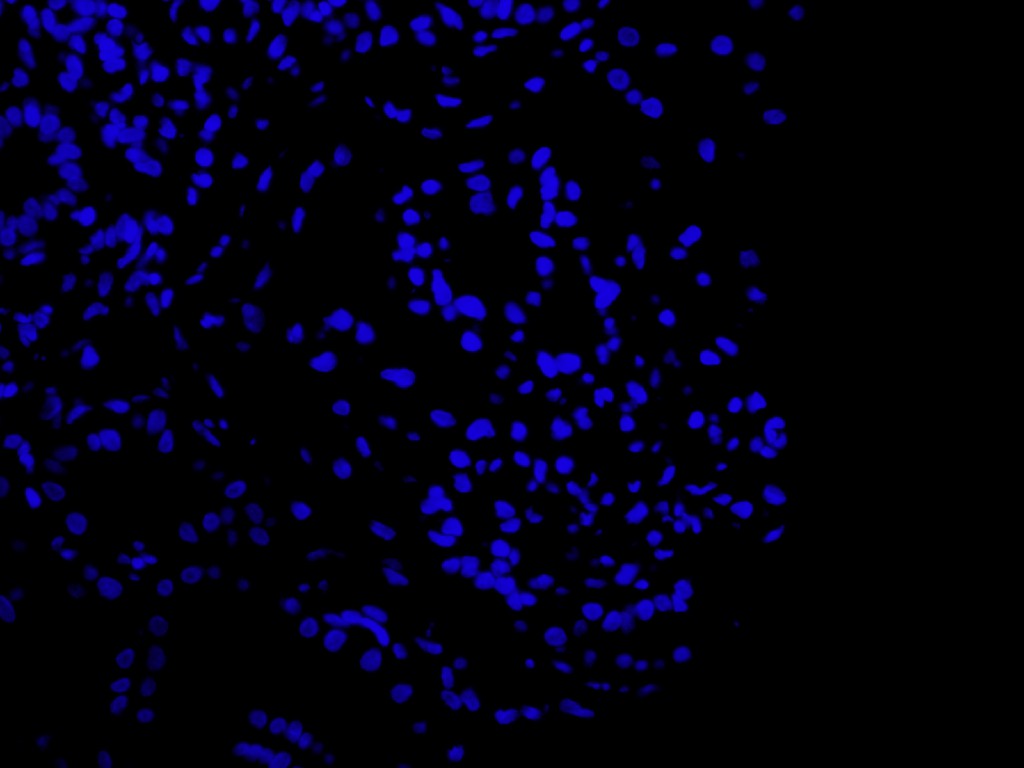

Supplement: Supplementary file 2 — Source data Fig. 1 [file 44321_2025_315_MOESM2_ESM.zip › Figure 1/F1A/1-GLDC-PDGFRbeta/Lee V/2 (3).jpg]

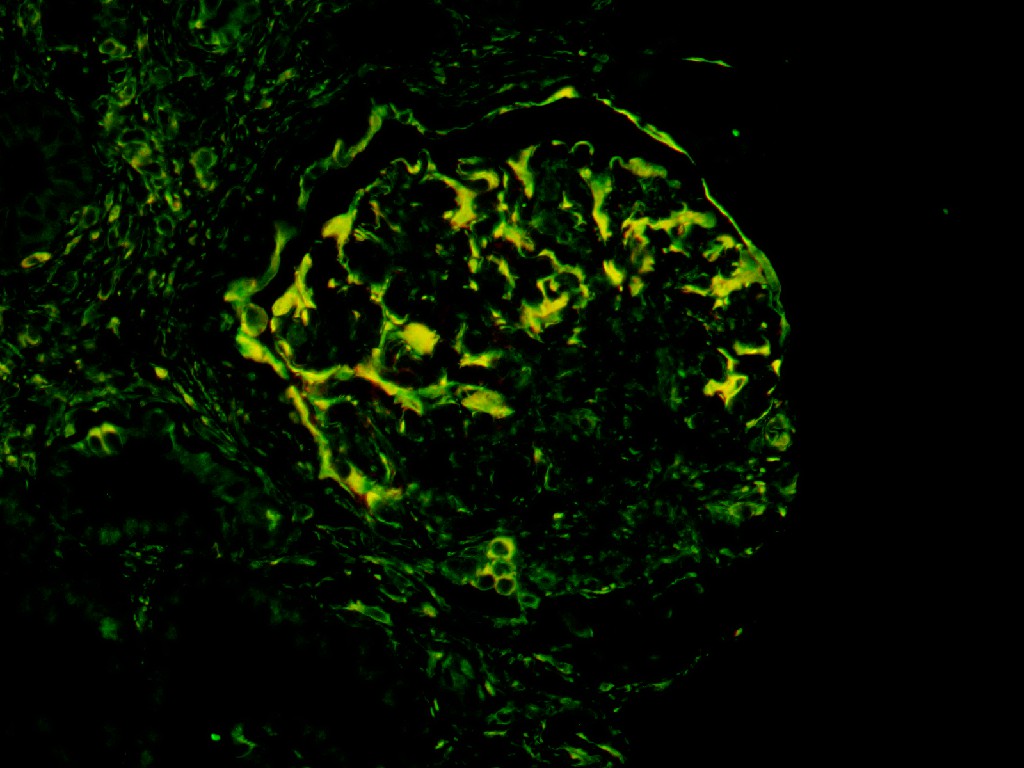

Supplement: Supplementary file 2 — Source data Fig. 1 [file 44321_2025_315_MOESM2_ESM.zip › Figure 1/F1A/1-GLDC-PDGFRbeta/Lee V/2 (4).jpg]

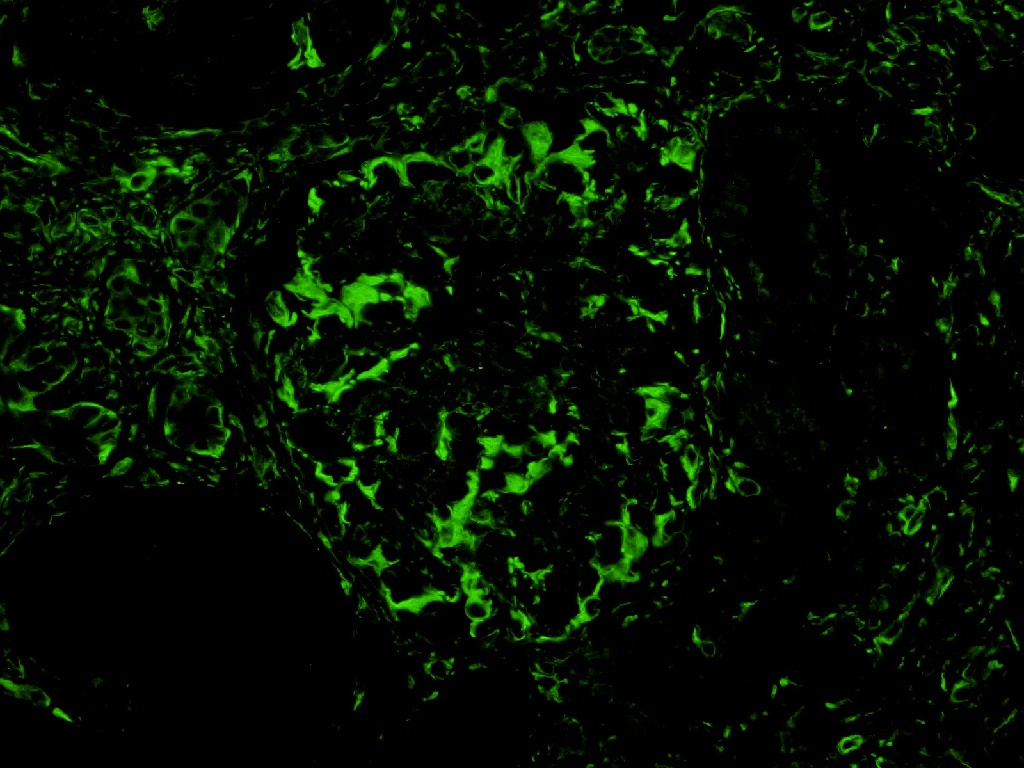

Supplement: Supplementary file 2 — Source data Fig. 1 [file 44321_2025_315_MOESM2_ESM.zip › Figure 1/F1A/1-GLDC-PDGFRbeta/Lee V/3 (1).jpg]

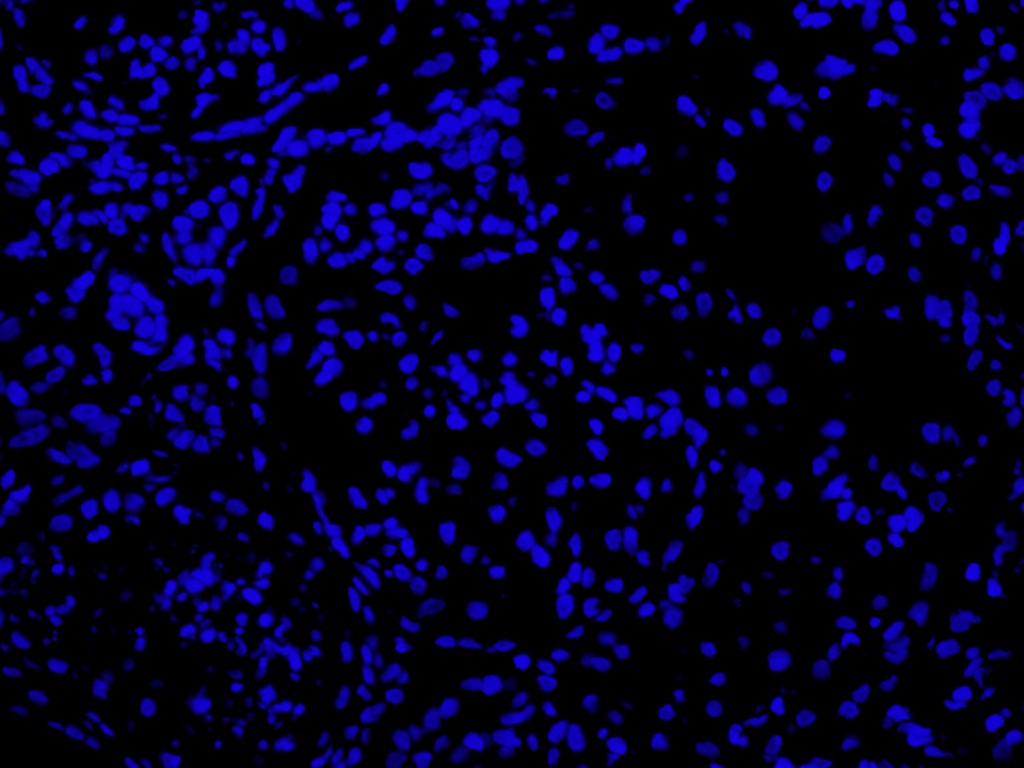

Supplement: Supplementary file 2 — Source data Fig. 1 [file 44321_2025_315_MOESM2_ESM.zip › Figure 1/F1A/1-GLDC-PDGFRbeta/Lee V/3 (2).jpg]

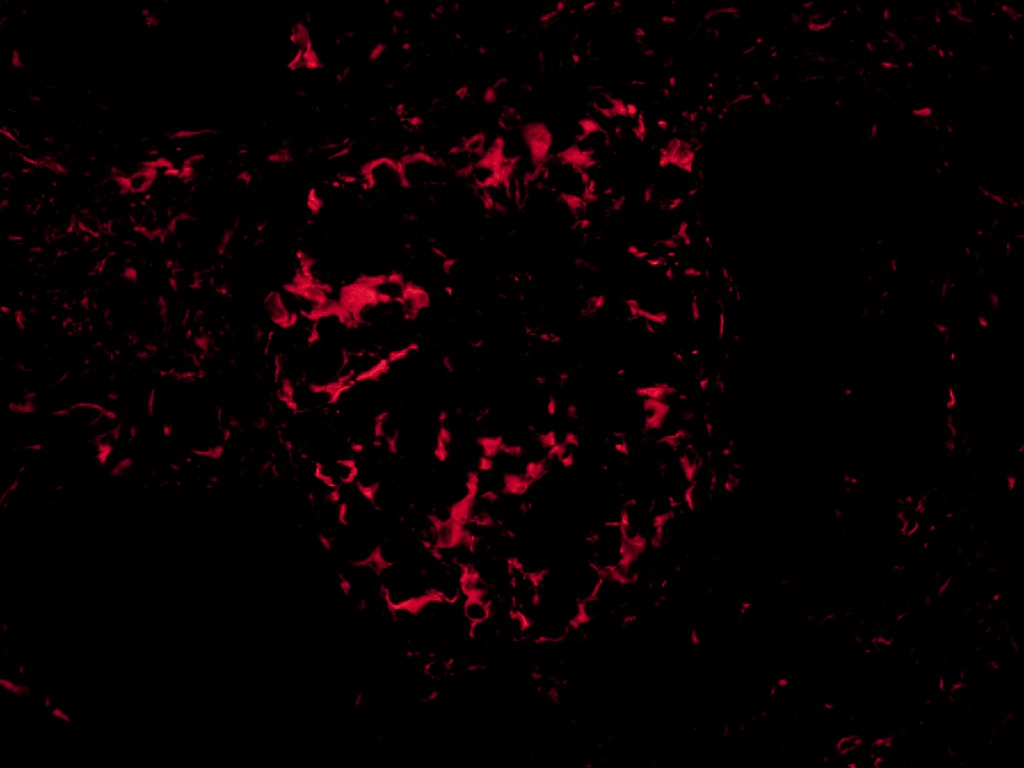

Supplement: Supplementary file 2 — Source data Fig. 1 [file 44321_2025_315_MOESM2_ESM.zip › Figure 1/F1A/1-GLDC-PDGFRbeta/Lee V/3 (3).jpg]

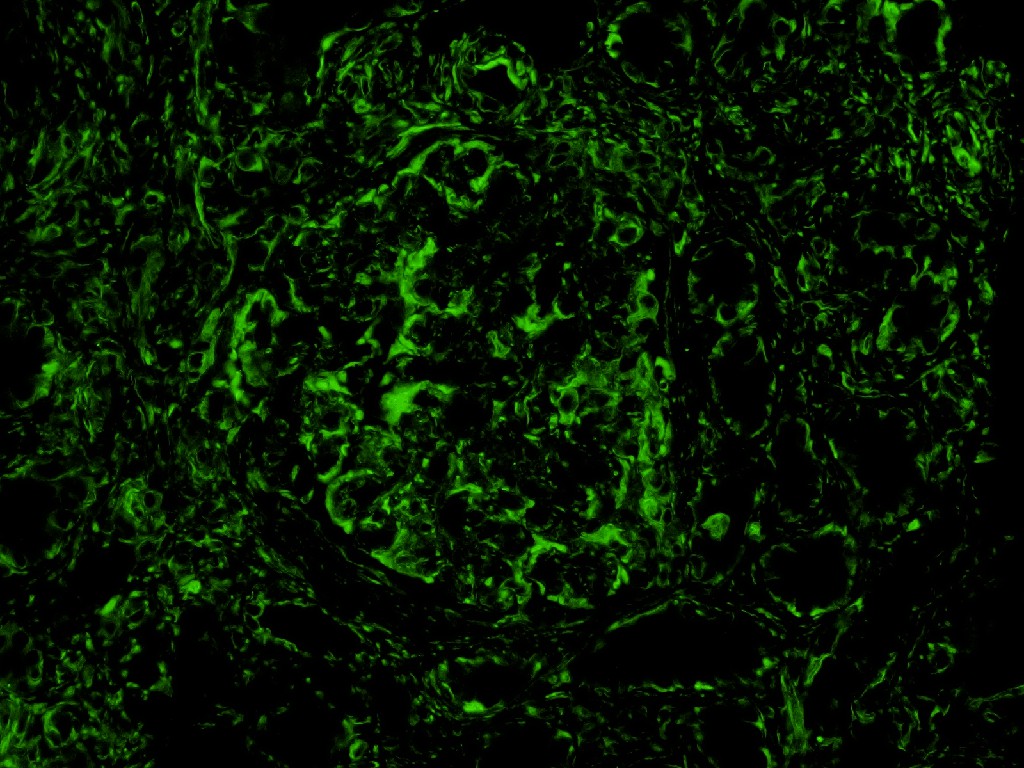

Supplement: Supplementary file 2 — Source data Fig. 1 [file 44321_2025_315_MOESM2_ESM.zip › Figure 1/F1A/1-GLDC-PDGFRbeta/Lee V/4 (1).jpg]

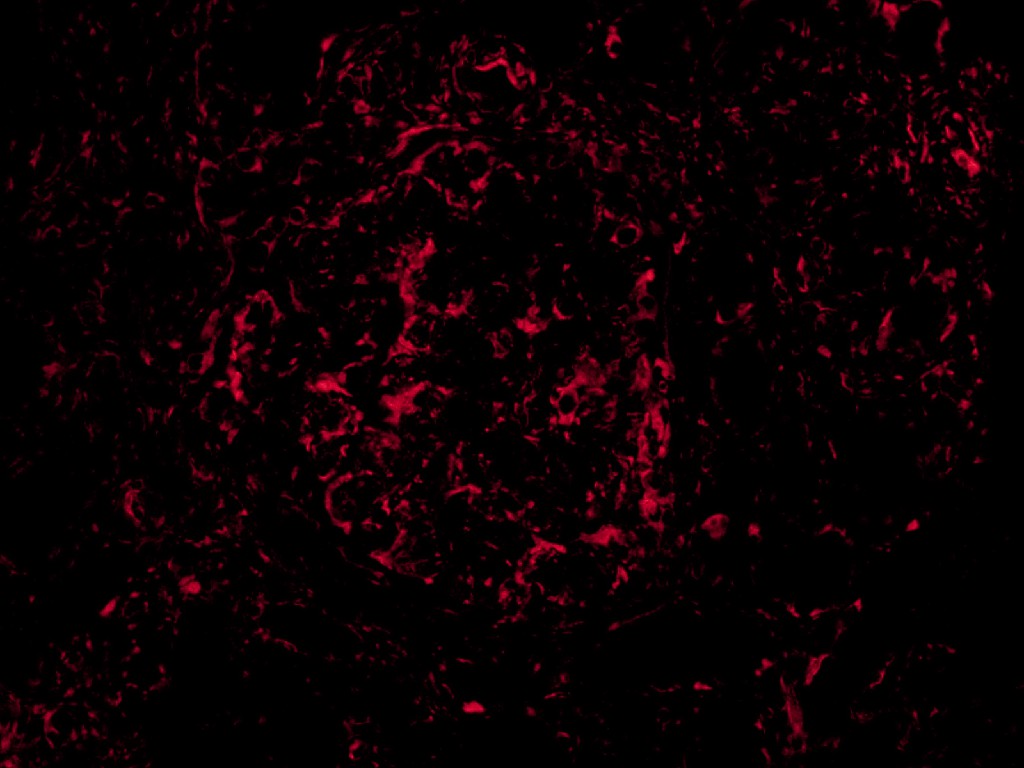

Supplement: Supplementary file 2 — Source data Fig. 1 [file 44321_2025_315_MOESM2_ESM.zip › Figure 1/F1A/1-GLDC-PDGFRbeta/Lee V/4 (2).jpg]

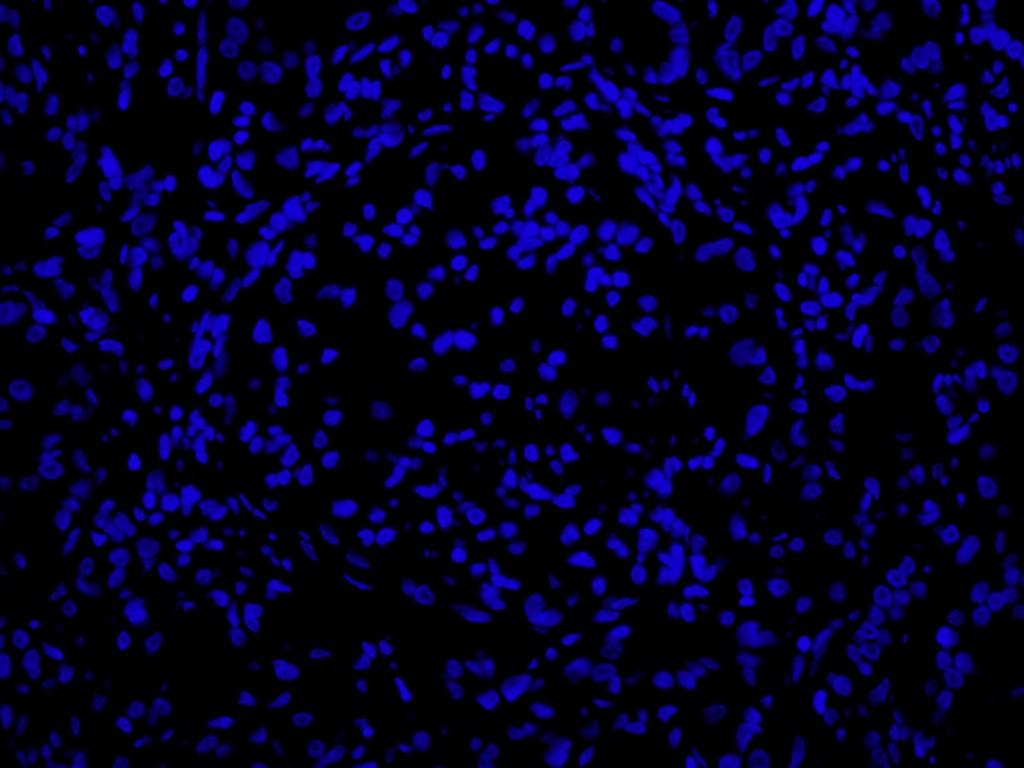

Supplement: Supplementary file 2 — Source data Fig. 1 [file 44321_2025_315_MOESM2_ESM.zip › Figure 1/F1A/1-GLDC-PDGFRbeta/Lee V/4 (3).jpg]

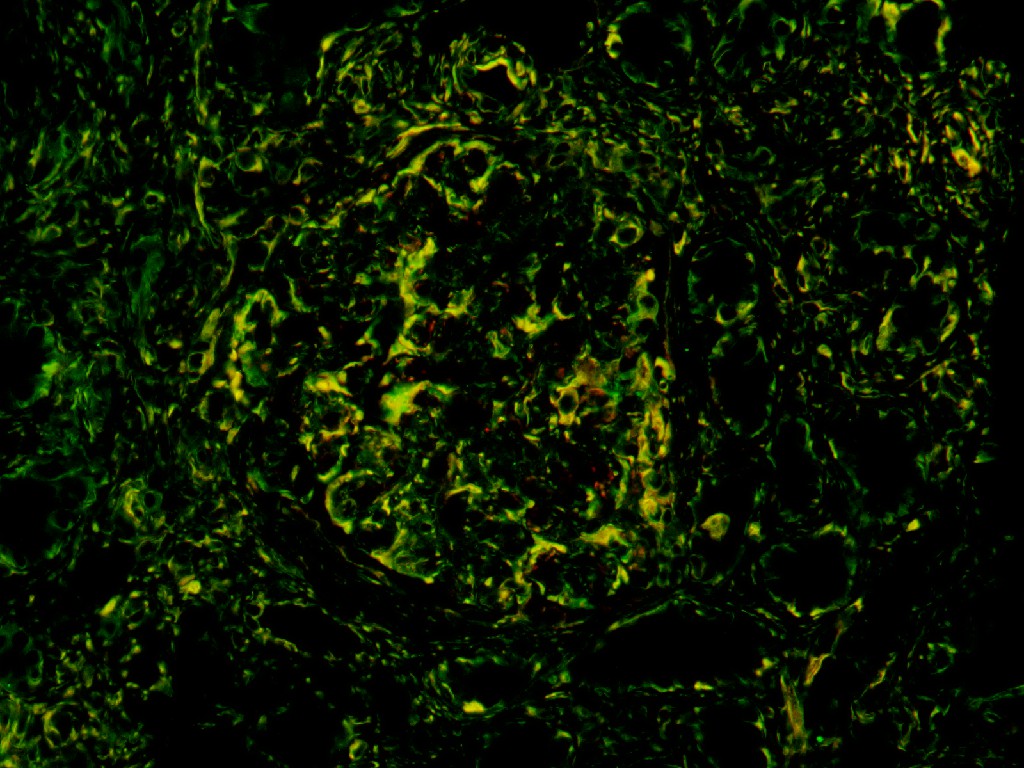

Supplement: Supplementary file 2 — Source data Fig. 1 [file 44321_2025_315_MOESM2_ESM.zip › Figure 1/F1A/1-GLDC-PDGFRbeta/Lee V/4 (4).jpg]

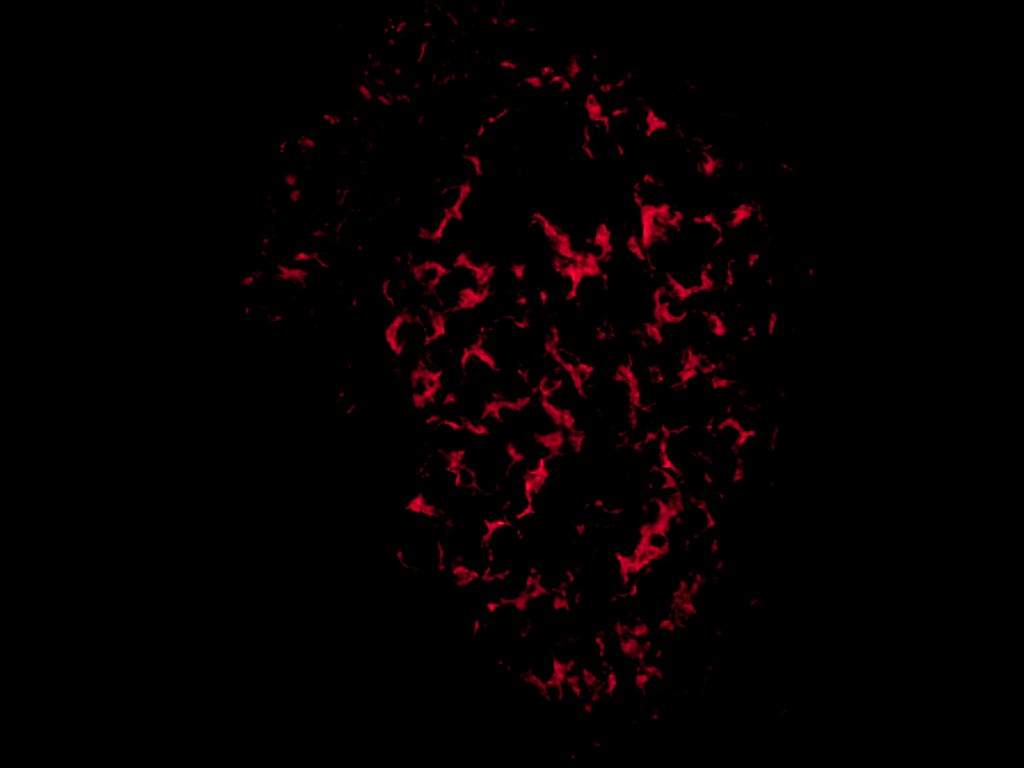

Supplement: Supplementary file 2 — Source data Fig. 1 [file 44321_2025_315_MOESM2_ESM.zip › Figure 1/F1A/1-GLDC-PDGFRbeta/Lee V/5 (1).jpg]

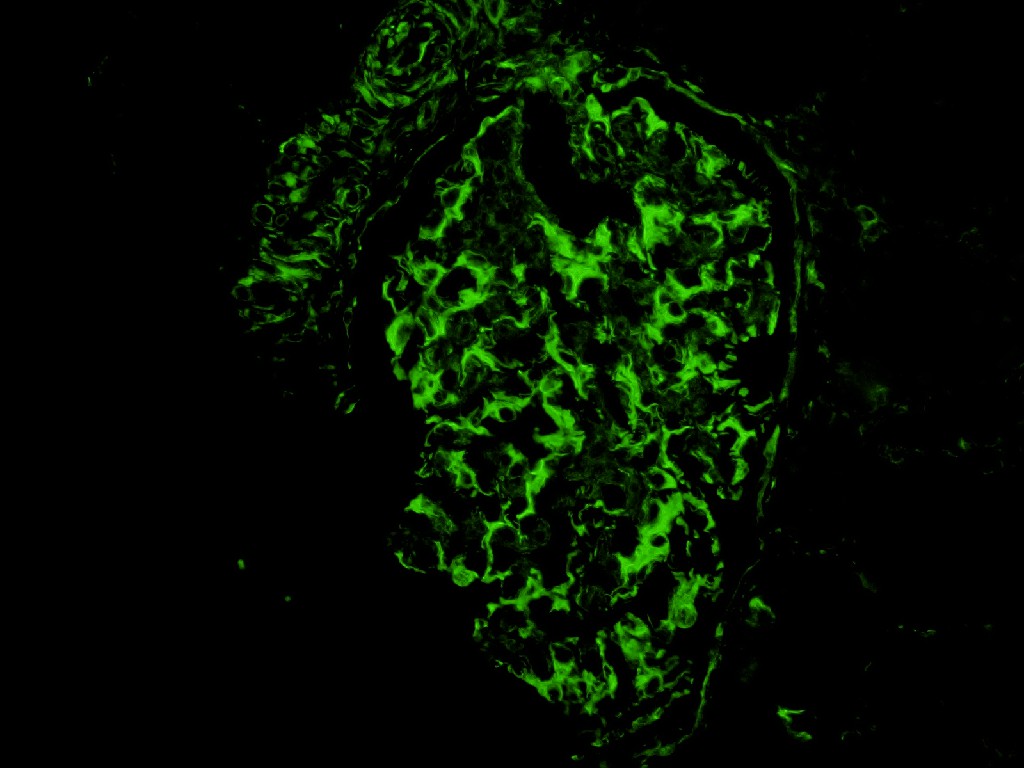

Supplement: Supplementary file 2 — Source data Fig. 1 [file 44321_2025_315_MOESM2_ESM.zip › Figure 1/F1A/1-GLDC-PDGFRbeta/Lee V/5 (2).jpg]

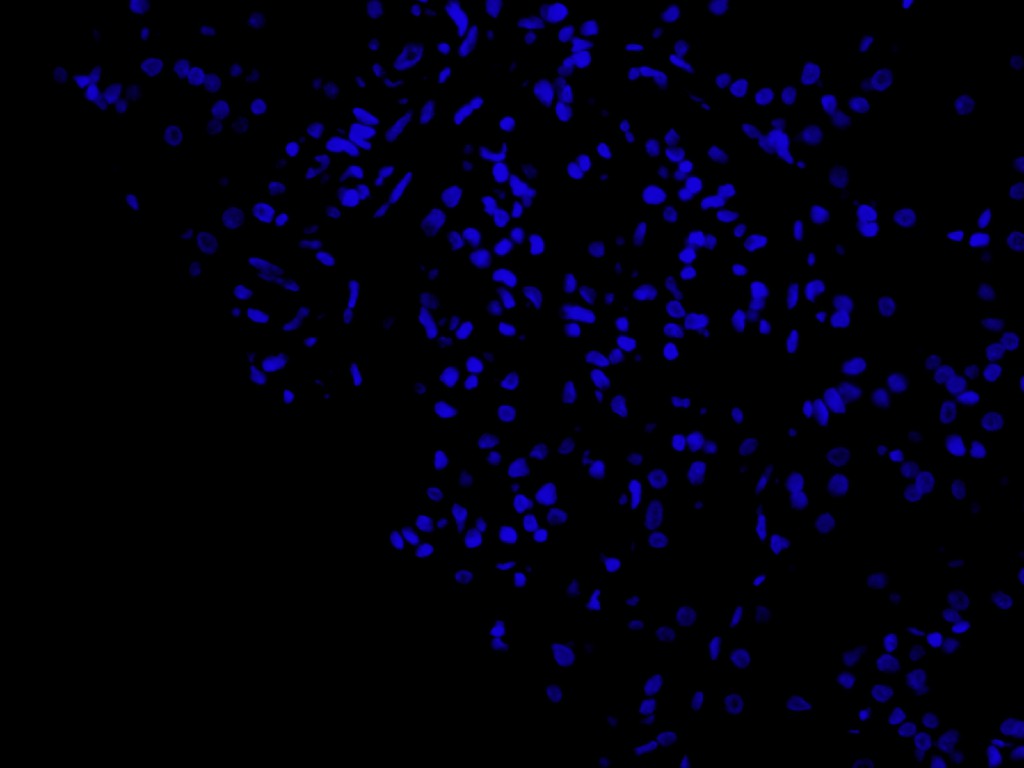

Supplement: Supplementary file 2 — Source data Fig. 1 [file 44321_2025_315_MOESM2_ESM.zip › Figure 1/F1A/1-GLDC-PDGFRbeta/Lee V/5 (3).jpg]

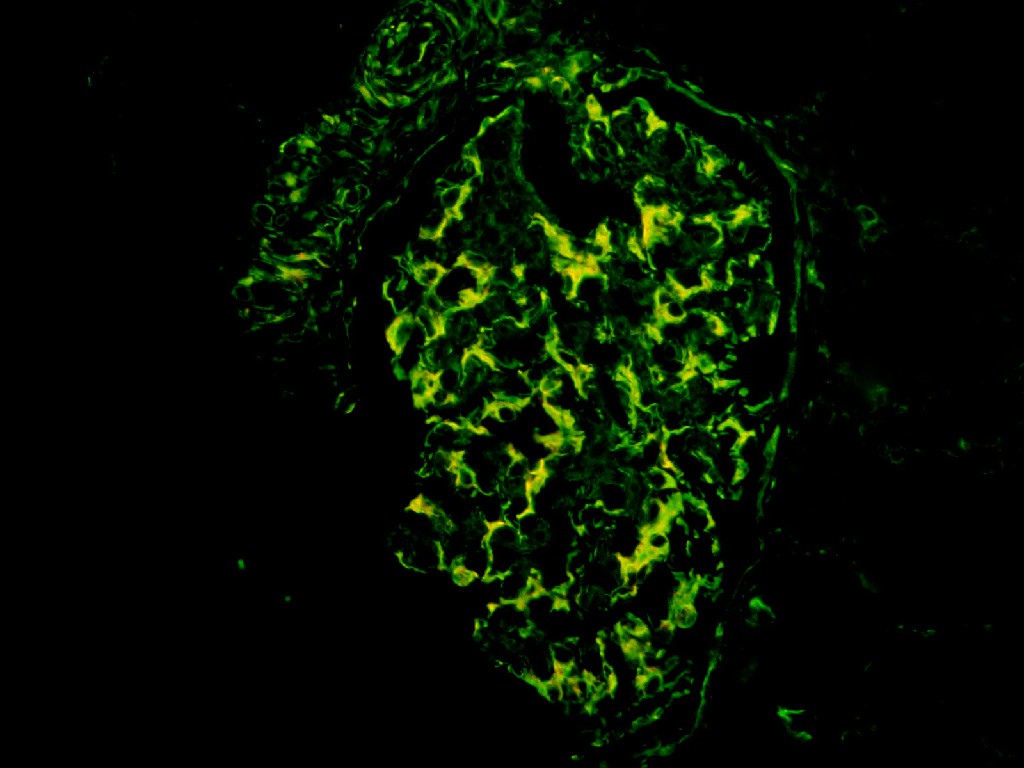

Supplement: Supplementary file 2 — Source data Fig. 1 [file 44321_2025_315_MOESM2_ESM.zip › Figure 1/F1A/1-GLDC-PDGFRbeta/Lee V/5 (4).jpg]

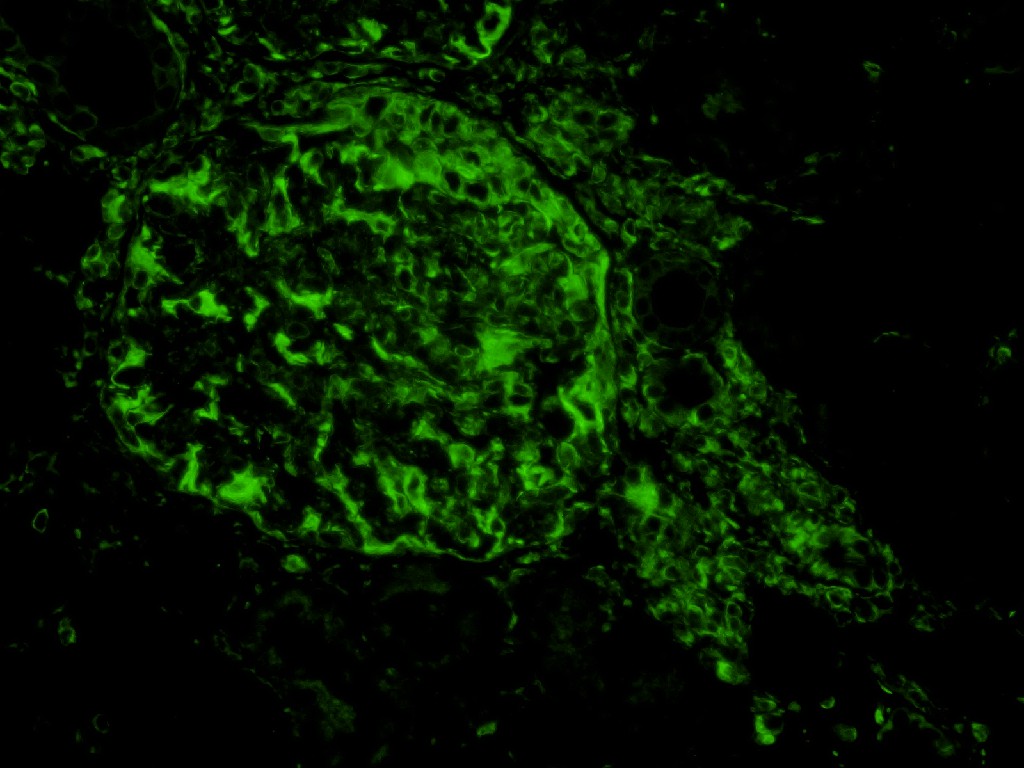

Supplement: Supplementary file 2 — Source data Fig. 1 [file 44321_2025_315_MOESM2_ESM.zip › Figure 1/F1A/1-GLDC-PDGFRbeta/Lee V/6 (1).jpg]

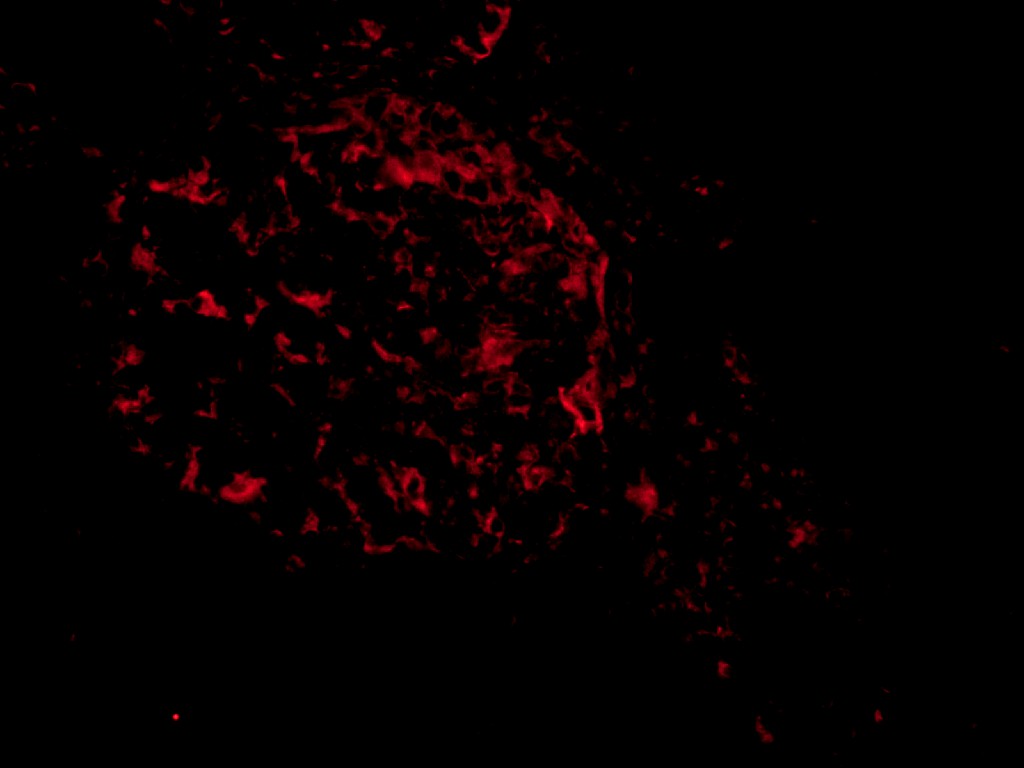

Supplement: Supplementary file 2 — Source data Fig. 1 [file 44321_2025_315_MOESM2_ESM.zip › Figure 1/F1A/1-GLDC-PDGFRbeta/Lee V/6 (2).jpg]

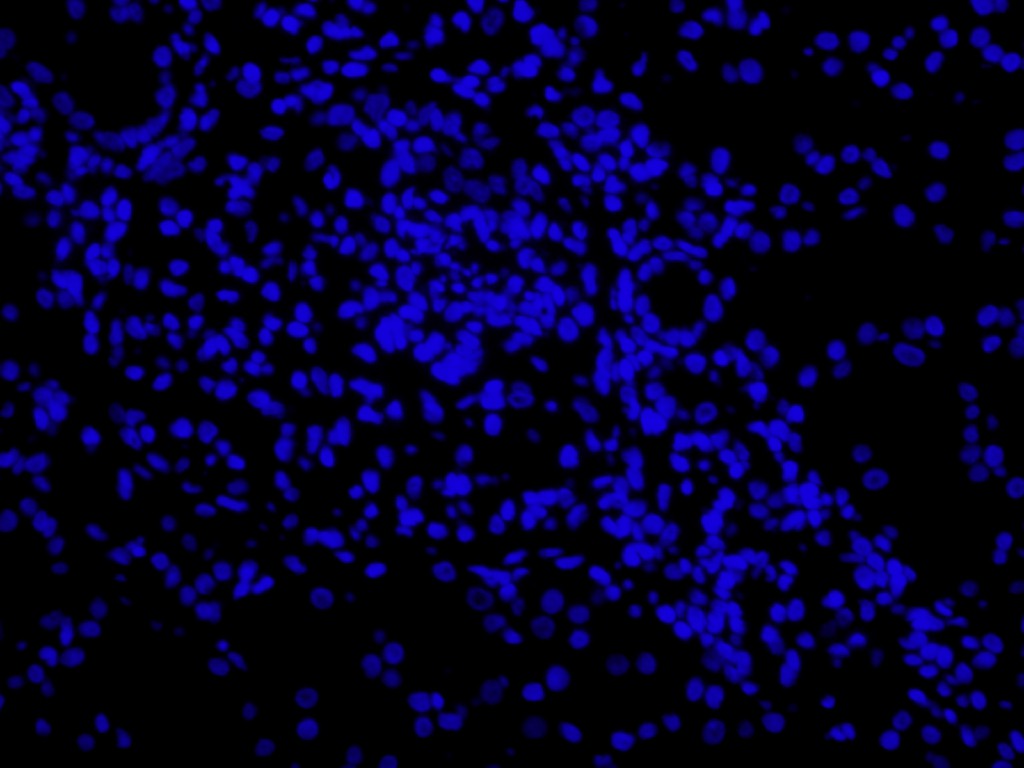

Supplement: Supplementary file 2 — Source data Fig. 1 [file 44321_2025_315_MOESM2_ESM.zip › Figure 1/F1A/1-GLDC-PDGFRbeta/Lee V/6 (3).jpg]

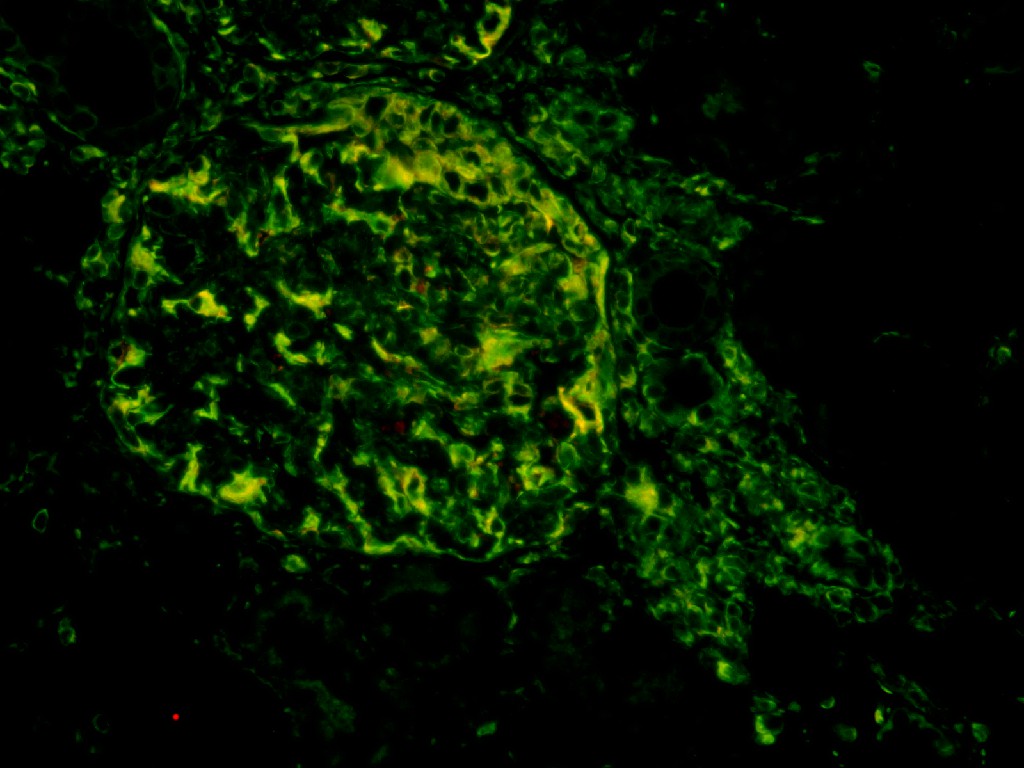

Supplement: Supplementary file 2 — Source data Fig. 1 [file 44321_2025_315_MOESM2_ESM.zip › Figure 1/F1A/1-GLDC-PDGFRbeta/Lee V/6 (4).jpg]

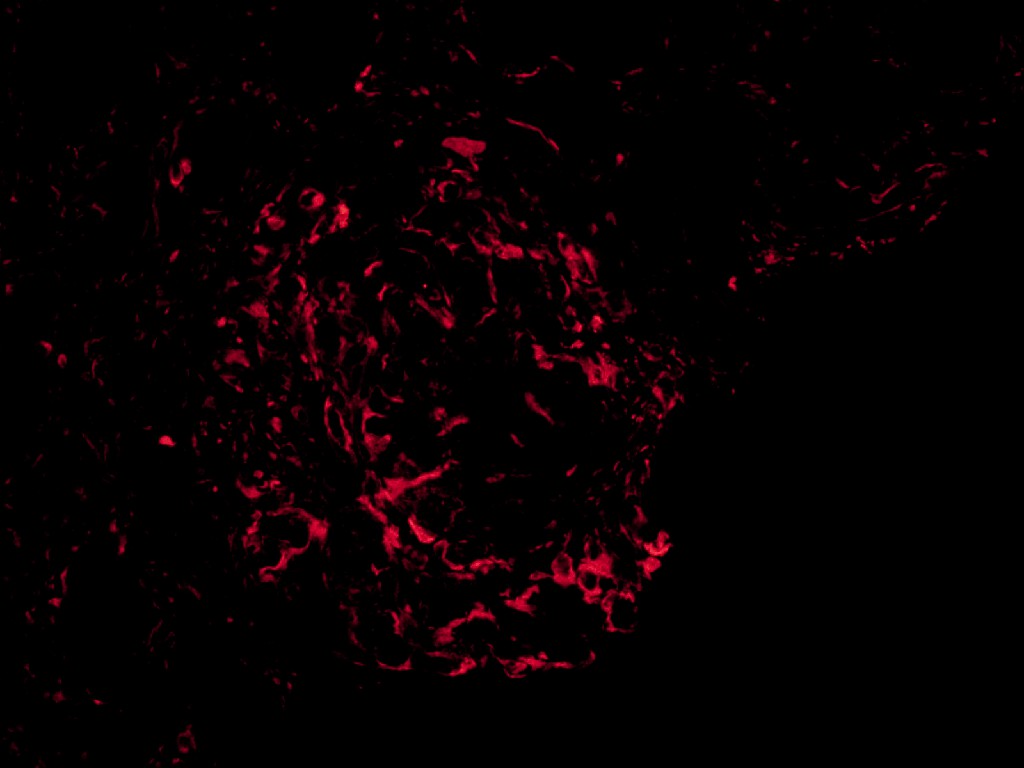

Supplement: Supplementary file 2 — Source data Fig. 1 [file 44321_2025_315_MOESM2_ESM.zip › Figure 1/F1A/1-GLDC-PDGFRbeta/Lee V/7 (1).jpg]

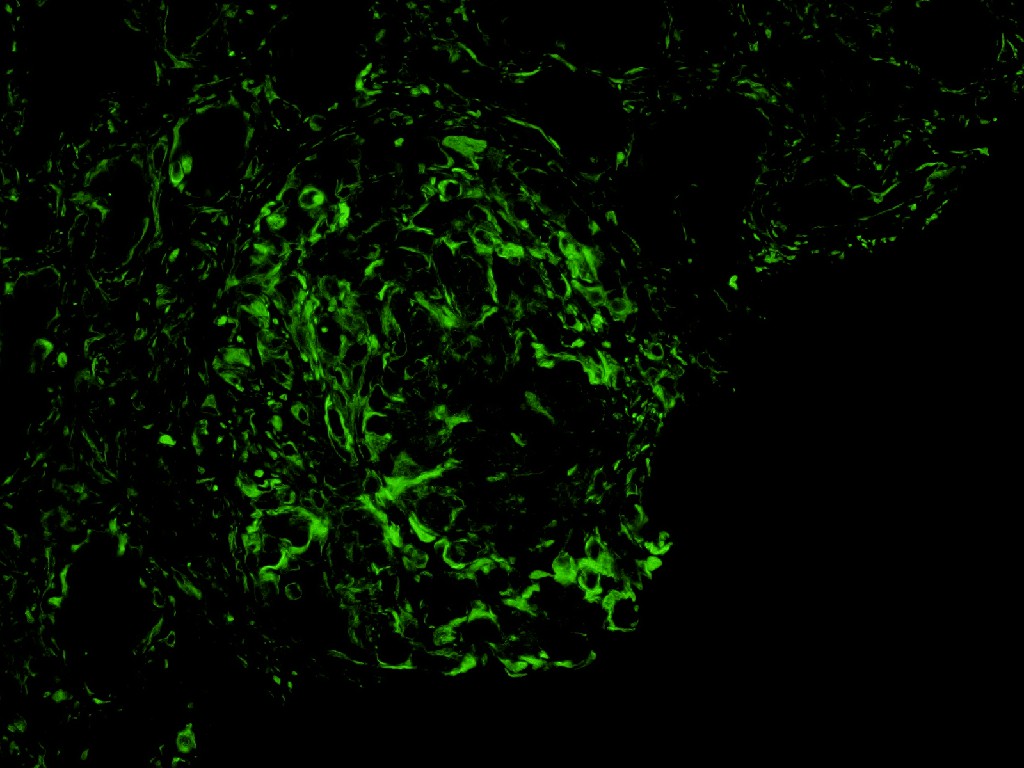

Supplement: Supplementary file 2 — Source data Fig. 1 [file 44321_2025_315_MOESM2_ESM.zip › Figure 1/F1A/1-GLDC-PDGFRbeta/Lee V/7 (2).jpg]

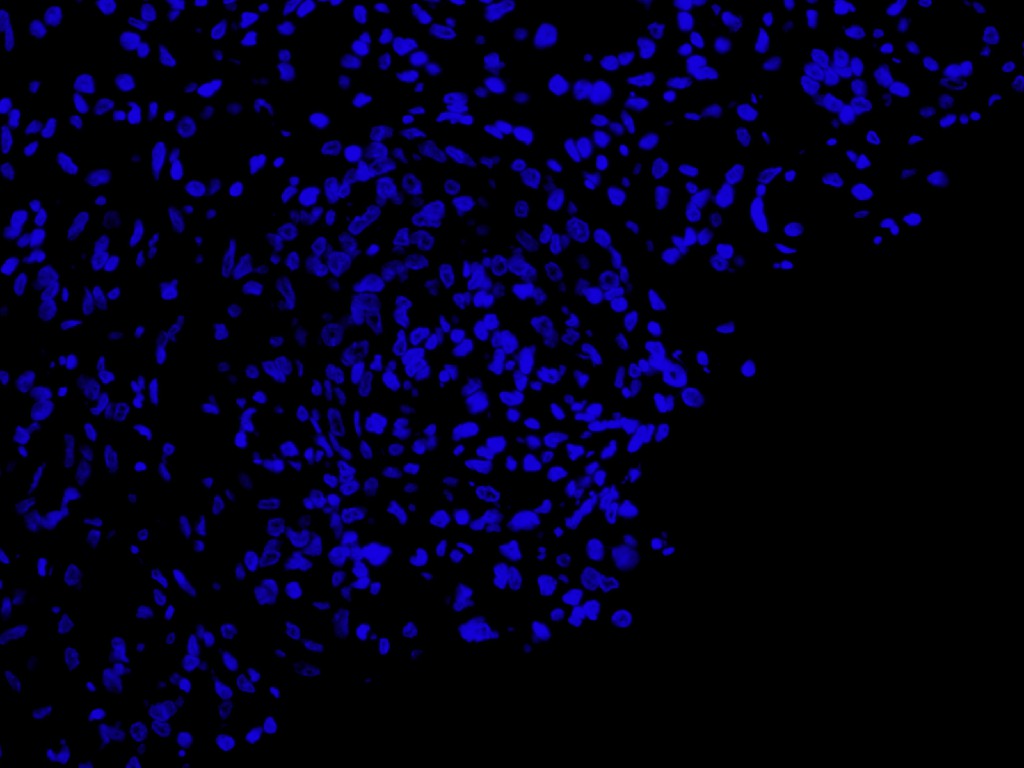

Supplement: Supplementary file 2 — Source data Fig. 1 [file 44321_2025_315_MOESM2_ESM.zip › Figure 1/F1A/1-GLDC-PDGFRbeta/Lee V/7 (3).jpg]

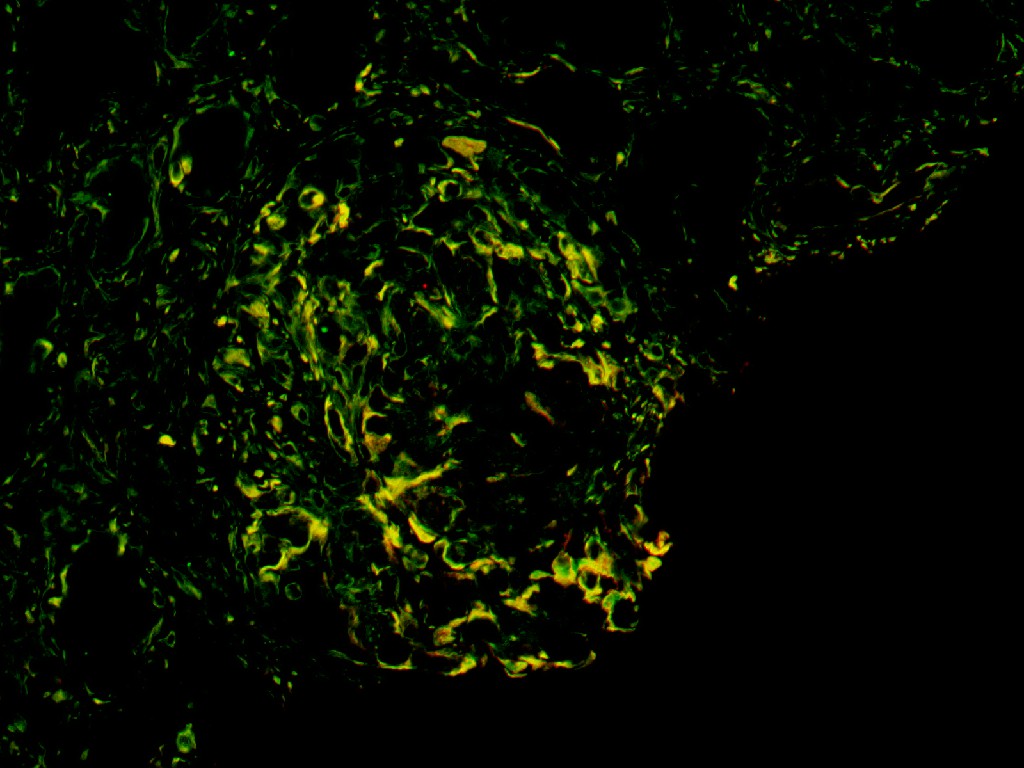

Supplement: Supplementary file 2 — Source data Fig. 1 [file 44321_2025_315_MOESM2_ESM.zip › Figure 1/F1A/1-GLDC-PDGFRbeta/Lee V/7 (4).jpg]

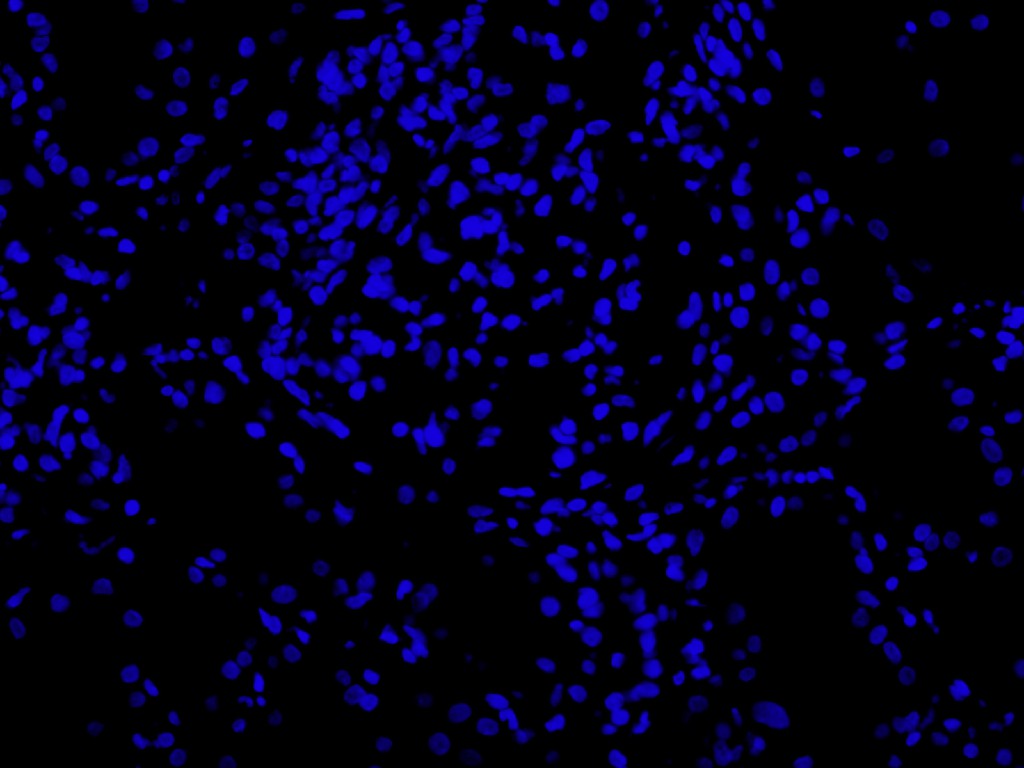

Supplement: Supplementary file 2 — Source data Fig. 1 [file 44321_2025_315_MOESM2_ESM.zip › Figure 1/F1A/1-GLDC-PDGFRbeta/Lee V/8 (1).jpg]

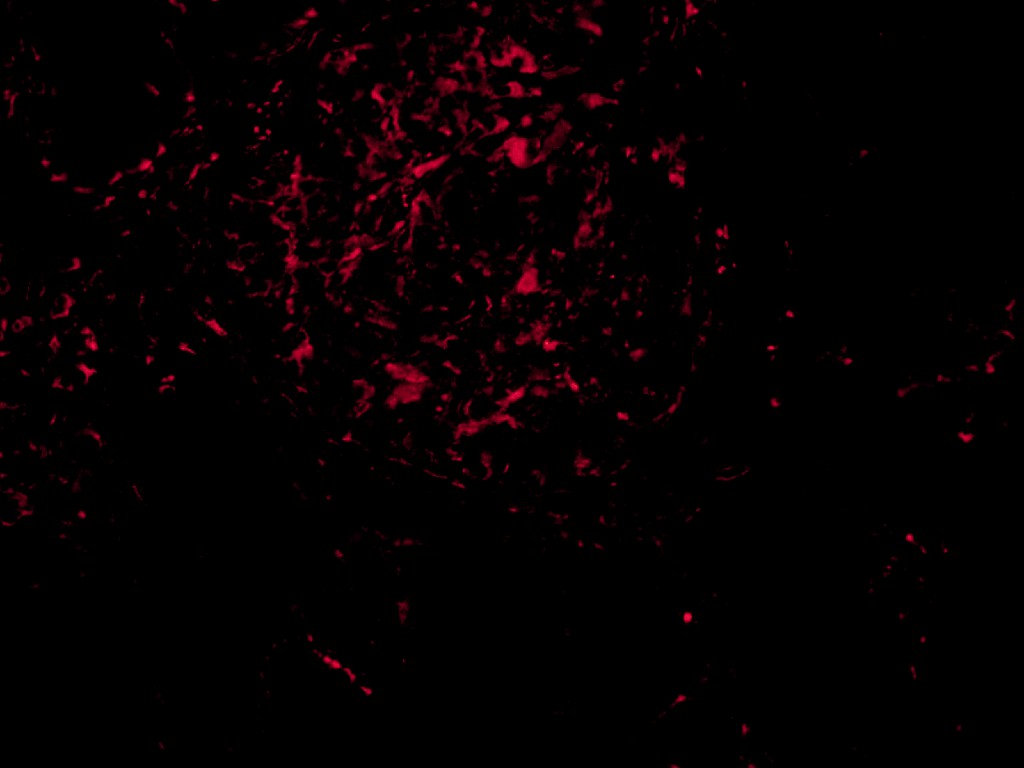

Supplement: Supplementary file 2 — Source data Fig. 1 [file 44321_2025_315_MOESM2_ESM.zip › Figure 1/F1A/1-GLDC-PDGFRbeta/Lee V/8 (2).jpg]

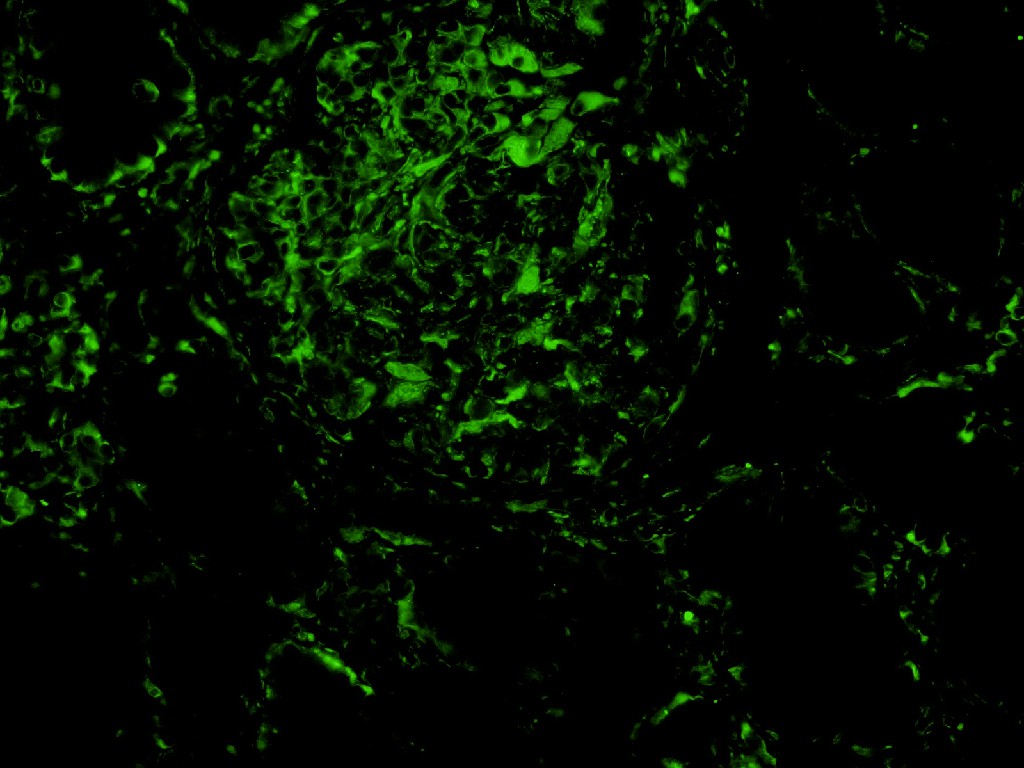

Supplement: Supplementary file 2 — Source data Fig. 1 [file 44321_2025_315_MOESM2_ESM.zip › Figure 1/F1A/1-GLDC-PDGFRbeta/Lee V/8 (3).jpg]

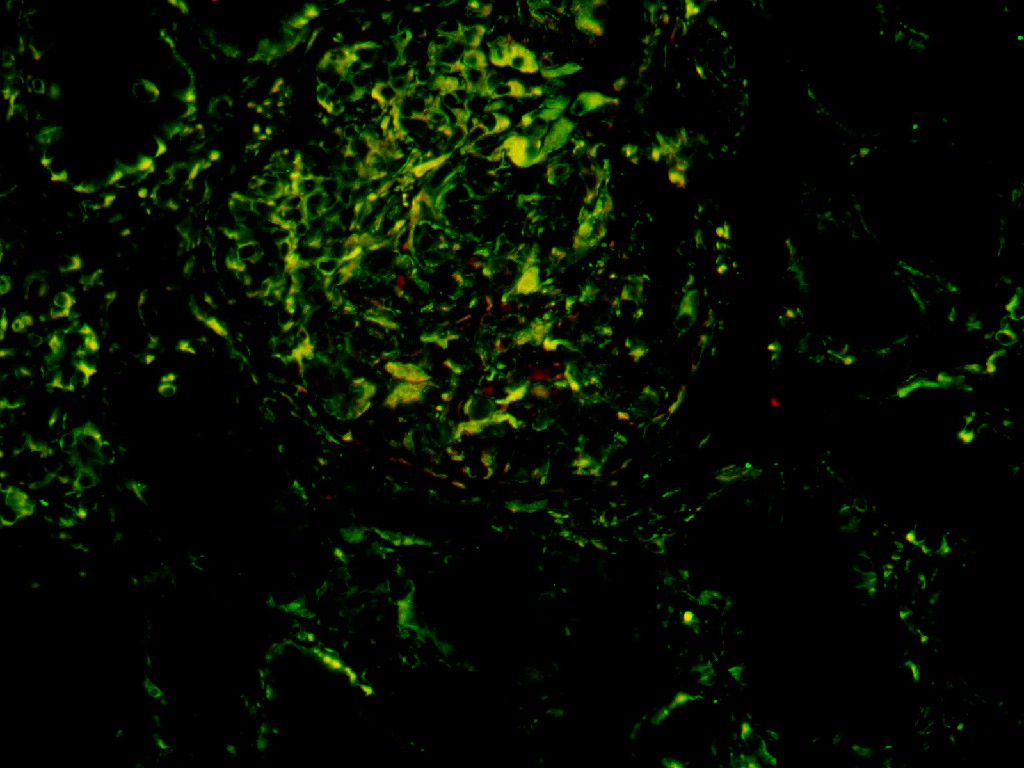

Supplement: Supplementary file 2 — Source data Fig. 1 [file 44321_2025_315_MOESM2_ESM.zip › Figure 1/F1A/1-GLDC-PDGFRbeta/Lee V/8 (4).jpg]

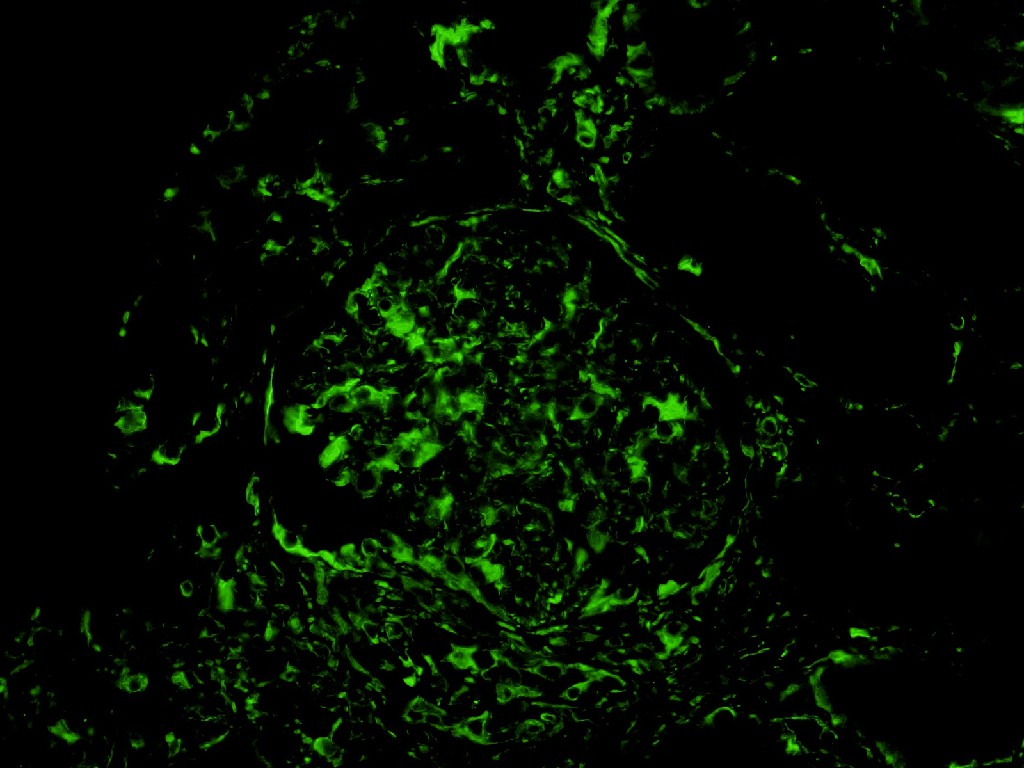

Supplement: Supplementary file 2 — Source data Fig. 1 [file 44321_2025_315_MOESM2_ESM.zip › Figure 1/F1A/1-GLDC-PDGFRbeta/Lee V/9 (1).jpg]

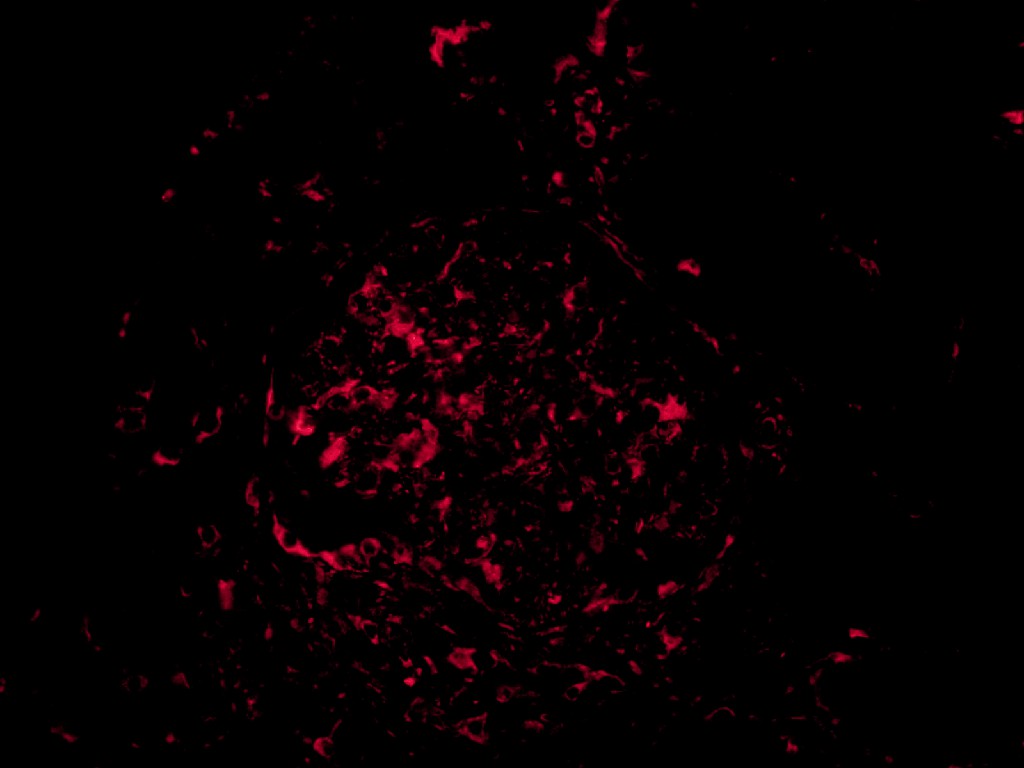

Supplement: Supplementary file 2 — Source data Fig. 1 [file 44321_2025_315_MOESM2_ESM.zip › Figure 1/F1A/1-GLDC-PDGFRbeta/Lee V/9 (2).jpg]

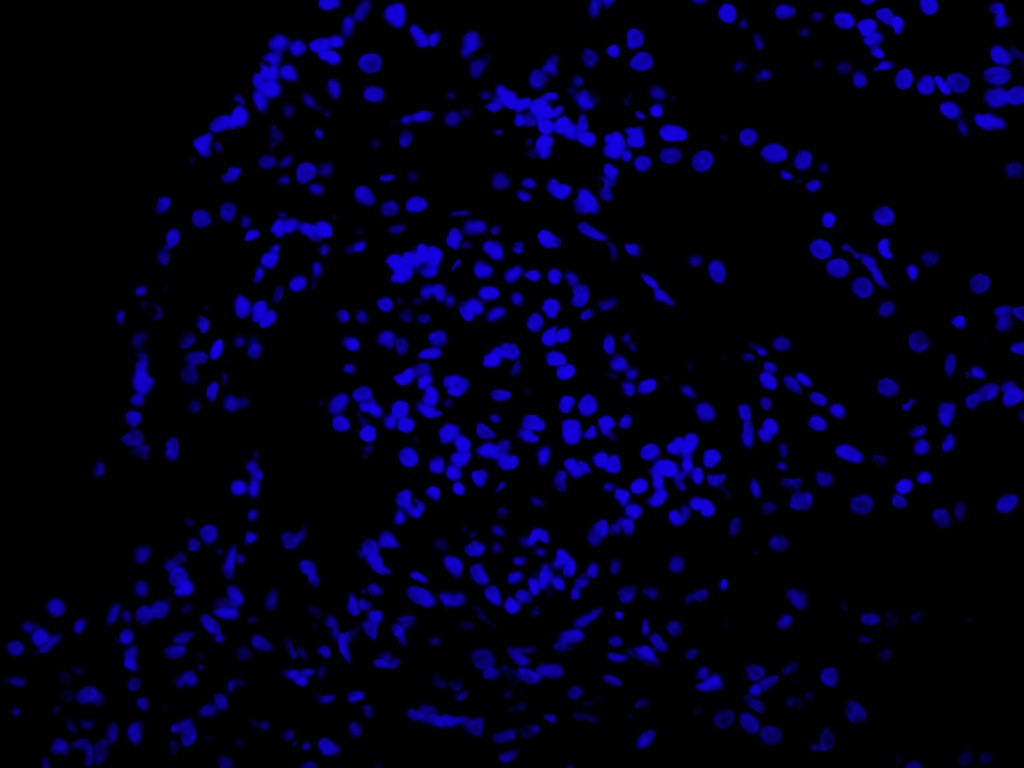

Supplement: Supplementary file 2 — Source data Fig. 1 [file 44321_2025_315_MOESM2_ESM.zip › Figure 1/F1A/1-GLDC-PDGFRbeta/Lee V/9 (3).jpg]

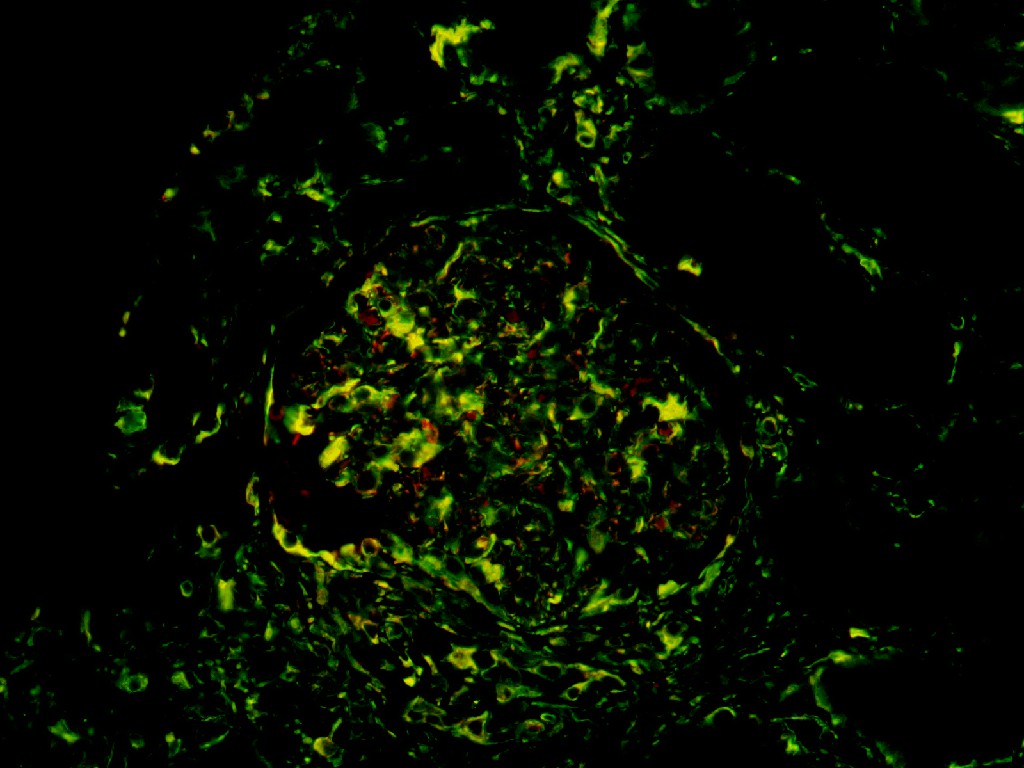

Supplement: Supplementary file 2 — Source data Fig. 1 [file 44321_2025_315_MOESM2_ESM.zip › Figure 1/F1A/1-GLDC-PDGFRbeta/Lee V/9 (4).jpg]

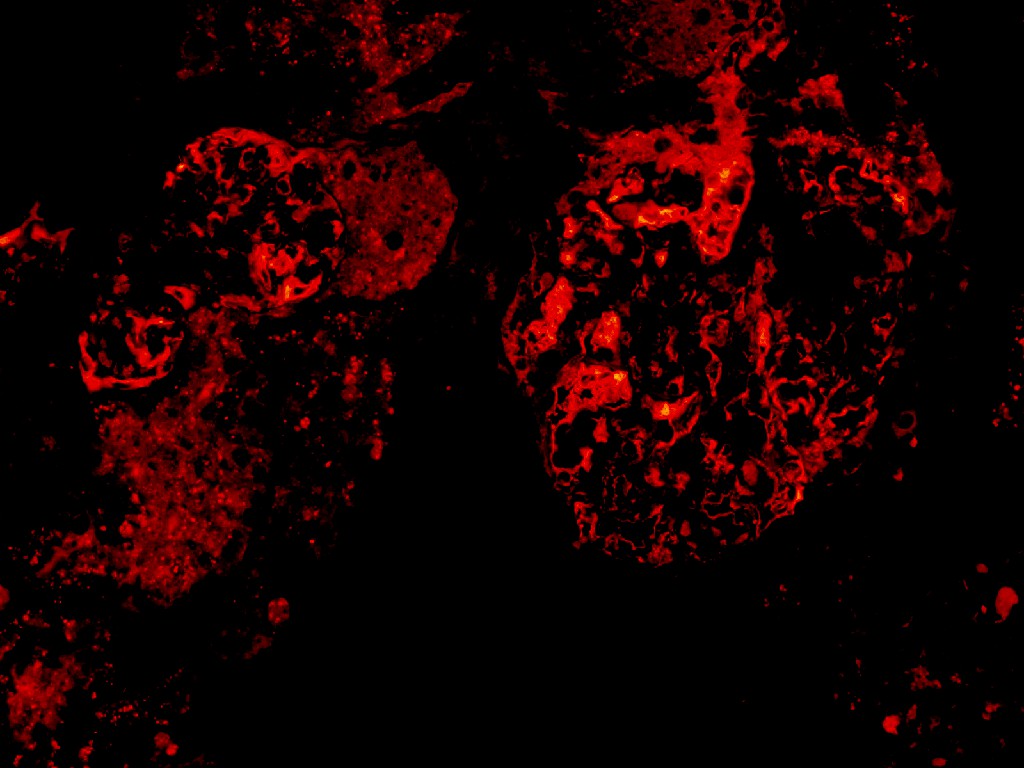

Supplement: Supplementary file 2 — Source data Fig. 1 [file 44321_2025_315_MOESM2_ESM.zip › Figure 1/F1A/2-IgA/Lee II(1).jpg]

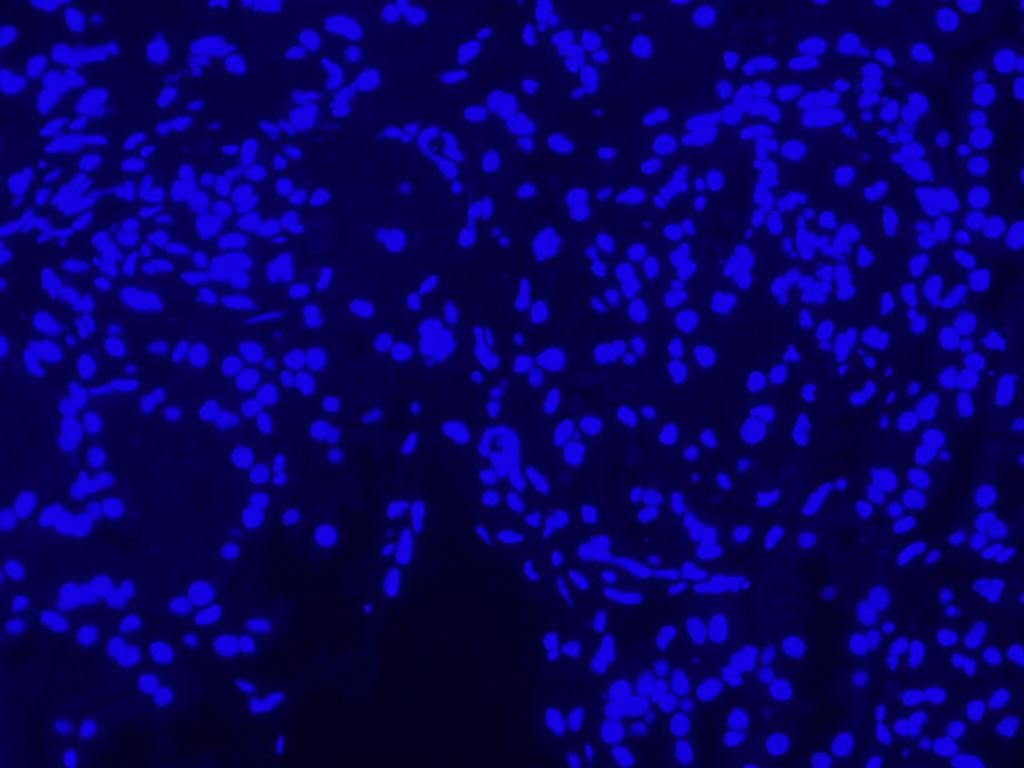

Supplement: Supplementary file 2 — Source data Fig. 1 [file 44321_2025_315_MOESM2_ESM.zip › Figure 1/F1A/2-IgA/Lee II(2).jpg]

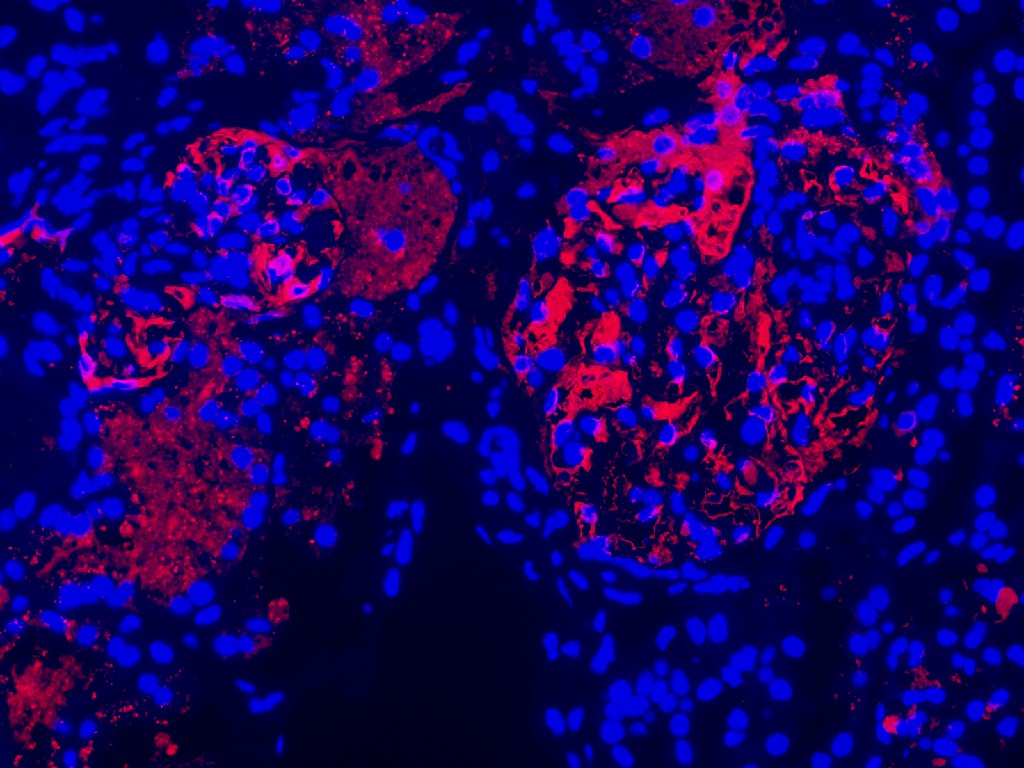

Supplement: Supplementary file 2 — Source data Fig. 1 [file 44321_2025_315_MOESM2_ESM.zip › Figure 1/F1A/2-IgA/Lee II(3).jpg]

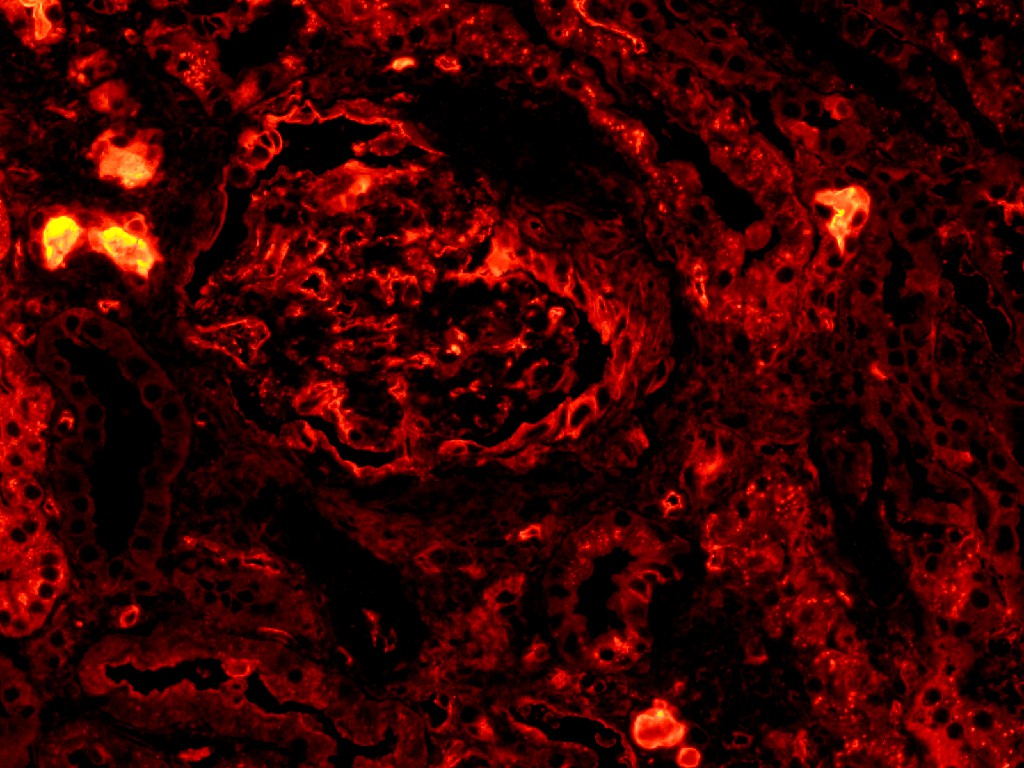

Supplement: Supplementary file 2 — Source data Fig. 1 [file 44321_2025_315_MOESM2_ESM.zip › Figure 1/F1A/2-IgA/Lee III (1).jpg]

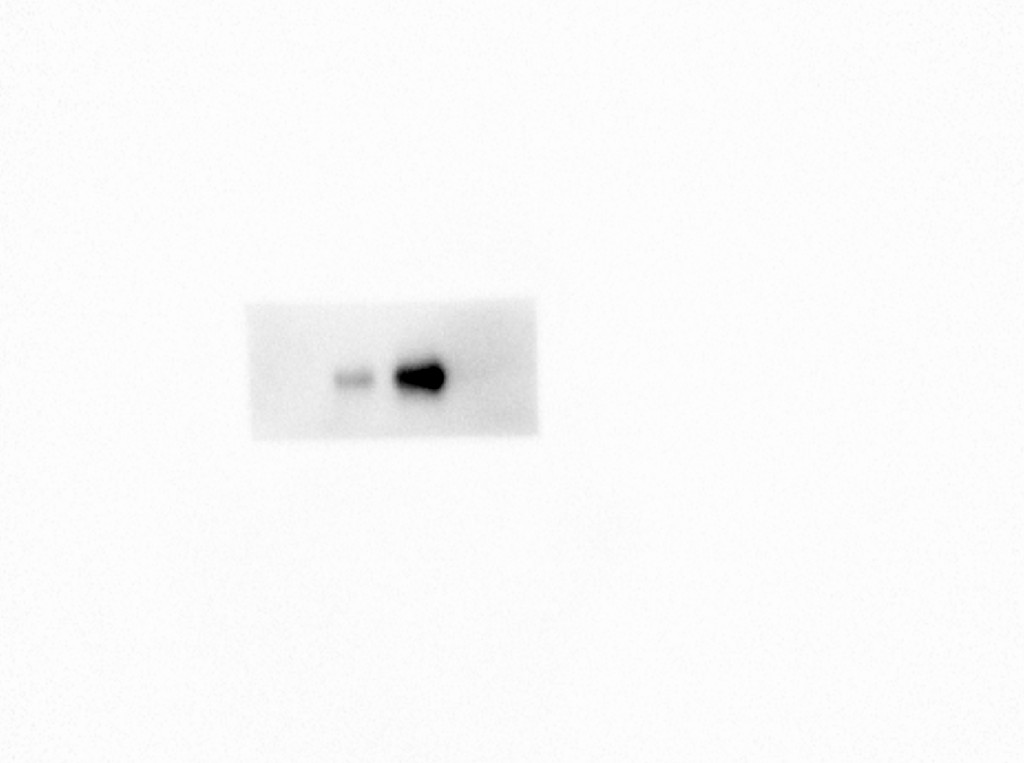

Supplement: Supplementary file 3 — Source data Fig. 2 [file 44321_2025_315_MOESM3_ESM.zip › Figure 2/F2C-WB/1-F2C left/1-1-GLDC.jpg]

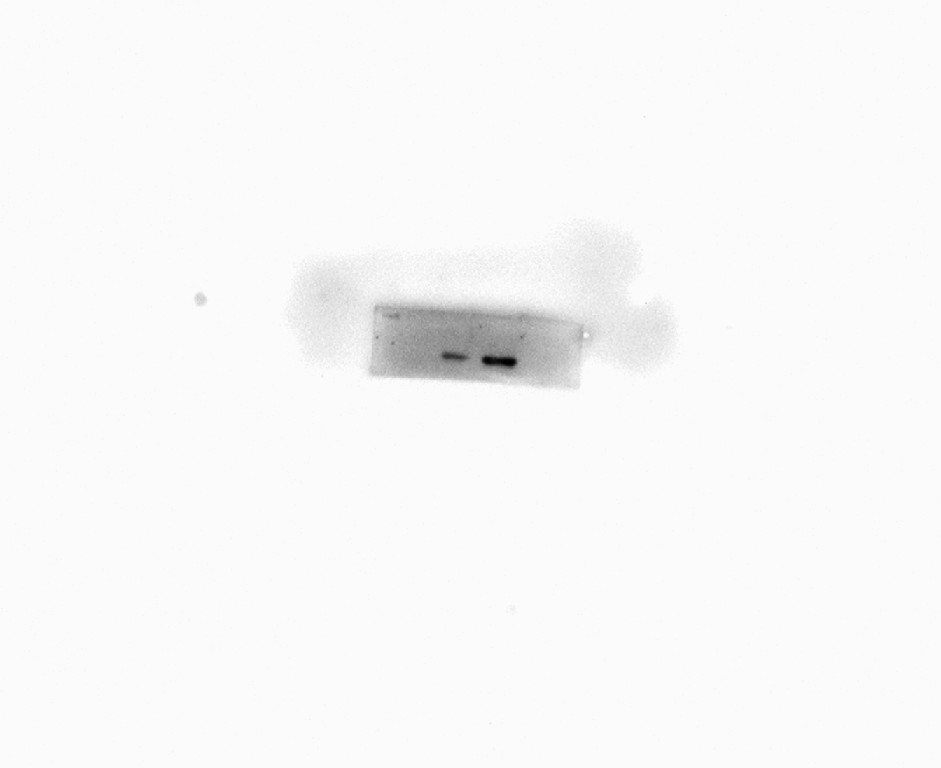

Supplement: Supplementary file 3 — Source data Fig. 2 [file 44321_2025_315_MOESM3_ESM.zip › Figure 2/F2C-WB/1-F2C left/1-2-GLDC.jpg]
