# Supplementary figures and images for: Glycine decarboxylase advances IgA nephropathy by boosting mesangial cell proliferation through the pyrimidine pathway (part 1 of 7)
Source: EMBO Mol Med. 2025 Oct 13;17(11):3039–63. doi: 10.1038/s44321-025-00315-2 (PMC12603144; doi:10.1038/s44321-025-00315-2)

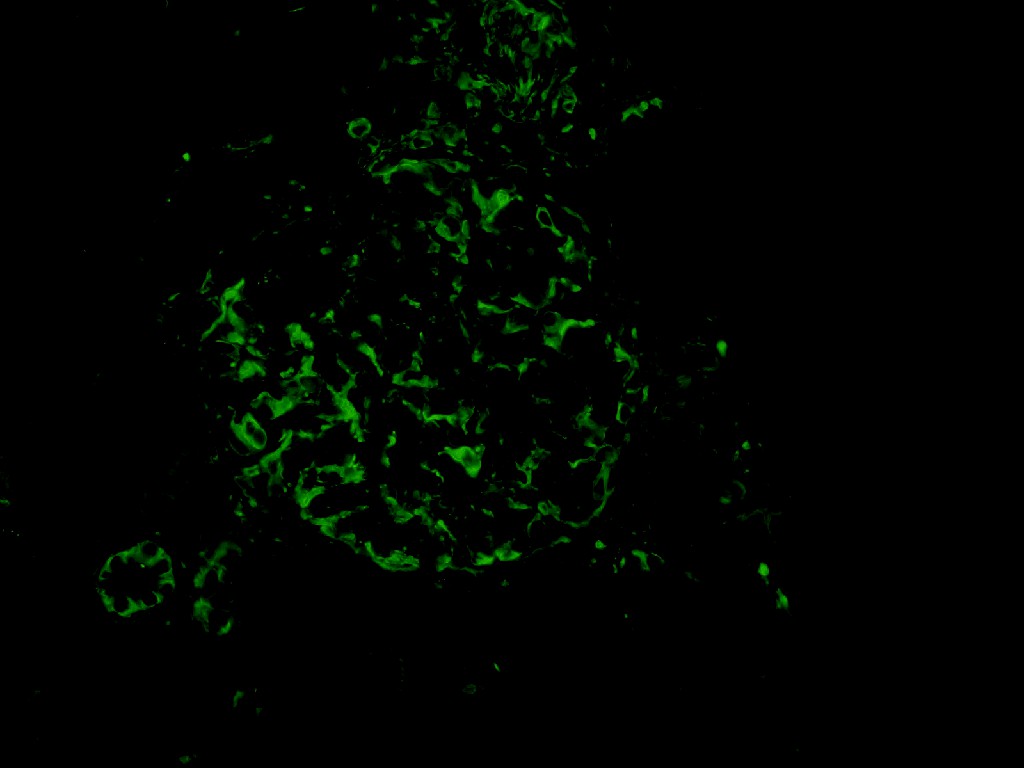

Supplement: Supplementary file 2 — Source data Fig. 1 [file 44321_2025_315_MOESM2_ESM.zip › Figure 1/F1A/1-GLDC-PDGFRbeta/Lee II/1 (1).jpg]

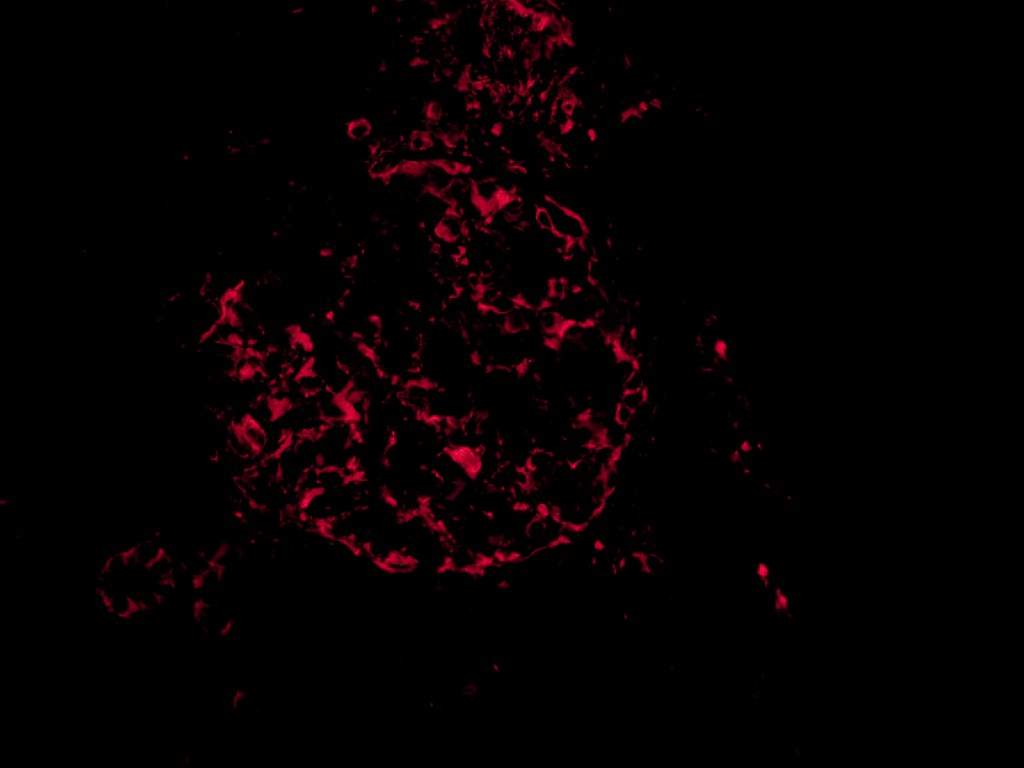

Supplement: Supplementary file 2 — Source data Fig. 1 [file 44321_2025_315_MOESM2_ESM.zip › Figure 1/F1A/1-GLDC-PDGFRbeta/Lee II/1 (2).jpg]

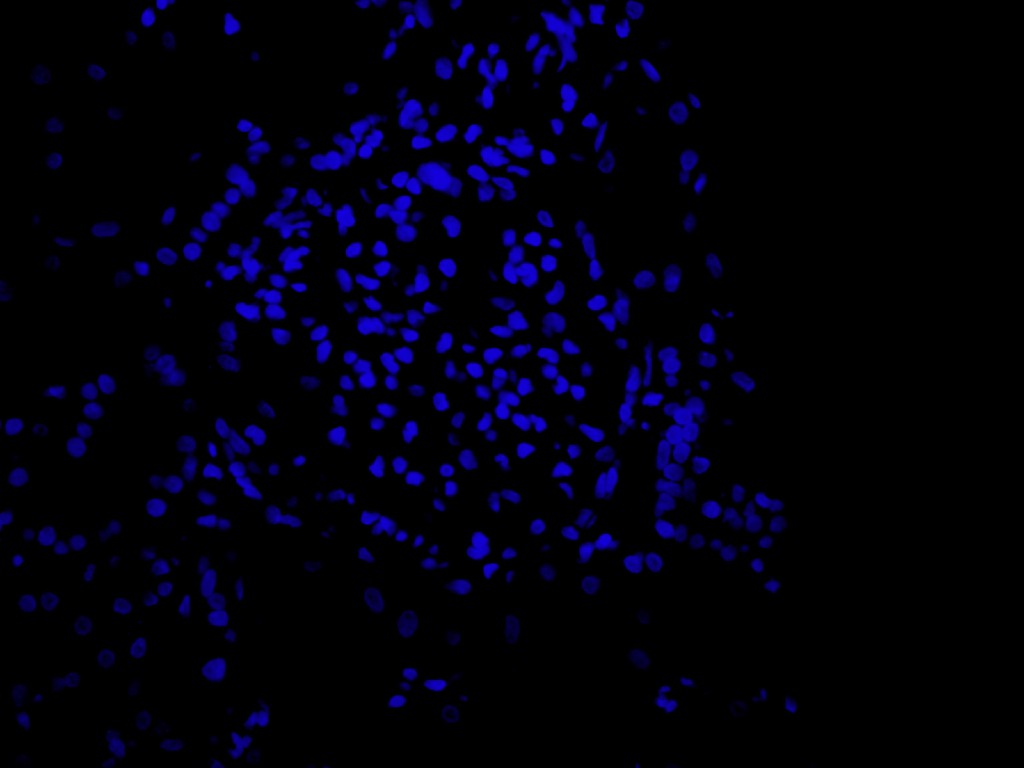

Supplement: Supplementary file 2 — Source data Fig. 1 [file 44321_2025_315_MOESM2_ESM.zip › Figure 1/F1A/1-GLDC-PDGFRbeta/Lee II/1 (3).jpg]

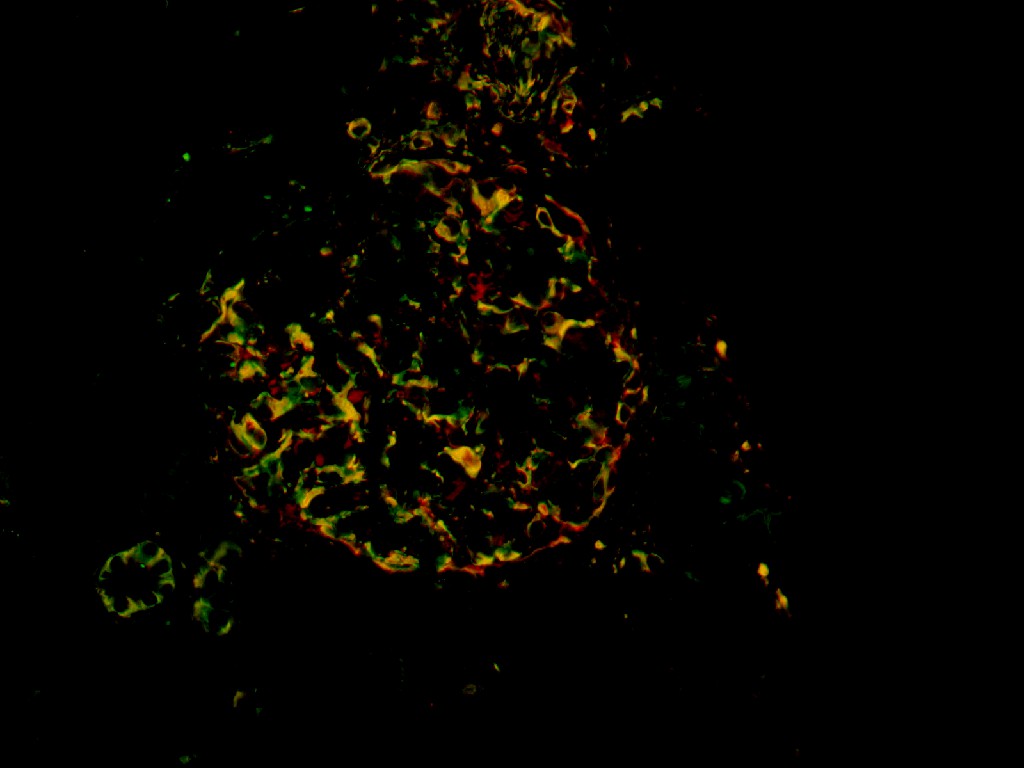

Supplement: Supplementary file 2 — Source data Fig. 1 [file 44321_2025_315_MOESM2_ESM.zip › Figure 1/F1A/1-GLDC-PDGFRbeta/Lee II/1 (4).jpg]

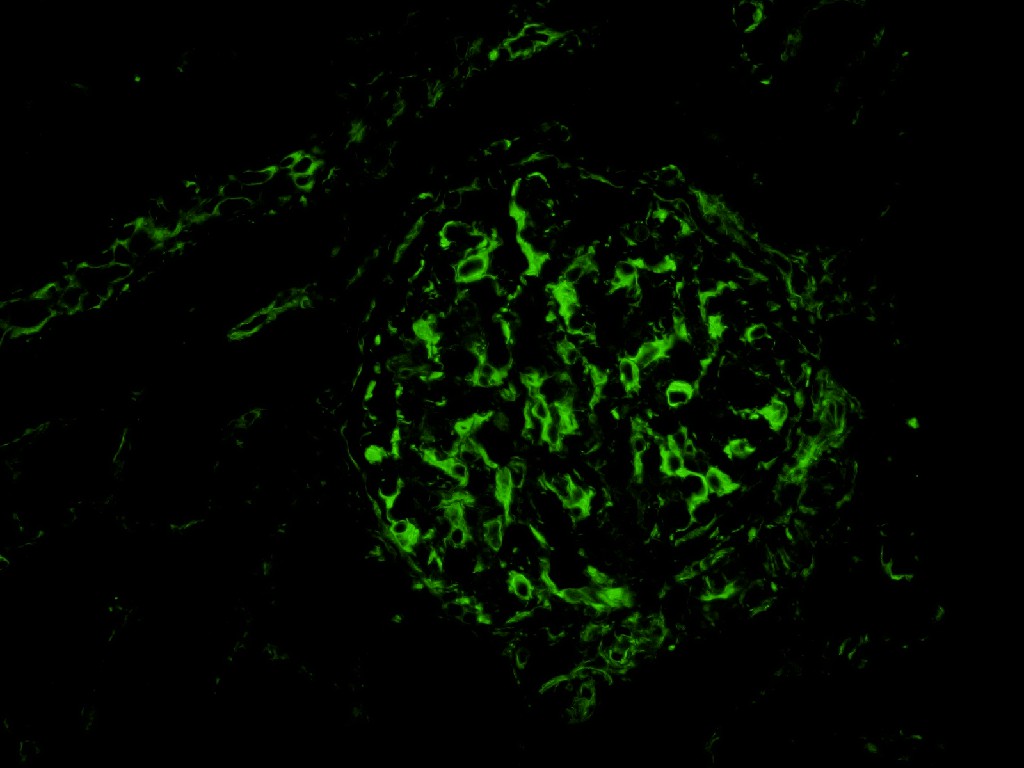

Supplement: Supplementary file 2 — Source data Fig. 1 [file 44321_2025_315_MOESM2_ESM.zip › Figure 1/F1A/1-GLDC-PDGFRbeta/Lee II/10 (1).jpg]

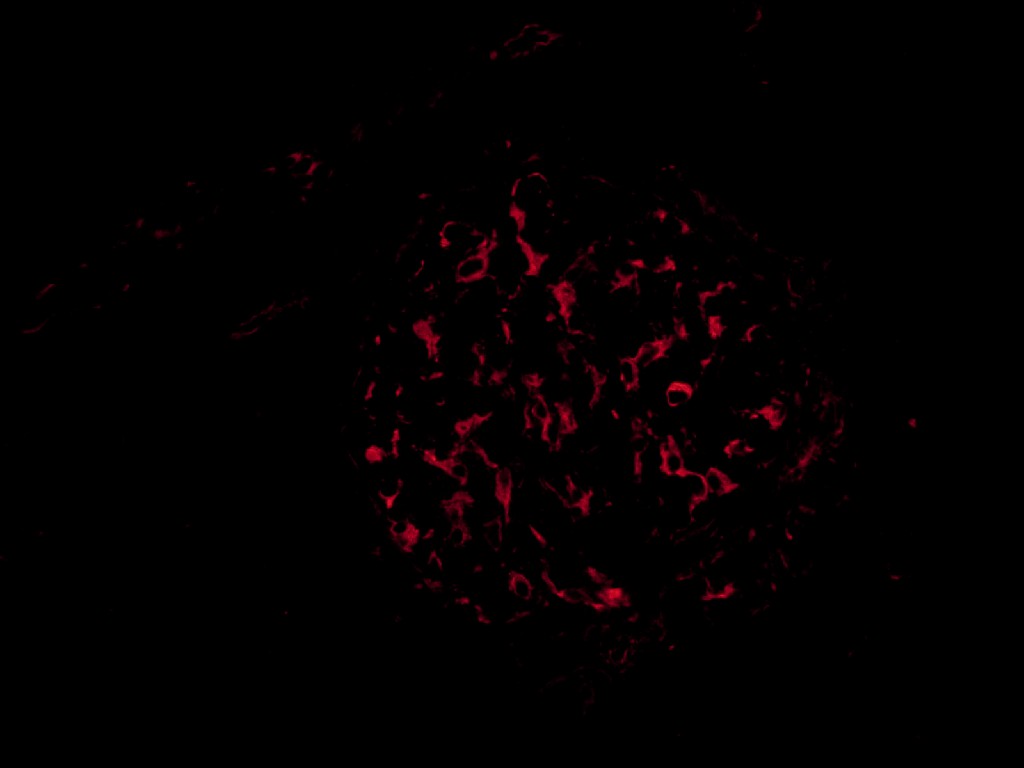

Supplement: Supplementary file 2 — Source data Fig. 1 [file 44321_2025_315_MOESM2_ESM.zip › Figure 1/F1A/1-GLDC-PDGFRbeta/Lee II/10 (2).jpg]

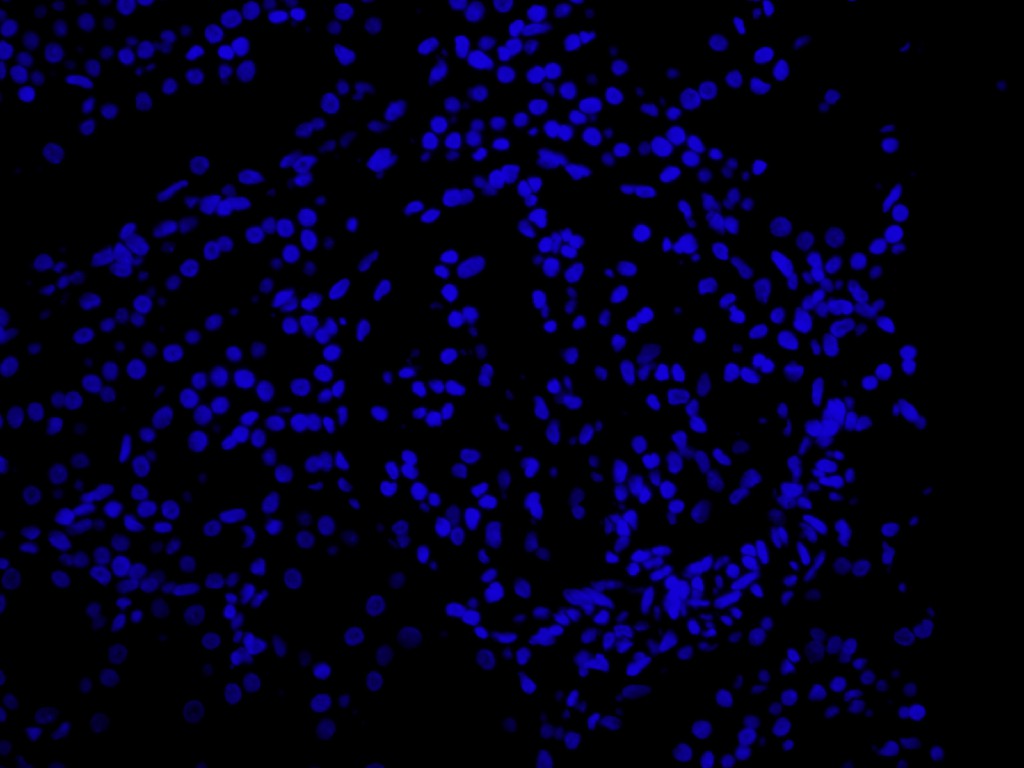

Supplement: Supplementary file 2 — Source data Fig. 1 [file 44321_2025_315_MOESM2_ESM.zip › Figure 1/F1A/1-GLDC-PDGFRbeta/Lee II/10 (3).jpg]

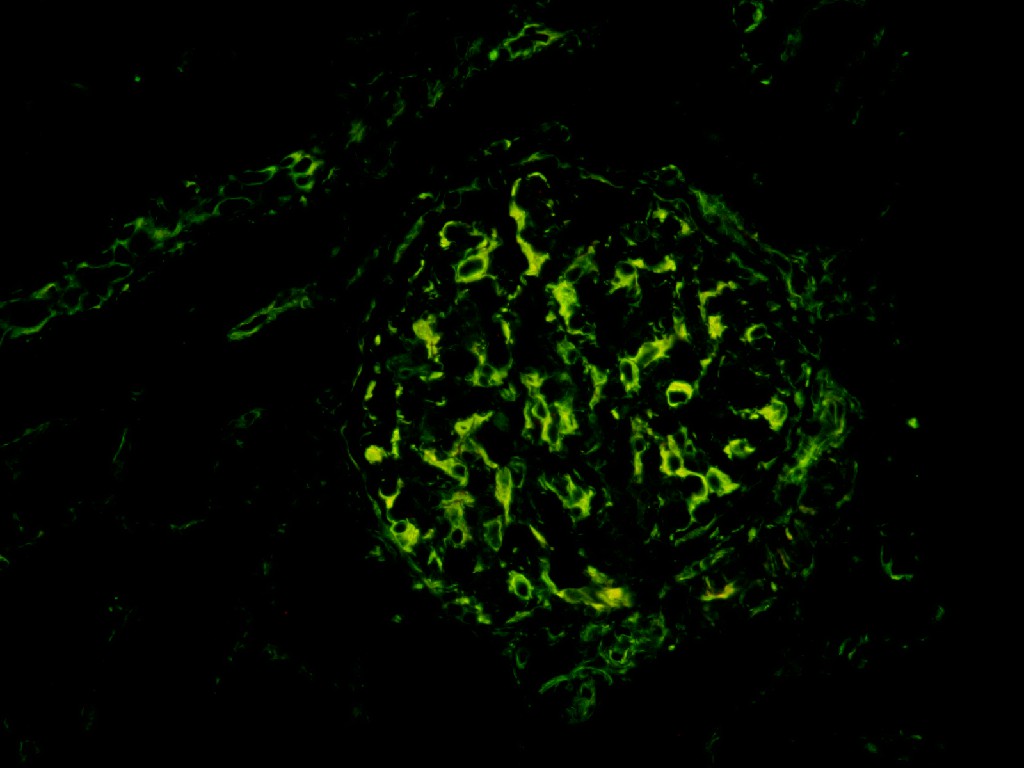

Supplement: Supplementary file 2 — Source data Fig. 1 [file 44321_2025_315_MOESM2_ESM.zip › Figure 1/F1A/1-GLDC-PDGFRbeta/Lee II/10 (4).jpg]

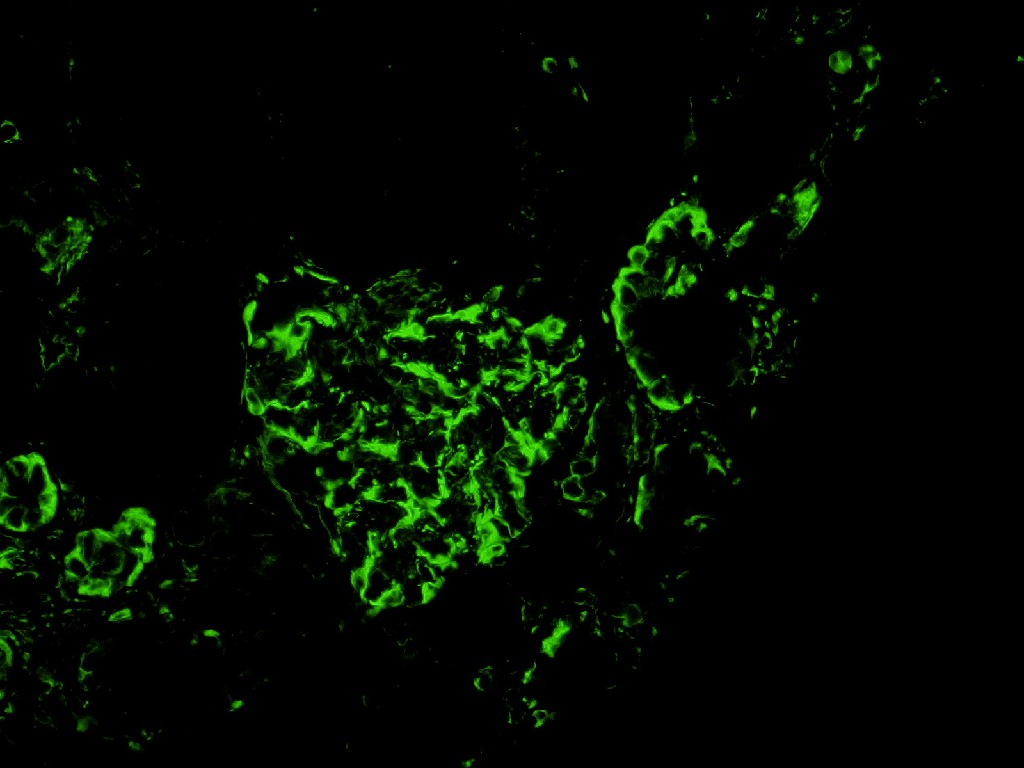

Supplement: Supplementary file 2 — Source data Fig. 1 [file 44321_2025_315_MOESM2_ESM.zip › Figure 1/F1A/1-GLDC-PDGFRbeta/Lee II/11 (1).jpg]

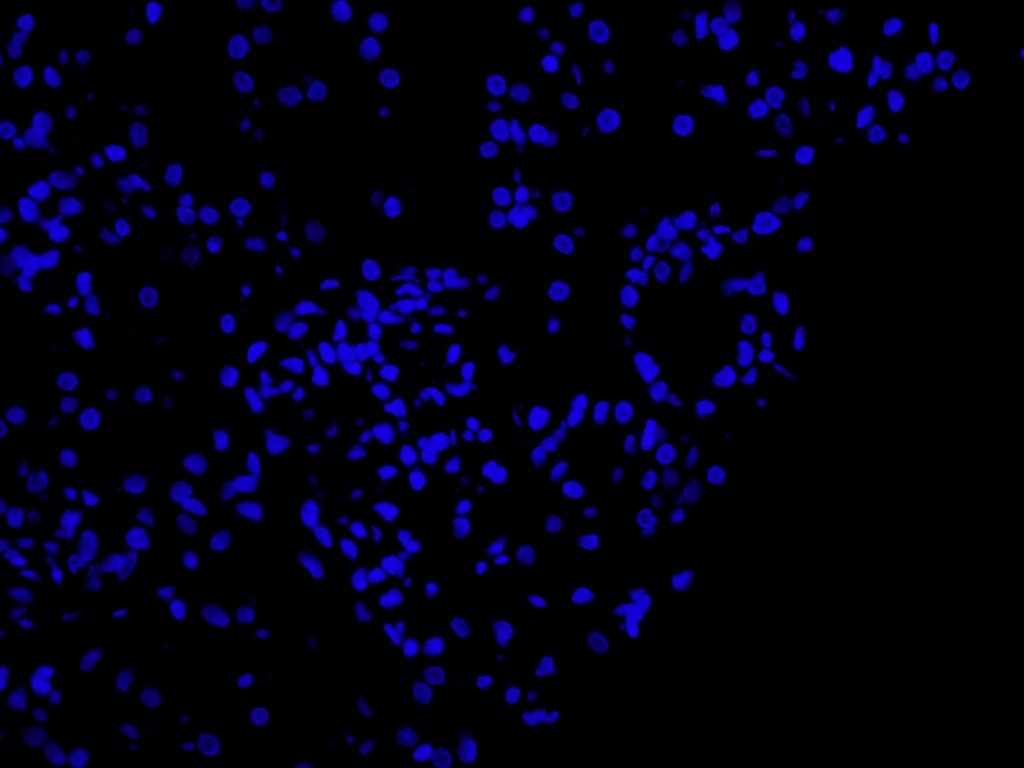

Supplement: Supplementary file 2 — Source data Fig. 1 [file 44321_2025_315_MOESM2_ESM.zip › Figure 1/F1A/1-GLDC-PDGFRbeta/Lee II/11 (2).jpg]

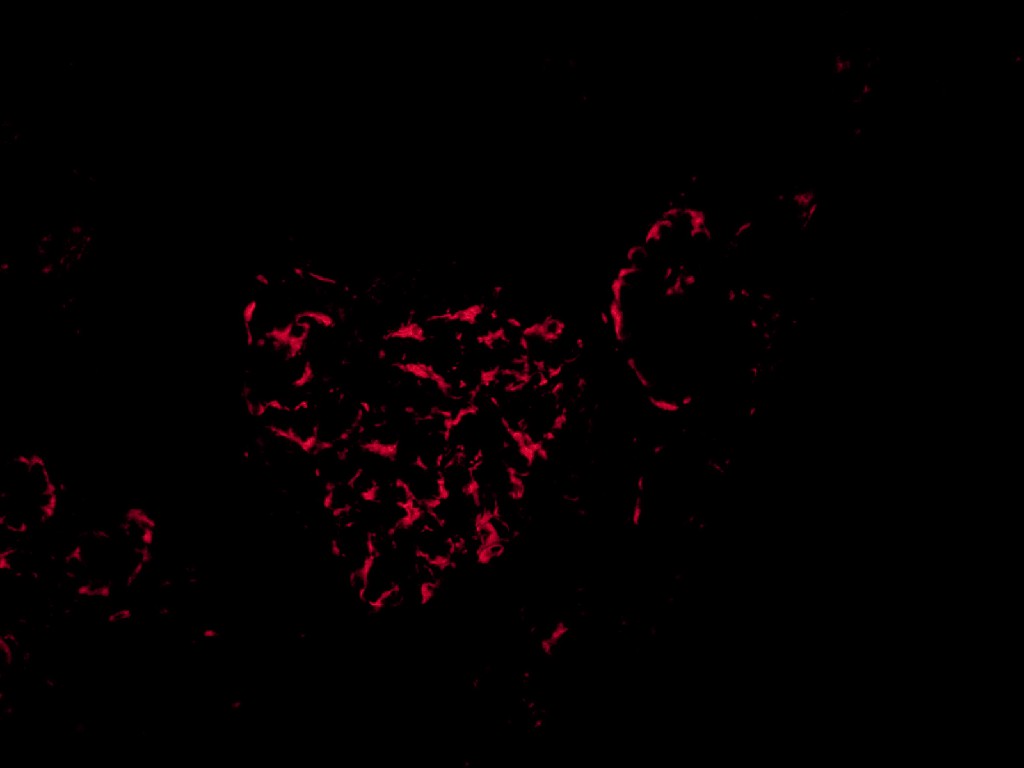

Supplement: Supplementary file 2 — Source data Fig. 1 [file 44321_2025_315_MOESM2_ESM.zip › Figure 1/F1A/1-GLDC-PDGFRbeta/Lee II/11 (3).jpg]

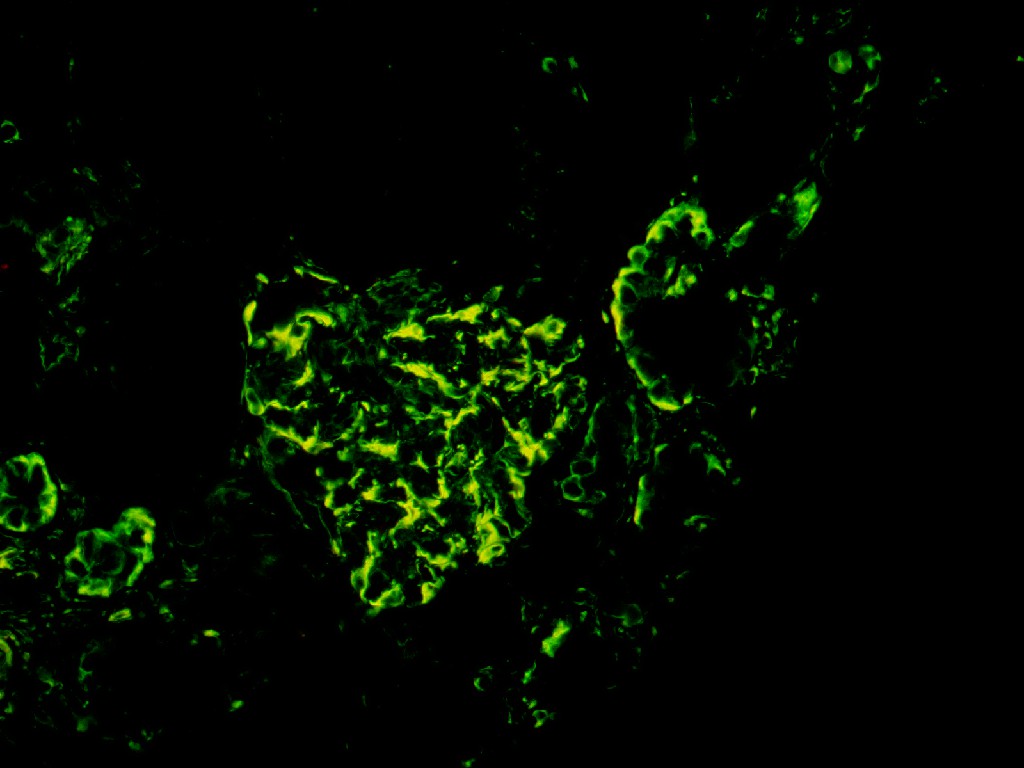

Supplement: Supplementary file 2 — Source data Fig. 1 [file 44321_2025_315_MOESM2_ESM.zip › Figure 1/F1A/1-GLDC-PDGFRbeta/Lee II/11 (4).jpg]

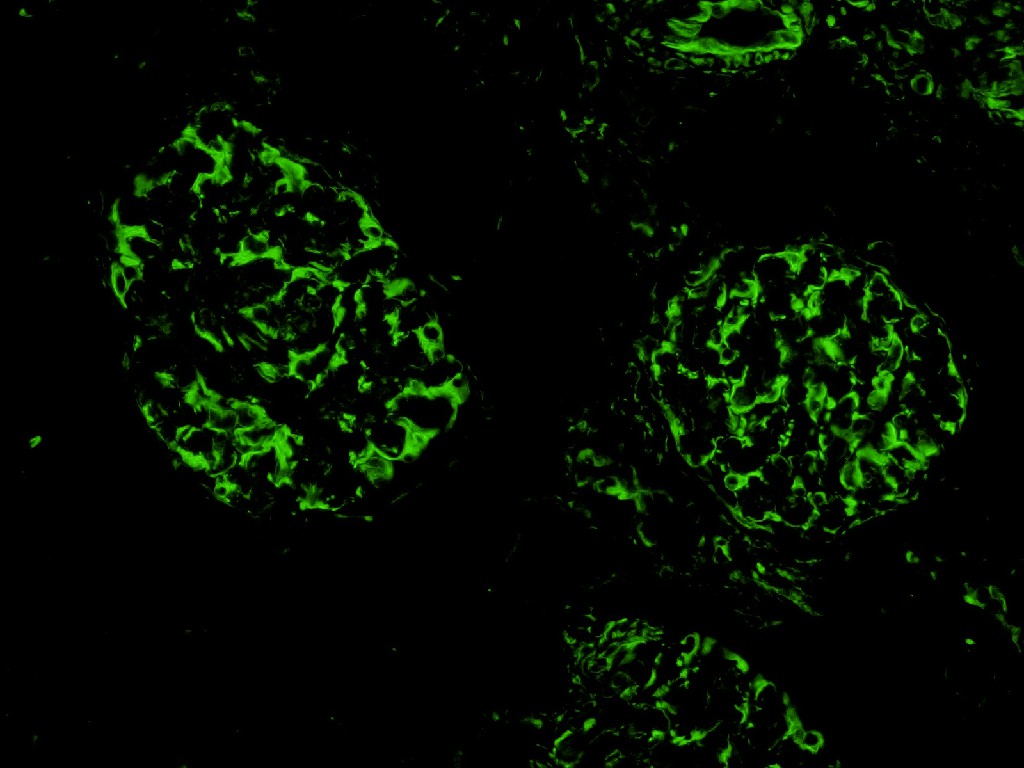

Supplement: Supplementary file 2 — Source data Fig. 1 [file 44321_2025_315_MOESM2_ESM.zip › Figure 1/F1A/1-GLDC-PDGFRbeta/Lee II/12 (1).jpg]

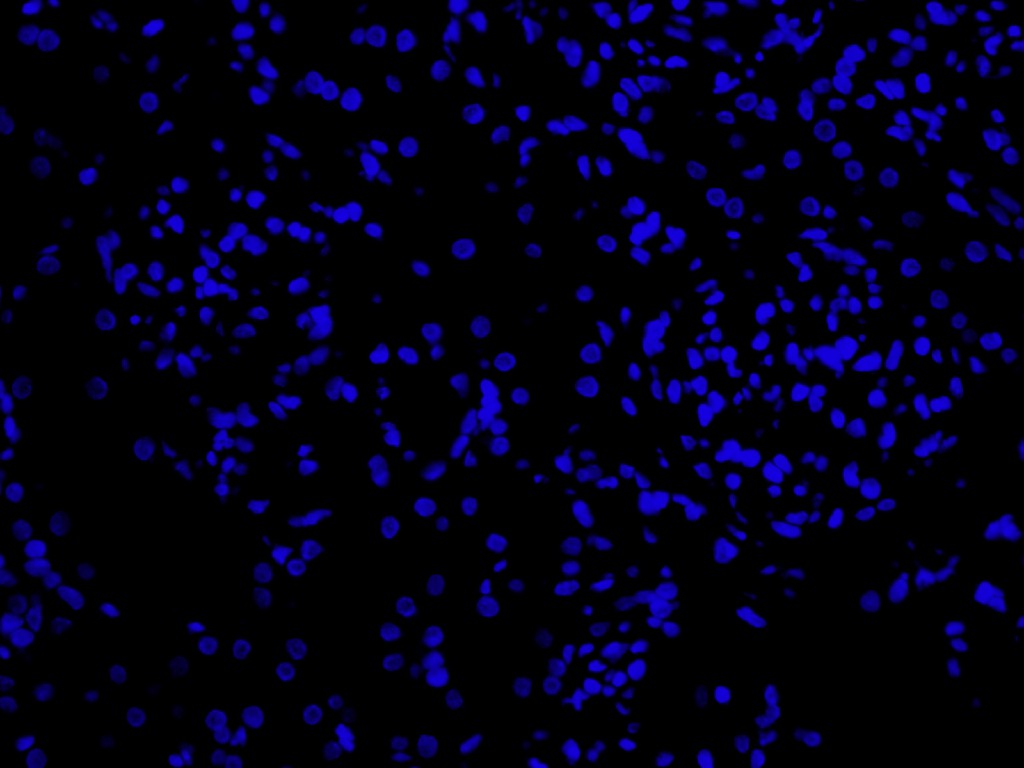

Supplement: Supplementary file 2 — Source data Fig. 1 [file 44321_2025_315_MOESM2_ESM.zip › Figure 1/F1A/1-GLDC-PDGFRbeta/Lee II/12 (2).jpg]

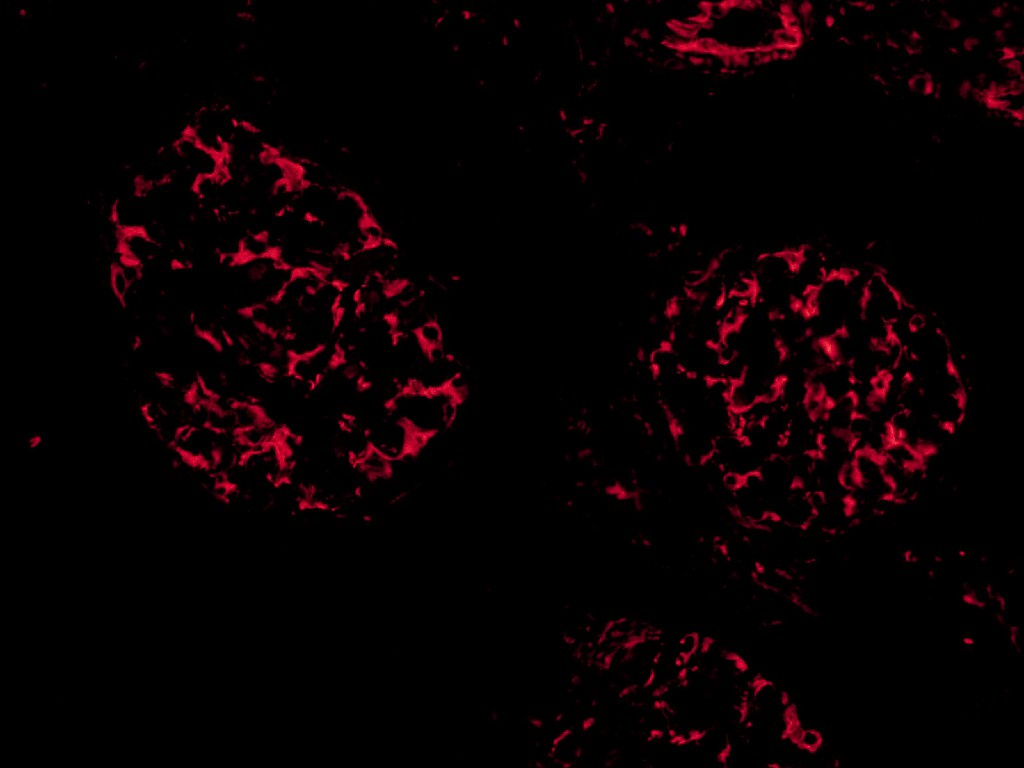

Supplement: Supplementary file 2 — Source data Fig. 1 [file 44321_2025_315_MOESM2_ESM.zip › Figure 1/F1A/1-GLDC-PDGFRbeta/Lee II/12 (3).jpg]

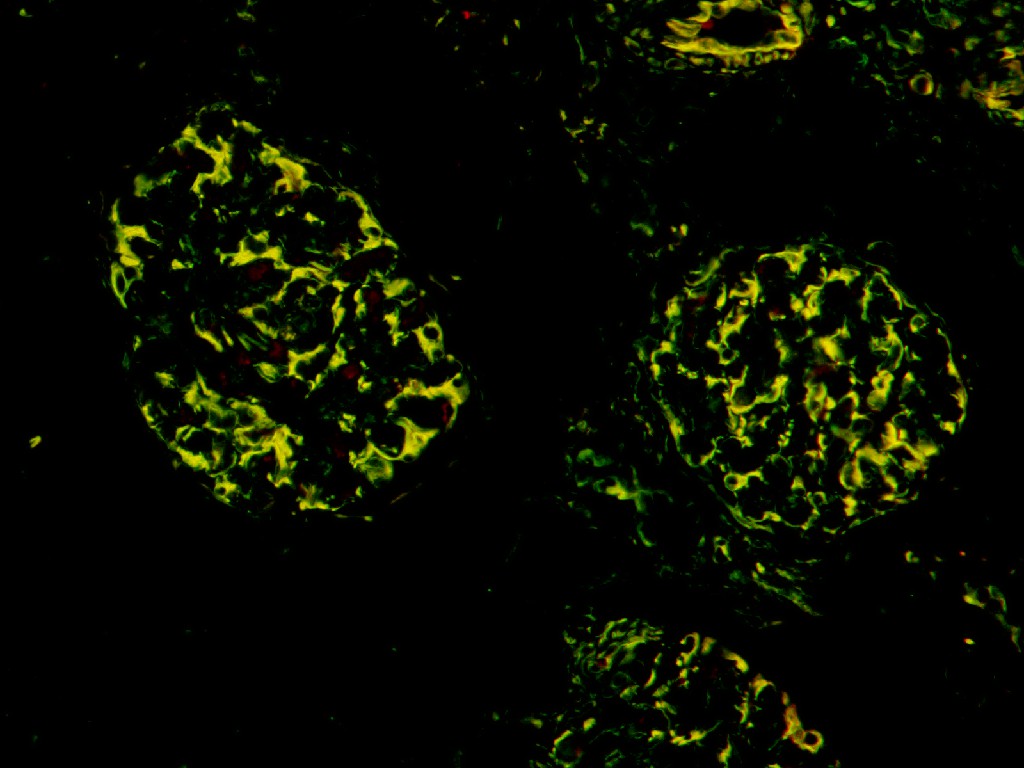

Supplement: Supplementary file 2 — Source data Fig. 1 [file 44321_2025_315_MOESM2_ESM.zip › Figure 1/F1A/1-GLDC-PDGFRbeta/Lee II/12 (4).jpg]

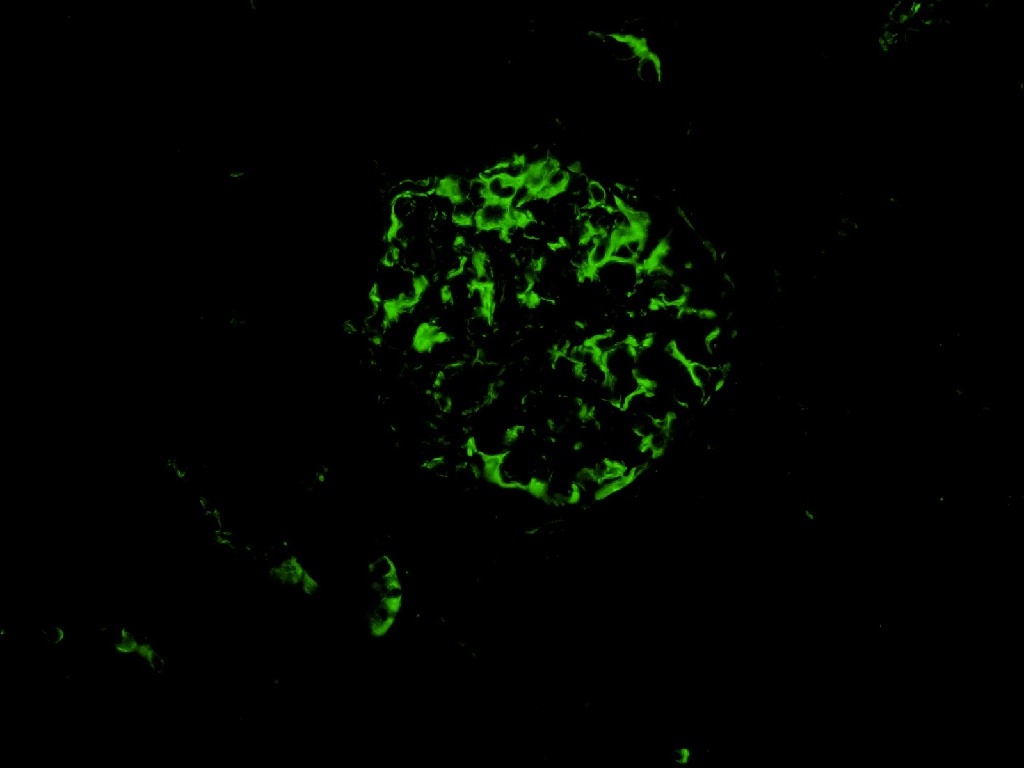

Supplement: Supplementary file 2 — Source data Fig. 1 [file 44321_2025_315_MOESM2_ESM.zip › Figure 1/F1A/1-GLDC-PDGFRbeta/Lee II/13 (1).jpg]

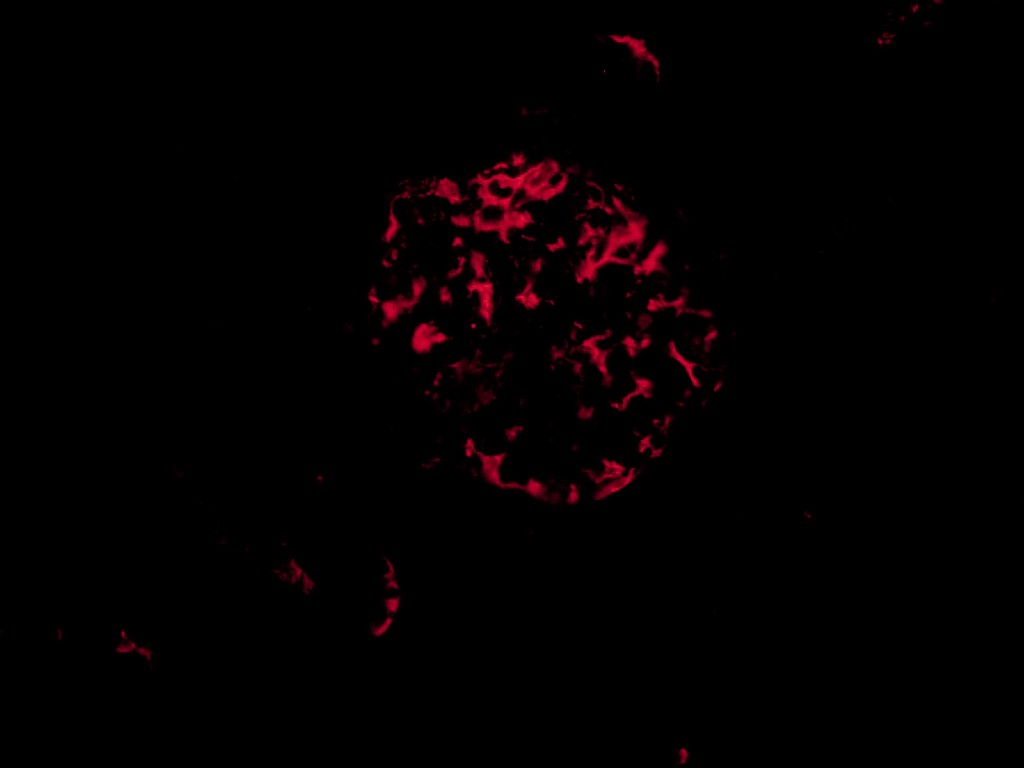

Supplement: Supplementary file 2 — Source data Fig. 1 [file 44321_2025_315_MOESM2_ESM.zip › Figure 1/F1A/1-GLDC-PDGFRbeta/Lee II/13 (2).jpg]

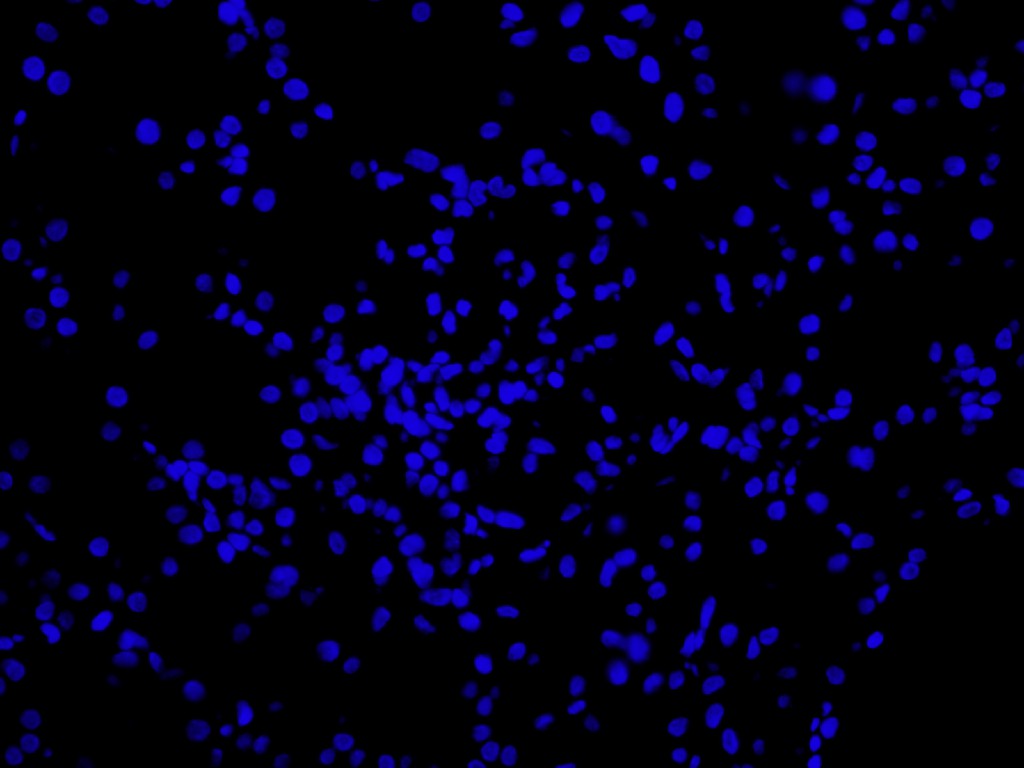

Supplement: Supplementary file 2 — Source data Fig. 1 [file 44321_2025_315_MOESM2_ESM.zip › Figure 1/F1A/1-GLDC-PDGFRbeta/Lee II/13 (3).jpg]

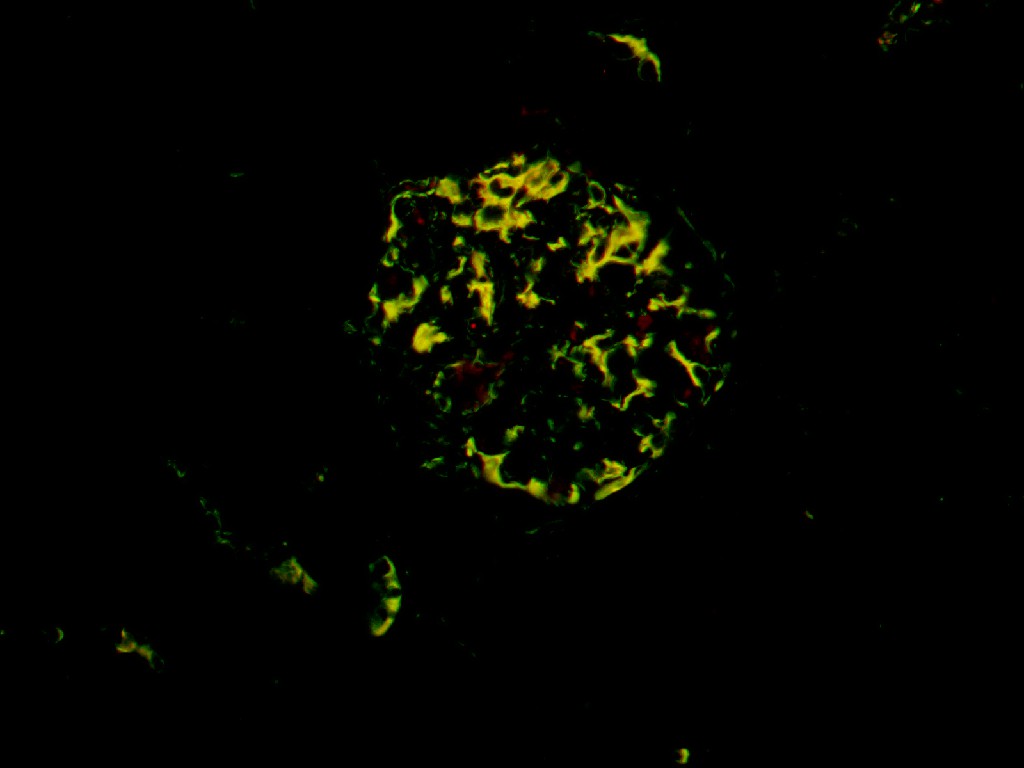

Supplement: Supplementary file 2 — Source data Fig. 1 [file 44321_2025_315_MOESM2_ESM.zip › Figure 1/F1A/1-GLDC-PDGFRbeta/Lee II/13 (4).jpg]

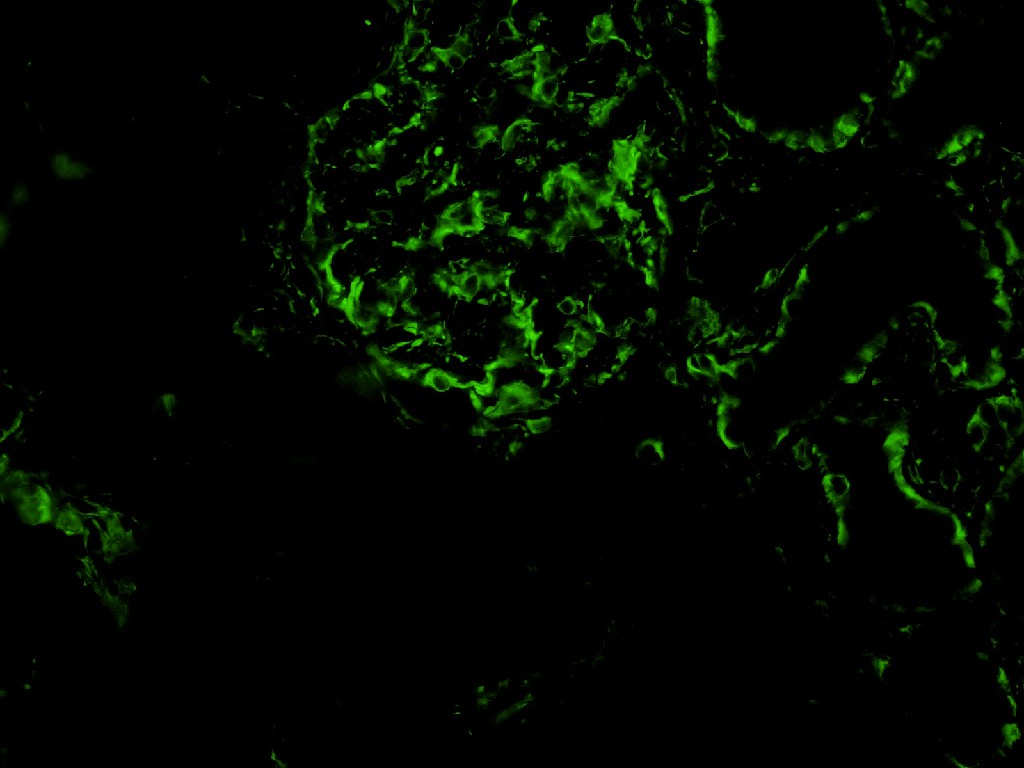

Supplement: Supplementary file 2 — Source data Fig. 1 [file 44321_2025_315_MOESM2_ESM.zip › Figure 1/F1A/1-GLDC-PDGFRbeta/Lee II/2 (1).jpg]

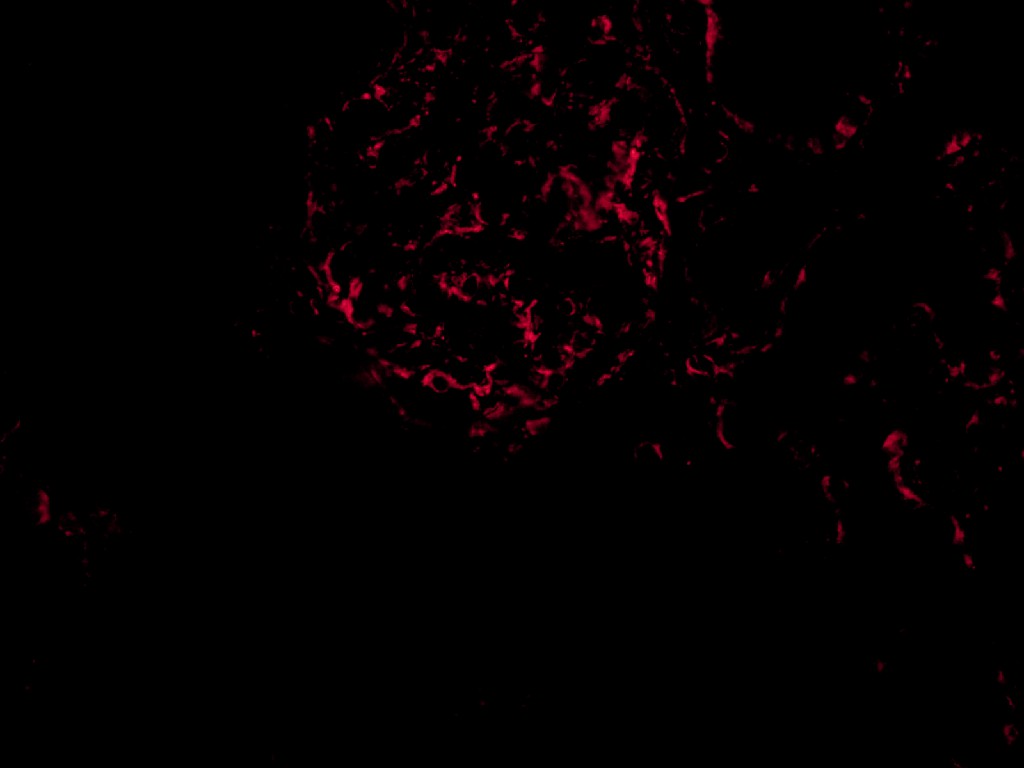

Supplement: Supplementary file 2 — Source data Fig. 1 [file 44321_2025_315_MOESM2_ESM.zip › Figure 1/F1A/1-GLDC-PDGFRbeta/Lee II/2 (2).jpg]

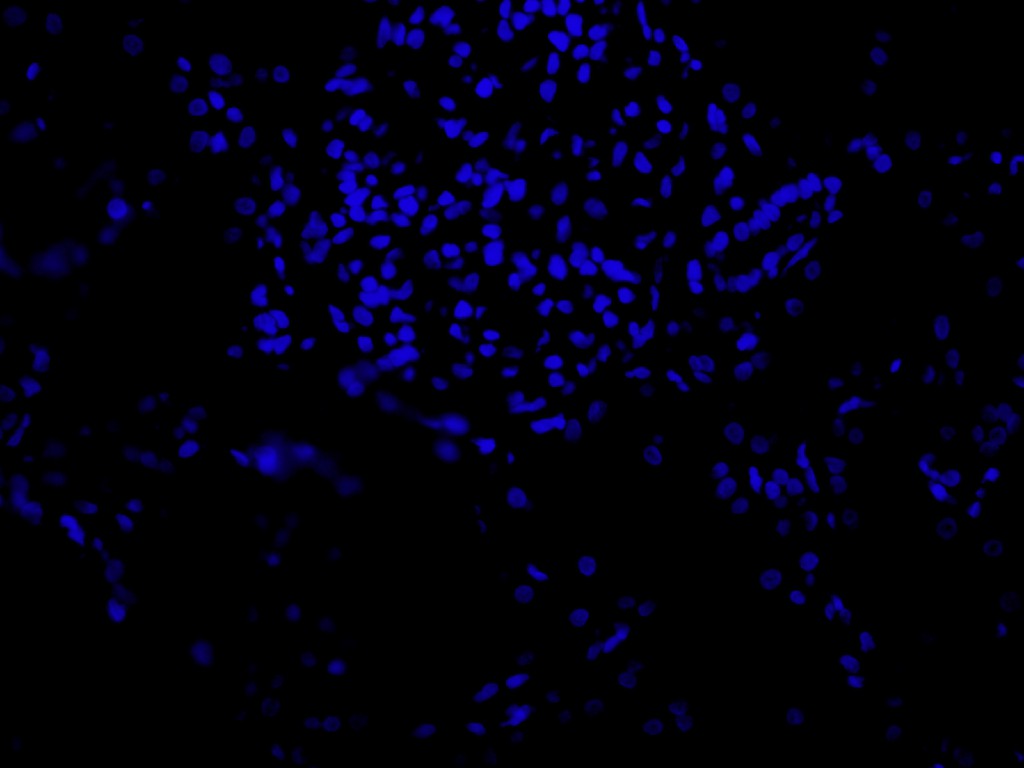

Supplement: Supplementary file 2 — Source data Fig. 1 [file 44321_2025_315_MOESM2_ESM.zip › Figure 1/F1A/1-GLDC-PDGFRbeta/Lee II/2 (3).jpg]

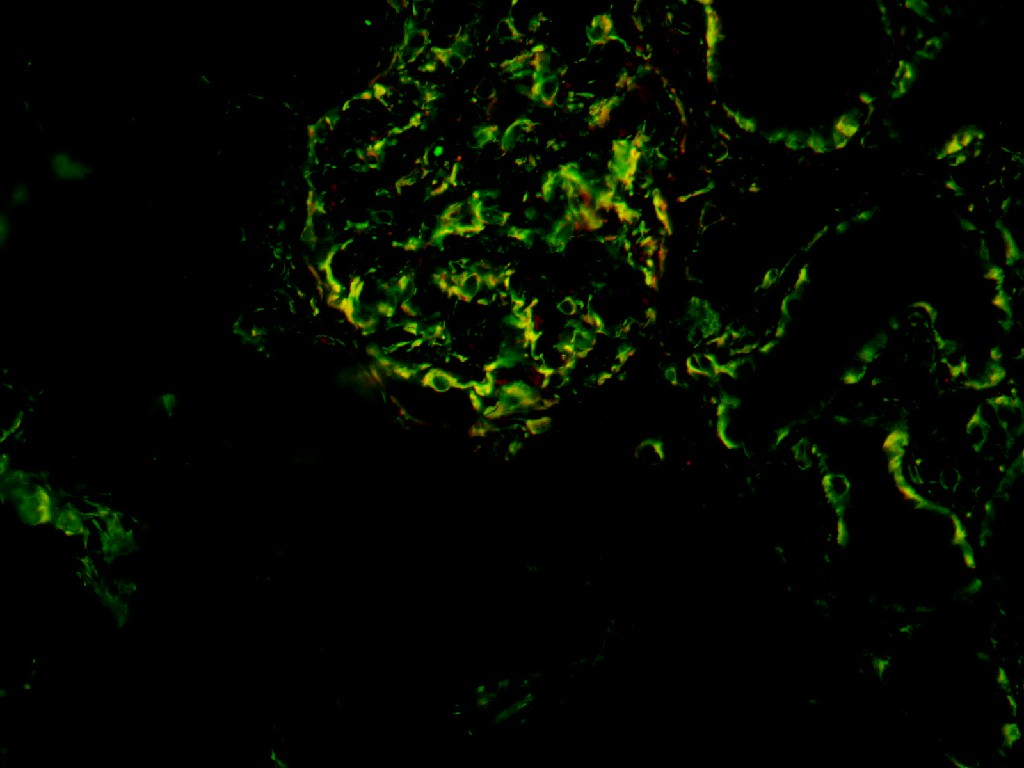

Supplement: Supplementary file 2 — Source data Fig. 1 [file 44321_2025_315_MOESM2_ESM.zip › Figure 1/F1A/1-GLDC-PDGFRbeta/Lee II/2 (4).jpg]

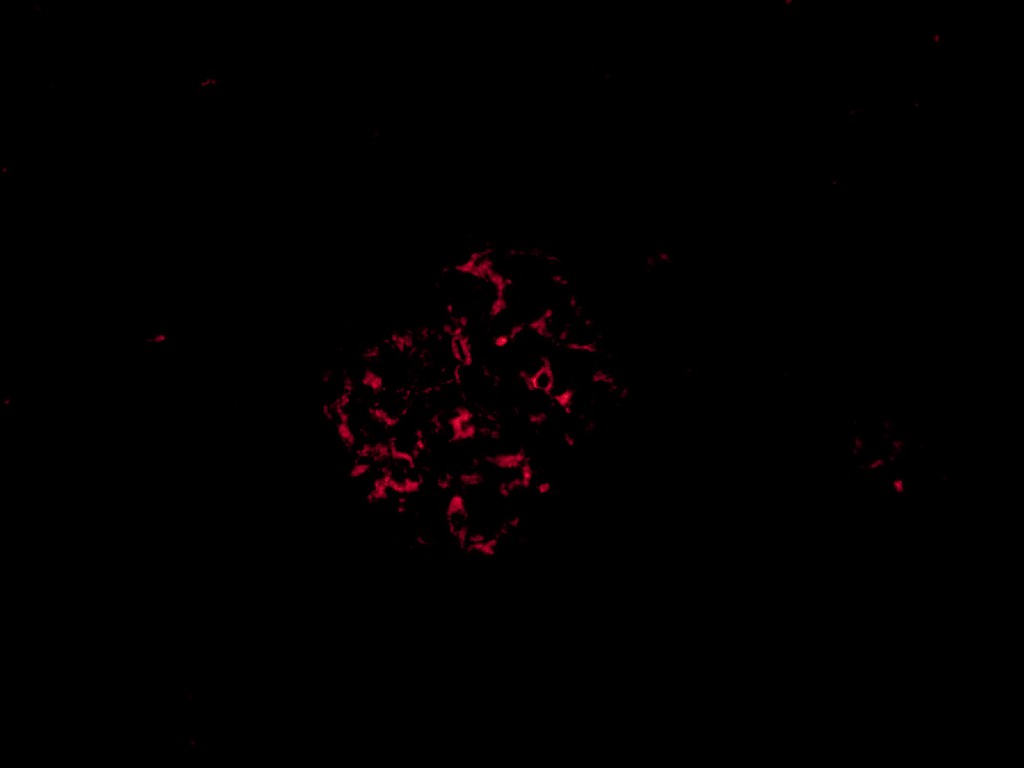

Supplement: Supplementary file 2 — Source data Fig. 1 [file 44321_2025_315_MOESM2_ESM.zip › Figure 1/F1A/1-GLDC-PDGFRbeta/Lee II/3 (1).jpg]

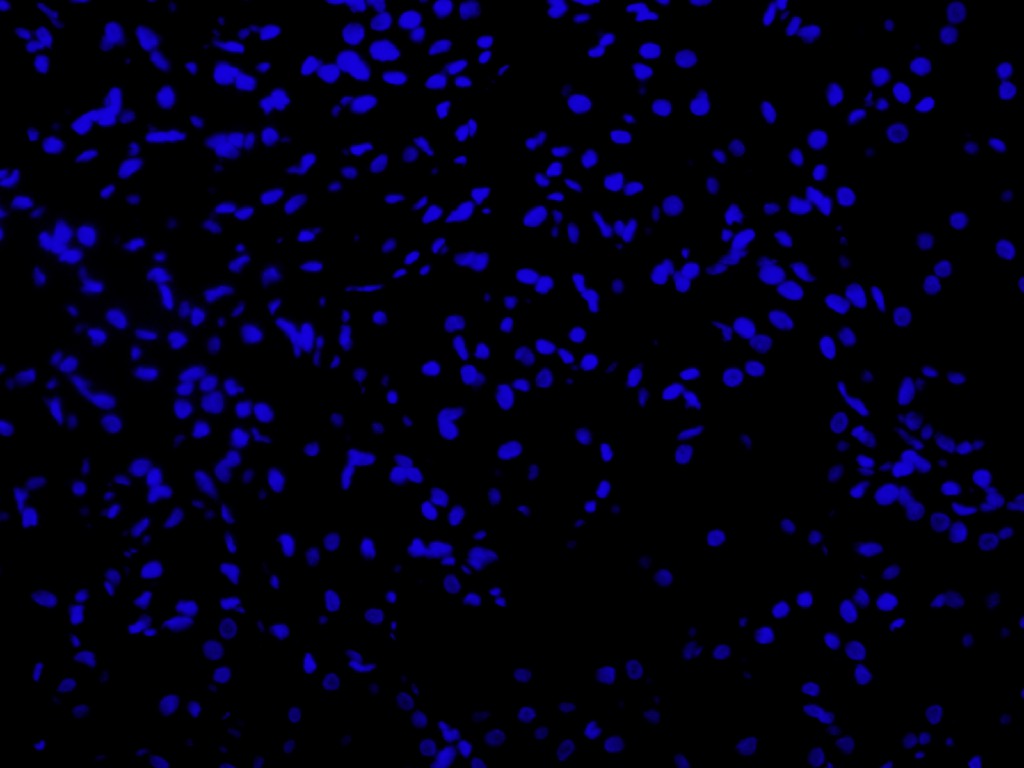

Supplement: Supplementary file 2 — Source data Fig. 1 [file 44321_2025_315_MOESM2_ESM.zip › Figure 1/F1A/1-GLDC-PDGFRbeta/Lee II/3 (2).jpg]

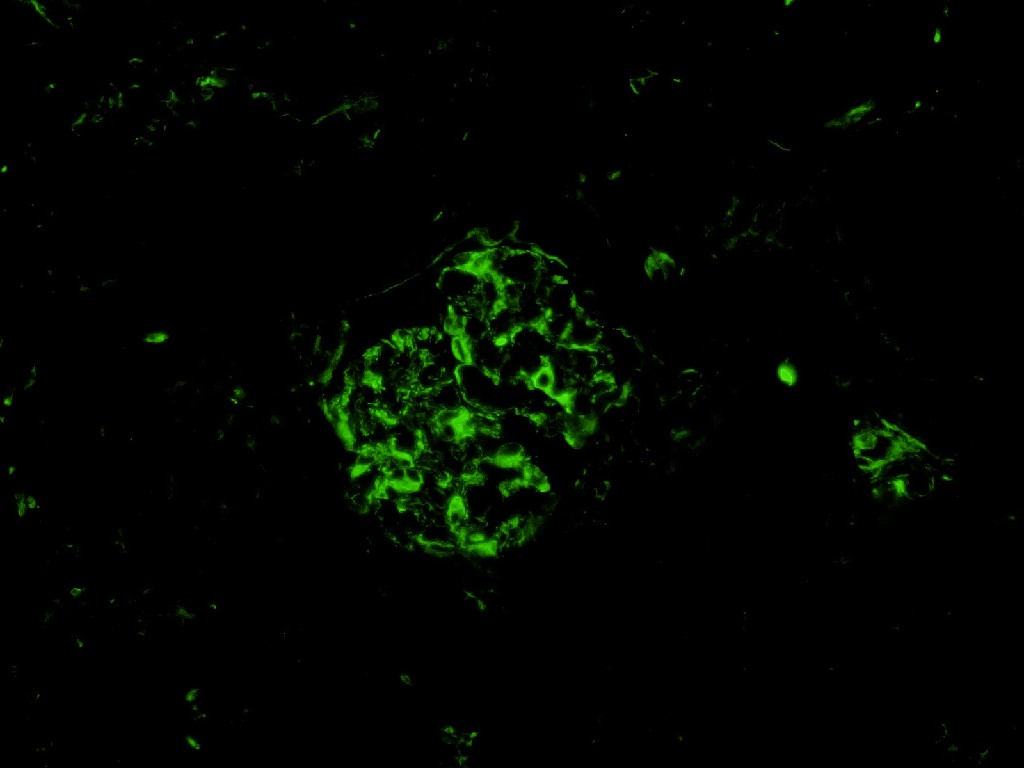

Supplement: Supplementary file 2 — Source data Fig. 1 [file 44321_2025_315_MOESM2_ESM.zip › Figure 1/F1A/1-GLDC-PDGFRbeta/Lee II/3 (3).jpg]

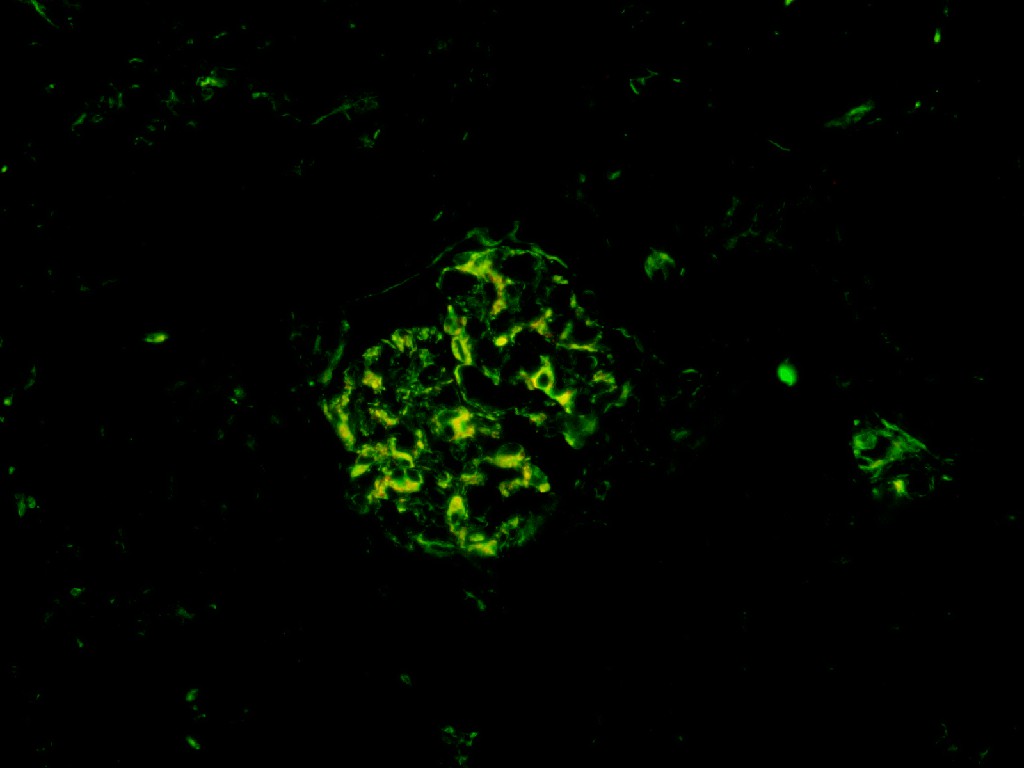

Supplement: Supplementary file 2 — Source data Fig. 1 [file 44321_2025_315_MOESM2_ESM.zip › Figure 1/F1A/1-GLDC-PDGFRbeta/Lee II/3 (4).jpg]

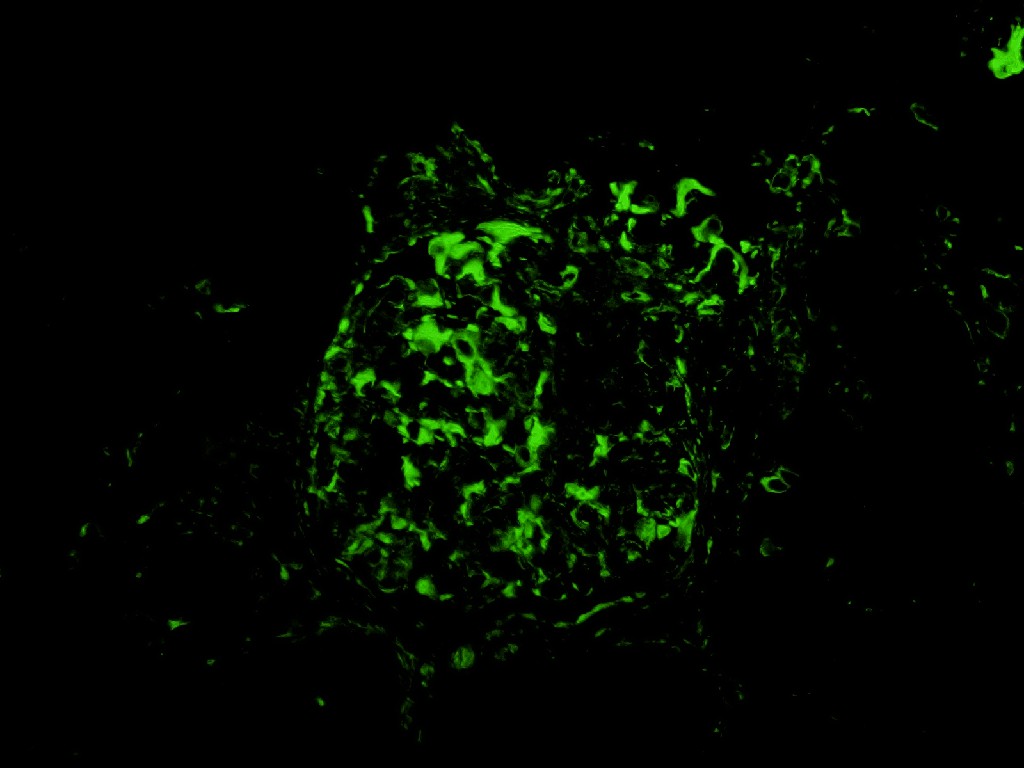

Supplement: Supplementary file 2 — Source data Fig. 1 [file 44321_2025_315_MOESM2_ESM.zip › Figure 1/F1A/1-GLDC-PDGFRbeta/Lee II/4 (1).jpg]

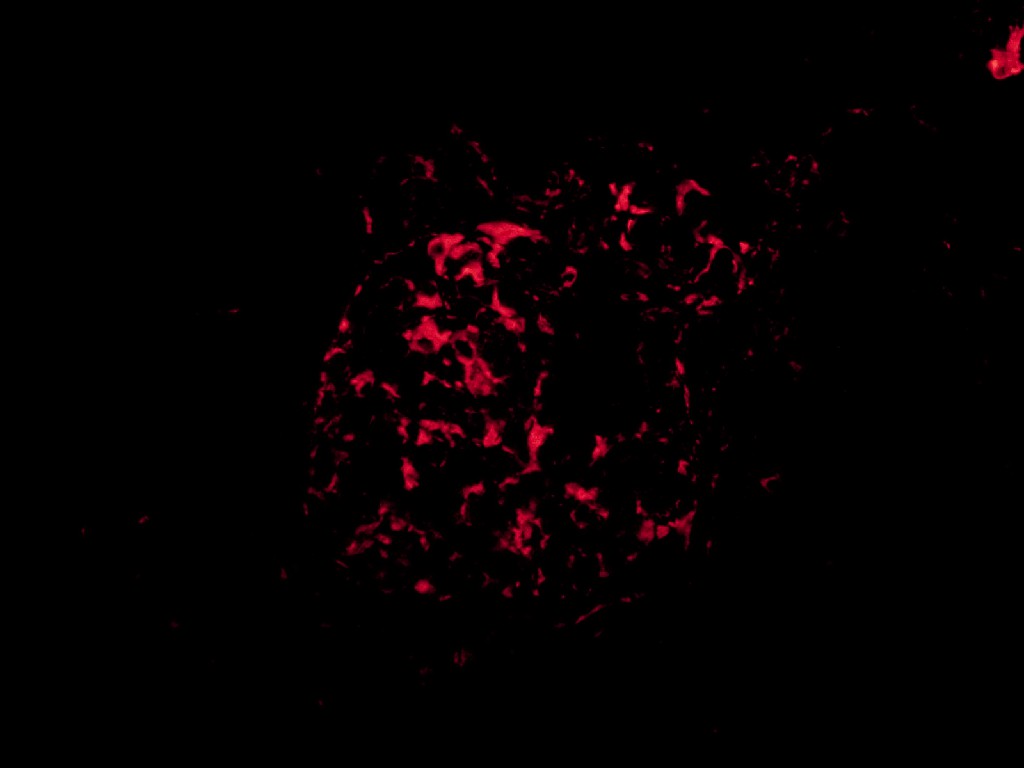

Supplement: Supplementary file 2 — Source data Fig. 1 [file 44321_2025_315_MOESM2_ESM.zip › Figure 1/F1A/1-GLDC-PDGFRbeta/Lee II/4 (2).jpg]

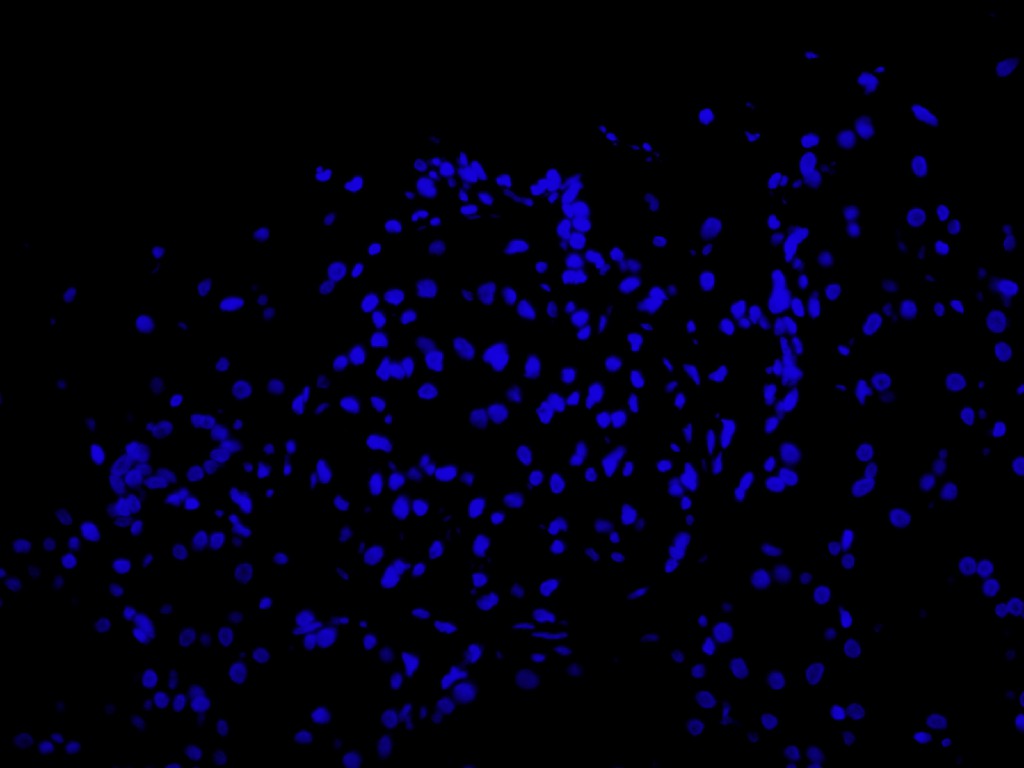

Supplement: Supplementary file 2 — Source data Fig. 1 [file 44321_2025_315_MOESM2_ESM.zip › Figure 1/F1A/1-GLDC-PDGFRbeta/Lee II/4 (3).jpg]

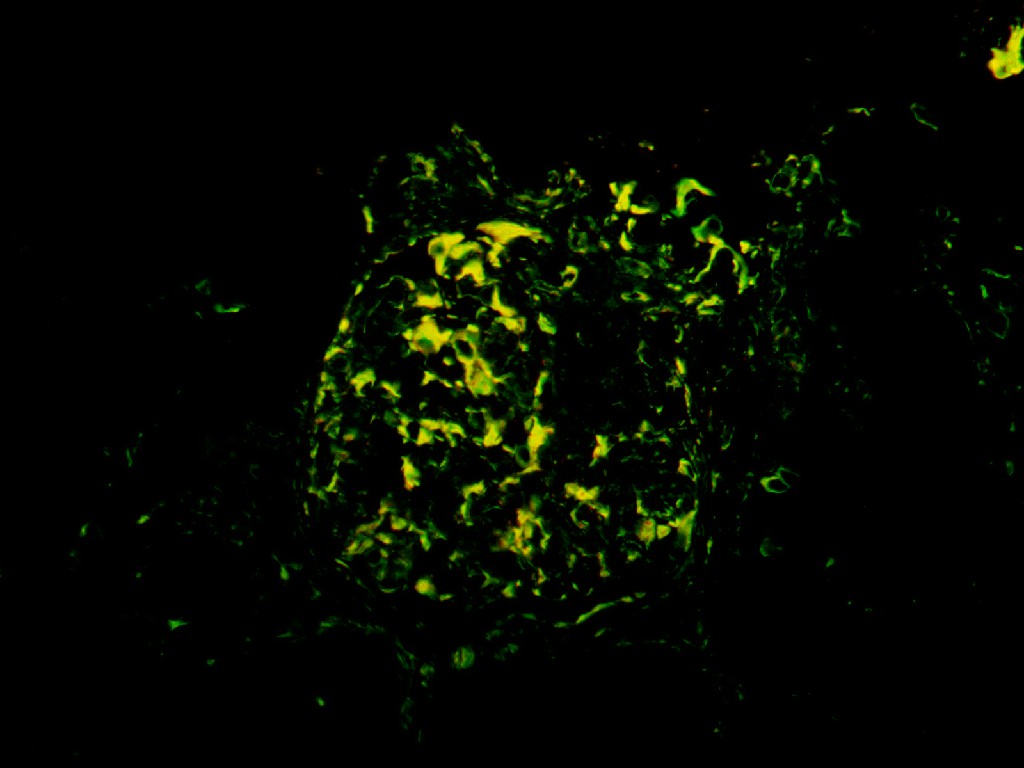

Supplement: Supplementary file 2 — Source data Fig. 1 [file 44321_2025_315_MOESM2_ESM.zip › Figure 1/F1A/1-GLDC-PDGFRbeta/Lee II/4 (4).jpg]

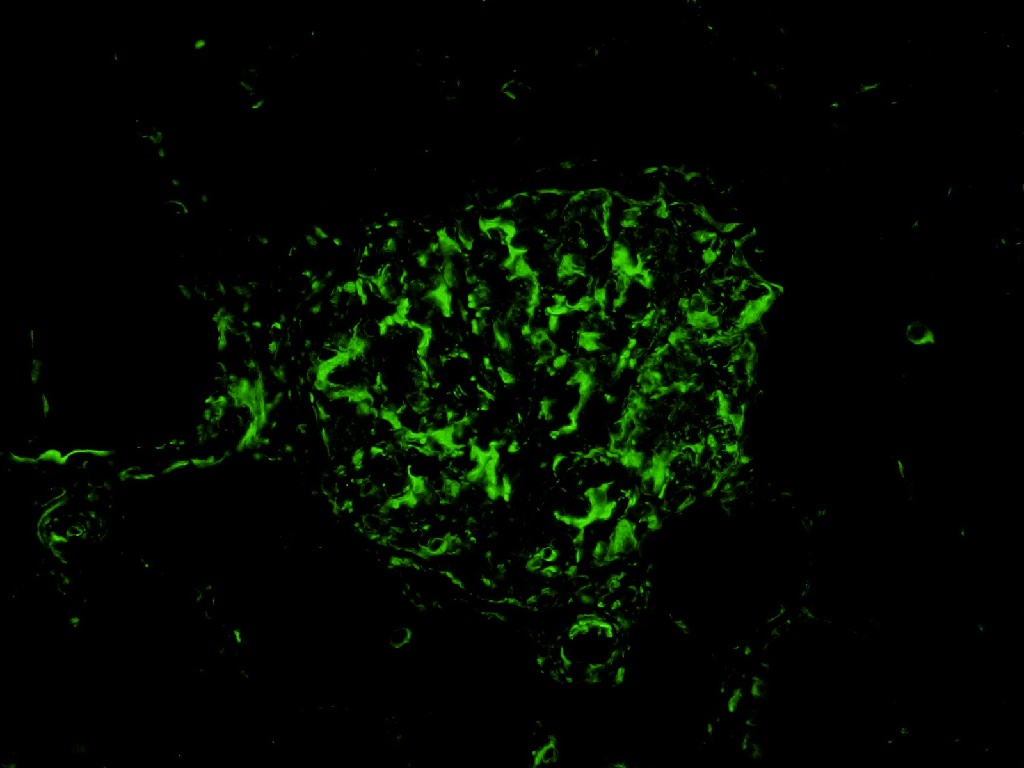

Supplement: Supplementary file 2 — Source data Fig. 1 [file 44321_2025_315_MOESM2_ESM.zip › Figure 1/F1A/1-GLDC-PDGFRbeta/Lee II/5 (1).jpg]

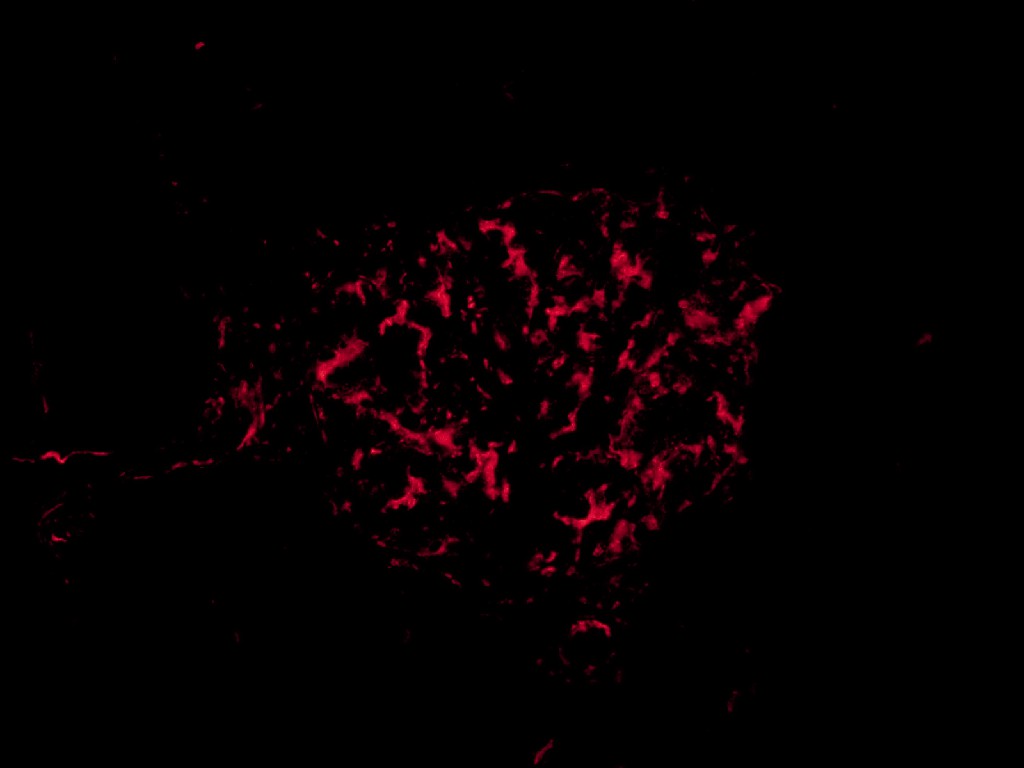

Supplement: Supplementary file 2 — Source data Fig. 1 [file 44321_2025_315_MOESM2_ESM.zip › Figure 1/F1A/1-GLDC-PDGFRbeta/Lee II/5 (2).jpg]

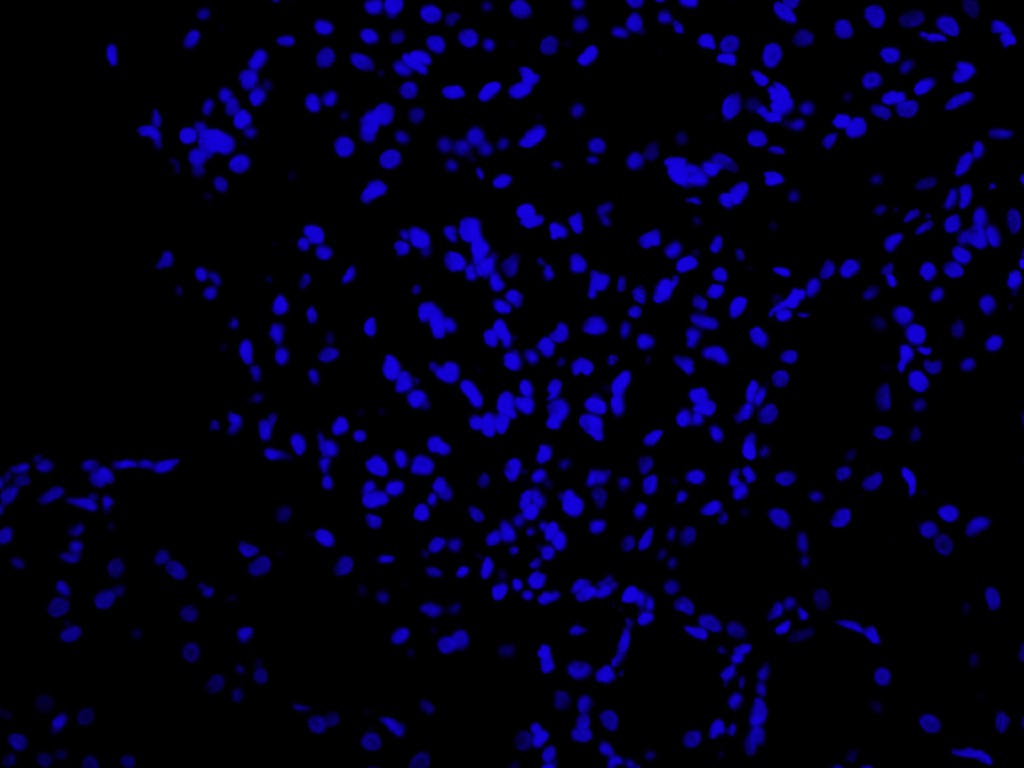

Supplement: Supplementary file 2 — Source data Fig. 1 [file 44321_2025_315_MOESM2_ESM.zip › Figure 1/F1A/1-GLDC-PDGFRbeta/Lee II/5 (3).jpg]

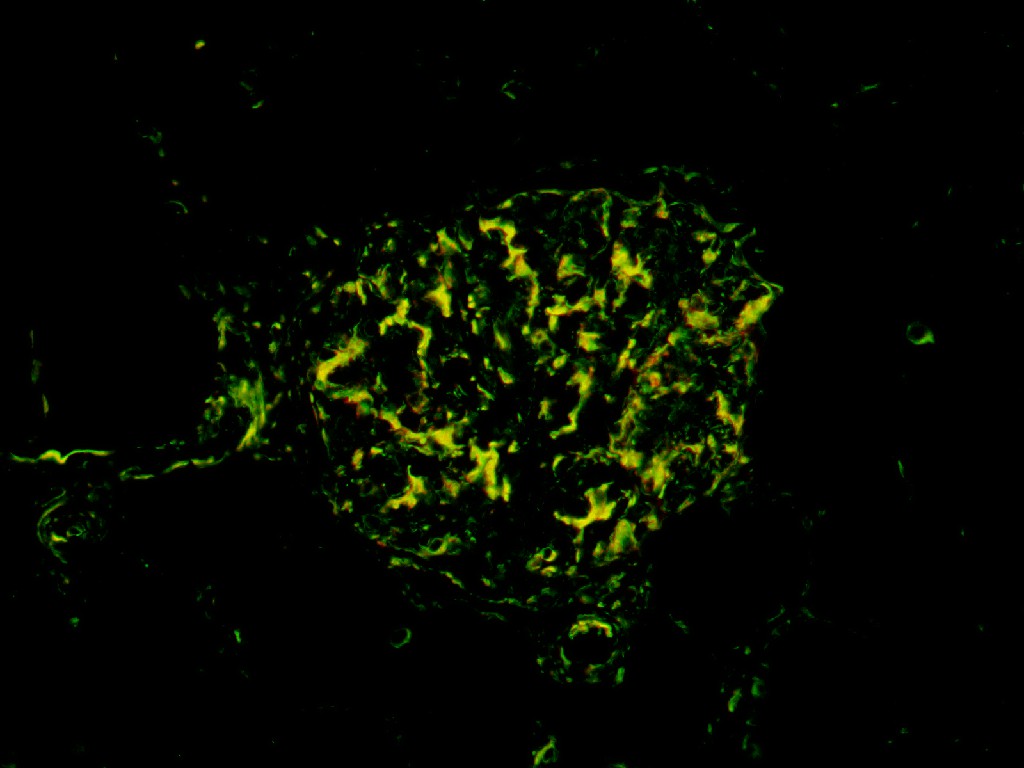

Supplement: Supplementary file 2 — Source data Fig. 1 [file 44321_2025_315_MOESM2_ESM.zip › Figure 1/F1A/1-GLDC-PDGFRbeta/Lee II/5 (4).jpg]

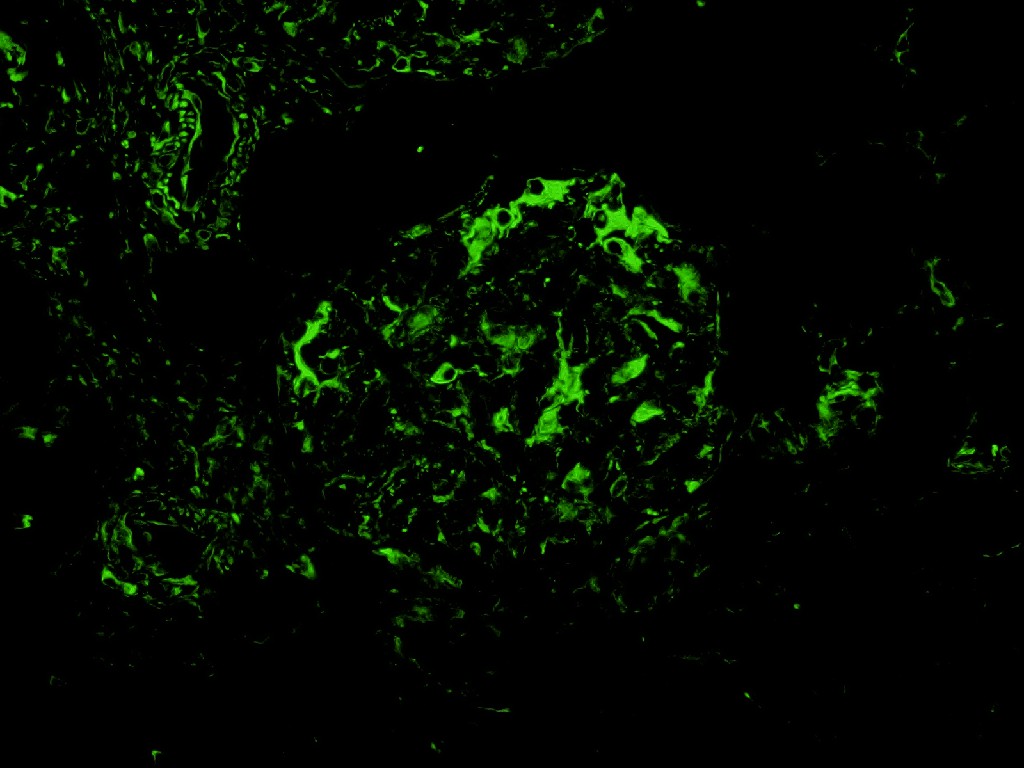

Supplement: Supplementary file 2 — Source data Fig. 1 [file 44321_2025_315_MOESM2_ESM.zip › Figure 1/F1A/1-GLDC-PDGFRbeta/Lee II/6 (1).jpg]

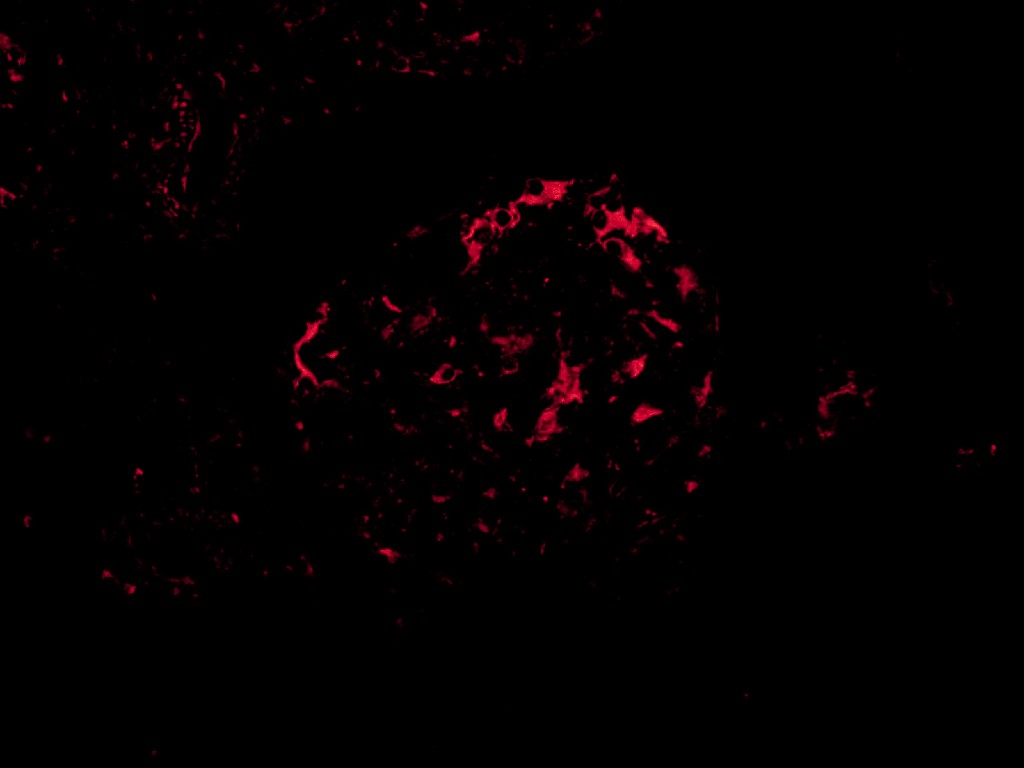

Supplement: Supplementary file 2 — Source data Fig. 1 [file 44321_2025_315_MOESM2_ESM.zip › Figure 1/F1A/1-GLDC-PDGFRbeta/Lee II/6 (2).jpg]

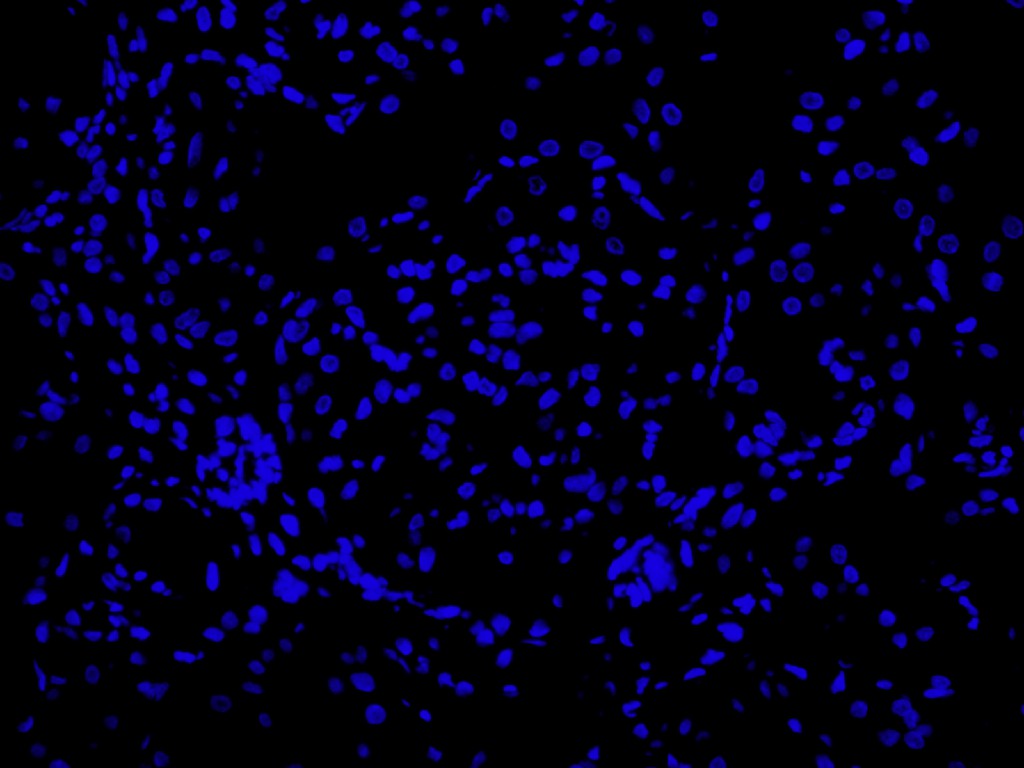

Supplement: Supplementary file 2 — Source data Fig. 1 [file 44321_2025_315_MOESM2_ESM.zip › Figure 1/F1A/1-GLDC-PDGFRbeta/Lee II/6 (3).jpg]

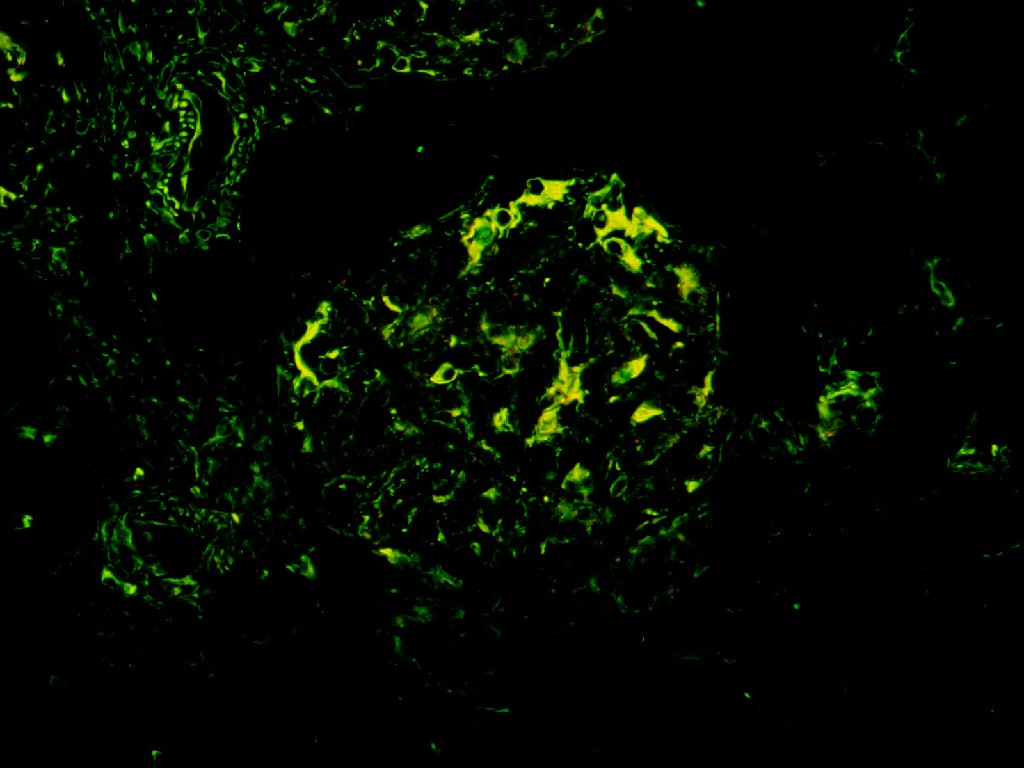

Supplement: Supplementary file 2 — Source data Fig. 1 [file 44321_2025_315_MOESM2_ESM.zip › Figure 1/F1A/1-GLDC-PDGFRbeta/Lee II/6 (4).jpg]

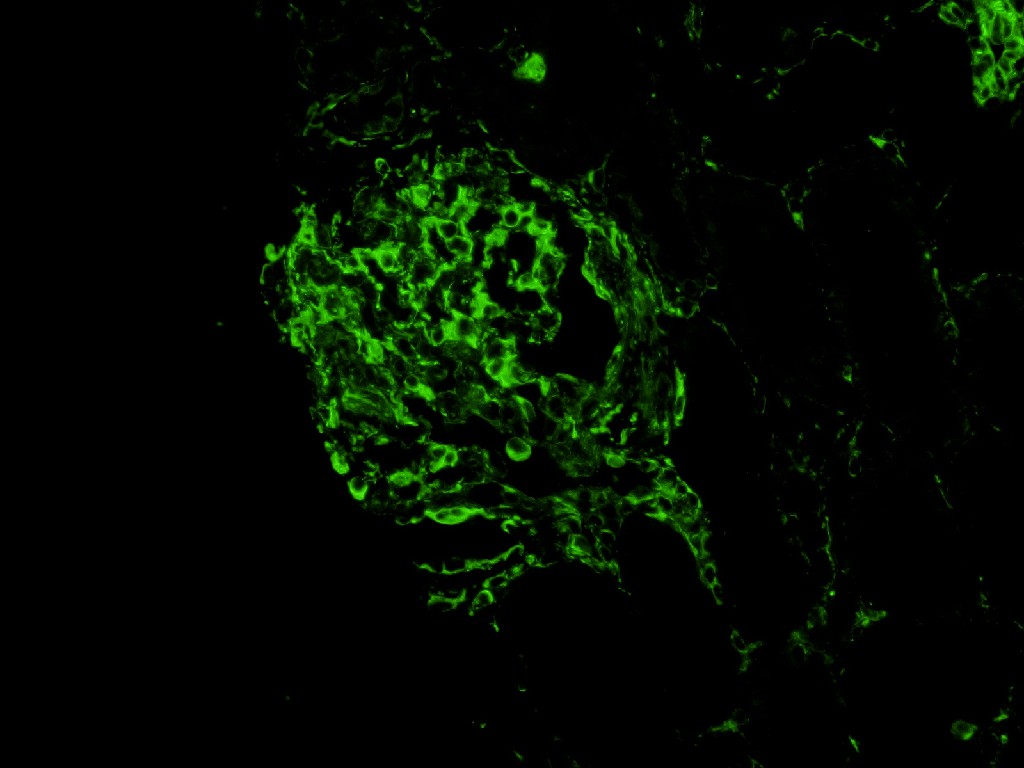

Supplement: Supplementary file 2 — Source data Fig. 1 [file 44321_2025_315_MOESM2_ESM.zip › Figure 1/F1A/1-GLDC-PDGFRbeta/Lee II/7 (1).jpg]

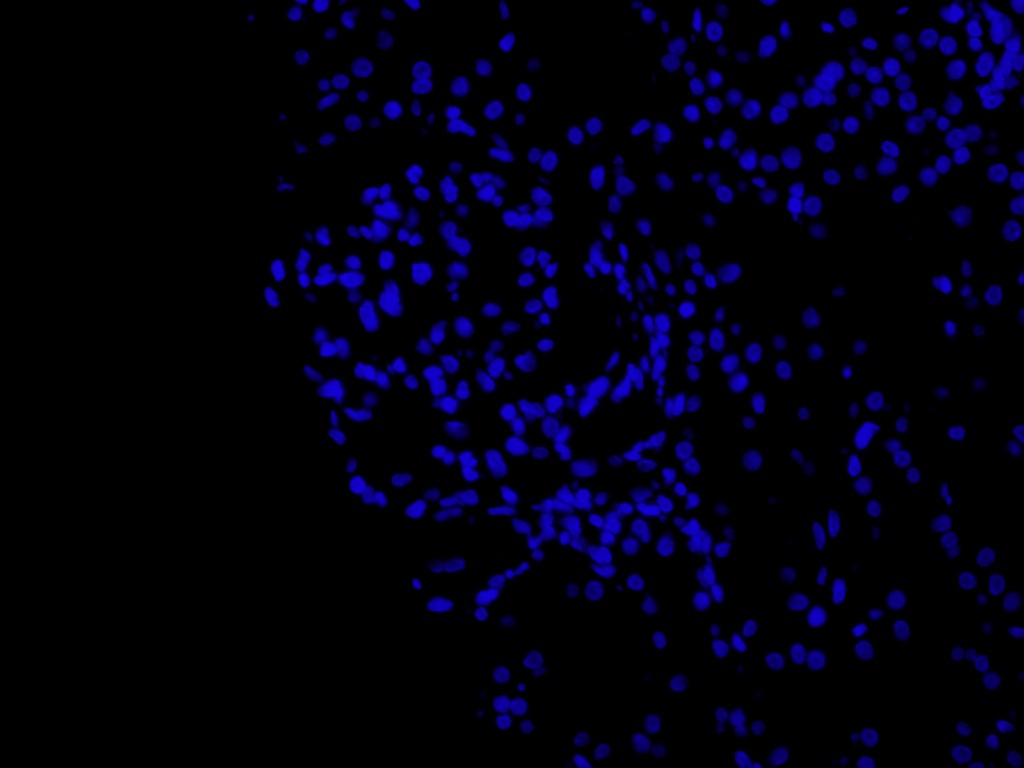

Supplement: Supplementary file 2 — Source data Fig. 1 [file 44321_2025_315_MOESM2_ESM.zip › Figure 1/F1A/1-GLDC-PDGFRbeta/Lee II/7 (2).jpg]

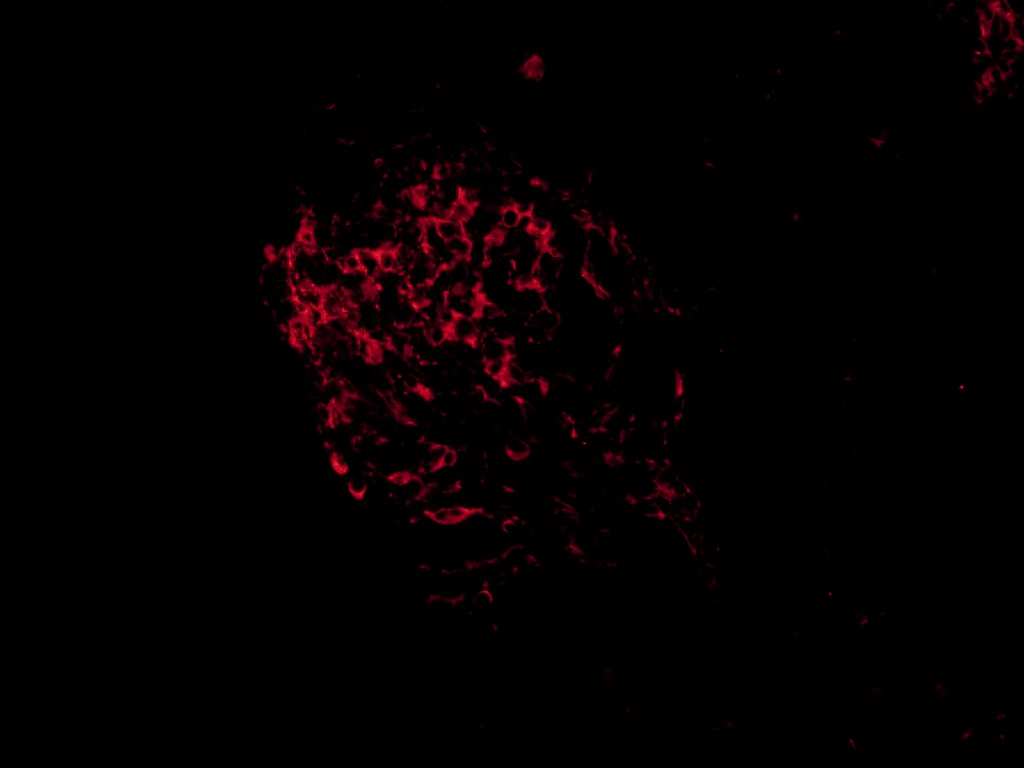

Supplement: Supplementary file 2 — Source data Fig. 1 [file 44321_2025_315_MOESM2_ESM.zip › Figure 1/F1A/1-GLDC-PDGFRbeta/Lee II/7 (3).jpg]

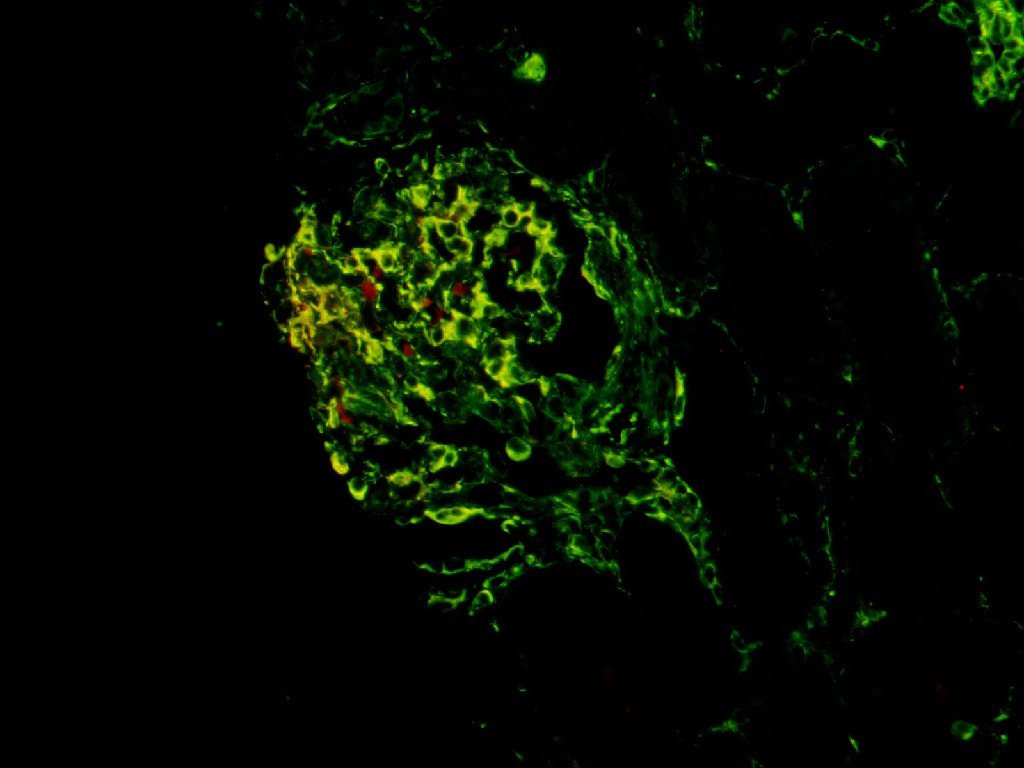

Supplement: Supplementary file 2 — Source data Fig. 1 [file 44321_2025_315_MOESM2_ESM.zip › Figure 1/F1A/1-GLDC-PDGFRbeta/Lee II/7 (4).jpg]

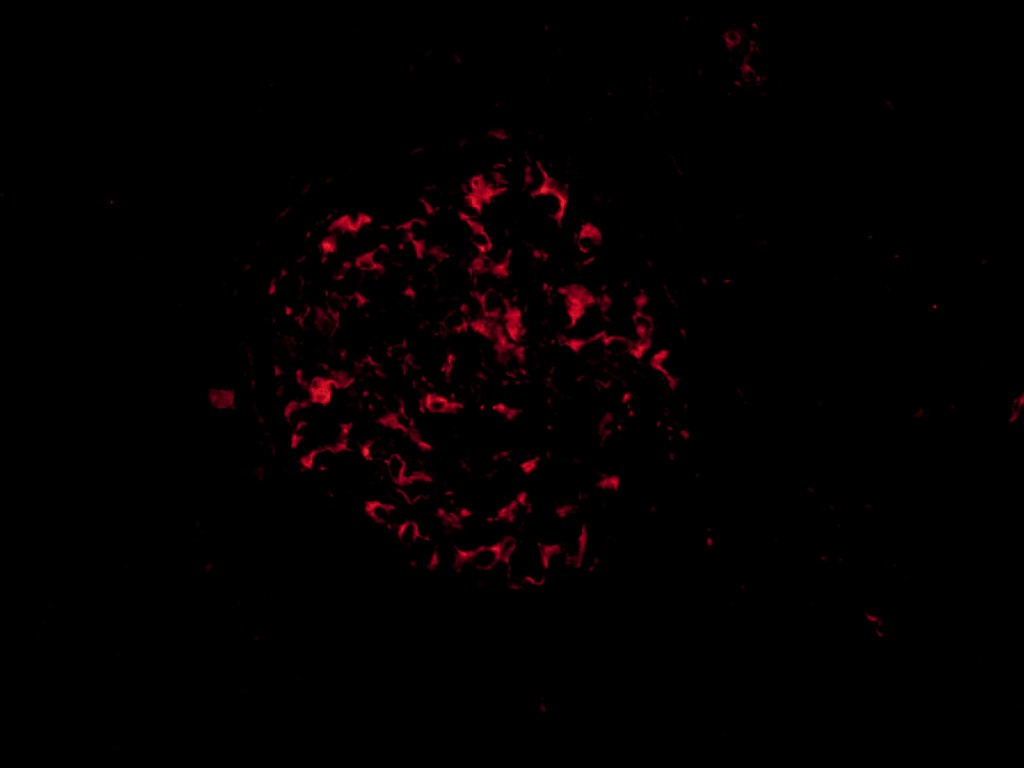

Supplement: Supplementary file 2 — Source data Fig. 1 [file 44321_2025_315_MOESM2_ESM.zip › Figure 1/F1A/1-GLDC-PDGFRbeta/Lee II/8 (1).jpg]

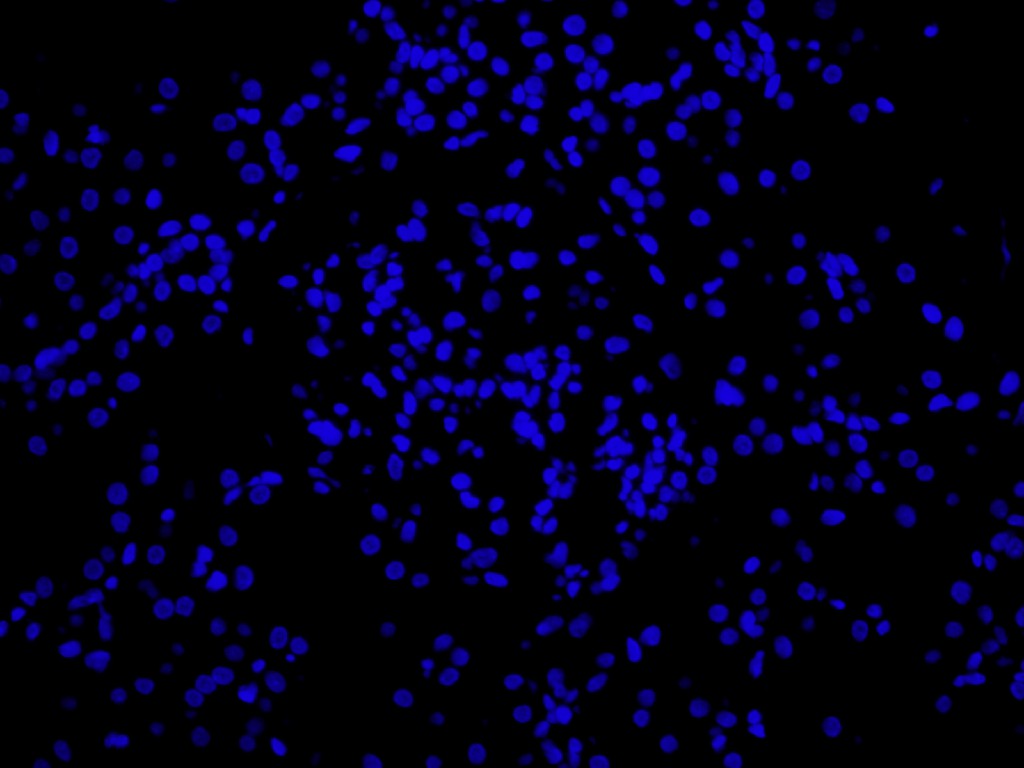

Supplement: Supplementary file 2 — Source data Fig. 1 [file 44321_2025_315_MOESM2_ESM.zip › Figure 1/F1A/1-GLDC-PDGFRbeta/Lee II/8 (2).jpg]

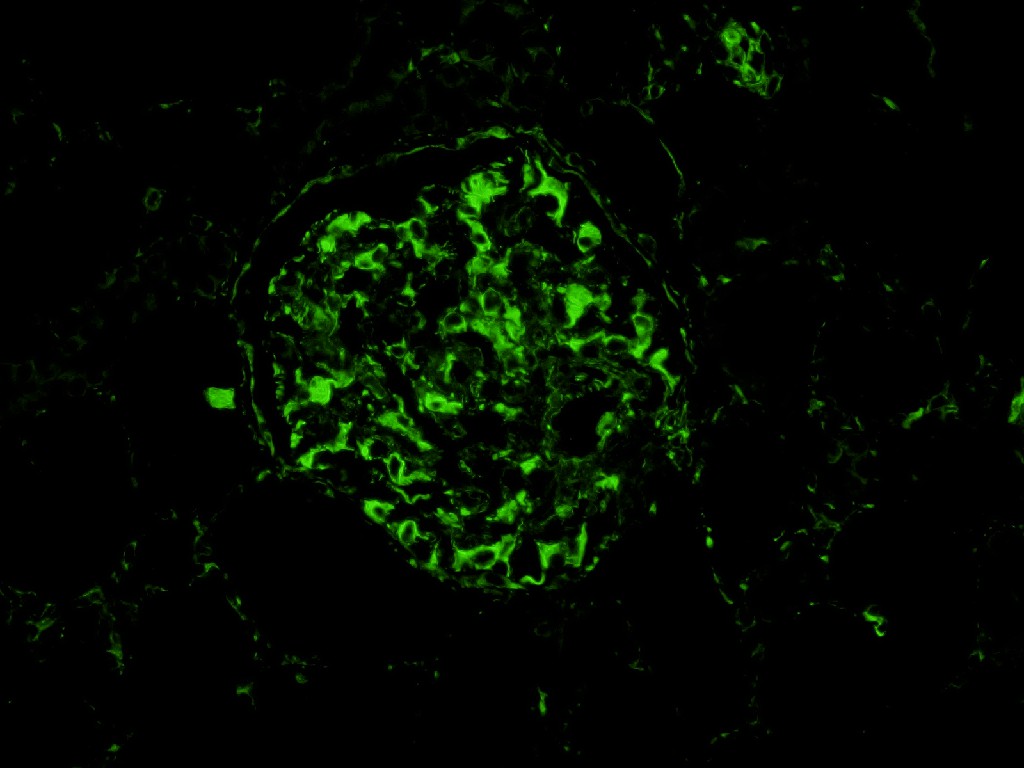

Supplement: Supplementary file 2 — Source data Fig. 1 [file 44321_2025_315_MOESM2_ESM.zip › Figure 1/F1A/1-GLDC-PDGFRbeta/Lee II/8 (3).jpg]

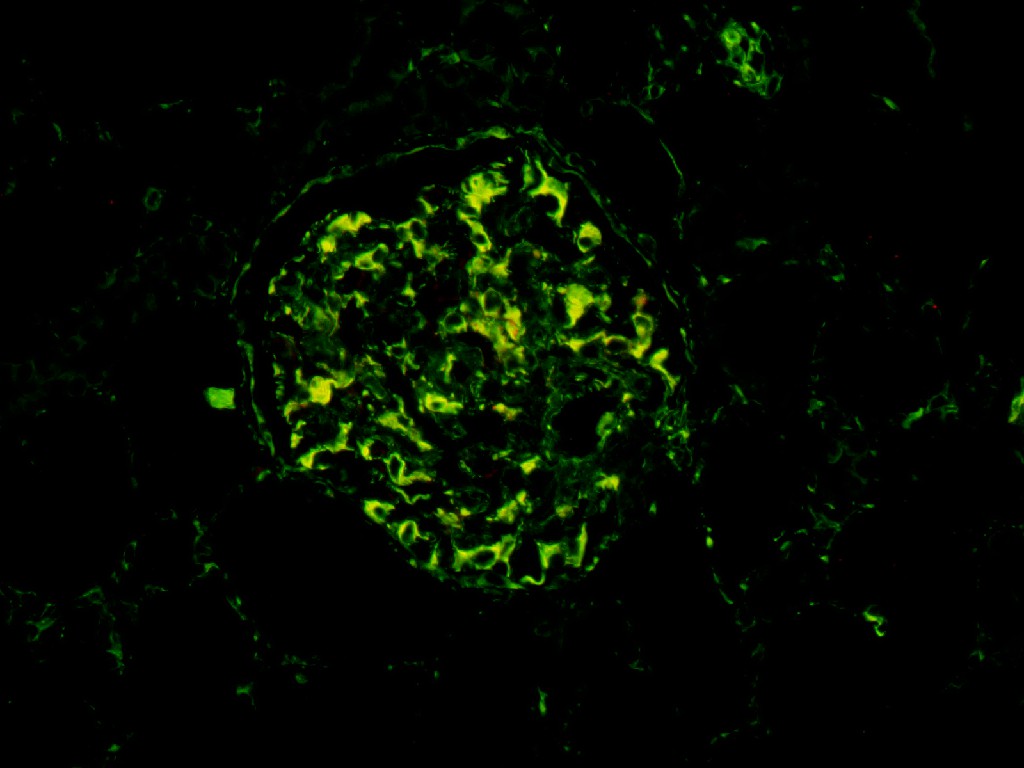

Supplement: Supplementary file 2 — Source data Fig. 1 [file 44321_2025_315_MOESM2_ESM.zip › Figure 1/F1A/1-GLDC-PDGFRbeta/Lee II/8 (4).jpg]

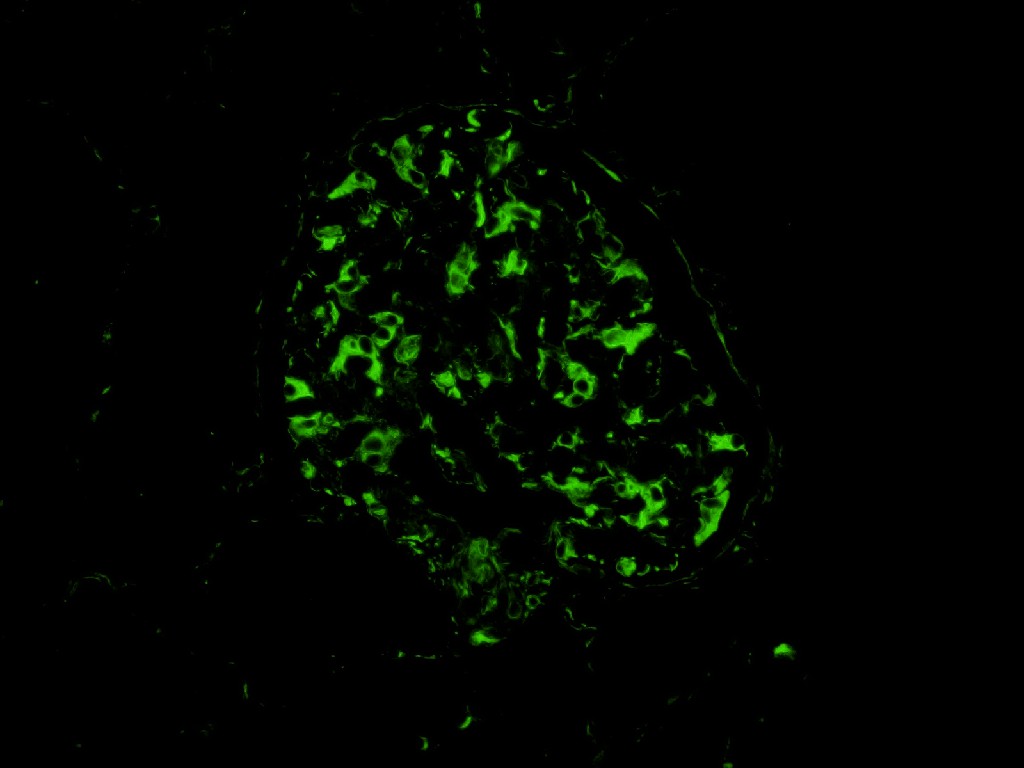

Supplement: Supplementary file 2 — Source data Fig. 1 [file 44321_2025_315_MOESM2_ESM.zip › Figure 1/F1A/1-GLDC-PDGFRbeta/Lee II/9 (1).jpg]

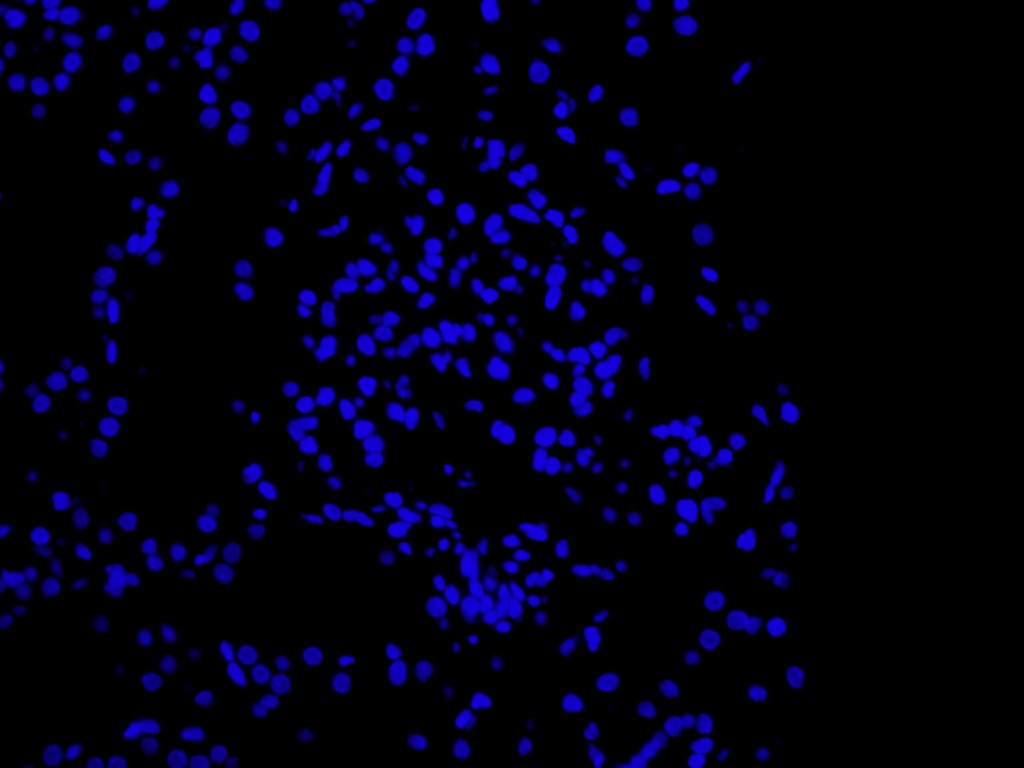

Supplement: Supplementary file 2 — Source data Fig. 1 [file 44321_2025_315_MOESM2_ESM.zip › Figure 1/F1A/1-GLDC-PDGFRbeta/Lee II/9 (2).jpg]

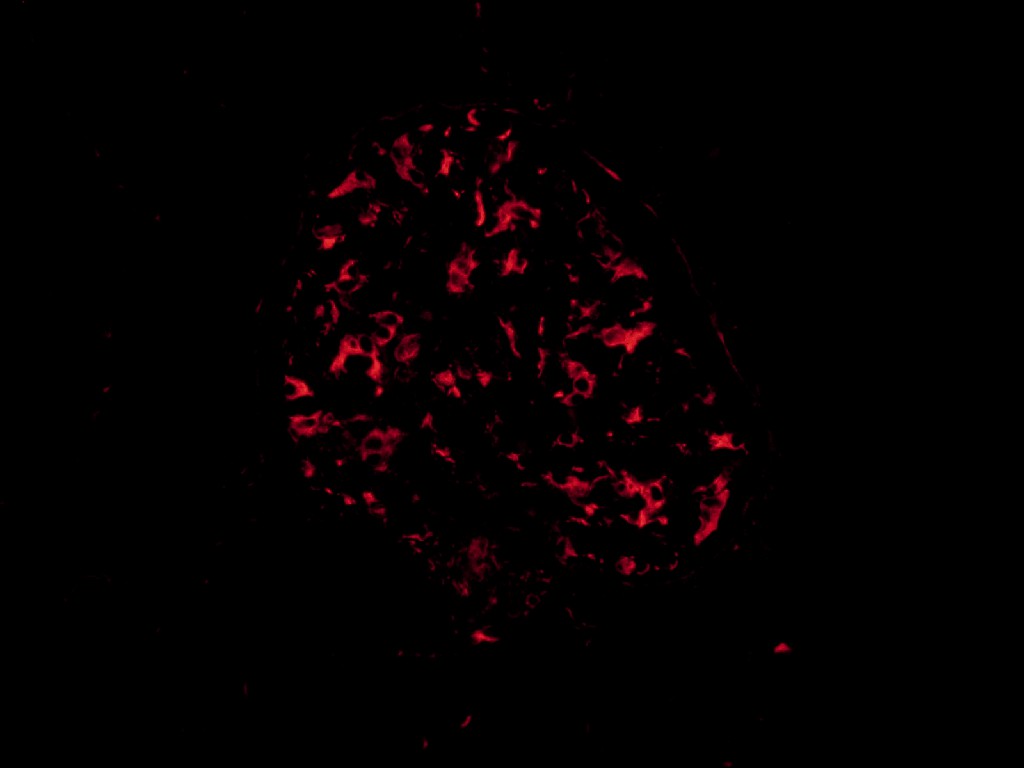

Supplement: Supplementary file 2 — Source data Fig. 1 [file 44321_2025_315_MOESM2_ESM.zip › Figure 1/F1A/1-GLDC-PDGFRbeta/Lee II/9 (3).jpg]

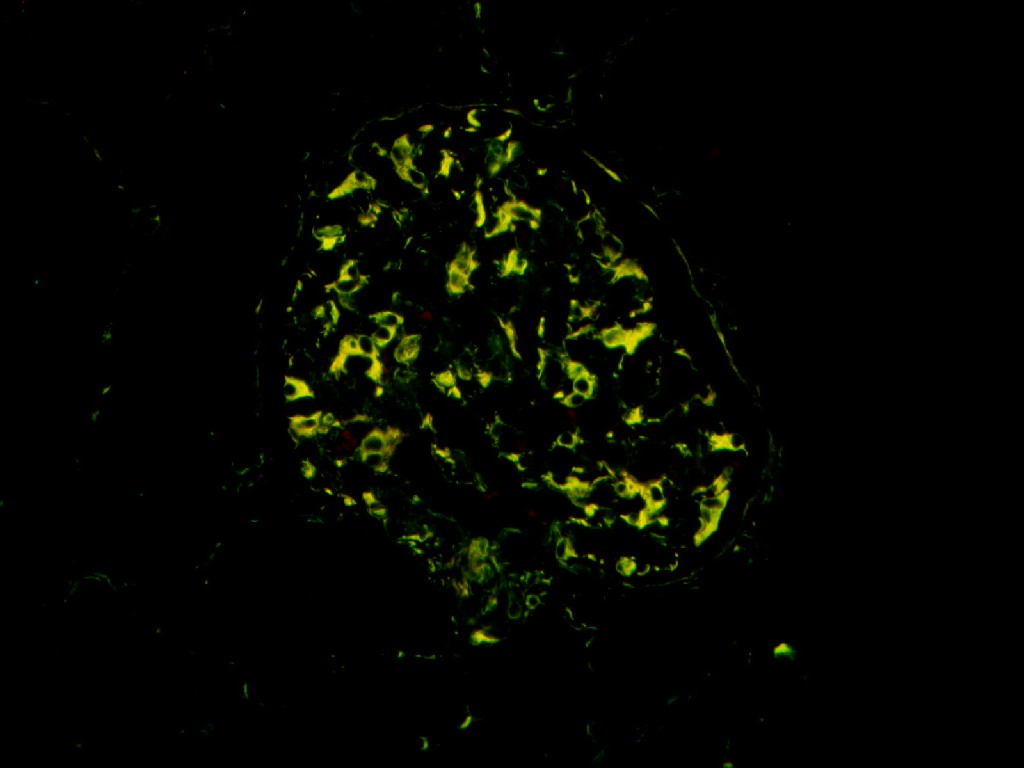

Supplement: Supplementary file 2 — Source data Fig. 1 [file 44321_2025_315_MOESM2_ESM.zip › Figure 1/F1A/1-GLDC-PDGFRbeta/Lee II/9 (4).jpg]

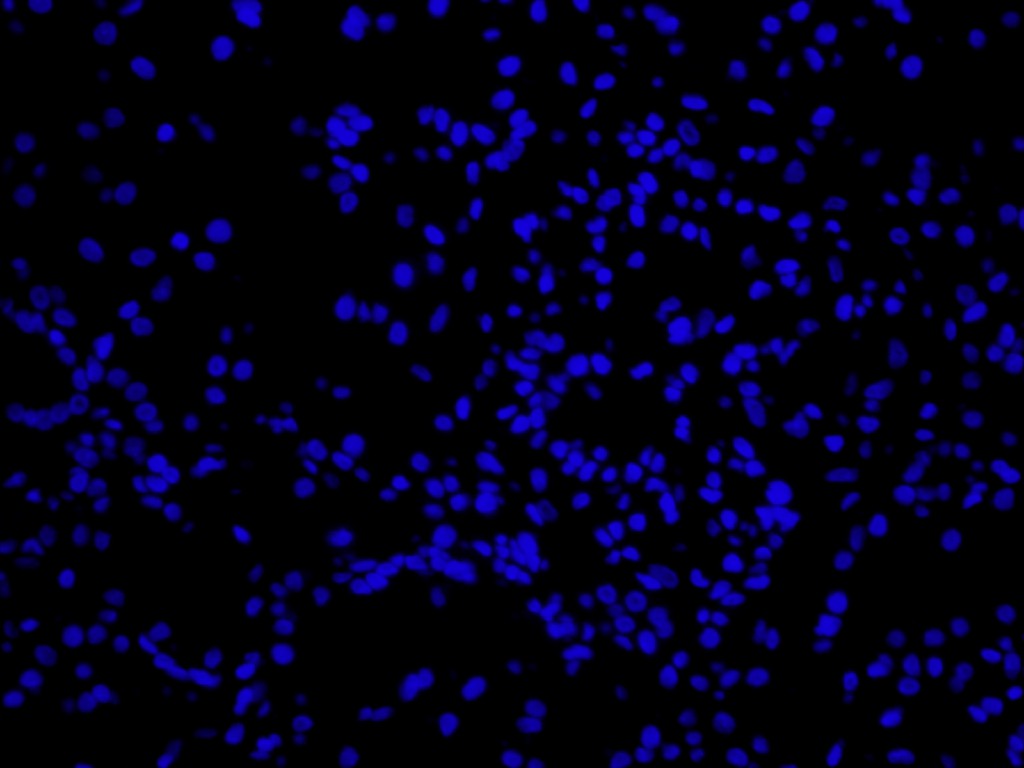

Supplement: Supplementary file 2 — Source data Fig. 1 [file 44321_2025_315_MOESM2_ESM.zip › Figure 1/F1A/1-GLDC-PDGFRbeta/Lee III/1 (1).jpg]

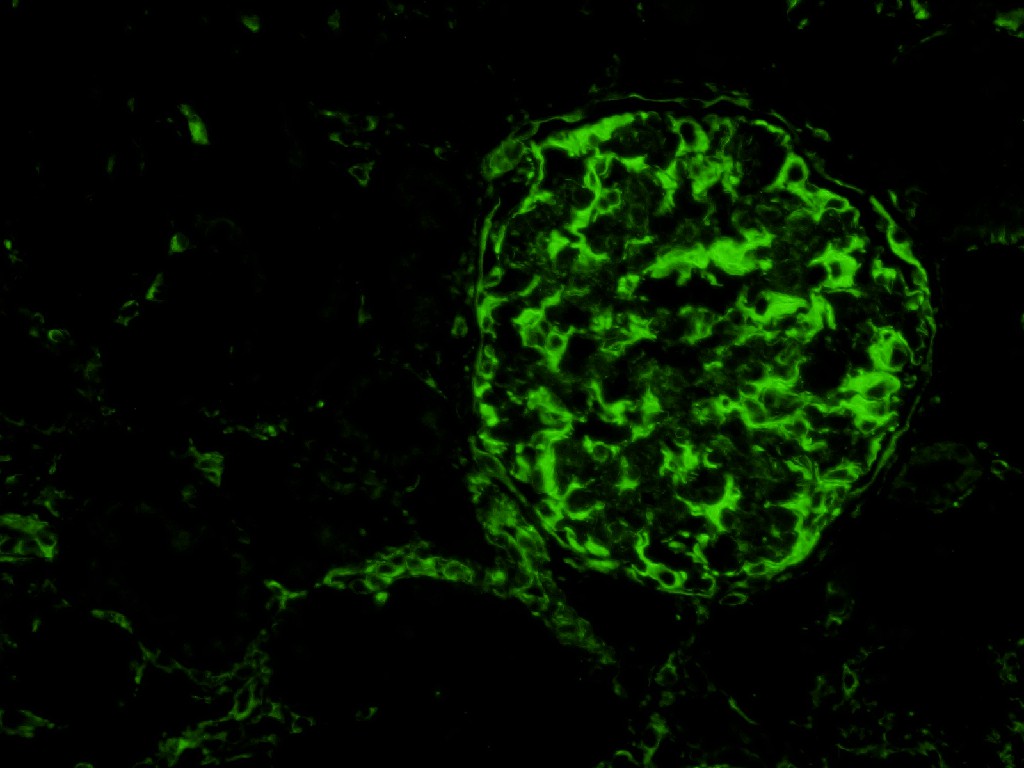

Supplement: Supplementary file 2 — Source data Fig. 1 [file 44321_2025_315_MOESM2_ESM.zip › Figure 1/F1A/1-GLDC-PDGFRbeta/Lee III/1 (2).jpg]

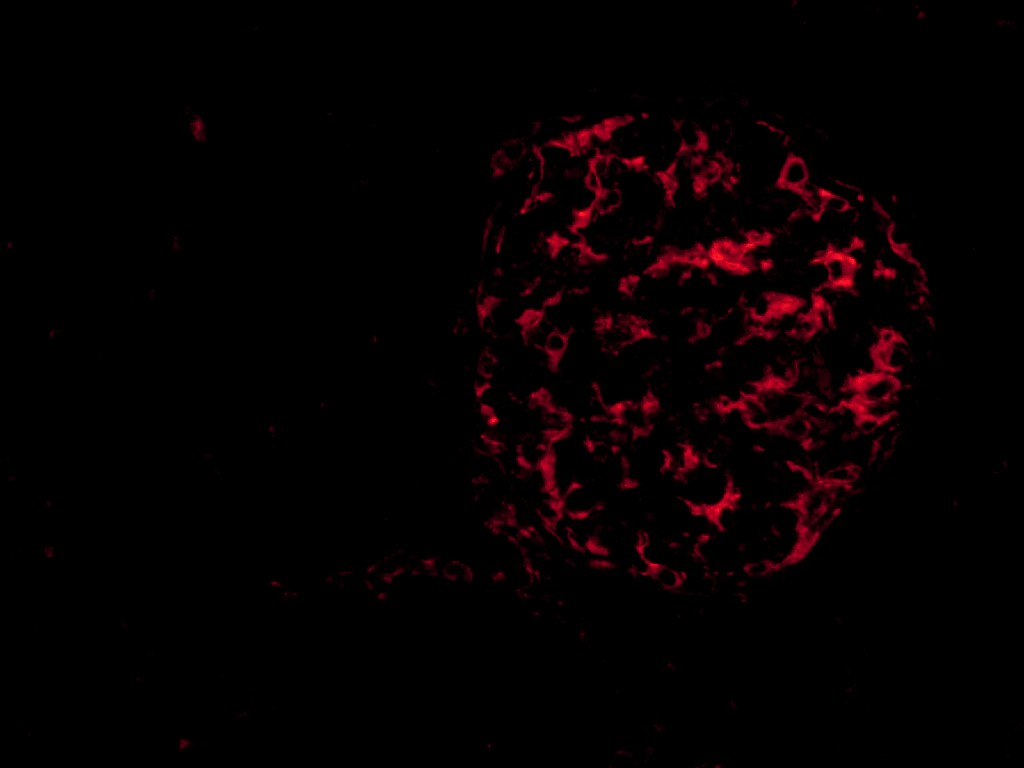

Supplement: Supplementary file 2 — Source data Fig. 1 [file 44321_2025_315_MOESM2_ESM.zip › Figure 1/F1A/1-GLDC-PDGFRbeta/Lee III/1 (3).jpg]

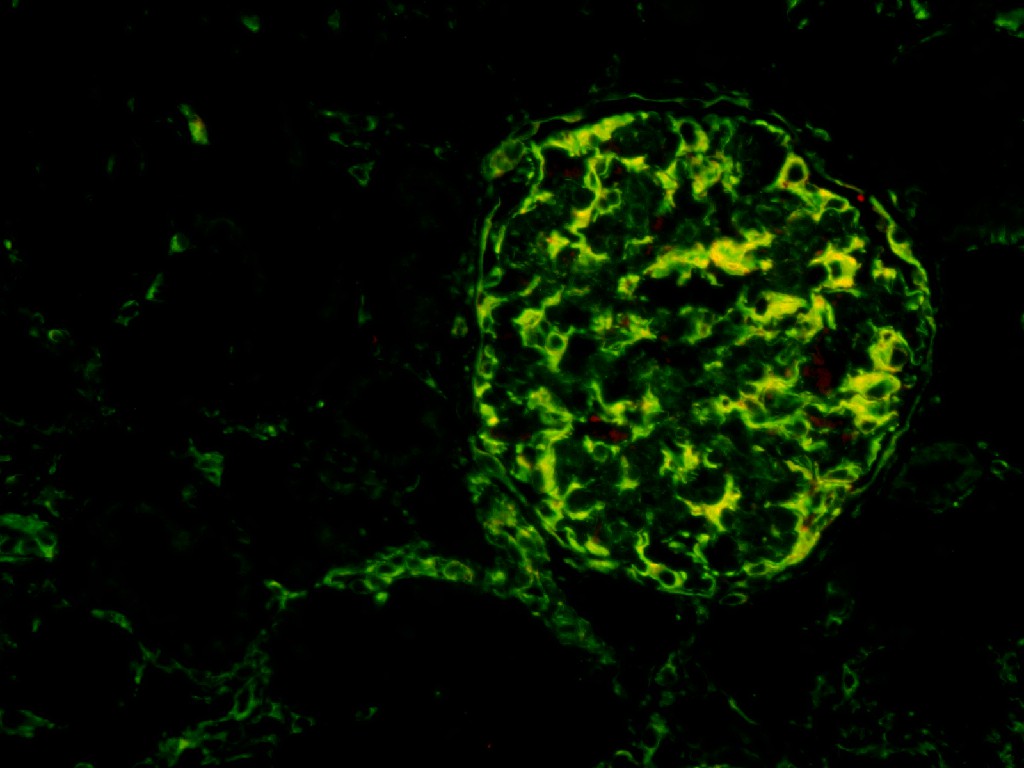

Supplement: Supplementary file 2 — Source data Fig. 1 [file 44321_2025_315_MOESM2_ESM.zip › Figure 1/F1A/1-GLDC-PDGFRbeta/Lee III/1 (4).jpg]

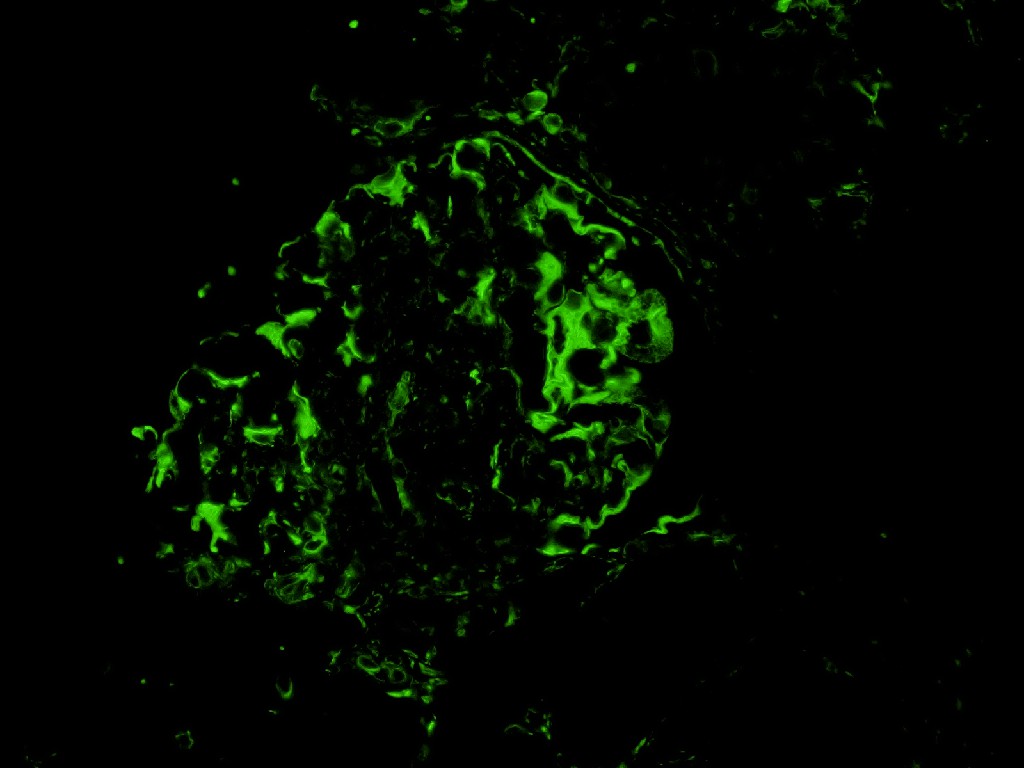

Supplement: Supplementary file 2 — Source data Fig. 1 [file 44321_2025_315_MOESM2_ESM.zip › Figure 1/F1A/1-GLDC-PDGFRbeta/Lee III/10 (1).jpg]

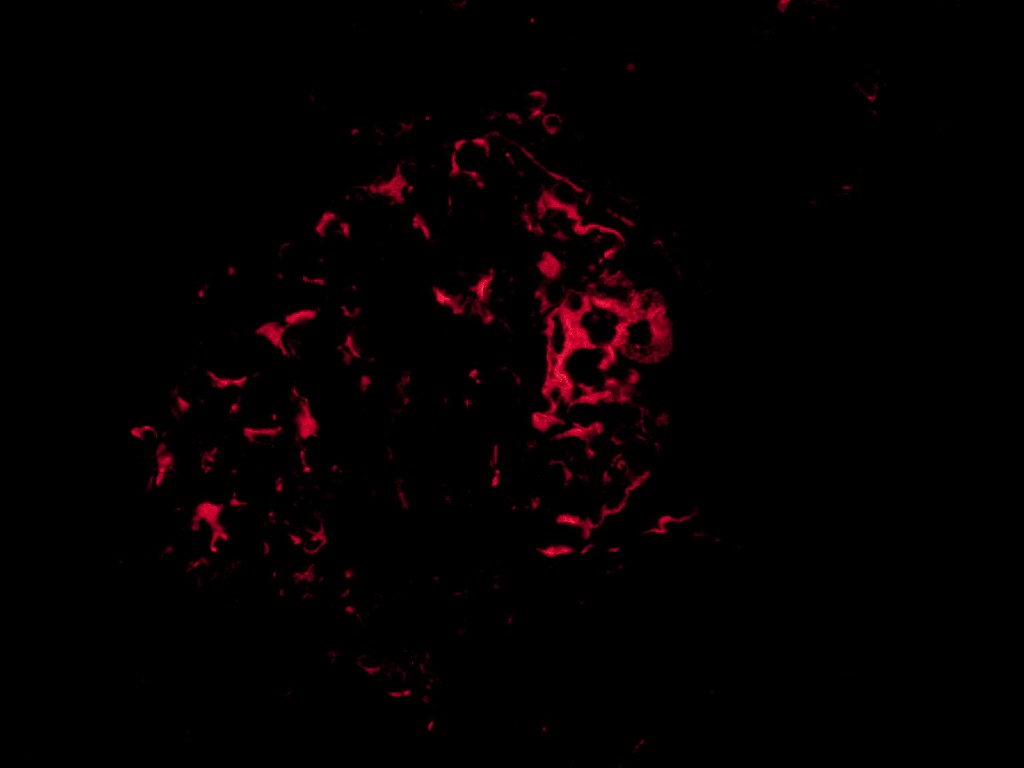

Supplement: Supplementary file 2 — Source data Fig. 1 [file 44321_2025_315_MOESM2_ESM.zip › Figure 1/F1A/1-GLDC-PDGFRbeta/Lee III/10 (2).jpg]

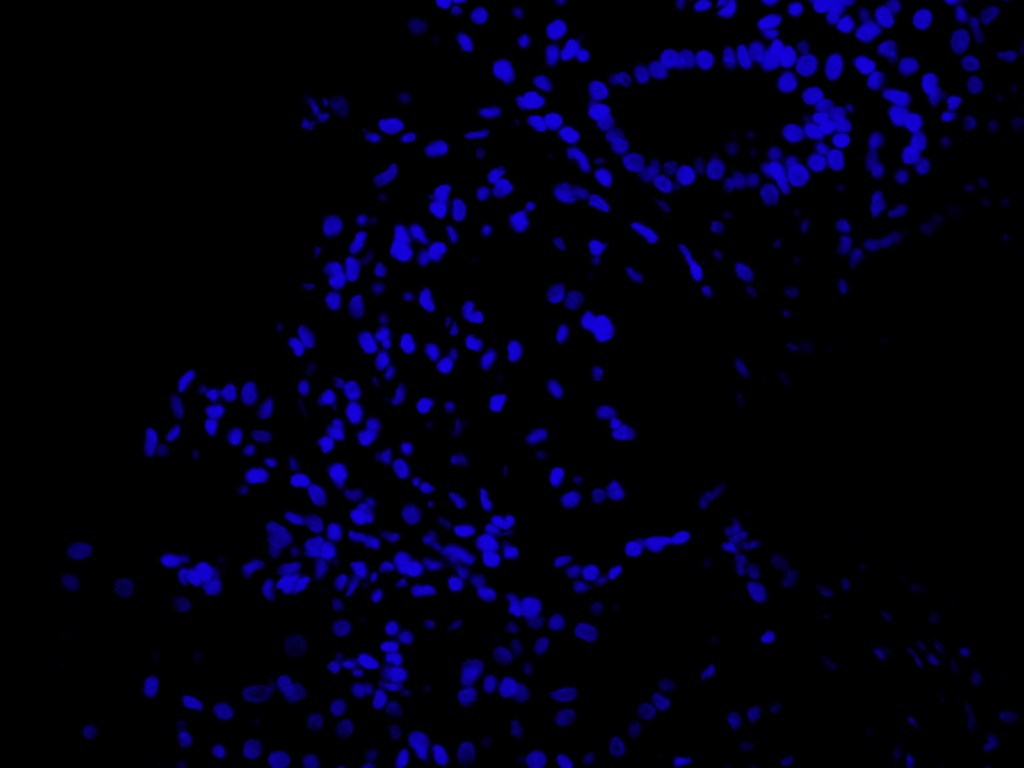

Supplement: Supplementary file 2 — Source data Fig. 1 [file 44321_2025_315_MOESM2_ESM.zip › Figure 1/F1A/1-GLDC-PDGFRbeta/Lee III/10 (3).jpg]

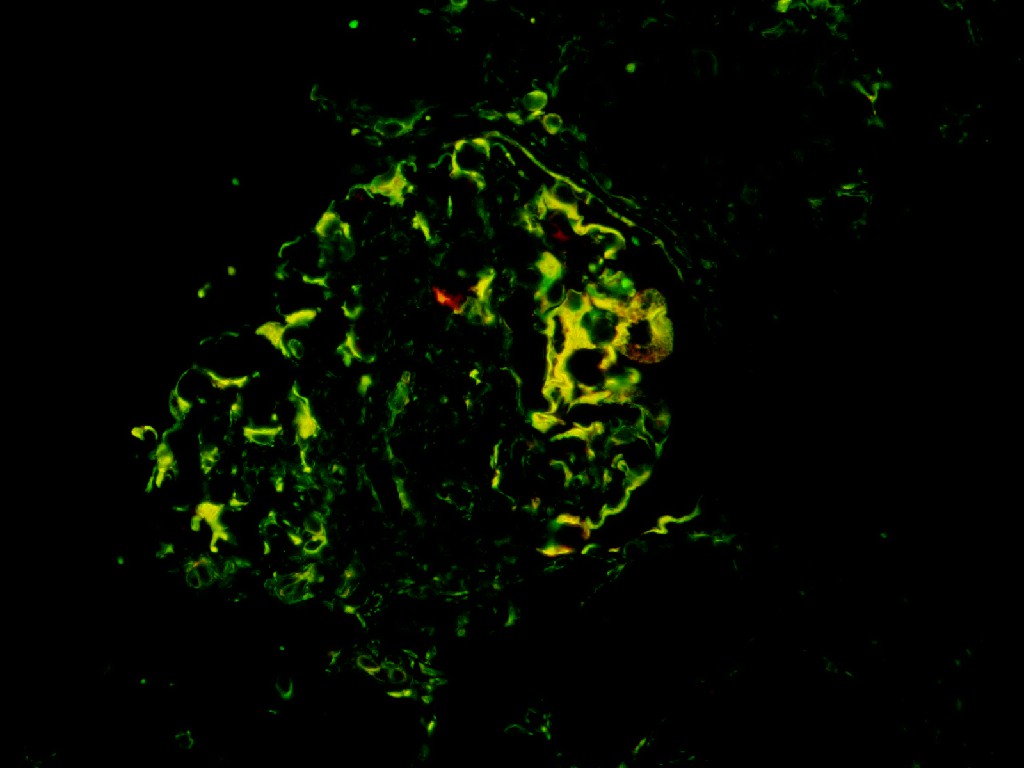

Supplement: Supplementary file 2 — Source data Fig. 1 [file 44321_2025_315_MOESM2_ESM.zip › Figure 1/F1A/1-GLDC-PDGFRbeta/Lee III/10 (4).jpg]

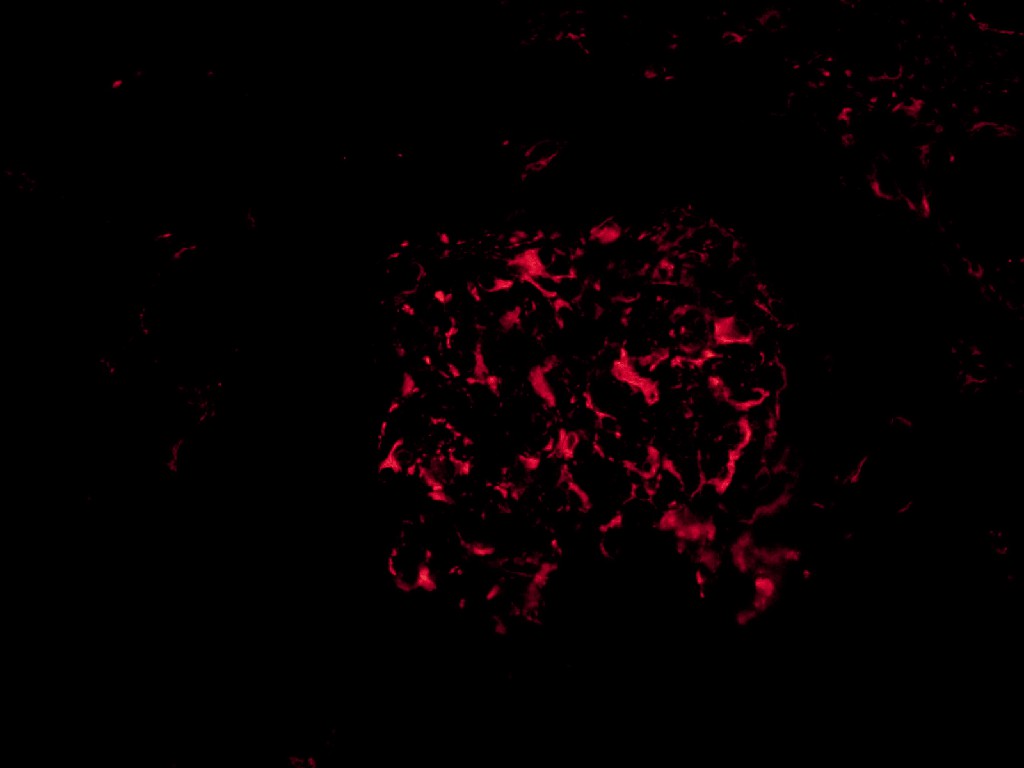

Supplement: Supplementary file 2 — Source data Fig. 1 [file 44321_2025_315_MOESM2_ESM.zip › Figure 1/F1A/1-GLDC-PDGFRbeta/Lee III/11 (1).jpg]

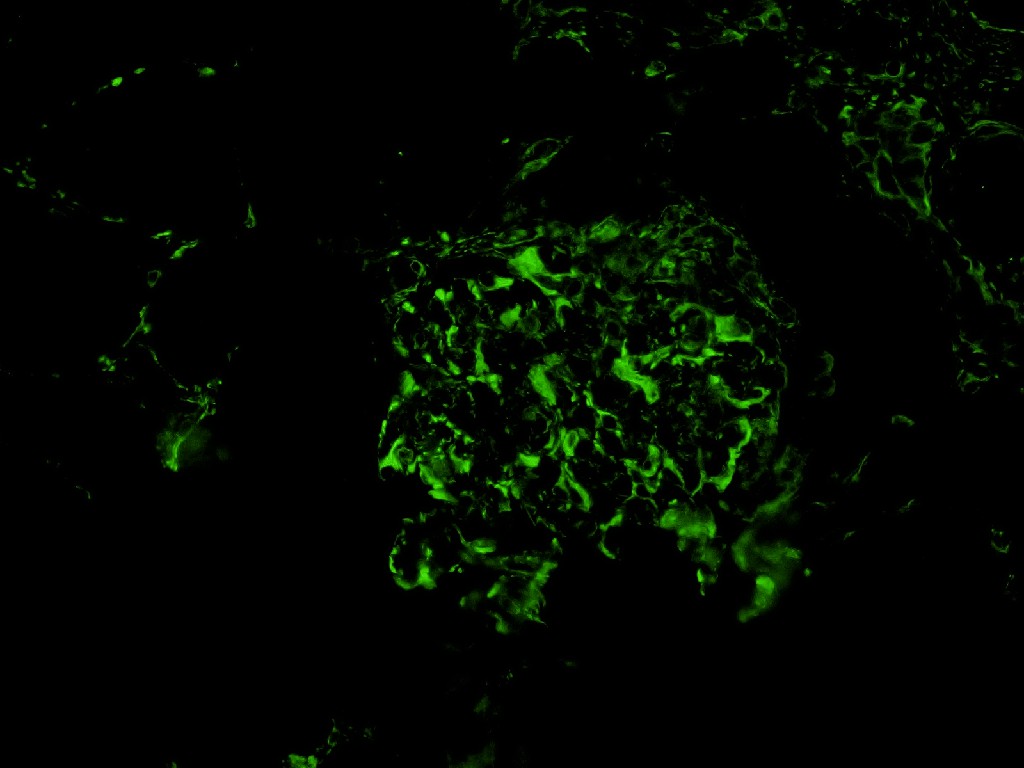

Supplement: Supplementary file 2 — Source data Fig. 1 [file 44321_2025_315_MOESM2_ESM.zip › Figure 1/F1A/1-GLDC-PDGFRbeta/Lee III/11 (2).jpg]

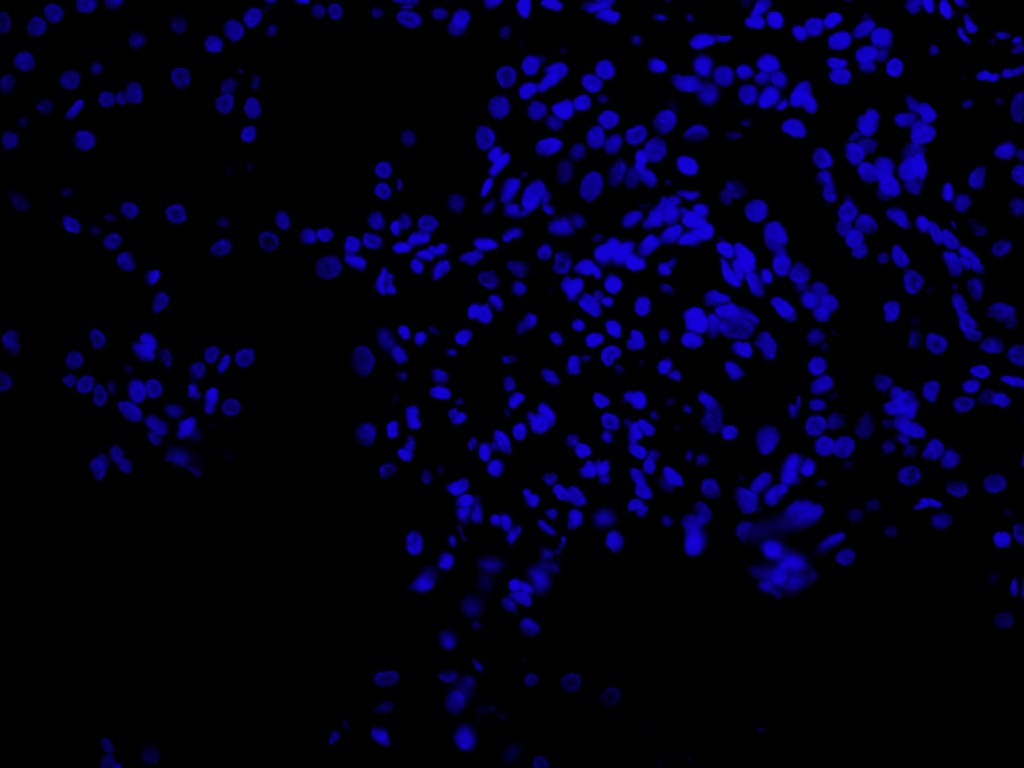

Supplement: Supplementary file 2 — Source data Fig. 1 [file 44321_2025_315_MOESM2_ESM.zip › Figure 1/F1A/1-GLDC-PDGFRbeta/Lee III/11 (3).jpg]

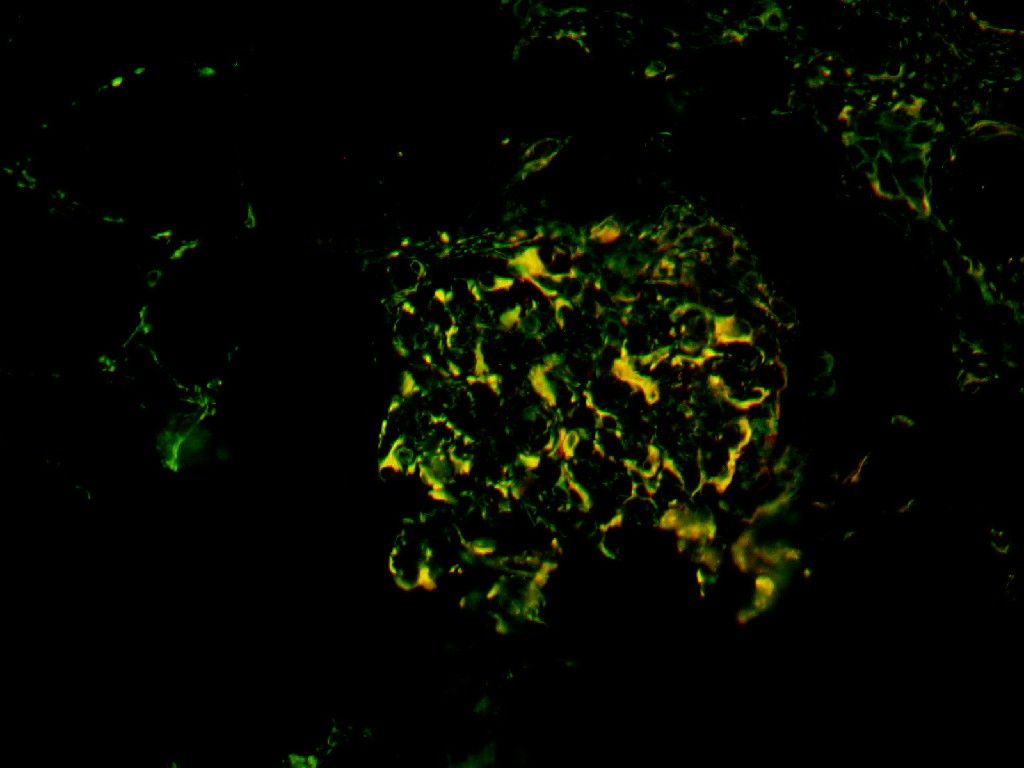

Supplement: Supplementary file 2 — Source data Fig. 1 [file 44321_2025_315_MOESM2_ESM.zip › Figure 1/F1A/1-GLDC-PDGFRbeta/Lee III/11 (4).jpg]

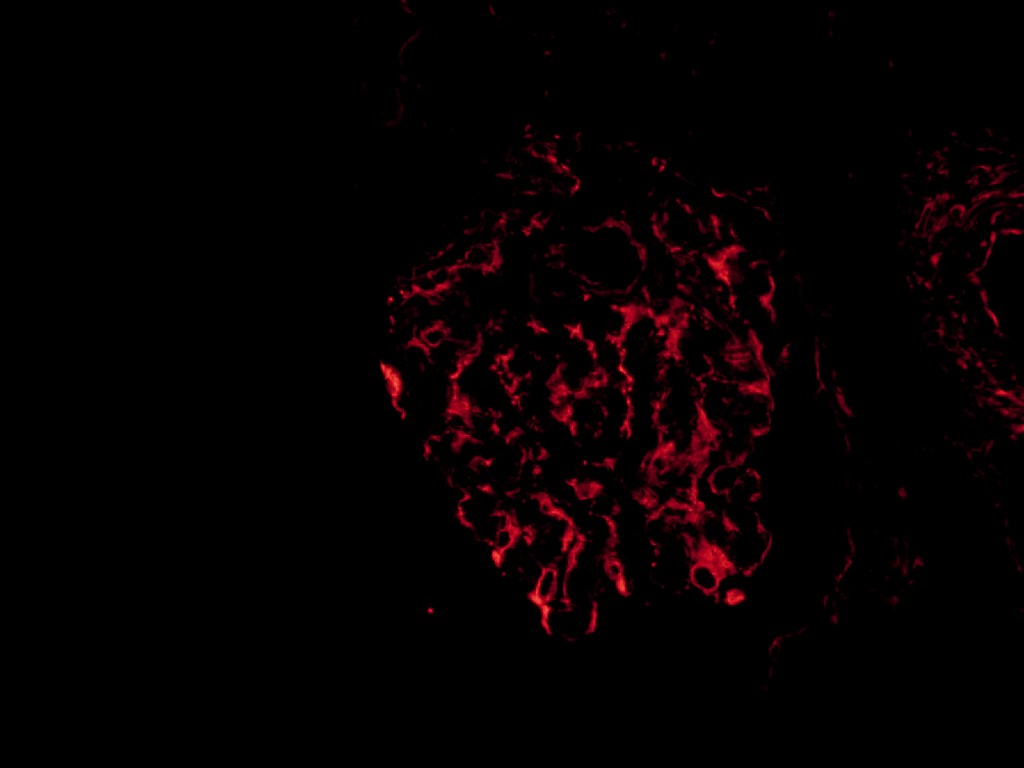

Supplement: Supplementary file 2 — Source data Fig. 1 [file 44321_2025_315_MOESM2_ESM.zip › Figure 1/F1A/1-GLDC-PDGFRbeta/Lee III/12 (1).jpg]

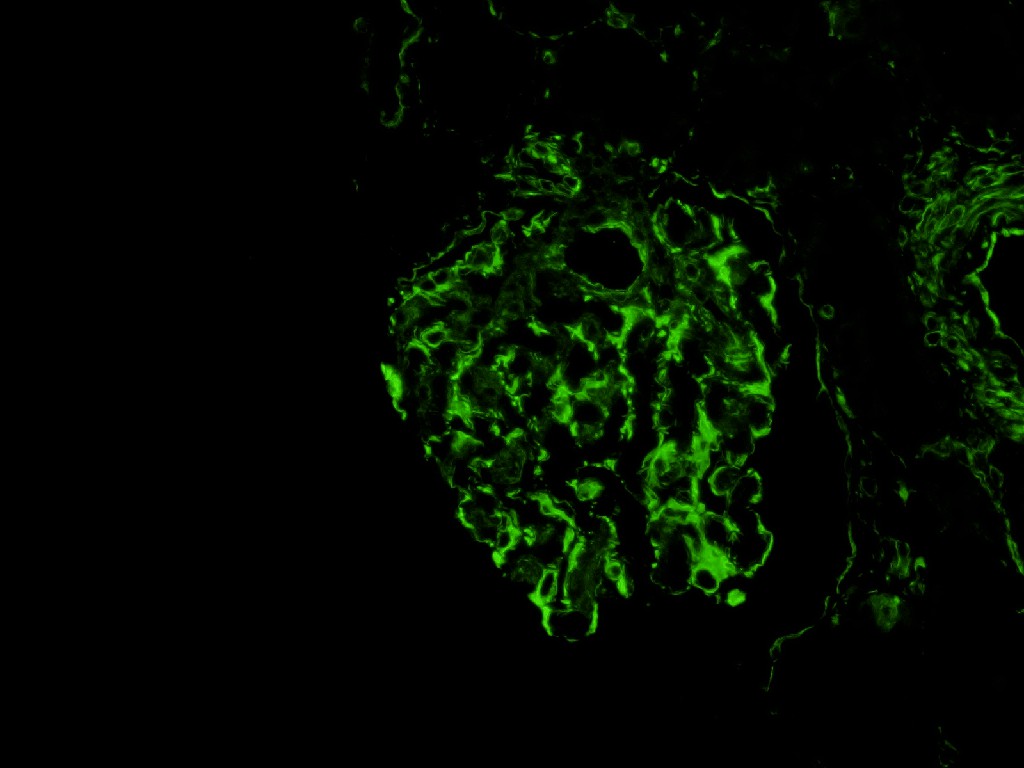

Supplement: Supplementary file 2 — Source data Fig. 1 [file 44321_2025_315_MOESM2_ESM.zip › Figure 1/F1A/1-GLDC-PDGFRbeta/Lee III/12 (2).jpg]

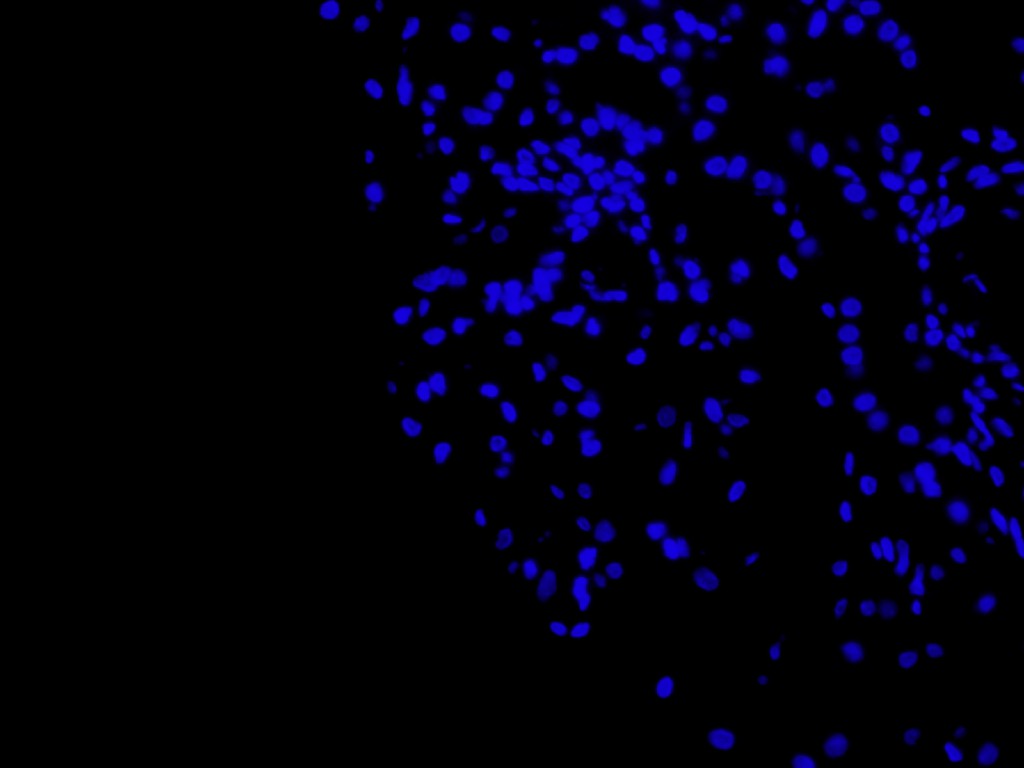

Supplement: Supplementary file 2 — Source data Fig. 1 [file 44321_2025_315_MOESM2_ESM.zip › Figure 1/F1A/1-GLDC-PDGFRbeta/Lee III/12 (3).jpg]

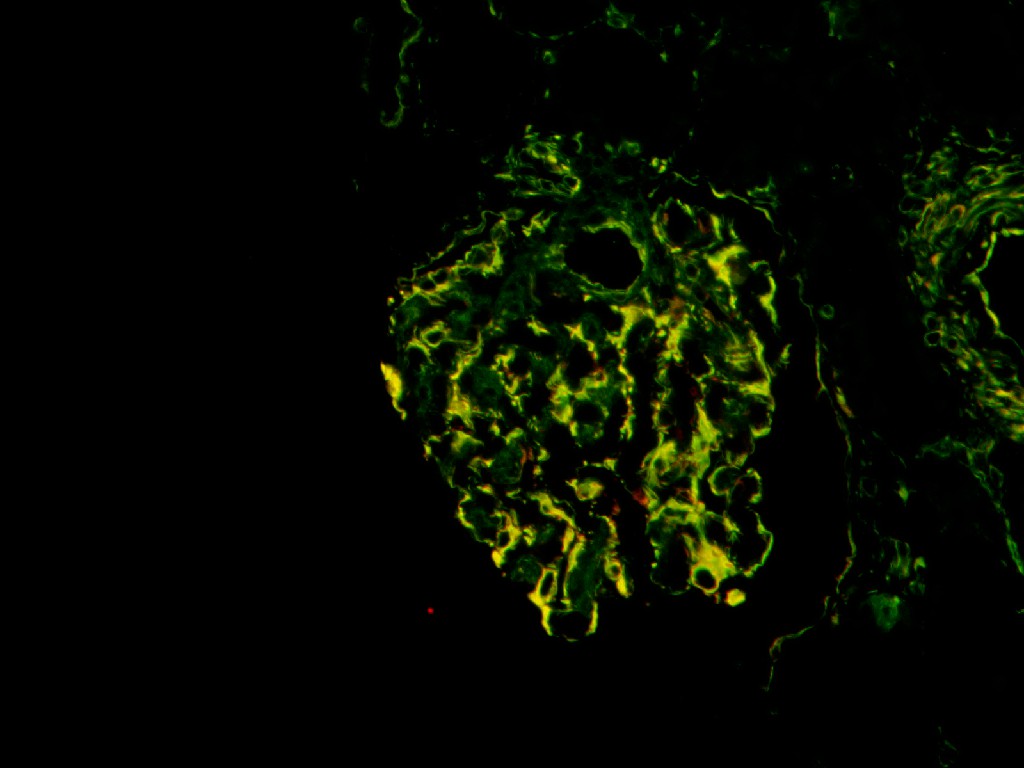

Supplement: Supplementary file 2 — Source data Fig. 1 [file 44321_2025_315_MOESM2_ESM.zip › Figure 1/F1A/1-GLDC-PDGFRbeta/Lee III/12 (4).jpg]

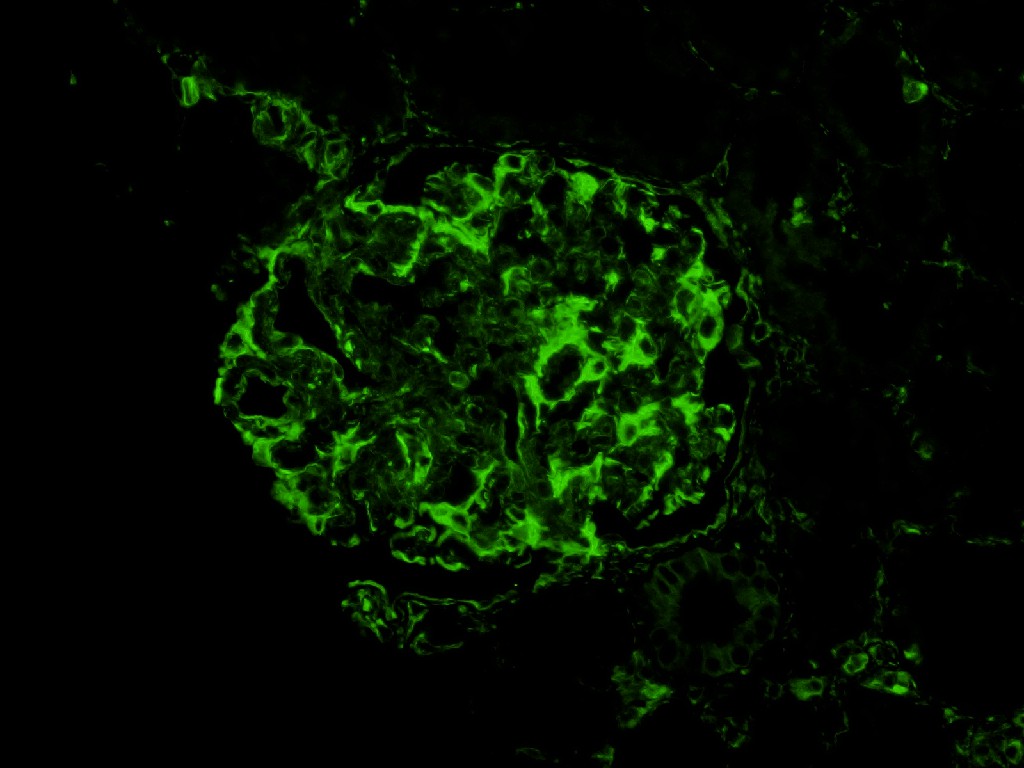

Supplement: Supplementary file 2 — Source data Fig. 1 [file 44321_2025_315_MOESM2_ESM.zip › Figure 1/F1A/1-GLDC-PDGFRbeta/Lee III/13 (1).jpg]

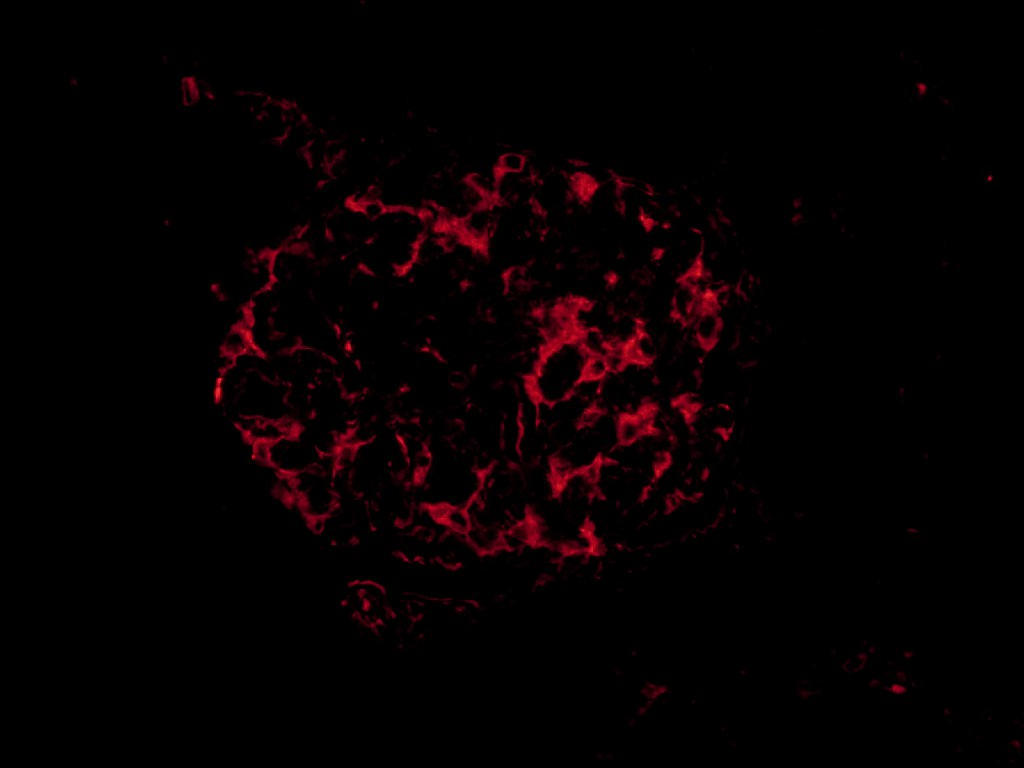

Supplement: Supplementary file 2 — Source data Fig. 1 [file 44321_2025_315_MOESM2_ESM.zip › Figure 1/F1A/1-GLDC-PDGFRbeta/Lee III/13 (2).jpg]

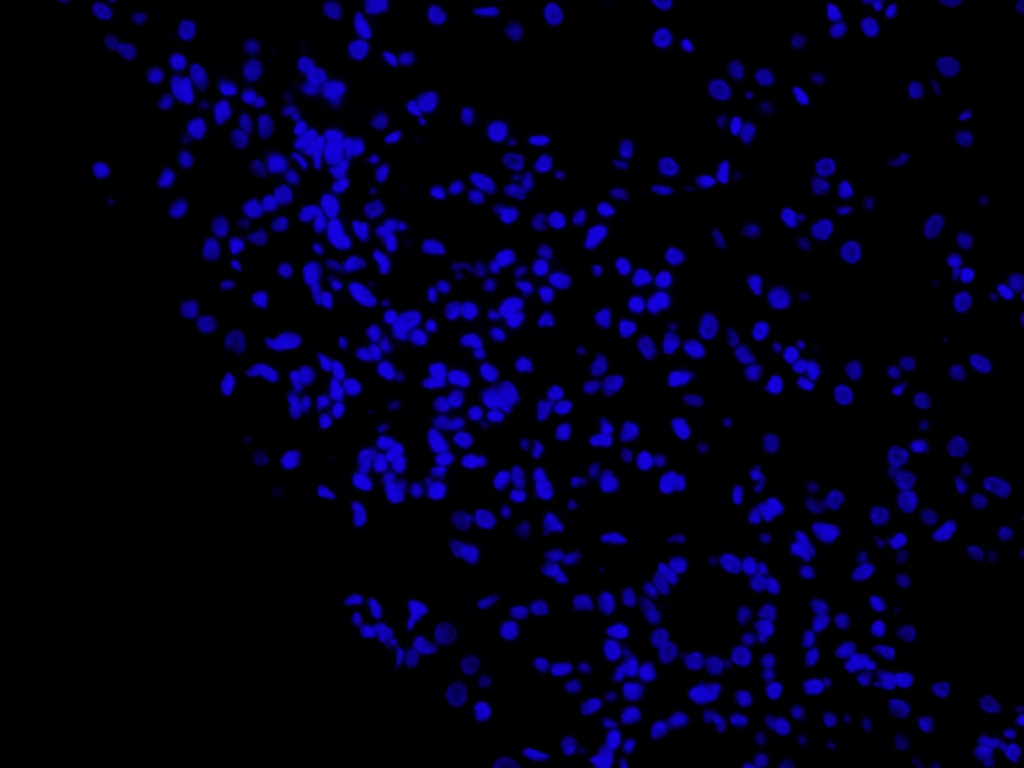

Supplement: Supplementary file 2 — Source data Fig. 1 [file 44321_2025_315_MOESM2_ESM.zip › Figure 1/F1A/1-GLDC-PDGFRbeta/Lee III/13 (3).jpg]

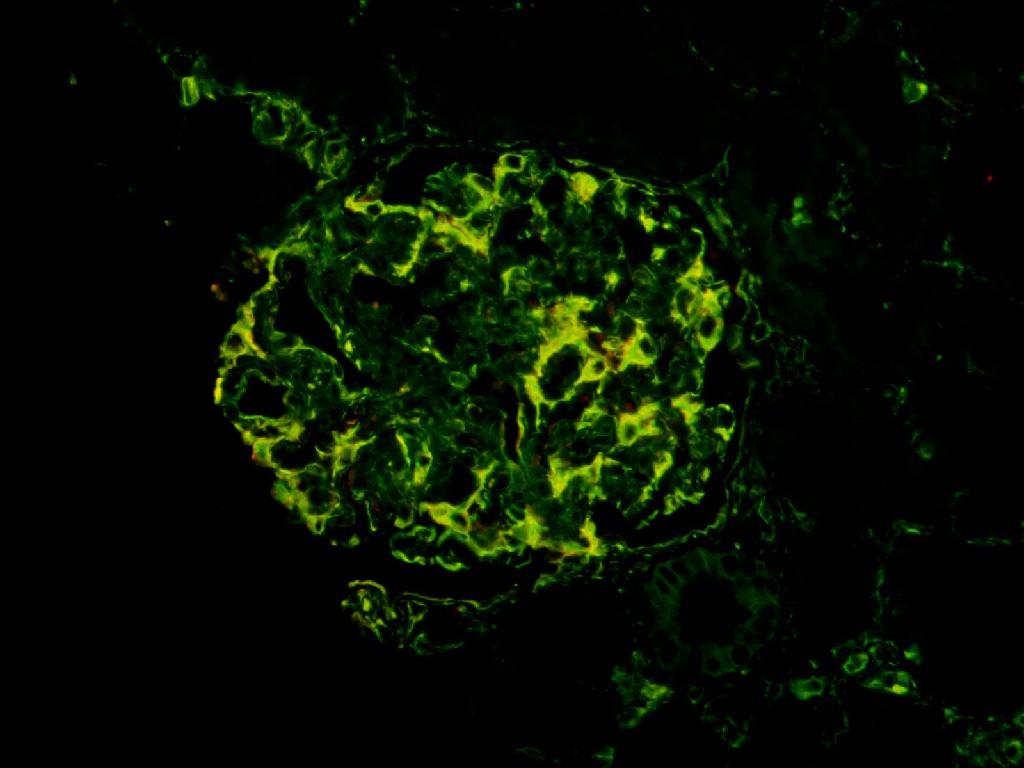

Supplement: Supplementary file 2 — Source data Fig. 1 [file 44321_2025_315_MOESM2_ESM.zip › Figure 1/F1A/1-GLDC-PDGFRbeta/Lee III/13 (4).jpg]

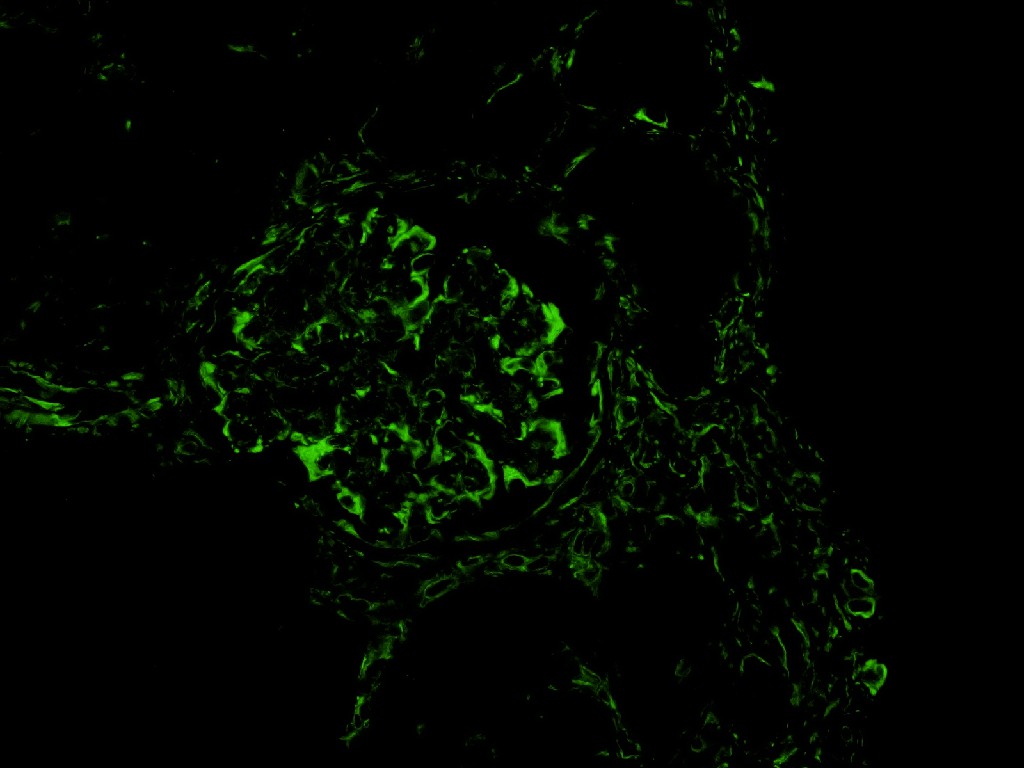

Supplement: Supplementary file 2 — Source data Fig. 1 [file 44321_2025_315_MOESM2_ESM.zip › Figure 1/F1A/1-GLDC-PDGFRbeta/Lee III/2 (1).jpg]

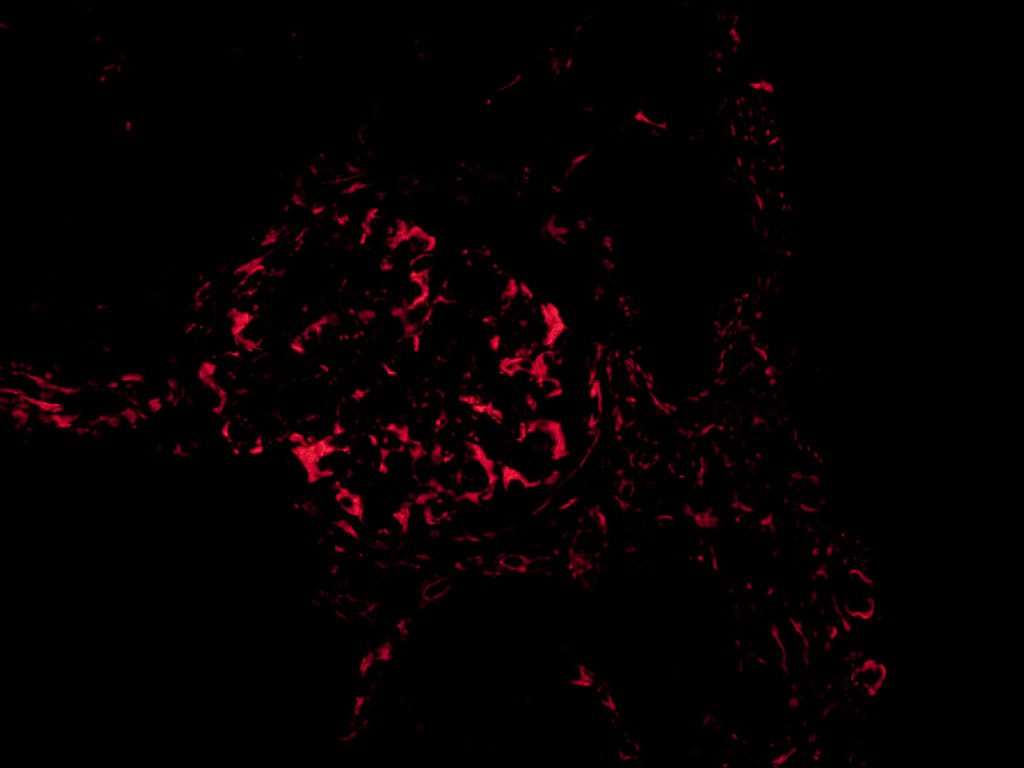

Supplement: Supplementary file 2 — Source data Fig. 1 [file 44321_2025_315_MOESM2_ESM.zip › Figure 1/F1A/1-GLDC-PDGFRbeta/Lee III/2 (2).jpg]

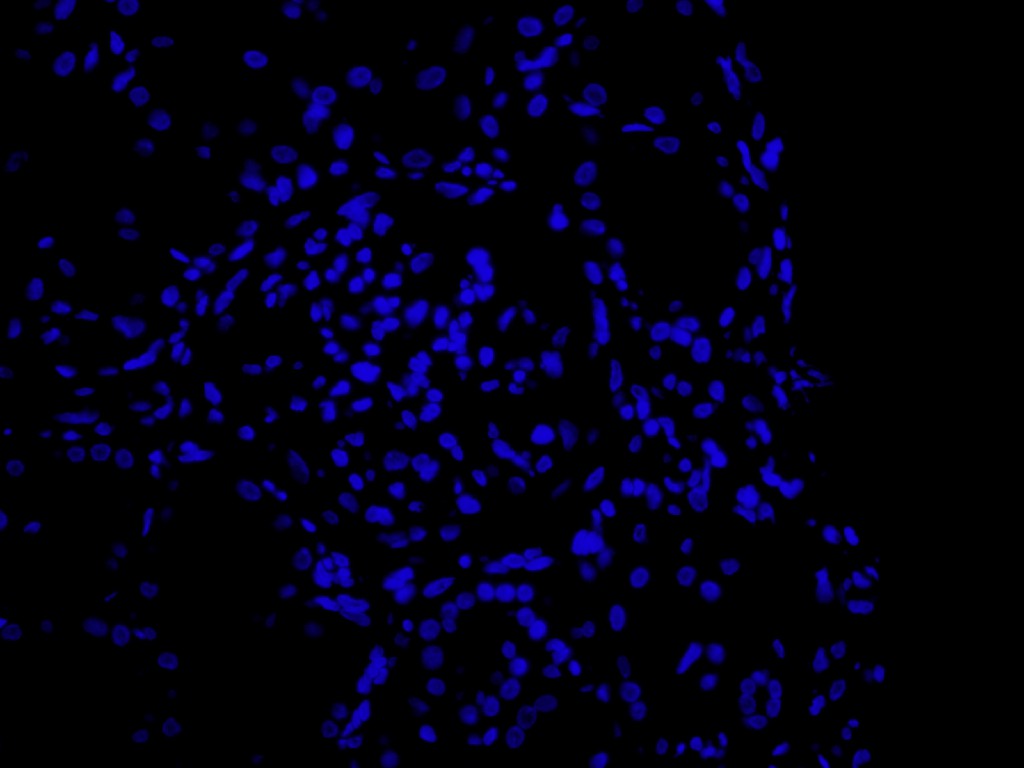

Supplement: Supplementary file 2 — Source data Fig. 1 [file 44321_2025_315_MOESM2_ESM.zip › Figure 1/F1A/1-GLDC-PDGFRbeta/Lee III/2 (3).jpg]

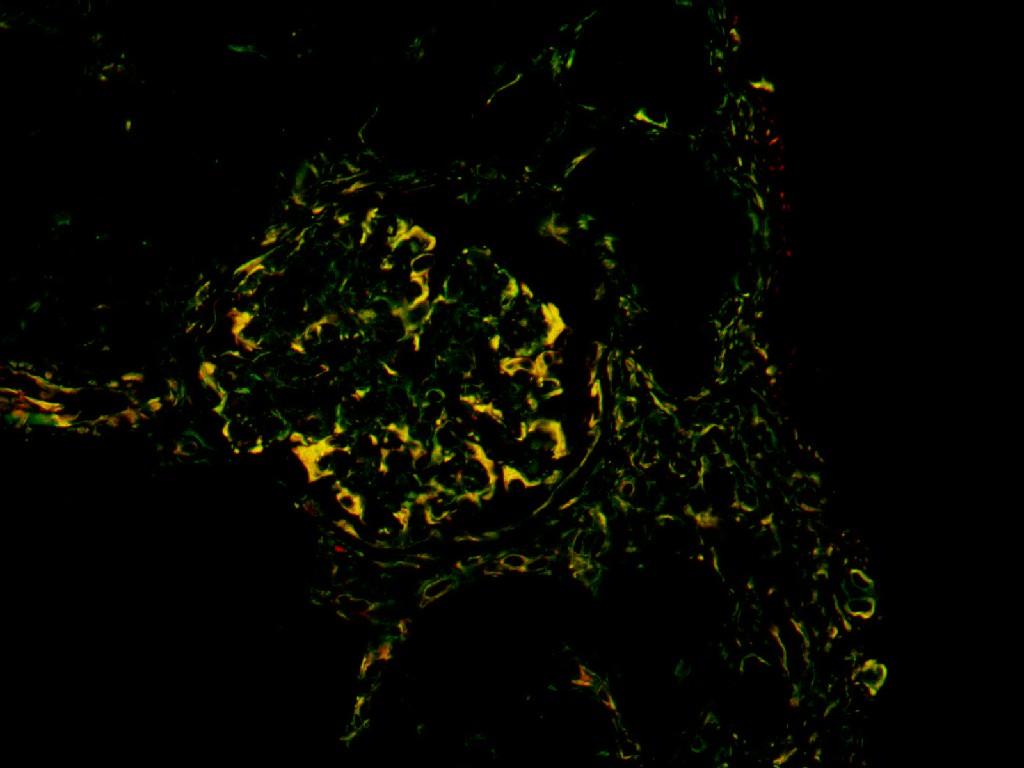

Supplement: Supplementary file 2 — Source data Fig. 1 [file 44321_2025_315_MOESM2_ESM.zip › Figure 1/F1A/1-GLDC-PDGFRbeta/Lee III/2 (4).jpg]

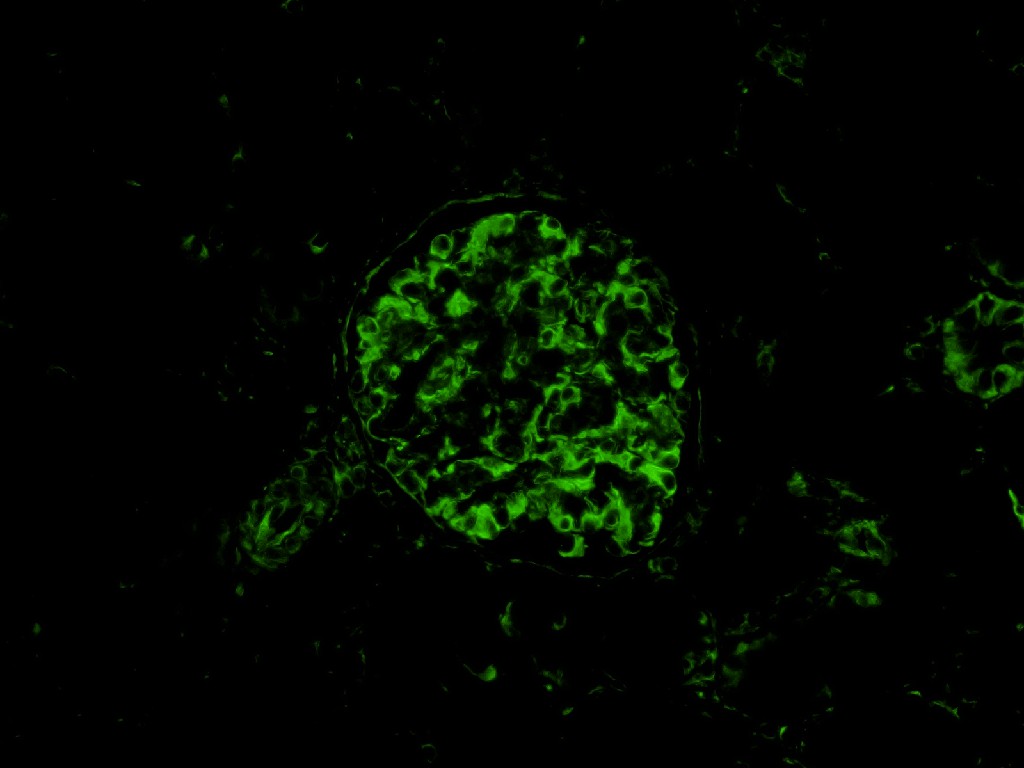

Supplement: Supplementary file 2 — Source data Fig. 1 [file 44321_2025_315_MOESM2_ESM.zip › Figure 1/F1A/1-GLDC-PDGFRbeta/Lee III/3 (1).jpg]

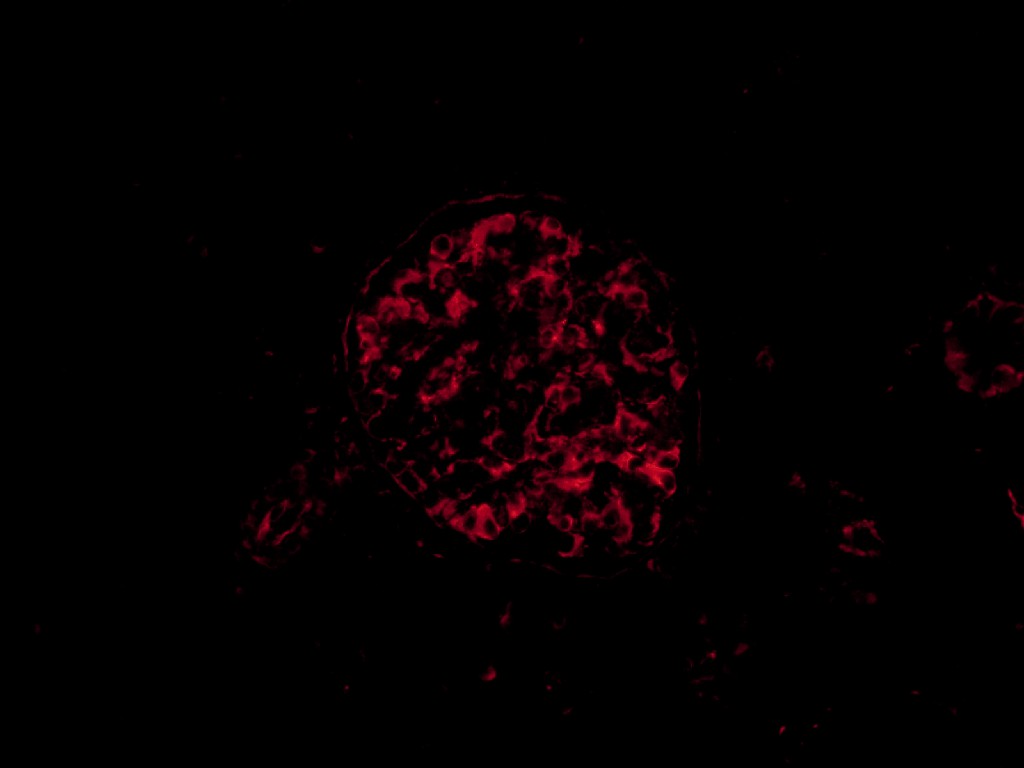

Supplement: Supplementary file 2 — Source data Fig. 1 [file 44321_2025_315_MOESM2_ESM.zip › Figure 1/F1A/1-GLDC-PDGFRbeta/Lee III/3 (2).jpg]

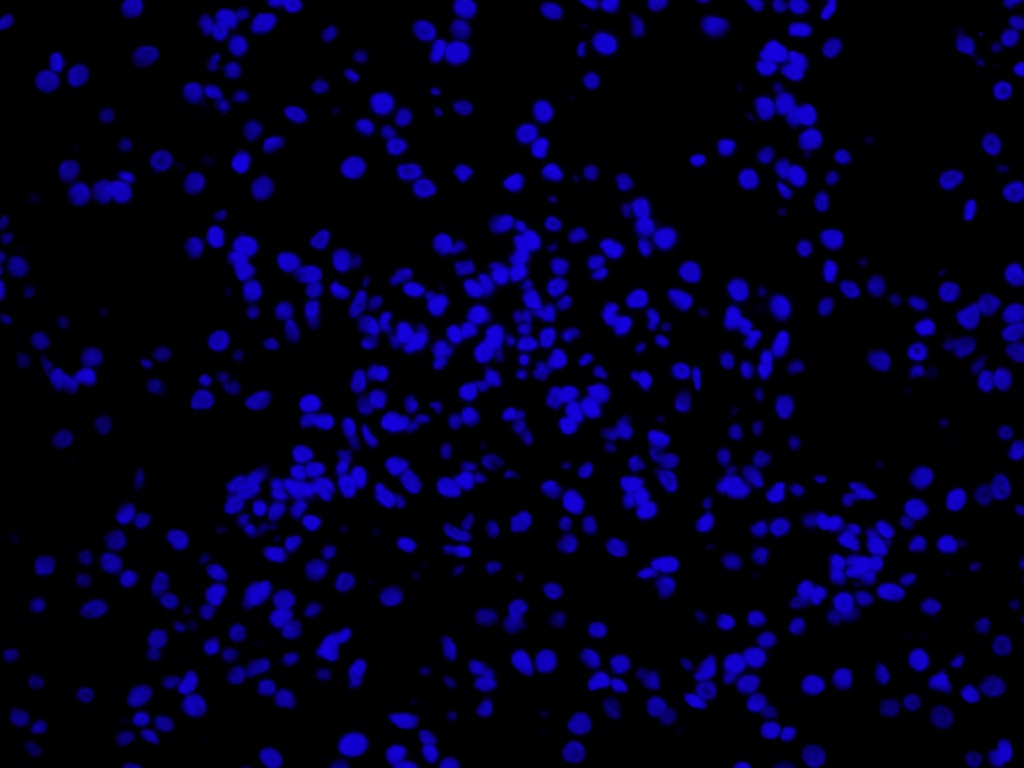

Supplement: Supplementary file 2 — Source data Fig. 1 [file 44321_2025_315_MOESM2_ESM.zip › Figure 1/F1A/1-GLDC-PDGFRbeta/Lee III/3 (3).jpg]

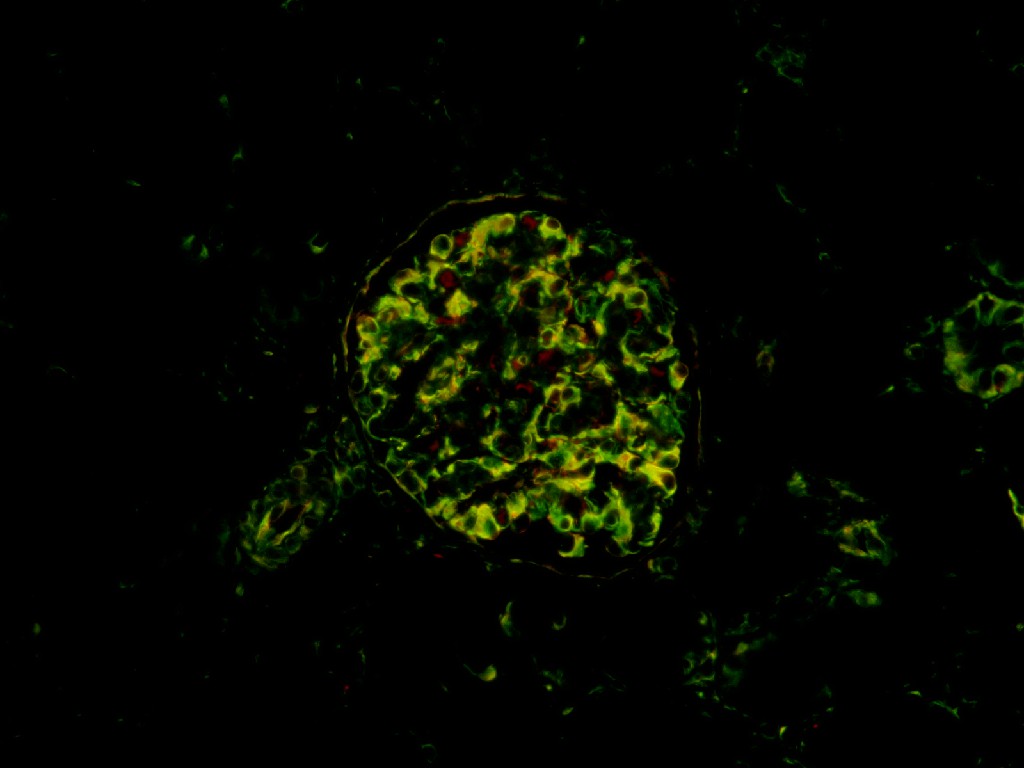

Supplement: Supplementary file 2 — Source data Fig. 1 [file 44321_2025_315_MOESM2_ESM.zip › Figure 1/F1A/1-GLDC-PDGFRbeta/Lee III/3 (4).jpg]

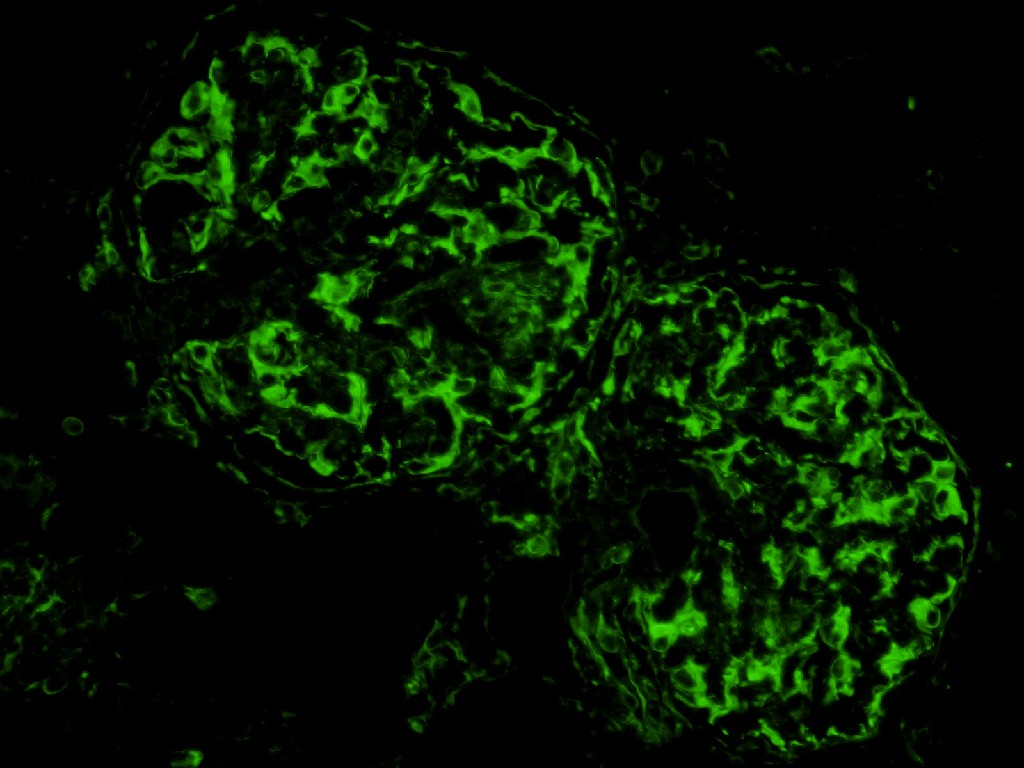

Supplement: Supplementary file 2 — Source data Fig. 1 [file 44321_2025_315_MOESM2_ESM.zip › Figure 1/F1A/1-GLDC-PDGFRbeta/Lee III/4 (1).jpg]

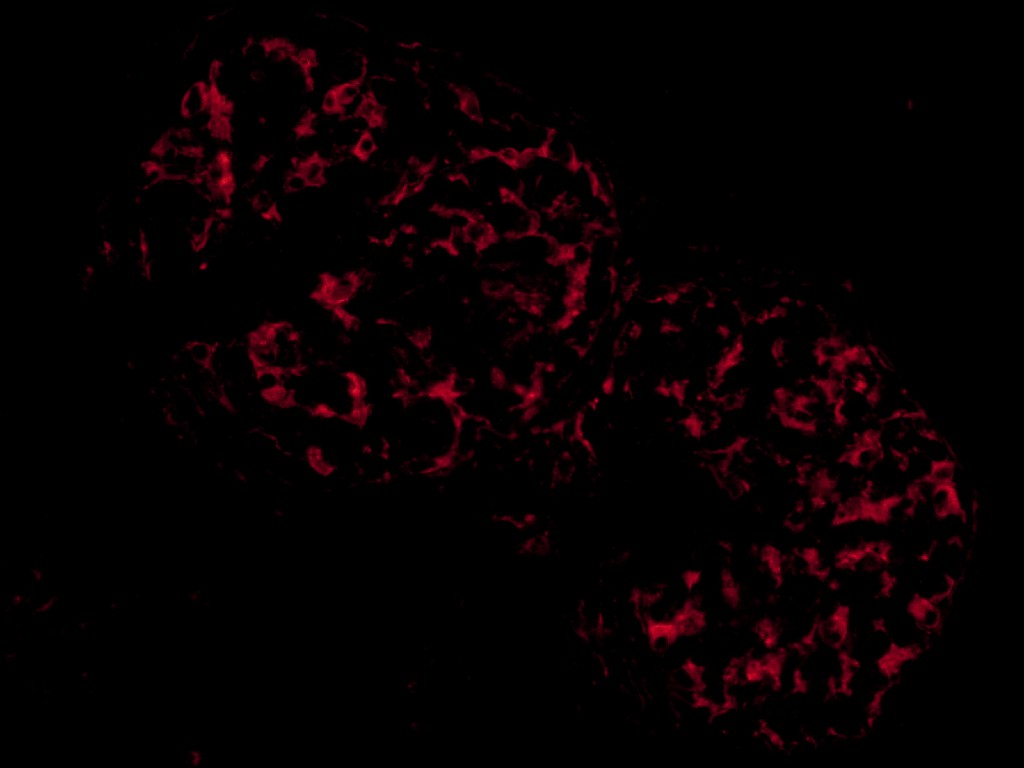

Supplement: Supplementary file 2 — Source data Fig. 1 [file 44321_2025_315_MOESM2_ESM.zip › Figure 1/F1A/1-GLDC-PDGFRbeta/Lee III/4 (2).jpg]

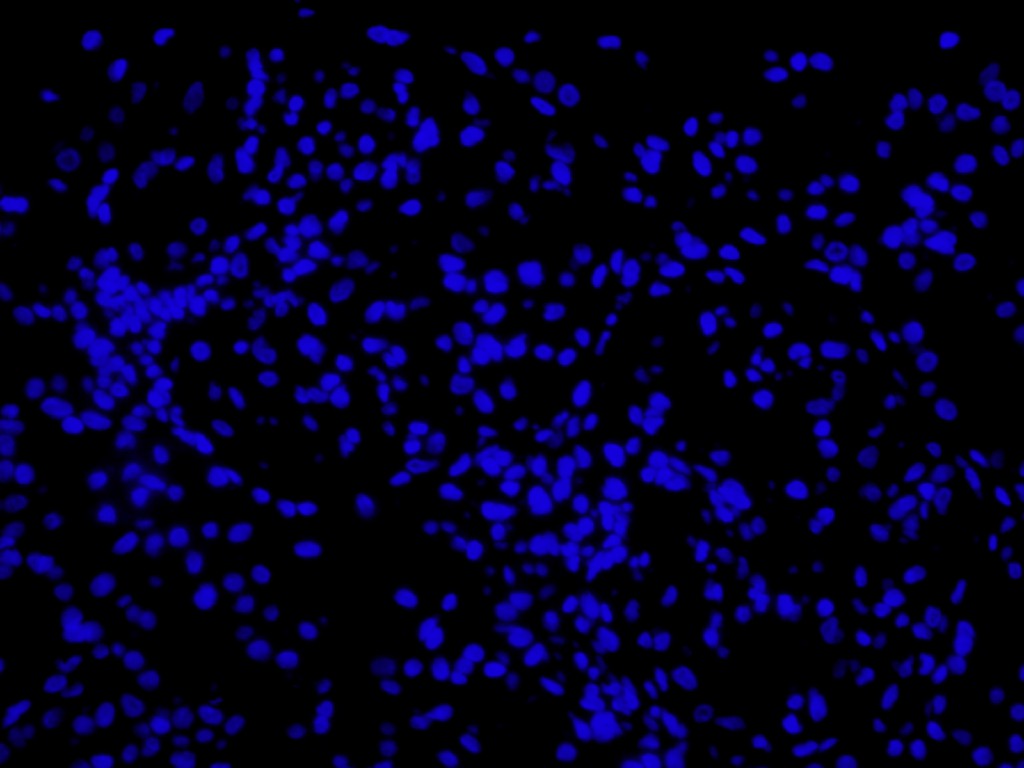

Supplement: Supplementary file 2 — Source data Fig. 1 [file 44321_2025_315_MOESM2_ESM.zip › Figure 1/F1A/1-GLDC-PDGFRbeta/Lee III/4 (3).jpg]

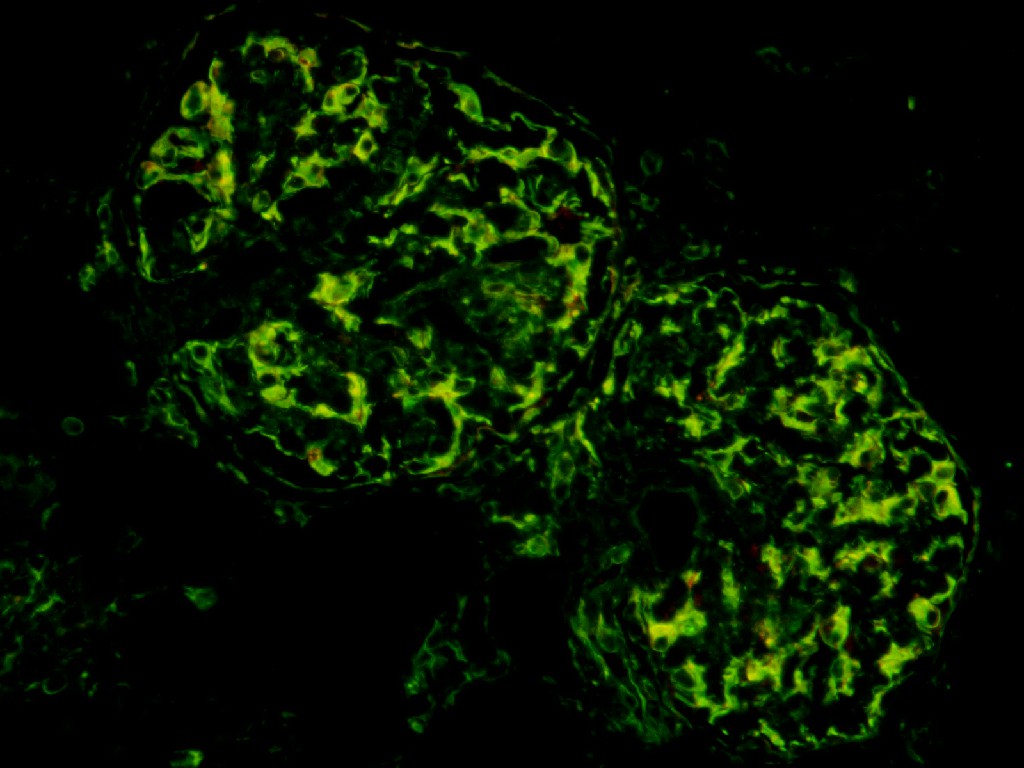

Supplement: Supplementary file 2 — Source data Fig. 1 [file 44321_2025_315_MOESM2_ESM.zip › Figure 1/F1A/1-GLDC-PDGFRbeta/Lee III/4 (4).jpg]

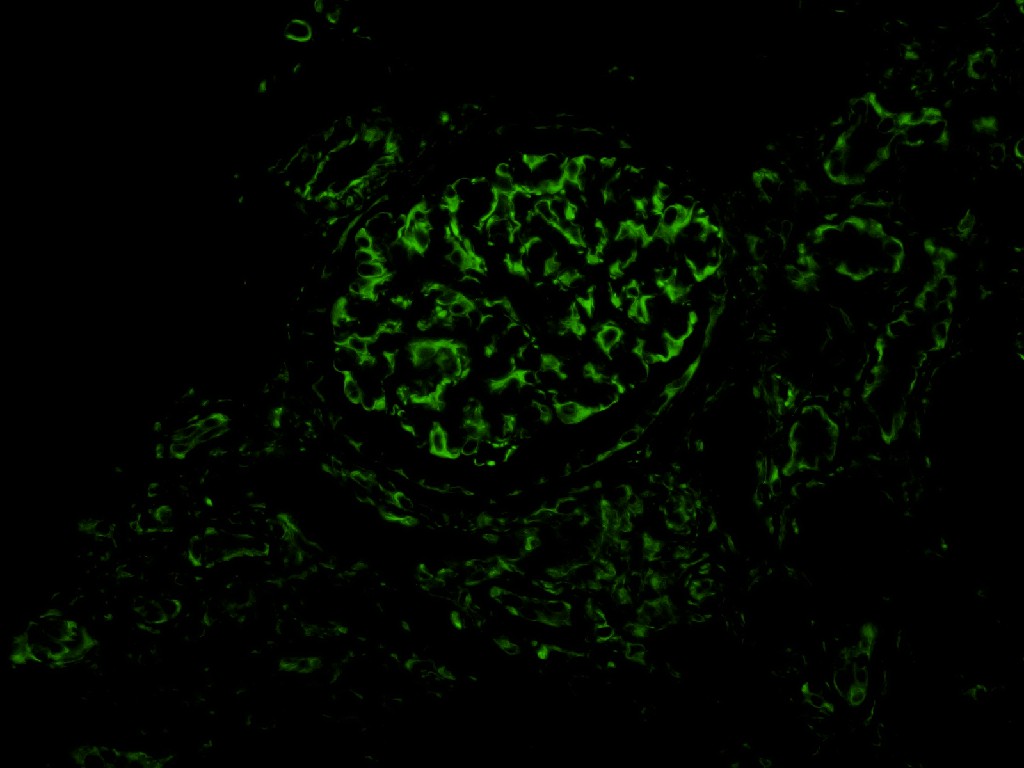

Supplement: Supplementary file 2 — Source data Fig. 1 [file 44321_2025_315_MOESM2_ESM.zip › Figure 1/F1A/1-GLDC-PDGFRbeta/Lee III/5 (1).jpg]

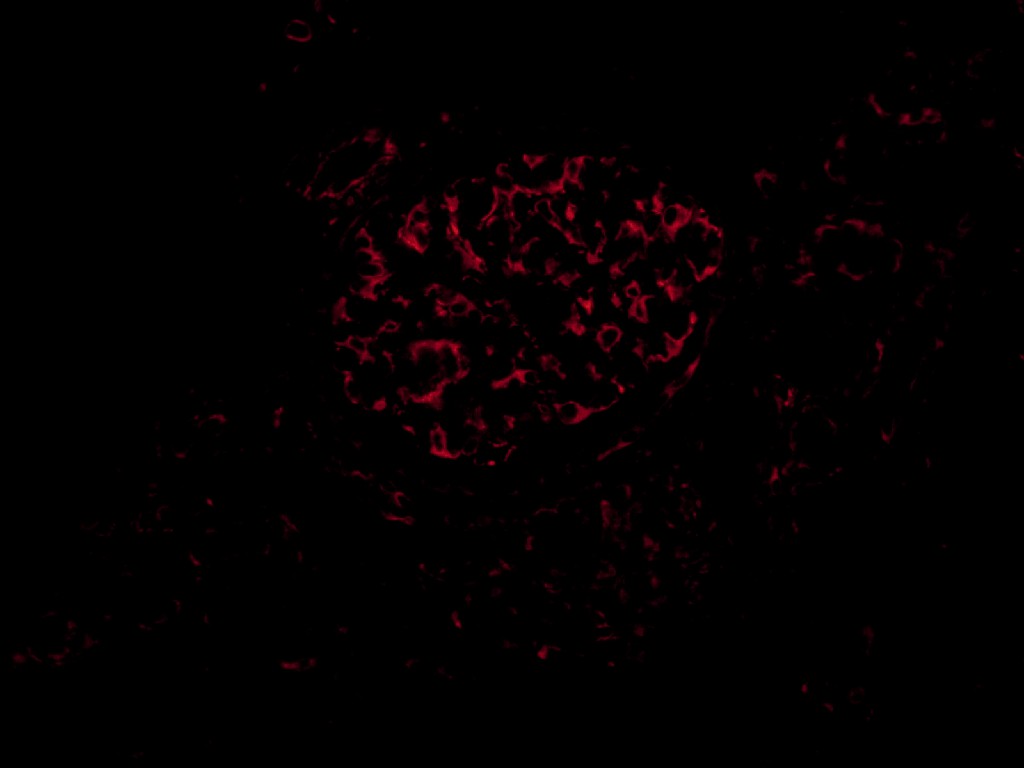

Supplement: Supplementary file 2 — Source data Fig. 1 [file 44321_2025_315_MOESM2_ESM.zip › Figure 1/F1A/1-GLDC-PDGFRbeta/Lee III/5 (2).jpg]

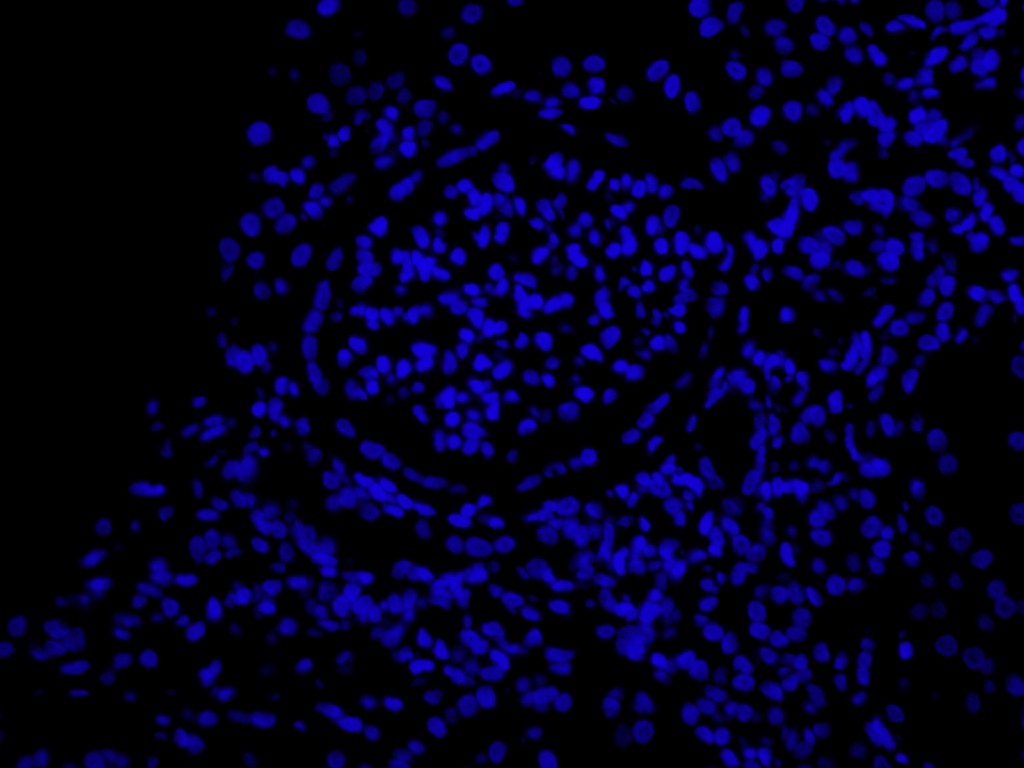

Supplement: Supplementary file 2 — Source data Fig. 1 [file 44321_2025_315_MOESM2_ESM.zip › Figure 1/F1A/1-GLDC-PDGFRbeta/Lee III/5 (3).jpg]

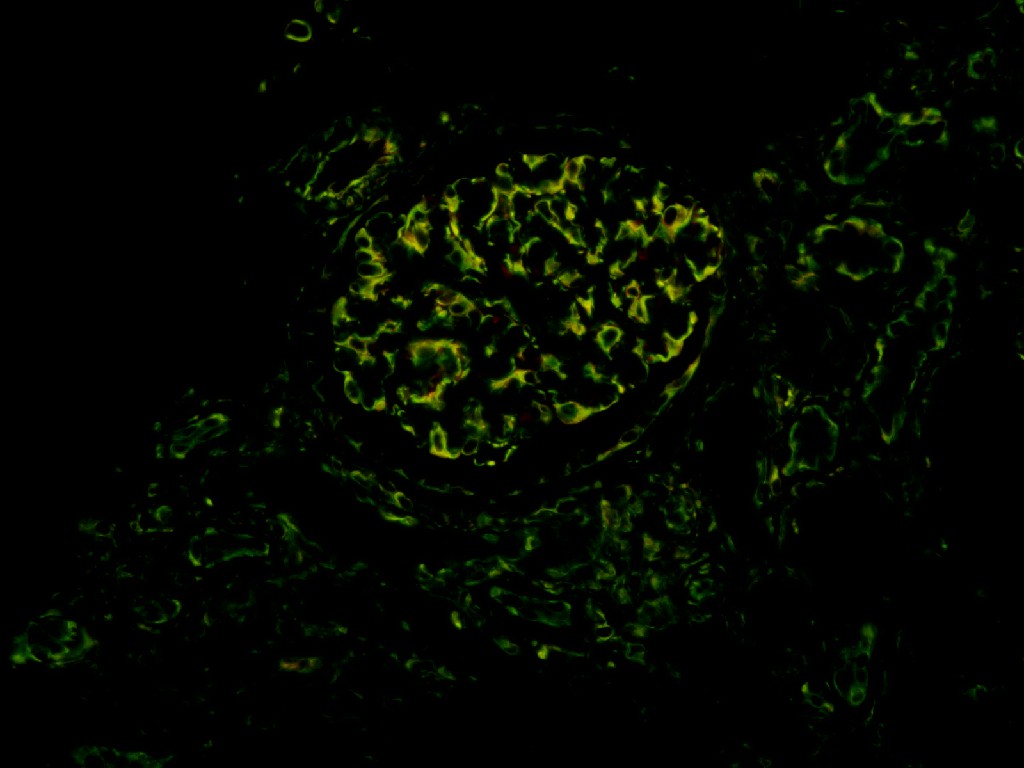

Supplement: Supplementary file 2 — Source data Fig. 1 [file 44321_2025_315_MOESM2_ESM.zip › Figure 1/F1A/1-GLDC-PDGFRbeta/Lee III/5 (4).jpg]

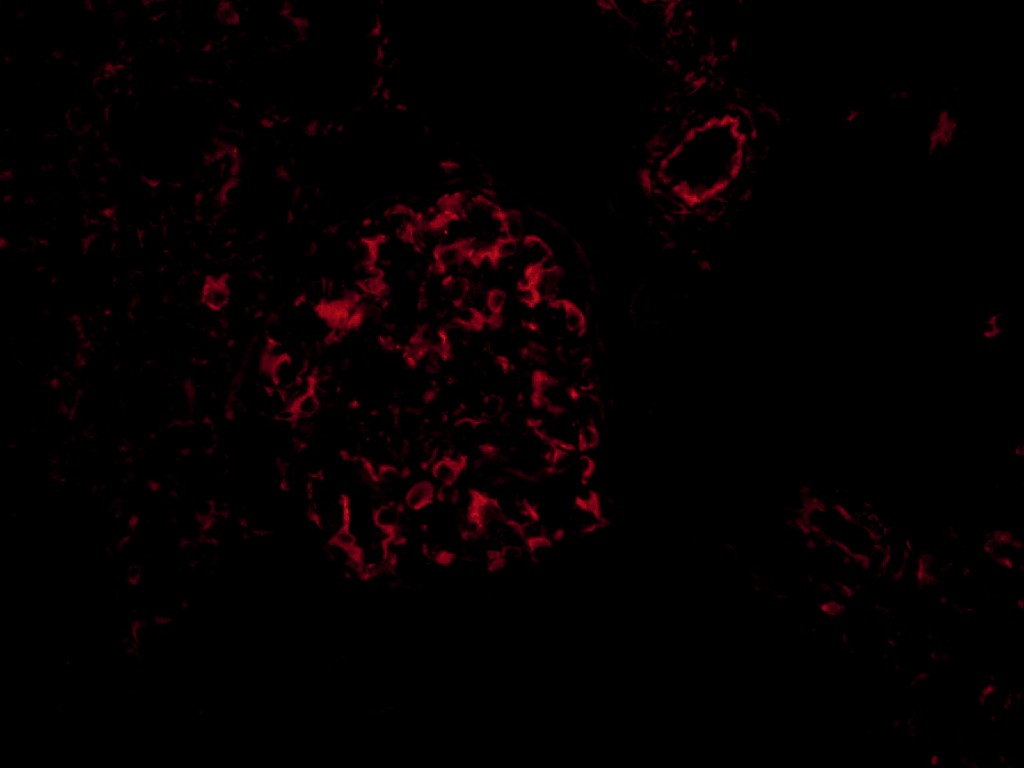

Supplement: Supplementary file 2 — Source data Fig. 1 [file 44321_2025_315_MOESM2_ESM.zip › Figure 1/F1A/1-GLDC-PDGFRbeta/Lee III/6 (1).jpg]

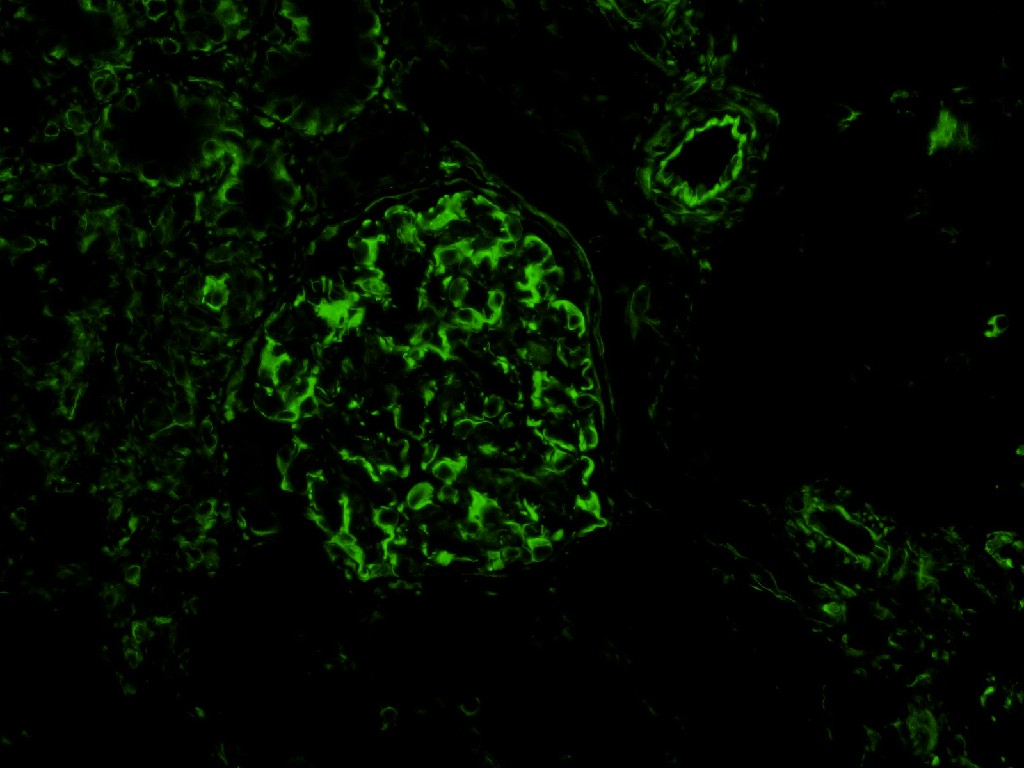

Supplement: Supplementary file 2 — Source data Fig. 1 [file 44321_2025_315_MOESM2_ESM.zip › Figure 1/F1A/1-GLDC-PDGFRbeta/Lee III/6 (2).jpg]

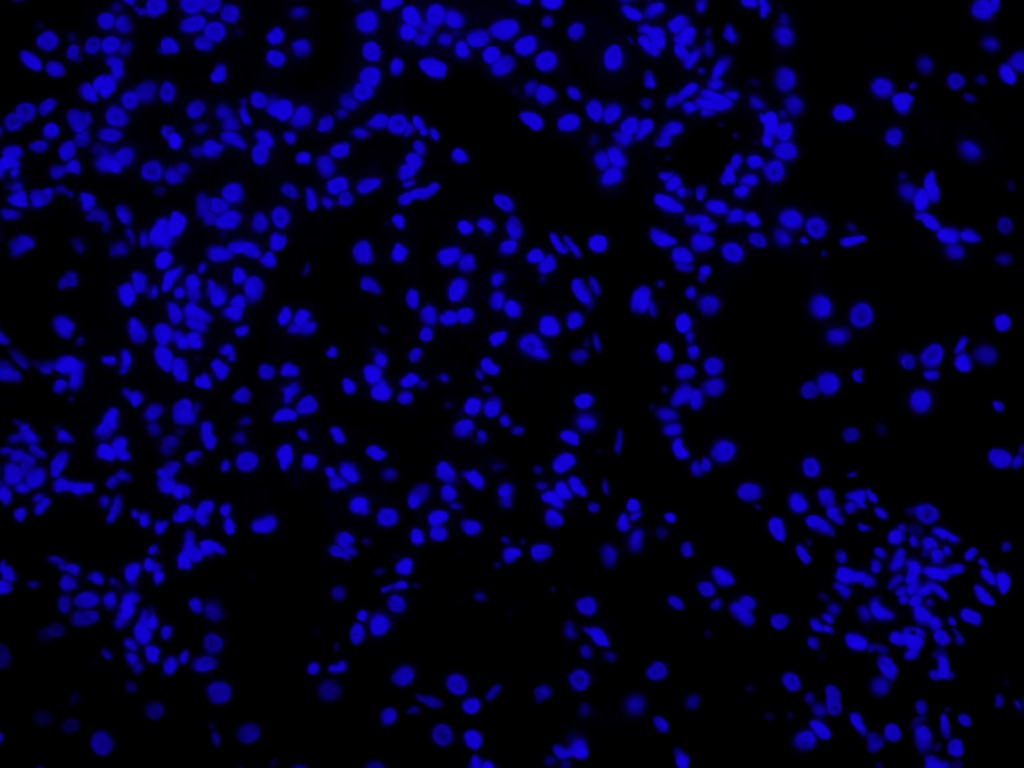

Supplement: Supplementary file 2 — Source data Fig. 1 [file 44321_2025_315_MOESM2_ESM.zip › Figure 1/F1A/1-GLDC-PDGFRbeta/Lee III/6 (3).jpg]

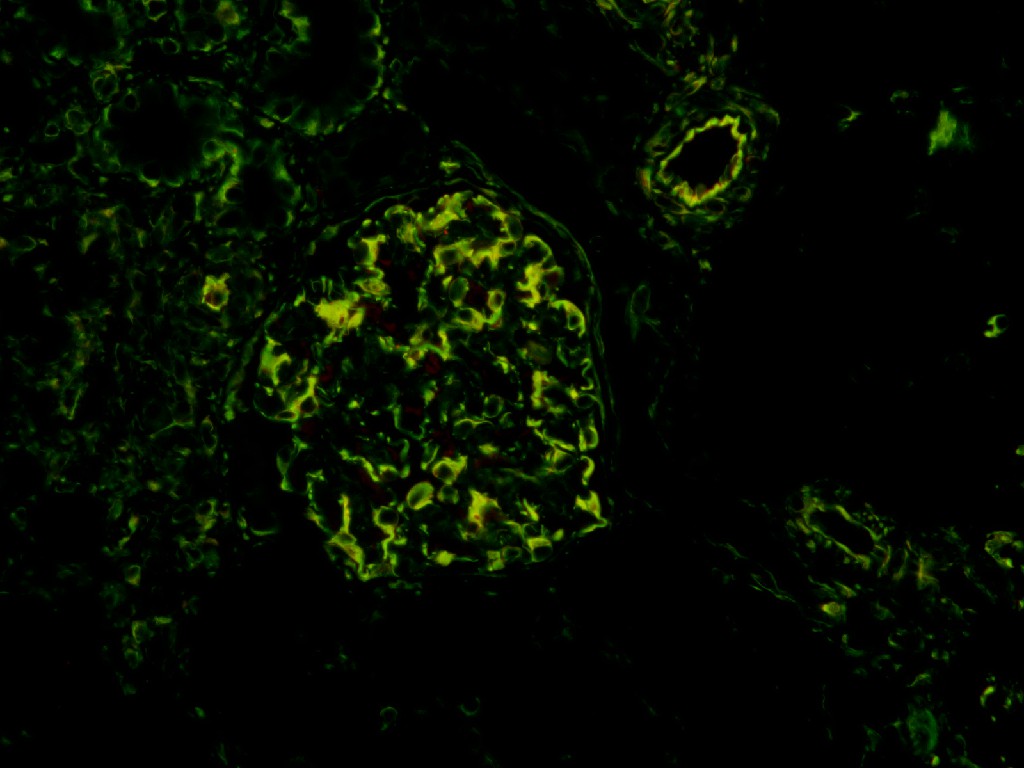

Supplement: Supplementary file 2 — Source data Fig. 1 [file 44321_2025_315_MOESM2_ESM.zip › Figure 1/F1A/1-GLDC-PDGFRbeta/Lee III/6 (4).jpg]

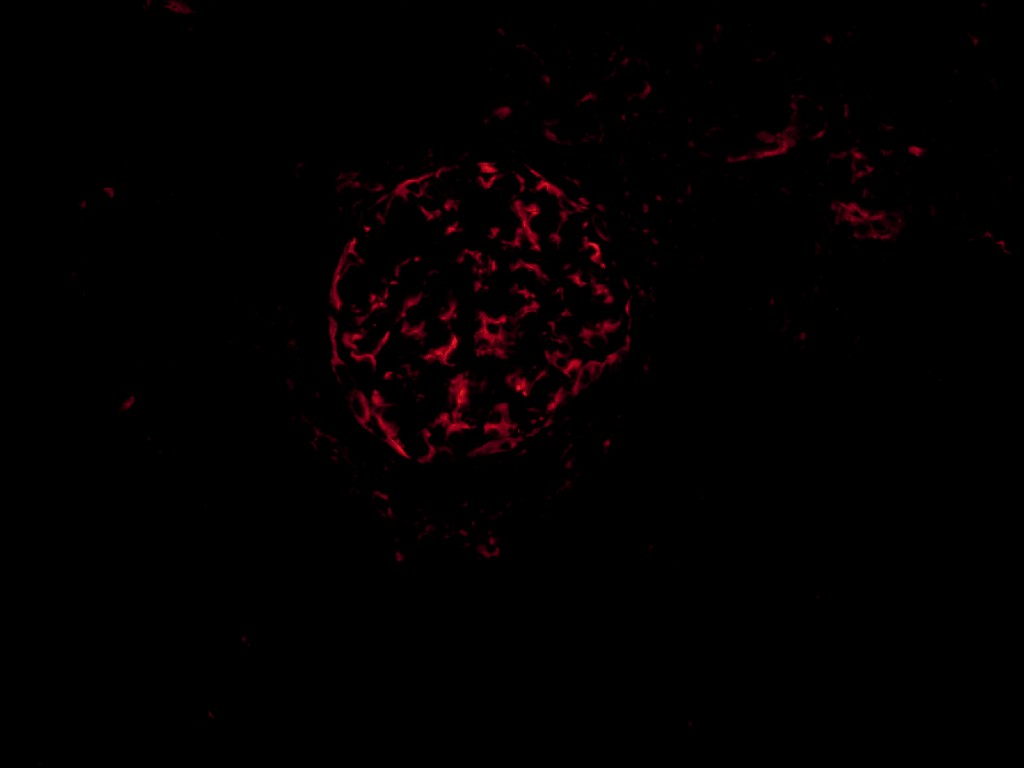

Supplement: Supplementary file 2 — Source data Fig. 1 [file 44321_2025_315_MOESM2_ESM.zip › Figure 1/F1A/1-GLDC-PDGFRbeta/Lee III/7 (1).jpg]

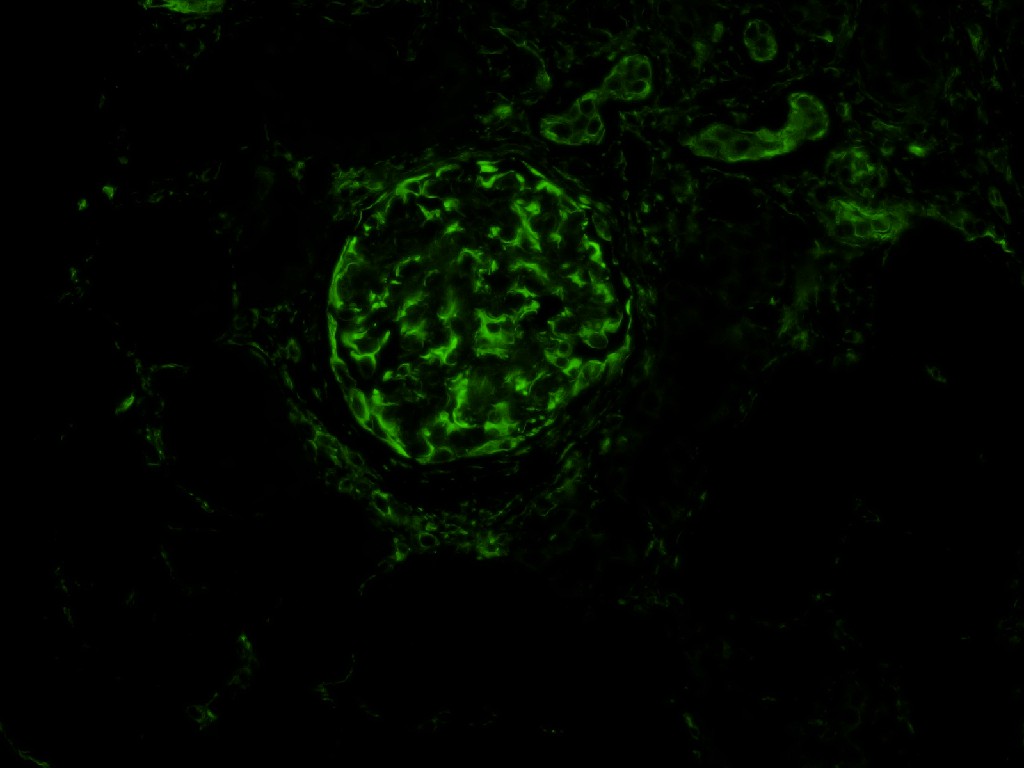

Supplement: Supplementary file 2 — Source data Fig. 1 [file 44321_2025_315_MOESM2_ESM.zip › Figure 1/F1A/1-GLDC-PDGFRbeta/Lee III/7 (2).jpg]

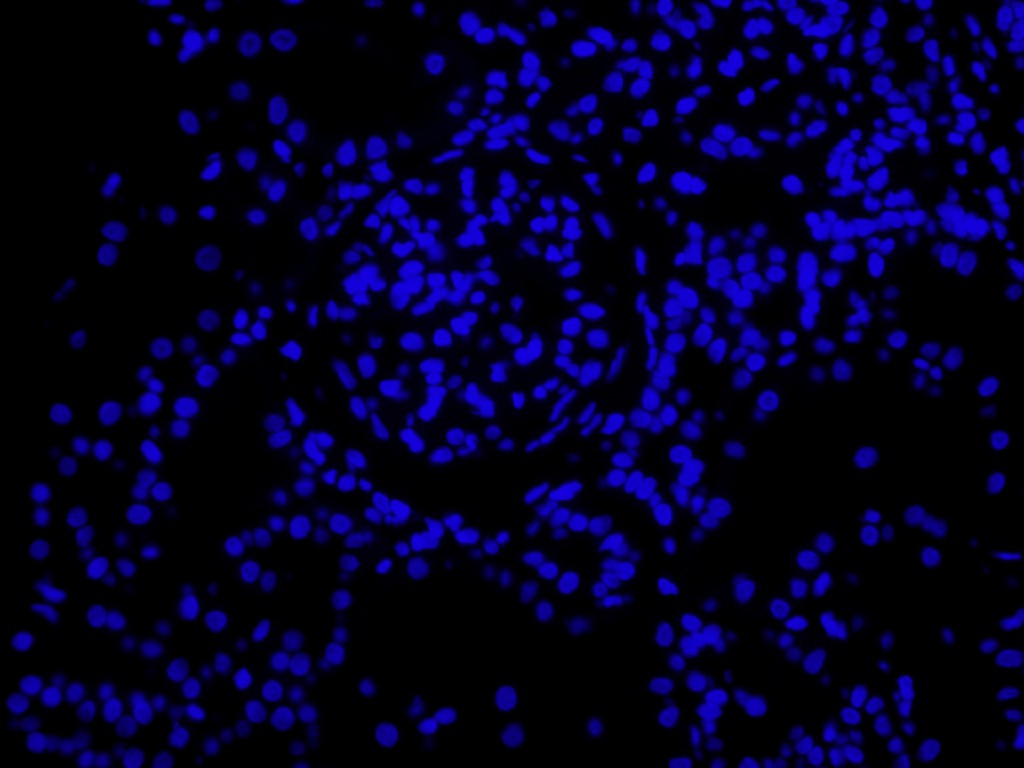

Supplement: Supplementary file 2 — Source data Fig. 1 [file 44321_2025_315_MOESM2_ESM.zip › Figure 1/F1A/1-GLDC-PDGFRbeta/Lee III/7 (3).jpg]

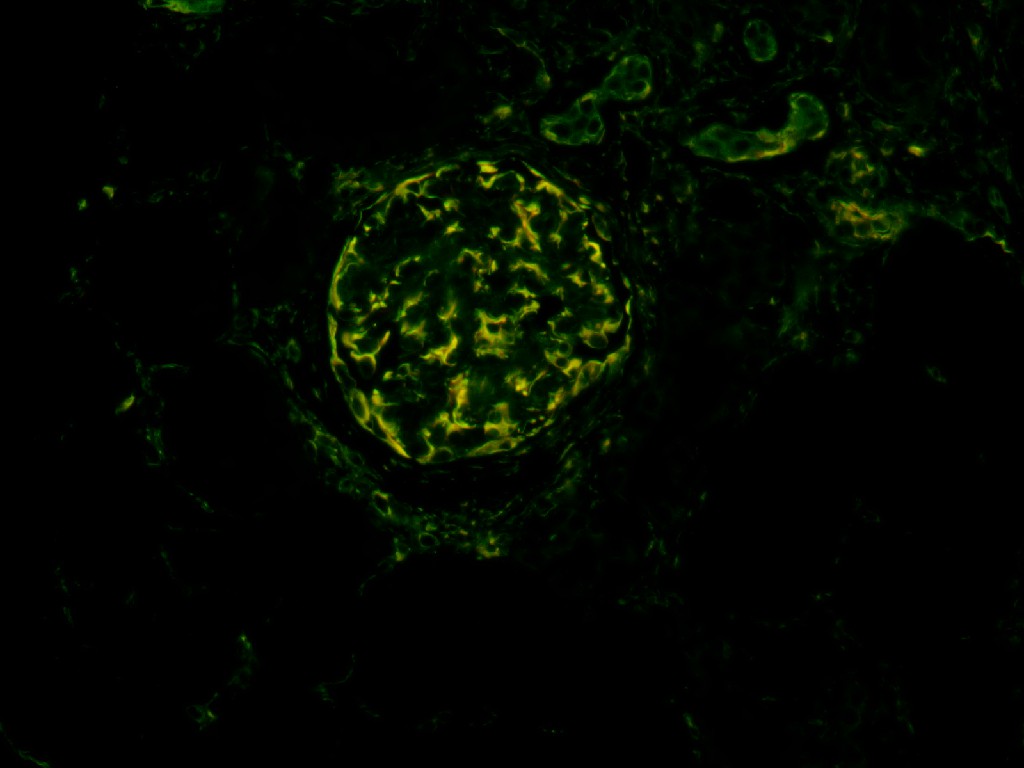

Supplement: Supplementary file 2 — Source data Fig. 1 [file 44321_2025_315_MOESM2_ESM.zip › Figure 1/F1A/1-GLDC-PDGFRbeta/Lee III/7 (4).jpg]

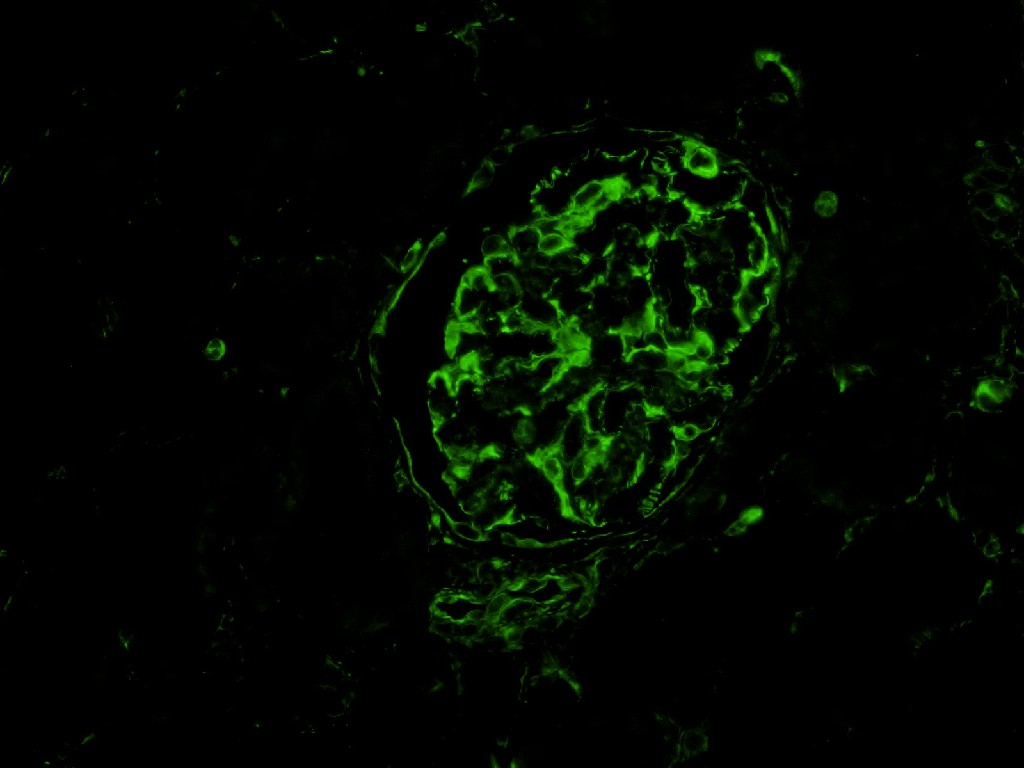

Supplement: Supplementary file 2 — Source data Fig. 1 [file 44321_2025_315_MOESM2_ESM.zip › Figure 1/F1A/1-GLDC-PDGFRbeta/Lee III/8 (1).jpg]

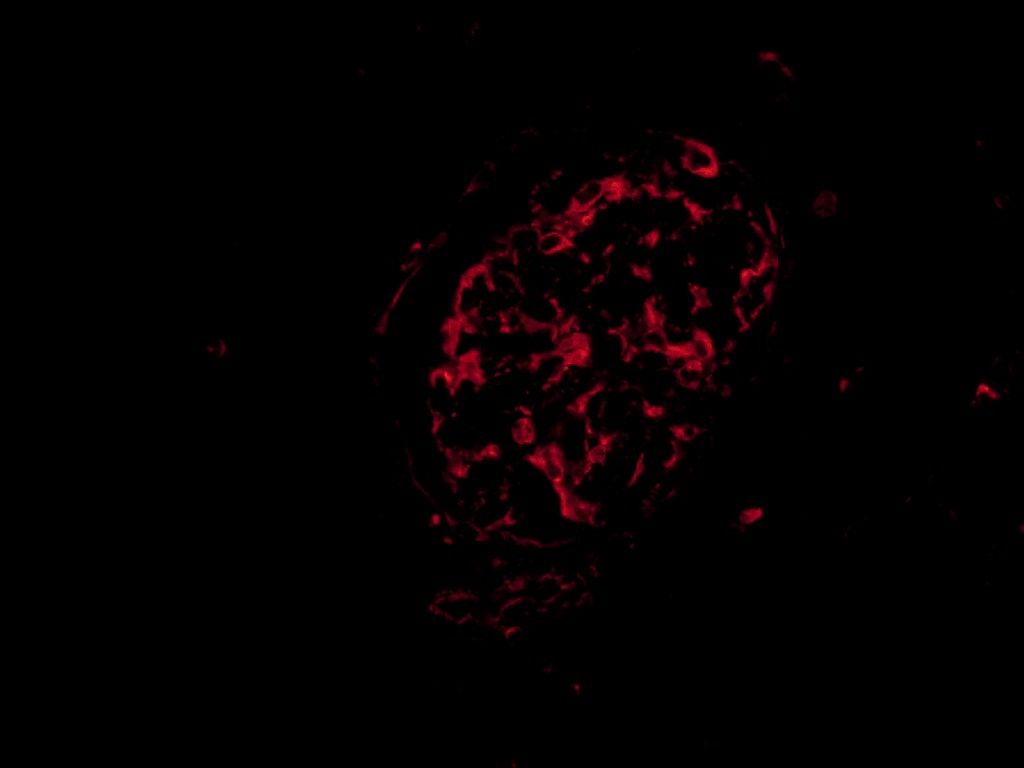

Supplement: Supplementary file 2 — Source data Fig. 1 [file 44321_2025_315_MOESM2_ESM.zip › Figure 1/F1A/1-GLDC-PDGFRbeta/Lee III/8 (2).jpg]

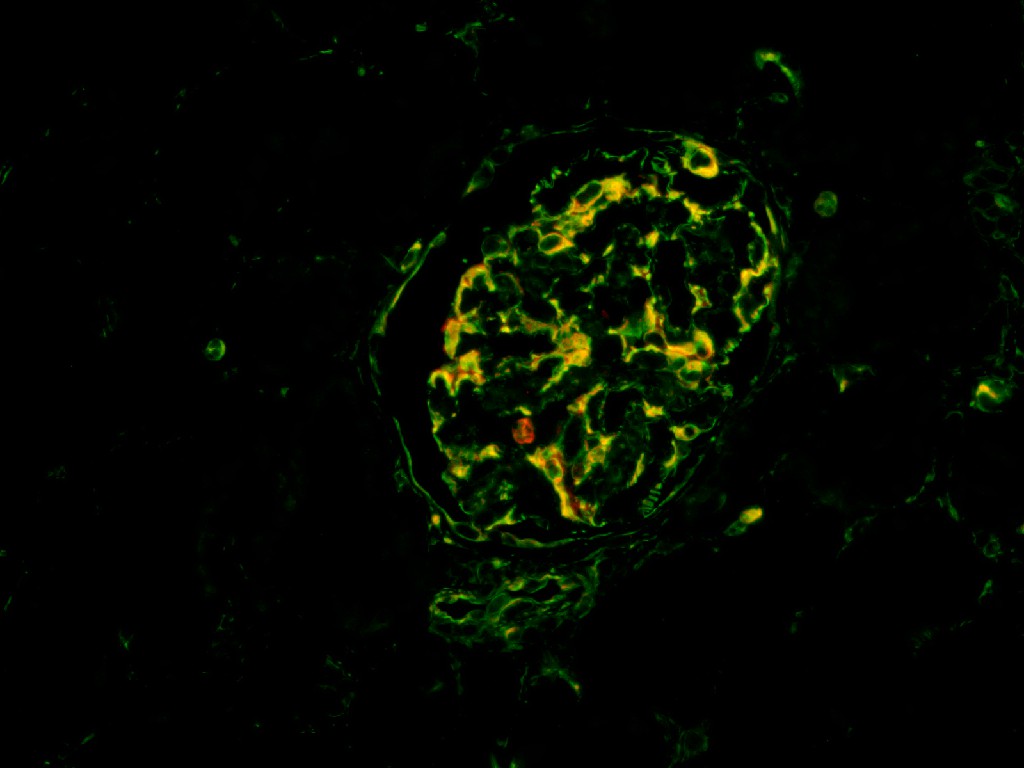

Supplement: Supplementary file 2 — Source data Fig. 1 [file 44321_2025_315_MOESM2_ESM.zip › Figure 1/F1A/1-GLDC-PDGFRbeta/Lee III/8 (3).jpg]

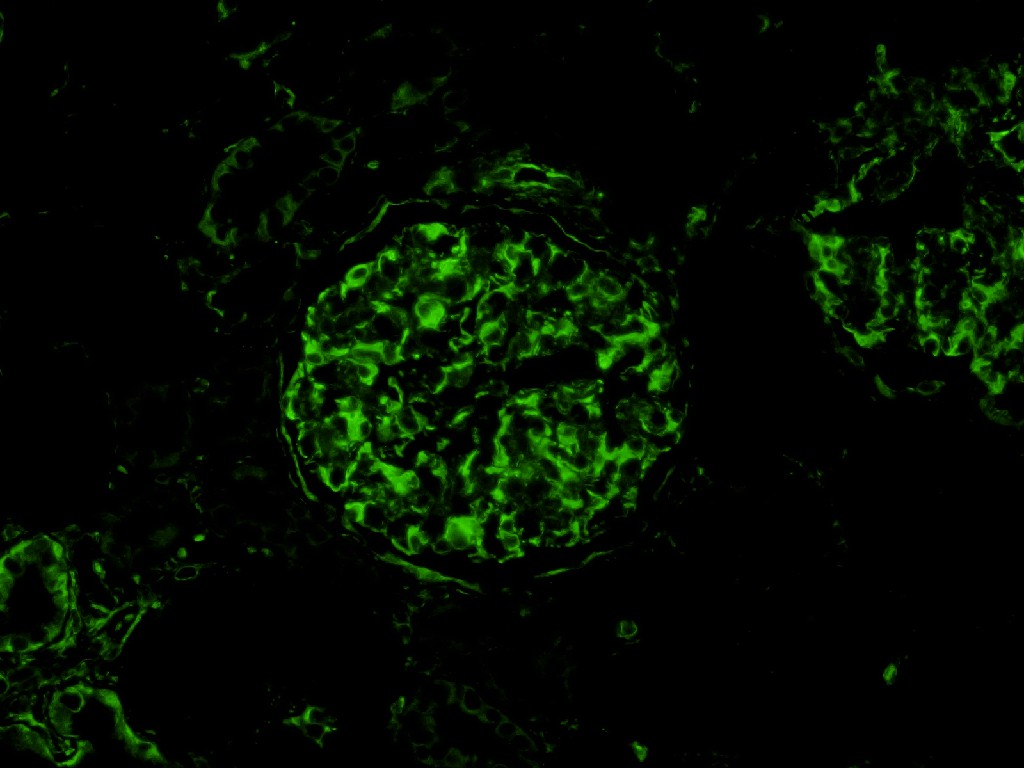

Supplement: Supplementary file 2 — Source data Fig. 1 [file 44321_2025_315_MOESM2_ESM.zip › Figure 1/F1A/1-GLDC-PDGFRbeta/Lee III/9 (1).jpg]
