# Supplementary figures and images for: Glycine decarboxylase advances IgA nephropathy by boosting mesangial cell proliferation through the pyrimidine pathway (part 3 of 7)
Source: EMBO Mol Med. 2025 Oct 13;17(11):3039–63. doi: 10.1038/s44321-025-00315-2 (PMC12603144; doi:10.1038/s44321-025-00315-2)

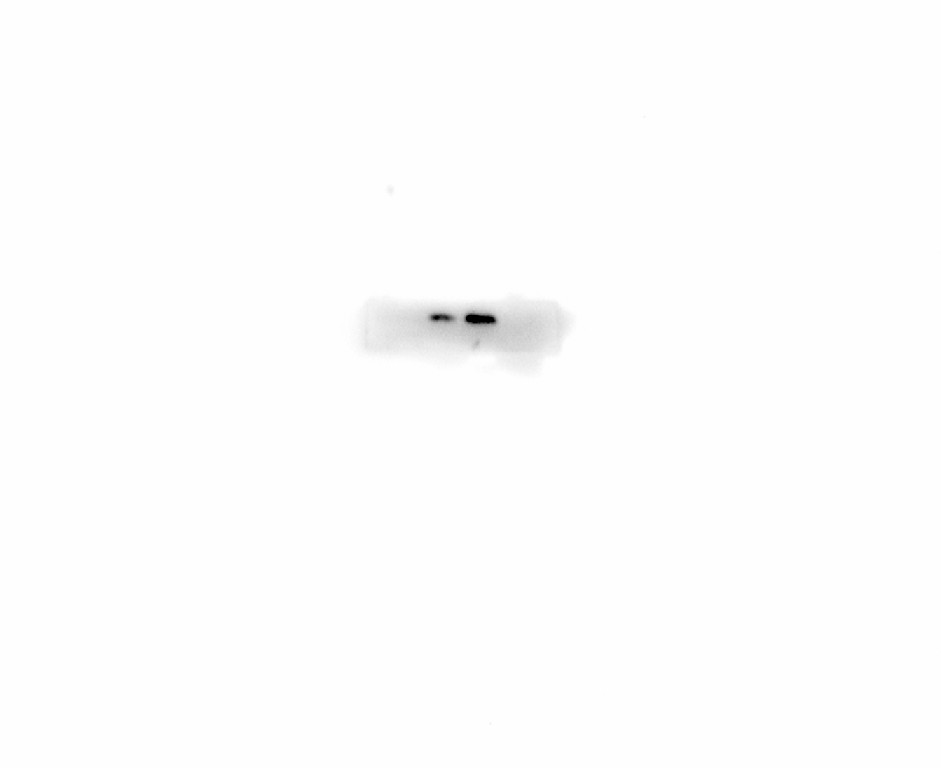

Supplement: Supplementary file 3 — Source data Fig. 2 [file 44321_2025_315_MOESM3_ESM.zip › Figure 2/F2C-WB/1-F2C left/1-3-GLDC.jpg]

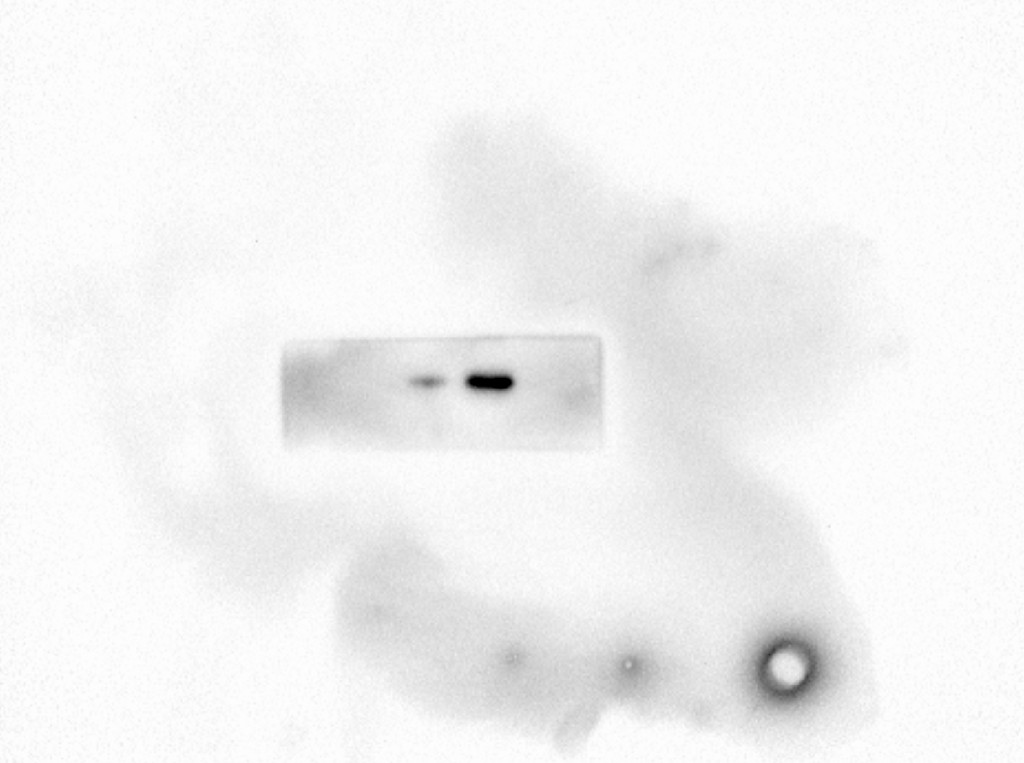

Supplement: Supplementary file 3 — Source data Fig. 2 [file 44321_2025_315_MOESM3_ESM.zip › Figure 2/F2C-WB/1-F2C left/2-1-PCNA.jpg]

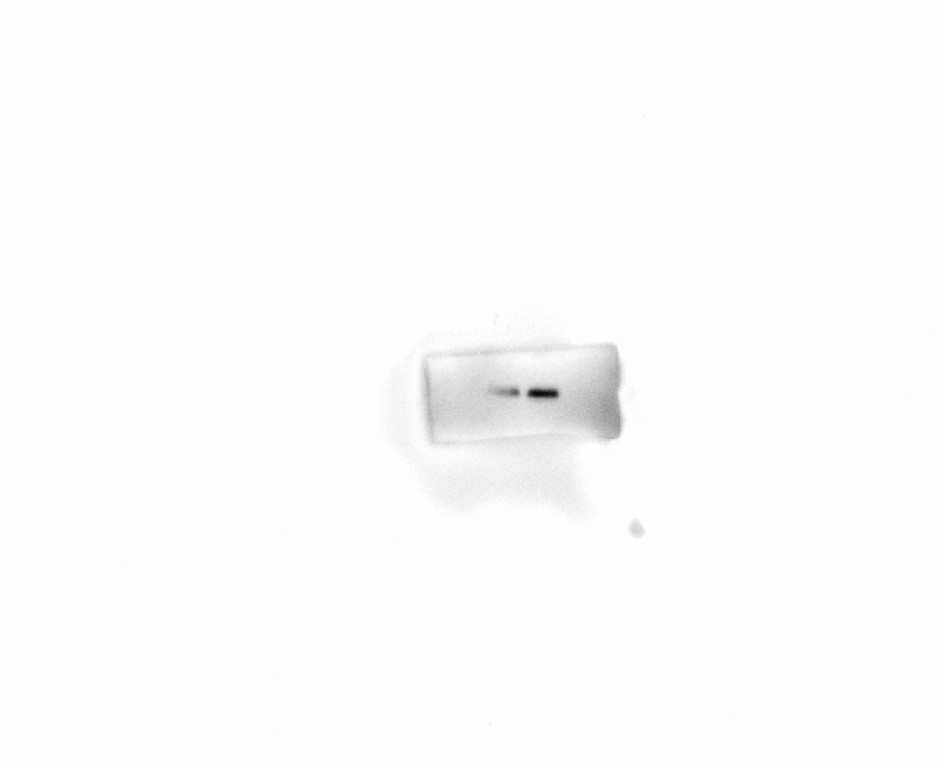

Supplement: Supplementary file 3 — Source data Fig. 2 [file 44321_2025_315_MOESM3_ESM.zip › Figure 2/F2C-WB/1-F2C left/2-2-PCNA.jpg]

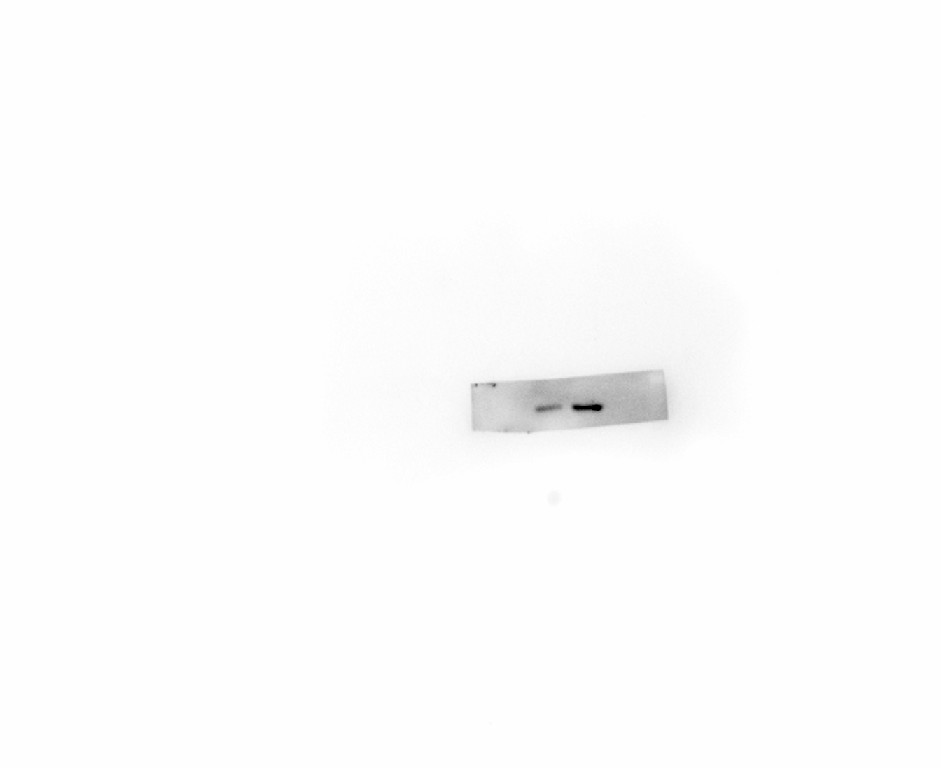

Supplement: Supplementary file 3 — Source data Fig. 2 [file 44321_2025_315_MOESM3_ESM.zip › Figure 2/F2C-WB/1-F2C left/2-3-PCNA.jpg]

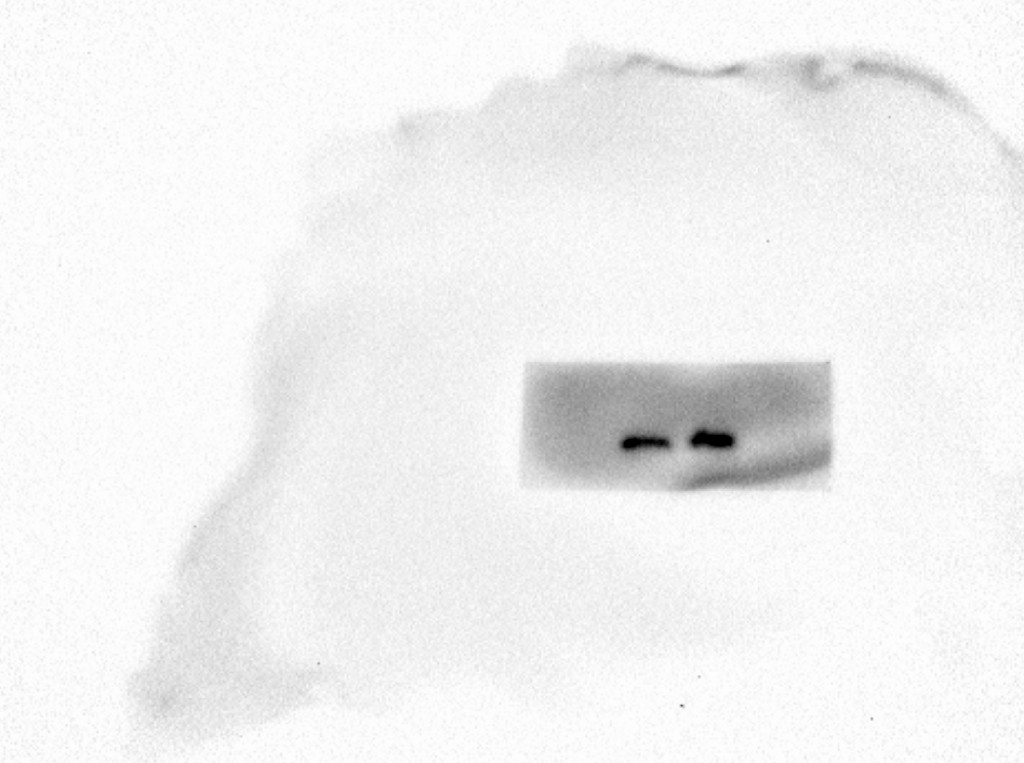

Supplement: Supplementary file 3 — Source data Fig. 2 [file 44321_2025_315_MOESM3_ESM.zip › Figure 2/F2C-WB/1-F2C left/3-1-Cyclin D1.jpg]

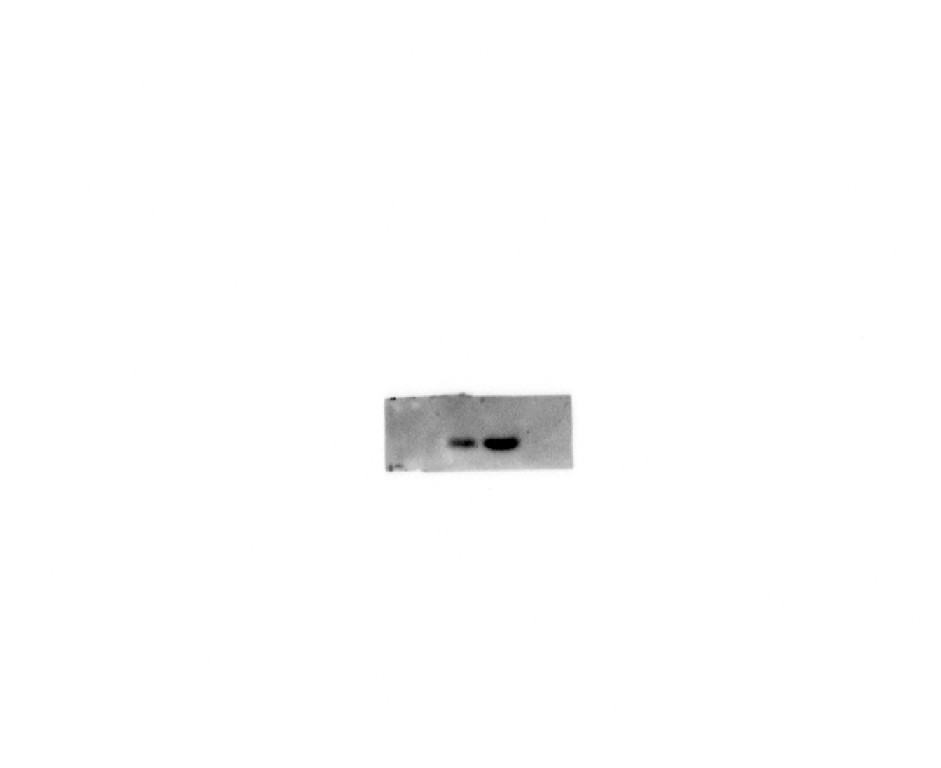

Supplement: Supplementary file 3 — Source data Fig. 2 [file 44321_2025_315_MOESM3_ESM.zip › Figure 2/F2C-WB/1-F2C left/3-2-Cyclin D1.jpg]

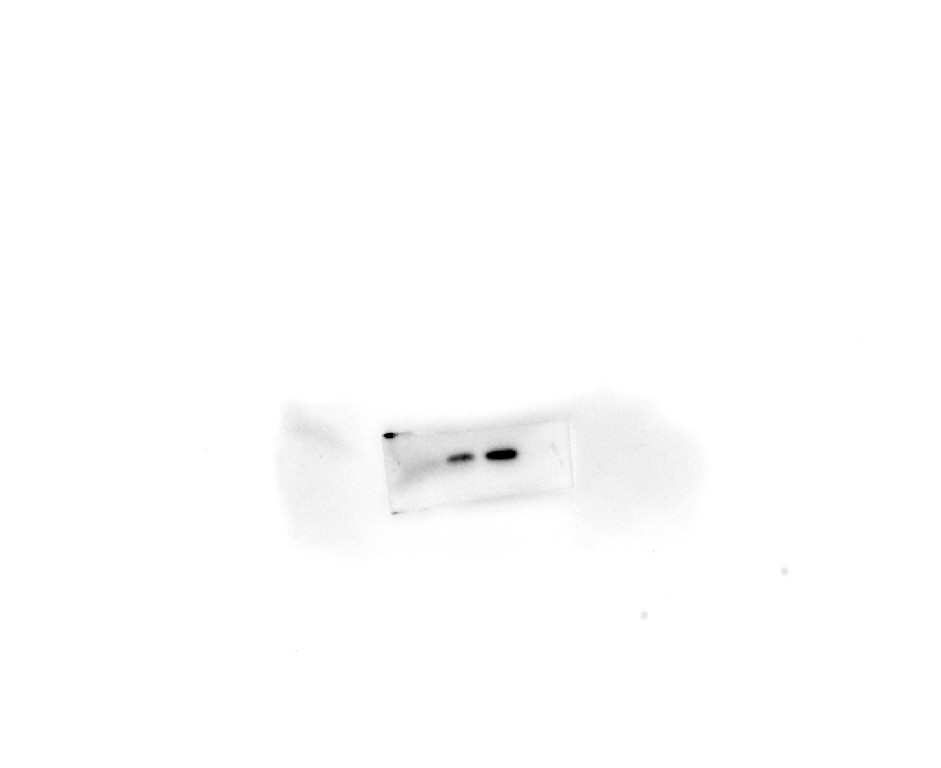

Supplement: Supplementary file 3 — Source data Fig. 2 [file 44321_2025_315_MOESM3_ESM.zip › Figure 2/F2C-WB/1-F2C left/3-3-Cyclin D1.jpg]

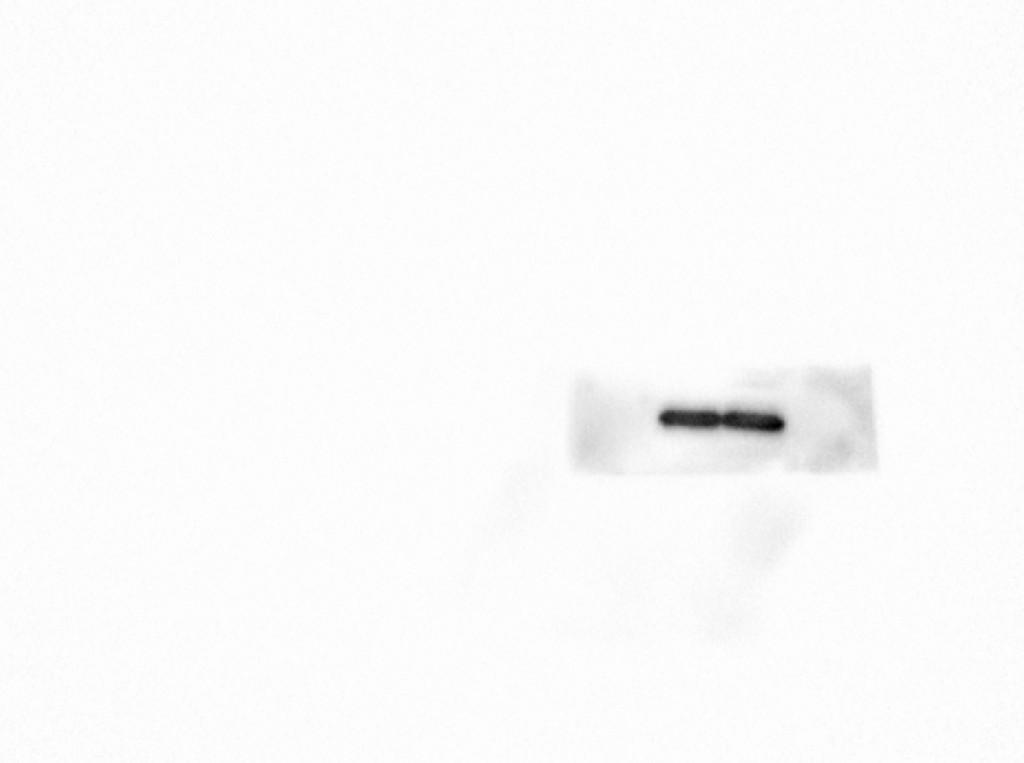

Supplement: Supplementary file 3 — Source data Fig. 2 [file 44321_2025_315_MOESM3_ESM.zip › Figure 2/F2C-WB/1-F2C left/4-1-beta-actin.jpg]

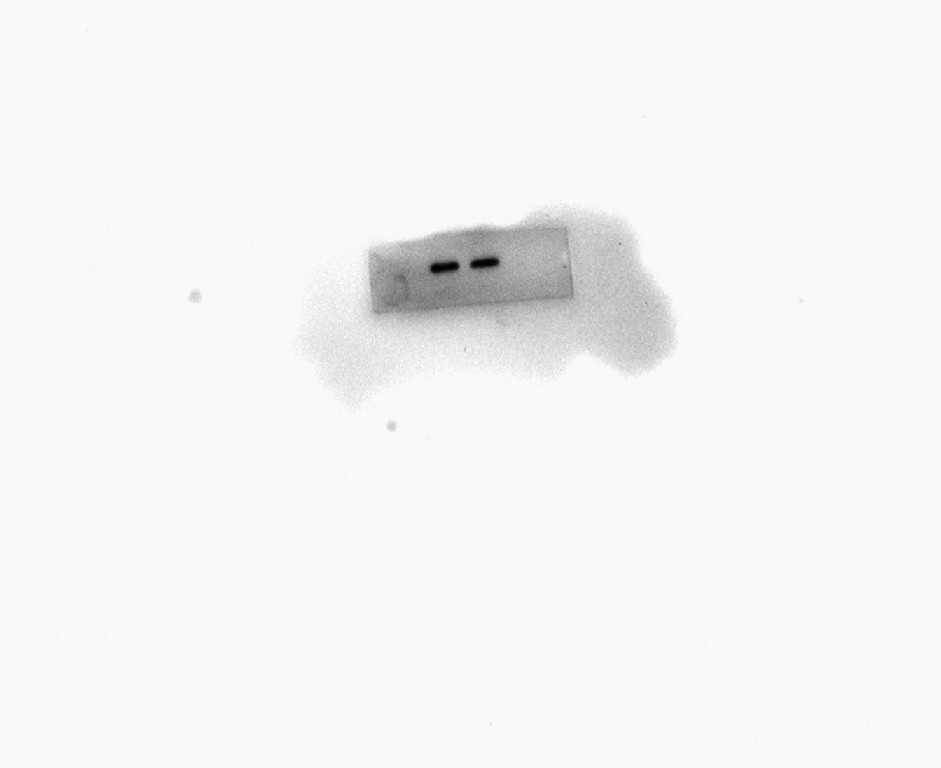

Supplement: Supplementary file 3 — Source data Fig. 2 [file 44321_2025_315_MOESM3_ESM.zip › Figure 2/F2C-WB/1-F2C left/4-2-beta actin.jpg]

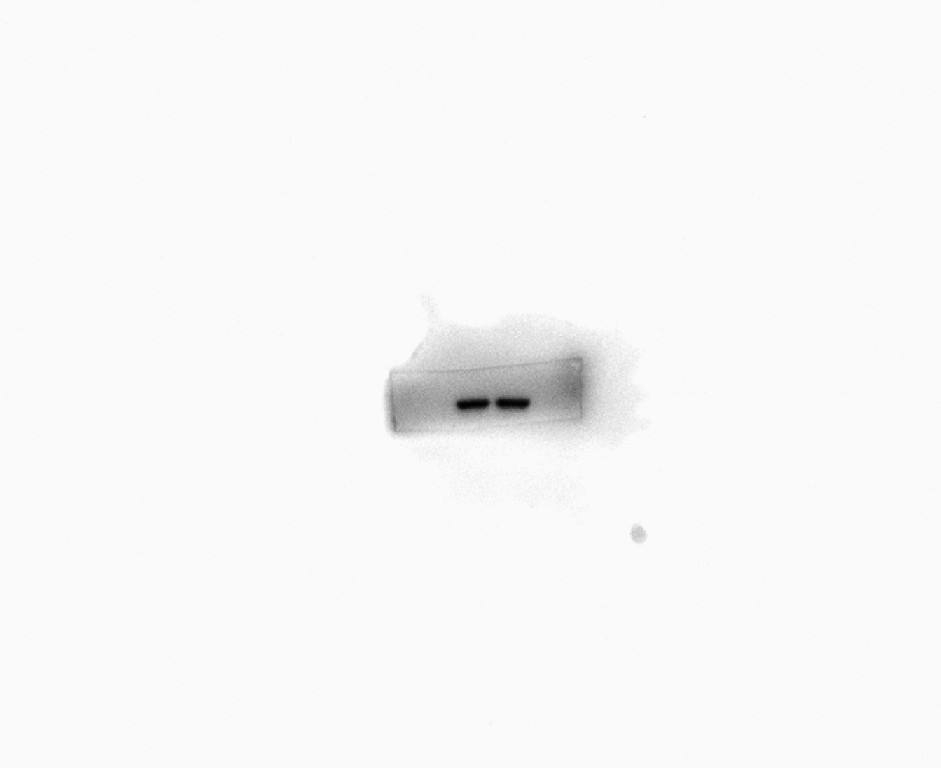

Supplement: Supplementary file 3 — Source data Fig. 2 [file 44321_2025_315_MOESM3_ESM.zip › Figure 2/F2C-WB/1-F2C left/4-3-beta-actin.jpg]

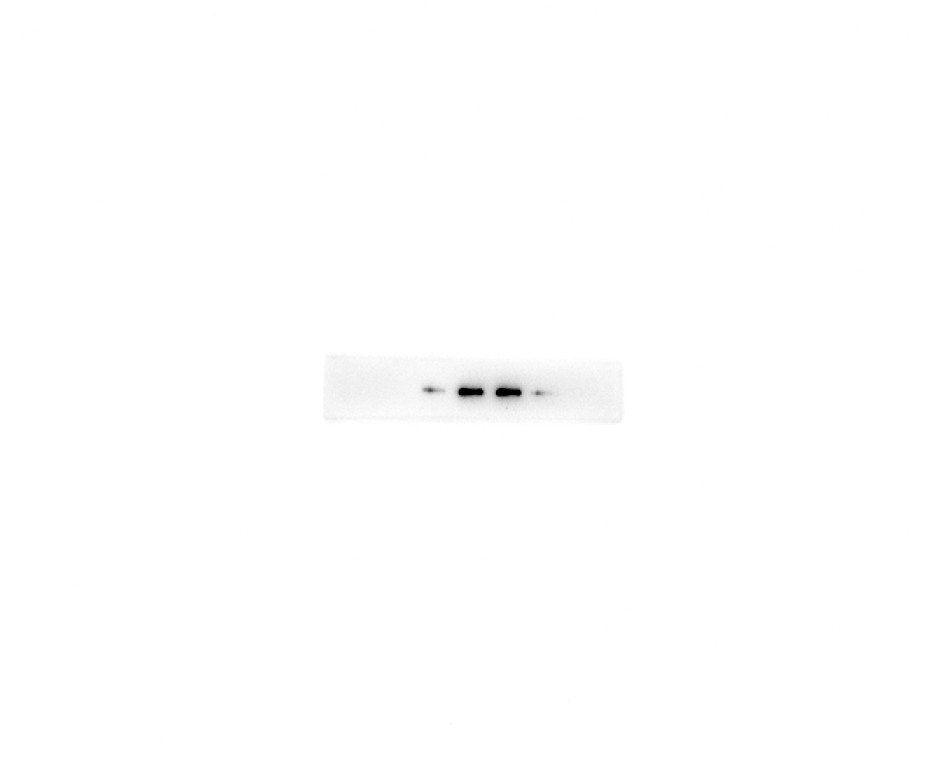

Supplement: Supplementary file 3 — Source data Fig. 2 [file 44321_2025_315_MOESM3_ESM.zip › Figure 2/F2C-WB/2-F2C right/1-1-GLDC.jpg]

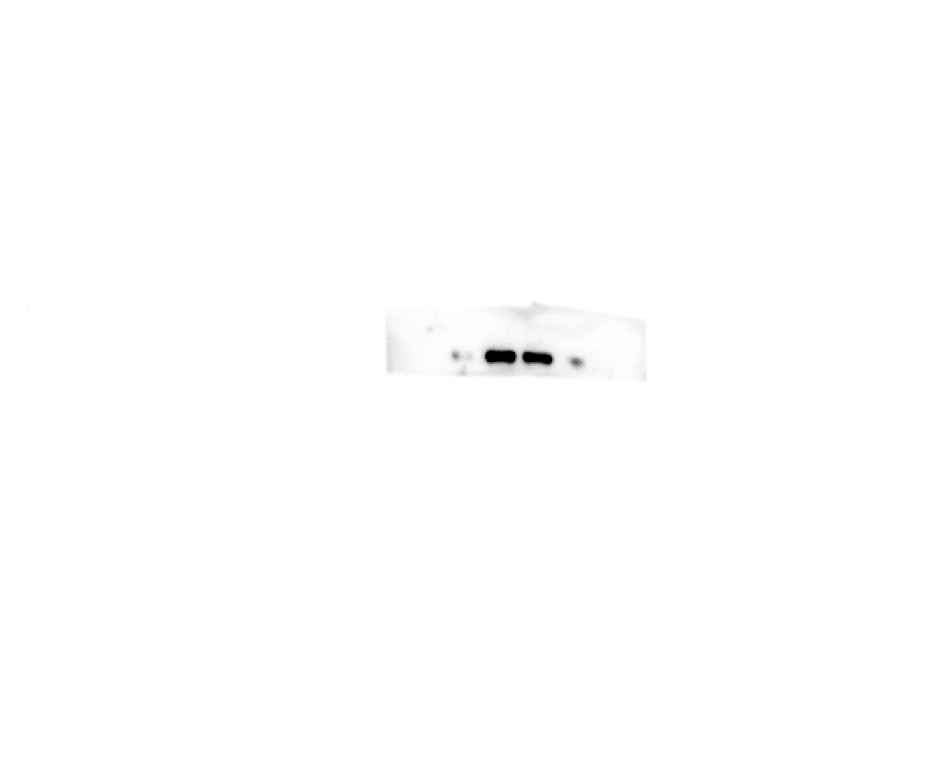

Supplement: Supplementary file 3 — Source data Fig. 2 [file 44321_2025_315_MOESM3_ESM.zip › Figure 2/F2C-WB/2-F2C right/1-2-GLDC.jpg]

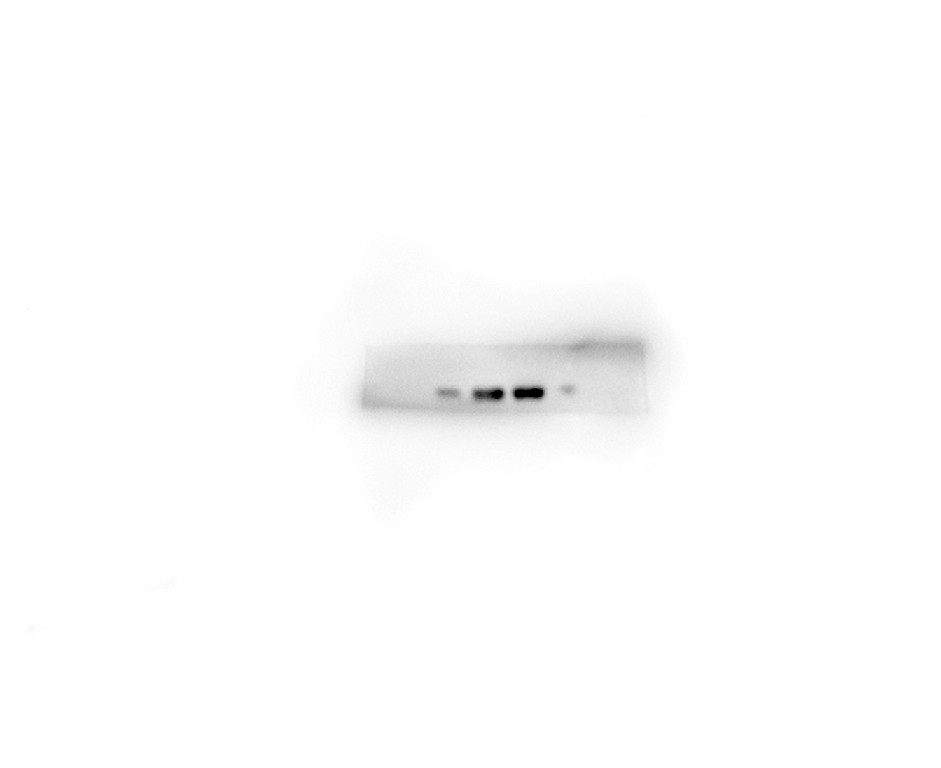

Supplement: Supplementary file 3 — Source data Fig. 2 [file 44321_2025_315_MOESM3_ESM.zip › Figure 2/F2C-WB/2-F2C right/1-3-GLDC.jpg]

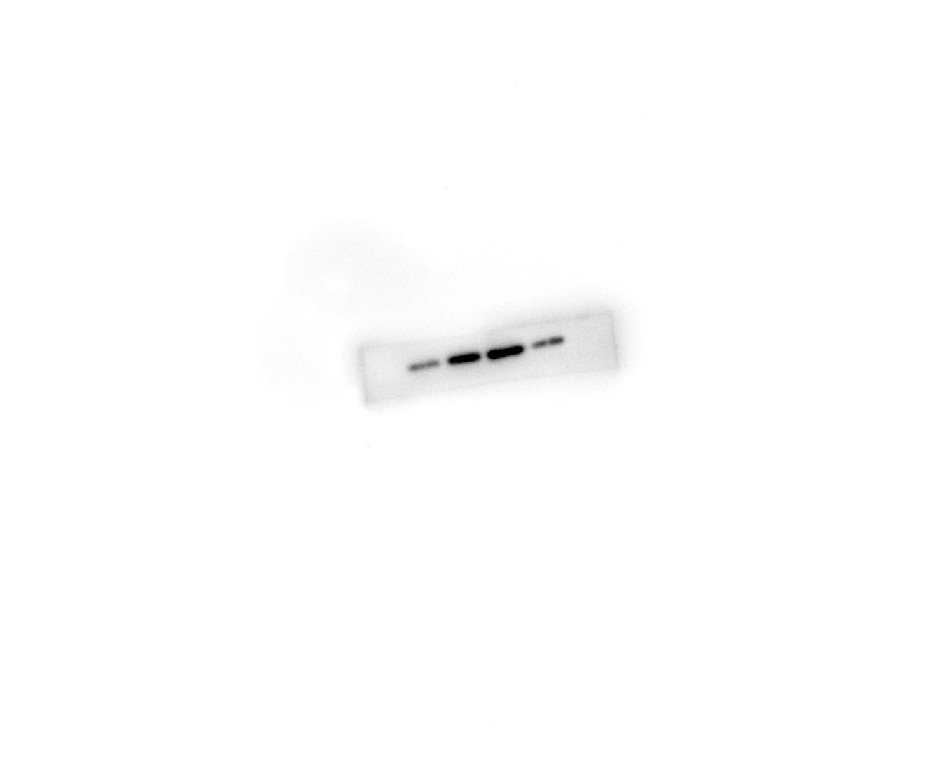

Supplement: Supplementary file 3 — Source data Fig. 2 [file 44321_2025_315_MOESM3_ESM.zip › Figure 2/F2C-WB/2-F2C right/2-1-PCNA.jpg]

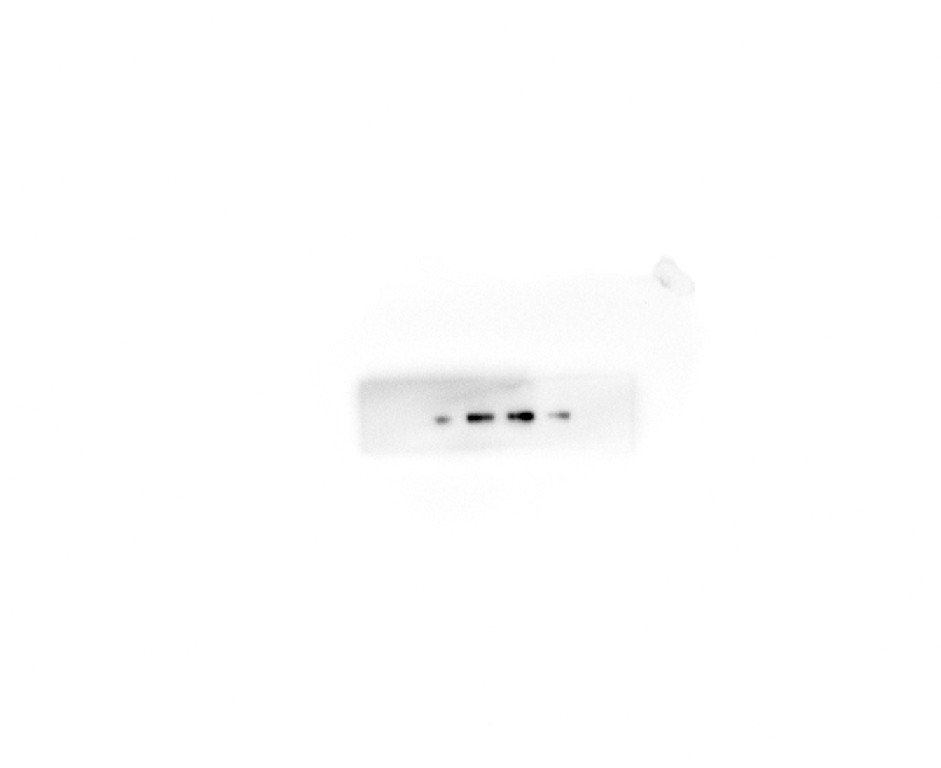

Supplement: Supplementary file 3 — Source data Fig. 2 [file 44321_2025_315_MOESM3_ESM.zip › Figure 2/F2C-WB/2-F2C right/2-2-PCNA.jpg]

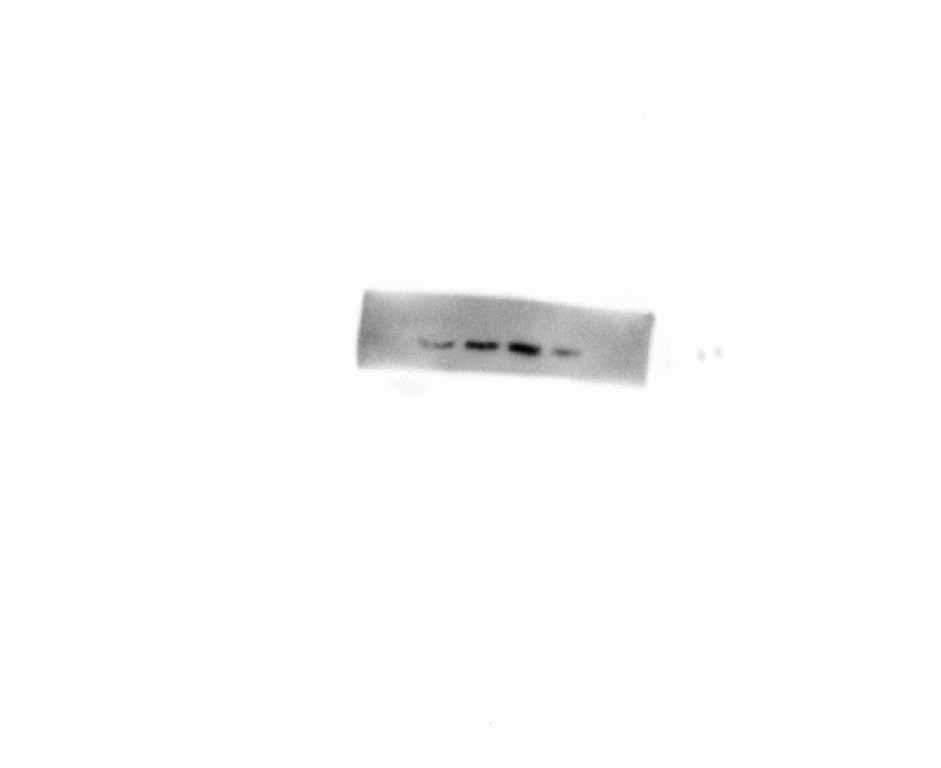

Supplement: Supplementary file 3 — Source data Fig. 2 [file 44321_2025_315_MOESM3_ESM.zip › Figure 2/F2C-WB/2-F2C right/2-3-PCNA.jpg]

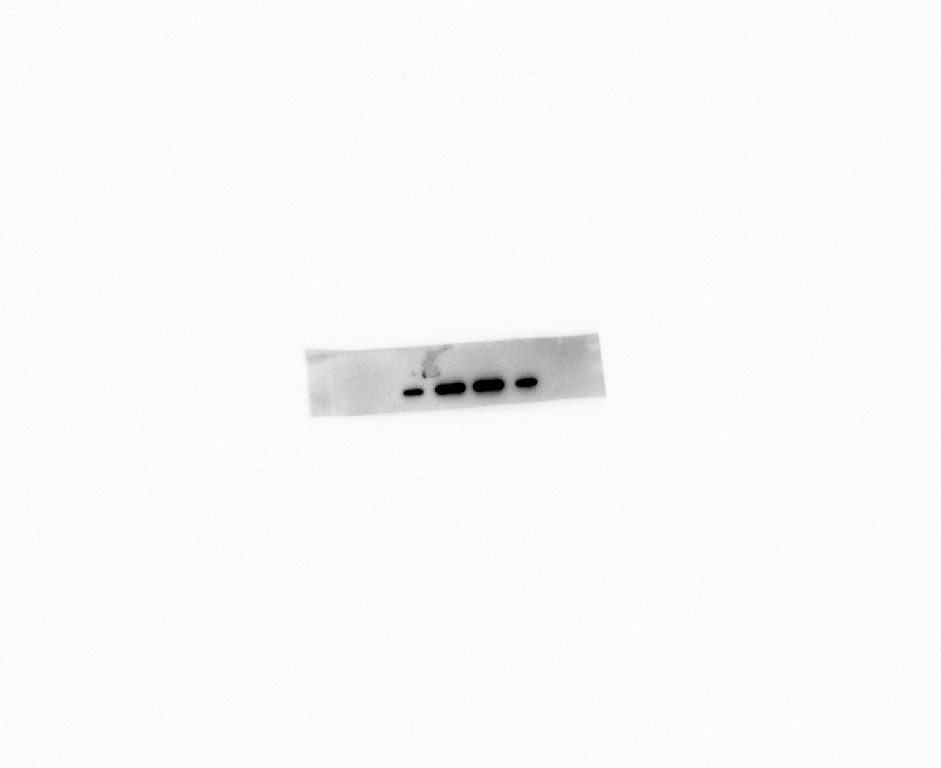

Supplement: Supplementary file 3 — Source data Fig. 2 [file 44321_2025_315_MOESM3_ESM.zip › Figure 2/F2C-WB/2-F2C right/3-1-cyclin-D1.jpg]

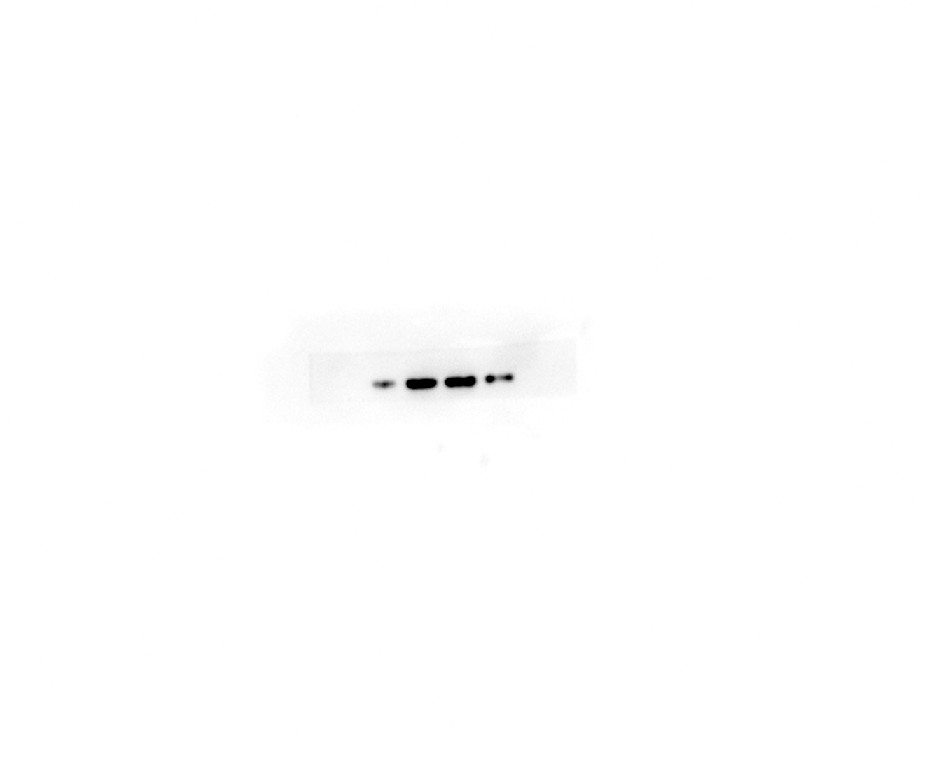

Supplement: Supplementary file 3 — Source data Fig. 2 [file 44321_2025_315_MOESM3_ESM.zip › Figure 2/F2C-WB/2-F2C right/3-2-cyclin D1.jpg]

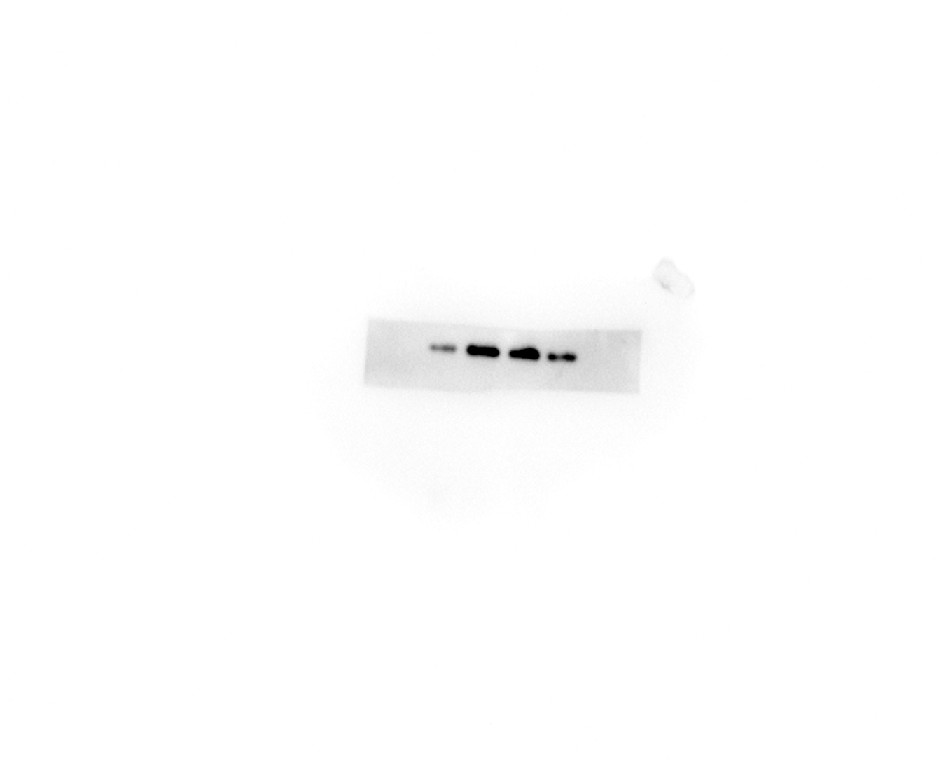

Supplement: Supplementary file 3 — Source data Fig. 2 [file 44321_2025_315_MOESM3_ESM.zip › Figure 2/F2C-WB/2-F2C right/3-3-cyclin D1.jpg]

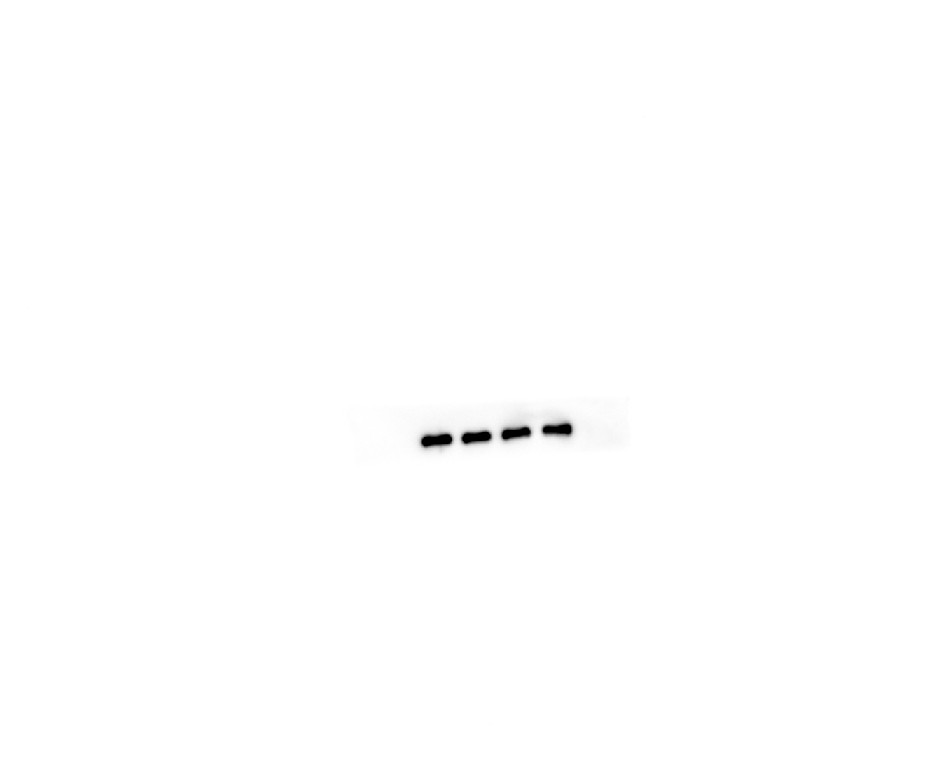

Supplement: Supplementary file 3 — Source data Fig. 2 [file 44321_2025_315_MOESM3_ESM.zip › Figure 2/F2C-WB/2-F2C right/4-1-beta-actin.jpg]

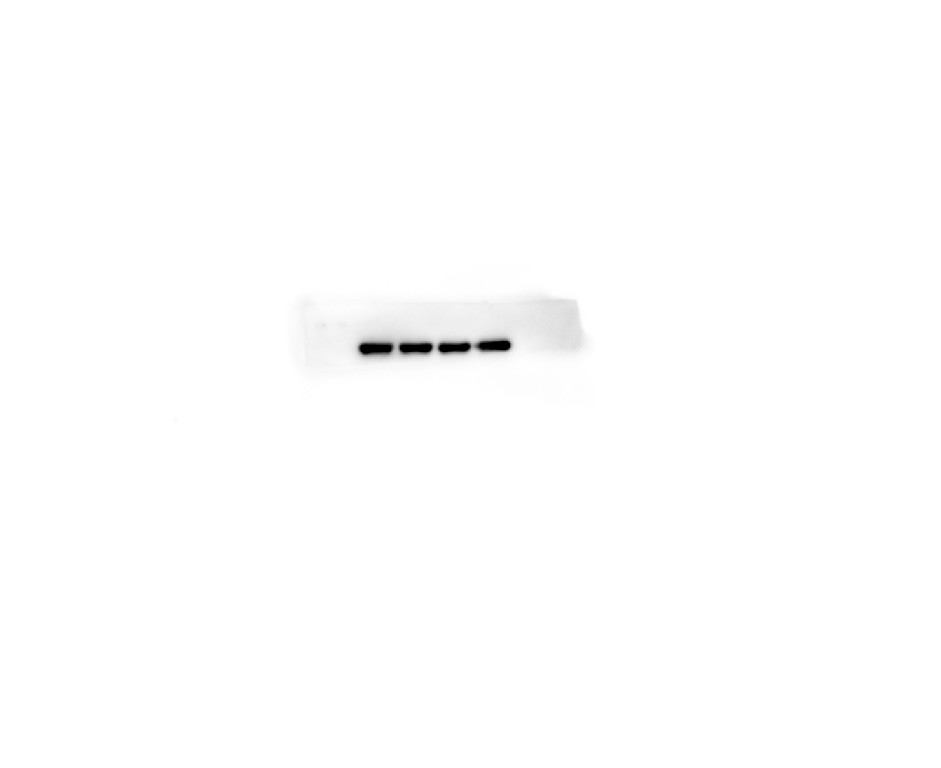

Supplement: Supplementary file 3 — Source data Fig. 2 [file 44321_2025_315_MOESM3_ESM.zip › Figure 2/F2C-WB/2-F2C right/4-2-beta actin.jpg]

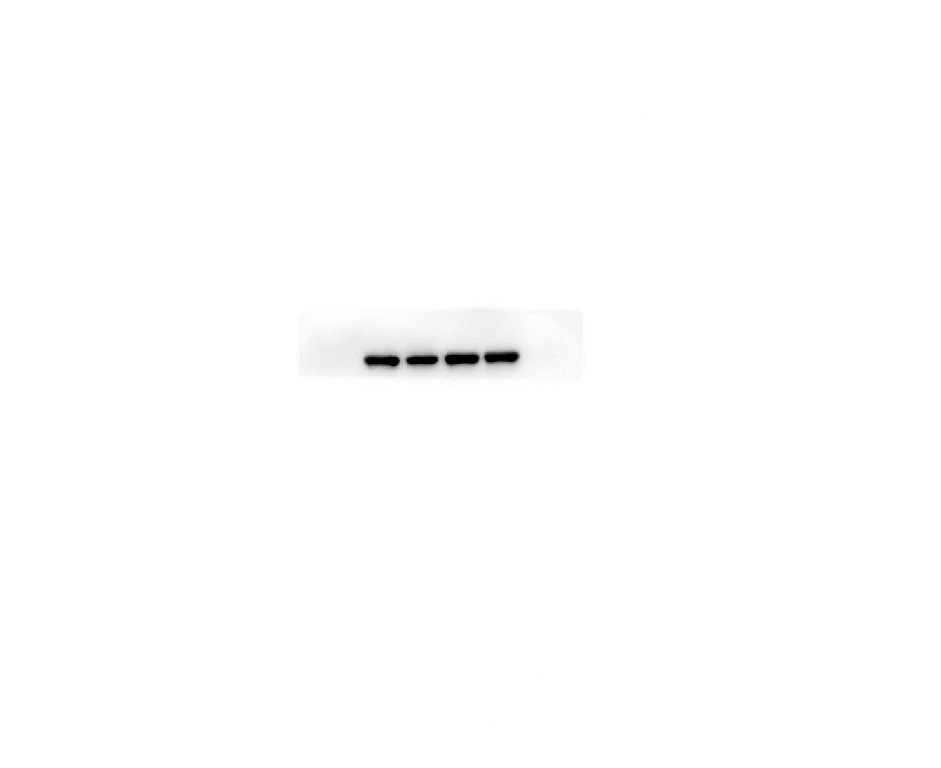

Supplement: Supplementary file 3 — Source data Fig. 2 [file 44321_2025_315_MOESM3_ESM.zip › Figure 2/F2C-WB/2-F2C right/4-3-beta actin.jpg]

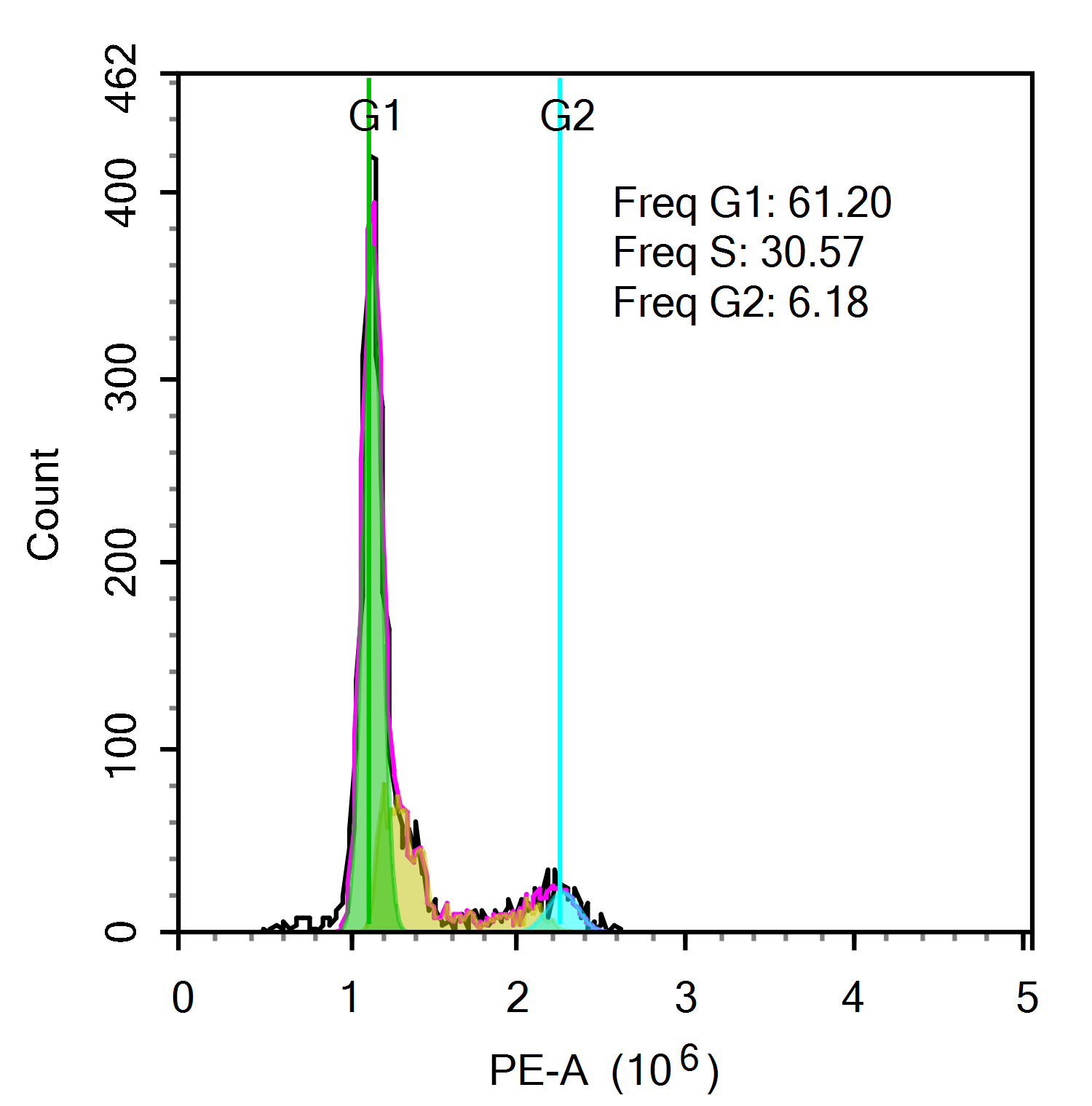

Supplement: Supplementary file 3 — Source data Fig. 2 [file 44321_2025_315_MOESM3_ESM.zip › Figure 2/F2D-cell cycle/1-NC (1).tiff]

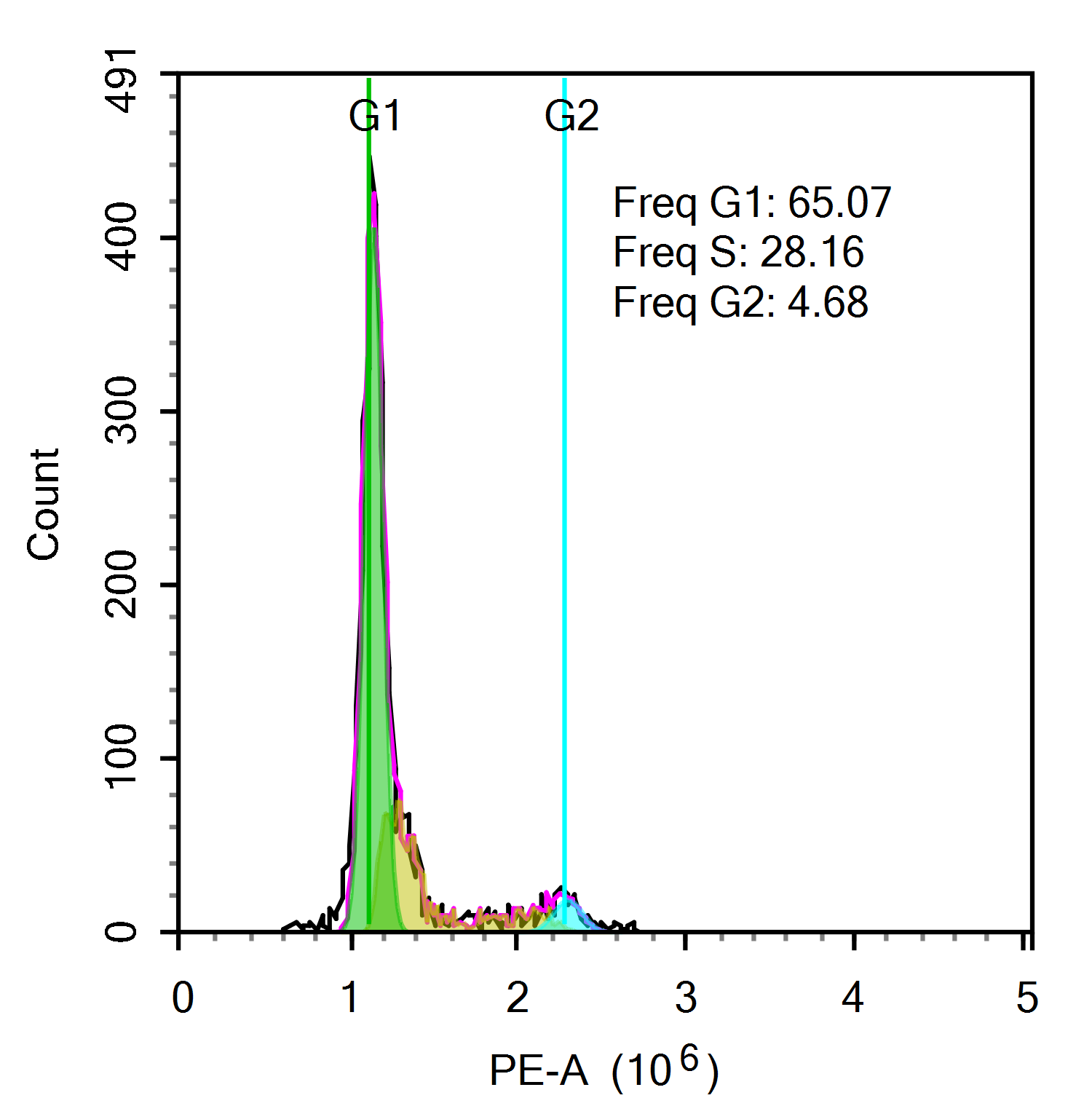

Supplement: Supplementary file 3 — Source data Fig. 2 [file 44321_2025_315_MOESM3_ESM.zip › Figure 2/F2D-cell cycle/1-NC (2).tiff]

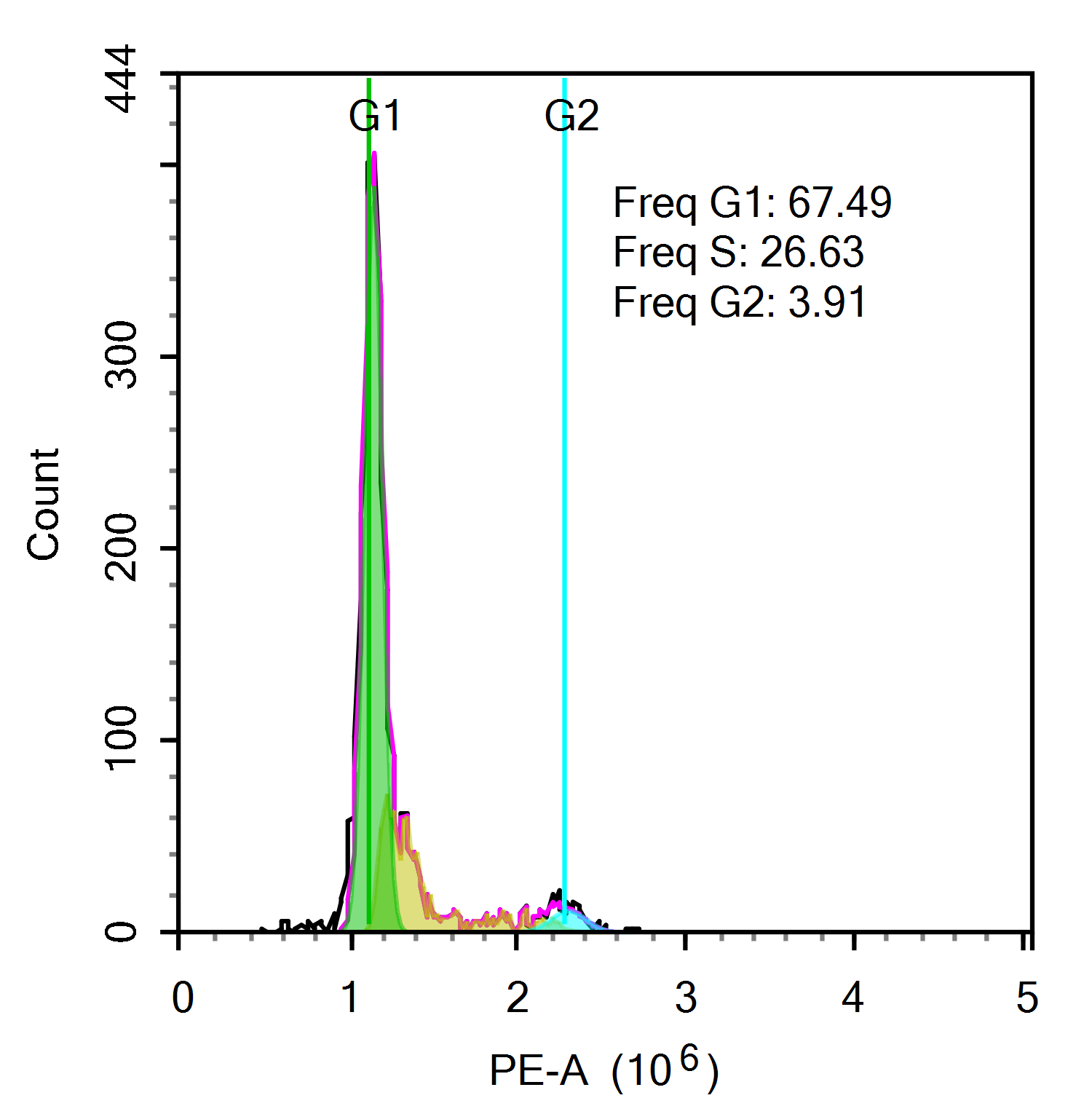

Supplement: Supplementary file 3 — Source data Fig. 2 [file 44321_2025_315_MOESM3_ESM.zip › Figure 2/F2D-cell cycle/1-NC (3).tiff]

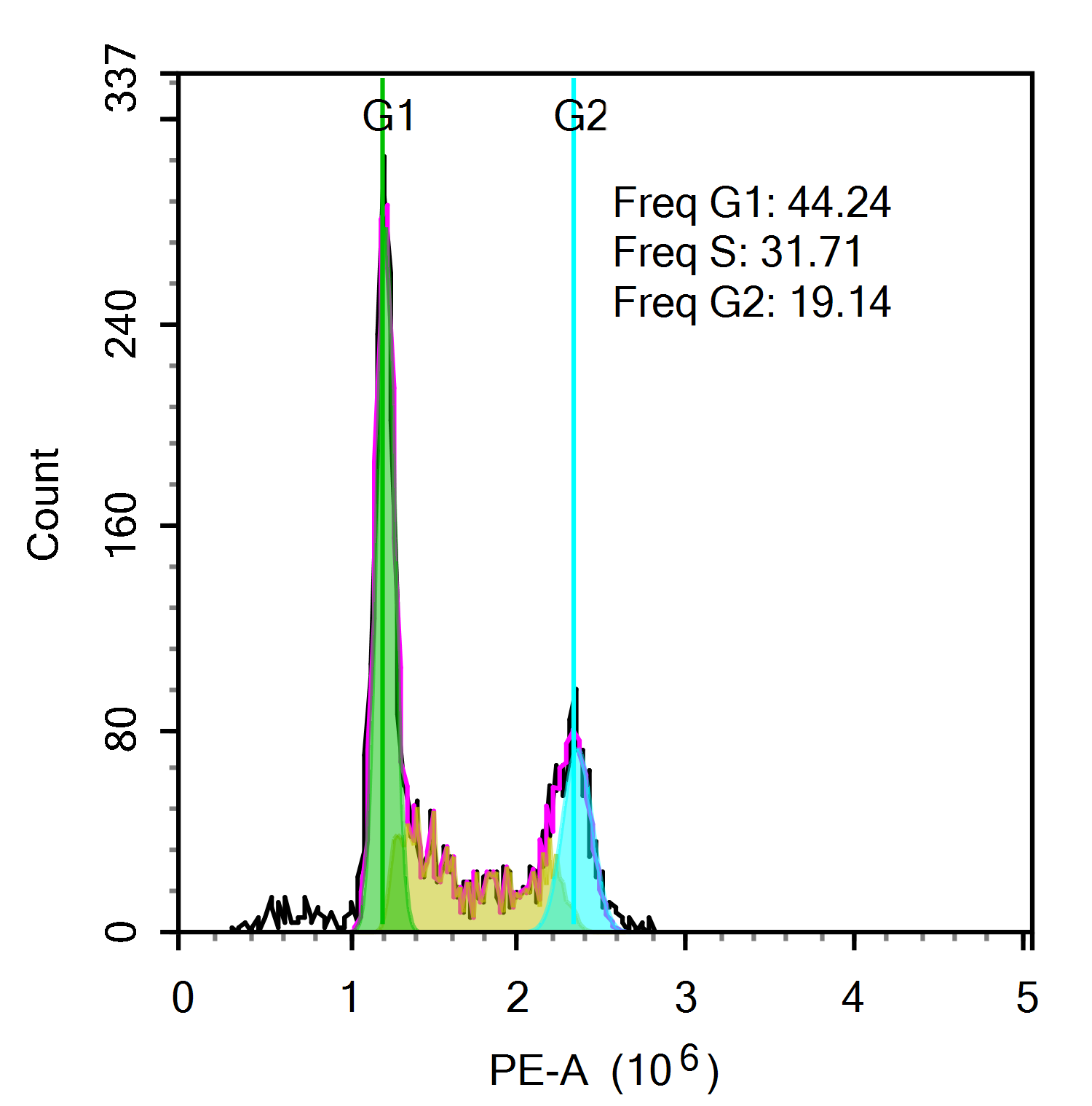

Supplement: Supplementary file 3 — Source data Fig. 2 [file 44321_2025_315_MOESM3_ESM.zip › Figure 2/F2D-cell cycle/2-GLDC (1).tiff]

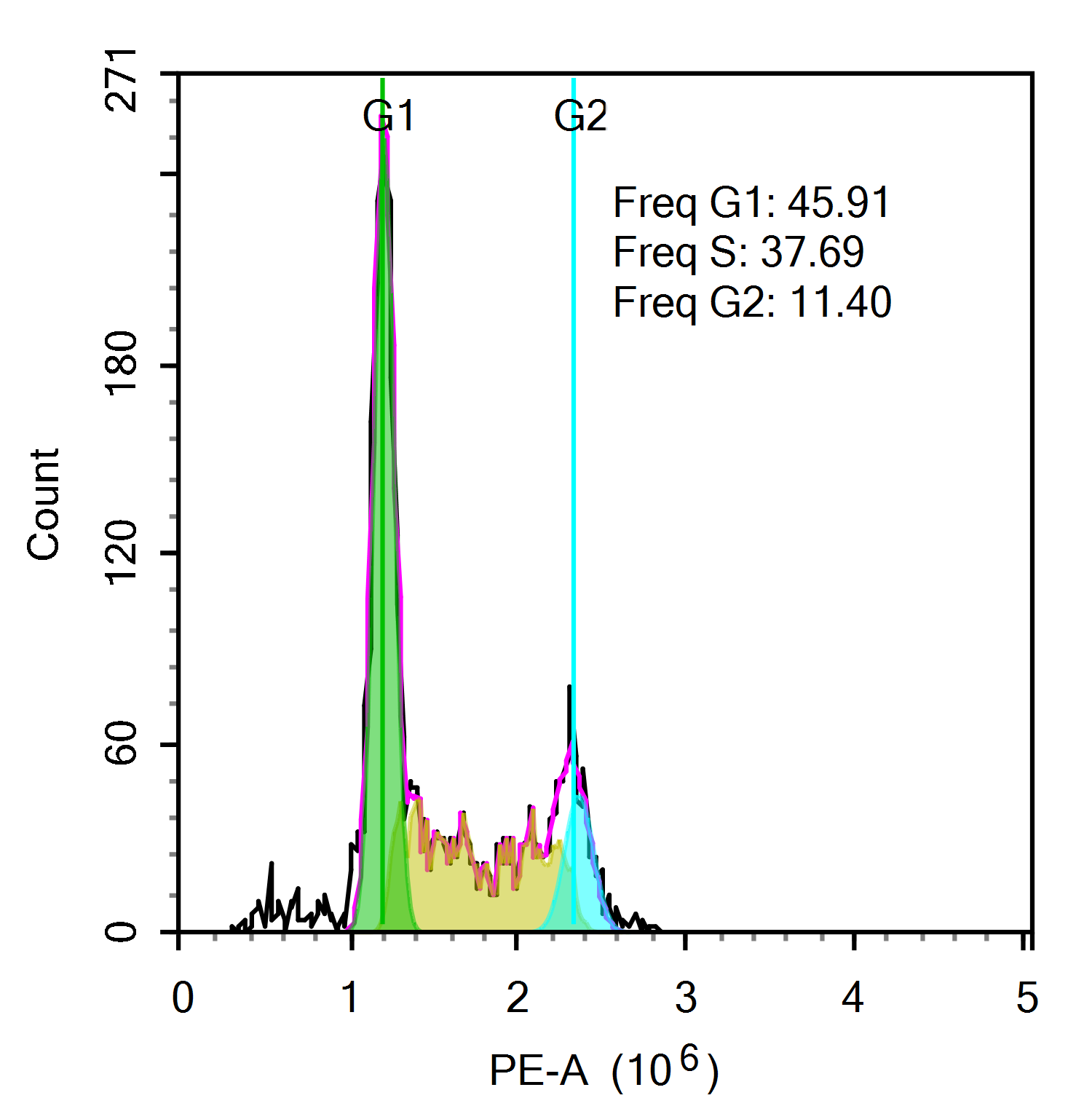

Supplement: Supplementary file 3 — Source data Fig. 2 [file 44321_2025_315_MOESM3_ESM.zip › Figure 2/F2D-cell cycle/2-GLDC (2).tiff]

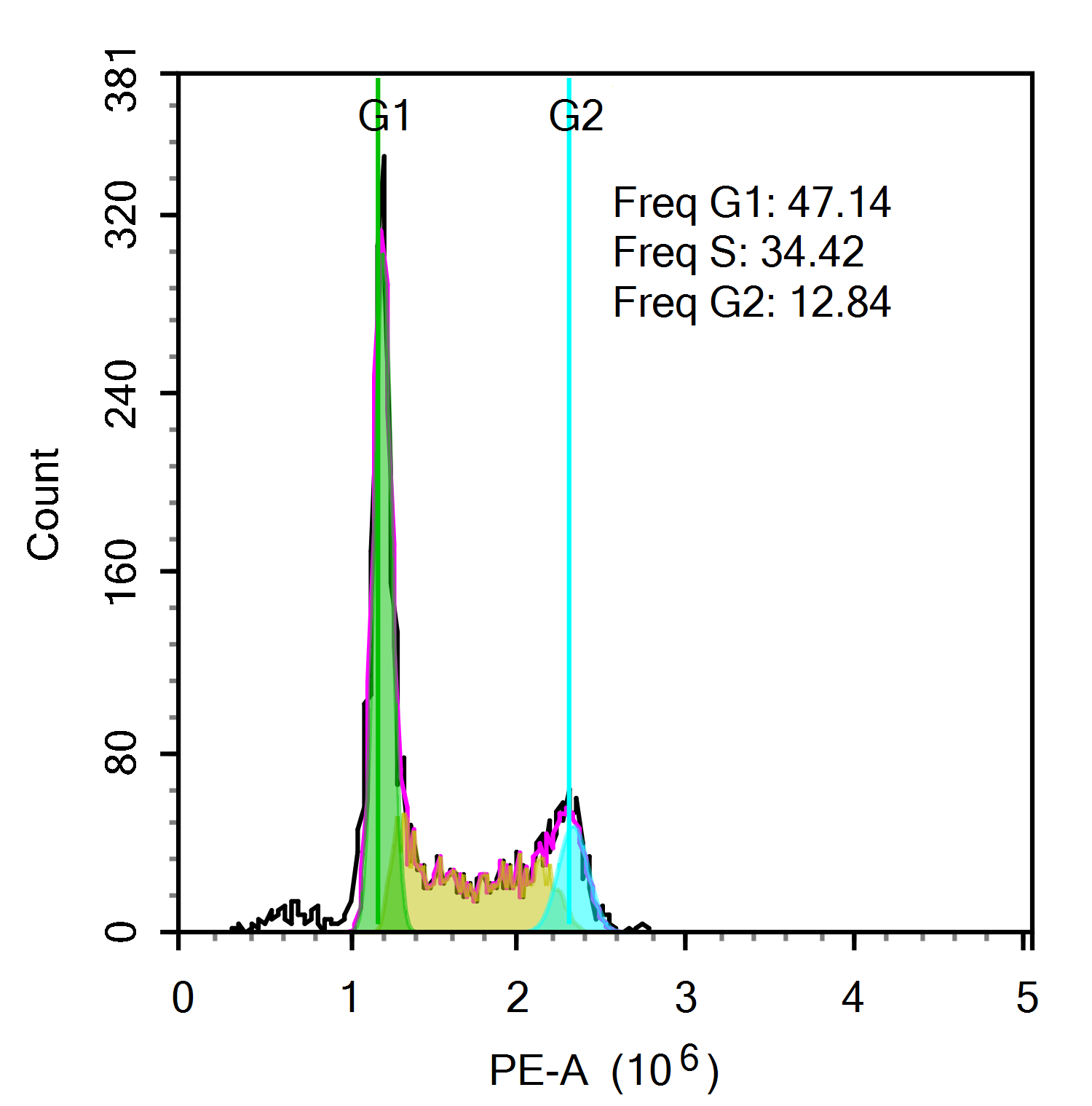

Supplement: Supplementary file 3 — Source data Fig. 2 [file 44321_2025_315_MOESM3_ESM.zip › Figure 2/F2D-cell cycle/2-GLDC (3).tiff]

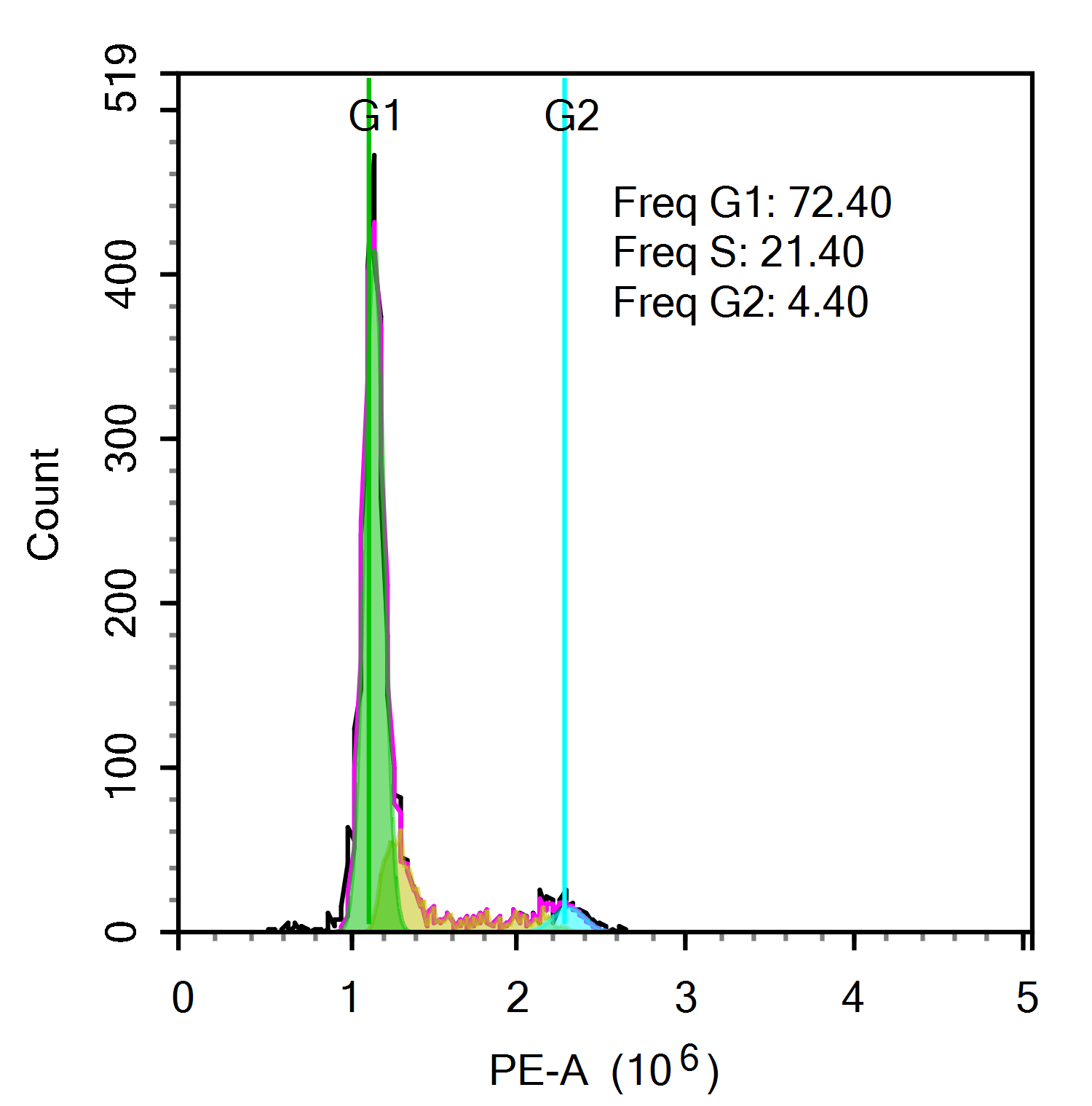

Supplement: Supplementary file 3 — Source data Fig. 2 [file 44321_2025_315_MOESM3_ESM.zip › Figure 2/F2E-cell cycle/1-ControL (1).tiff]

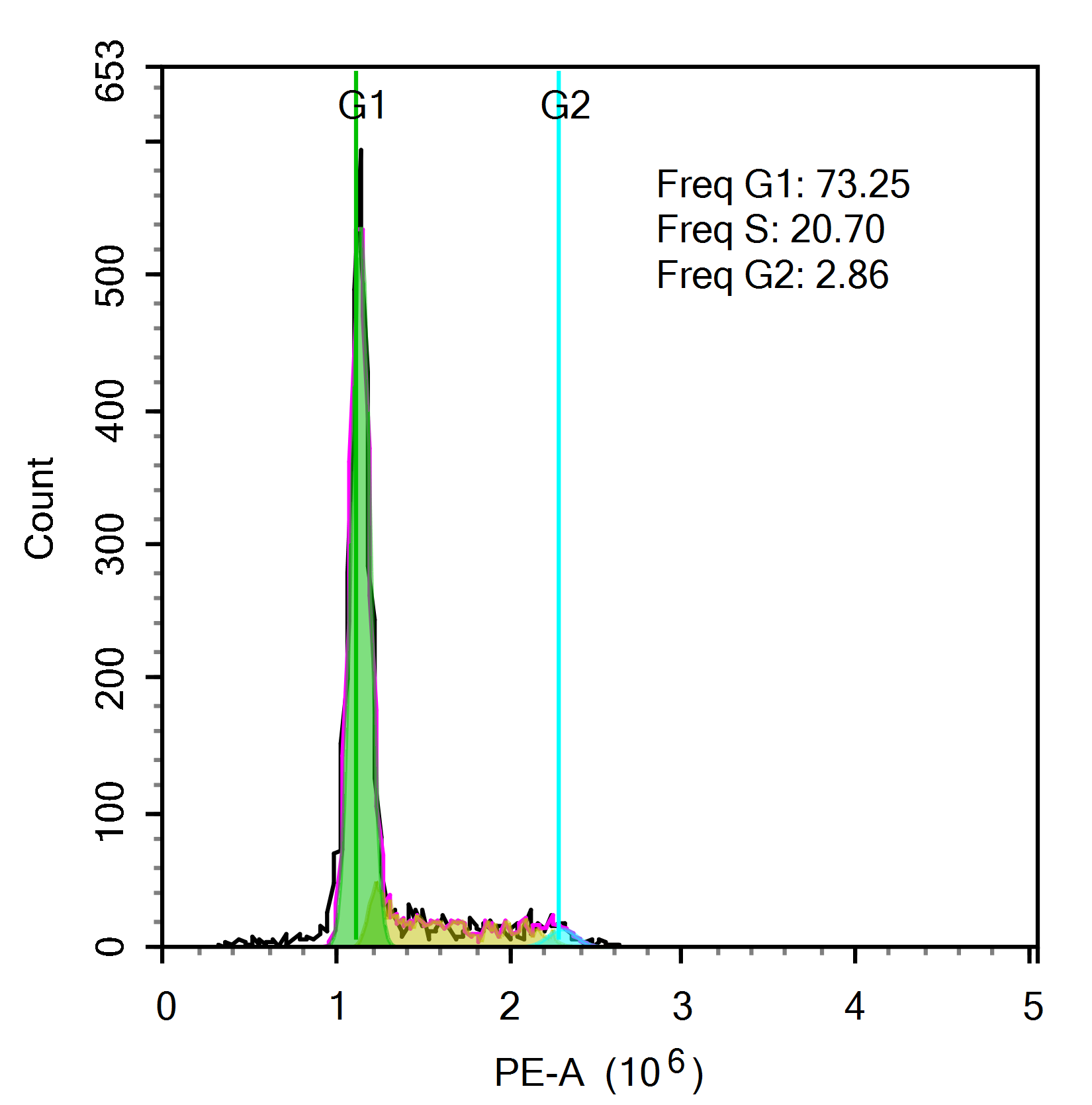

Supplement: Supplementary file 3 — Source data Fig. 2 [file 44321_2025_315_MOESM3_ESM.zip › Figure 2/F2E-cell cycle/1-ControL (2).tiff]

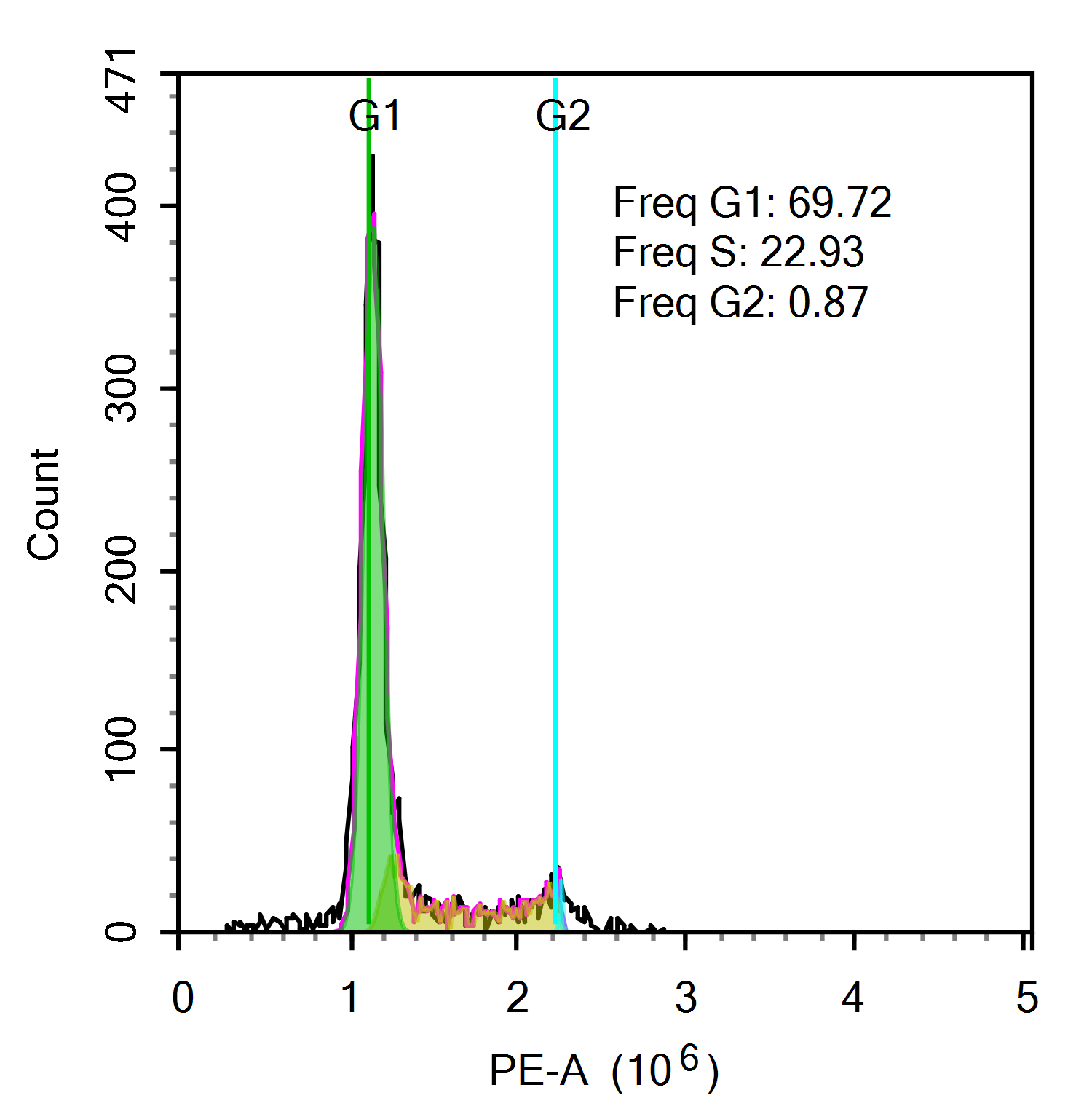

Supplement: Supplementary file 3 — Source data Fig. 2 [file 44321_2025_315_MOESM3_ESM.zip › Figure 2/F2E-cell cycle/1-ControL (3).tiff]

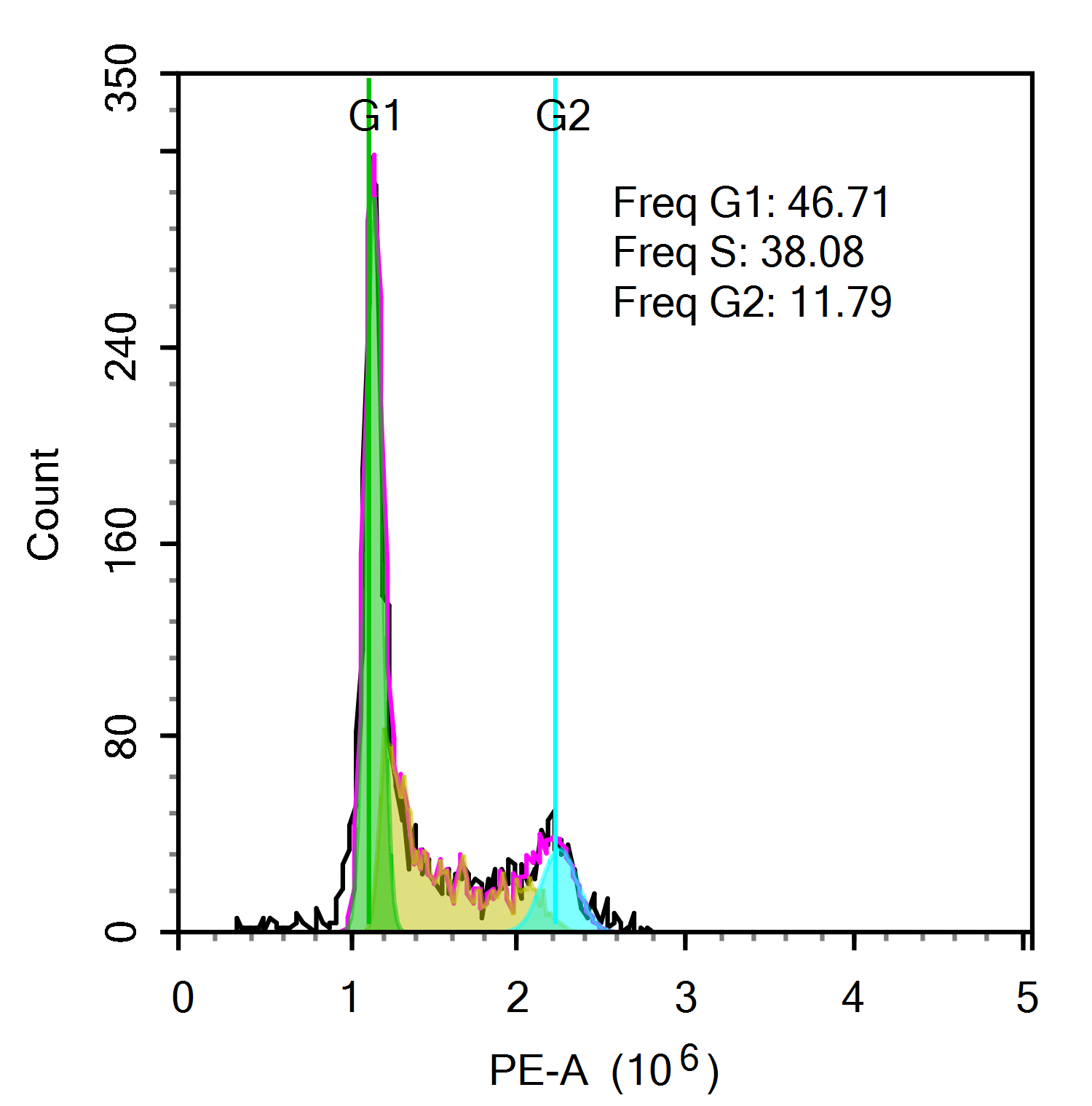

Supplement: Supplementary file 3 — Source data Fig. 2 [file 44321_2025_315_MOESM3_ESM.zip › Figure 2/F2E-cell cycle/2-pIgA(1).tiff]

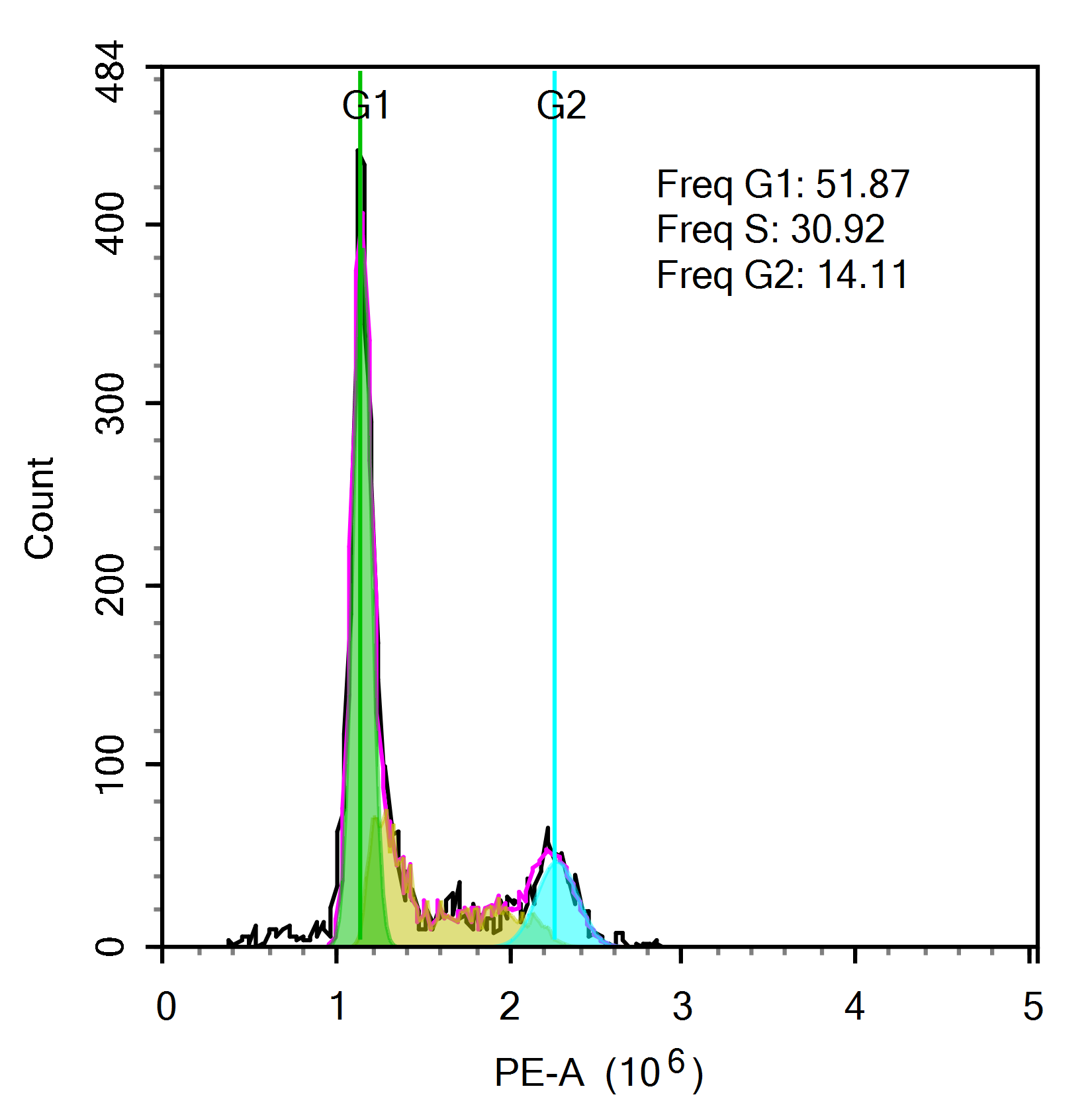

Supplement: Supplementary file 3 — Source data Fig. 2 [file 44321_2025_315_MOESM3_ESM.zip › Figure 2/F2E-cell cycle/2-pIgA(2).tiff]

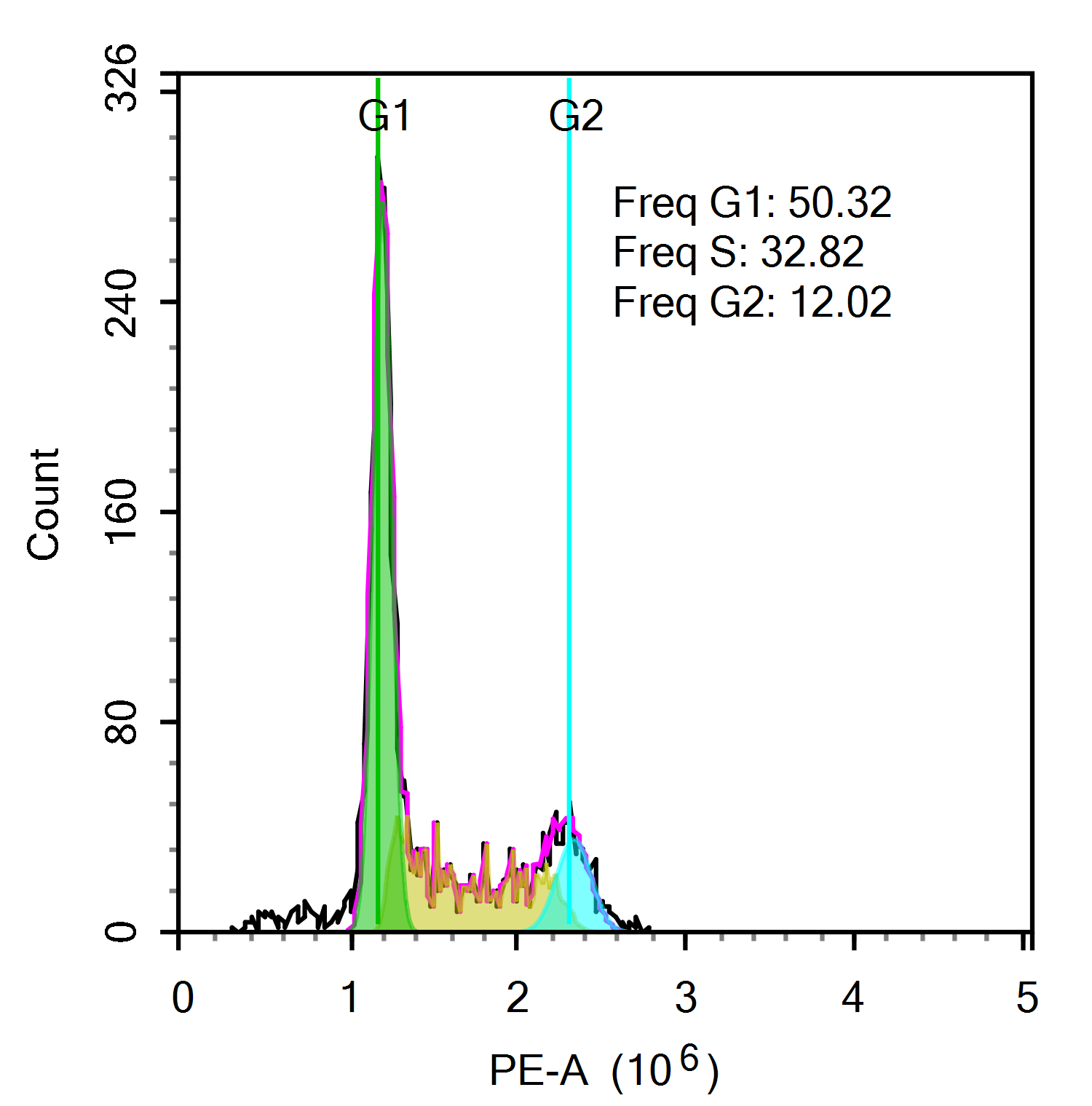

Supplement: Supplementary file 3 — Source data Fig. 2 [file 44321_2025_315_MOESM3_ESM.zip › Figure 2/F2E-cell cycle/2-pIgA(3).tiff]

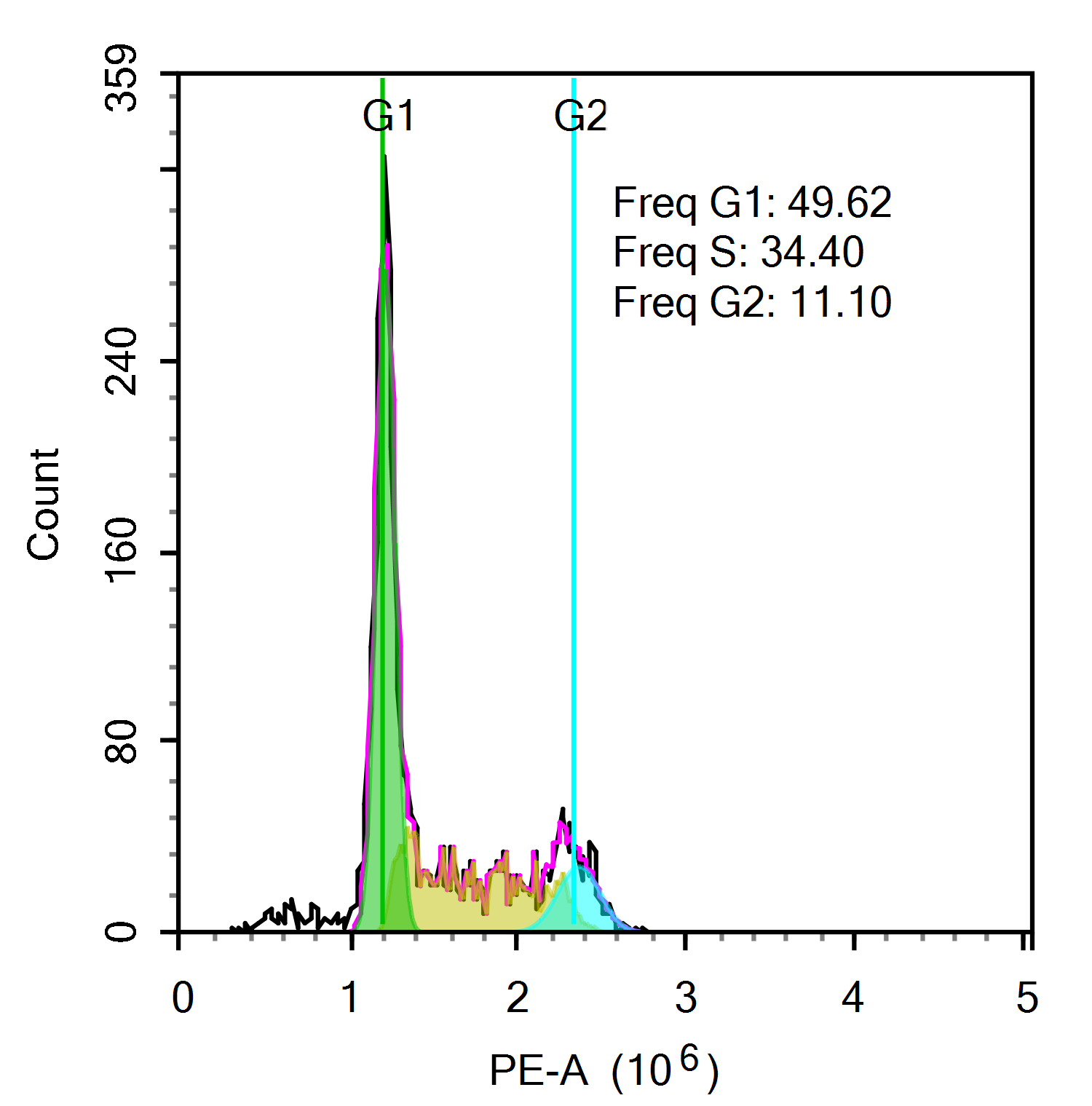

Supplement: Supplementary file 3 — Source data Fig. 2 [file 44321_2025_315_MOESM3_ESM.zip › Figure 2/F2E-cell cycle/3-pIgA+si-NC-1(1).tiff]

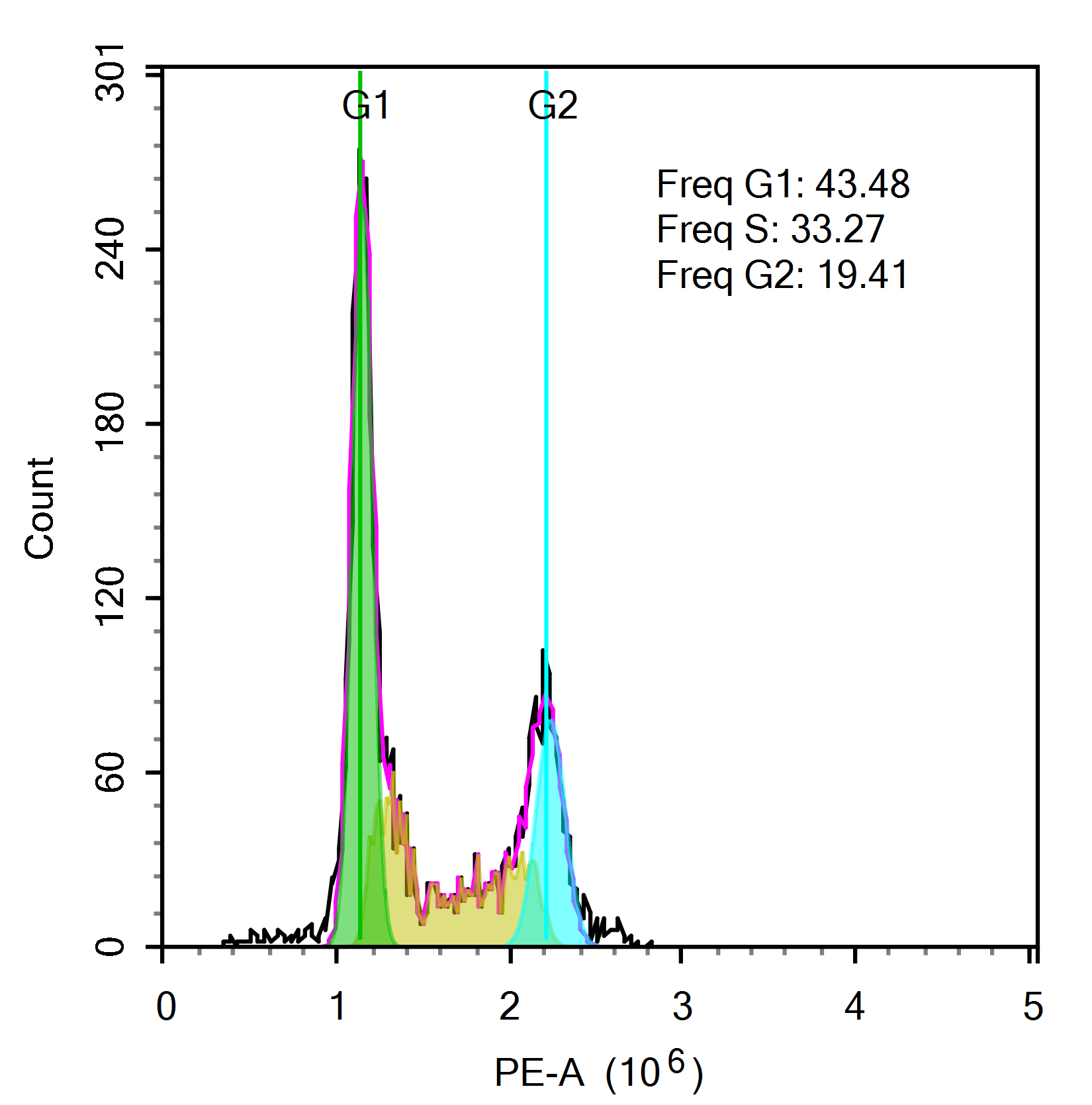

Supplement: Supplementary file 3 — Source data Fig. 2 [file 44321_2025_315_MOESM3_ESM.zip › Figure 2/F2E-cell cycle/3-pIgA+si-NC-1(2).tiff]

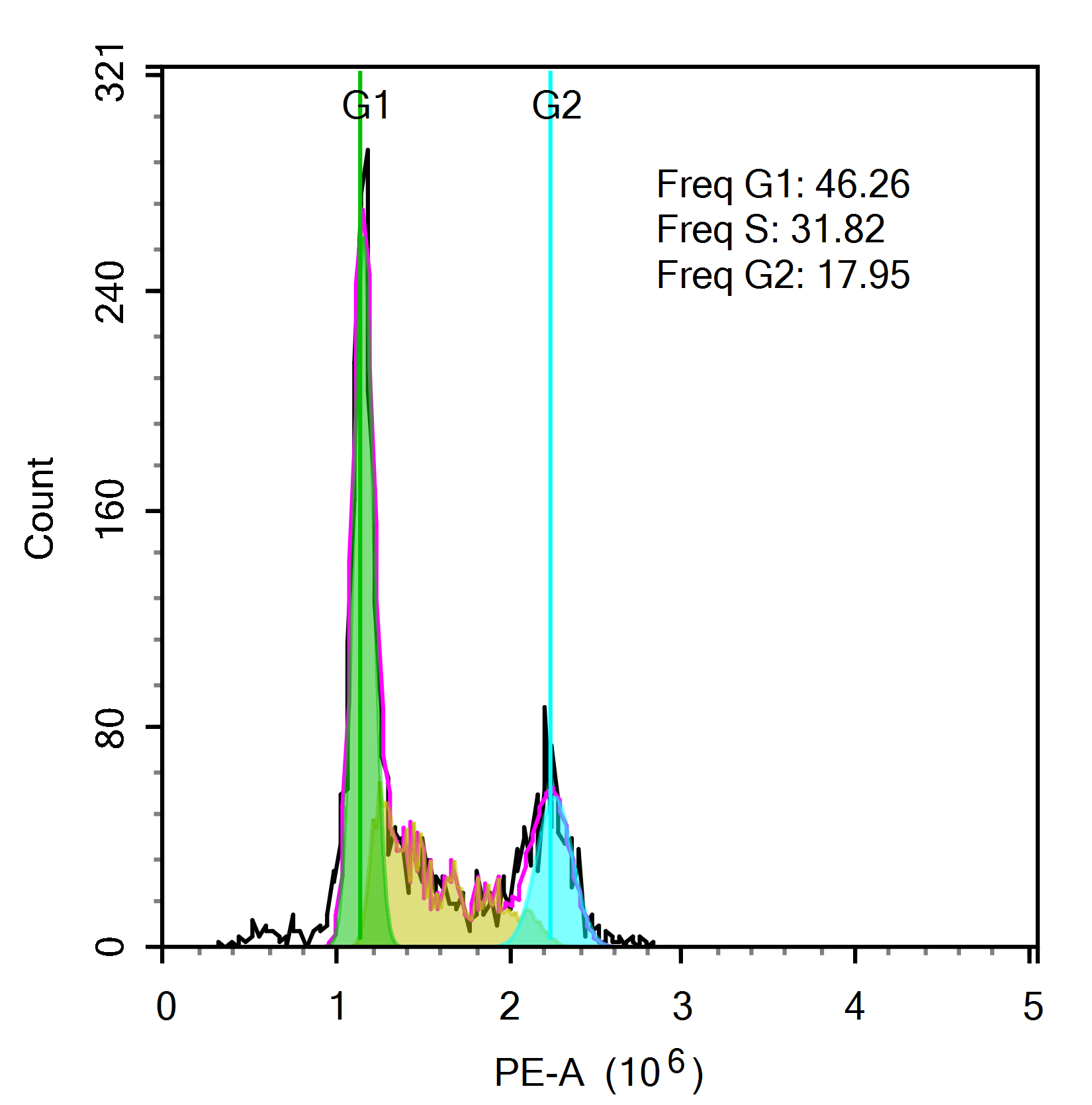

Supplement: Supplementary file 3 — Source data Fig. 2 [file 44321_2025_315_MOESM3_ESM.zip › Figure 2/F2E-cell cycle/3-pIgA+si-NC-1(3).tiff]

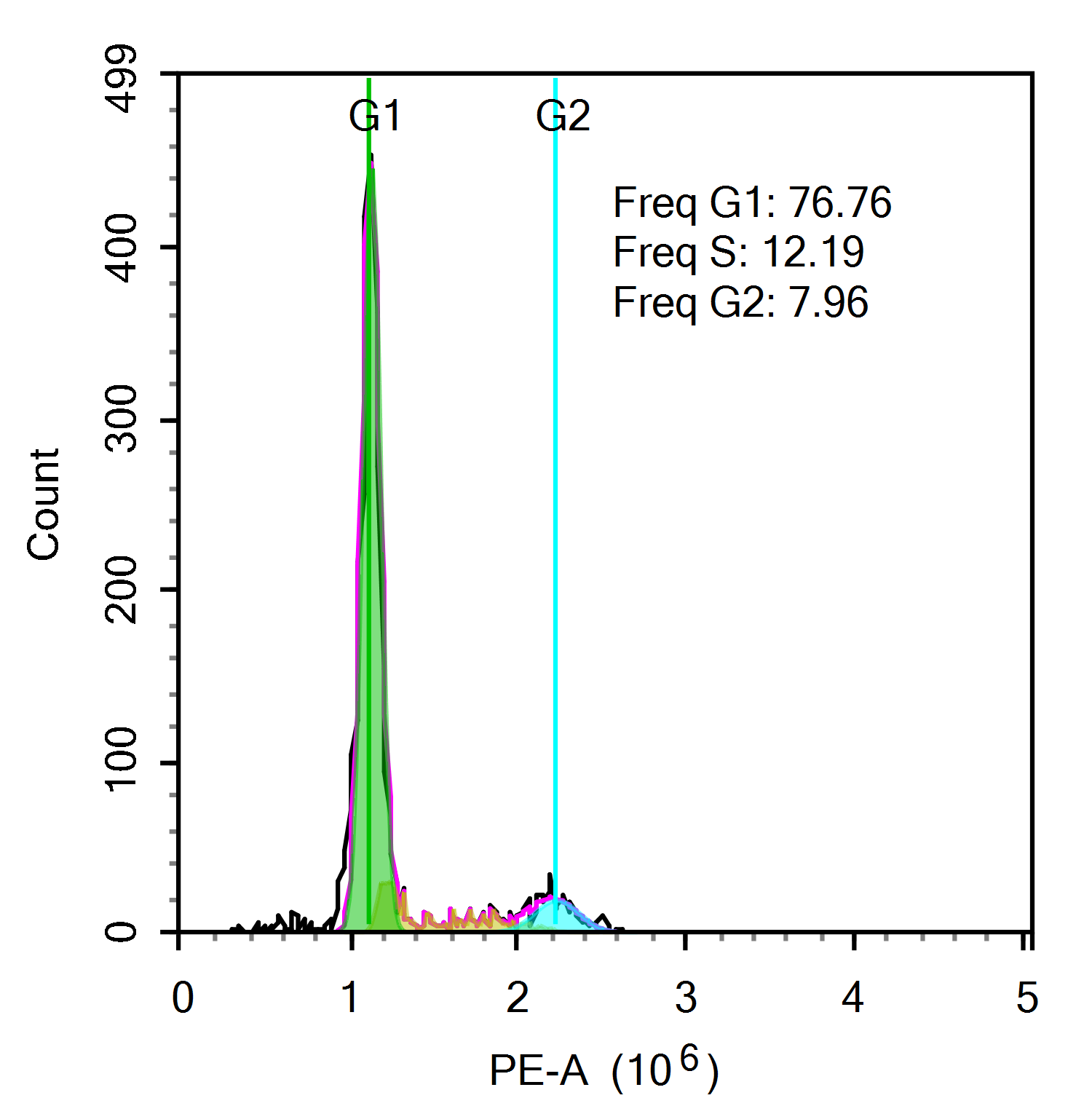

Supplement: Supplementary file 3 — Source data Fig. 2 [file 44321_2025_315_MOESM3_ESM.zip › Figure 2/F2E-cell cycle/4-pIgA+si-GLDC(1).tiff]

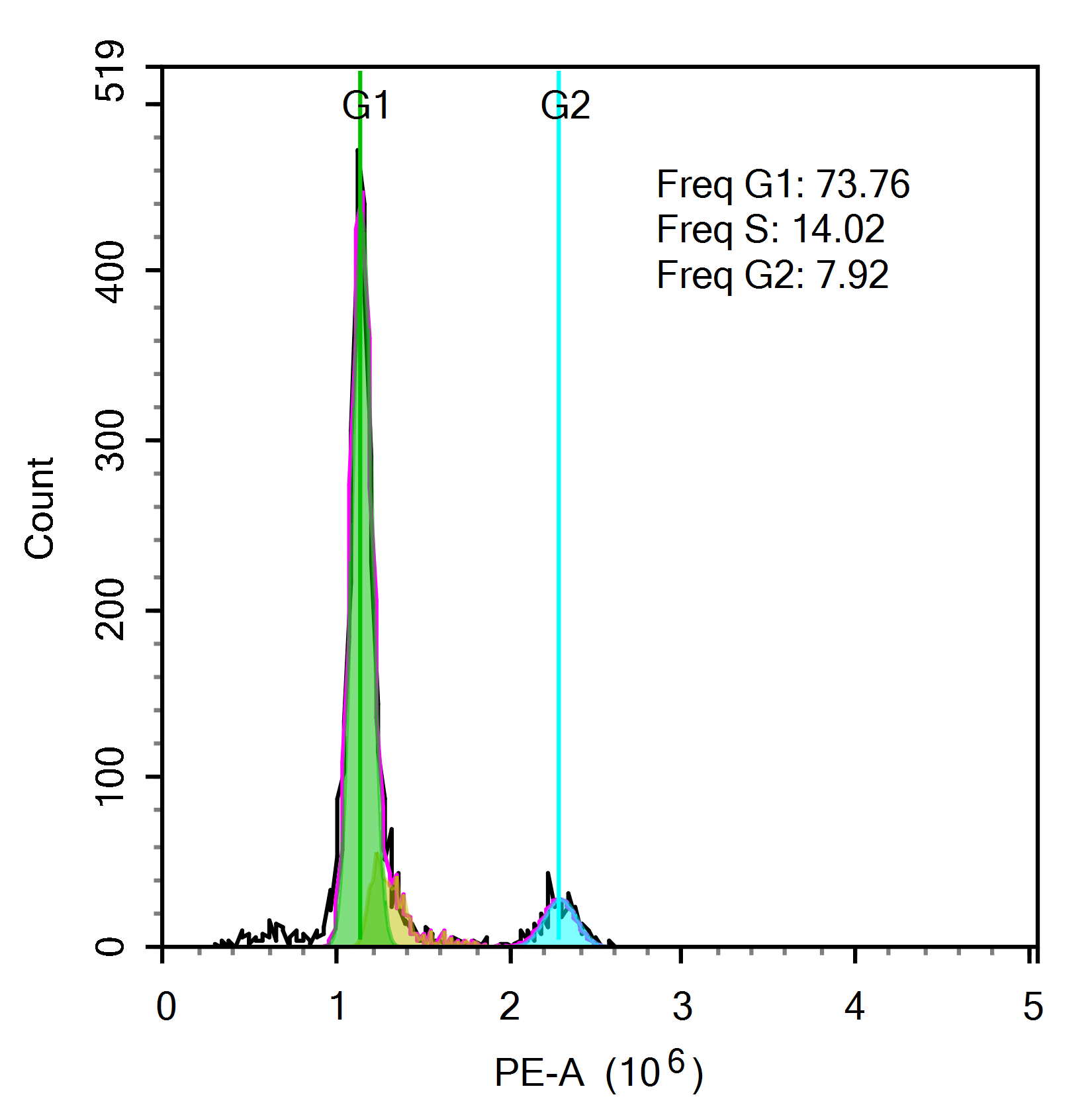

Supplement: Supplementary file 3 — Source data Fig. 2 [file 44321_2025_315_MOESM3_ESM.zip › Figure 2/F2E-cell cycle/4-pIgA+si-GLDC(2).tiff]

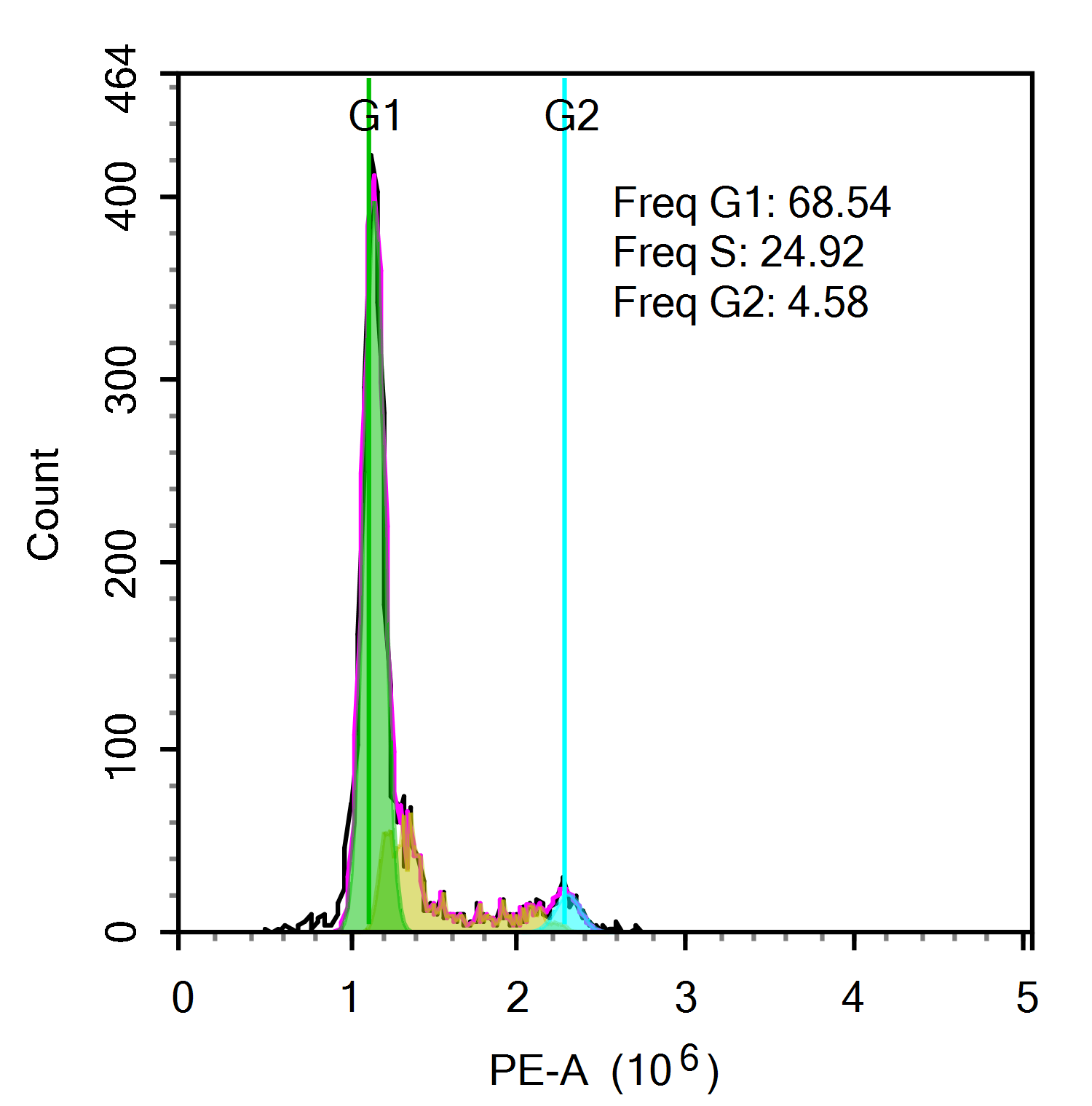

Supplement: Supplementary file 3 — Source data Fig. 2 [file 44321_2025_315_MOESM3_ESM.zip › Figure 2/F2E-cell cycle/4-pIgA+si-GLDC(3).tiff]

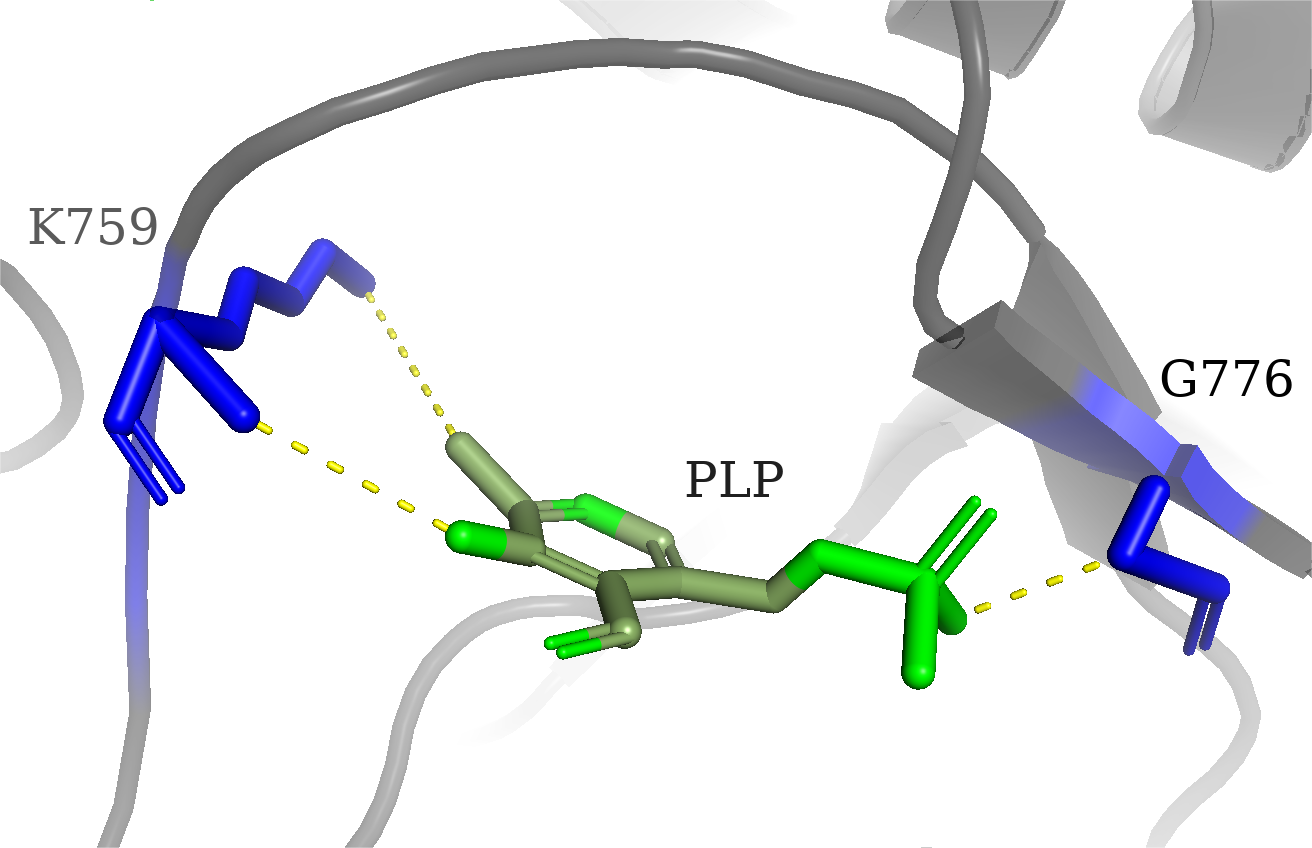

Supplement: Supplementary file 4 — Source data Fig. 3 [file 44321_2025_315_MOESM4_ESM.zip › Figure 3/F3A/Molecular docking.png]

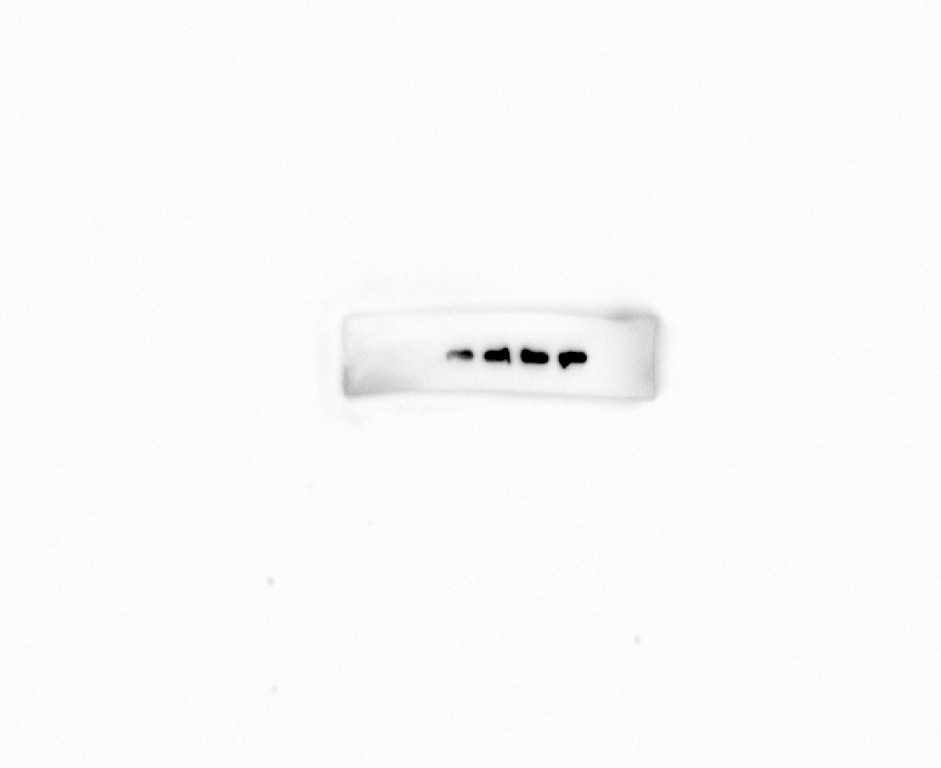

Supplement: Supplementary file 4 — Source data Fig. 3 [file 44321_2025_315_MOESM4_ESM.zip › Figure 3/F3B-WB/1-1-GLDC.jpg]

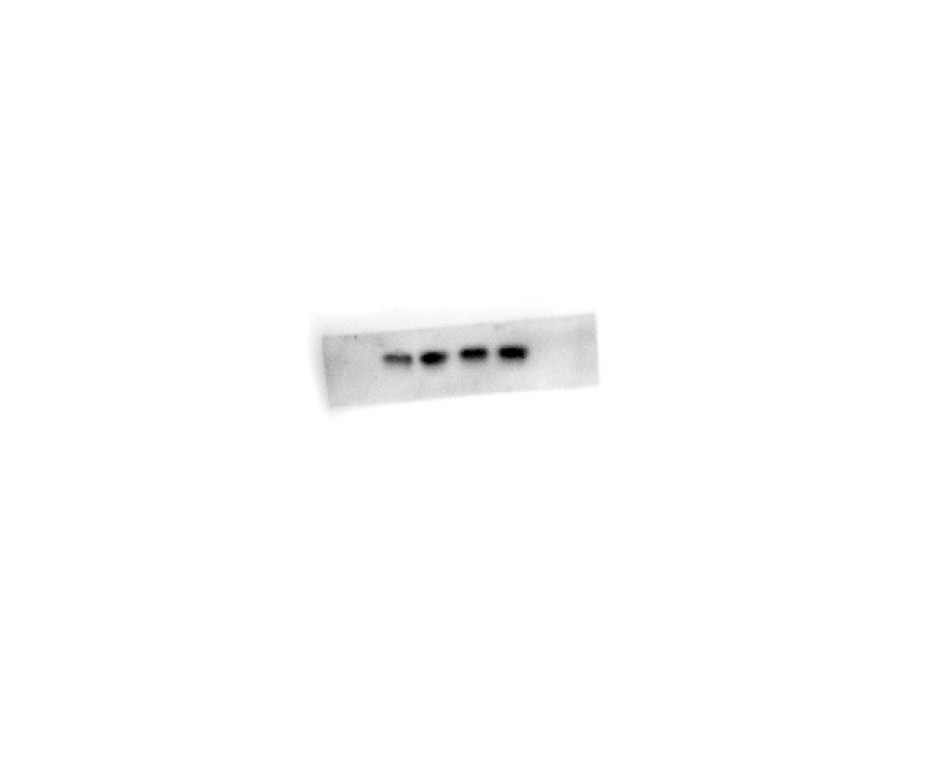

Supplement: Supplementary file 4 — Source data Fig. 3 [file 44321_2025_315_MOESM4_ESM.zip › Figure 3/F3B-WB/1-2-GLDC.jpg]

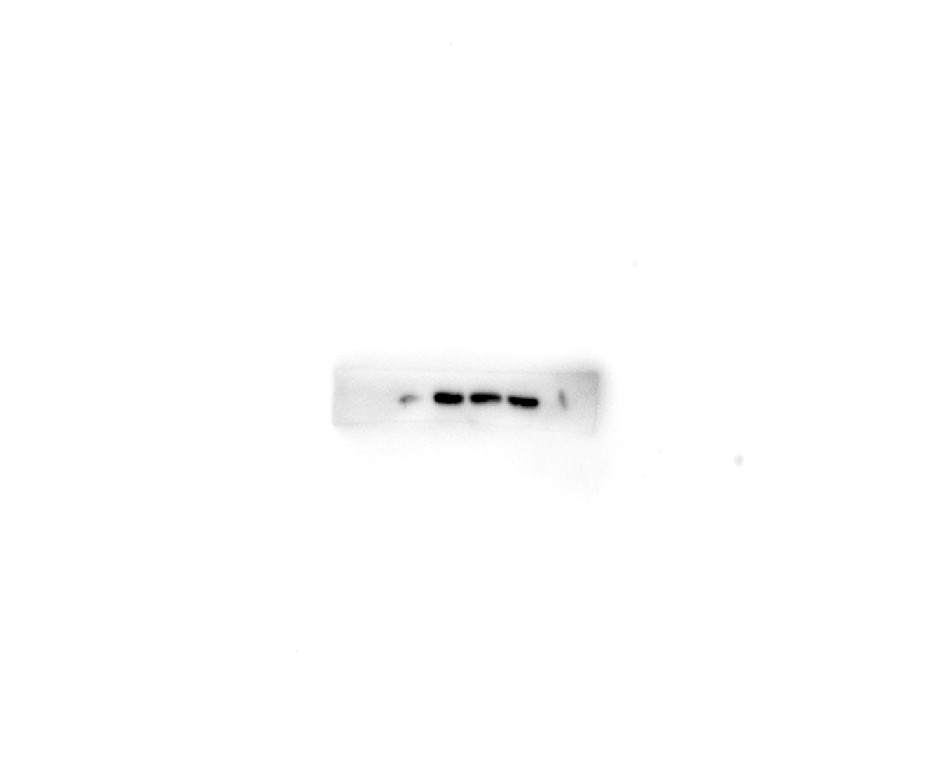

Supplement: Supplementary file 4 — Source data Fig. 3 [file 44321_2025_315_MOESM4_ESM.zip › Figure 3/F3B-WB/1-3-GLDC.jpg]

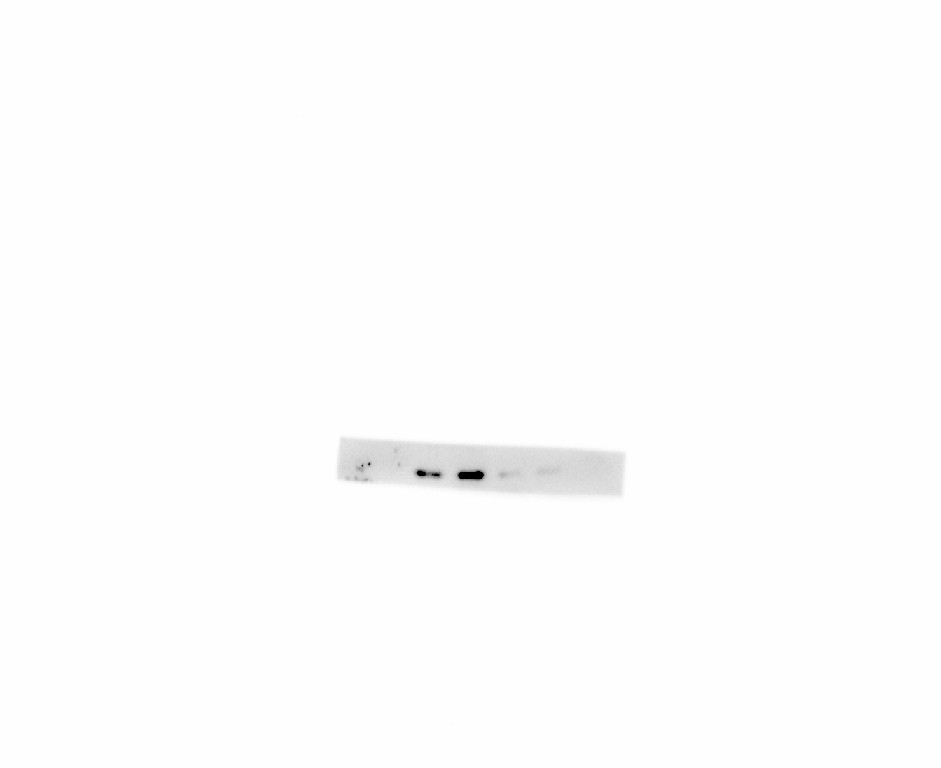

Supplement: Supplementary file 4 — Source data Fig. 3 [file 44321_2025_315_MOESM4_ESM.zip › Figure 3/F3B-WB/2-1-pcna.jpg]

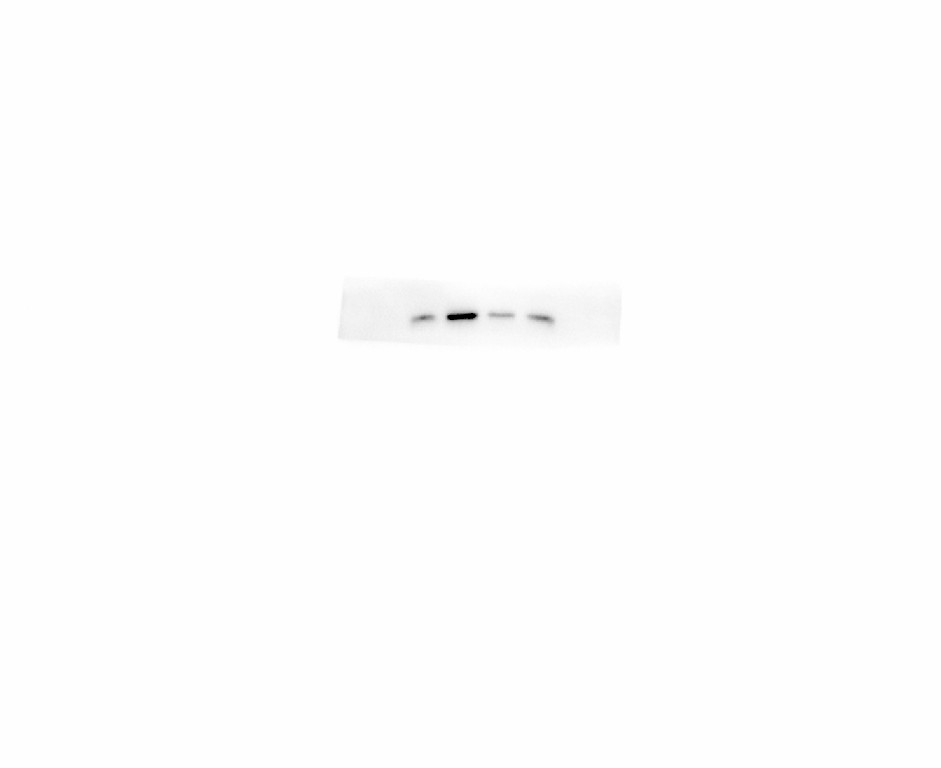

Supplement: Supplementary file 4 — Source data Fig. 3 [file 44321_2025_315_MOESM4_ESM.zip › Figure 3/F3B-WB/2-2-PCNA.jpg]

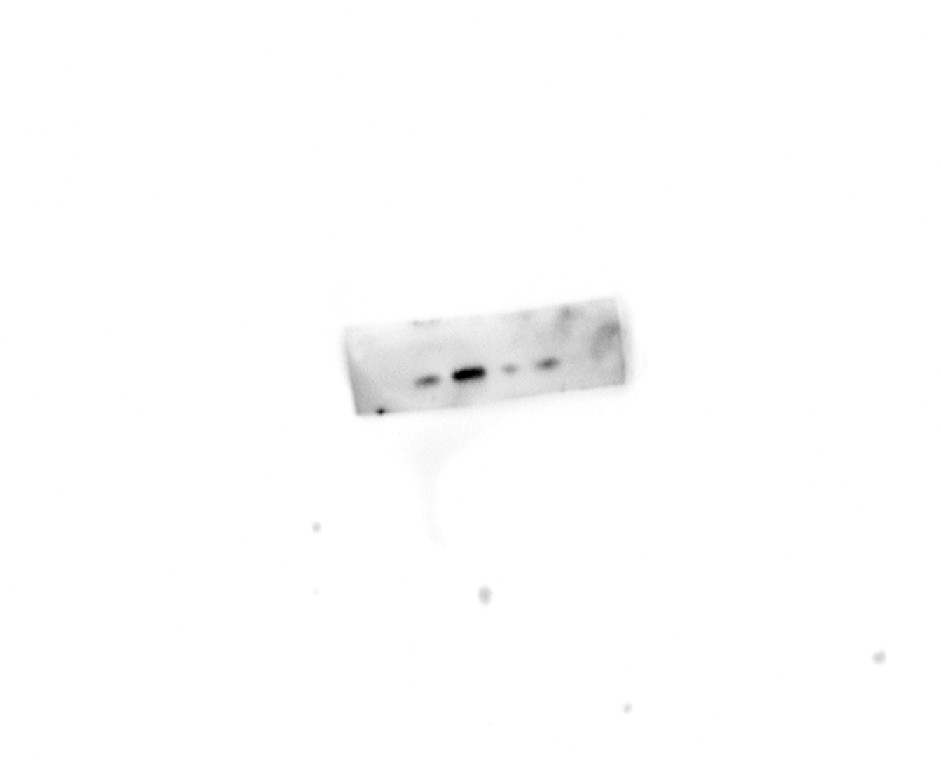

Supplement: Supplementary file 4 — Source data Fig. 3 [file 44321_2025_315_MOESM4_ESM.zip › Figure 3/F3B-WB/2-3-PCNA.jpg]

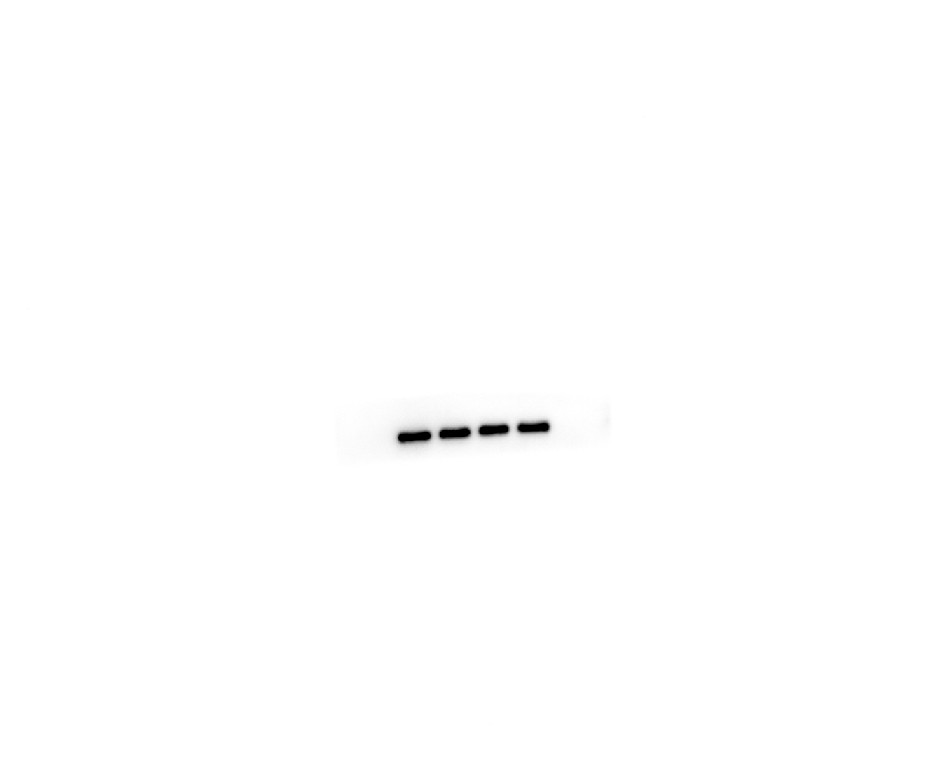

Supplement: Supplementary file 4 — Source data Fig. 3 [file 44321_2025_315_MOESM4_ESM.zip › Figure 3/F3B-WB/3-1-beta-actin.jpg]

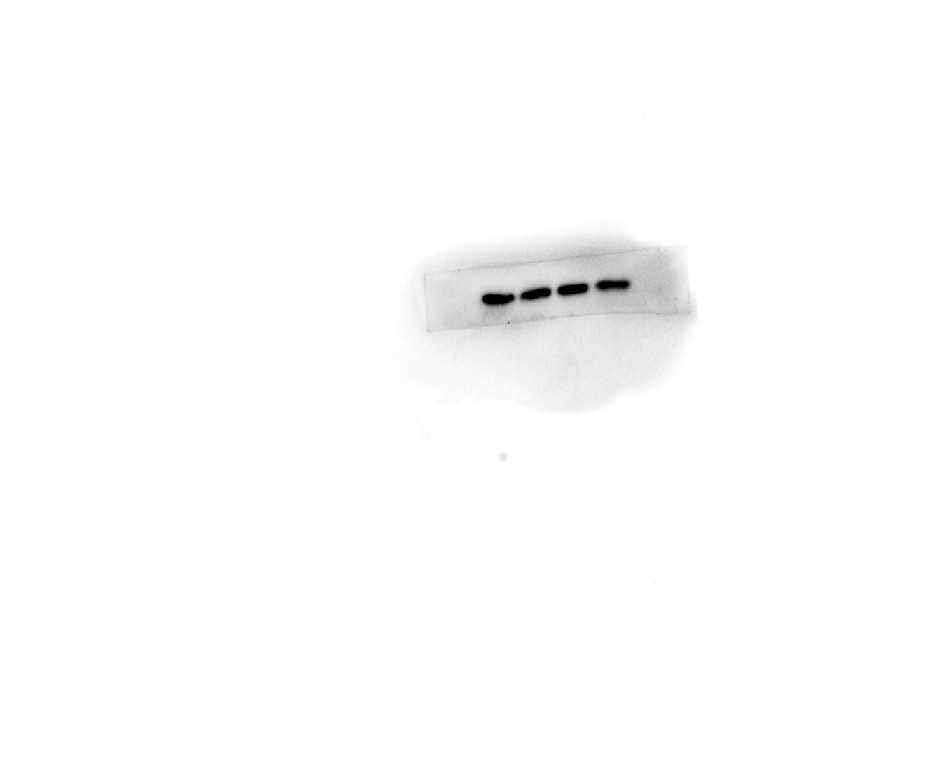

Supplement: Supplementary file 4 — Source data Fig. 3 [file 44321_2025_315_MOESM4_ESM.zip › Figure 3/F3B-WB/3-2-beta-actin.jpg]

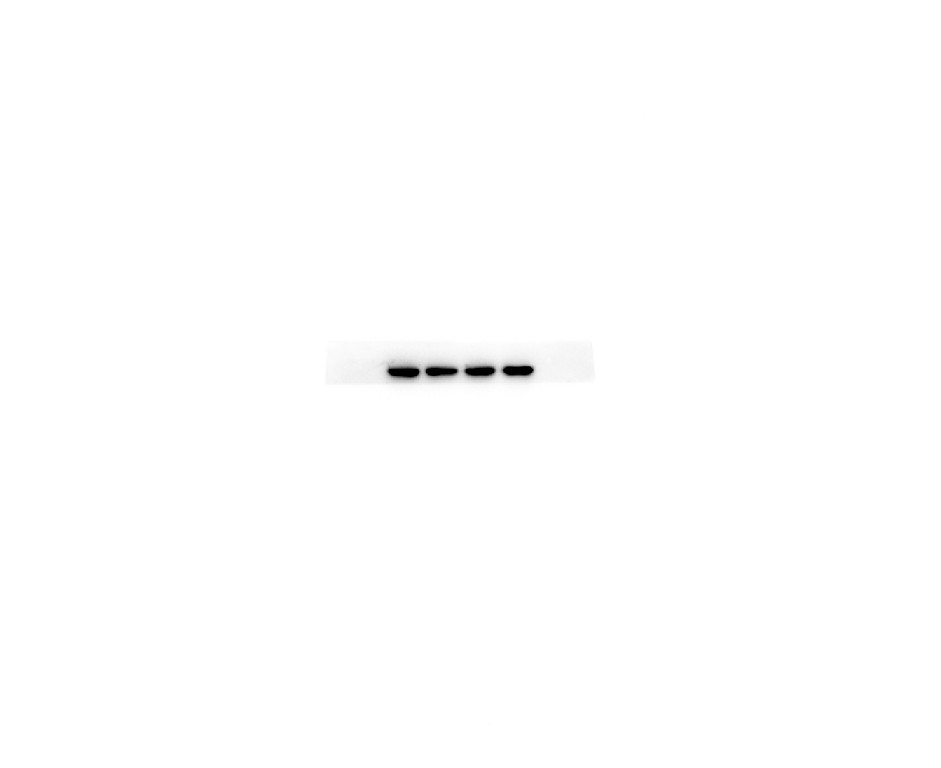

Supplement: Supplementary file 4 — Source data Fig. 3 [file 44321_2025_315_MOESM4_ESM.zip › Figure 3/F3B-WB/3-3-beta-actin.jpg]

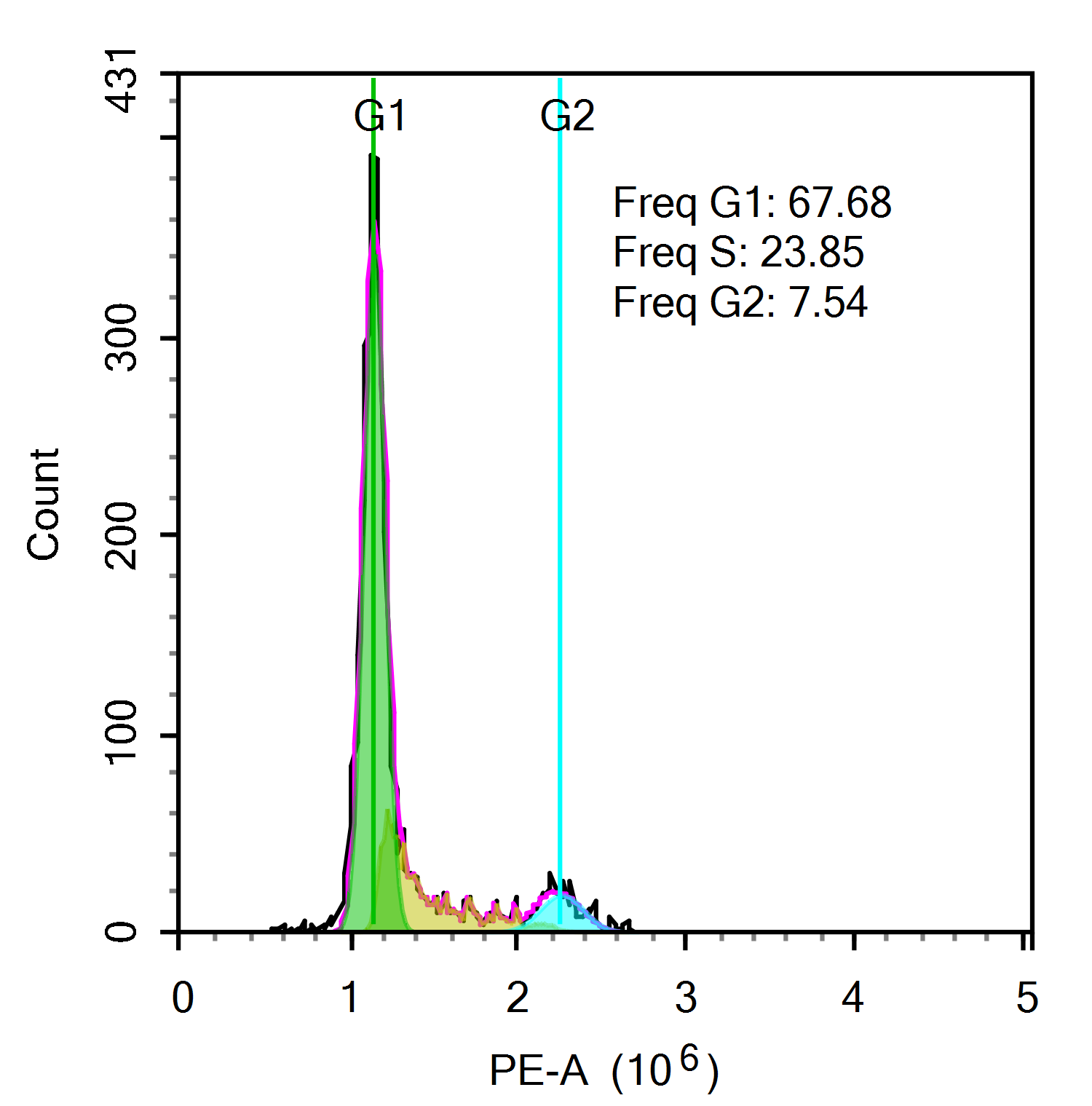

Supplement: Supplementary file 4 — Source data Fig. 3 [file 44321_2025_315_MOESM4_ESM.zip › Figure 3/F3C-cell cycle/1-1.tiff]

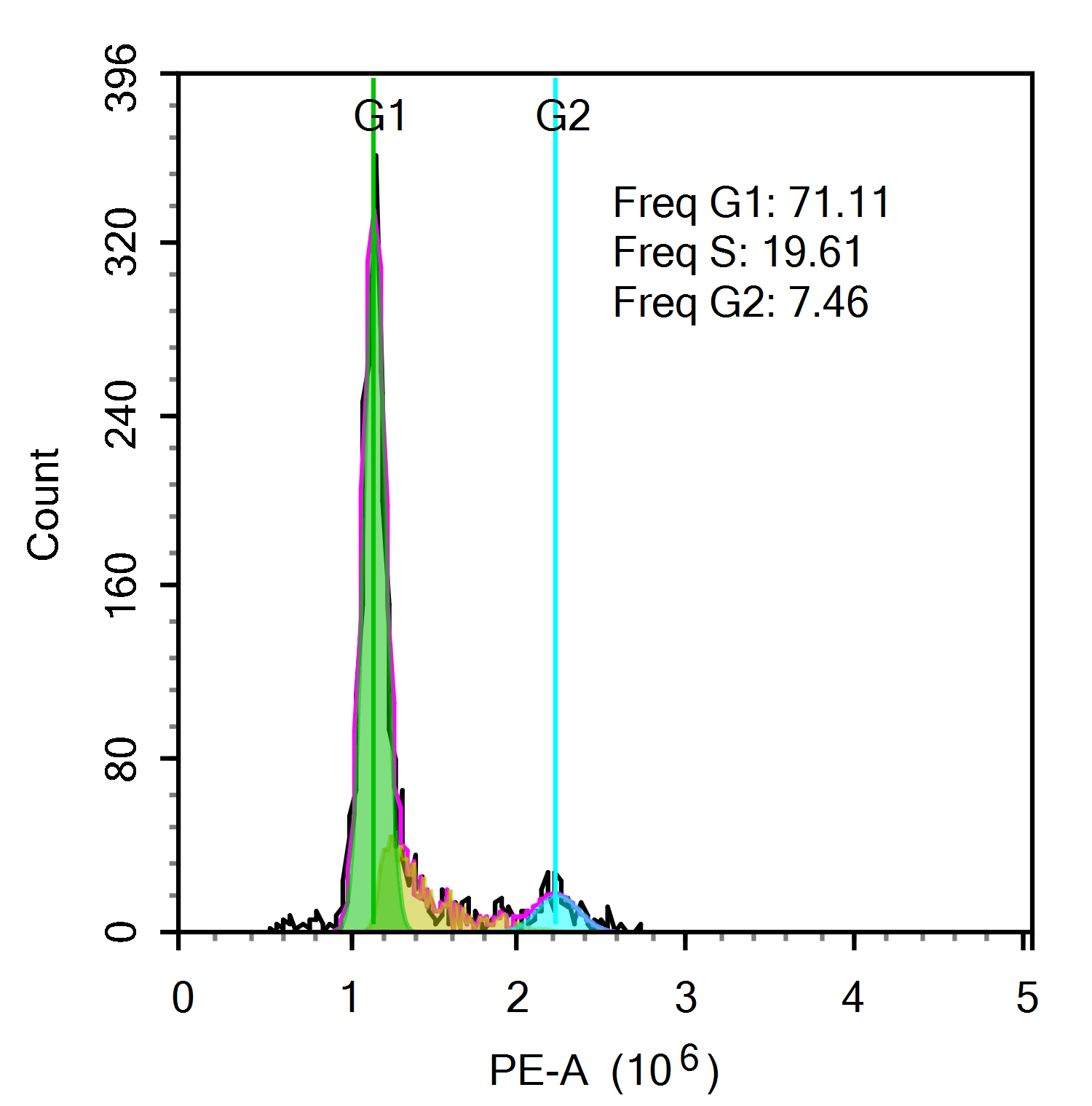

Supplement: Supplementary file 4 — Source data Fig. 3 [file 44321_2025_315_MOESM4_ESM.zip › Figure 3/F3C-cell cycle/1-2.tiff]

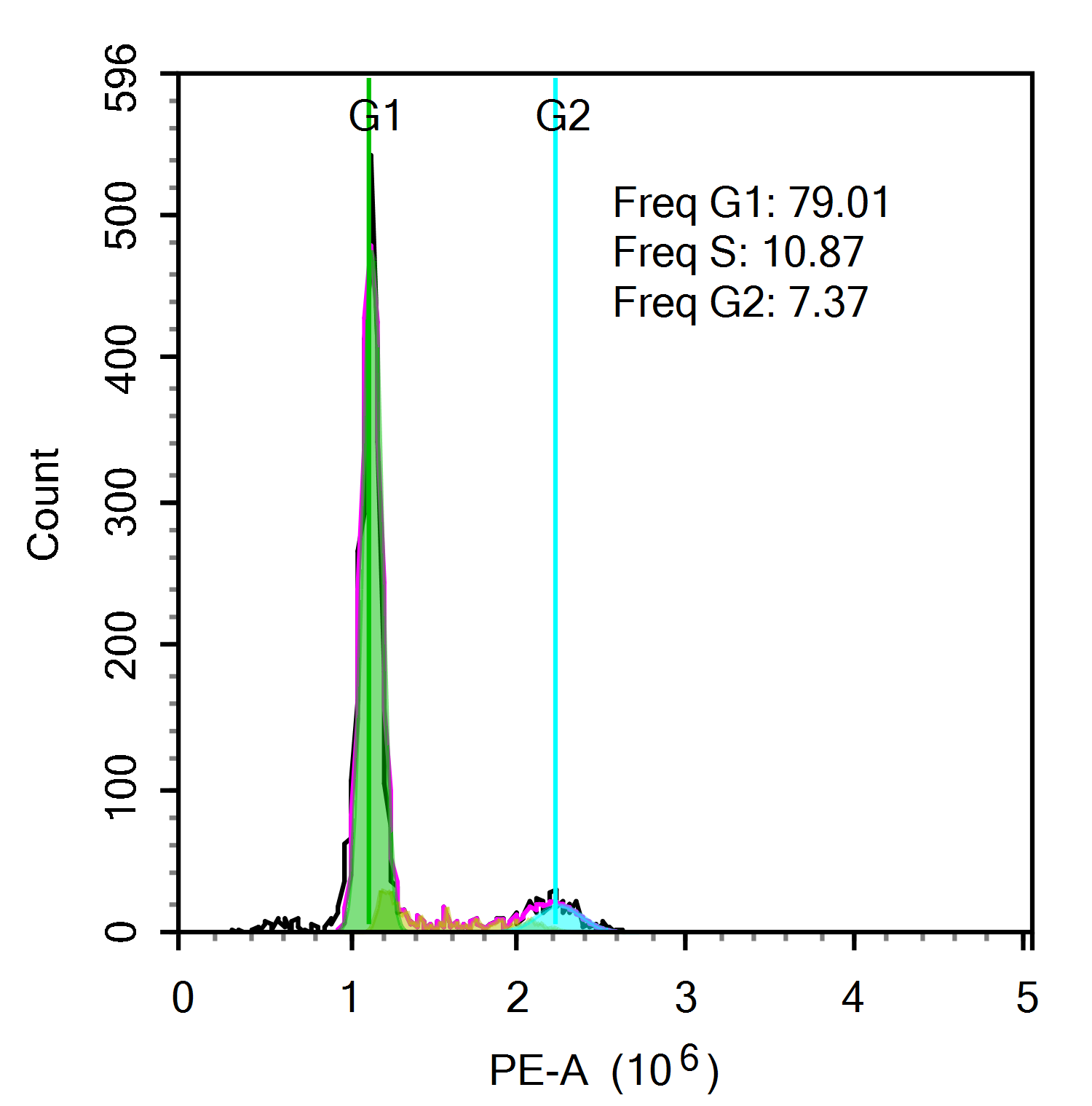

Supplement: Supplementary file 4 — Source data Fig. 3 [file 44321_2025_315_MOESM4_ESM.zip › Figure 3/F3C-cell cycle/1-3.tiff]

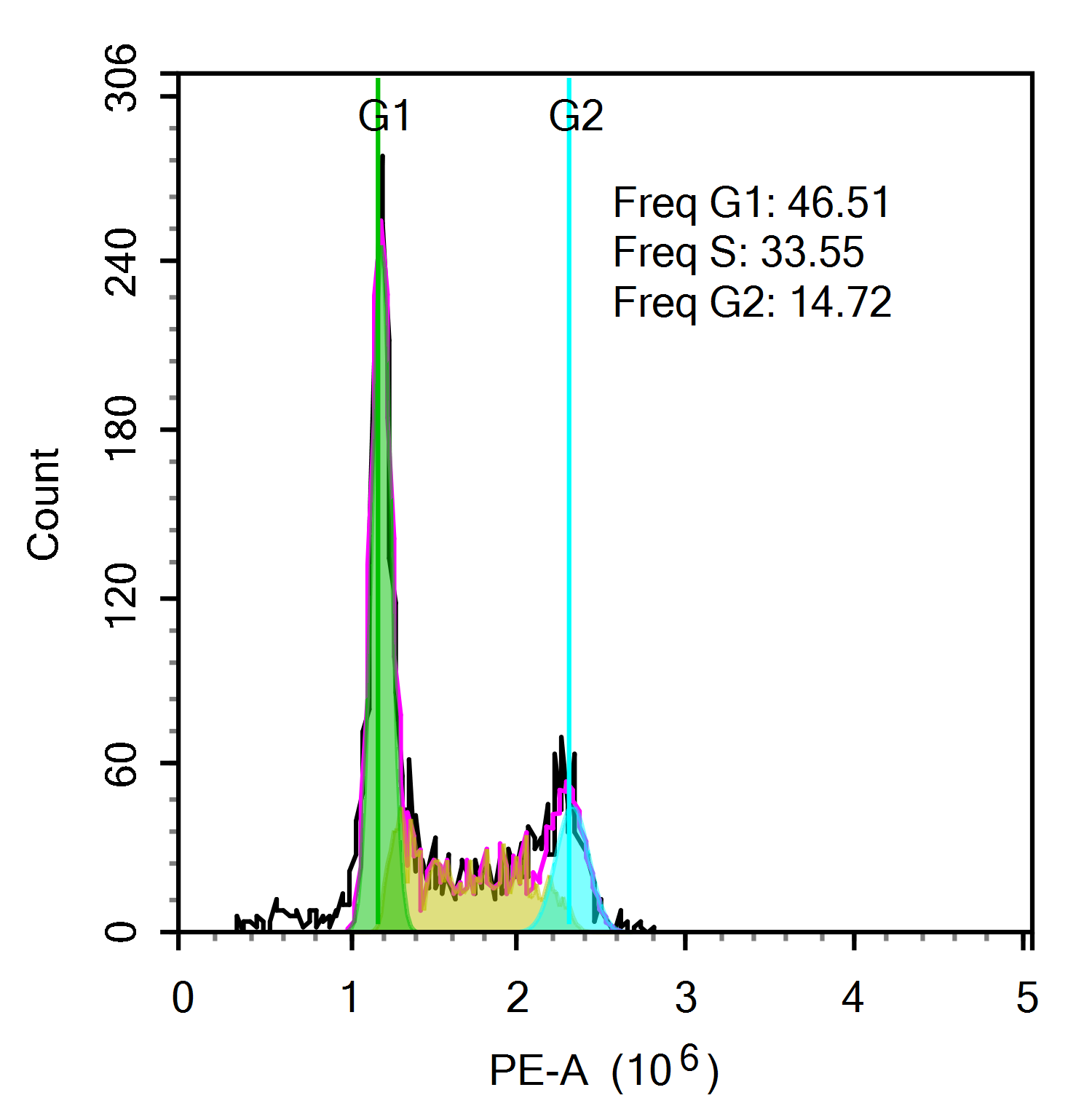

Supplement: Supplementary file 4 — Source data Fig. 3 [file 44321_2025_315_MOESM4_ESM.zip › Figure 3/F3C-cell cycle/2-1.tiff]

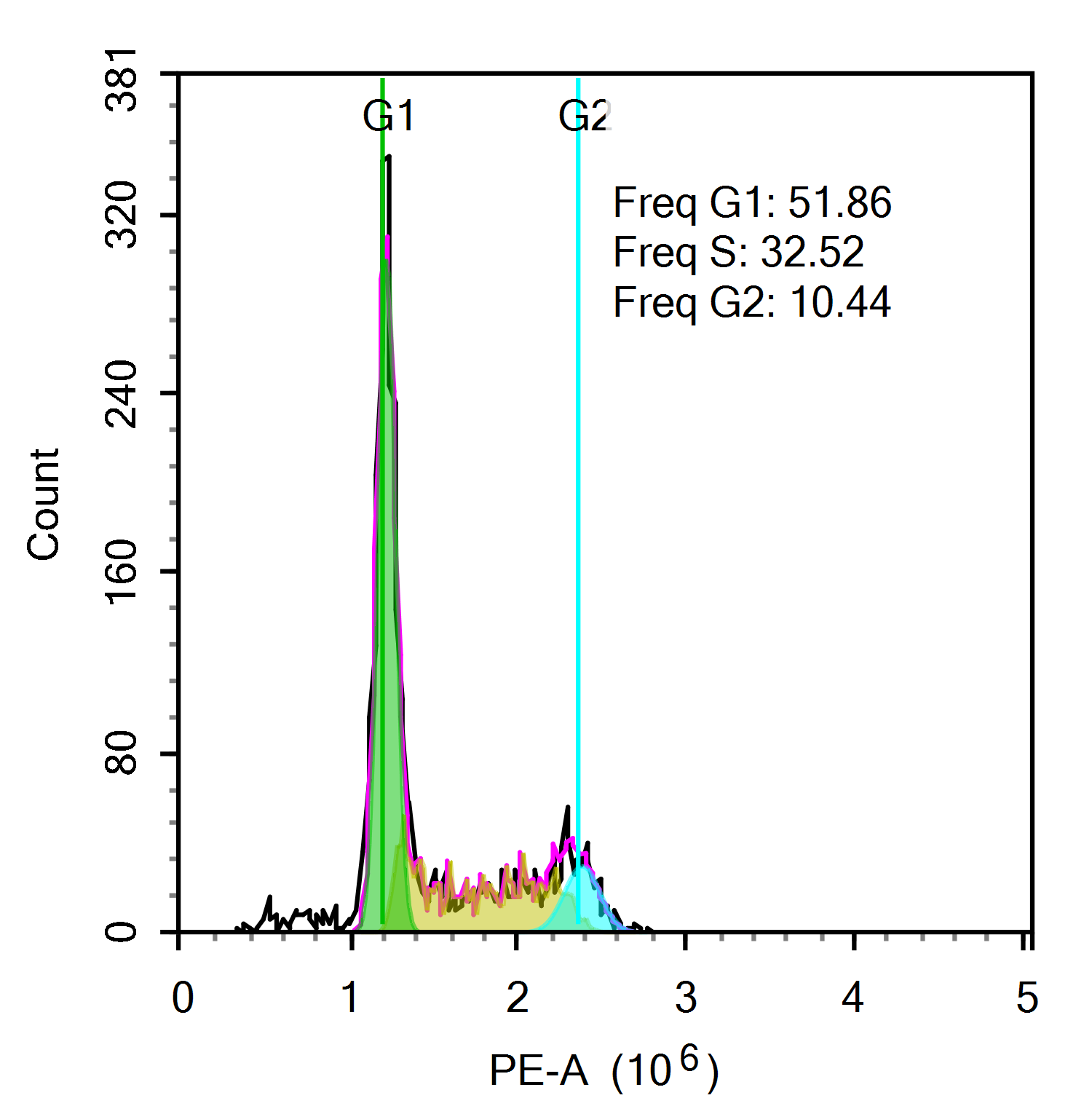

Supplement: Supplementary file 4 — Source data Fig. 3 [file 44321_2025_315_MOESM4_ESM.zip › Figure 3/F3C-cell cycle/2-2.tiff]

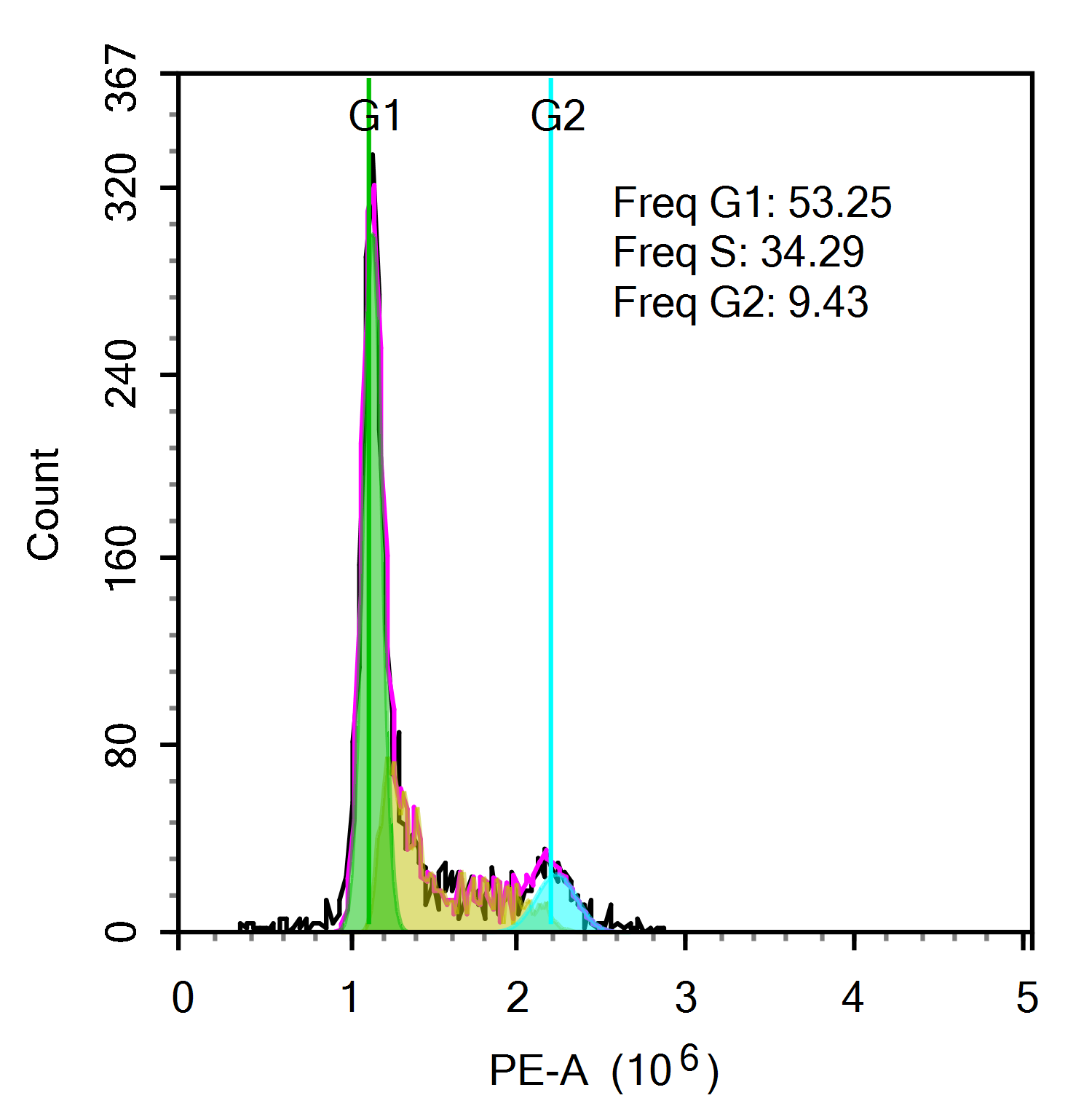

Supplement: Supplementary file 4 — Source data Fig. 3 [file 44321_2025_315_MOESM4_ESM.zip › Figure 3/F3C-cell cycle/2-3.tiff]

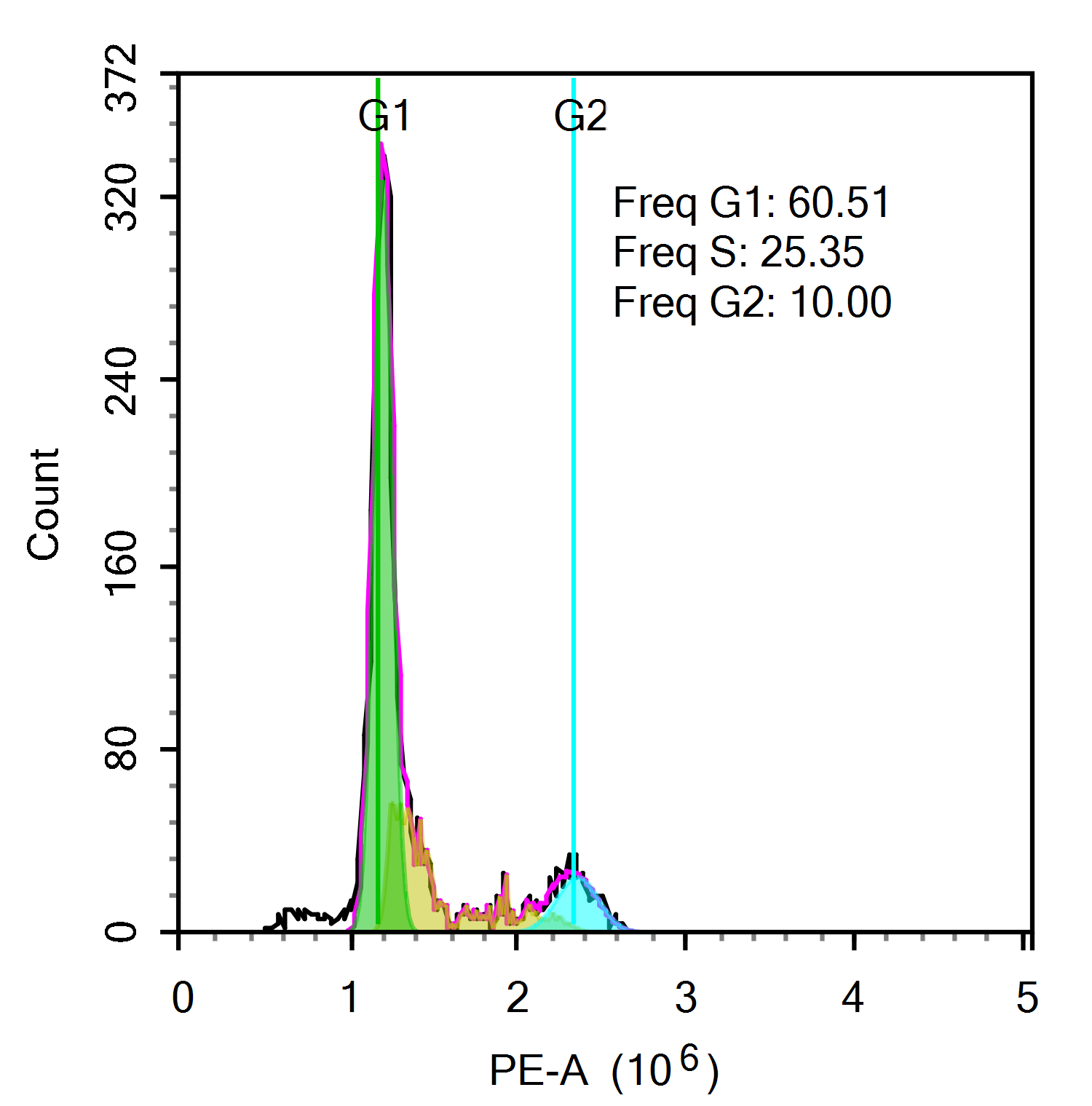

Supplement: Supplementary file 4 — Source data Fig. 3 [file 44321_2025_315_MOESM4_ESM.zip › Figure 3/F3C-cell cycle/3-1.tiff]

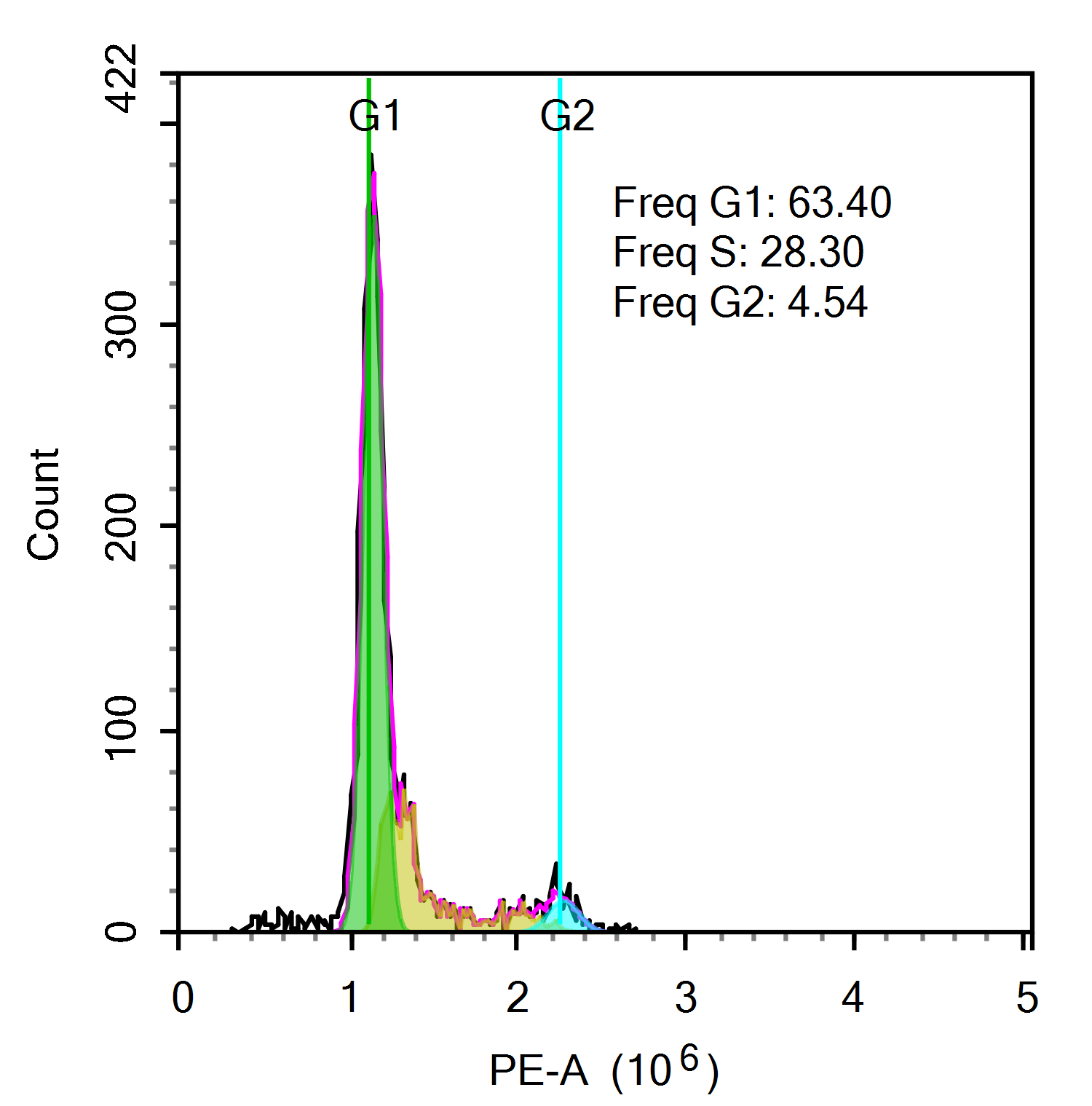

Supplement: Supplementary file 4 — Source data Fig. 3 [file 44321_2025_315_MOESM4_ESM.zip › Figure 3/F3C-cell cycle/3-2.tiff]

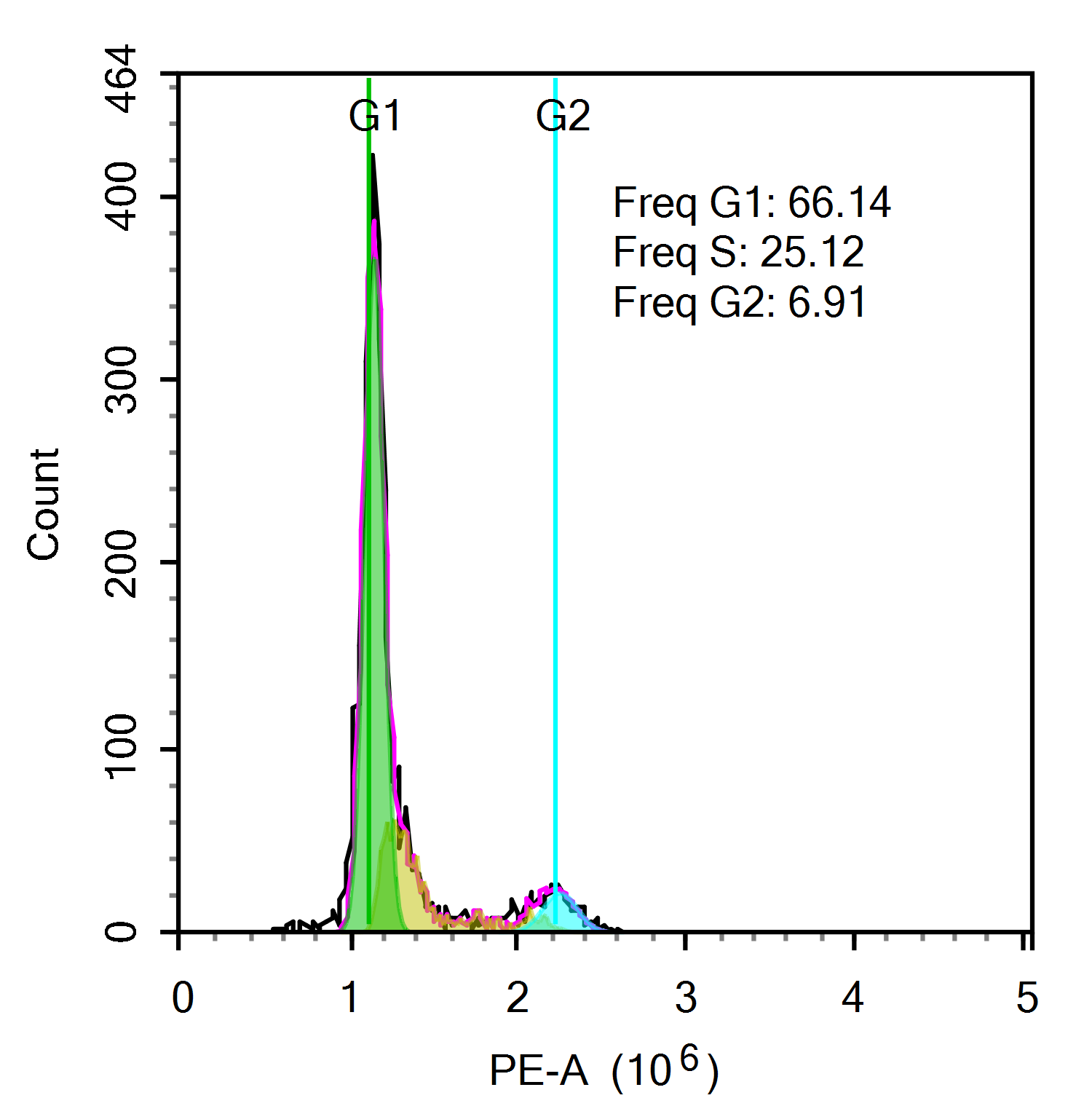

Supplement: Supplementary file 4 — Source data Fig. 3 [file 44321_2025_315_MOESM4_ESM.zip › Figure 3/F3C-cell cycle/3-3.tiff]

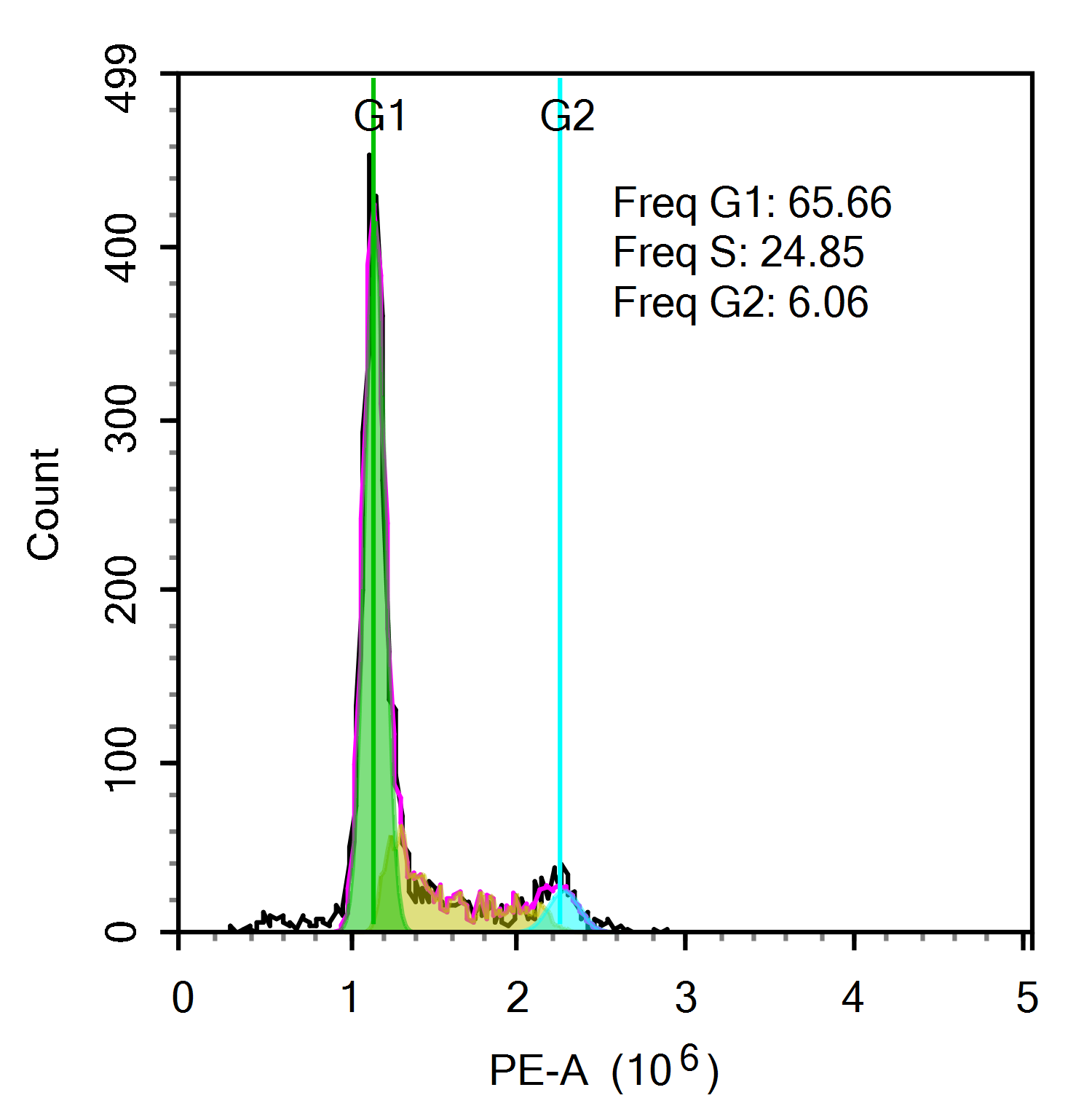

Supplement: Supplementary file 4 — Source data Fig. 3 [file 44321_2025_315_MOESM4_ESM.zip › Figure 3/F3C-cell cycle/4-1.tiff]

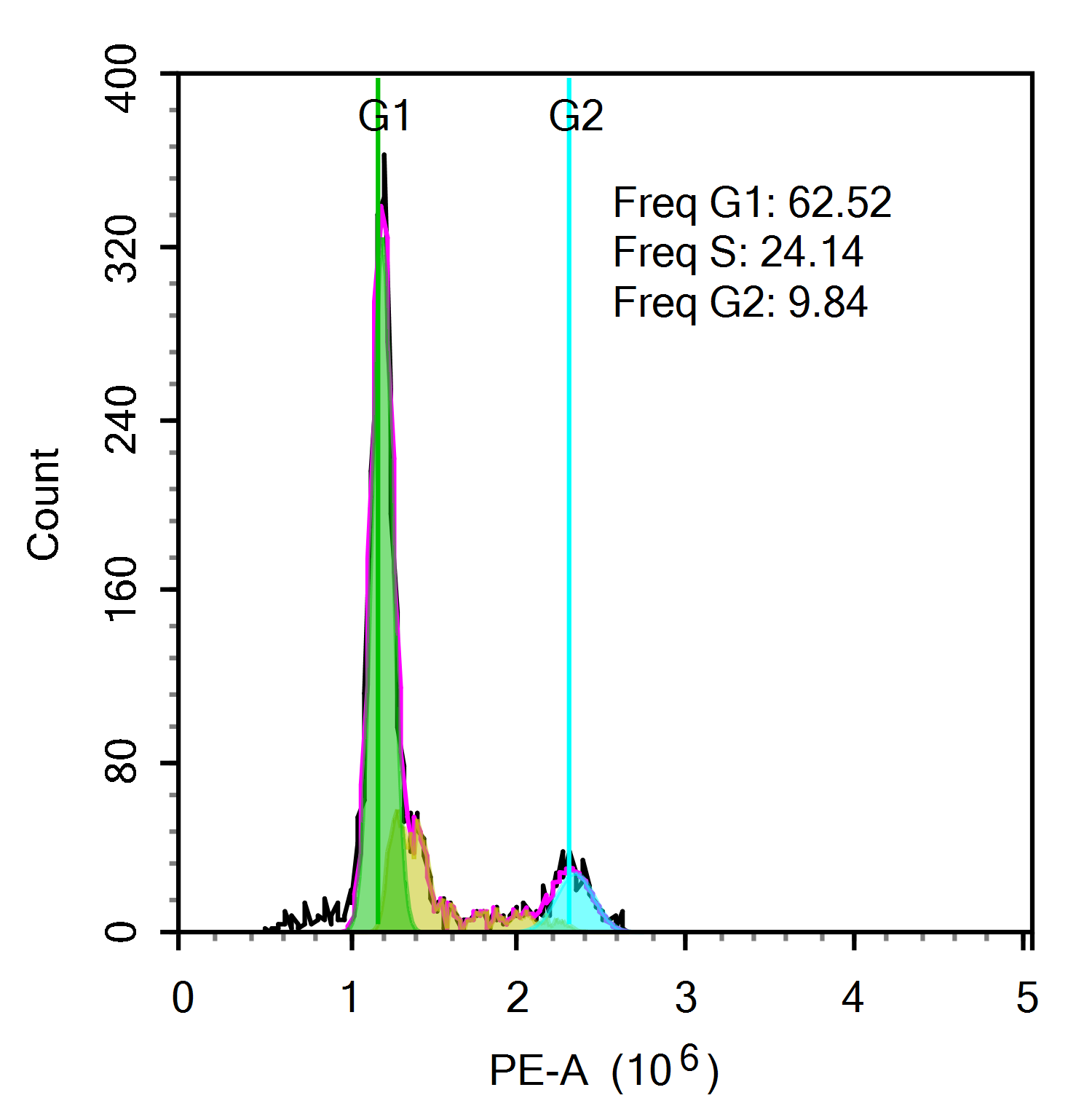

Supplement: Supplementary file 4 — Source data Fig. 3 [file 44321_2025_315_MOESM4_ESM.zip › Figure 3/F3C-cell cycle/4-2.tiff]

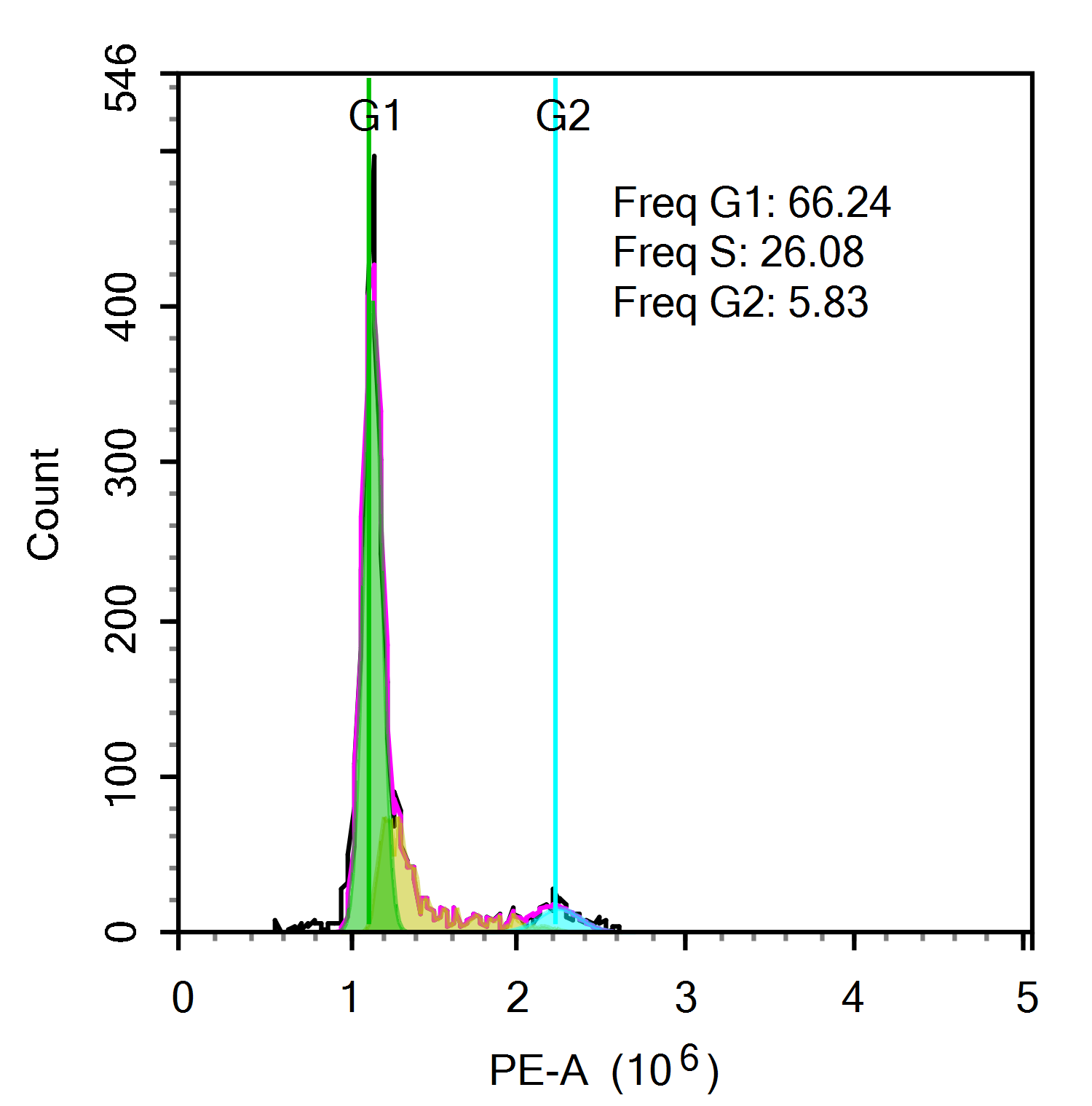

Supplement: Supplementary file 4 — Source data Fig. 3 [file 44321_2025_315_MOESM4_ESM.zip › Figure 3/F3C-cell cycle/4-3.tiff]

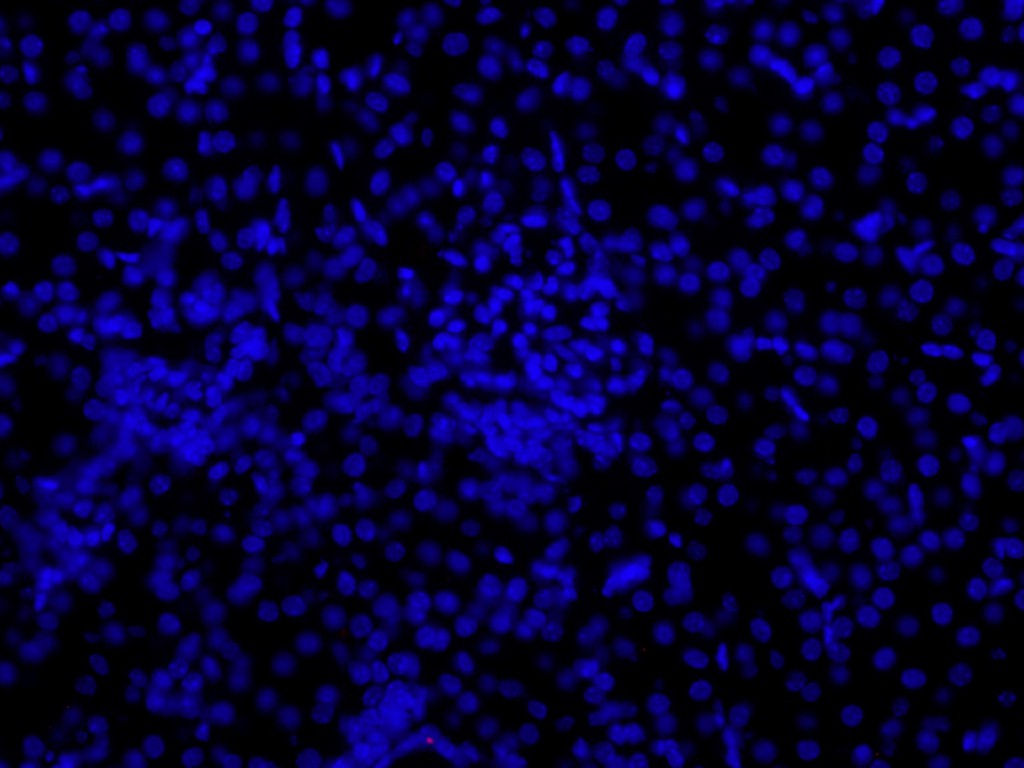

Supplement: Supplementary file 7 — Source data Fig. 6 [file 44321_2025_315_MOESM7_ESM.zip › Figure 6/F6A/1-IgA/1-1 (1).jpg]

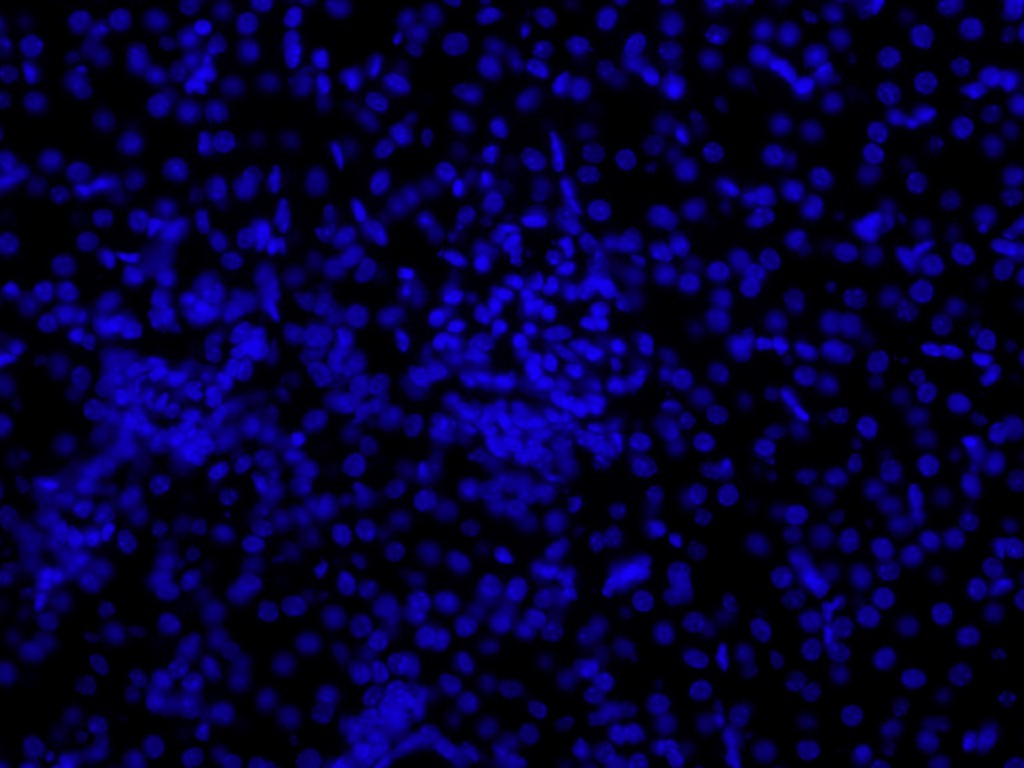

Supplement: Supplementary file 7 — Source data Fig. 6 [file 44321_2025_315_MOESM7_ESM.zip › Figure 6/F6A/1-IgA/1-1 (2).jpg]

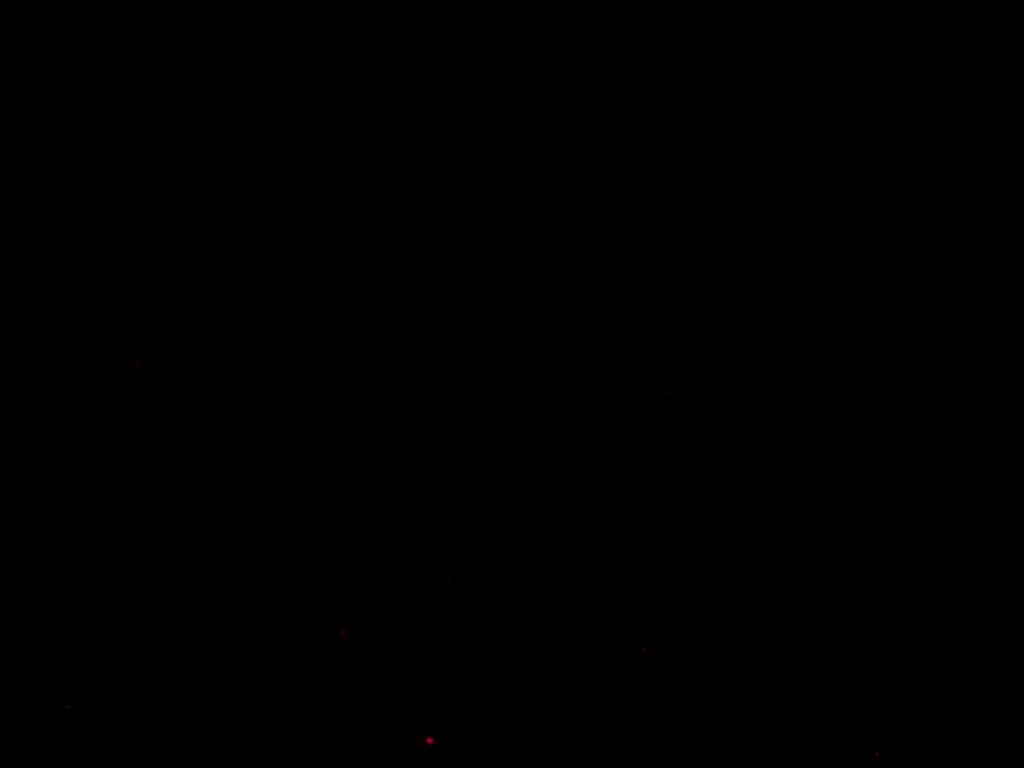

Supplement: Supplementary file 7 — Source data Fig. 6 [file 44321_2025_315_MOESM7_ESM.zip › Figure 6/F6A/1-IgA/1-1 (3).jpg]

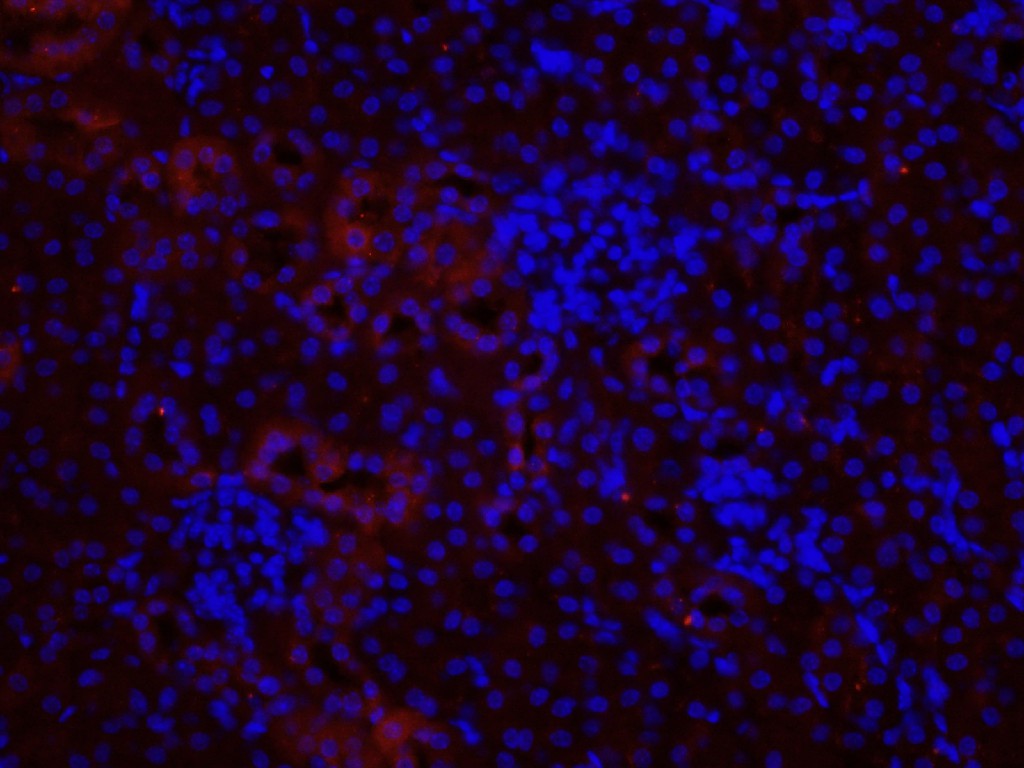

Supplement: Supplementary file 7 — Source data Fig. 6 [file 44321_2025_315_MOESM7_ESM.zip › Figure 6/F6A/1-IgA/1-2 (1).jpg]

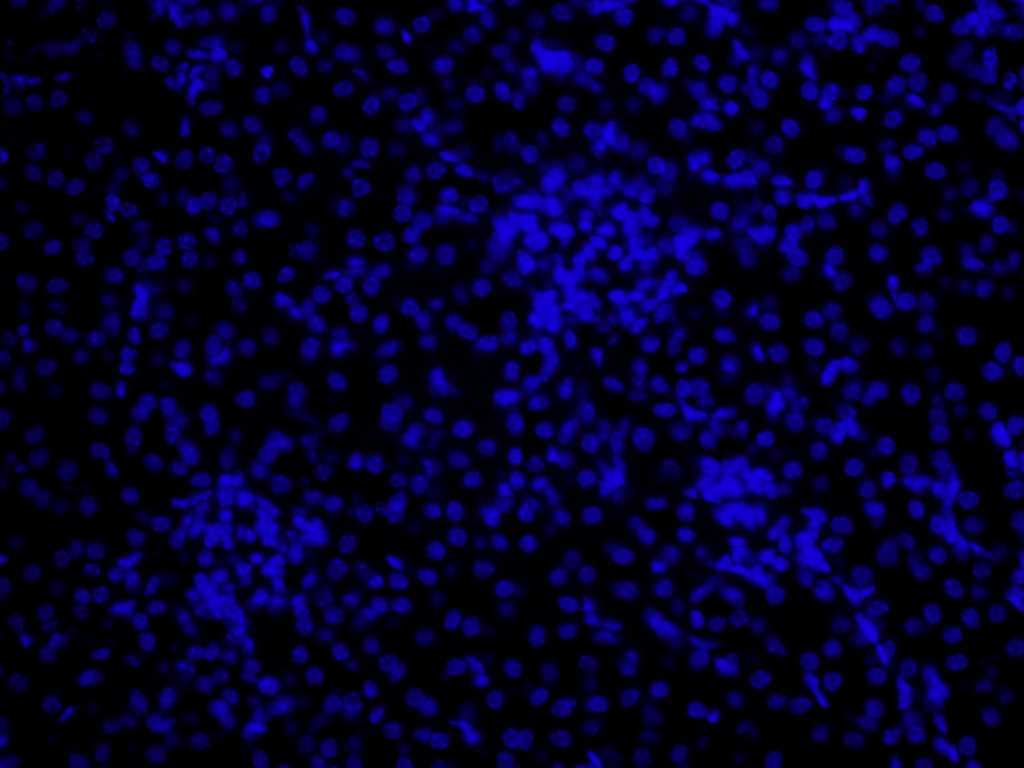

Supplement: Supplementary file 7 — Source data Fig. 6 [file 44321_2025_315_MOESM7_ESM.zip › Figure 6/F6A/1-IgA/1-2 (2).jpg]

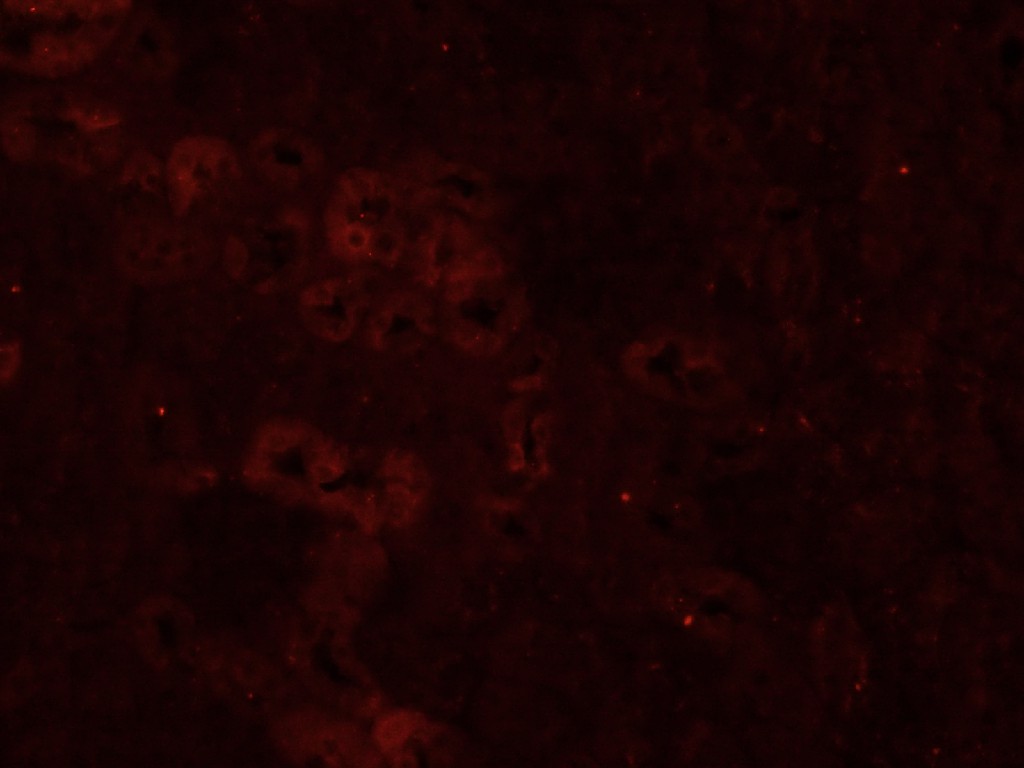

Supplement: Supplementary file 7 — Source data Fig. 6 [file 44321_2025_315_MOESM7_ESM.zip › Figure 6/F6A/1-IgA/1-2 (3).jpg]

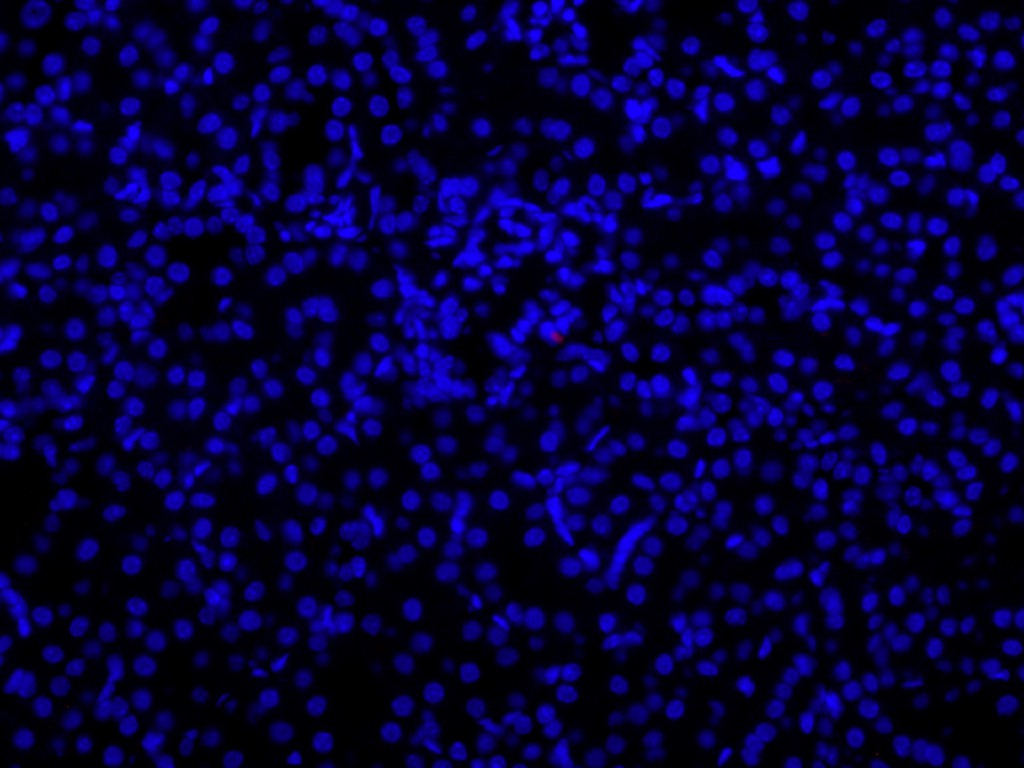

Supplement: Supplementary file 7 — Source data Fig. 6 [file 44321_2025_315_MOESM7_ESM.zip › Figure 6/F6A/1-IgA/1-3 (1).jpg]

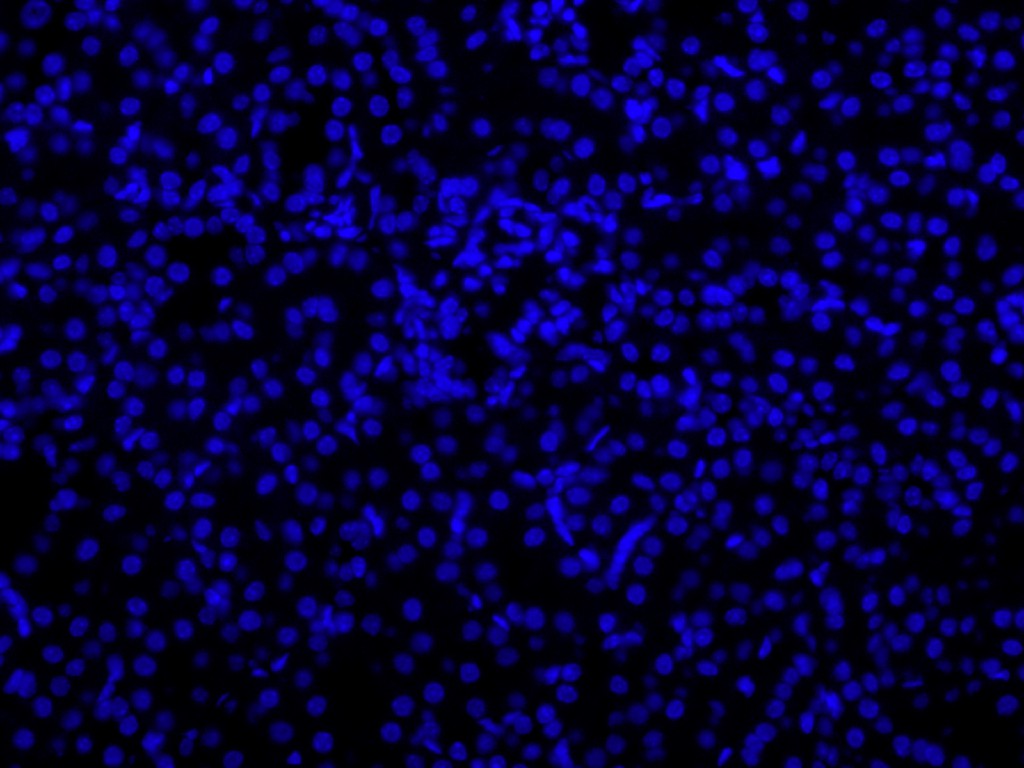

Supplement: Supplementary file 7 — Source data Fig. 6 [file 44321_2025_315_MOESM7_ESM.zip › Figure 6/F6A/1-IgA/1-3 (2).jpg]

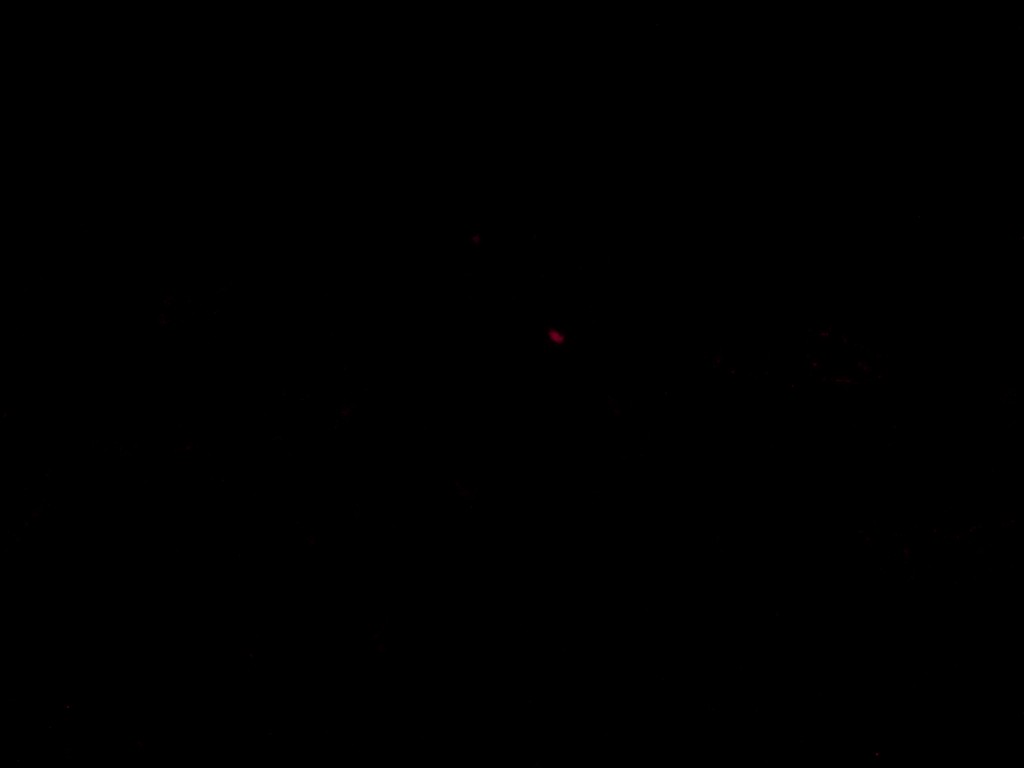

Supplement: Supplementary file 7 — Source data Fig. 6 [file 44321_2025_315_MOESM7_ESM.zip › Figure 6/F6A/1-IgA/1-3 (3).jpg]

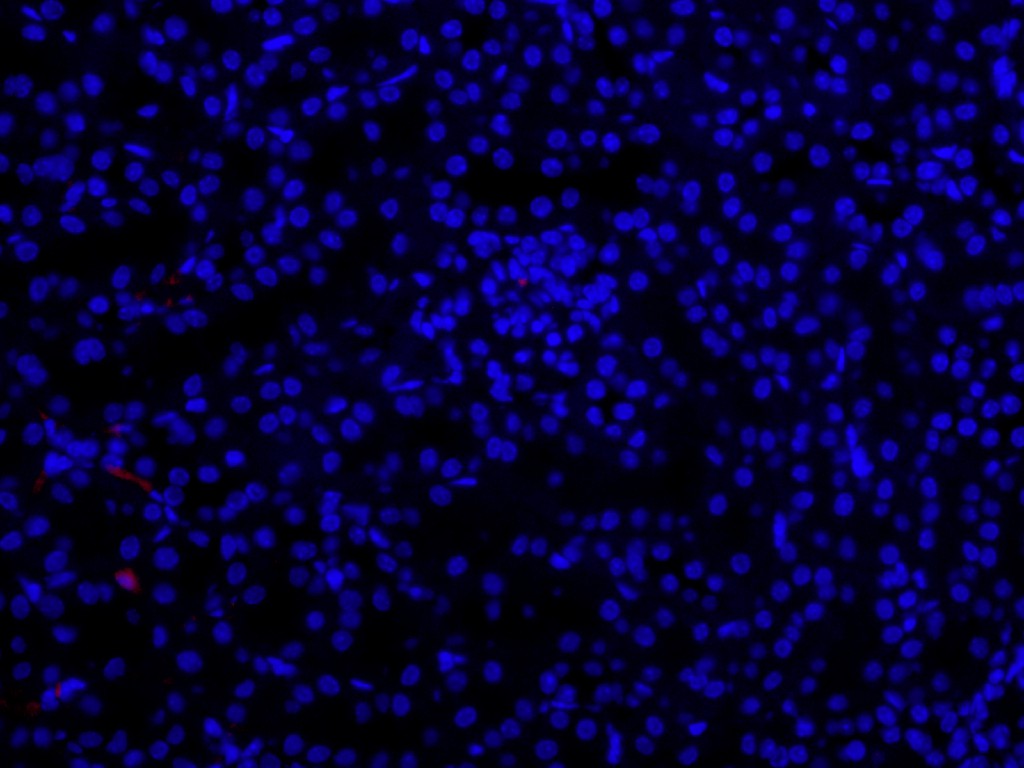

Supplement: Supplementary file 7 — Source data Fig. 6 [file 44321_2025_315_MOESM7_ESM.zip › Figure 6/F6A/1-IgA/1-4 (1).jpg]

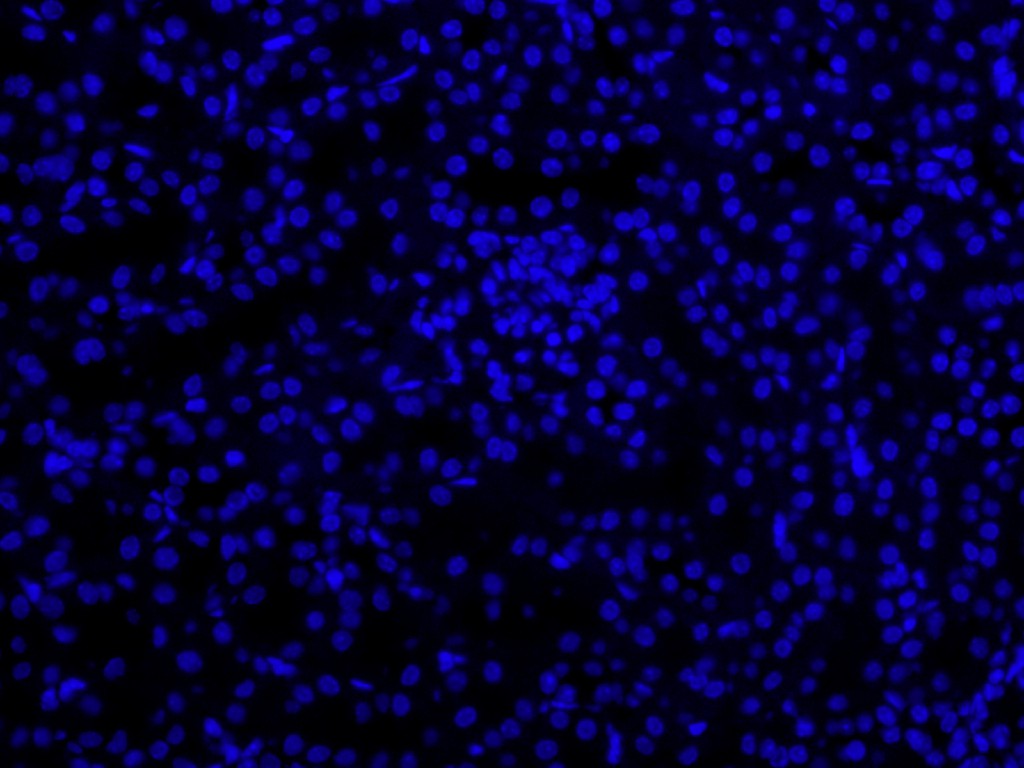

Supplement: Supplementary file 7 — Source data Fig. 6 [file 44321_2025_315_MOESM7_ESM.zip › Figure 6/F6A/1-IgA/1-4 (2).jpg]

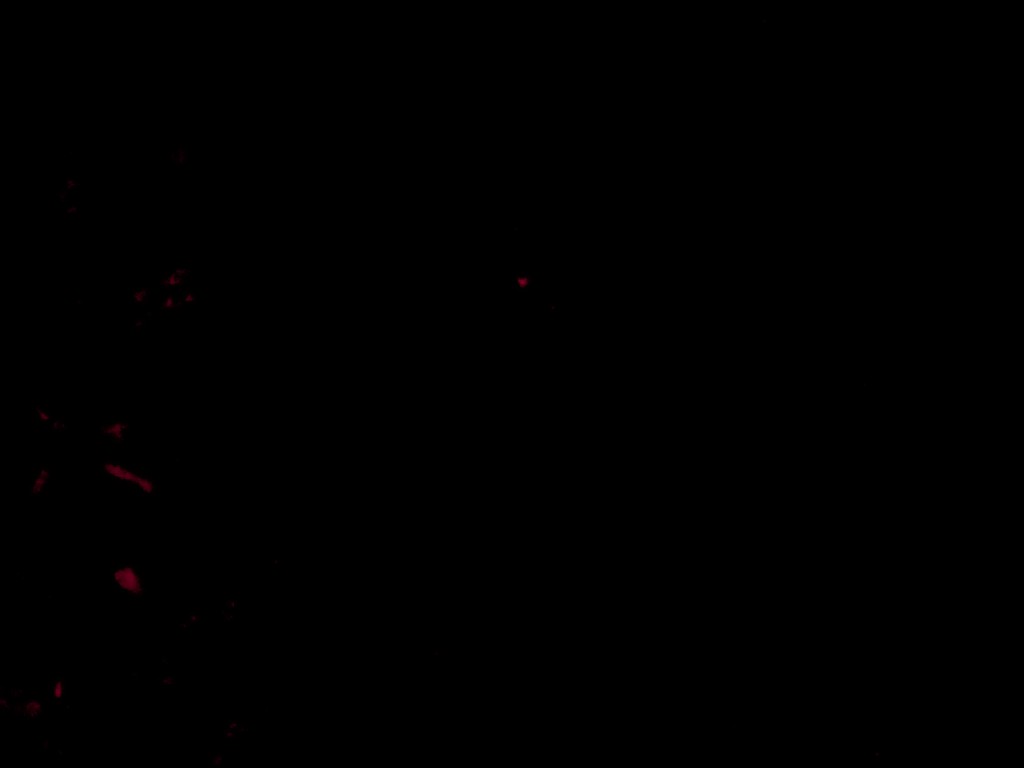

Supplement: Supplementary file 7 — Source data Fig. 6 [file 44321_2025_315_MOESM7_ESM.zip › Figure 6/F6A/1-IgA/1-4 (3).jpg]

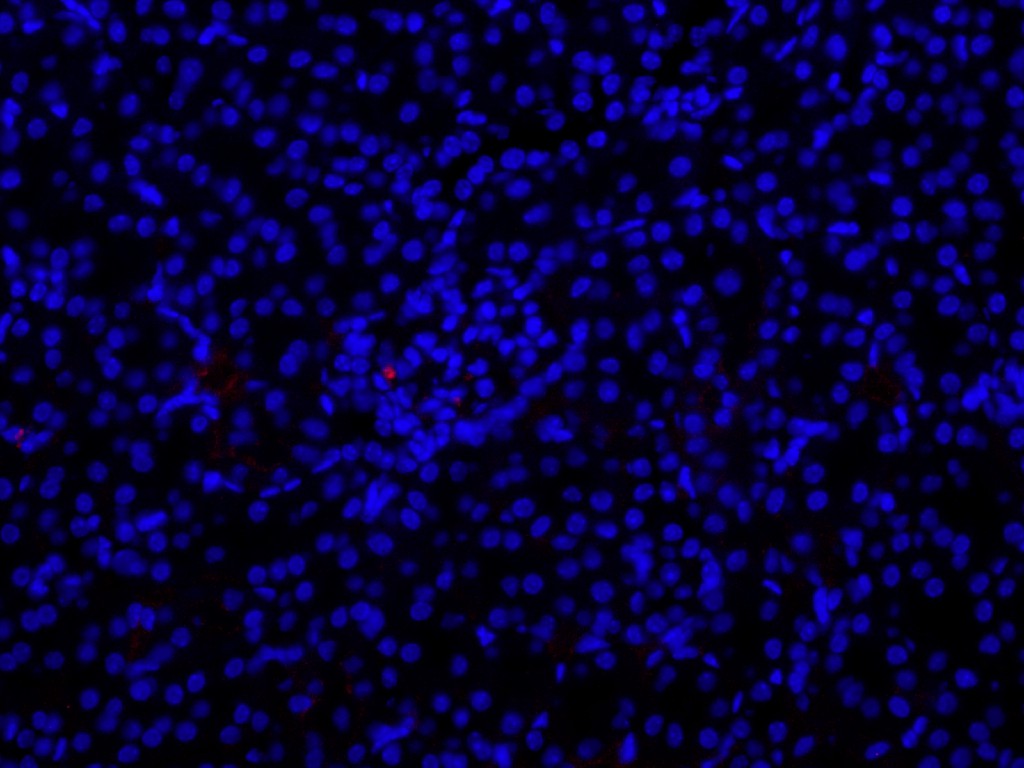

Supplement: Supplementary file 7 — Source data Fig. 6 [file 44321_2025_315_MOESM7_ESM.zip › Figure 6/F6A/1-IgA/1-5 (1).jpg]

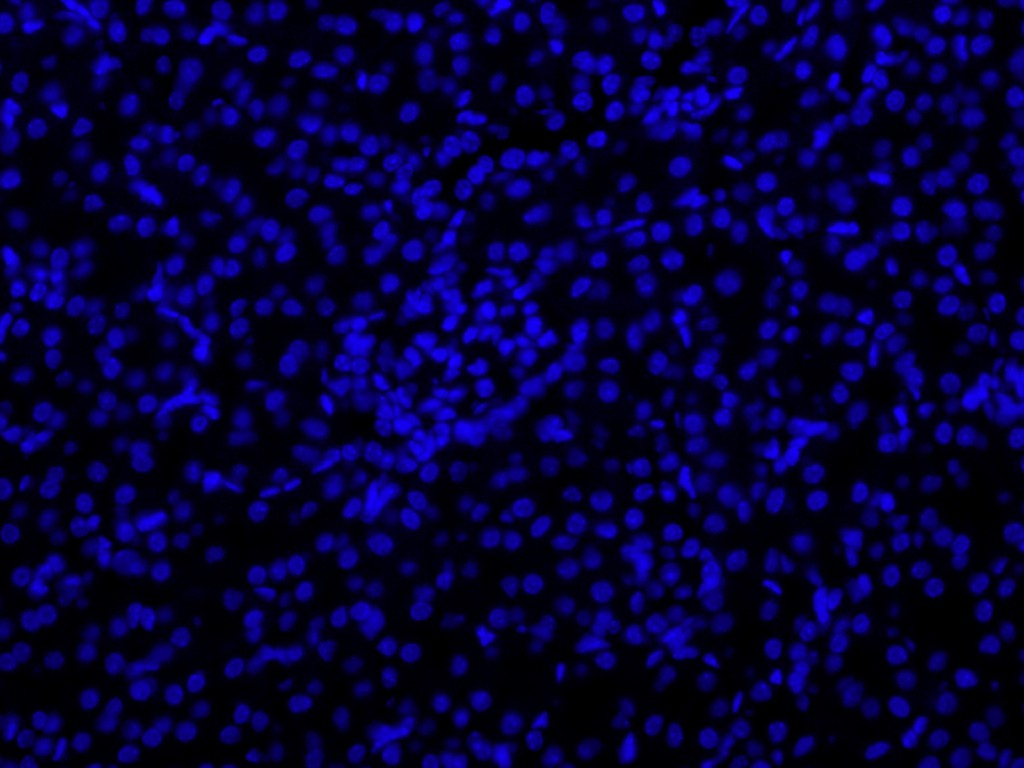

Supplement: Supplementary file 7 — Source data Fig. 6 [file 44321_2025_315_MOESM7_ESM.zip › Figure 6/F6A/1-IgA/1-5 (2).jpg]

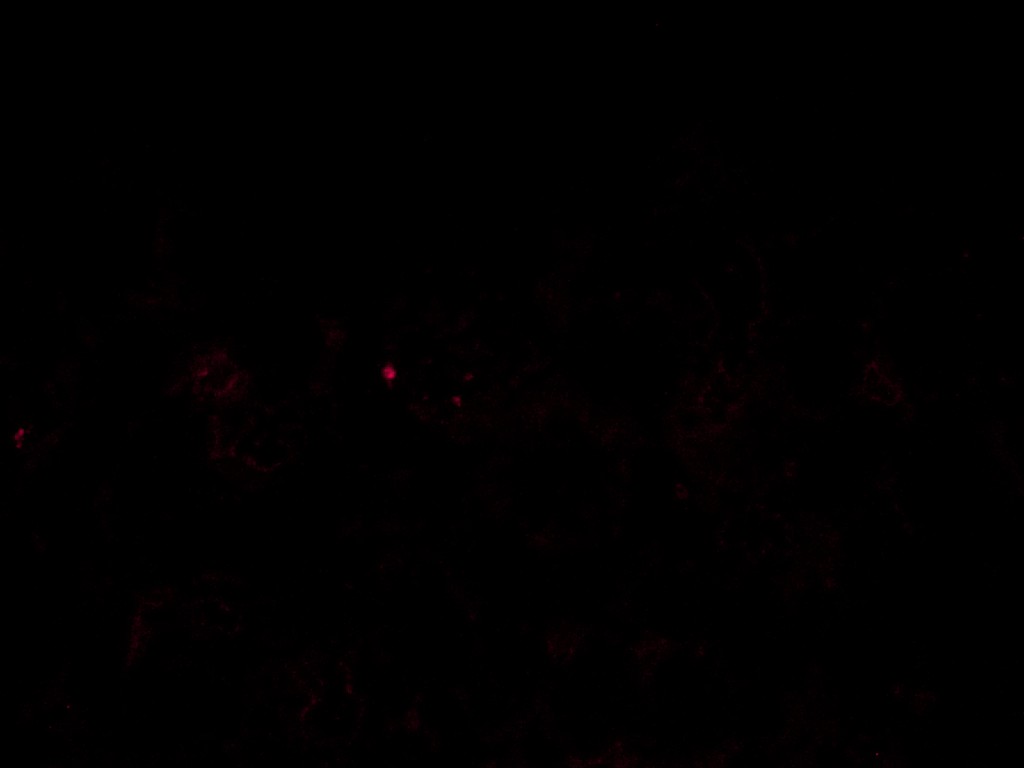

Supplement: Supplementary file 7 — Source data Fig. 6 [file 44321_2025_315_MOESM7_ESM.zip › Figure 6/F6A/1-IgA/1-5 (3).jpg]

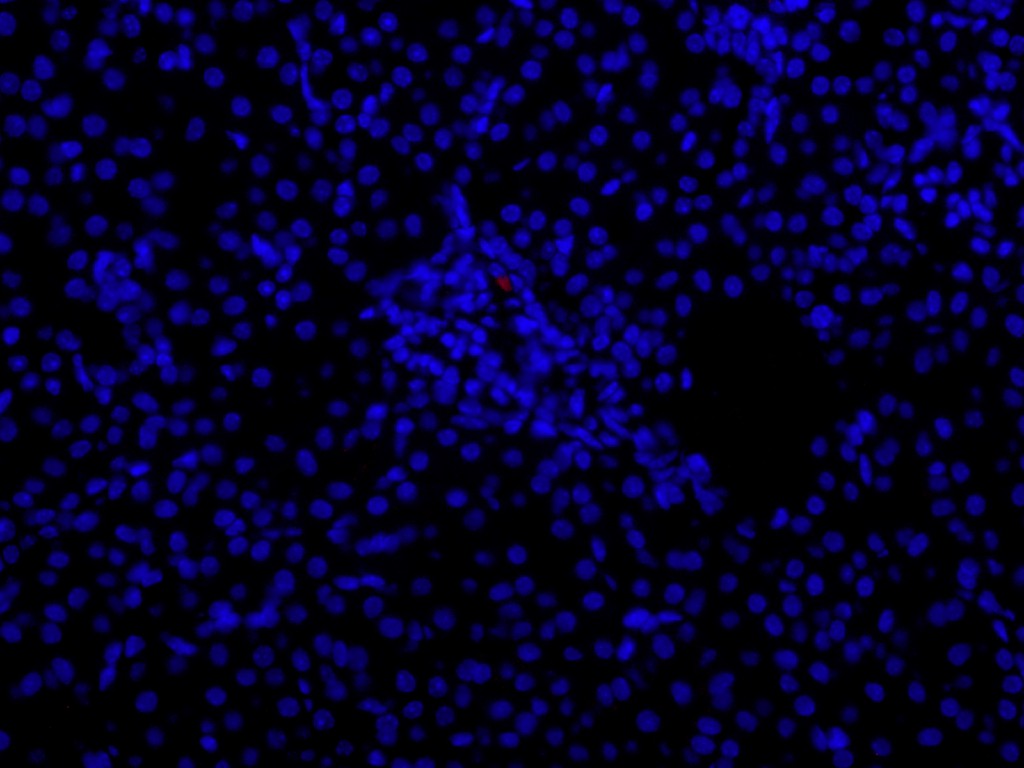

Supplement: Supplementary file 7 — Source data Fig. 6 [file 44321_2025_315_MOESM7_ESM.zip › Figure 6/F6A/1-IgA/1-6 (1).jpg]

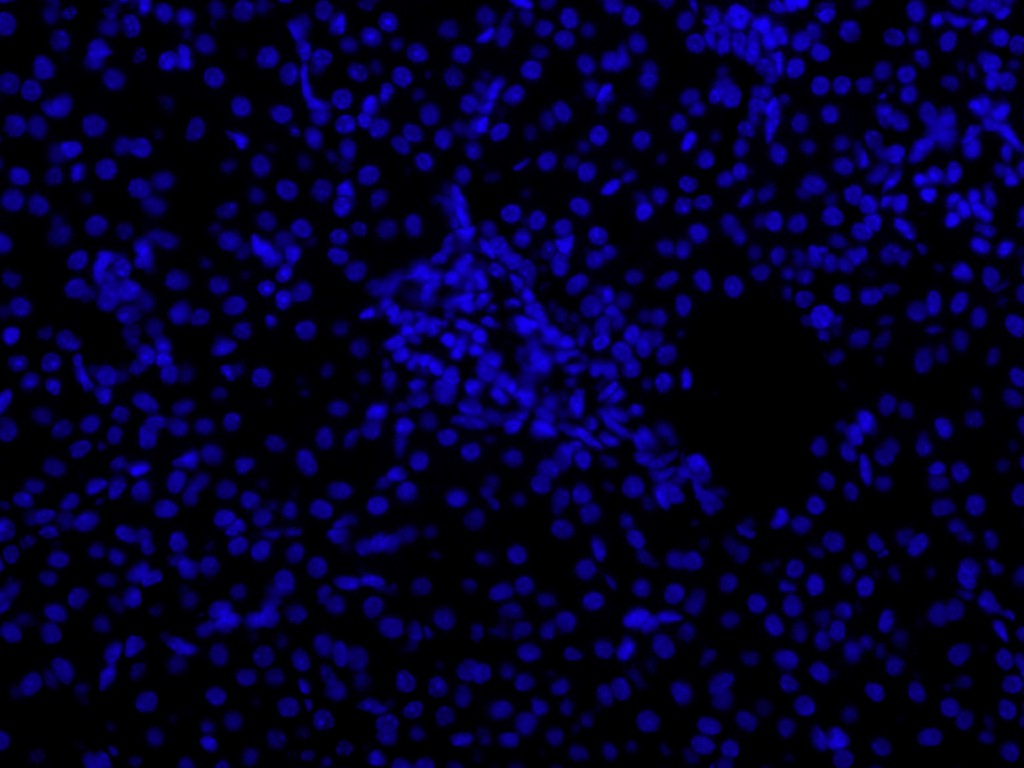

Supplement: Supplementary file 7 — Source data Fig. 6 [file 44321_2025_315_MOESM7_ESM.zip › Figure 6/F6A/1-IgA/1-6 (2).jpg]

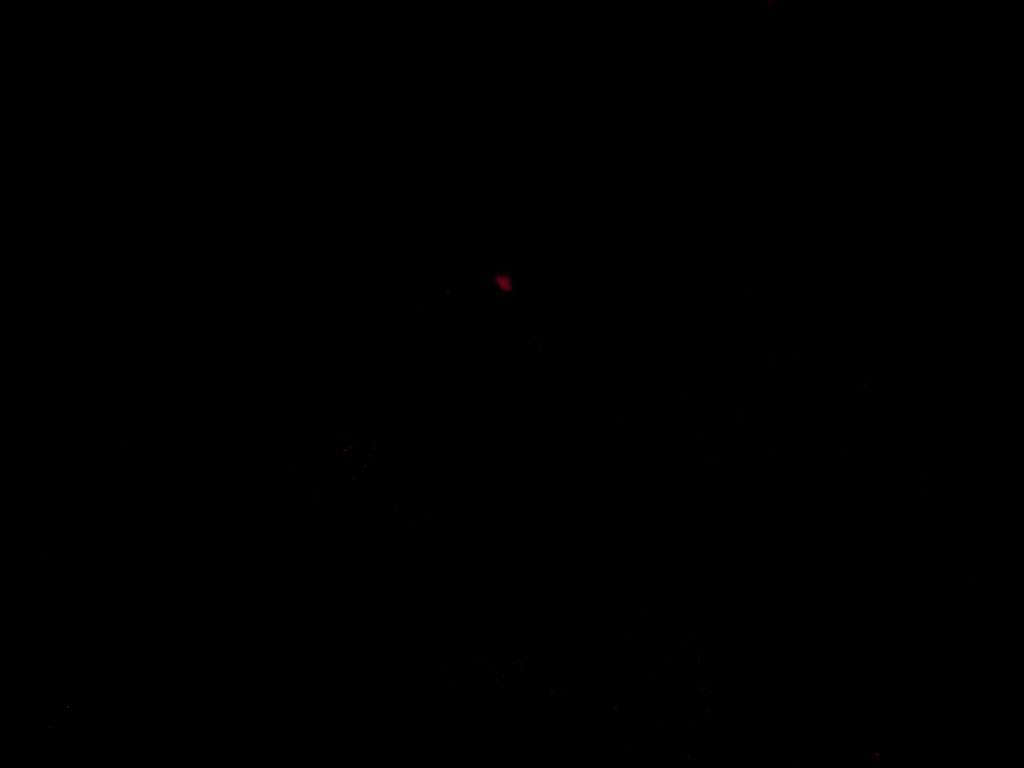

Supplement: Supplementary file 7 — Source data Fig. 6 [file 44321_2025_315_MOESM7_ESM.zip › Figure 6/F6A/1-IgA/1-6 (3).jpg]

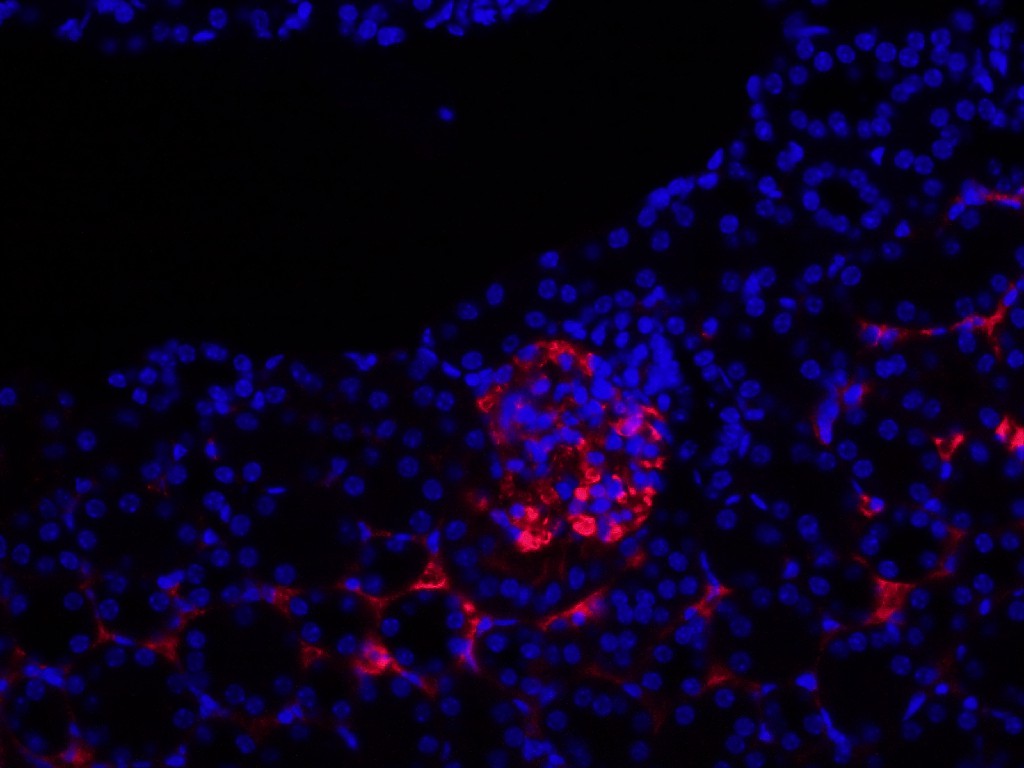

Supplement: Supplementary file 7 — Source data Fig. 6 [file 44321_2025_315_MOESM7_ESM.zip › Figure 6/F6A/1-IgA/2-1 (1).jpg]

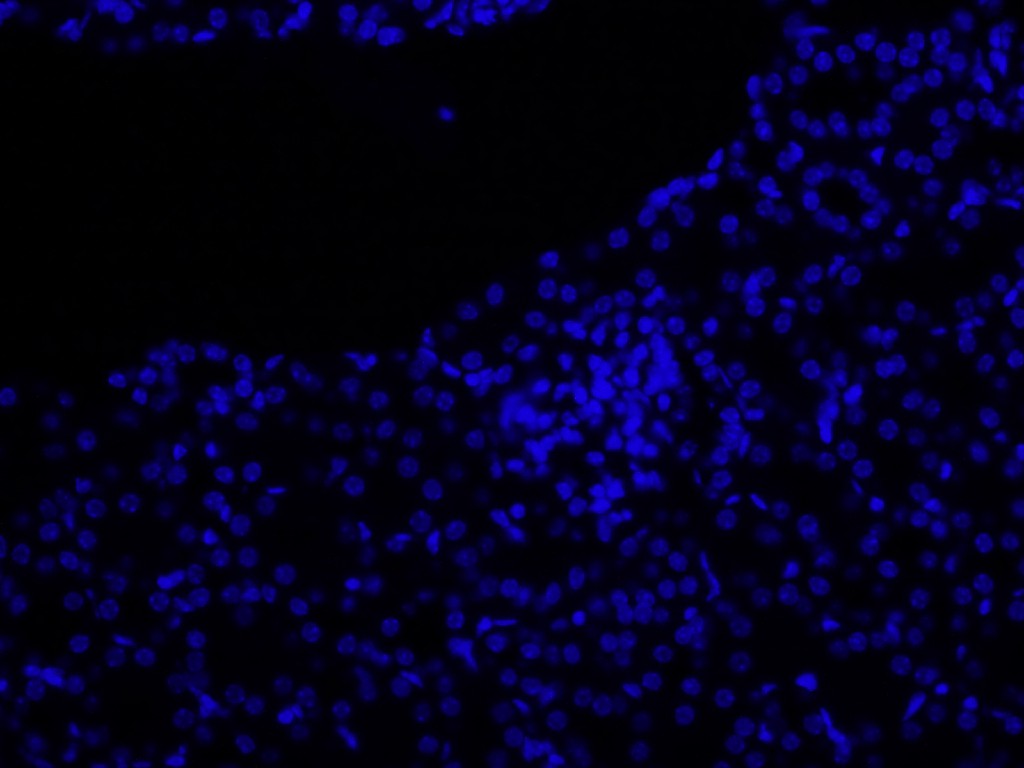

Supplement: Supplementary file 7 — Source data Fig. 6 [file 44321_2025_315_MOESM7_ESM.zip › Figure 6/F6A/1-IgA/2-1 (2).jpg]

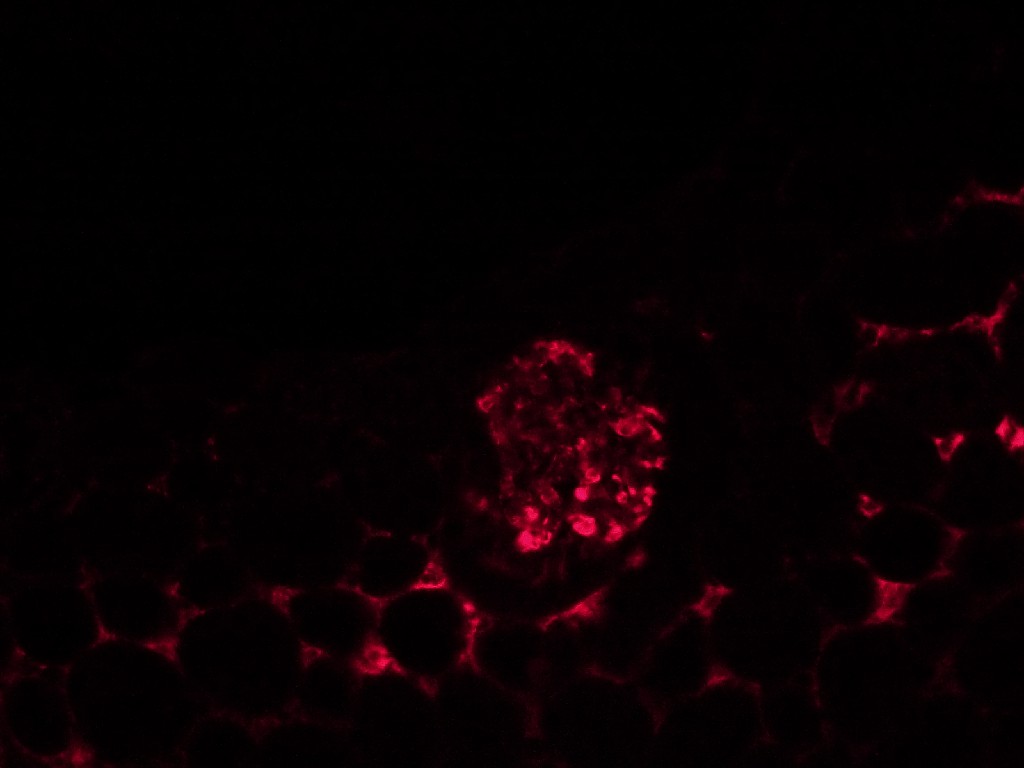

Supplement: Supplementary file 7 — Source data Fig. 6 [file 44321_2025_315_MOESM7_ESM.zip › Figure 6/F6A/1-IgA/2-1 (3).jpg]

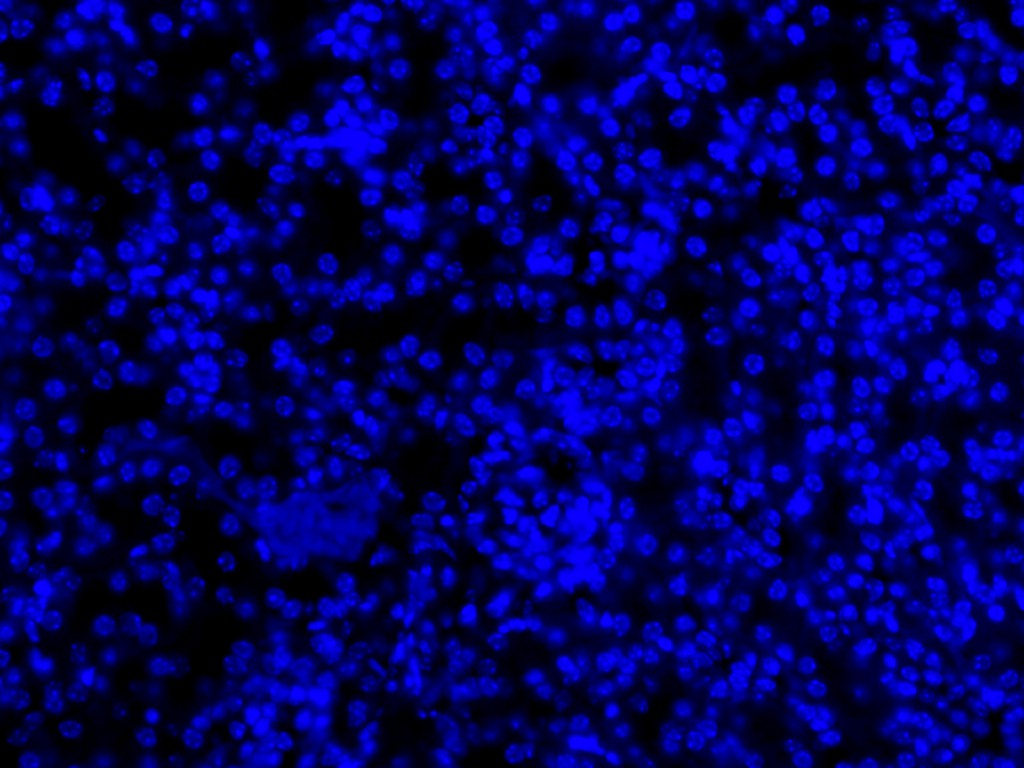

Supplement: Supplementary file 7 — Source data Fig. 6 [file 44321_2025_315_MOESM7_ESM.zip › Figure 6/F6A/1-IgA/2-2 (1).jpg]

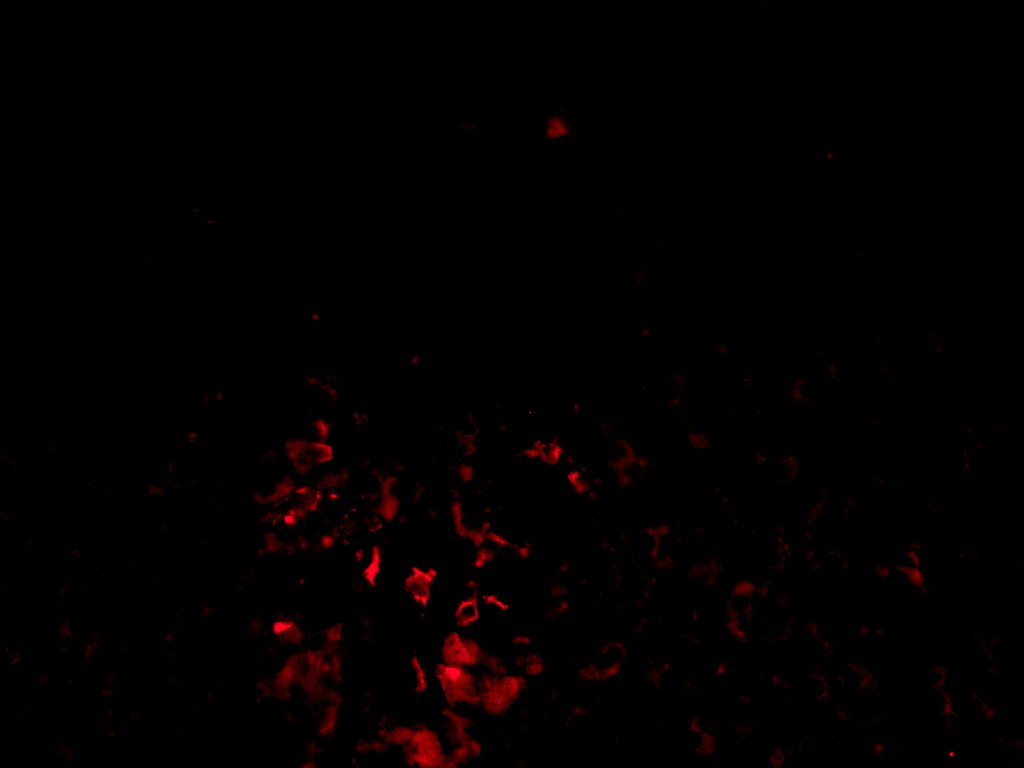

Supplement: Supplementary file 7 — Source data Fig. 6 [file 44321_2025_315_MOESM7_ESM.zip › Figure 6/F6A/1-IgA/2-2 (2).jpg]

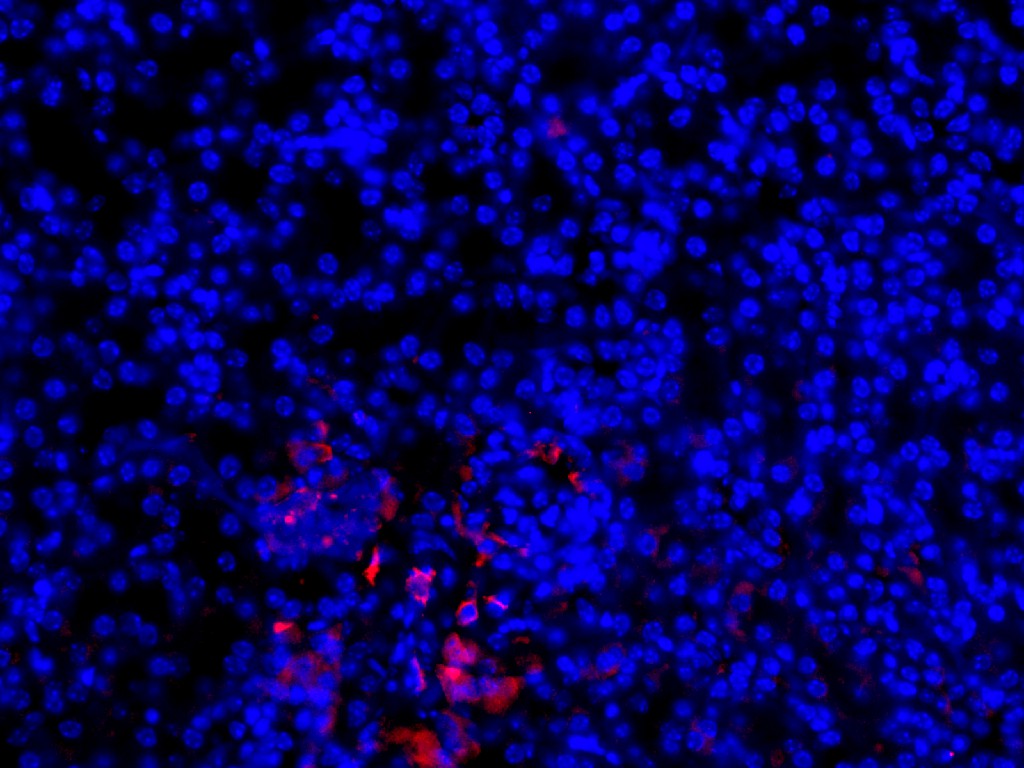

Supplement: Supplementary file 7 — Source data Fig. 6 [file 44321_2025_315_MOESM7_ESM.zip › Figure 6/F6A/1-IgA/2-2 (3).jpg]

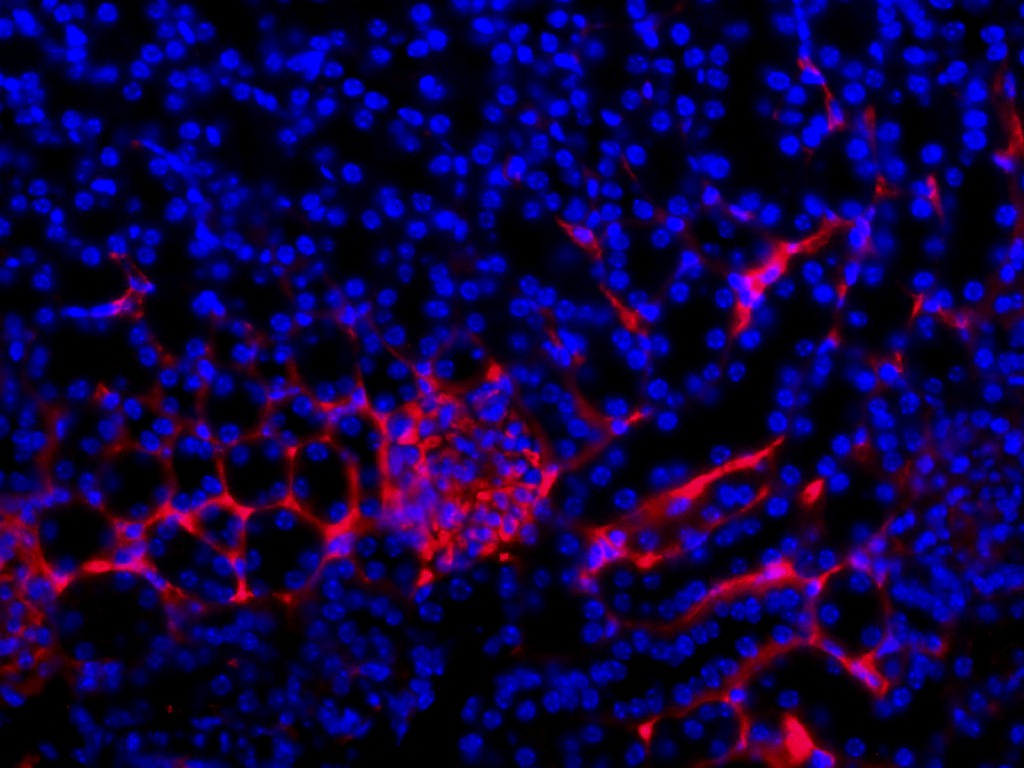

Supplement: Supplementary file 7 — Source data Fig. 6 [file 44321_2025_315_MOESM7_ESM.zip › Figure 6/F6A/1-IgA/2-3 (1).jpg]

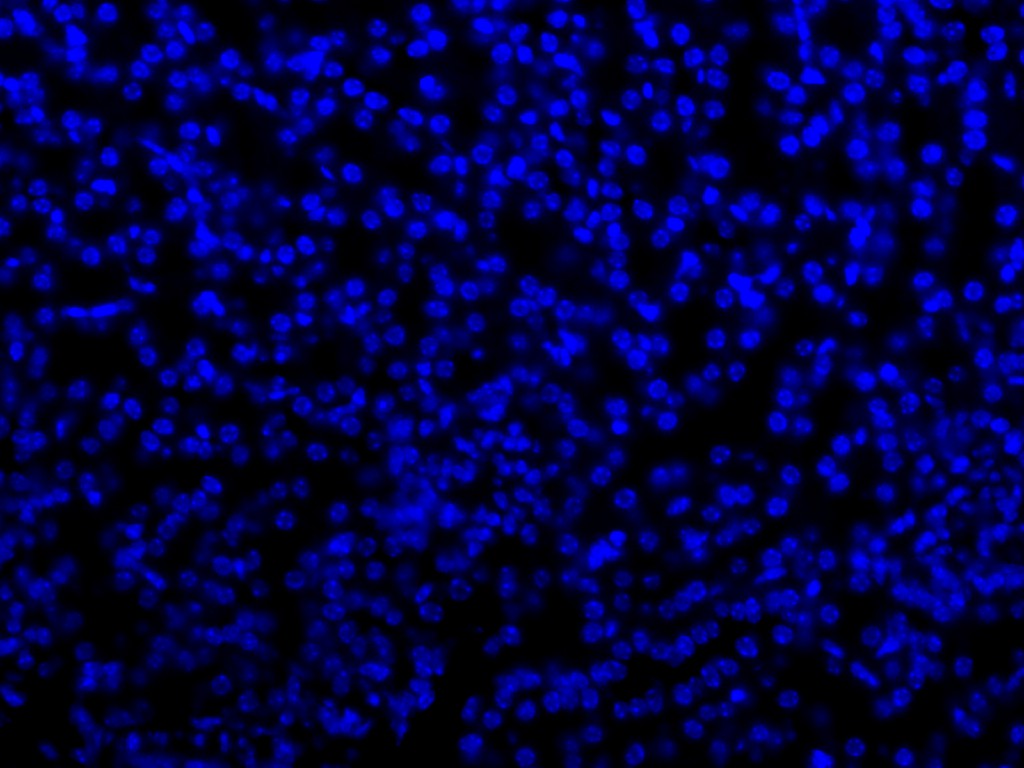

Supplement: Supplementary file 7 — Source data Fig. 6 [file 44321_2025_315_MOESM7_ESM.zip › Figure 6/F6A/1-IgA/2-3 (2).jpg]

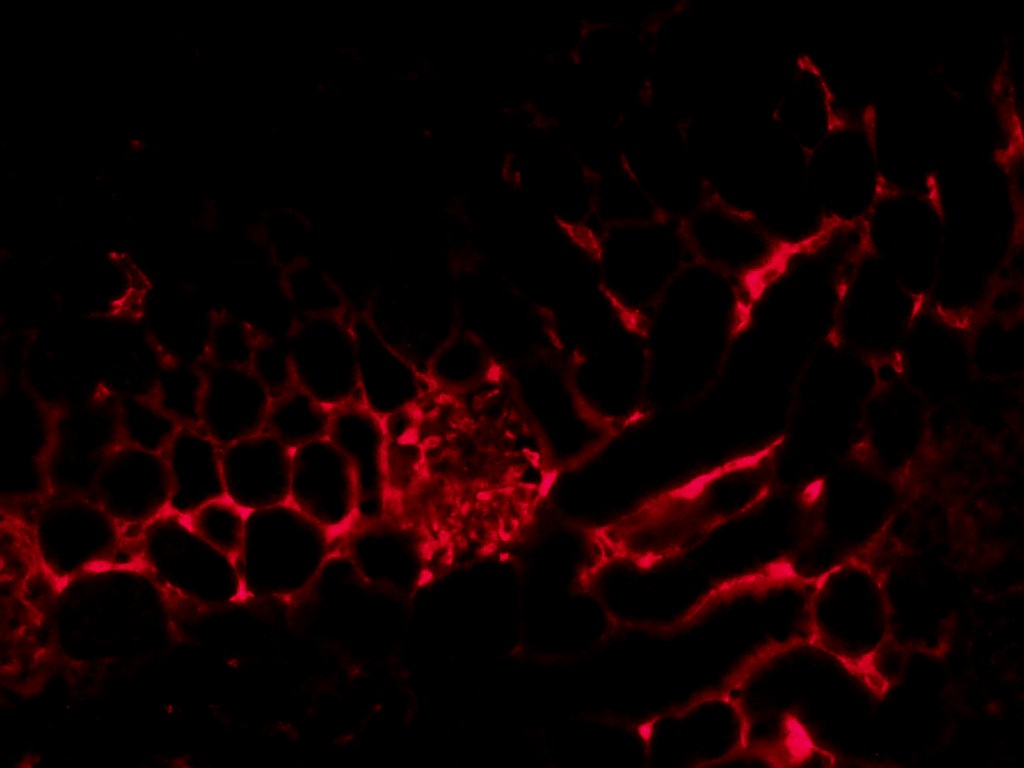

Supplement: Supplementary file 7 — Source data Fig. 6 [file 44321_2025_315_MOESM7_ESM.zip › Figure 6/F6A/1-IgA/2-3 (3).jpg]

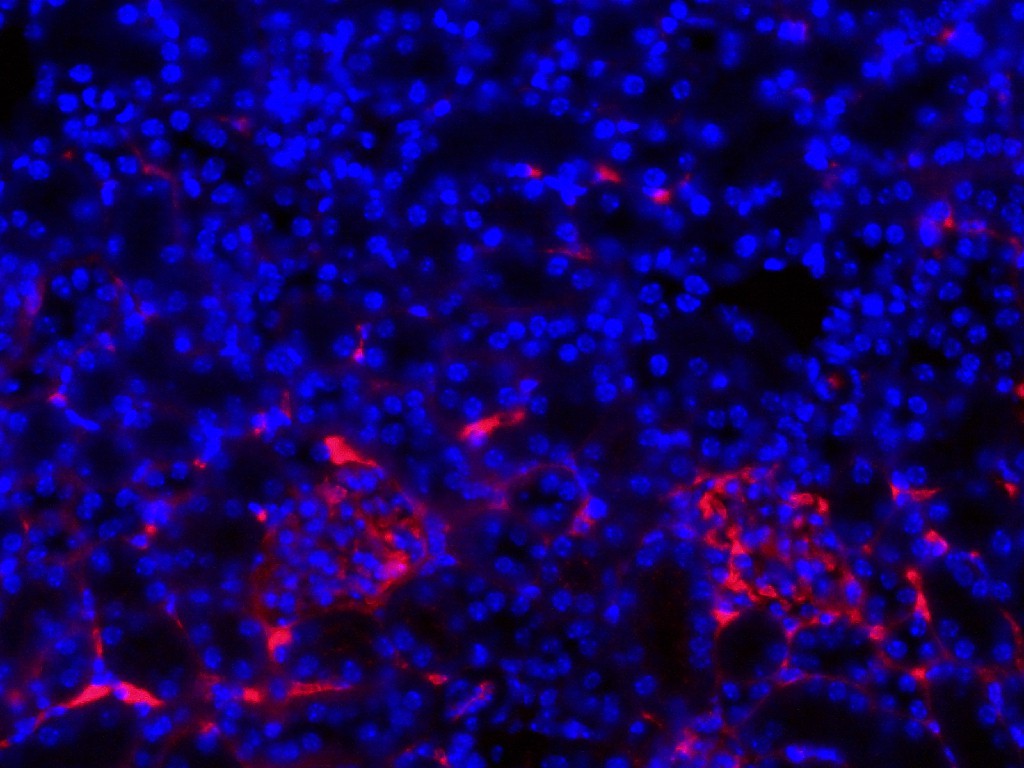

Supplement: Supplementary file 7 — Source data Fig. 6 [file 44321_2025_315_MOESM7_ESM.zip › Figure 6/F6A/1-IgA/2-4 (1).jpg]

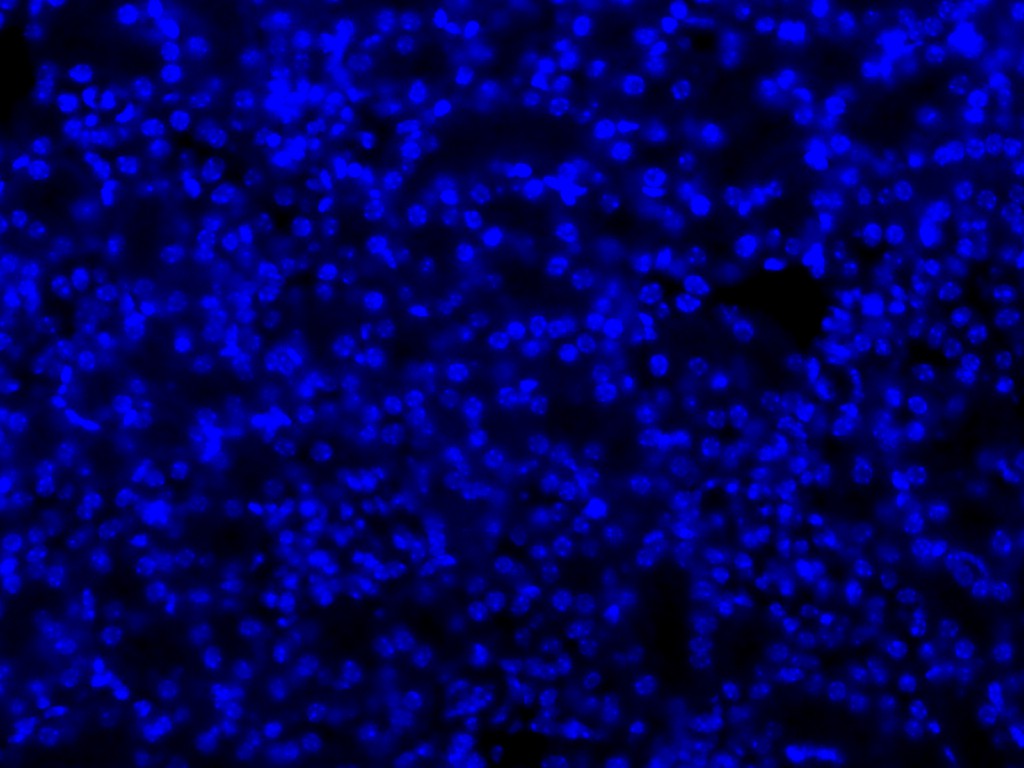

Supplement: Supplementary file 7 — Source data Fig. 6 [file 44321_2025_315_MOESM7_ESM.zip › Figure 6/F6A/1-IgA/2-4 (2).jpg]

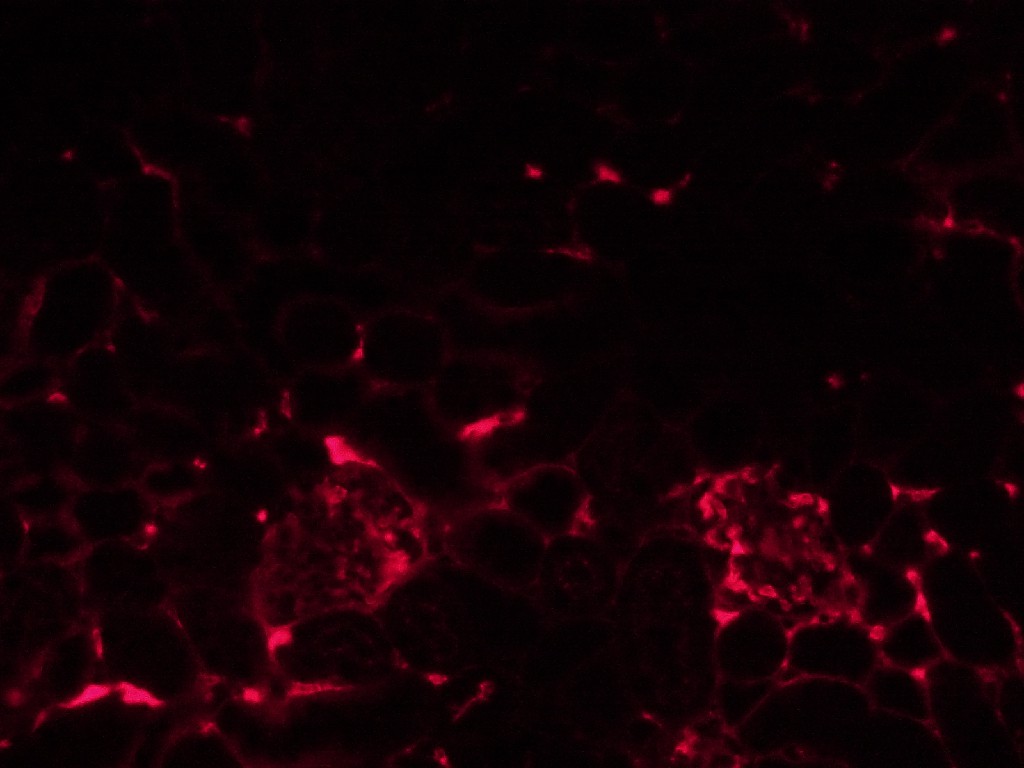

Supplement: Supplementary file 7 — Source data Fig. 6 [file 44321_2025_315_MOESM7_ESM.zip › Figure 6/F6A/1-IgA/2-4 (3).jpg]

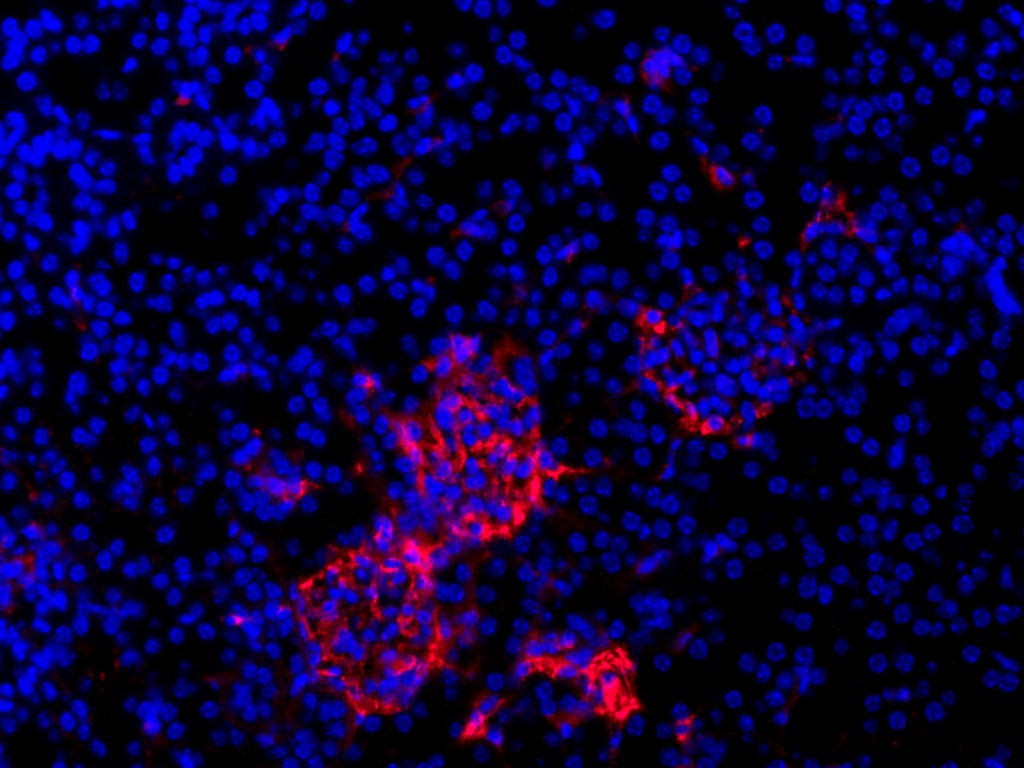

Supplement: Supplementary file 7 — Source data Fig. 6 [file 44321_2025_315_MOESM7_ESM.zip › Figure 6/F6A/1-IgA/2-5 (1).jpg]

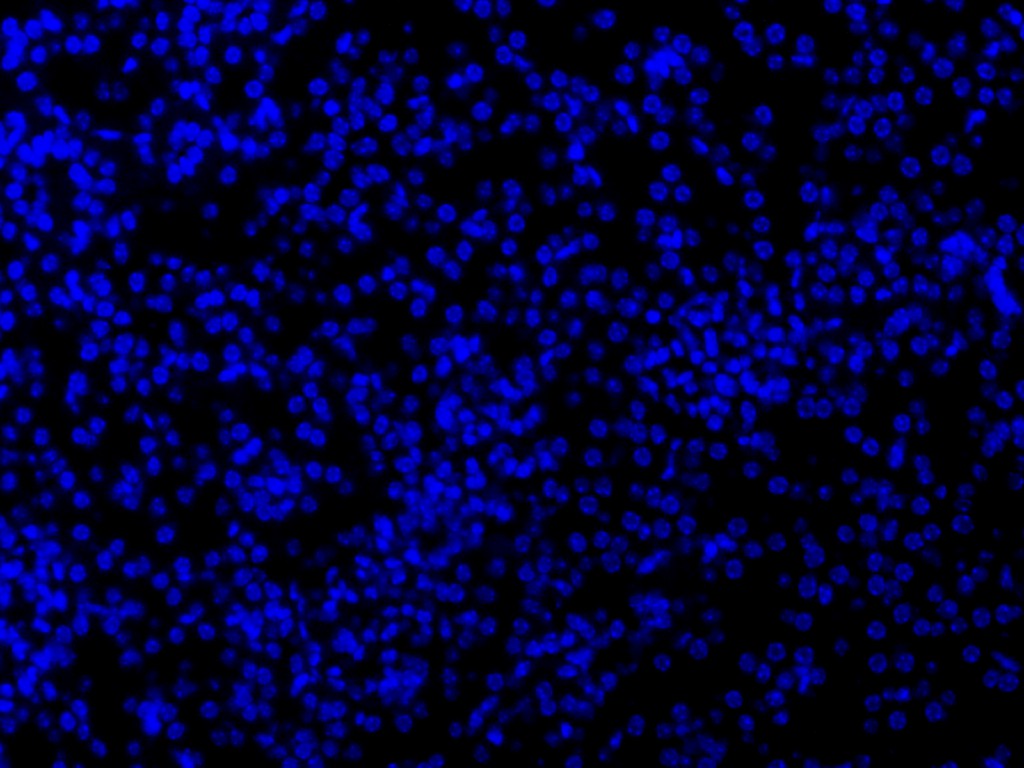

Supplement: Supplementary file 7 — Source data Fig. 6 [file 44321_2025_315_MOESM7_ESM.zip › Figure 6/F6A/1-IgA/2-5 (2).jpg]

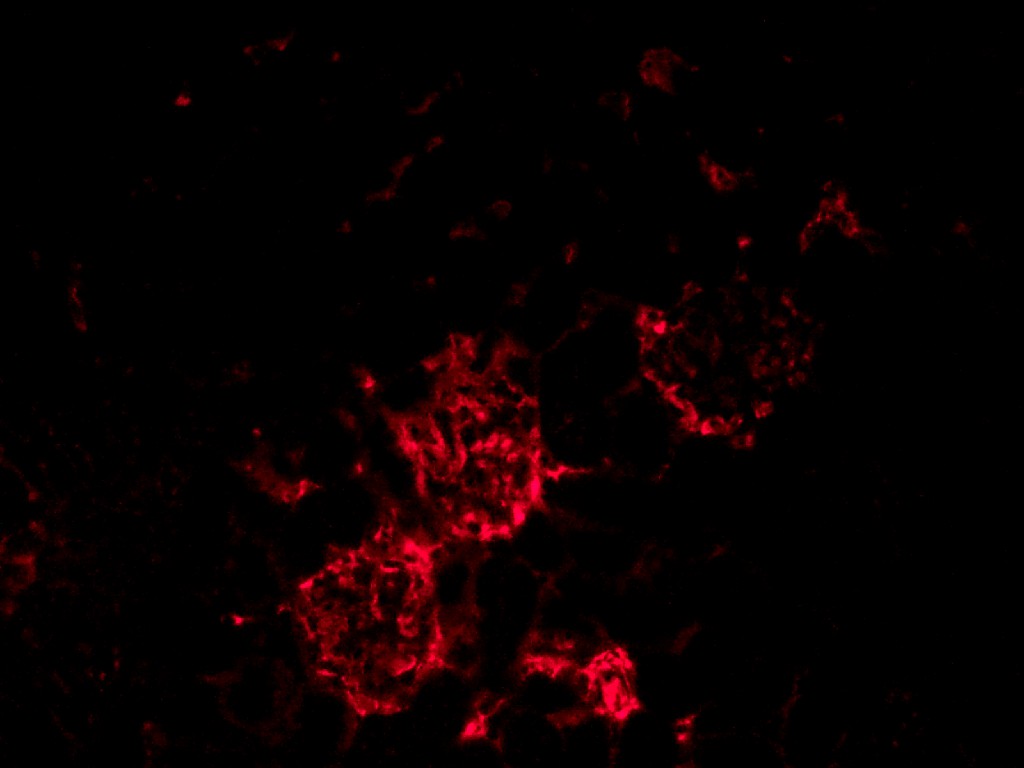

Supplement: Supplementary file 7 — Source data Fig. 6 [file 44321_2025_315_MOESM7_ESM.zip › Figure 6/F6A/1-IgA/2-5 (3).jpg]

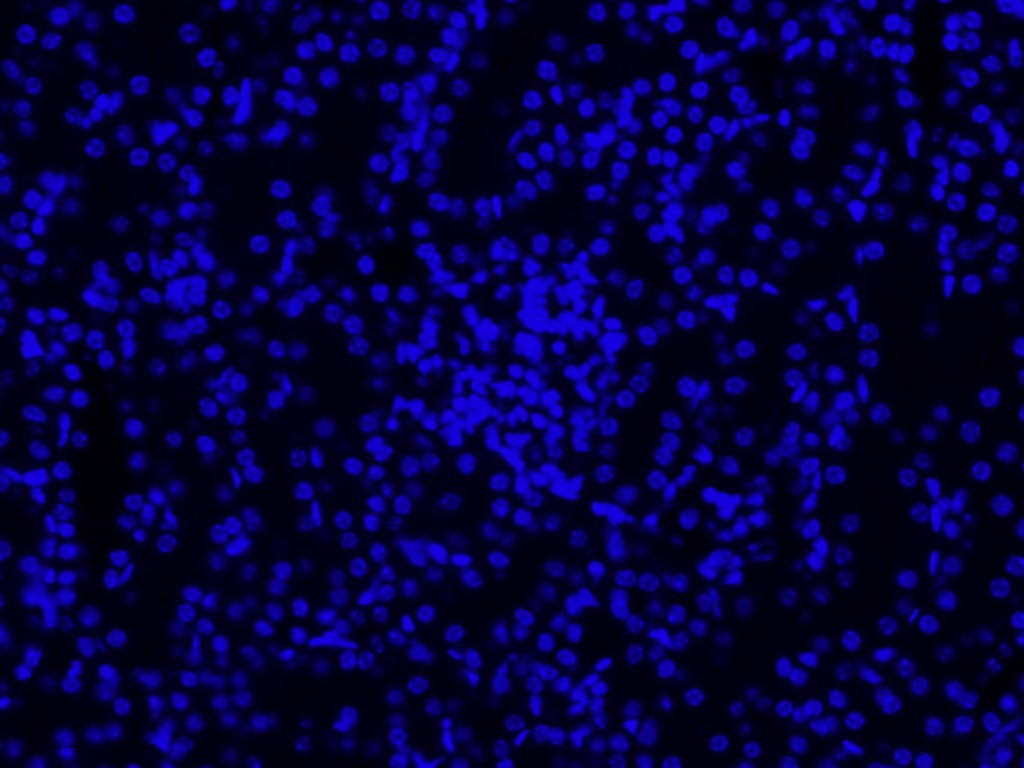

Supplement: Supplementary file 7 — Source data Fig. 6 [file 44321_2025_315_MOESM7_ESM.zip › Figure 6/F6A/1-IgA/2-6 (1).jpg]

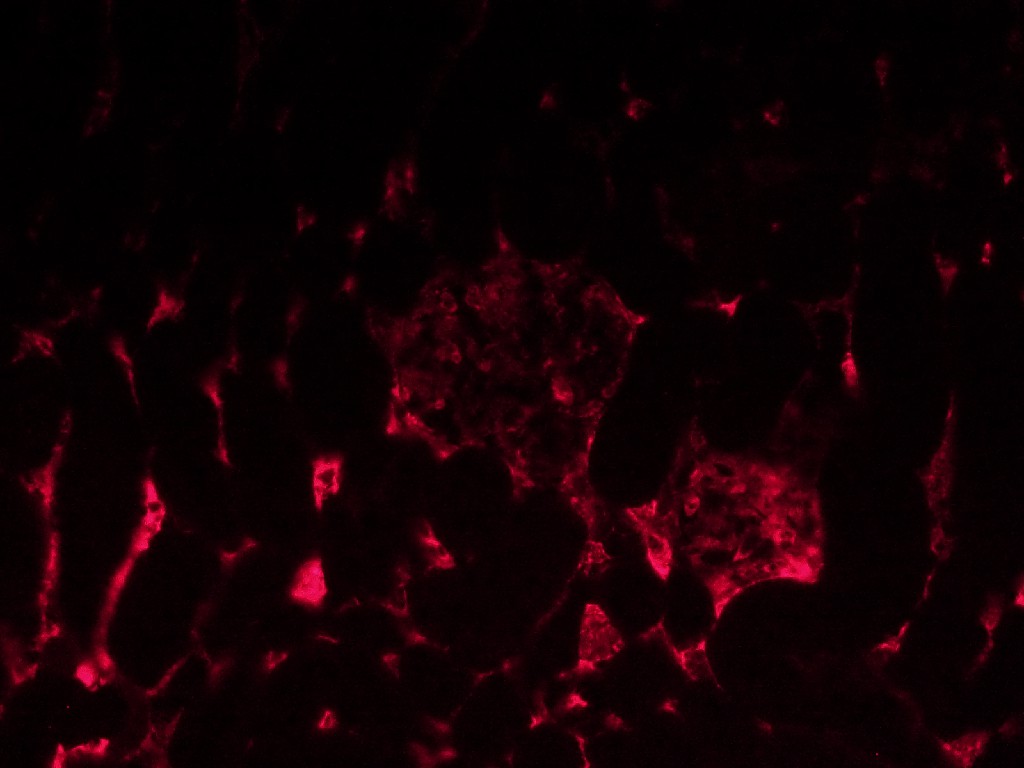

Supplement: Supplementary file 7 — Source data Fig. 6 [file 44321_2025_315_MOESM7_ESM.zip › Figure 6/F6A/1-IgA/2-6 (2).jpg]

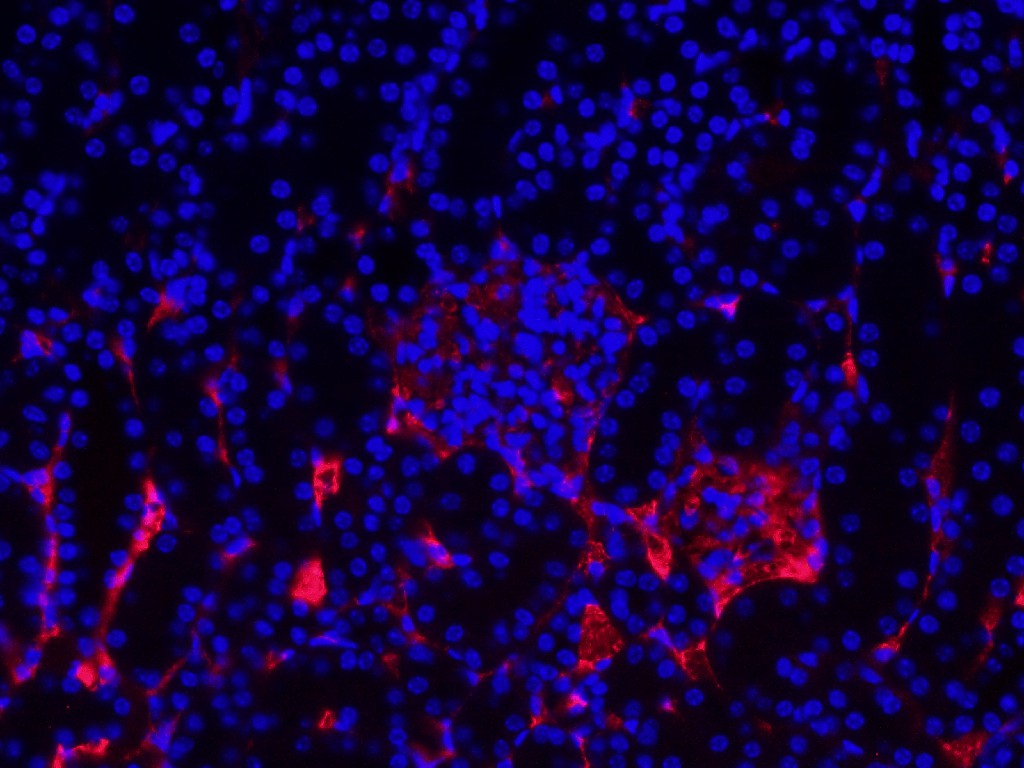

Supplement: Supplementary file 7 — Source data Fig. 6 [file 44321_2025_315_MOESM7_ESM.zip › Figure 6/F6A/1-IgA/2-6 (3).jpg]

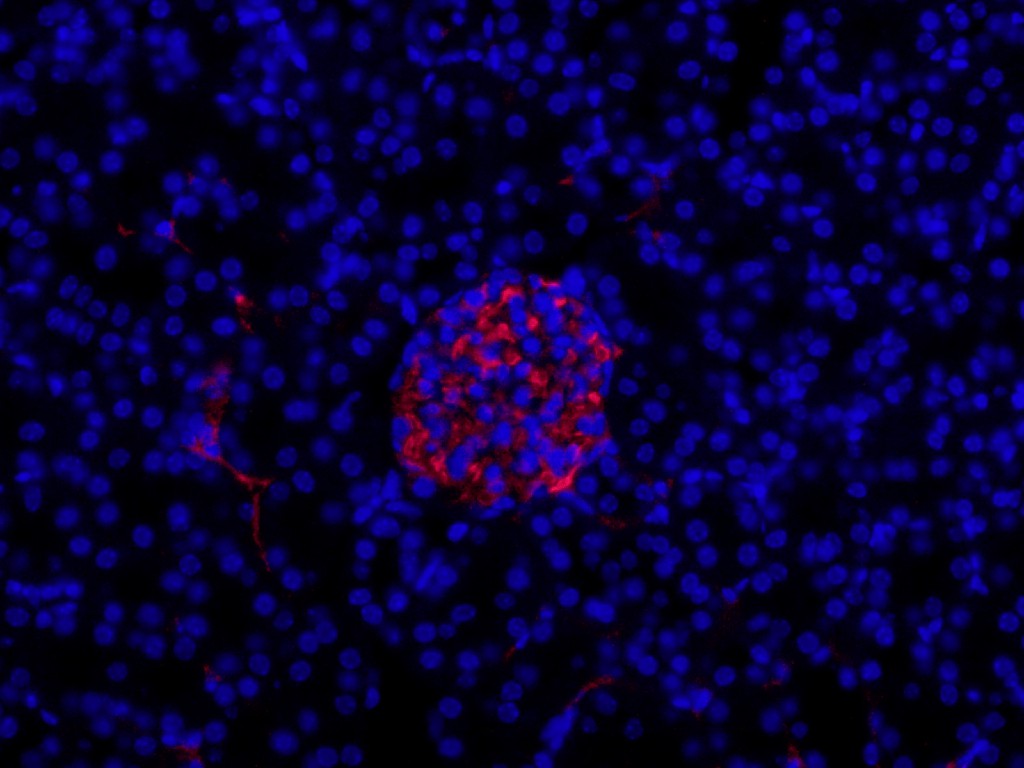

Supplement: Supplementary file 7 — Source data Fig. 6 [file 44321_2025_315_MOESM7_ESM.zip › Figure 6/F6A/1-IgA/3-1 (1).jpg]

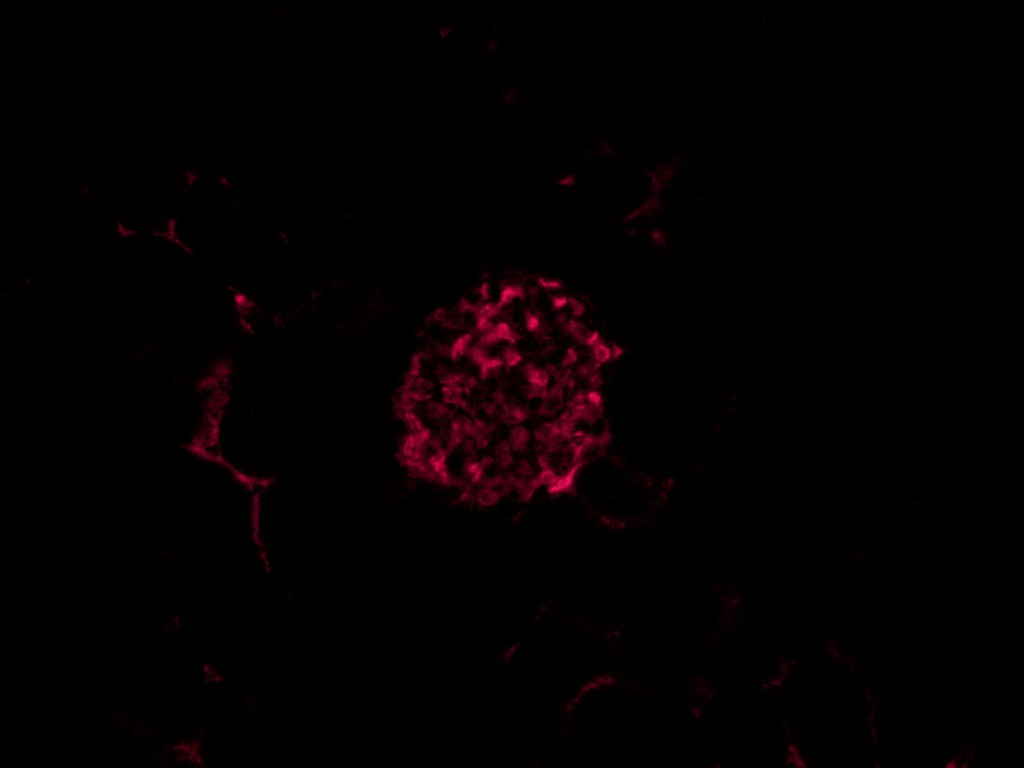

Supplement: Supplementary file 7 — Source data Fig. 6 [file 44321_2025_315_MOESM7_ESM.zip › Figure 6/F6A/1-IgA/3-1 (2).jpg]
