# Supplementary figures and images for: Glycine decarboxylase advances IgA nephropathy by boosting mesangial cell proliferation through the pyrimidine pathway (part 5 of 7)
Source: EMBO Mol Med. 2025 Oct 13;17(11):3039–63. doi: 10.1038/s44321-025-00315-2 (PMC12603144; doi:10.1038/s44321-025-00315-2)

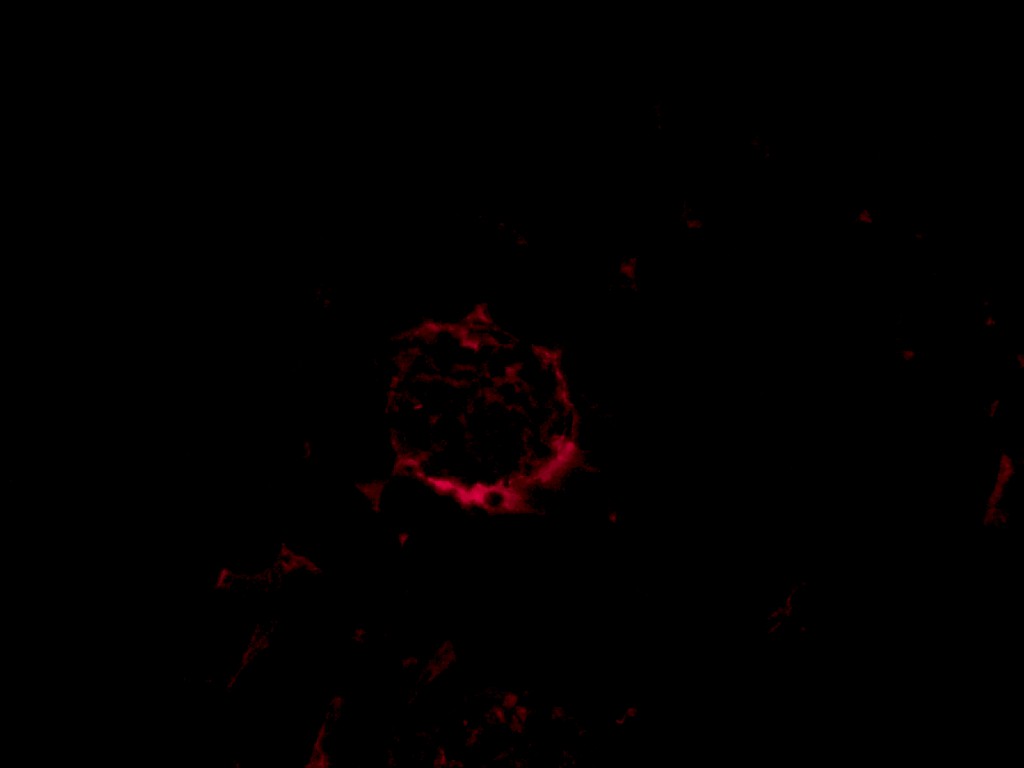

Supplement: Supplementary file 7 — Source data Fig. 6 [file 44321_2025_315_MOESM7_ESM.zip › Figure 6/F6A/2-C3/4-5 (2).jpg]

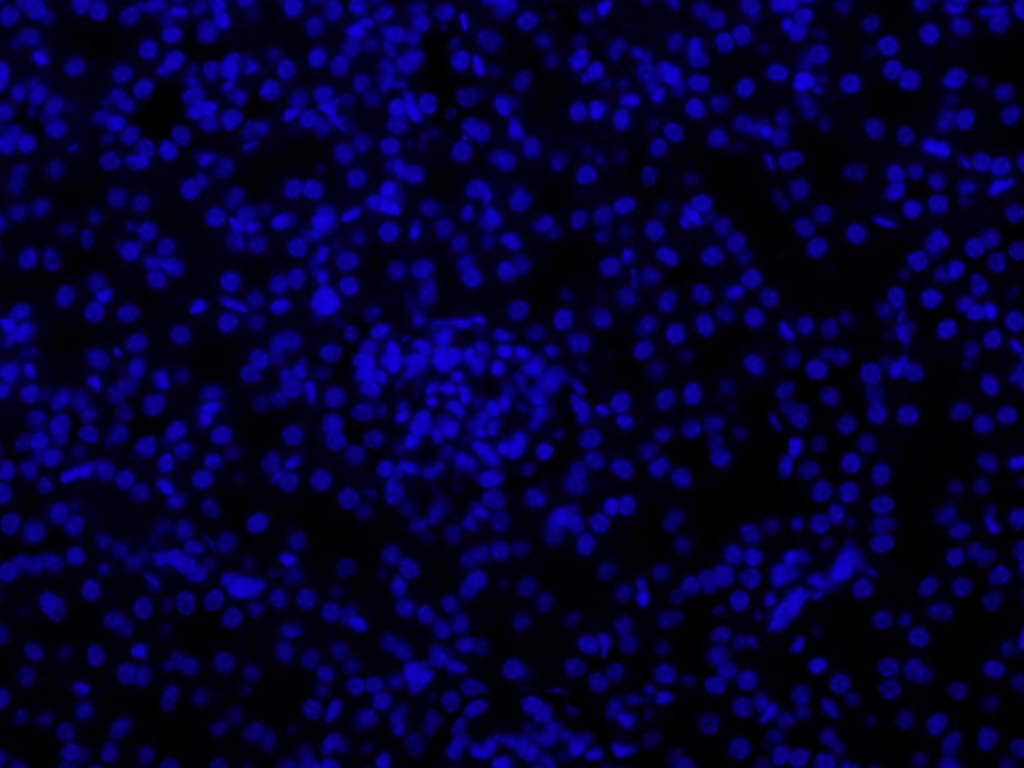

Supplement: Supplementary file 7 — Source data Fig. 6 [file 44321_2025_315_MOESM7_ESM.zip › Figure 6/F6A/2-C3/4-5 (3).jpg]

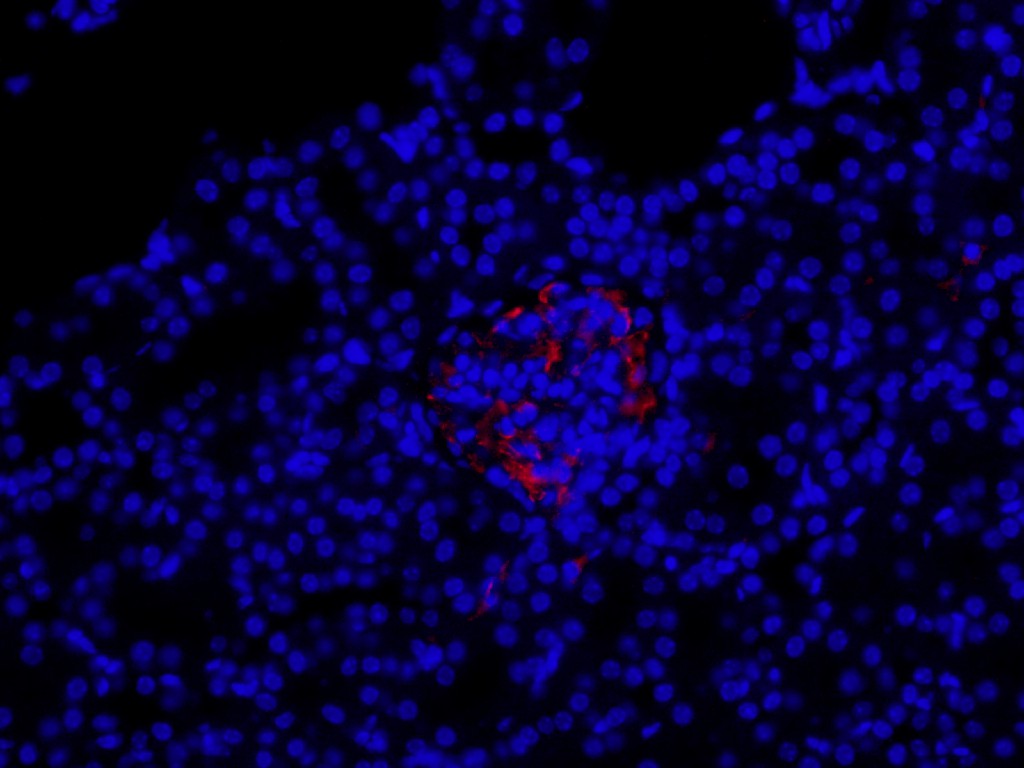

Supplement: Supplementary file 7 — Source data Fig. 6 [file 44321_2025_315_MOESM7_ESM.zip › Figure 6/F6A/2-C3/4-6 (1).jpg]

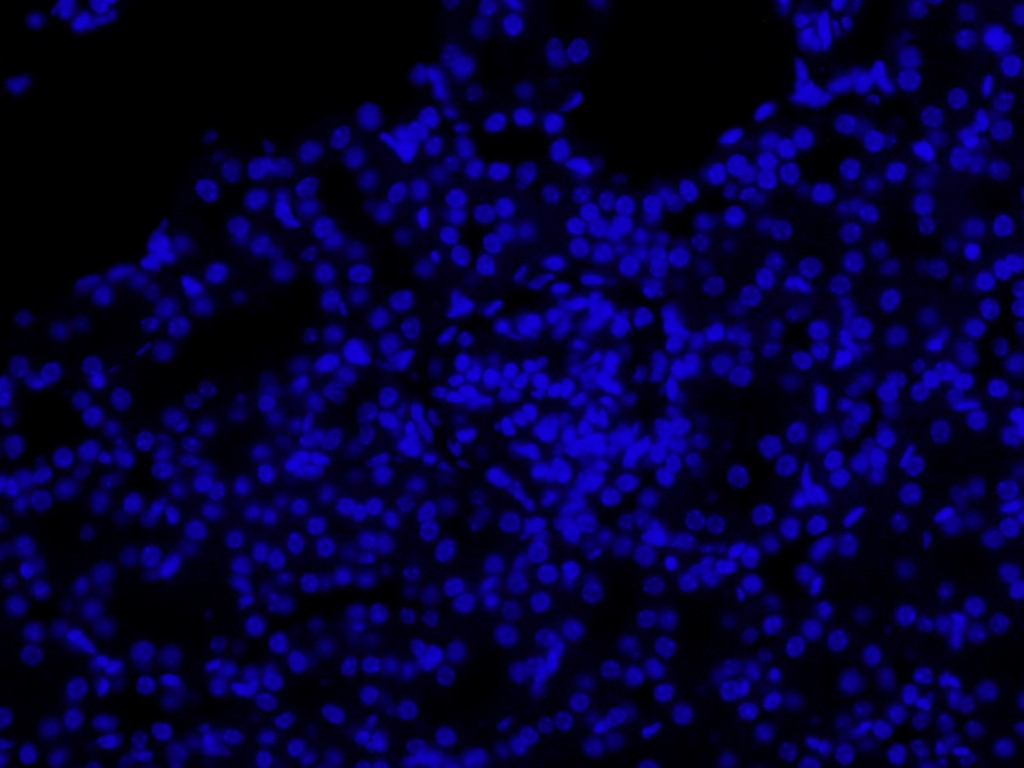

Supplement: Supplementary file 7 — Source data Fig. 6 [file 44321_2025_315_MOESM7_ESM.zip › Figure 6/F6A/2-C3/4-6 (2).jpg]

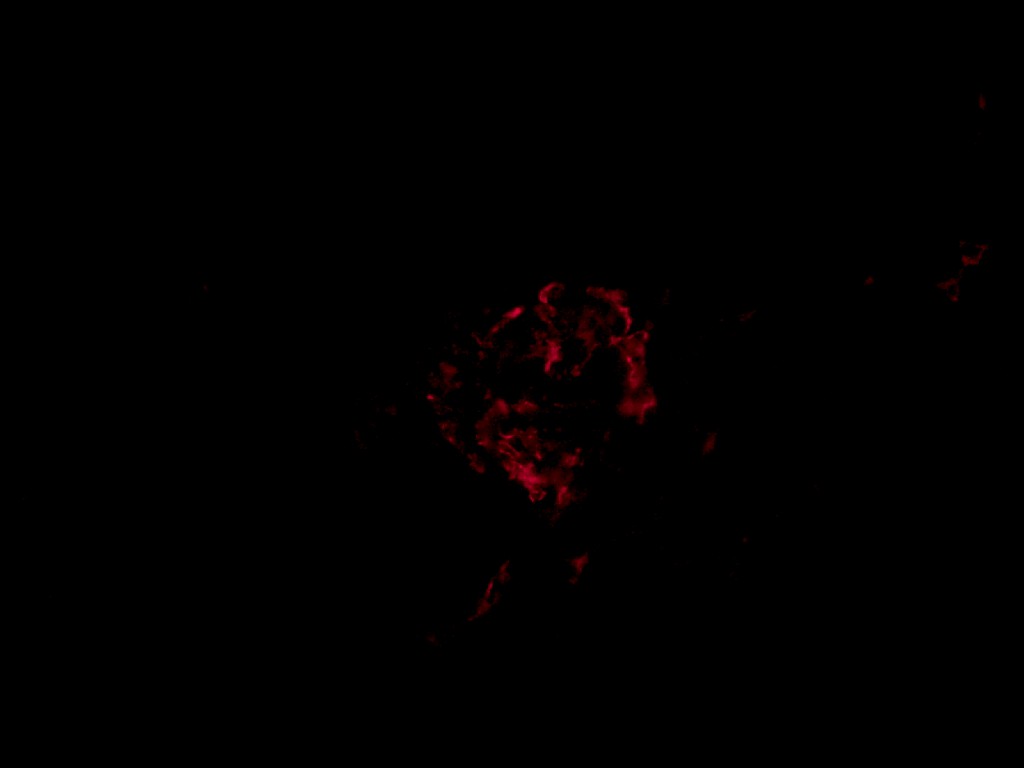

Supplement: Supplementary file 7 — Source data Fig. 6 [file 44321_2025_315_MOESM7_ESM.zip › Figure 6/F6A/2-C3/4-6 (3).jpg]

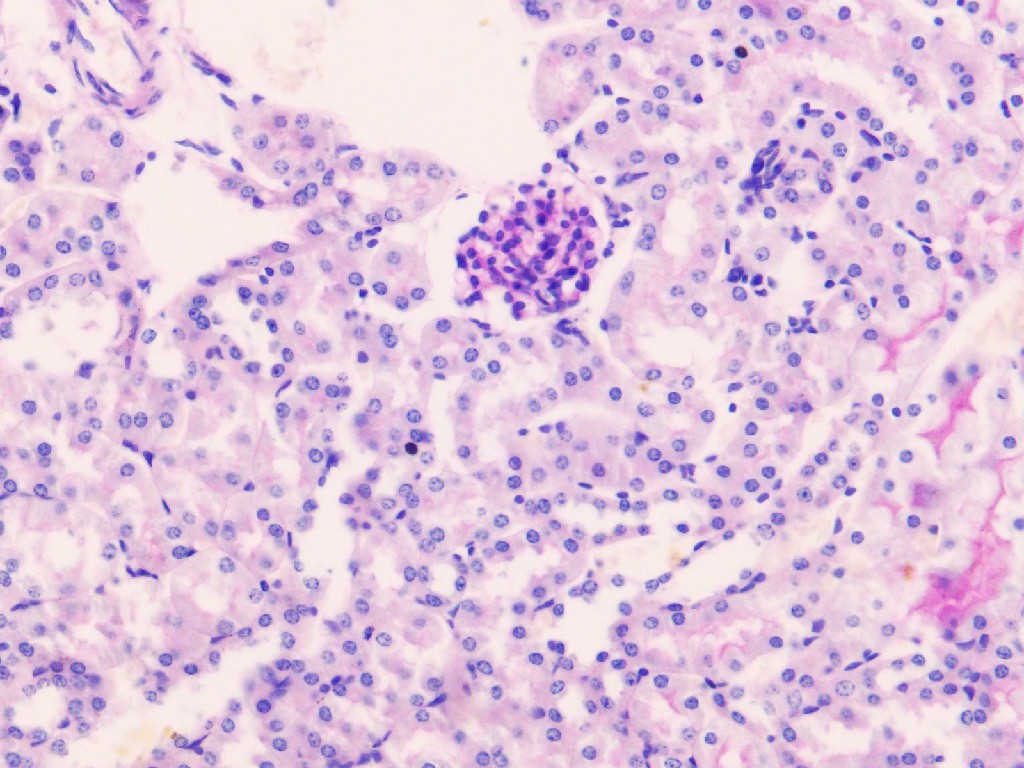

Supplement: Supplementary file 7 — Source data Fig. 6 [file 44321_2025_315_MOESM7_ESM.zip › Figure 6/F6A/3-PAS/1.jpg]

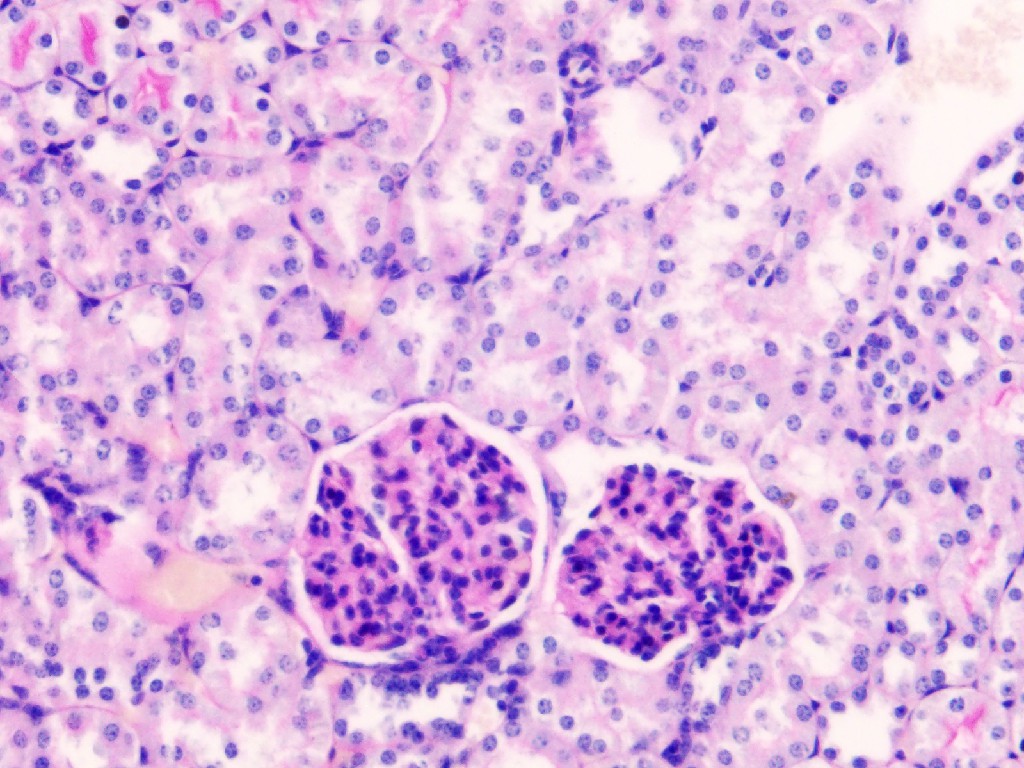

Supplement: Supplementary file 7 — Source data Fig. 6 [file 44321_2025_315_MOESM7_ESM.zip › Figure 6/F6A/3-PAS/2.jpg]

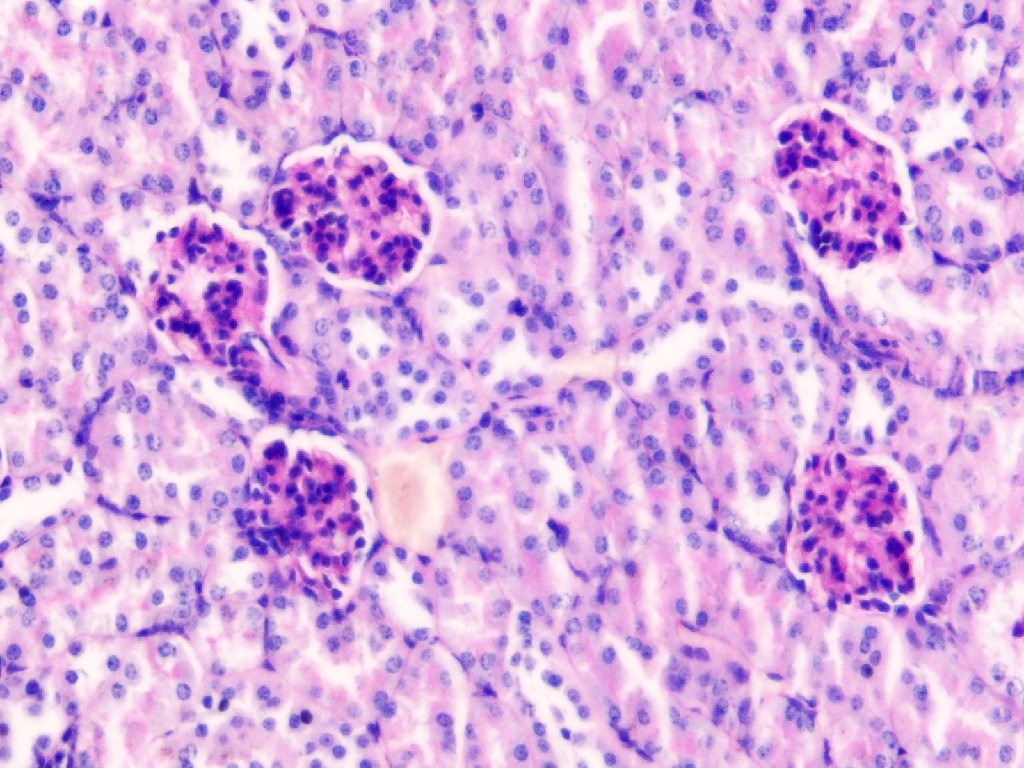

Supplement: Supplementary file 7 — Source data Fig. 6 [file 44321_2025_315_MOESM7_ESM.zip › Figure 6/F6A/3-PAS/3.jpg]

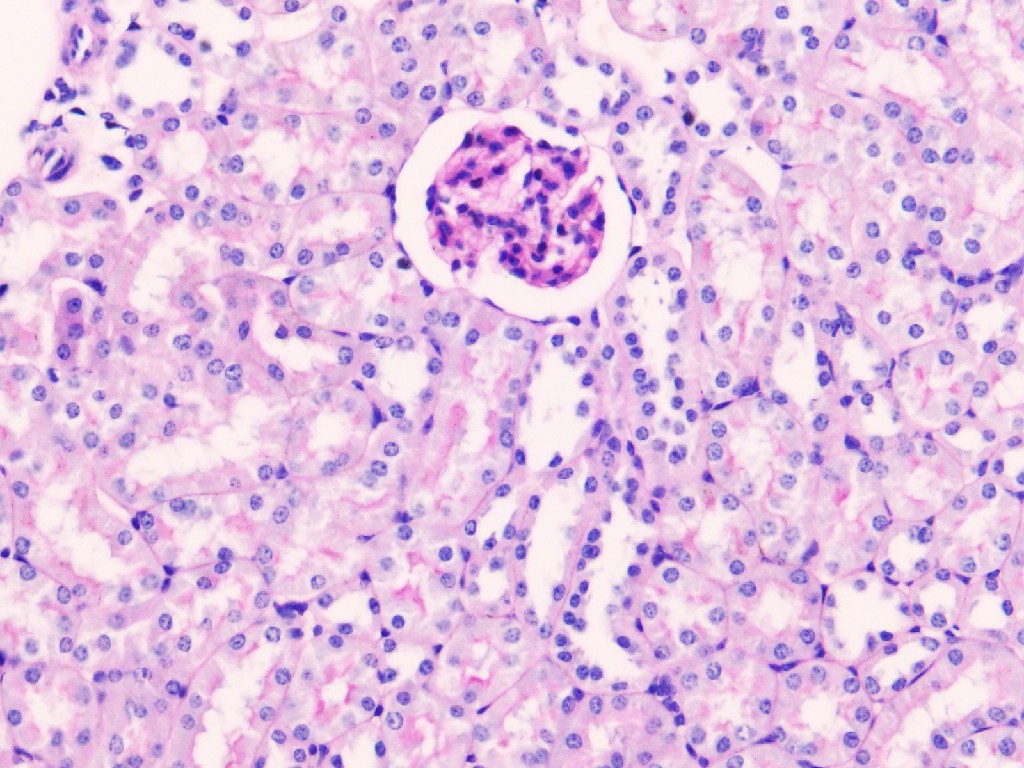

Supplement: Supplementary file 7 — Source data Fig. 6 [file 44321_2025_315_MOESM7_ESM.zip › Figure 6/F6A/3-PAS/4.jpg]

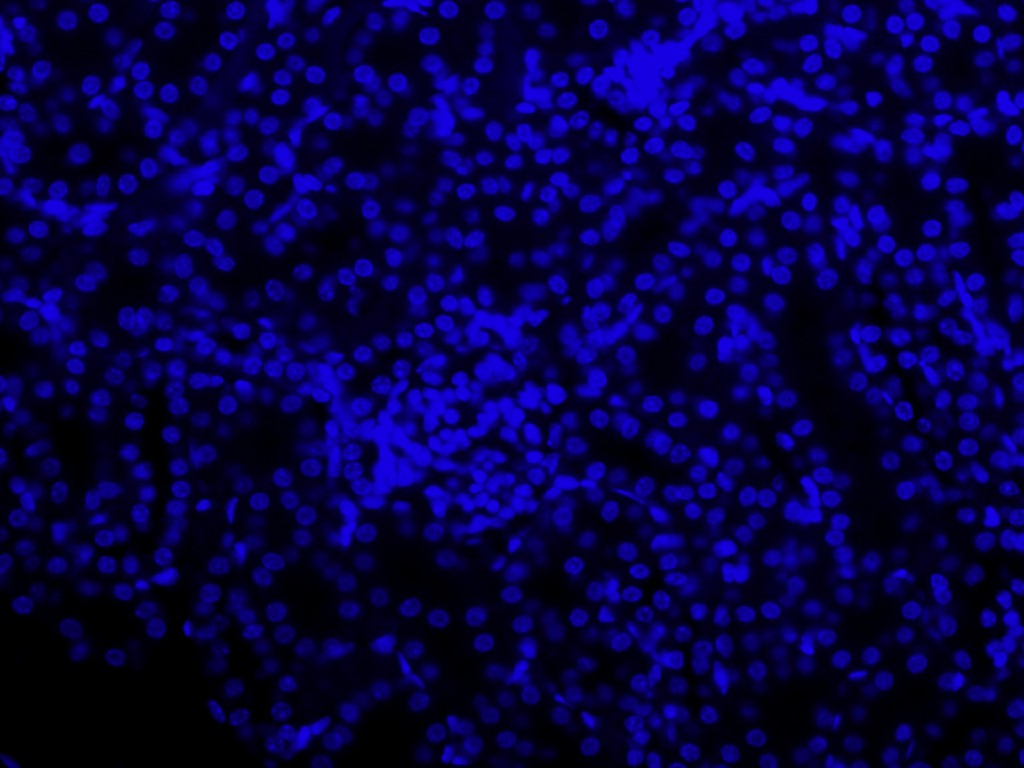

Supplement: Supplementary file 7 — Source data Fig. 6 [file 44321_2025_315_MOESM7_ESM.zip › Figure 6/F6B/1-GLDC-PDGFRbeta/1-1 (1).jpg]

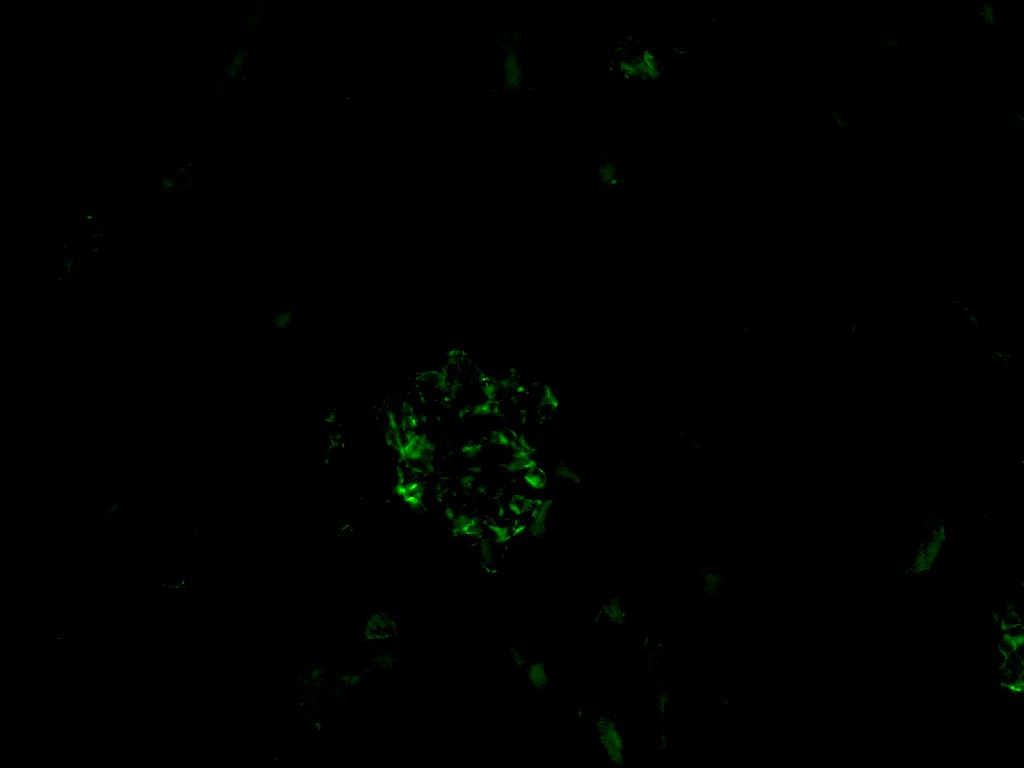

Supplement: Supplementary file 7 — Source data Fig. 6 [file 44321_2025_315_MOESM7_ESM.zip › Figure 6/F6B/1-GLDC-PDGFRbeta/1-1 (2).jpg]

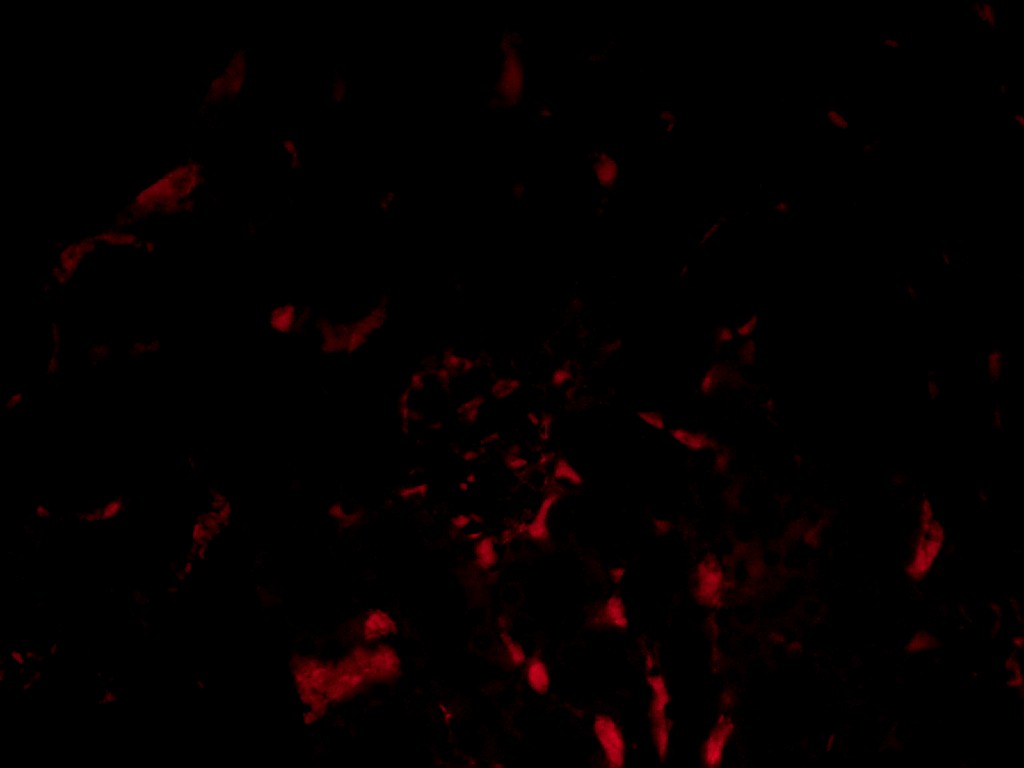

Supplement: Supplementary file 7 — Source data Fig. 6 [file 44321_2025_315_MOESM7_ESM.zip › Figure 6/F6B/1-GLDC-PDGFRbeta/1-1 (3).jpg]

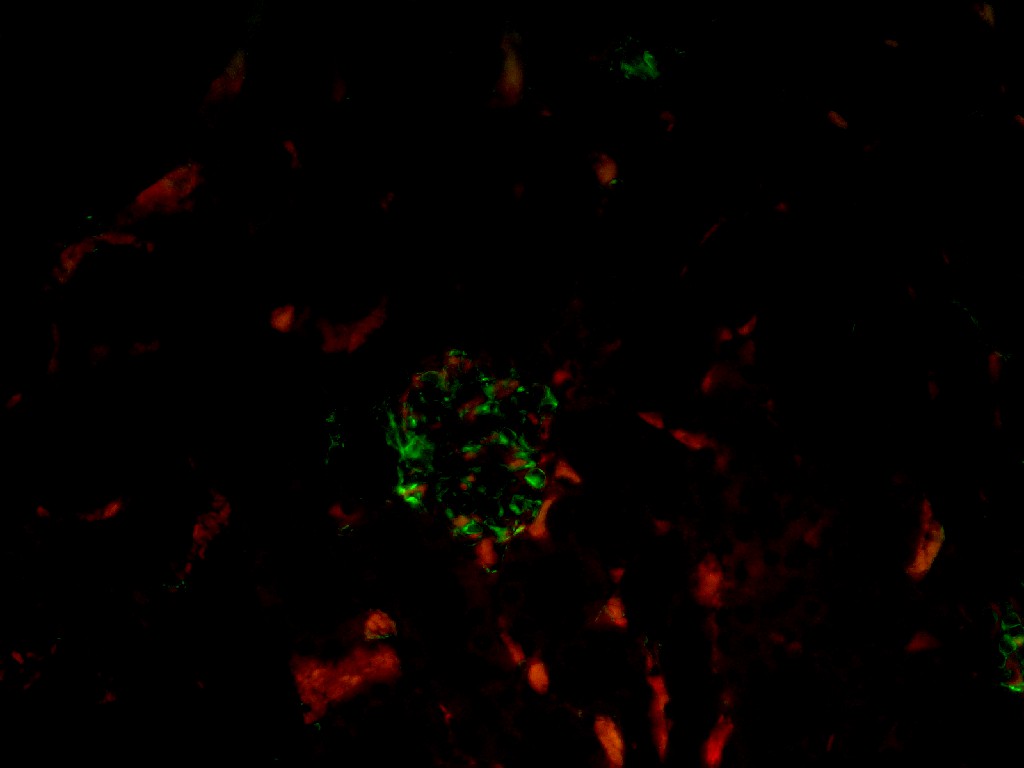

Supplement: Supplementary file 7 — Source data Fig. 6 [file 44321_2025_315_MOESM7_ESM.zip › Figure 6/F6B/1-GLDC-PDGFRbeta/1-1 (4).jpg]

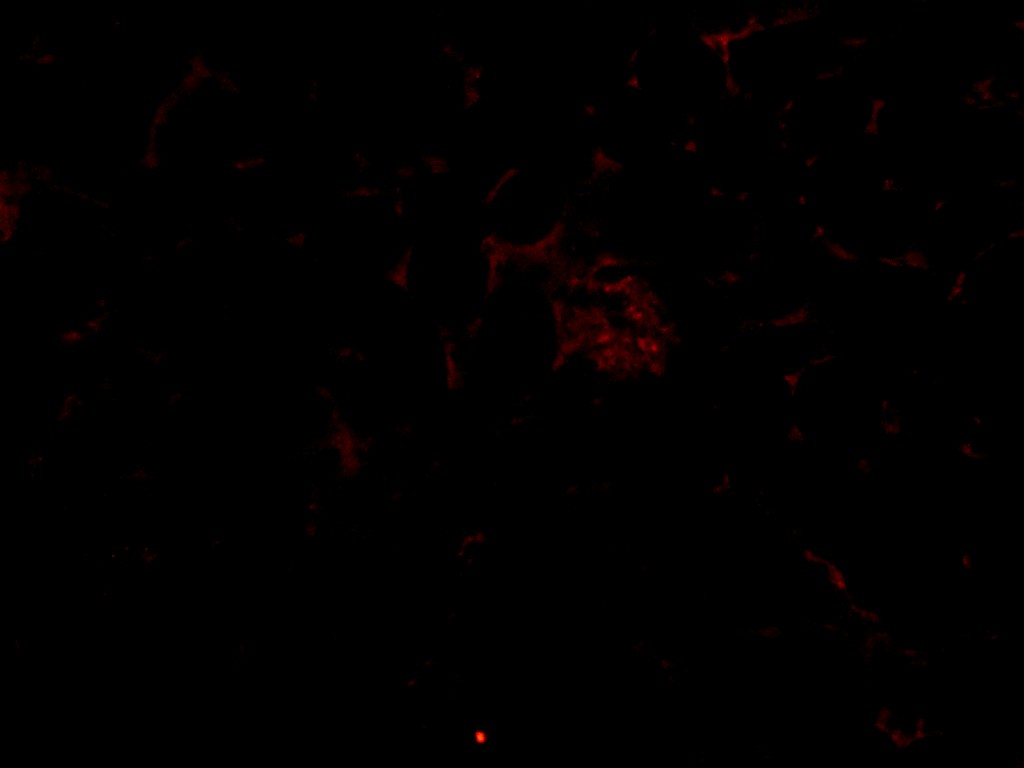

Supplement: Supplementary file 7 — Source data Fig. 6 [file 44321_2025_315_MOESM7_ESM.zip › Figure 6/F6B/1-GLDC-PDGFRbeta/1-2 (1).jpg]

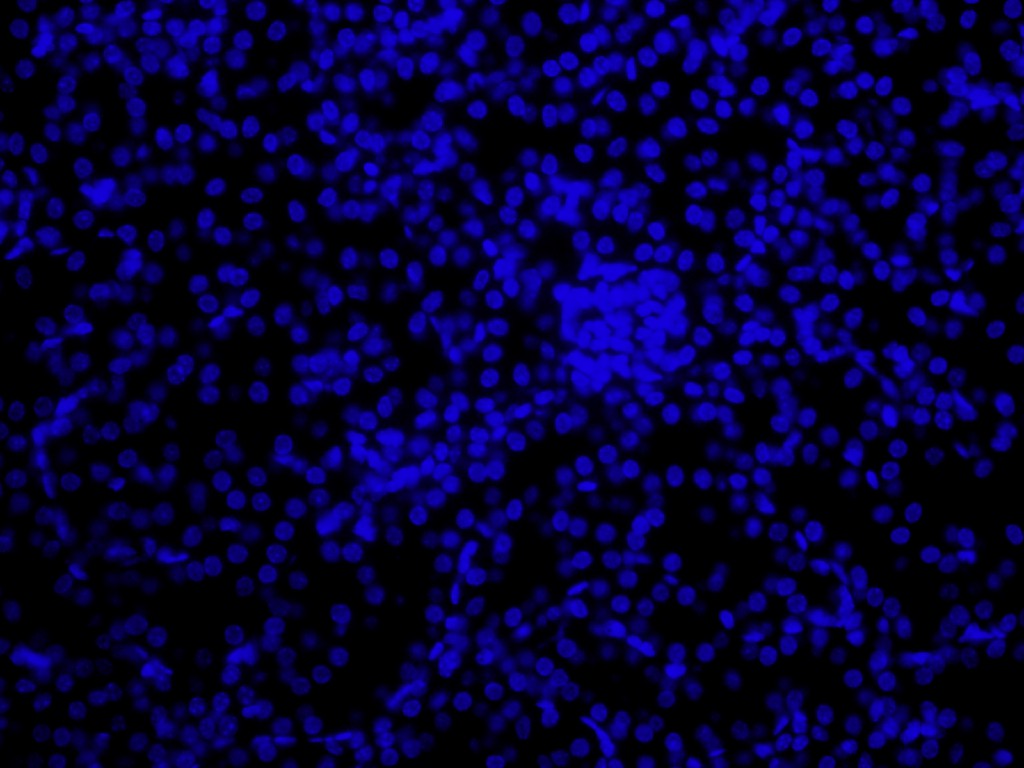

Supplement: Supplementary file 7 — Source data Fig. 6 [file 44321_2025_315_MOESM7_ESM.zip › Figure 6/F6B/1-GLDC-PDGFRbeta/1-2 (2).jpg]

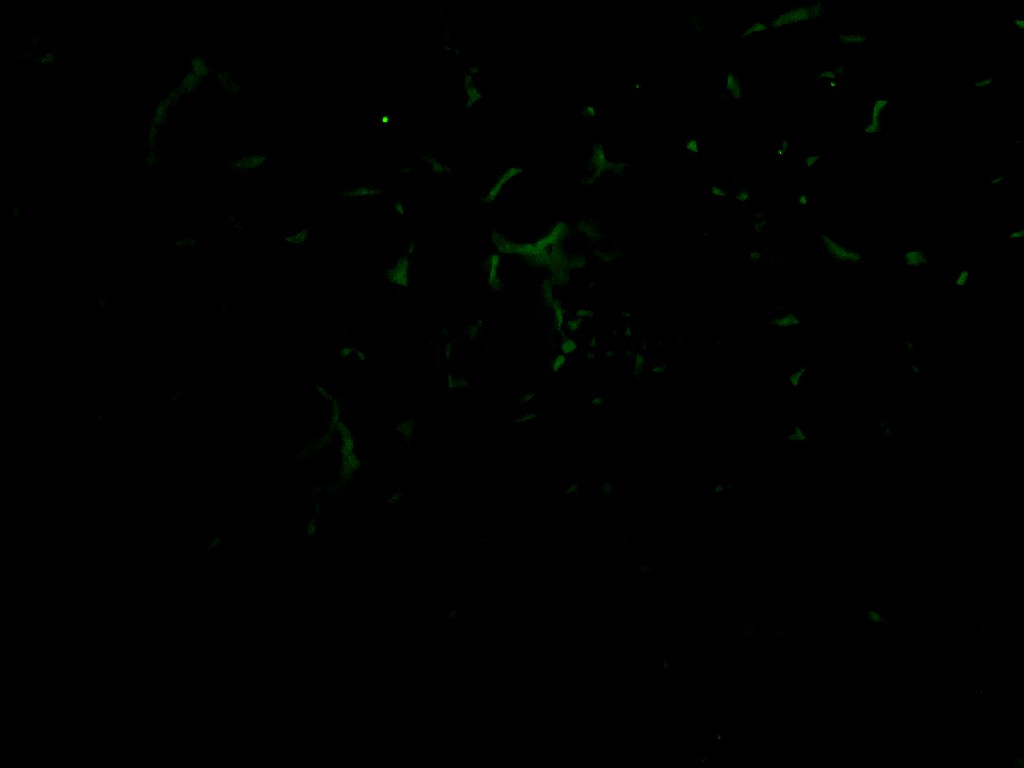

Supplement: Supplementary file 7 — Source data Fig. 6 [file 44321_2025_315_MOESM7_ESM.zip › Figure 6/F6B/1-GLDC-PDGFRbeta/1-2 (3).jpg]

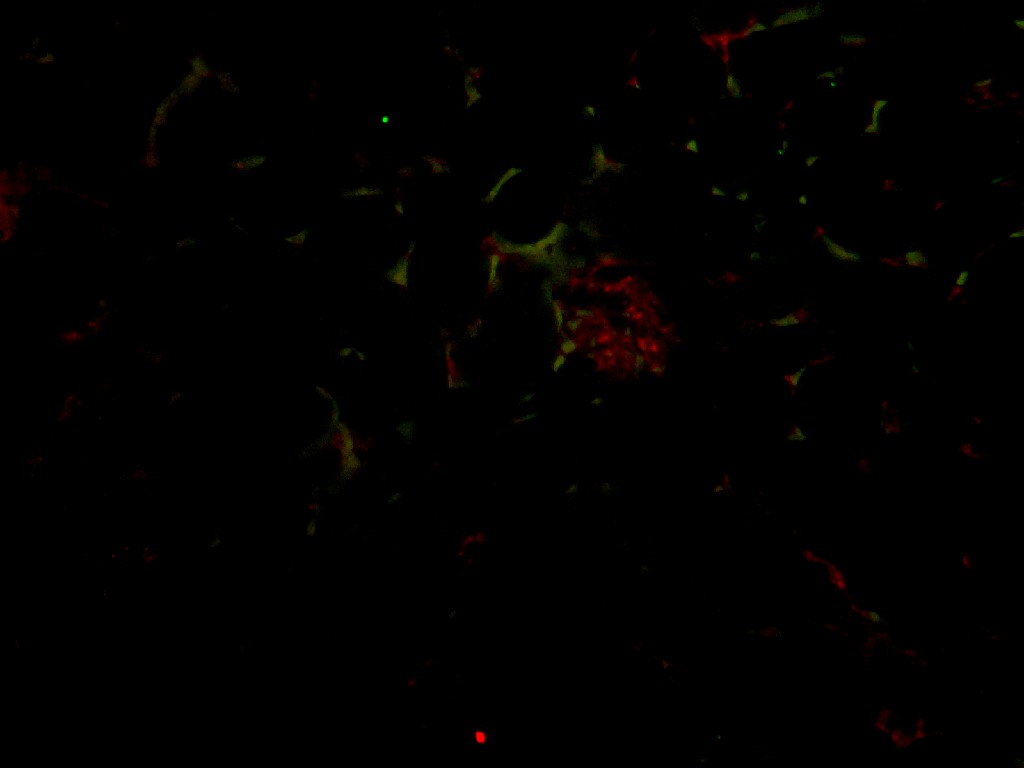

Supplement: Supplementary file 7 — Source data Fig. 6 [file 44321_2025_315_MOESM7_ESM.zip › Figure 6/F6B/1-GLDC-PDGFRbeta/1-2 (4).jpg]

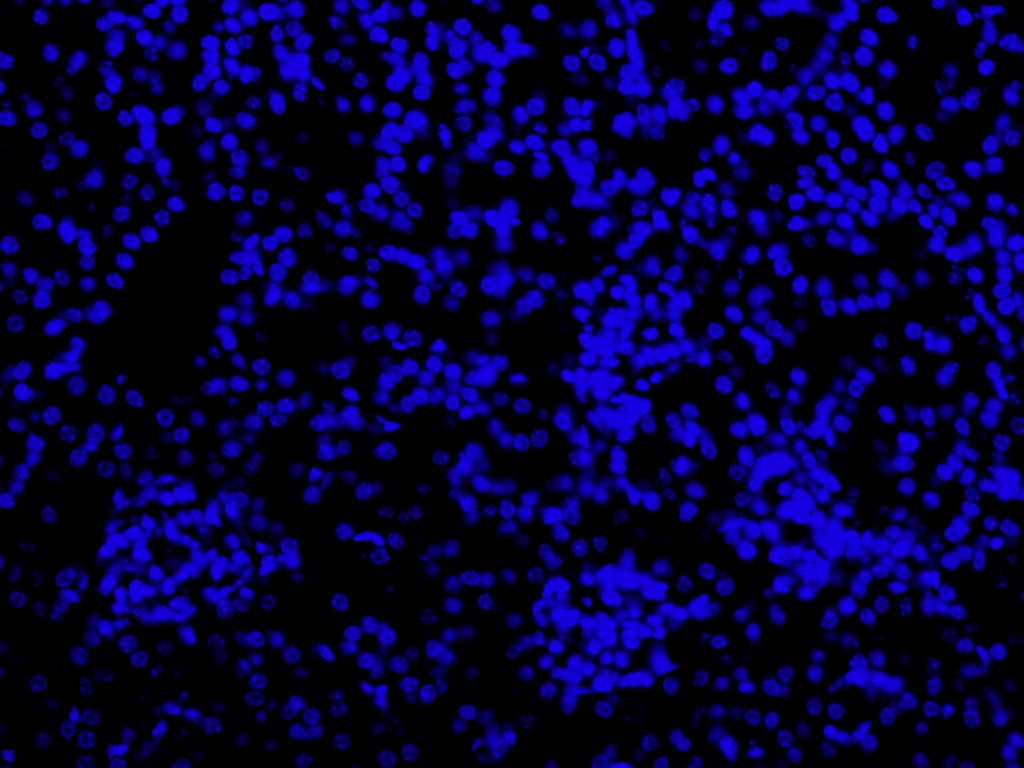

Supplement: Supplementary file 7 — Source data Fig. 6 [file 44321_2025_315_MOESM7_ESM.zip › Figure 6/F6B/1-GLDC-PDGFRbeta/2-1 (1).jpg]

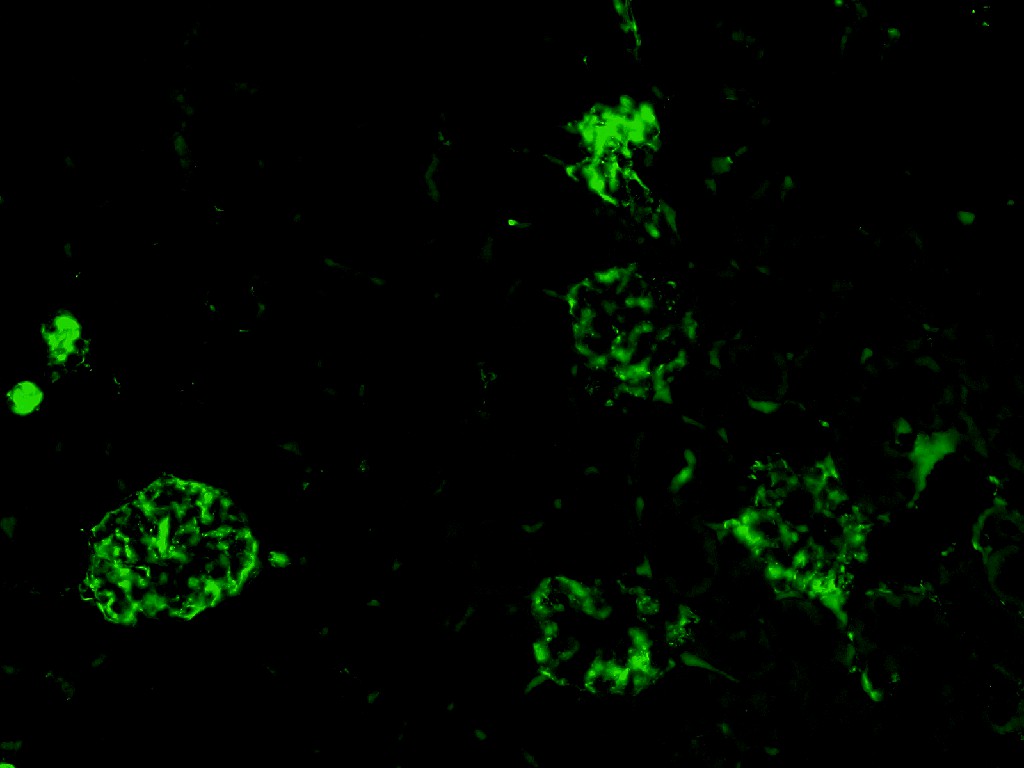

Supplement: Supplementary file 7 — Source data Fig. 6 [file 44321_2025_315_MOESM7_ESM.zip › Figure 6/F6B/1-GLDC-PDGFRbeta/2-1 (2).jpg]

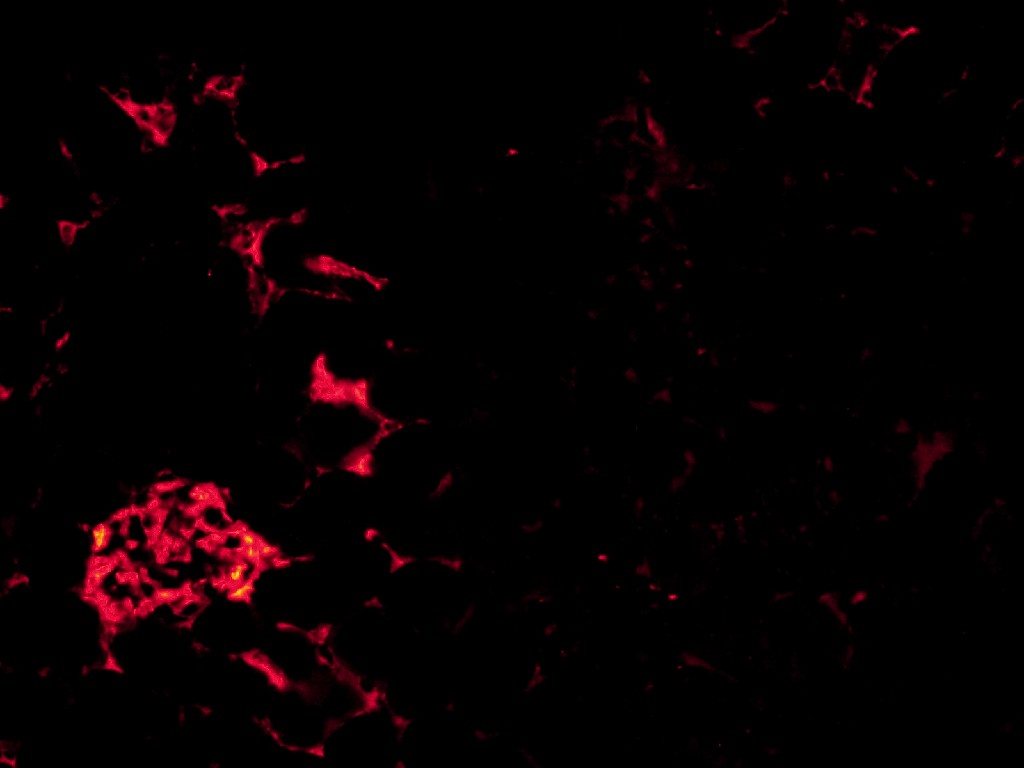

Supplement: Supplementary file 7 — Source data Fig. 6 [file 44321_2025_315_MOESM7_ESM.zip › Figure 6/F6B/1-GLDC-PDGFRbeta/2-1 (3).jpg]

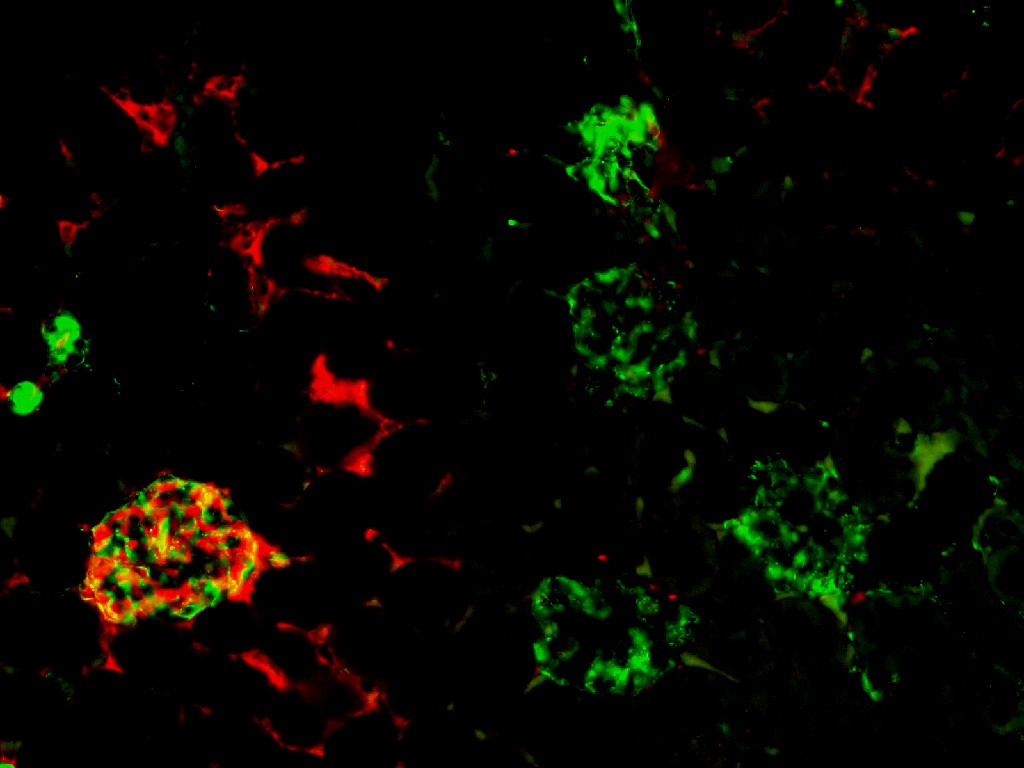

Supplement: Supplementary file 7 — Source data Fig. 6 [file 44321_2025_315_MOESM7_ESM.zip › Figure 6/F6B/1-GLDC-PDGFRbeta/2-1 (4).jpg]

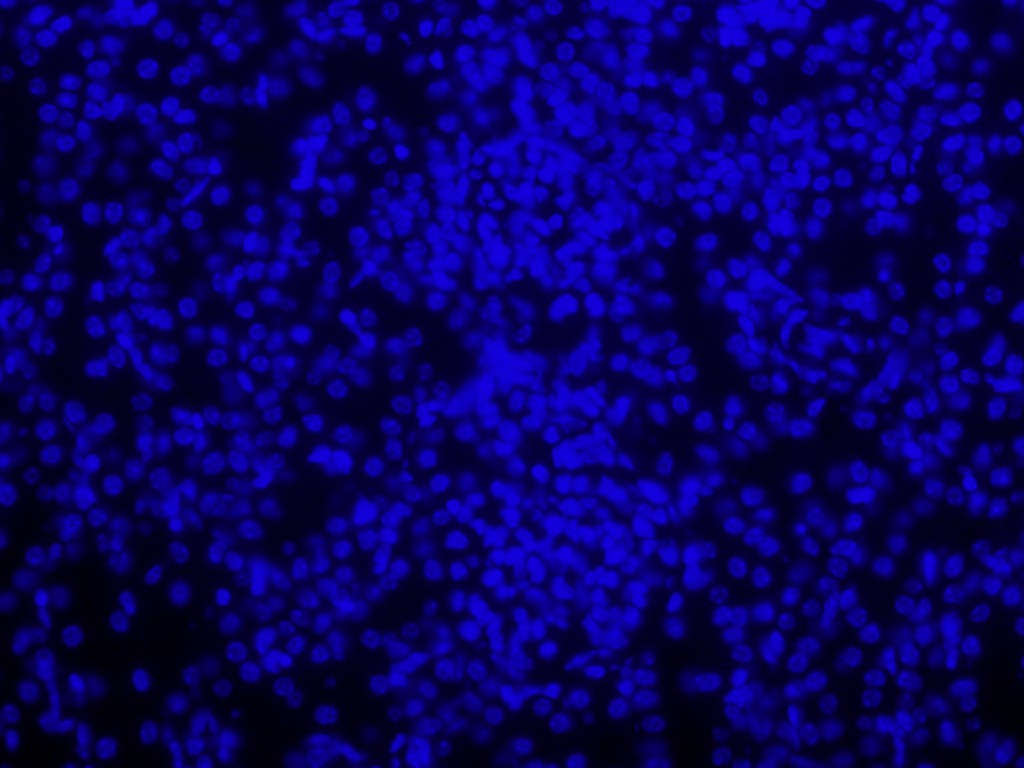

Supplement: Supplementary file 7 — Source data Fig. 6 [file 44321_2025_315_MOESM7_ESM.zip › Figure 6/F6B/1-GLDC-PDGFRbeta/2-2 (1).jpg]

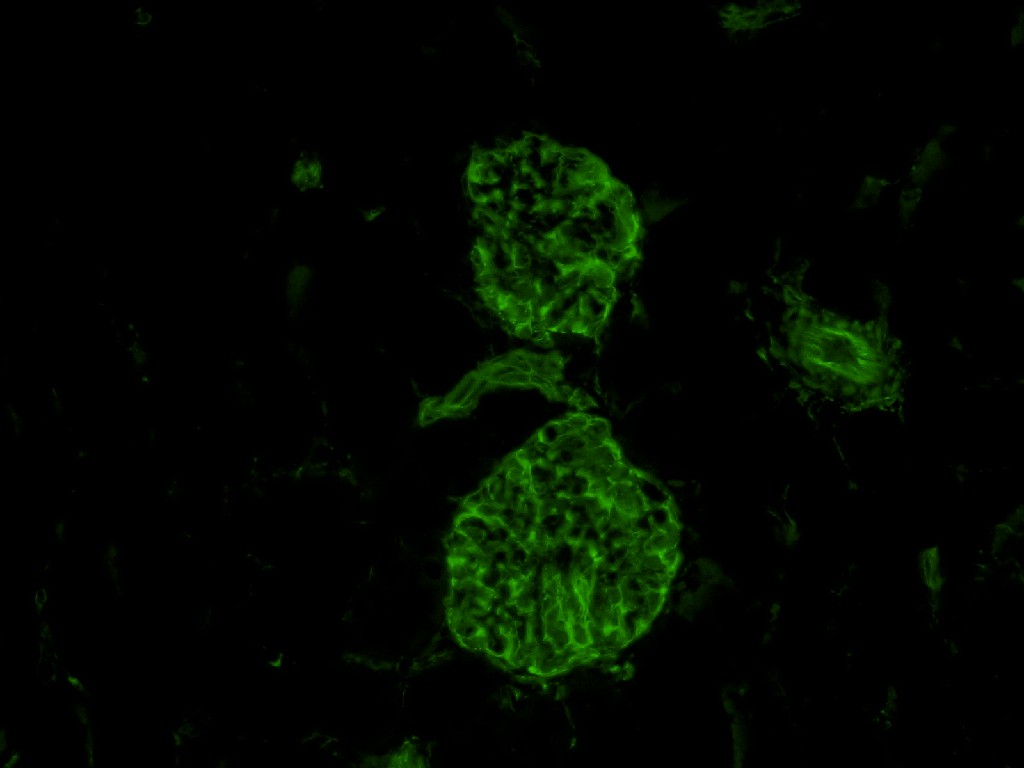

Supplement: Supplementary file 7 — Source data Fig. 6 [file 44321_2025_315_MOESM7_ESM.zip › Figure 6/F6B/1-GLDC-PDGFRbeta/2-2 (2).jpg]

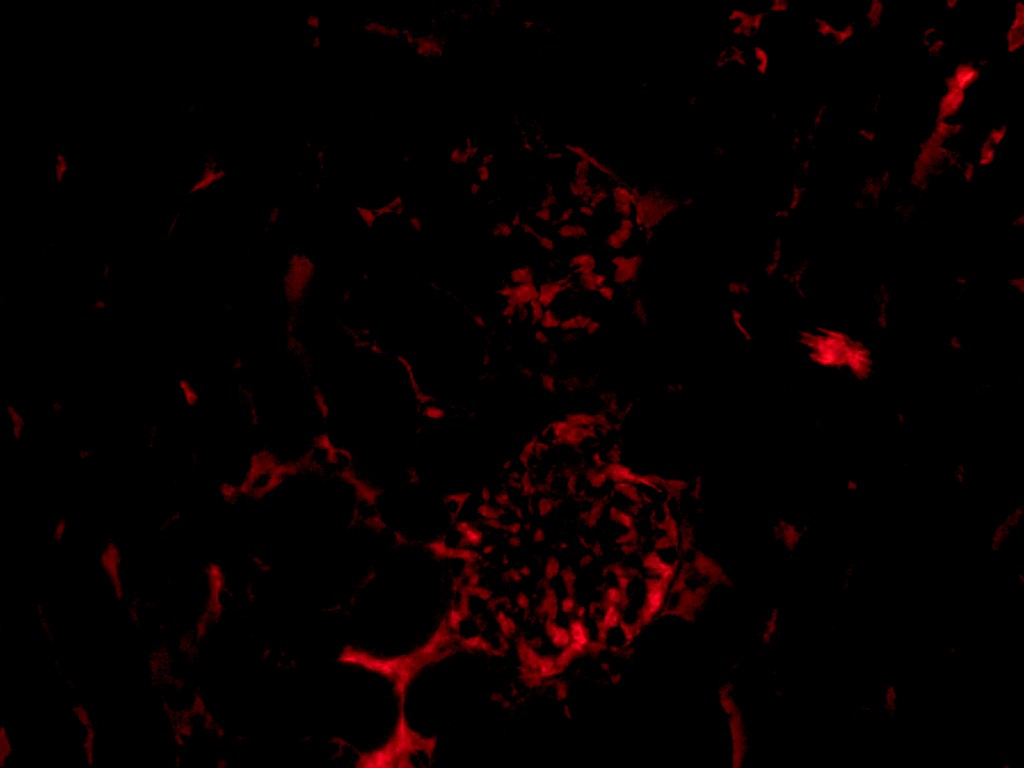

Supplement: Supplementary file 7 — Source data Fig. 6 [file 44321_2025_315_MOESM7_ESM.zip › Figure 6/F6B/1-GLDC-PDGFRbeta/2-2 (3).jpg]

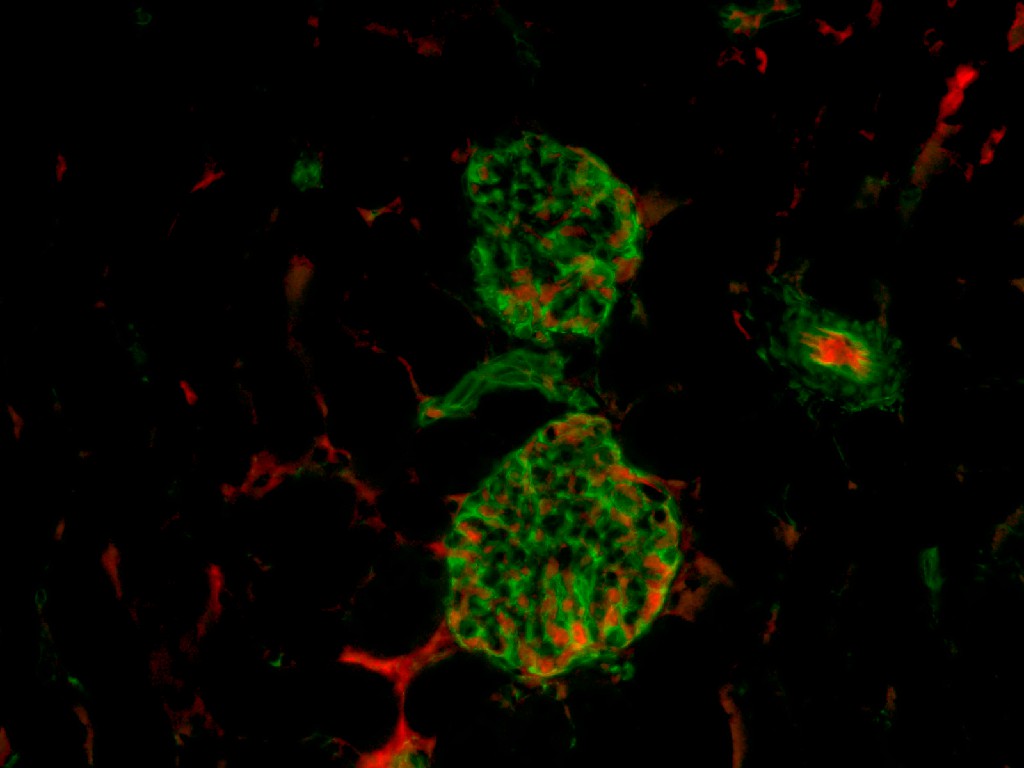

Supplement: Supplementary file 7 — Source data Fig. 6 [file 44321_2025_315_MOESM7_ESM.zip › Figure 6/F6B/1-GLDC-PDGFRbeta/2-2 (4).jpg]

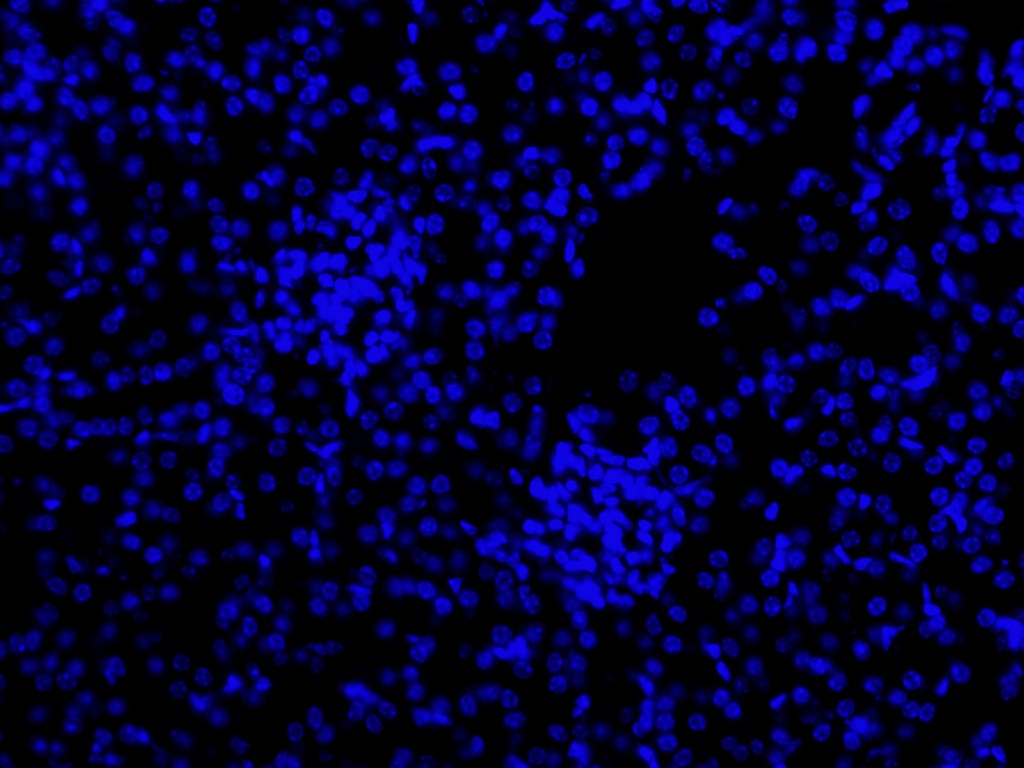

Supplement: Supplementary file 7 — Source data Fig. 6 [file 44321_2025_315_MOESM7_ESM.zip › Figure 6/F6B/1-GLDC-PDGFRbeta/3-1 (1).jpg]

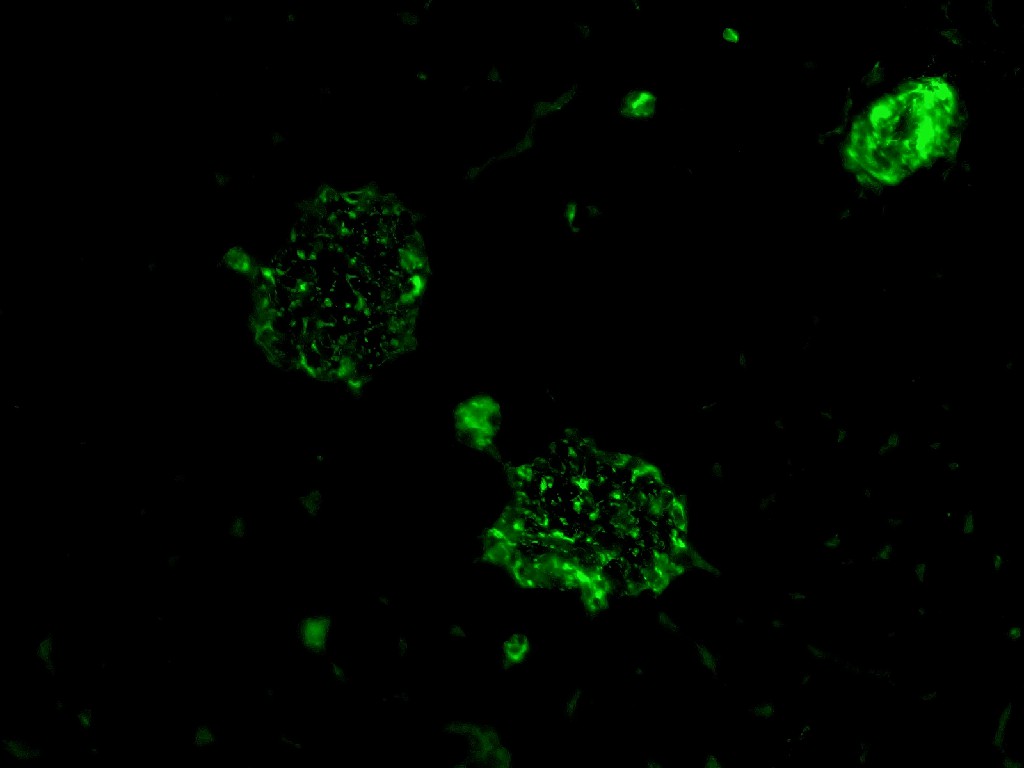

Supplement: Supplementary file 7 — Source data Fig. 6 [file 44321_2025_315_MOESM7_ESM.zip › Figure 6/F6B/1-GLDC-PDGFRbeta/3-1 (2).jpg]

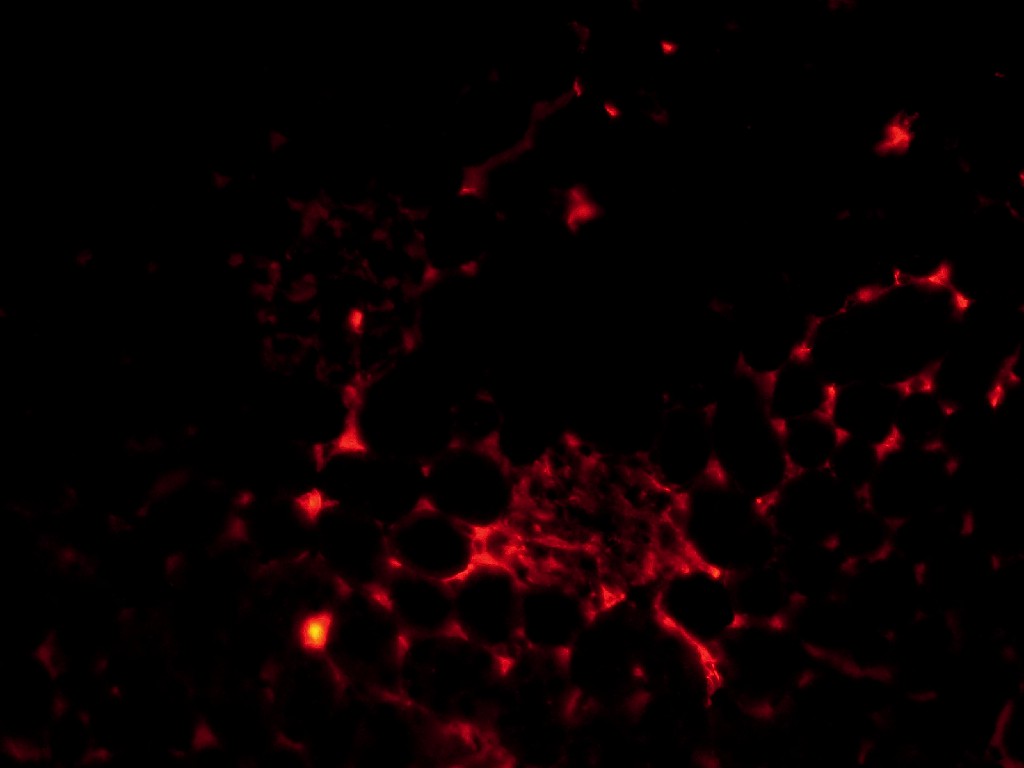

Supplement: Supplementary file 7 — Source data Fig. 6 [file 44321_2025_315_MOESM7_ESM.zip › Figure 6/F6B/1-GLDC-PDGFRbeta/3-1 (3).jpg]

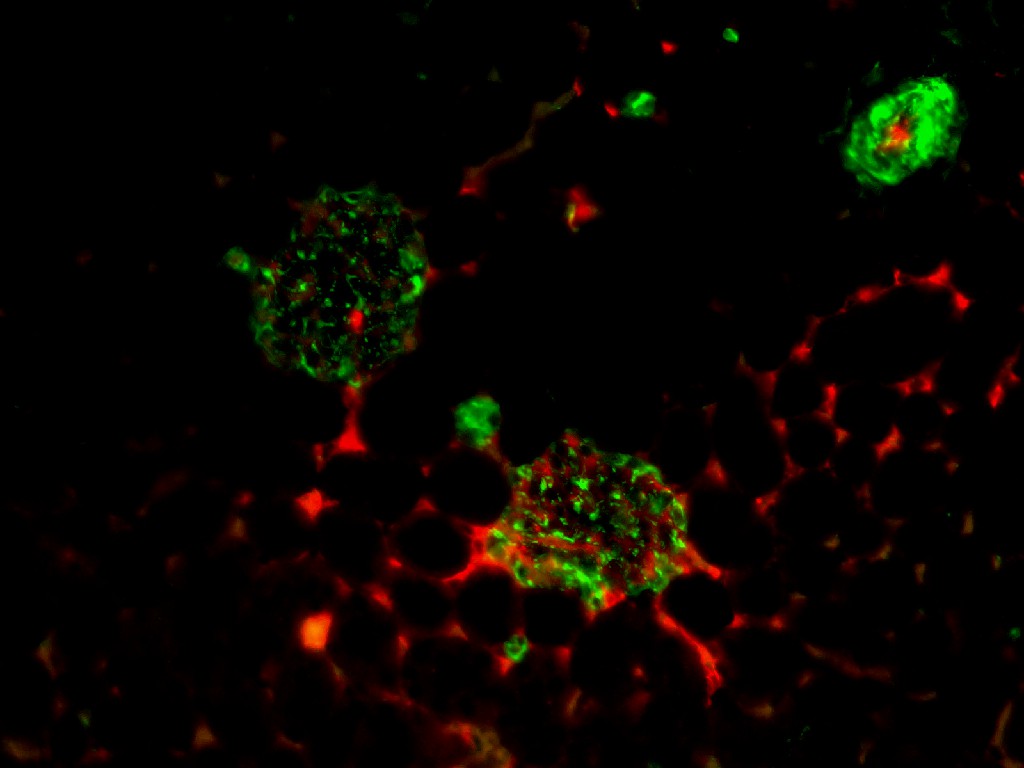

Supplement: Supplementary file 7 — Source data Fig. 6 [file 44321_2025_315_MOESM7_ESM.zip › Figure 6/F6B/1-GLDC-PDGFRbeta/3-1 (4).jpg]

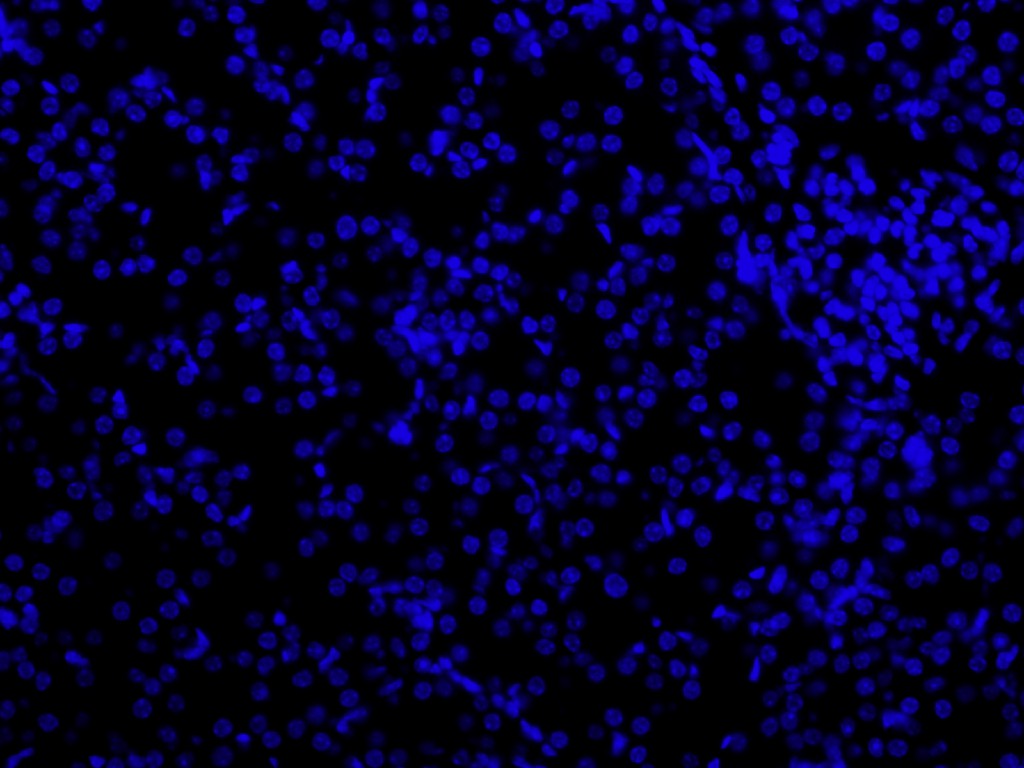

Supplement: Supplementary file 7 — Source data Fig. 6 [file 44321_2025_315_MOESM7_ESM.zip › Figure 6/F6B/1-GLDC-PDGFRbeta/3-2 (1).jpg]

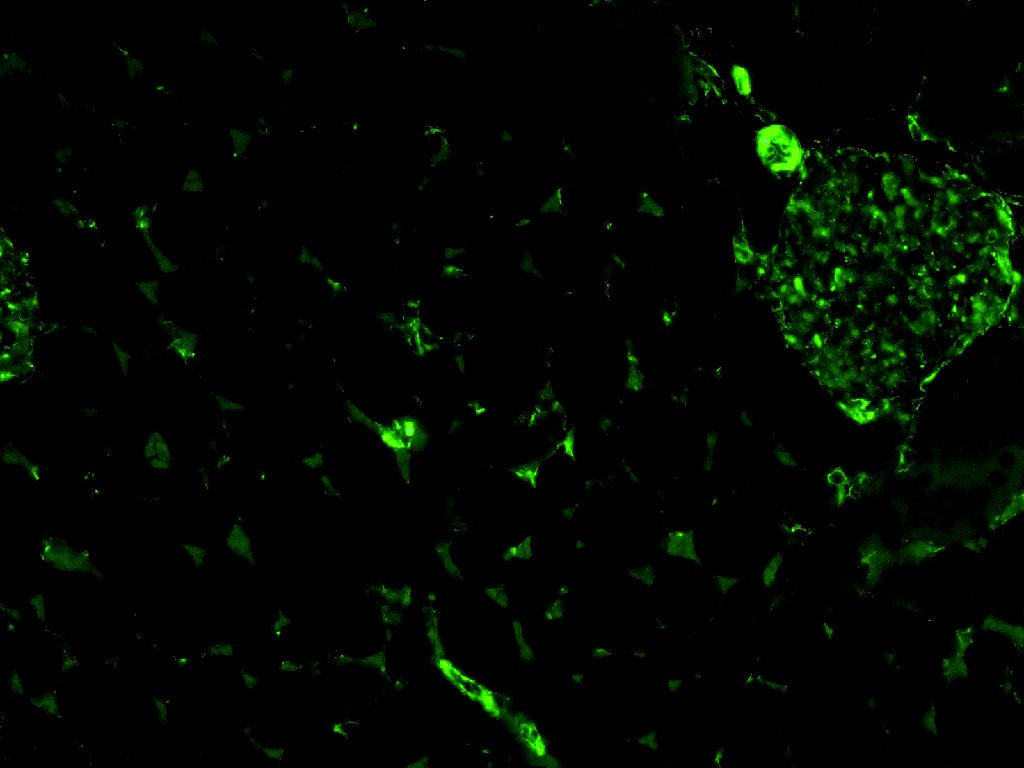

Supplement: Supplementary file 7 — Source data Fig. 6 [file 44321_2025_315_MOESM7_ESM.zip › Figure 6/F6B/1-GLDC-PDGFRbeta/3-2 (2).jpg]

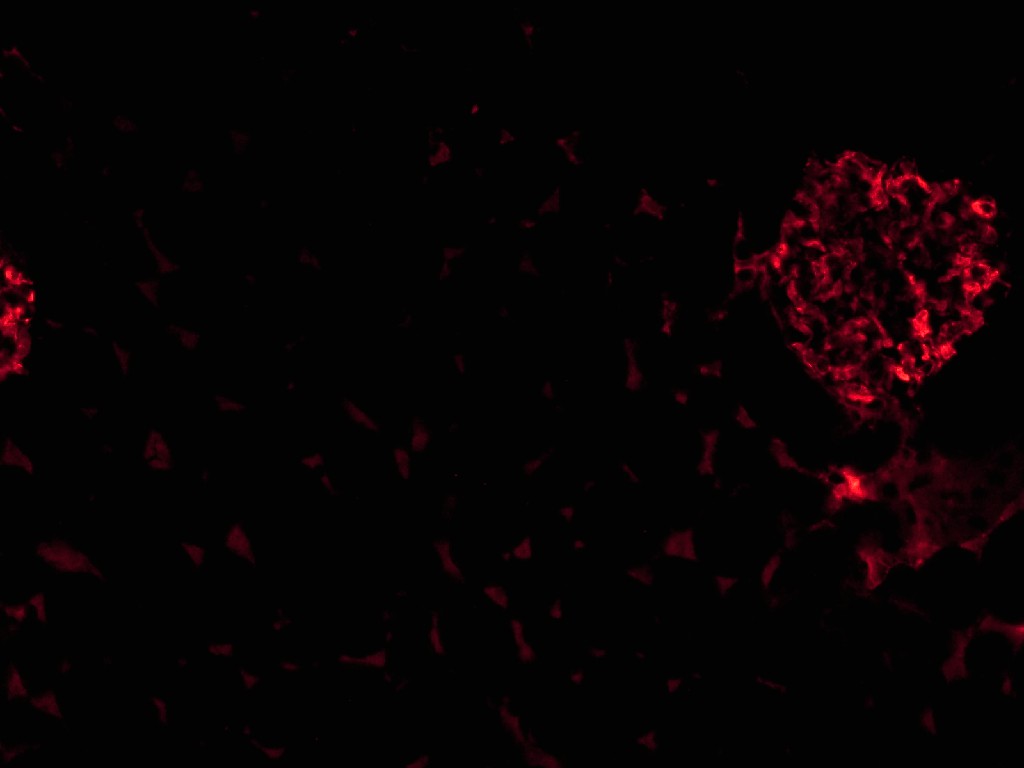

Supplement: Supplementary file 7 — Source data Fig. 6 [file 44321_2025_315_MOESM7_ESM.zip › Figure 6/F6B/1-GLDC-PDGFRbeta/3-2 (3).jpg]

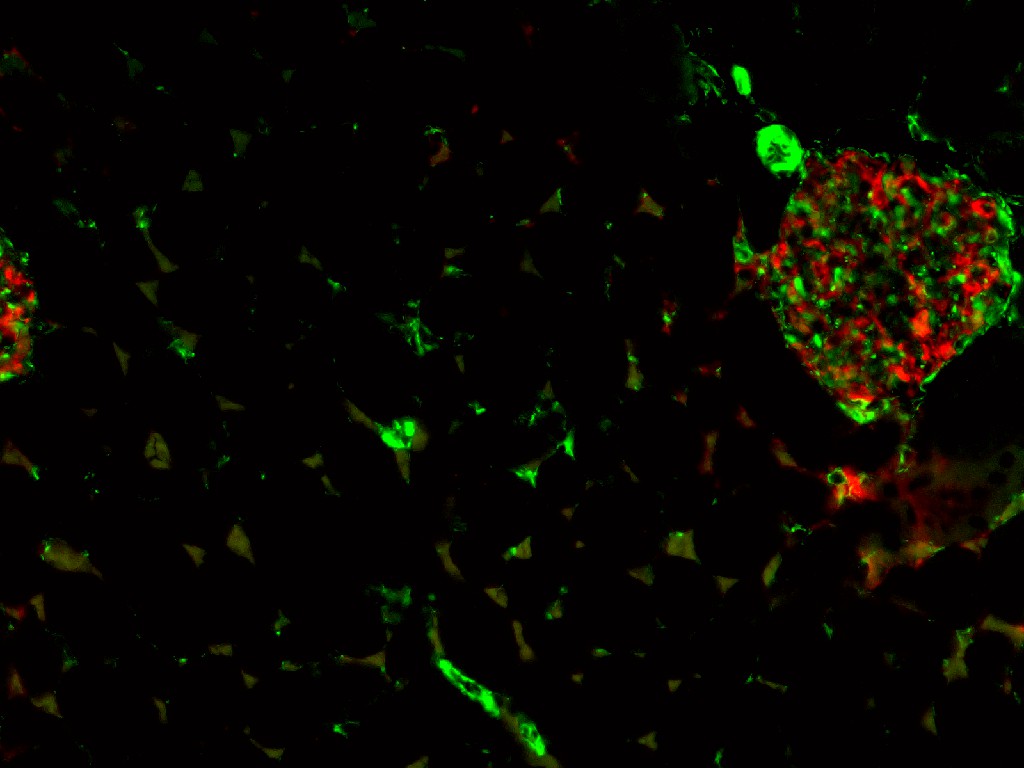

Supplement: Supplementary file 7 — Source data Fig. 6 [file 44321_2025_315_MOESM7_ESM.zip › Figure 6/F6B/1-GLDC-PDGFRbeta/3-2 (4).jpg]

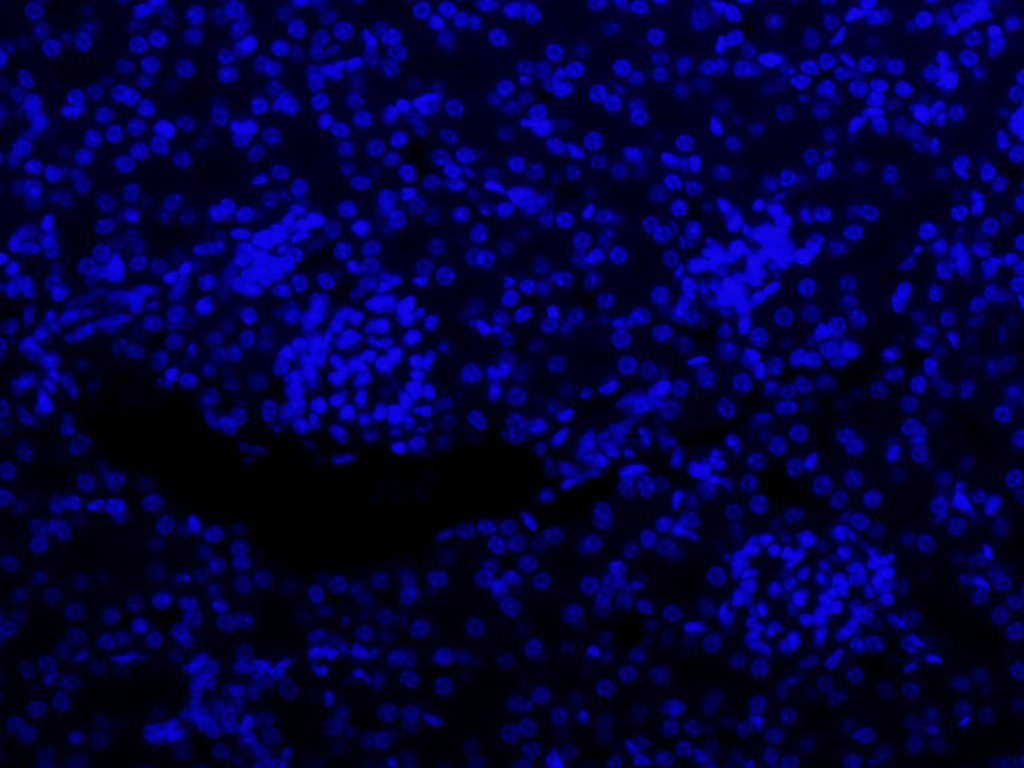

Supplement: Supplementary file 7 — Source data Fig. 6 [file 44321_2025_315_MOESM7_ESM.zip › Figure 6/F6B/1-GLDC-PDGFRbeta/4-1 (1).jpg]

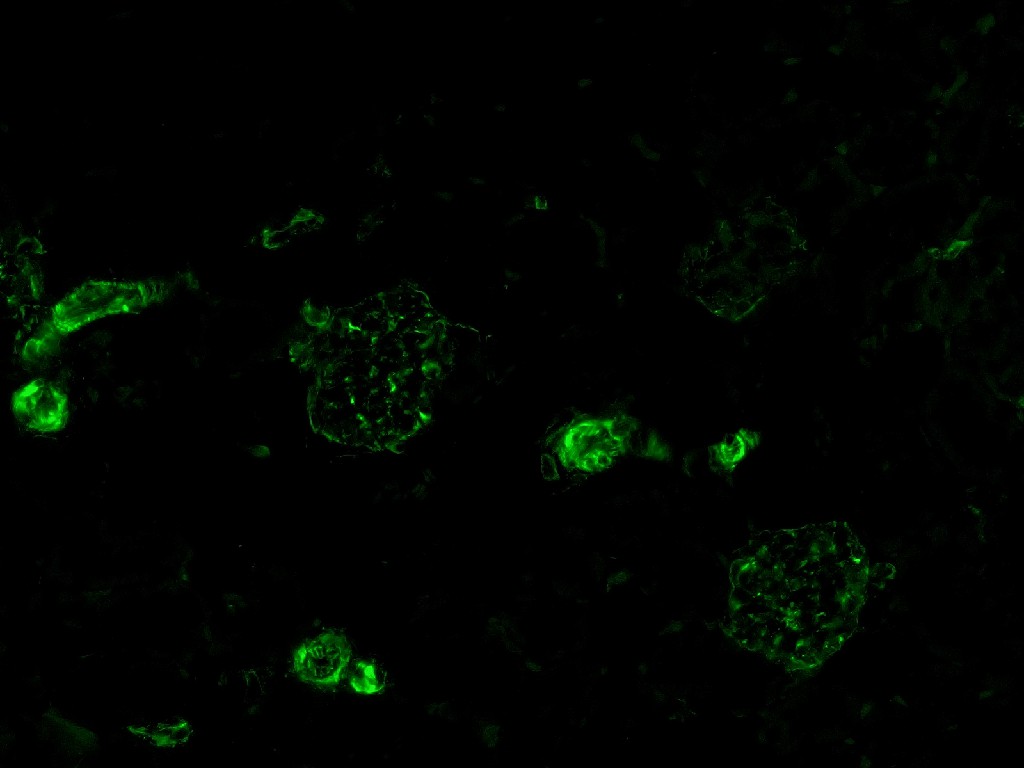

Supplement: Supplementary file 7 — Source data Fig. 6 [file 44321_2025_315_MOESM7_ESM.zip › Figure 6/F6B/1-GLDC-PDGFRbeta/4-1 (2).jpg]

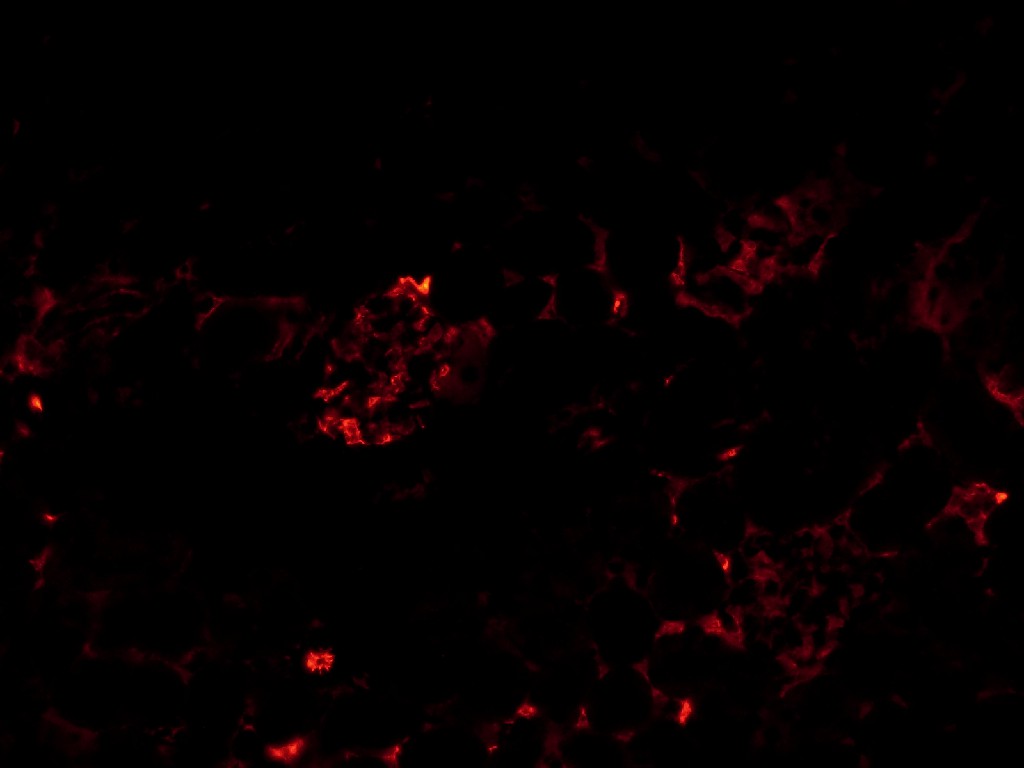

Supplement: Supplementary file 7 — Source data Fig. 6 [file 44321_2025_315_MOESM7_ESM.zip › Figure 6/F6B/1-GLDC-PDGFRbeta/4-1 (3).jpg]

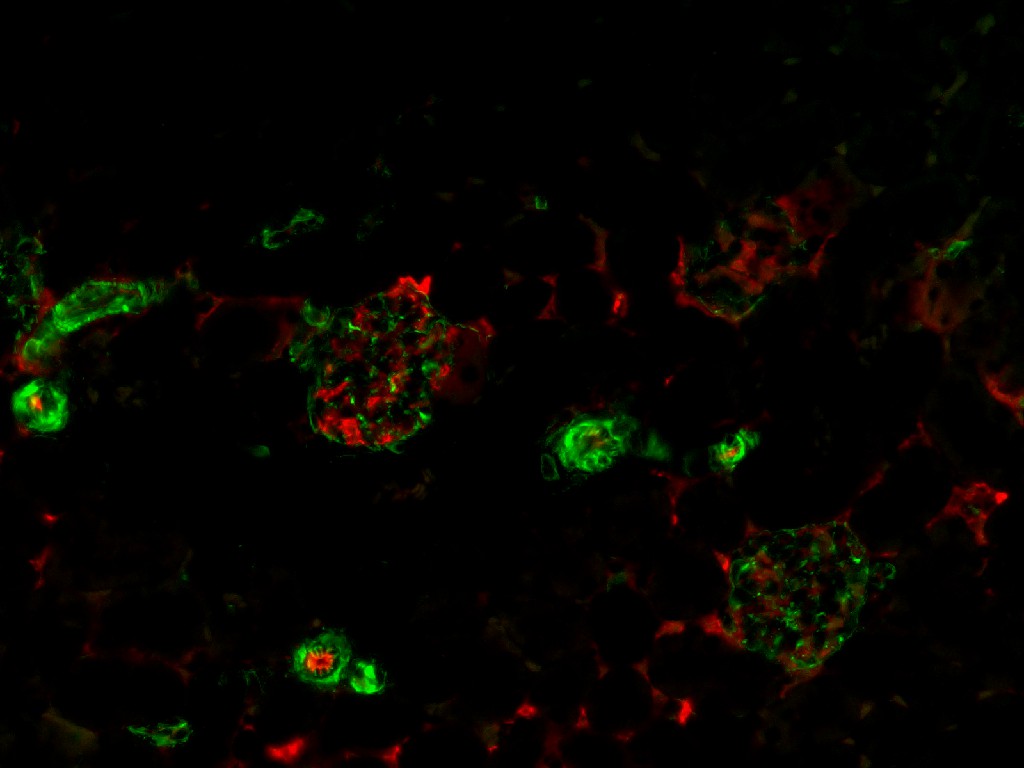

Supplement: Supplementary file 7 — Source data Fig. 6 [file 44321_2025_315_MOESM7_ESM.zip › Figure 6/F6B/1-GLDC-PDGFRbeta/4-1 (4).jpg]

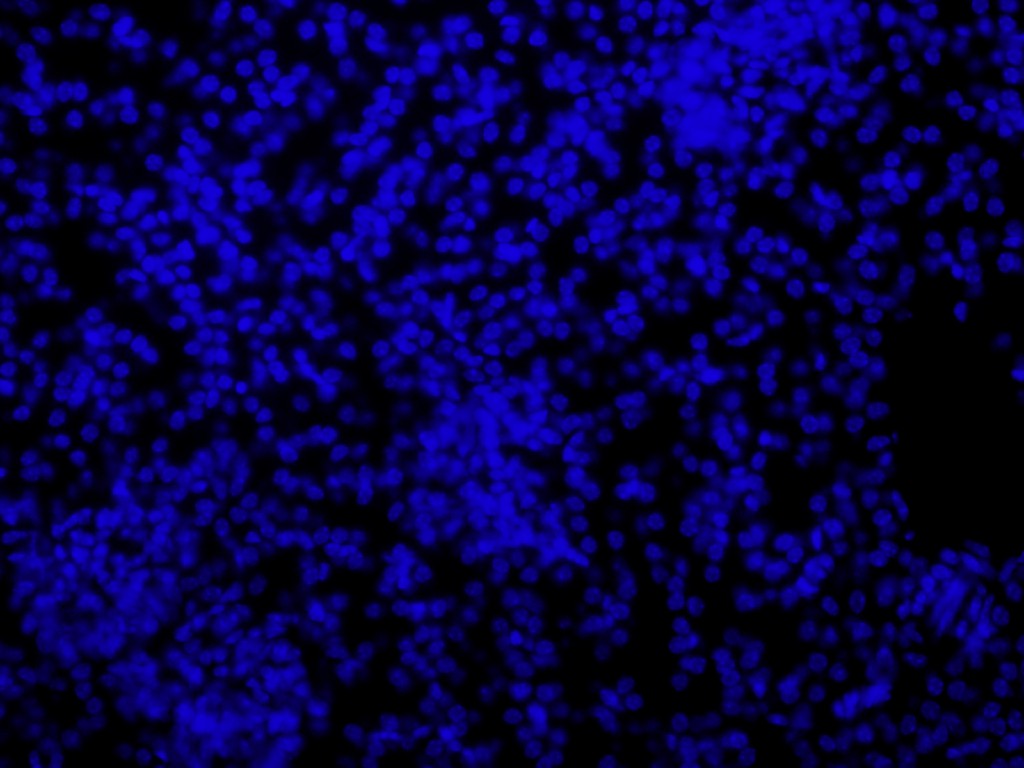

Supplement: Supplementary file 7 — Source data Fig. 6 [file 44321_2025_315_MOESM7_ESM.zip › Figure 6/F6B/1-GLDC-PDGFRbeta/4-2 (1).jpg]

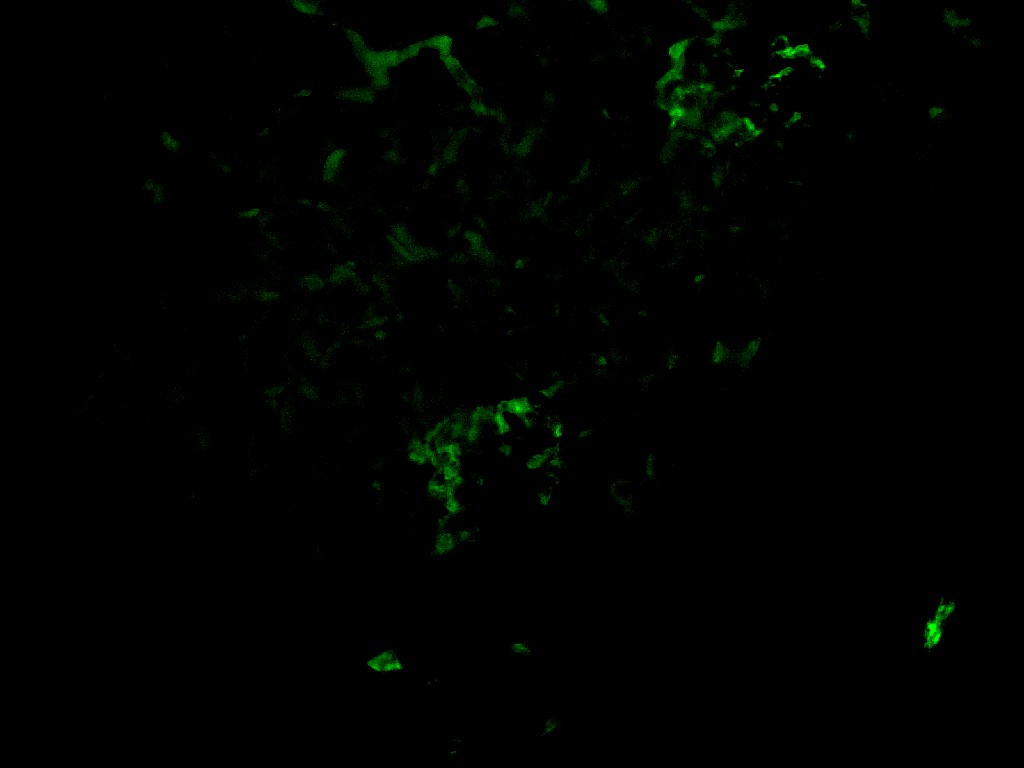

Supplement: Supplementary file 7 — Source data Fig. 6 [file 44321_2025_315_MOESM7_ESM.zip › Figure 6/F6B/1-GLDC-PDGFRbeta/4-2 (2).jpg]

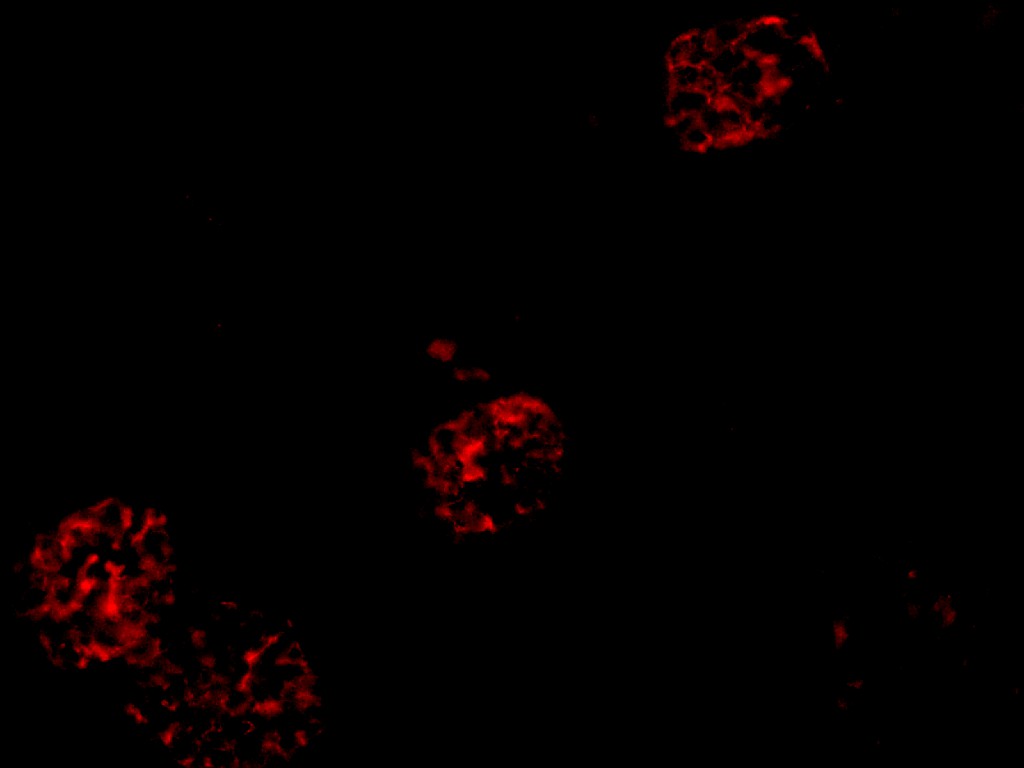

Supplement: Supplementary file 7 — Source data Fig. 6 [file 44321_2025_315_MOESM7_ESM.zip › Figure 6/F6B/1-GLDC-PDGFRbeta/4-2 (3).jpg]

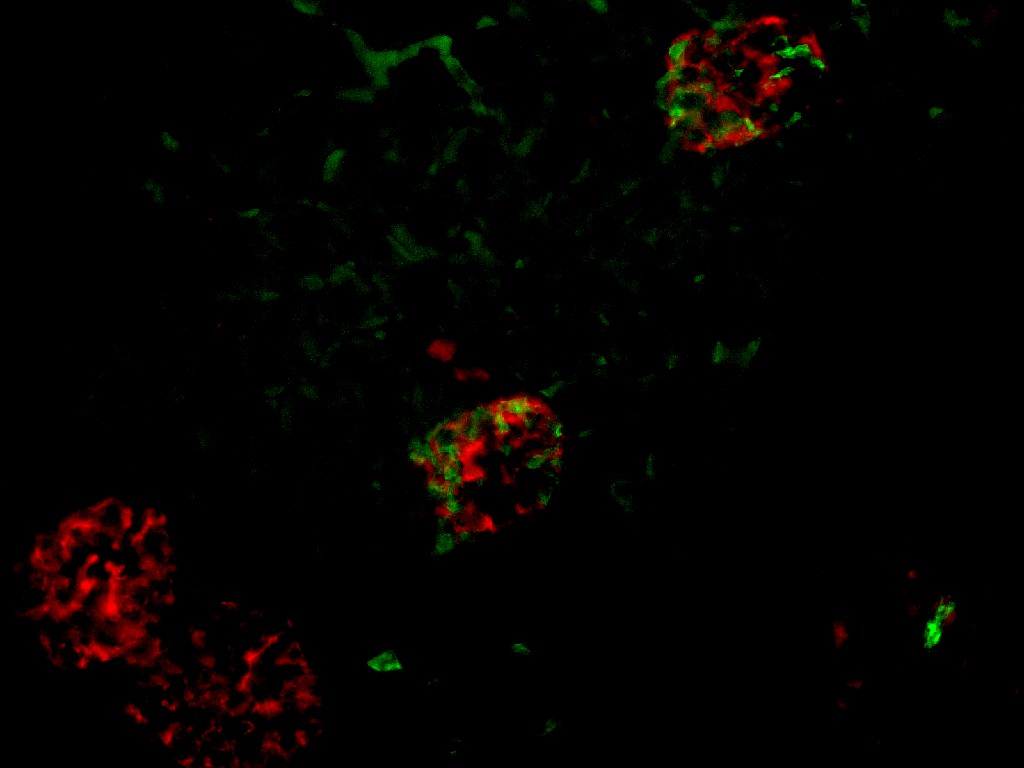

Supplement: Supplementary file 7 — Source data Fig. 6 [file 44321_2025_315_MOESM7_ESM.zip › Figure 6/F6B/1-GLDC-PDGFRbeta/4-2 (4).jpg]

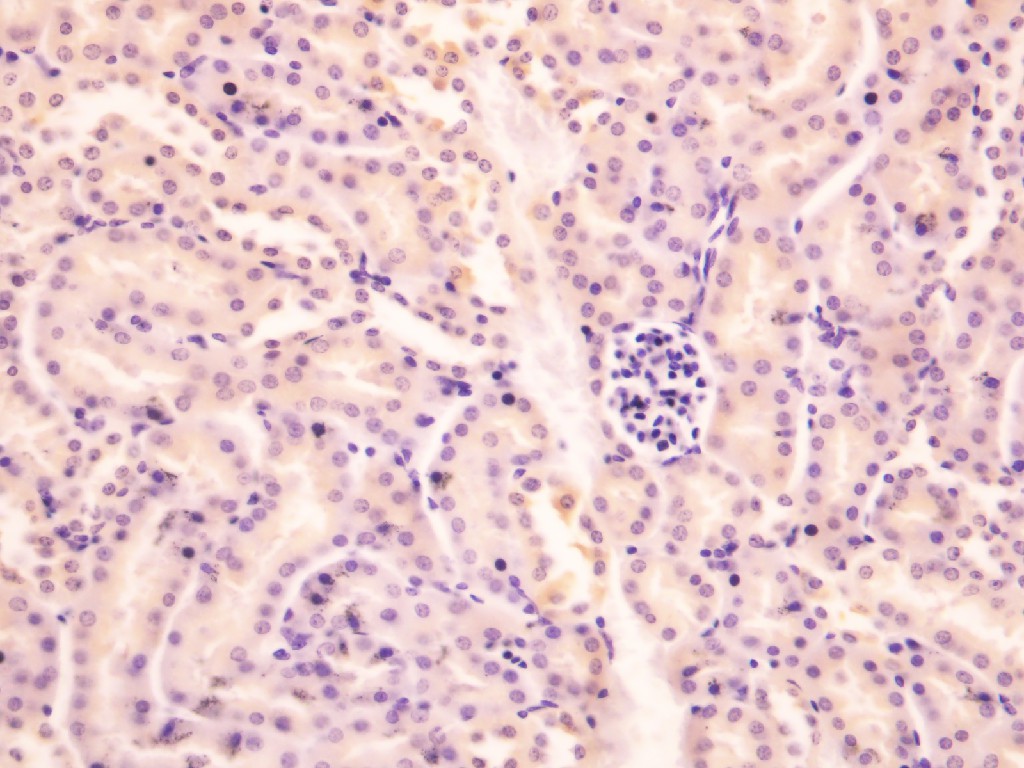

Supplement: Supplementary file 7 — Source data Fig. 6 [file 44321_2025_315_MOESM7_ESM.zip › Figure 6/F6B/2-CD68/1 (1).jpg]

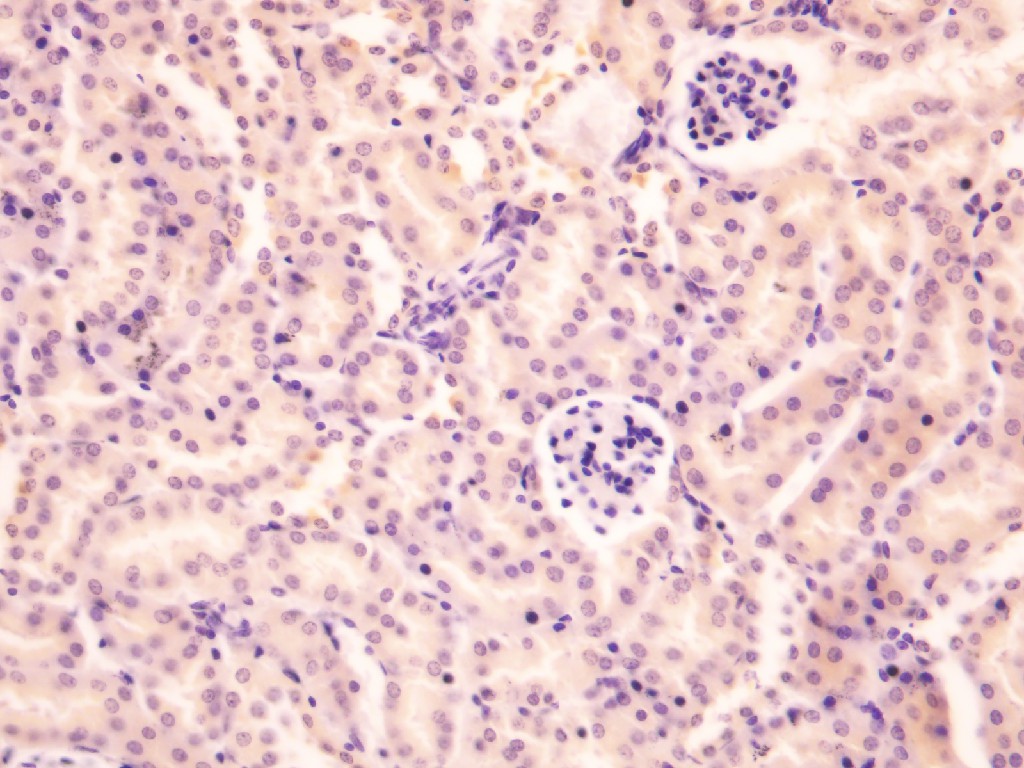

Supplement: Supplementary file 7 — Source data Fig. 6 [file 44321_2025_315_MOESM7_ESM.zip › Figure 6/F6B/2-CD68/1 (2).jpg]

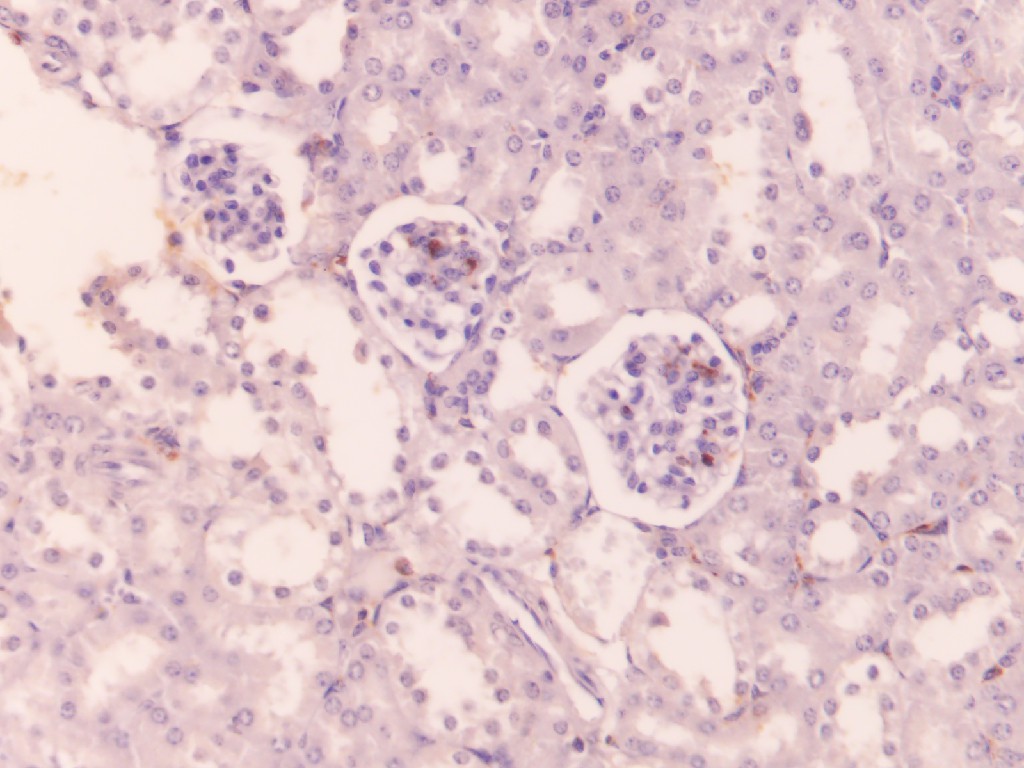

Supplement: Supplementary file 7 — Source data Fig. 6 [file 44321_2025_315_MOESM7_ESM.zip › Figure 6/F6B/2-CD68/2 (1).jpg]

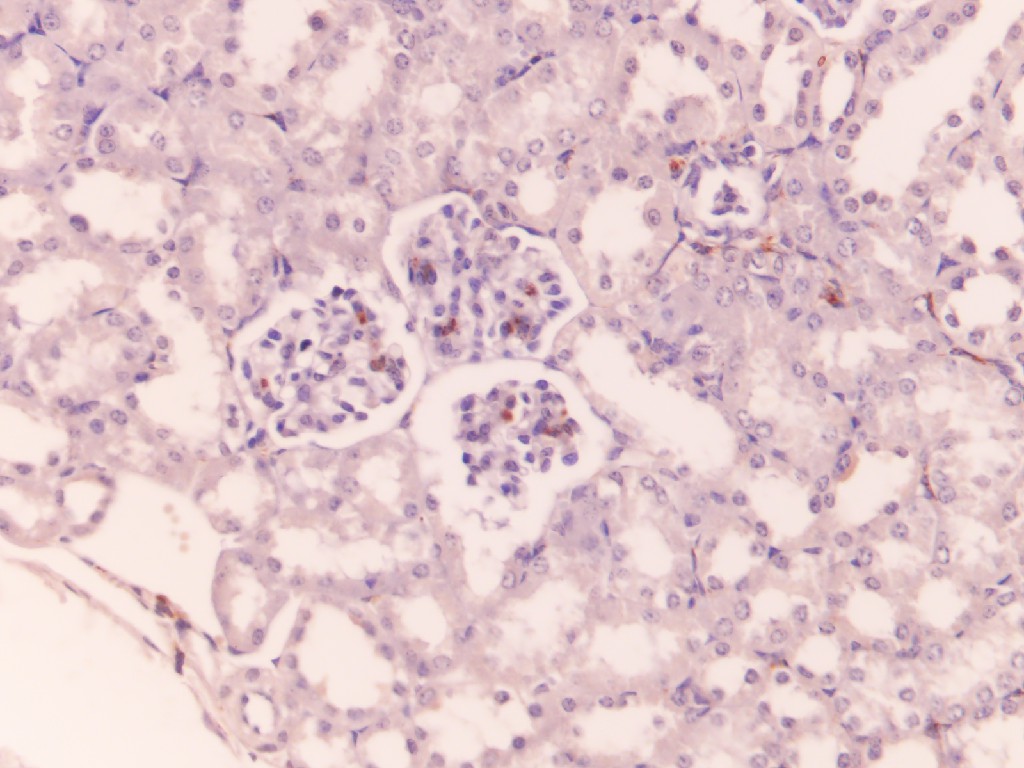

Supplement: Supplementary file 7 — Source data Fig. 6 [file 44321_2025_315_MOESM7_ESM.zip › Figure 6/F6B/2-CD68/2 (2).jpg]

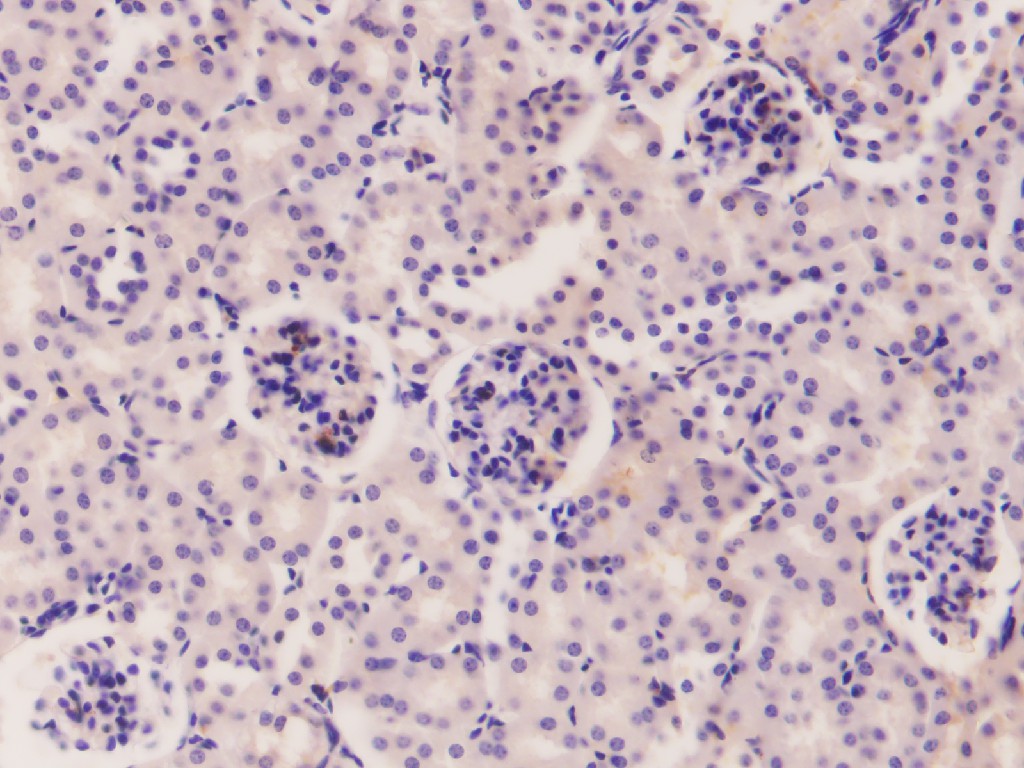

Supplement: Supplementary file 7 — Source data Fig. 6 [file 44321_2025_315_MOESM7_ESM.zip › Figure 6/F6B/2-CD68/3 (1).jpg]

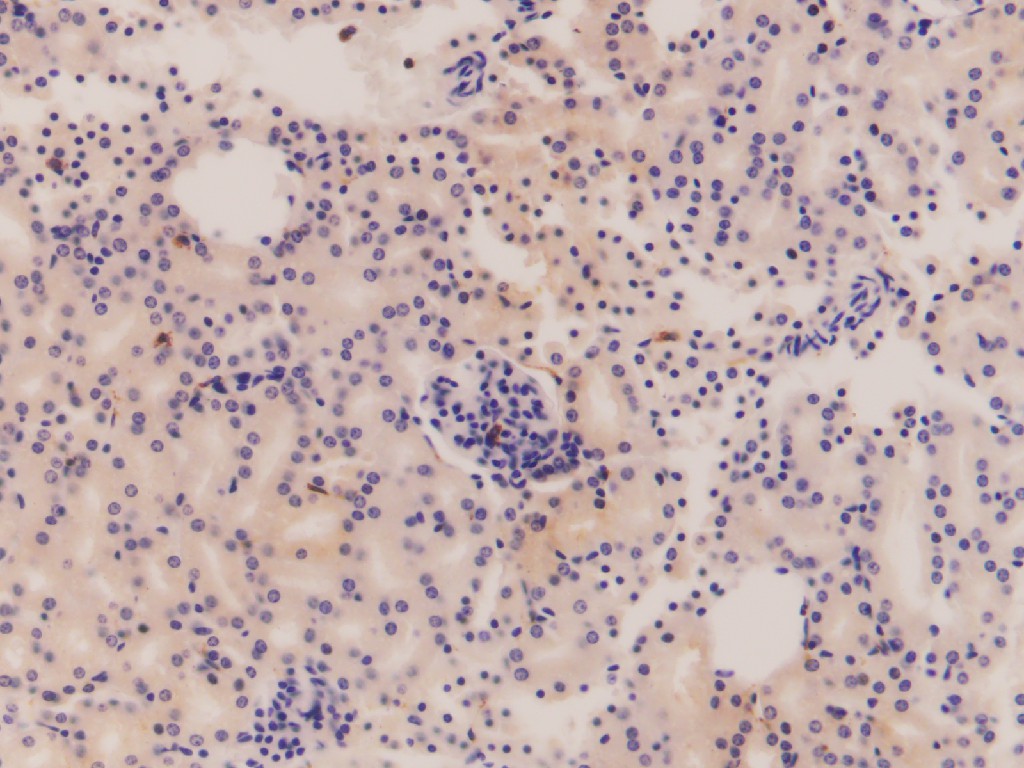

Supplement: Supplementary file 7 — Source data Fig. 6 [file 44321_2025_315_MOESM7_ESM.zip › Figure 6/F6B/2-CD68/3 (2).jpg]

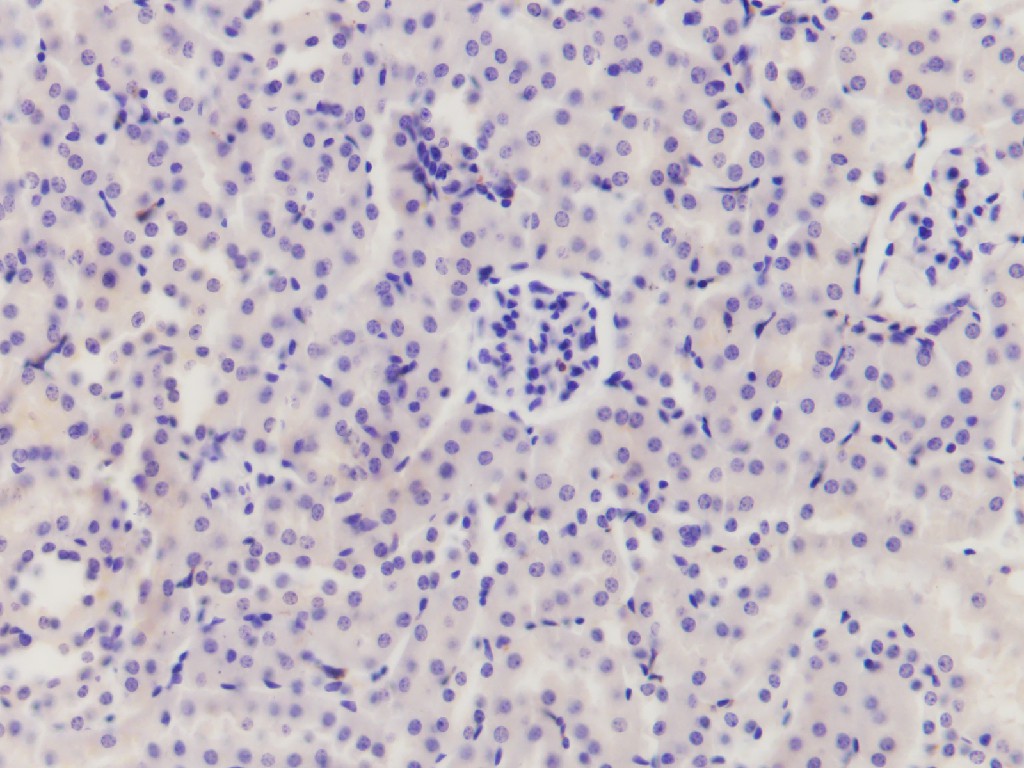

Supplement: Supplementary file 7 — Source data Fig. 6 [file 44321_2025_315_MOESM7_ESM.zip › Figure 6/F6B/2-CD68/4 (1).jpg]

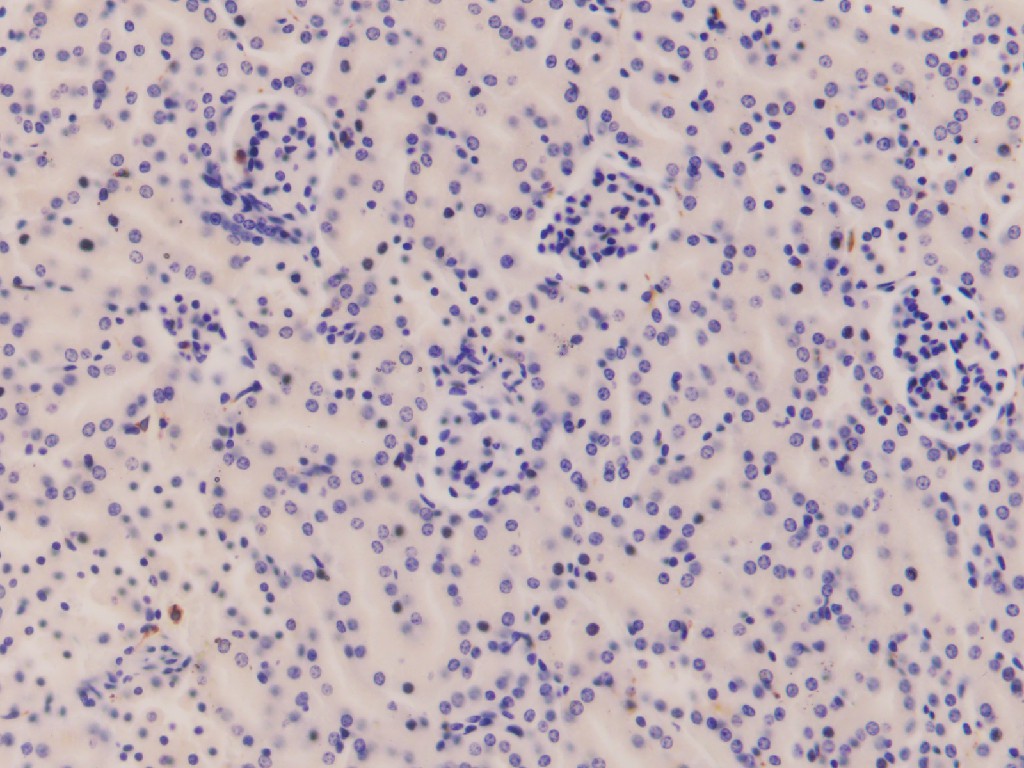

Supplement: Supplementary file 7 — Source data Fig. 6 [file 44321_2025_315_MOESM7_ESM.zip › Figure 6/F6B/2-CD68/4 (2).jpg]

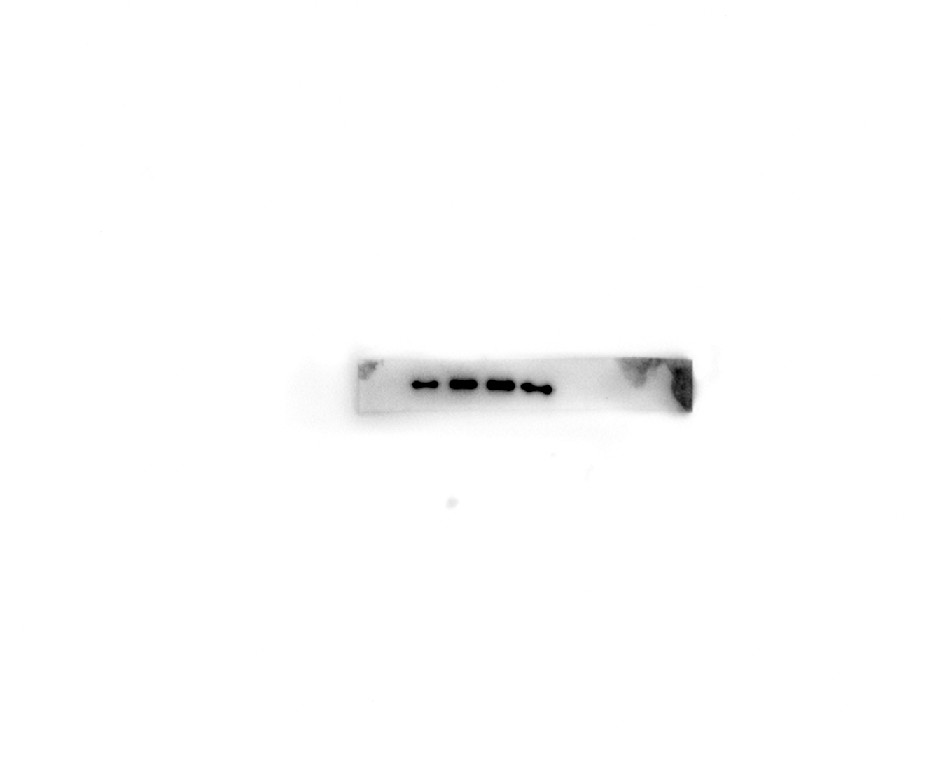

Supplement: Supplementary file 7 — Source data Fig. 6 [file 44321_2025_315_MOESM7_ESM.zip › Figure 6/F6C/1-1-MCP-1.jpg]

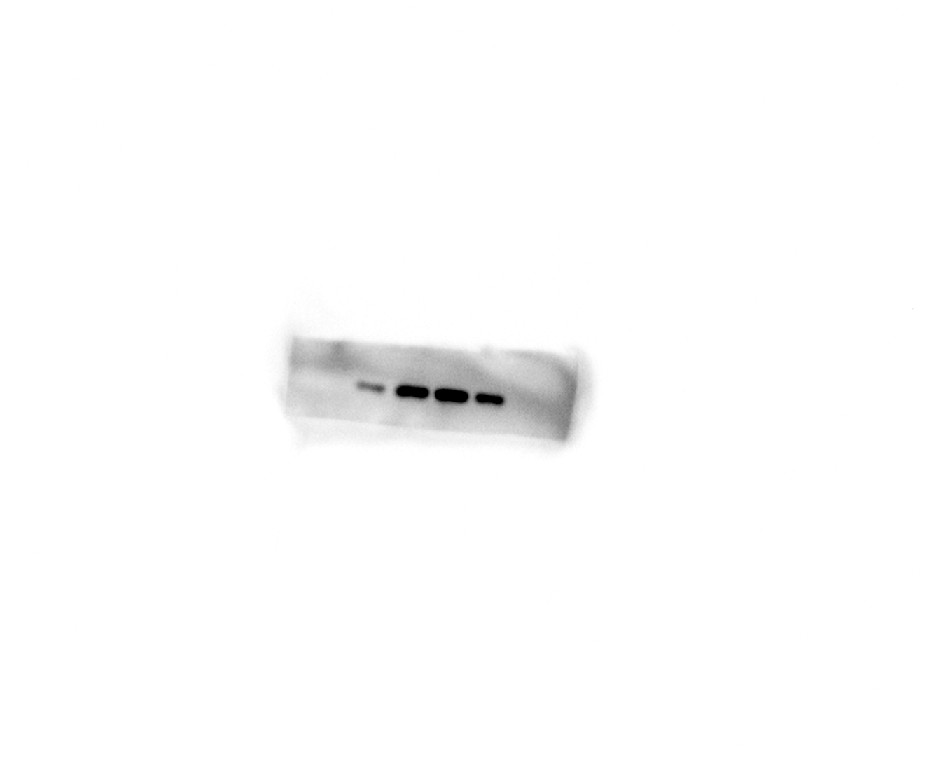

Supplement: Supplementary file 7 — Source data Fig. 6 [file 44321_2025_315_MOESM7_ESM.zip › Figure 6/F6C/1-2-MCP-1.jpg]

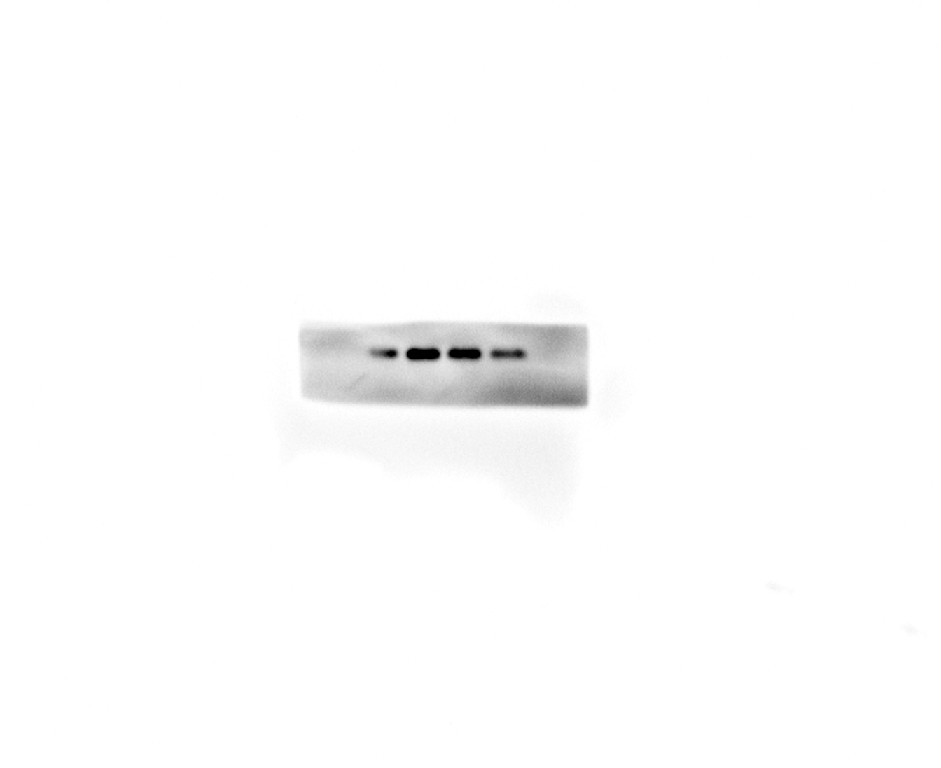

Supplement: Supplementary file 7 — Source data Fig. 6 [file 44321_2025_315_MOESM7_ESM.zip › Figure 6/F6C/1-3-MCP-1.jpg]

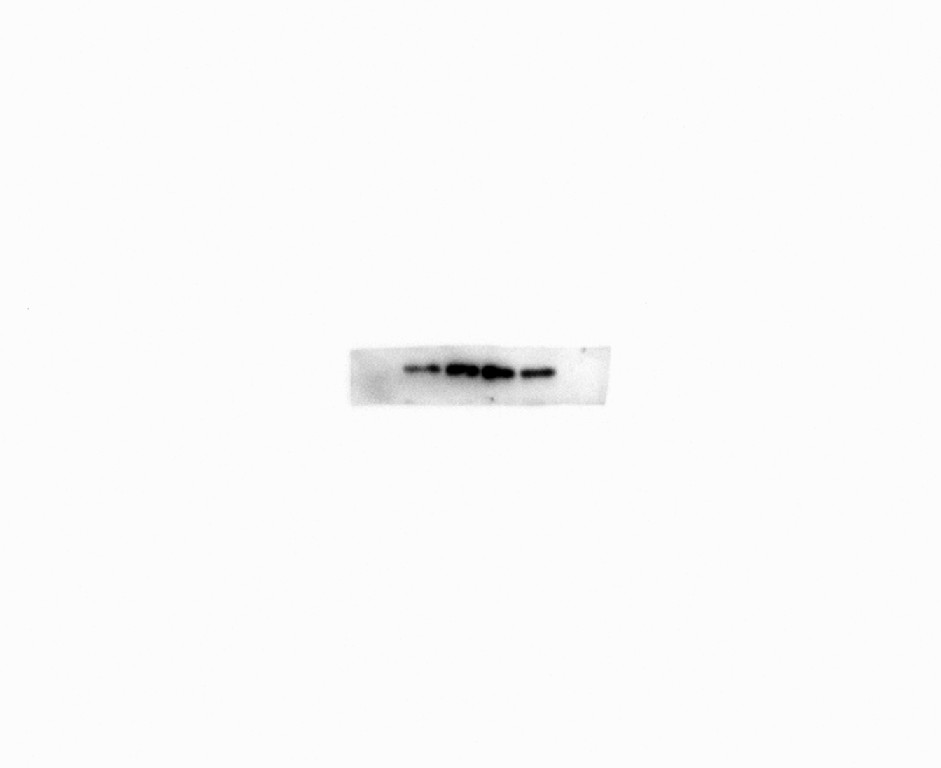

Supplement: Supplementary file 7 — Source data Fig. 6 [file 44321_2025_315_MOESM7_ESM.zip › Figure 6/F6C/1-4-MCP-1.jpg]

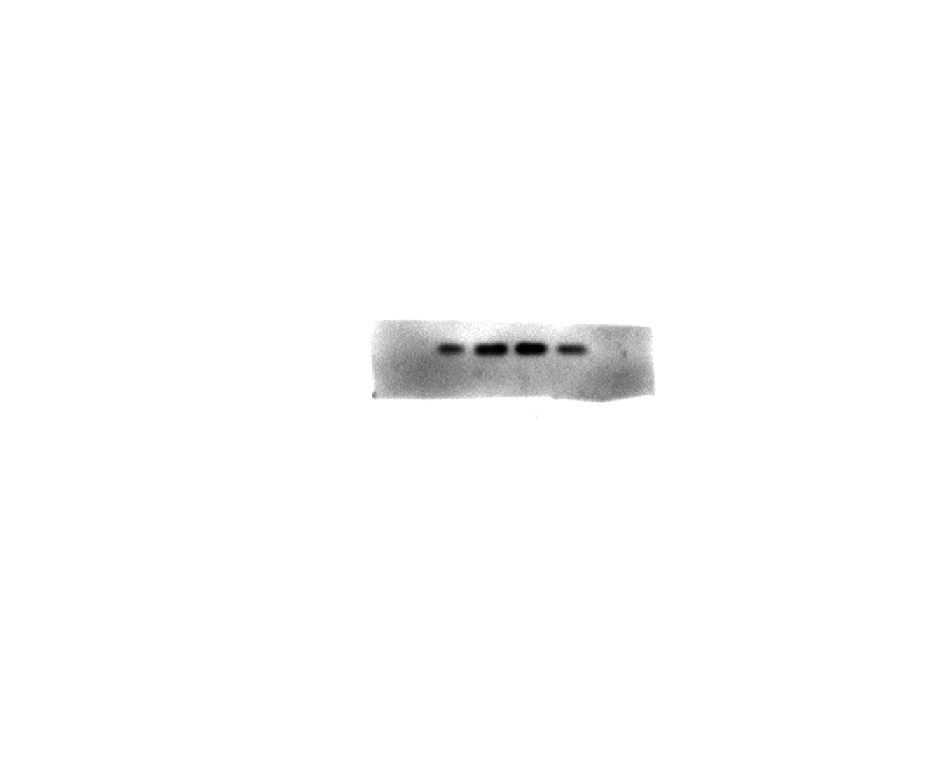

Supplement: Supplementary file 7 — Source data Fig. 6 [file 44321_2025_315_MOESM7_ESM.zip › Figure 6/F6C/1-5-MCP-1.jpg]

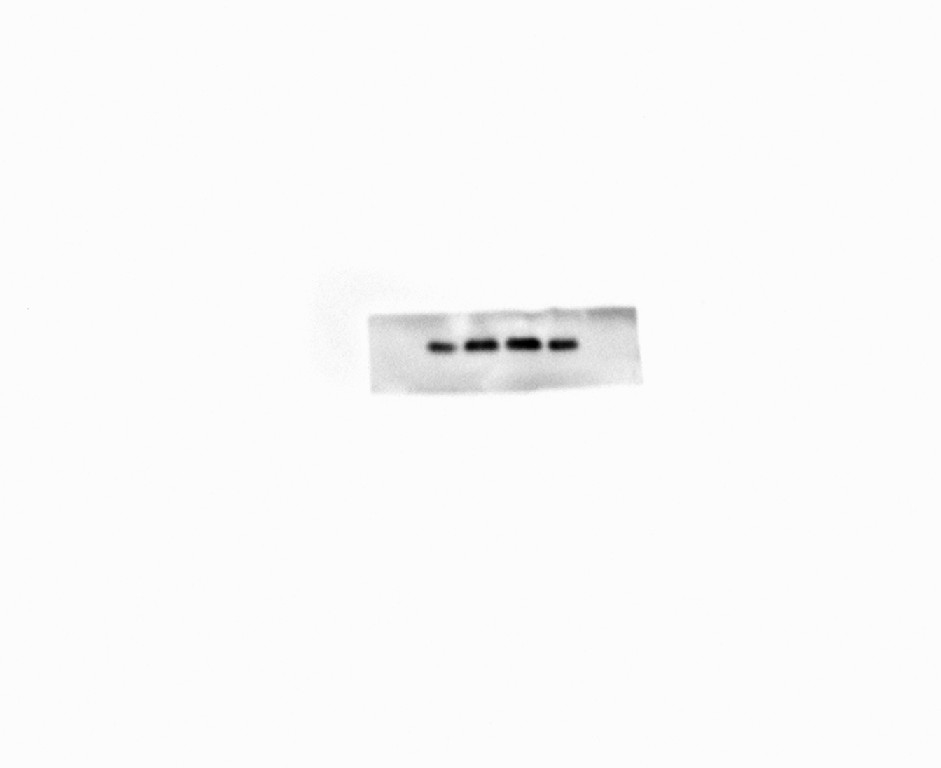

Supplement: Supplementary file 7 — Source data Fig. 6 [file 44321_2025_315_MOESM7_ESM.zip › Figure 6/F6C/1-6-MCP-1.jpg]

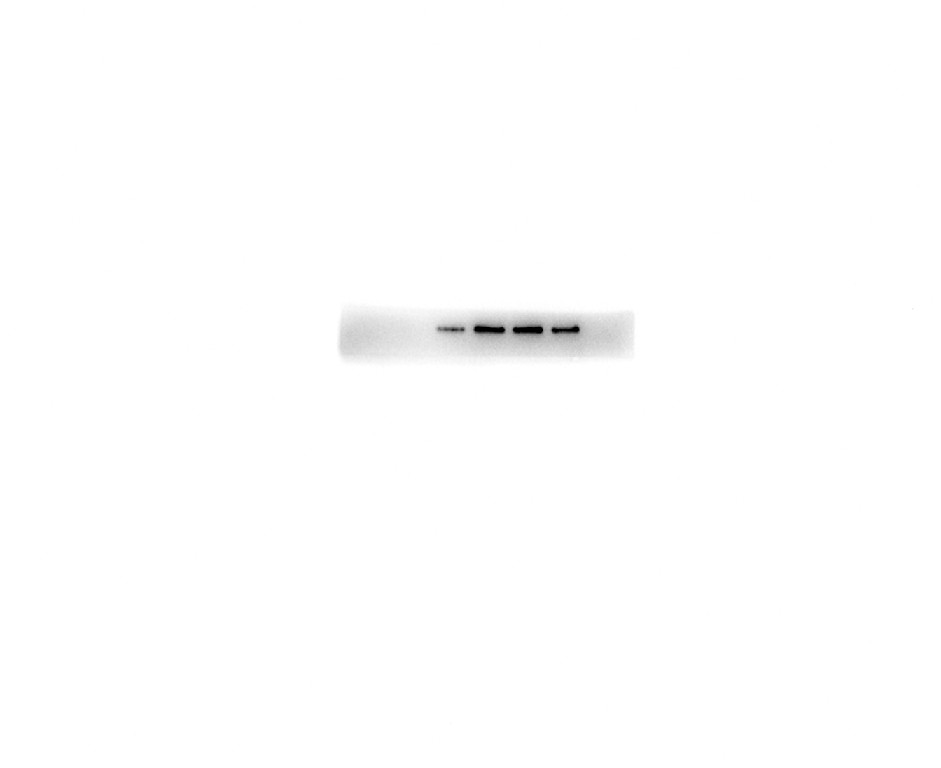

Supplement: Supplementary file 7 — Source data Fig. 6 [file 44321_2025_315_MOESM7_ESM.zip › Figure 6/F6C/2-1-IL-6.jpg]

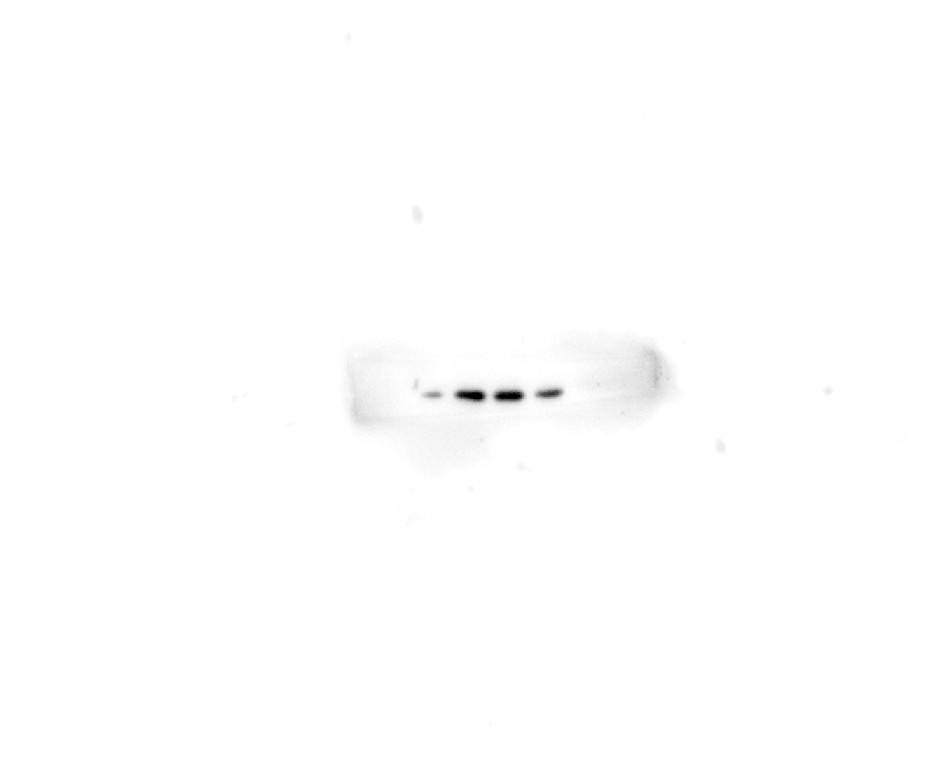

Supplement: Supplementary file 7 — Source data Fig. 6 [file 44321_2025_315_MOESM7_ESM.zip › Figure 6/F6C/2-2-IL-6.jpg]

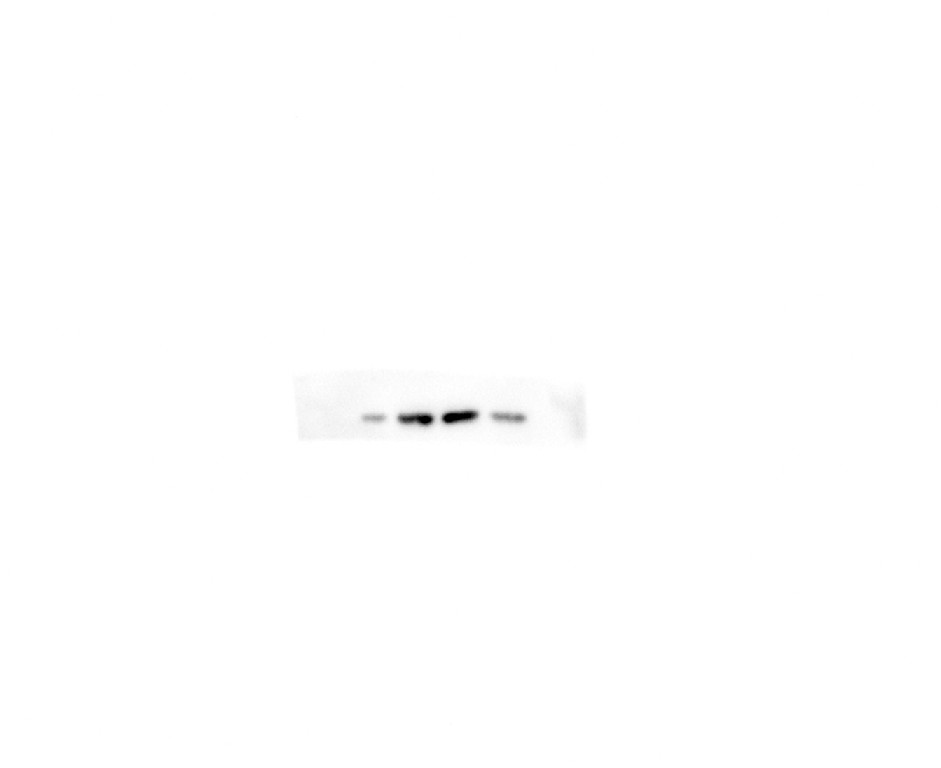

Supplement: Supplementary file 7 — Source data Fig. 6 [file 44321_2025_315_MOESM7_ESM.zip › Figure 6/F6C/2-3-IL-6.jpg]

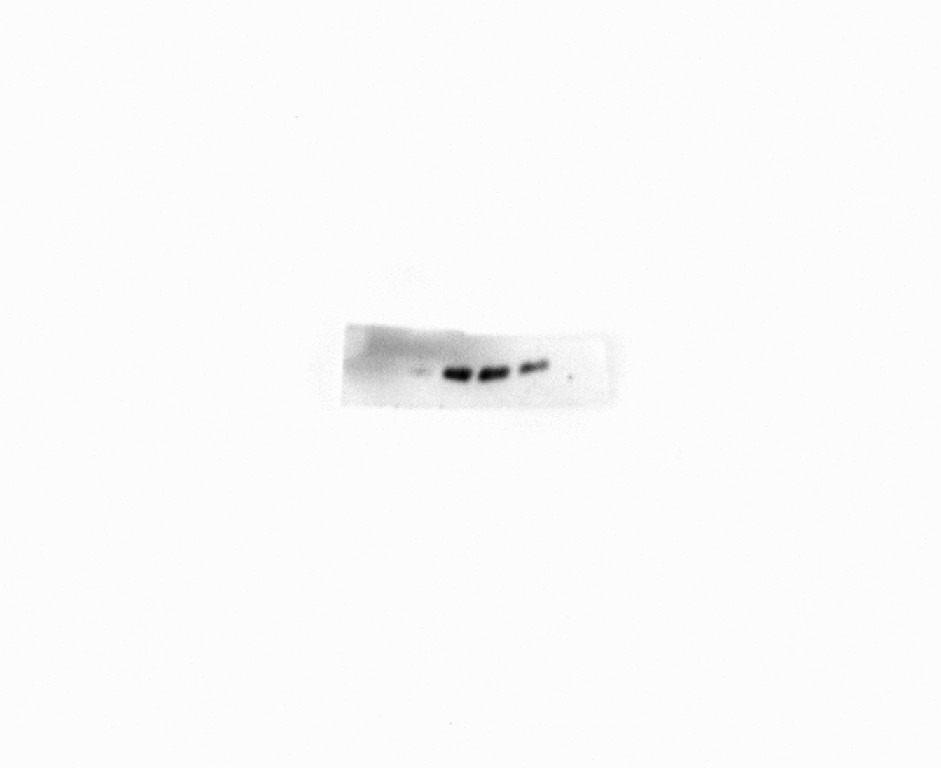

Supplement: Supplementary file 7 — Source data Fig. 6 [file 44321_2025_315_MOESM7_ESM.zip › Figure 6/F6C/2-4-IL-6.jpg]

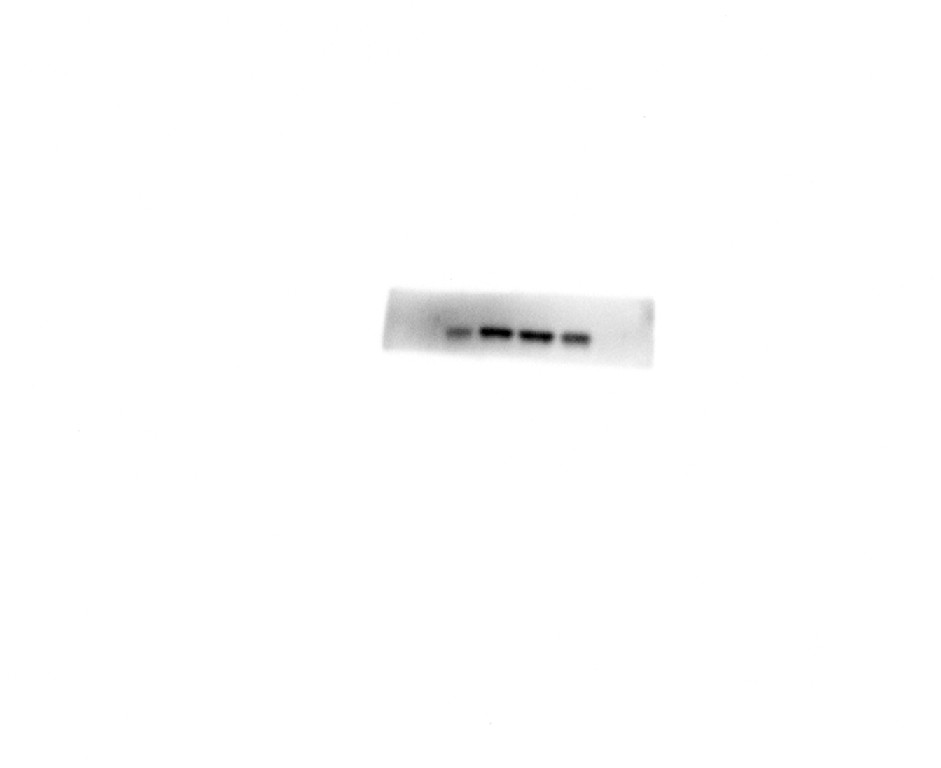

Supplement: Supplementary file 7 — Source data Fig. 6 [file 44321_2025_315_MOESM7_ESM.zip › Figure 6/F6C/2-5-IL-6.jpg]

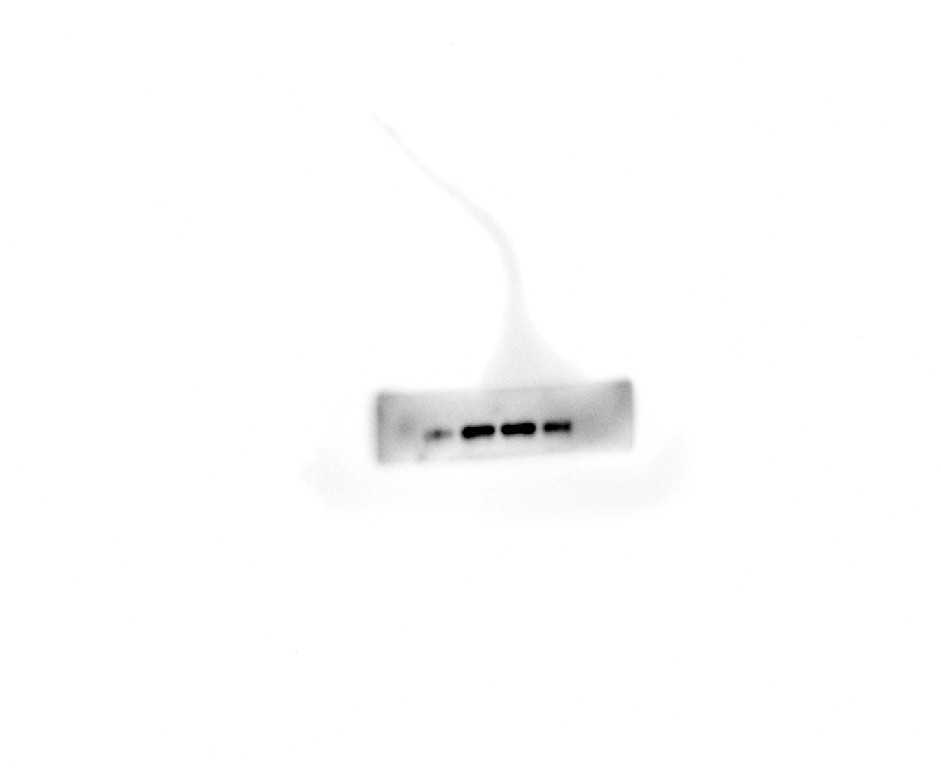

Supplement: Supplementary file 7 — Source data Fig. 6 [file 44321_2025_315_MOESM7_ESM.zip › Figure 6/F6C/2-6-IL-6.jpg]

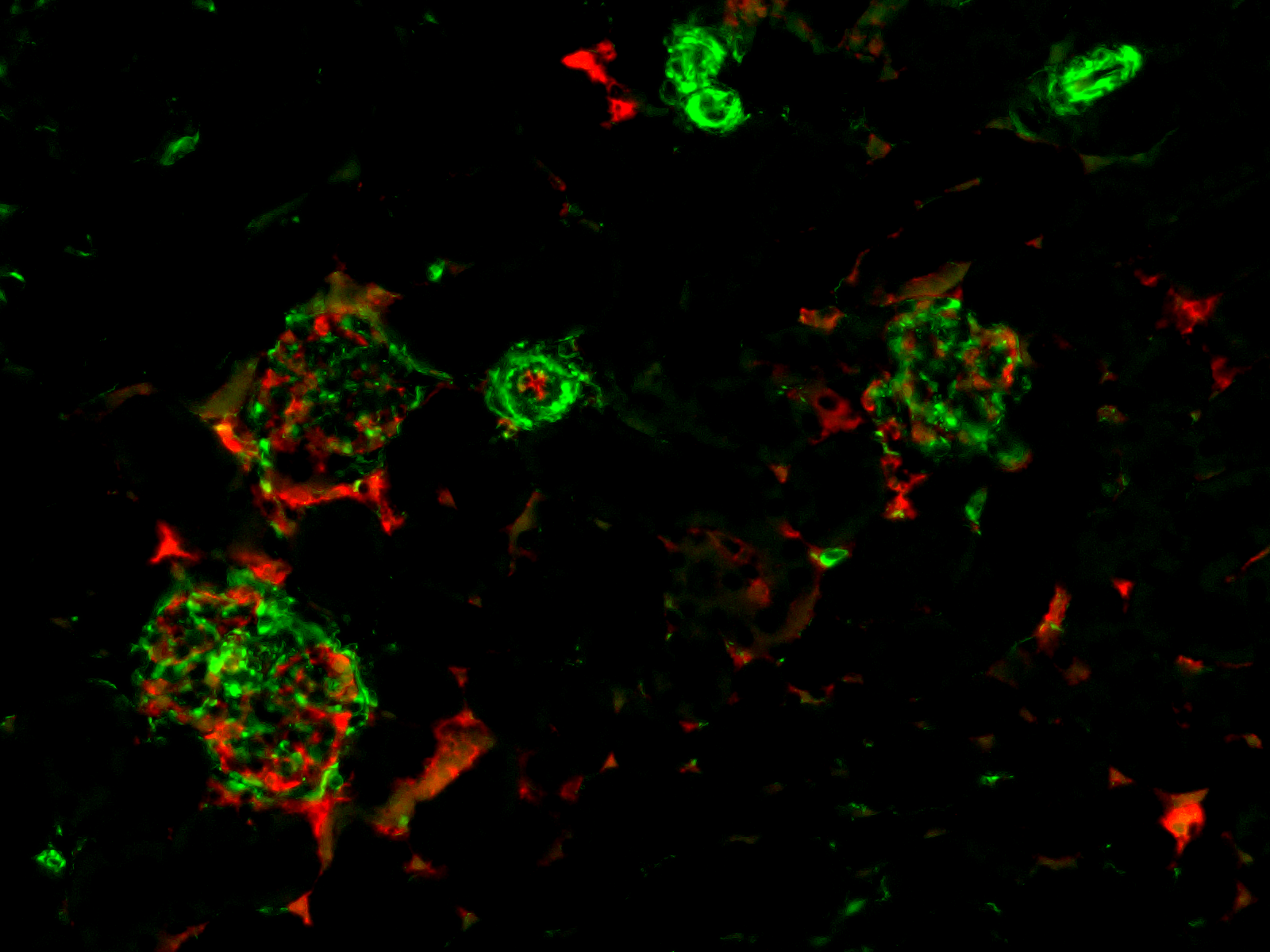

Supplement: Supplementary file 9 — Figure EV1 Source Data [file 44321_2025_315_MOESM9_ESM.zip › Figure EV1/EV1E/IgAN (4).tif]

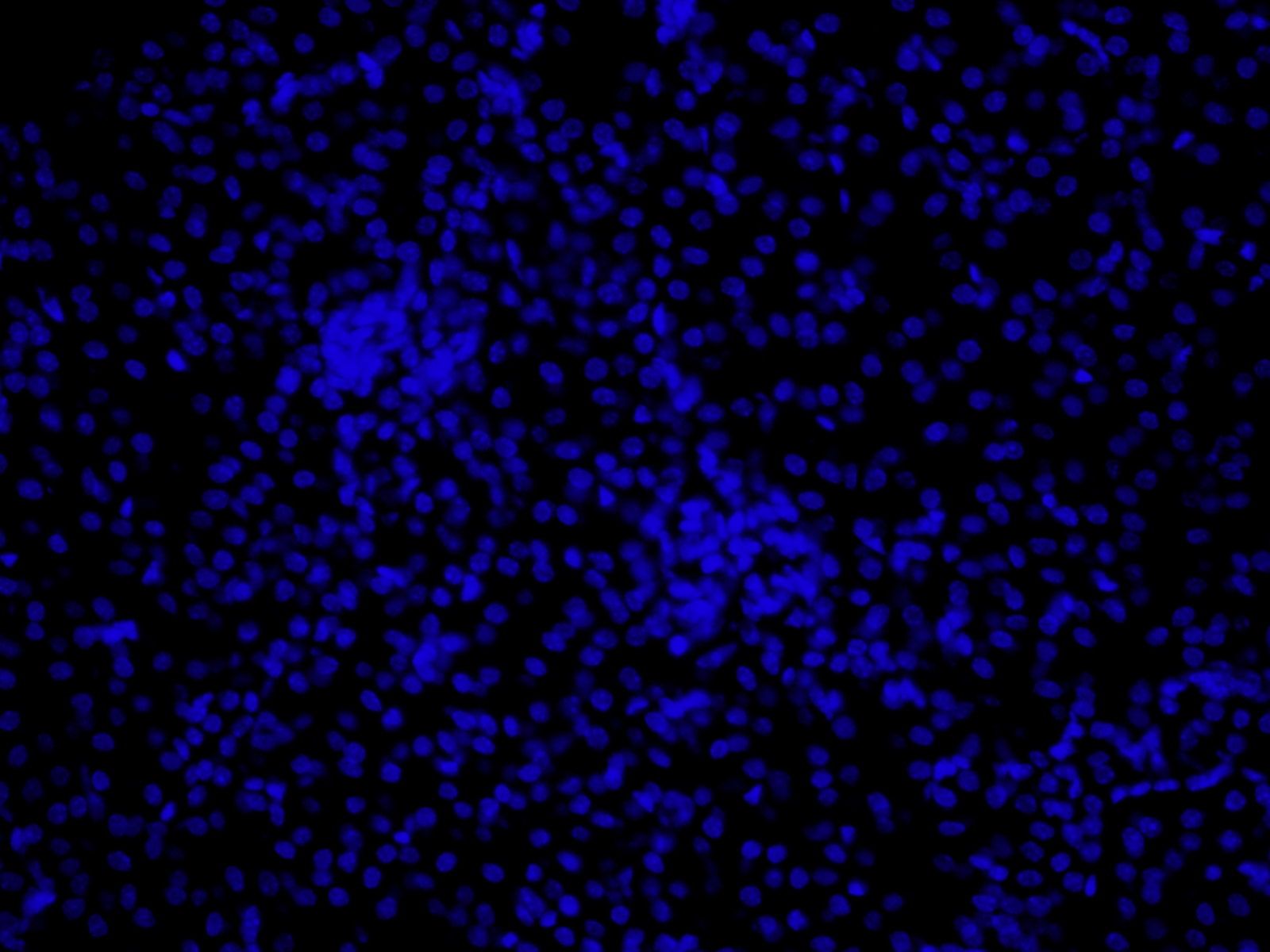

Supplement: Supplementary file 9 — Figure EV1 Source Data [file 44321_2025_315_MOESM9_ESM.zip › Figure EV1/EV1E/Control (1).tif]

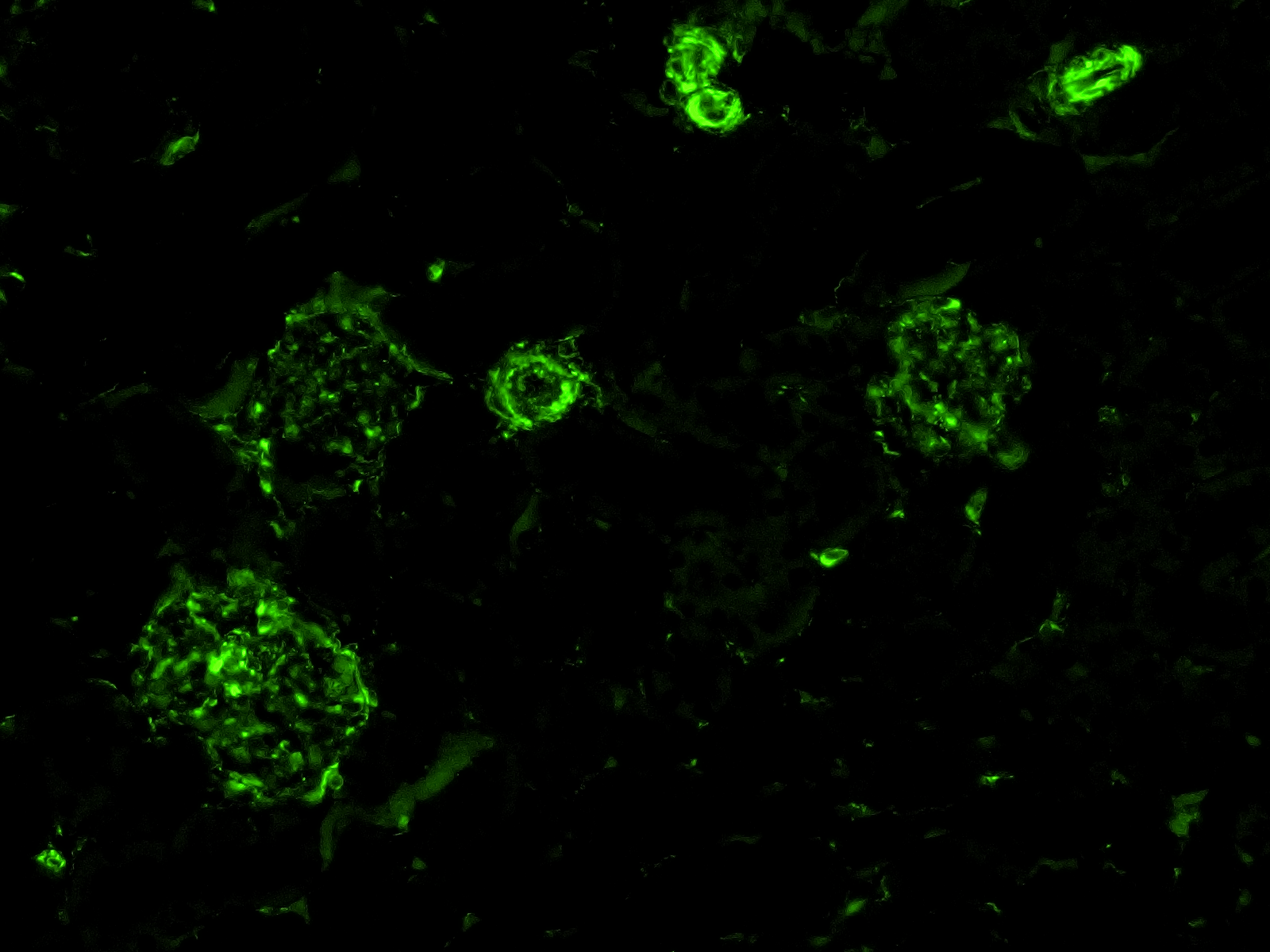

Supplement: Supplementary file 9 — Figure EV1 Source Data [file 44321_2025_315_MOESM9_ESM.zip › Figure EV1/EV1E/IgAN (2).tif]

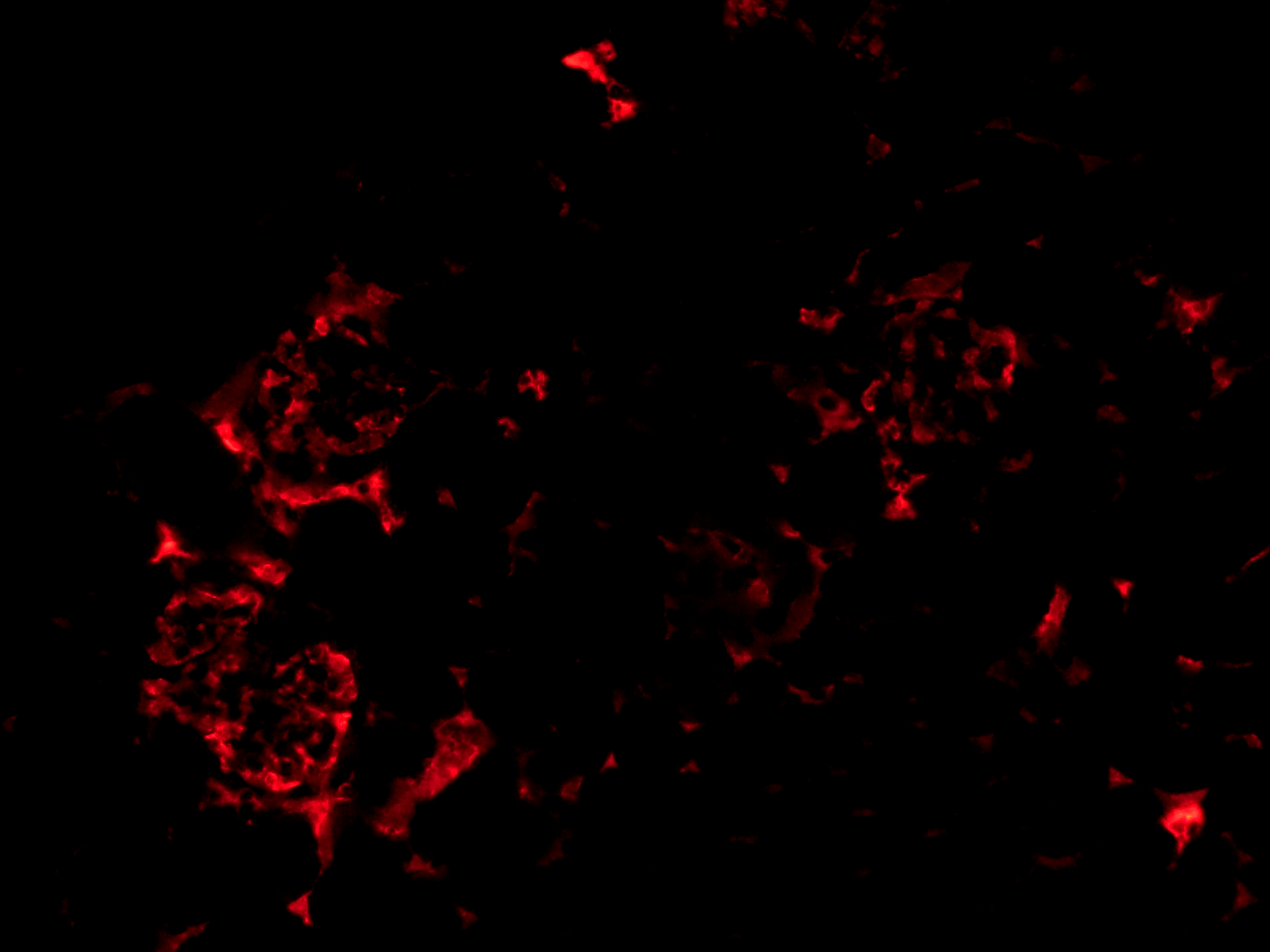

Supplement: Supplementary file 9 — Figure EV1 Source Data [file 44321_2025_315_MOESM9_ESM.zip › Figure EV1/EV1E/IgAN (3).tif]

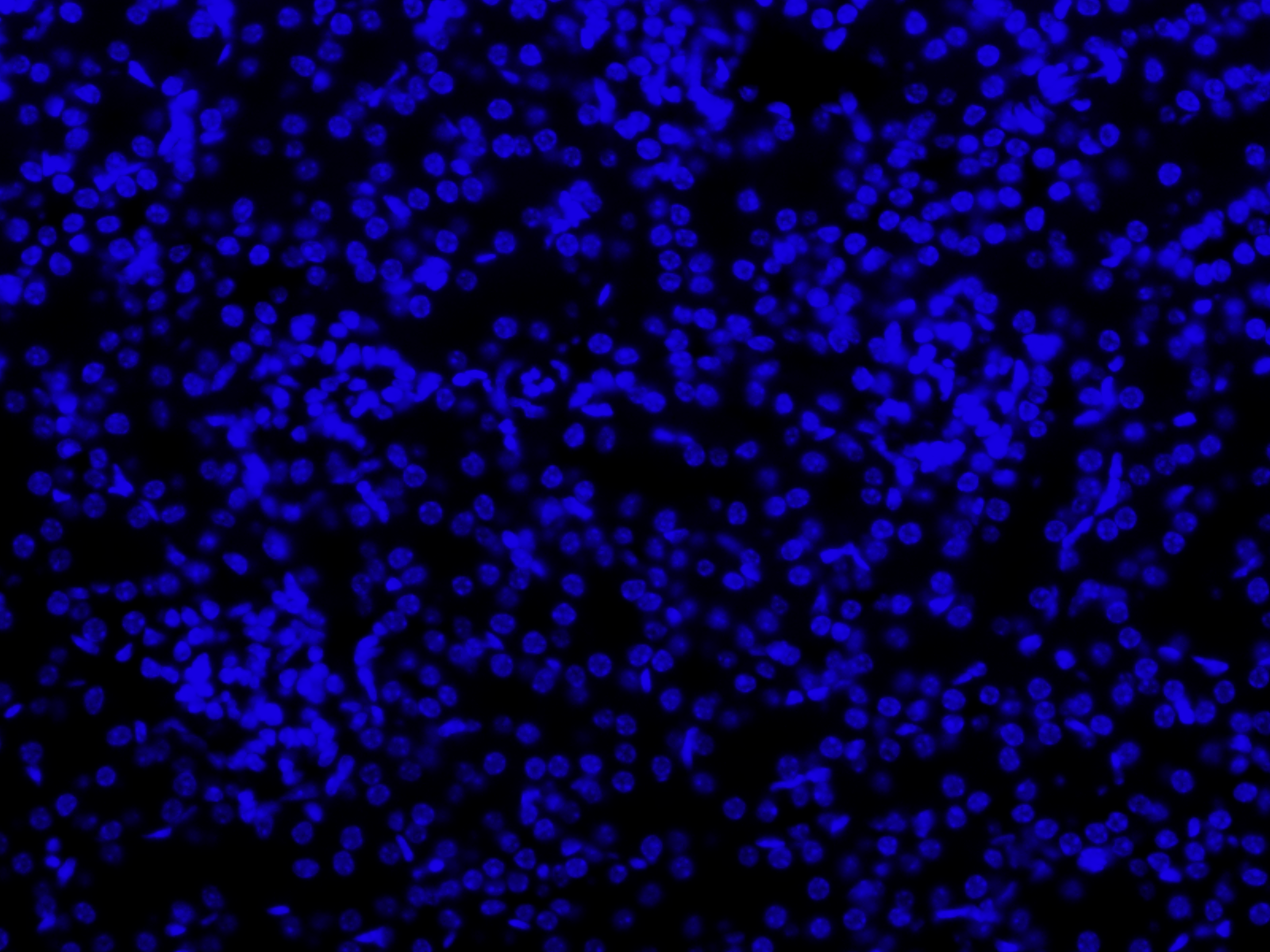

Supplement: Supplementary file 9 — Figure EV1 Source Data [file 44321_2025_315_MOESM9_ESM.zip › Figure EV1/EV1E/IgAN (1).tif]

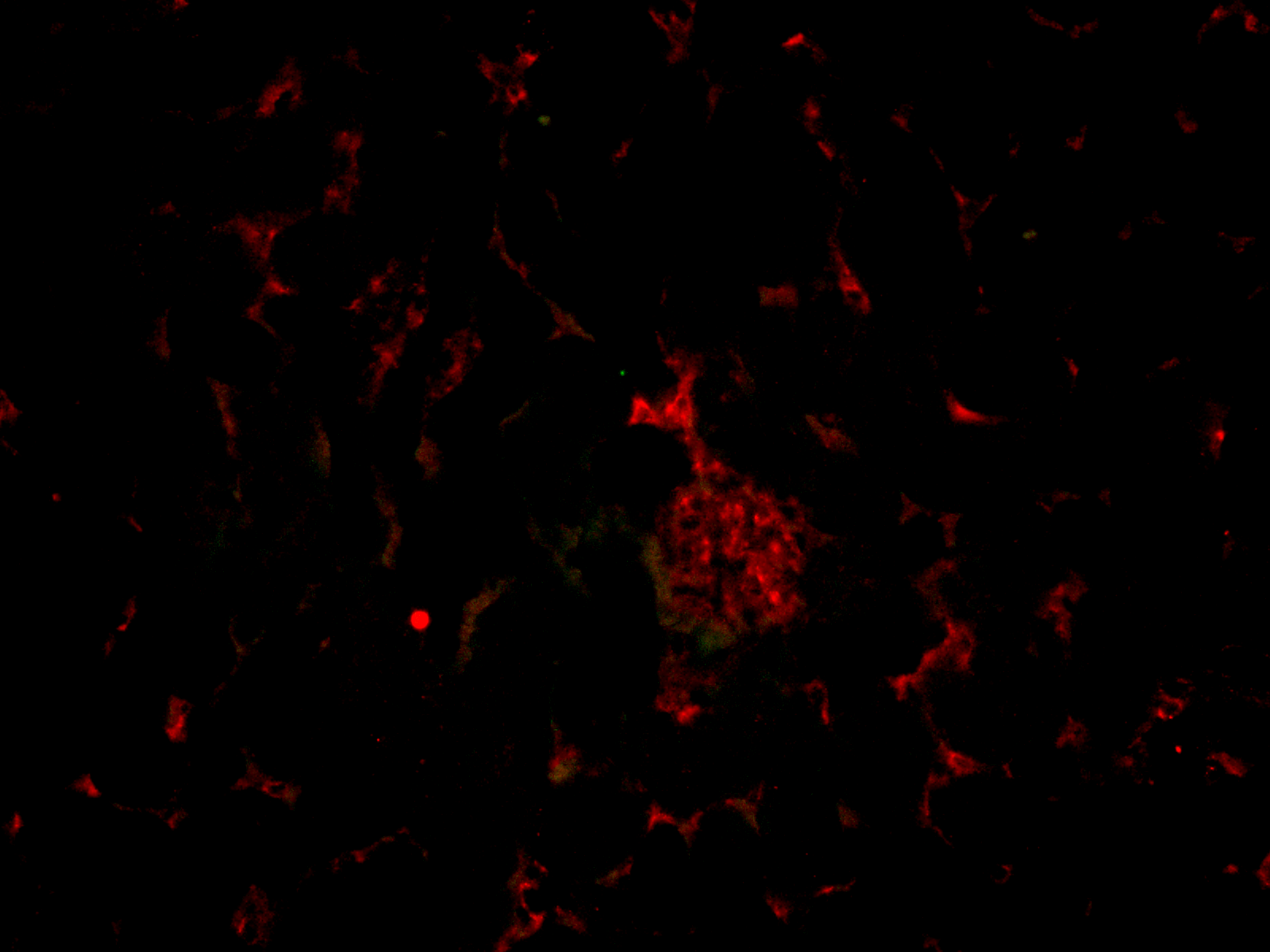

Supplement: Supplementary file 9 — Figure EV1 Source Data [file 44321_2025_315_MOESM9_ESM.zip › Figure EV1/EV1E/Control (4).tif]

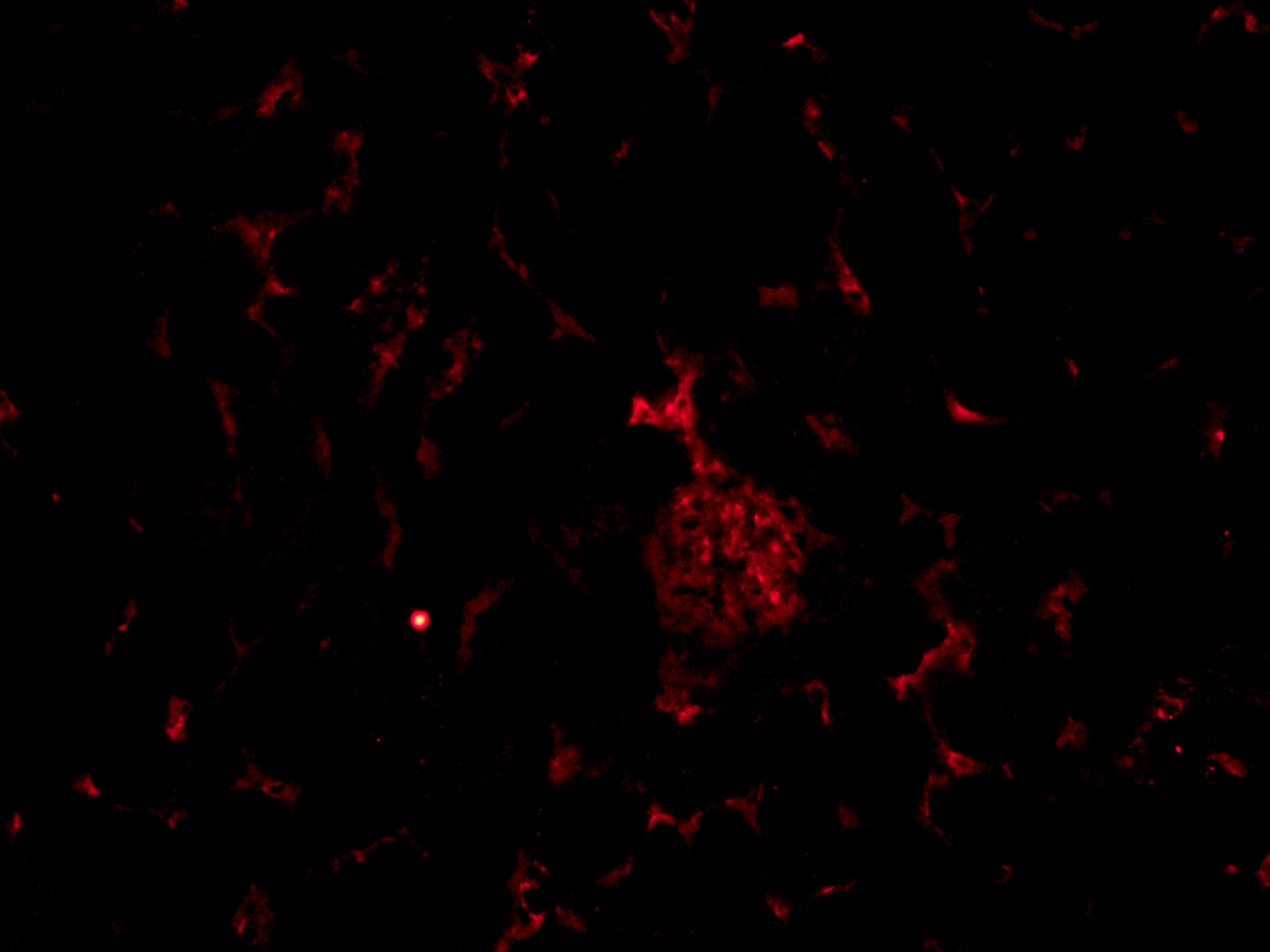

Supplement: Supplementary file 9 — Figure EV1 Source Data [file 44321_2025_315_MOESM9_ESM.zip › Figure EV1/EV1E/Control (3).tif]

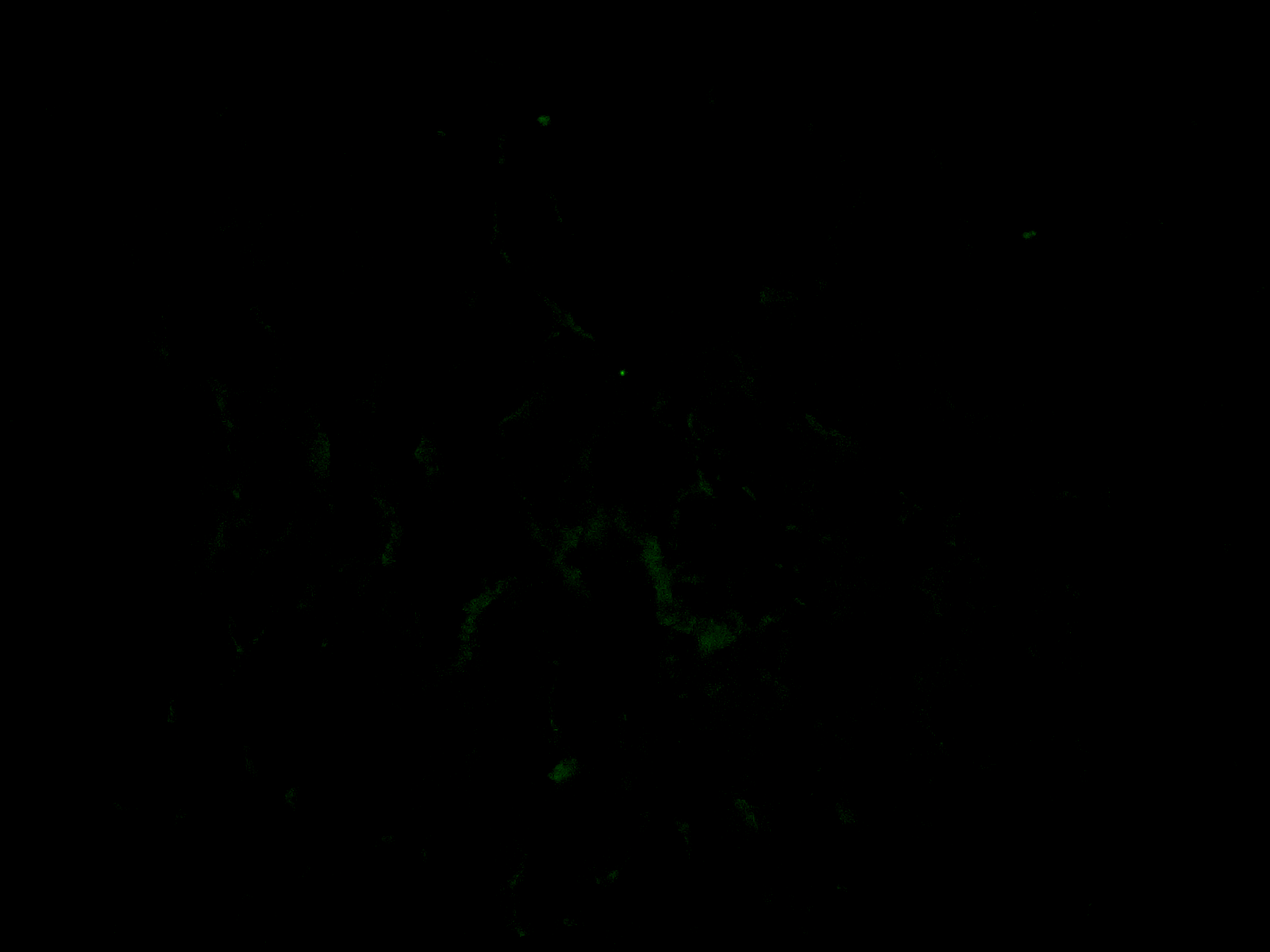

Supplement: Supplementary file 9 — Figure EV1 Source Data [file 44321_2025_315_MOESM9_ESM.zip › Figure EV1/EV1E/Control (2).tif]

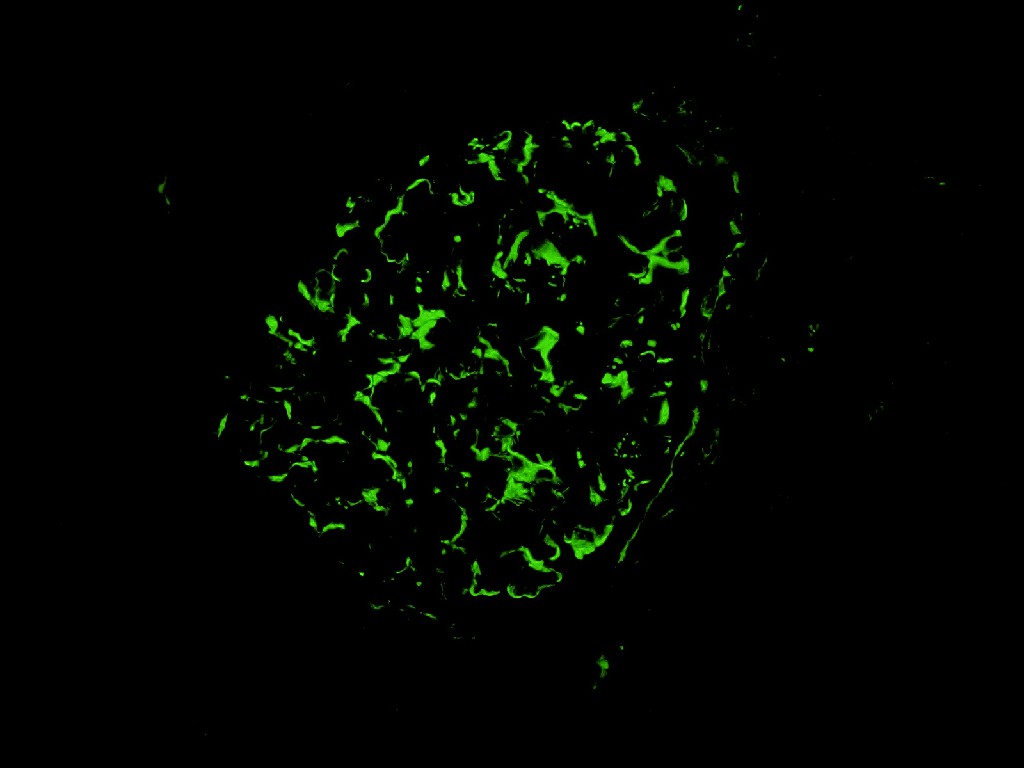

Supplement: Supplementary file 9 — Figure EV1 Source Data [file 44321_2025_315_MOESM9_ESM.zip › Figure EV1/EV1D/3-Claudin-1-GLDC/LEE III/3 (1).jpg]

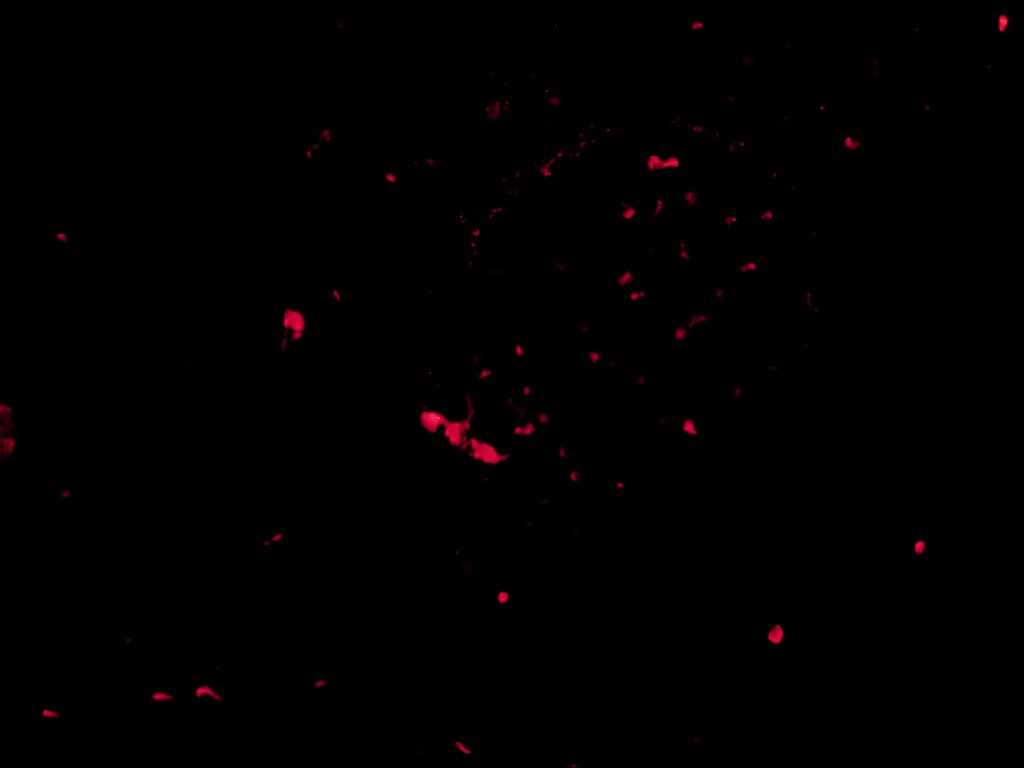

Supplement: Supplementary file 9 — Figure EV1 Source Data [file 44321_2025_315_MOESM9_ESM.zip › Figure EV1/EV1D/3-Claudin-1-GLDC/LEE III/6 (2).jpg]

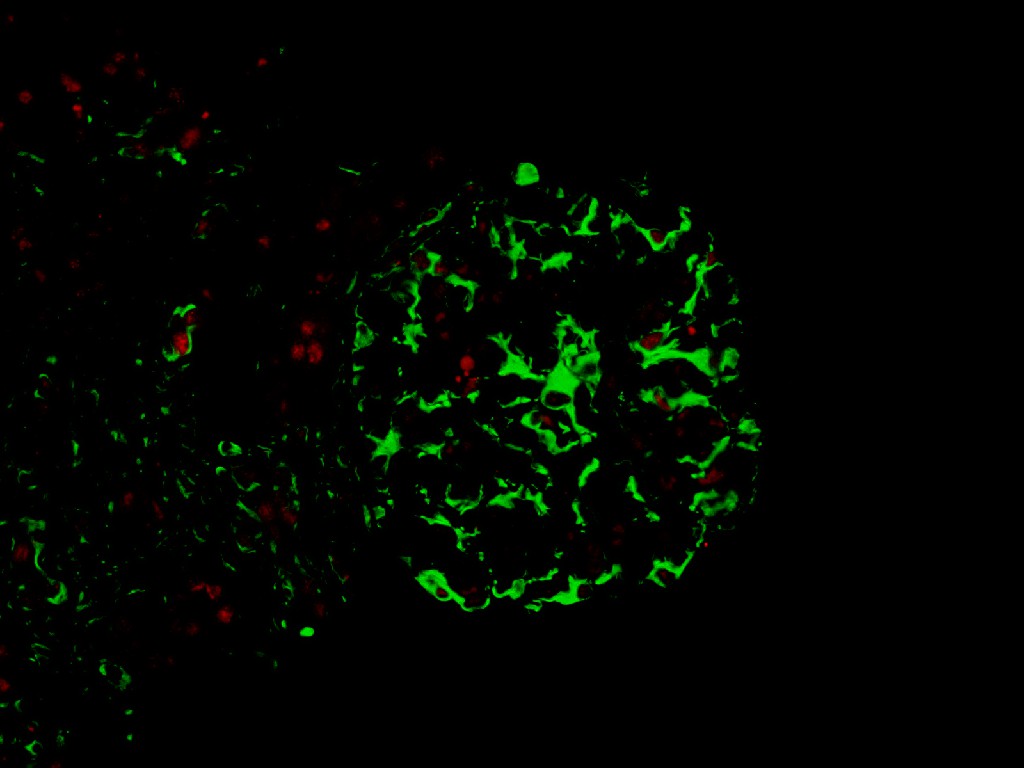

Supplement: Supplementary file 9 — Figure EV1 Source Data [file 44321_2025_315_MOESM9_ESM.zip › Figure EV1/EV1D/3-Claudin-1-GLDC/LEE III/5 (4).jpg]

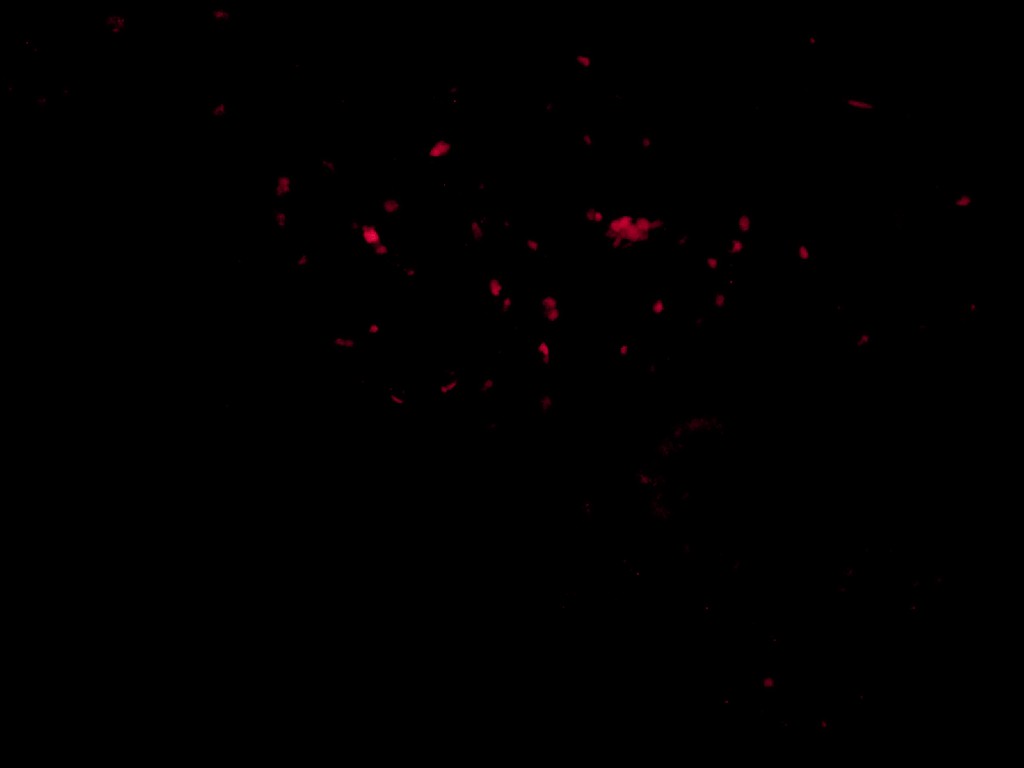

Supplement: Supplementary file 9 — Figure EV1 Source Data [file 44321_2025_315_MOESM9_ESM.zip › Figure EV1/EV1D/3-Claudin-1-GLDC/LEE III/2 (1).jpg]

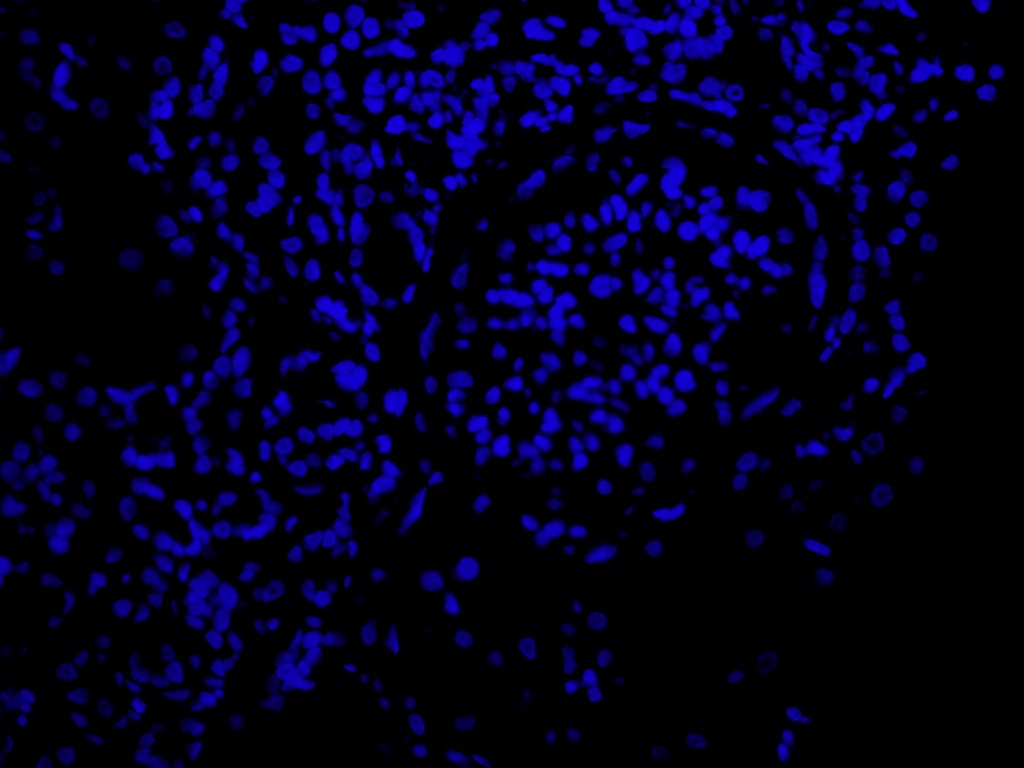

Supplement: Supplementary file 9 — Figure EV1 Source Data [file 44321_2025_315_MOESM9_ESM.zip › Figure EV1/EV1D/3-Claudin-1-GLDC/LEE III/6 (3).jpg]

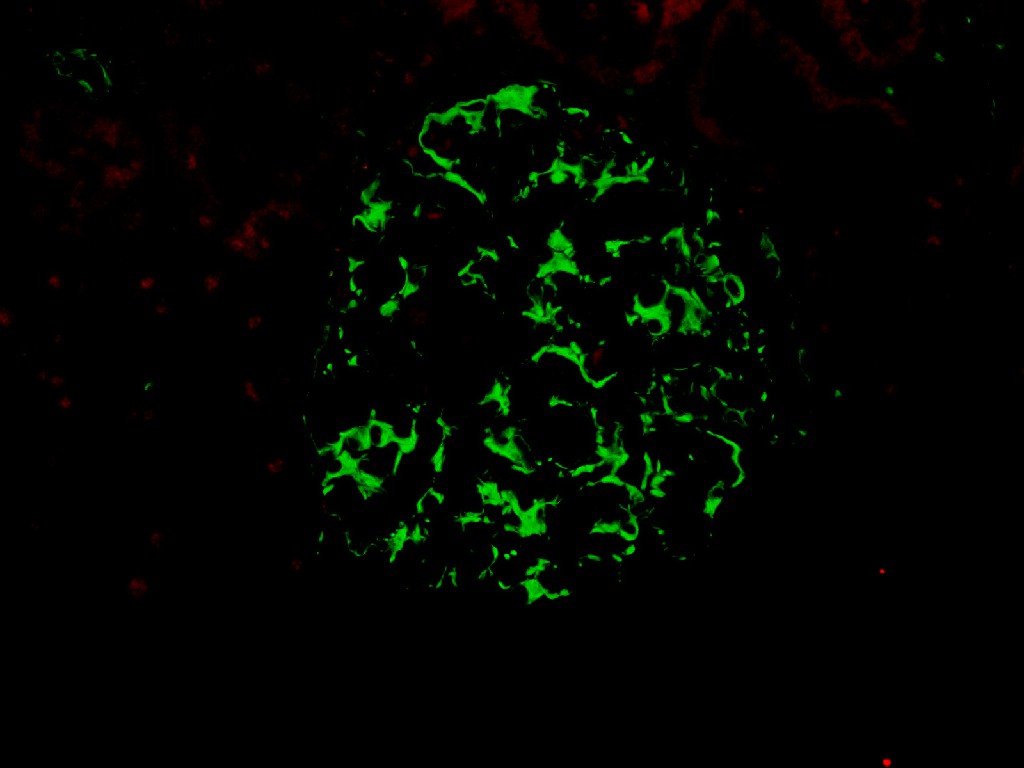

Supplement: Supplementary file 9 — Figure EV1 Source Data [file 44321_2025_315_MOESM9_ESM.zip › Figure EV1/EV1D/3-Claudin-1-GLDC/LEE III/4 (4).jpg]

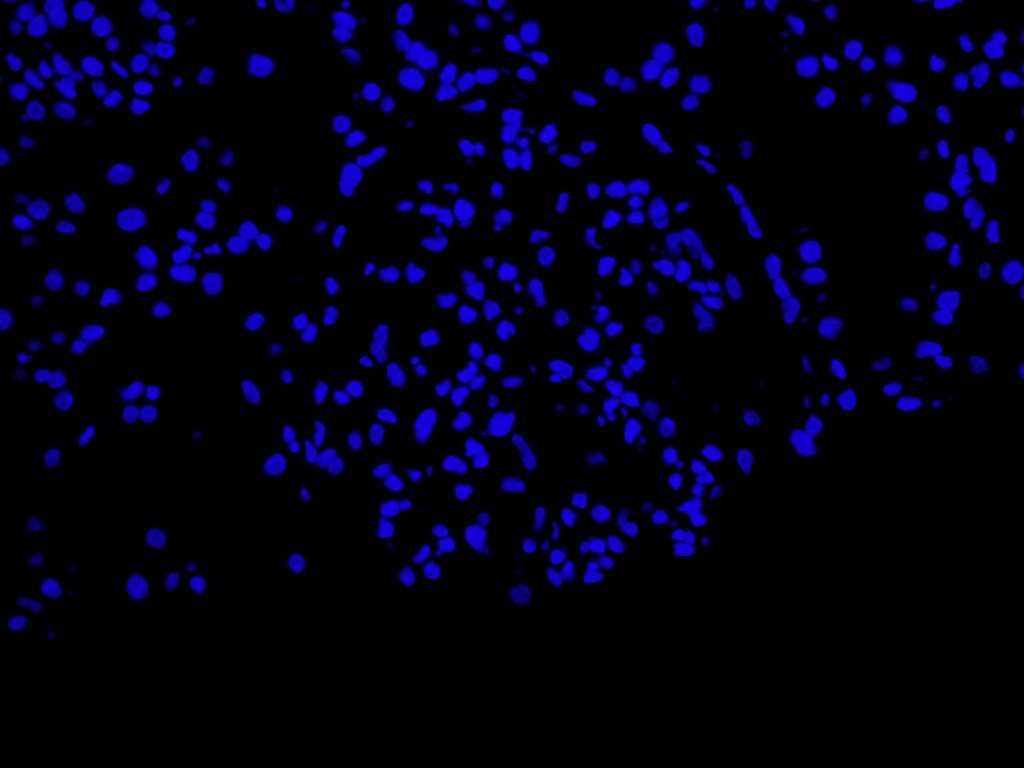

Supplement: Supplementary file 9 — Figure EV1 Source Data [file 44321_2025_315_MOESM9_ESM.zip › Figure EV1/EV1D/3-Claudin-1-GLDC/LEE III/4 (3).jpg]

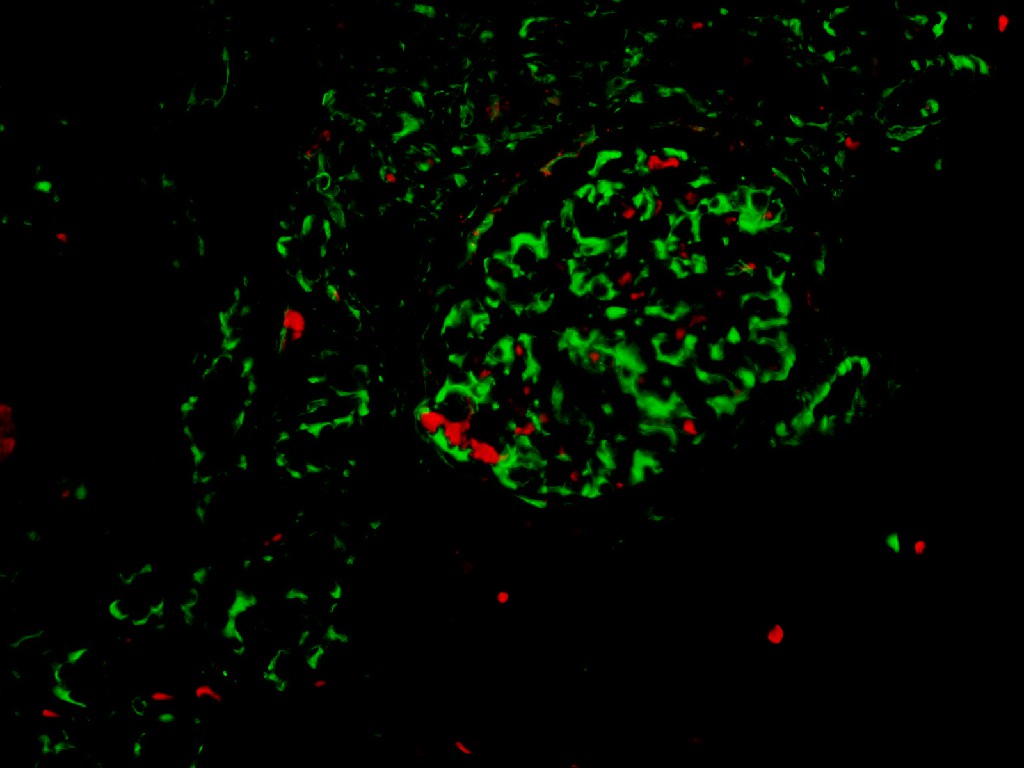

Supplement: Supplementary file 9 — Figure EV1 Source Data [file 44321_2025_315_MOESM9_ESM.zip › Figure EV1/EV1D/3-Claudin-1-GLDC/LEE III/6 (4).jpg]

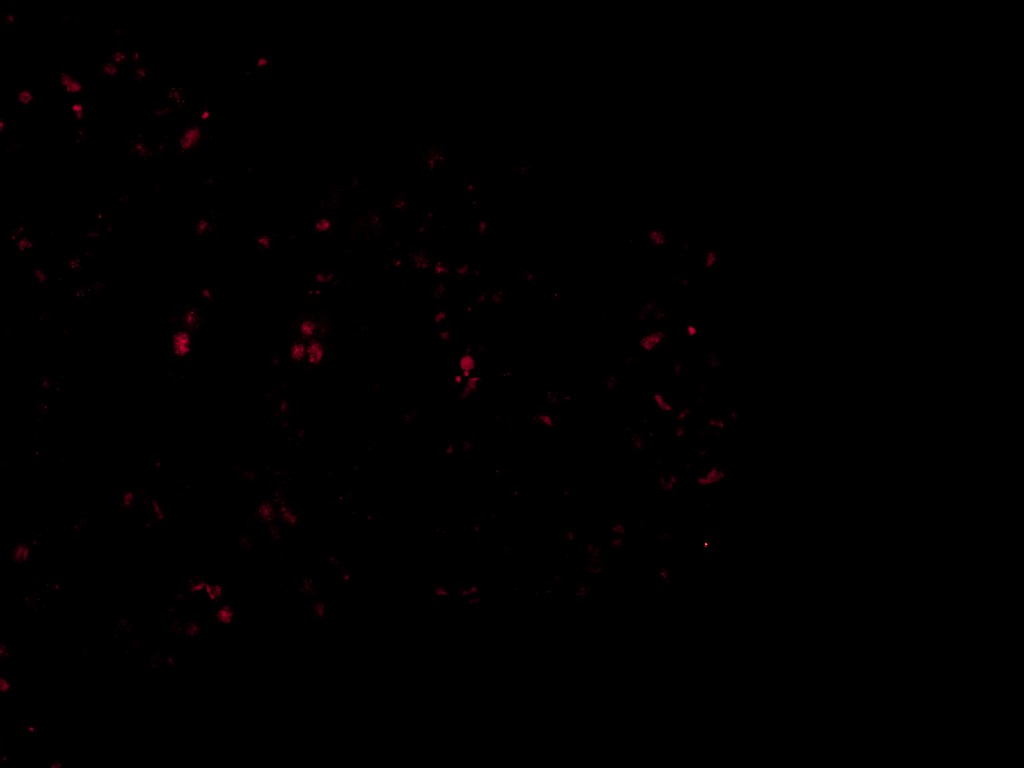

Supplement: Supplementary file 9 — Figure EV1 Source Data [file 44321_2025_315_MOESM9_ESM.zip › Figure EV1/EV1D/3-Claudin-1-GLDC/LEE III/5 (2).jpg]

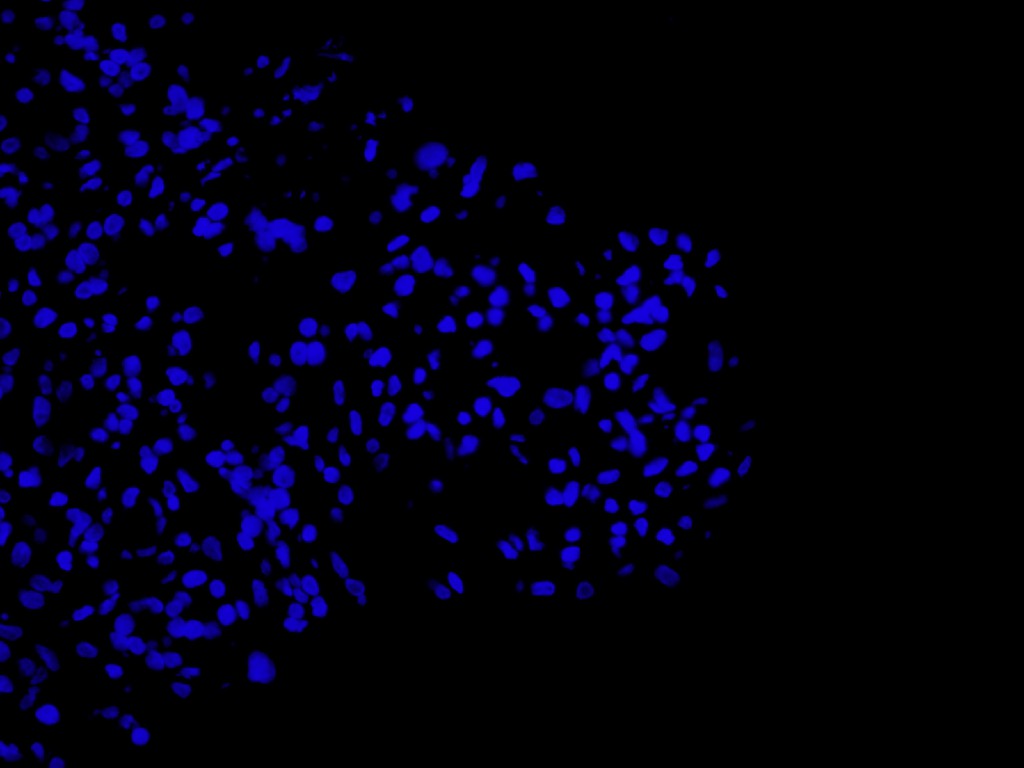

Supplement: Supplementary file 9 — Figure EV1 Source Data [file 44321_2025_315_MOESM9_ESM.zip › Figure EV1/EV1D/3-Claudin-1-GLDC/LEE III/5 (3).jpg]

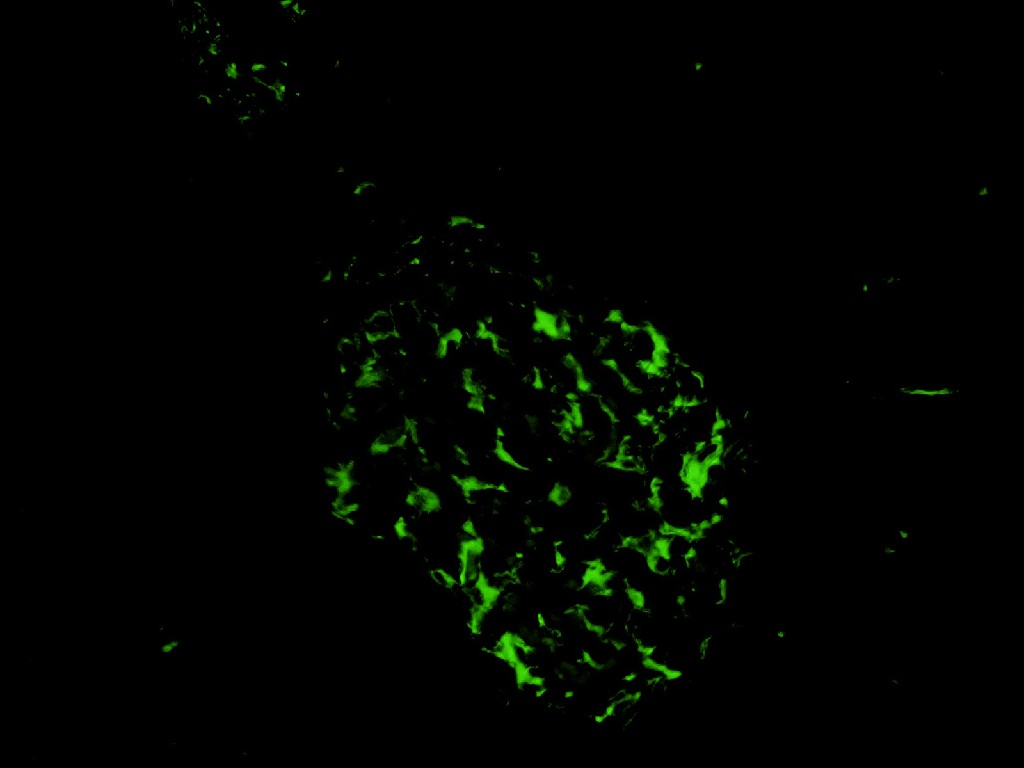

Supplement: Supplementary file 9 — Figure EV1 Source Data [file 44321_2025_315_MOESM9_ESM.zip › Figure EV1/EV1D/3-Claudin-1-GLDC/LEE III/1 (1).jpg]

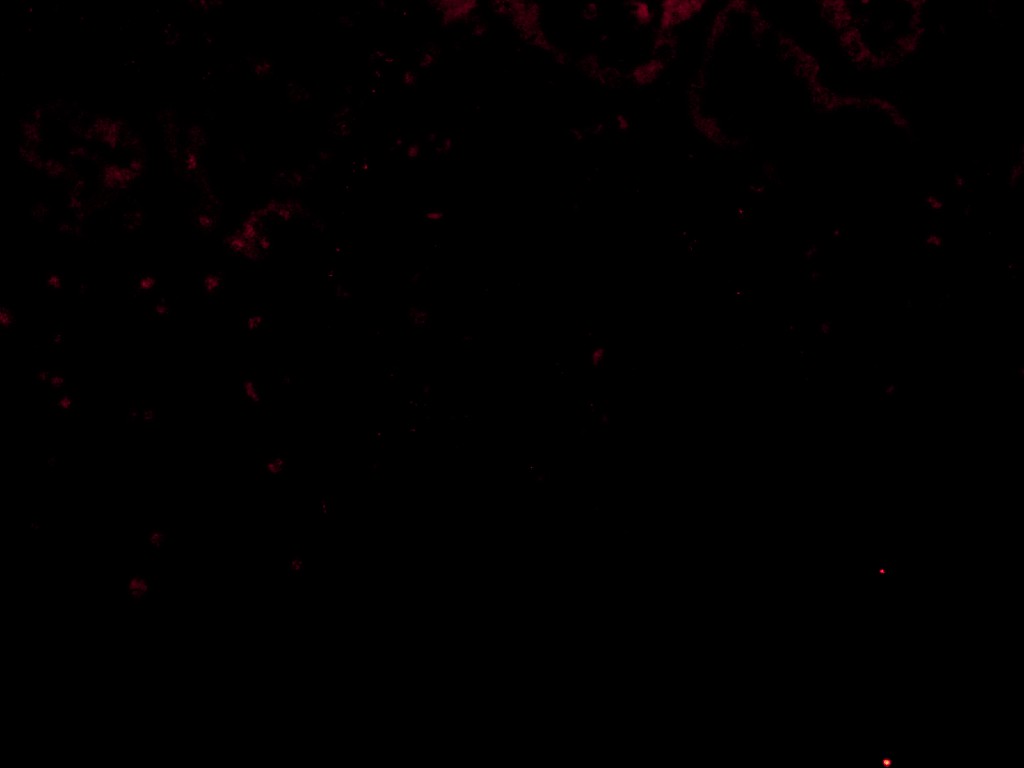

Supplement: Supplementary file 9 — Figure EV1 Source Data [file 44321_2025_315_MOESM9_ESM.zip › Figure EV1/EV1D/3-Claudin-1-GLDC/LEE III/4 (2).jpg]

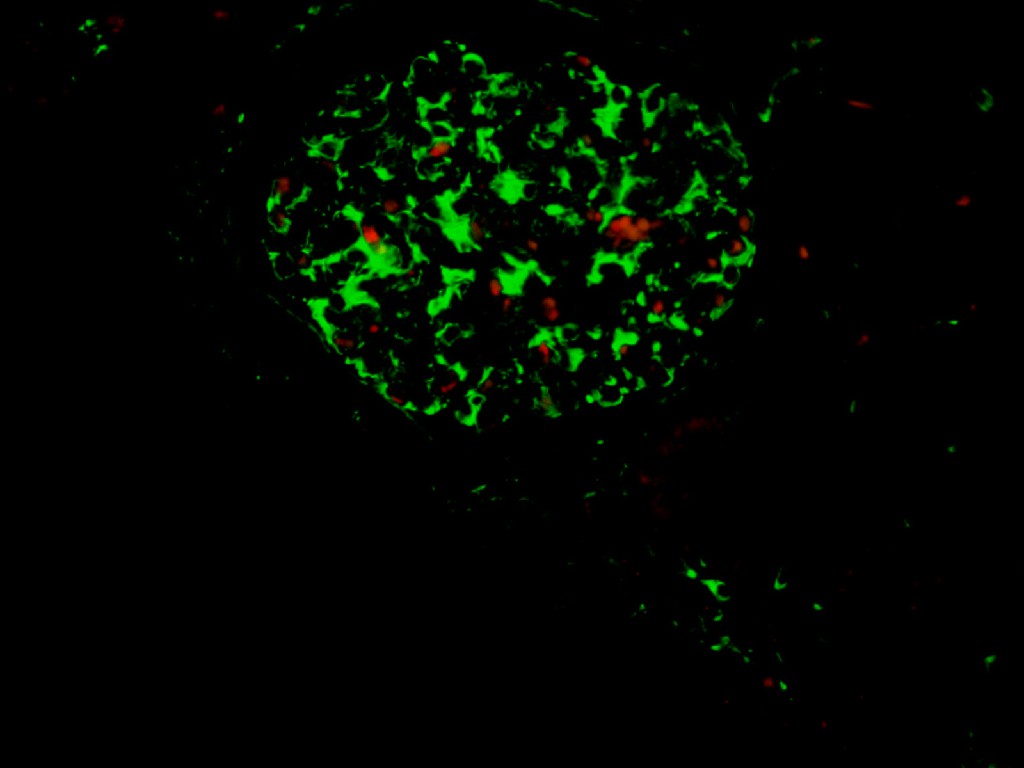

Supplement: Supplementary file 9 — Figure EV1 Source Data [file 44321_2025_315_MOESM9_ESM.zip › Figure EV1/EV1D/3-Claudin-1-GLDC/LEE III/2 (4).jpg]

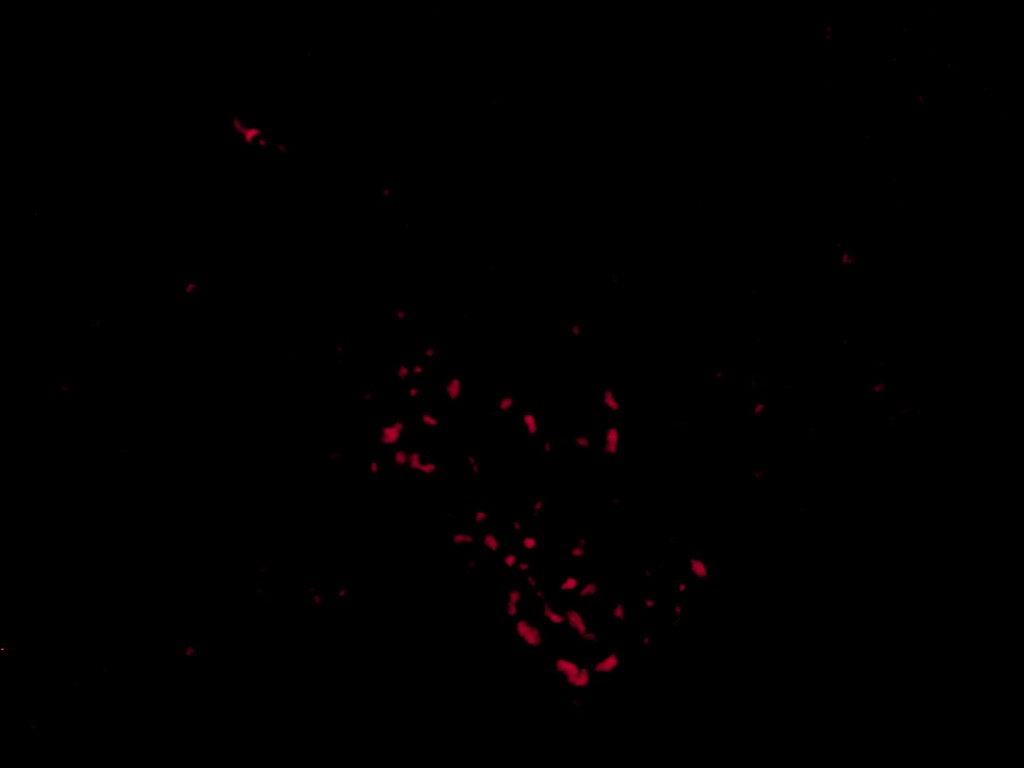

Supplement: Supplementary file 9 — Figure EV1 Source Data [file 44321_2025_315_MOESM9_ESM.zip › Figure EV1/EV1D/3-Claudin-1-GLDC/LEE III/1 (2).jpg]

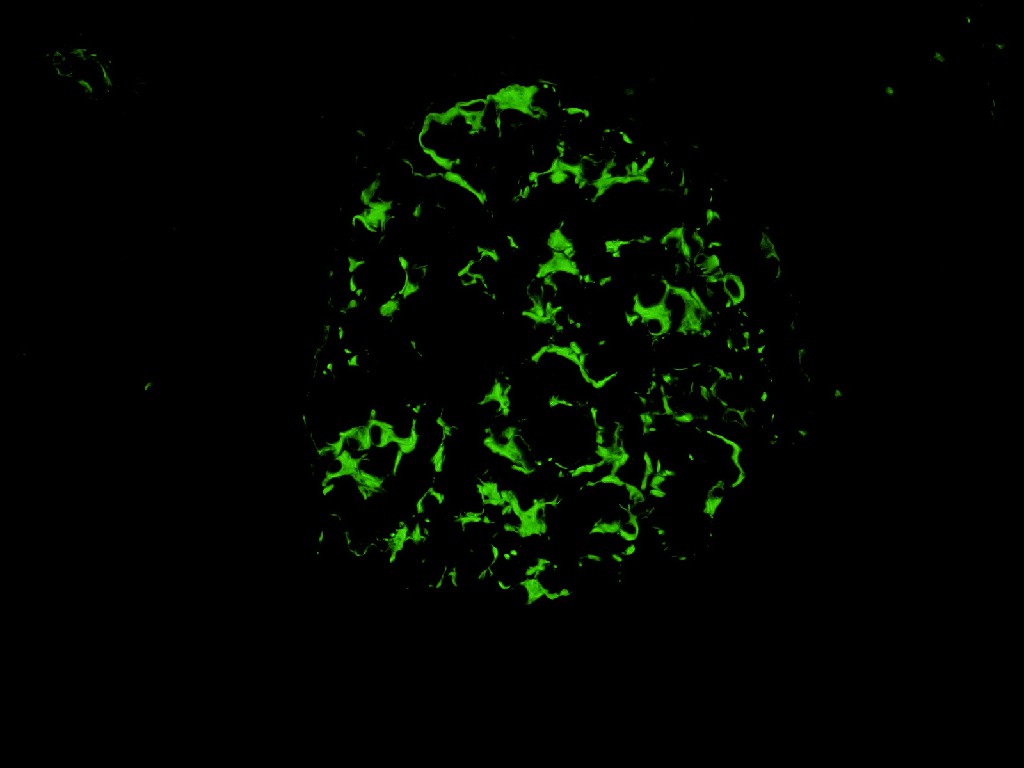

Supplement: Supplementary file 9 — Figure EV1 Source Data [file 44321_2025_315_MOESM9_ESM.zip › Figure EV1/EV1D/3-Claudin-1-GLDC/LEE III/4 (1).jpg]

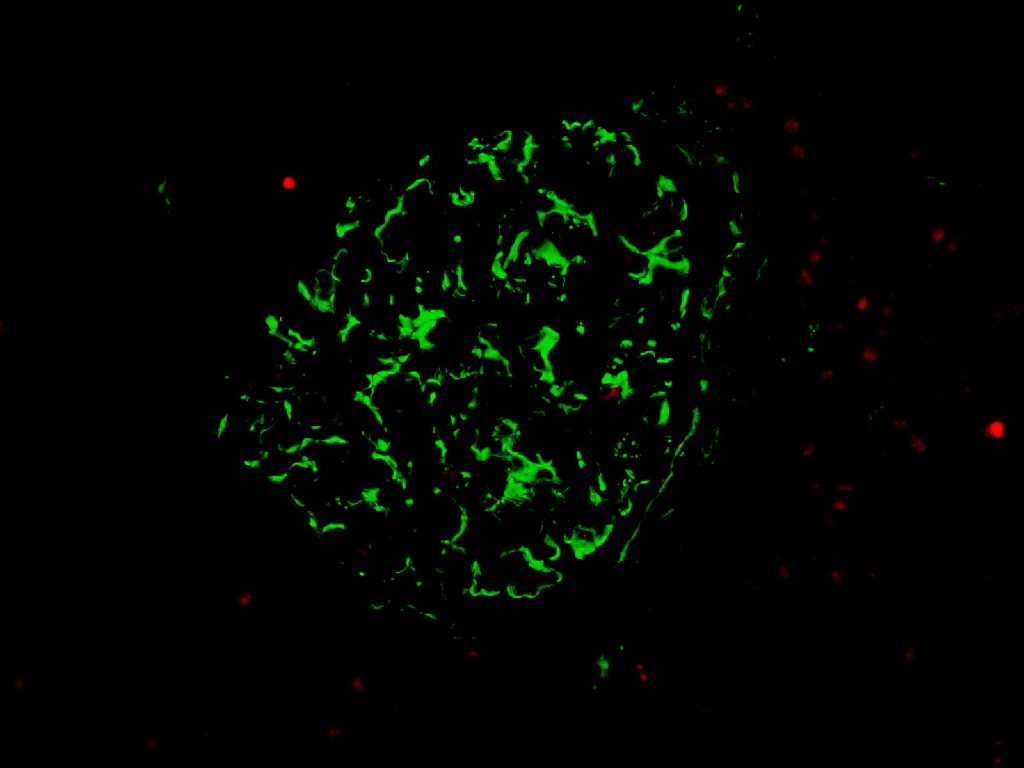

Supplement: Supplementary file 9 — Figure EV1 Source Data [file 44321_2025_315_MOESM9_ESM.zip › Figure EV1/EV1D/3-Claudin-1-GLDC/LEE III/3 (4).jpg]

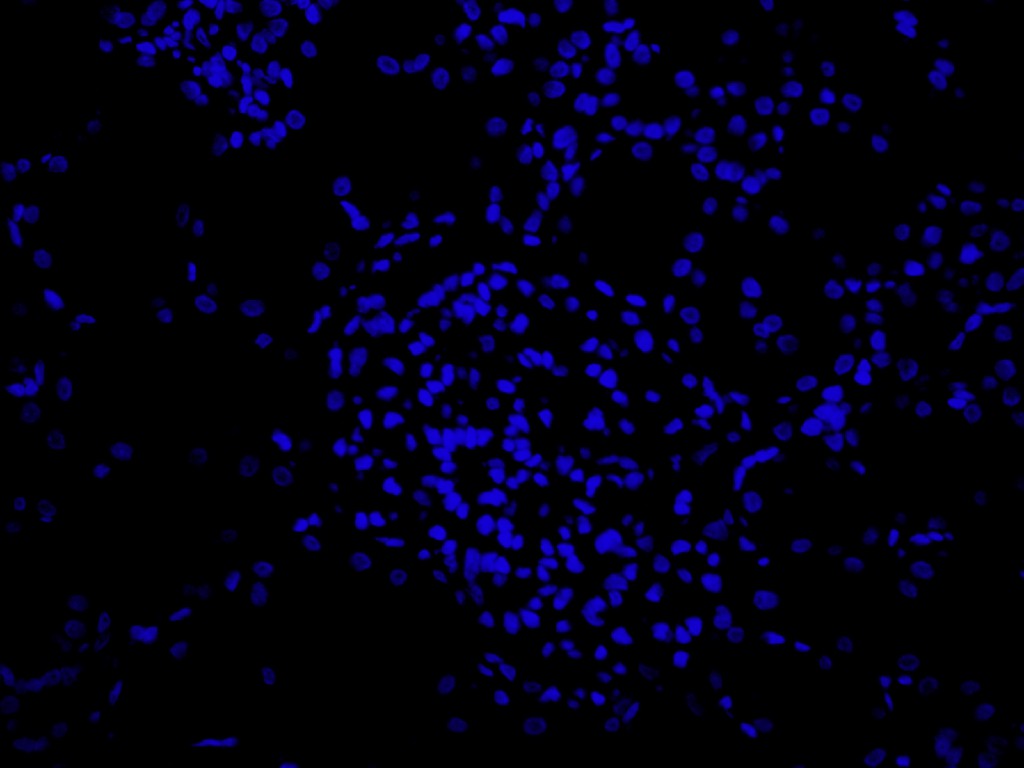

Supplement: Supplementary file 9 — Figure EV1 Source Data [file 44321_2025_315_MOESM9_ESM.zip › Figure EV1/EV1D/3-Claudin-1-GLDC/LEE III/1 (3).jpg]

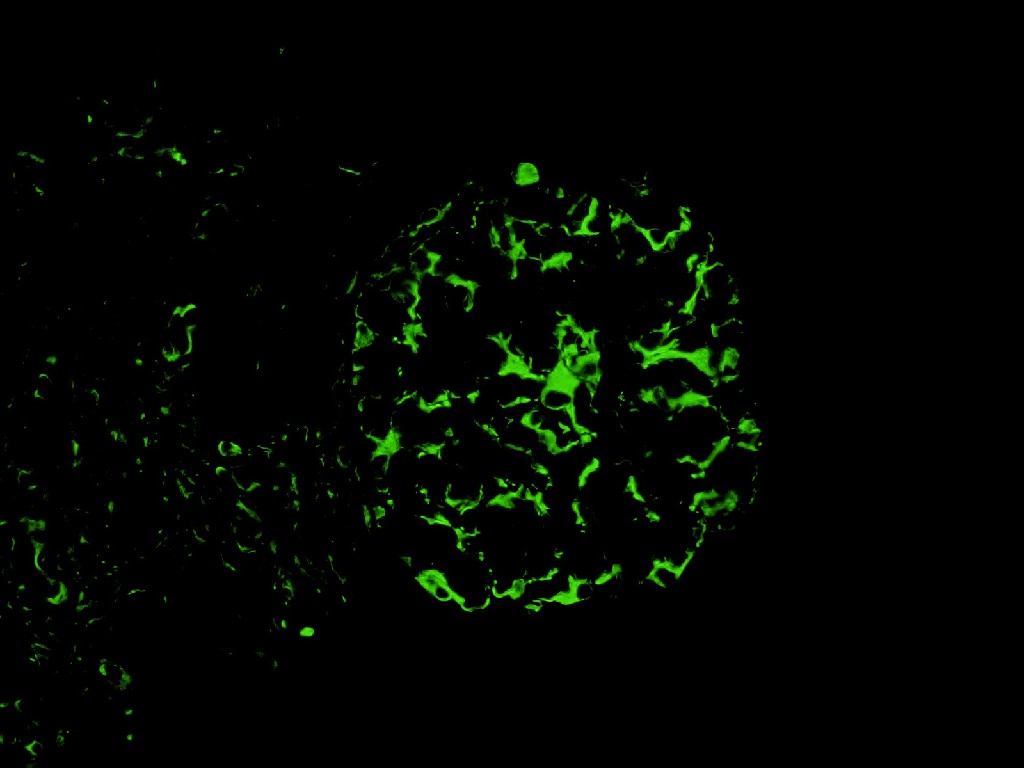

Supplement: Supplementary file 9 — Figure EV1 Source Data [file 44321_2025_315_MOESM9_ESM.zip › Figure EV1/EV1D/3-Claudin-1-GLDC/LEE III/5 (1).jpg]

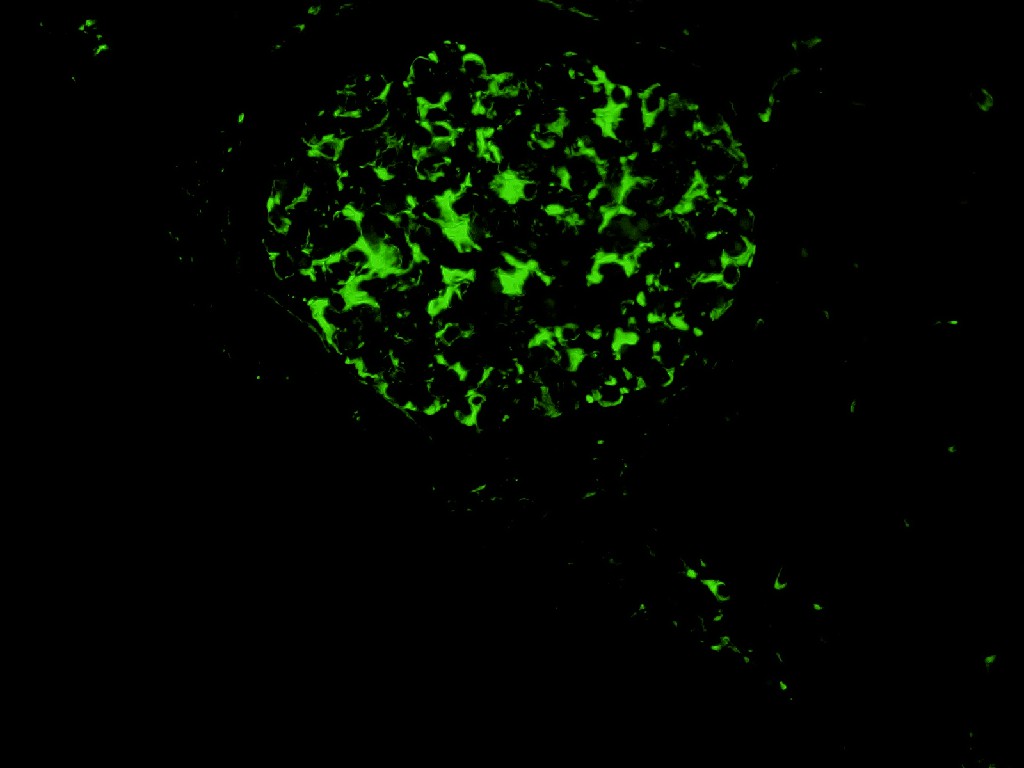

Supplement: Supplementary file 9 — Figure EV1 Source Data [file 44321_2025_315_MOESM9_ESM.zip › Figure EV1/EV1D/3-Claudin-1-GLDC/LEE III/2 (2).jpg]

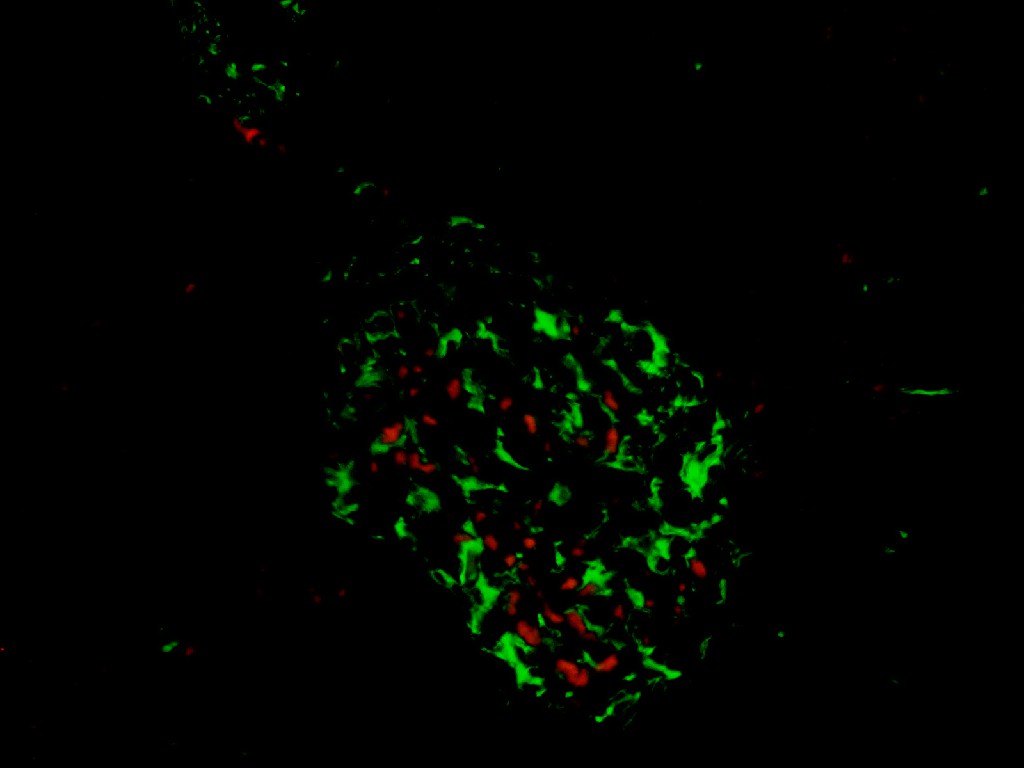

Supplement: Supplementary file 9 — Figure EV1 Source Data [file 44321_2025_315_MOESM9_ESM.zip › Figure EV1/EV1D/3-Claudin-1-GLDC/LEE III/1 (4).jpg]

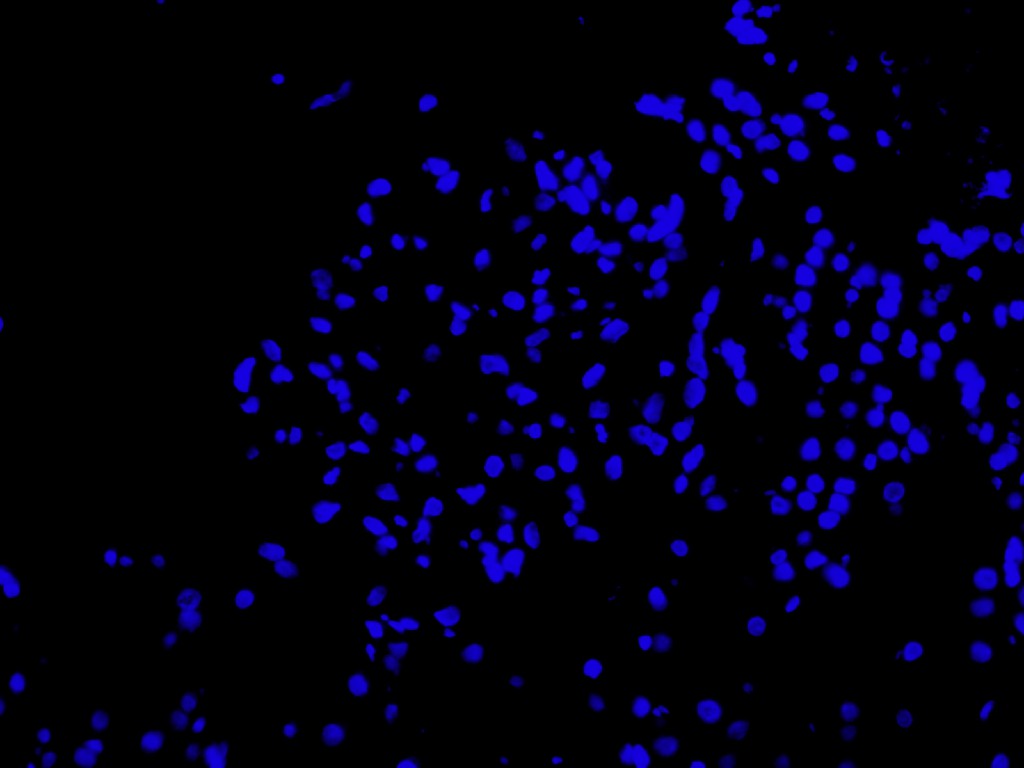

Supplement: Supplementary file 9 — Figure EV1 Source Data [file 44321_2025_315_MOESM9_ESM.zip › Figure EV1/EV1D/3-Claudin-1-GLDC/LEE III/3 (3).jpg]

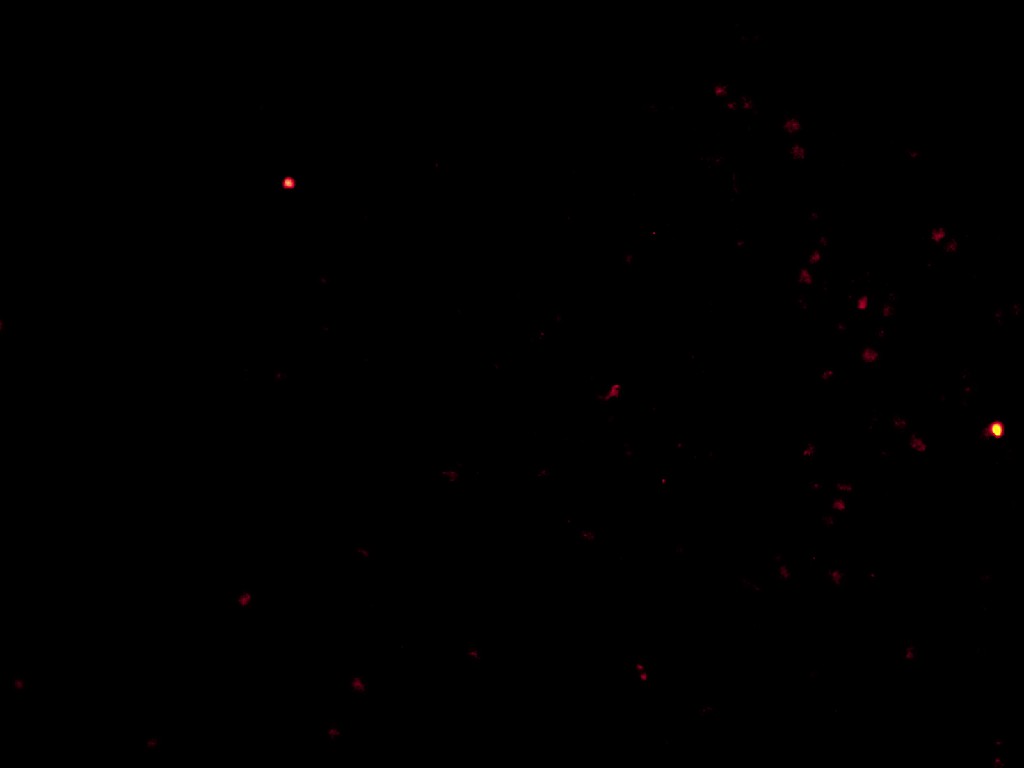

Supplement: Supplementary file 9 — Figure EV1 Source Data [file 44321_2025_315_MOESM9_ESM.zip › Figure EV1/EV1D/3-Claudin-1-GLDC/LEE III/3 (2).jpg]

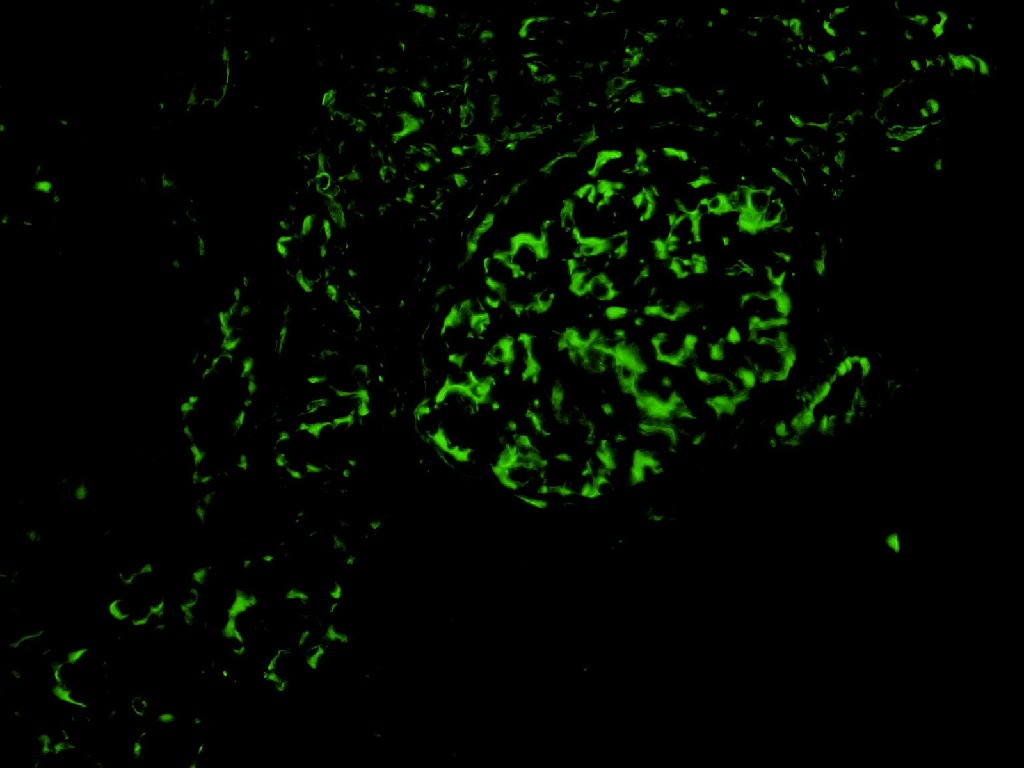

Supplement: Supplementary file 9 — Figure EV1 Source Data [file 44321_2025_315_MOESM9_ESM.zip › Figure EV1/EV1D/3-Claudin-1-GLDC/LEE III/6 (1).jpg]

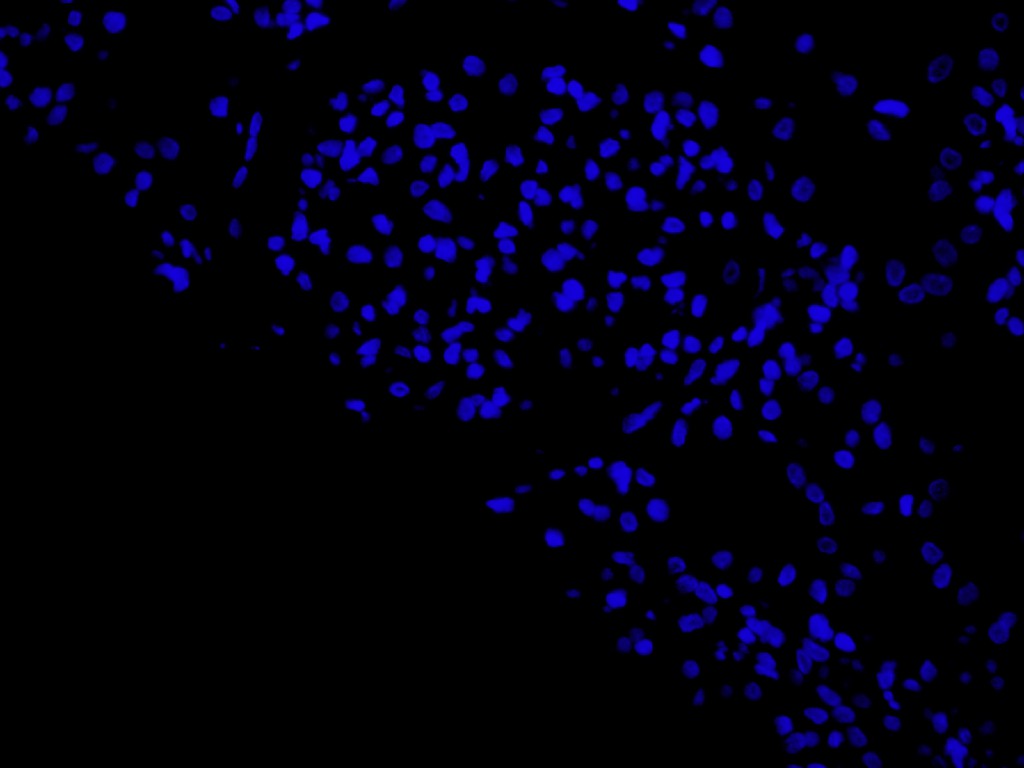

Supplement: Supplementary file 9 — Figure EV1 Source Data [file 44321_2025_315_MOESM9_ESM.zip › Figure EV1/EV1D/3-Claudin-1-GLDC/LEE III/2 (3).jpg]

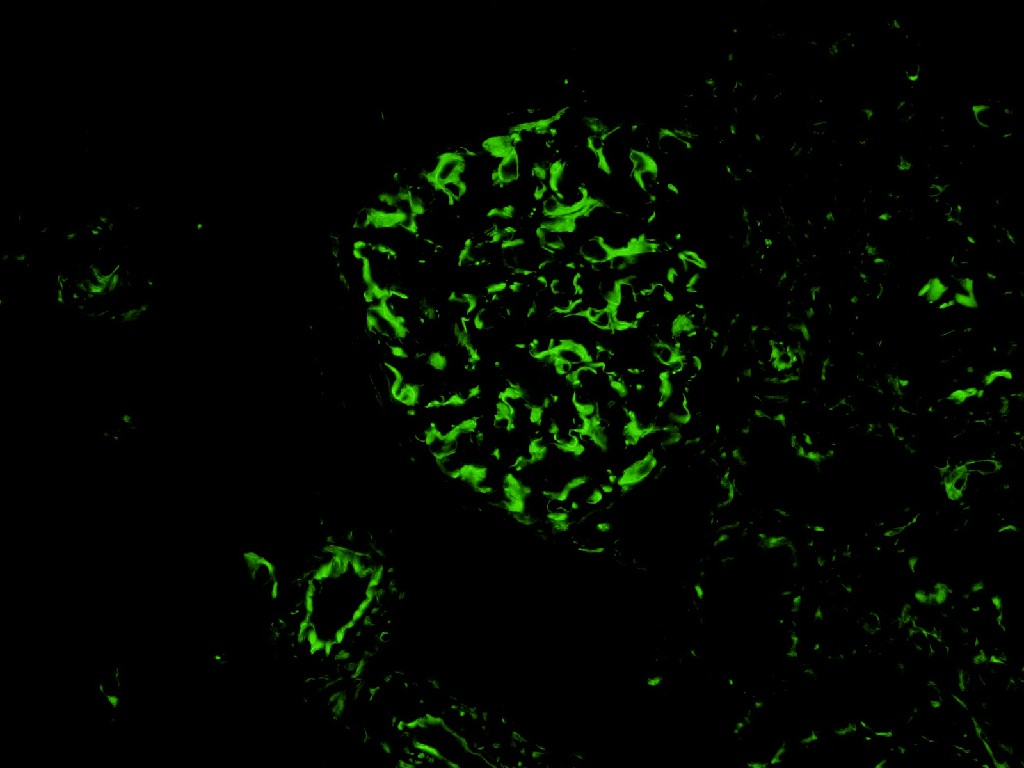

Supplement: Supplementary file 9 — Figure EV1 Source Data [file 44321_2025_315_MOESM9_ESM.zip › Figure EV1/EV1D/3-Claudin-1-GLDC/LEE II/2 (1).jpg]

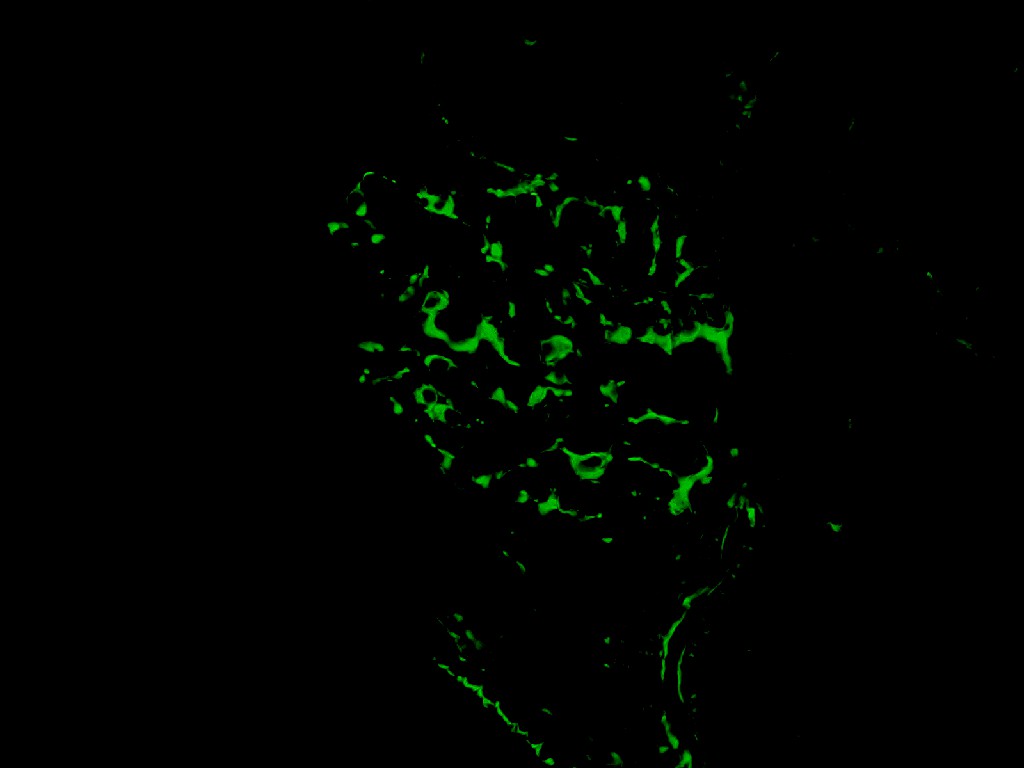

Supplement: Supplementary file 9 — Figure EV1 Source Data [file 44321_2025_315_MOESM9_ESM.zip › Figure EV1/EV1D/3-Claudin-1-GLDC/LEE II/1 (1).jpg]

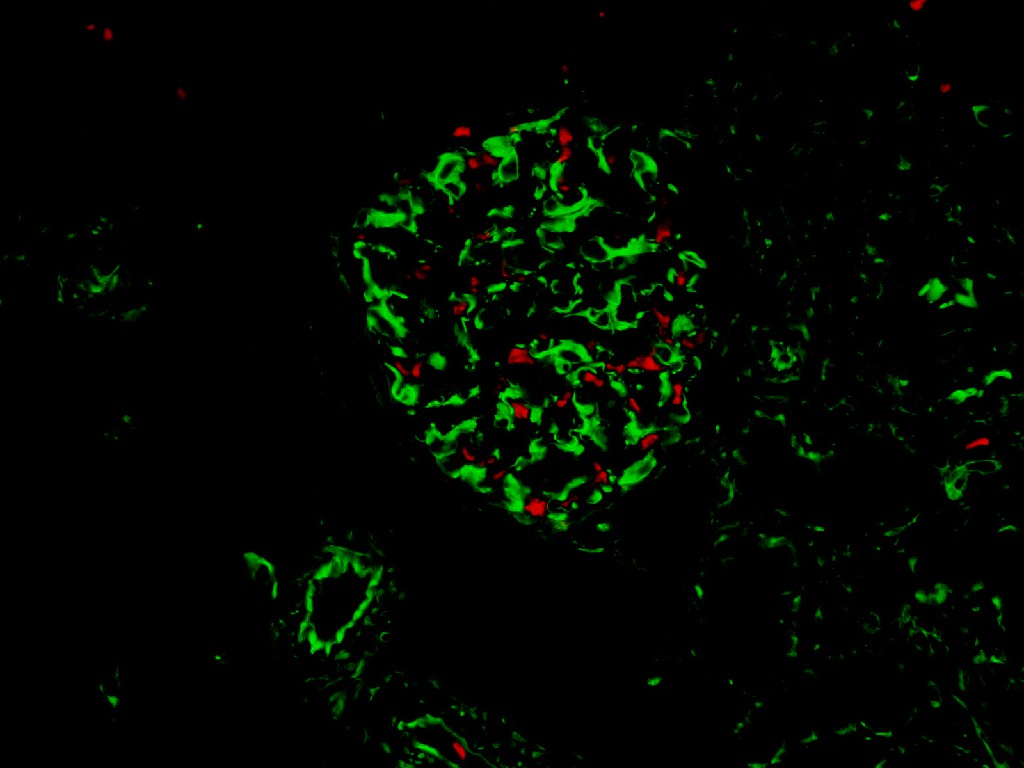

Supplement: Supplementary file 9 — Figure EV1 Source Data [file 44321_2025_315_MOESM9_ESM.zip › Figure EV1/EV1D/3-Claudin-1-GLDC/LEE II/2 (4).jpg]

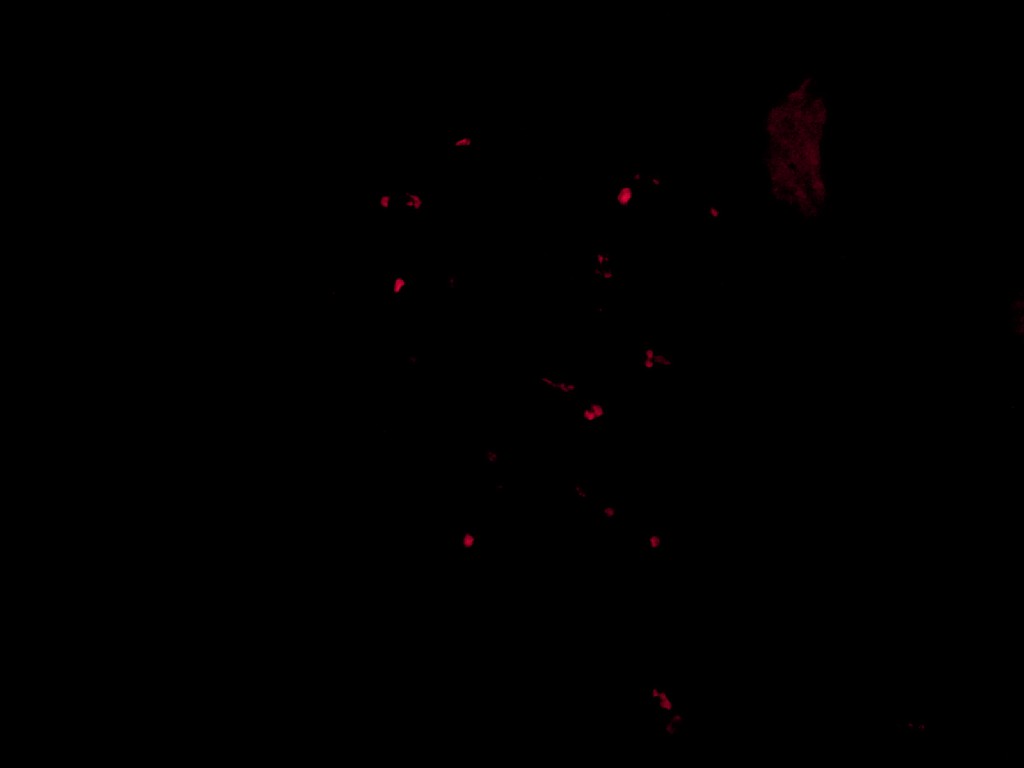

Supplement: Supplementary file 9 — Figure EV1 Source Data [file 44321_2025_315_MOESM9_ESM.zip › Figure EV1/EV1D/3-Claudin-1-GLDC/LEE II/1 (2).jpg]

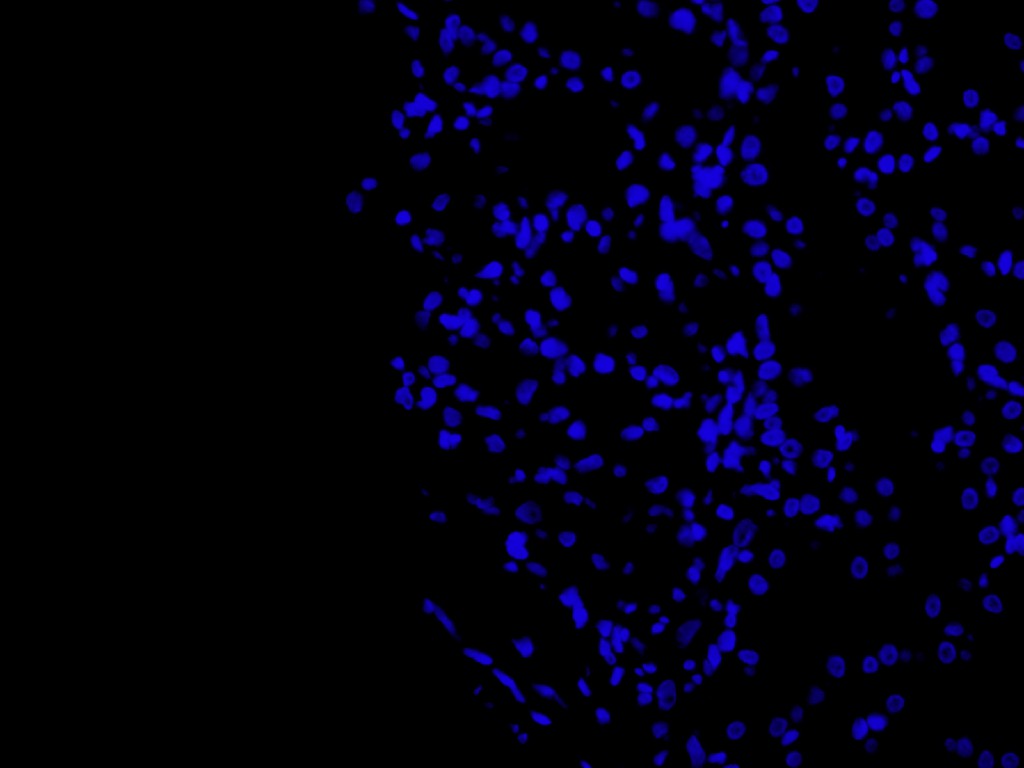

Supplement: Supplementary file 9 — Figure EV1 Source Data [file 44321_2025_315_MOESM9_ESM.zip › Figure EV1/EV1D/3-Claudin-1-GLDC/LEE II/1 (3).jpg]

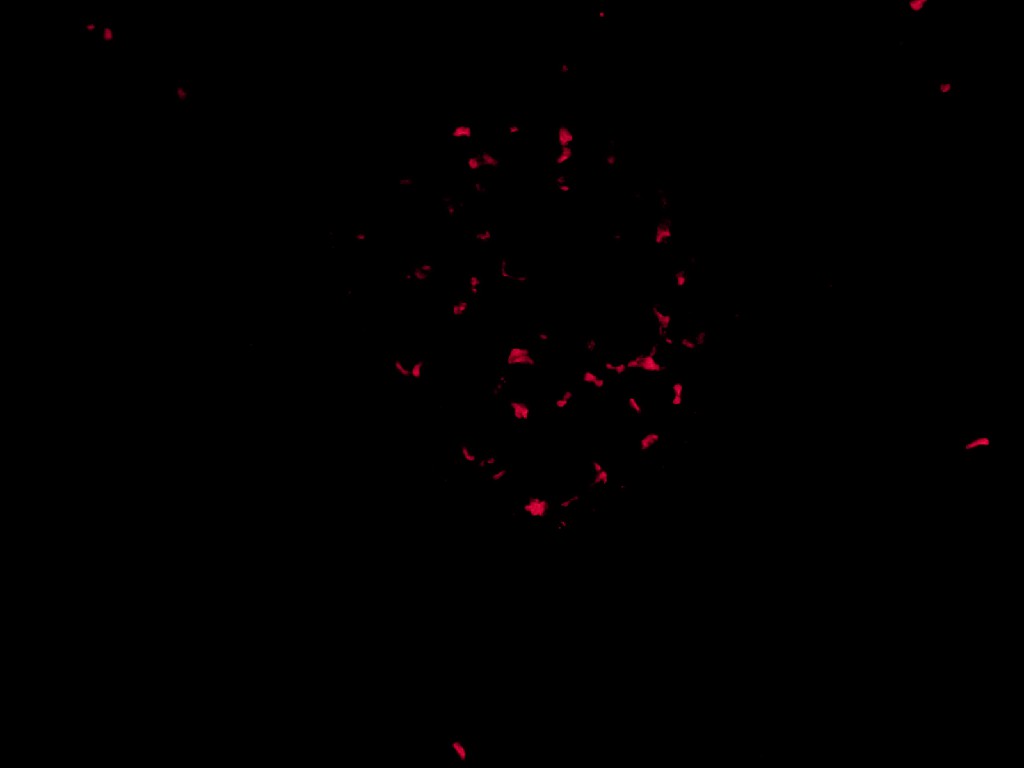

Supplement: Supplementary file 9 — Figure EV1 Source Data [file 44321_2025_315_MOESM9_ESM.zip › Figure EV1/EV1D/3-Claudin-1-GLDC/LEE II/2 (2).jpg]

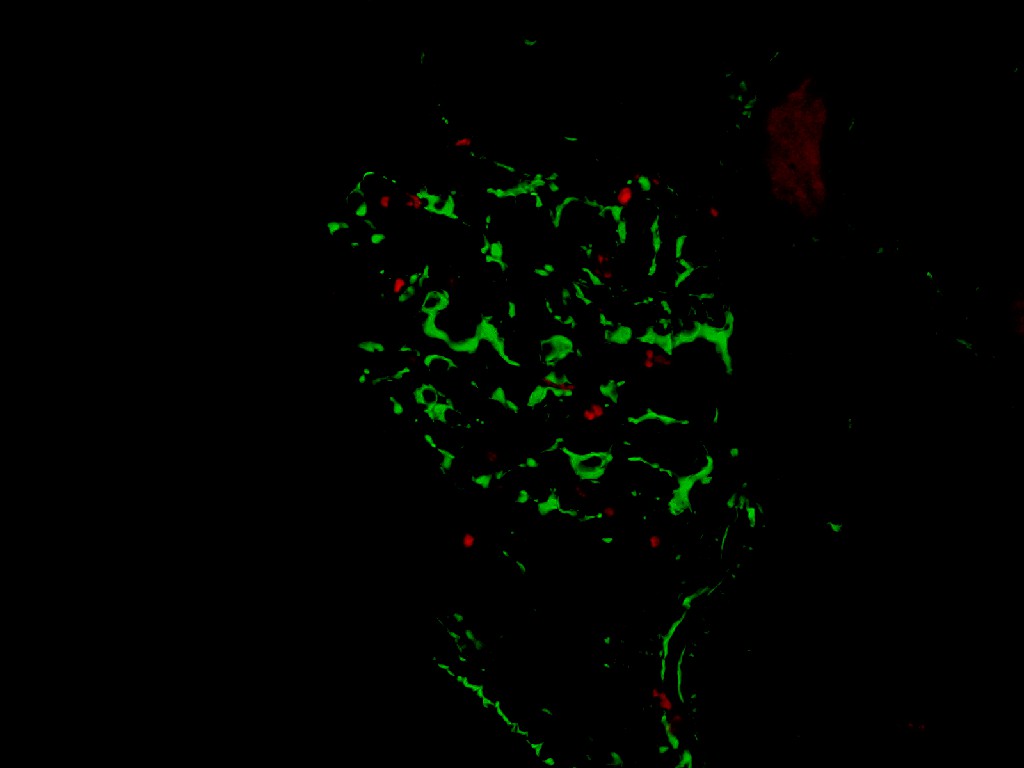

Supplement: Supplementary file 9 — Figure EV1 Source Data [file 44321_2025_315_MOESM9_ESM.zip › Figure EV1/EV1D/3-Claudin-1-GLDC/LEE II/1 (4).jpg]
