# Supplementary figures and images for: Glycine decarboxylase advances IgA nephropathy by boosting mesangial cell proliferation through the pyrimidine pathway (part 4 of 7)
Source: EMBO Mol Med. 2025 Oct 13;17(11):3039–63. doi: 10.1038/s44321-025-00315-2 (PMC12603144; doi:10.1038/s44321-025-00315-2)

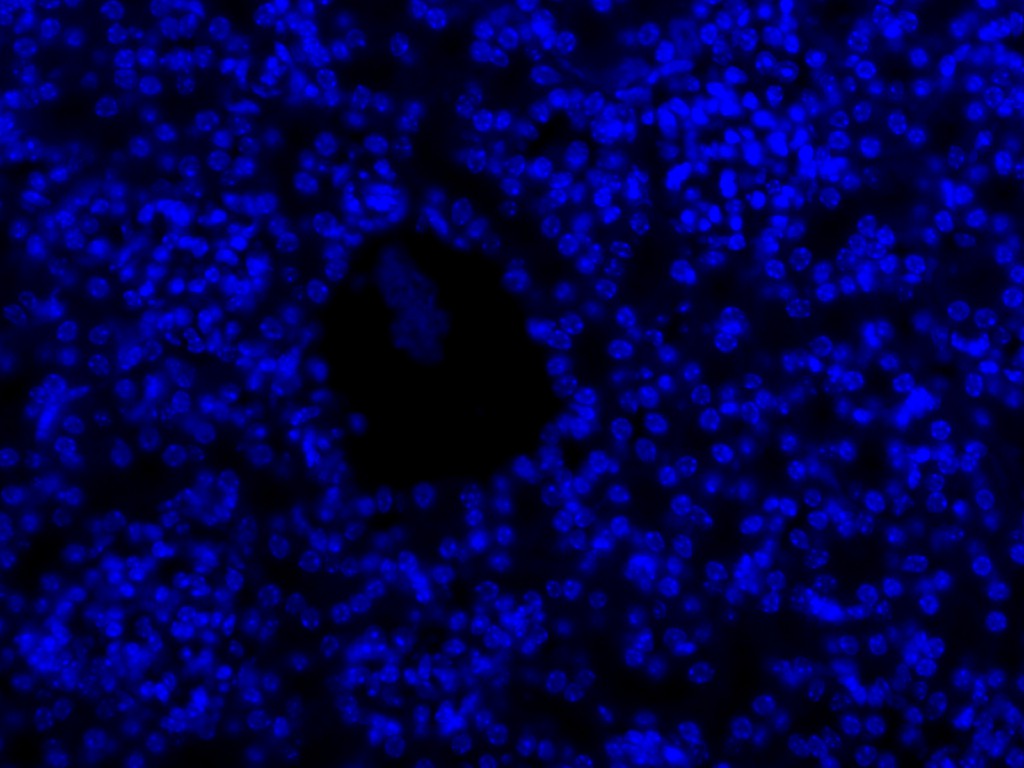

Supplement: Supplementary file 7 — Source data Fig. 6 [file 44321_2025_315_MOESM7_ESM.zip › Figure 6/F6A/1-IgA/3-2 (1).jpg]

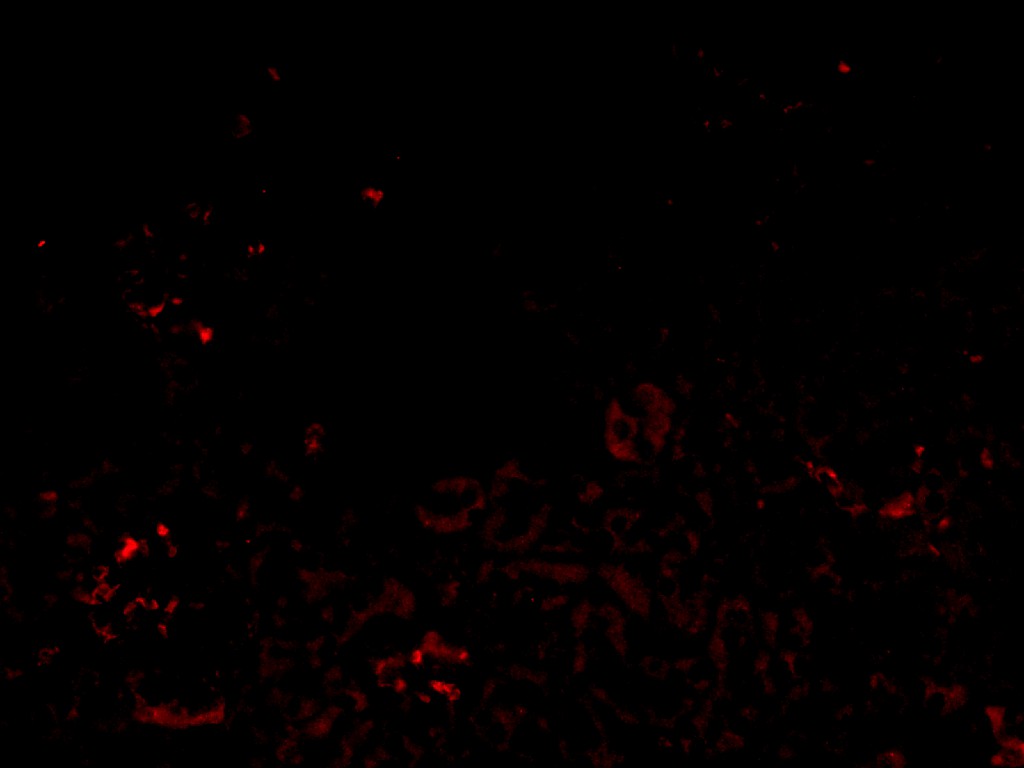

Supplement: Supplementary file 7 — Source data Fig. 6 [file 44321_2025_315_MOESM7_ESM.zip › Figure 6/F6A/1-IgA/3-2 (2).jpg]

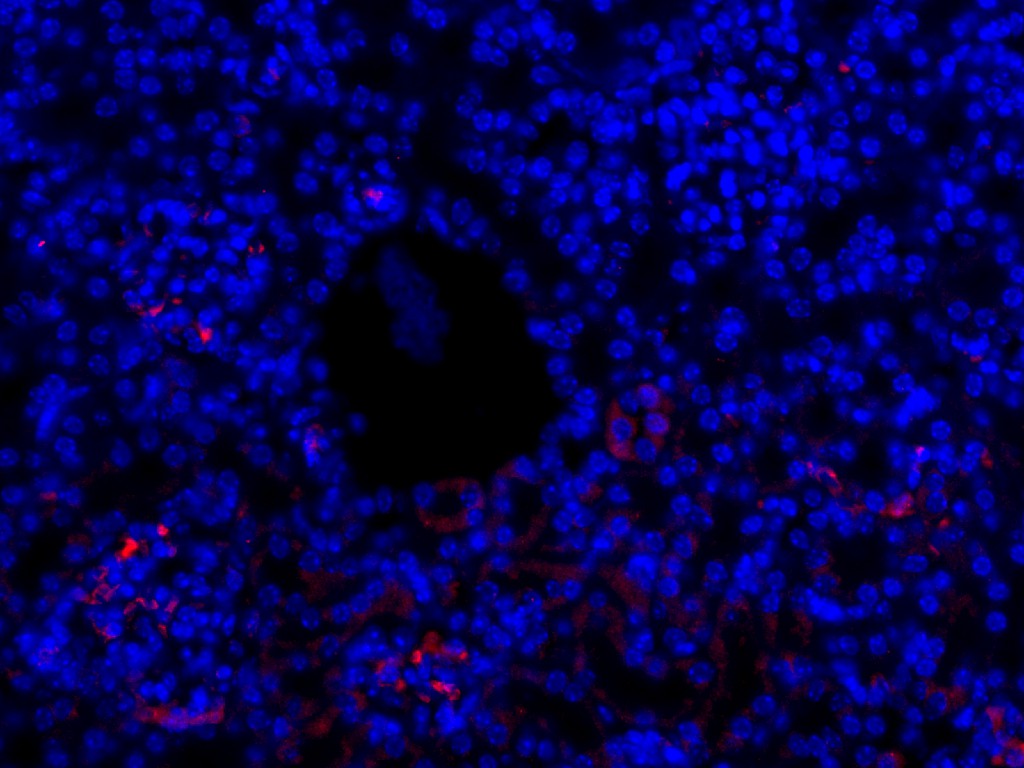

Supplement: Supplementary file 7 — Source data Fig. 6 [file 44321_2025_315_MOESM7_ESM.zip › Figure 6/F6A/1-IgA/3-2 (3).jpg]

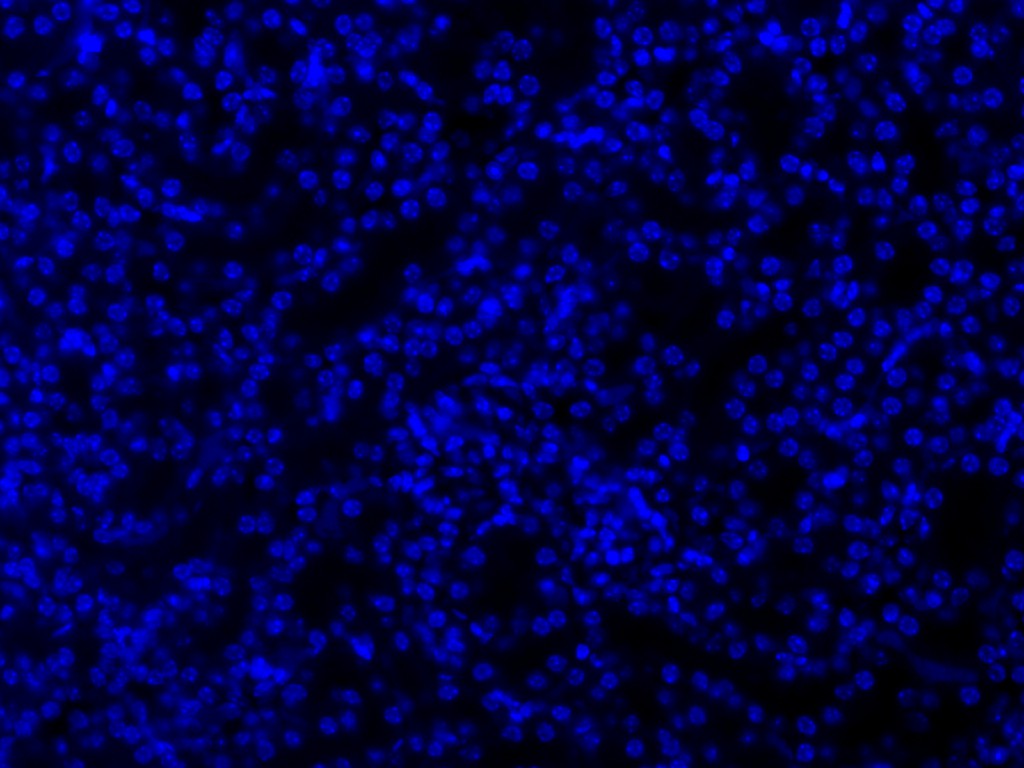

Supplement: Supplementary file 7 — Source data Fig. 6 [file 44321_2025_315_MOESM7_ESM.zip › Figure 6/F6A/1-IgA/3-3 (1).jpg]

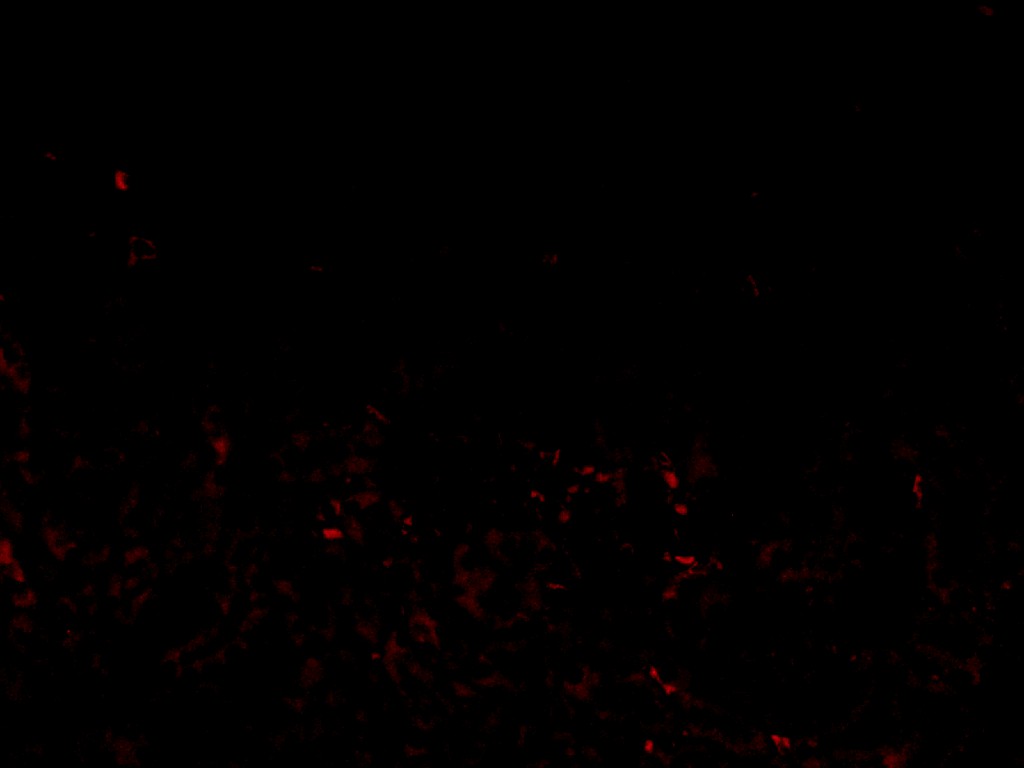

Supplement: Supplementary file 7 — Source data Fig. 6 [file 44321_2025_315_MOESM7_ESM.zip › Figure 6/F6A/1-IgA/3-3 (2).jpg]

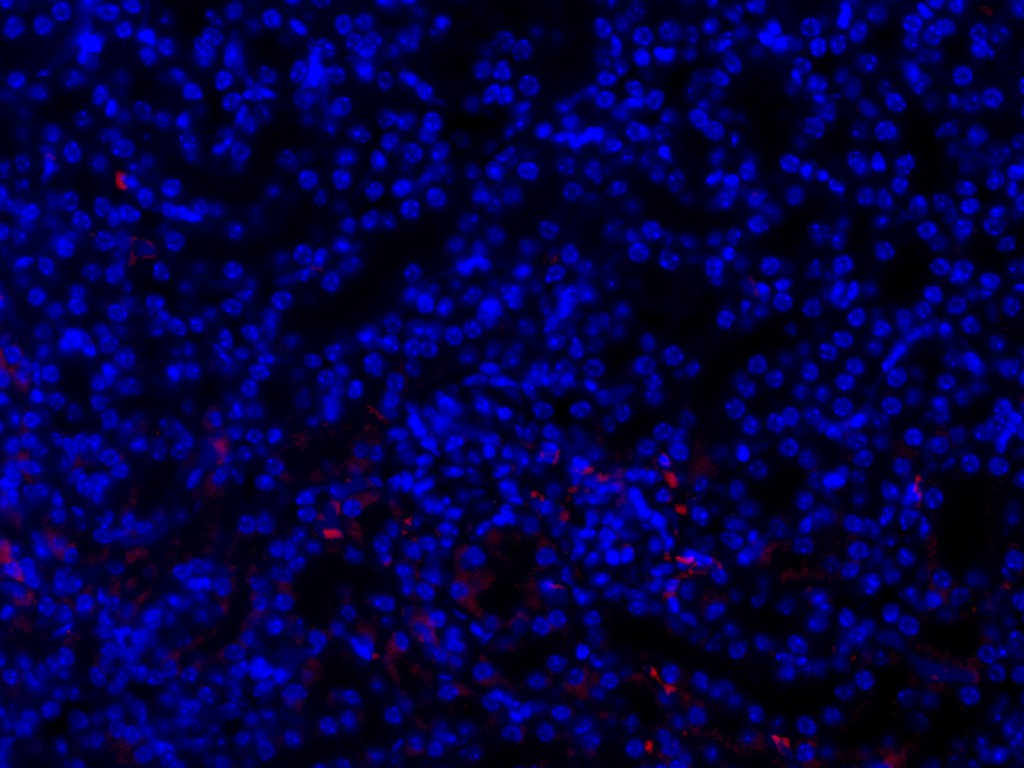

Supplement: Supplementary file 7 — Source data Fig. 6 [file 44321_2025_315_MOESM7_ESM.zip › Figure 6/F6A/1-IgA/3-3 (3).jpg]

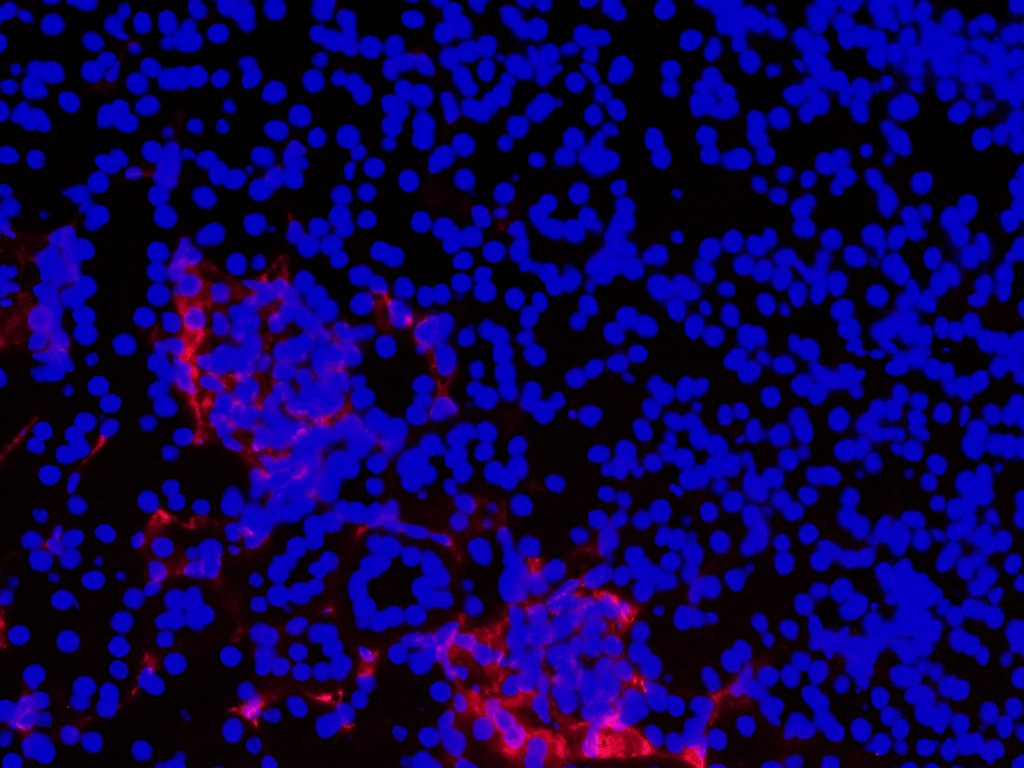

Supplement: Supplementary file 7 — Source data Fig. 6 [file 44321_2025_315_MOESM7_ESM.zip › Figure 6/F6A/1-IgA/3-4 (1).jpg]

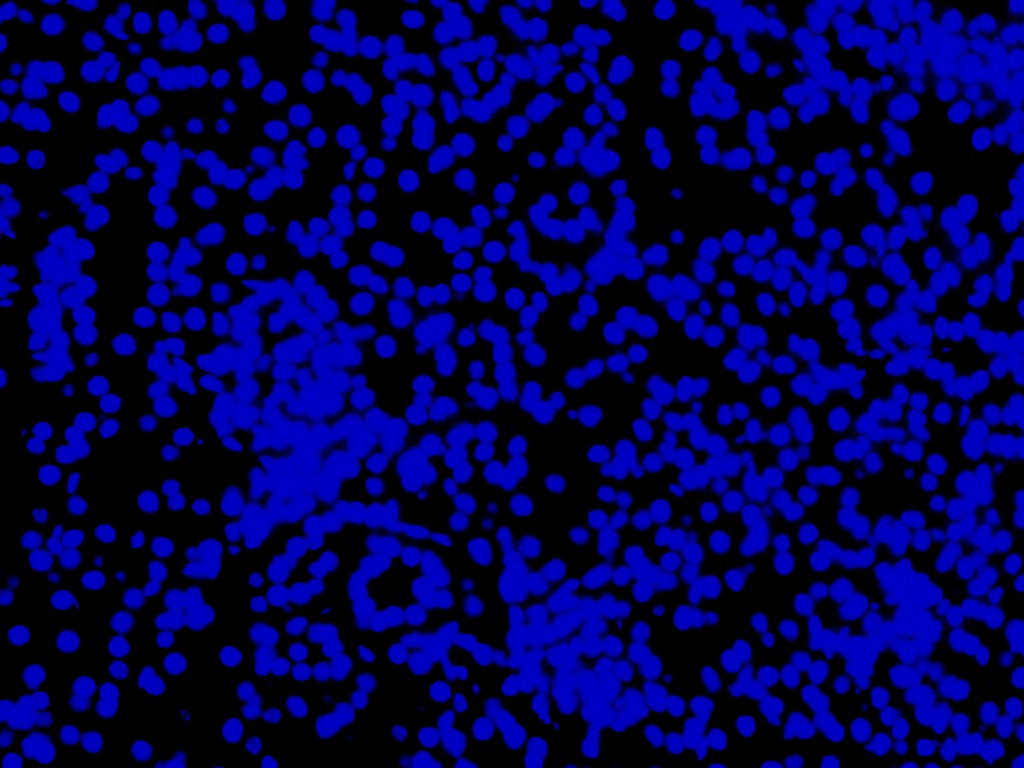

Supplement: Supplementary file 7 — Source data Fig. 6 [file 44321_2025_315_MOESM7_ESM.zip › Figure 6/F6A/1-IgA/3-4 (2).jpg]

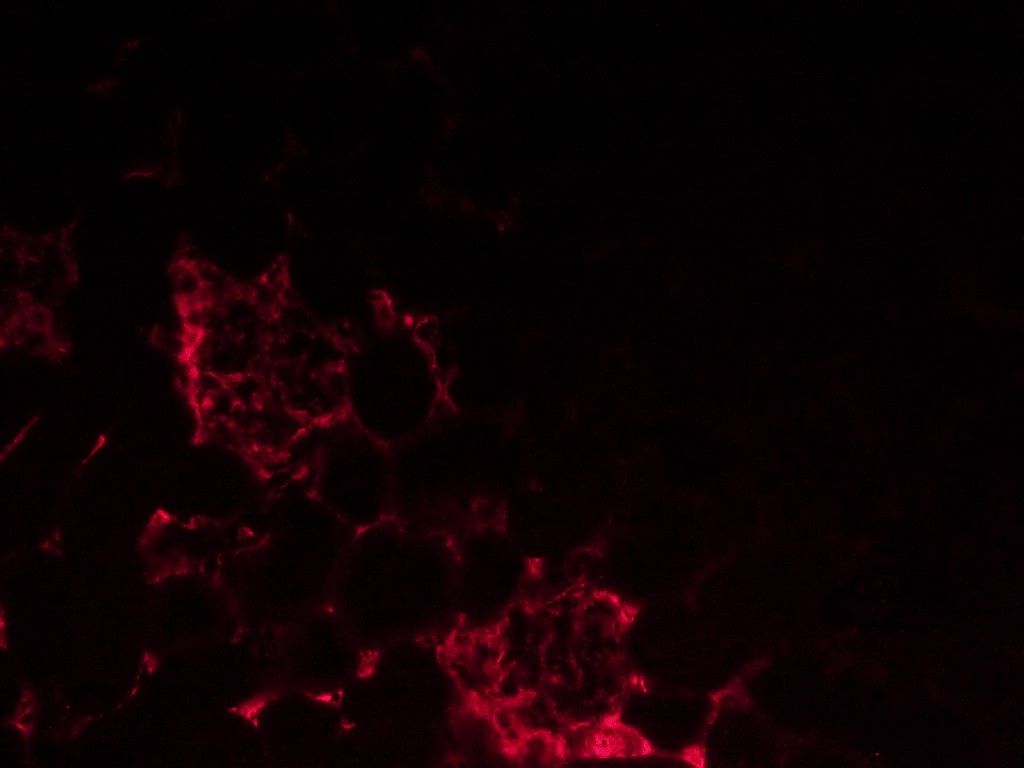

Supplement: Supplementary file 7 — Source data Fig. 6 [file 44321_2025_315_MOESM7_ESM.zip › Figure 6/F6A/1-IgA/3-4 (3).jpg]

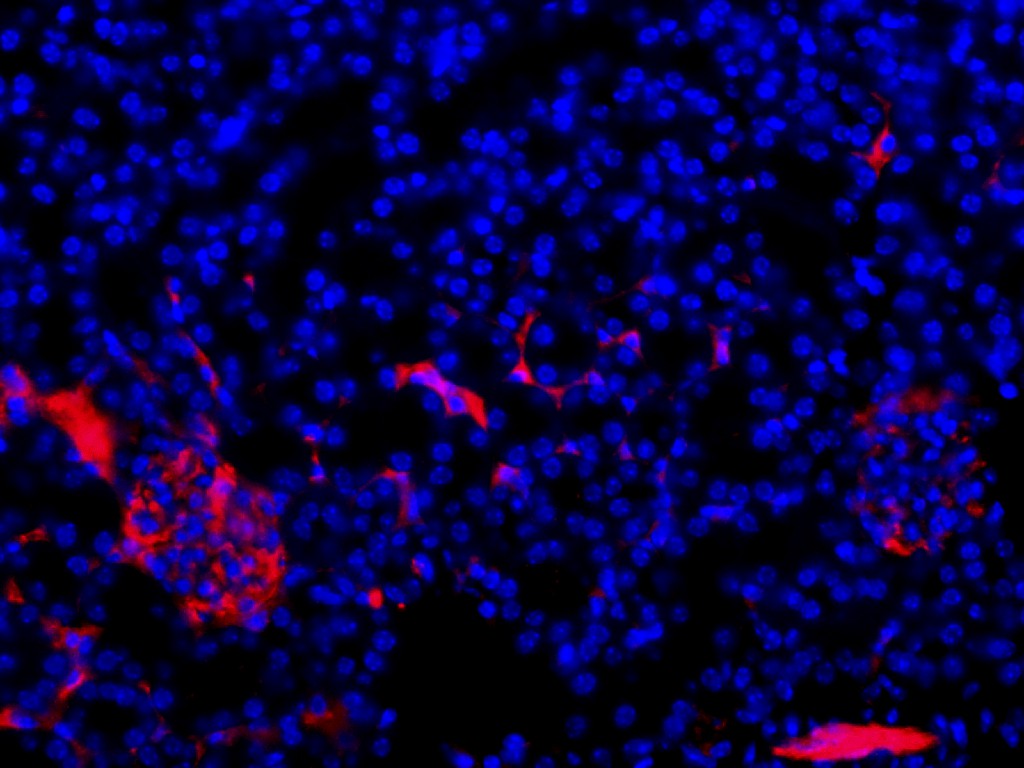

Supplement: Supplementary file 7 — Source data Fig. 6 [file 44321_2025_315_MOESM7_ESM.zip › Figure 6/F6A/1-IgA/3-5 (1).jpg]

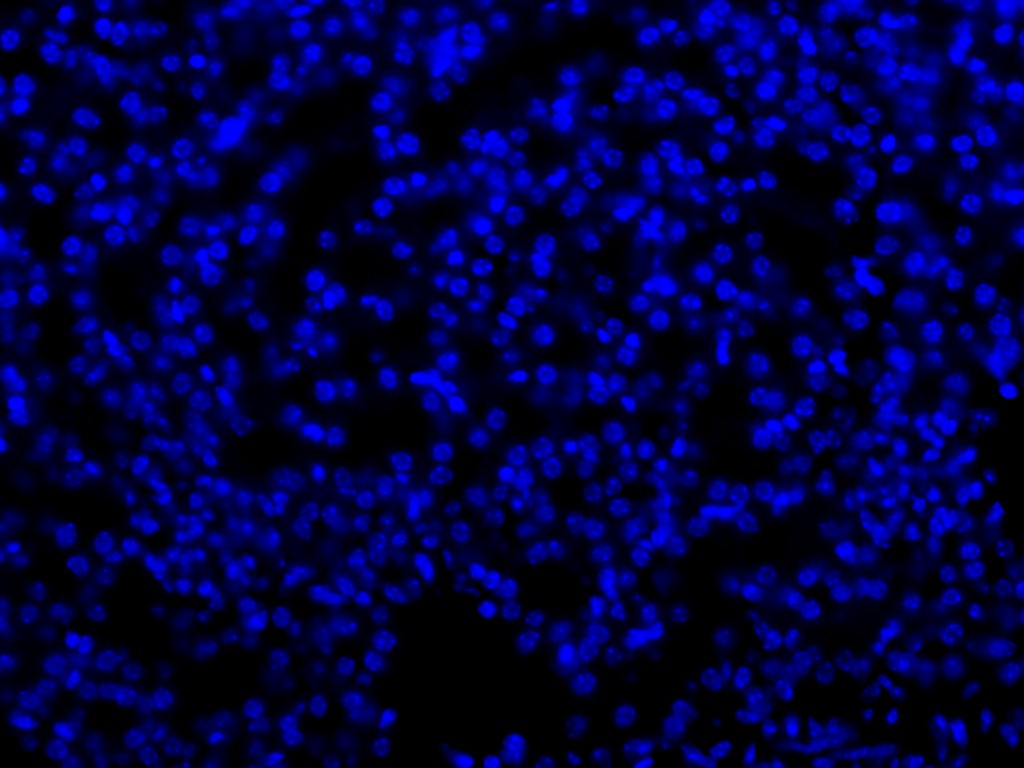

Supplement: Supplementary file 7 — Source data Fig. 6 [file 44321_2025_315_MOESM7_ESM.zip › Figure 6/F6A/1-IgA/3-5 (2).jpg]

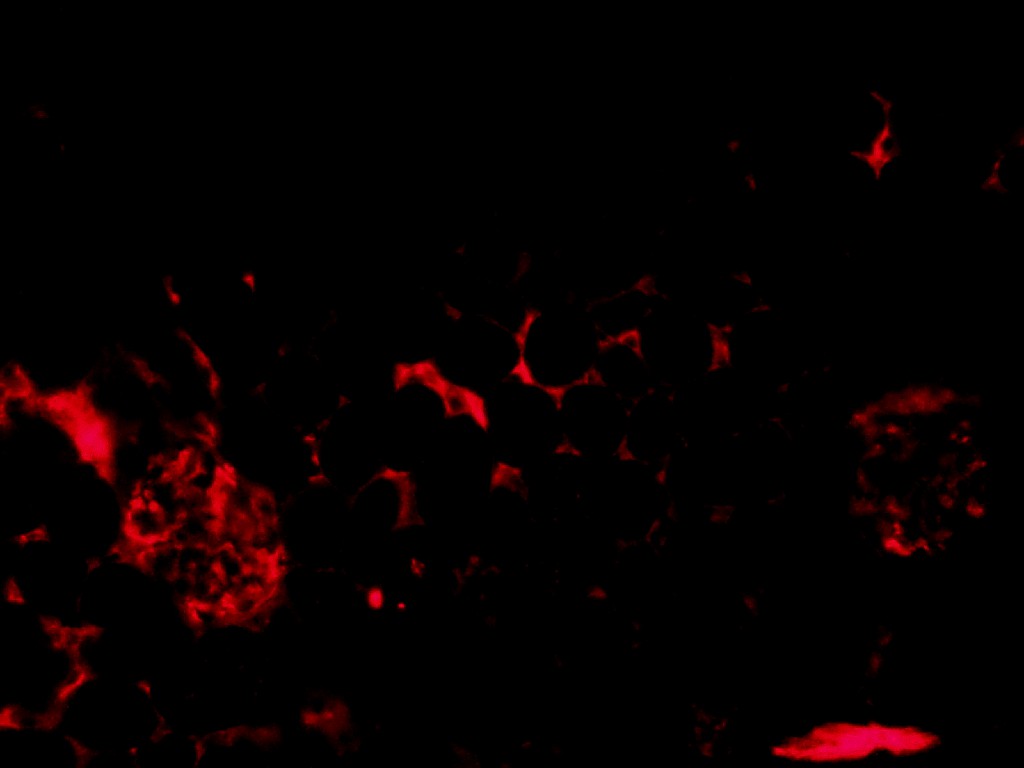

Supplement: Supplementary file 7 — Source data Fig. 6 [file 44321_2025_315_MOESM7_ESM.zip › Figure 6/F6A/1-IgA/3-5 (3).jpg]

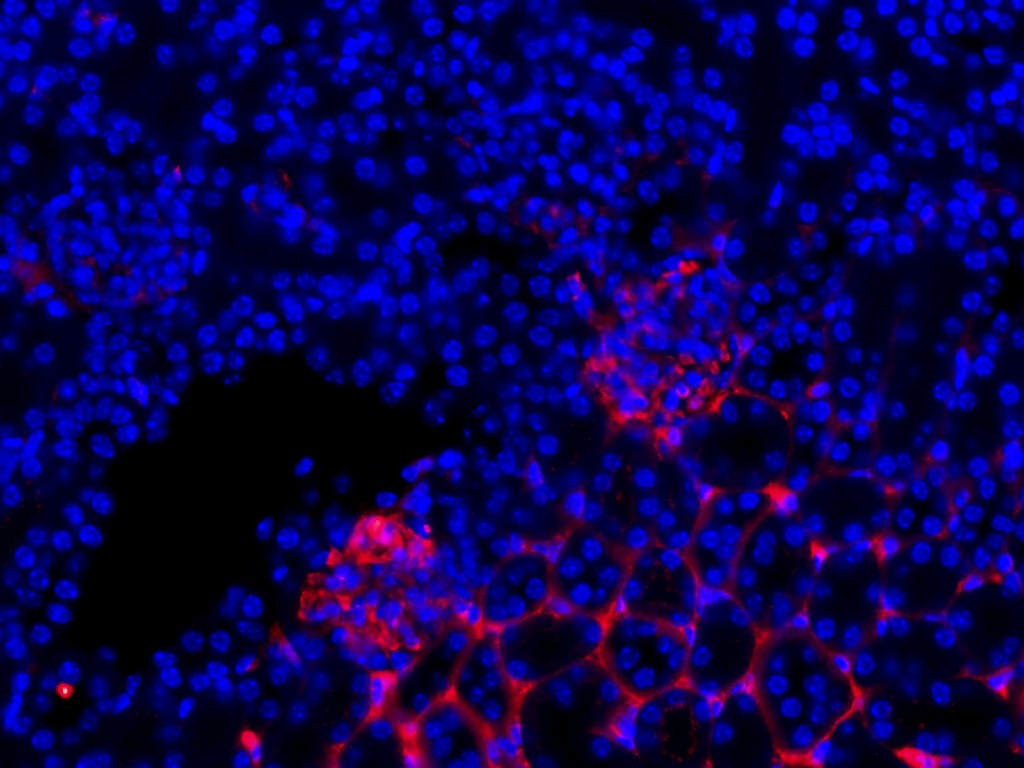

Supplement: Supplementary file 7 — Source data Fig. 6 [file 44321_2025_315_MOESM7_ESM.zip › Figure 6/F6A/1-IgA/3-6 (1).jpg]

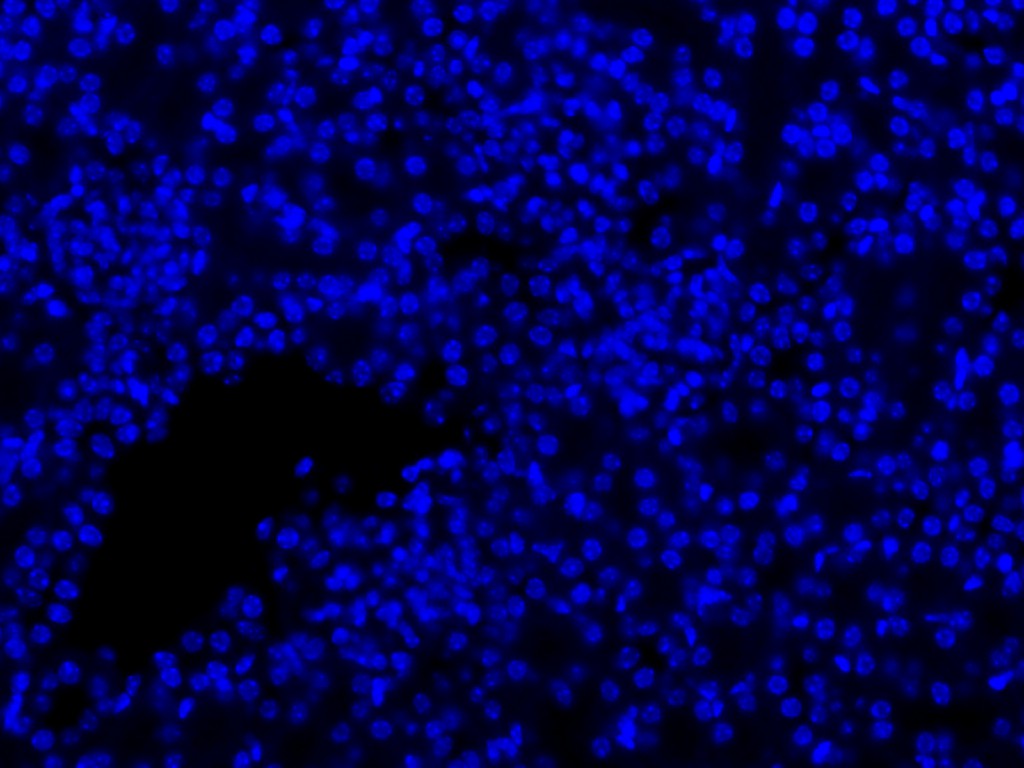

Supplement: Supplementary file 7 — Source data Fig. 6 [file 44321_2025_315_MOESM7_ESM.zip › Figure 6/F6A/1-IgA/3-6 (2).jpg]

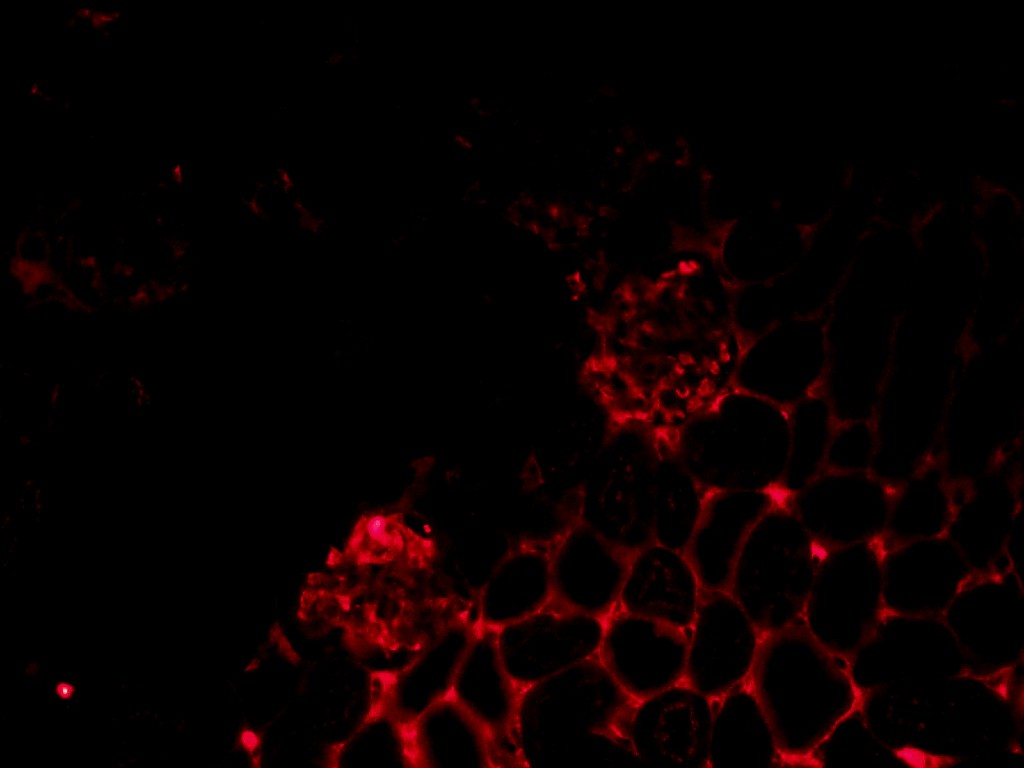

Supplement: Supplementary file 7 — Source data Fig. 6 [file 44321_2025_315_MOESM7_ESM.zip › Figure 6/F6A/1-IgA/3-6 (3).jpg]

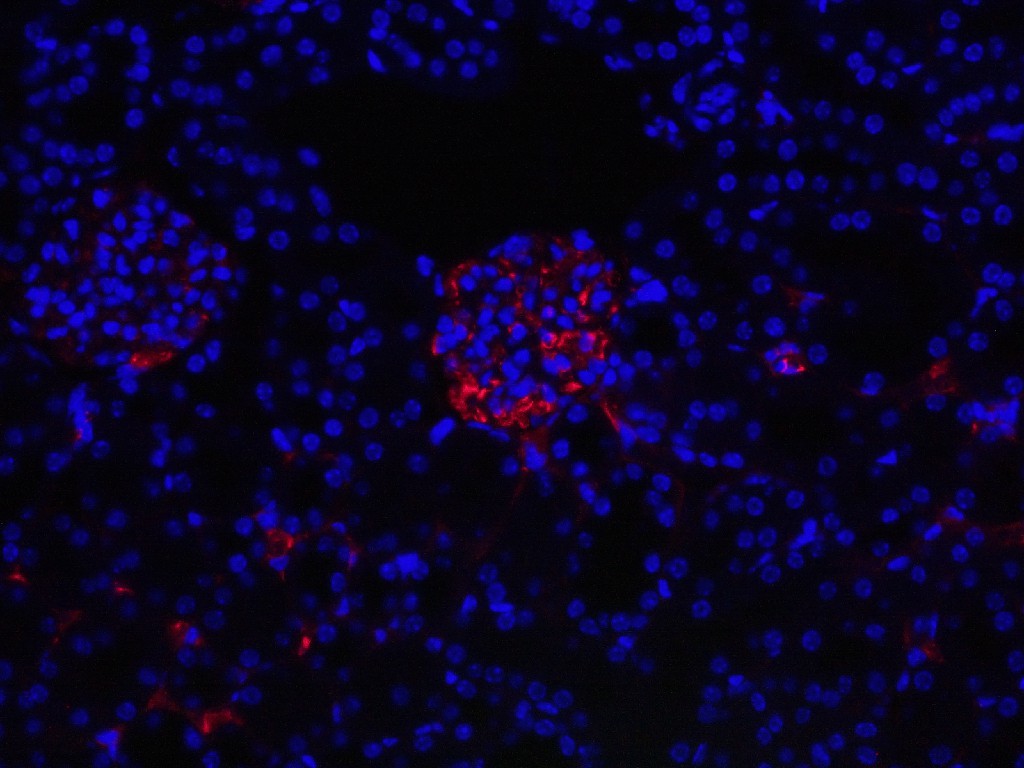

Supplement: Supplementary file 7 — Source data Fig. 6 [file 44321_2025_315_MOESM7_ESM.zip › Figure 6/F6A/1-IgA/4-1 (1).jpg]

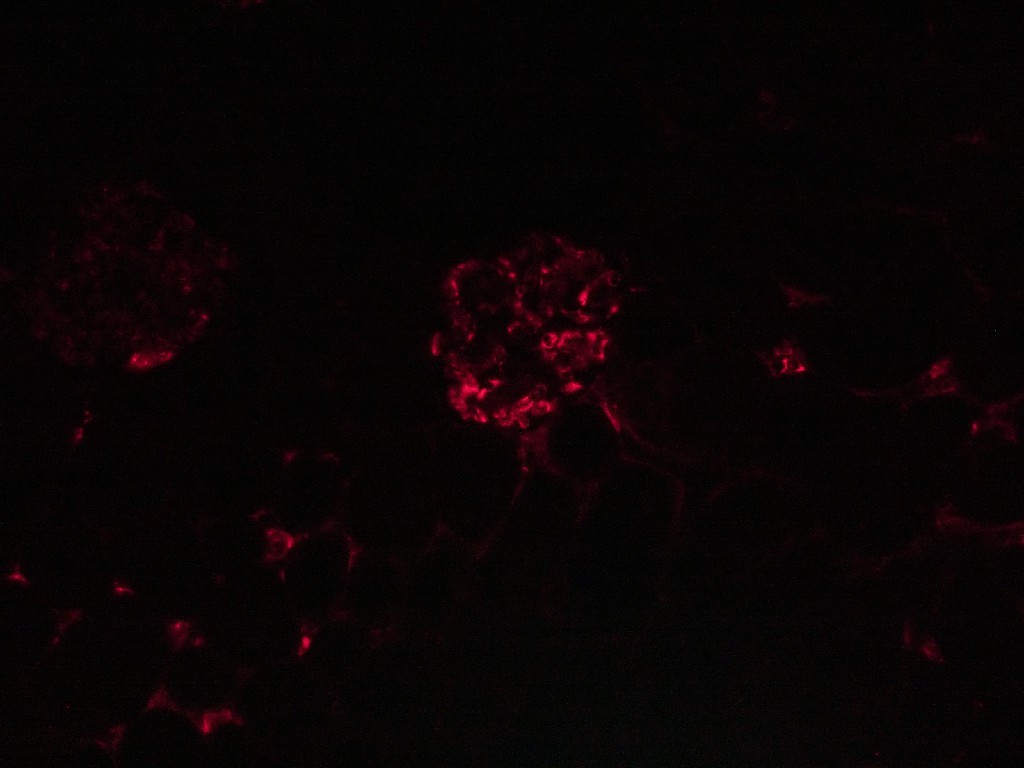

Supplement: Supplementary file 7 — Source data Fig. 6 [file 44321_2025_315_MOESM7_ESM.zip › Figure 6/F6A/1-IgA/4-1 (2).jpg]

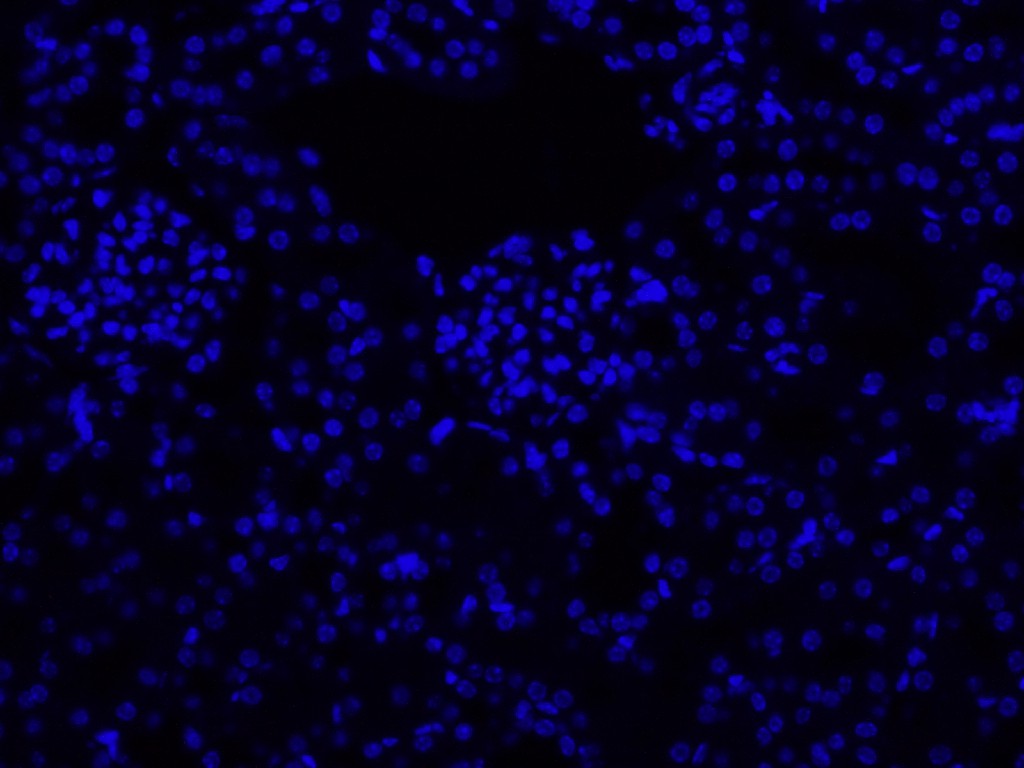

Supplement: Supplementary file 7 — Source data Fig. 6 [file 44321_2025_315_MOESM7_ESM.zip › Figure 6/F6A/1-IgA/4-1 (3).jpg]

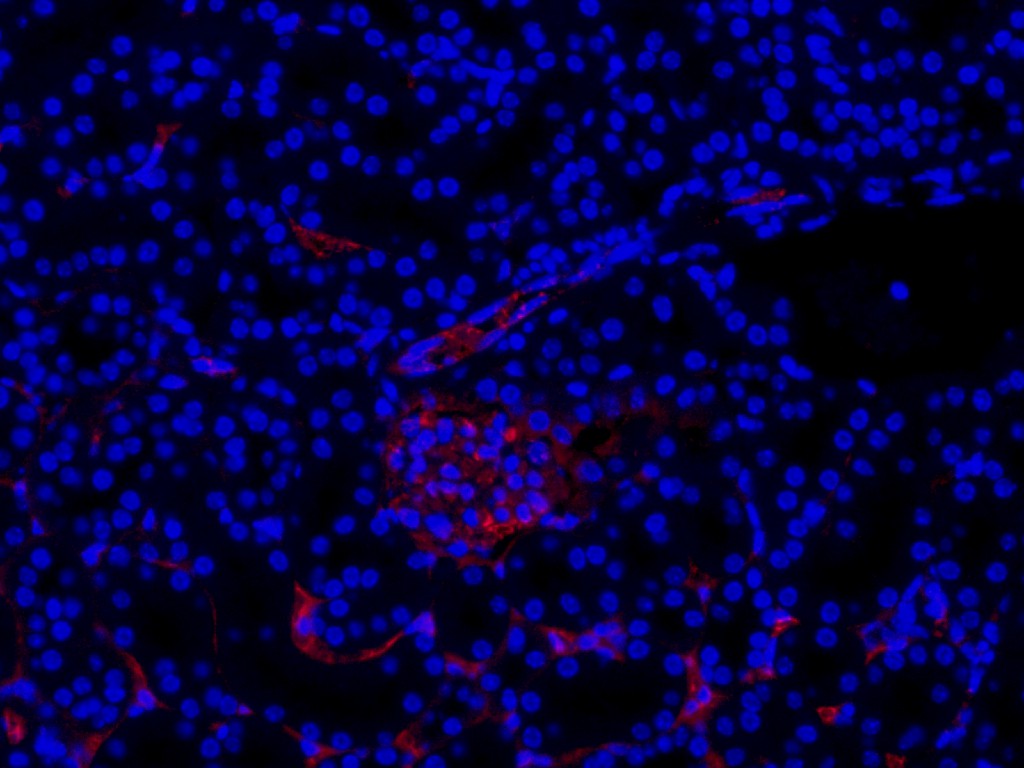

Supplement: Supplementary file 7 — Source data Fig. 6 [file 44321_2025_315_MOESM7_ESM.zip › Figure 6/F6A/1-IgA/4-2 (1).jpg]

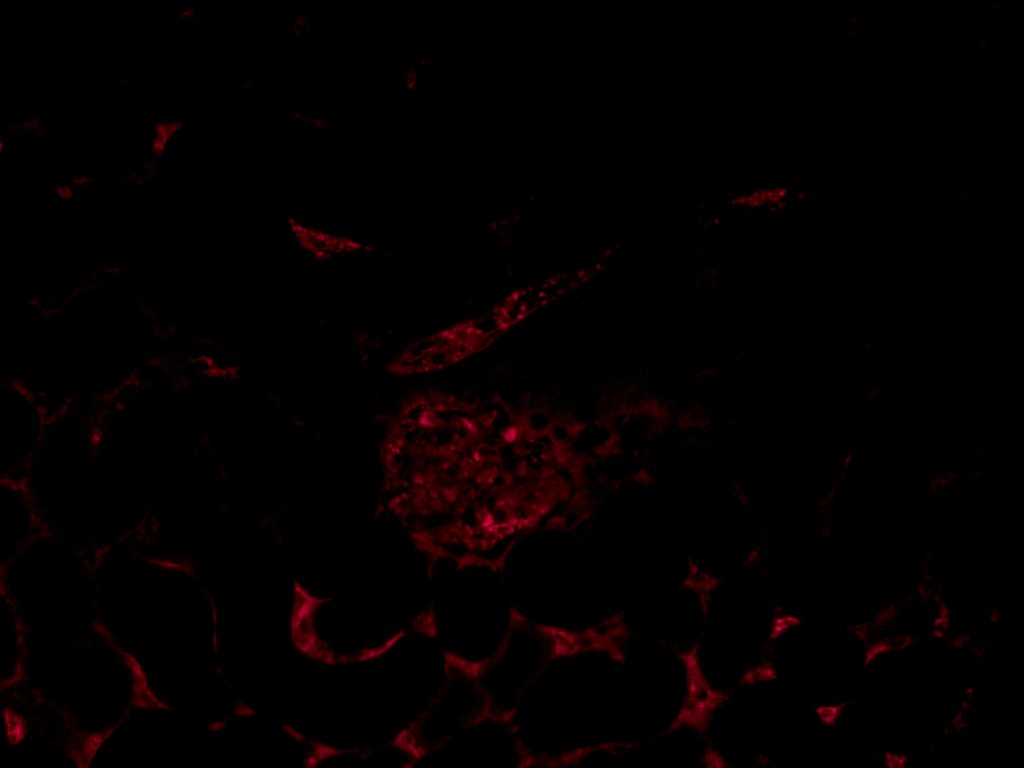

Supplement: Supplementary file 7 — Source data Fig. 6 [file 44321_2025_315_MOESM7_ESM.zip › Figure 6/F6A/1-IgA/4-2 (2).jpg]

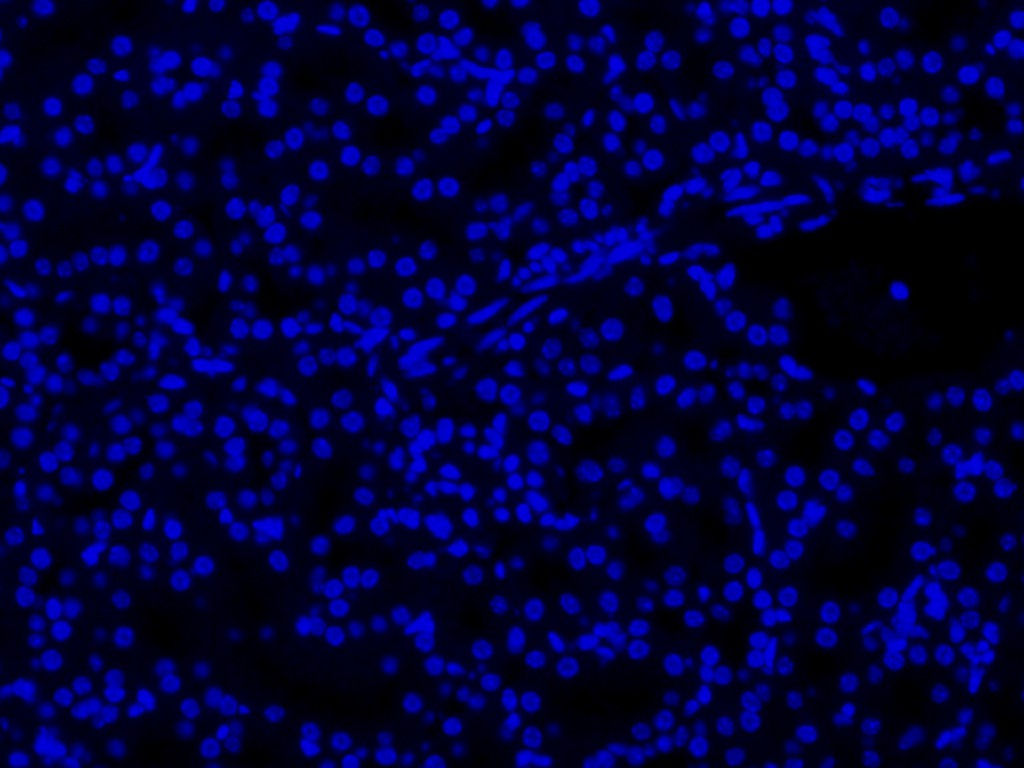

Supplement: Supplementary file 7 — Source data Fig. 6 [file 44321_2025_315_MOESM7_ESM.zip › Figure 6/F6A/1-IgA/4-2 (3).jpg]

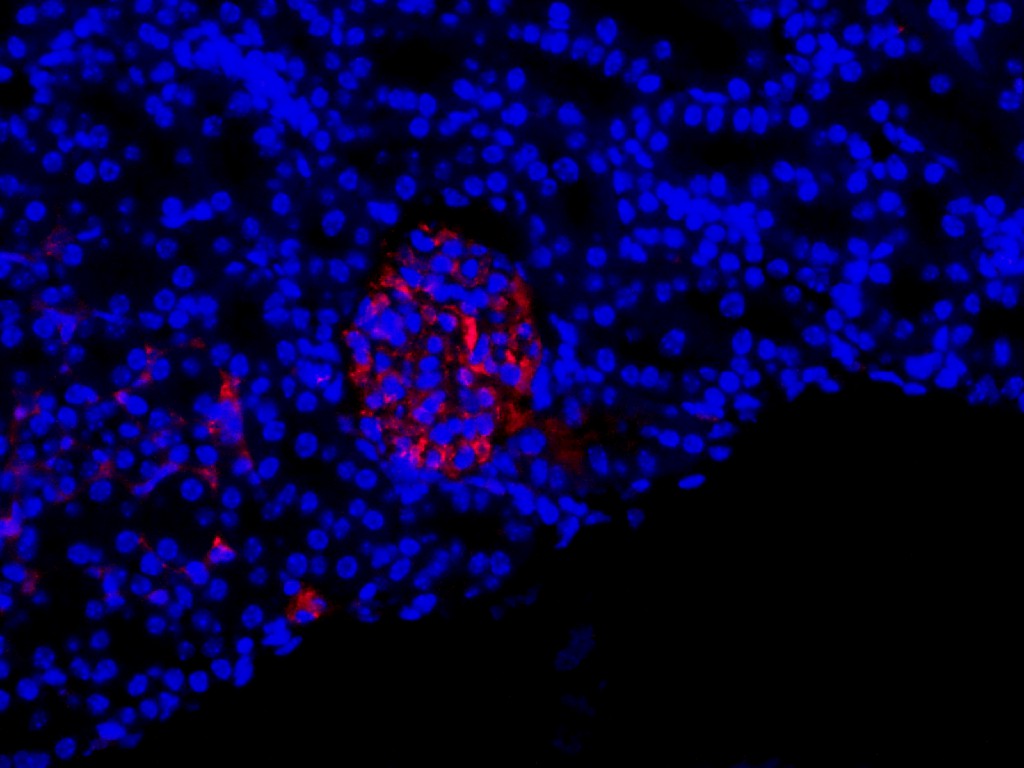

Supplement: Supplementary file 7 — Source data Fig. 6 [file 44321_2025_315_MOESM7_ESM.zip › Figure 6/F6A/1-IgA/4-3 (1).jpg]

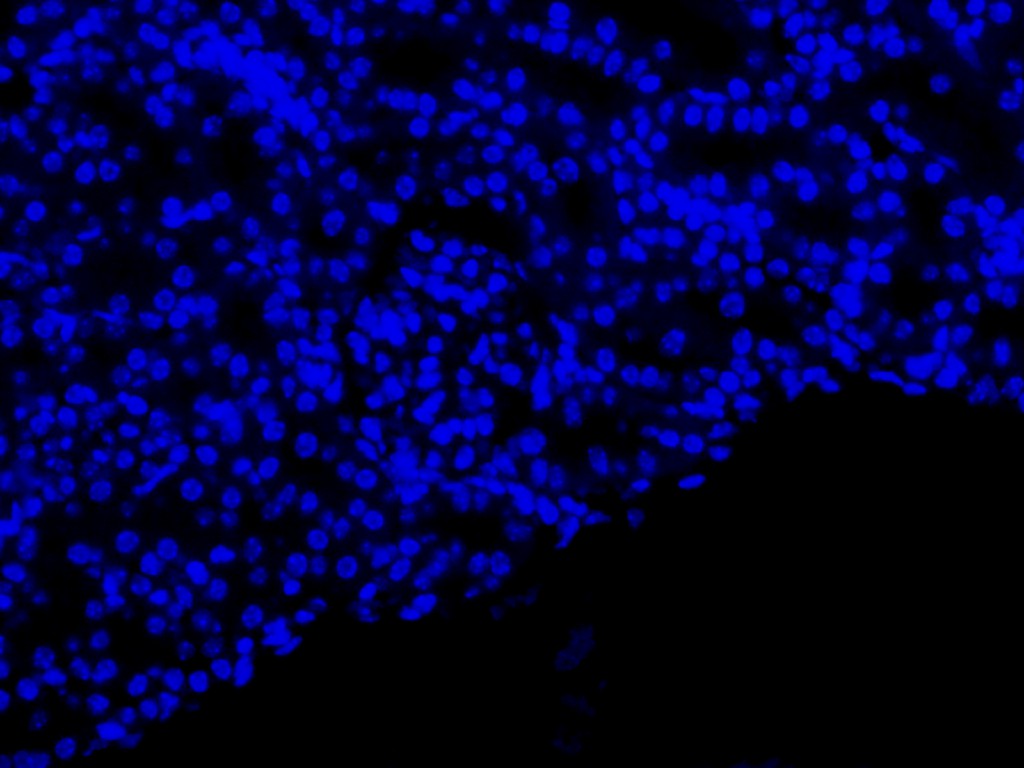

Supplement: Supplementary file 7 — Source data Fig. 6 [file 44321_2025_315_MOESM7_ESM.zip › Figure 6/F6A/1-IgA/4-3 (2).jpg]

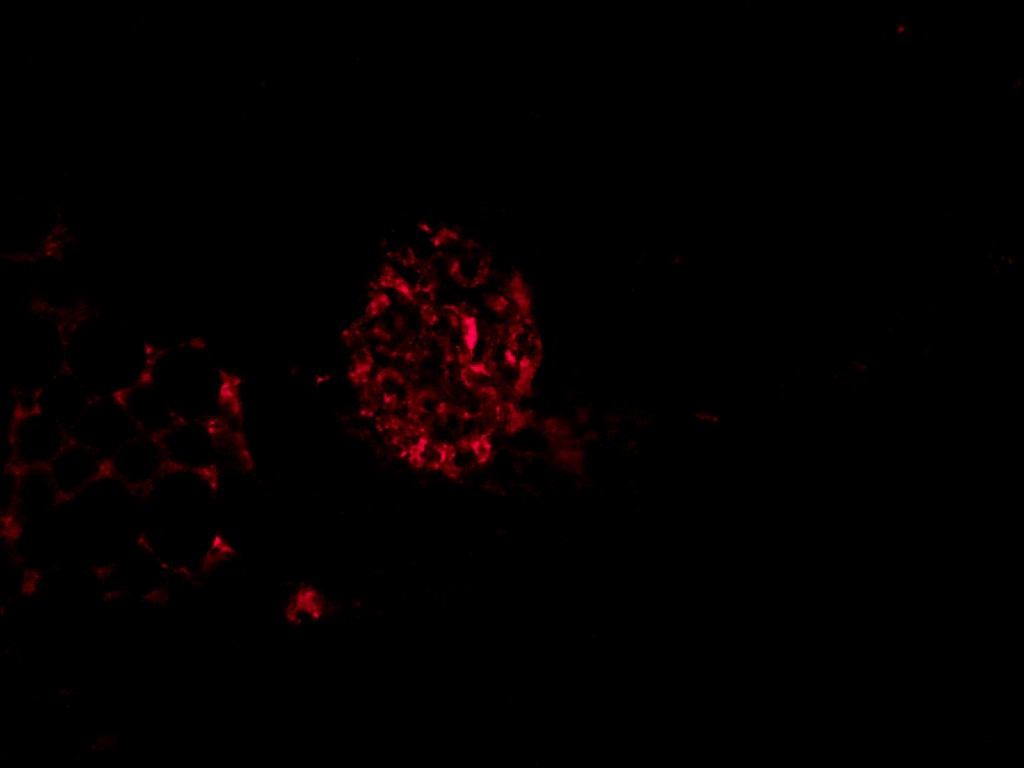

Supplement: Supplementary file 7 — Source data Fig. 6 [file 44321_2025_315_MOESM7_ESM.zip › Figure 6/F6A/1-IgA/4-3 (3).jpg]

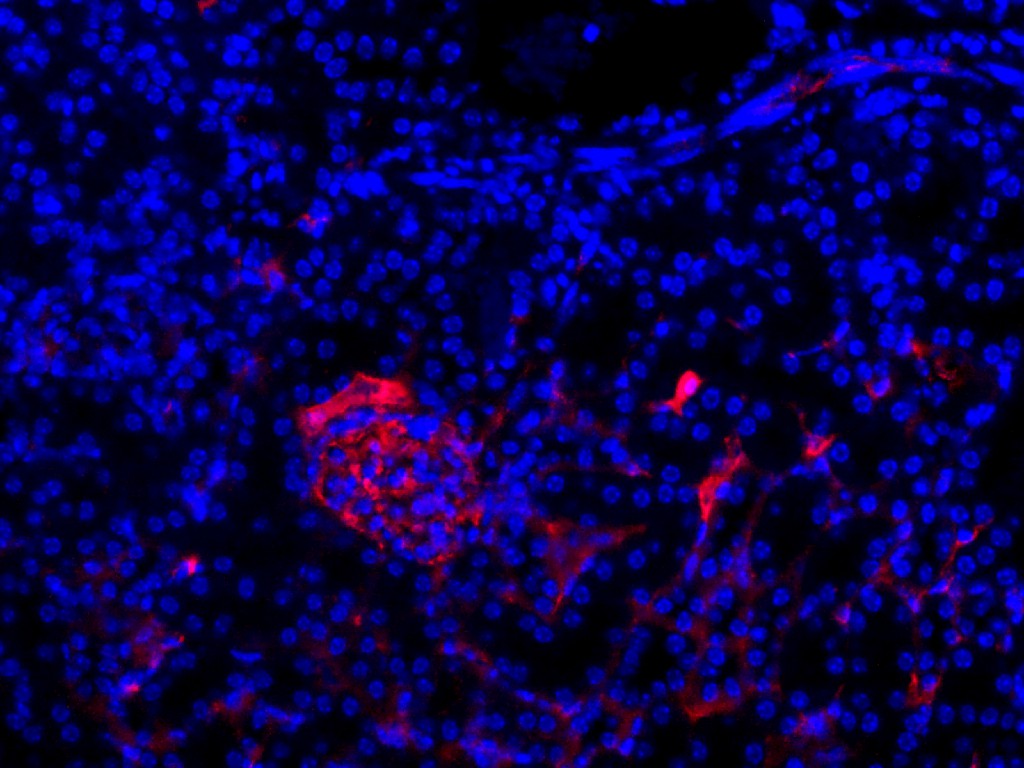

Supplement: Supplementary file 7 — Source data Fig. 6 [file 44321_2025_315_MOESM7_ESM.zip › Figure 6/F6A/1-IgA/4-4 (1).jpg]

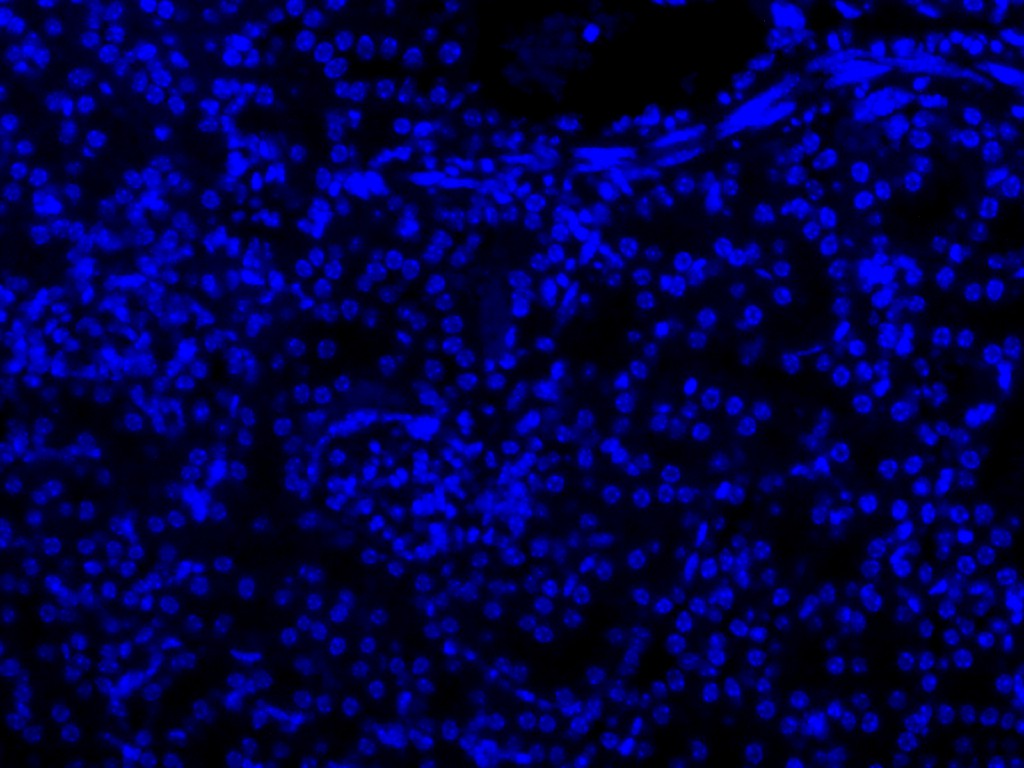

Supplement: Supplementary file 7 — Source data Fig. 6 [file 44321_2025_315_MOESM7_ESM.zip › Figure 6/F6A/1-IgA/4-4 (2).jpg]

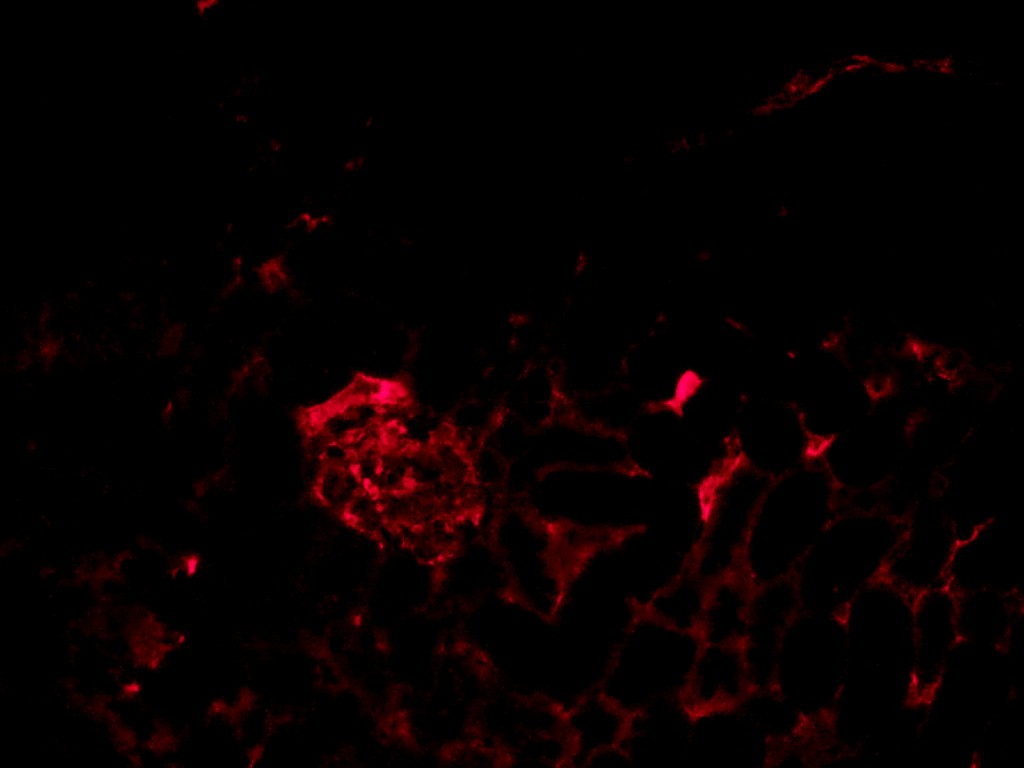

Supplement: Supplementary file 7 — Source data Fig. 6 [file 44321_2025_315_MOESM7_ESM.zip › Figure 6/F6A/1-IgA/4-4 (3).jpg]

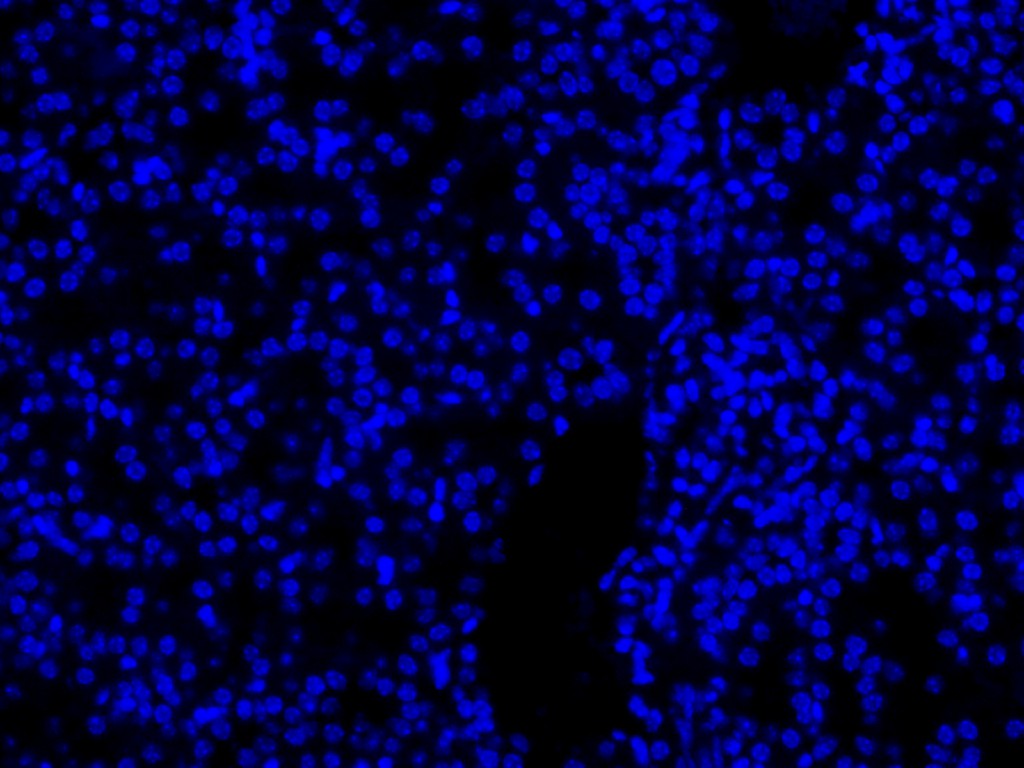

Supplement: Supplementary file 7 — Source data Fig. 6 [file 44321_2025_315_MOESM7_ESM.zip › Figure 6/F6A/1-IgA/4-5 (1).jpg]

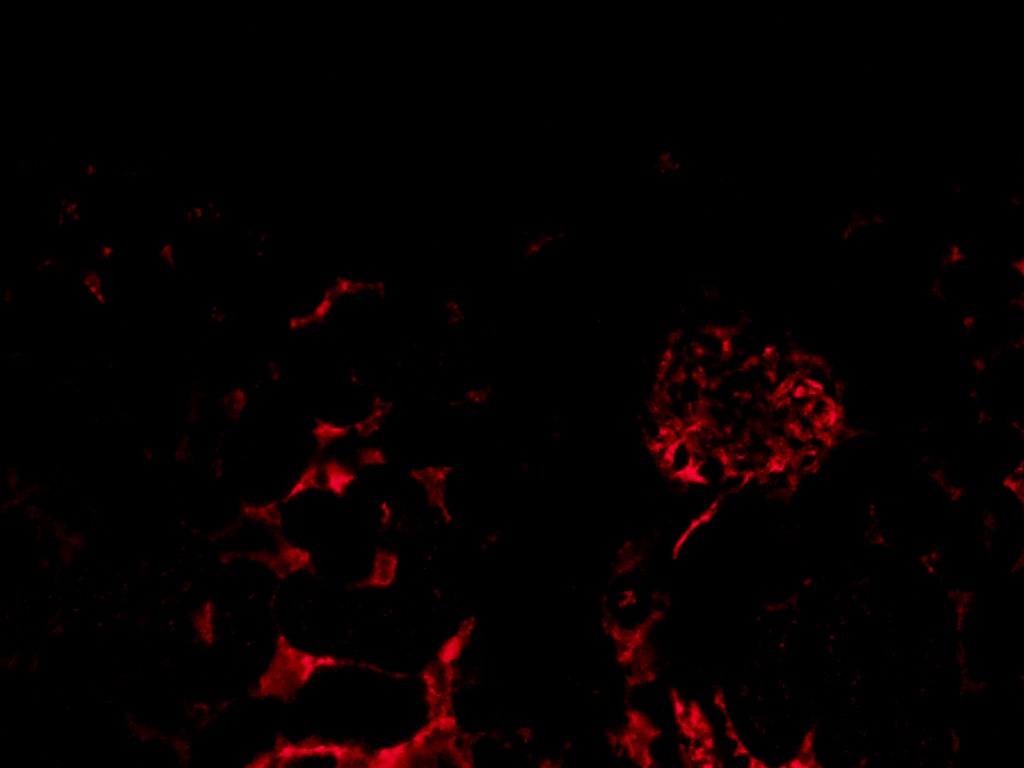

Supplement: Supplementary file 7 — Source data Fig. 6 [file 44321_2025_315_MOESM7_ESM.zip › Figure 6/F6A/1-IgA/4-5 (2).jpg]

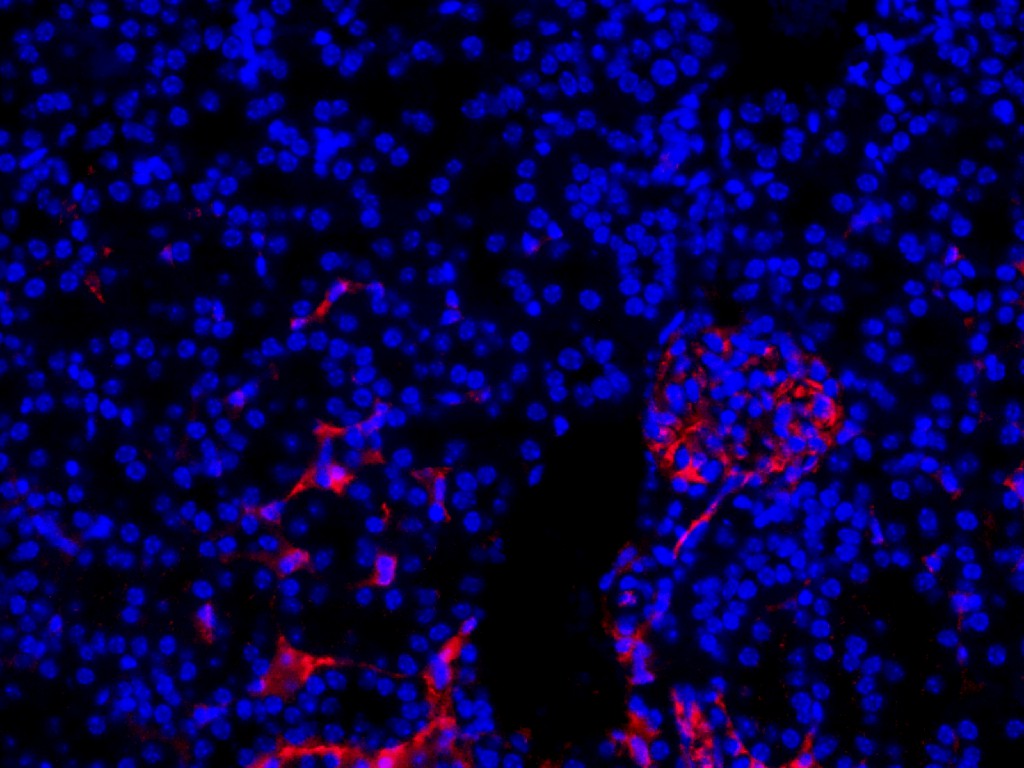

Supplement: Supplementary file 7 — Source data Fig. 6 [file 44321_2025_315_MOESM7_ESM.zip › Figure 6/F6A/1-IgA/4-5 (3).jpg]

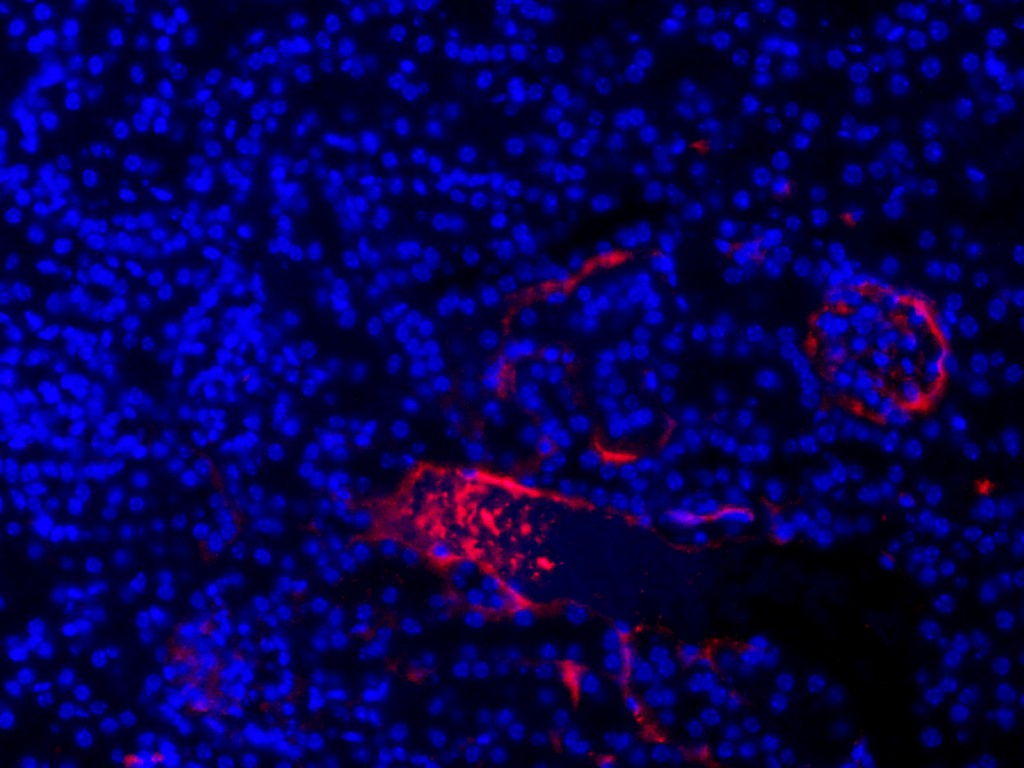

Supplement: Supplementary file 7 — Source data Fig. 6 [file 44321_2025_315_MOESM7_ESM.zip › Figure 6/F6A/1-IgA/4-6 (1).jpg]

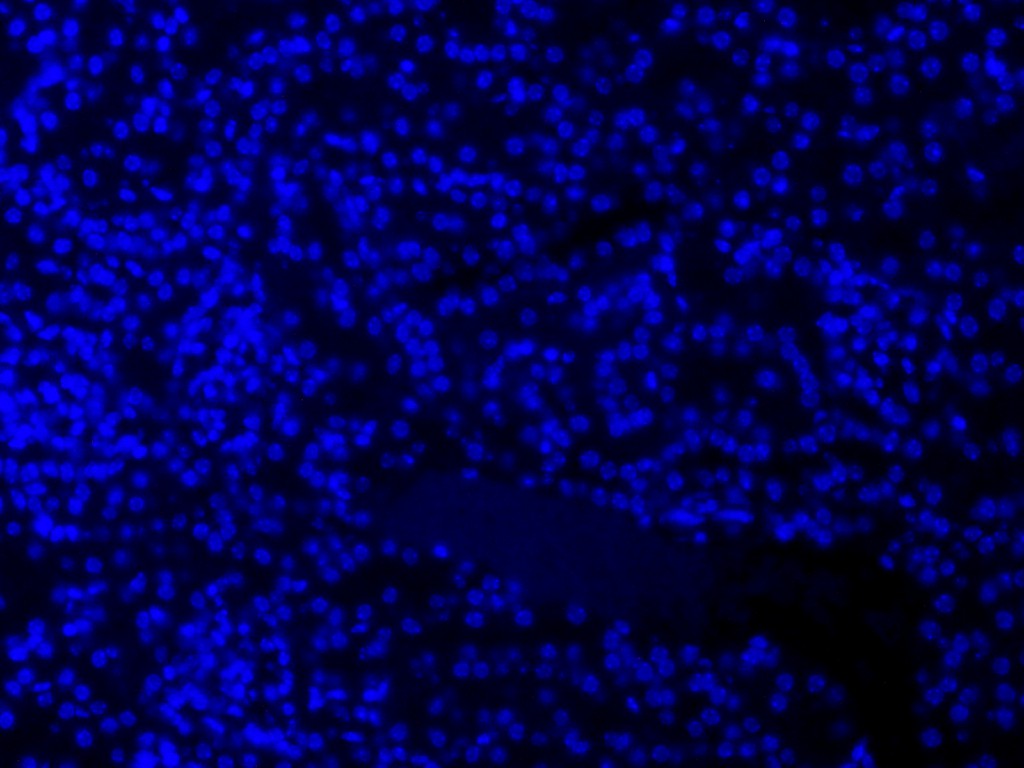

Supplement: Supplementary file 7 — Source data Fig. 6 [file 44321_2025_315_MOESM7_ESM.zip › Figure 6/F6A/1-IgA/4-6 (2).jpg]

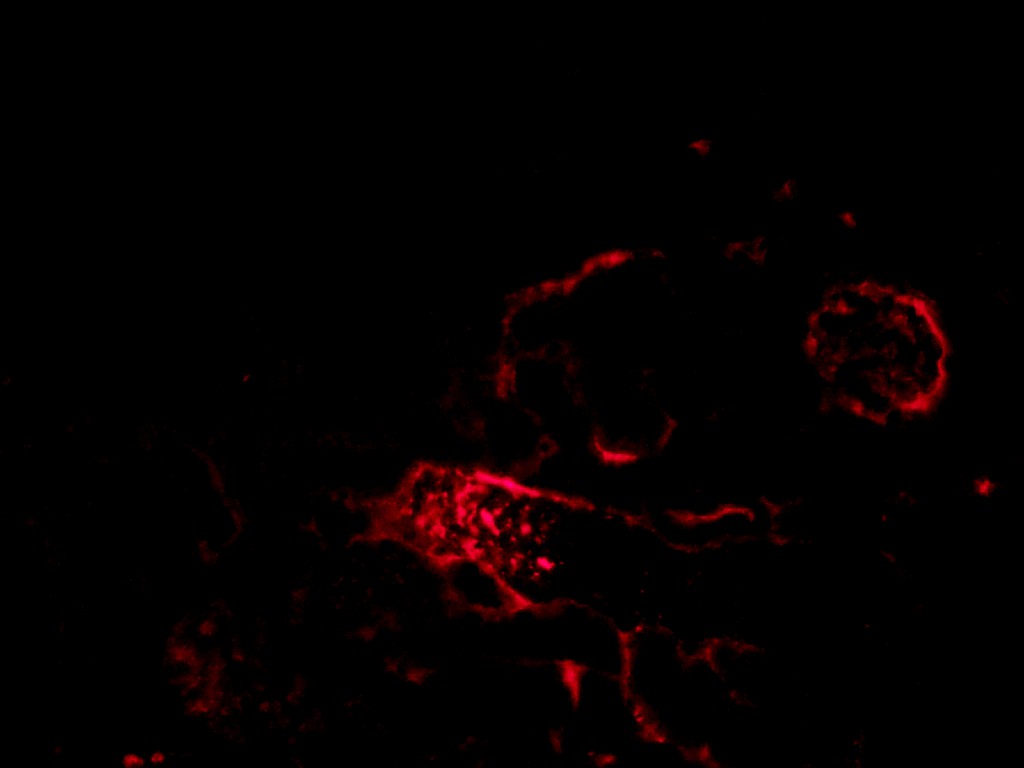

Supplement: Supplementary file 7 — Source data Fig. 6 [file 44321_2025_315_MOESM7_ESM.zip › Figure 6/F6A/1-IgA/4-6 (3).jpg]

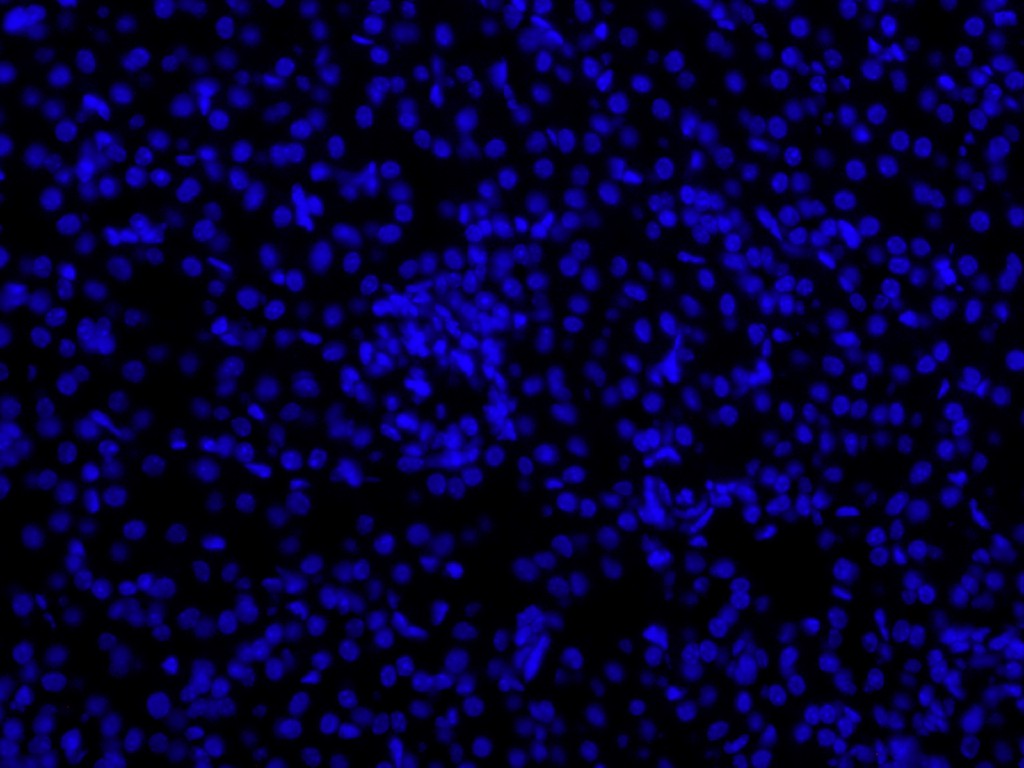

Supplement: Supplementary file 7 — Source data Fig. 6 [file 44321_2025_315_MOESM7_ESM.zip › Figure 6/F6A/2-C3/1-1 (1).jpg]

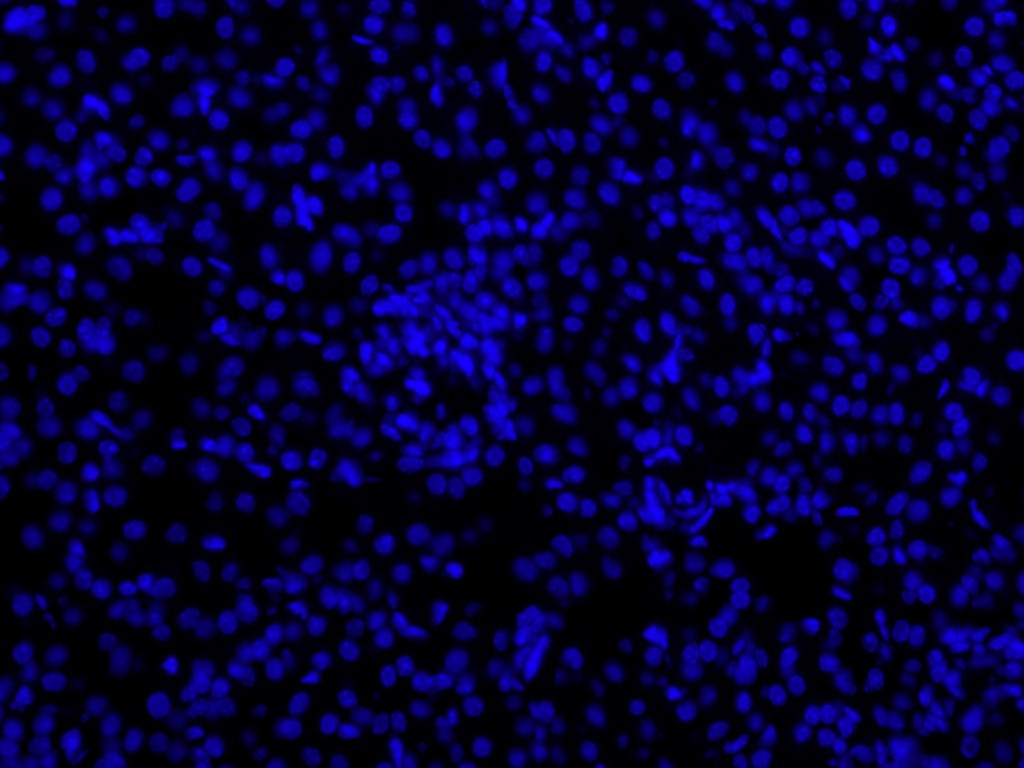

Supplement: Supplementary file 7 — Source data Fig. 6 [file 44321_2025_315_MOESM7_ESM.zip › Figure 6/F6A/2-C3/1-1 (2).jpg]

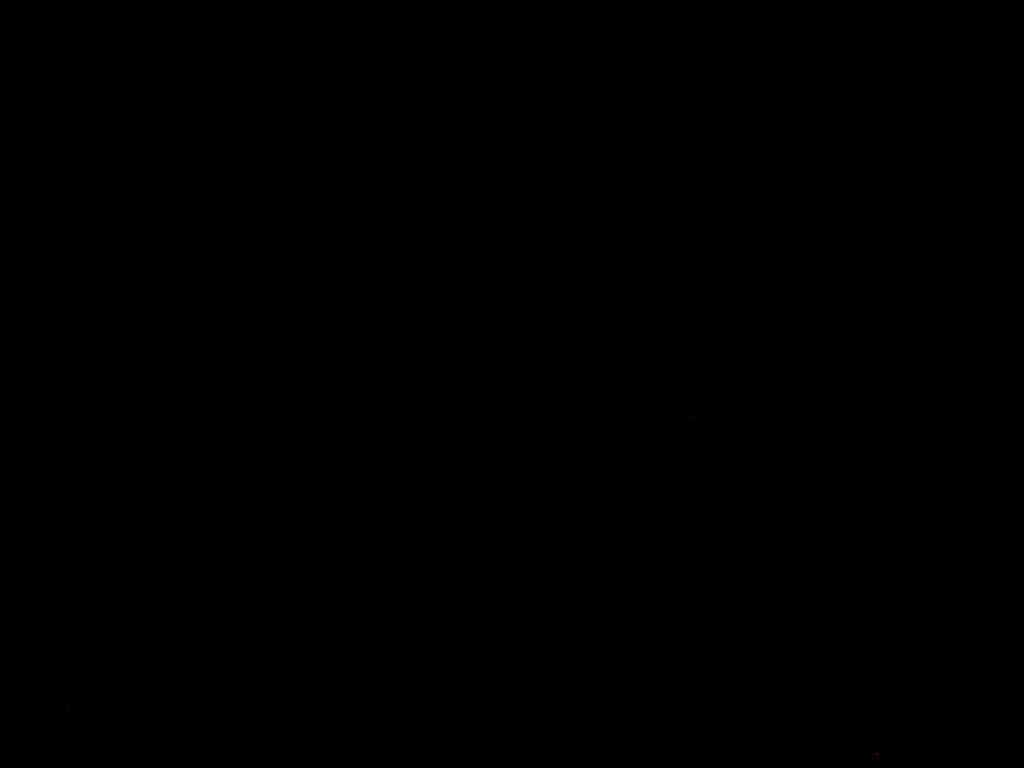

Supplement: Supplementary file 7 — Source data Fig. 6 [file 44321_2025_315_MOESM7_ESM.zip › Figure 6/F6A/2-C3/1-1 (3).jpg]

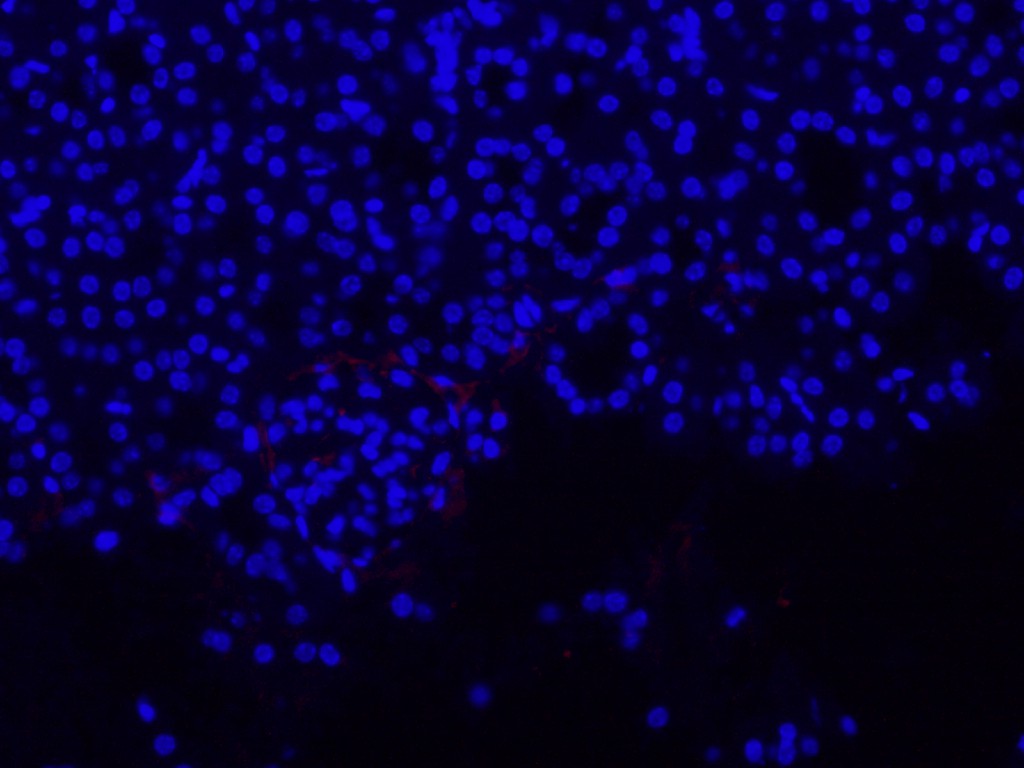

Supplement: Supplementary file 7 — Source data Fig. 6 [file 44321_2025_315_MOESM7_ESM.zip › Figure 6/F6A/2-C3/1-2 (1).jpg]

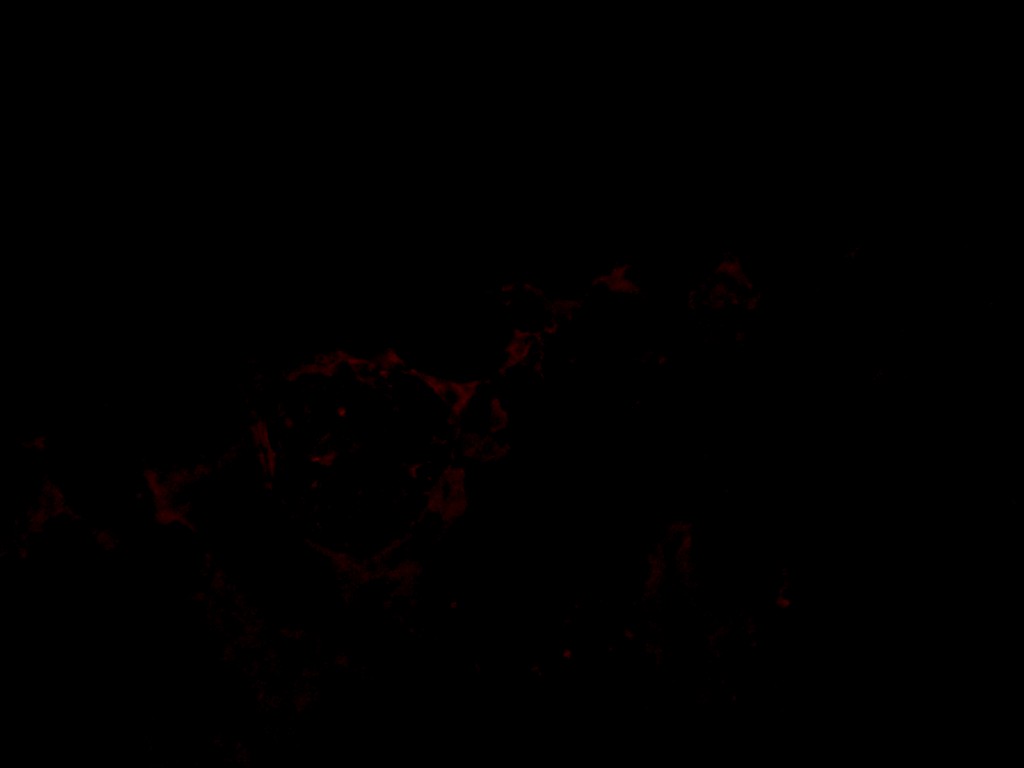

Supplement: Supplementary file 7 — Source data Fig. 6 [file 44321_2025_315_MOESM7_ESM.zip › Figure 6/F6A/2-C3/1-2 (2).jpg]

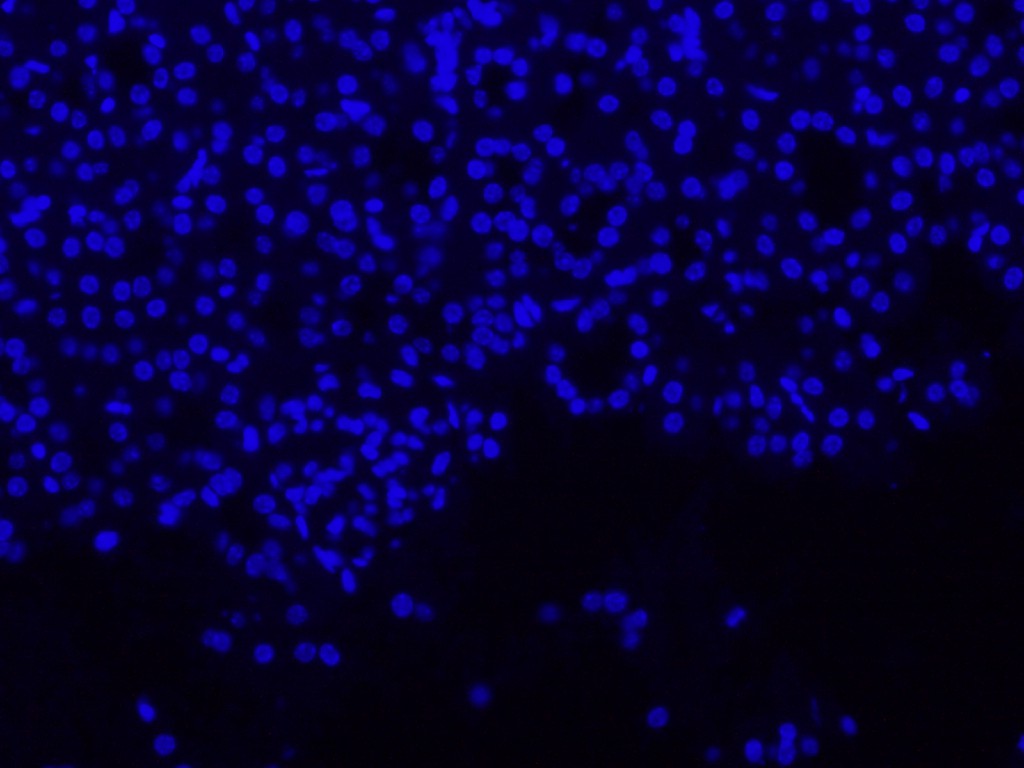

Supplement: Supplementary file 7 — Source data Fig. 6 [file 44321_2025_315_MOESM7_ESM.zip › Figure 6/F6A/2-C3/1-2 (3).jpg]

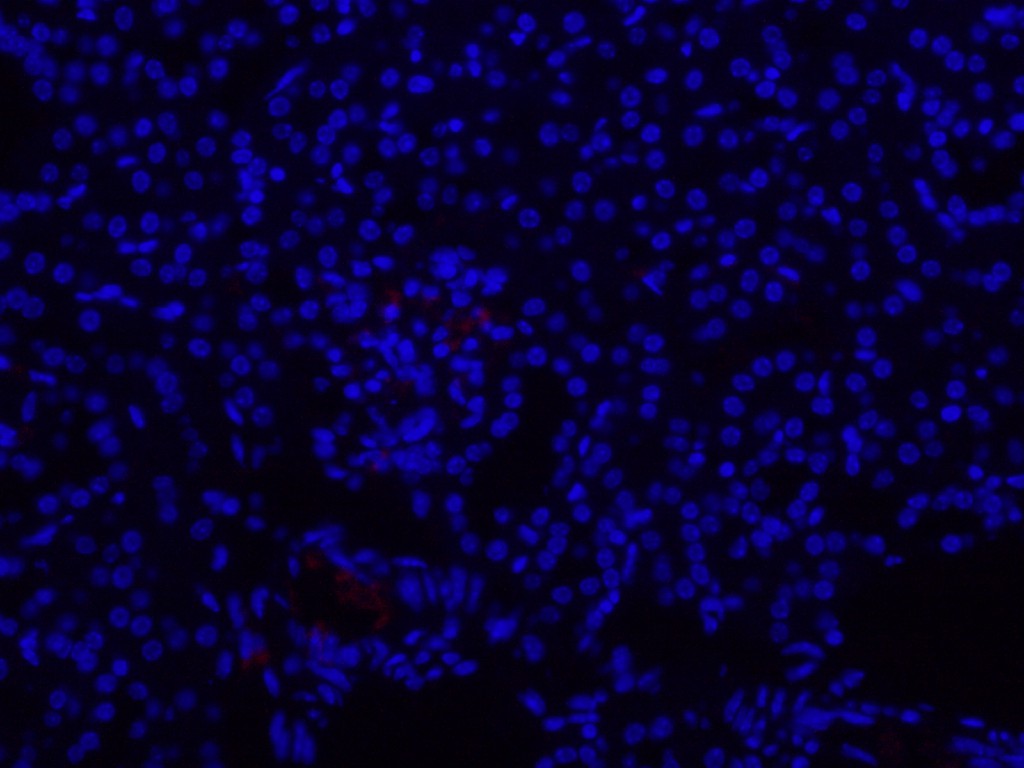

Supplement: Supplementary file 7 — Source data Fig. 6 [file 44321_2025_315_MOESM7_ESM.zip › Figure 6/F6A/2-C3/1-3 (1).jpg]

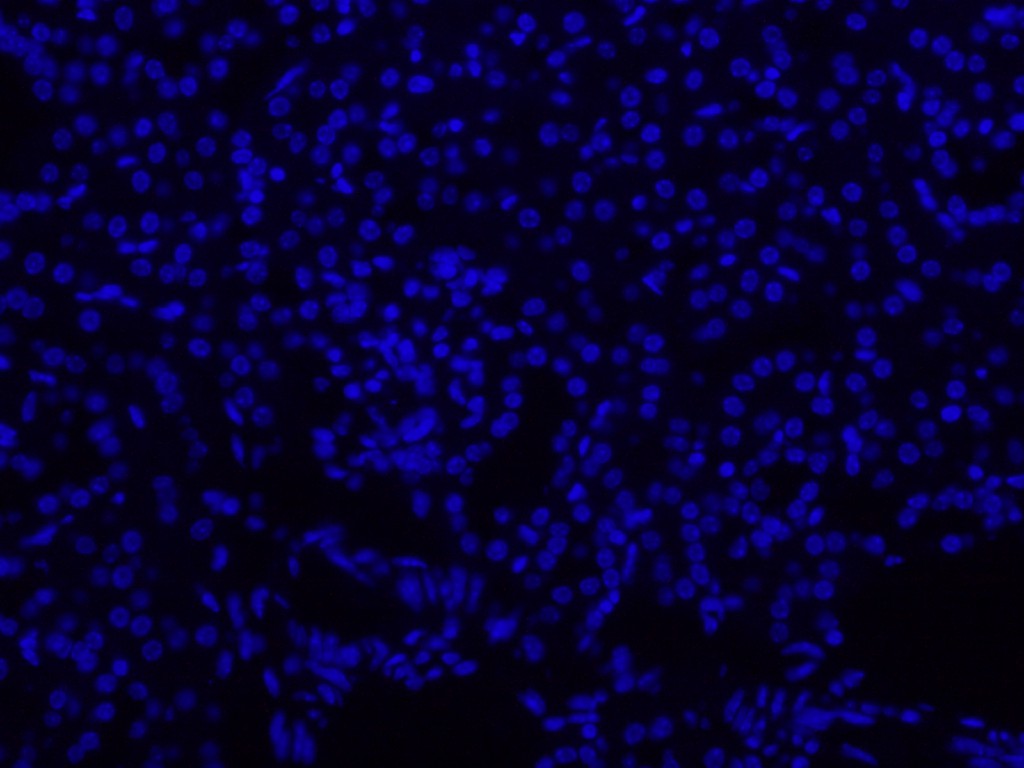

Supplement: Supplementary file 7 — Source data Fig. 6 [file 44321_2025_315_MOESM7_ESM.zip › Figure 6/F6A/2-C3/1-3 (2).jpg]

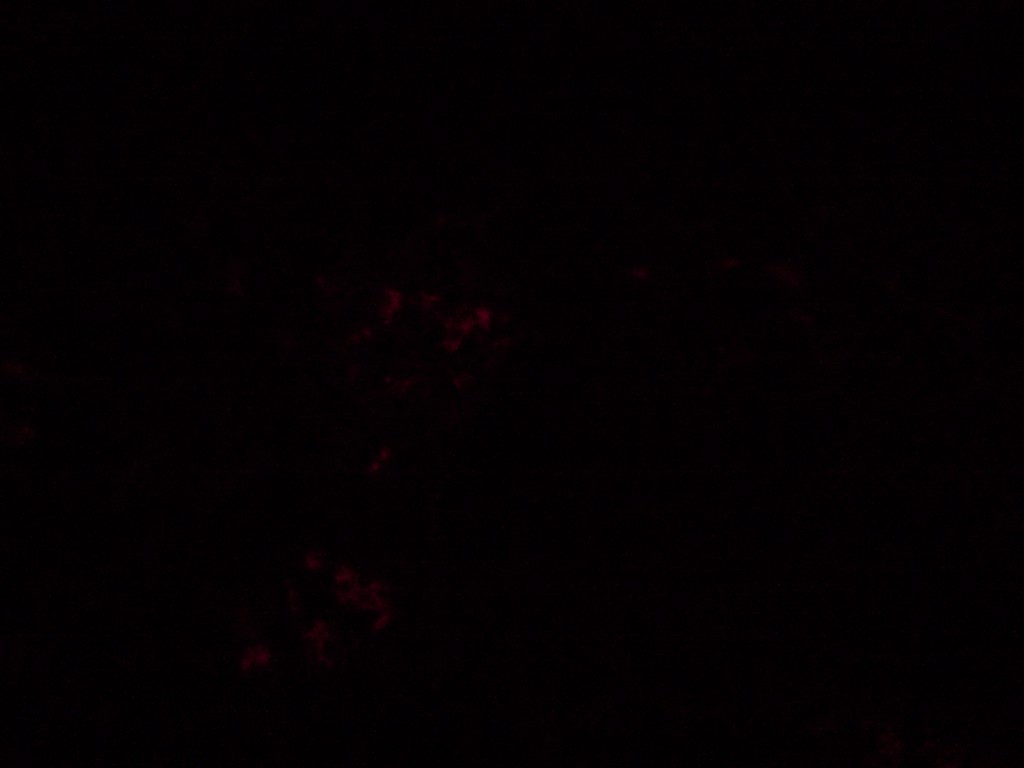

Supplement: Supplementary file 7 — Source data Fig. 6 [file 44321_2025_315_MOESM7_ESM.zip › Figure 6/F6A/2-C3/1-3 (3).jpg]

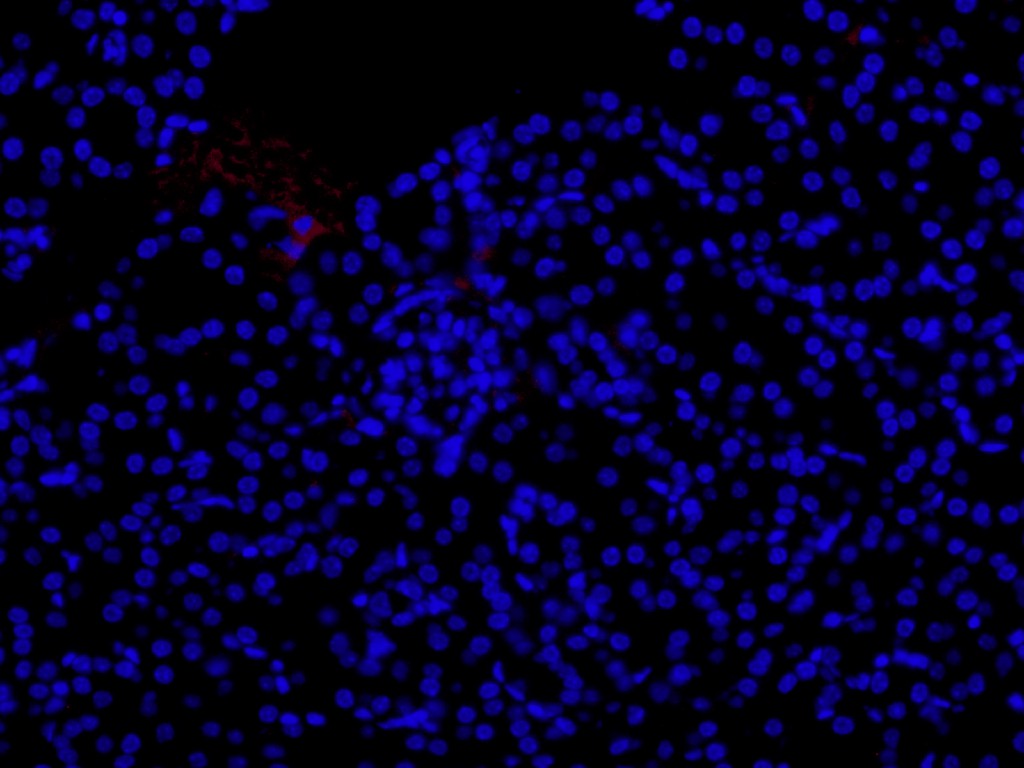

Supplement: Supplementary file 7 — Source data Fig. 6 [file 44321_2025_315_MOESM7_ESM.zip › Figure 6/F6A/2-C3/1-4 (1).jpg]

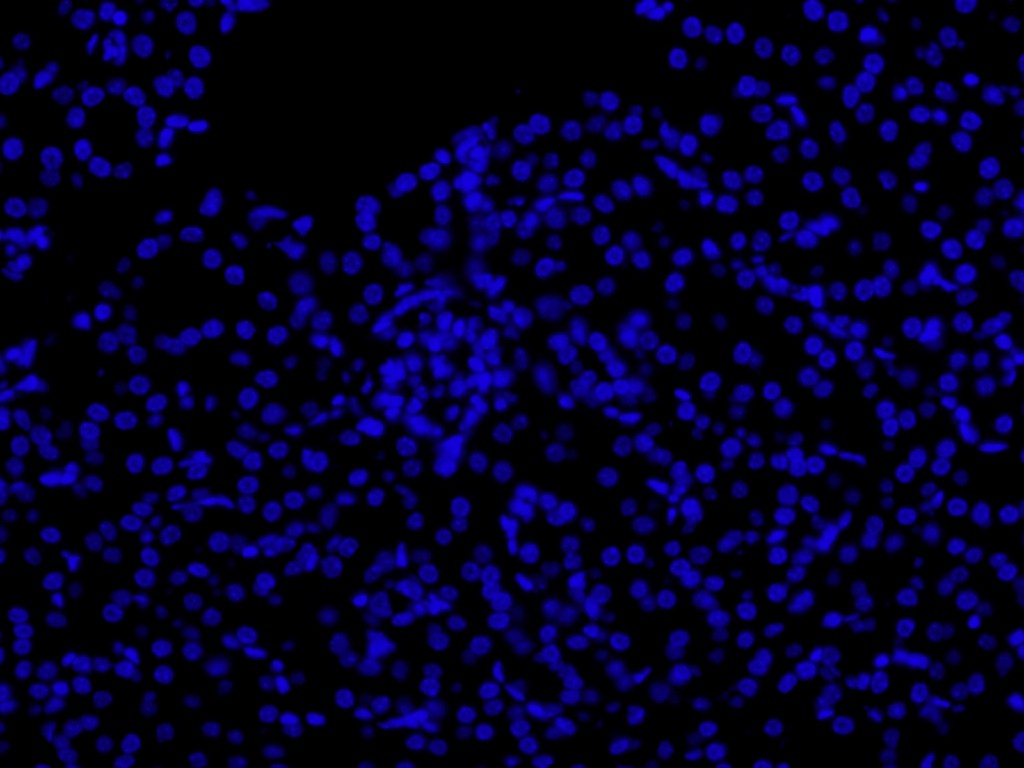

Supplement: Supplementary file 7 — Source data Fig. 6 [file 44321_2025_315_MOESM7_ESM.zip › Figure 6/F6A/2-C3/1-4 (2).jpg]

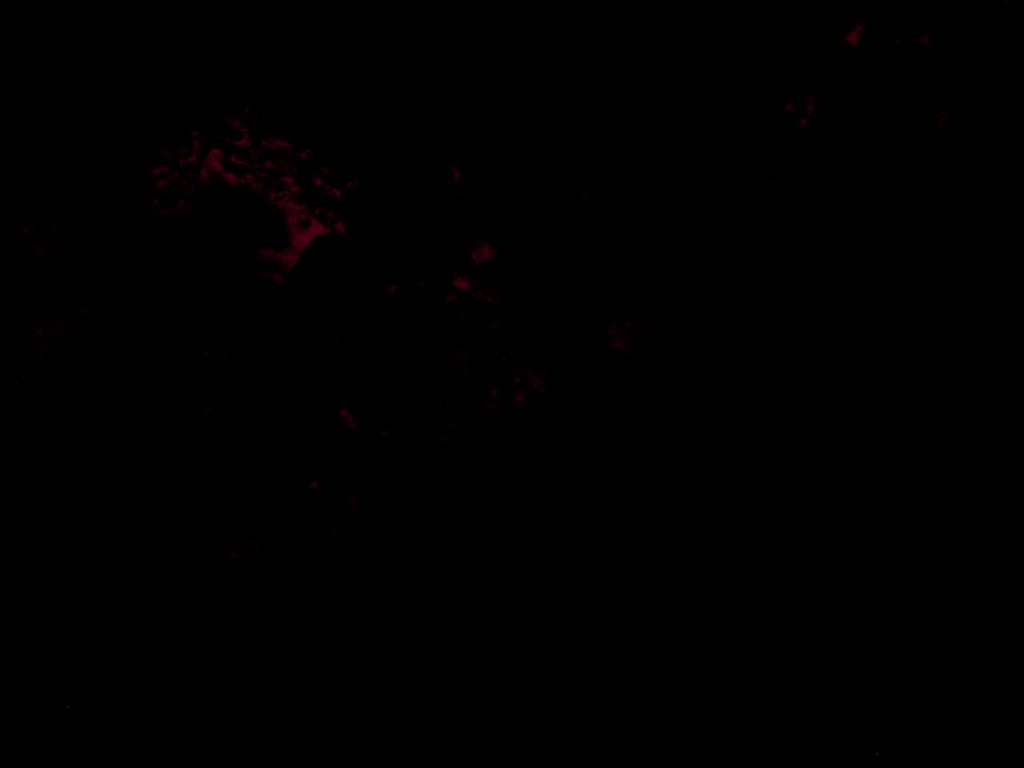

Supplement: Supplementary file 7 — Source data Fig. 6 [file 44321_2025_315_MOESM7_ESM.zip › Figure 6/F6A/2-C3/1-4 (3).jpg]

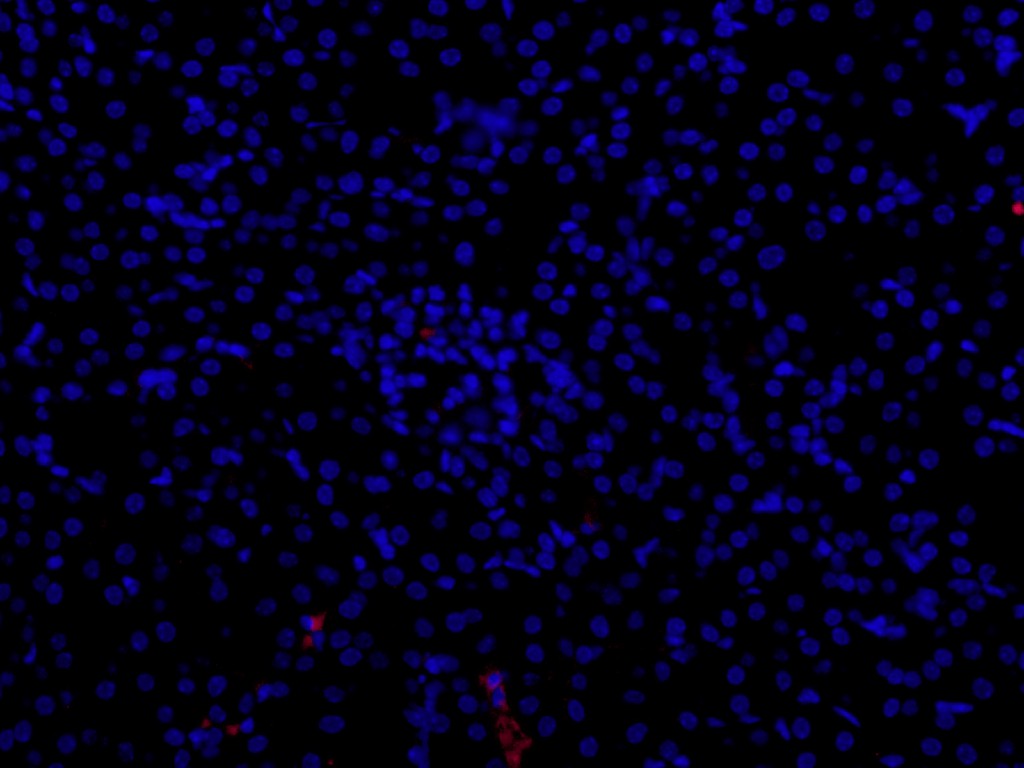

Supplement: Supplementary file 7 — Source data Fig. 6 [file 44321_2025_315_MOESM7_ESM.zip › Figure 6/F6A/2-C3/1-5 (1).jpg]

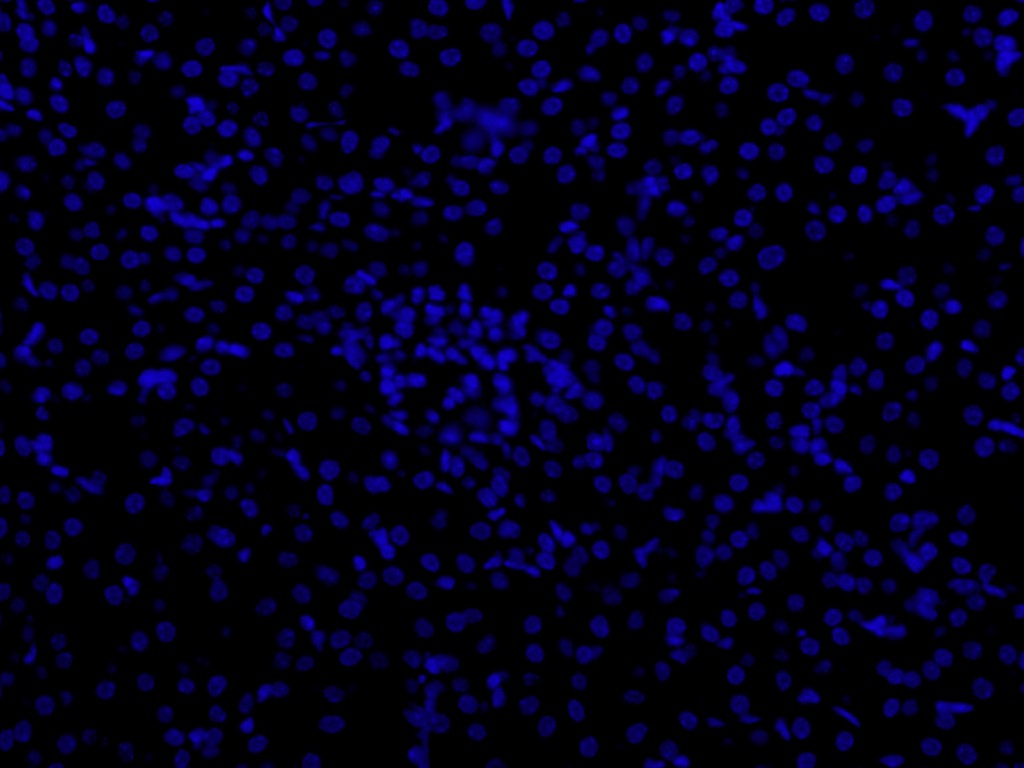

Supplement: Supplementary file 7 — Source data Fig. 6 [file 44321_2025_315_MOESM7_ESM.zip › Figure 6/F6A/2-C3/1-5 (2).jpg]

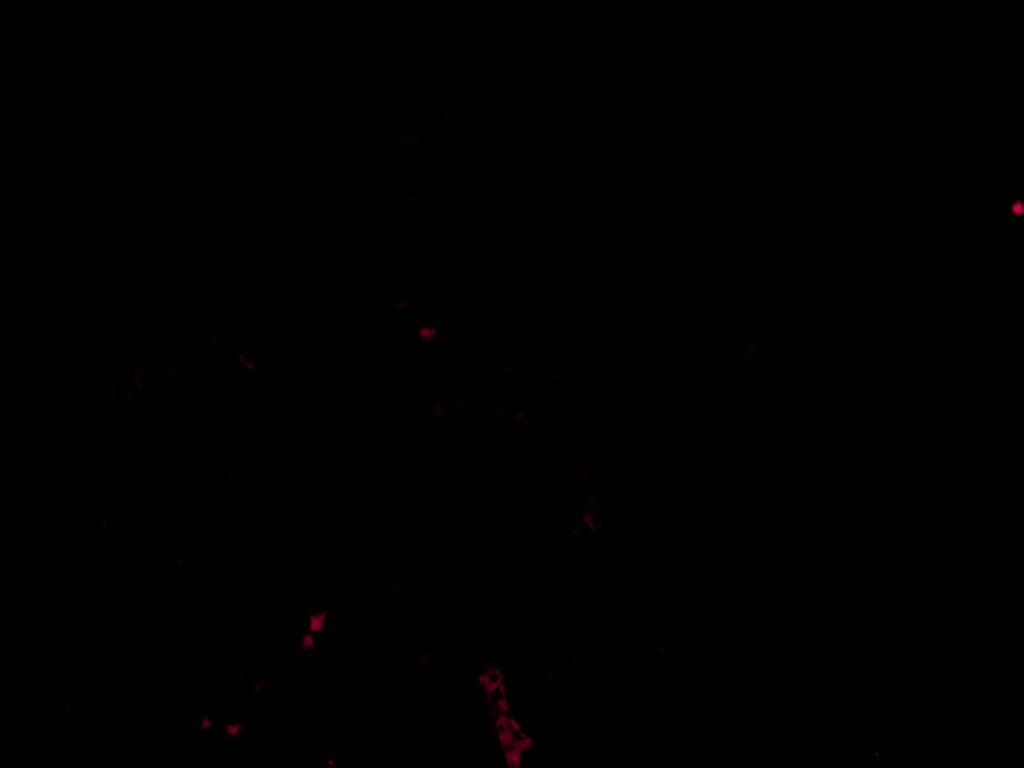

Supplement: Supplementary file 7 — Source data Fig. 6 [file 44321_2025_315_MOESM7_ESM.zip › Figure 6/F6A/2-C3/1-5 (3).jpg]

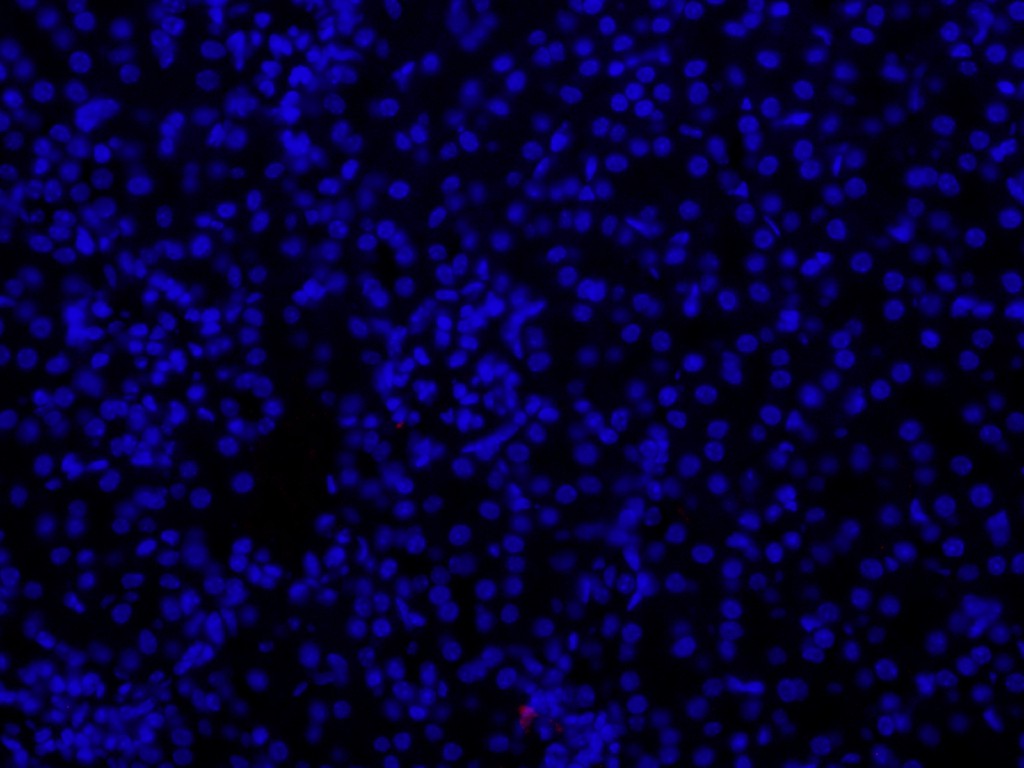

Supplement: Supplementary file 7 — Source data Fig. 6 [file 44321_2025_315_MOESM7_ESM.zip › Figure 6/F6A/2-C3/1-6 (1).jpg]

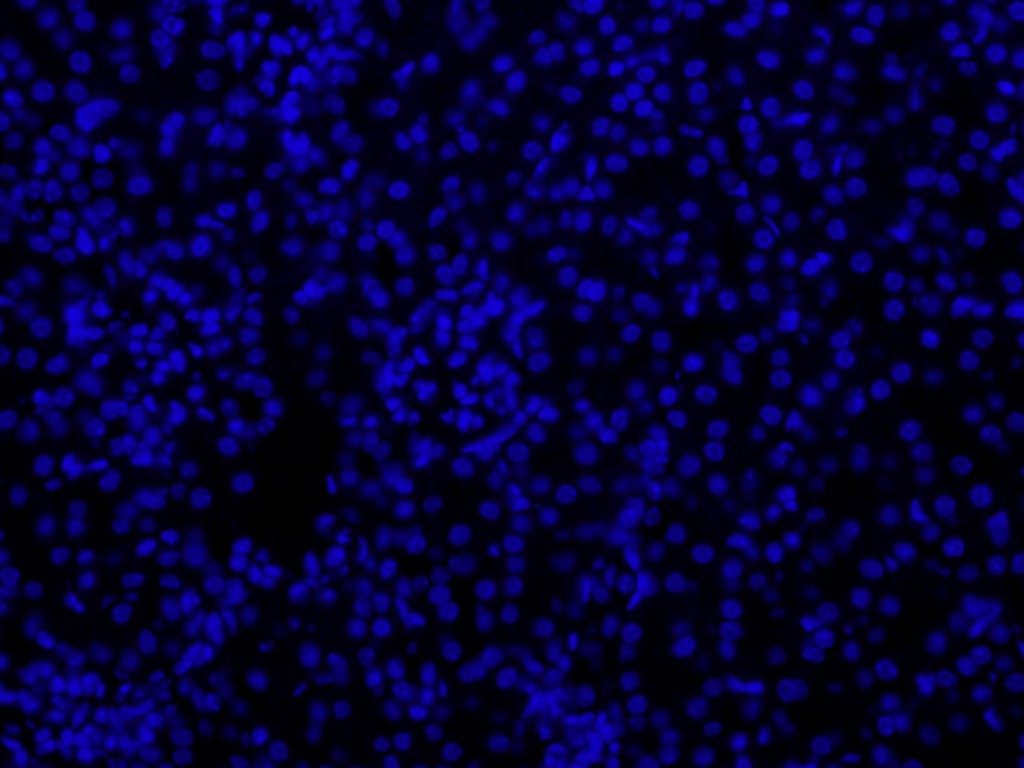

Supplement: Supplementary file 7 — Source data Fig. 6 [file 44321_2025_315_MOESM7_ESM.zip › Figure 6/F6A/2-C3/1-6 (2).jpg]

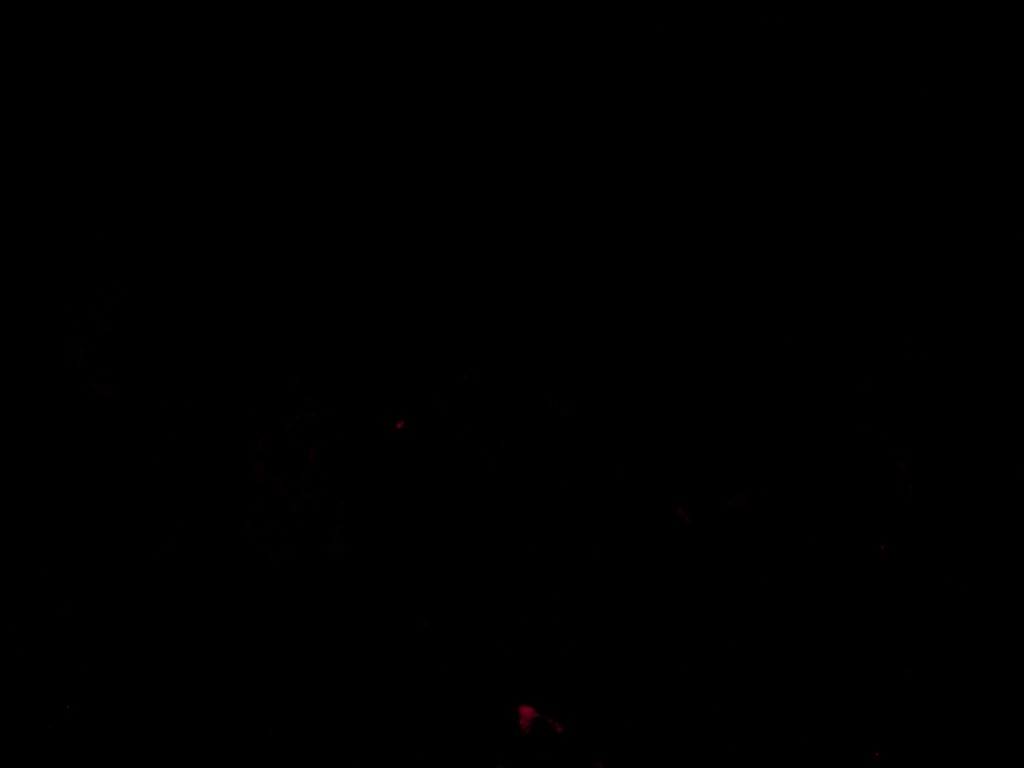

Supplement: Supplementary file 7 — Source data Fig. 6 [file 44321_2025_315_MOESM7_ESM.zip › Figure 6/F6A/2-C3/1-6 (3).jpg]

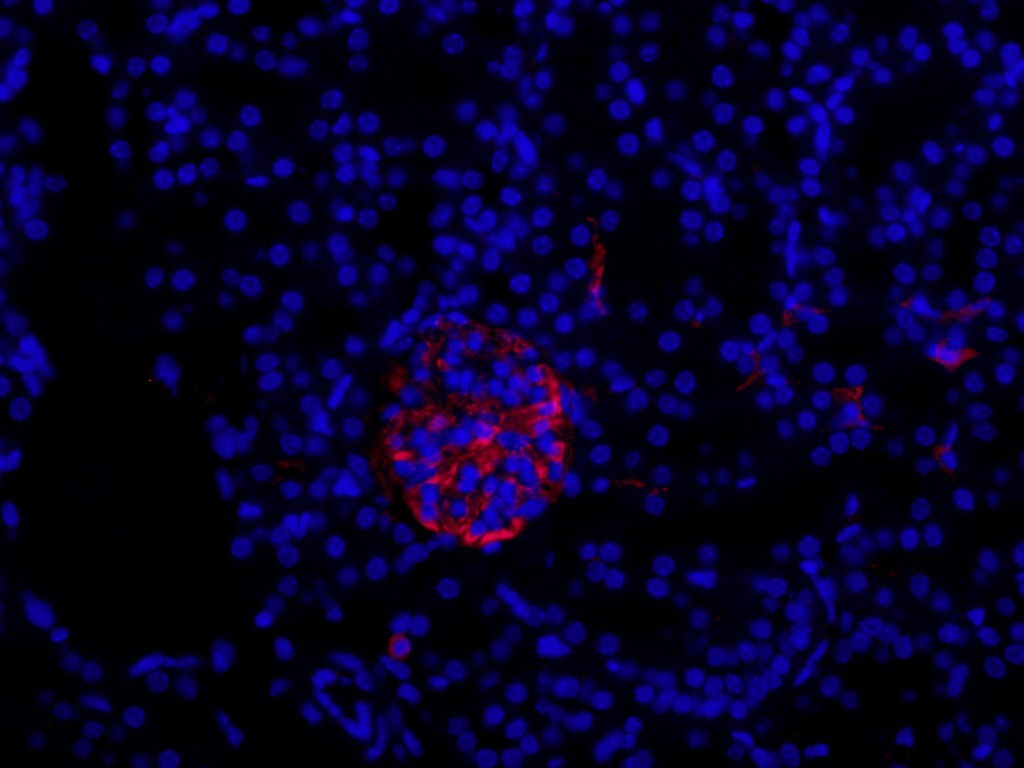

Supplement: Supplementary file 7 — Source data Fig. 6 [file 44321_2025_315_MOESM7_ESM.zip › Figure 6/F6A/2-C3/2-1 (1).jpg]

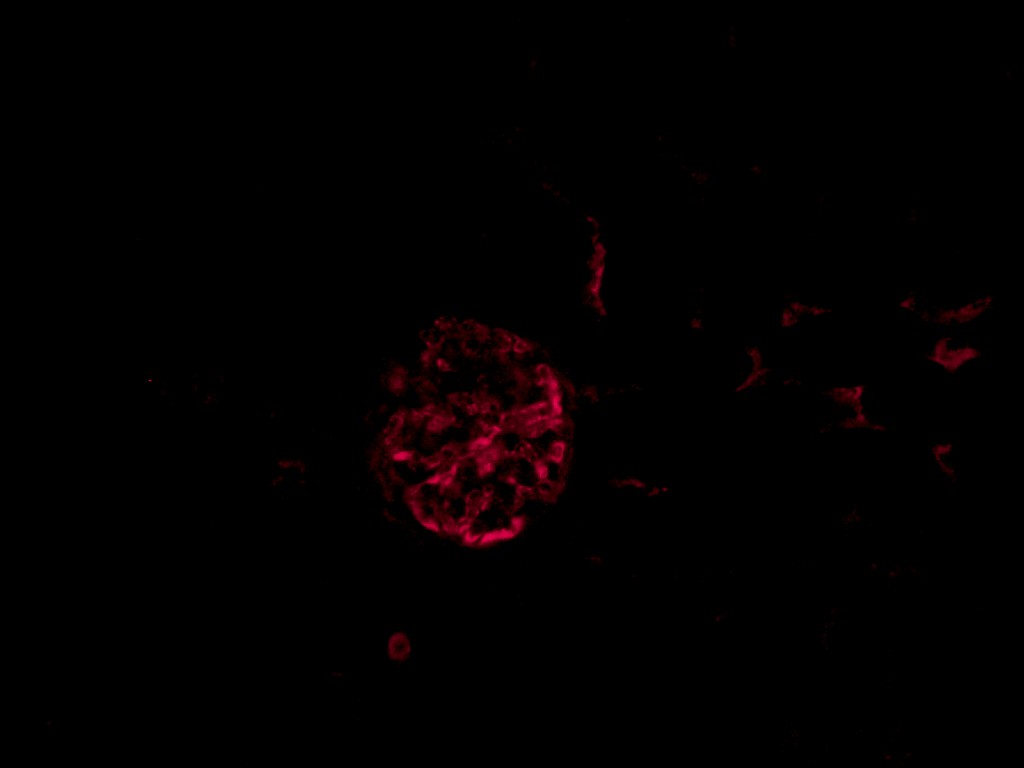

Supplement: Supplementary file 7 — Source data Fig. 6 [file 44321_2025_315_MOESM7_ESM.zip › Figure 6/F6A/2-C3/2-1 (2).jpg]

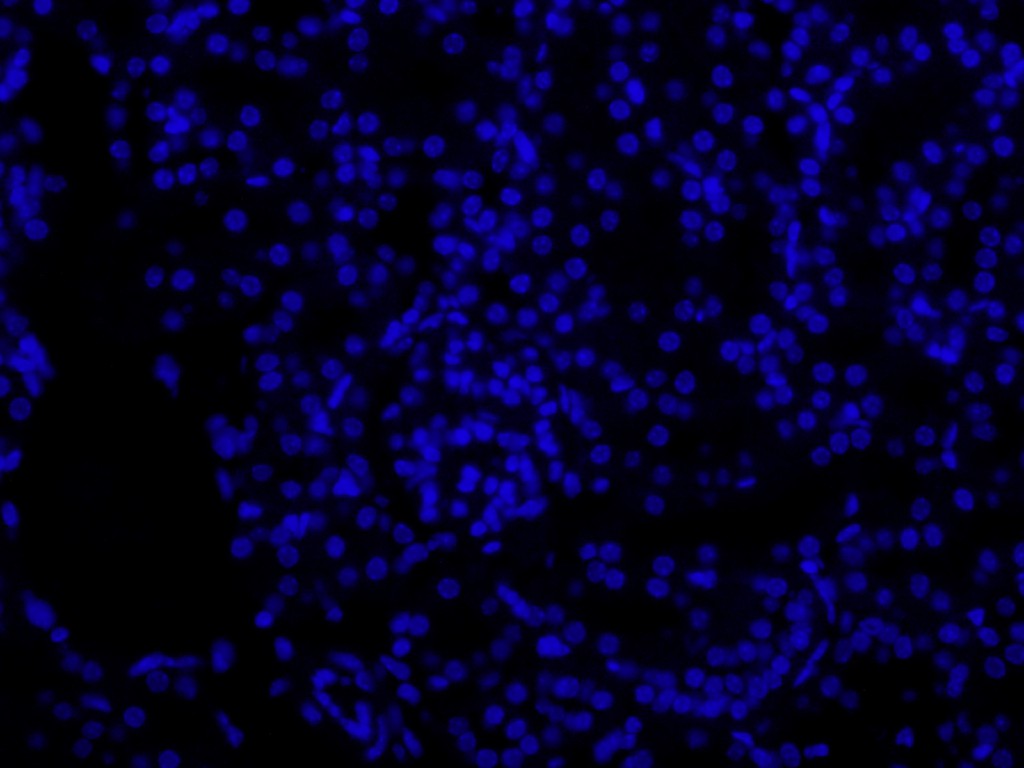

Supplement: Supplementary file 7 — Source data Fig. 6 [file 44321_2025_315_MOESM7_ESM.zip › Figure 6/F6A/2-C3/2-1 (3).jpg]

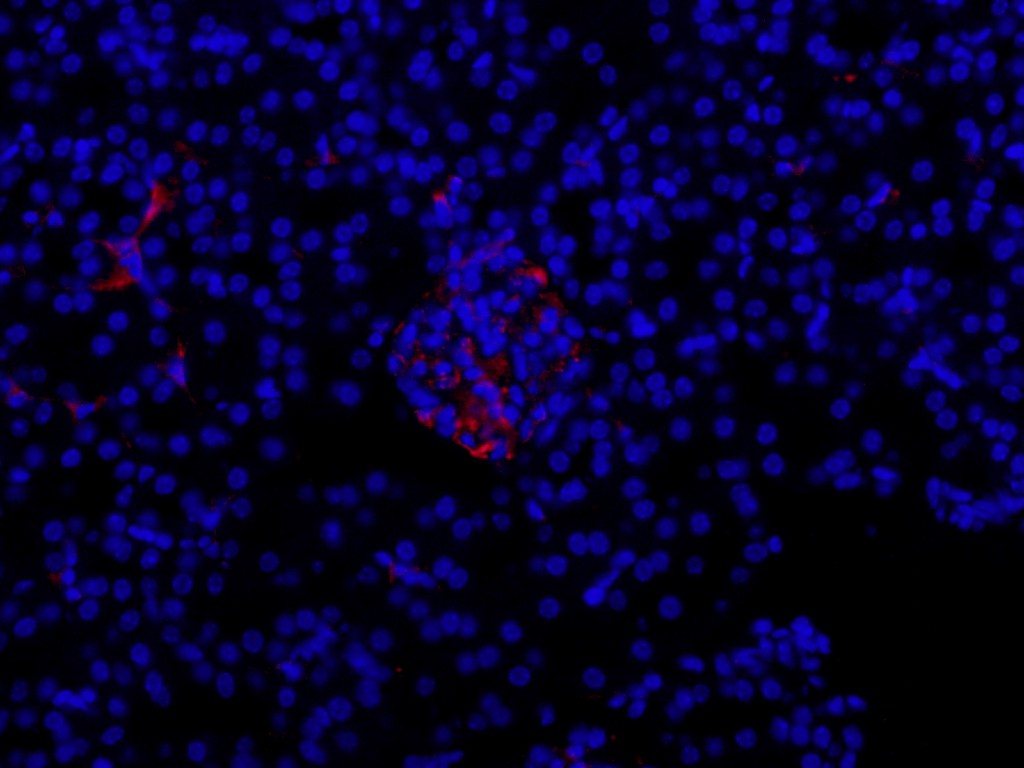

Supplement: Supplementary file 7 — Source data Fig. 6 [file 44321_2025_315_MOESM7_ESM.zip › Figure 6/F6A/2-C3/2-2 (1).jpg]

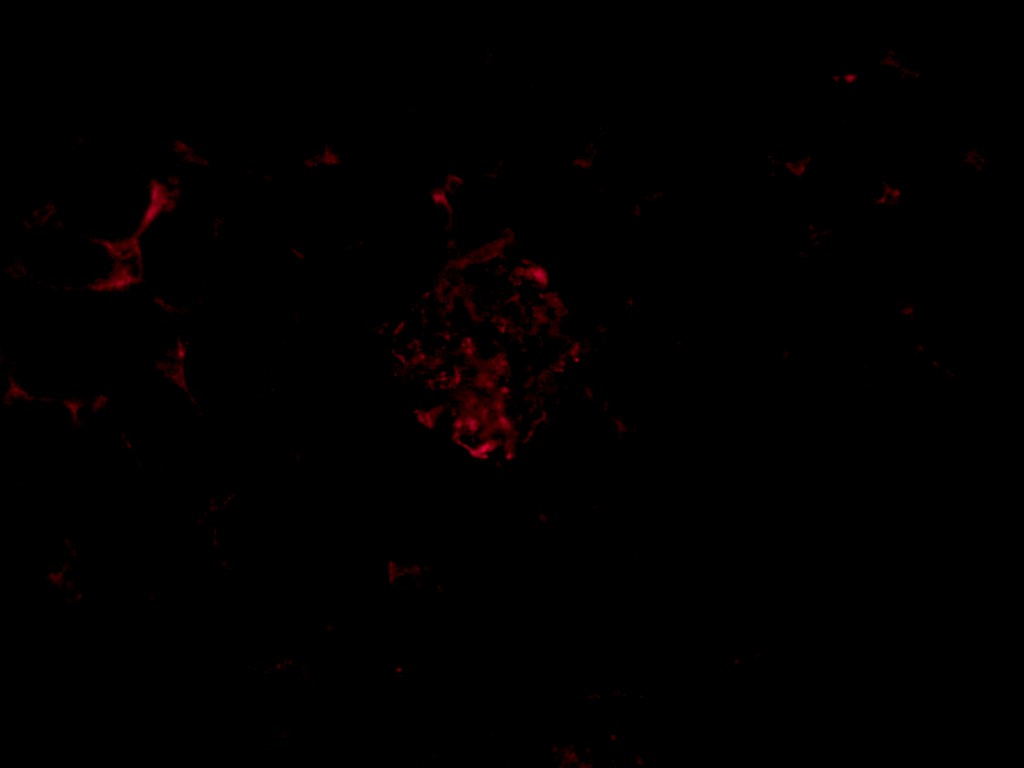

Supplement: Supplementary file 7 — Source data Fig. 6 [file 44321_2025_315_MOESM7_ESM.zip › Figure 6/F6A/2-C3/2-2 (2).jpg]

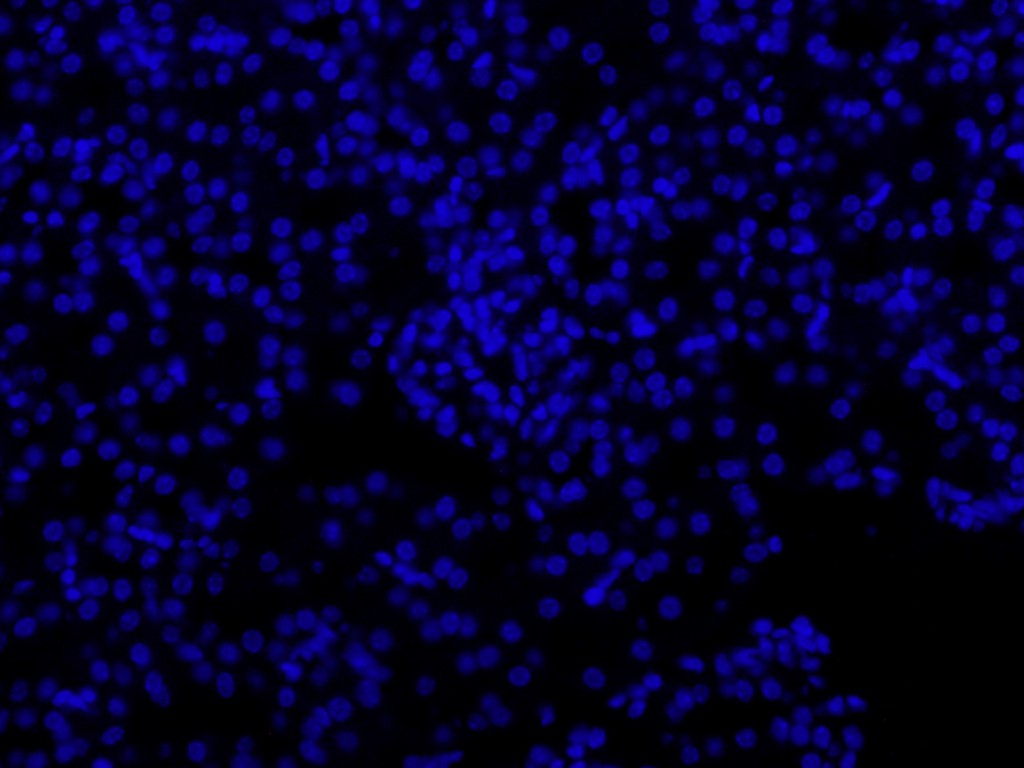

Supplement: Supplementary file 7 — Source data Fig. 6 [file 44321_2025_315_MOESM7_ESM.zip › Figure 6/F6A/2-C3/2-2 (3).jpg]

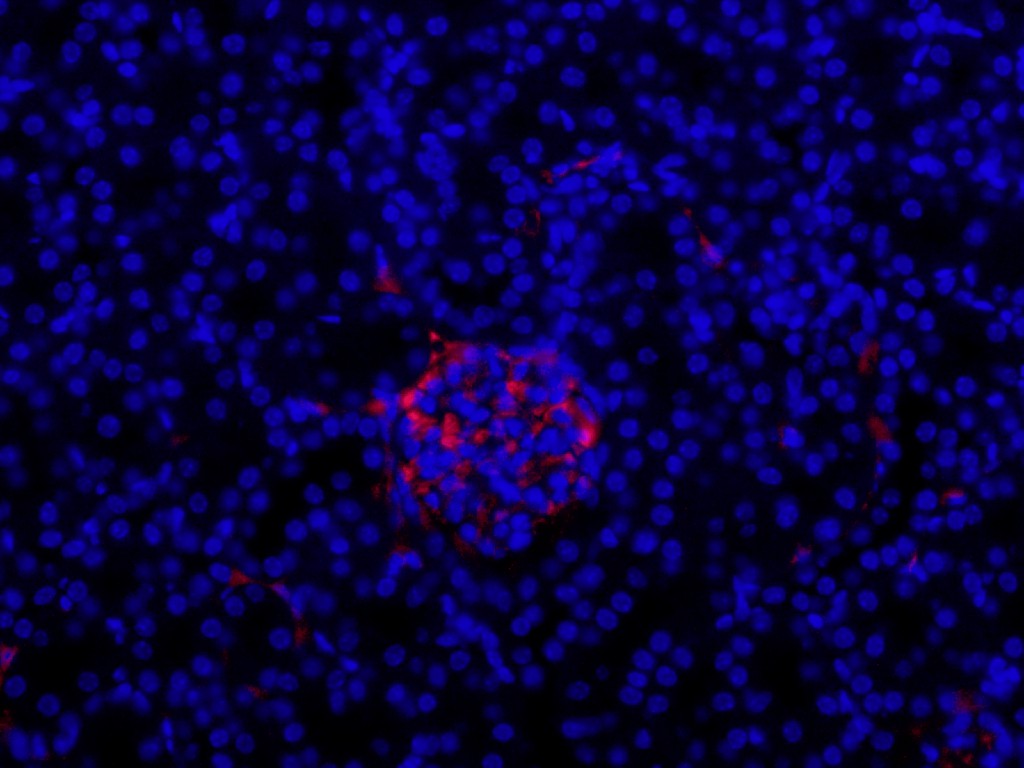

Supplement: Supplementary file 7 — Source data Fig. 6 [file 44321_2025_315_MOESM7_ESM.zip › Figure 6/F6A/2-C3/2-3 (1).jpg]

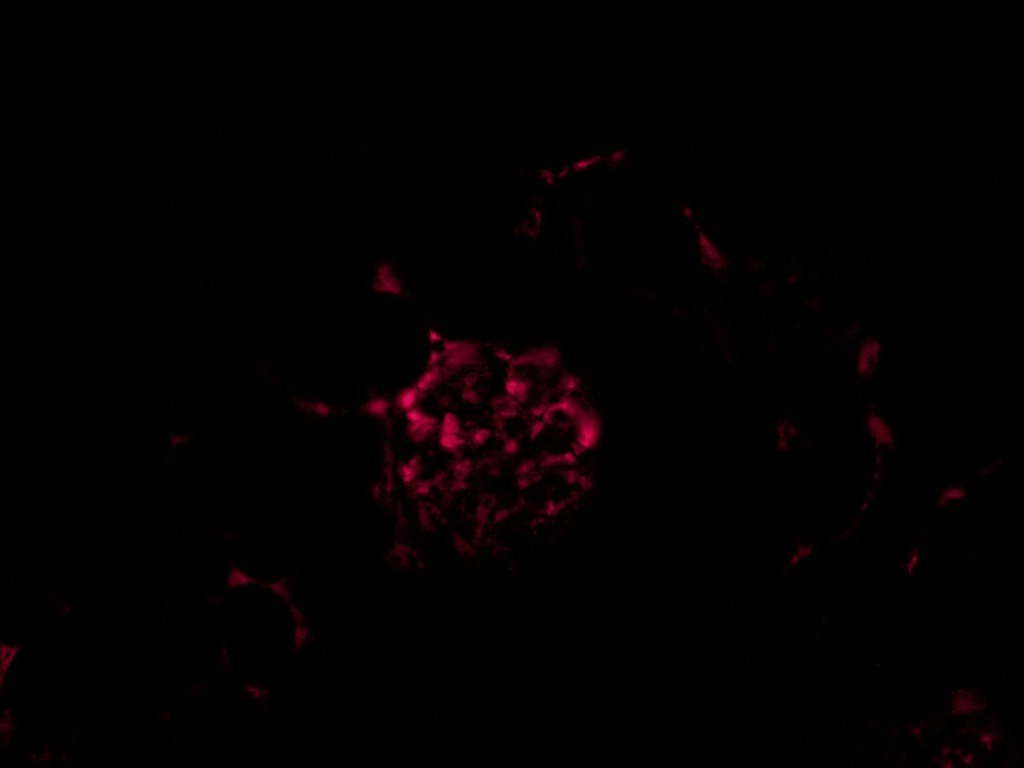

Supplement: Supplementary file 7 — Source data Fig. 6 [file 44321_2025_315_MOESM7_ESM.zip › Figure 6/F6A/2-C3/2-3 (2).jpg]

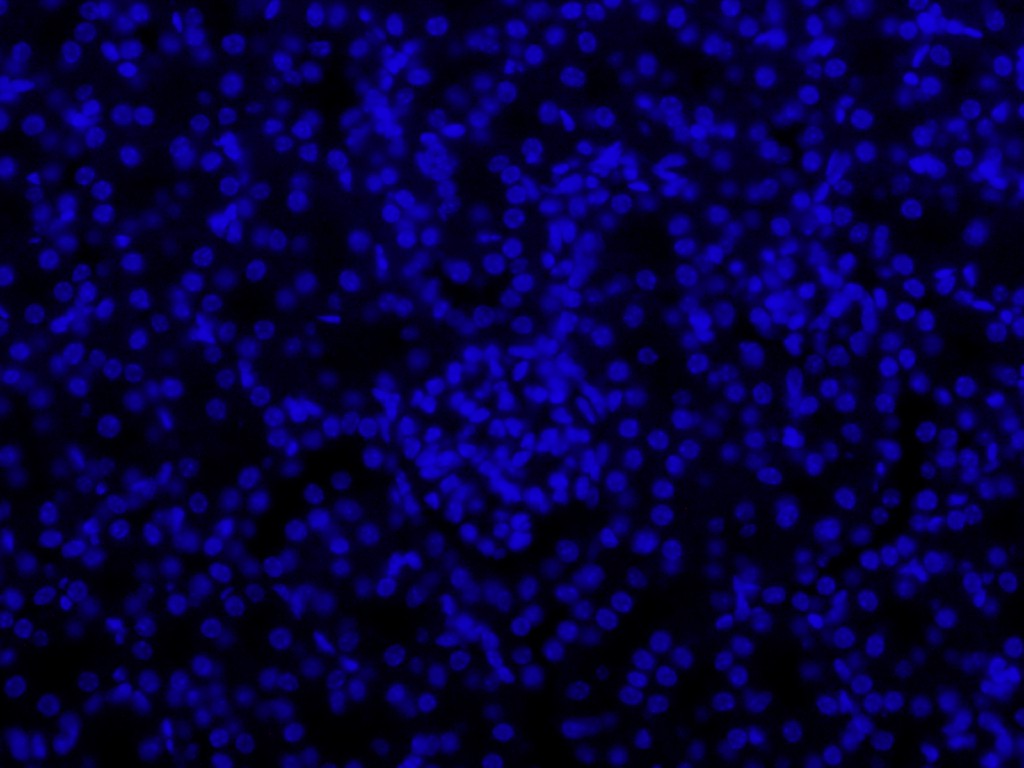

Supplement: Supplementary file 7 — Source data Fig. 6 [file 44321_2025_315_MOESM7_ESM.zip › Figure 6/F6A/2-C3/2-3 (3).jpg]

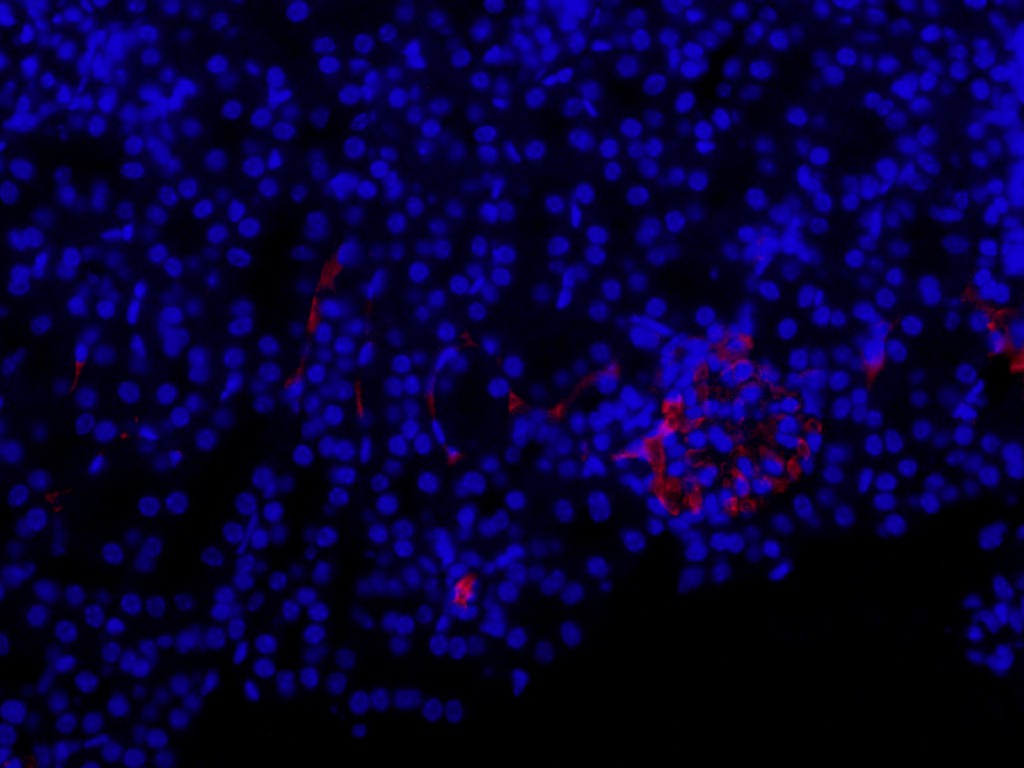

Supplement: Supplementary file 7 — Source data Fig. 6 [file 44321_2025_315_MOESM7_ESM.zip › Figure 6/F6A/2-C3/2-4 (1).jpg]

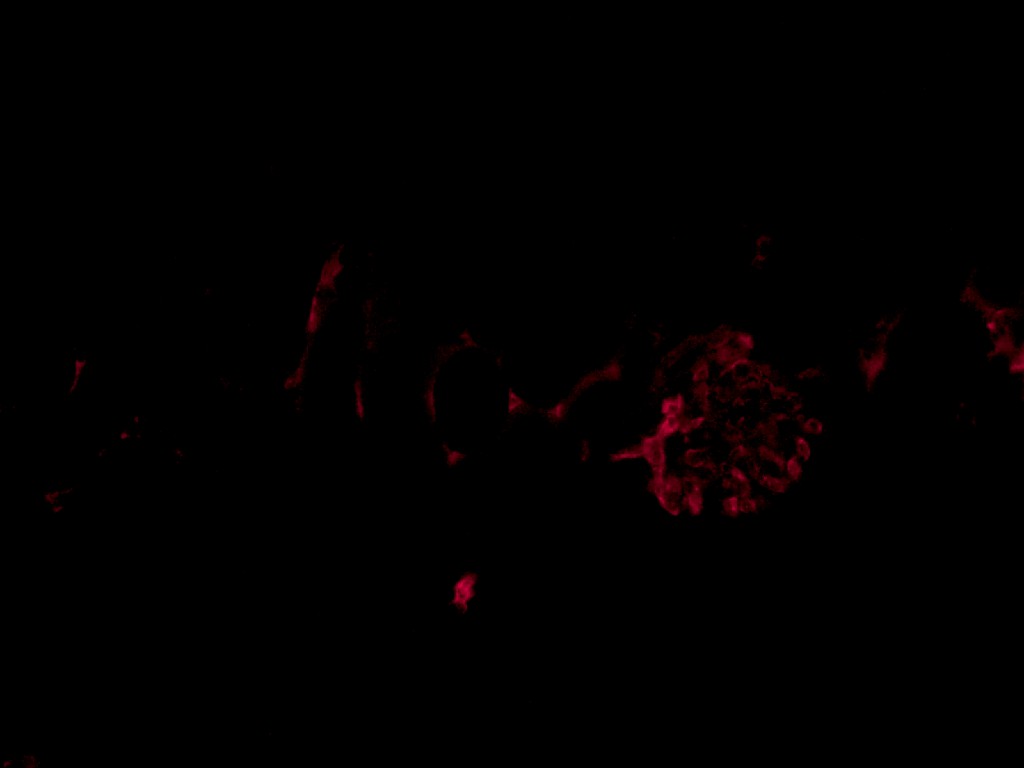

Supplement: Supplementary file 7 — Source data Fig. 6 [file 44321_2025_315_MOESM7_ESM.zip › Figure 6/F6A/2-C3/2-4 (2).jpg]

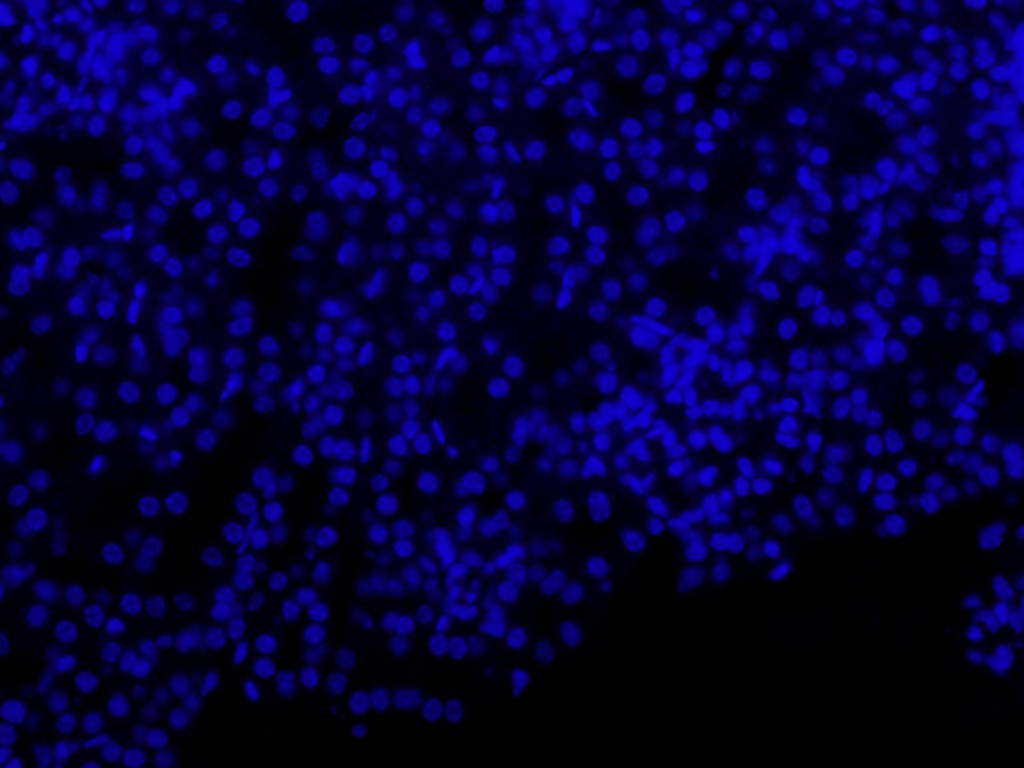

Supplement: Supplementary file 7 — Source data Fig. 6 [file 44321_2025_315_MOESM7_ESM.zip › Figure 6/F6A/2-C3/2-4 (3).jpg]

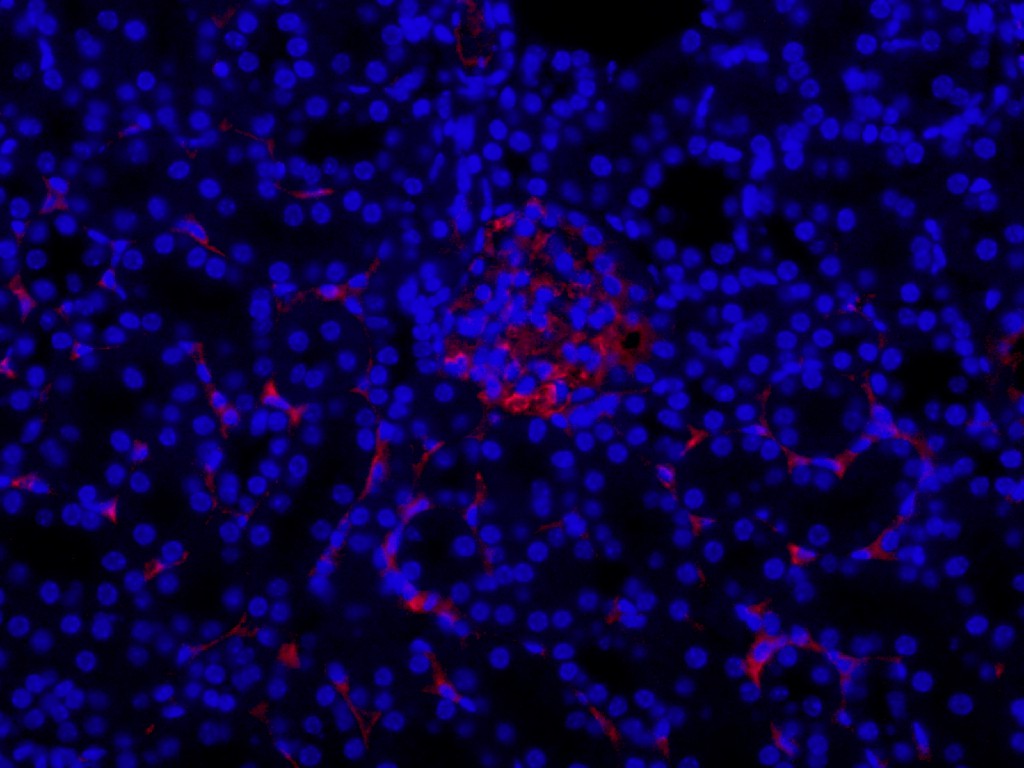

Supplement: Supplementary file 7 — Source data Fig. 6 [file 44321_2025_315_MOESM7_ESM.zip › Figure 6/F6A/2-C3/2-5 (1).jpg]

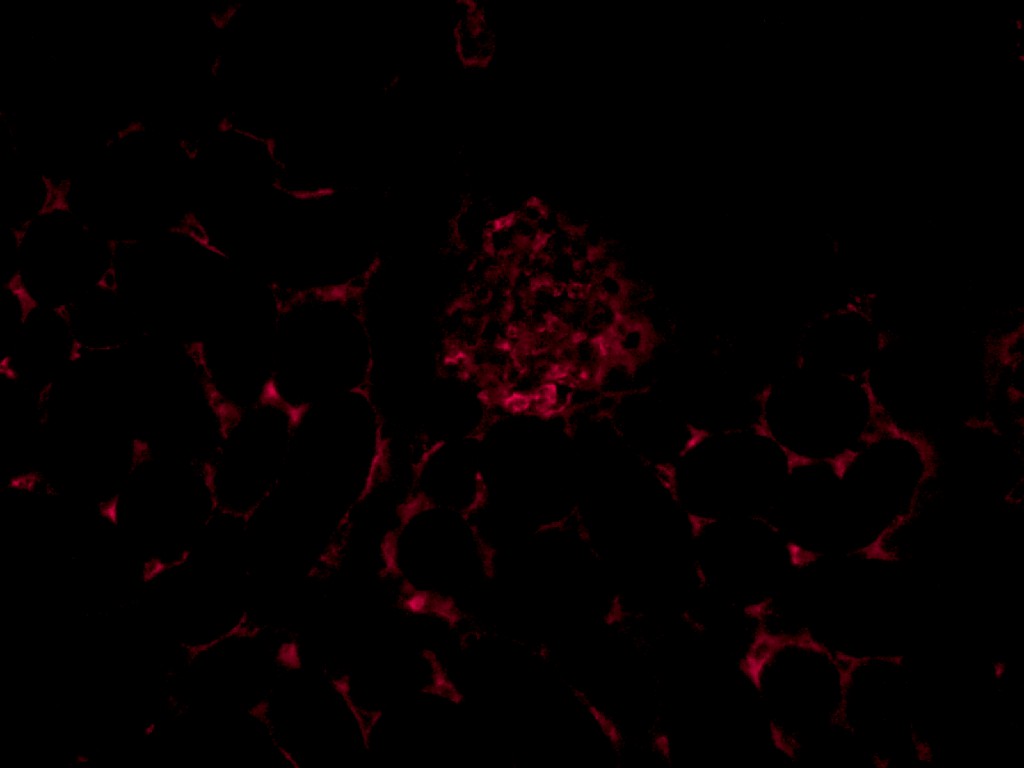

Supplement: Supplementary file 7 — Source data Fig. 6 [file 44321_2025_315_MOESM7_ESM.zip › Figure 6/F6A/2-C3/2-5 (2).jpg]

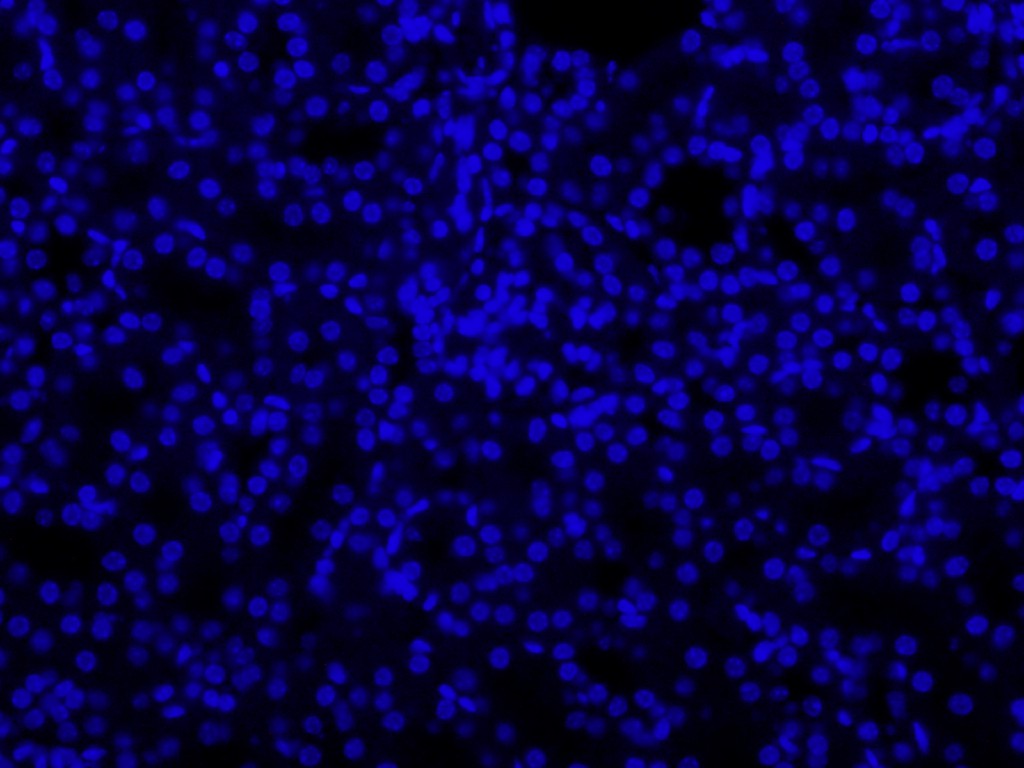

Supplement: Supplementary file 7 — Source data Fig. 6 [file 44321_2025_315_MOESM7_ESM.zip › Figure 6/F6A/2-C3/2-5 (3).jpg]

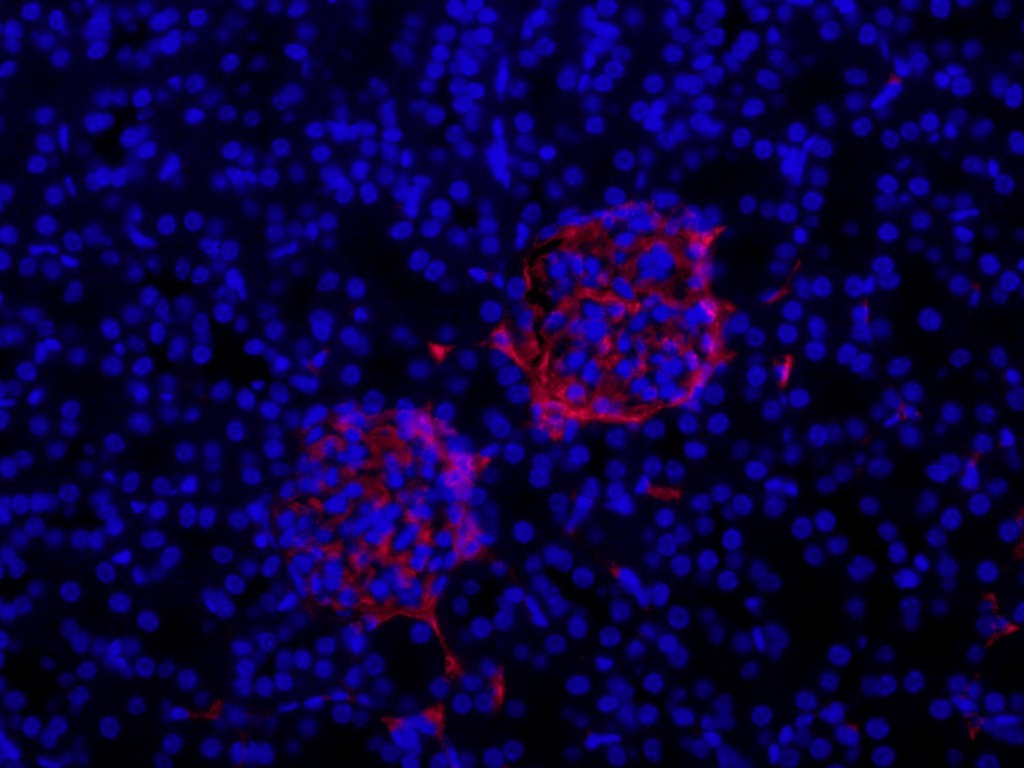

Supplement: Supplementary file 7 — Source data Fig. 6 [file 44321_2025_315_MOESM7_ESM.zip › Figure 6/F6A/2-C3/2-6 (1).jpg]

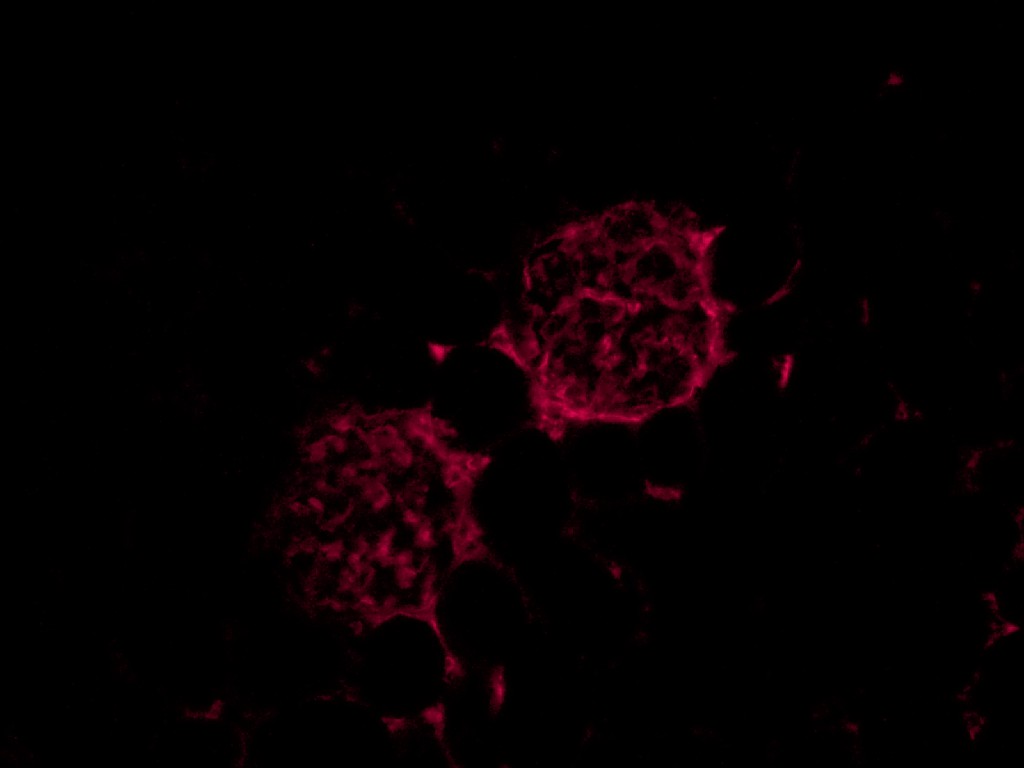

Supplement: Supplementary file 7 — Source data Fig. 6 [file 44321_2025_315_MOESM7_ESM.zip › Figure 6/F6A/2-C3/2-6 (2).jpg]

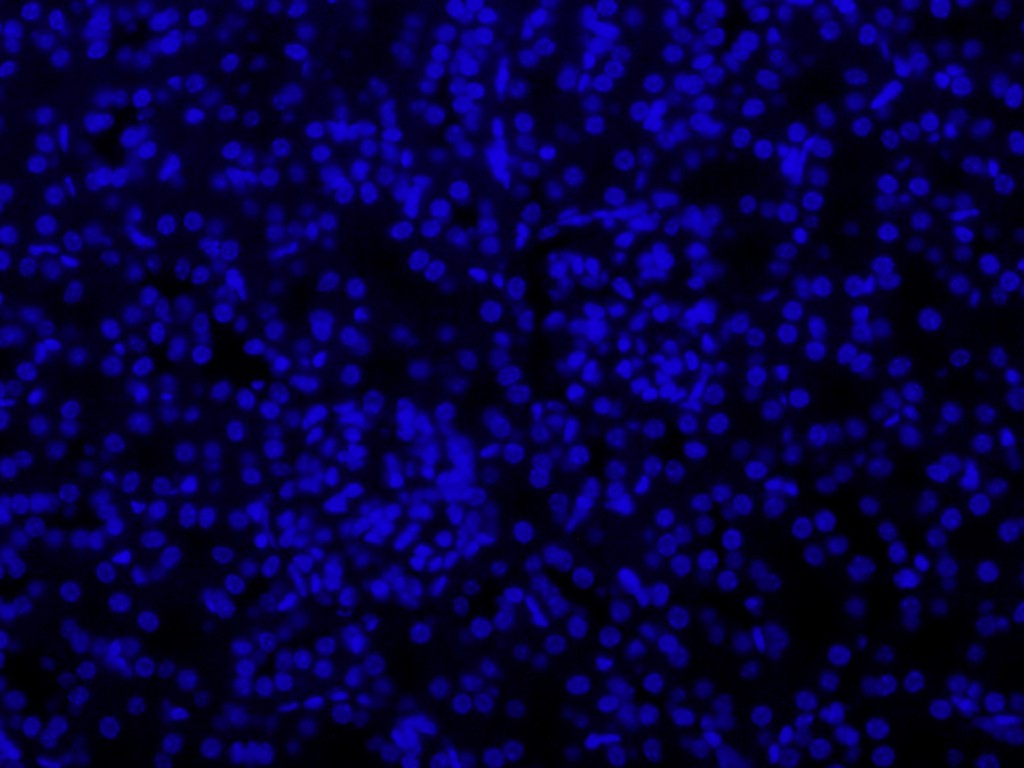

Supplement: Supplementary file 7 — Source data Fig. 6 [file 44321_2025_315_MOESM7_ESM.zip › Figure 6/F6A/2-C3/2-6 (3).jpg]

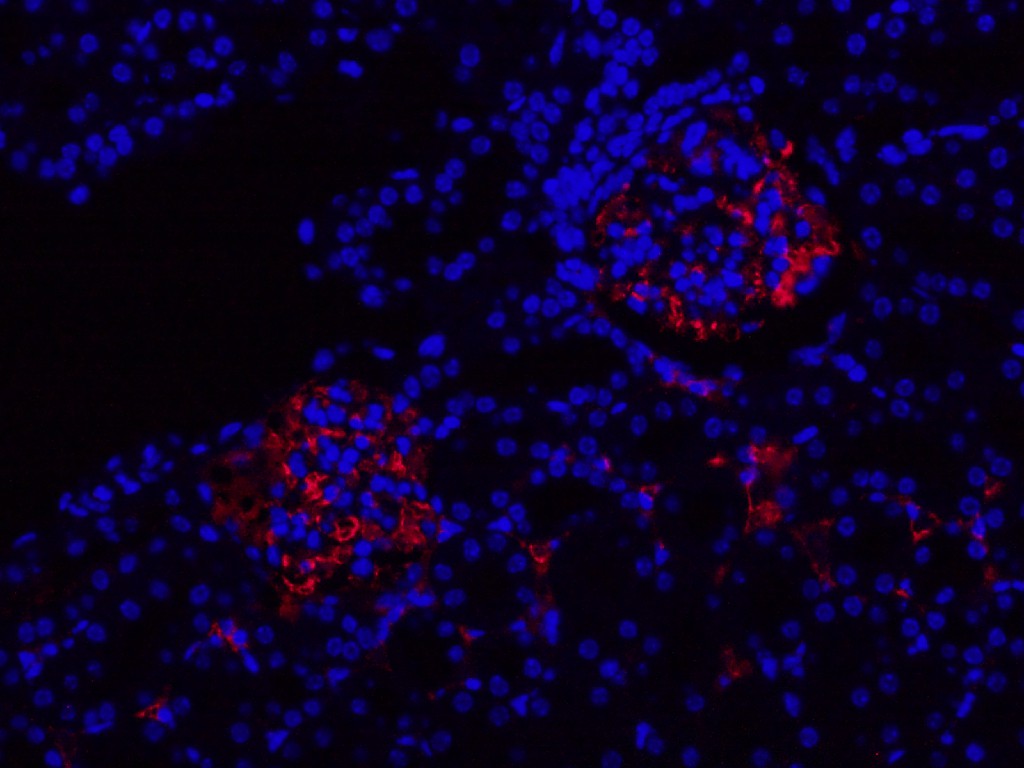

Supplement: Supplementary file 7 — Source data Fig. 6 [file 44321_2025_315_MOESM7_ESM.zip › Figure 6/F6A/2-C3/3-1 (1).jpg]

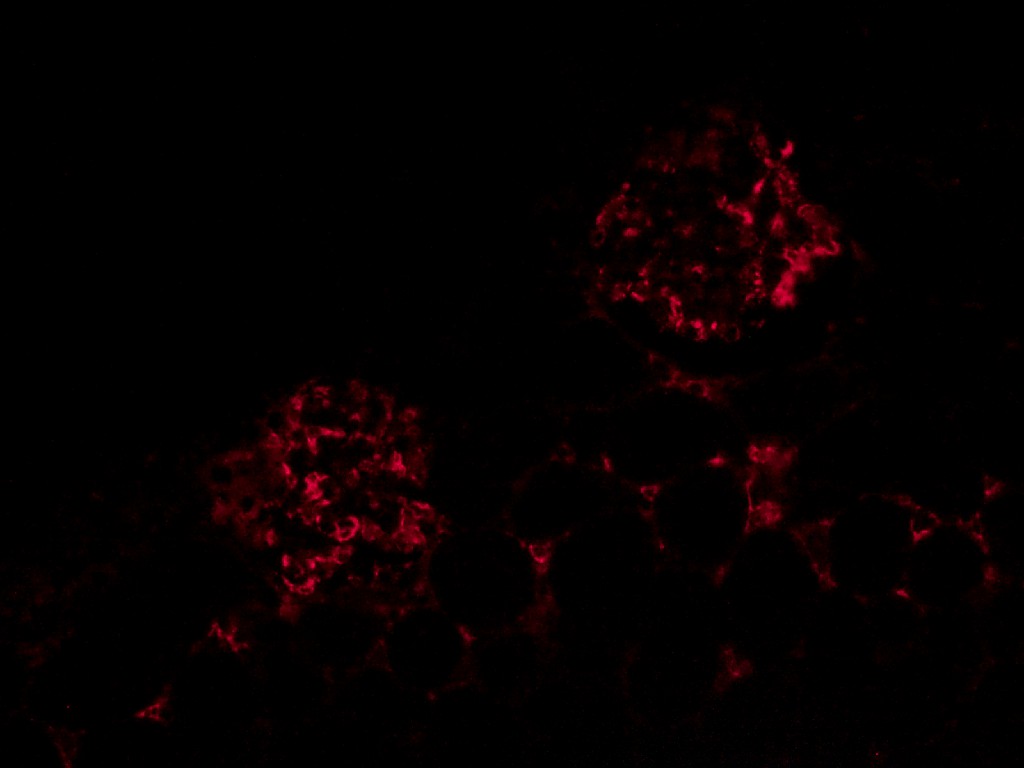

Supplement: Supplementary file 7 — Source data Fig. 6 [file 44321_2025_315_MOESM7_ESM.zip › Figure 6/F6A/2-C3/3-1 (2).jpg]

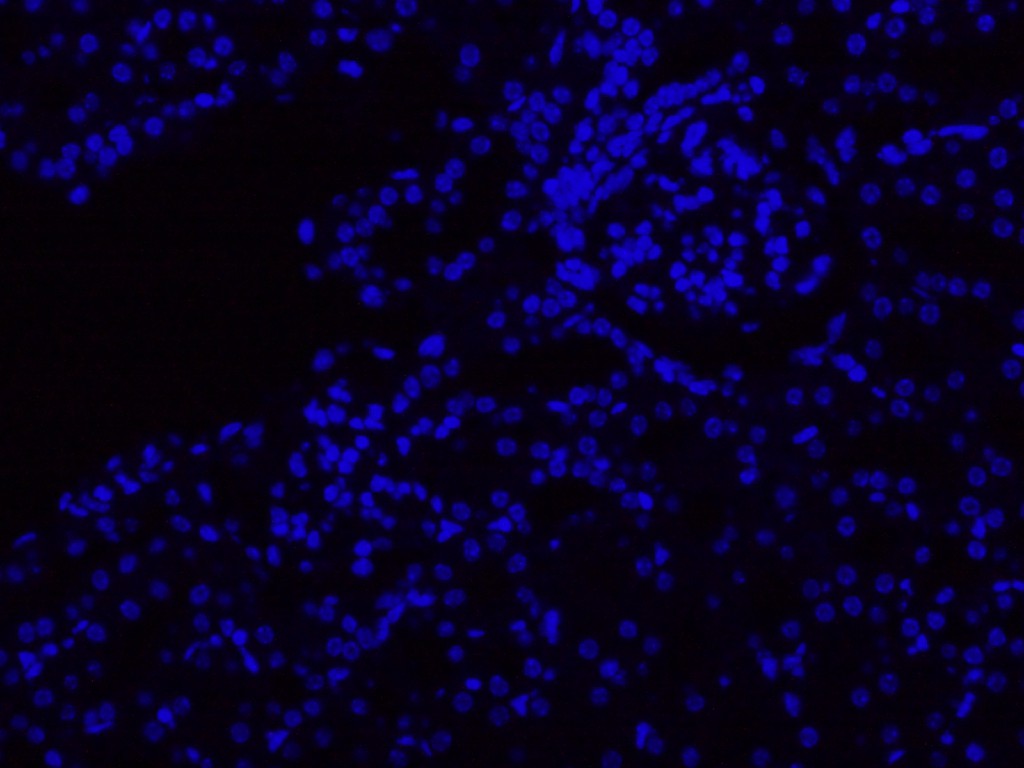

Supplement: Supplementary file 7 — Source data Fig. 6 [file 44321_2025_315_MOESM7_ESM.zip › Figure 6/F6A/2-C3/3-1 (3).jpg]

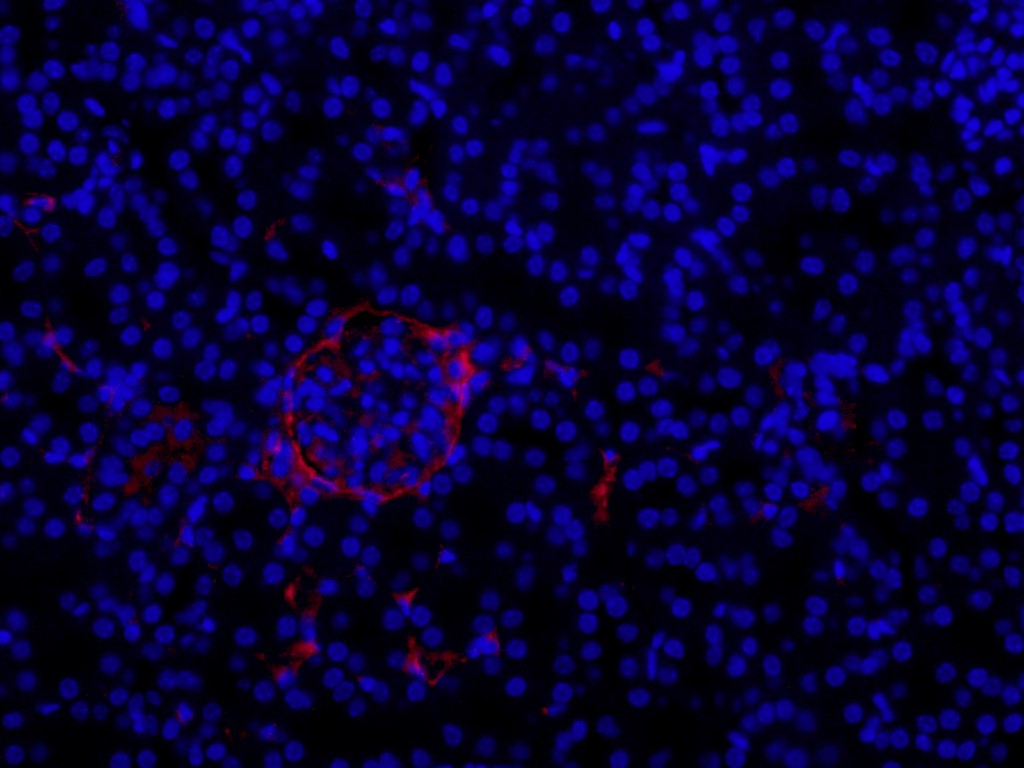

Supplement: Supplementary file 7 — Source data Fig. 6 [file 44321_2025_315_MOESM7_ESM.zip › Figure 6/F6A/2-C3/3-2 (1).jpg]

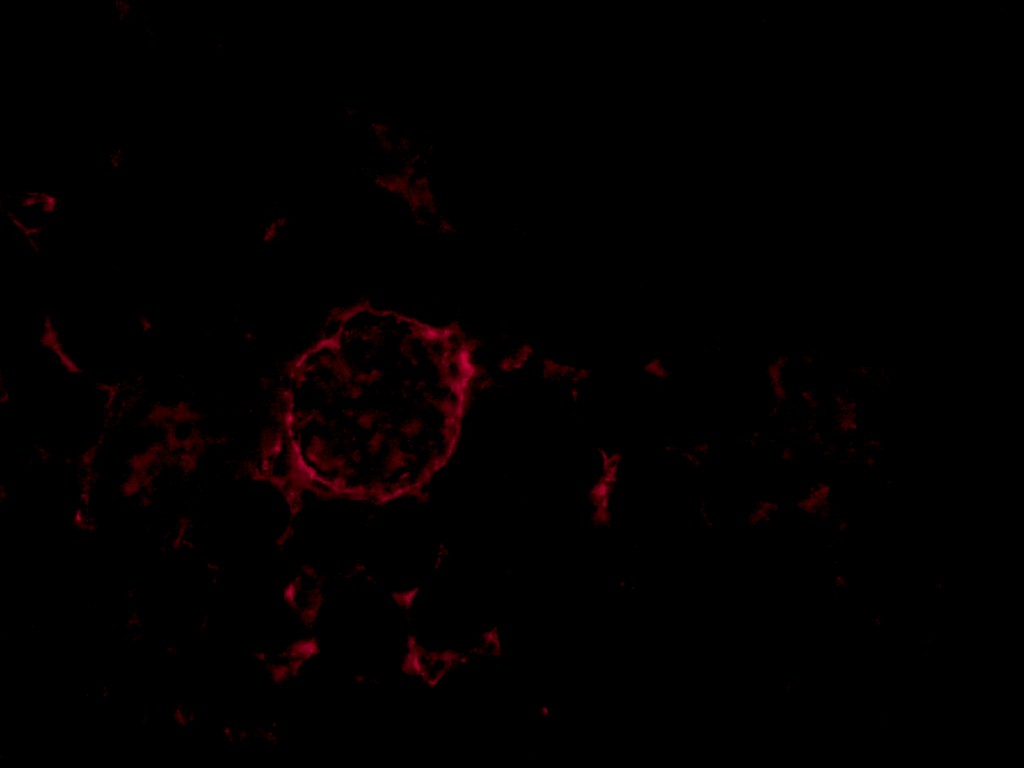

Supplement: Supplementary file 7 — Source data Fig. 6 [file 44321_2025_315_MOESM7_ESM.zip › Figure 6/F6A/2-C3/3-2 (2).jpg]

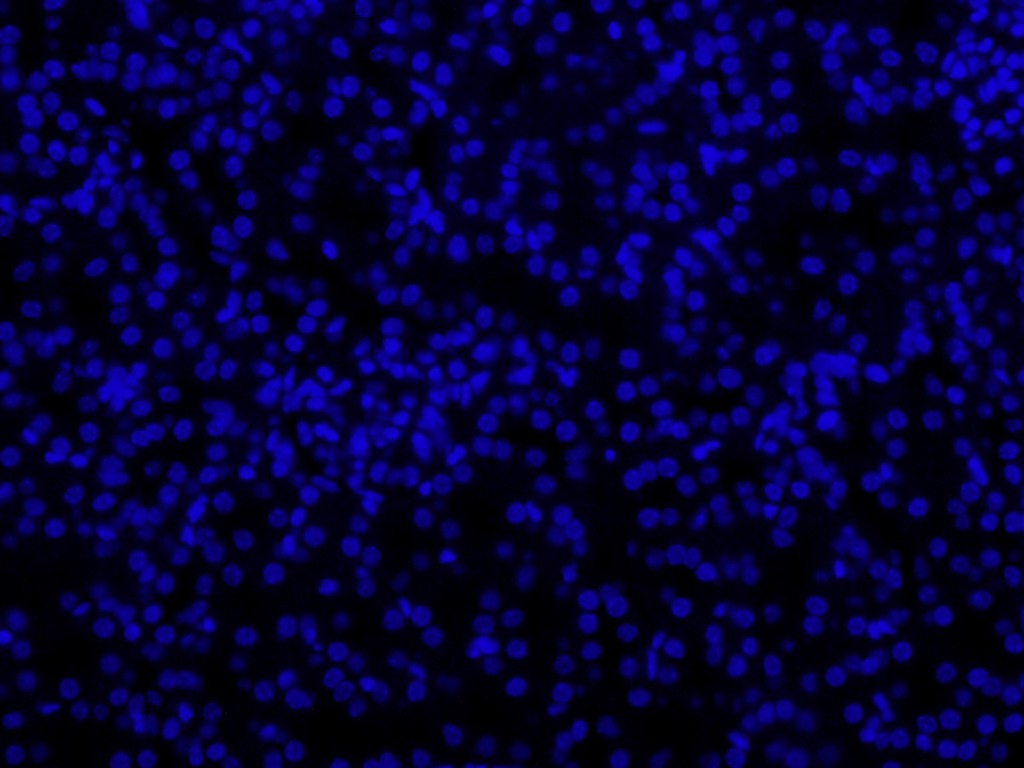

Supplement: Supplementary file 7 — Source data Fig. 6 [file 44321_2025_315_MOESM7_ESM.zip › Figure 6/F6A/2-C3/3-2 (3).jpg]

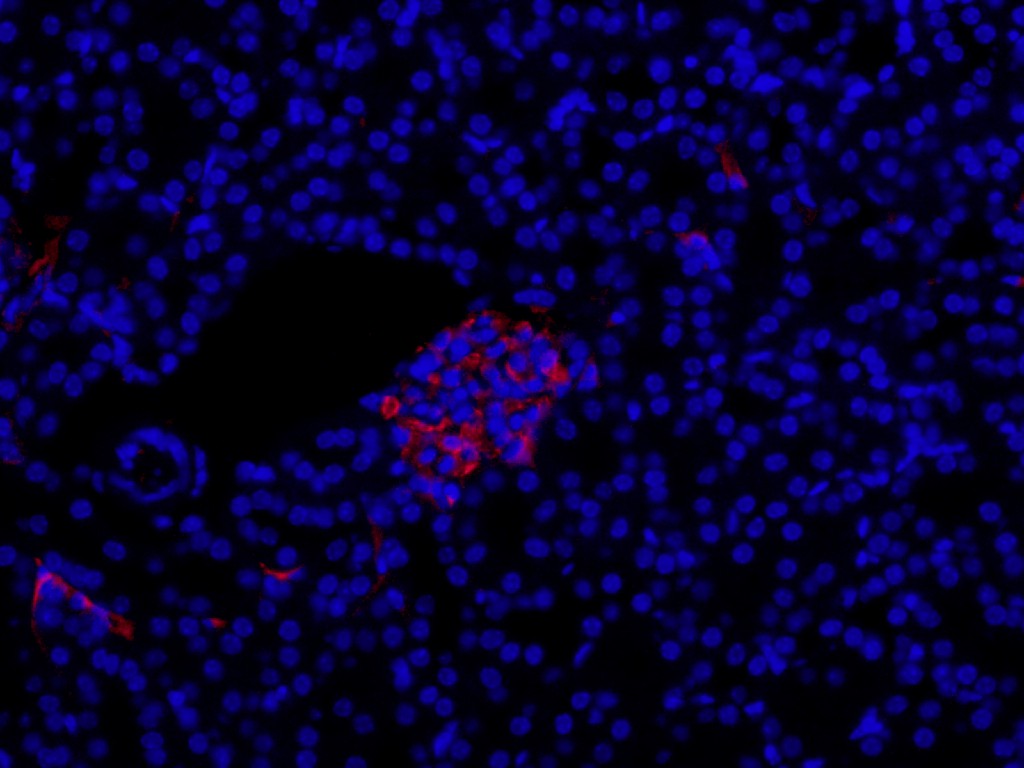

Supplement: Supplementary file 7 — Source data Fig. 6 [file 44321_2025_315_MOESM7_ESM.zip › Figure 6/F6A/2-C3/3-3 (1).jpg]

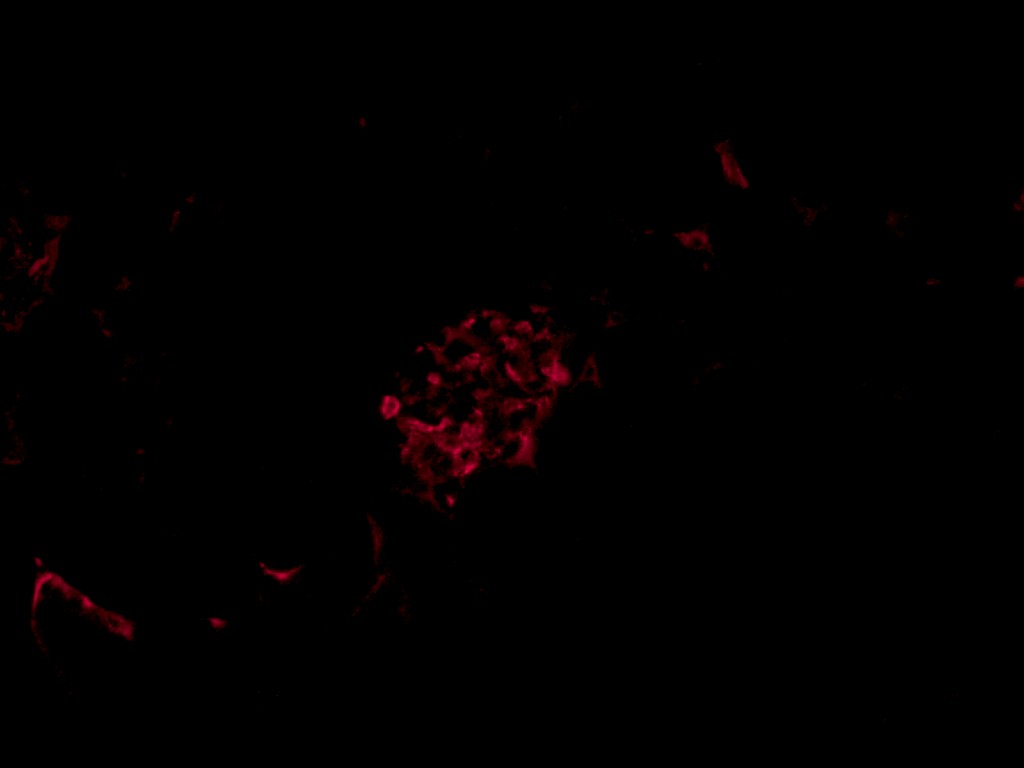

Supplement: Supplementary file 7 — Source data Fig. 6 [file 44321_2025_315_MOESM7_ESM.zip › Figure 6/F6A/2-C3/3-3 (2).jpg]

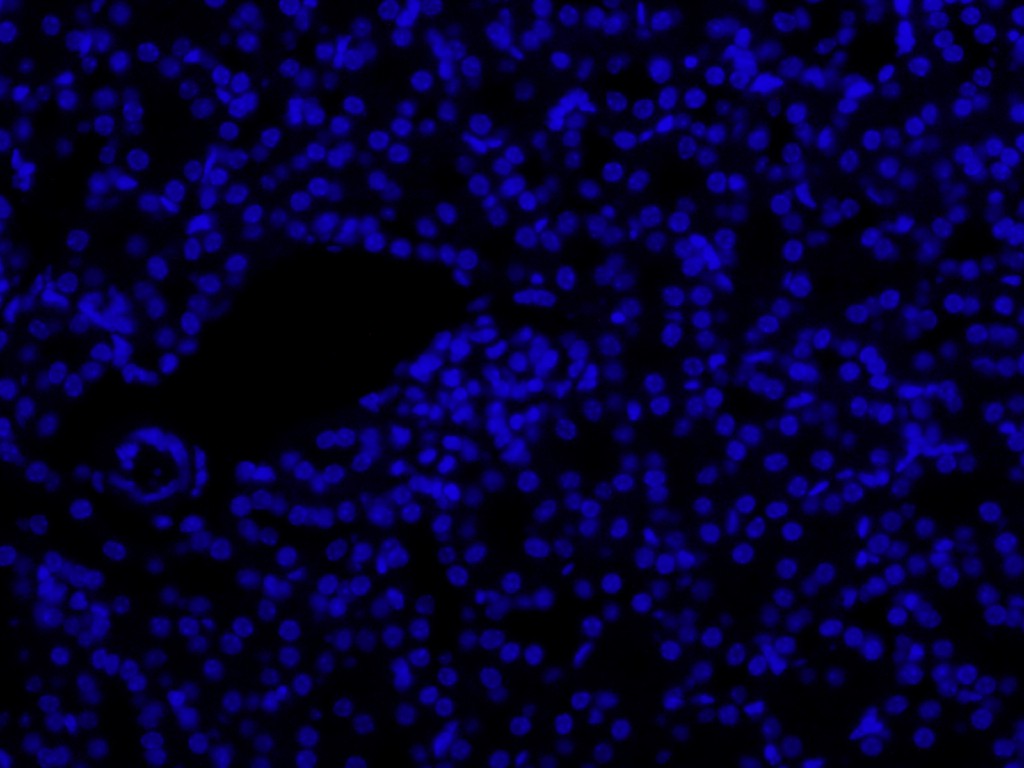

Supplement: Supplementary file 7 — Source data Fig. 6 [file 44321_2025_315_MOESM7_ESM.zip › Figure 6/F6A/2-C3/3-3 (3).jpg]

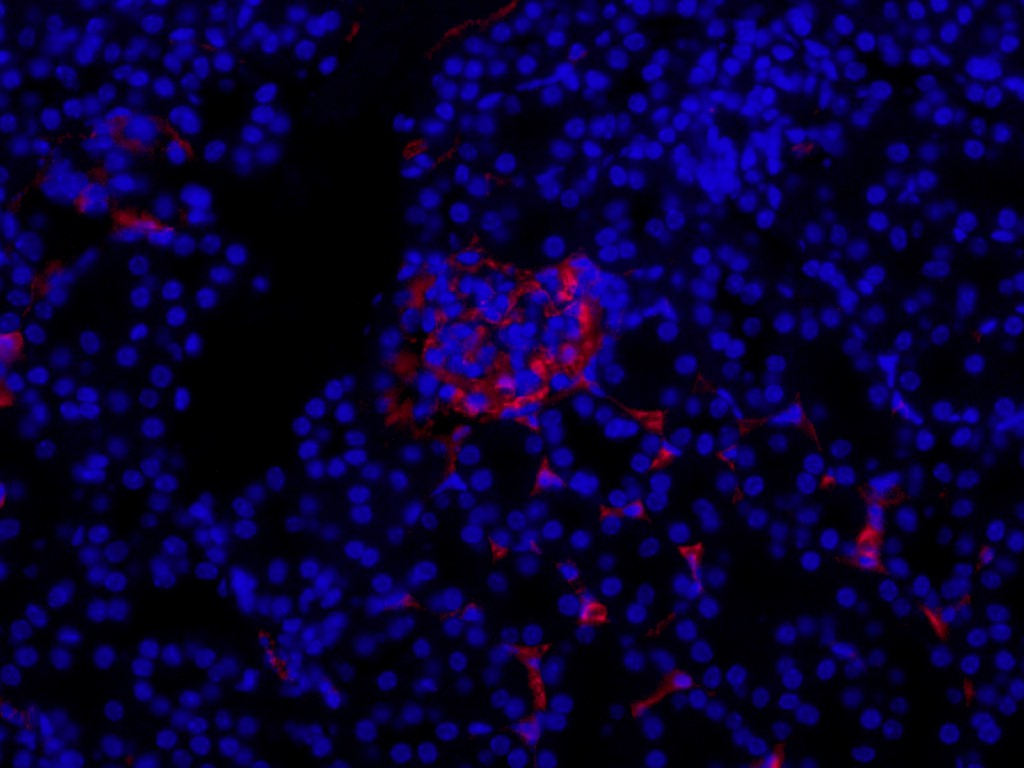

Supplement: Supplementary file 7 — Source data Fig. 6 [file 44321_2025_315_MOESM7_ESM.zip › Figure 6/F6A/2-C3/3-4 (1).jpg]

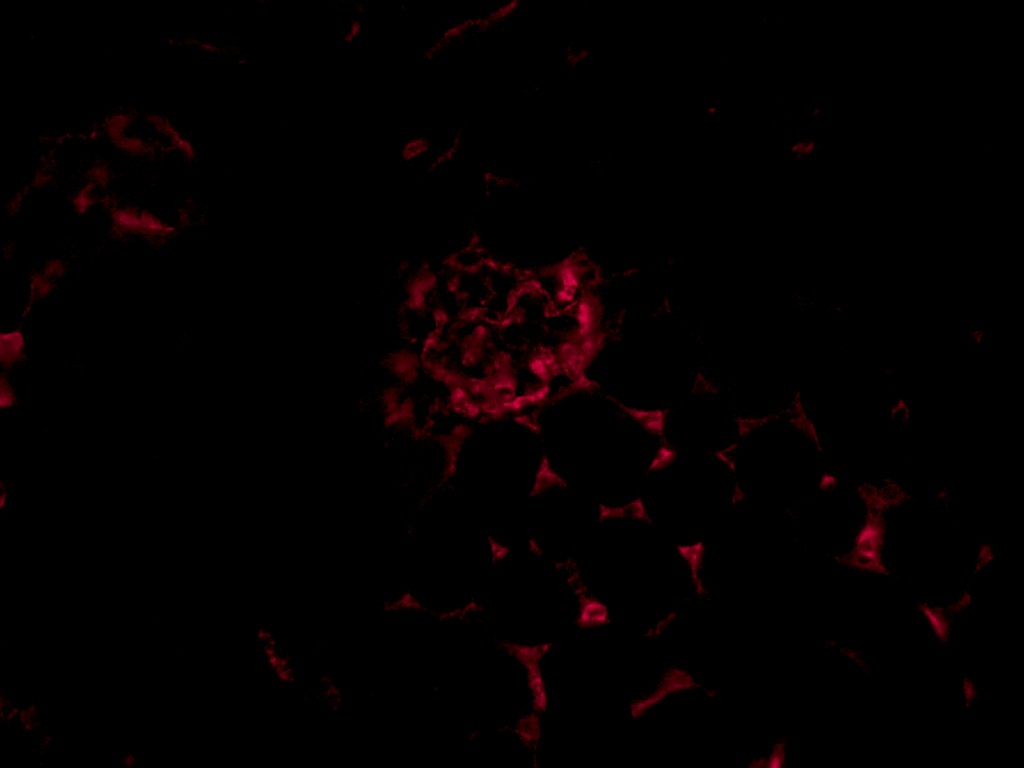

Supplement: Supplementary file 7 — Source data Fig. 6 [file 44321_2025_315_MOESM7_ESM.zip › Figure 6/F6A/2-C3/3-4 (2).jpg]

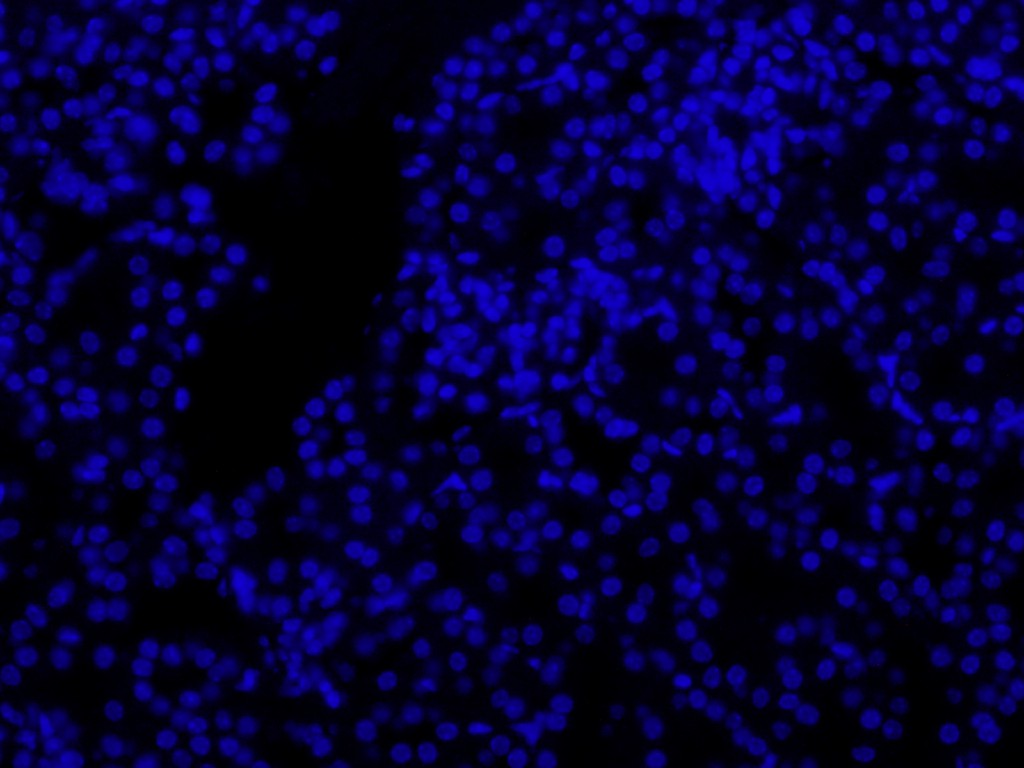

Supplement: Supplementary file 7 — Source data Fig. 6 [file 44321_2025_315_MOESM7_ESM.zip › Figure 6/F6A/2-C3/3-4 (3).jpg]

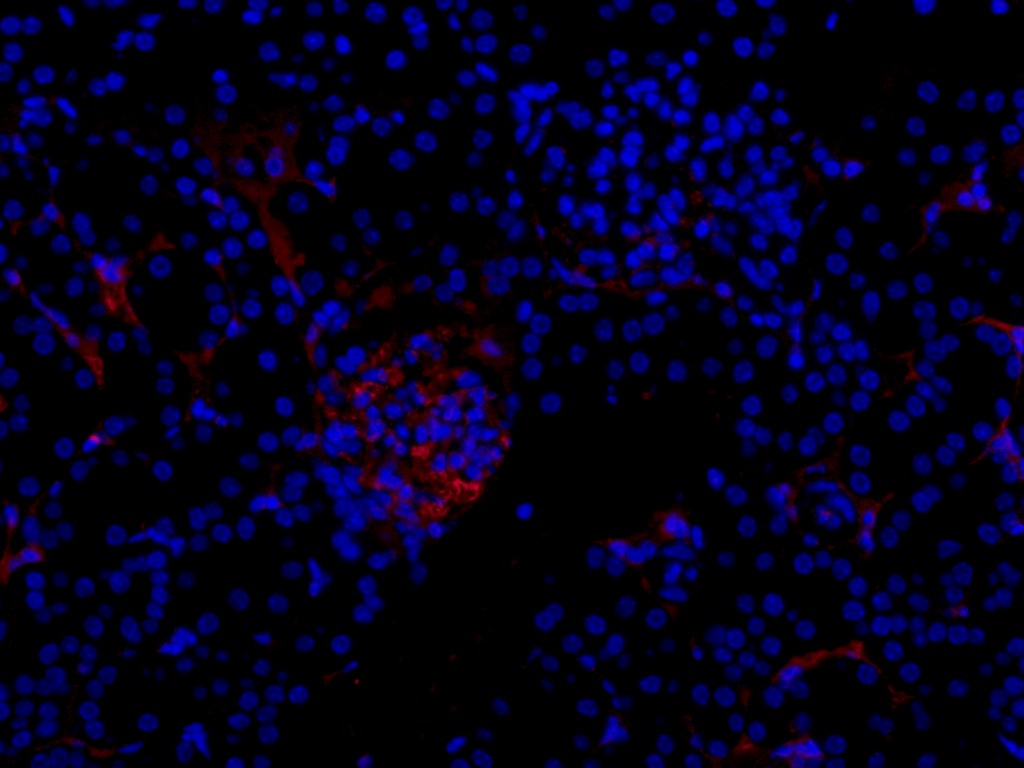

Supplement: Supplementary file 7 — Source data Fig. 6 [file 44321_2025_315_MOESM7_ESM.zip › Figure 6/F6A/2-C3/3-5 (1).jpg]

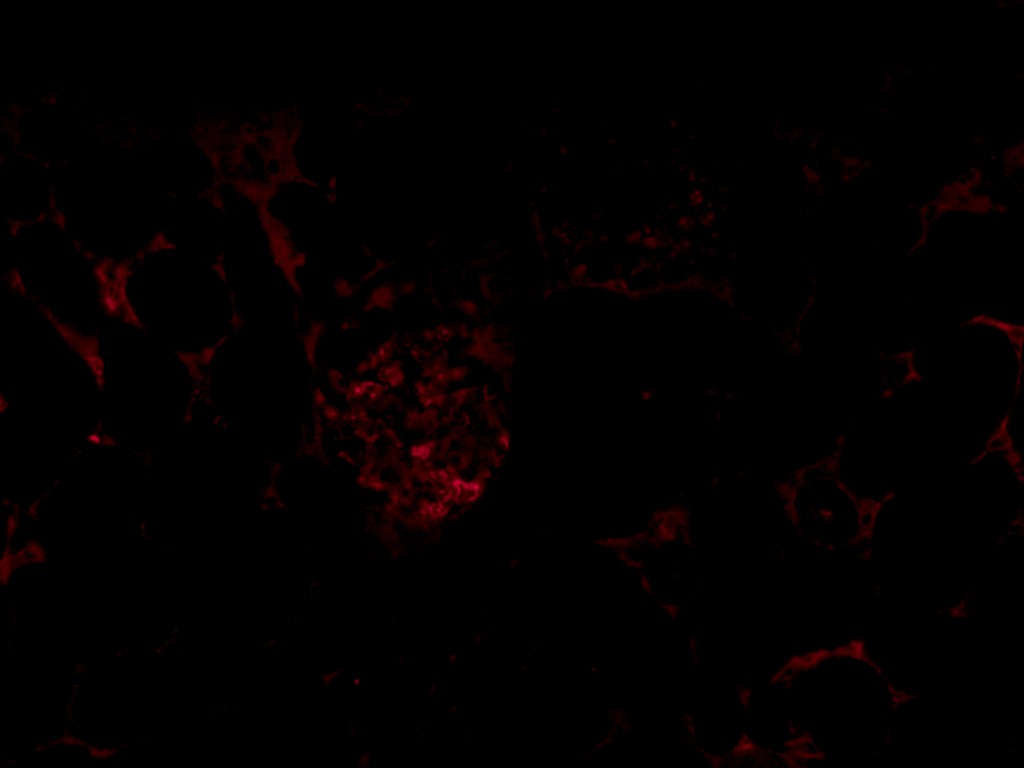

Supplement: Supplementary file 7 — Source data Fig. 6 [file 44321_2025_315_MOESM7_ESM.zip › Figure 6/F6A/2-C3/3-5 (2).jpg]

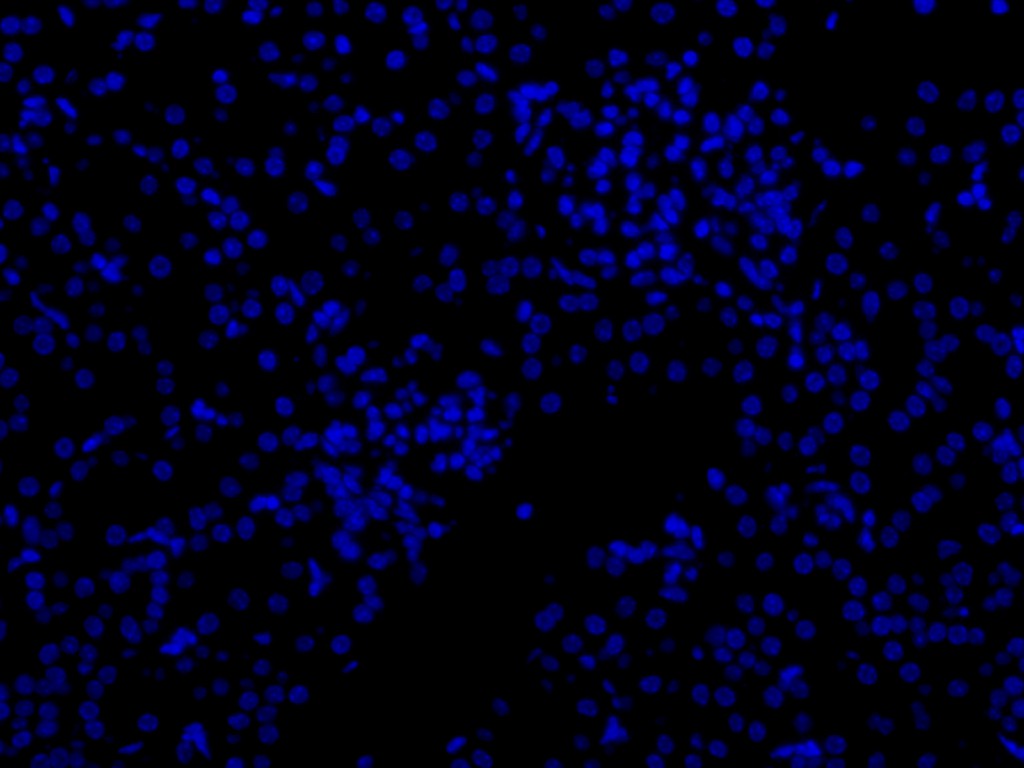

Supplement: Supplementary file 7 — Source data Fig. 6 [file 44321_2025_315_MOESM7_ESM.zip › Figure 6/F6A/2-C3/3-5 (3).jpg]

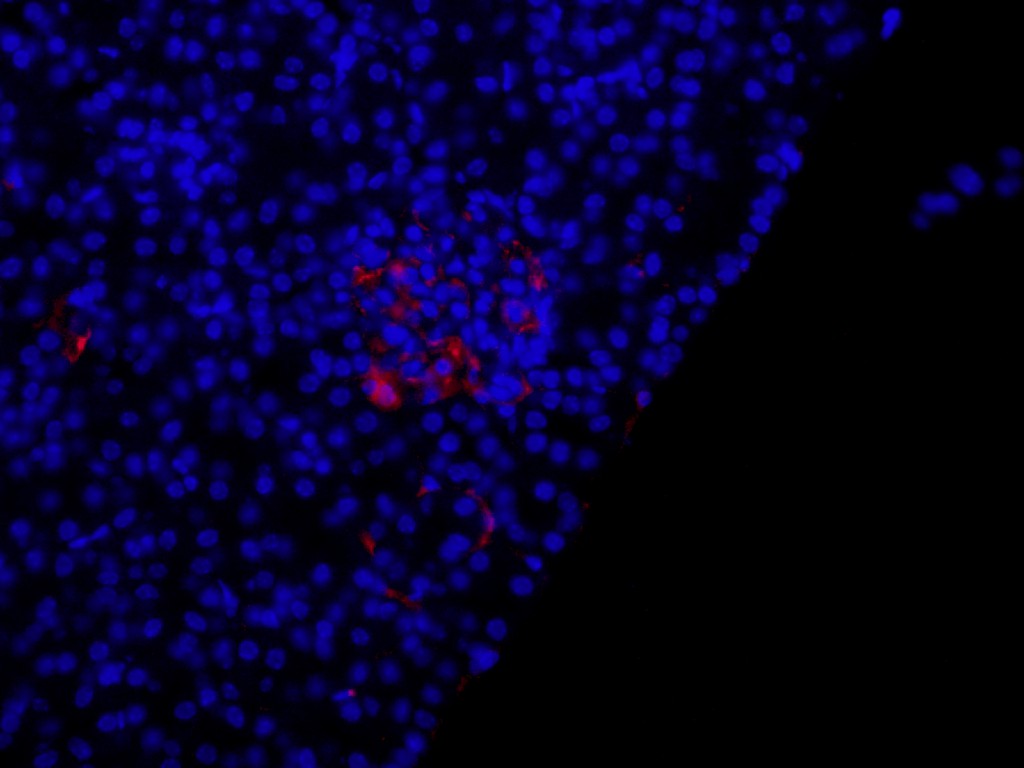

Supplement: Supplementary file 7 — Source data Fig. 6 [file 44321_2025_315_MOESM7_ESM.zip › Figure 6/F6A/2-C3/3-6 (1).jpg]

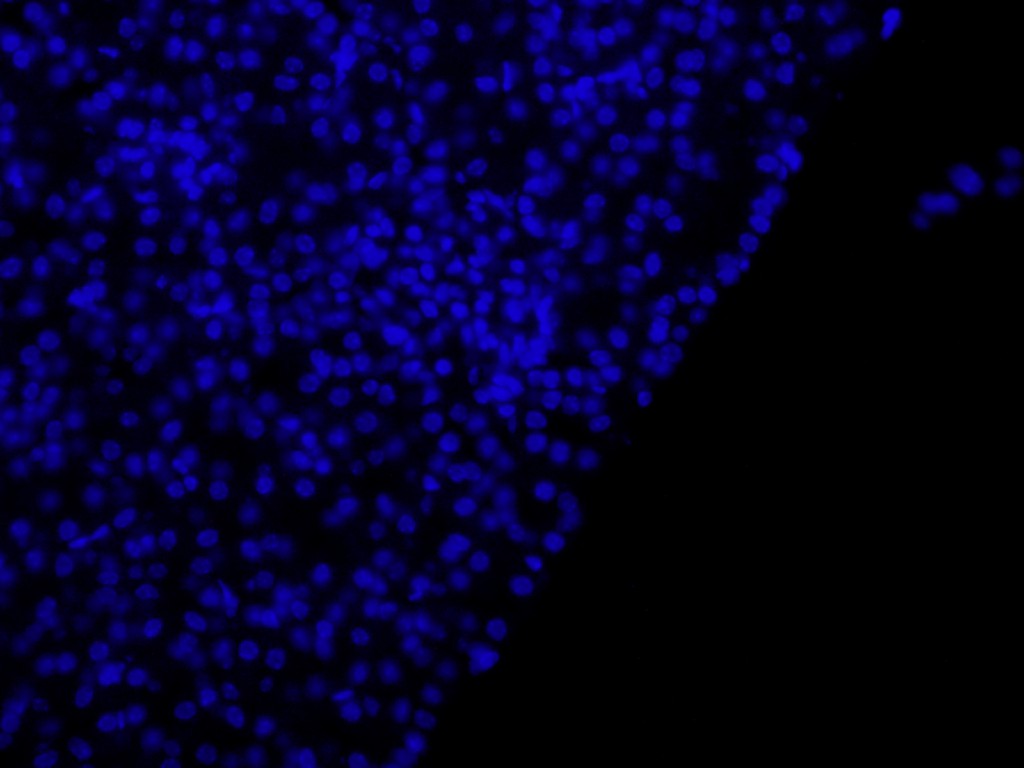

Supplement: Supplementary file 7 — Source data Fig. 6 [file 44321_2025_315_MOESM7_ESM.zip › Figure 6/F6A/2-C3/3-6 (2).jpg]

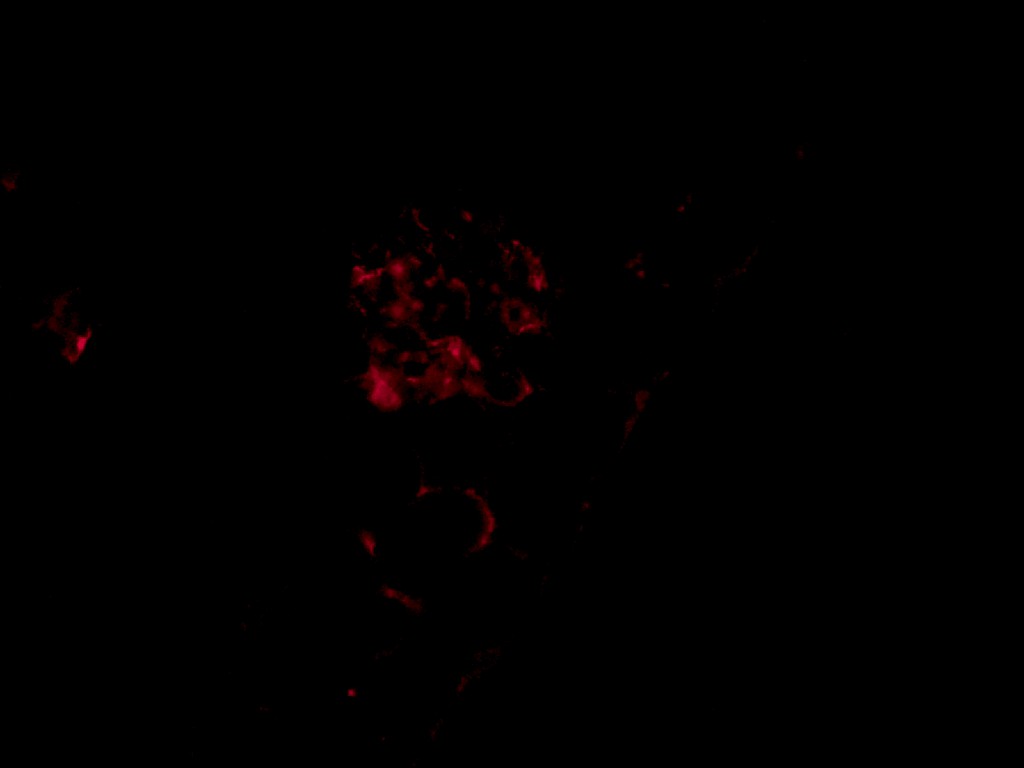

Supplement: Supplementary file 7 — Source data Fig. 6 [file 44321_2025_315_MOESM7_ESM.zip › Figure 6/F6A/2-C3/3-6 (3).jpg]

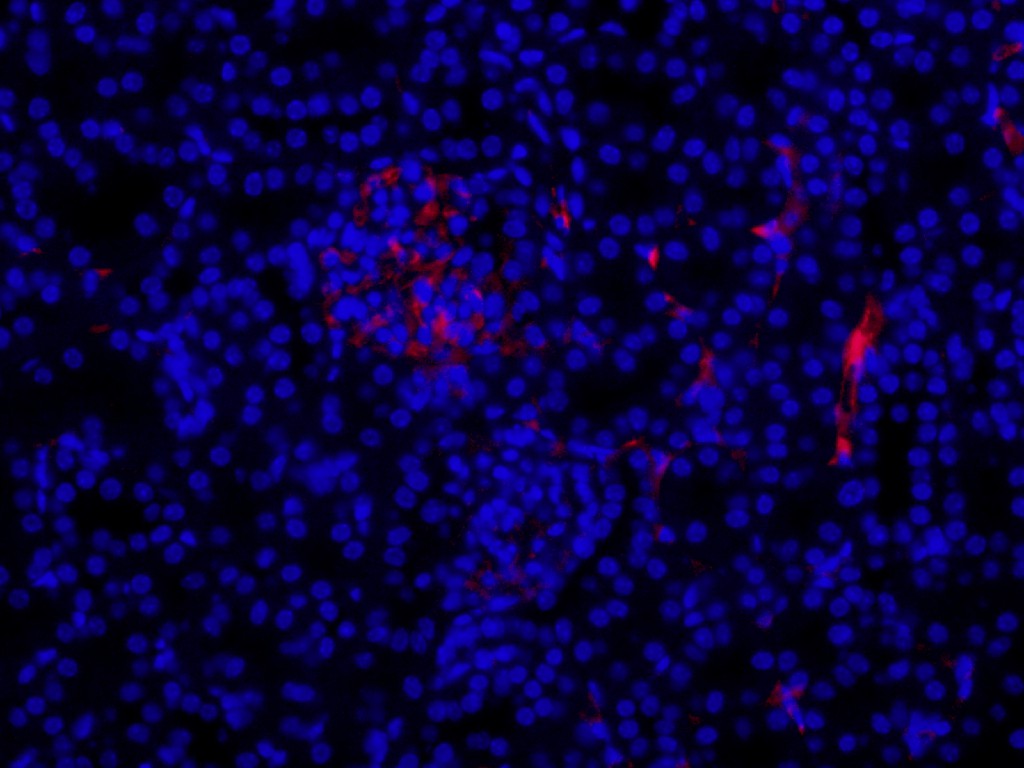

Supplement: Supplementary file 7 — Source data Fig. 6 [file 44321_2025_315_MOESM7_ESM.zip › Figure 6/F6A/2-C3/4-1 (1).jpg]

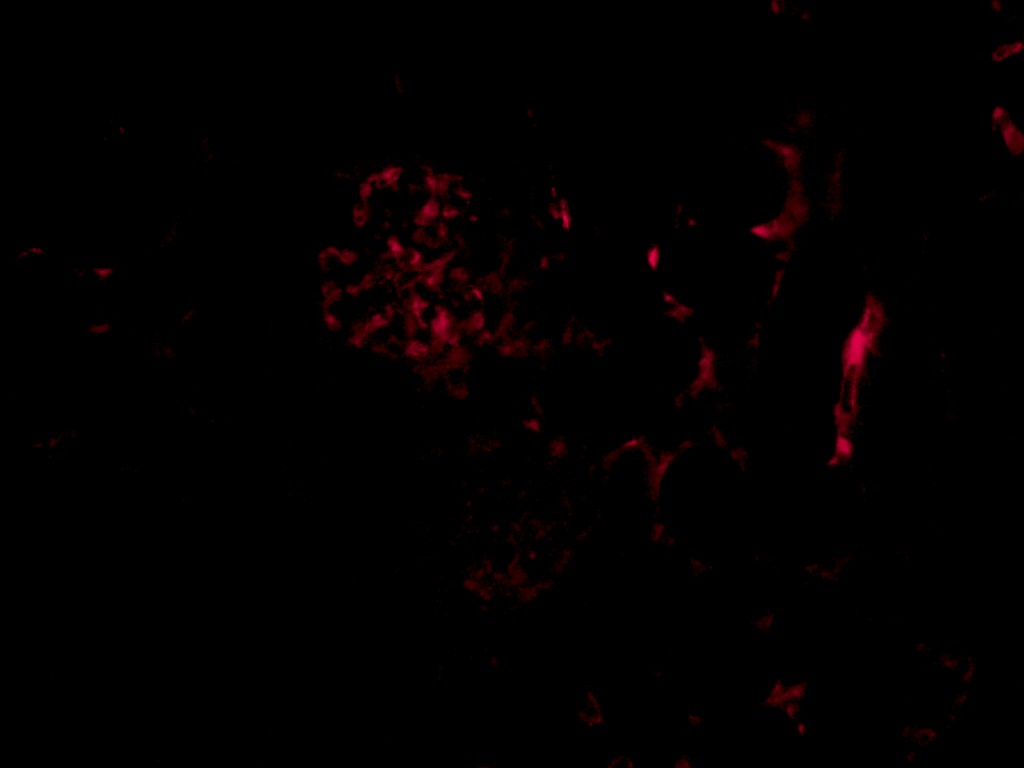

Supplement: Supplementary file 7 — Source data Fig. 6 [file 44321_2025_315_MOESM7_ESM.zip › Figure 6/F6A/2-C3/4-1 (2).jpg]

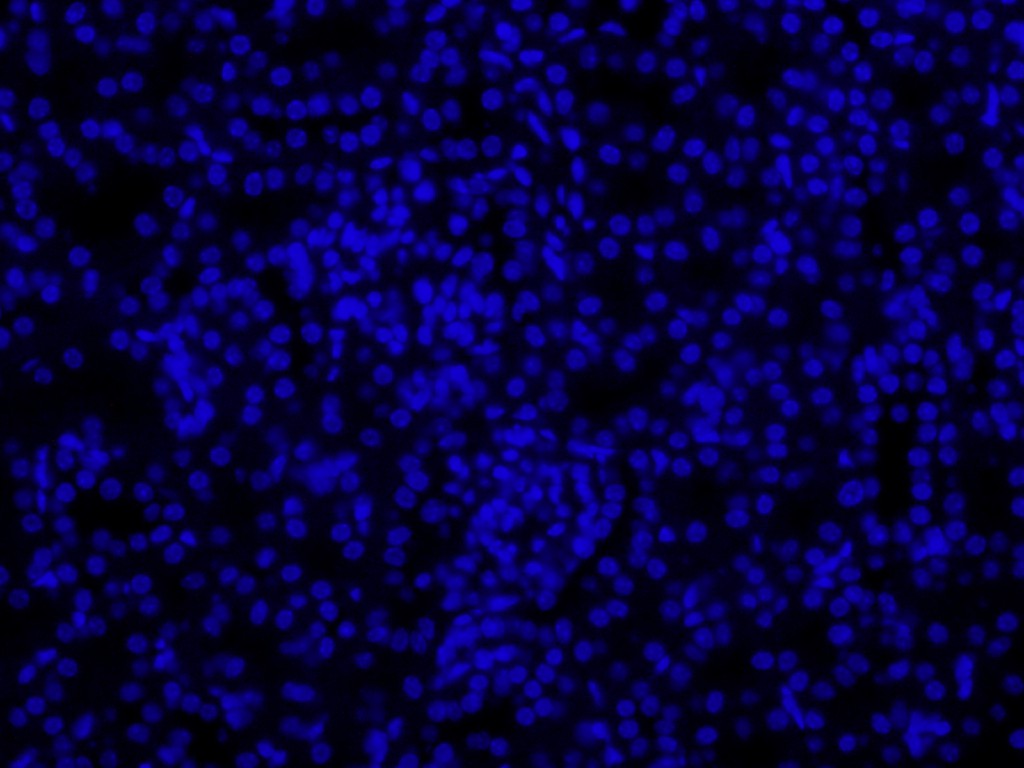

Supplement: Supplementary file 7 — Source data Fig. 6 [file 44321_2025_315_MOESM7_ESM.zip › Figure 6/F6A/2-C3/4-1 (3).jpg]

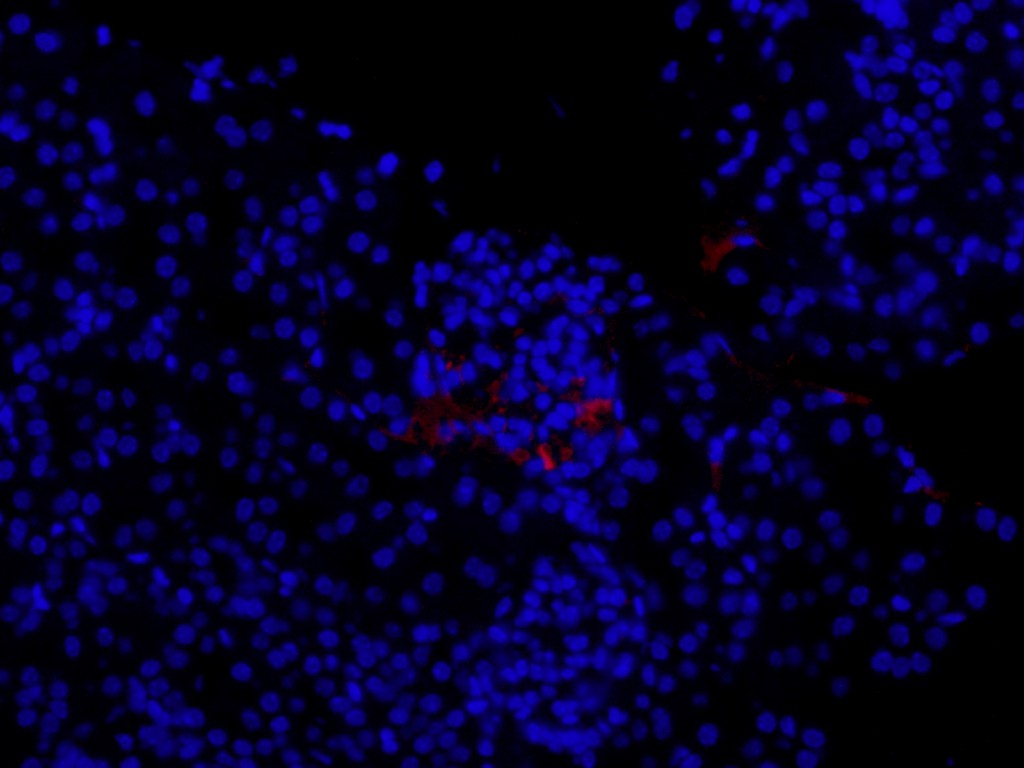

Supplement: Supplementary file 7 — Source data Fig. 6 [file 44321_2025_315_MOESM7_ESM.zip › Figure 6/F6A/2-C3/4-2 (1).jpg]

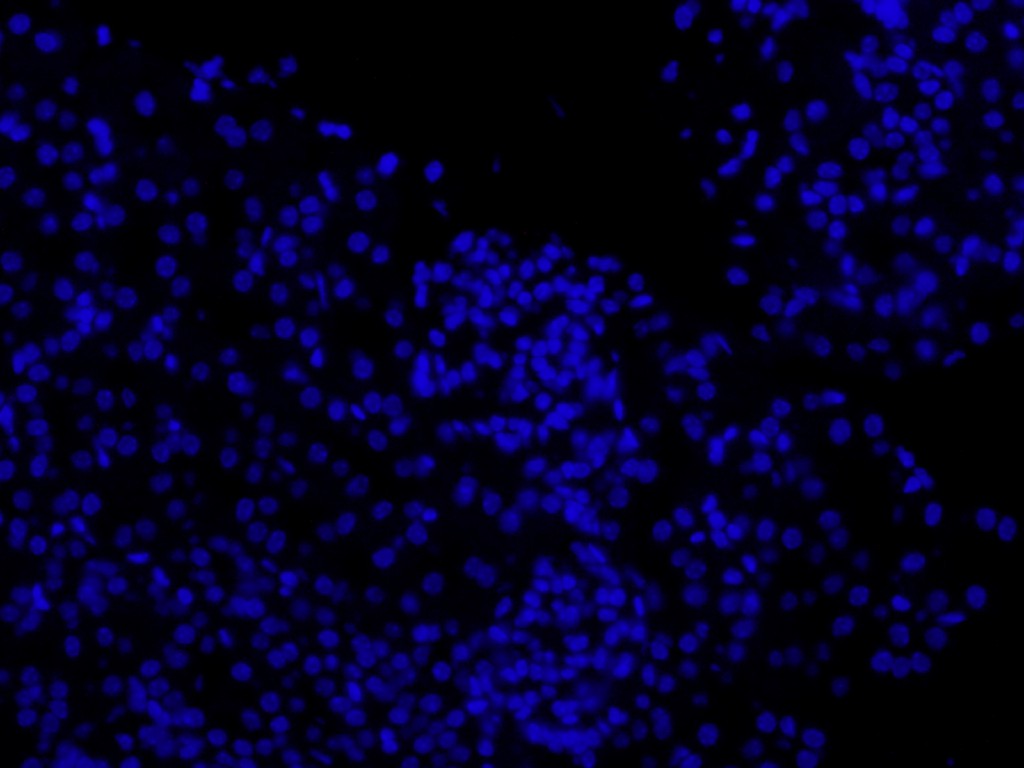

Supplement: Supplementary file 7 — Source data Fig. 6 [file 44321_2025_315_MOESM7_ESM.zip › Figure 6/F6A/2-C3/4-2 (2).jpg]

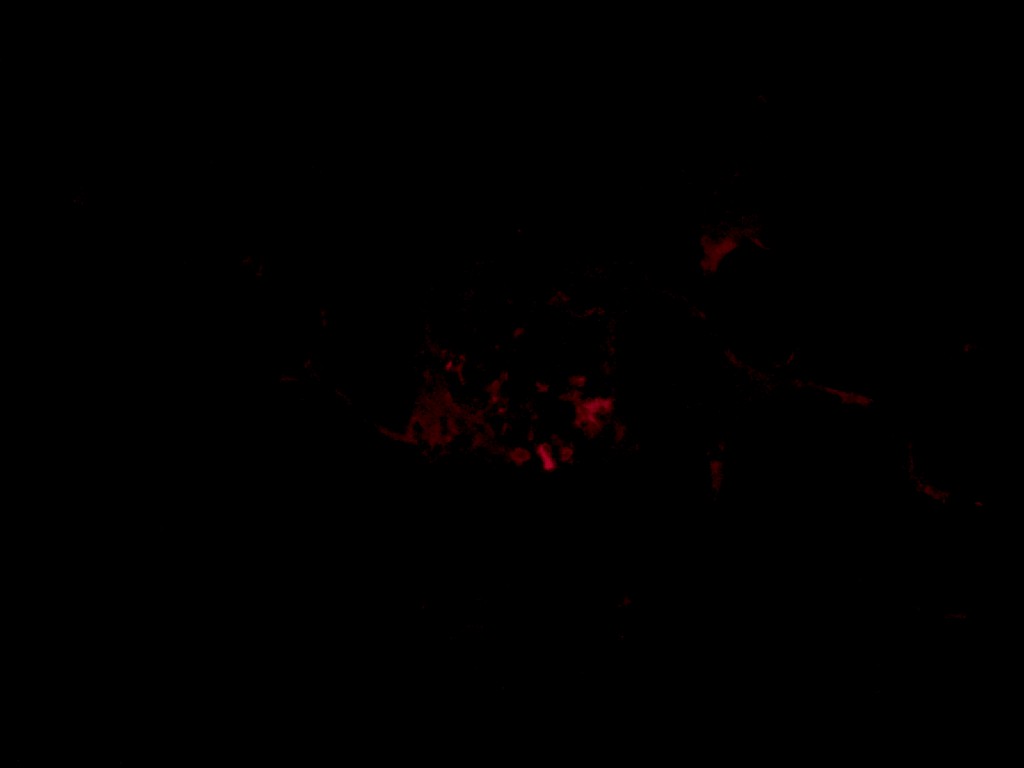

Supplement: Supplementary file 7 — Source data Fig. 6 [file 44321_2025_315_MOESM7_ESM.zip › Figure 6/F6A/2-C3/4-2 (3).jpg]

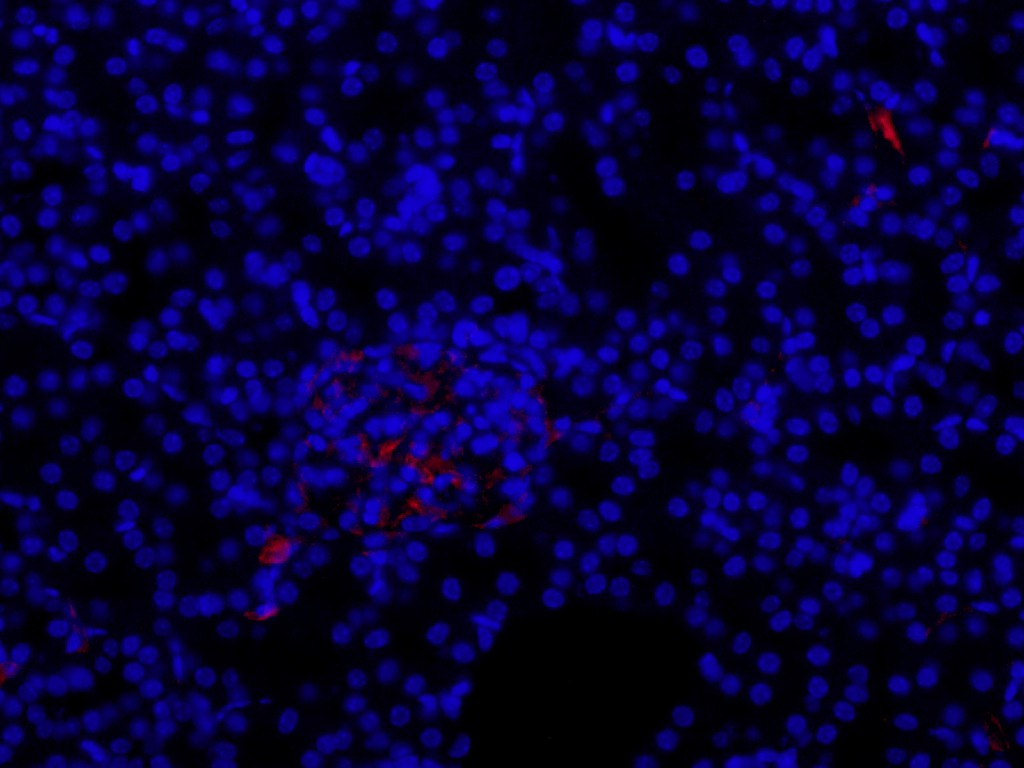

Supplement: Supplementary file 7 — Source data Fig. 6 [file 44321_2025_315_MOESM7_ESM.zip › Figure 6/F6A/2-C3/4-3 (1).jpg]

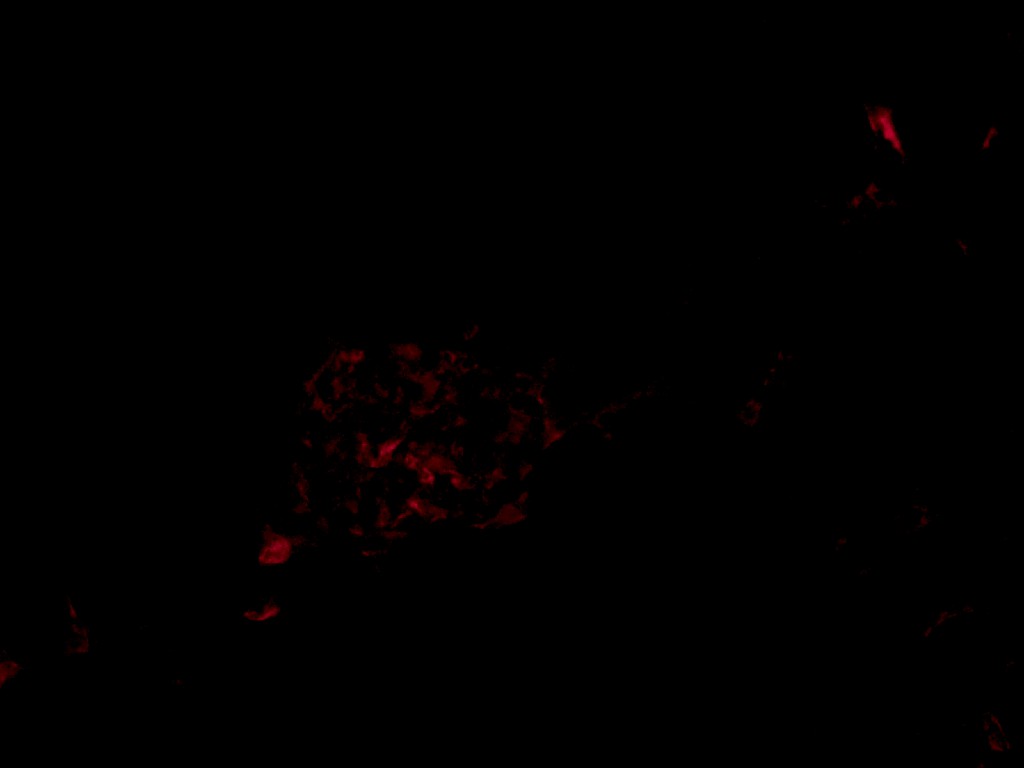

Supplement: Supplementary file 7 — Source data Fig. 6 [file 44321_2025_315_MOESM7_ESM.zip › Figure 6/F6A/2-C3/4-3 (2).jpg]

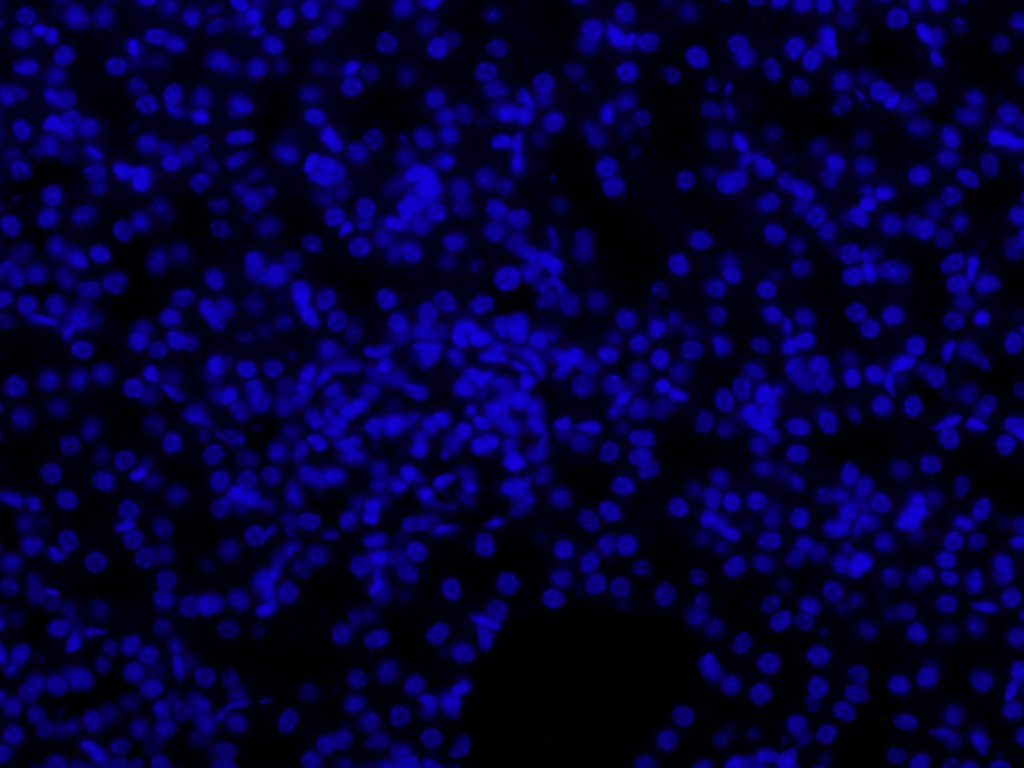

Supplement: Supplementary file 7 — Source data Fig. 6 [file 44321_2025_315_MOESM7_ESM.zip › Figure 6/F6A/2-C3/4-3 (3).jpg]

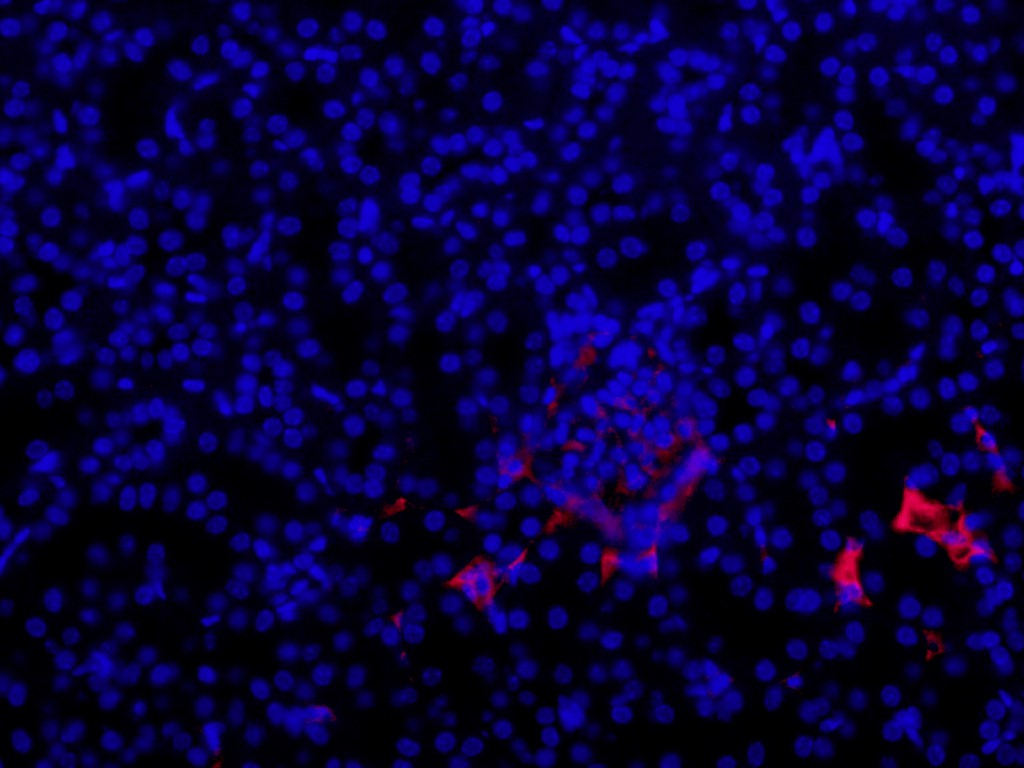

Supplement: Supplementary file 7 — Source data Fig. 6 [file 44321_2025_315_MOESM7_ESM.zip › Figure 6/F6A/2-C3/4-4 (1).jpg]

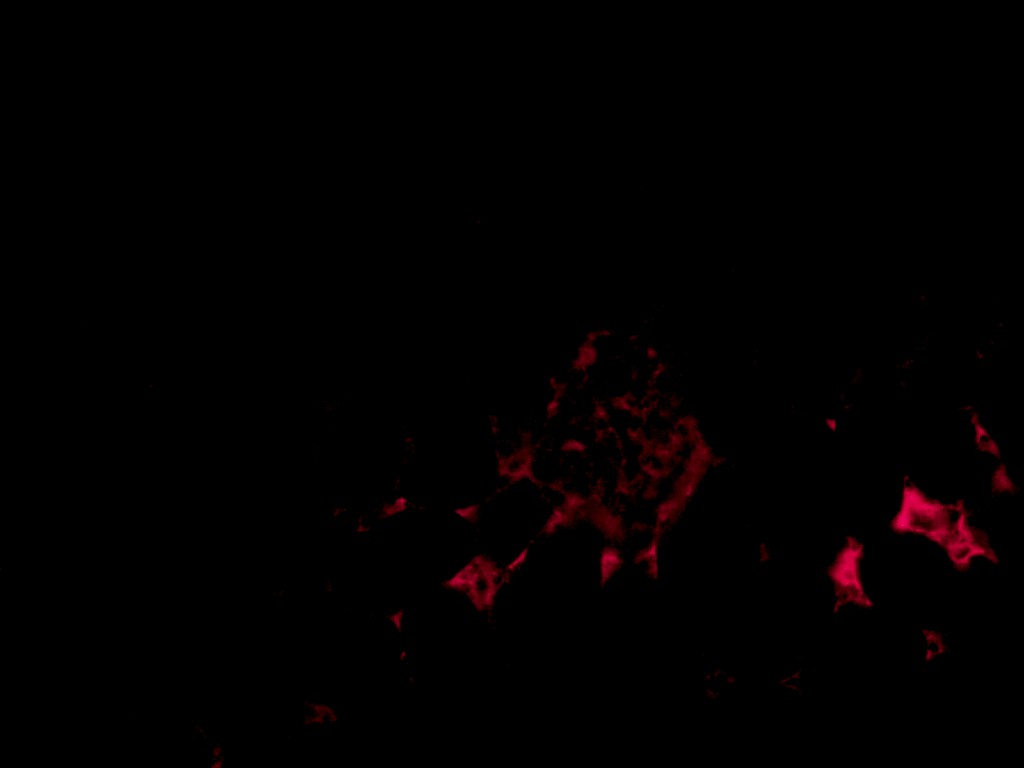

Supplement: Supplementary file 7 — Source data Fig. 6 [file 44321_2025_315_MOESM7_ESM.zip › Figure 6/F6A/2-C3/4-4 (2).jpg]

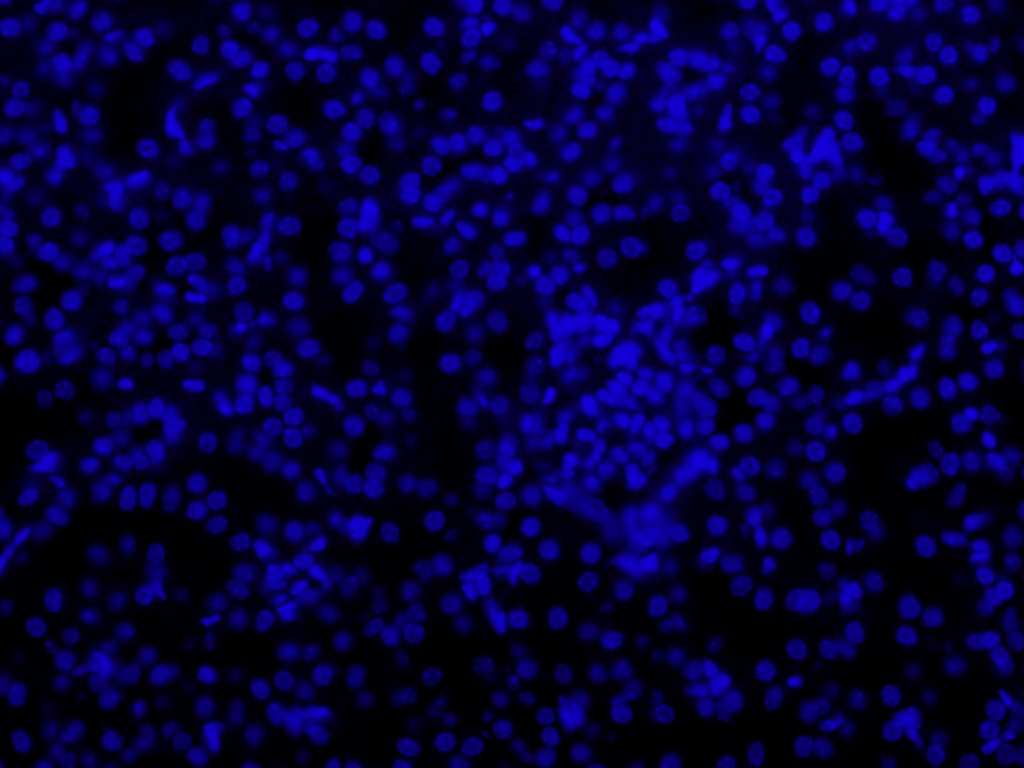

Supplement: Supplementary file 7 — Source data Fig. 6 [file 44321_2025_315_MOESM7_ESM.zip › Figure 6/F6A/2-C3/4-4 (3).jpg]

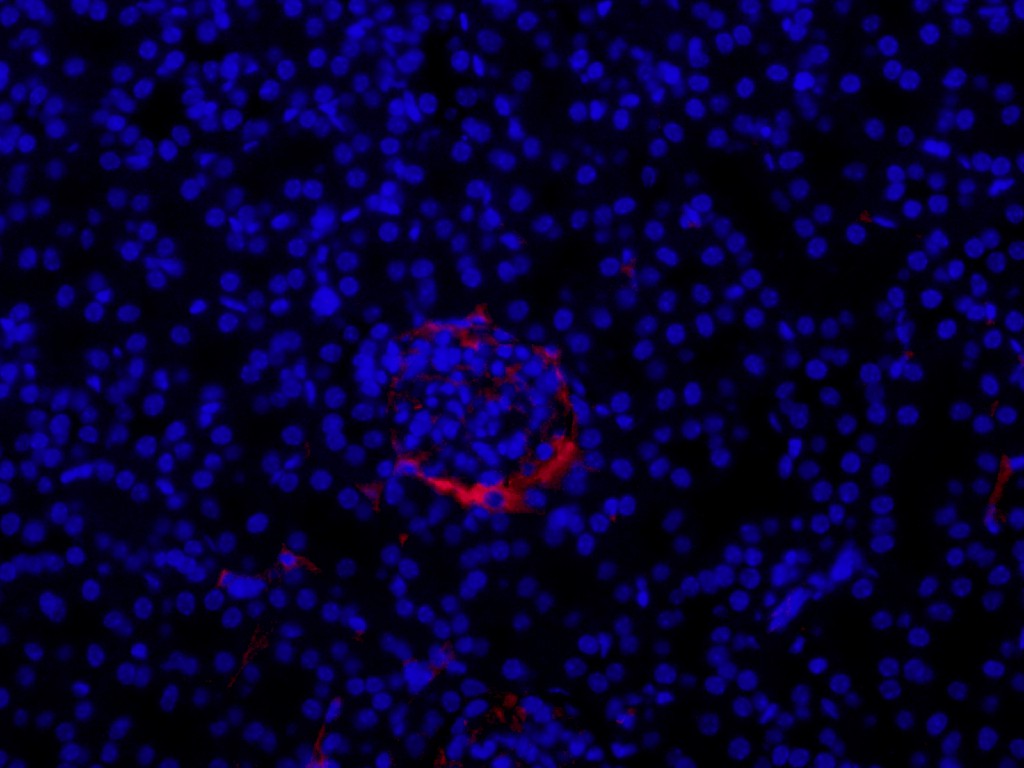

Supplement: Supplementary file 7 — Source data Fig. 6 [file 44321_2025_315_MOESM7_ESM.zip › Figure 6/F6A/2-C3/4-5 (1).jpg]
