# Supplementary figures and images for: Glycine decarboxylase advances IgA nephropathy by boosting mesangial cell proliferation through the pyrimidine pathway (part 7 of 7)
Source: EMBO Mol Med. 2025 Oct 13;17(11):3039–63. doi: 10.1038/s44321-025-00315-2 (PMC12603144; doi:10.1038/s44321-025-00315-2)

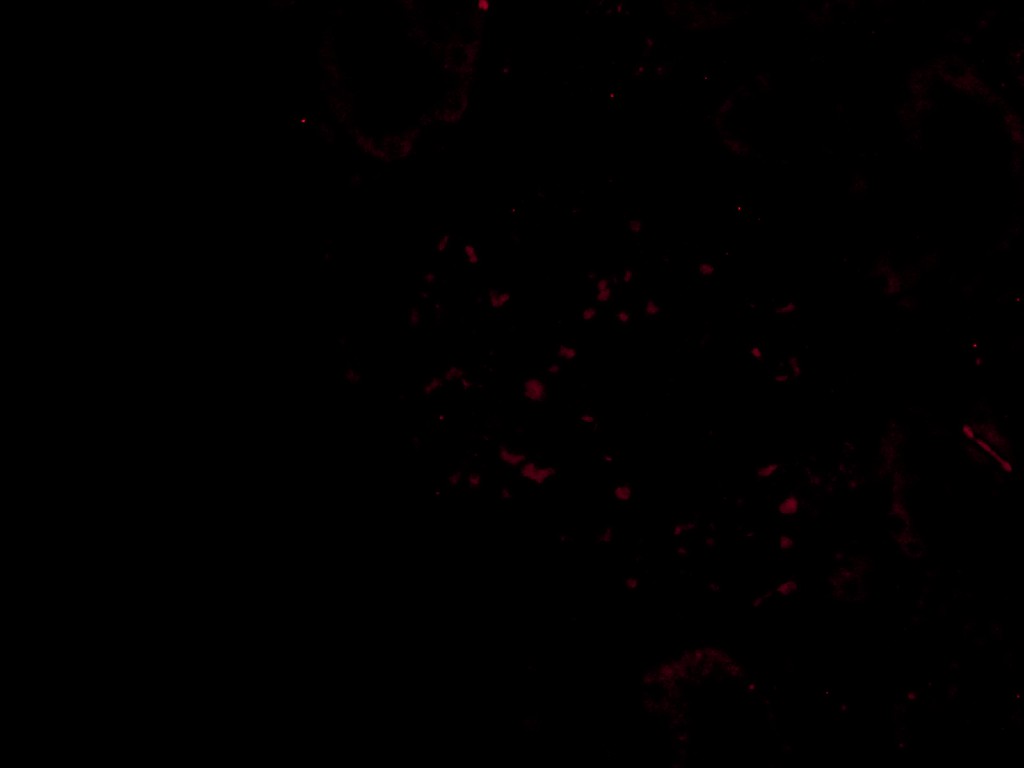

Supplement: Supplementary file 9 — Figure EV1 Source Data [file 44321_2025_315_MOESM9_ESM.zip › Figure EV1/EV1D/2-CD31-GLDC/LEE II/2 (2).jpg]

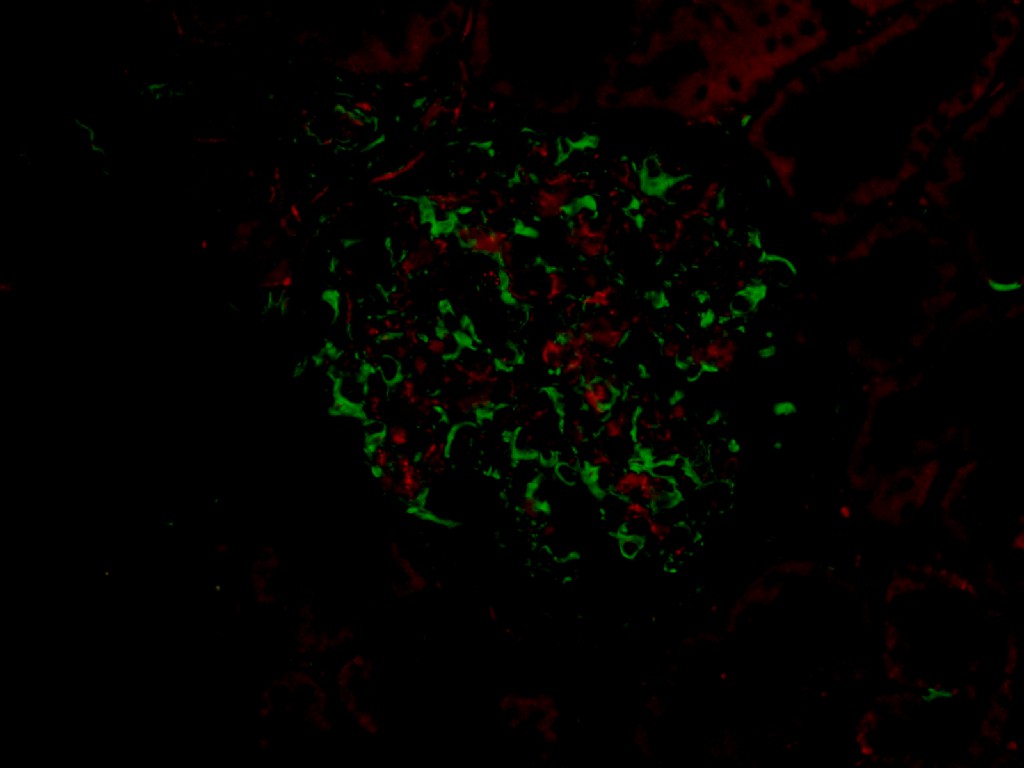

Supplement: Supplementary file 9 — Figure EV1 Source Data [file 44321_2025_315_MOESM9_ESM.zip › Figure EV1/EV1D/2-CD31-GLDC/LEE II/1 (4).jpg]

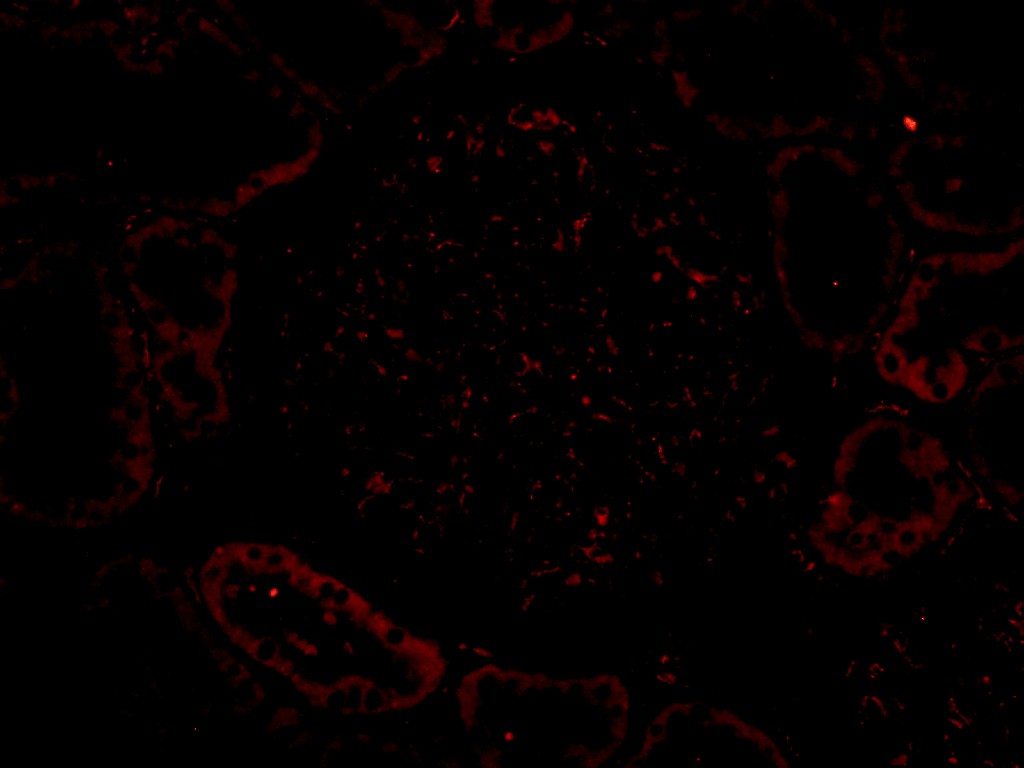

Supplement: Supplementary file 9 — Figure EV1 Source Data [file 44321_2025_315_MOESM9_ESM.zip › Figure EV1/EV1D/2-CD31-GLDC/LEE II/7 (1).jpg]

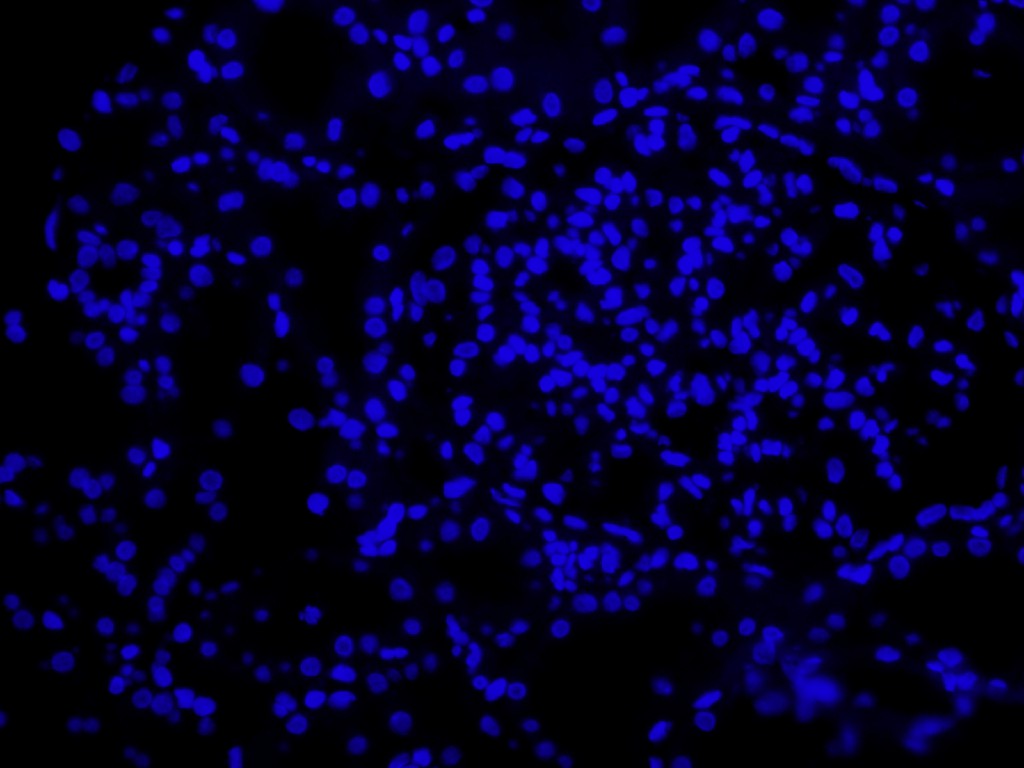

Supplement: Supplementary file 9 — Figure EV1 Source Data [file 44321_2025_315_MOESM9_ESM.zip › Figure EV1/EV1D/2-CD31-GLDC/LEE II/3 (3).jpg]

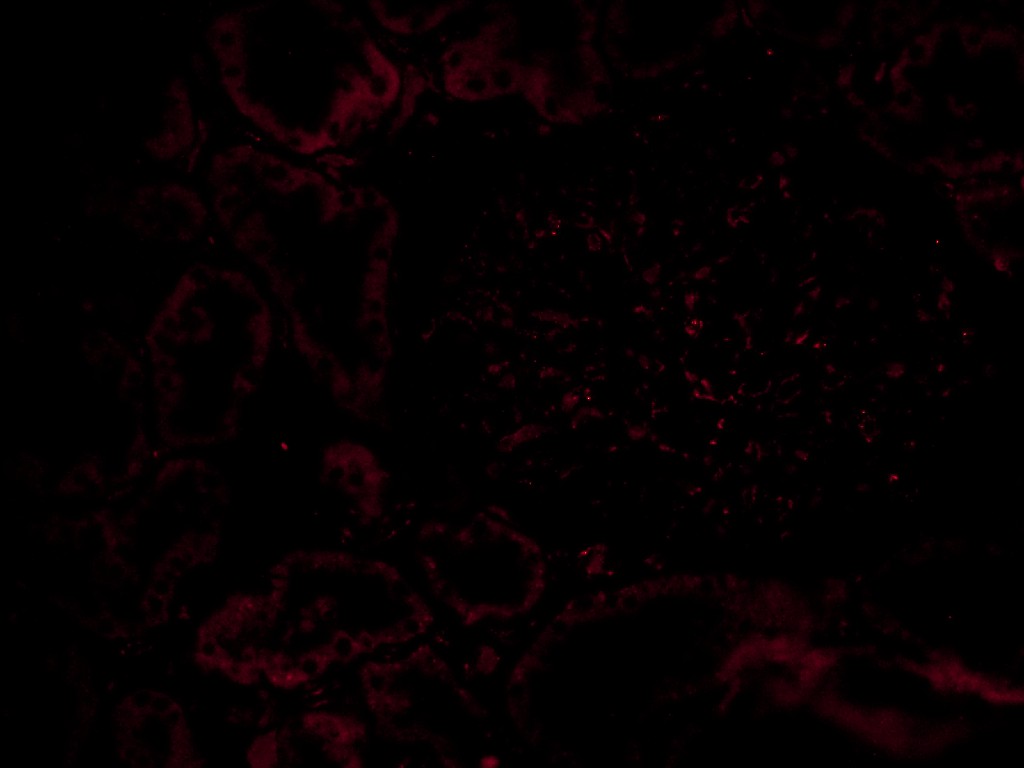

Supplement: Supplementary file 9 — Figure EV1 Source Data [file 44321_2025_315_MOESM9_ESM.zip › Figure EV1/EV1D/2-CD31-GLDC/LEE II/3 (2).jpg]

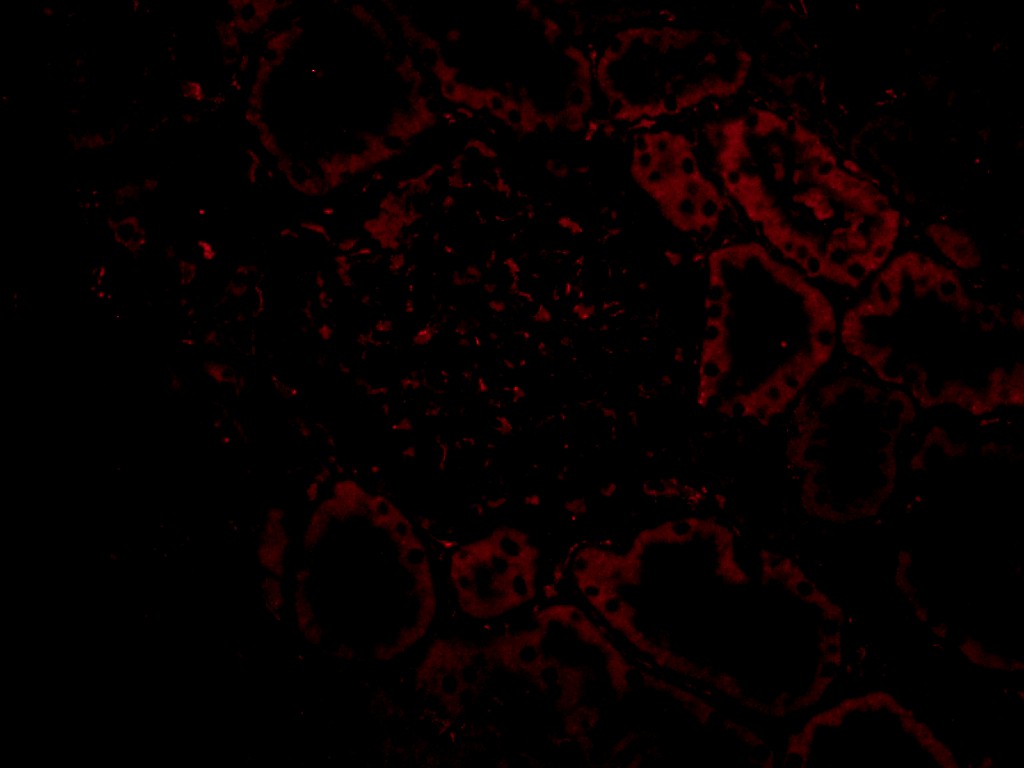

Supplement: Supplementary file 9 — Figure EV1 Source Data [file 44321_2025_315_MOESM9_ESM.zip › Figure EV1/EV1D/2-CD31-GLDC/LEE II/6 (1).jpg]

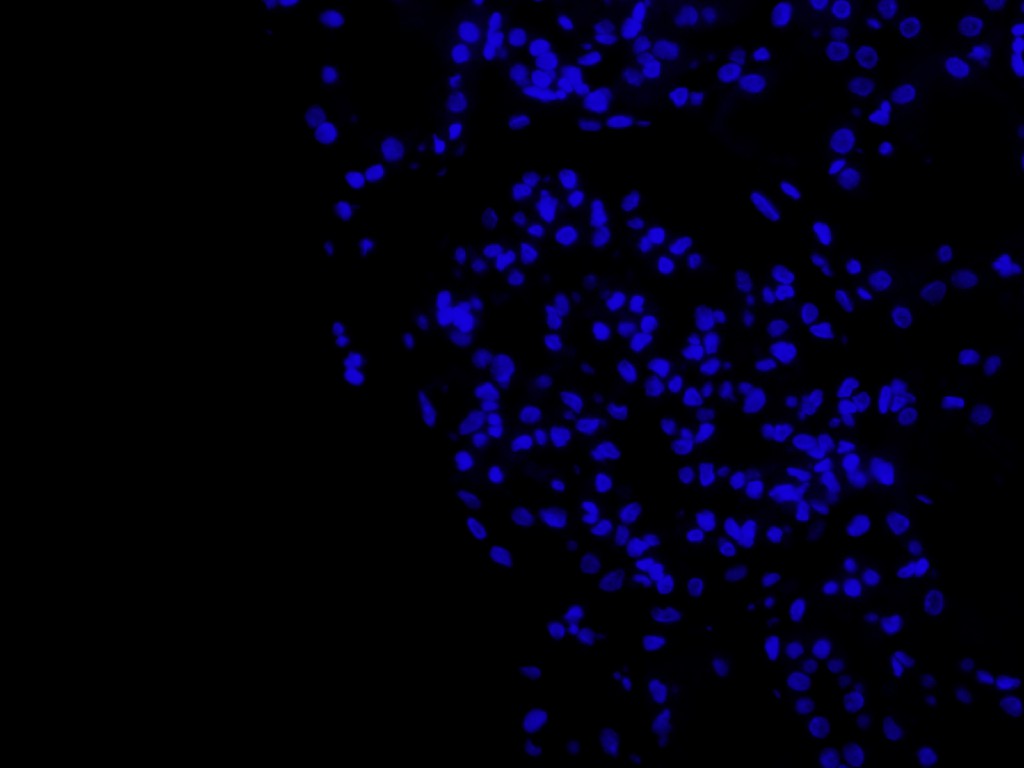

Supplement: Supplementary file 9 — Figure EV1 Source Data [file 44321_2025_315_MOESM9_ESM.zip › Figure EV1/EV1D/2-CD31-GLDC/LEE II/2 (3).jpg]

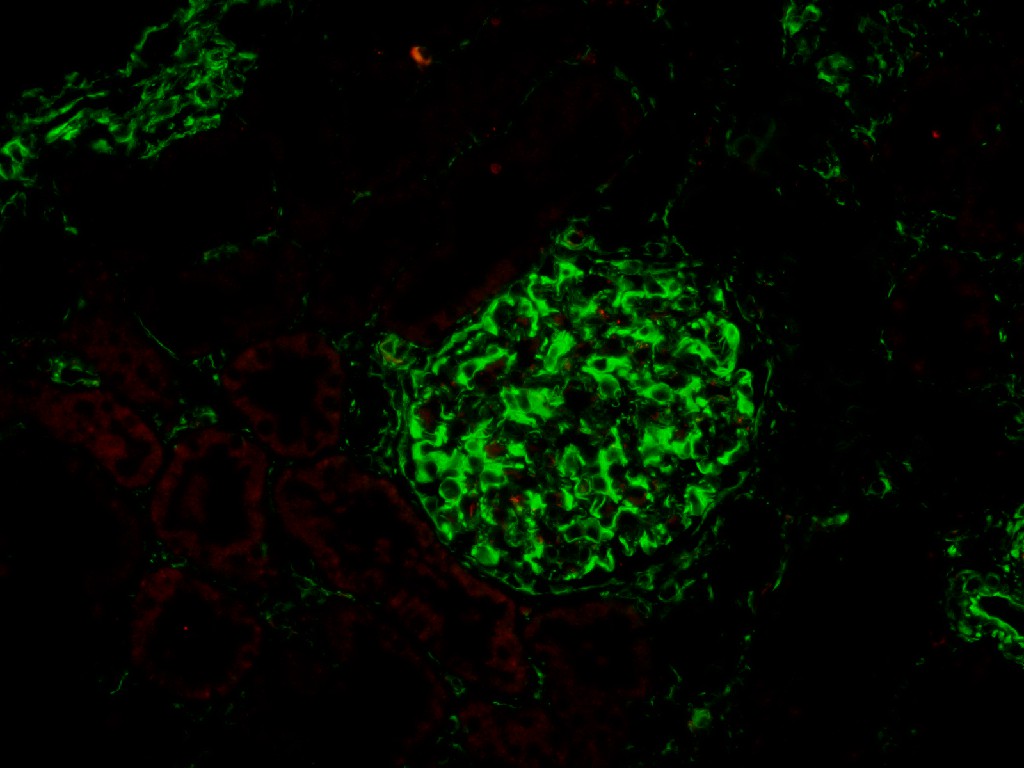

Supplement: Supplementary file 9 — Figure EV1 Source Data [file 44321_2025_315_MOESM9_ESM.zip › Figure EV1/EV1D/2-CD31-GLDC/LEE II/9 (4).jpg]

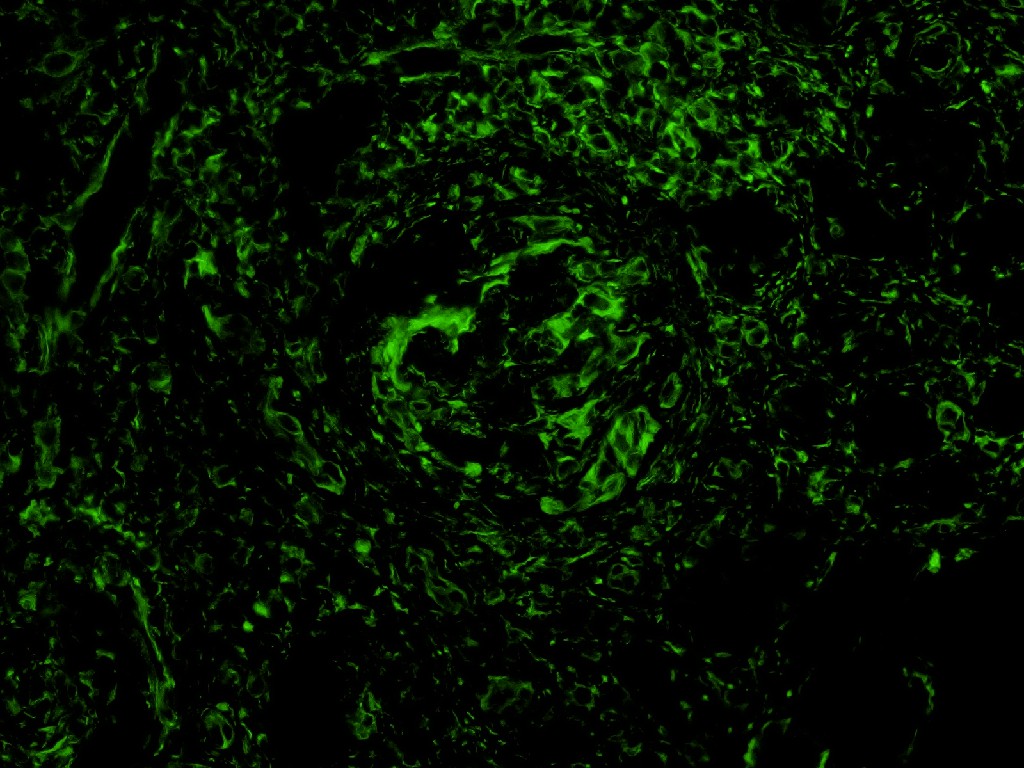

Supplement: Supplementary file 9 — Figure EV1 Source Data [file 44321_2025_315_MOESM9_ESM.zip › Figure EV1/EV1D/2-CD31-GLDC/LEE V/2 (1).jpg]

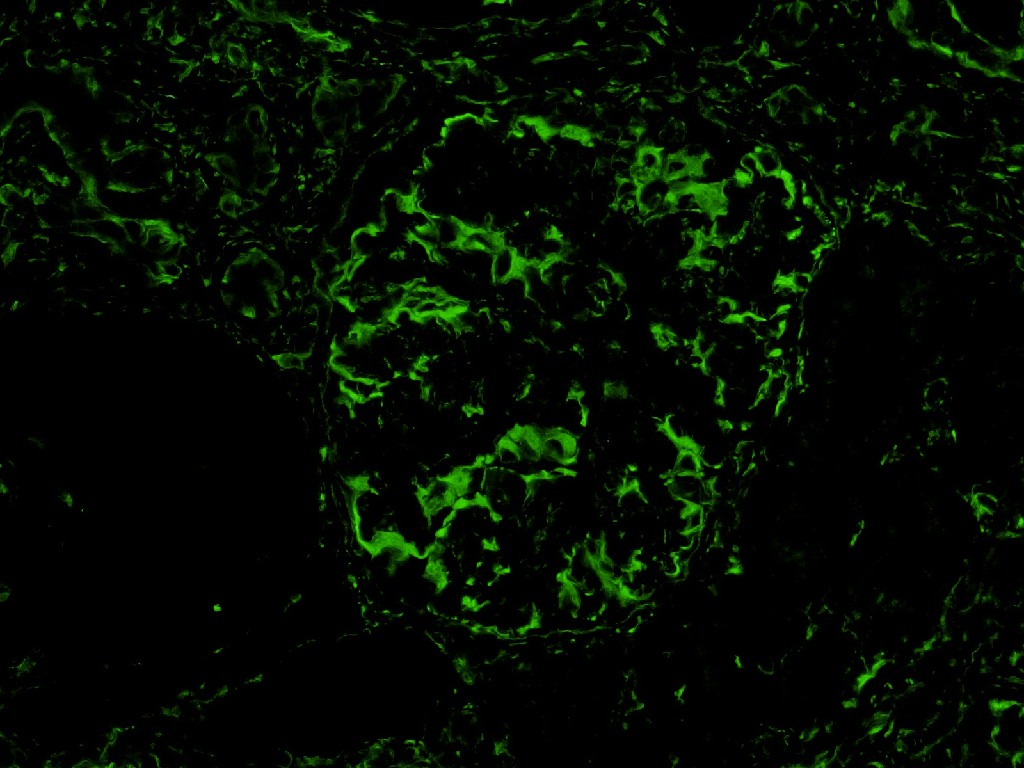

Supplement: Supplementary file 9 — Figure EV1 Source Data [file 44321_2025_315_MOESM9_ESM.zip › Figure EV1/EV1D/2-CD31-GLDC/LEE V/1 (1).jpg]

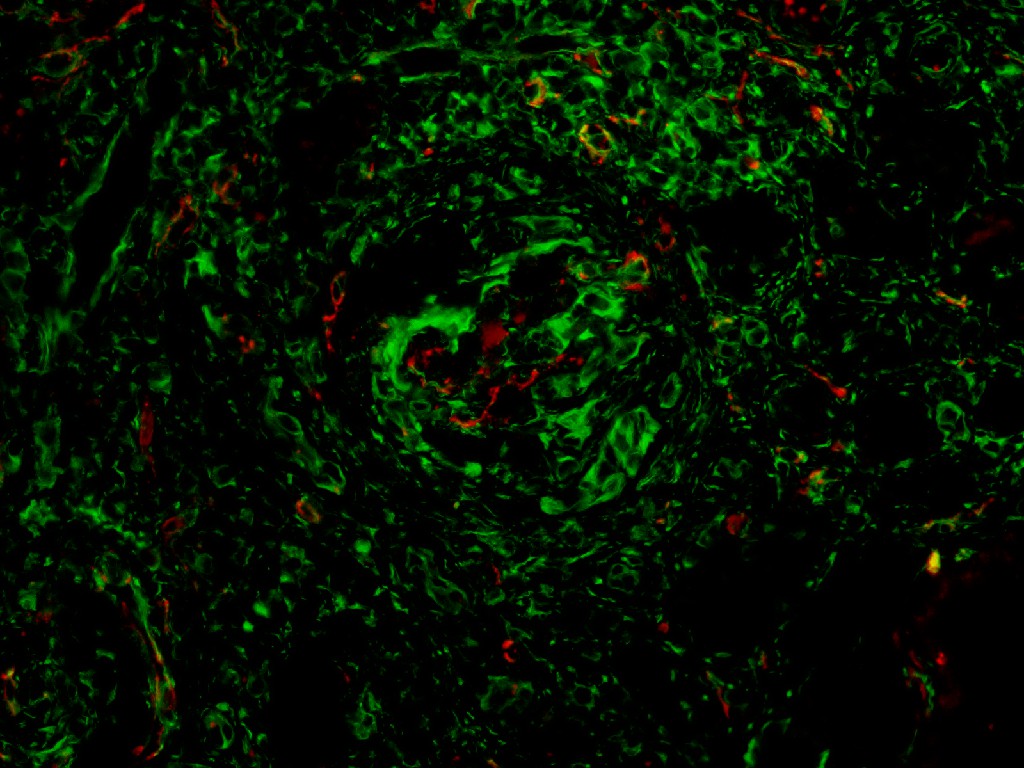

Supplement: Supplementary file 9 — Figure EV1 Source Data [file 44321_2025_315_MOESM9_ESM.zip › Figure EV1/EV1D/2-CD31-GLDC/LEE V/2 (4).jpg]

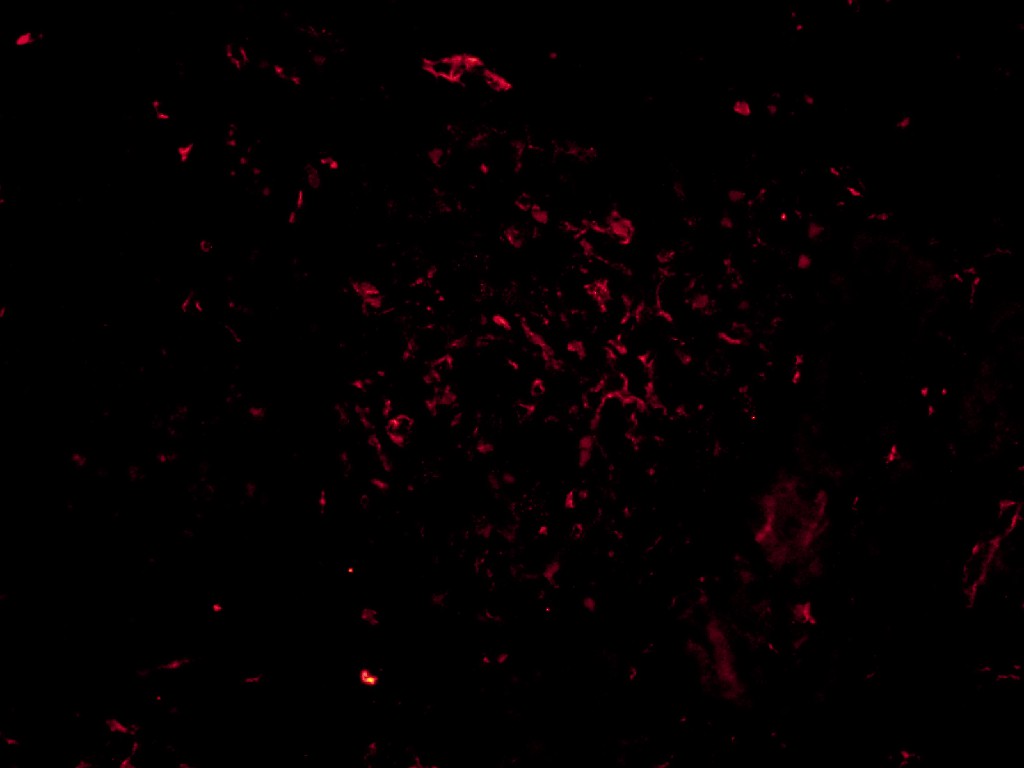

Supplement: Supplementary file 9 — Figure EV1 Source Data [file 44321_2025_315_MOESM9_ESM.zip › Figure EV1/EV1D/2-CD31-GLDC/LEE V/1 (2).jpg]

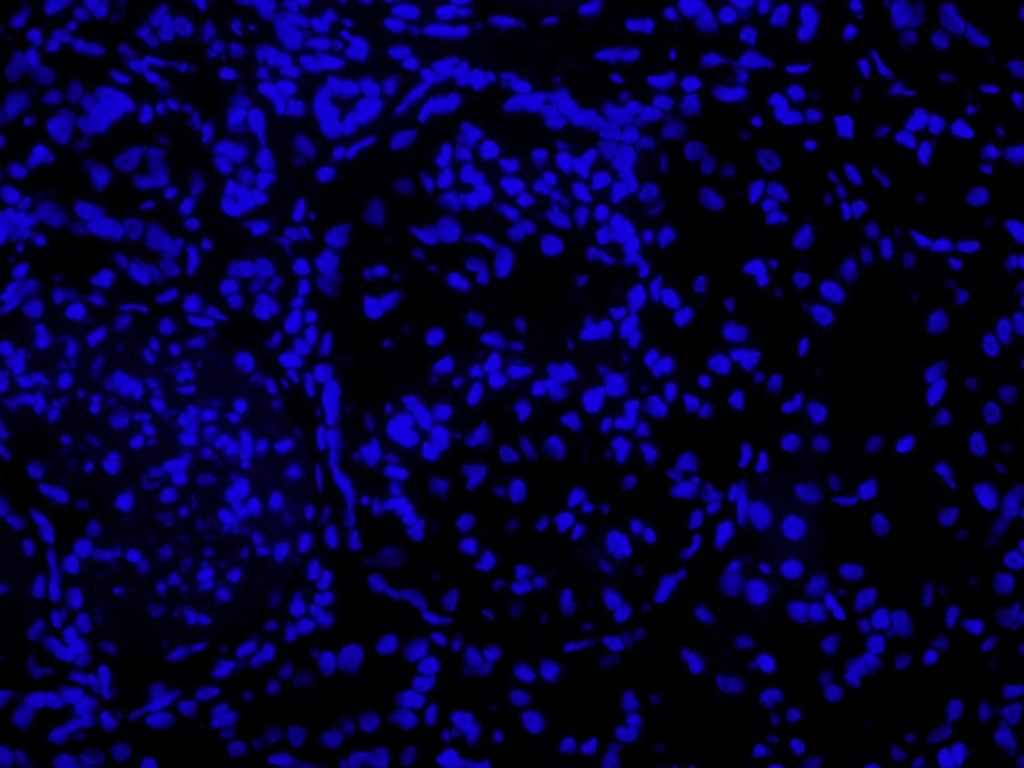

Supplement: Supplementary file 9 — Figure EV1 Source Data [file 44321_2025_315_MOESM9_ESM.zip › Figure EV1/EV1D/2-CD31-GLDC/LEE V/1 (3).jpg]

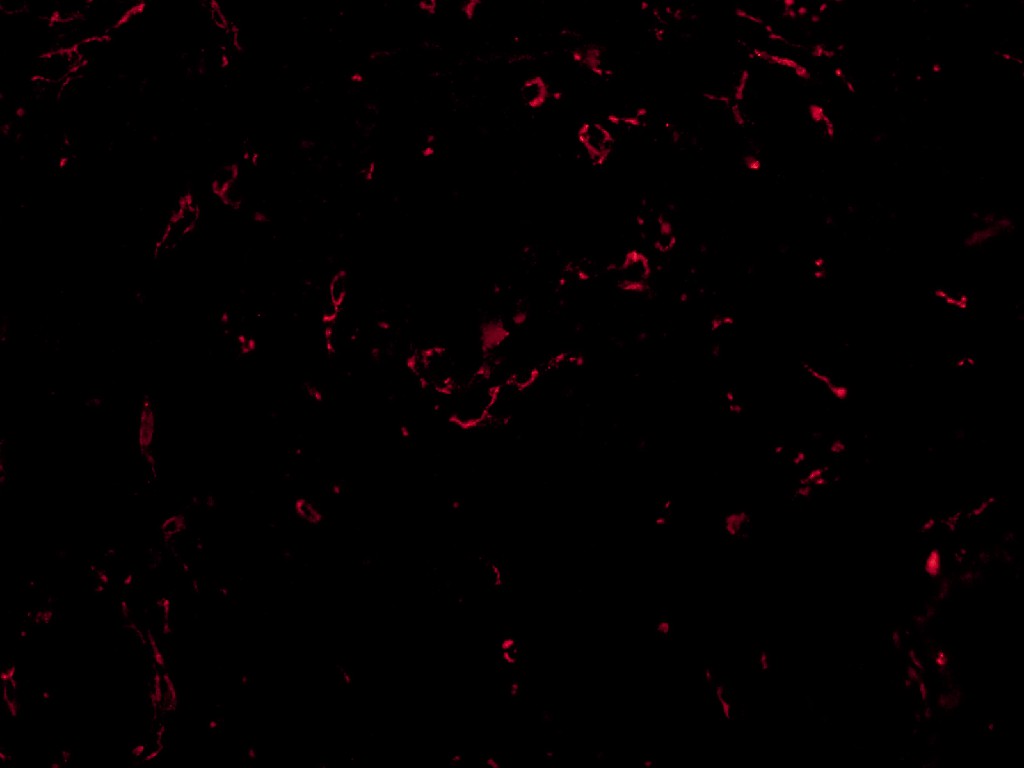

Supplement: Supplementary file 9 — Figure EV1 Source Data [file 44321_2025_315_MOESM9_ESM.zip › Figure EV1/EV1D/2-CD31-GLDC/LEE V/2 (2).jpg]

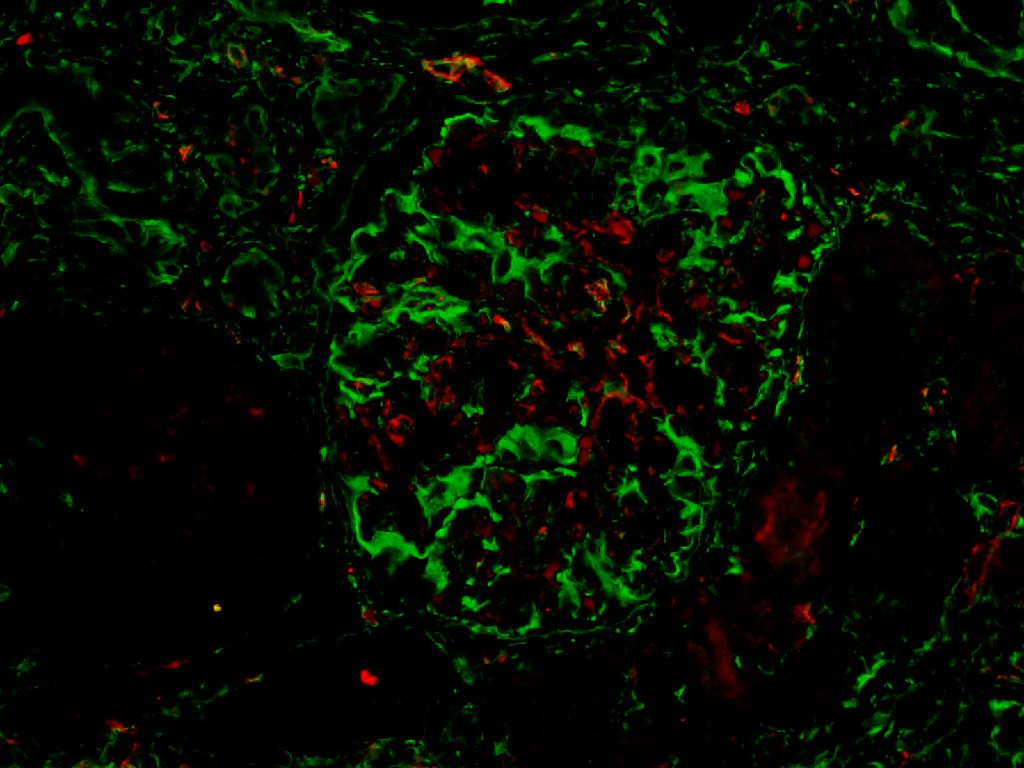

Supplement: Supplementary file 9 — Figure EV1 Source Data [file 44321_2025_315_MOESM9_ESM.zip › Figure EV1/EV1D/2-CD31-GLDC/LEE V/1 (4).jpg]

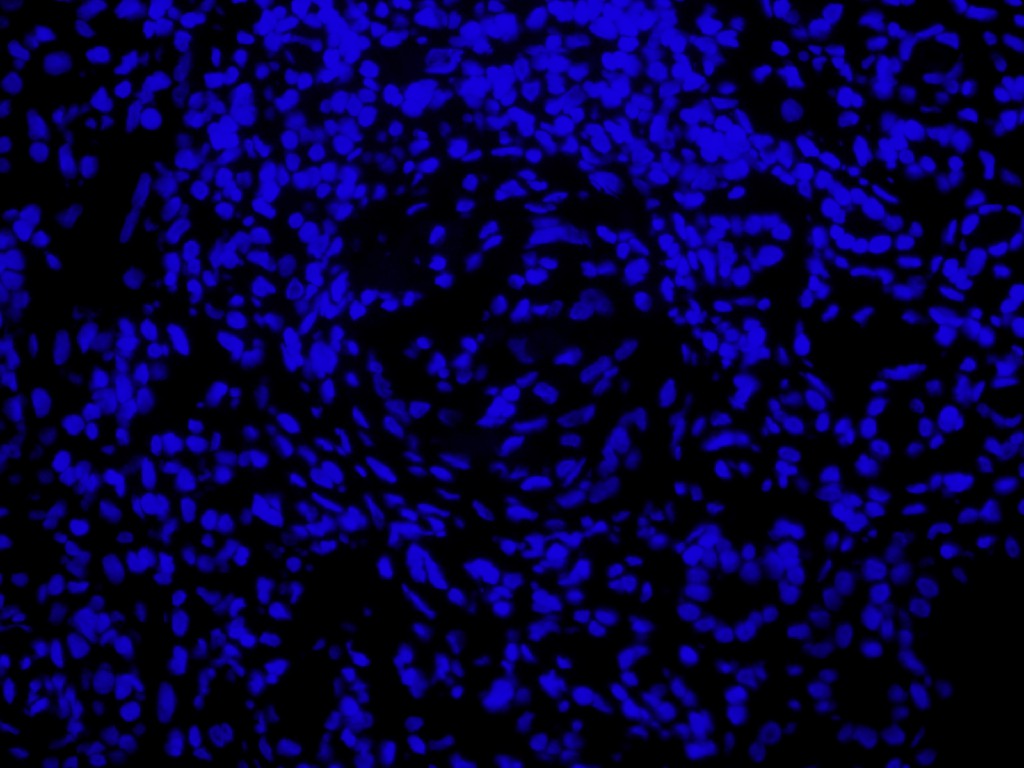

Supplement: Supplementary file 9 — Figure EV1 Source Data [file 44321_2025_315_MOESM9_ESM.zip › Figure EV1/EV1D/2-CD31-GLDC/LEE V/2 (3).jpg]

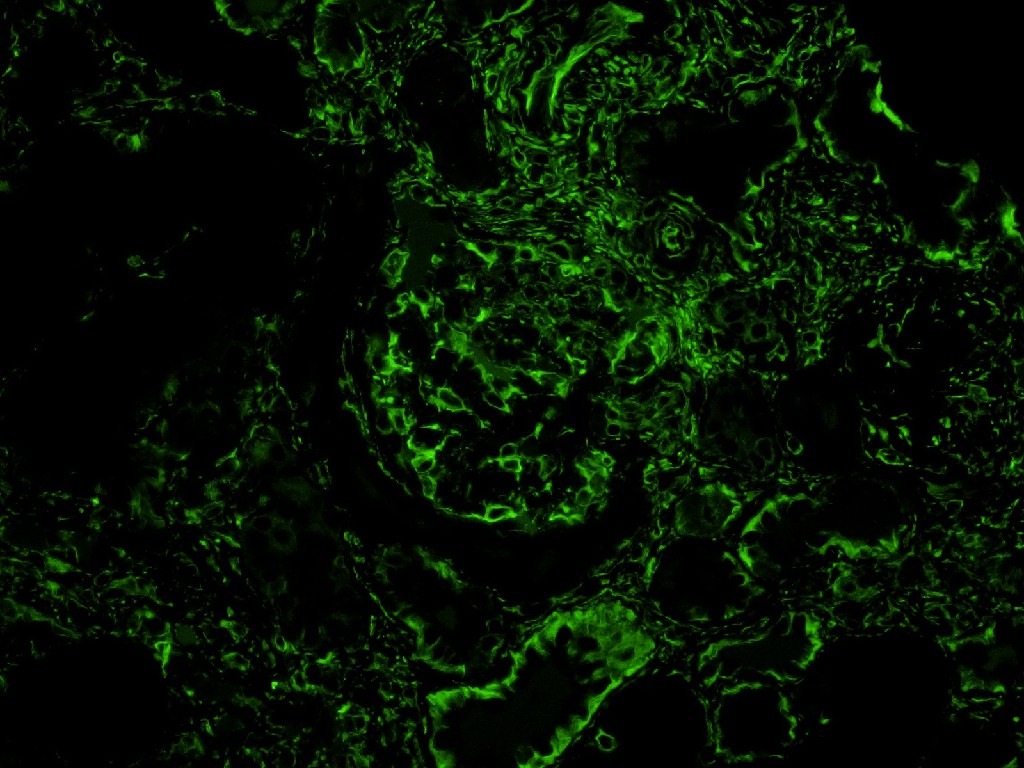

Supplement: Supplementary file 9 — Figure EV1 Source Data [file 44321_2025_315_MOESM9_ESM.zip › Figure EV1/EV1D/2-CD31-GLDC/LEE IV/3 (1).jpg]

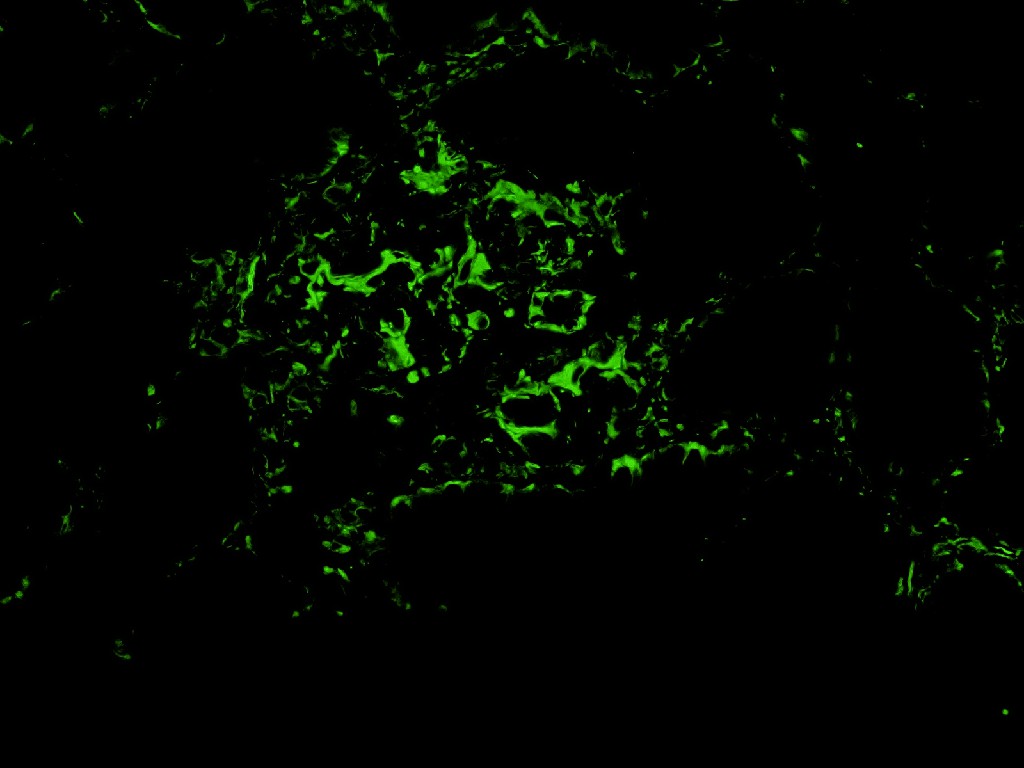

Supplement: Supplementary file 9 — Figure EV1 Source Data [file 44321_2025_315_MOESM9_ESM.zip › Figure EV1/EV1D/2-CD31-GLDC/LEE IV/2 (1).jpg]

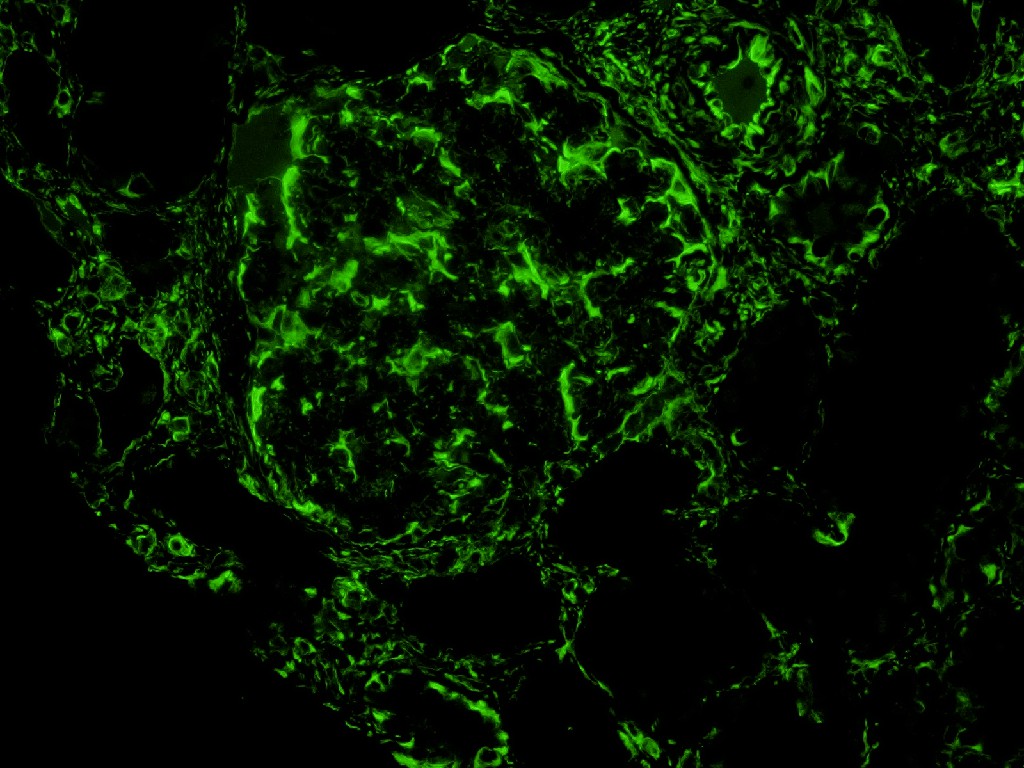

Supplement: Supplementary file 9 — Figure EV1 Source Data [file 44321_2025_315_MOESM9_ESM.zip › Figure EV1/EV1D/2-CD31-GLDC/LEE IV/1 (1).jpg]

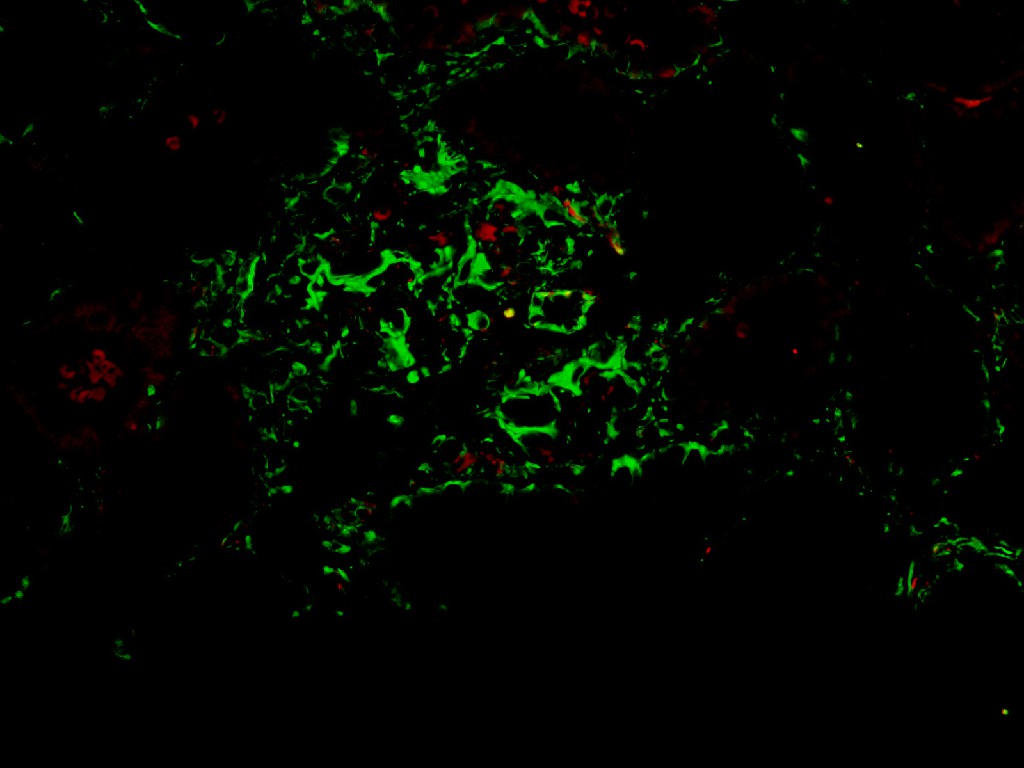

Supplement: Supplementary file 9 — Figure EV1 Source Data [file 44321_2025_315_MOESM9_ESM.zip › Figure EV1/EV1D/2-CD31-GLDC/LEE IV/2 (4).jpg]

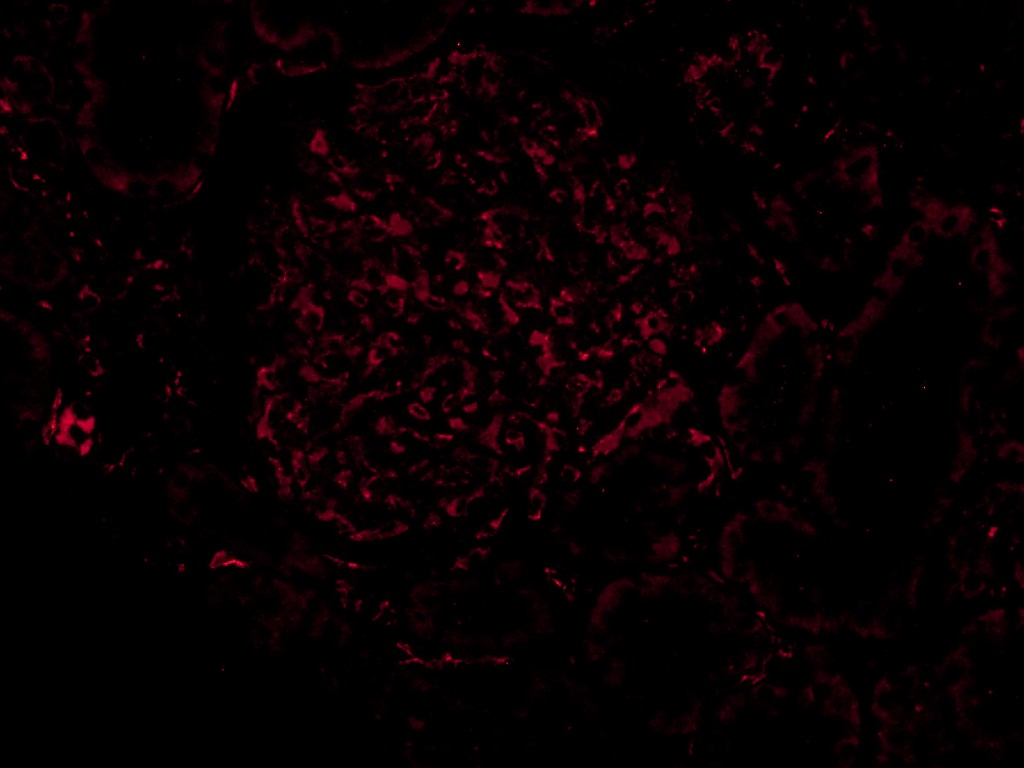

Supplement: Supplementary file 9 — Figure EV1 Source Data [file 44321_2025_315_MOESM9_ESM.zip › Figure EV1/EV1D/2-CD31-GLDC/LEE IV/1 (2).jpg]

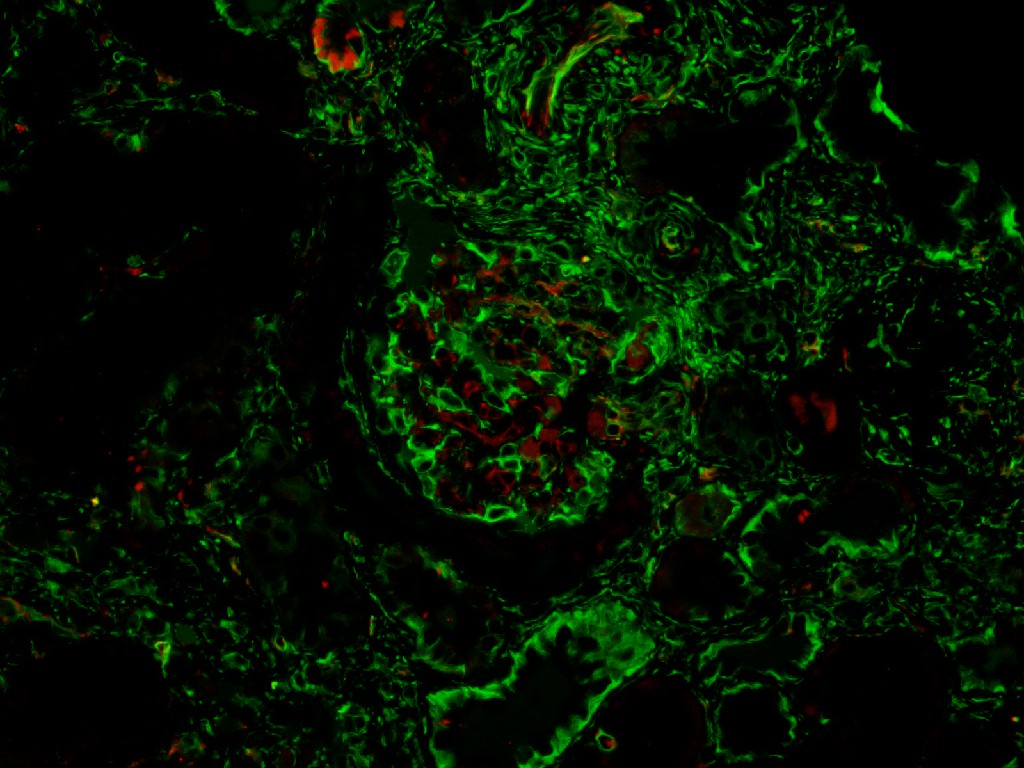

Supplement: Supplementary file 9 — Figure EV1 Source Data [file 44321_2025_315_MOESM9_ESM.zip › Figure EV1/EV1D/2-CD31-GLDC/LEE IV/3 (4).jpg]

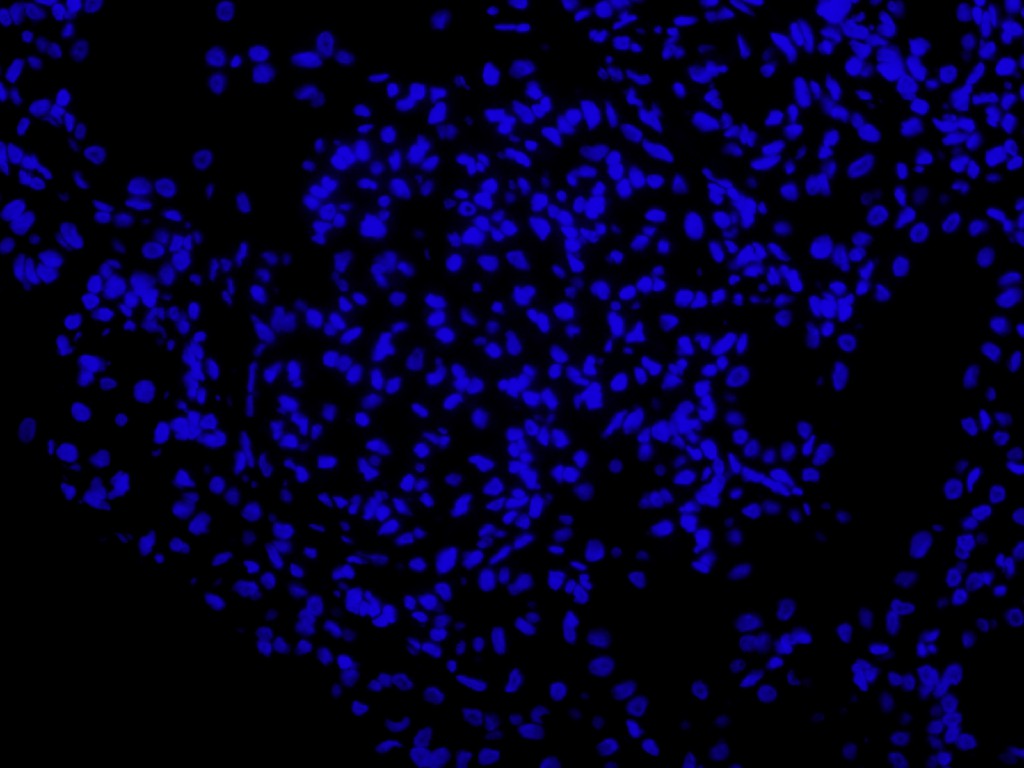

Supplement: Supplementary file 9 — Figure EV1 Source Data [file 44321_2025_315_MOESM9_ESM.zip › Figure EV1/EV1D/2-CD31-GLDC/LEE IV/1 (3).jpg]

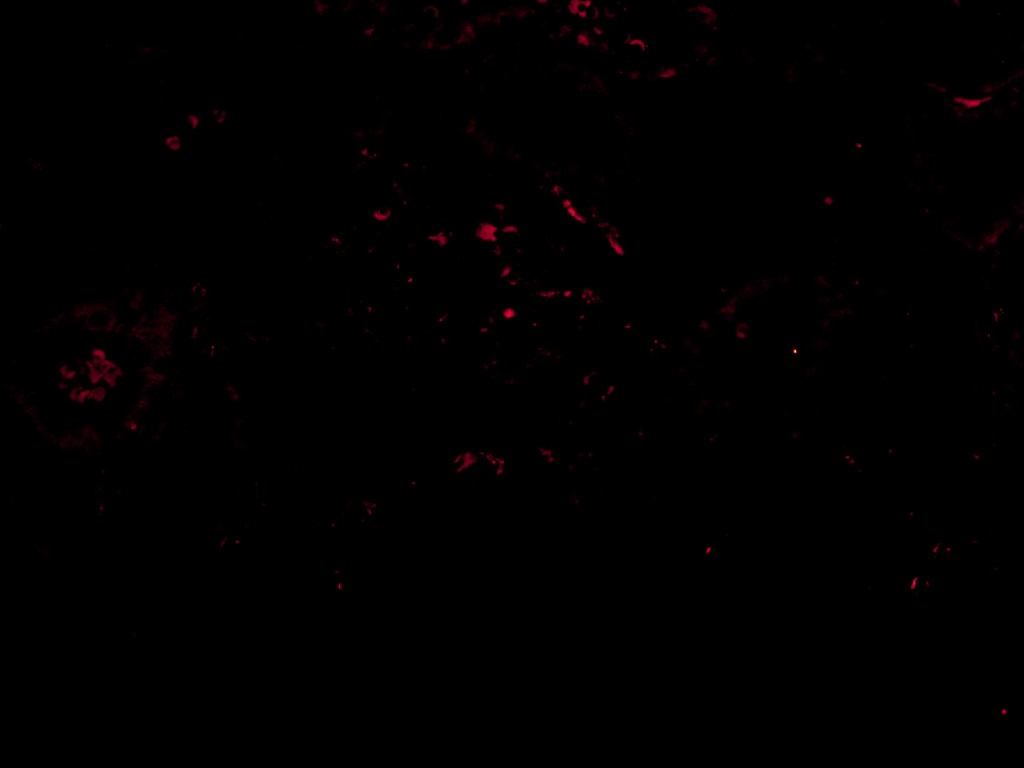

Supplement: Supplementary file 9 — Figure EV1 Source Data [file 44321_2025_315_MOESM9_ESM.zip › Figure EV1/EV1D/2-CD31-GLDC/LEE IV/2 (2).jpg]

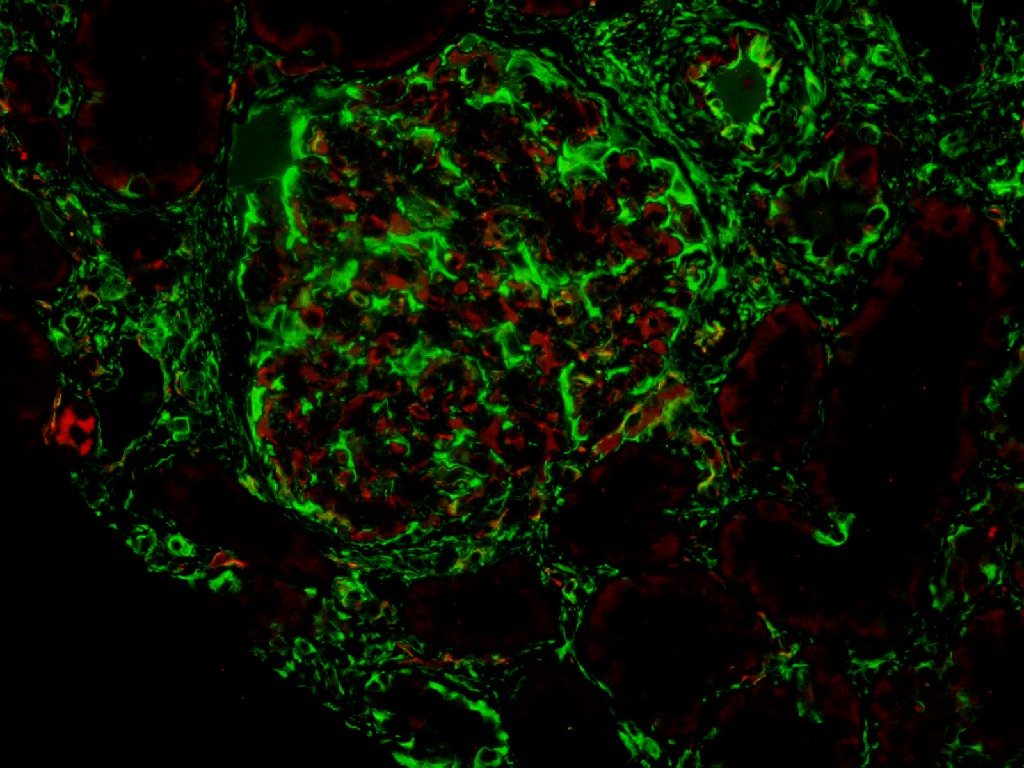

Supplement: Supplementary file 9 — Figure EV1 Source Data [file 44321_2025_315_MOESM9_ESM.zip › Figure EV1/EV1D/2-CD31-GLDC/LEE IV/1 (4).jpg]

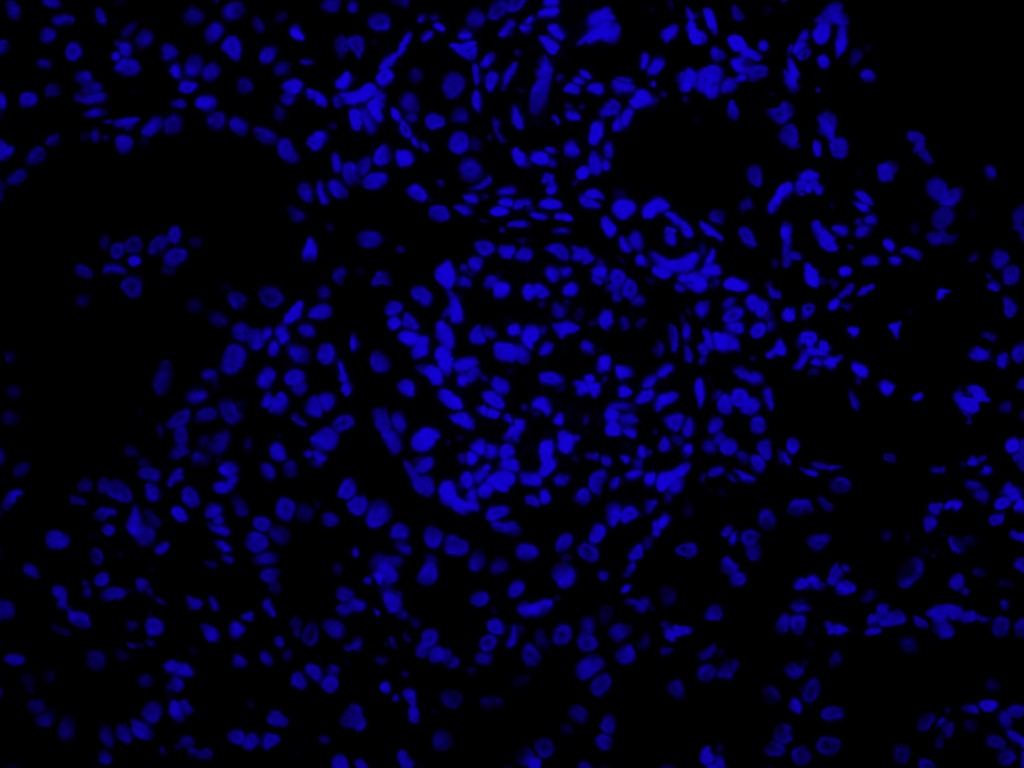

Supplement: Supplementary file 9 — Figure EV1 Source Data [file 44321_2025_315_MOESM9_ESM.zip › Figure EV1/EV1D/2-CD31-GLDC/LEE IV/3 (3).jpg]

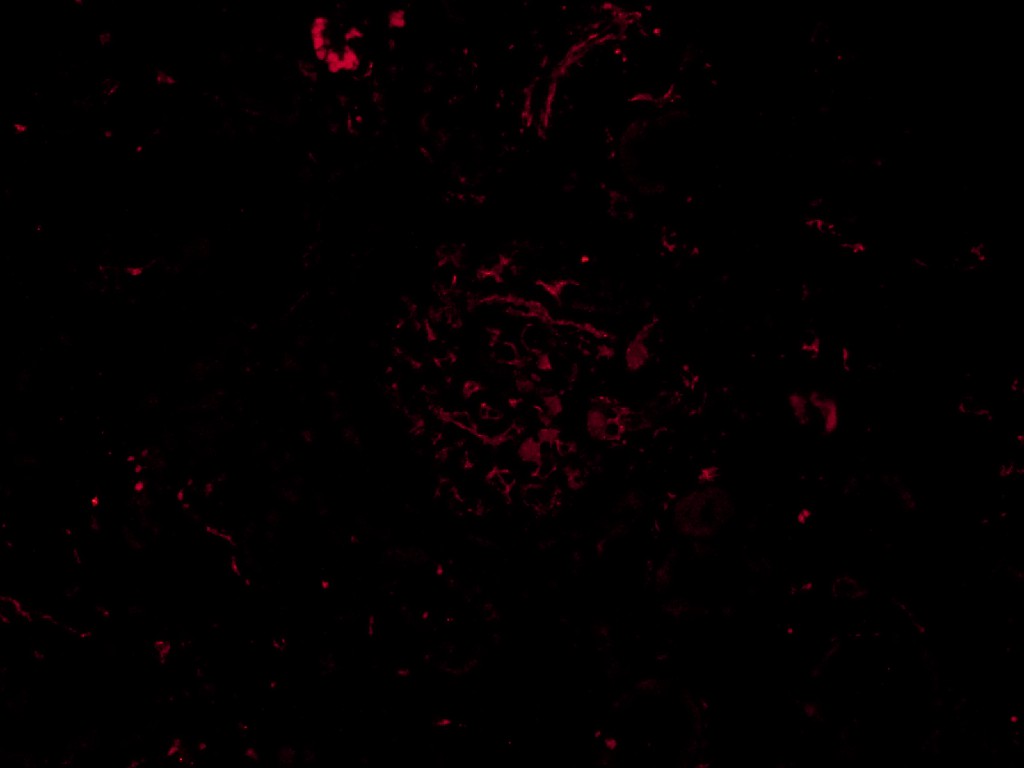

Supplement: Supplementary file 9 — Figure EV1 Source Data [file 44321_2025_315_MOESM9_ESM.zip › Figure EV1/EV1D/2-CD31-GLDC/LEE IV/3 (2).jpg]

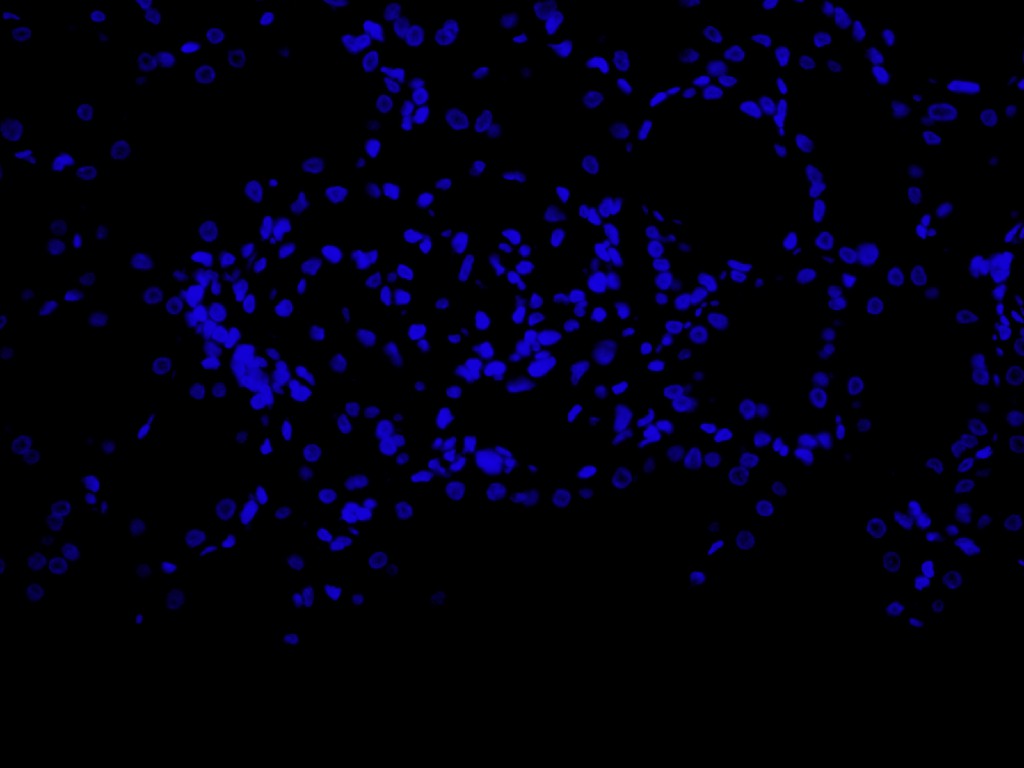

Supplement: Supplementary file 9 — Figure EV1 Source Data [file 44321_2025_315_MOESM9_ESM.zip › Figure EV1/EV1D/2-CD31-GLDC/LEE IV/2 (3).jpg]

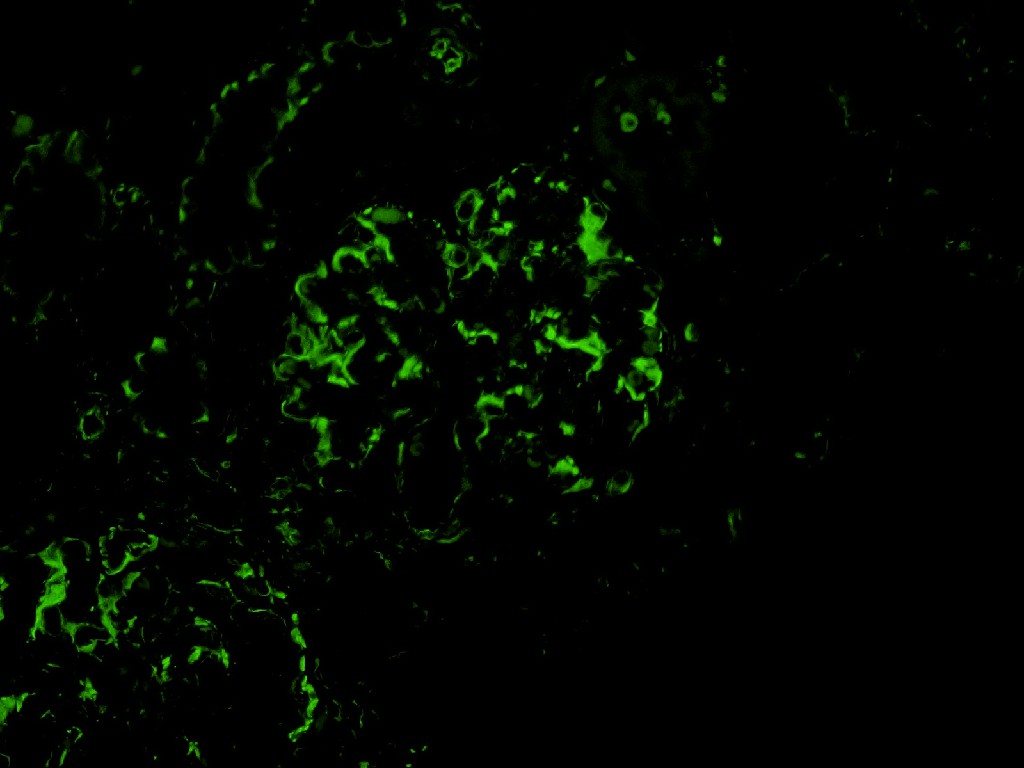

Supplement: Supplementary file 9 — Figure EV1 Source Data [file 44321_2025_315_MOESM9_ESM.zip › Figure EV1/EV1D/1-Podocin-GLDC/LEE III/3 (1).jpg]

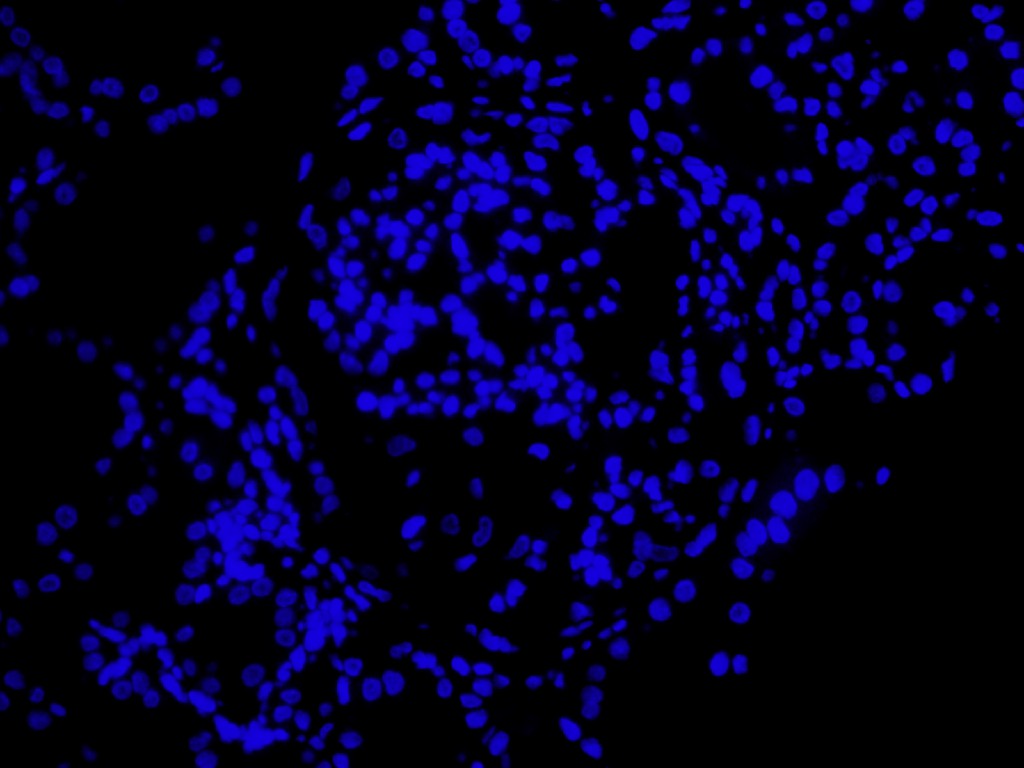

Supplement: Supplementary file 9 — Figure EV1 Source Data [file 44321_2025_315_MOESM9_ESM.zip › Figure EV1/EV1D/1-Podocin-GLDC/LEE III/7 (3).jpg]

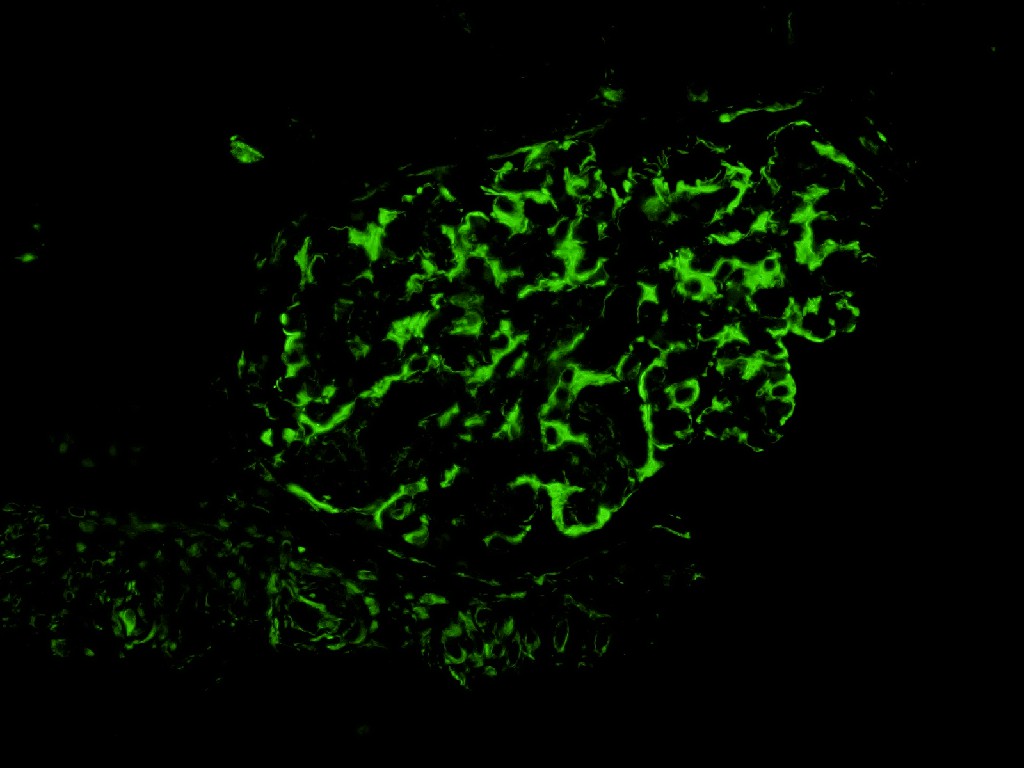

Supplement: Supplementary file 9 — Figure EV1 Source Data [file 44321_2025_315_MOESM9_ESM.zip › Figure EV1/EV1D/1-Podocin-GLDC/LEE III/10 (1).jpg]

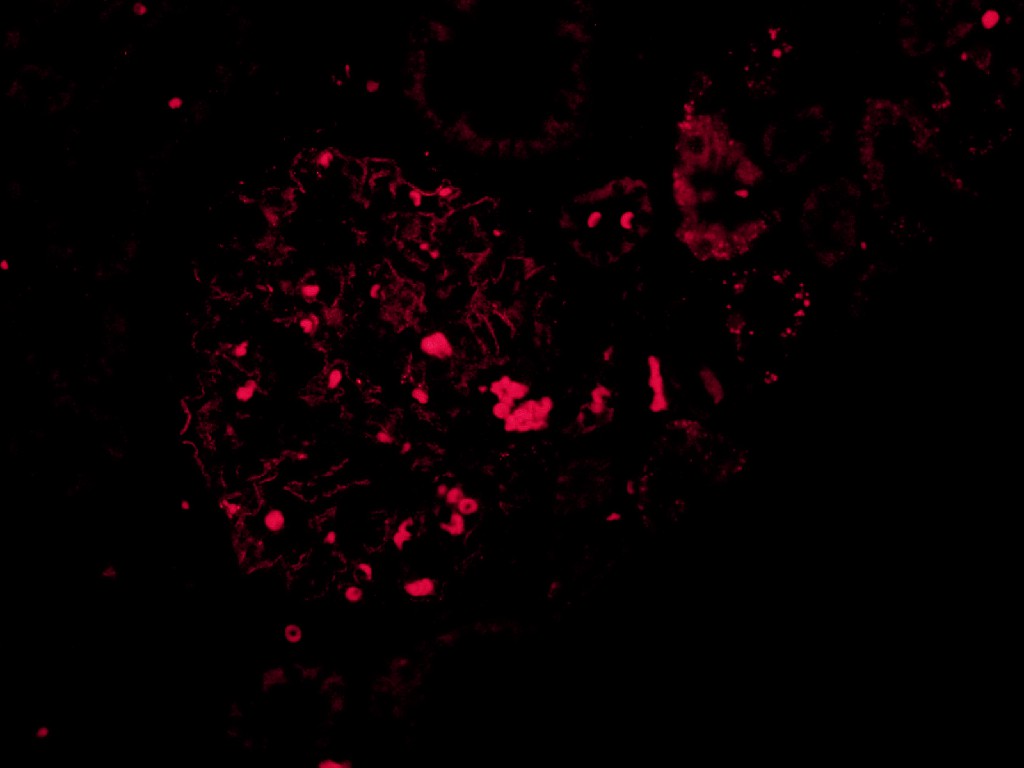

Supplement: Supplementary file 9 — Figure EV1 Source Data [file 44321_2025_315_MOESM9_ESM.zip › Figure EV1/EV1D/1-Podocin-GLDC/LEE III/6 (2).jpg]

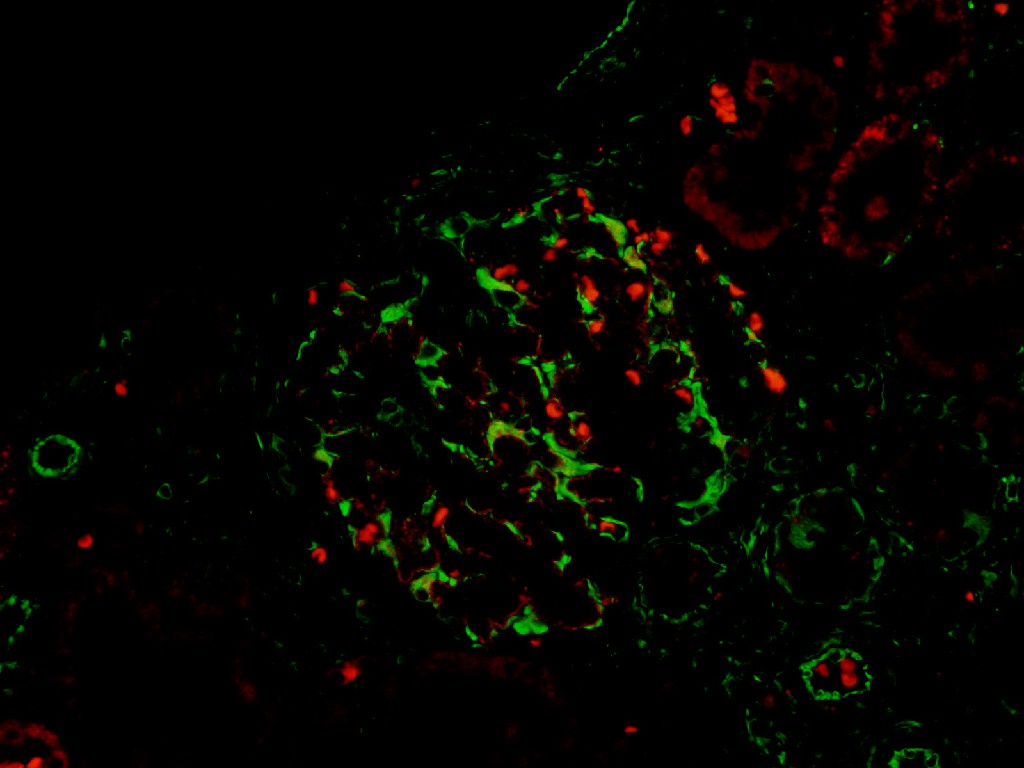

Supplement: Supplementary file 9 — Figure EV1 Source Data [file 44321_2025_315_MOESM9_ESM.zip › Figure EV1/EV1D/1-Podocin-GLDC/LEE III/5 (4).jpg]

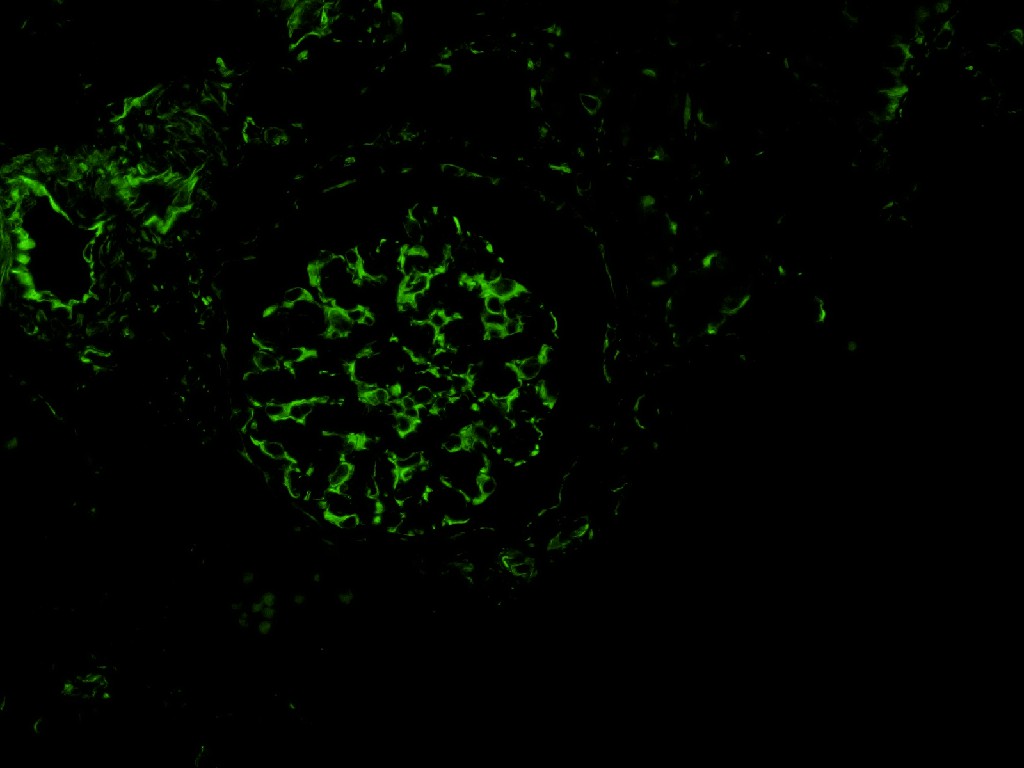

Supplement: Supplementary file 9 — Figure EV1 Source Data [file 44321_2025_315_MOESM9_ESM.zip › Figure EV1/EV1D/1-Podocin-GLDC/LEE III/2 (1).jpg]

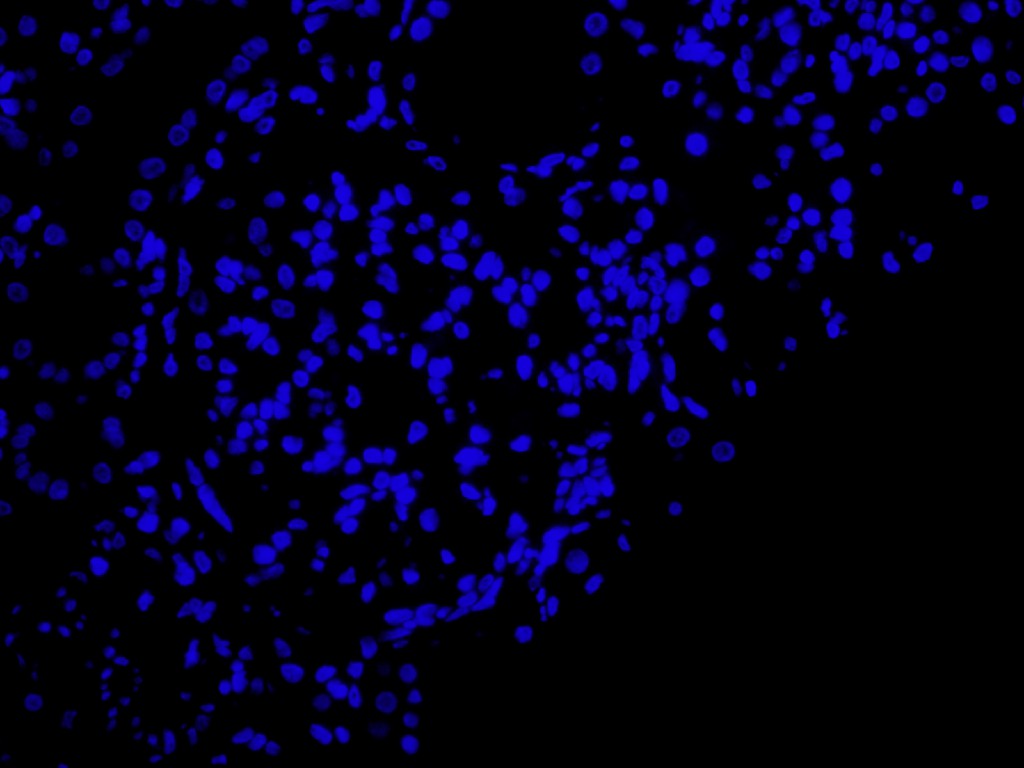

Supplement: Supplementary file 9 — Figure EV1 Source Data [file 44321_2025_315_MOESM9_ESM.zip › Figure EV1/EV1D/1-Podocin-GLDC/LEE III/6 (3).jpg]

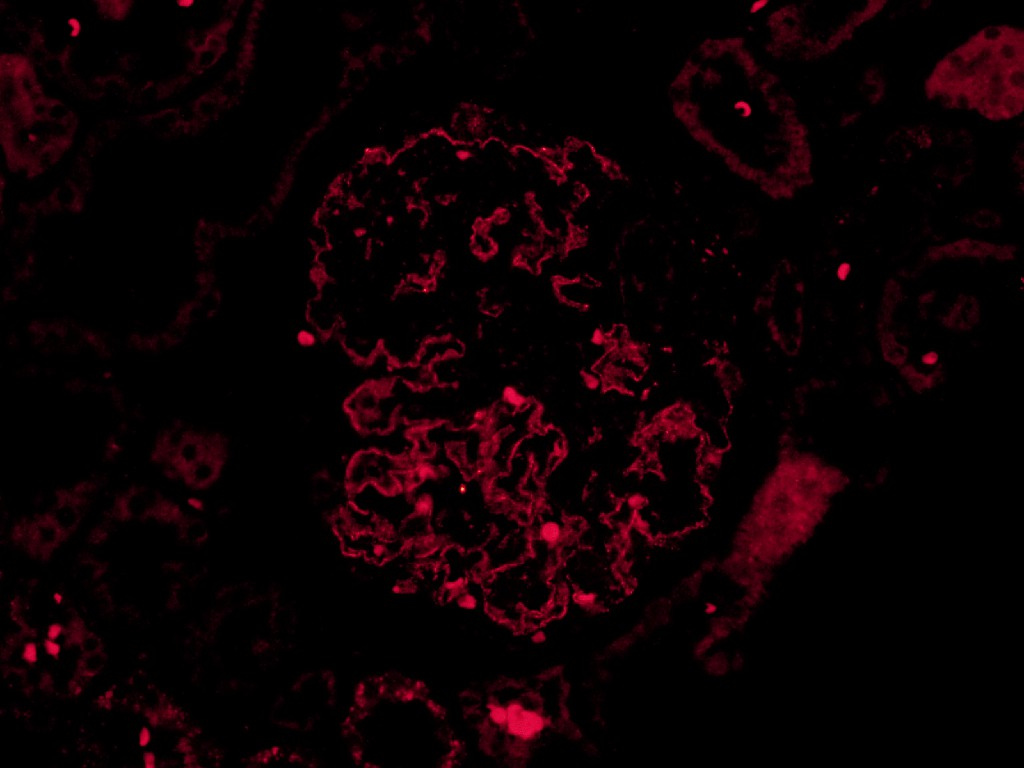

Supplement: Supplementary file 9 — Figure EV1 Source Data [file 44321_2025_315_MOESM9_ESM.zip › Figure EV1/EV1D/1-Podocin-GLDC/LEE III/7 (2).jpg]

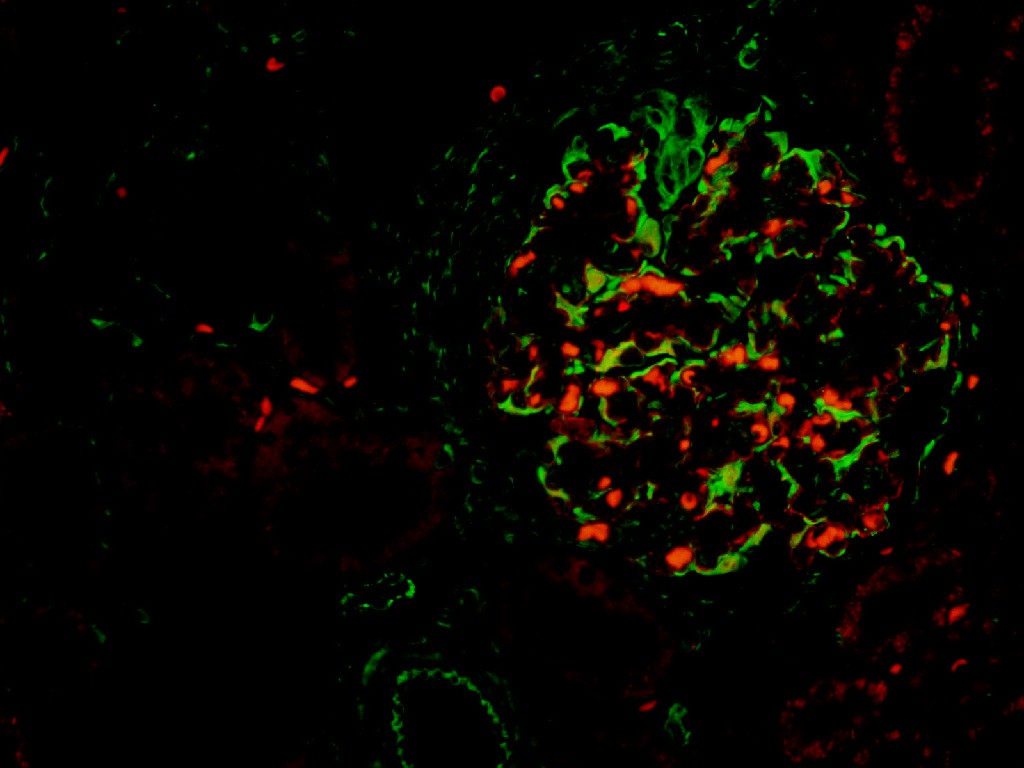

Supplement: Supplementary file 9 — Figure EV1 Source Data [file 44321_2025_315_MOESM9_ESM.zip › Figure EV1/EV1D/1-Podocin-GLDC/LEE III/4 (4).jpg]

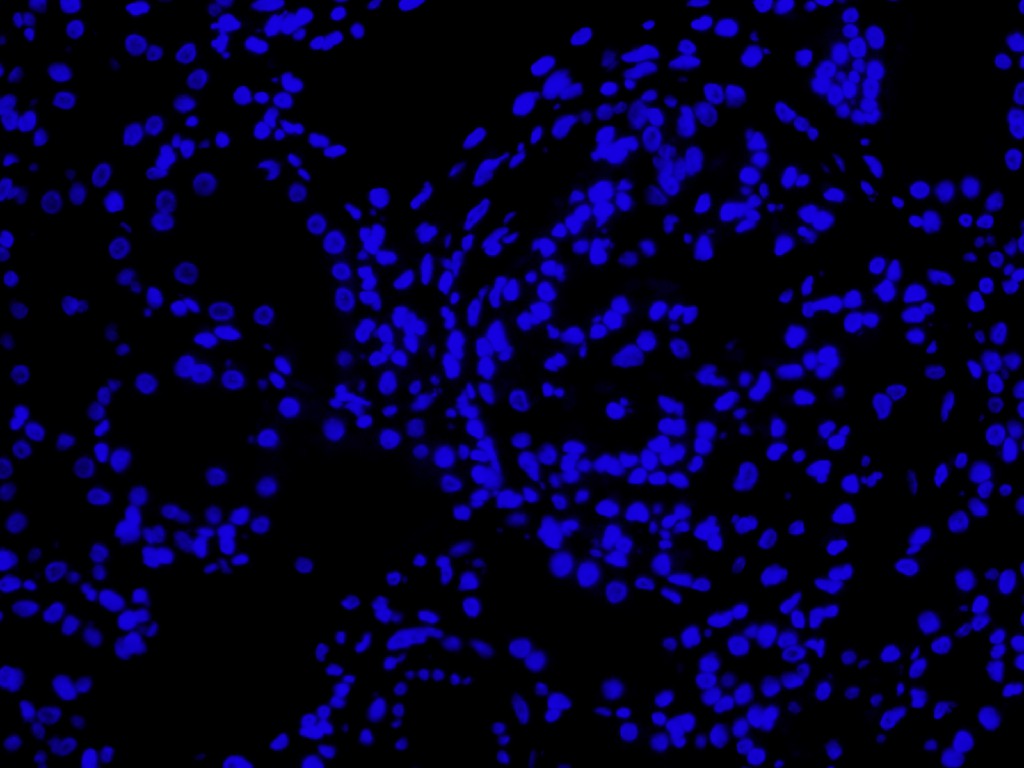

Supplement: Supplementary file 9 — Figure EV1 Source Data [file 44321_2025_315_MOESM9_ESM.zip › Figure EV1/EV1D/1-Podocin-GLDC/LEE III/4 (3).jpg]

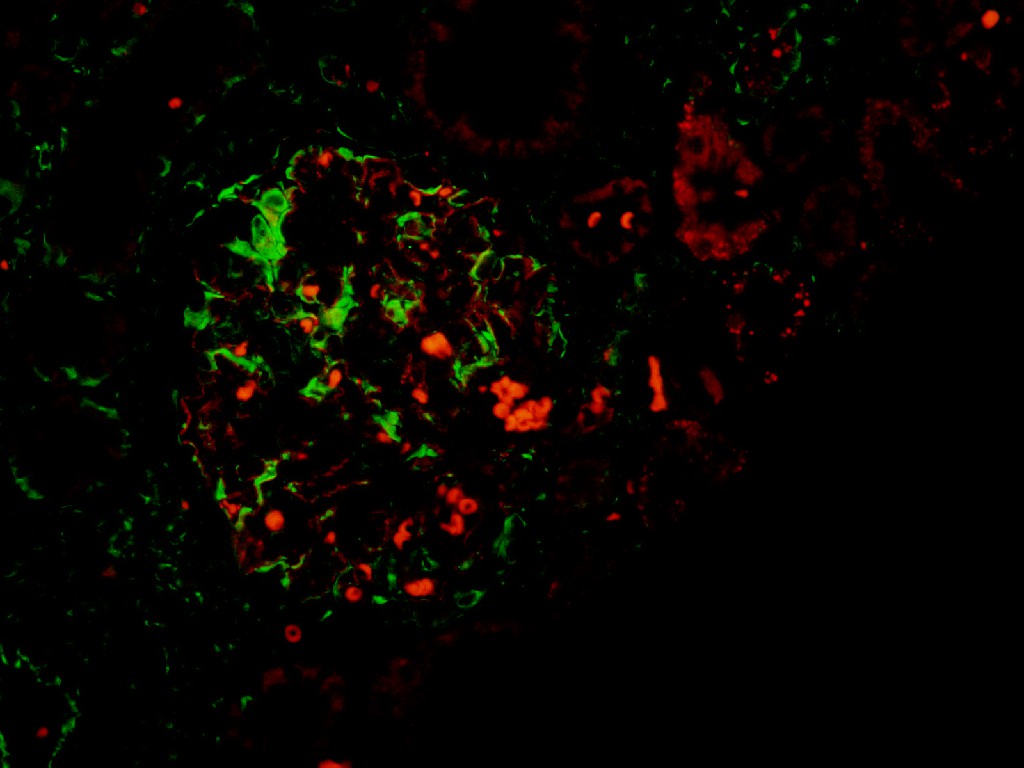

Supplement: Supplementary file 9 — Figure EV1 Source Data [file 44321_2025_315_MOESM9_ESM.zip › Figure EV1/EV1D/1-Podocin-GLDC/LEE III/6 (4).jpg]

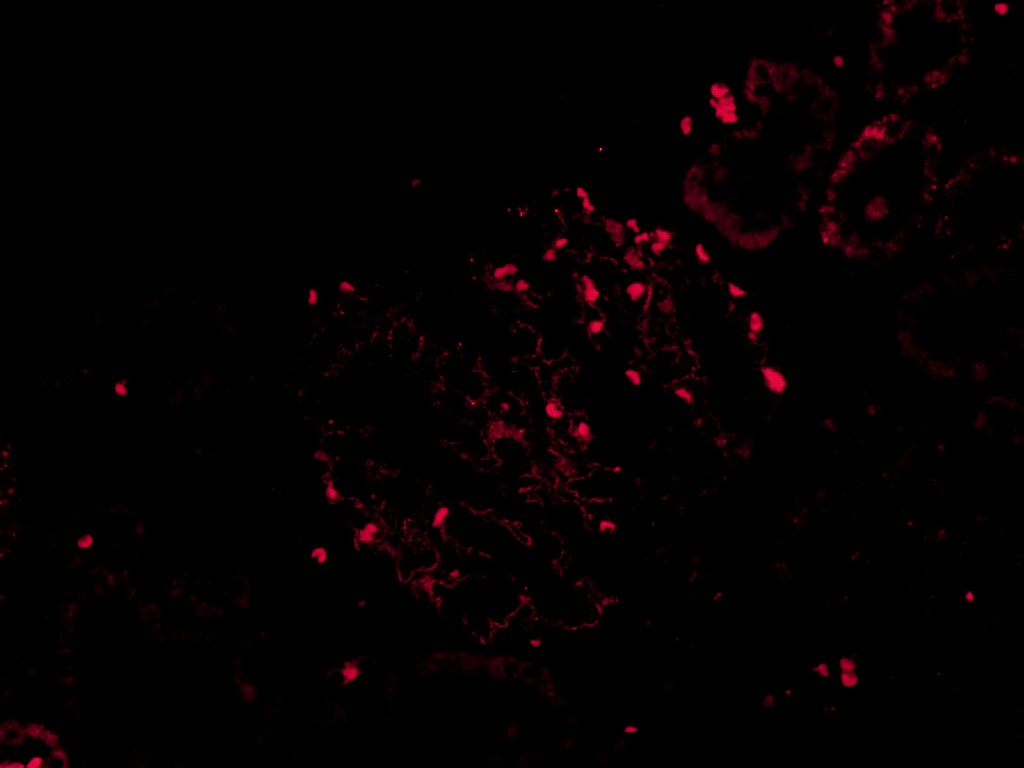

Supplement: Supplementary file 9 — Figure EV1 Source Data [file 44321_2025_315_MOESM9_ESM.zip › Figure EV1/EV1D/1-Podocin-GLDC/LEE III/5 (2).jpg]

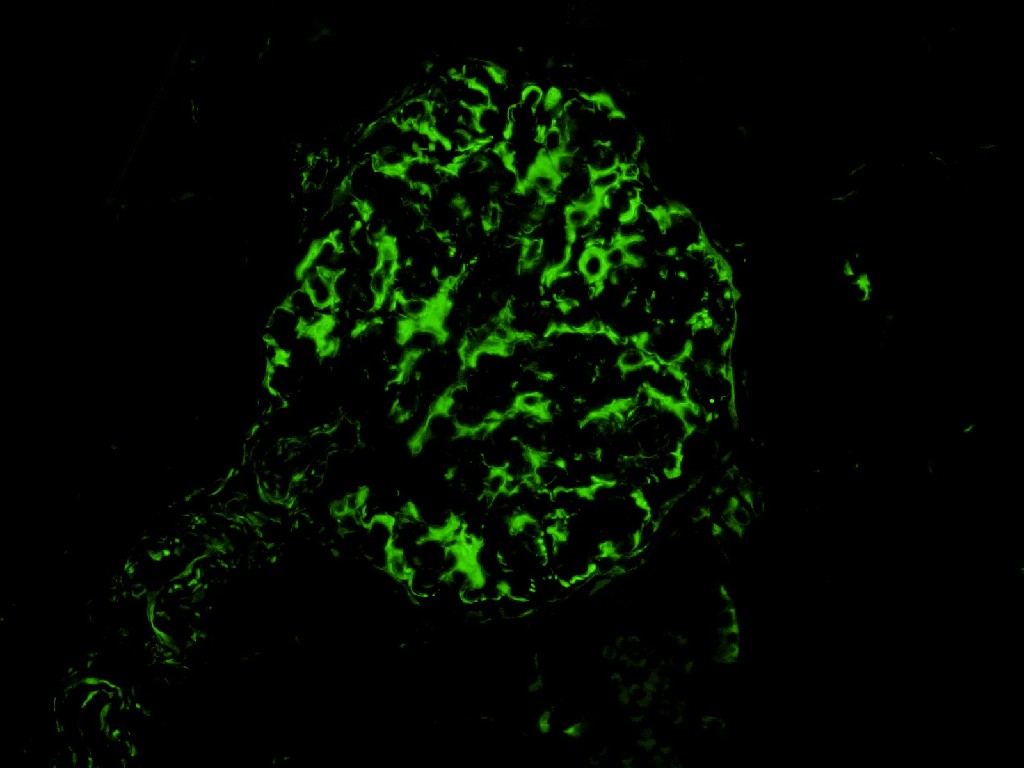

Supplement: Supplementary file 9 — Figure EV1 Source Data [file 44321_2025_315_MOESM9_ESM.zip › Figure EV1/EV1D/1-Podocin-GLDC/LEE III/9 (1).jpg]

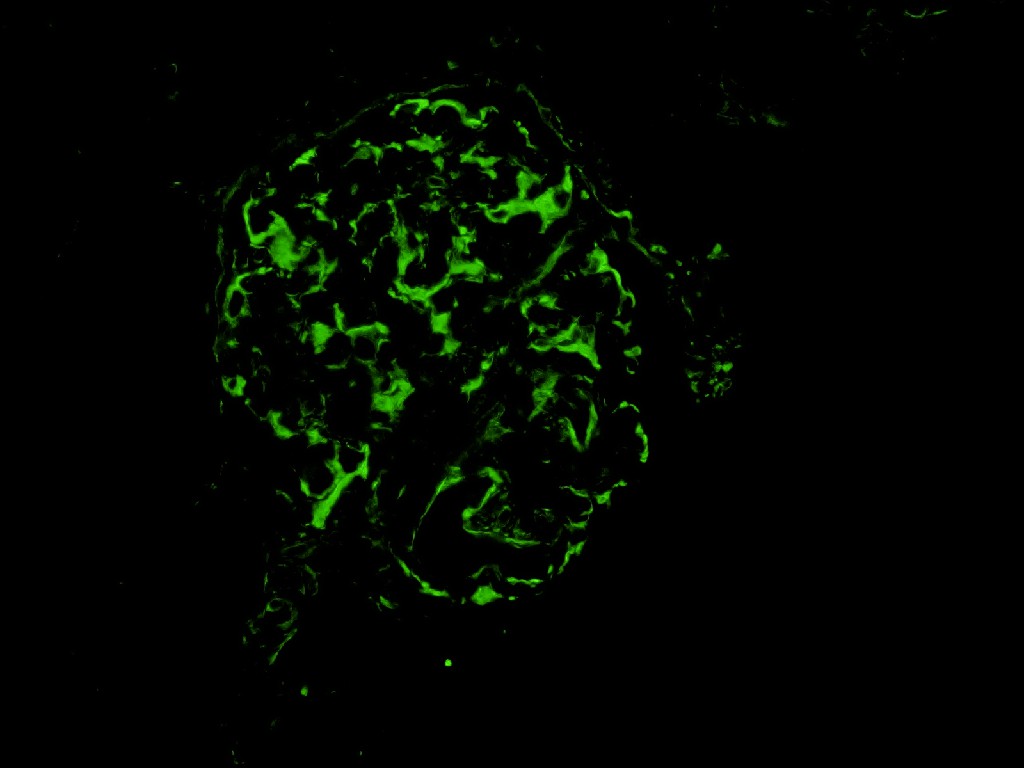

Supplement: Supplementary file 9 — Figure EV1 Source Data [file 44321_2025_315_MOESM9_ESM.zip › Figure EV1/EV1D/1-Podocin-GLDC/LEE III/8 (1).jpg]

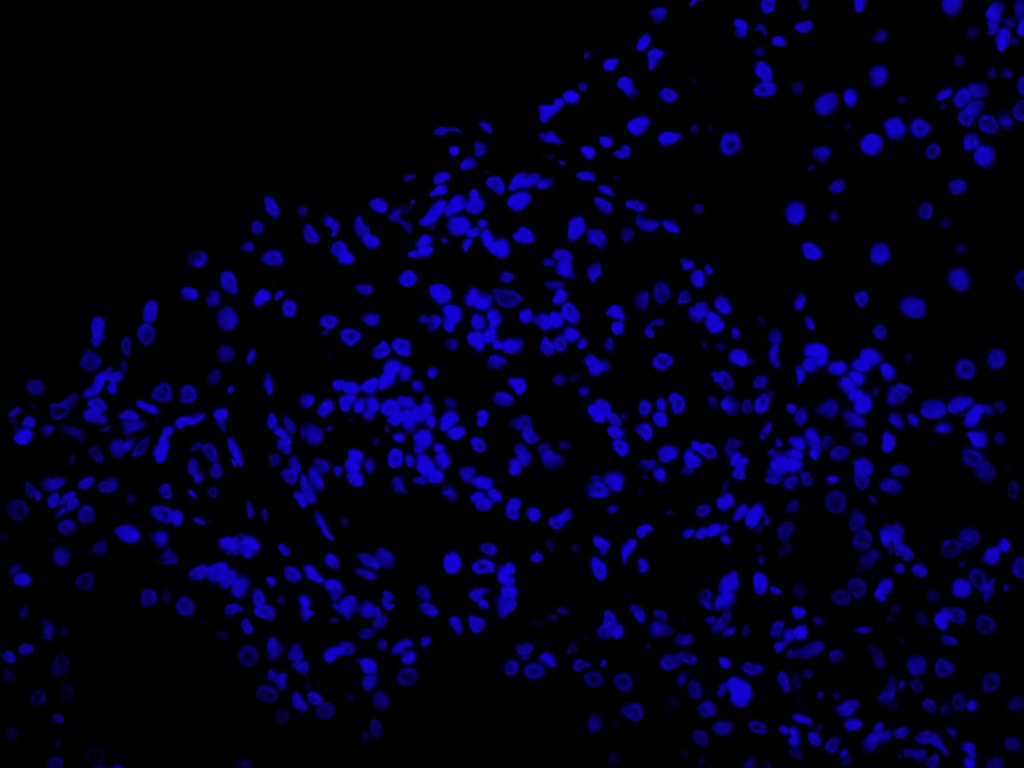

Supplement: Supplementary file 9 — Figure EV1 Source Data [file 44321_2025_315_MOESM9_ESM.zip › Figure EV1/EV1D/1-Podocin-GLDC/LEE III/5 (3).jpg]

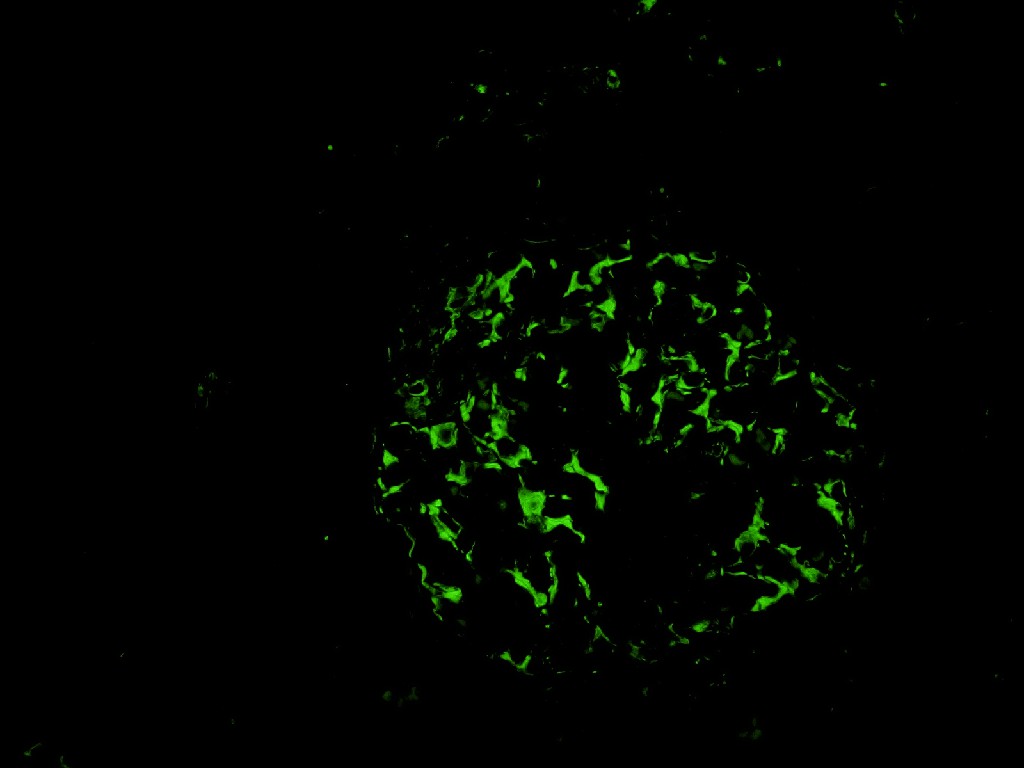

Supplement: Supplementary file 9 — Figure EV1 Source Data [file 44321_2025_315_MOESM9_ESM.zip › Figure EV1/EV1D/1-Podocin-GLDC/LEE III/1 (1).jpg]

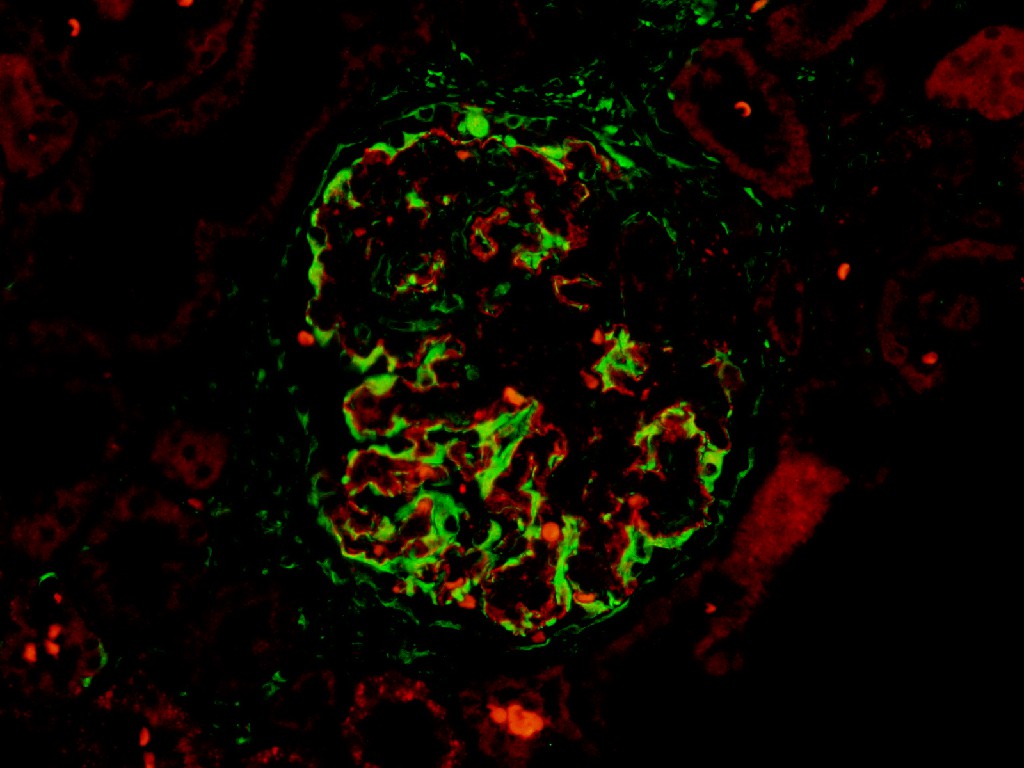

Supplement: Supplementary file 9 — Figure EV1 Source Data [file 44321_2025_315_MOESM9_ESM.zip › Figure EV1/EV1D/1-Podocin-GLDC/LEE III/7 (4).jpg]

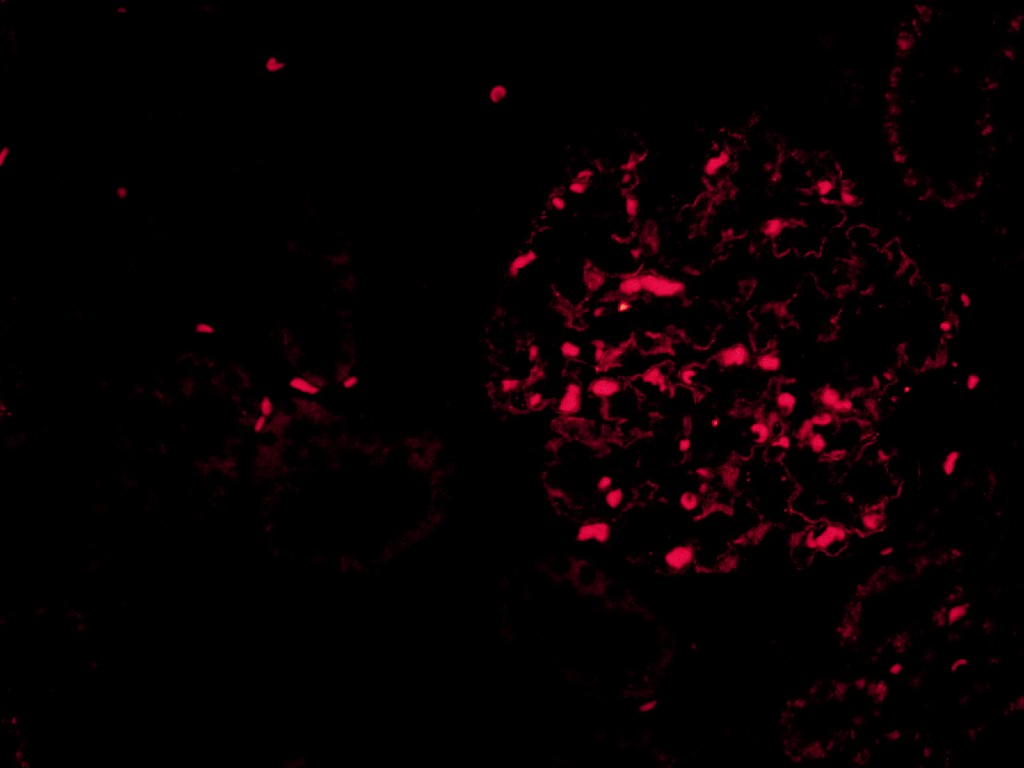

Supplement: Supplementary file 9 — Figure EV1 Source Data [file 44321_2025_315_MOESM9_ESM.zip › Figure EV1/EV1D/1-Podocin-GLDC/LEE III/4 (2).jpg]

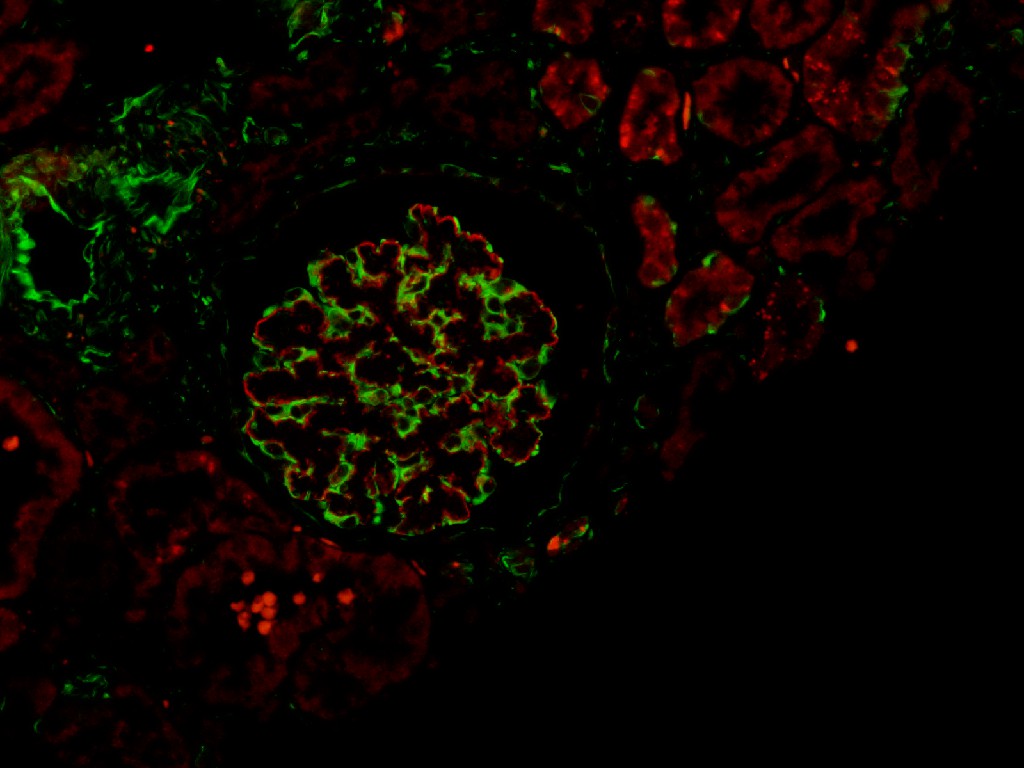

Supplement: Supplementary file 9 — Figure EV1 Source Data [file 44321_2025_315_MOESM9_ESM.zip › Figure EV1/EV1D/1-Podocin-GLDC/LEE III/2 (4).jpg]

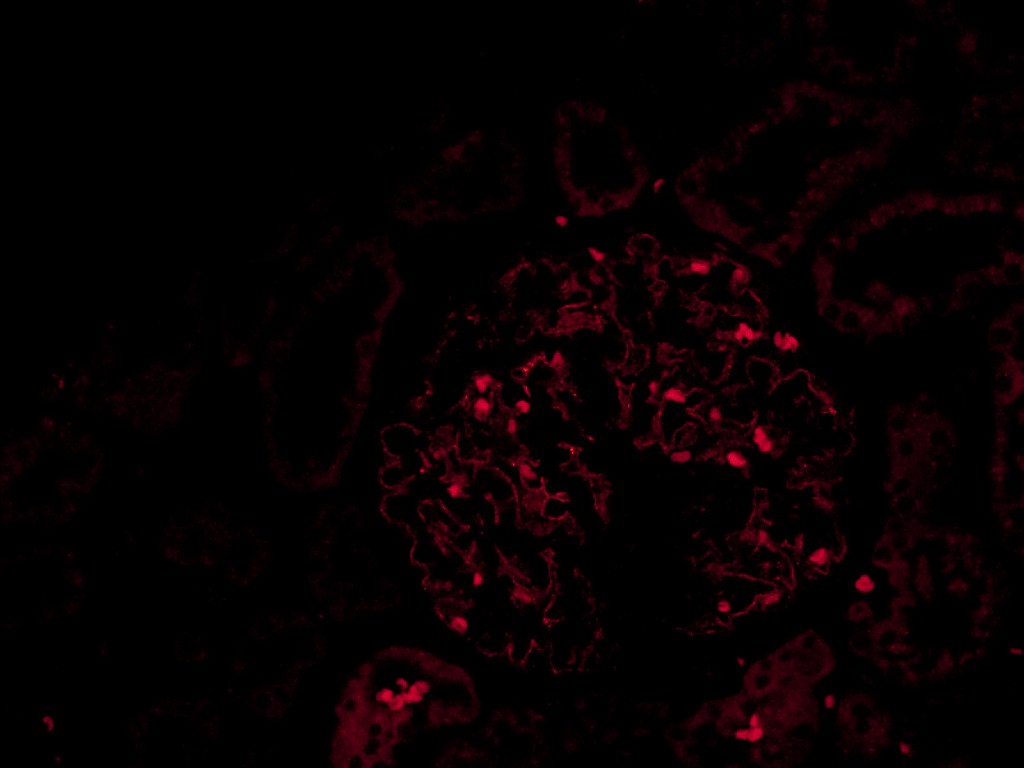

Supplement: Supplementary file 9 — Figure EV1 Source Data [file 44321_2025_315_MOESM9_ESM.zip › Figure EV1/EV1D/1-Podocin-GLDC/LEE III/1 (2).jpg]

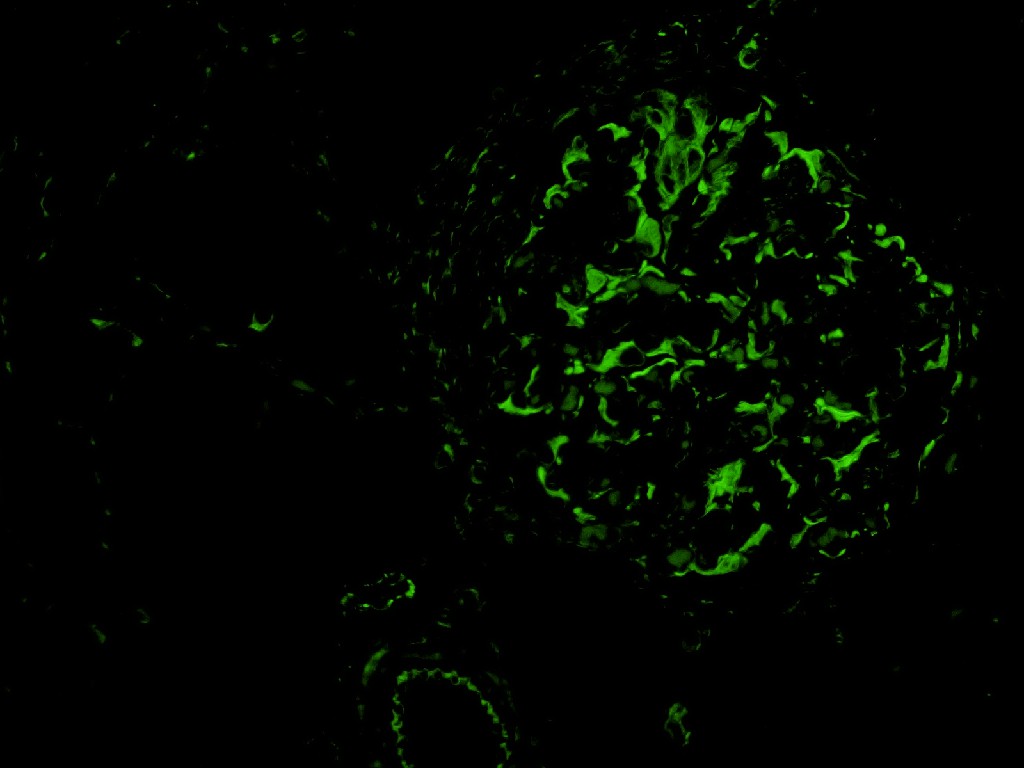

Supplement: Supplementary file 9 — Figure EV1 Source Data [file 44321_2025_315_MOESM9_ESM.zip › Figure EV1/EV1D/1-Podocin-GLDC/LEE III/4 (1).jpg]

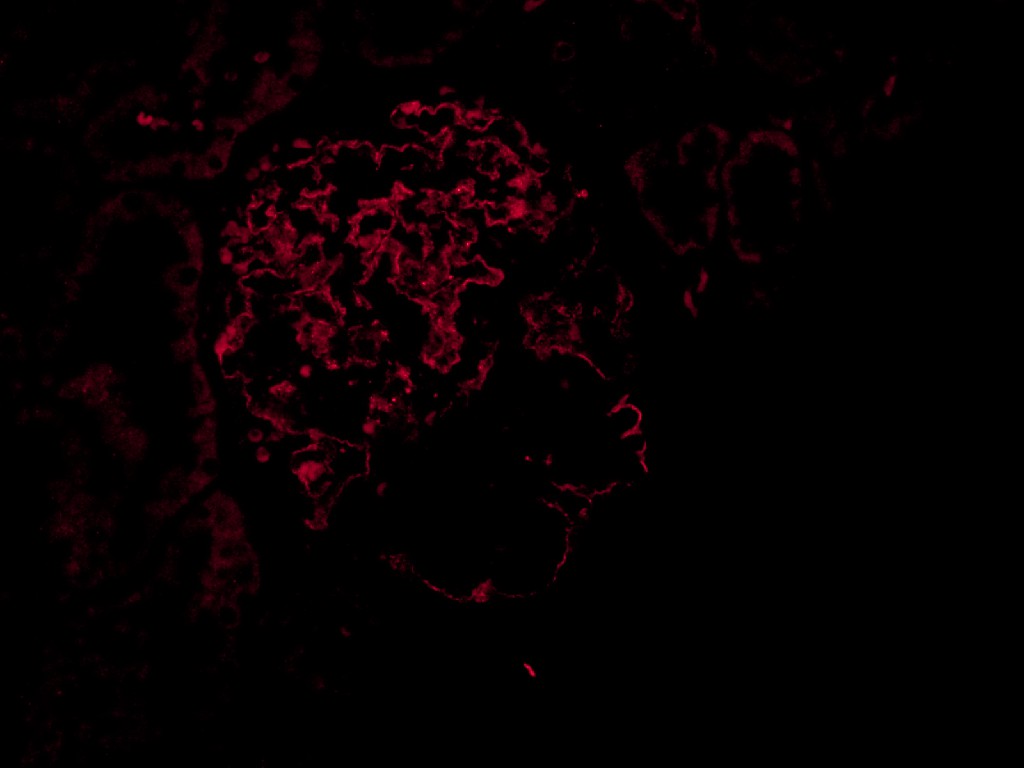

Supplement: Supplementary file 9 — Figure EV1 Source Data [file 44321_2025_315_MOESM9_ESM.zip › Figure EV1/EV1D/1-Podocin-GLDC/LEE III/8 (2).jpg]

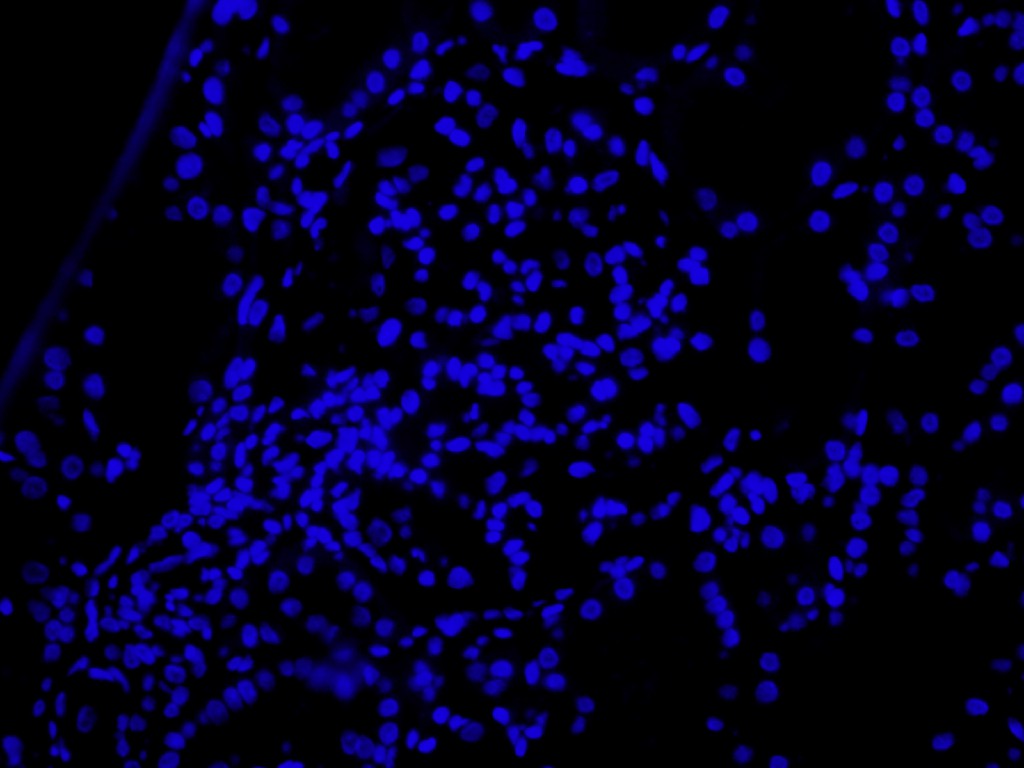

Supplement: Supplementary file 9 — Figure EV1 Source Data [file 44321_2025_315_MOESM9_ESM.zip › Figure EV1/EV1D/1-Podocin-GLDC/LEE III/9 (3).jpg]

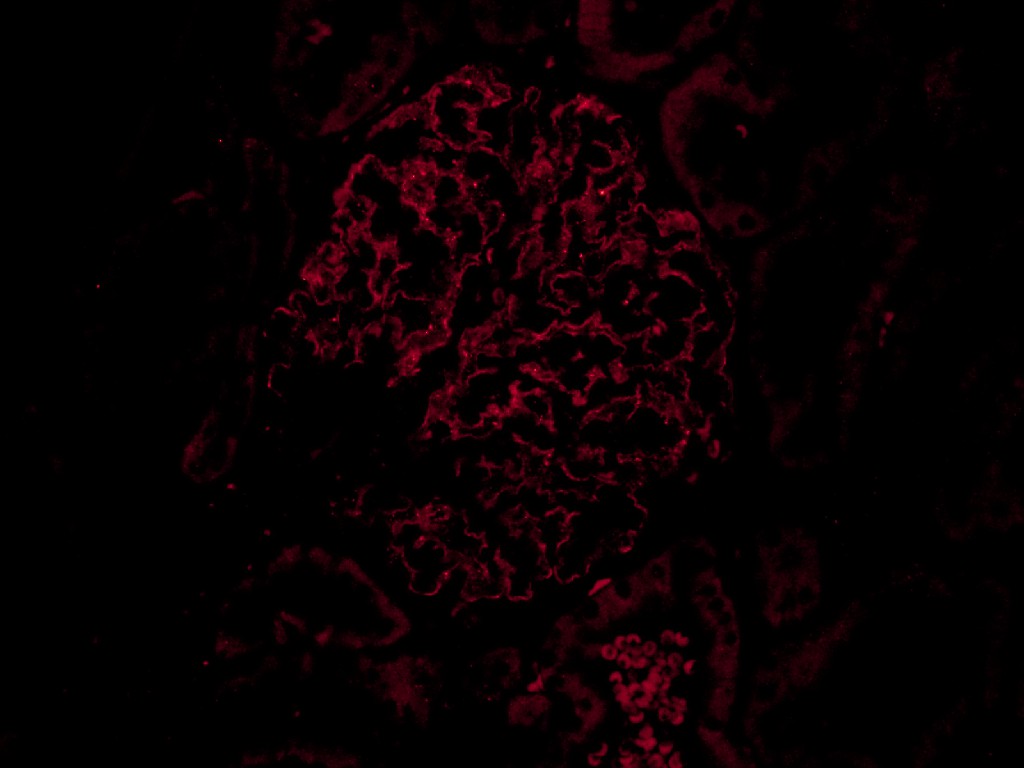

Supplement: Supplementary file 9 — Figure EV1 Source Data [file 44321_2025_315_MOESM9_ESM.zip › Figure EV1/EV1D/1-Podocin-GLDC/LEE III/9 (2).jpg]

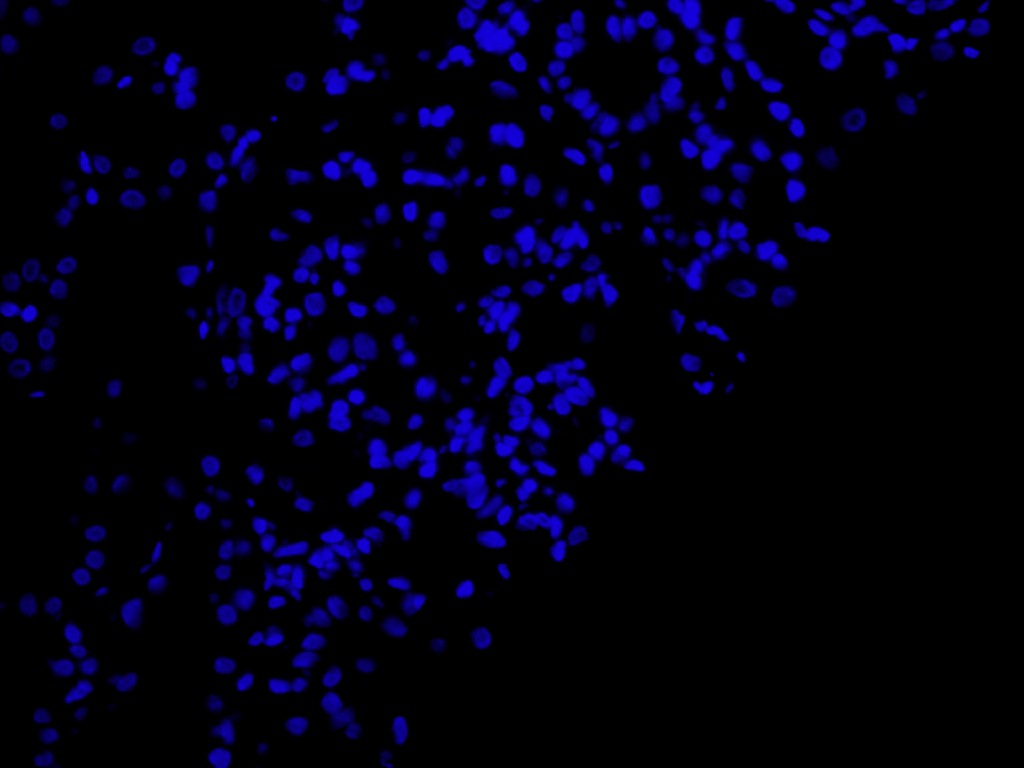

Supplement: Supplementary file 9 — Figure EV1 Source Data [file 44321_2025_315_MOESM9_ESM.zip › Figure EV1/EV1D/1-Podocin-GLDC/LEE III/8 (3).jpg]

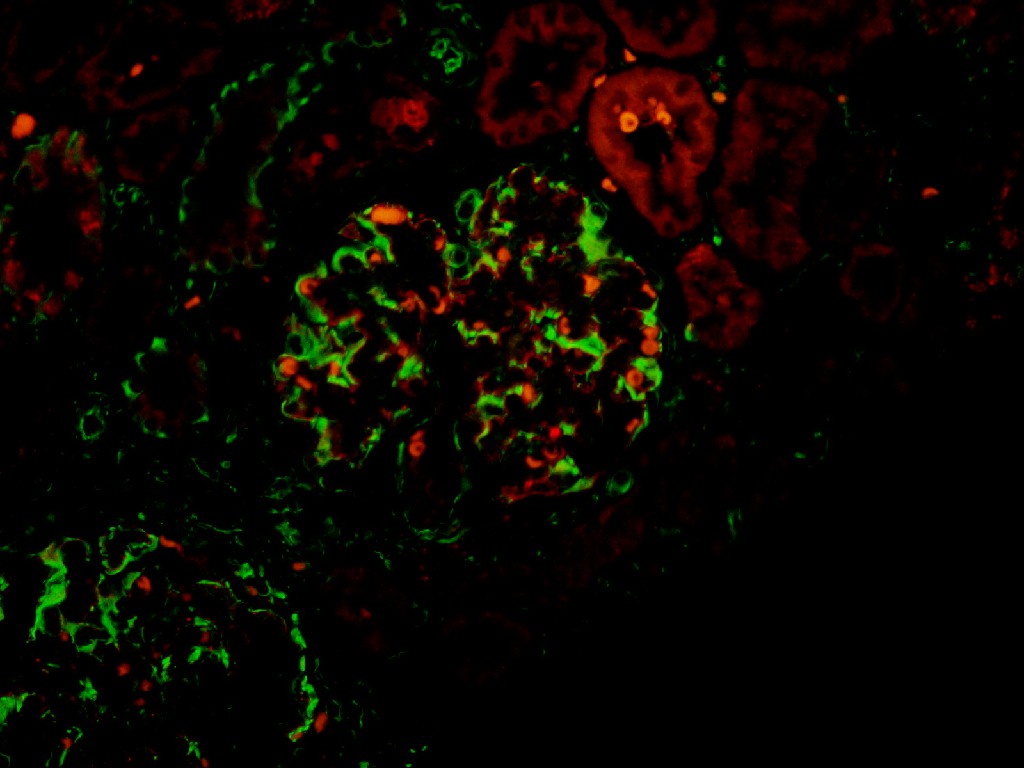

Supplement: Supplementary file 9 — Figure EV1 Source Data [file 44321_2025_315_MOESM9_ESM.zip › Figure EV1/EV1D/1-Podocin-GLDC/LEE III/3 (4).jpg]

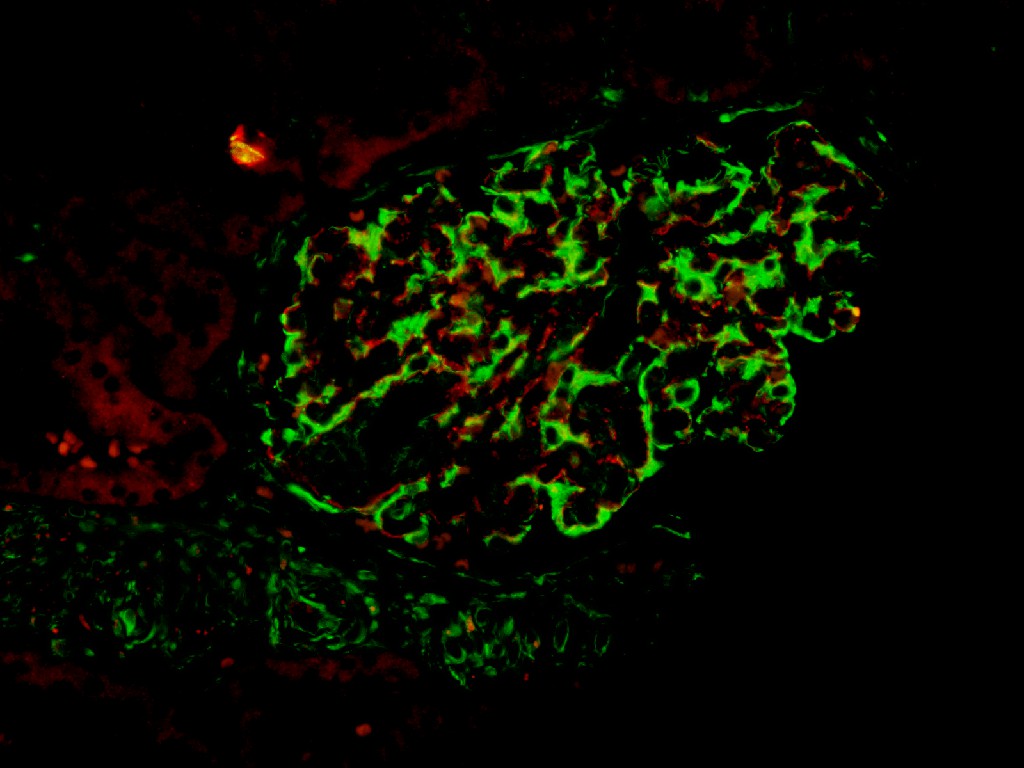

Supplement: Supplementary file 9 — Figure EV1 Source Data [file 44321_2025_315_MOESM9_ESM.zip › Figure EV1/EV1D/1-Podocin-GLDC/LEE III/10 (4).jpg]

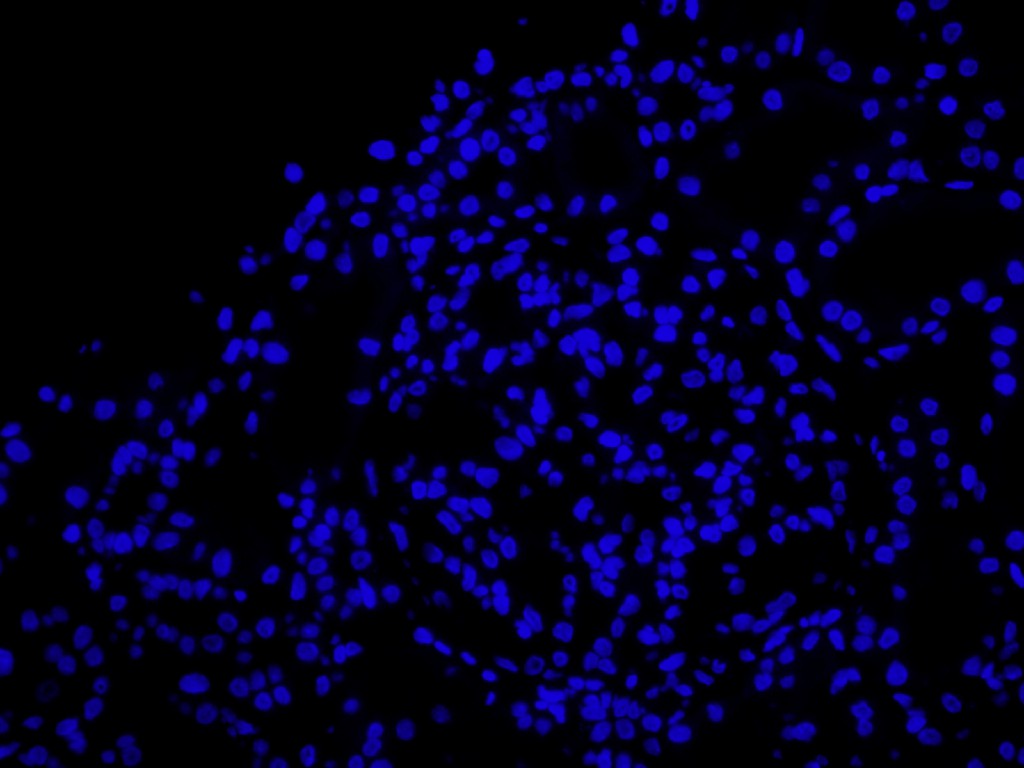

Supplement: Supplementary file 9 — Figure EV1 Source Data [file 44321_2025_315_MOESM9_ESM.zip › Figure EV1/EV1D/1-Podocin-GLDC/LEE III/1 (3).jpg]

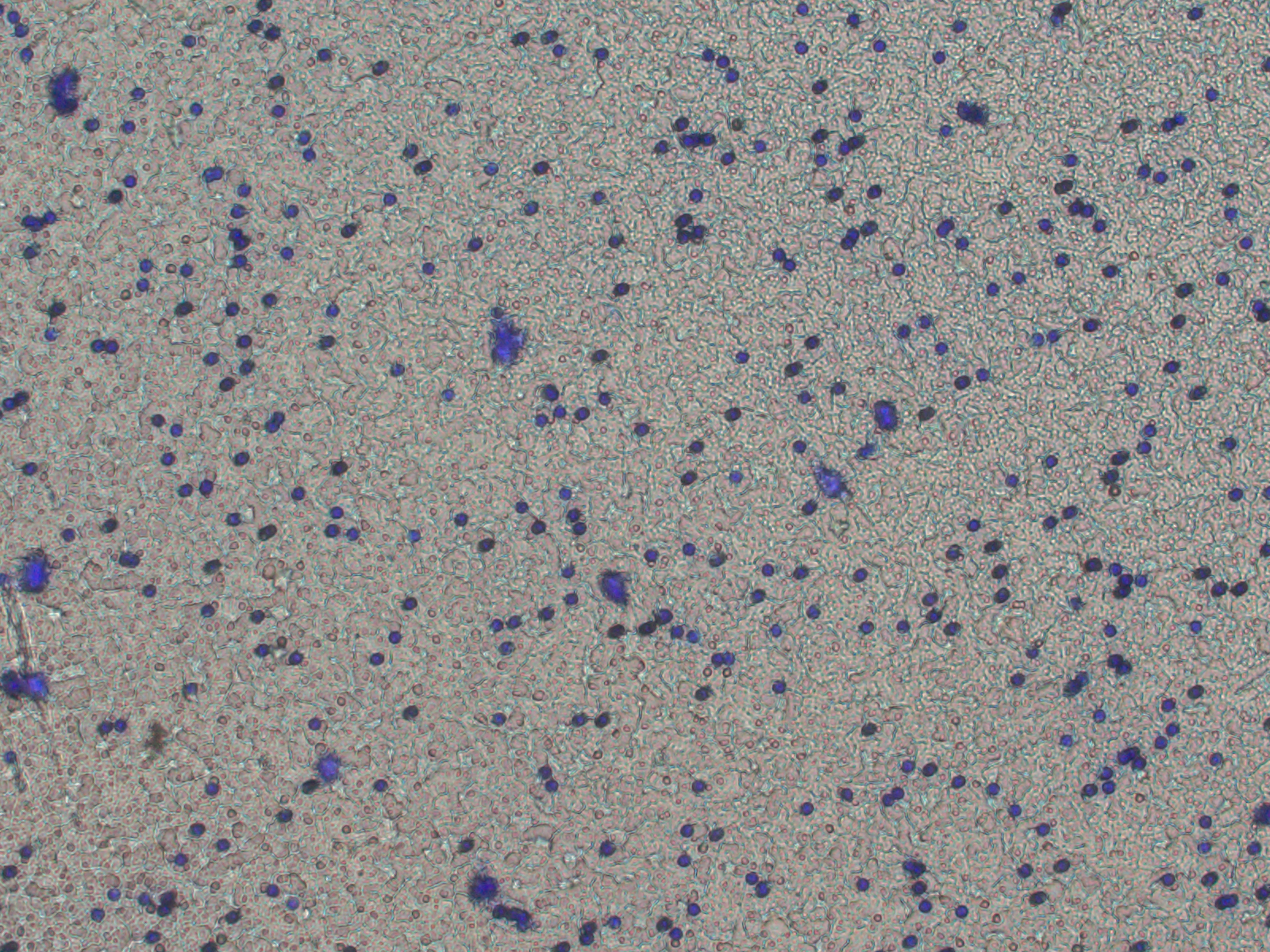

Supplement: Supplementary file 10 — Figure EV2 Source Data [file 44321_2025_315_MOESM10_ESM.zip › Figure EV2/EV2B/1-Control.tif]

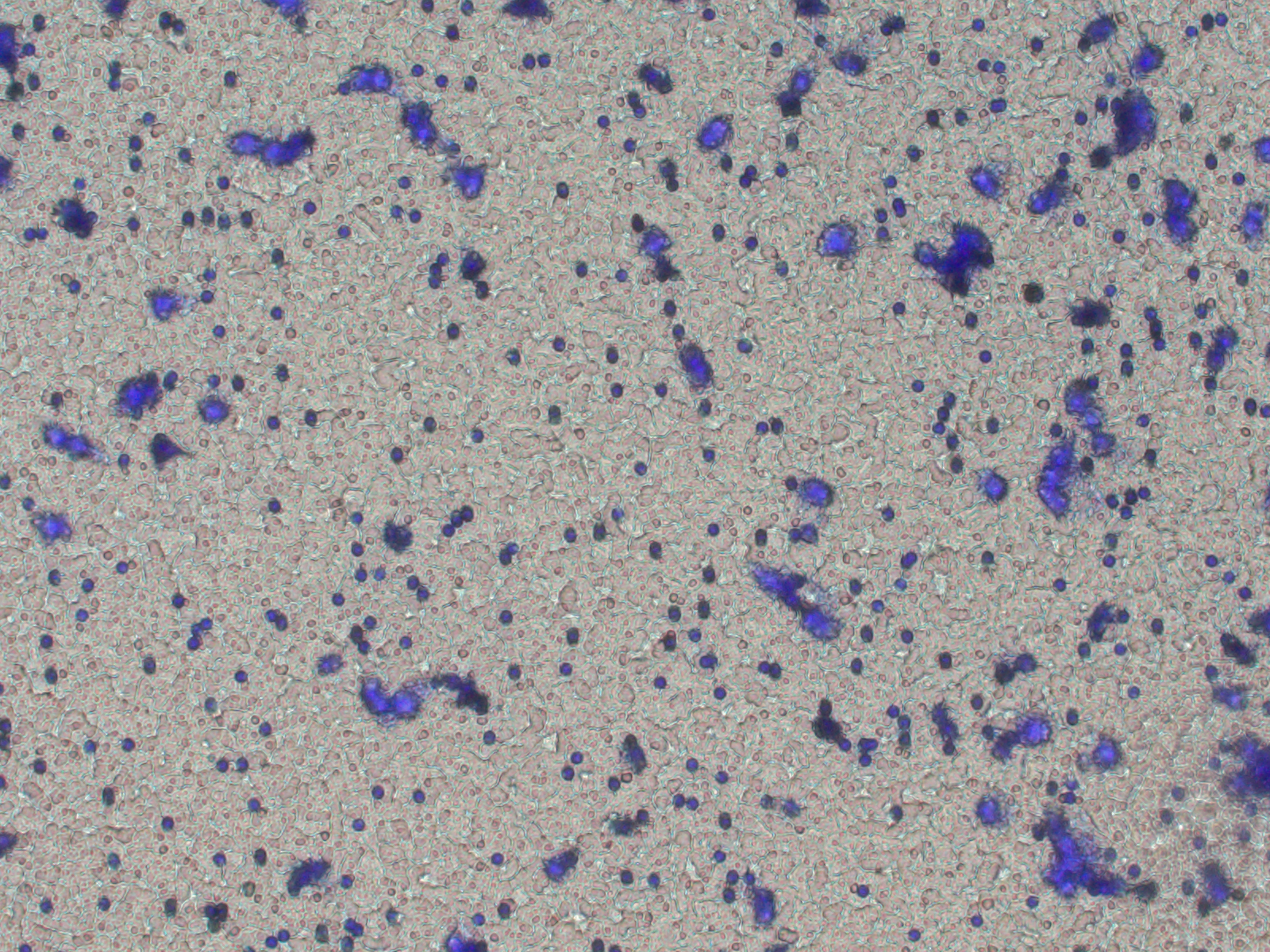

Supplement: Supplementary file 10 — Figure EV2 Source Data [file 44321_2025_315_MOESM10_ESM.zip › Figure EV2/EV2B/2-pIgA.tif]

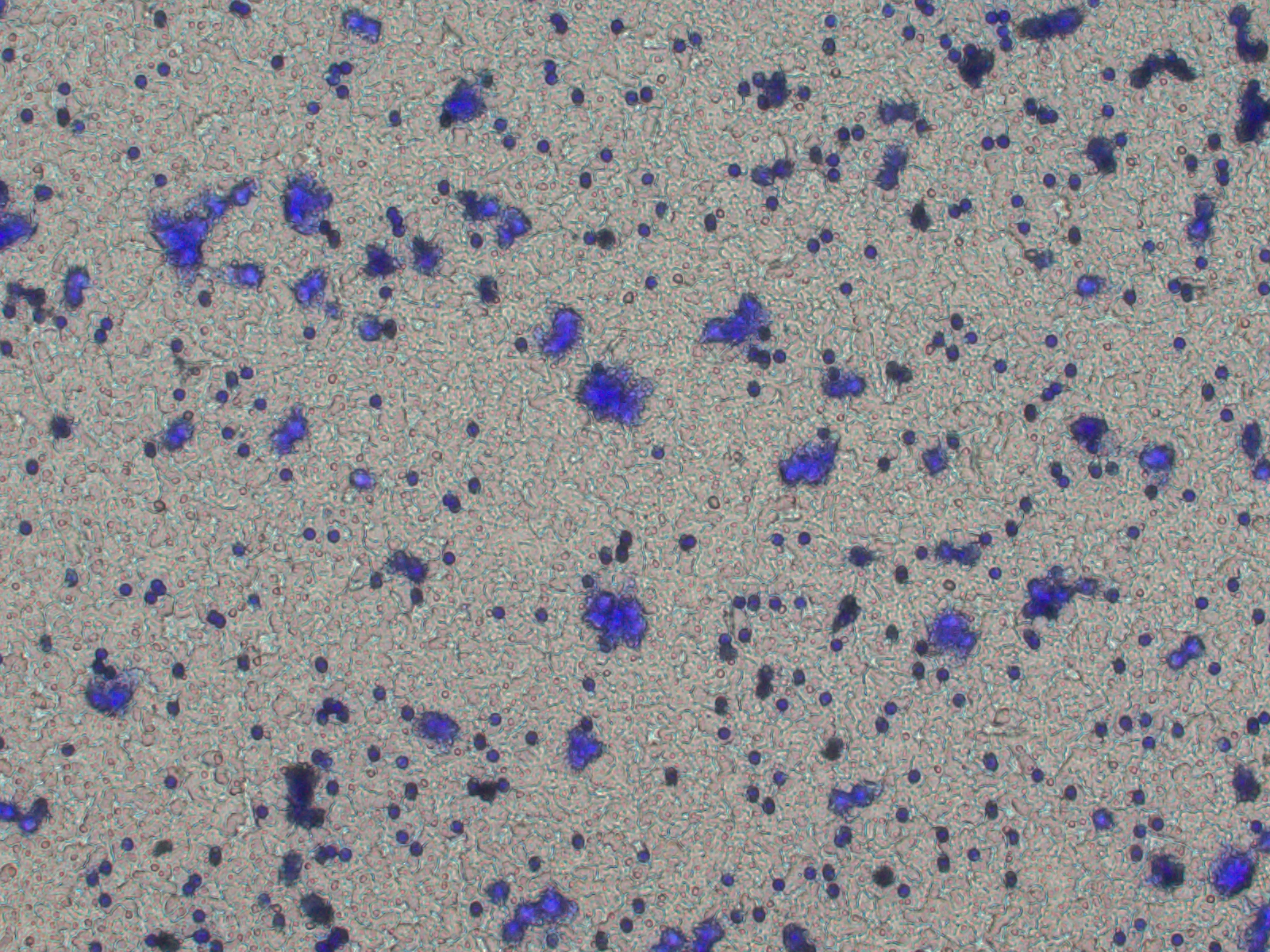

Supplement: Supplementary file 10 — Figure EV2 Source Data [file 44321_2025_315_MOESM10_ESM.zip › Figure EV2/EV2B/3-pIgA+si-NC.tif]

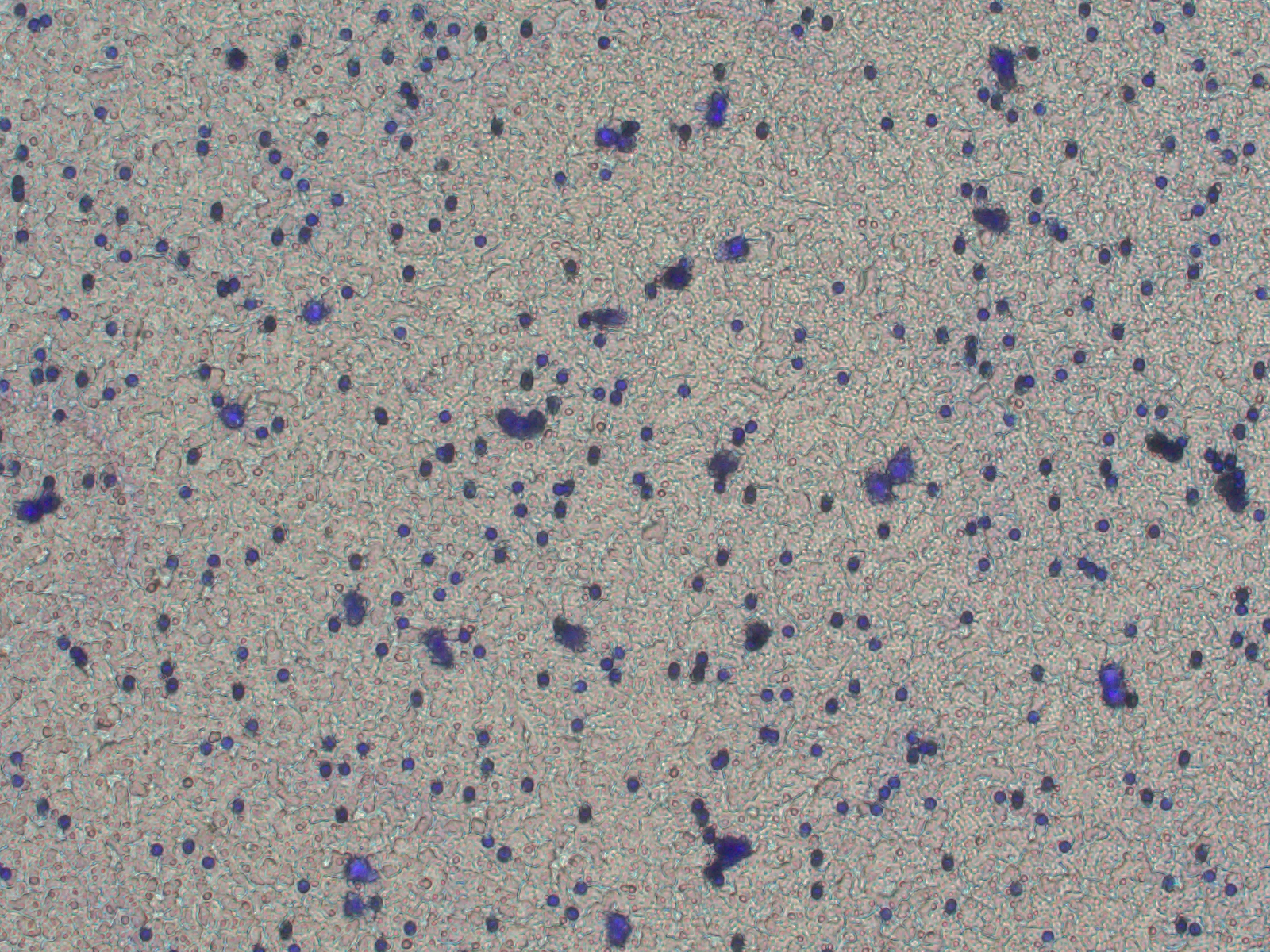

Supplement: Supplementary file 10 — Figure EV2 Source Data [file 44321_2025_315_MOESM10_ESM.zip › Figure EV2/EV2B/4-pIgA+si-GLDC.tif]

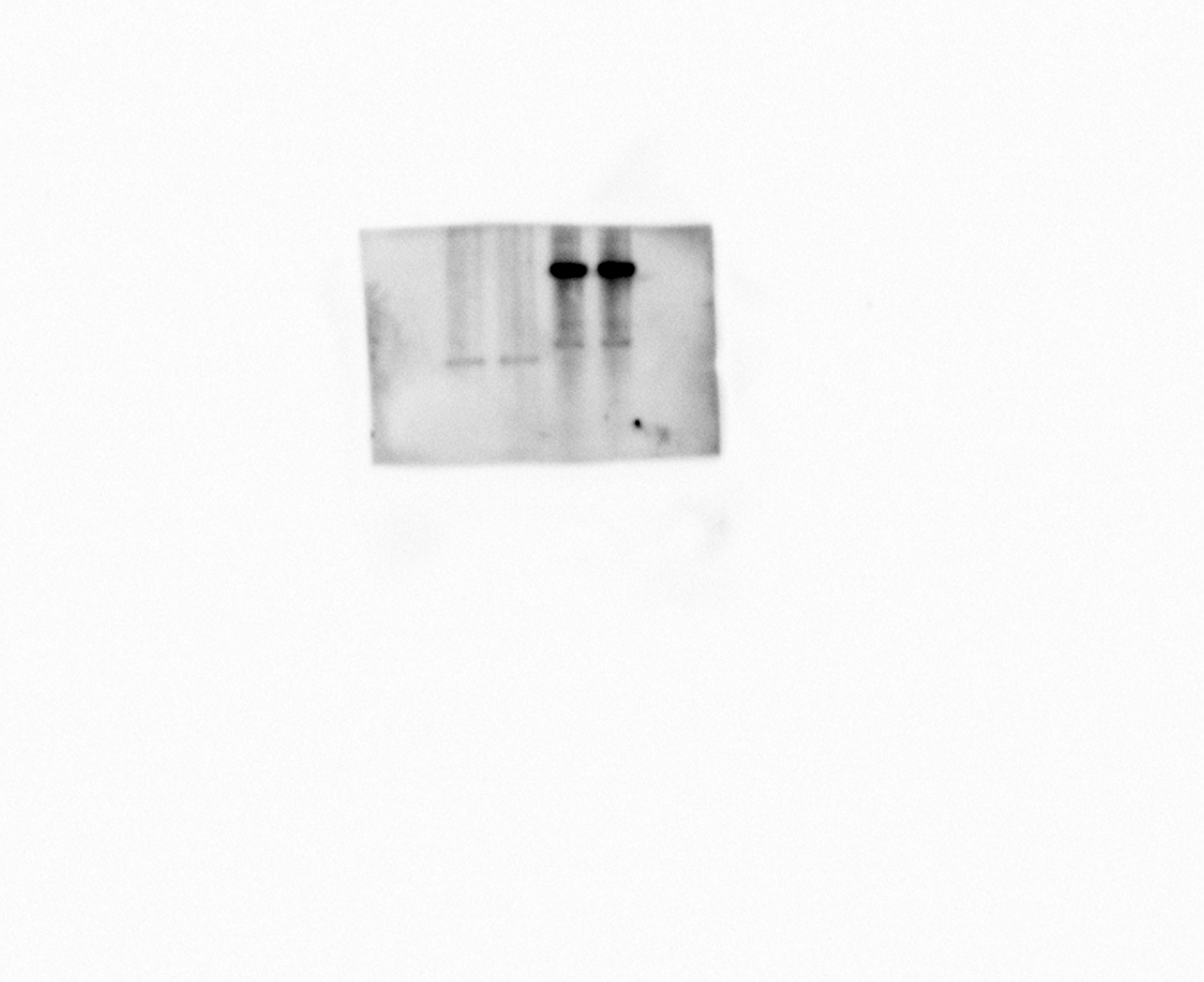

Supplement: Supplementary file 10 — Figure EV2 Source Data [file 44321_2025_315_MOESM10_ESM.zip › Figure EV2/EV2A/3.Tif]

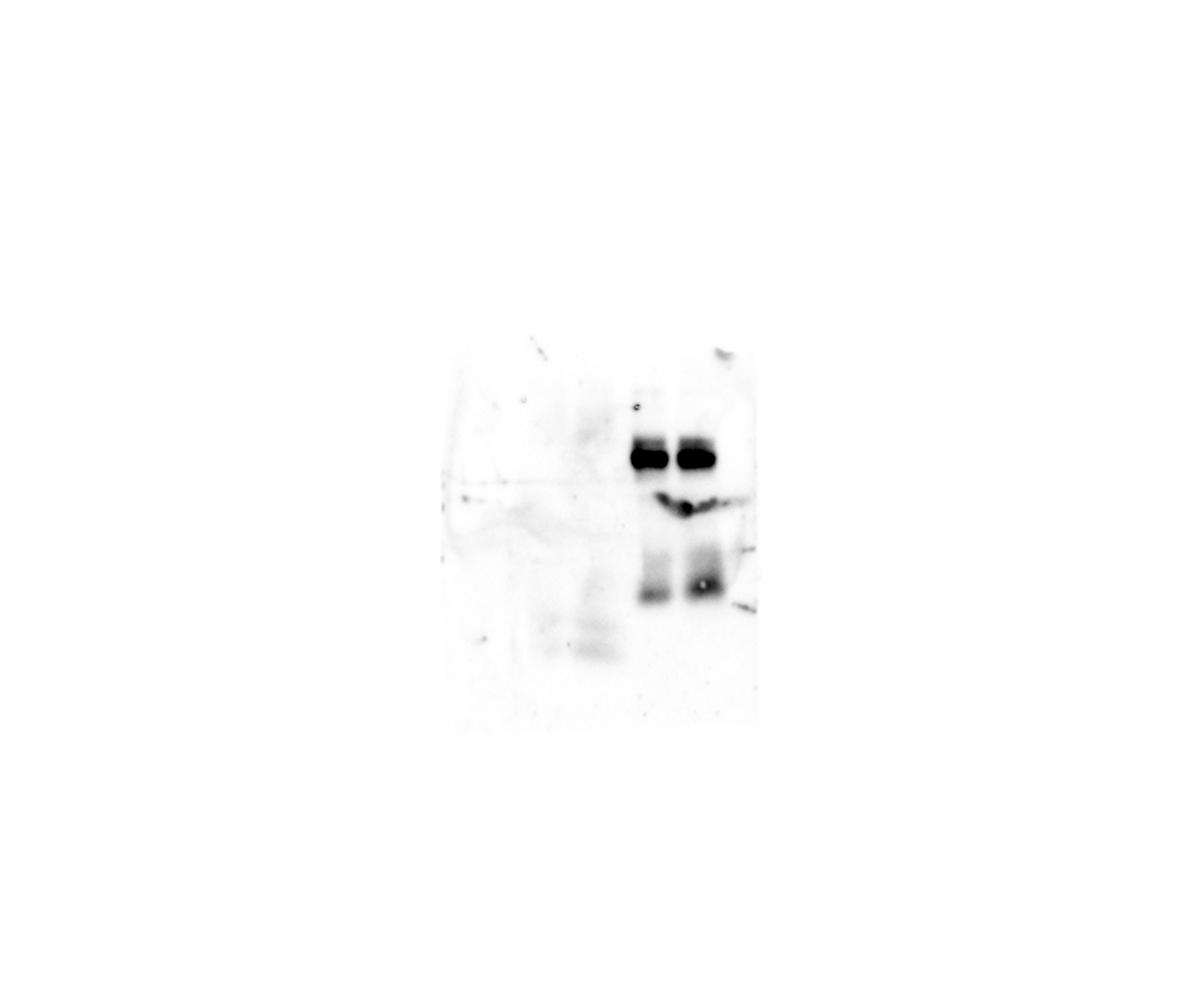

Supplement: Supplementary file 10 — Figure EV2 Source Data [file 44321_2025_315_MOESM10_ESM.zip › Figure EV2/EV2A/2.Tif]

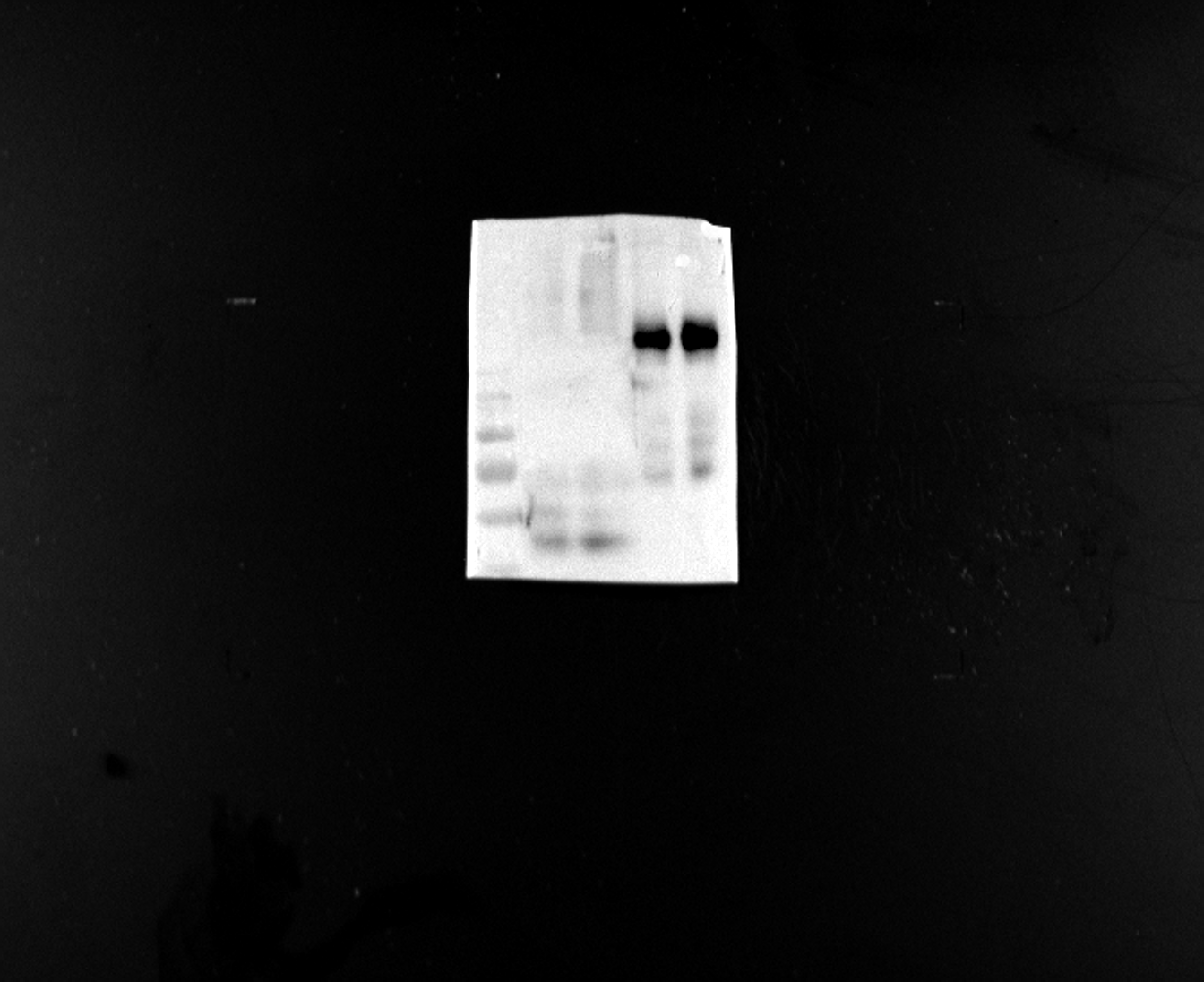

Supplement: Supplementary file 10 — Figure EV2 Source Data [file 44321_2025_315_MOESM10_ESM.zip › Figure EV2/EV2A/1.Tif]

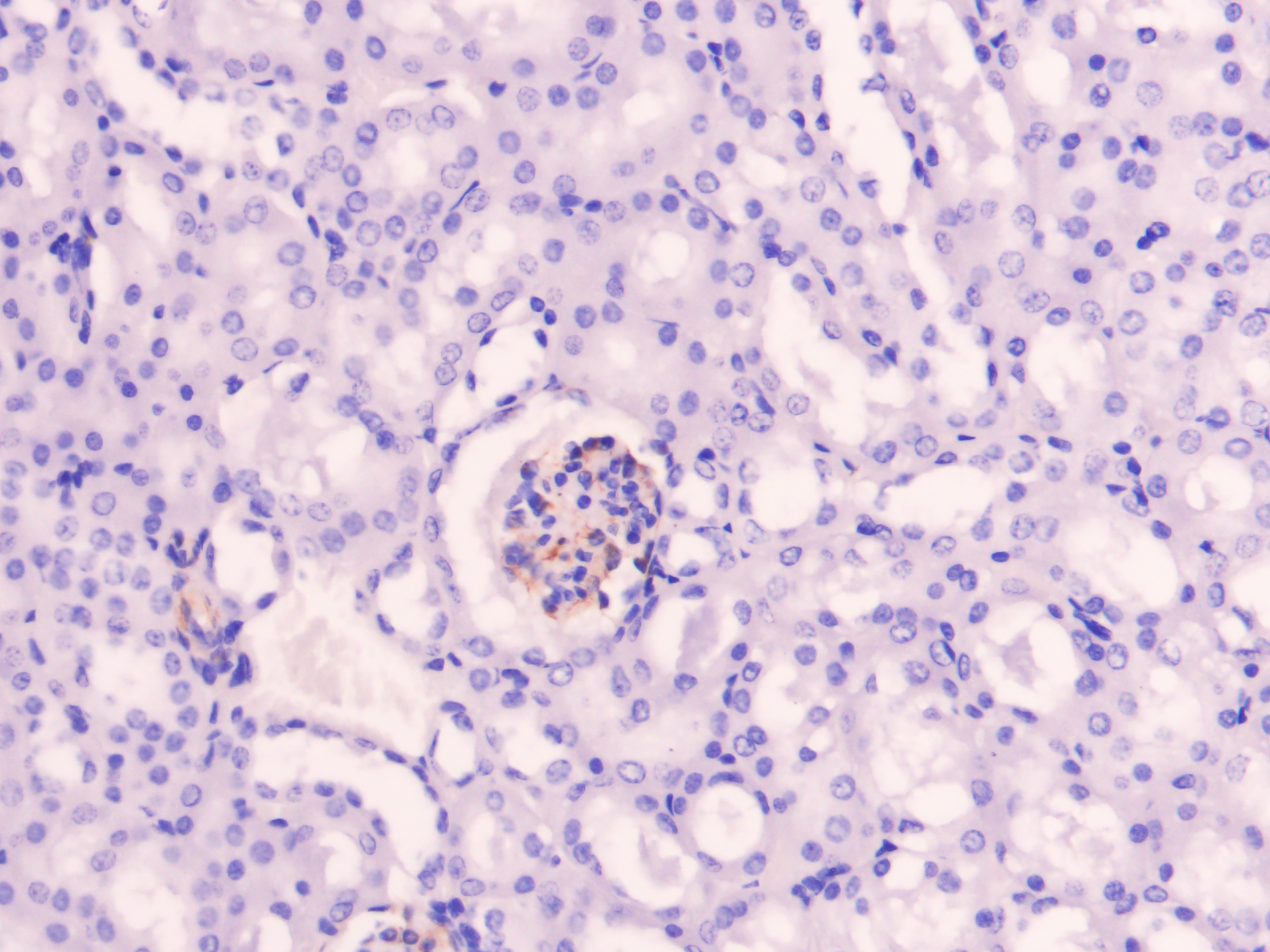

Supplement: Supplementary file 12 — Figure EV4 Source Data [file 44321_2025_315_MOESM12_ESM.zip › Figure EV4/EV4A-CAD/1-Control.tif]

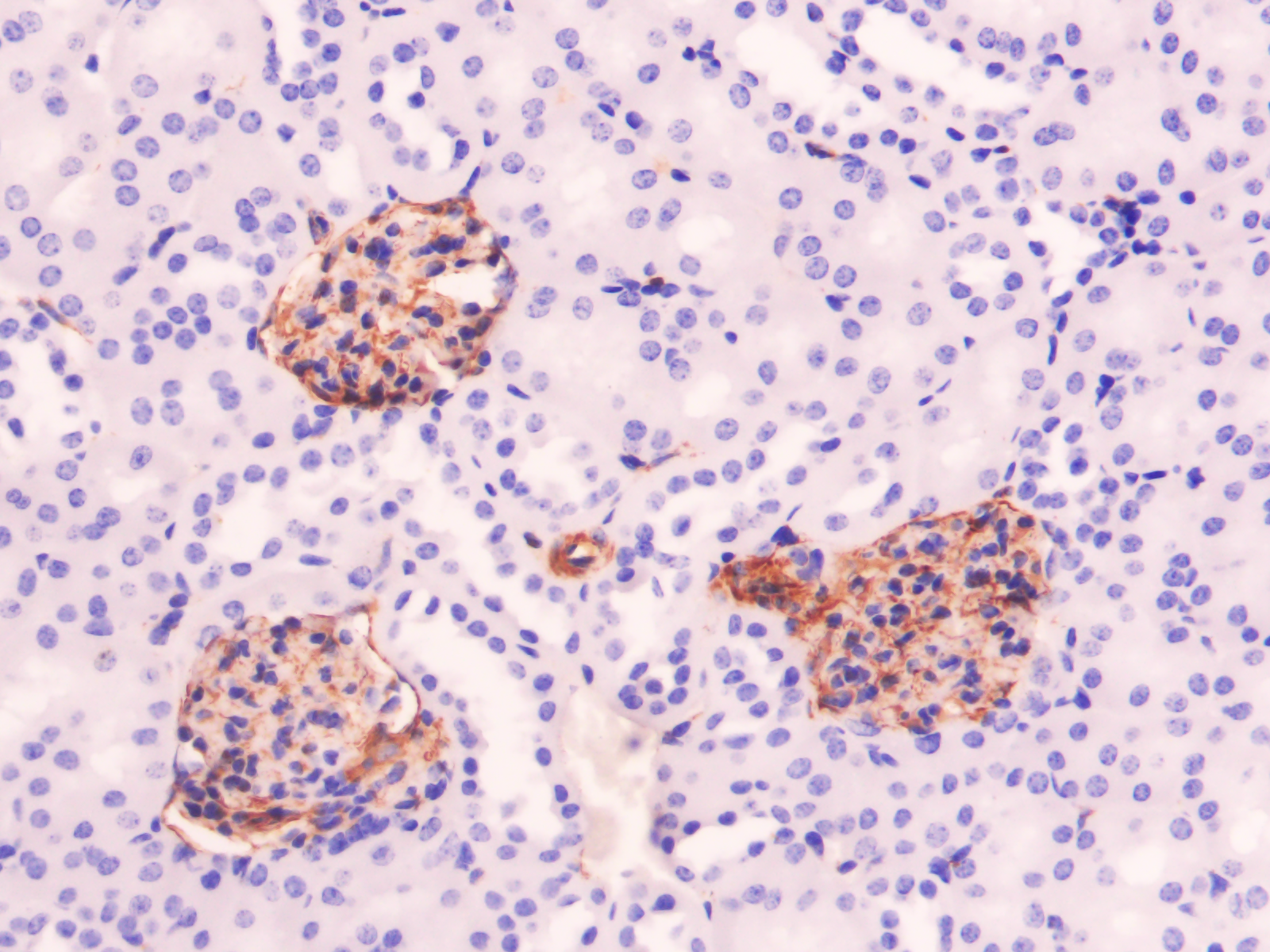

Supplement: Supplementary file 12 — Figure EV4 Source Data [file 44321_2025_315_MOESM12_ESM.zip › Figure EV4/EV4A-CAD/2-IgAN.tif]

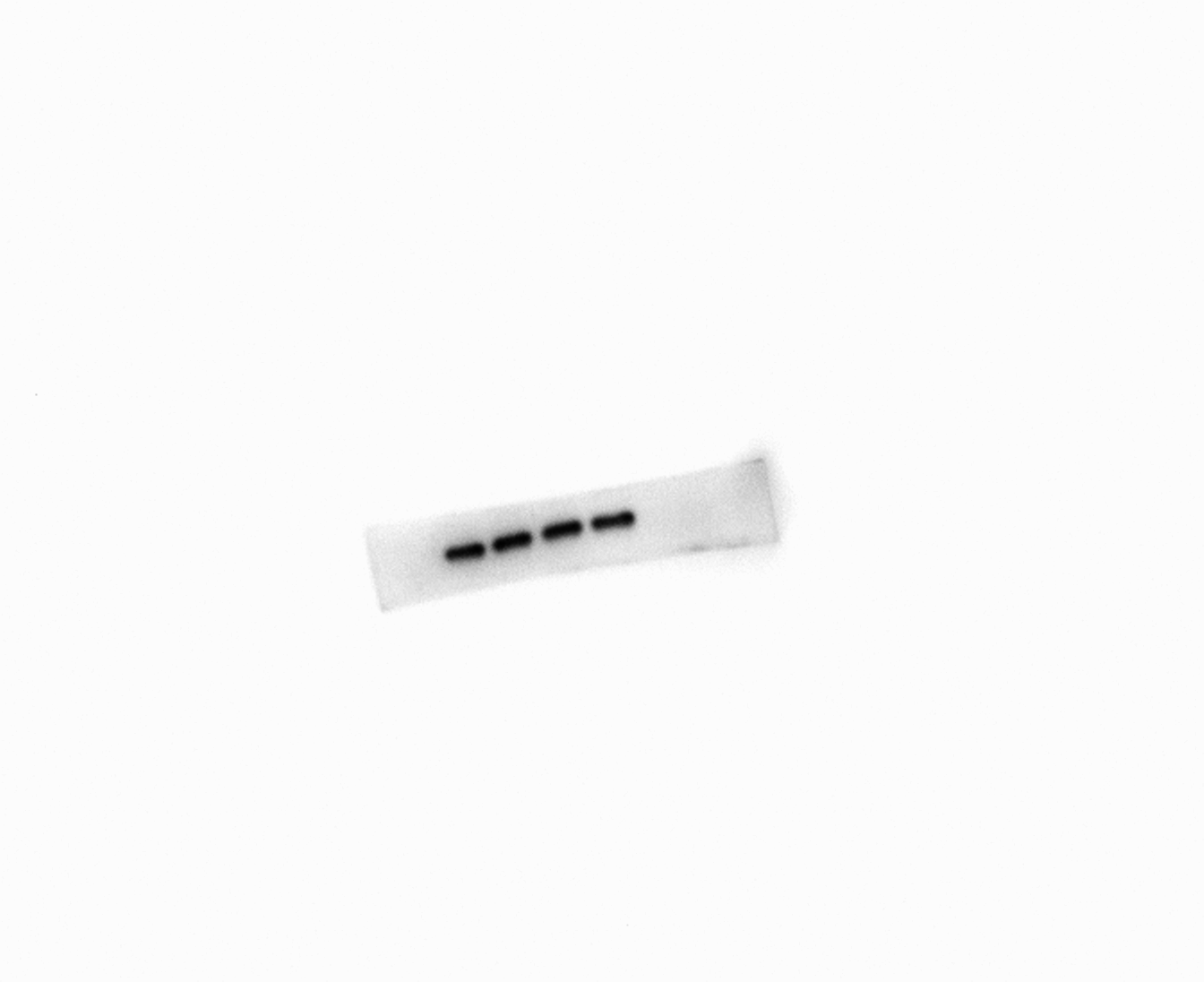

Supplement: Supplementary file 12 — Figure EV4 Source Data [file 44321_2025_315_MOESM12_ESM.zip › Figure EV4/EV4B-WB/3-2-beta-actin.tif]

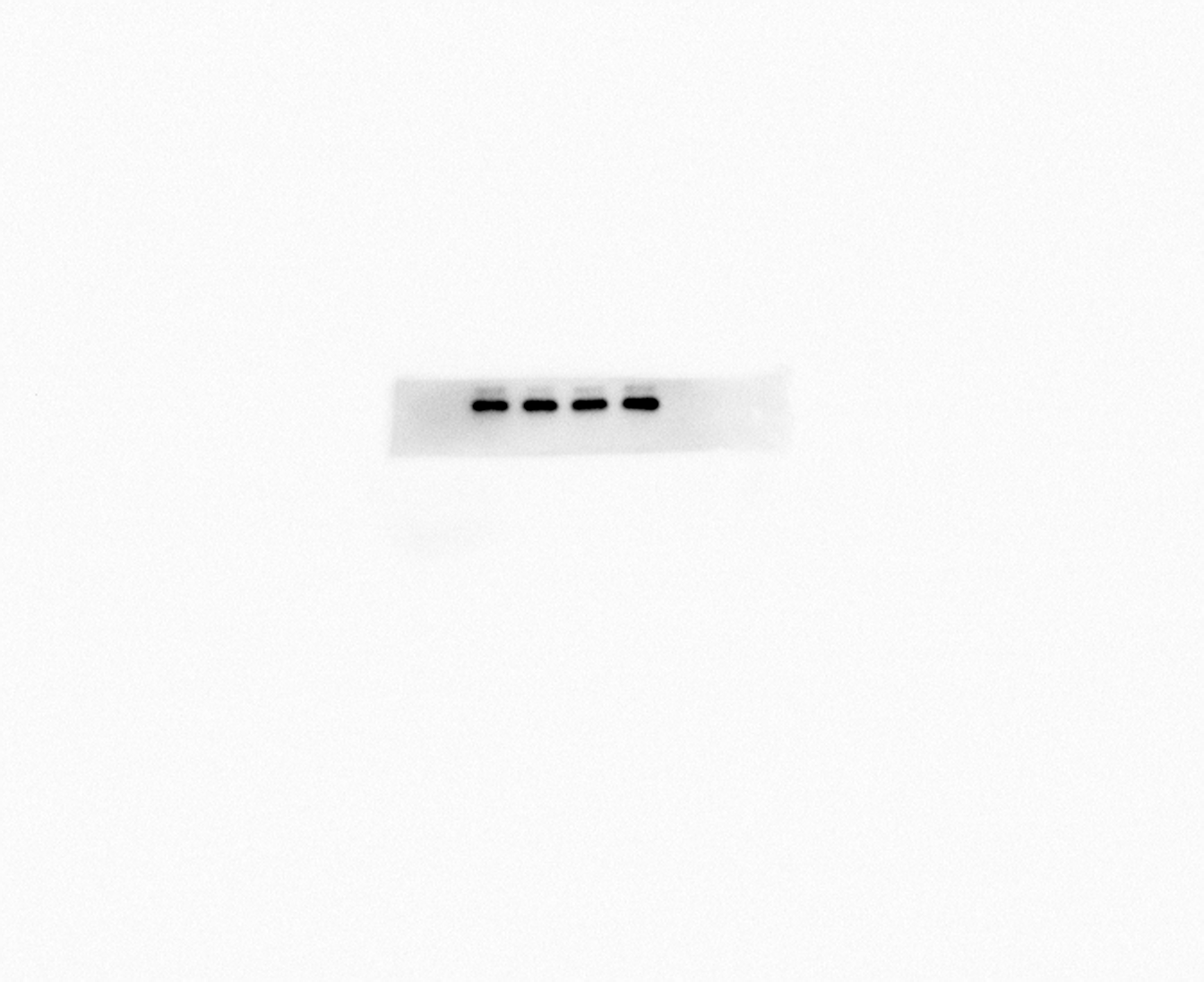

Supplement: Supplementary file 12 — Figure EV4 Source Data [file 44321_2025_315_MOESM12_ESM.zip › Figure EV4/EV4B-WB/3-3-beta-actin.Tif]

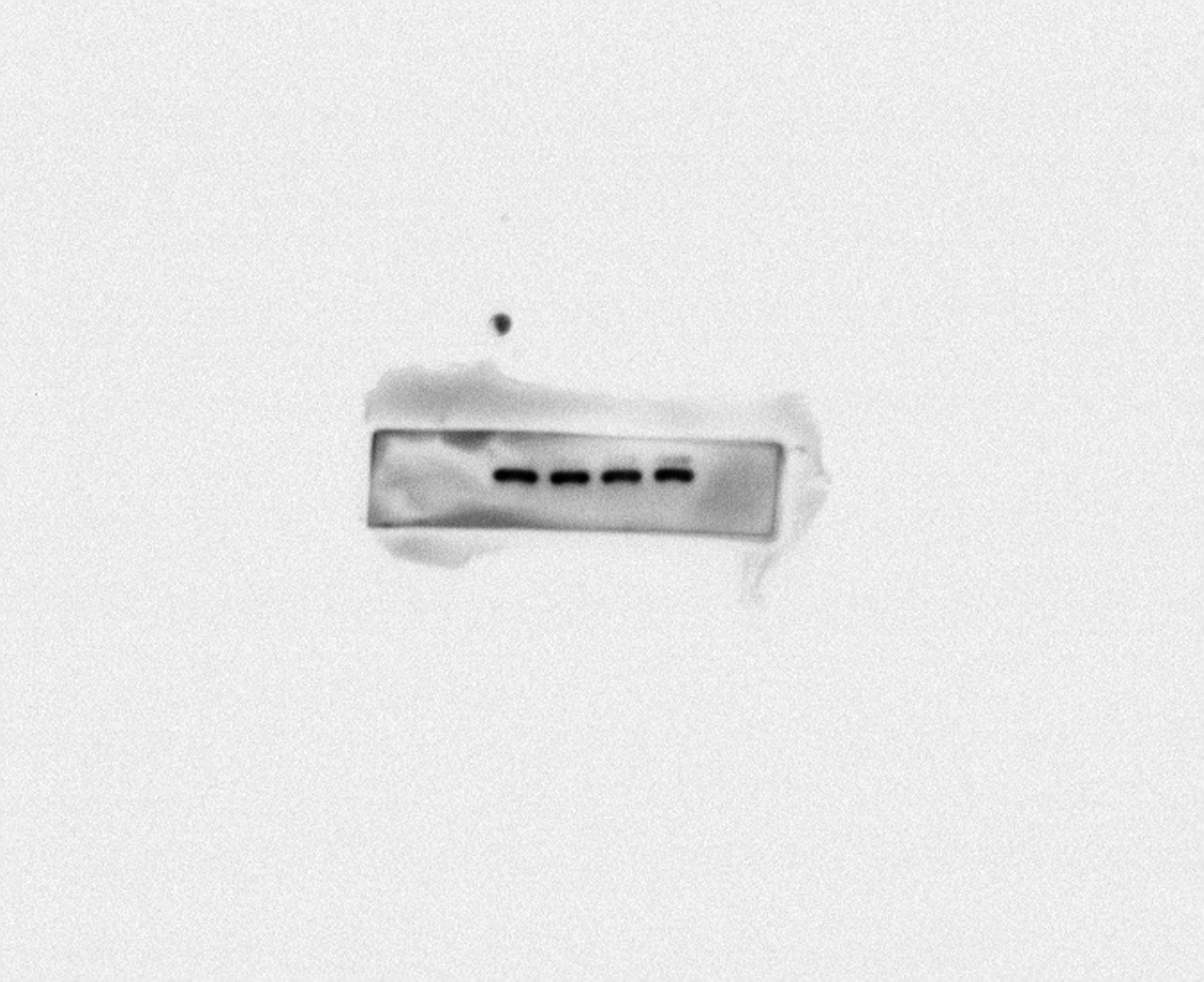

Supplement: Supplementary file 12 — Figure EV4 Source Data [file 44321_2025_315_MOESM12_ESM.zip › Figure EV4/EV4B-WB/3-1-beta-actin.tif]

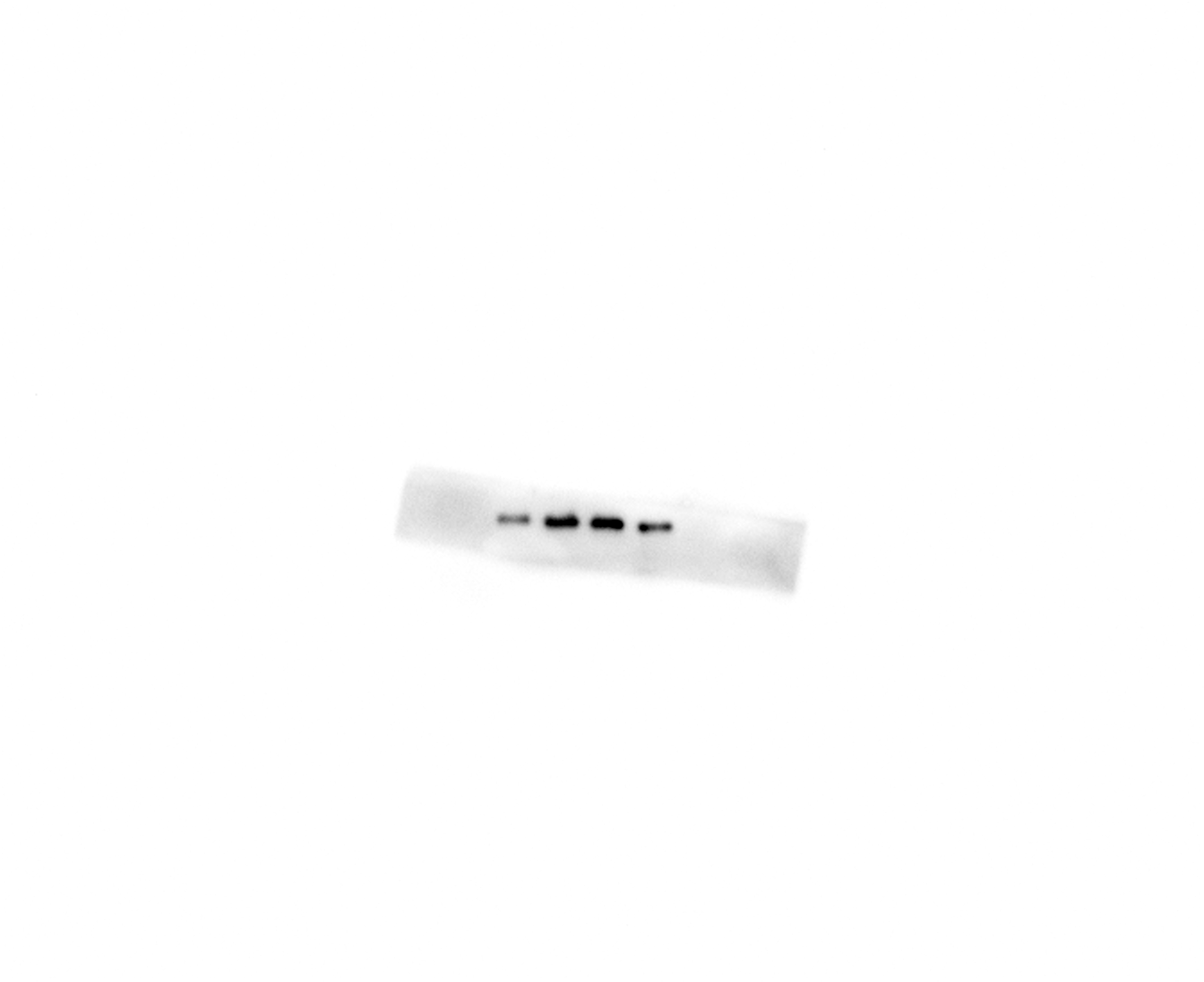

Supplement: Supplementary file 12 — Figure EV4 Source Data [file 44321_2025_315_MOESM12_ESM.zip › Figure EV4/EV4B-WB/2-1-DHODH.Tif]

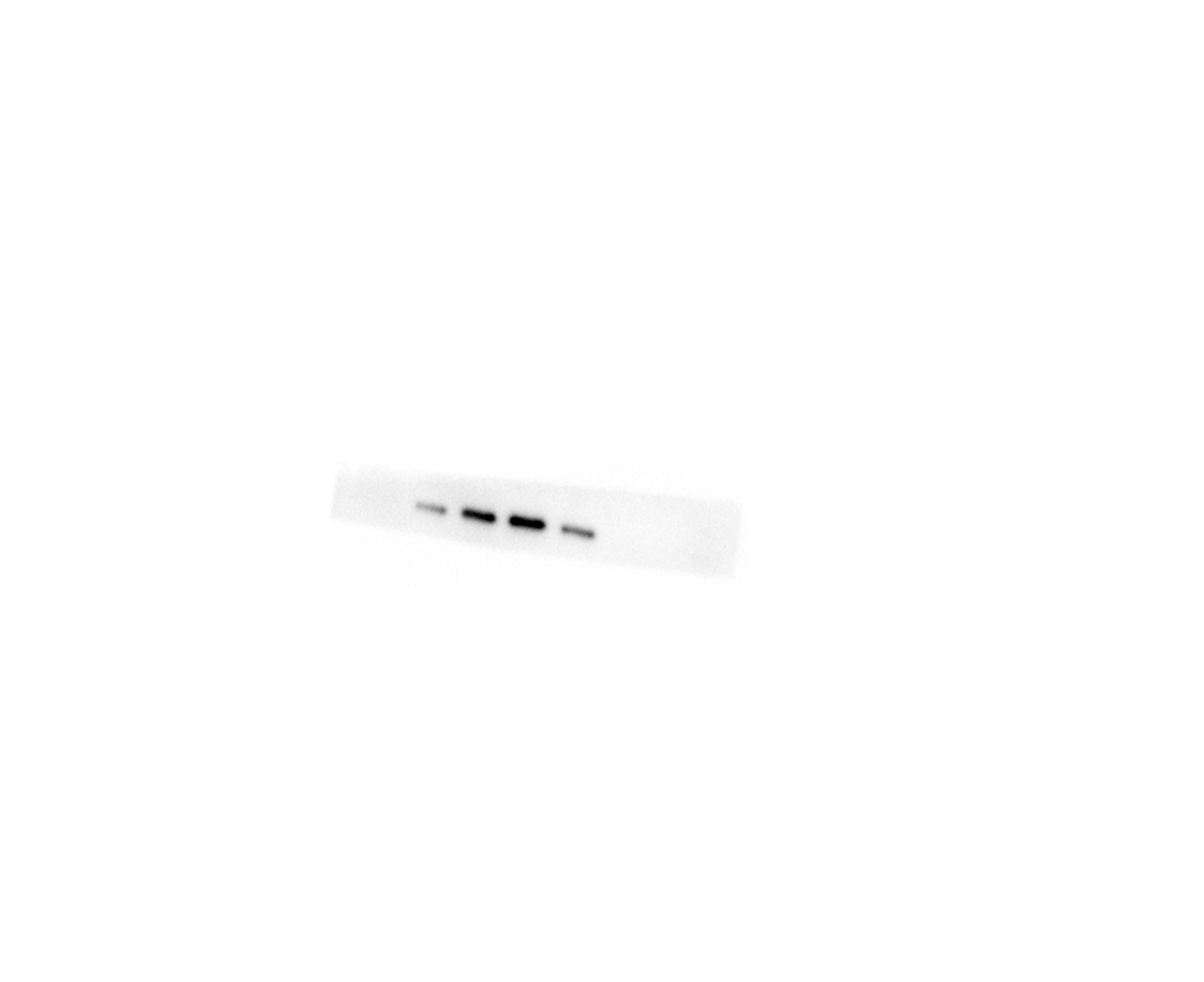

Supplement: Supplementary file 12 — Figure EV4 Source Data [file 44321_2025_315_MOESM12_ESM.zip › Figure EV4/EV4B-WB/1-2-CAD.Tif]

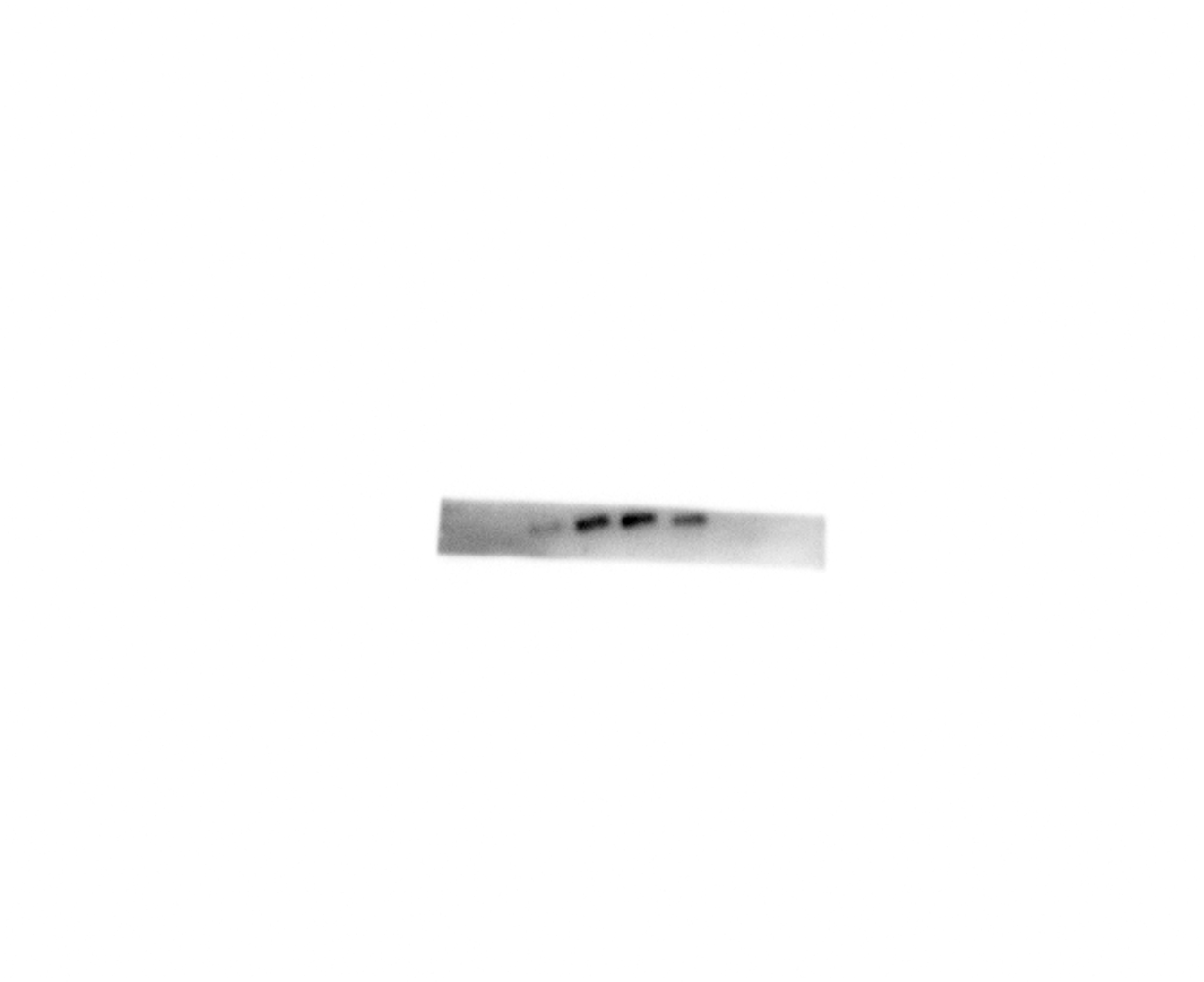

Supplement: Supplementary file 12 — Figure EV4 Source Data [file 44321_2025_315_MOESM12_ESM.zip › Figure EV4/EV4B-WB/2-2-DHODH.tif]

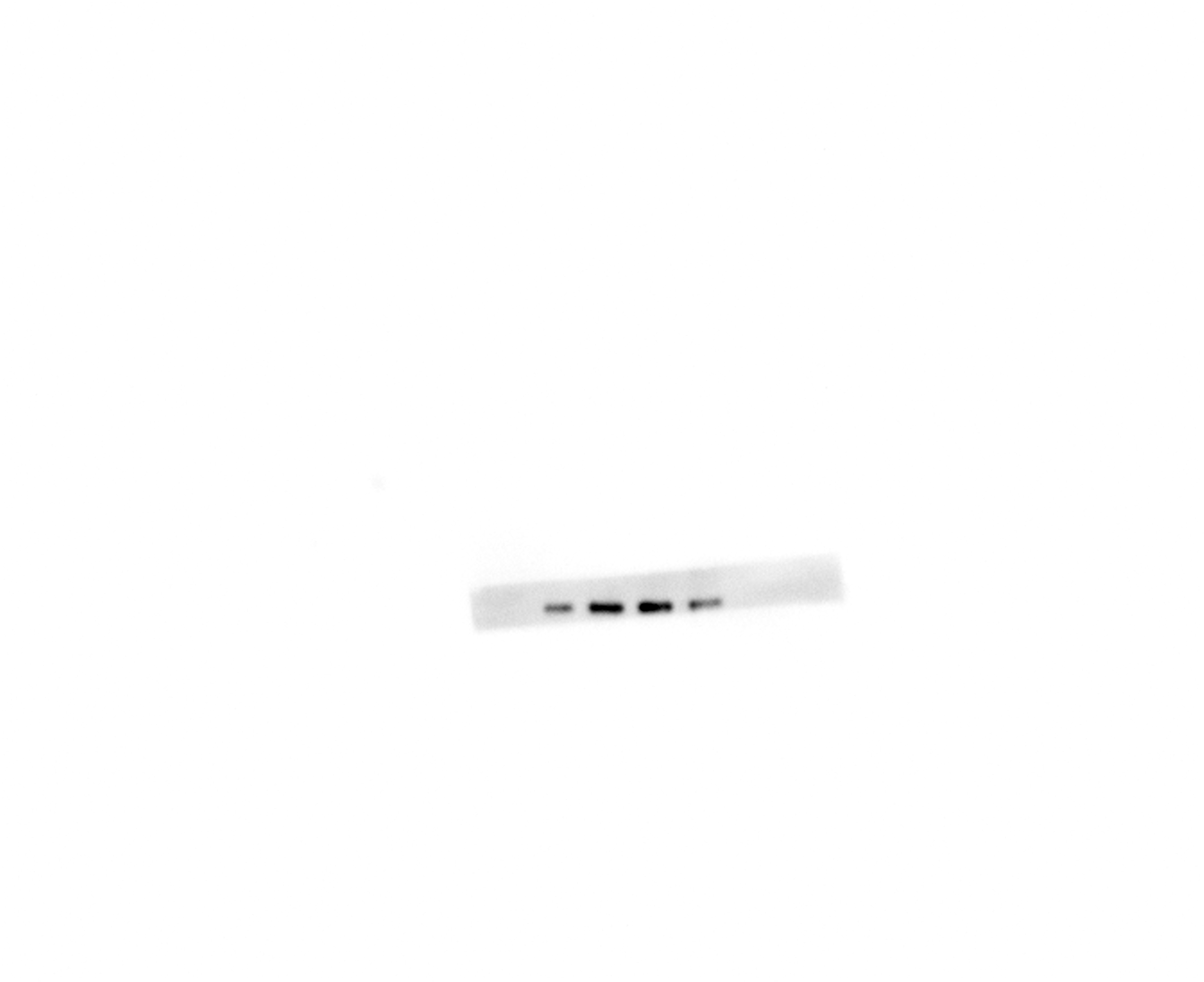

Supplement: Supplementary file 12 — Figure EV4 Source Data [file 44321_2025_315_MOESM12_ESM.zip › Figure EV4/EV4B-WB/1-3-CAD.Tif]

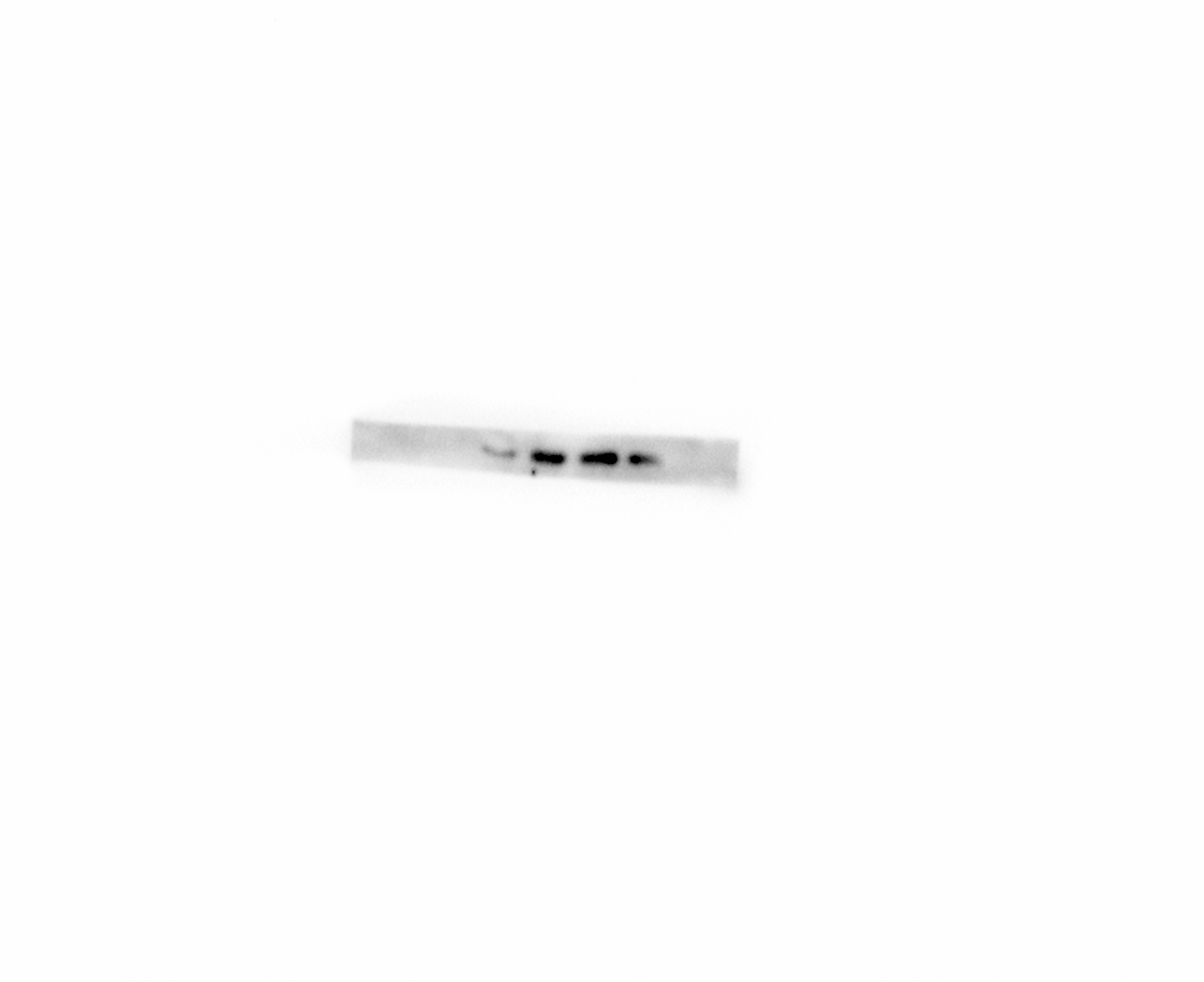

Supplement: Supplementary file 12 — Figure EV4 Source Data [file 44321_2025_315_MOESM12_ESM.zip › Figure EV4/EV4B-WB/2-3-DHODH.tif]

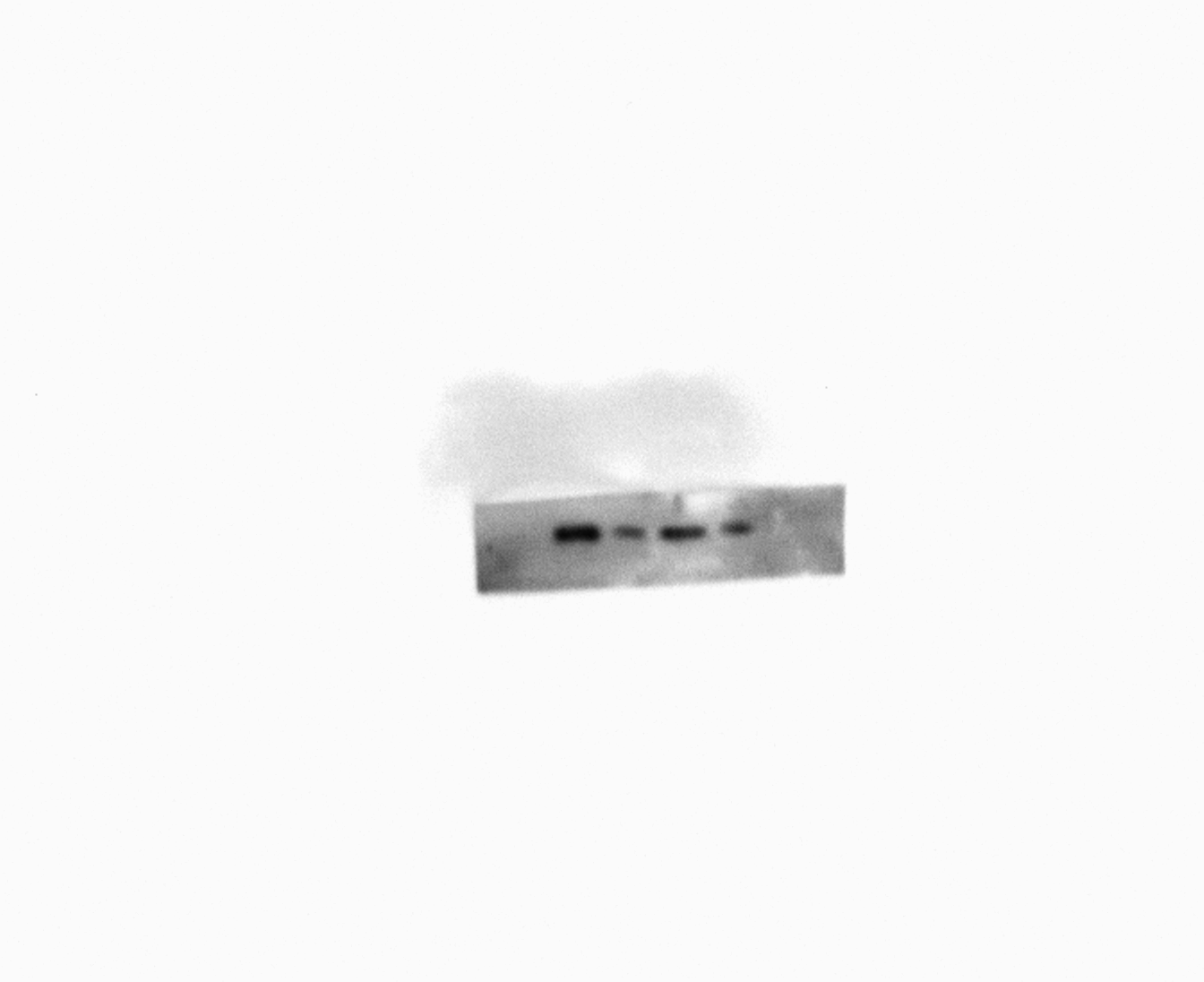

Supplement: Supplementary file 13 — Figure EV5 Source Data [file 44321_2025_315_MOESM13_ESM.zip › Figure EV5/EV5G-WB/2-3-cyclin D1.Tif]

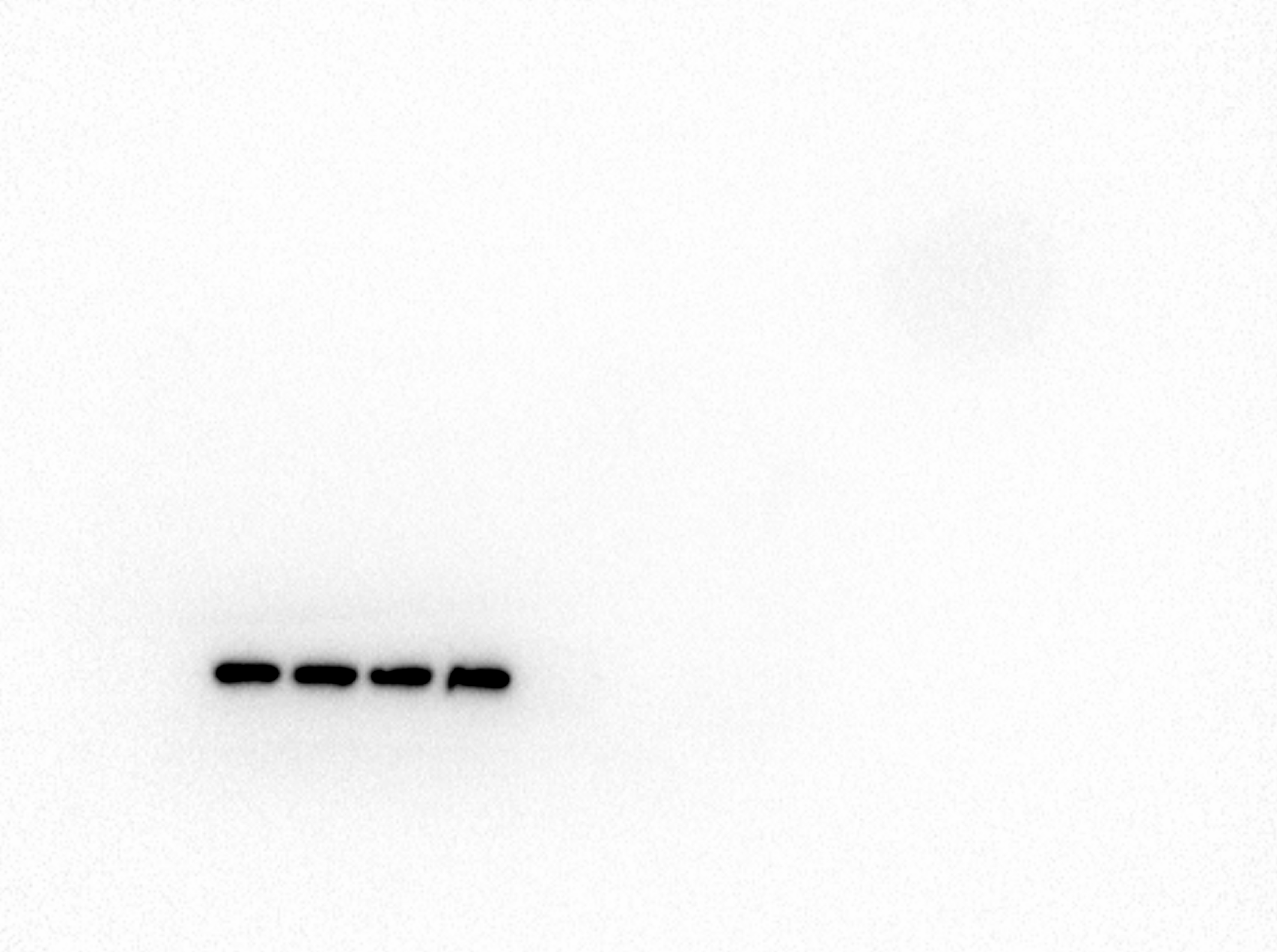

Supplement: Supplementary file 13 — Figure EV5 Source Data [file 44321_2025_315_MOESM13_ESM.zip › Figure EV5/EV5G-WB/3-2-beta-actin.tif]

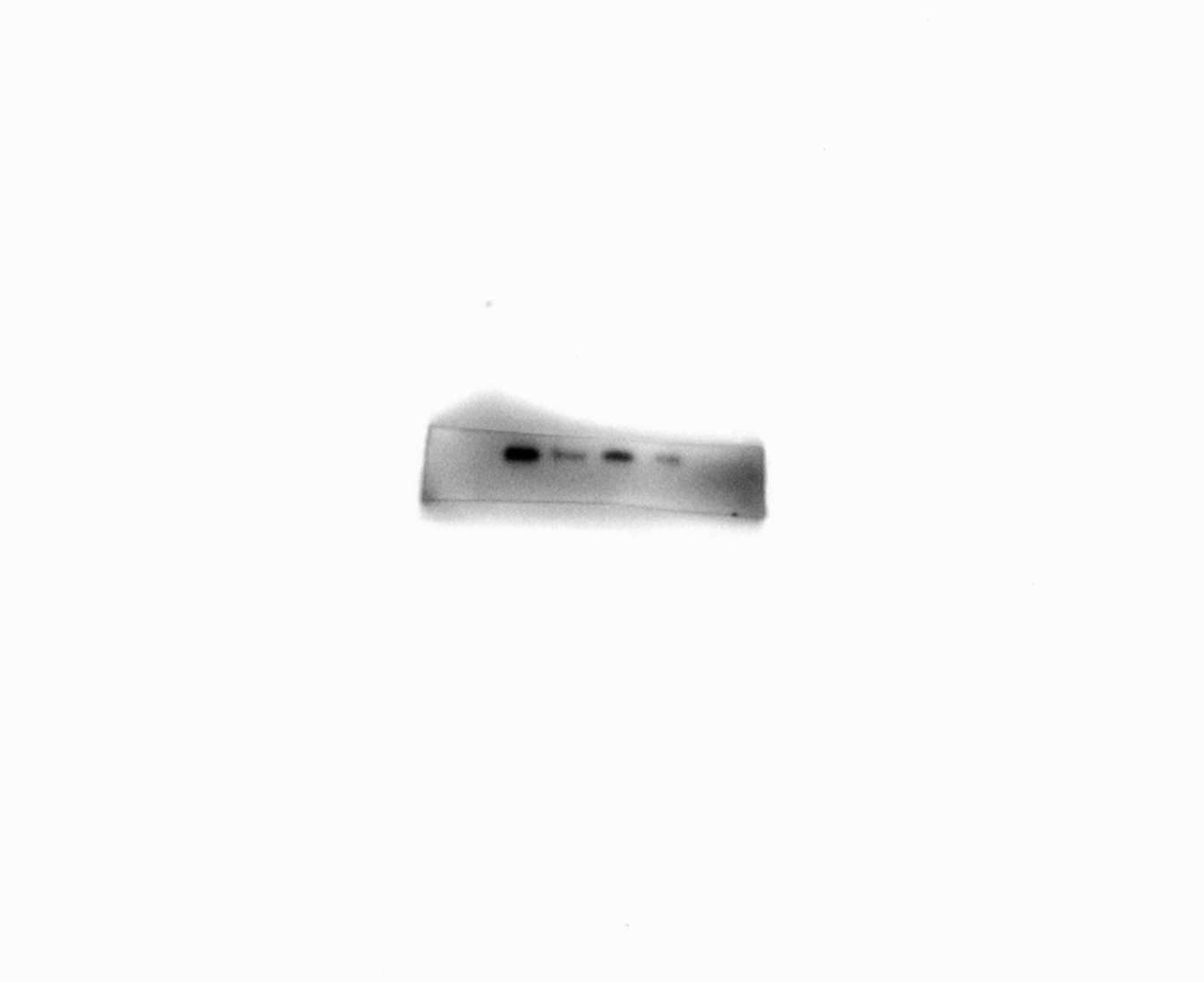

Supplement: Supplementary file 13 — Figure EV5 Source Data [file 44321_2025_315_MOESM13_ESM.zip › Figure EV5/EV5G-WB/1-1-PCNA.Tif]

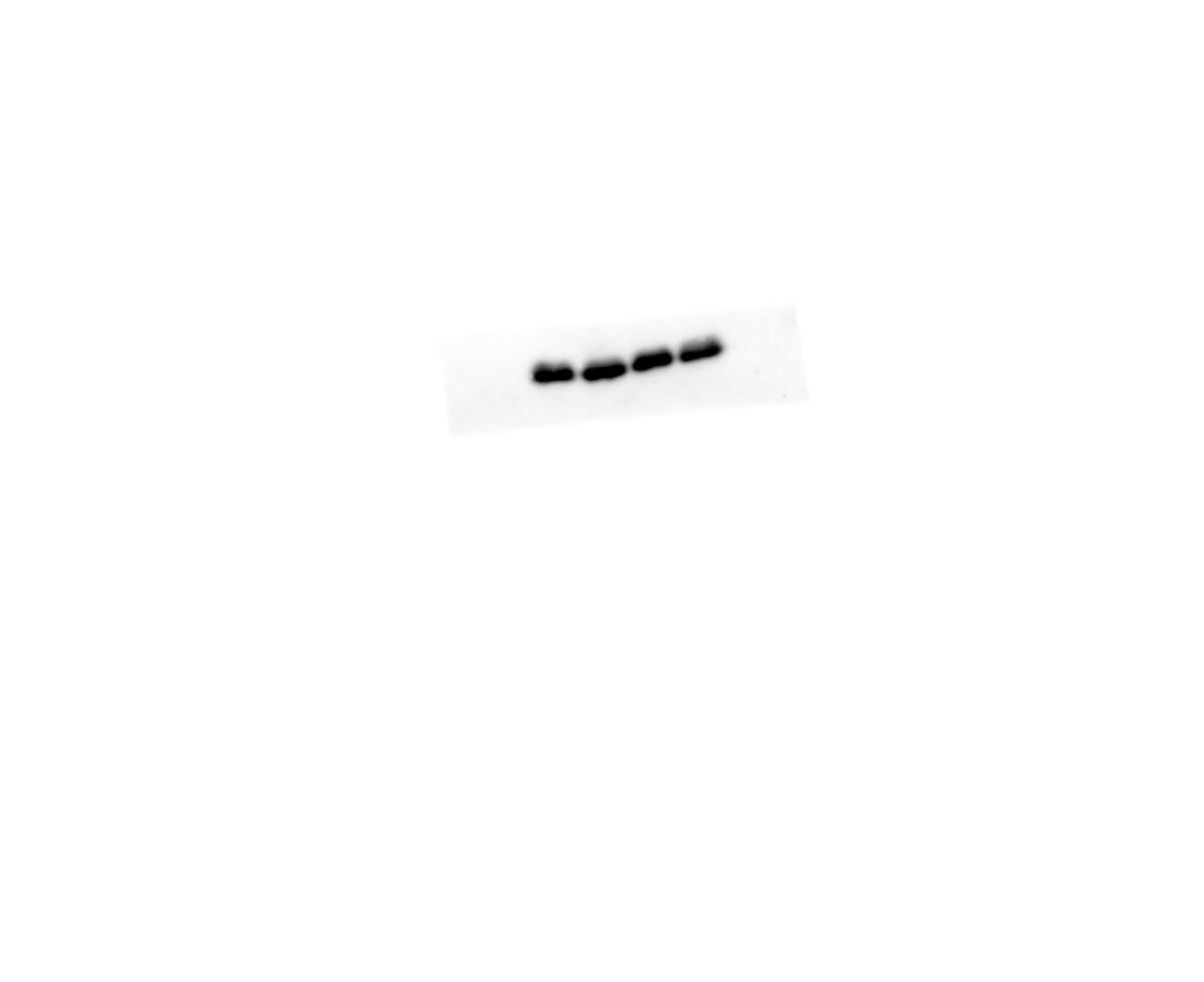

Supplement: Supplementary file 13 — Figure EV5 Source Data [file 44321_2025_315_MOESM13_ESM.zip › Figure EV5/EV5G-WB/3-3-beta-actin.Tif]

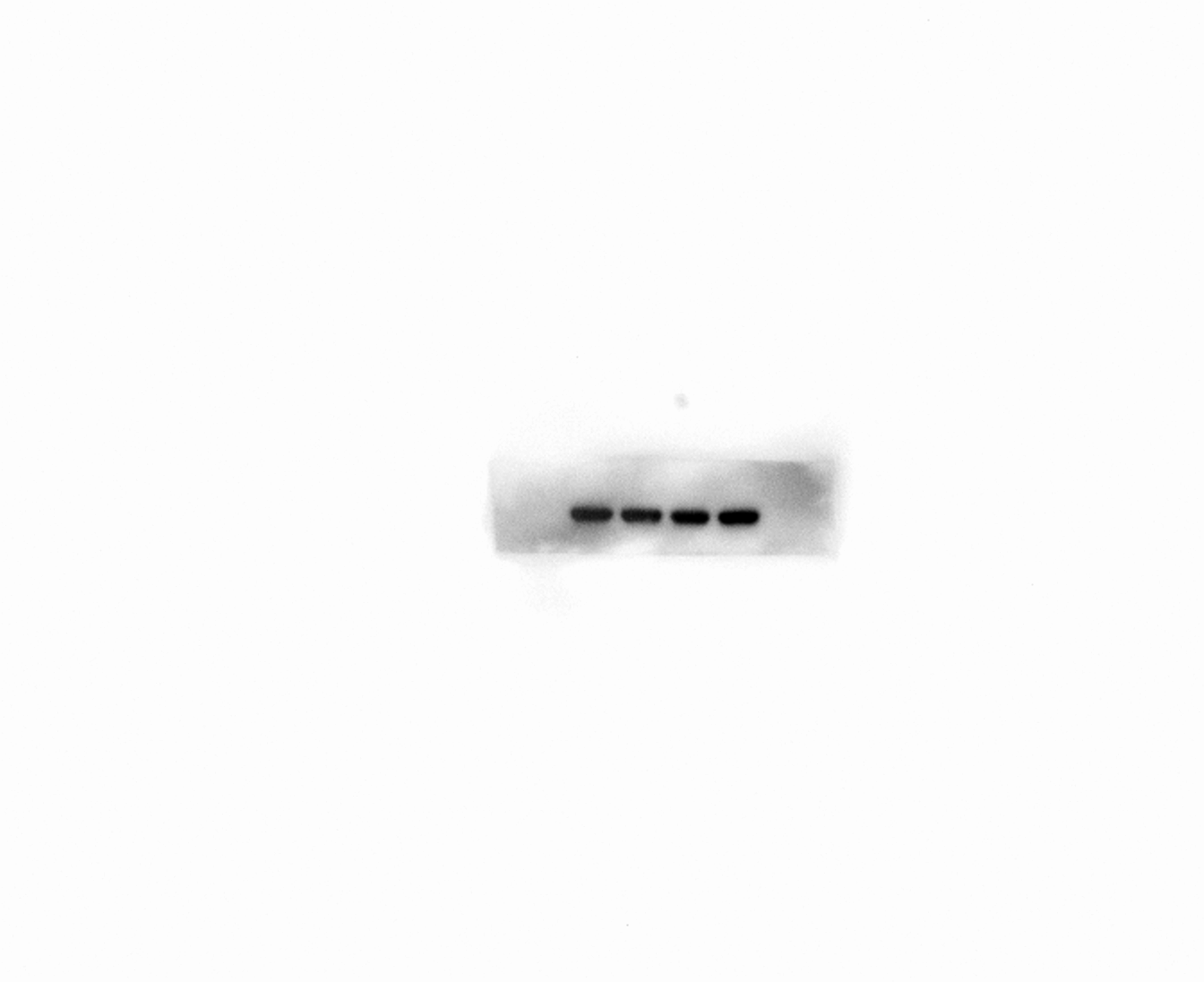

Supplement: Supplementary file 13 — Figure EV5 Source Data [file 44321_2025_315_MOESM13_ESM.zip › Figure EV5/EV5G-WB/3-1-beta-actin.Tif]

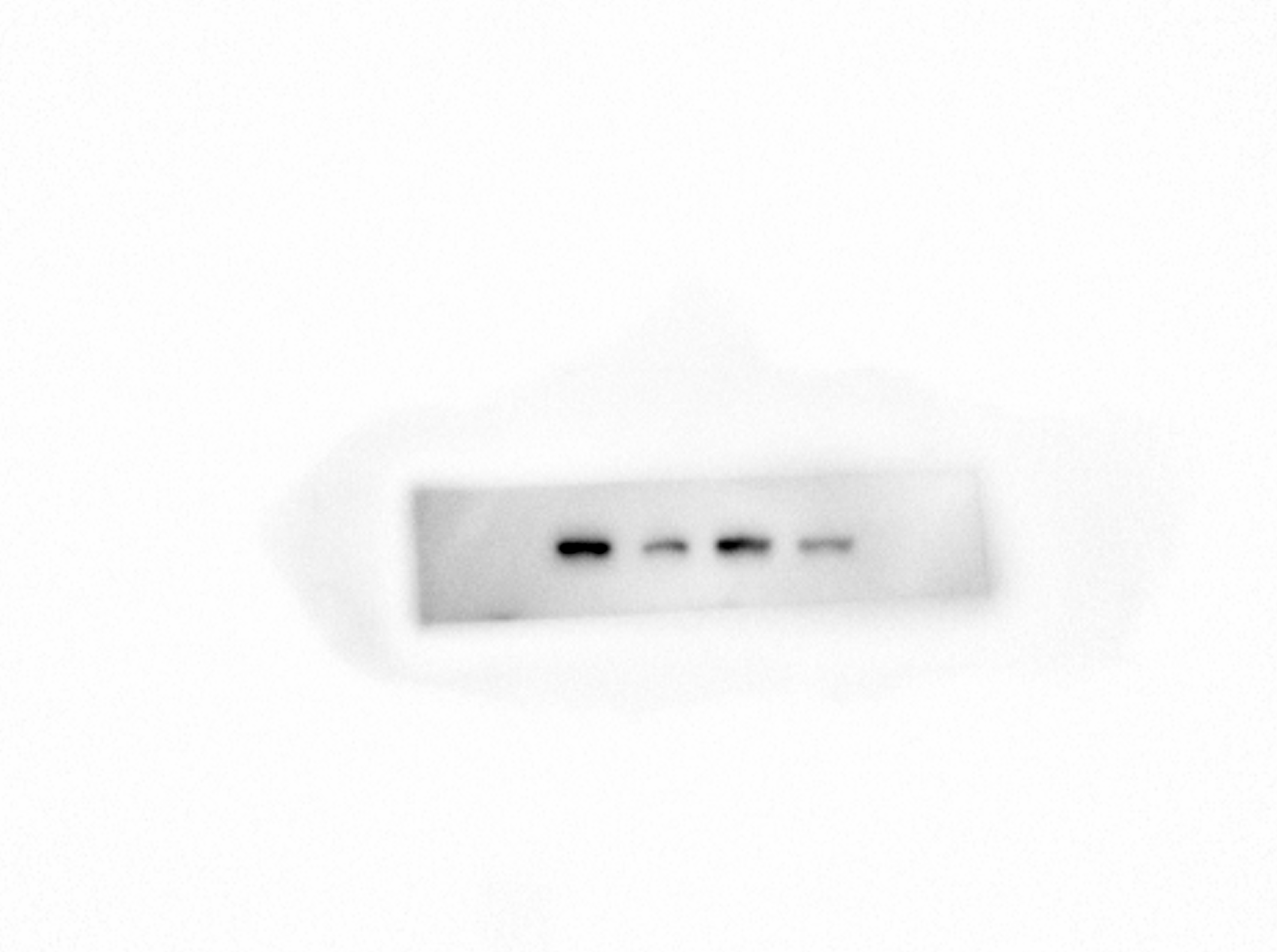

Supplement: Supplementary file 13 — Figure EV5 Source Data [file 44321_2025_315_MOESM13_ESM.zip › Figure EV5/EV5G-WB/1-2-PCNA.tif]

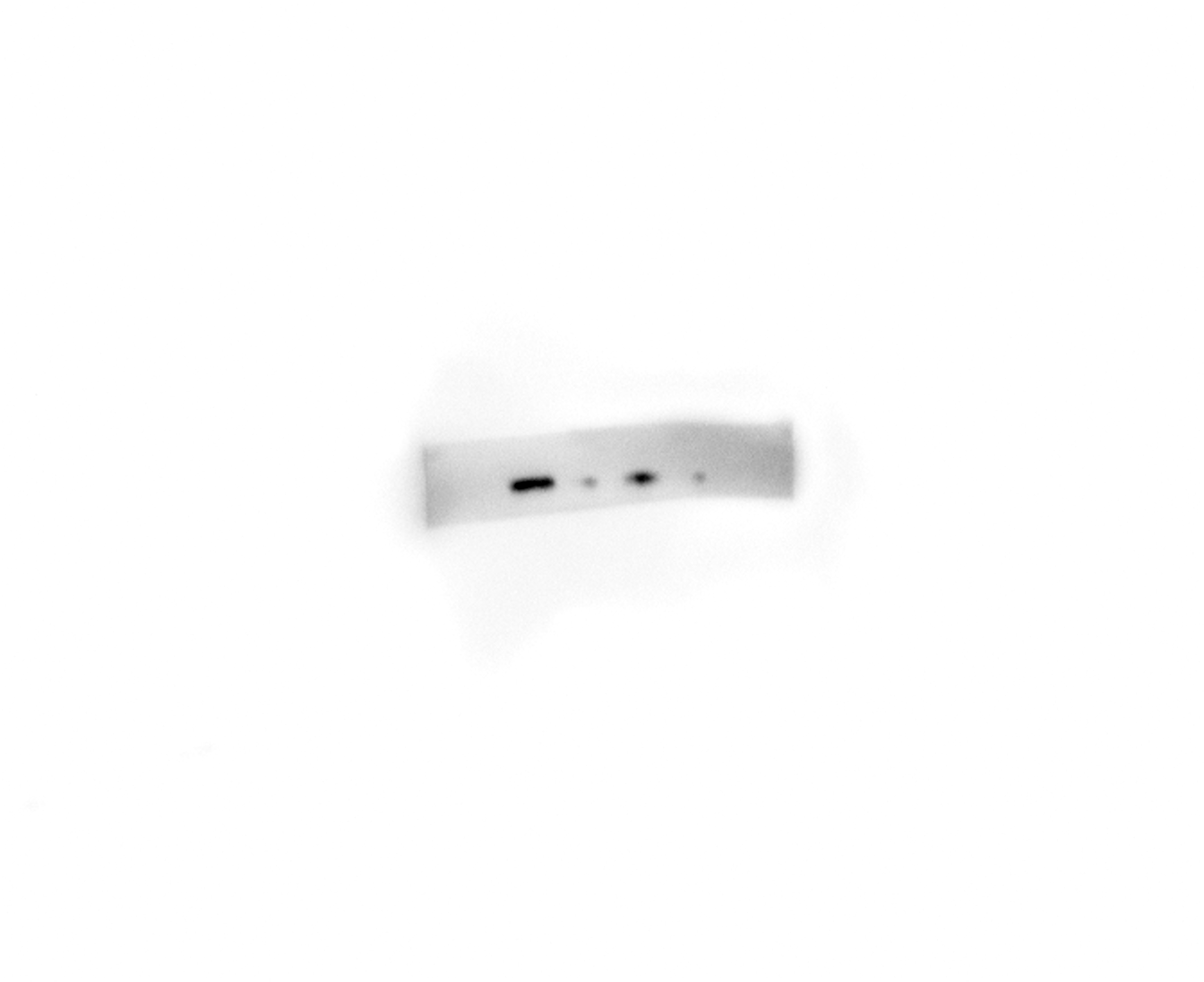

Supplement: Supplementary file 13 — Figure EV5 Source Data [file 44321_2025_315_MOESM13_ESM.zip › Figure EV5/EV5G-WB/1-3-PCNA.Tif]

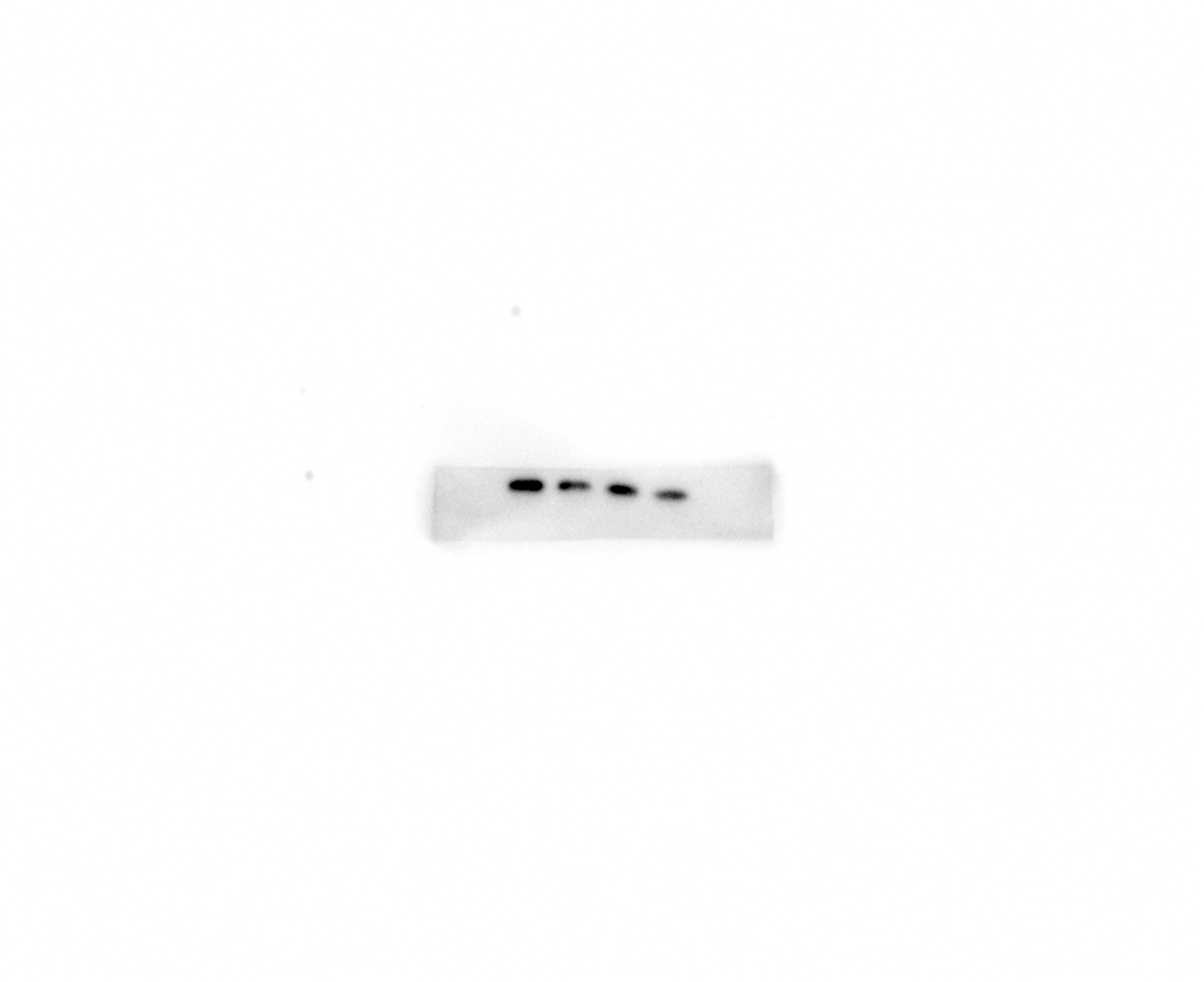

Supplement: Supplementary file 13 — Figure EV5 Source Data [file 44321_2025_315_MOESM13_ESM.zip › Figure EV5/EV5G-WB/2-1-cyclin D1.Tif]

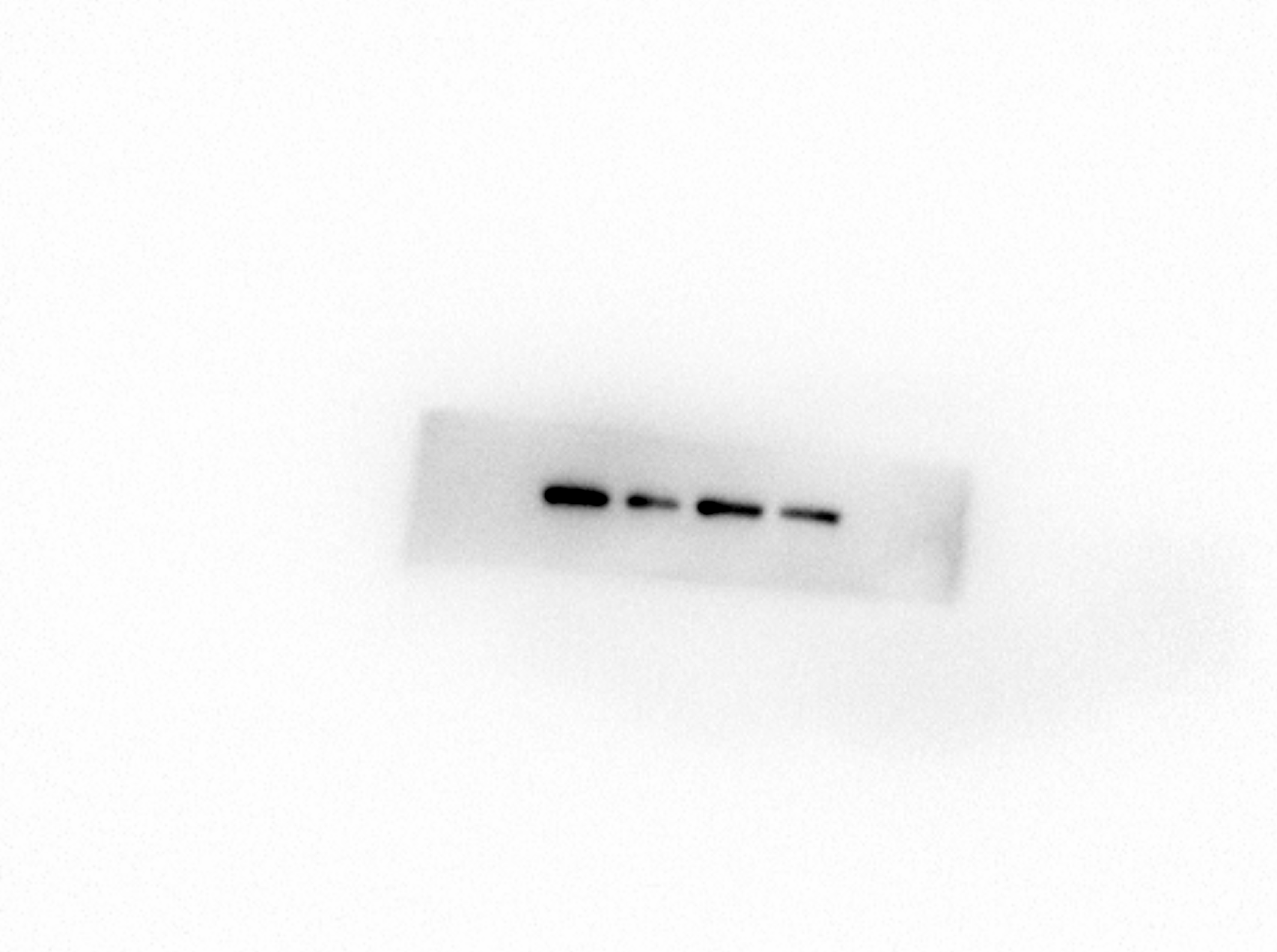

Supplement: Supplementary file 13 — Figure EV5 Source Data [file 44321_2025_315_MOESM13_ESM.zip › Figure EV5/EV5G-WB/2-2-Cyclin D1.tif]

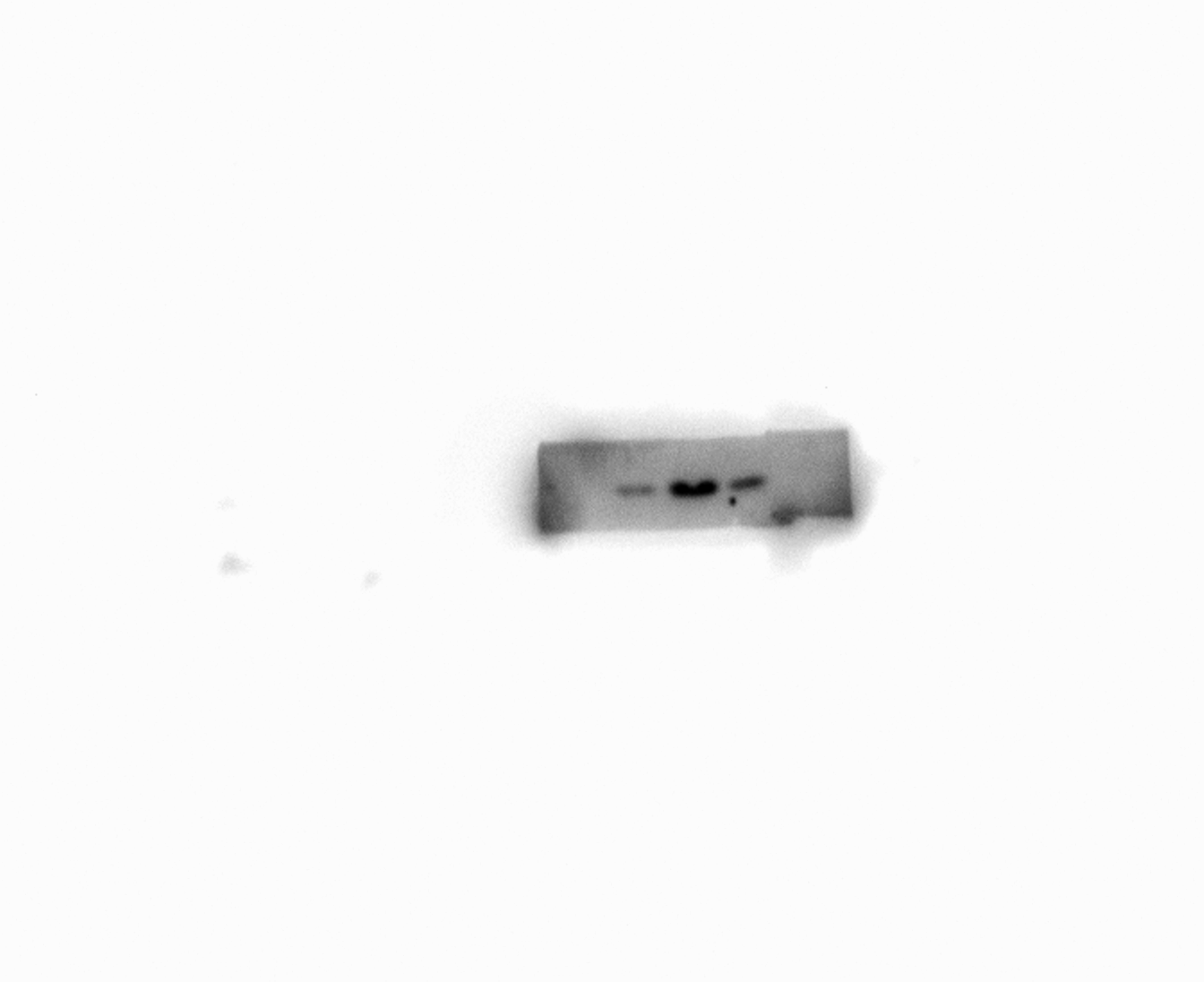

Supplement: Supplementary file 13 — Figure EV5 Source Data [file 44321_2025_315_MOESM13_ESM.zip › Figure EV5/EV5C-WB/2-3-cyclin D1.Tif]

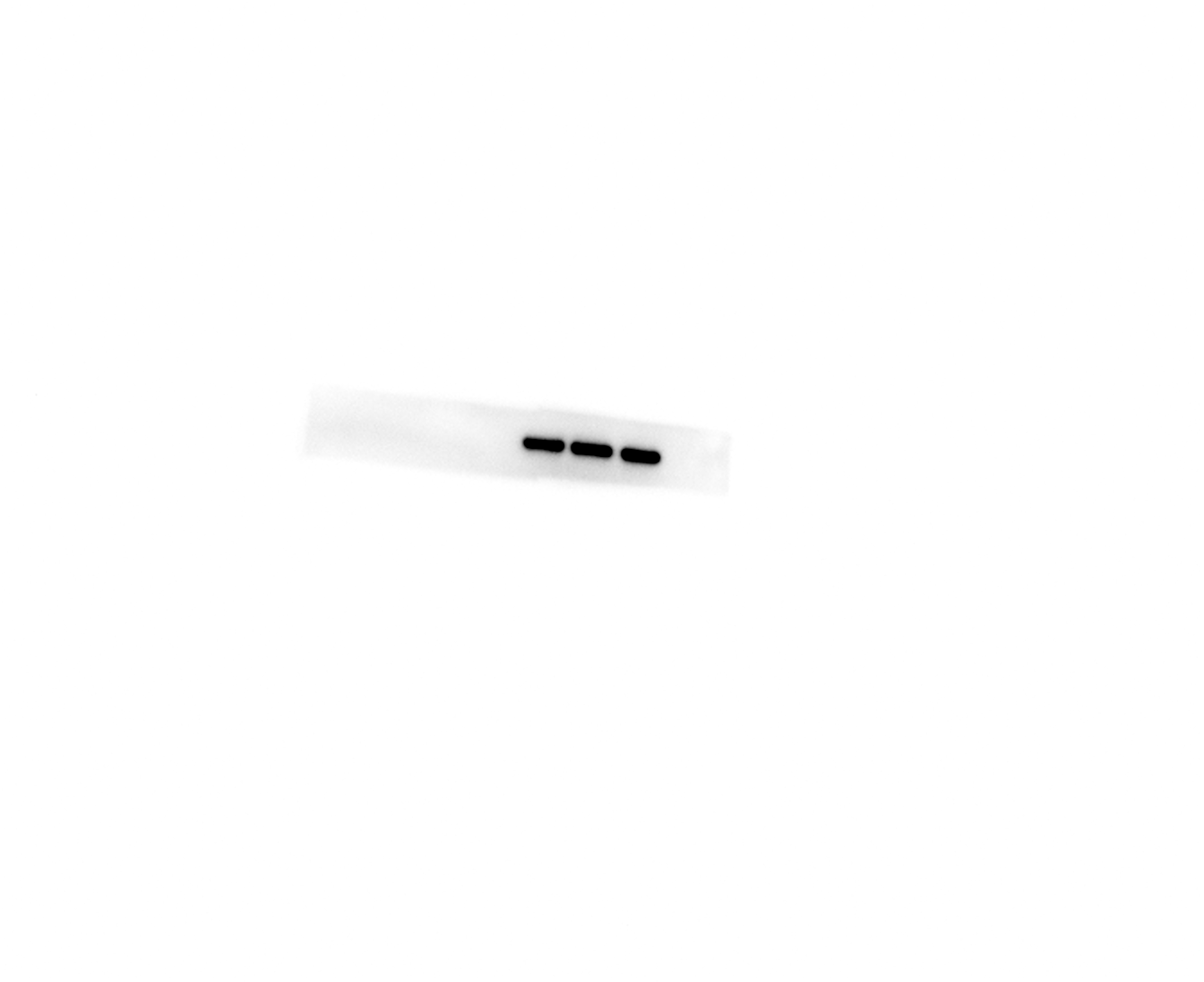

Supplement: Supplementary file 13 — Figure EV5 Source Data [file 44321_2025_315_MOESM13_ESM.zip › Figure EV5/EV5C-WB/3-2-beta-actin.Tif]

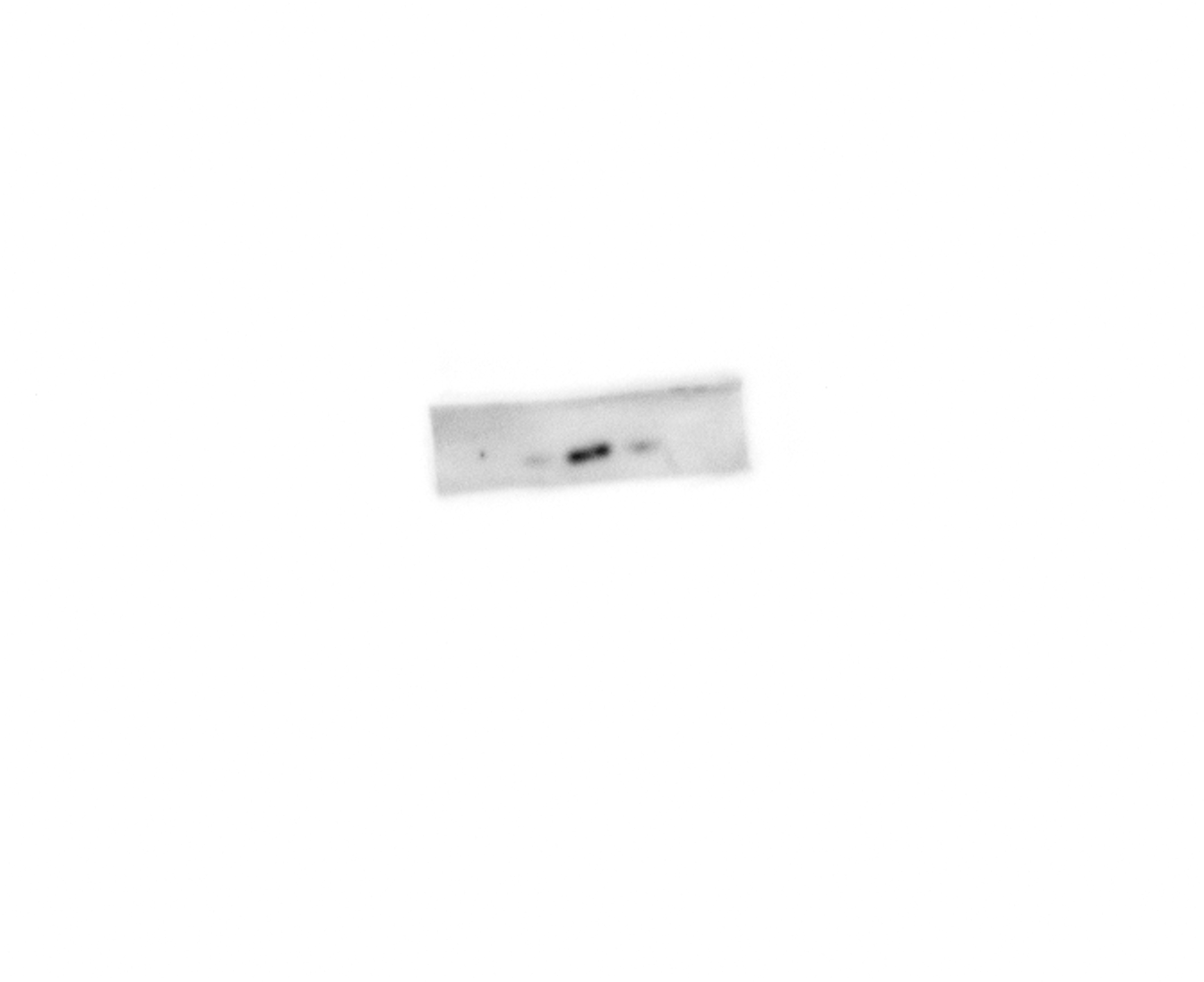

Supplement: Supplementary file 13 — Figure EV5 Source Data [file 44321_2025_315_MOESM13_ESM.zip › Figure EV5/EV5C-WB/1-1-PCNA.Tif]

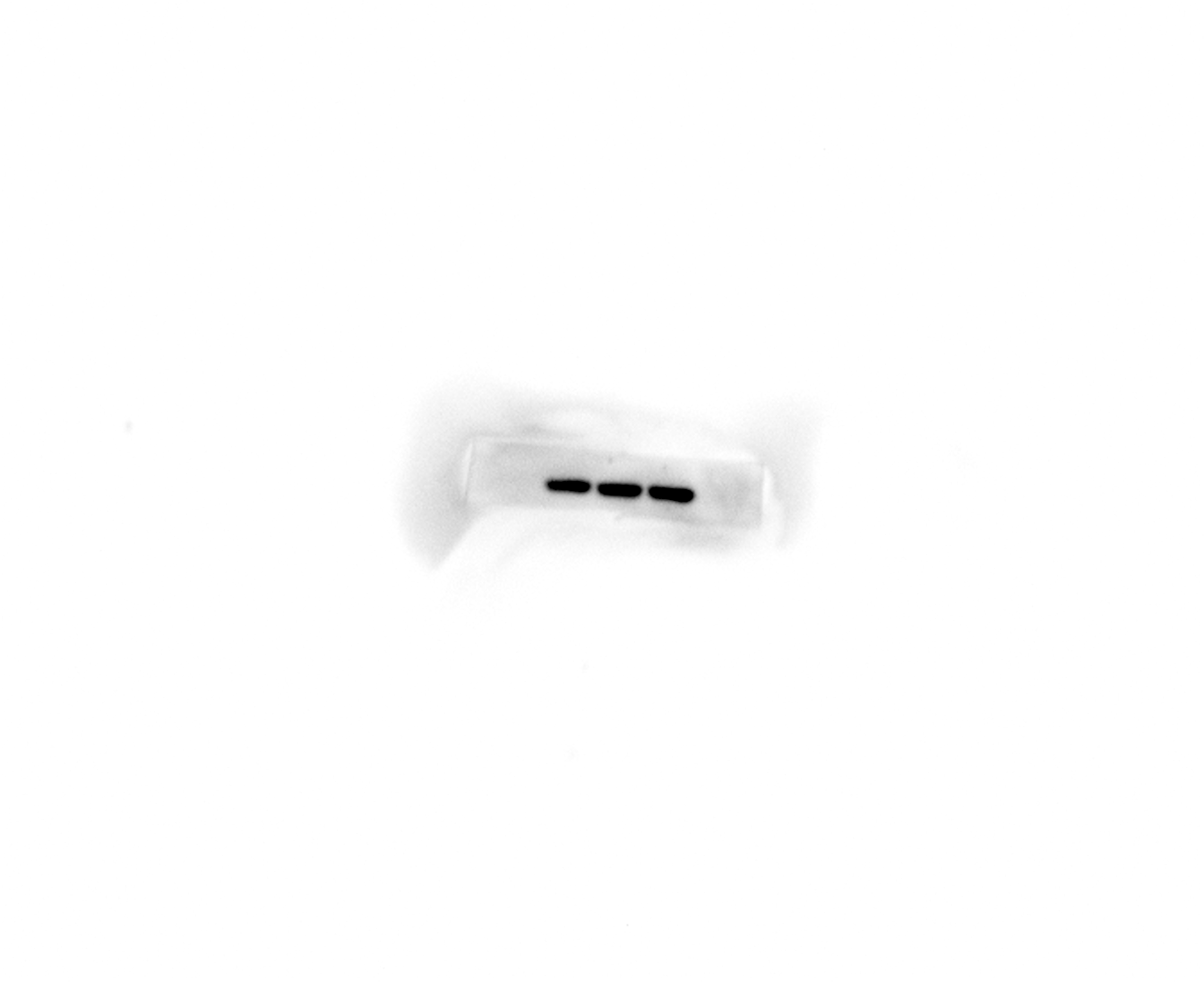

Supplement: Supplementary file 13 — Figure EV5 Source Data [file 44321_2025_315_MOESM13_ESM.zip › Figure EV5/EV5C-WB/3-3-beta-actin.Tif]

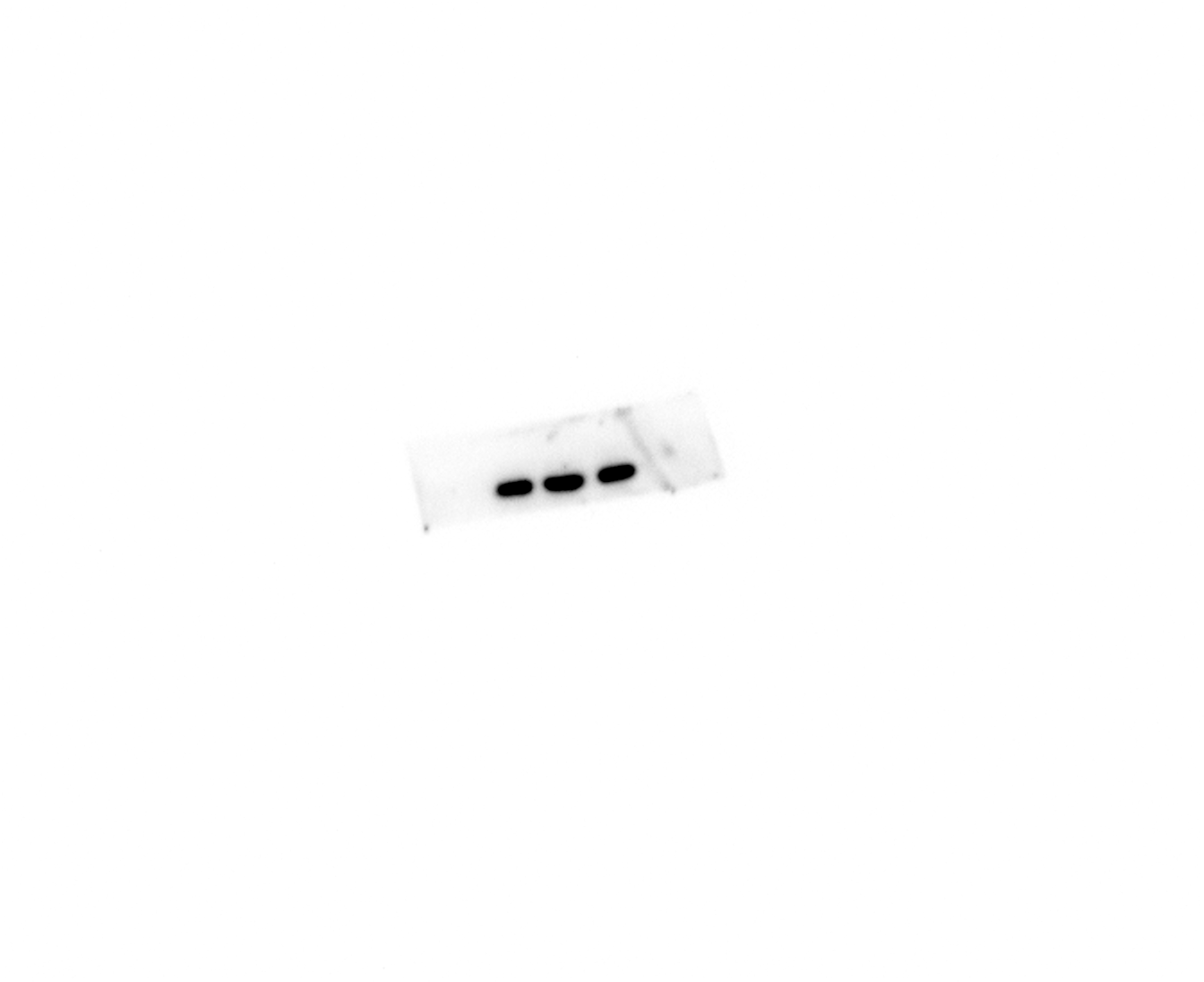

Supplement: Supplementary file 13 — Figure EV5 Source Data [file 44321_2025_315_MOESM13_ESM.zip › Figure EV5/EV5C-WB/3-1-beta-actin.Tif]

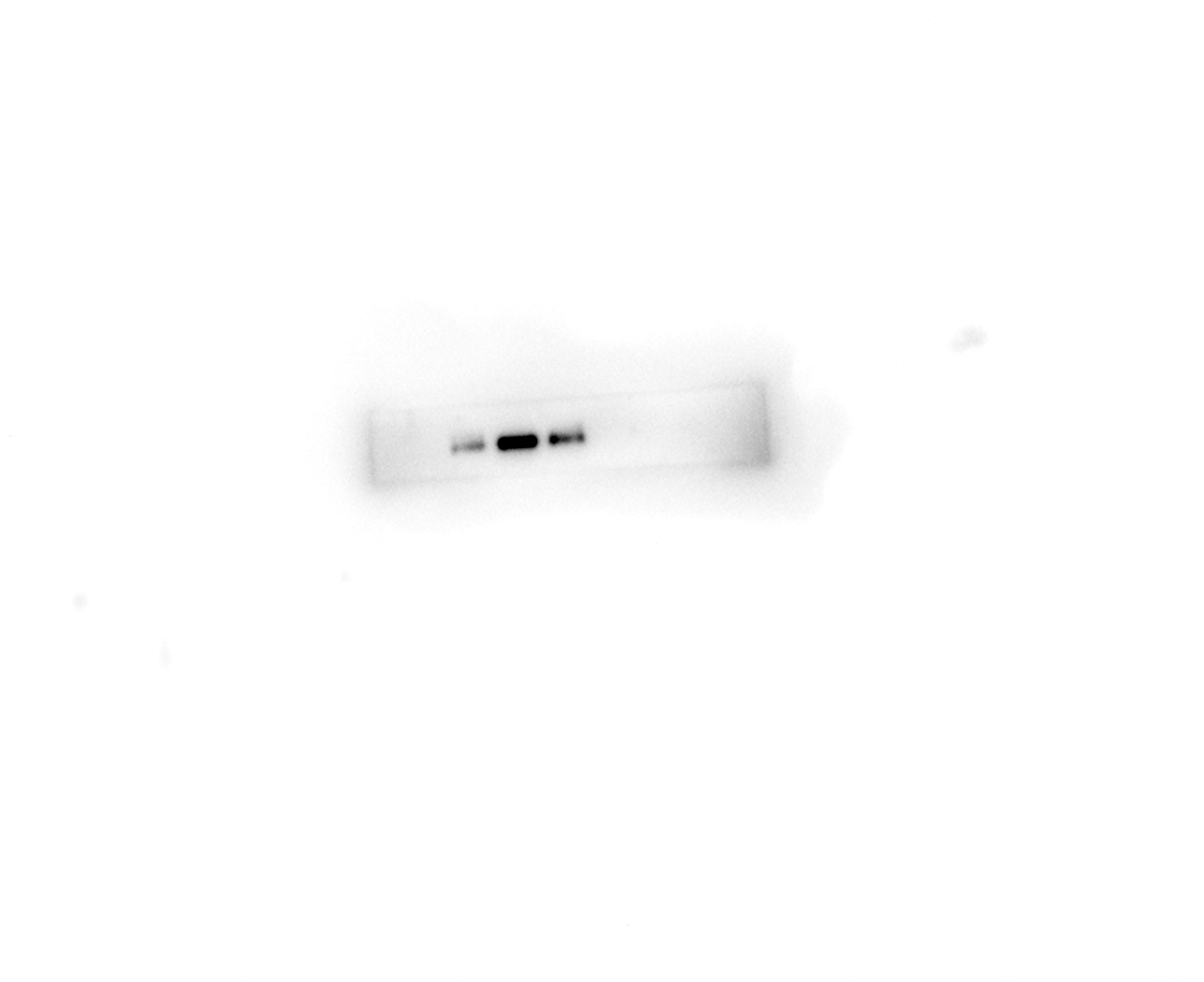

Supplement: Supplementary file 13 — Figure EV5 Source Data [file 44321_2025_315_MOESM13_ESM.zip › Figure EV5/EV5C-WB/1-2-PCNA.Tif]

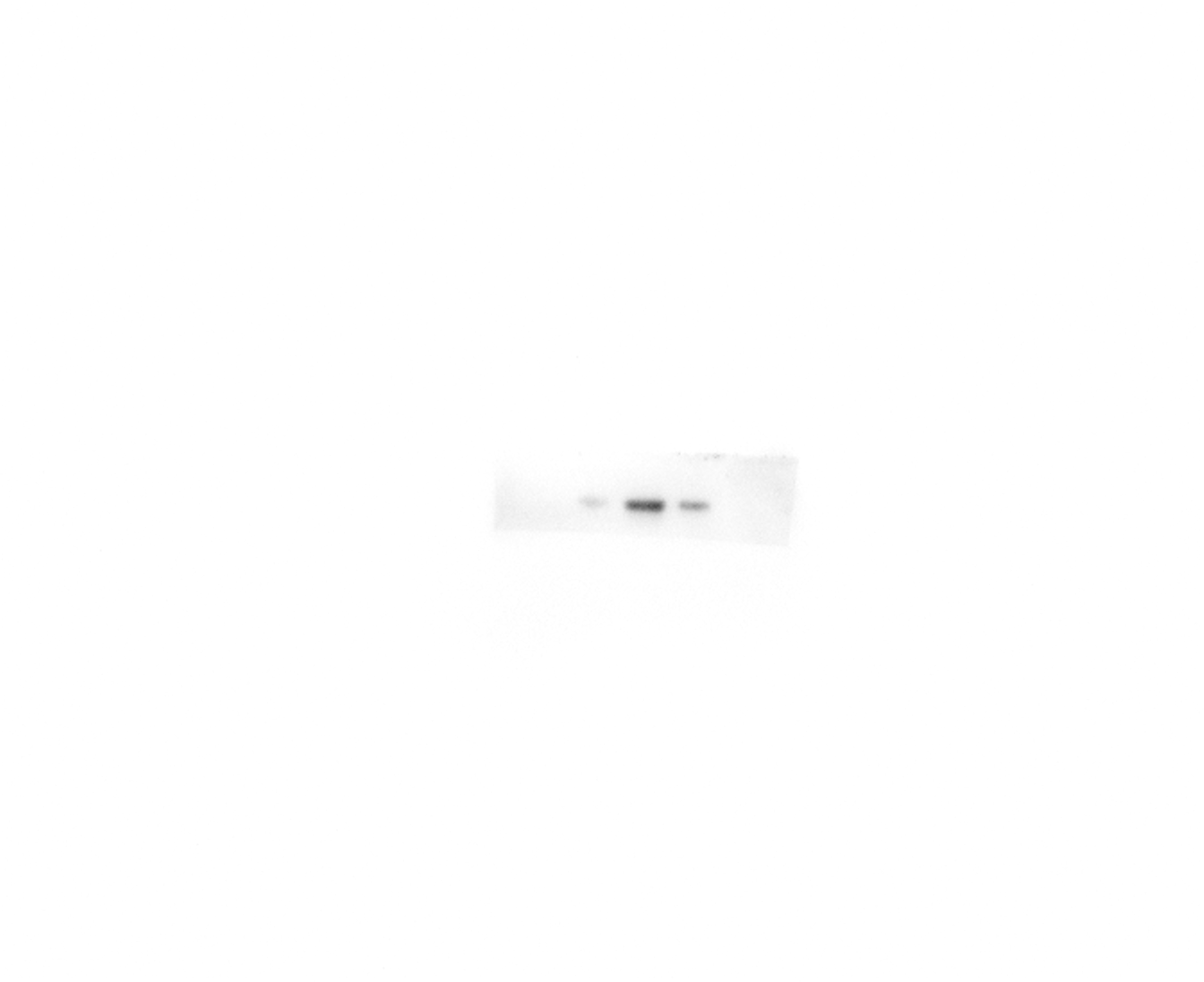

Supplement: Supplementary file 13 — Figure EV5 Source Data [file 44321_2025_315_MOESM13_ESM.zip › Figure EV5/EV5C-WB/1-3-PCNA.Tif]

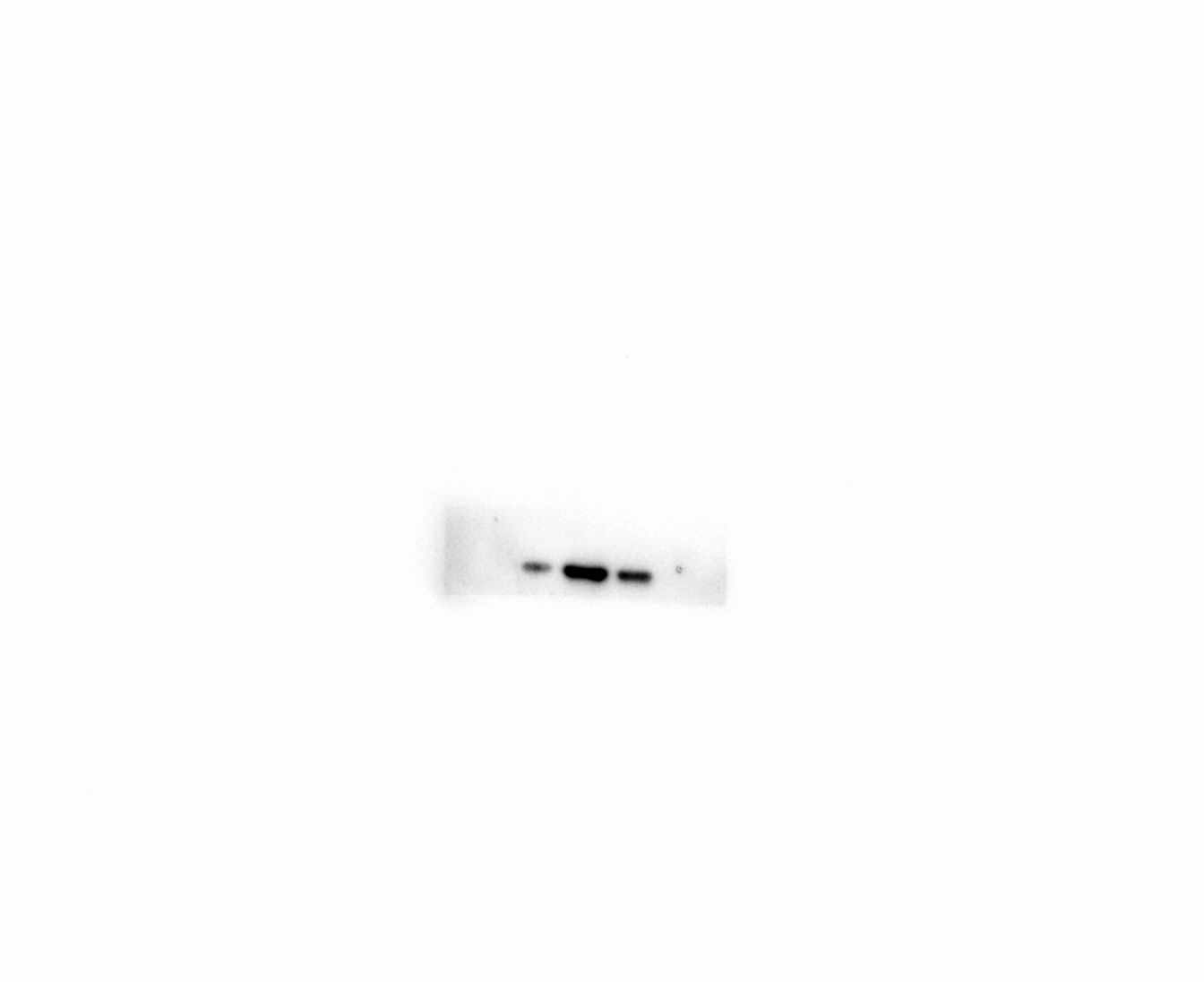

Supplement: Supplementary file 13 — Figure EV5 Source Data [file 44321_2025_315_MOESM13_ESM.zip › Figure EV5/EV5C-WB/2-1-cyclin D1.Tif]

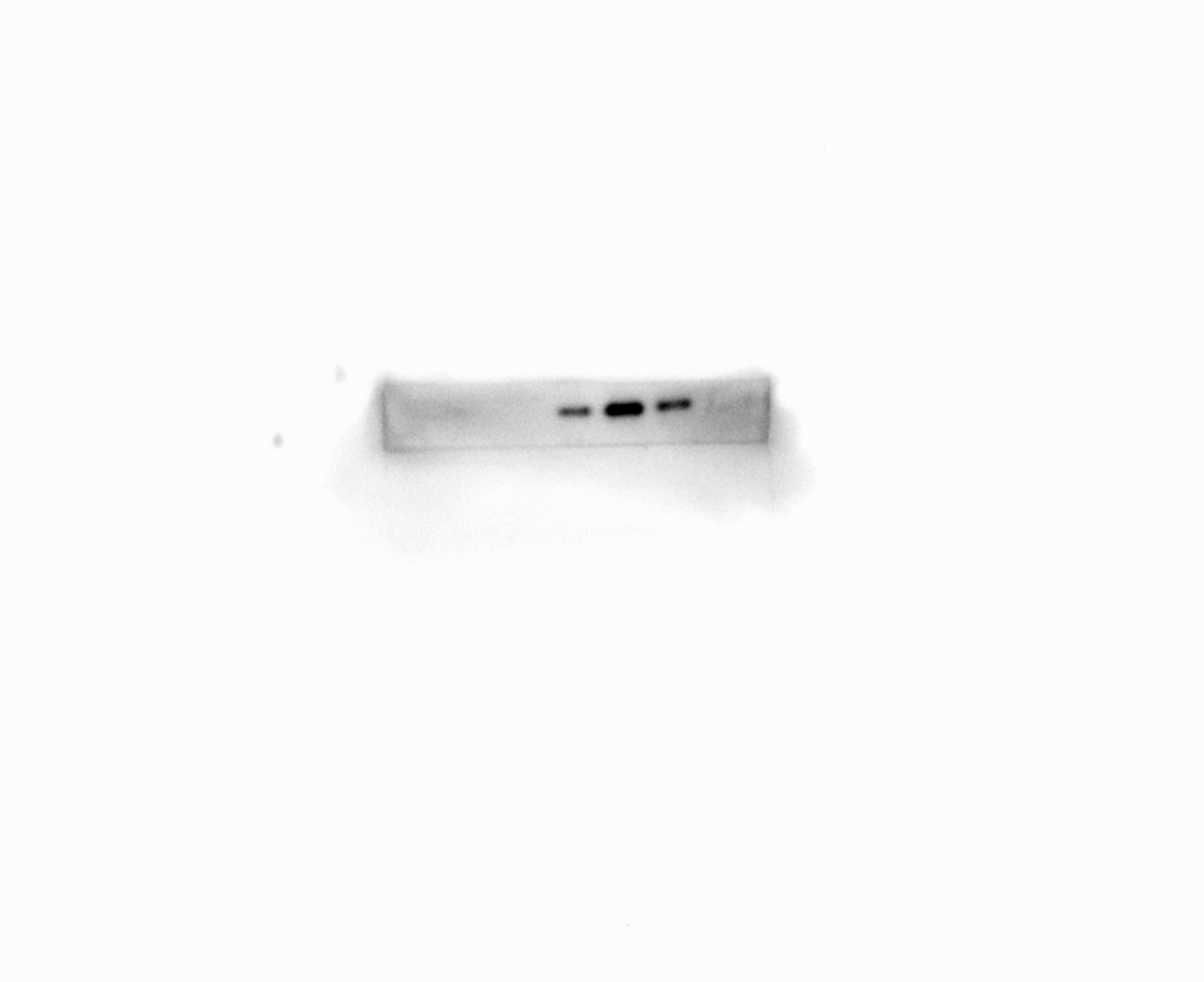

Supplement: Supplementary file 13 — Figure EV5 Source Data [file 44321_2025_315_MOESM13_ESM.zip › Figure EV5/EV5C-WB/2-2-cyclin D1.Tif]
