# Supplementary figures and images for: Glycine decarboxylase advances IgA nephropathy by boosting mesangial cell proliferation through the pyrimidine pathway (part 6 of 7)
Source: EMBO Mol Med. 2025 Oct 13;17(11):3039–63. doi: 10.1038/s44321-025-00315-2 (PMC12603144; doi:10.1038/s44321-025-00315-2)

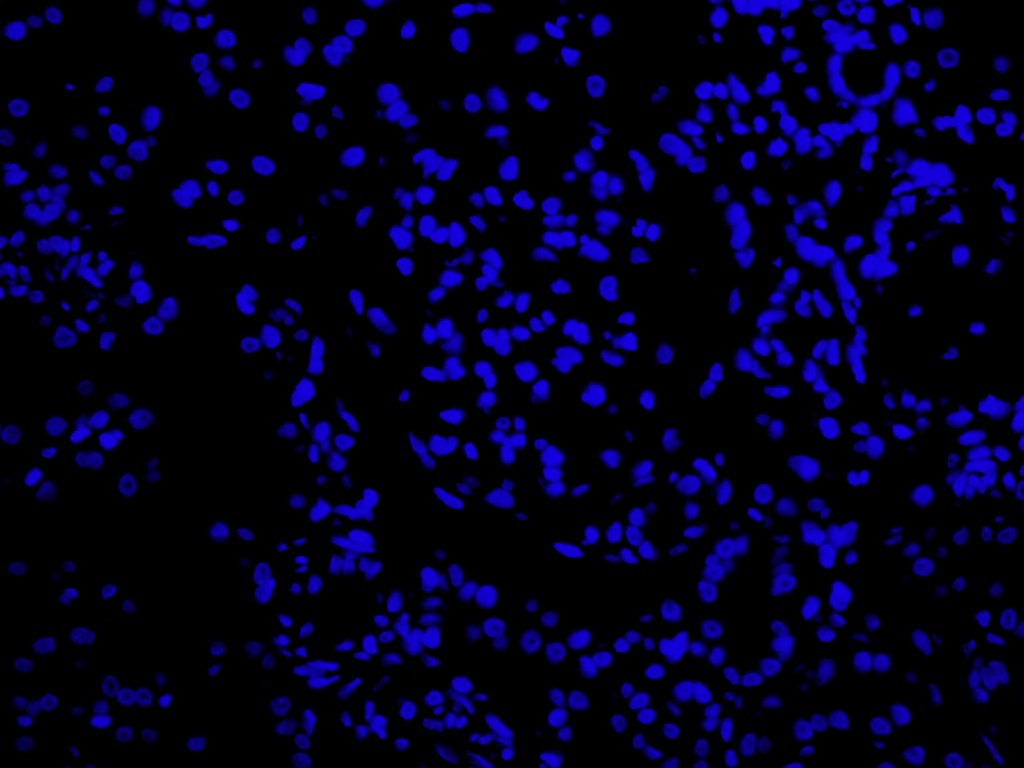

Supplement: Supplementary file 9 — Figure EV1 Source Data [file 44321_2025_315_MOESM9_ESM.zip › Figure EV1/EV1D/3-Claudin-1-GLDC/LEE II/2 (3).jpg]

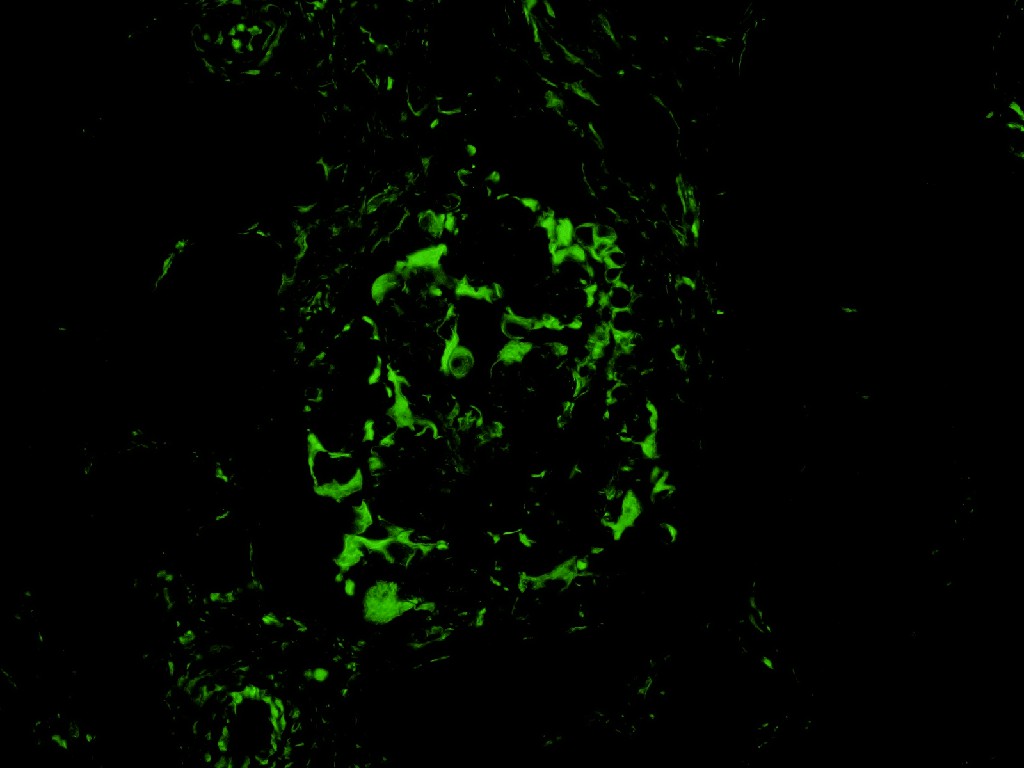

Supplement: Supplementary file 9 — Figure EV1 Source Data [file 44321_2025_315_MOESM9_ESM.zip › Figure EV1/EV1D/3-Claudin-1-GLDC/LEE V/2 (1).jpg]

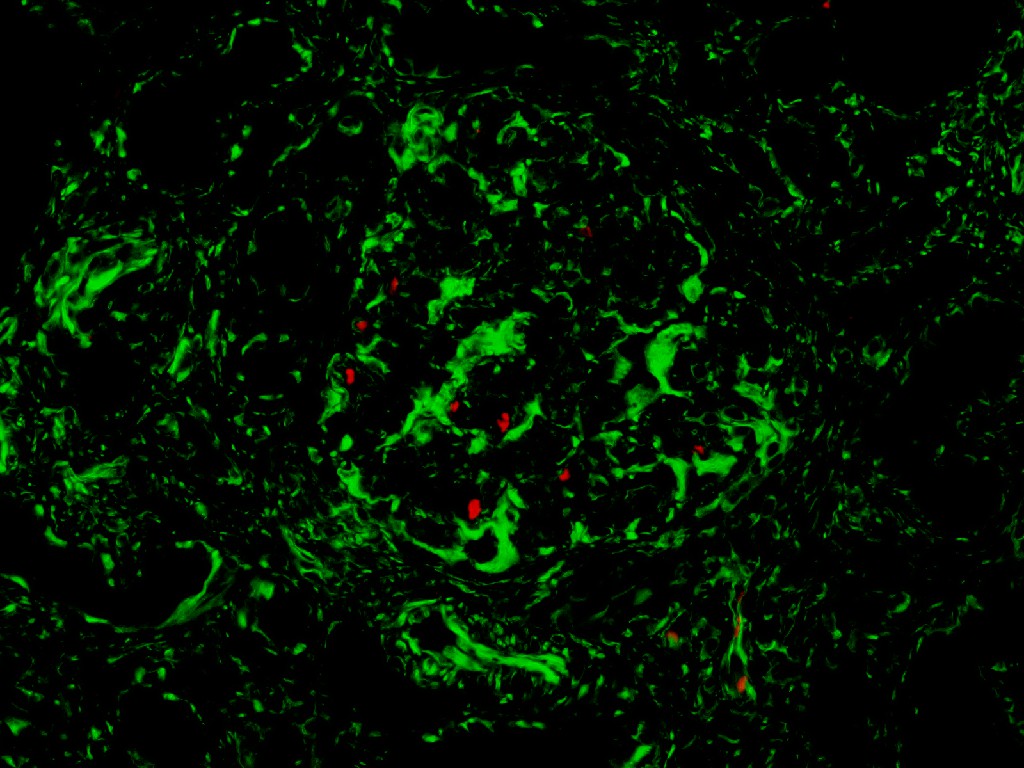

Supplement: Supplementary file 9 — Figure EV1 Source Data [file 44321_2025_315_MOESM9_ESM.zip › Figure EV1/EV1D/3-Claudin-1-GLDC/LEE V/1 (1).jpg]

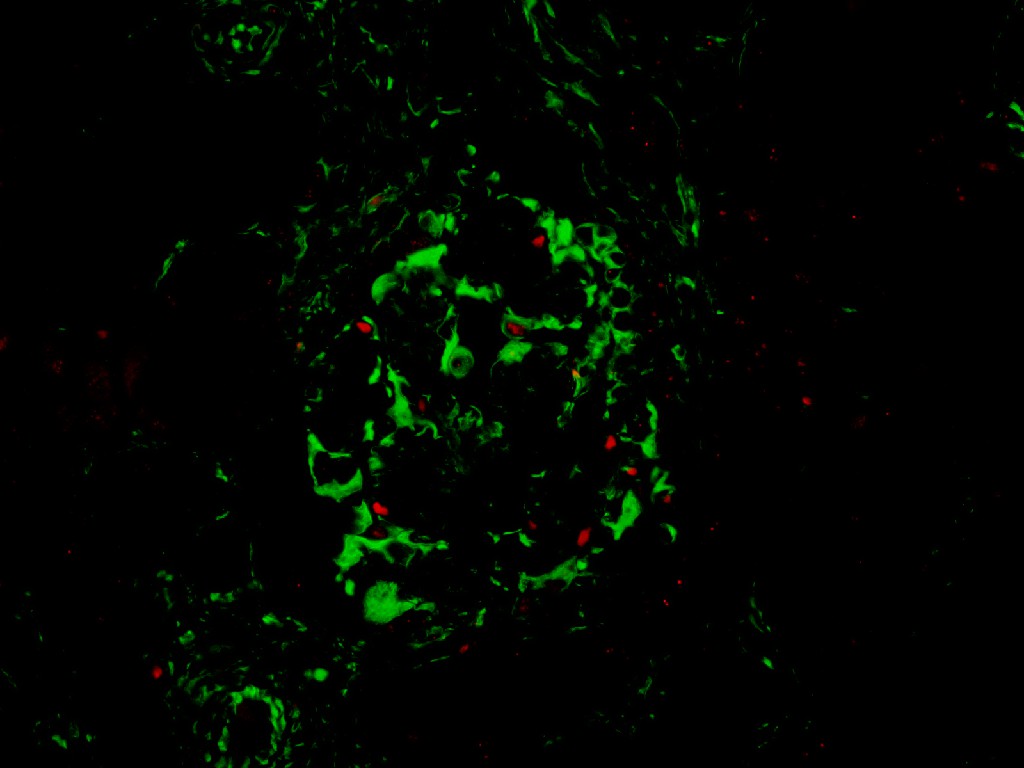

Supplement: Supplementary file 9 — Figure EV1 Source Data [file 44321_2025_315_MOESM9_ESM.zip › Figure EV1/EV1D/3-Claudin-1-GLDC/LEE V/2 (4).jpg]

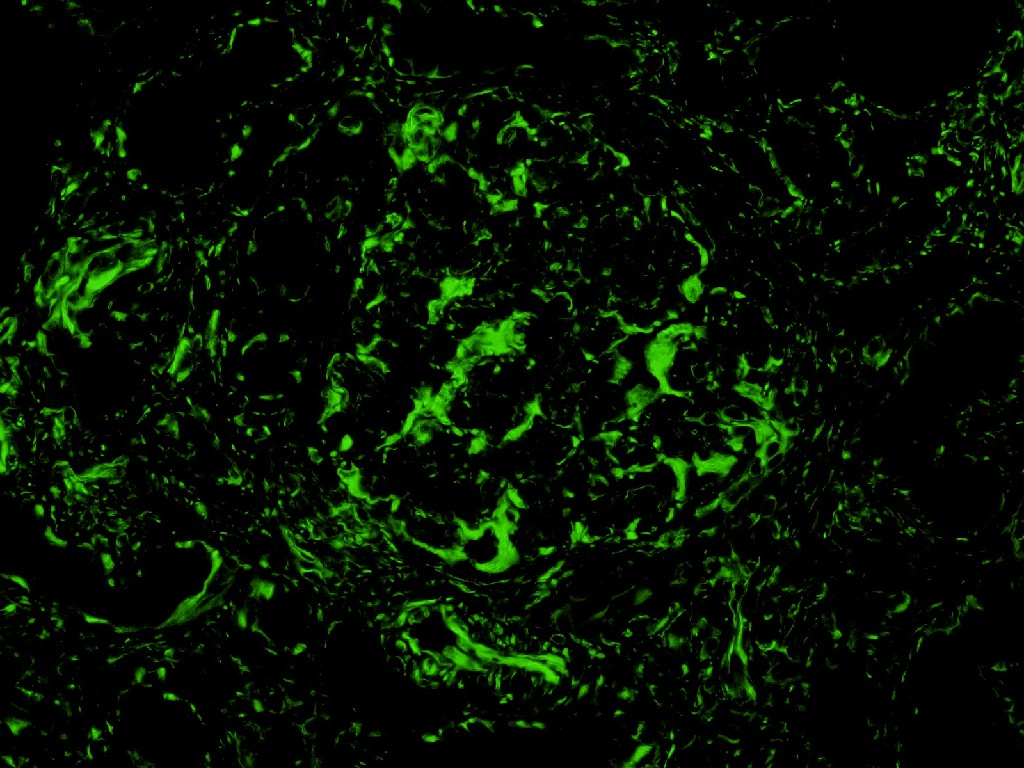

Supplement: Supplementary file 9 — Figure EV1 Source Data [file 44321_2025_315_MOESM9_ESM.zip › Figure EV1/EV1D/3-Claudin-1-GLDC/LEE V/1 (2).jpg]

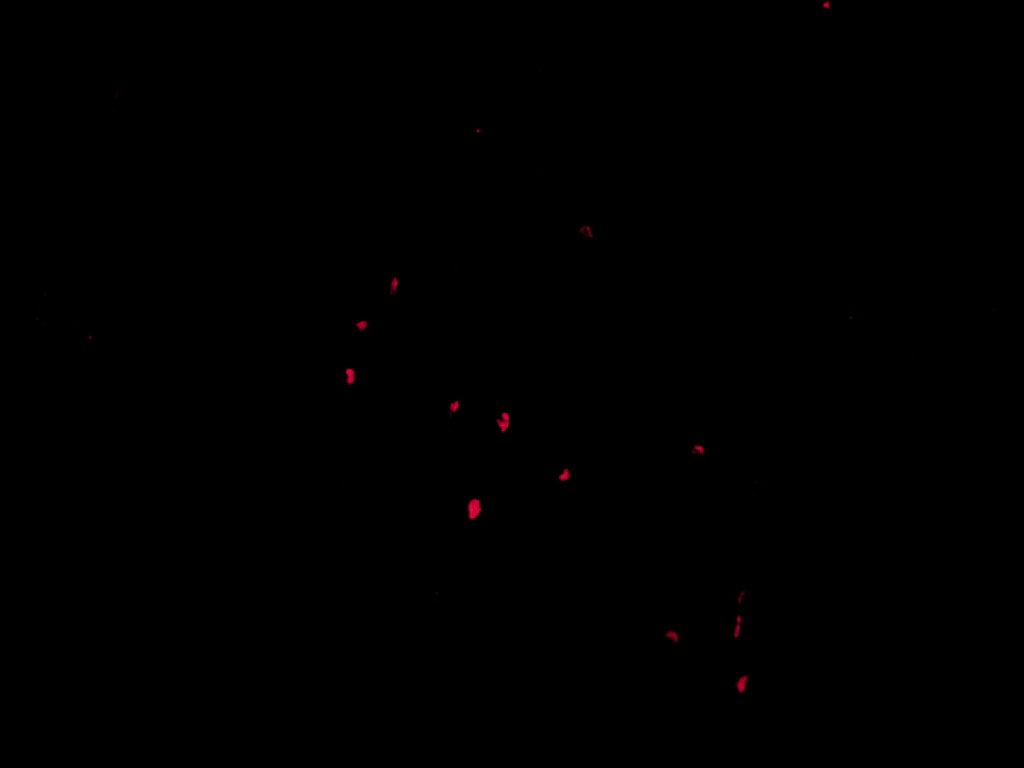

Supplement: Supplementary file 9 — Figure EV1 Source Data [file 44321_2025_315_MOESM9_ESM.zip › Figure EV1/EV1D/3-Claudin-1-GLDC/LEE V/1 (3).jpg]

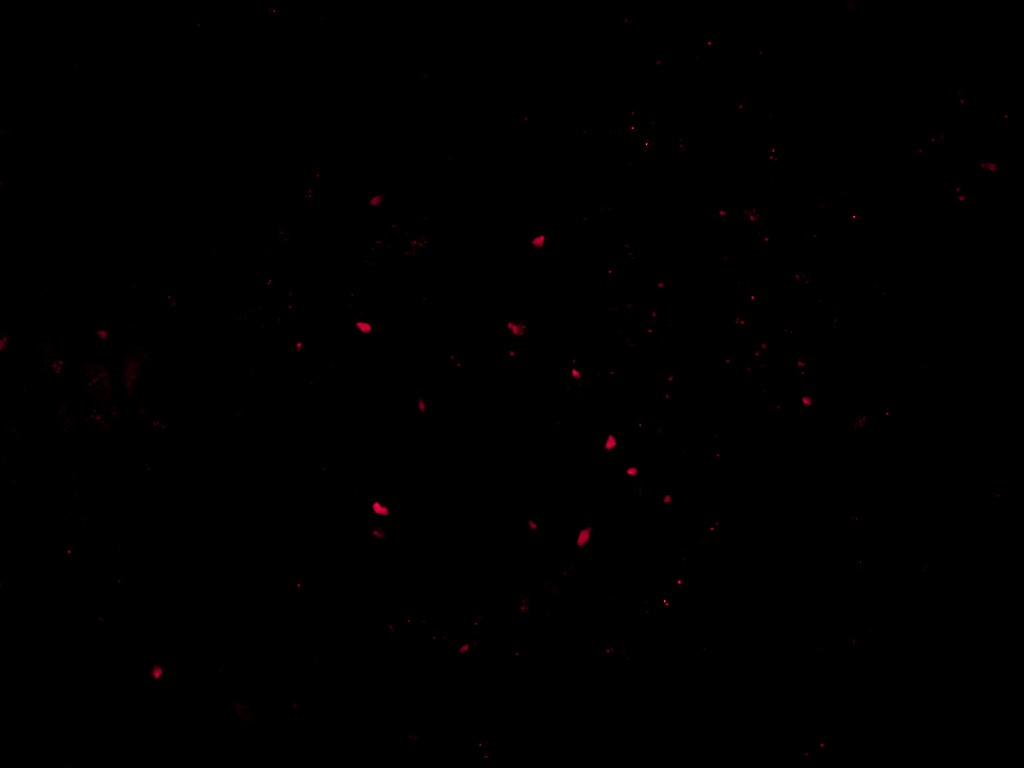

Supplement: Supplementary file 9 — Figure EV1 Source Data [file 44321_2025_315_MOESM9_ESM.zip › Figure EV1/EV1D/3-Claudin-1-GLDC/LEE V/2 (2).jpg]

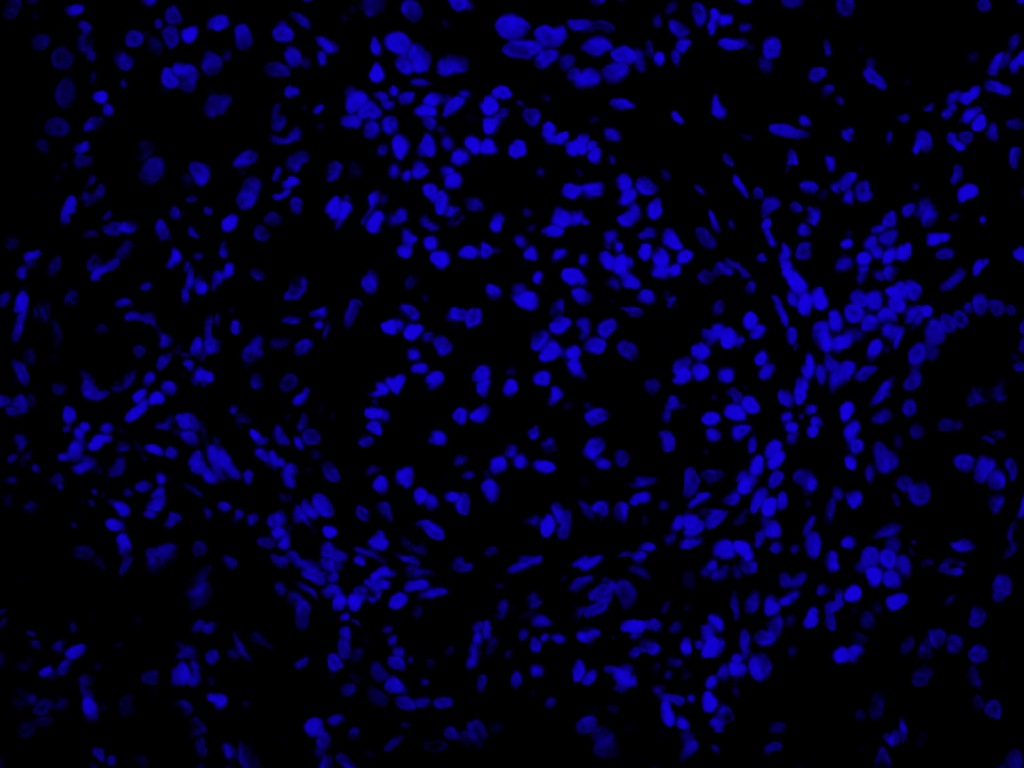

Supplement: Supplementary file 9 — Figure EV1 Source Data [file 44321_2025_315_MOESM9_ESM.zip › Figure EV1/EV1D/3-Claudin-1-GLDC/LEE V/1 (4).jpg]

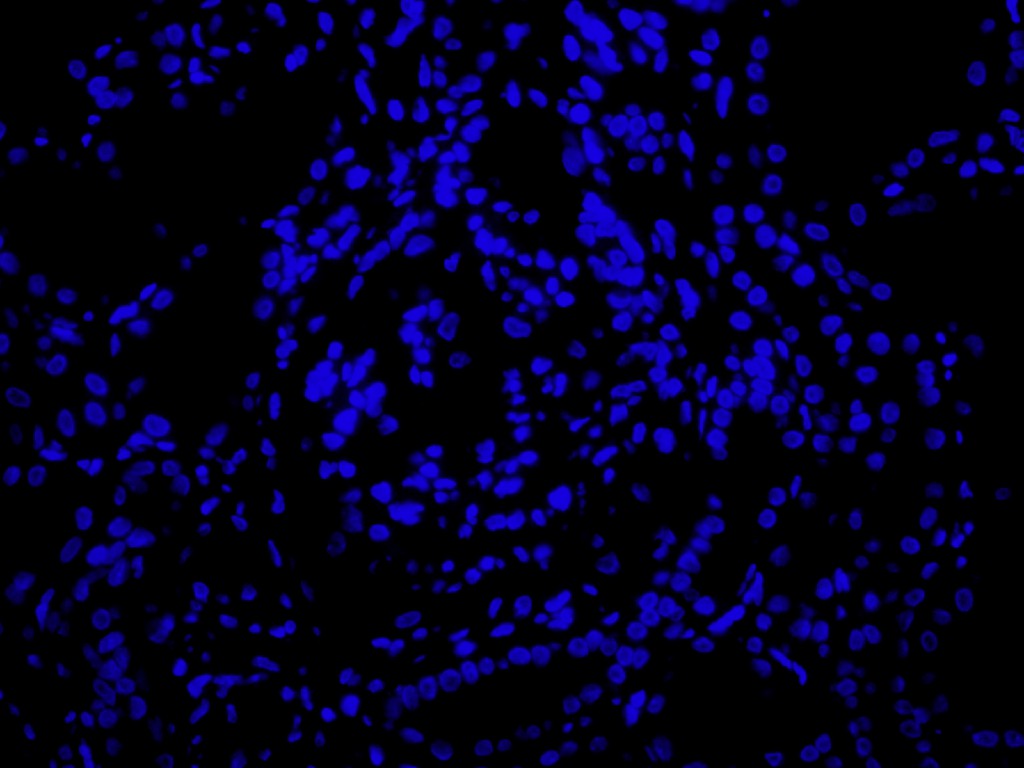

Supplement: Supplementary file 9 — Figure EV1 Source Data [file 44321_2025_315_MOESM9_ESM.zip › Figure EV1/EV1D/3-Claudin-1-GLDC/LEE V/2 (3).jpg]

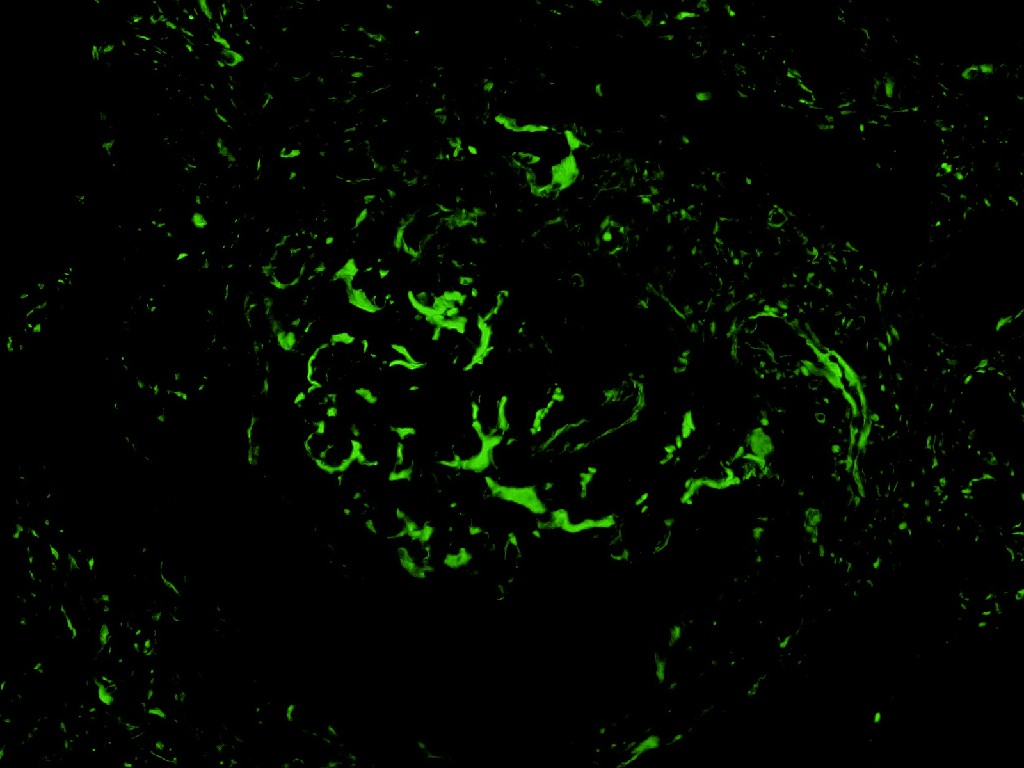

Supplement: Supplementary file 9 — Figure EV1 Source Data [file 44321_2025_315_MOESM9_ESM.zip › Figure EV1/EV1D/3-Claudin-1-GLDC/LEE IV/3 (1).jpg]

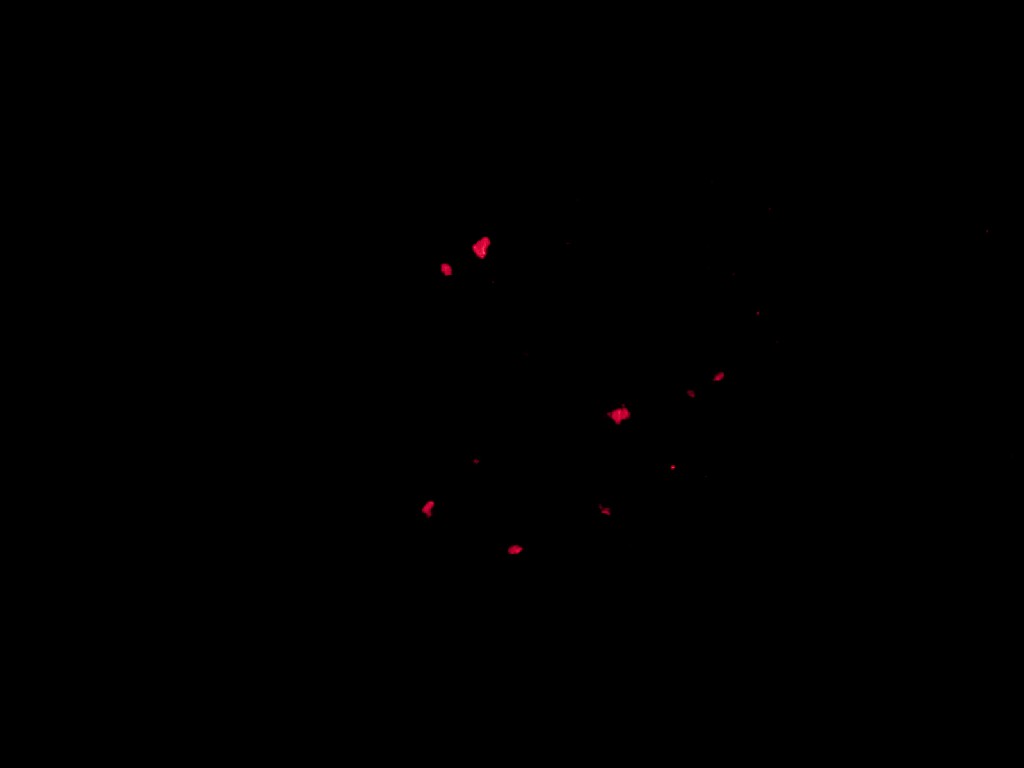

Supplement: Supplementary file 9 — Figure EV1 Source Data [file 44321_2025_315_MOESM9_ESM.zip › Figure EV1/EV1D/3-Claudin-1-GLDC/LEE IV/6 (2).jpg]

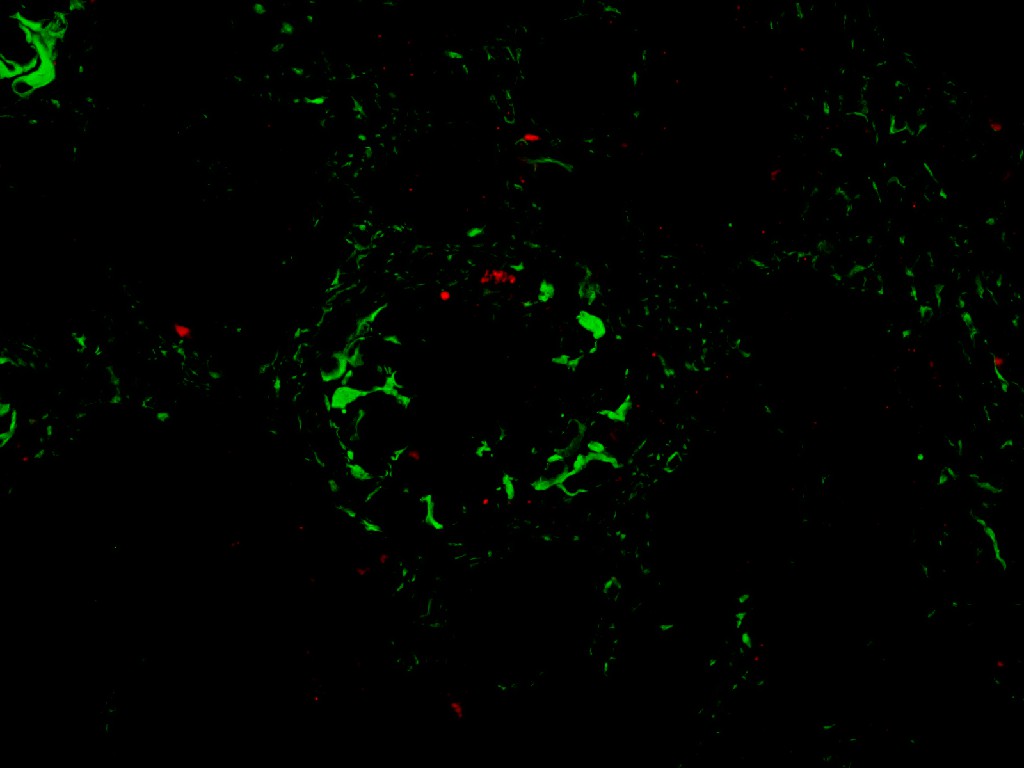

Supplement: Supplementary file 9 — Figure EV1 Source Data [file 44321_2025_315_MOESM9_ESM.zip › Figure EV1/EV1D/3-Claudin-1-GLDC/LEE IV/5 (4).jpg]

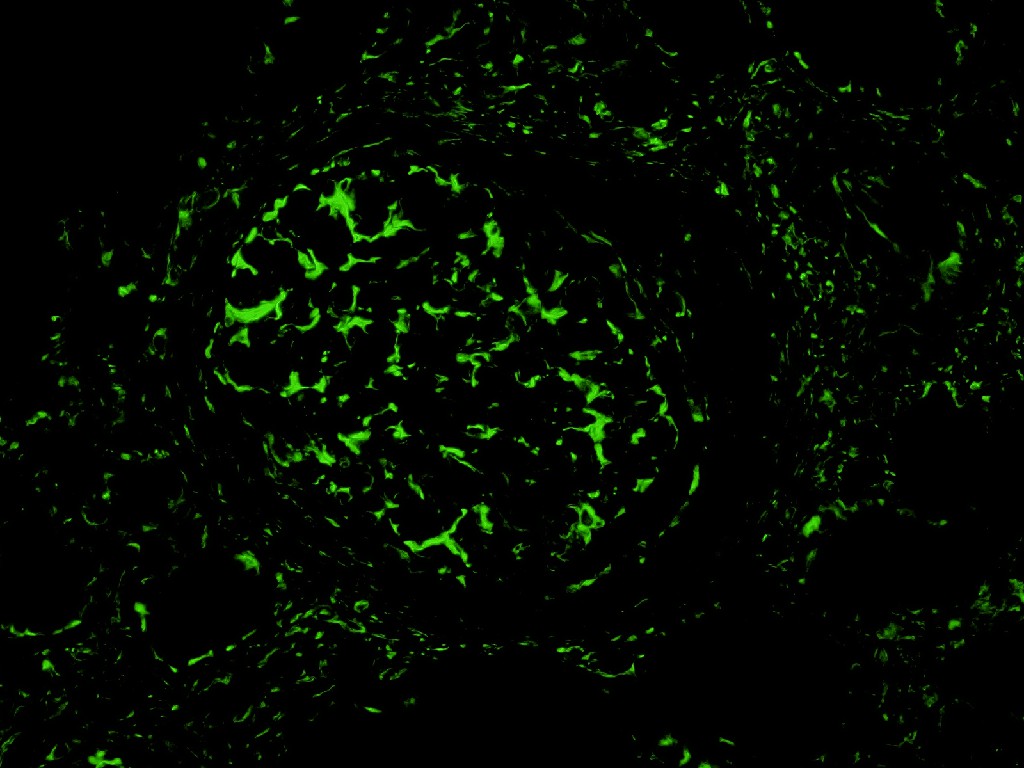

Supplement: Supplementary file 9 — Figure EV1 Source Data [file 44321_2025_315_MOESM9_ESM.zip › Figure EV1/EV1D/3-Claudin-1-GLDC/LEE IV/2 (1).jpg]

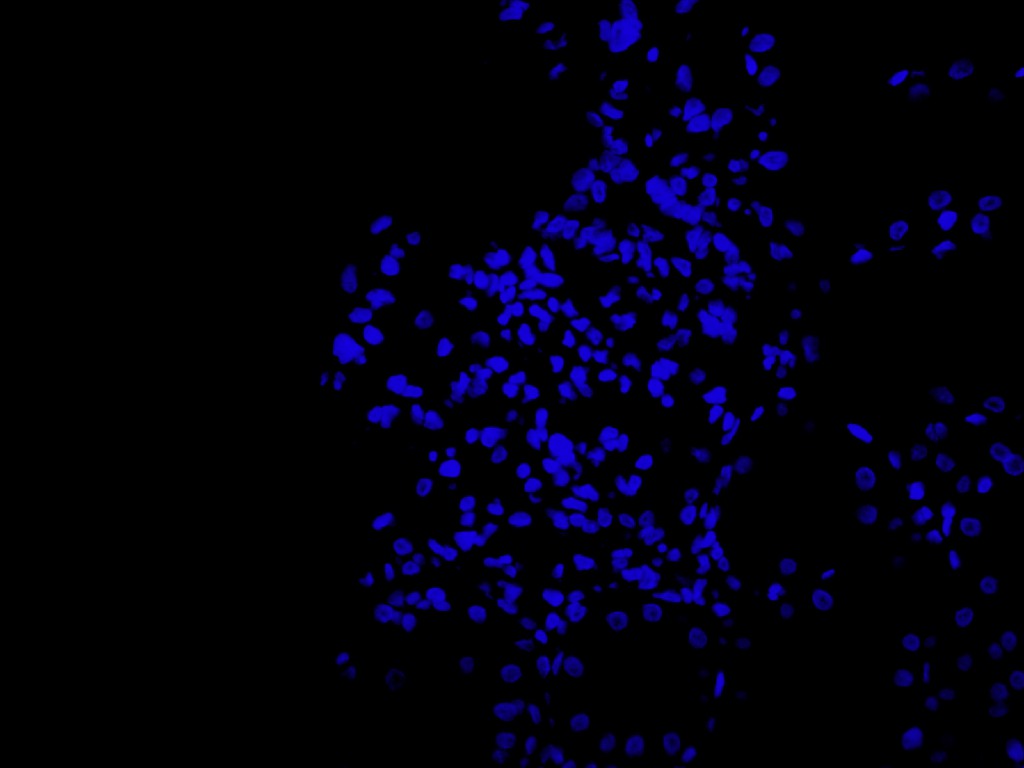

Supplement: Supplementary file 9 — Figure EV1 Source Data [file 44321_2025_315_MOESM9_ESM.zip › Figure EV1/EV1D/3-Claudin-1-GLDC/LEE IV/6 (3).jpg]

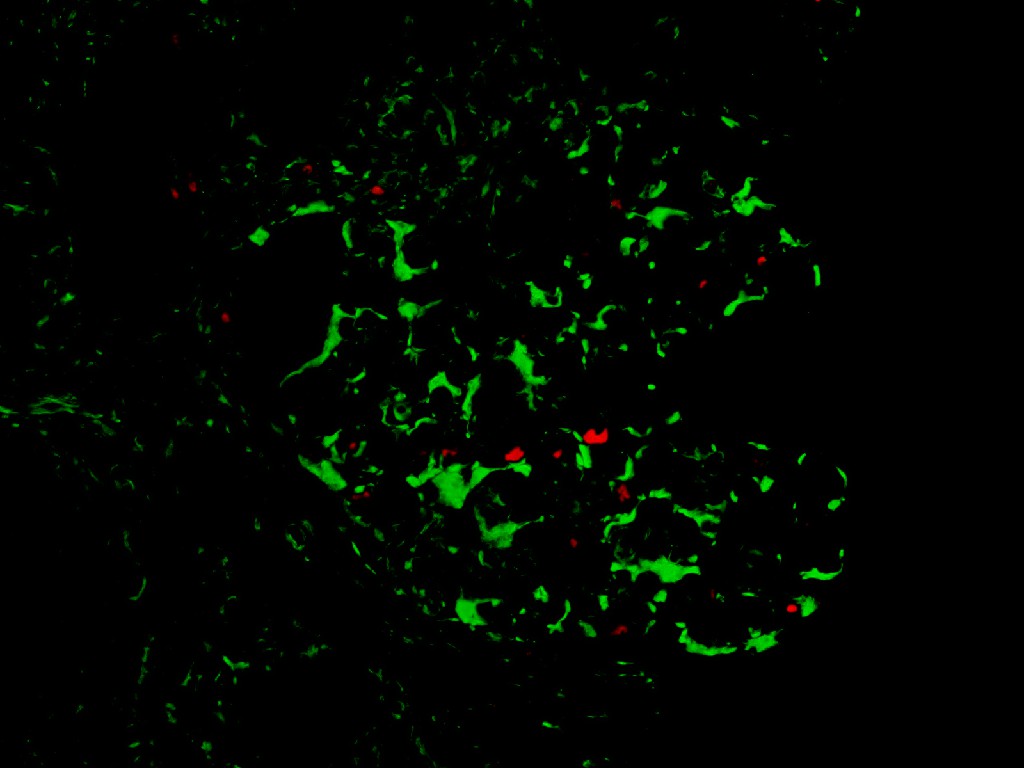

Supplement: Supplementary file 9 — Figure EV1 Source Data [file 44321_2025_315_MOESM9_ESM.zip › Figure EV1/EV1D/3-Claudin-1-GLDC/LEE IV/4 (4).jpg]

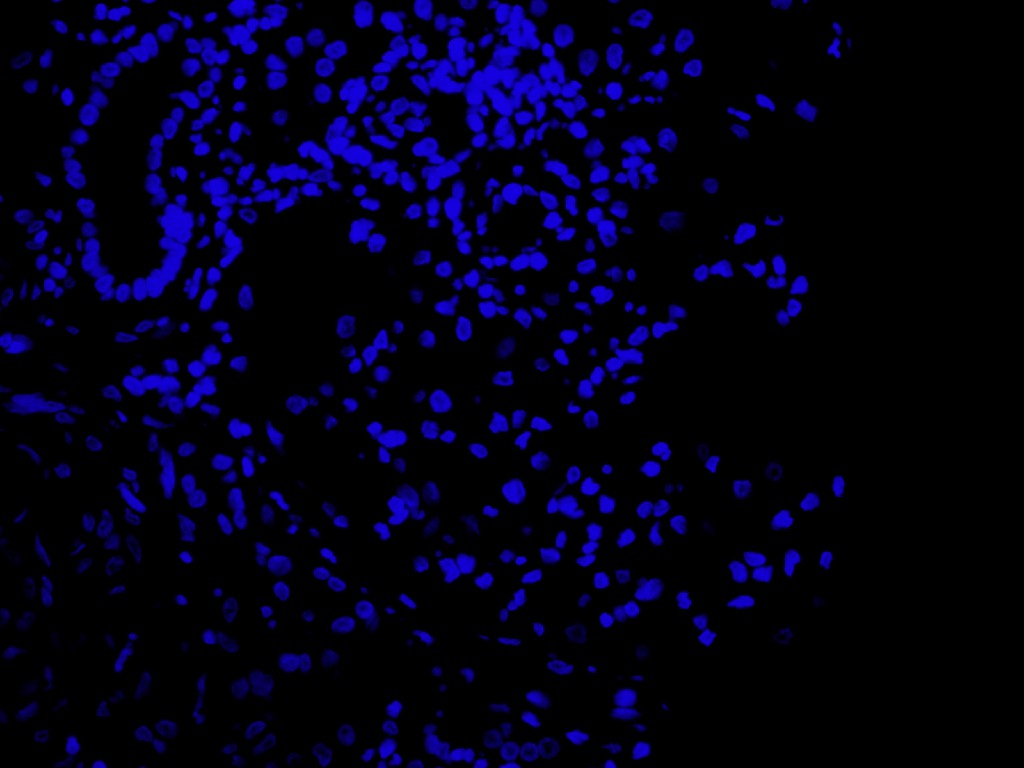

Supplement: Supplementary file 9 — Figure EV1 Source Data [file 44321_2025_315_MOESM9_ESM.zip › Figure EV1/EV1D/3-Claudin-1-GLDC/LEE IV/4 (3).jpg]

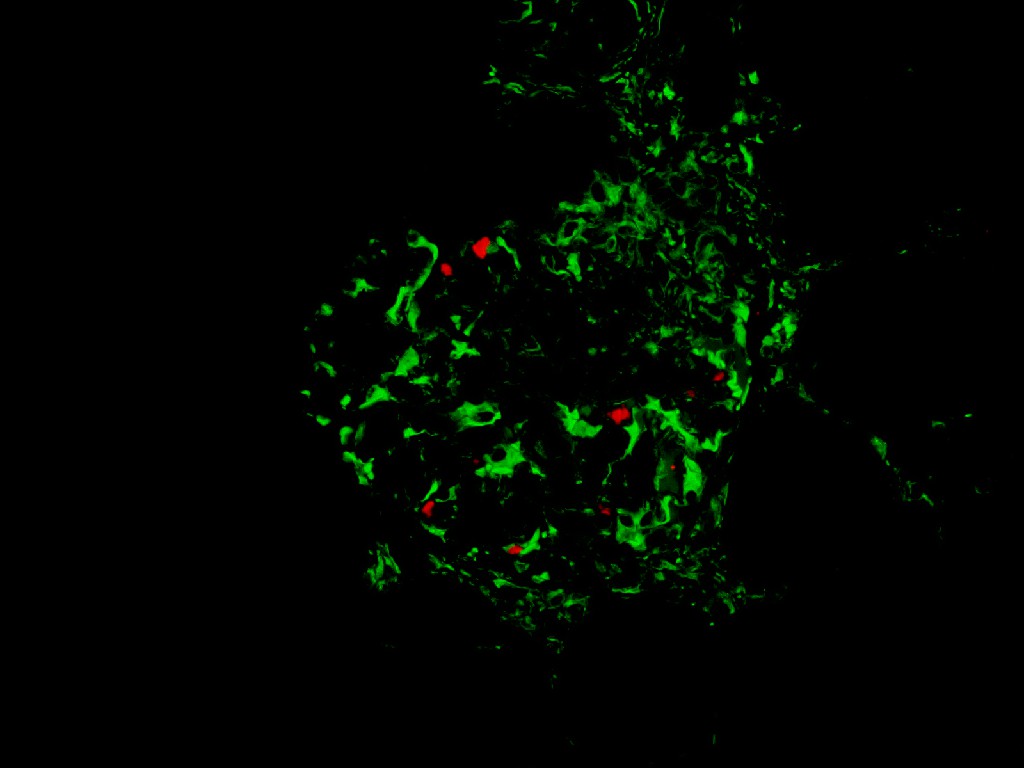

Supplement: Supplementary file 9 — Figure EV1 Source Data [file 44321_2025_315_MOESM9_ESM.zip › Figure EV1/EV1D/3-Claudin-1-GLDC/LEE IV/6 (4).jpg]

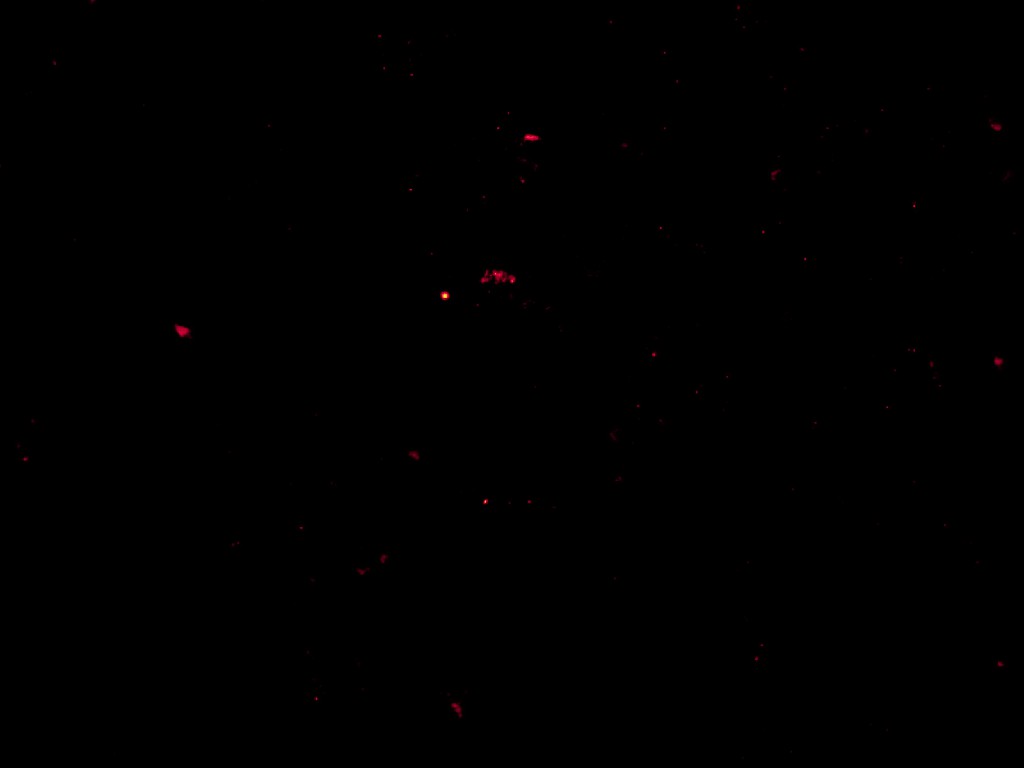

Supplement: Supplementary file 9 — Figure EV1 Source Data [file 44321_2025_315_MOESM9_ESM.zip › Figure EV1/EV1D/3-Claudin-1-GLDC/LEE IV/5 (2).jpg]

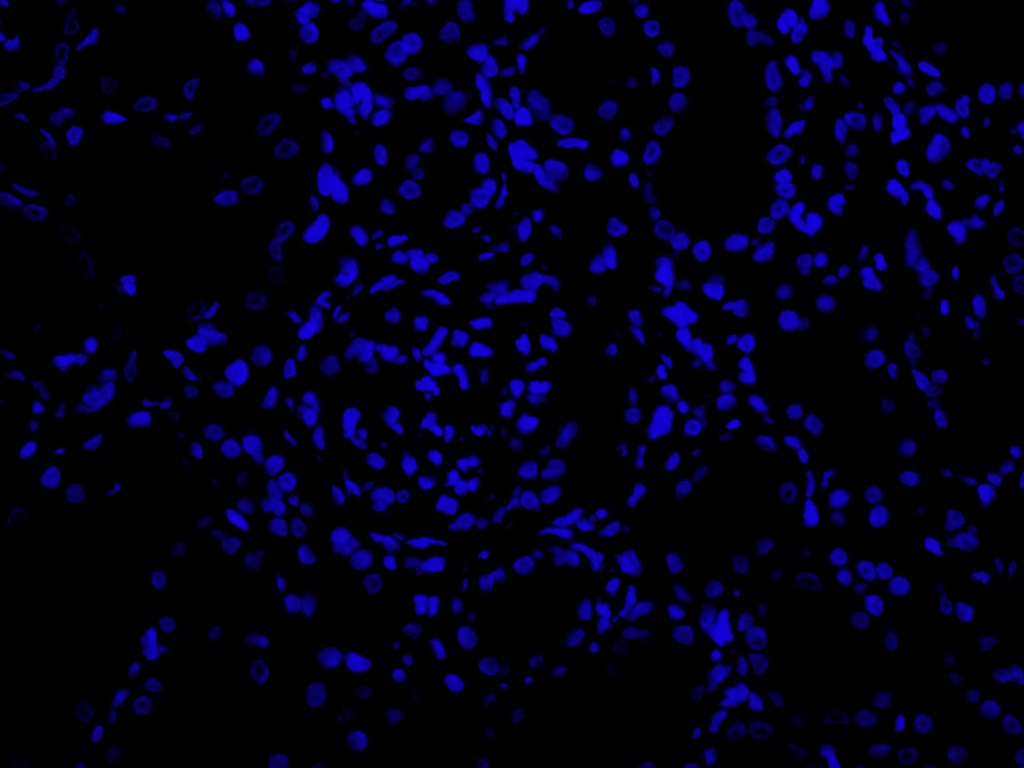

Supplement: Supplementary file 9 — Figure EV1 Source Data [file 44321_2025_315_MOESM9_ESM.zip › Figure EV1/EV1D/3-Claudin-1-GLDC/LEE IV/5 (3).jpg]

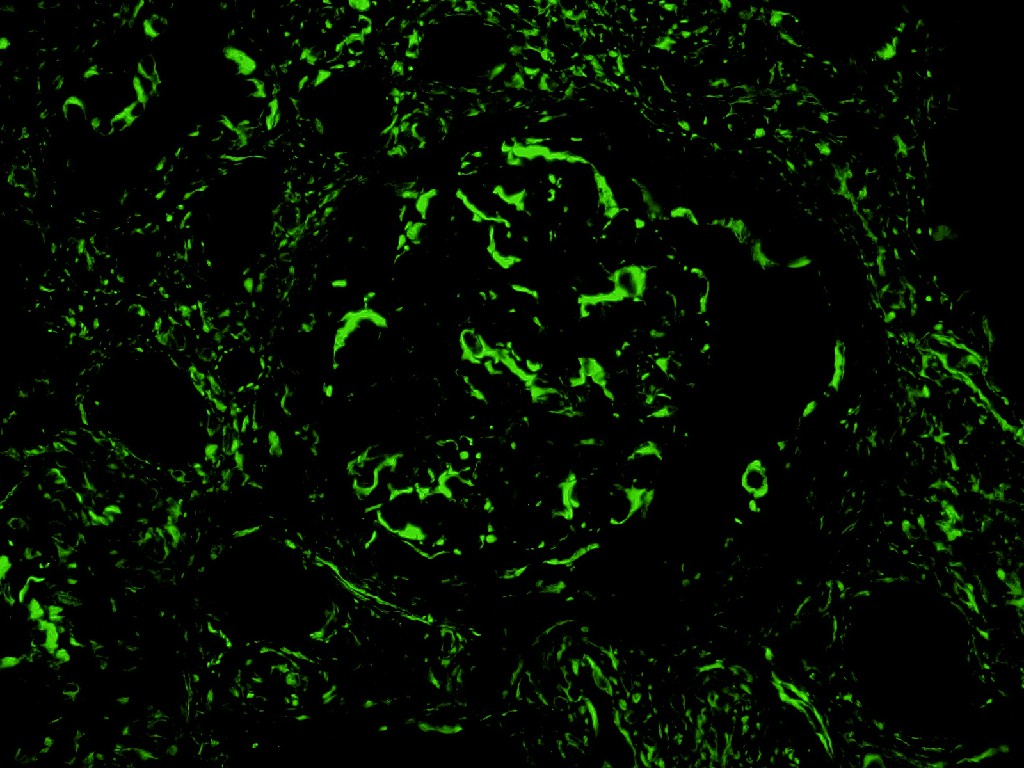

Supplement: Supplementary file 9 — Figure EV1 Source Data [file 44321_2025_315_MOESM9_ESM.zip › Figure EV1/EV1D/3-Claudin-1-GLDC/LEE IV/1 (1).jpg]

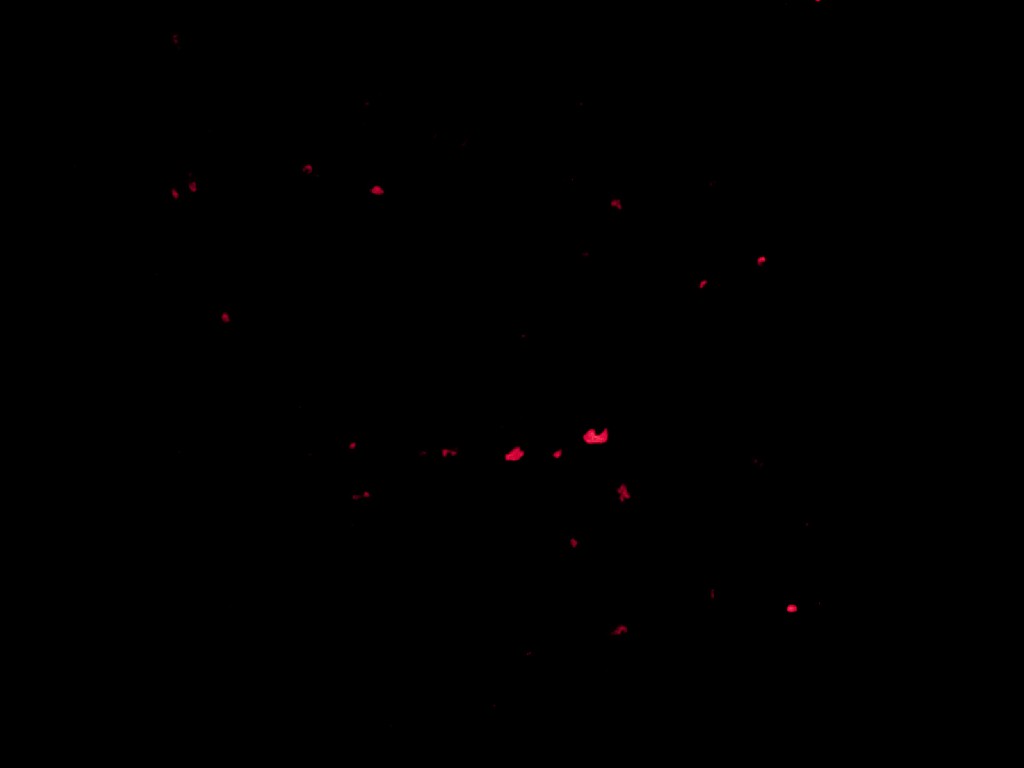

Supplement: Supplementary file 9 — Figure EV1 Source Data [file 44321_2025_315_MOESM9_ESM.zip › Figure EV1/EV1D/3-Claudin-1-GLDC/LEE IV/4 (2).jpg]

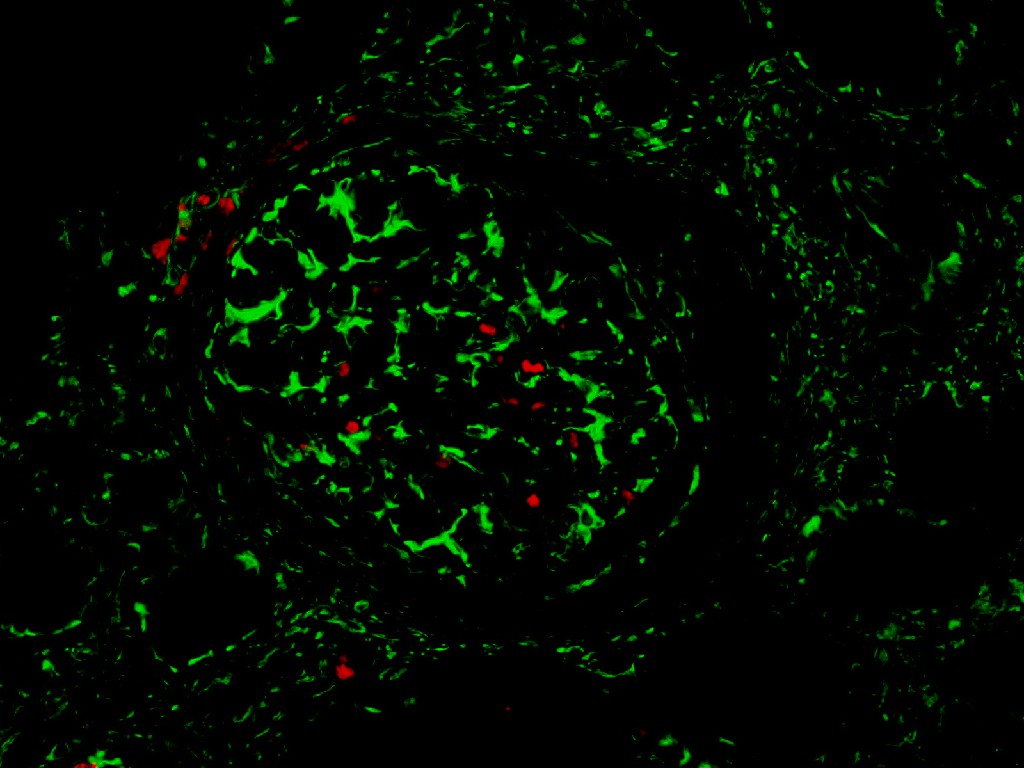

Supplement: Supplementary file 9 — Figure EV1 Source Data [file 44321_2025_315_MOESM9_ESM.zip › Figure EV1/EV1D/3-Claudin-1-GLDC/LEE IV/2 (4).jpg]

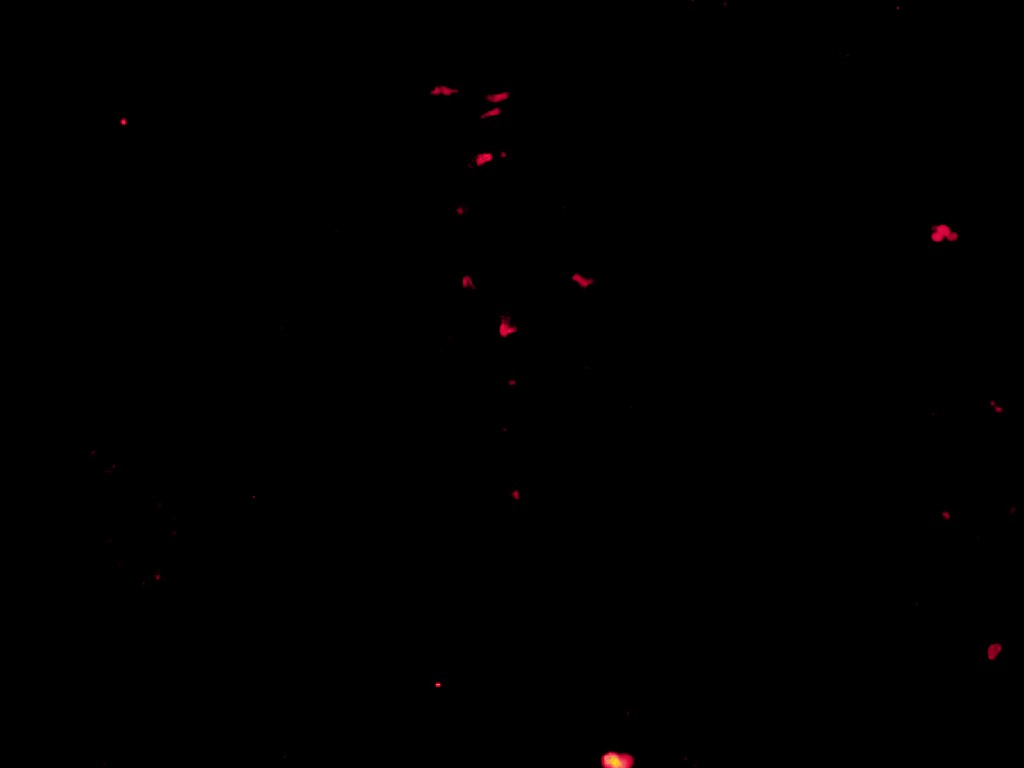

Supplement: Supplementary file 9 — Figure EV1 Source Data [file 44321_2025_315_MOESM9_ESM.zip › Figure EV1/EV1D/3-Claudin-1-GLDC/LEE IV/1 (2).jpg]

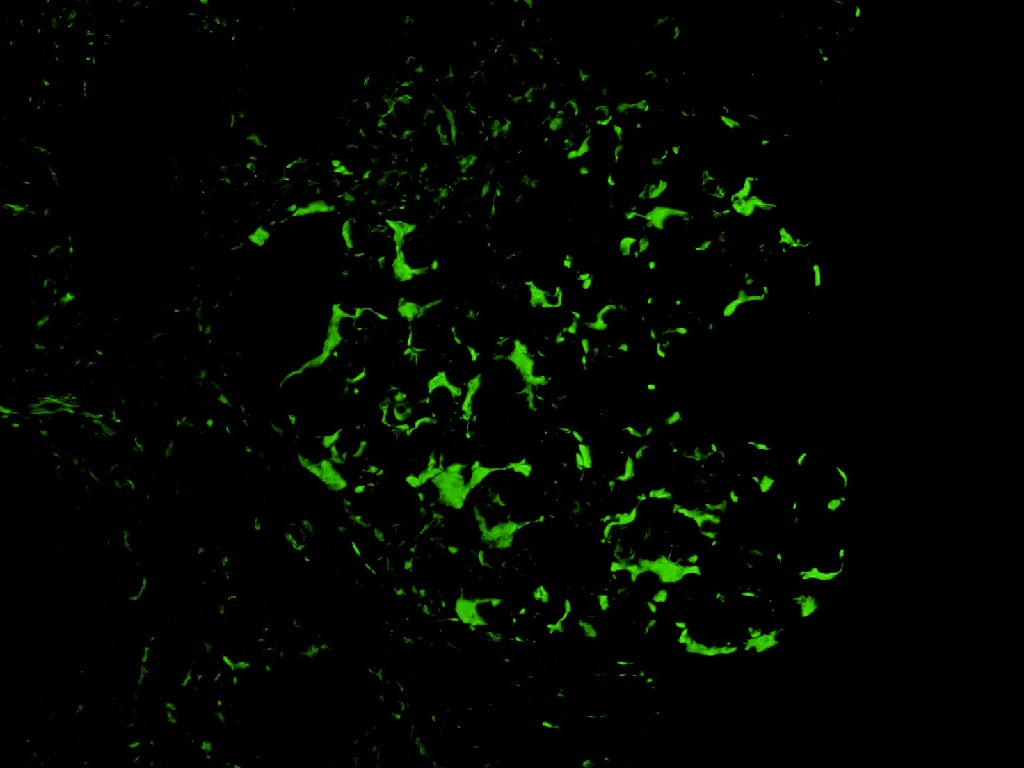

Supplement: Supplementary file 9 — Figure EV1 Source Data [file 44321_2025_315_MOESM9_ESM.zip › Figure EV1/EV1D/3-Claudin-1-GLDC/LEE IV/4 (1).jpg]

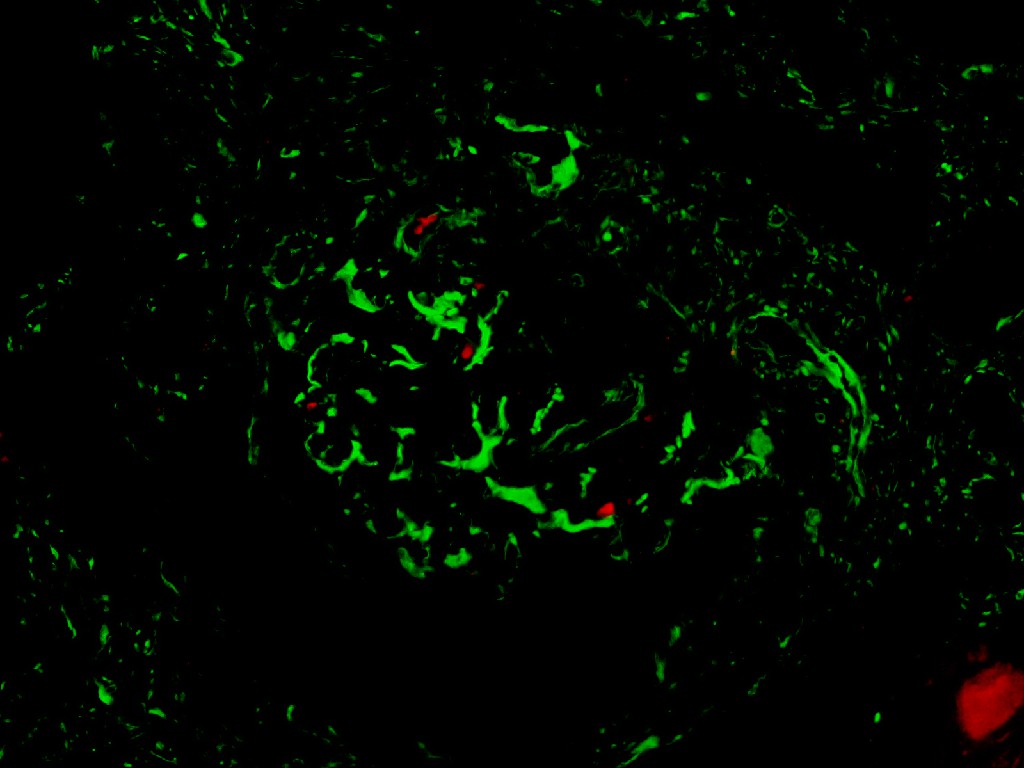

Supplement: Supplementary file 9 — Figure EV1 Source Data [file 44321_2025_315_MOESM9_ESM.zip › Figure EV1/EV1D/3-Claudin-1-GLDC/LEE IV/3 (4).jpg]

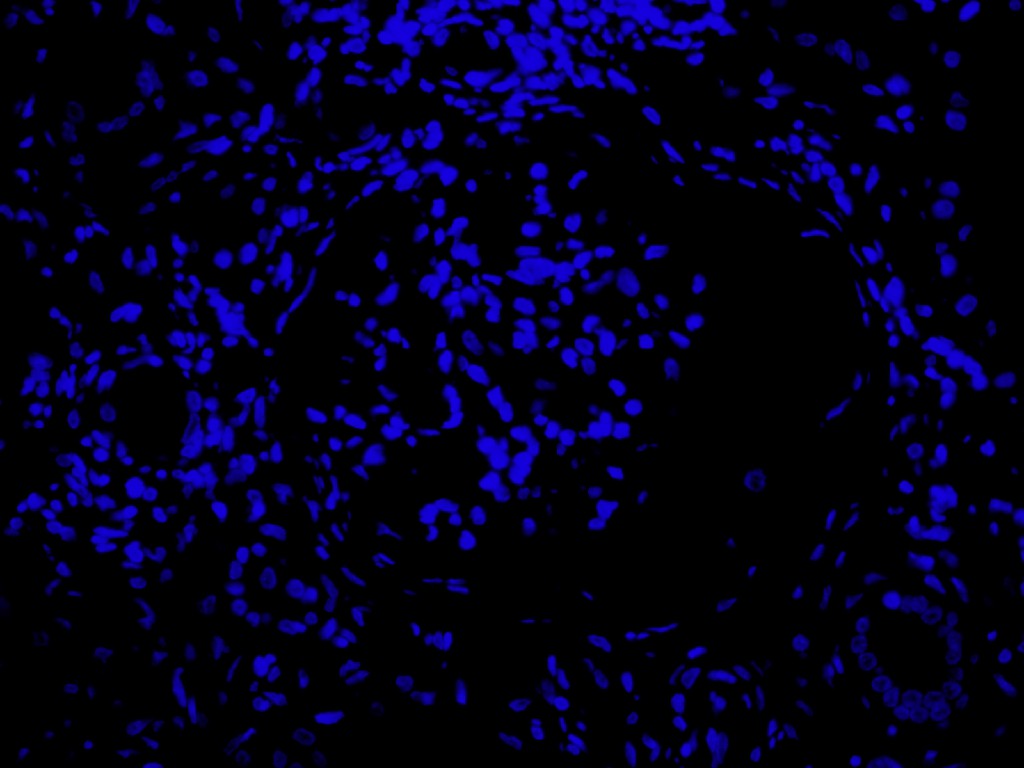

Supplement: Supplementary file 9 — Figure EV1 Source Data [file 44321_2025_315_MOESM9_ESM.zip › Figure EV1/EV1D/3-Claudin-1-GLDC/LEE IV/1 (3).jpg]

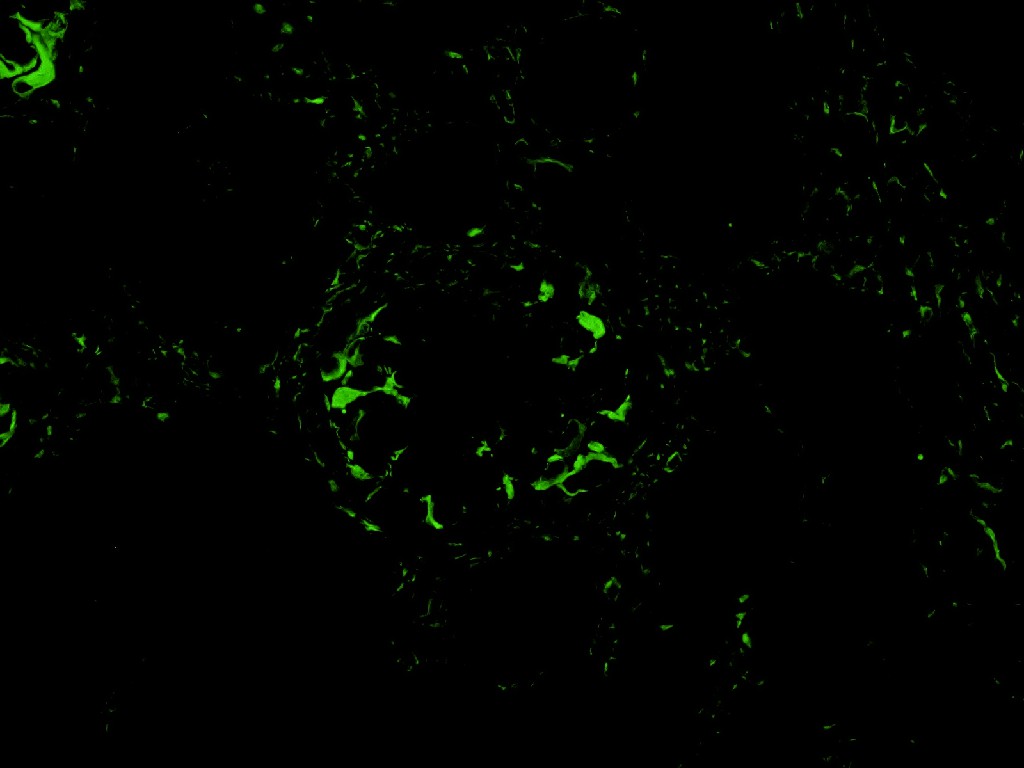

Supplement: Supplementary file 9 — Figure EV1 Source Data [file 44321_2025_315_MOESM9_ESM.zip › Figure EV1/EV1D/3-Claudin-1-GLDC/LEE IV/5 (1).jpg]

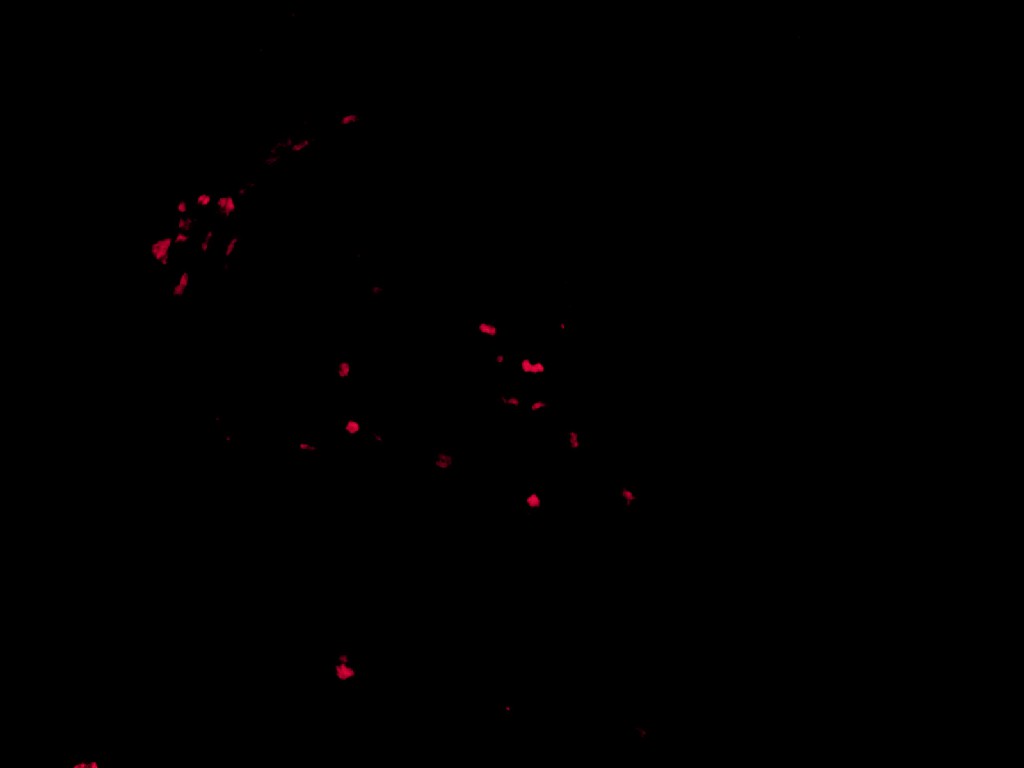

Supplement: Supplementary file 9 — Figure EV1 Source Data [file 44321_2025_315_MOESM9_ESM.zip › Figure EV1/EV1D/3-Claudin-1-GLDC/LEE IV/2 (2).jpg]

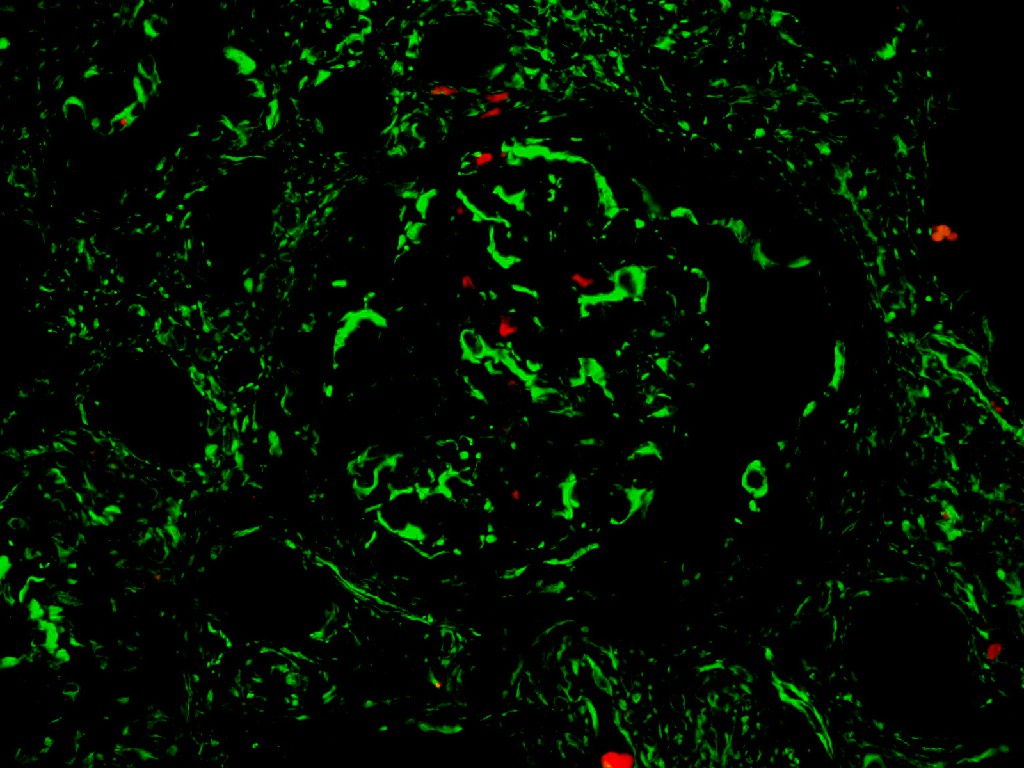

Supplement: Supplementary file 9 — Figure EV1 Source Data [file 44321_2025_315_MOESM9_ESM.zip › Figure EV1/EV1D/3-Claudin-1-GLDC/LEE IV/1 (4).jpg]

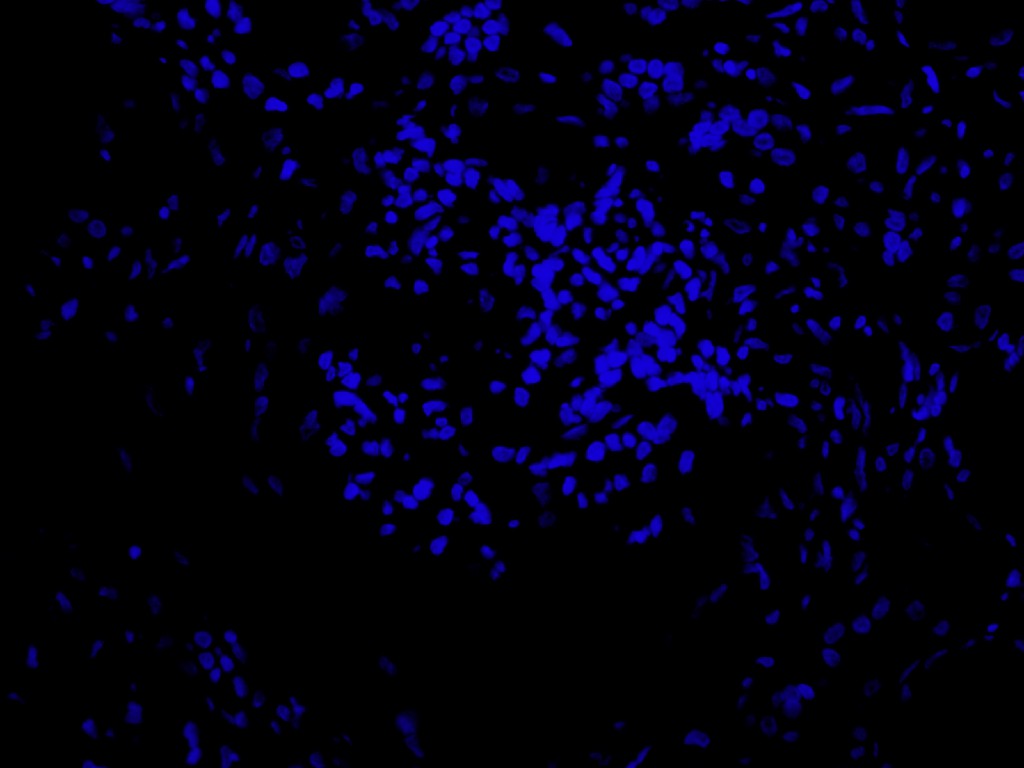

Supplement: Supplementary file 9 — Figure EV1 Source Data [file 44321_2025_315_MOESM9_ESM.zip › Figure EV1/EV1D/3-Claudin-1-GLDC/LEE IV/3 (3).jpg]

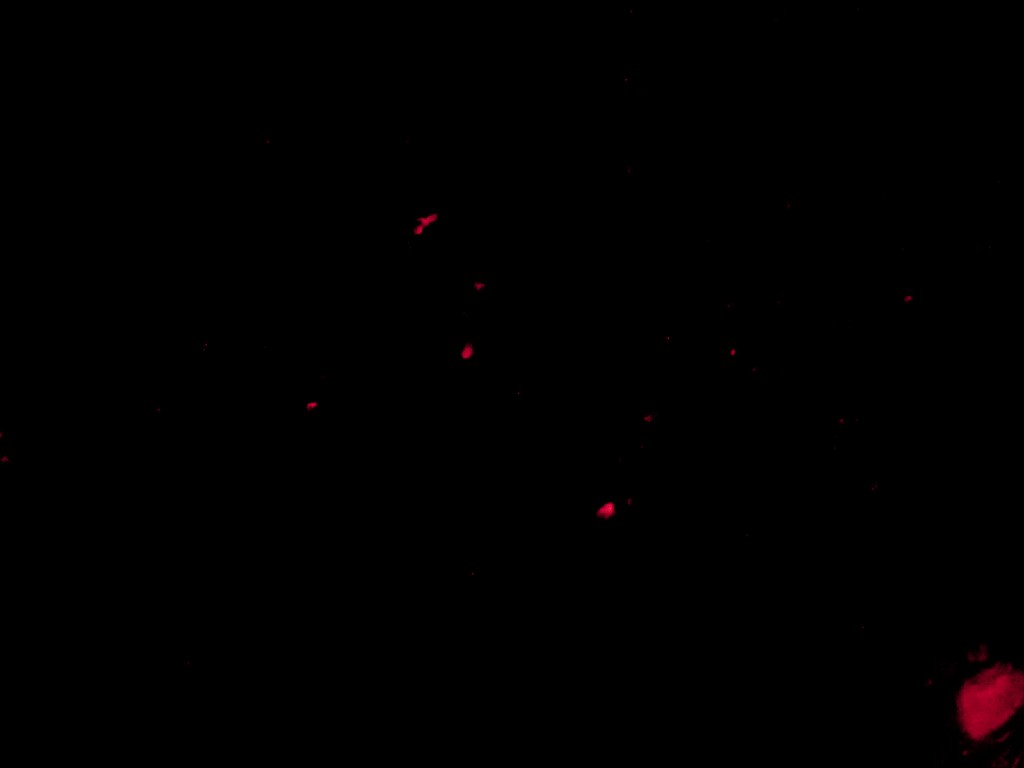

Supplement: Supplementary file 9 — Figure EV1 Source Data [file 44321_2025_315_MOESM9_ESM.zip › Figure EV1/EV1D/3-Claudin-1-GLDC/LEE IV/3 (2).jpg]

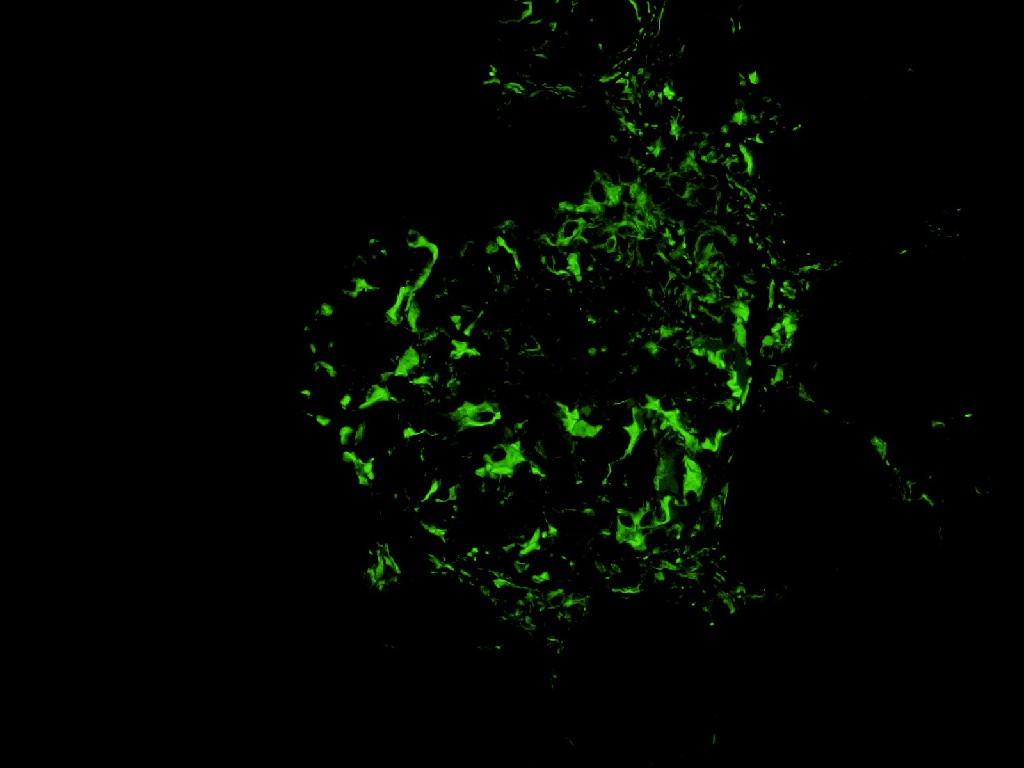

Supplement: Supplementary file 9 — Figure EV1 Source Data [file 44321_2025_315_MOESM9_ESM.zip › Figure EV1/EV1D/3-Claudin-1-GLDC/LEE IV/6 (1).jpg]

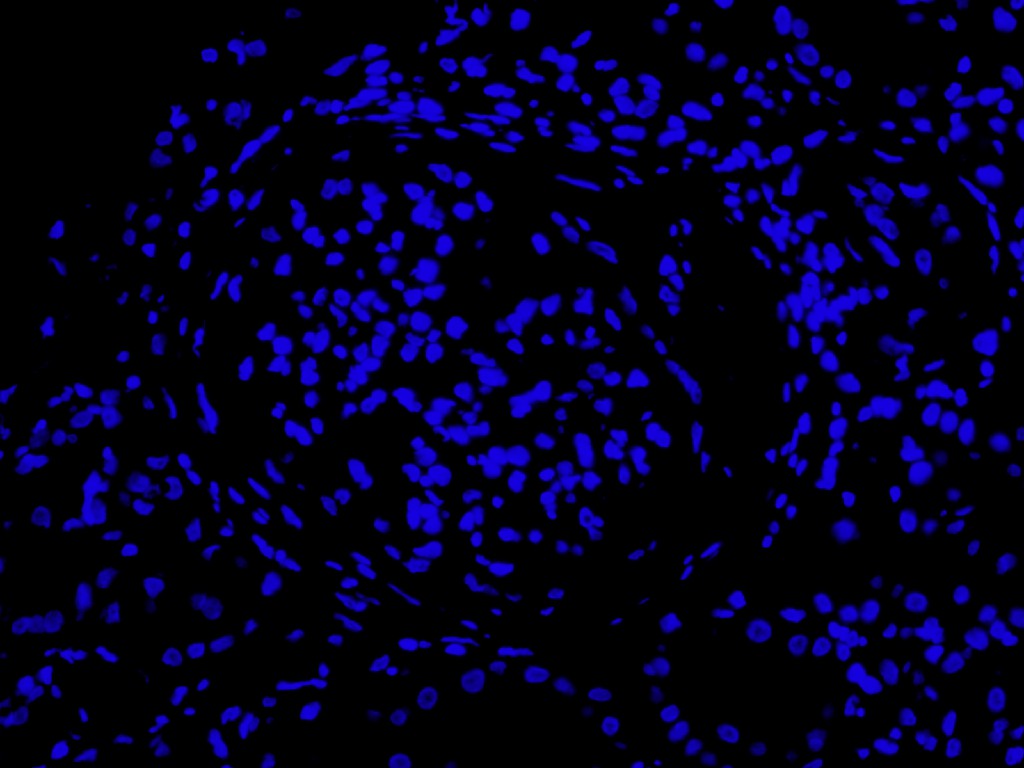

Supplement: Supplementary file 9 — Figure EV1 Source Data [file 44321_2025_315_MOESM9_ESM.zip › Figure EV1/EV1D/3-Claudin-1-GLDC/LEE IV/2 (3).jpg]

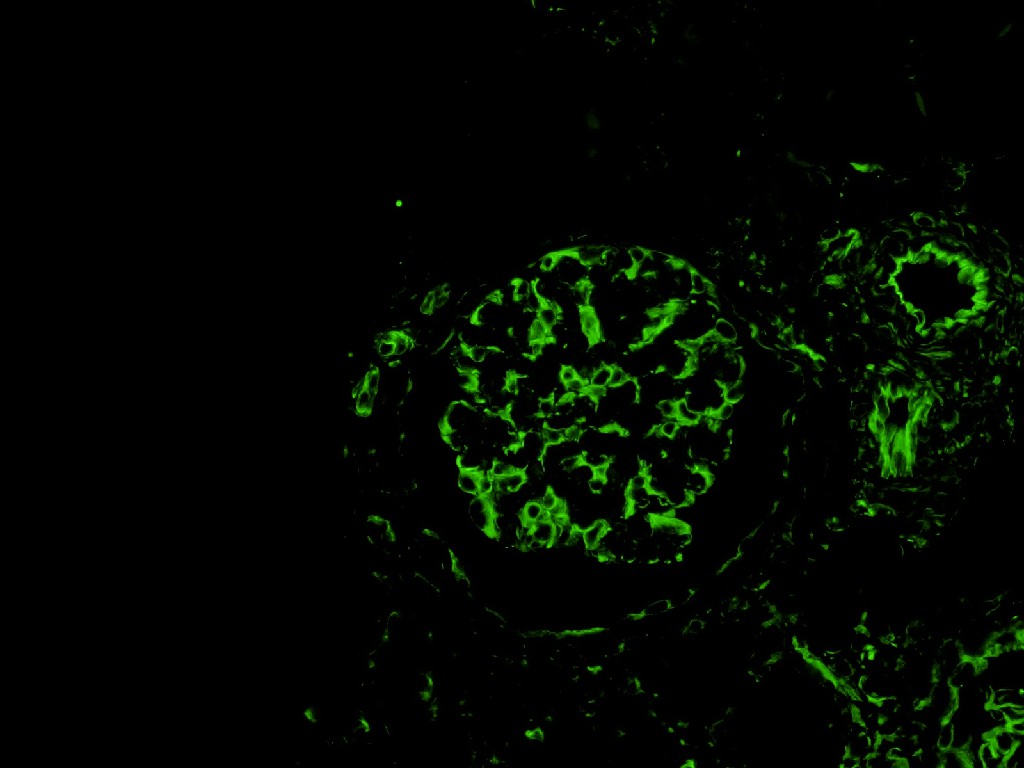

Supplement: Supplementary file 9 — Figure EV1 Source Data [file 44321_2025_315_MOESM9_ESM.zip › Figure EV1/EV1D/2-CD31-GLDC/LEE III/3 (1).jpg]

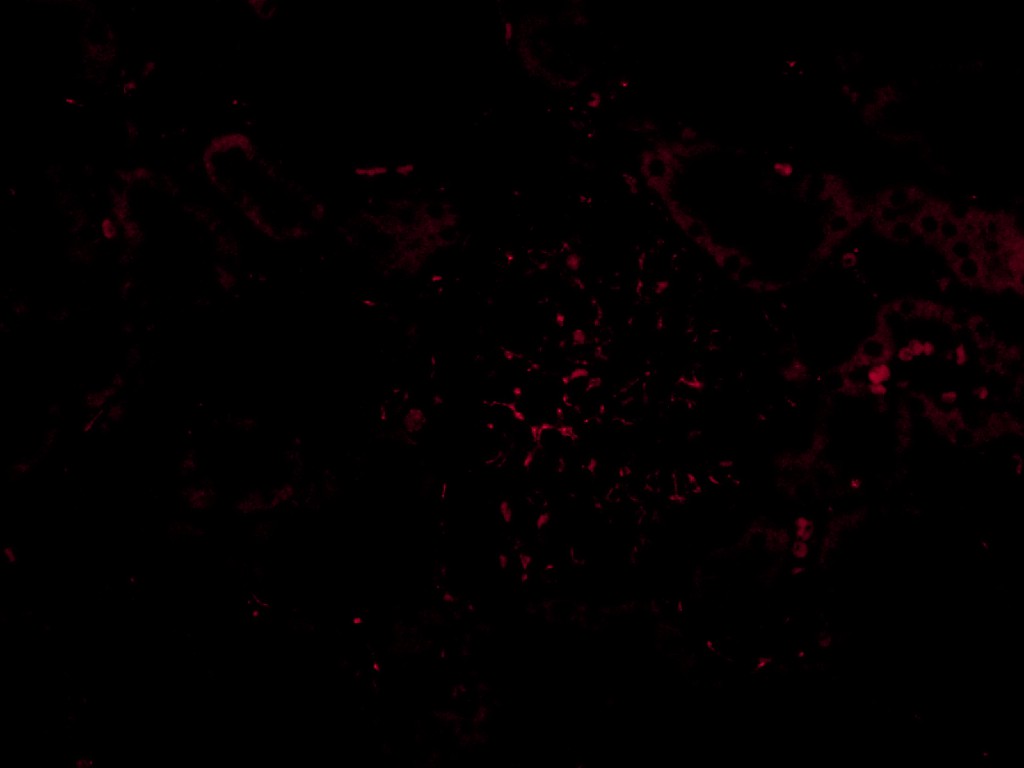

Supplement: Supplementary file 9 — Figure EV1 Source Data [file 44321_2025_315_MOESM9_ESM.zip › Figure EV1/EV1D/2-CD31-GLDC/LEE III/7 (3).jpg]

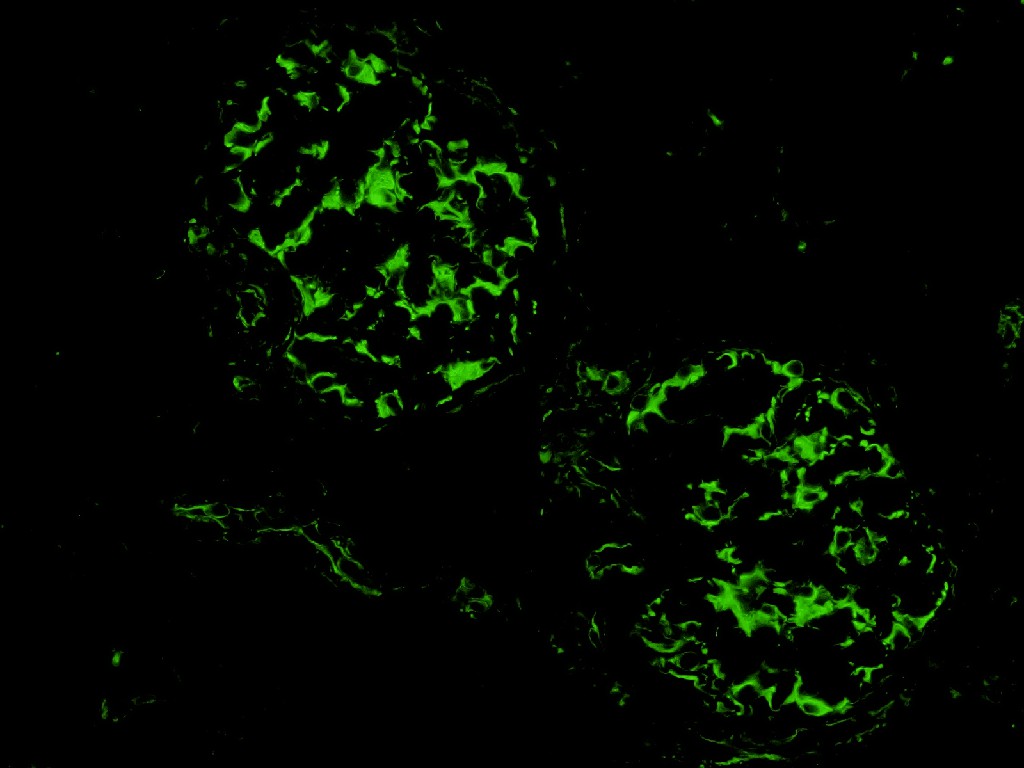

Supplement: Supplementary file 9 — Figure EV1 Source Data [file 44321_2025_315_MOESM9_ESM.zip › Figure EV1/EV1D/2-CD31-GLDC/LEE III/10 (1).jpg]

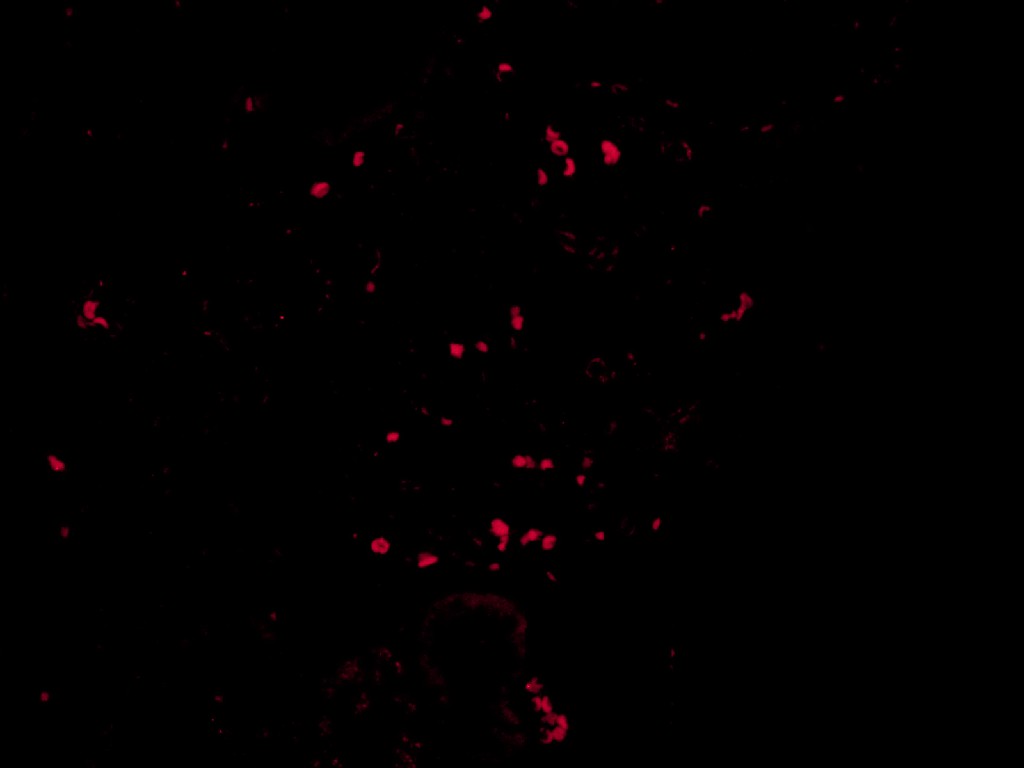

Supplement: Supplementary file 9 — Figure EV1 Source Data [file 44321_2025_315_MOESM9_ESM.zip › Figure EV1/EV1D/2-CD31-GLDC/LEE III/6 (2).jpg]

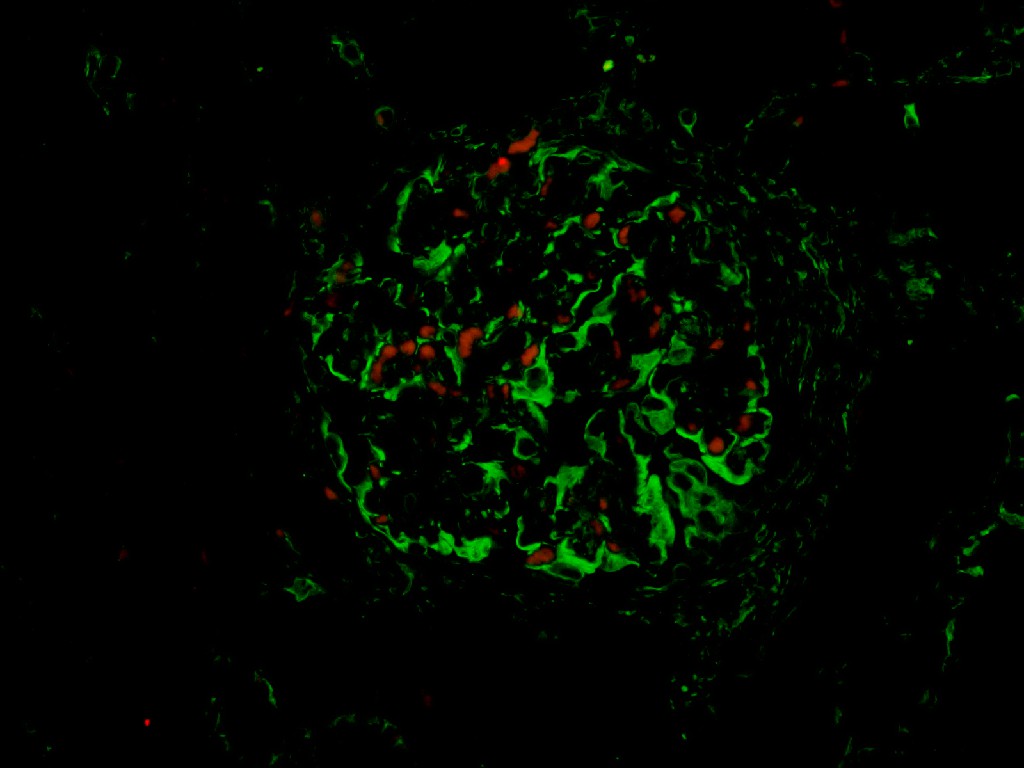

Supplement: Supplementary file 9 — Figure EV1 Source Data [file 44321_2025_315_MOESM9_ESM.zip › Figure EV1/EV1D/2-CD31-GLDC/LEE III/5 (4).jpg]

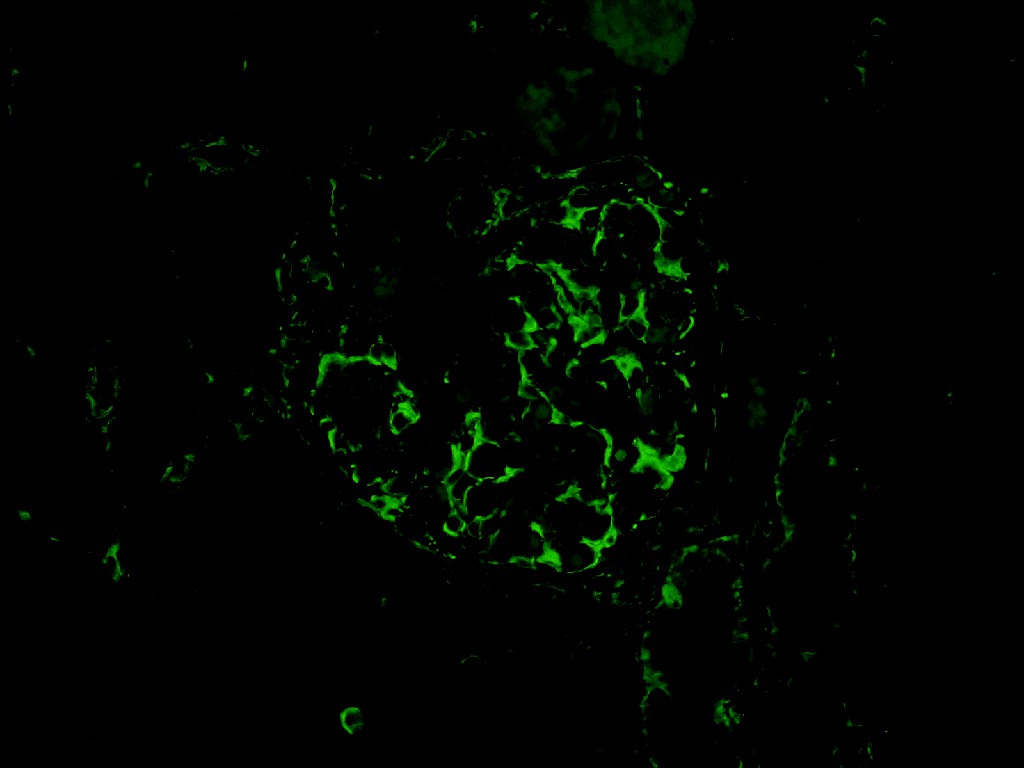

Supplement: Supplementary file 9 — Figure EV1 Source Data [file 44321_2025_315_MOESM9_ESM.zip › Figure EV1/EV1D/2-CD31-GLDC/LEE III/2 (1).jpg]

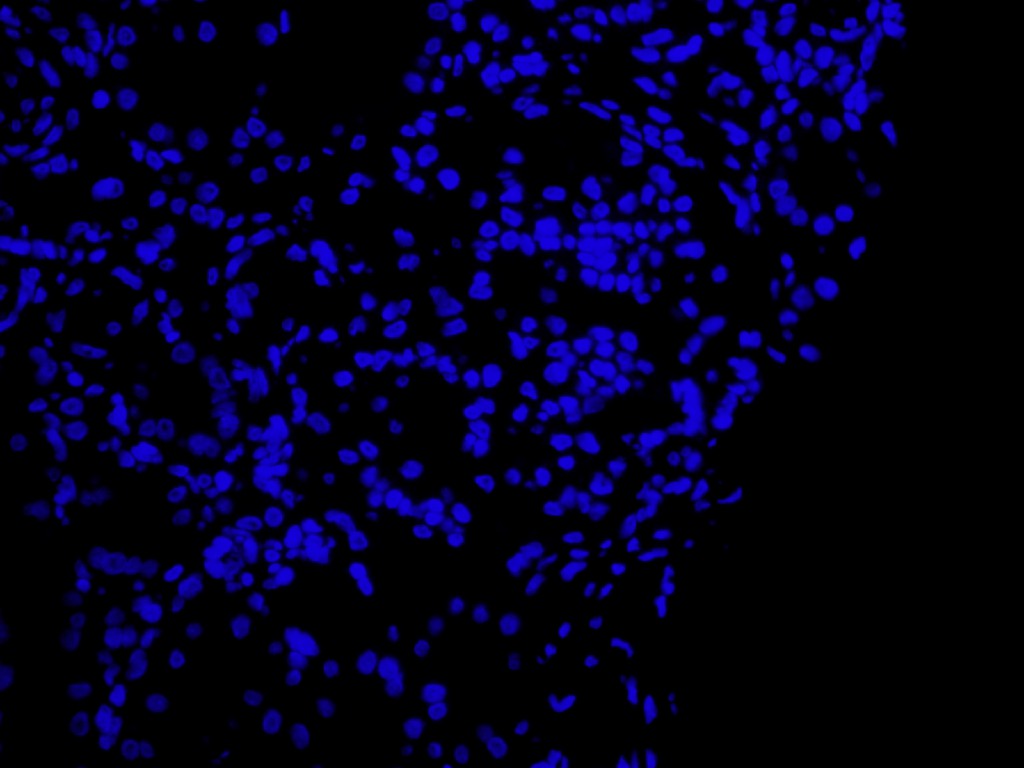

Supplement: Supplementary file 9 — Figure EV1 Source Data [file 44321_2025_315_MOESM9_ESM.zip › Figure EV1/EV1D/2-CD31-GLDC/LEE III/6 (3).jpg]

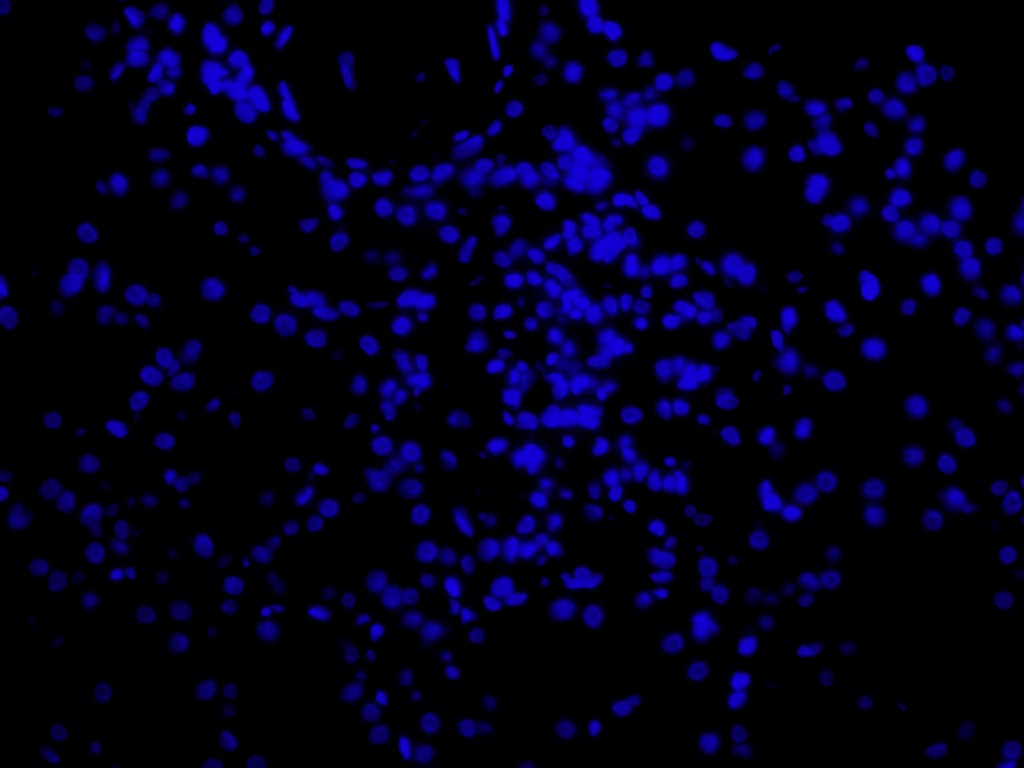

Supplement: Supplementary file 9 — Figure EV1 Source Data [file 44321_2025_315_MOESM9_ESM.zip › Figure EV1/EV1D/2-CD31-GLDC/LEE III/7 (2).jpg]

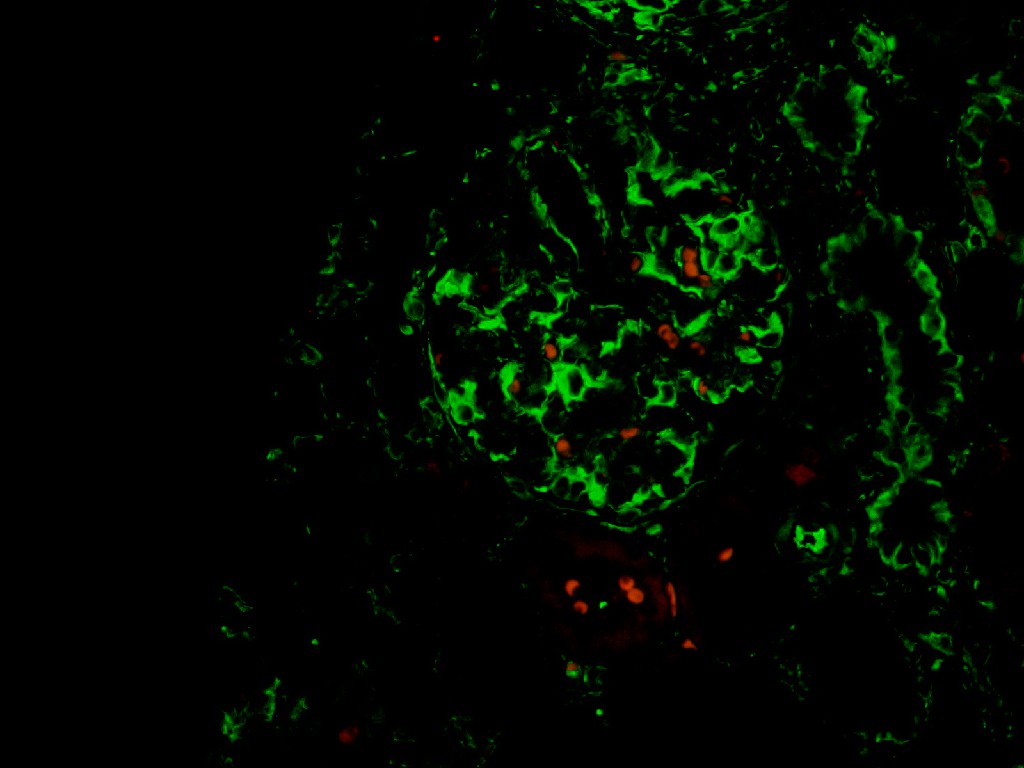

Supplement: Supplementary file 9 — Figure EV1 Source Data [file 44321_2025_315_MOESM9_ESM.zip › Figure EV1/EV1D/2-CD31-GLDC/LEE III/4 (4).jpg]

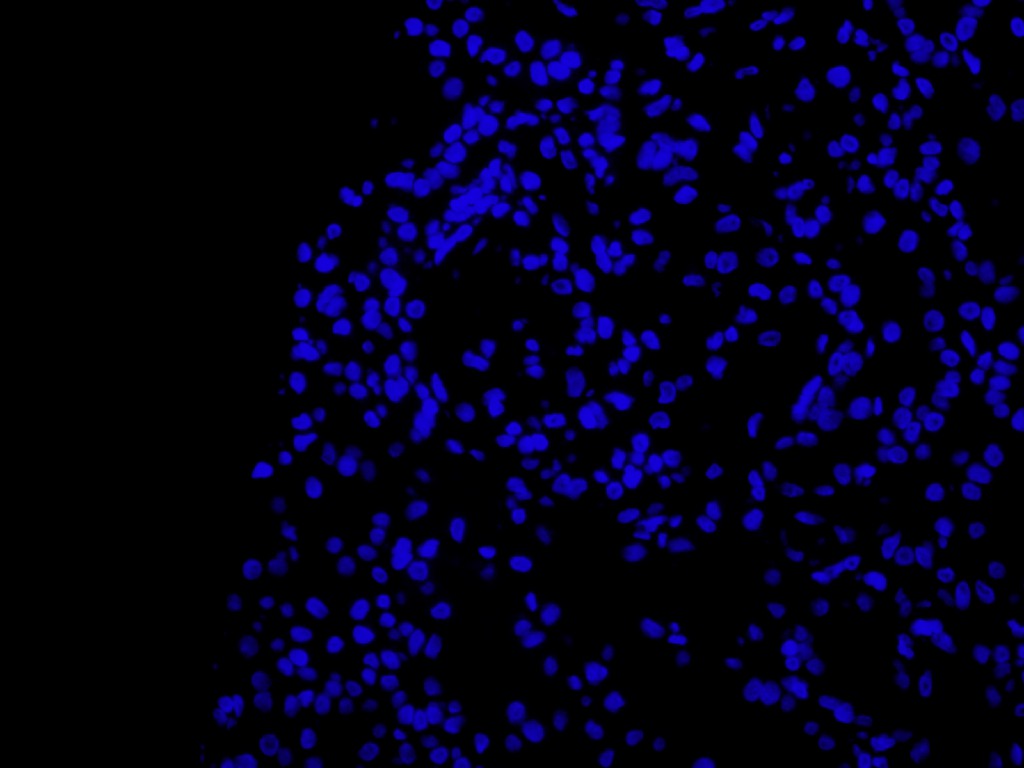

Supplement: Supplementary file 9 — Figure EV1 Source Data [file 44321_2025_315_MOESM9_ESM.zip › Figure EV1/EV1D/2-CD31-GLDC/LEE III/4 (3).jpg]

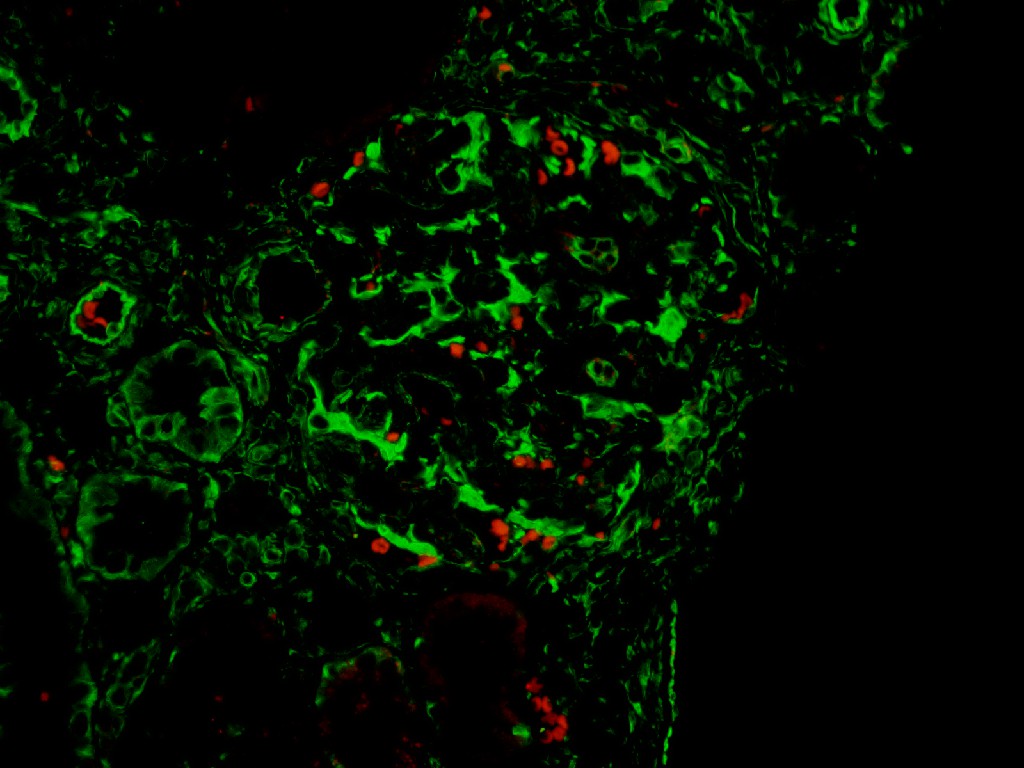

Supplement: Supplementary file 9 — Figure EV1 Source Data [file 44321_2025_315_MOESM9_ESM.zip › Figure EV1/EV1D/2-CD31-GLDC/LEE III/6 (4).jpg]

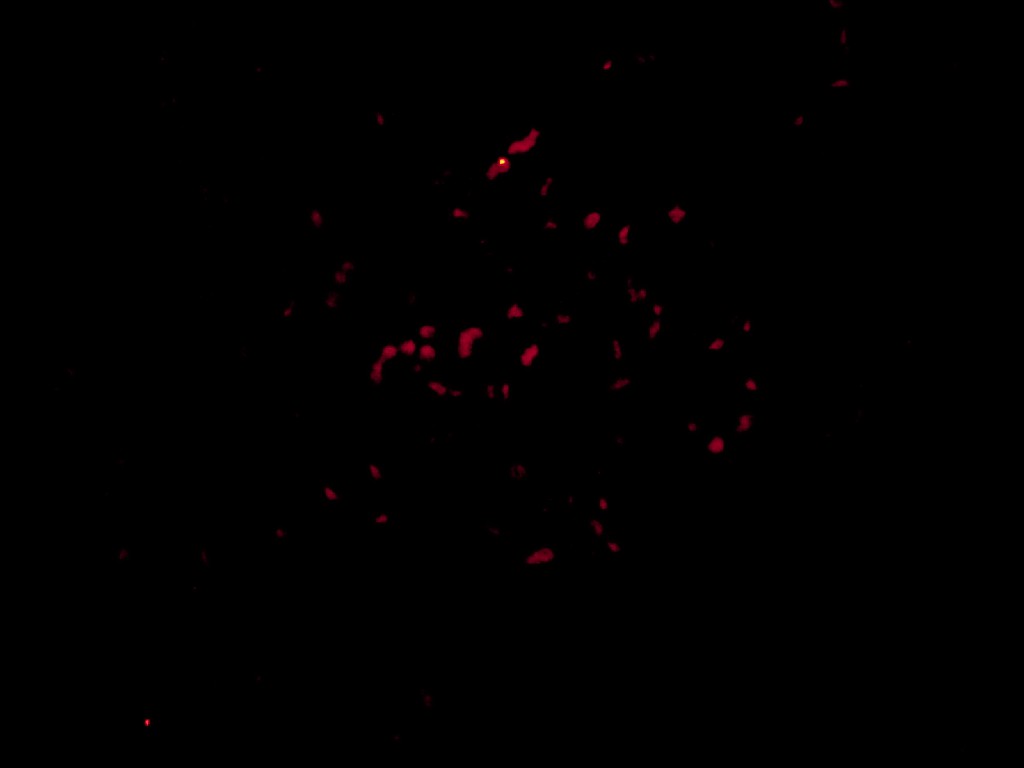

Supplement: Supplementary file 9 — Figure EV1 Source Data [file 44321_2025_315_MOESM9_ESM.zip › Figure EV1/EV1D/2-CD31-GLDC/LEE III/5 (2).jpg]

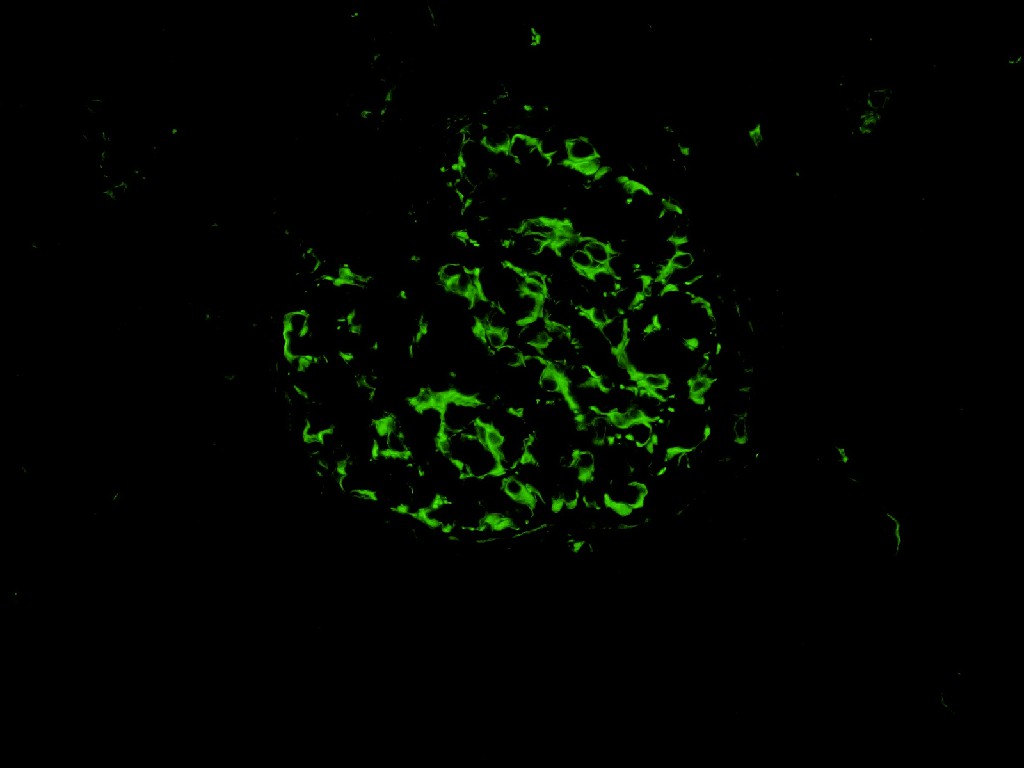

Supplement: Supplementary file 9 — Figure EV1 Source Data [file 44321_2025_315_MOESM9_ESM.zip › Figure EV1/EV1D/2-CD31-GLDC/LEE III/9 (1).jpg]

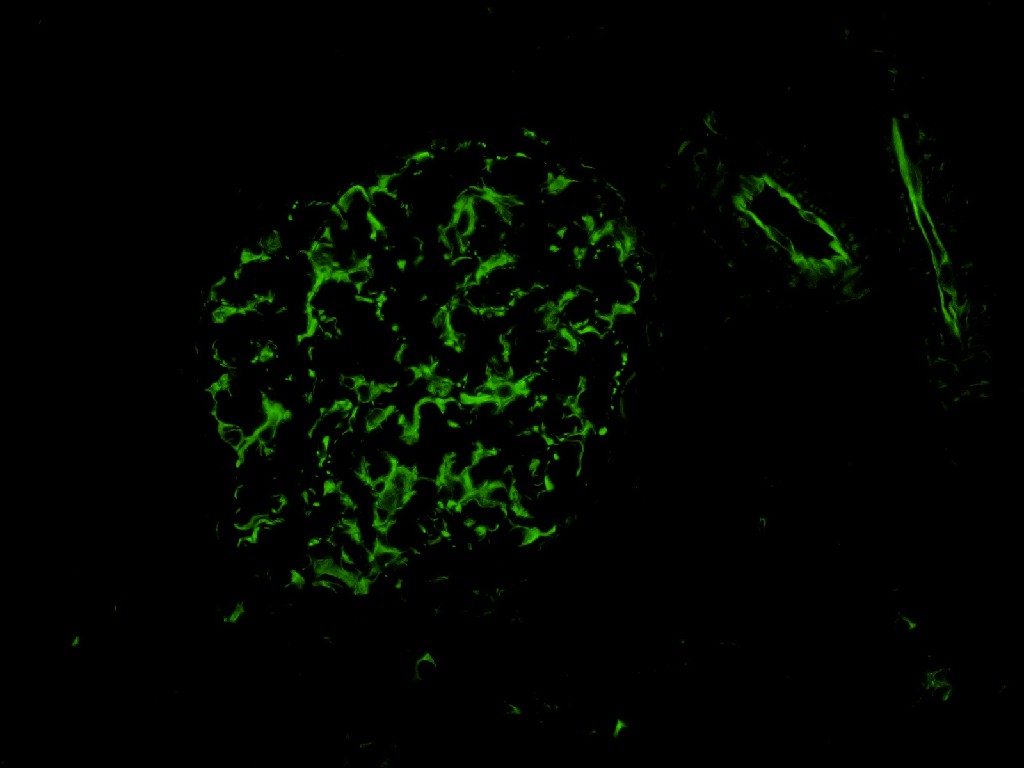

Supplement: Supplementary file 9 — Figure EV1 Source Data [file 44321_2025_315_MOESM9_ESM.zip › Figure EV1/EV1D/2-CD31-GLDC/LEE III/8 (1).jpg]

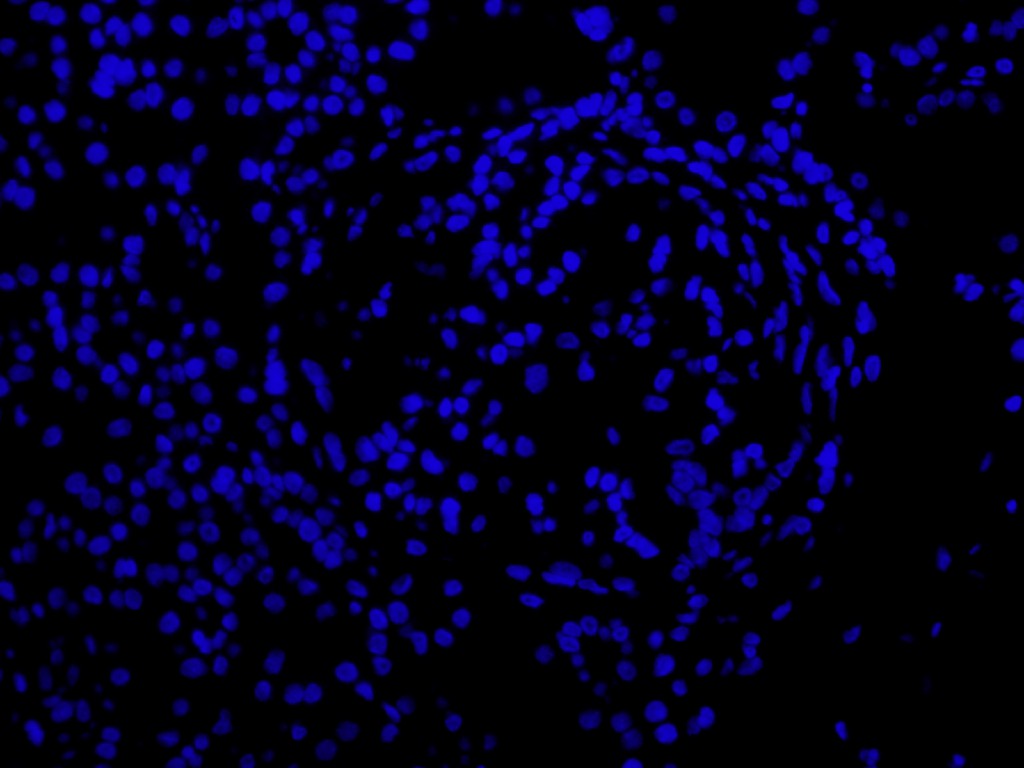

Supplement: Supplementary file 9 — Figure EV1 Source Data [file 44321_2025_315_MOESM9_ESM.zip › Figure EV1/EV1D/2-CD31-GLDC/LEE III/5 (3).jpg]

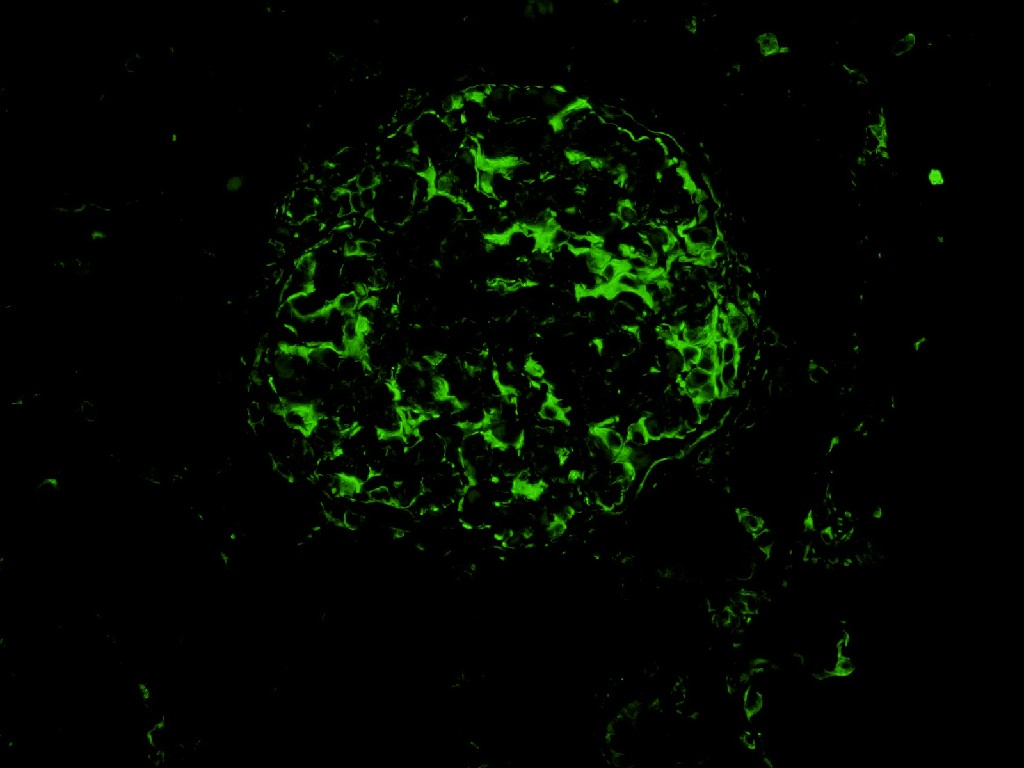

Supplement: Supplementary file 9 — Figure EV1 Source Data [file 44321_2025_315_MOESM9_ESM.zip › Figure EV1/EV1D/2-CD31-GLDC/LEE III/1 (1).jpg]

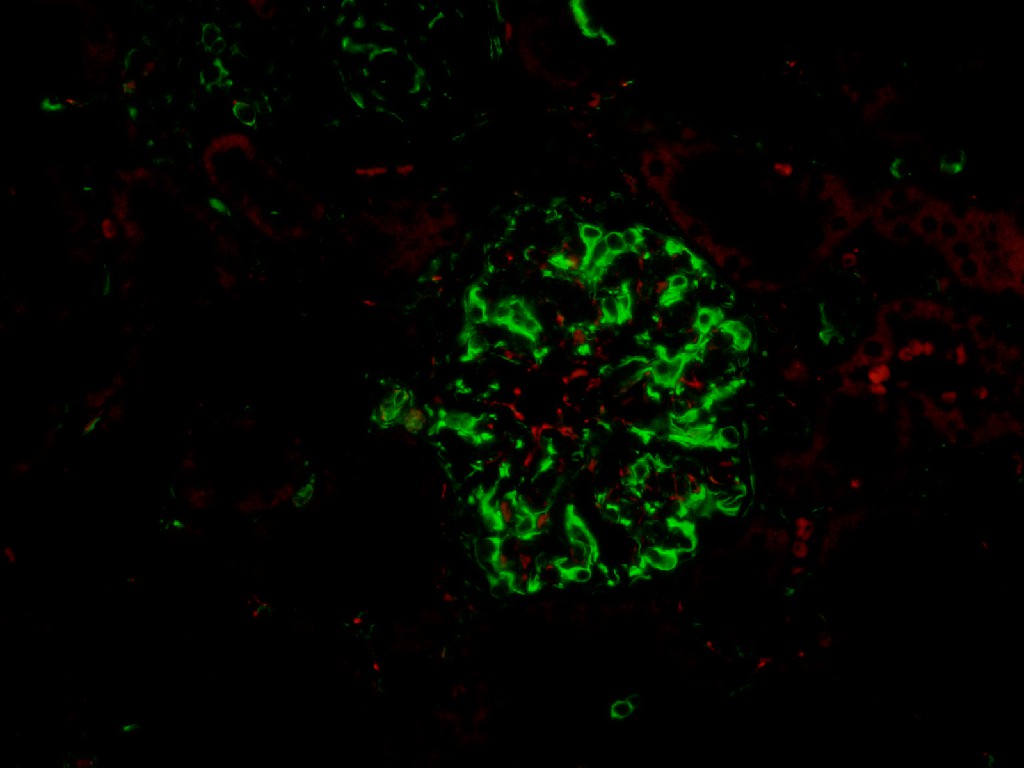

Supplement: Supplementary file 9 — Figure EV1 Source Data [file 44321_2025_315_MOESM9_ESM.zip › Figure EV1/EV1D/2-CD31-GLDC/LEE III/7 (4).jpg]

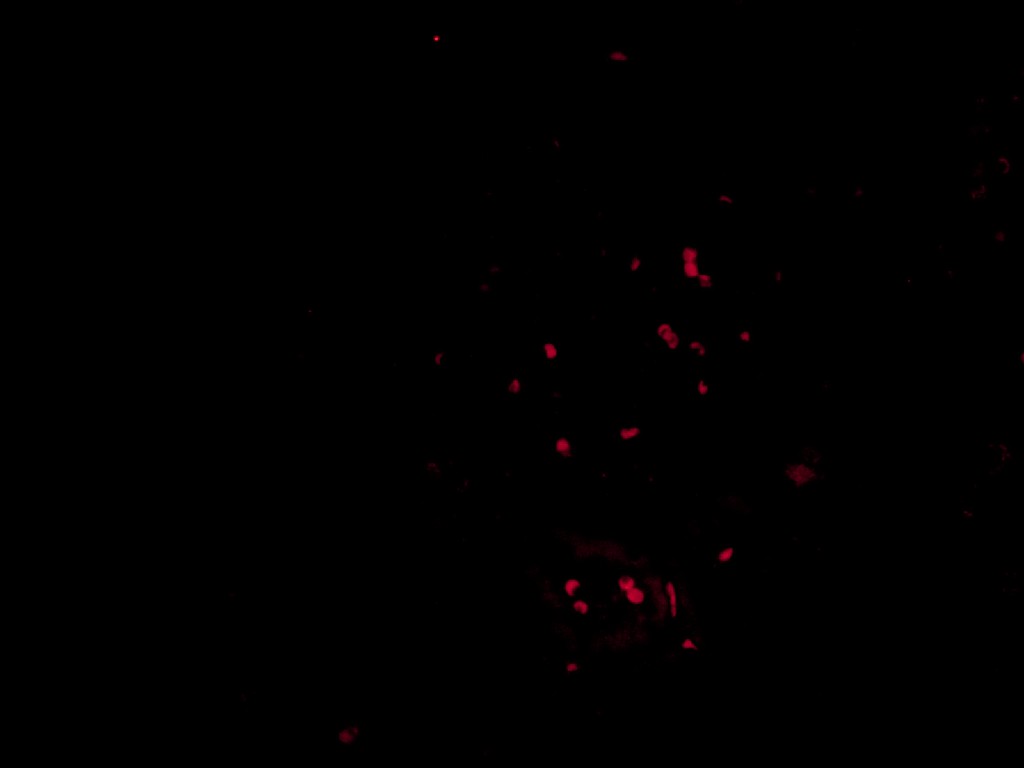

Supplement: Supplementary file 9 — Figure EV1 Source Data [file 44321_2025_315_MOESM9_ESM.zip › Figure EV1/EV1D/2-CD31-GLDC/LEE III/4 (2).jpg]

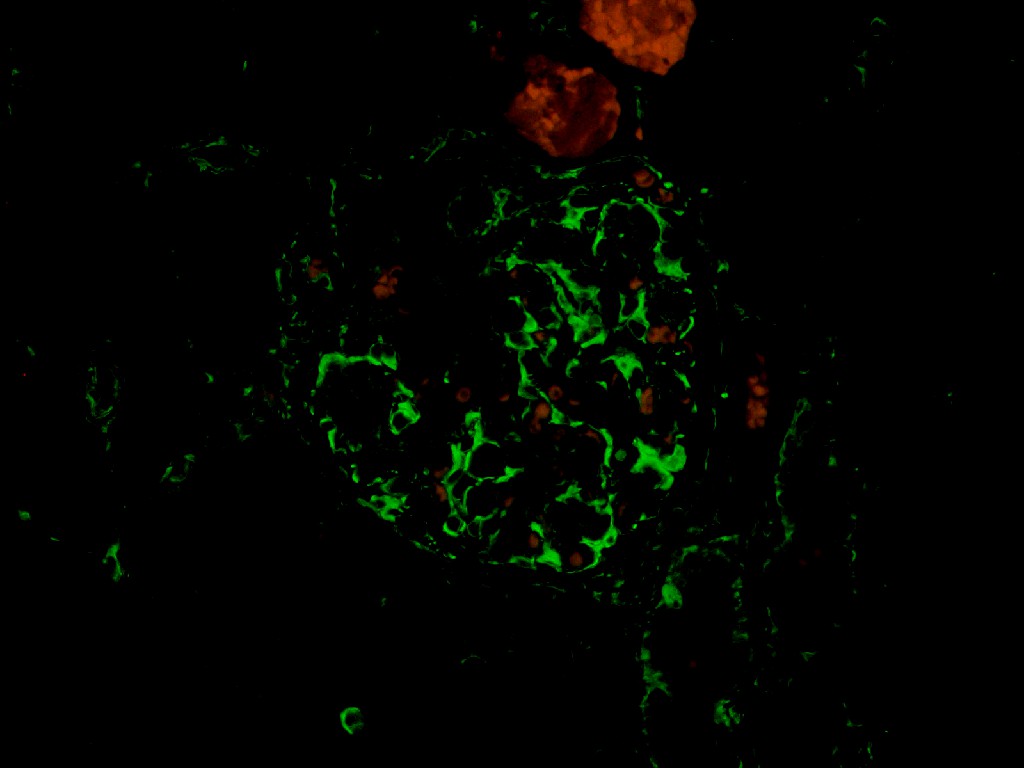

Supplement: Supplementary file 9 — Figure EV1 Source Data [file 44321_2025_315_MOESM9_ESM.zip › Figure EV1/EV1D/2-CD31-GLDC/LEE III/2 (4).jpg]

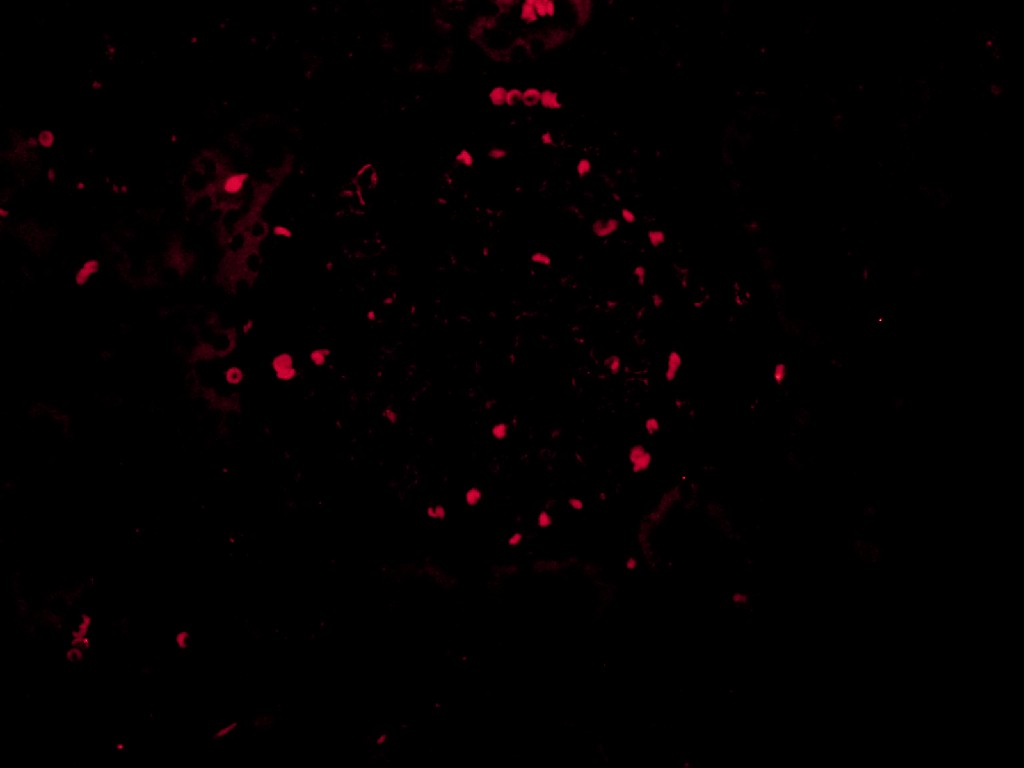

Supplement: Supplementary file 9 — Figure EV1 Source Data [file 44321_2025_315_MOESM9_ESM.zip › Figure EV1/EV1D/2-CD31-GLDC/LEE III/1 (2).jpg]

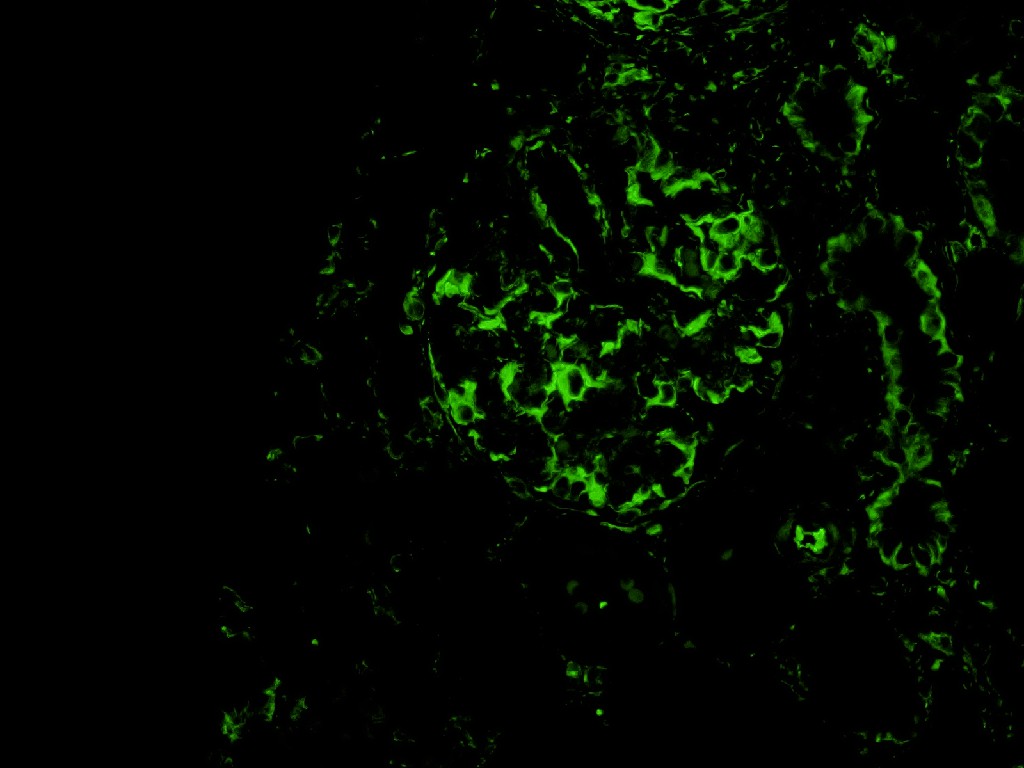

Supplement: Supplementary file 9 — Figure EV1 Source Data [file 44321_2025_315_MOESM9_ESM.zip › Figure EV1/EV1D/2-CD31-GLDC/LEE III/4 (1).jpg]

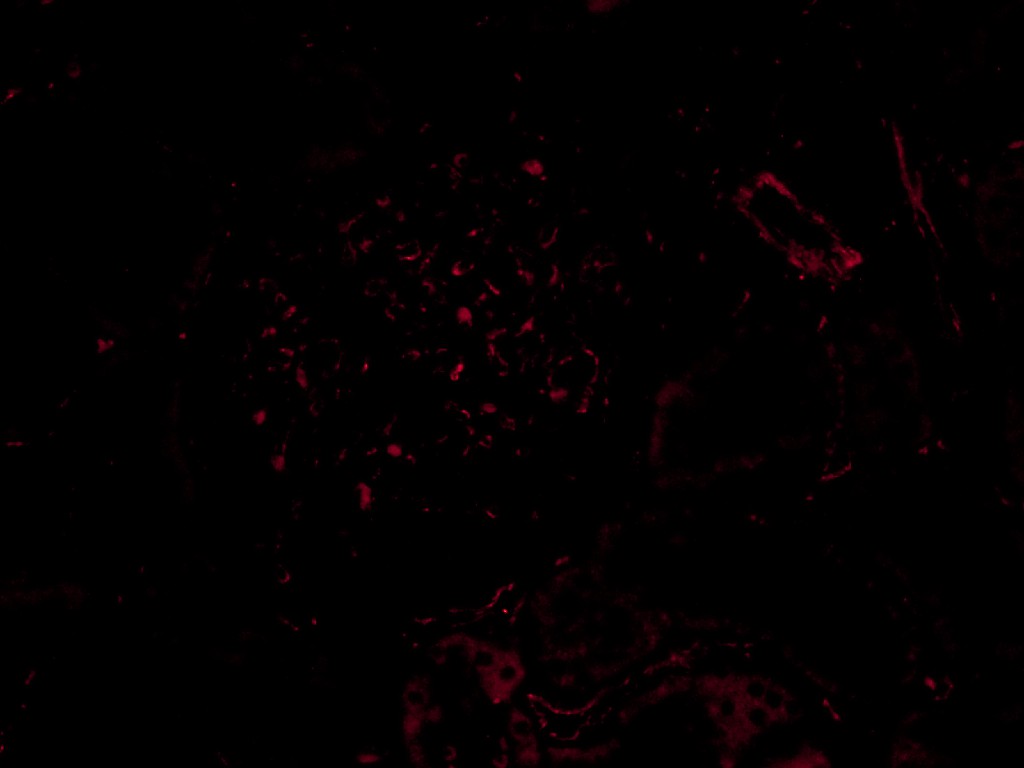

Supplement: Supplementary file 9 — Figure EV1 Source Data [file 44321_2025_315_MOESM9_ESM.zip › Figure EV1/EV1D/2-CD31-GLDC/LEE III/8 (2).jpg]

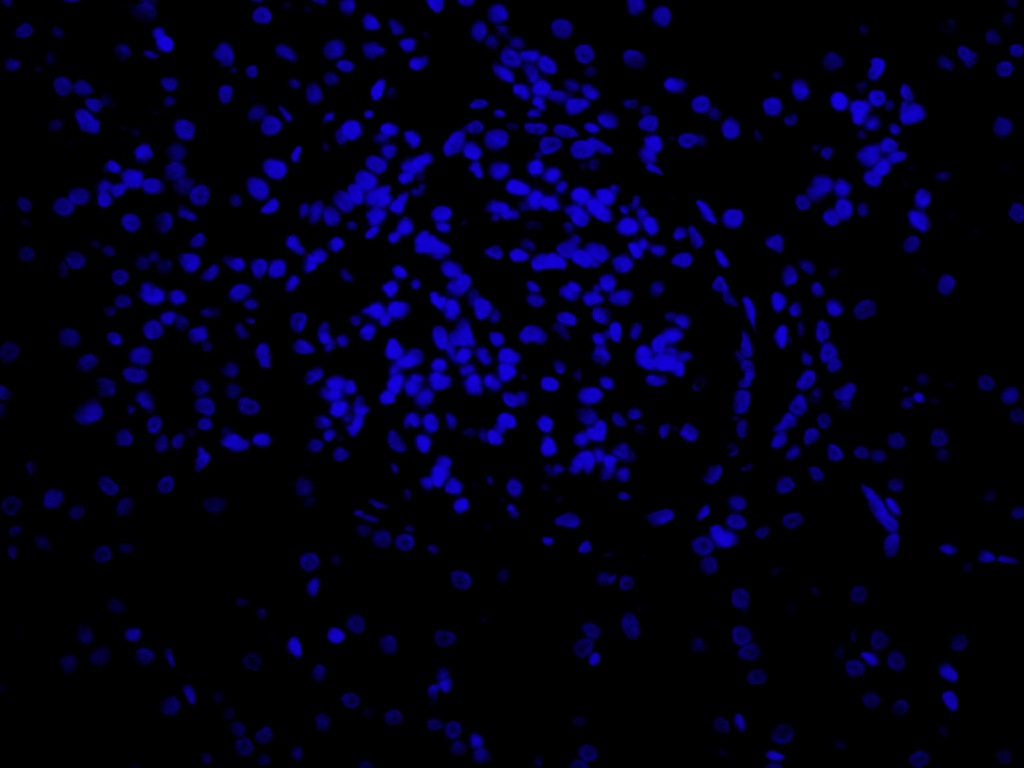

Supplement: Supplementary file 9 — Figure EV1 Source Data [file 44321_2025_315_MOESM9_ESM.zip › Figure EV1/EV1D/2-CD31-GLDC/LEE III/9 (3).jpg]

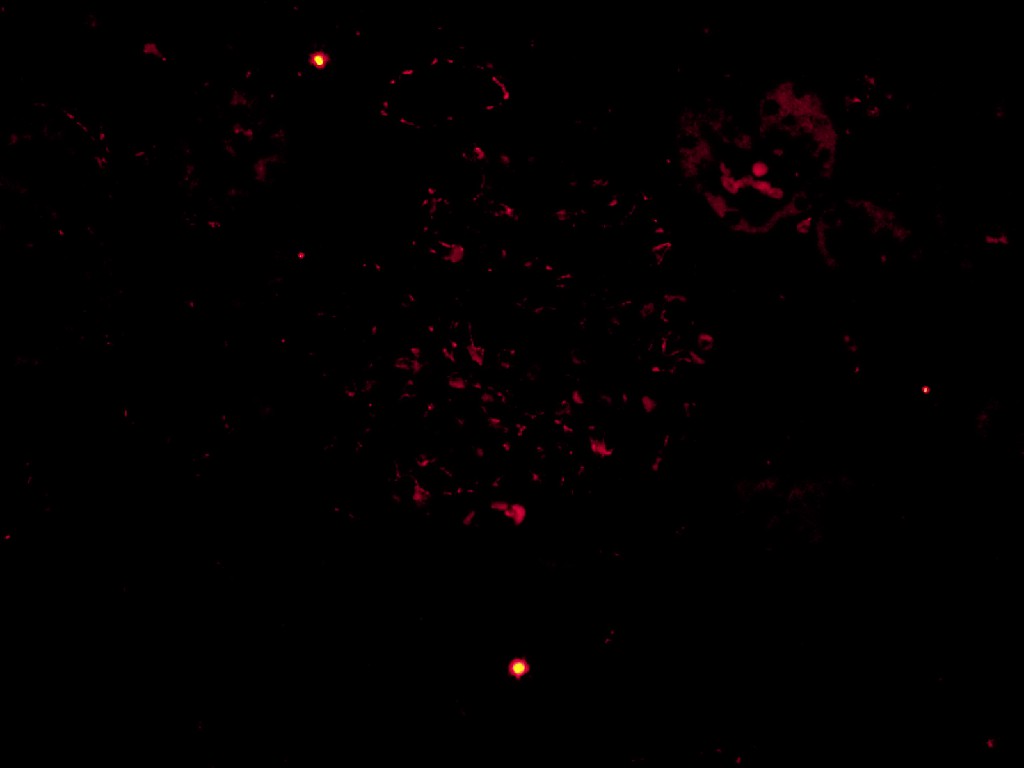

Supplement: Supplementary file 9 — Figure EV1 Source Data [file 44321_2025_315_MOESM9_ESM.zip › Figure EV1/EV1D/2-CD31-GLDC/LEE III/9 (2).jpg]

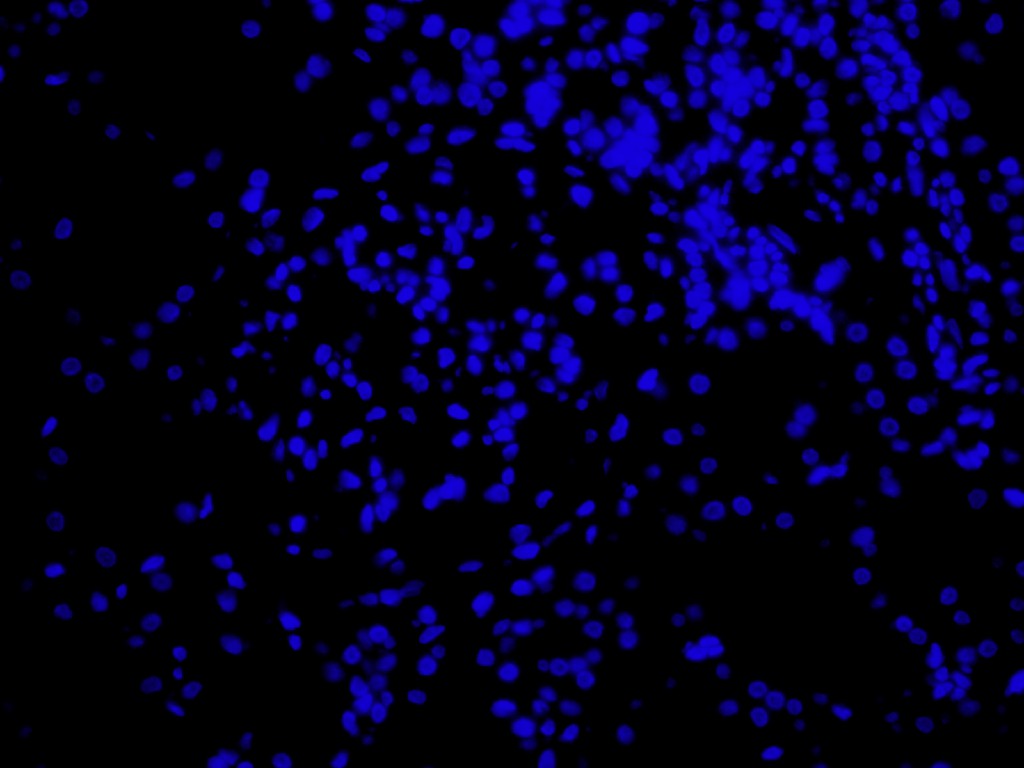

Supplement: Supplementary file 9 — Figure EV1 Source Data [file 44321_2025_315_MOESM9_ESM.zip › Figure EV1/EV1D/2-CD31-GLDC/LEE III/8 (3).jpg]

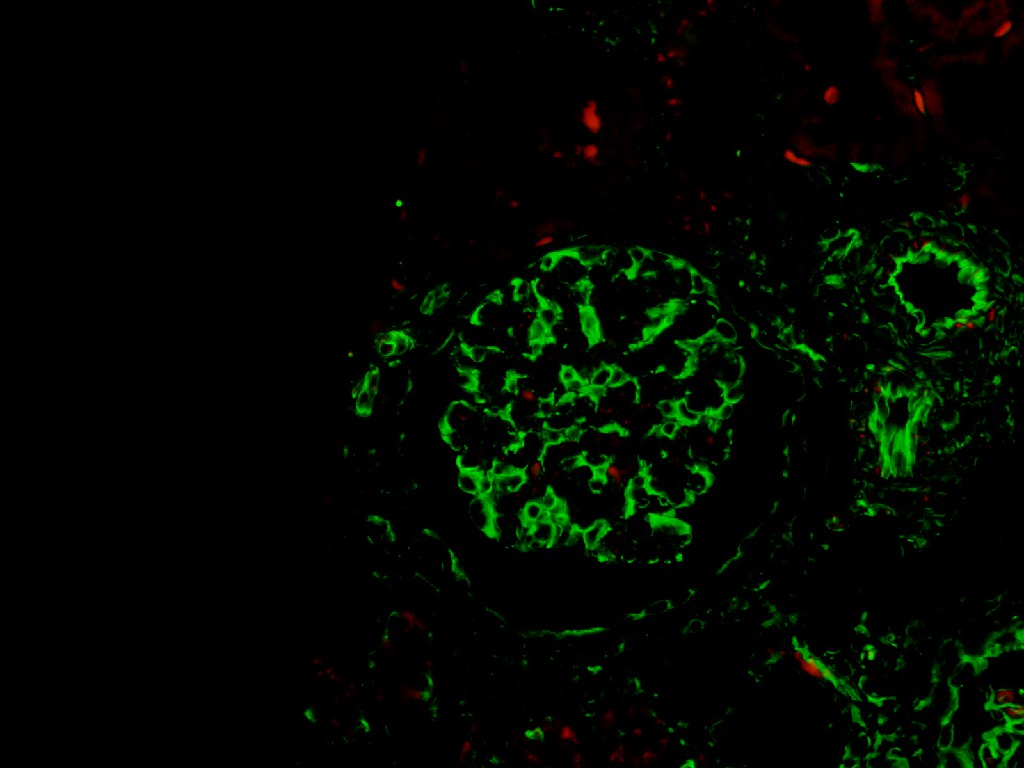

Supplement: Supplementary file 9 — Figure EV1 Source Data [file 44321_2025_315_MOESM9_ESM.zip › Figure EV1/EV1D/2-CD31-GLDC/LEE III/3 (4).jpg]

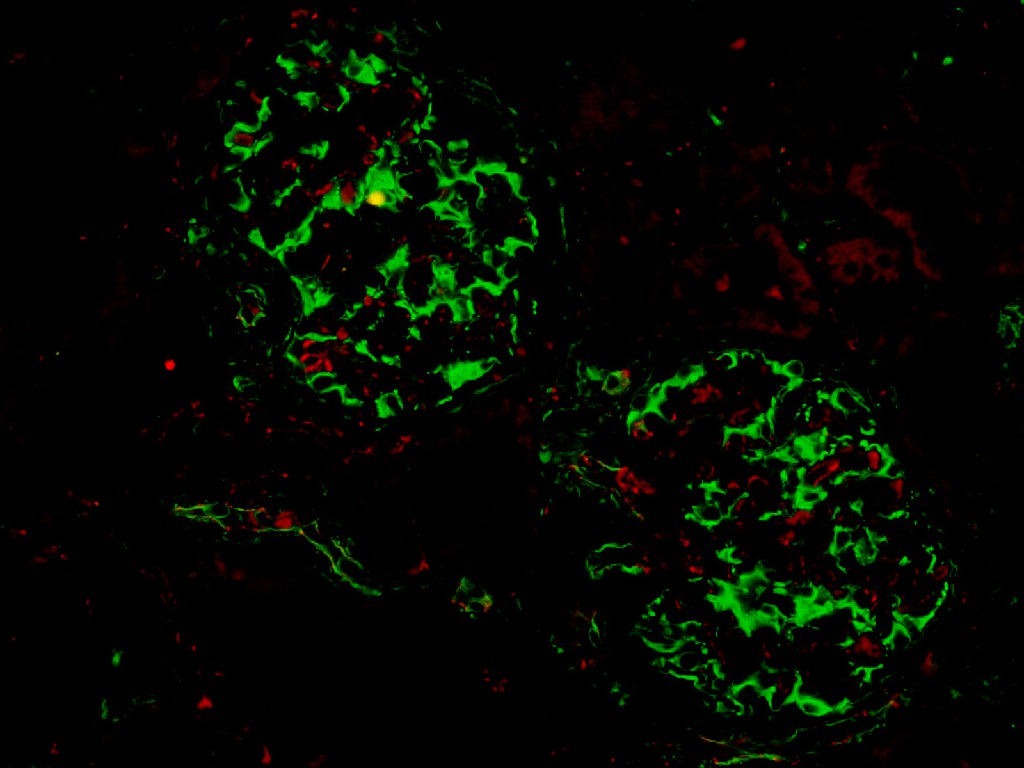

Supplement: Supplementary file 9 — Figure EV1 Source Data [file 44321_2025_315_MOESM9_ESM.zip › Figure EV1/EV1D/2-CD31-GLDC/LEE III/10 (4).jpg]

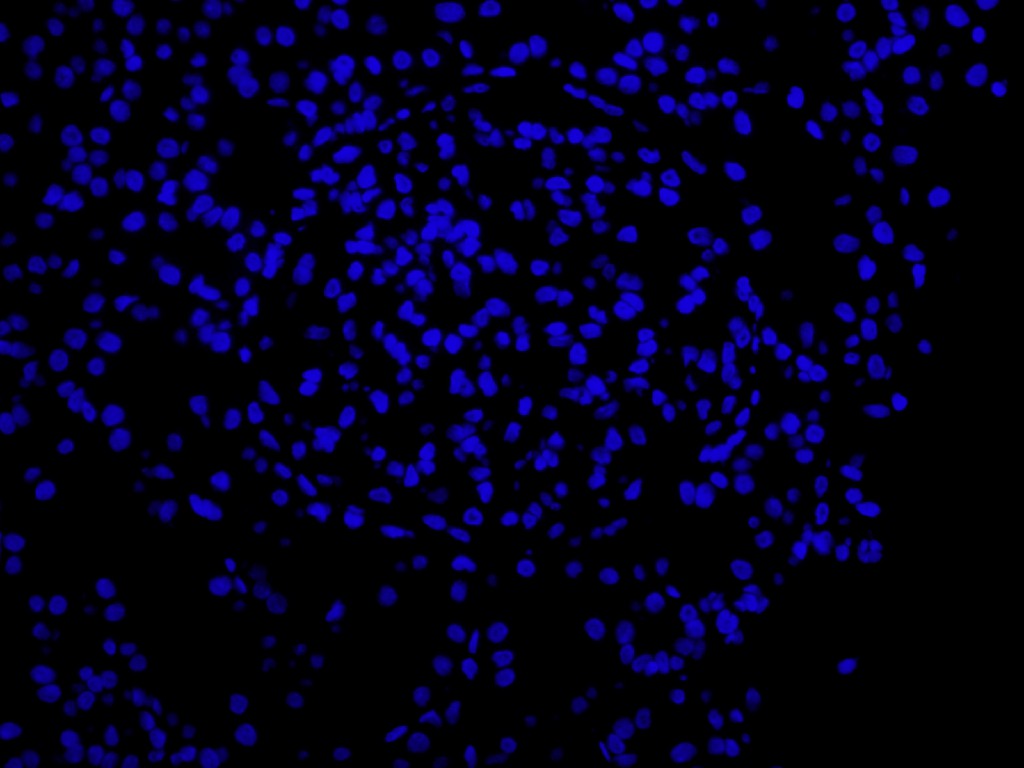

Supplement: Supplementary file 9 — Figure EV1 Source Data [file 44321_2025_315_MOESM9_ESM.zip › Figure EV1/EV1D/2-CD31-GLDC/LEE III/1 (3).jpg]

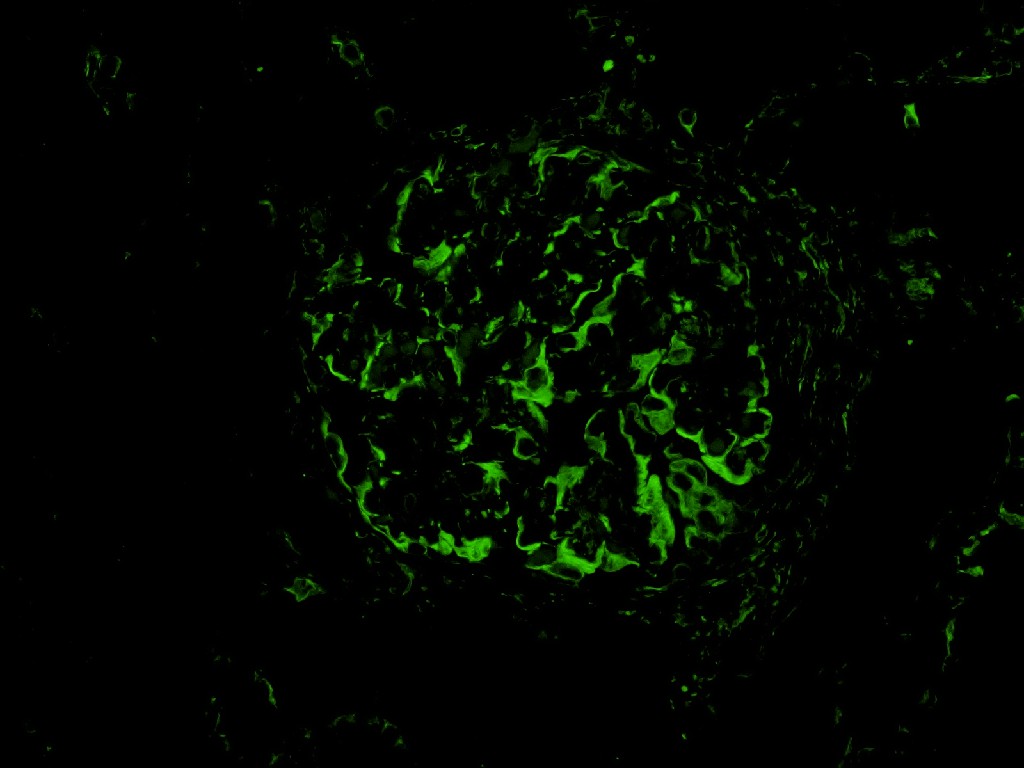

Supplement: Supplementary file 9 — Figure EV1 Source Data [file 44321_2025_315_MOESM9_ESM.zip › Figure EV1/EV1D/2-CD31-GLDC/LEE III/5 (1).jpg]

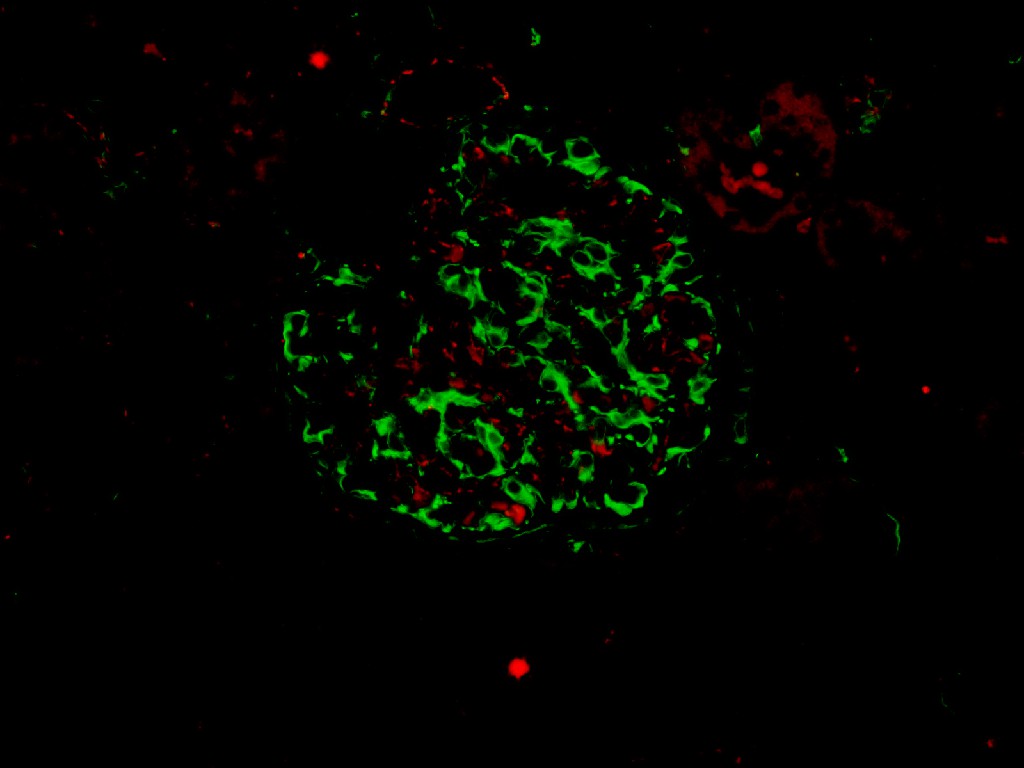

Supplement: Supplementary file 9 — Figure EV1 Source Data [file 44321_2025_315_MOESM9_ESM.zip › Figure EV1/EV1D/2-CD31-GLDC/LEE III/8 (4).jpg]

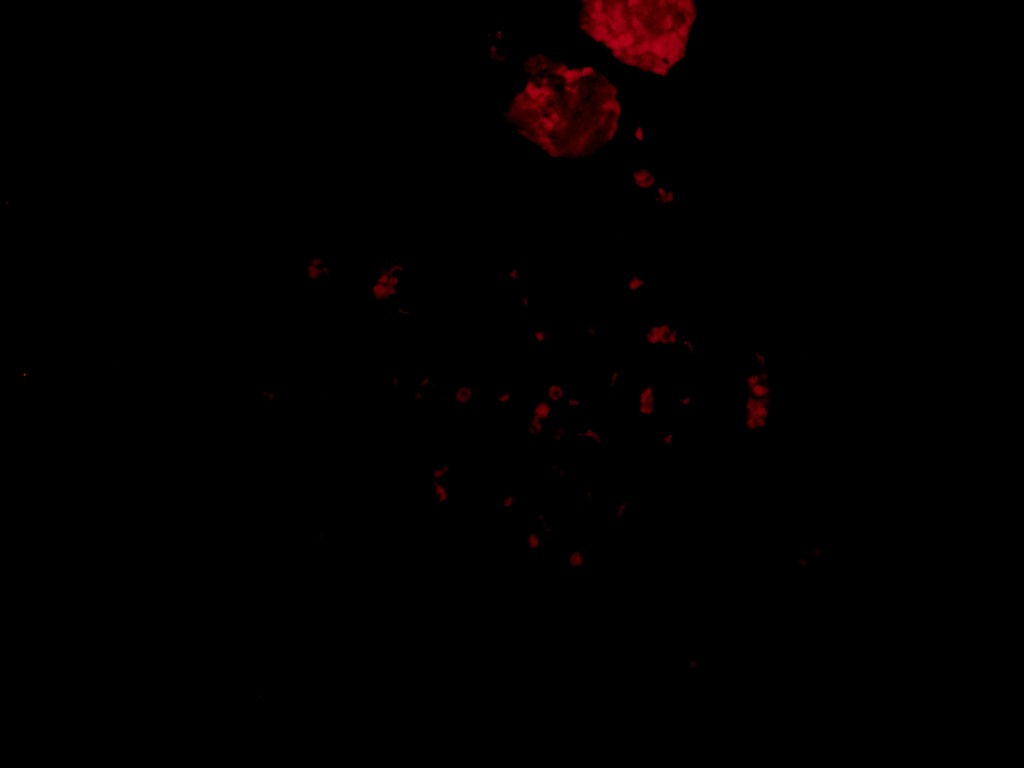

Supplement: Supplementary file 9 — Figure EV1 Source Data [file 44321_2025_315_MOESM9_ESM.zip › Figure EV1/EV1D/2-CD31-GLDC/LEE III/2 (2).jpg]

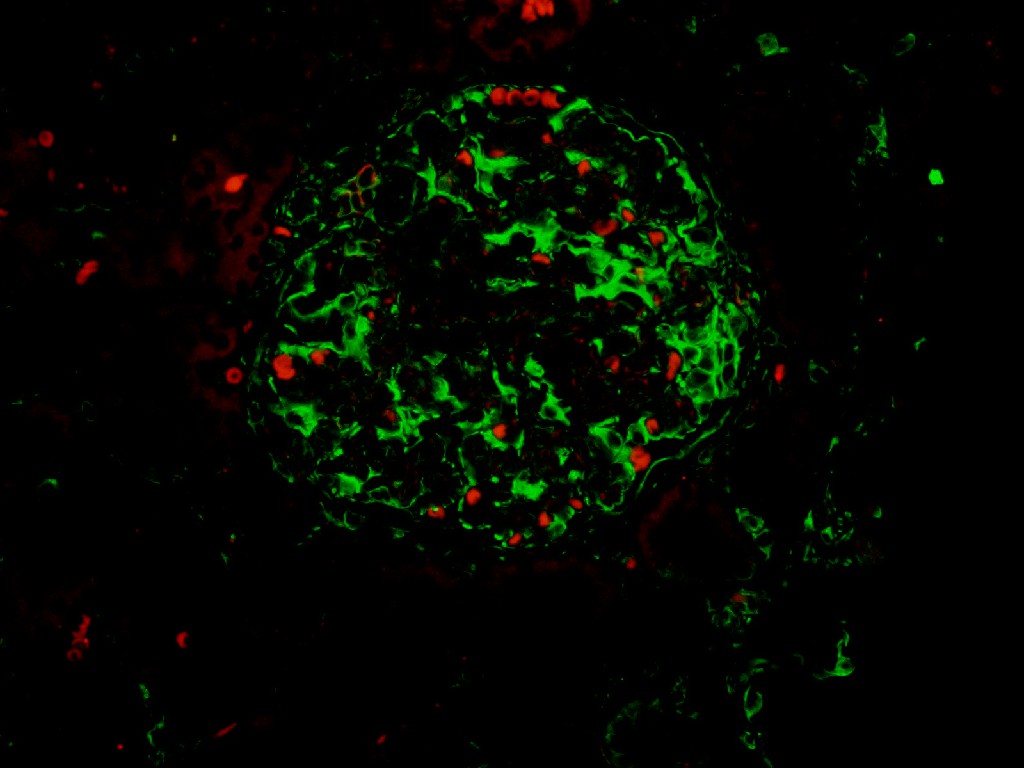

Supplement: Supplementary file 9 — Figure EV1 Source Data [file 44321_2025_315_MOESM9_ESM.zip › Figure EV1/EV1D/2-CD31-GLDC/LEE III/1 (4).jpg]

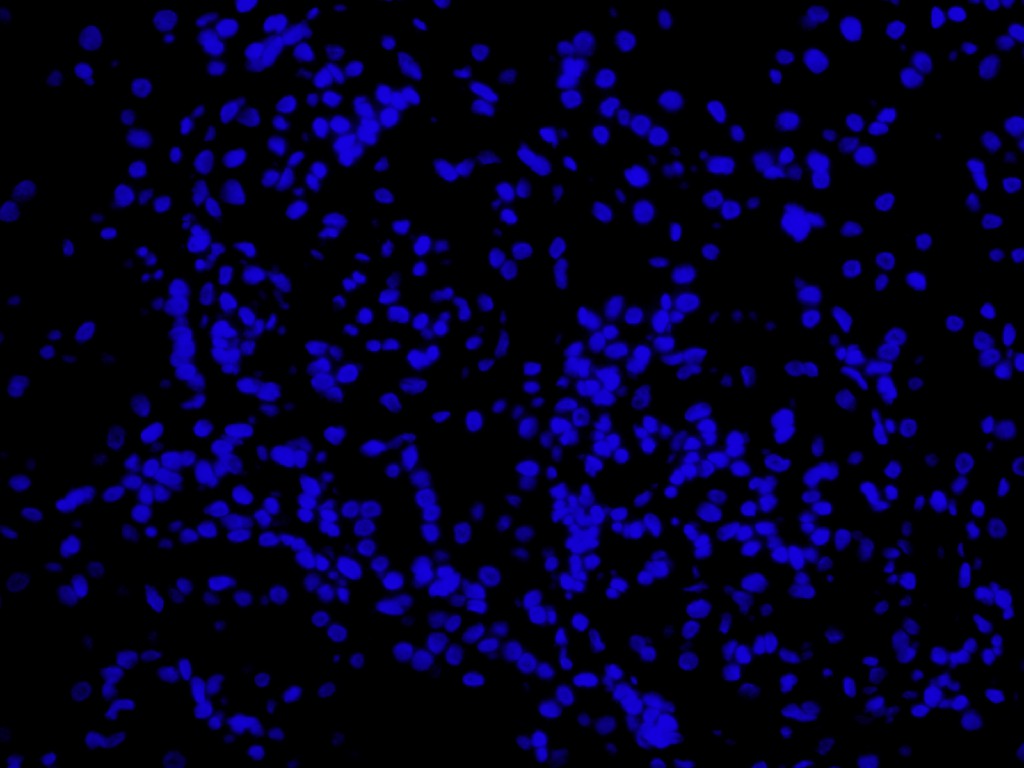

Supplement: Supplementary file 9 — Figure EV1 Source Data [file 44321_2025_315_MOESM9_ESM.zip › Figure EV1/EV1D/2-CD31-GLDC/LEE III/10 (3).jpg]

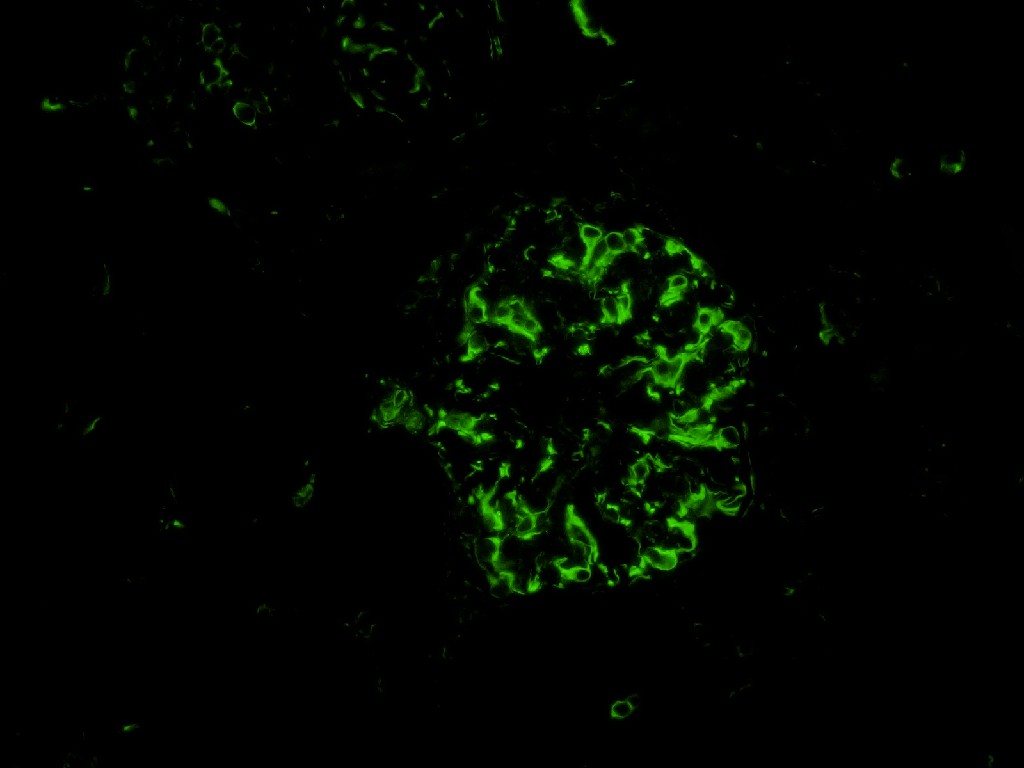

Supplement: Supplementary file 9 — Figure EV1 Source Data [file 44321_2025_315_MOESM9_ESM.zip › Figure EV1/EV1D/2-CD31-GLDC/LEE III/7 (1).jpg]

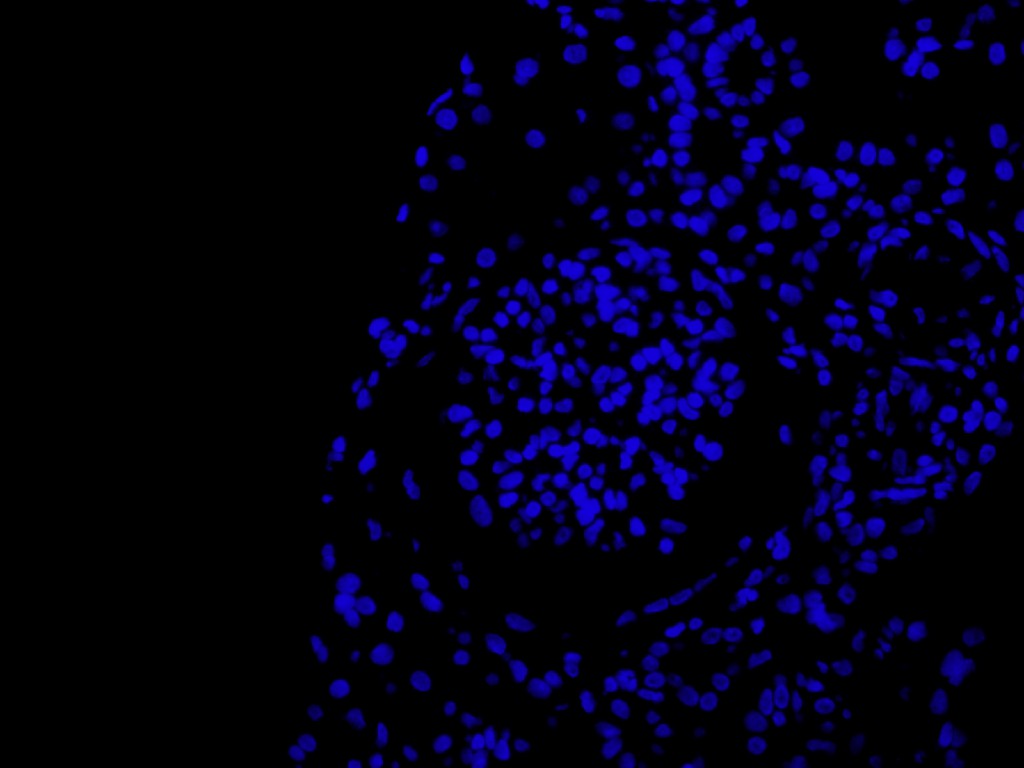

Supplement: Supplementary file 9 — Figure EV1 Source Data [file 44321_2025_315_MOESM9_ESM.zip › Figure EV1/EV1D/2-CD31-GLDC/LEE III/3 (3).jpg]

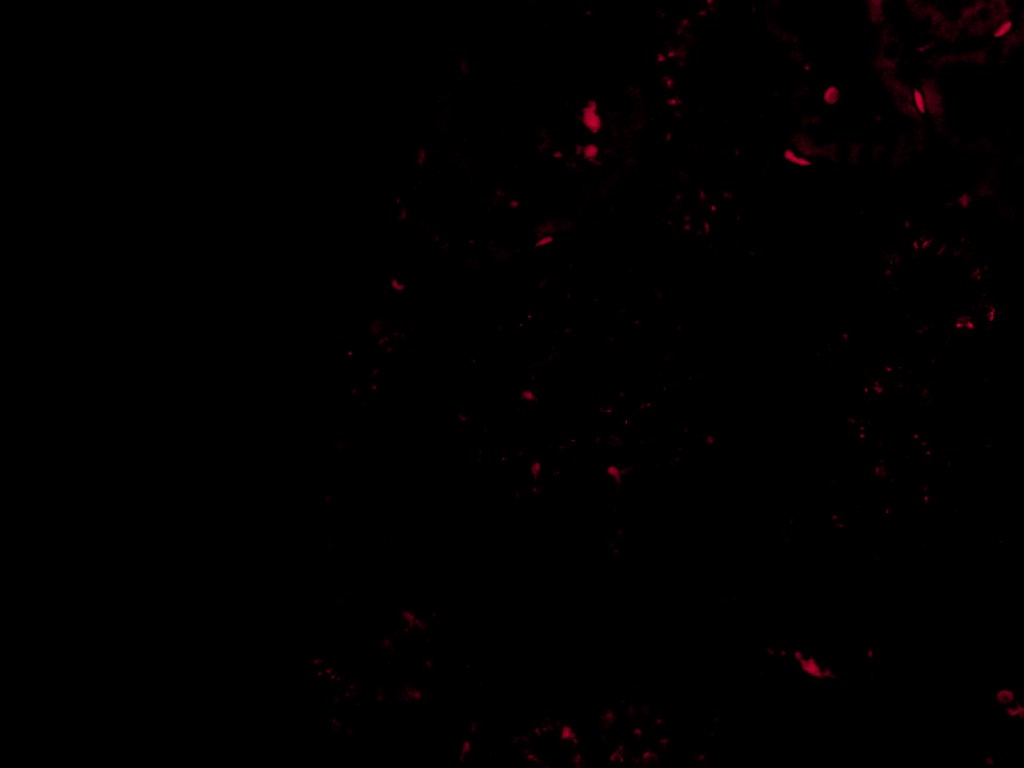

Supplement: Supplementary file 9 — Figure EV1 Source Data [file 44321_2025_315_MOESM9_ESM.zip › Figure EV1/EV1D/2-CD31-GLDC/LEE III/3 (2).jpg]

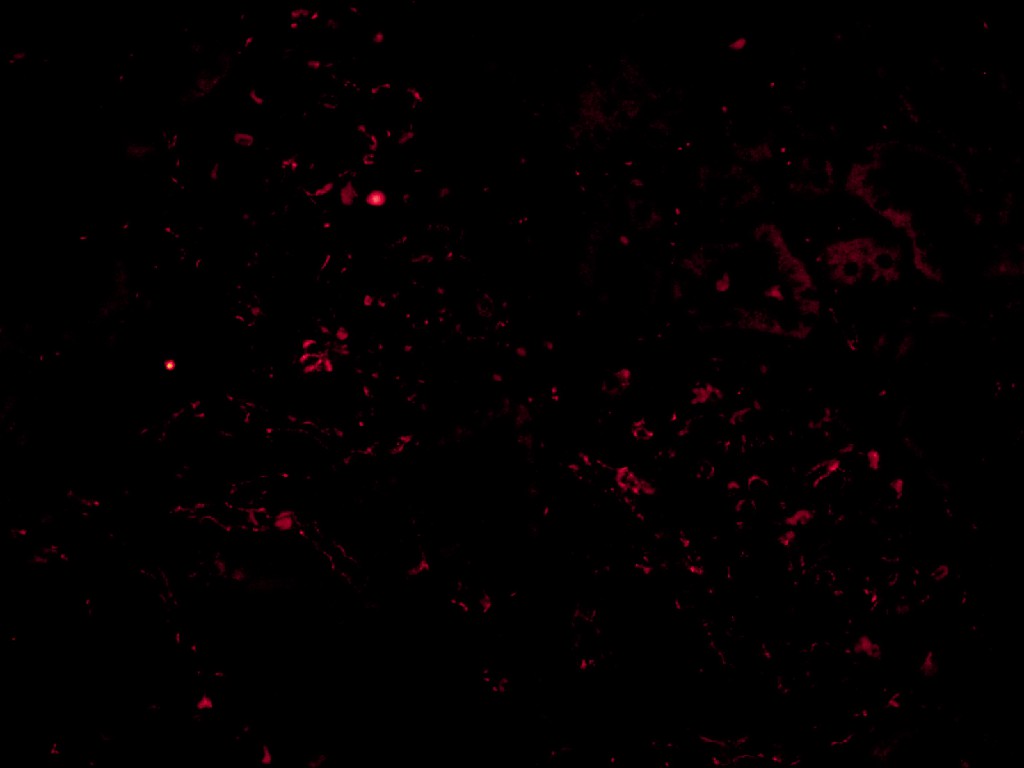

Supplement: Supplementary file 9 — Figure EV1 Source Data [file 44321_2025_315_MOESM9_ESM.zip › Figure EV1/EV1D/2-CD31-GLDC/LEE III/10 (2).jpg]

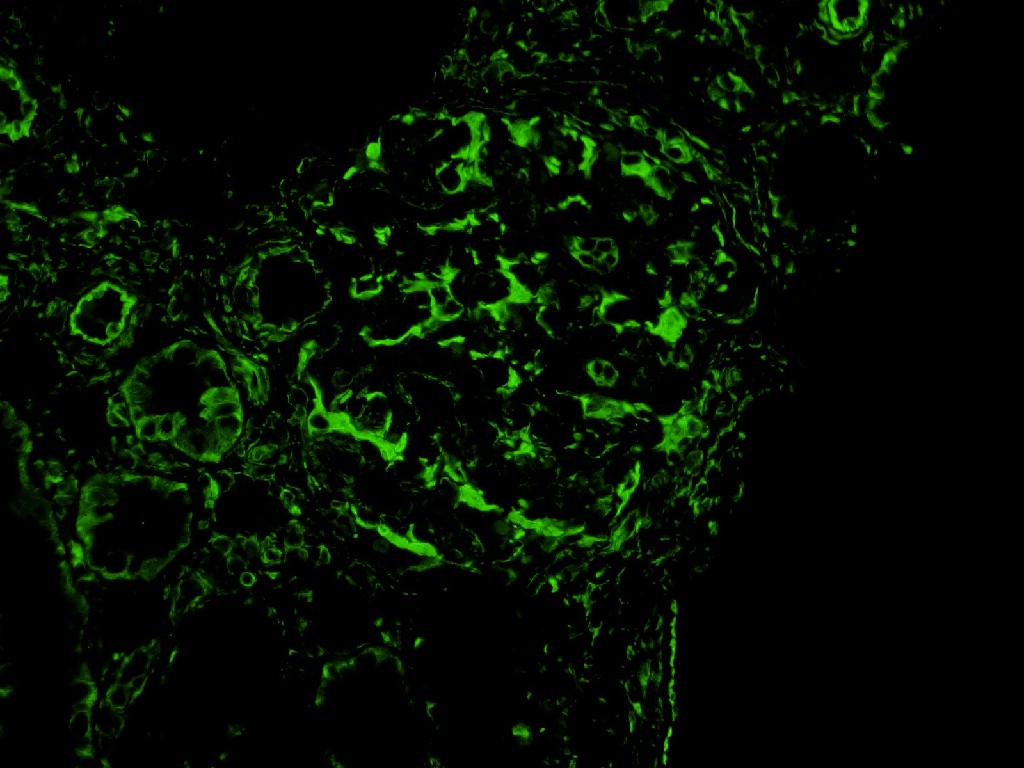

Supplement: Supplementary file 9 — Figure EV1 Source Data [file 44321_2025_315_MOESM9_ESM.zip › Figure EV1/EV1D/2-CD31-GLDC/LEE III/6 (1).jpg]

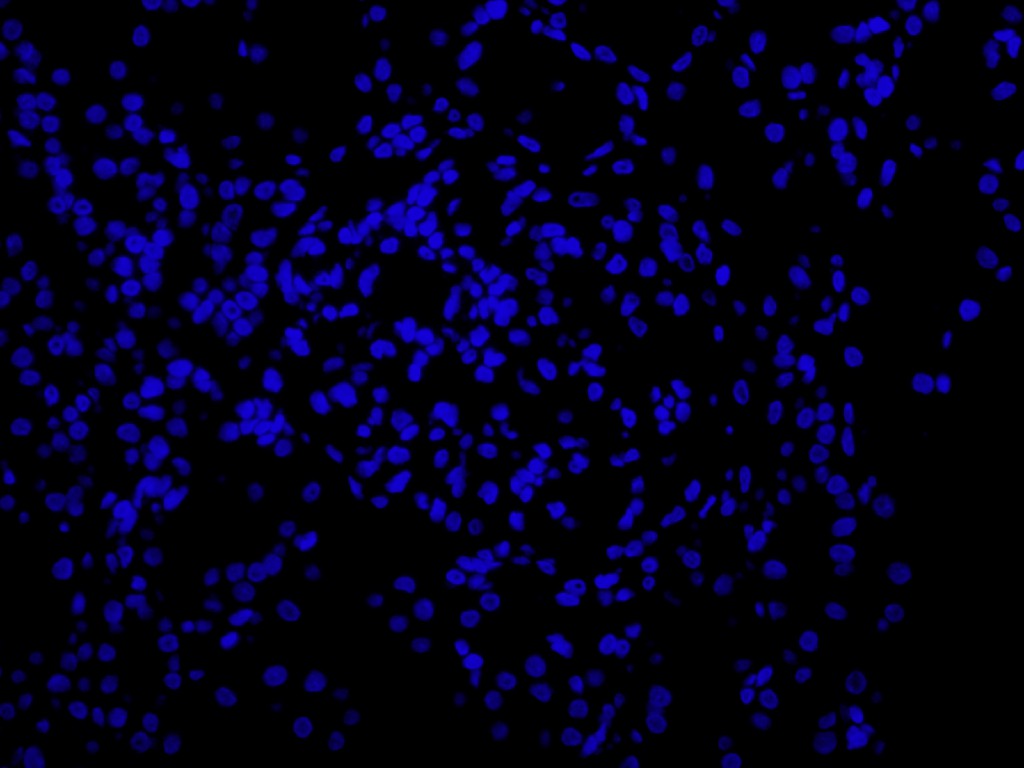

Supplement: Supplementary file 9 — Figure EV1 Source Data [file 44321_2025_315_MOESM9_ESM.zip › Figure EV1/EV1D/2-CD31-GLDC/LEE III/2 (3).jpg]

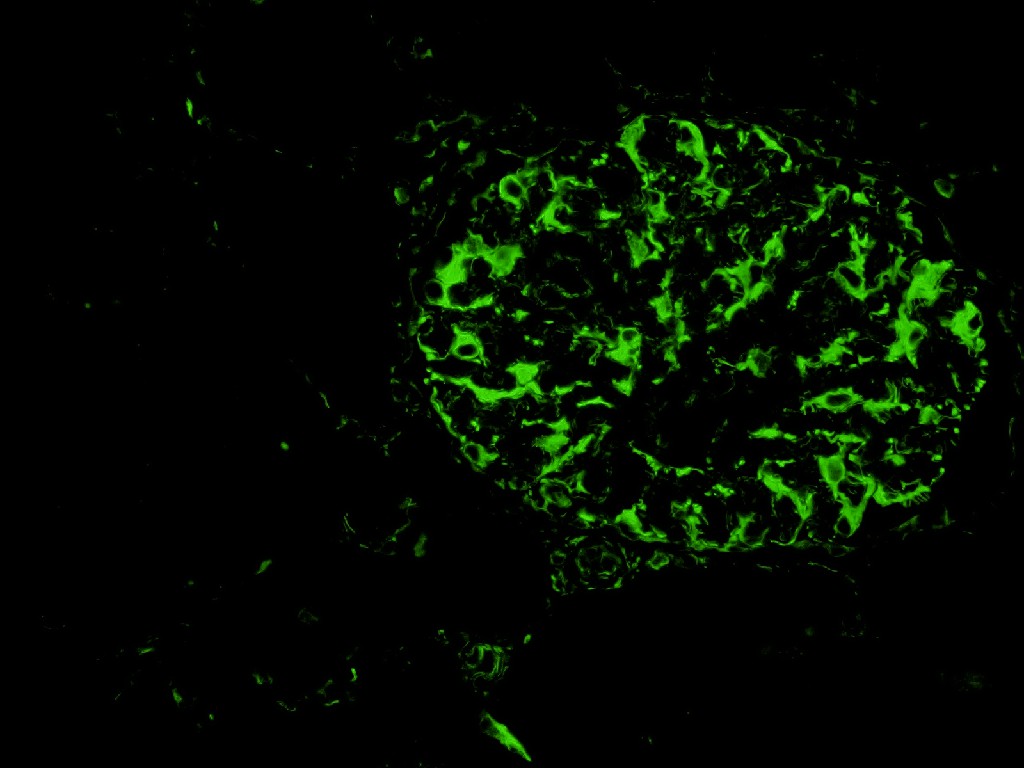

Supplement: Supplementary file 9 — Figure EV1 Source Data [file 44321_2025_315_MOESM9_ESM.zip › Figure EV1/EV1D/2-CD31-GLDC/LEE II/3 (1).jpg]

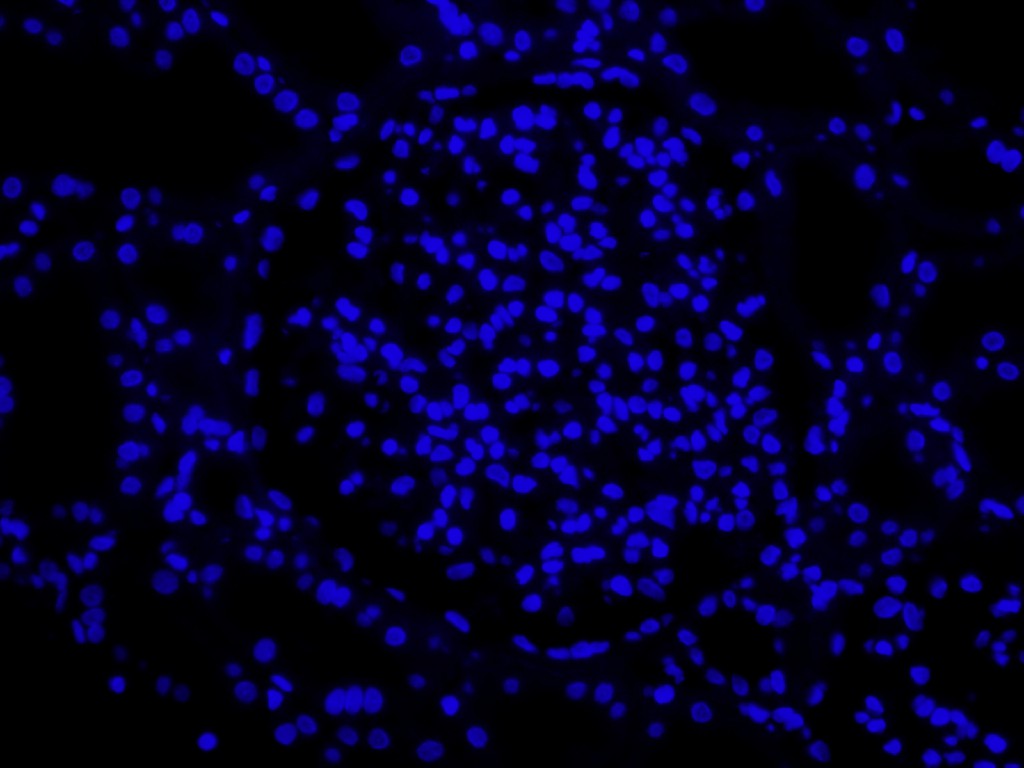

Supplement: Supplementary file 9 — Figure EV1 Source Data [file 44321_2025_315_MOESM9_ESM.zip › Figure EV1/EV1D/2-CD31-GLDC/LEE II/7 (3).jpg]

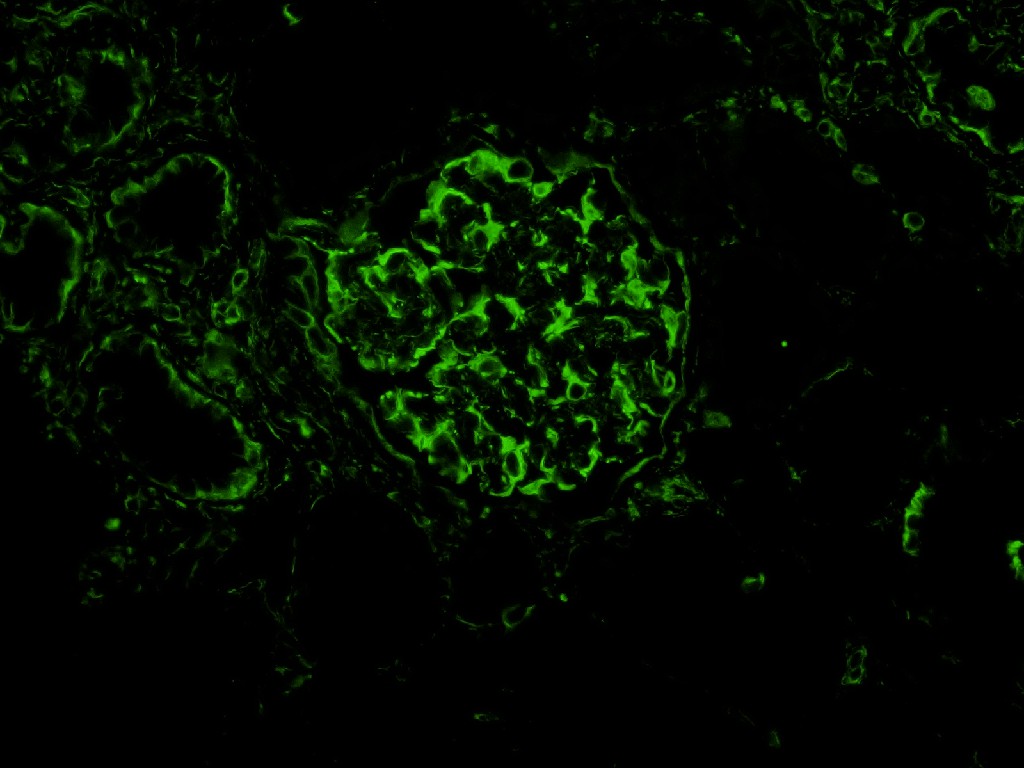

Supplement: Supplementary file 9 — Figure EV1 Source Data [file 44321_2025_315_MOESM9_ESM.zip › Figure EV1/EV1D/2-CD31-GLDC/LEE II/6 (2).jpg]

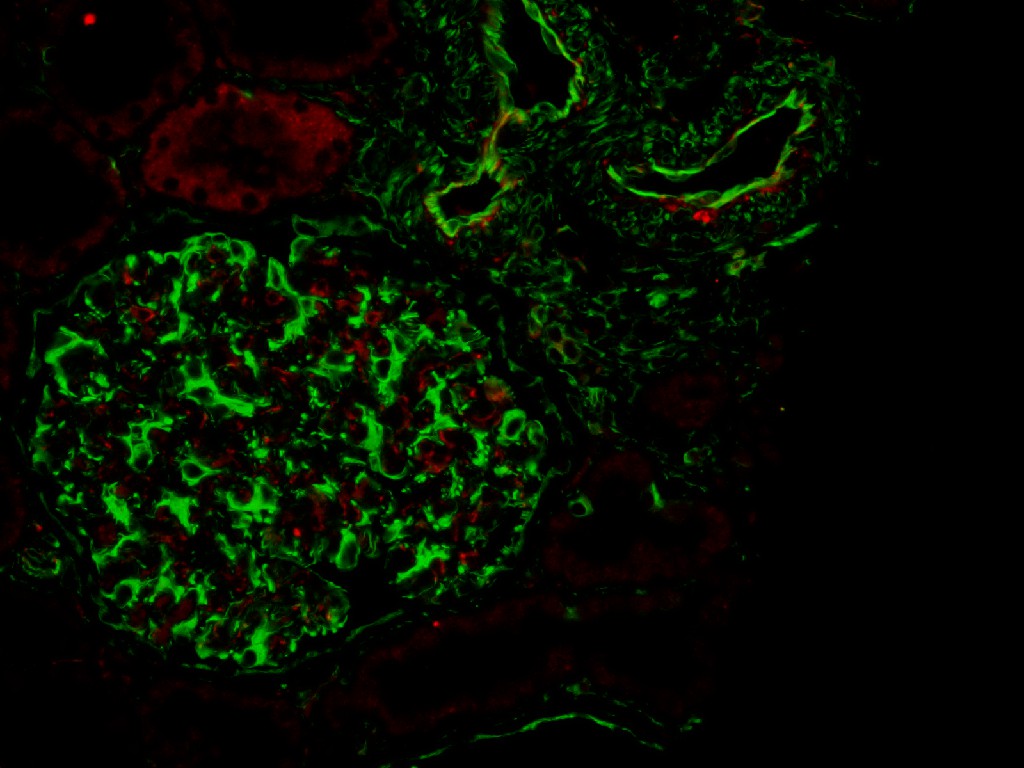

Supplement: Supplementary file 9 — Figure EV1 Source Data [file 44321_2025_315_MOESM9_ESM.zip › Figure EV1/EV1D/2-CD31-GLDC/LEE II/5 (4).jpg]

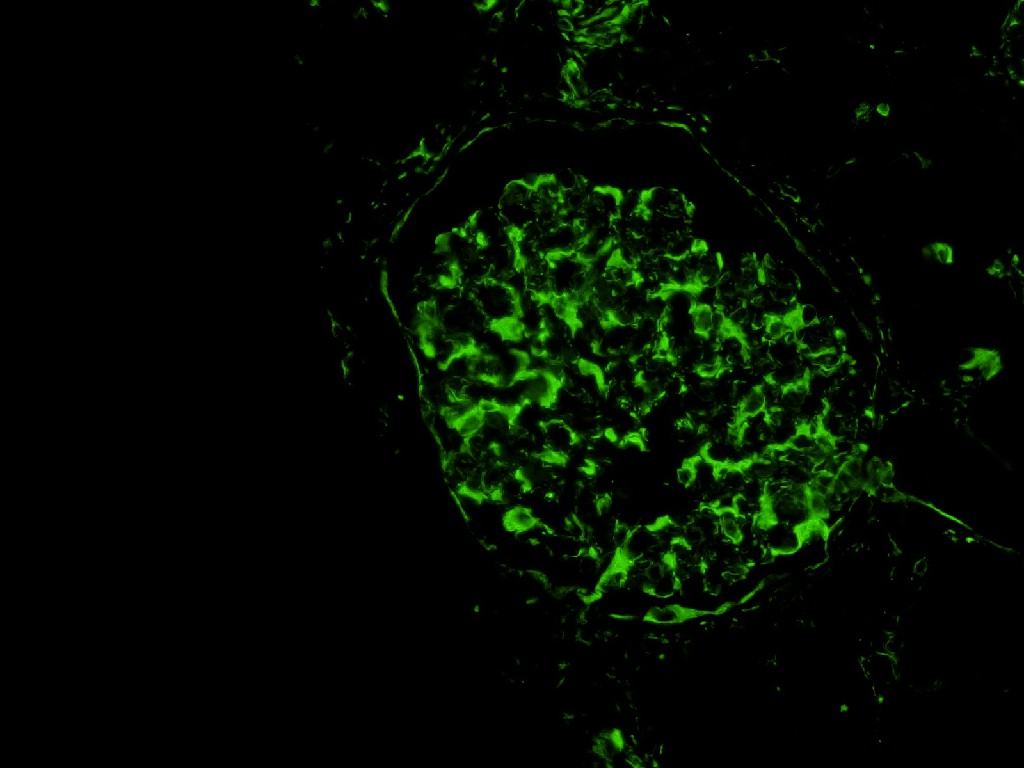

Supplement: Supplementary file 9 — Figure EV1 Source Data [file 44321_2025_315_MOESM9_ESM.zip › Figure EV1/EV1D/2-CD31-GLDC/LEE II/2 (1).jpg]

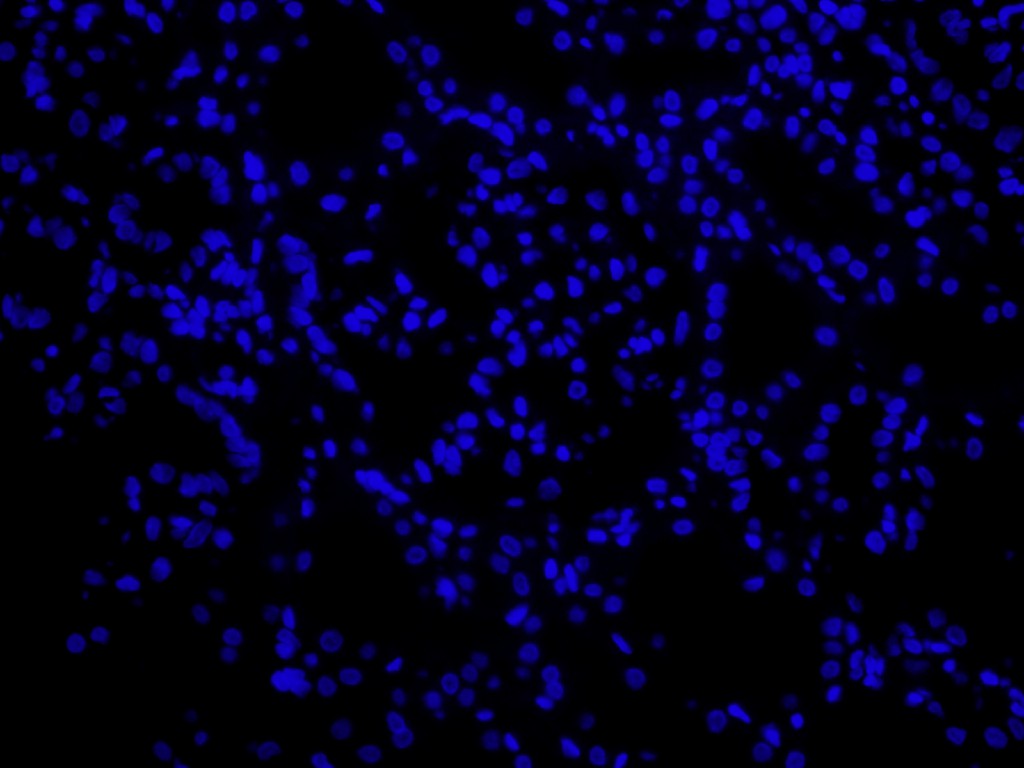

Supplement: Supplementary file 9 — Figure EV1 Source Data [file 44321_2025_315_MOESM9_ESM.zip › Figure EV1/EV1D/2-CD31-GLDC/LEE II/6 (3).jpg]

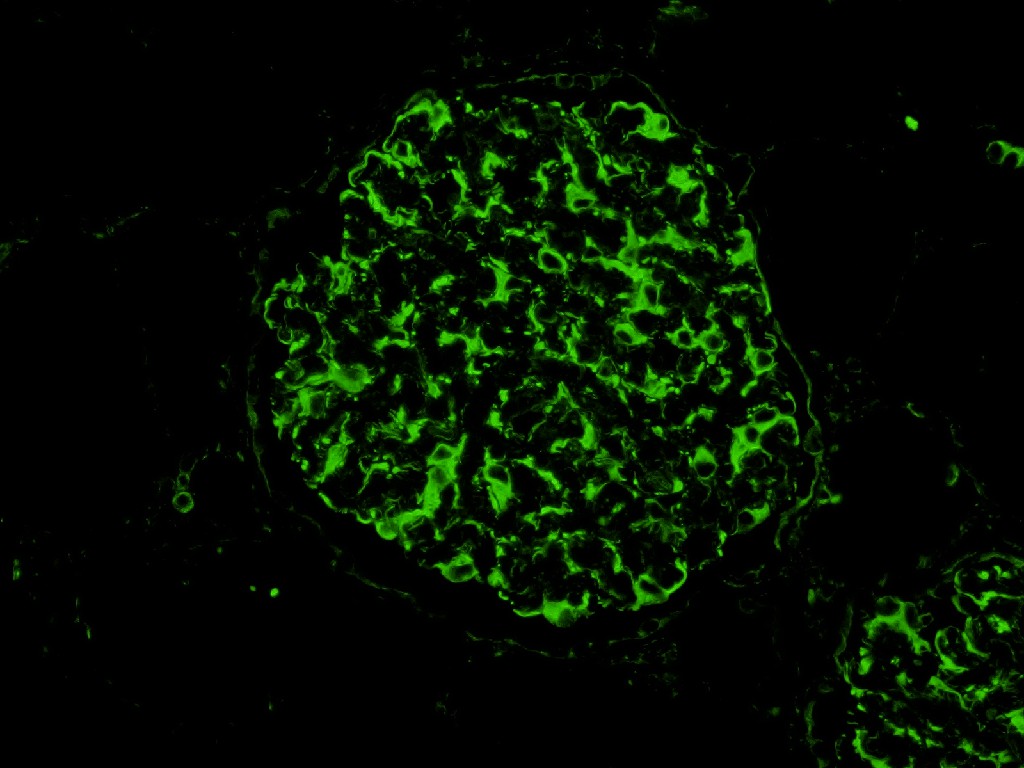

Supplement: Supplementary file 9 — Figure EV1 Source Data [file 44321_2025_315_MOESM9_ESM.zip › Figure EV1/EV1D/2-CD31-GLDC/LEE II/7 (2).jpg]

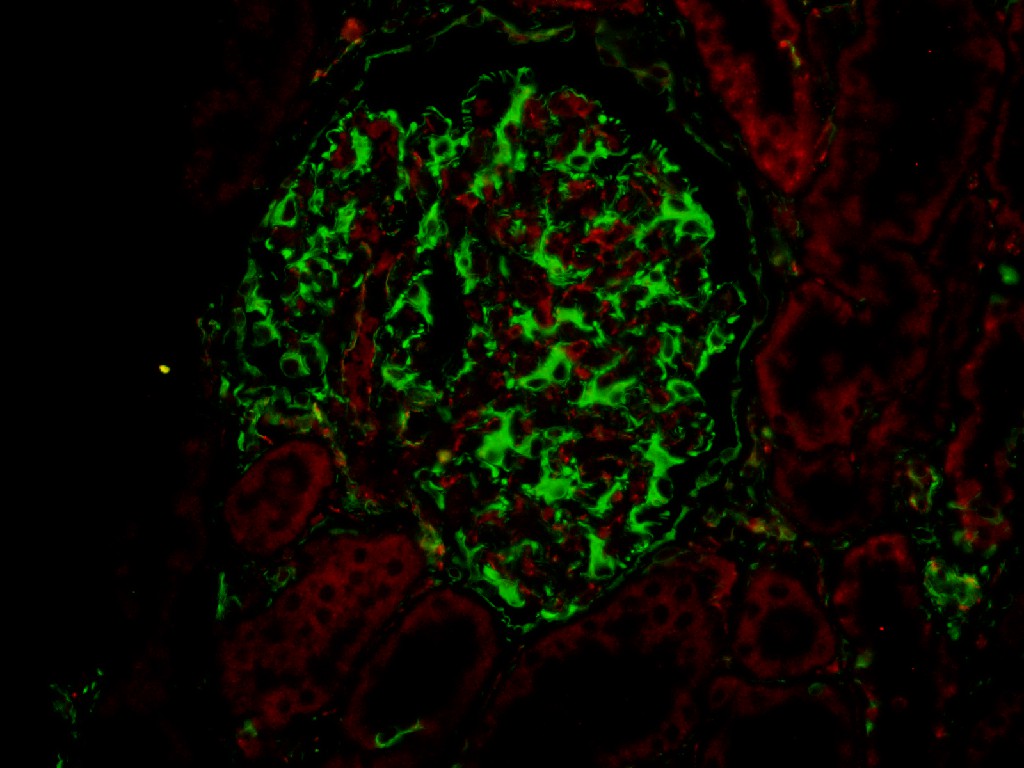

Supplement: Supplementary file 9 — Figure EV1 Source Data [file 44321_2025_315_MOESM9_ESM.zip › Figure EV1/EV1D/2-CD31-GLDC/LEE II/4 (4).jpg]

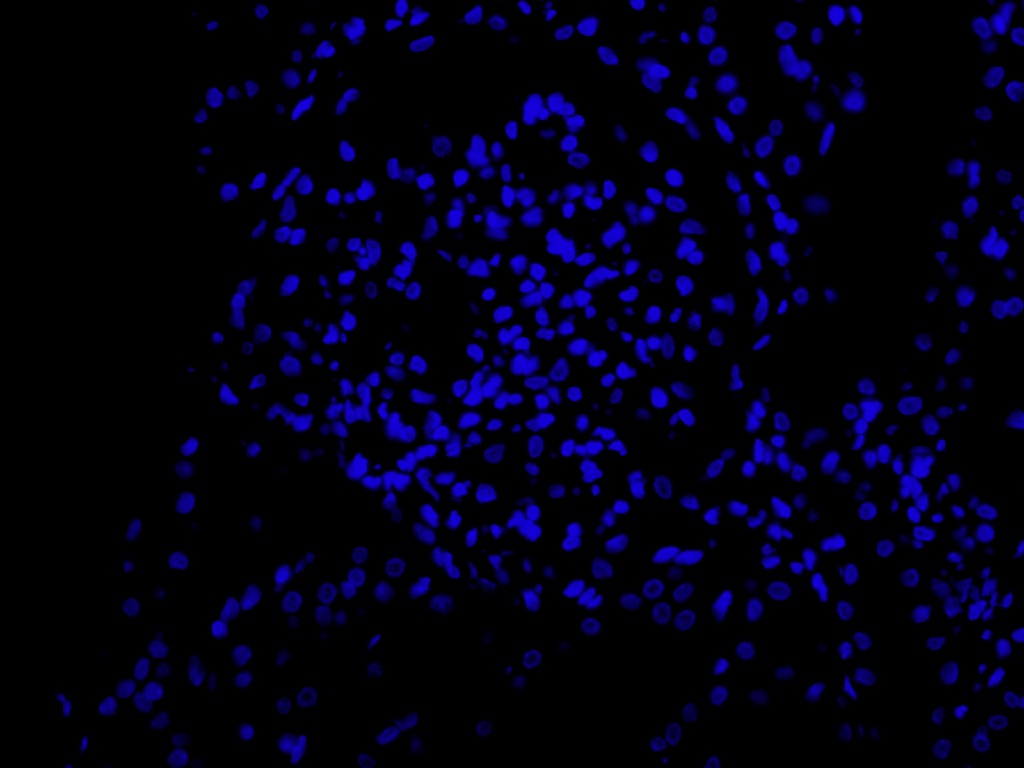

Supplement: Supplementary file 9 — Figure EV1 Source Data [file 44321_2025_315_MOESM9_ESM.zip › Figure EV1/EV1D/2-CD31-GLDC/LEE II/4 (3).jpg]

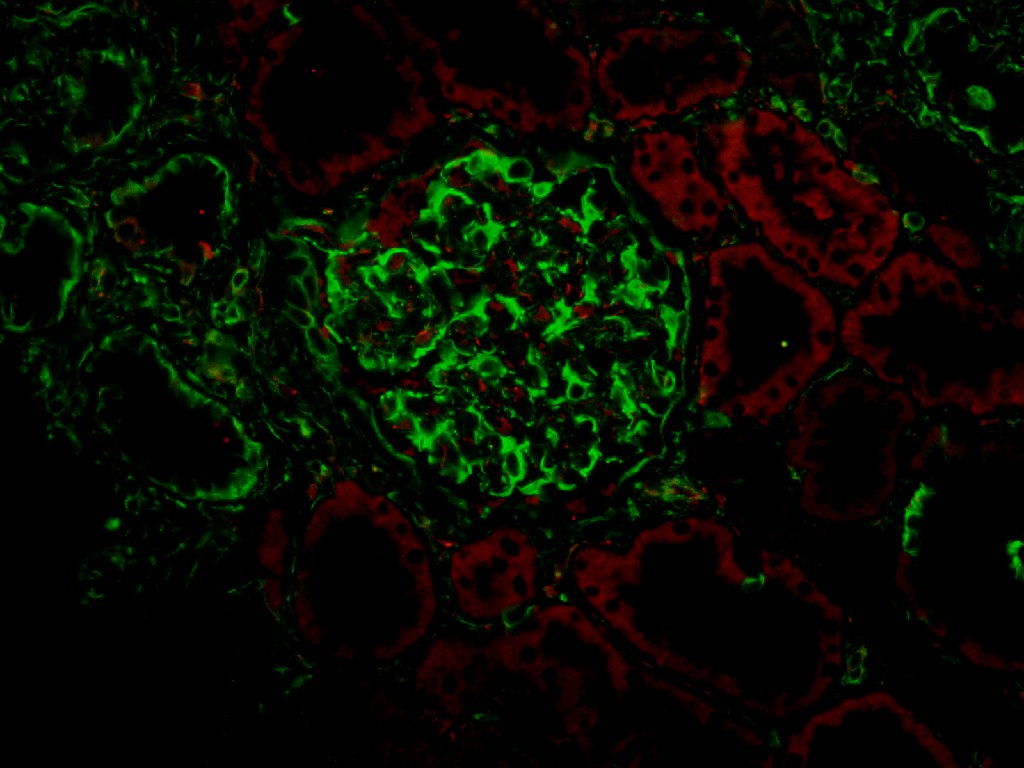

Supplement: Supplementary file 9 — Figure EV1 Source Data [file 44321_2025_315_MOESM9_ESM.zip › Figure EV1/EV1D/2-CD31-GLDC/LEE II/6 (4).jpg]

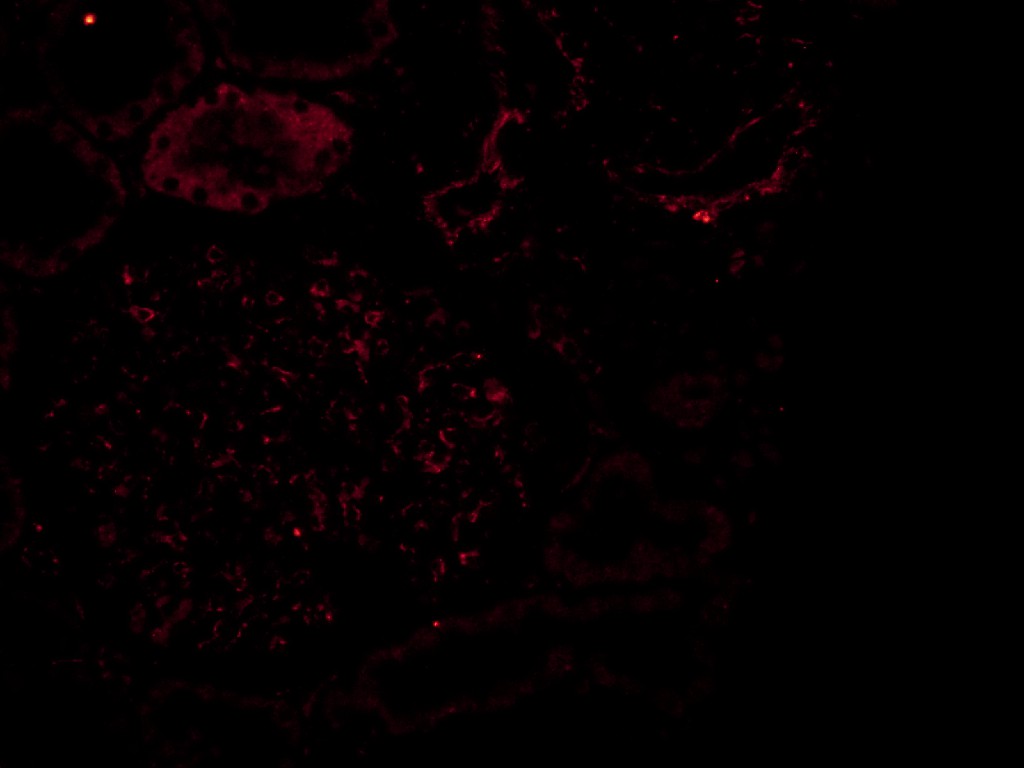

Supplement: Supplementary file 9 — Figure EV1 Source Data [file 44321_2025_315_MOESM9_ESM.zip › Figure EV1/EV1D/2-CD31-GLDC/LEE II/5 (2).jpg]

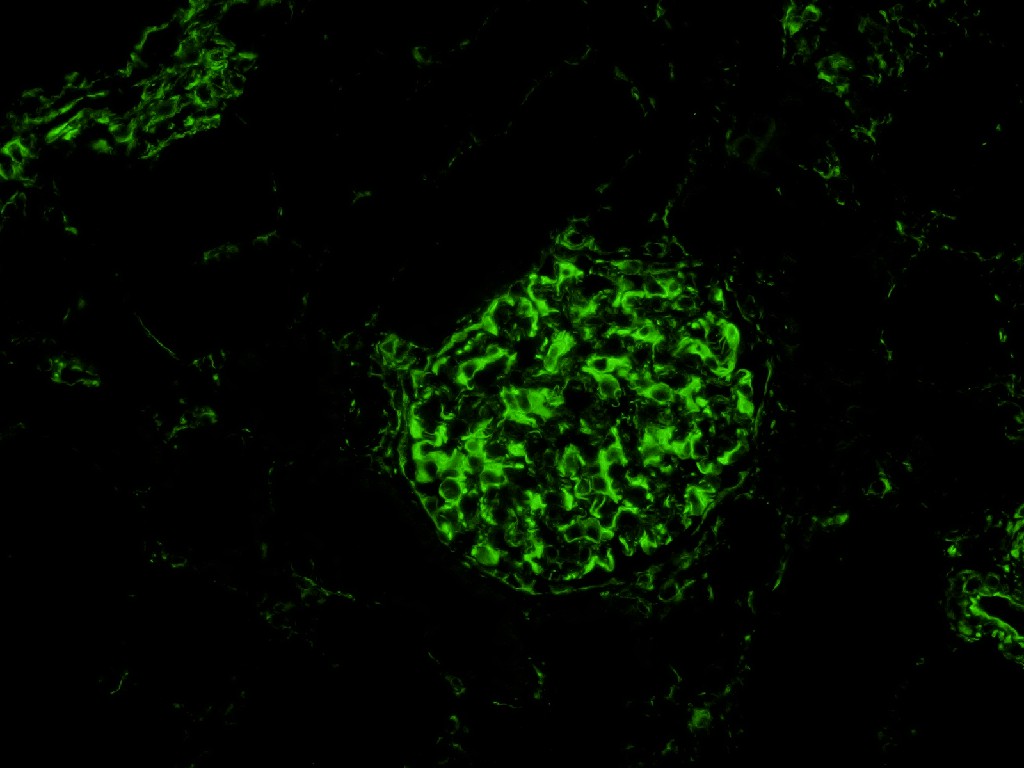

Supplement: Supplementary file 9 — Figure EV1 Source Data [file 44321_2025_315_MOESM9_ESM.zip › Figure EV1/EV1D/2-CD31-GLDC/LEE II/9 (1).jpg]

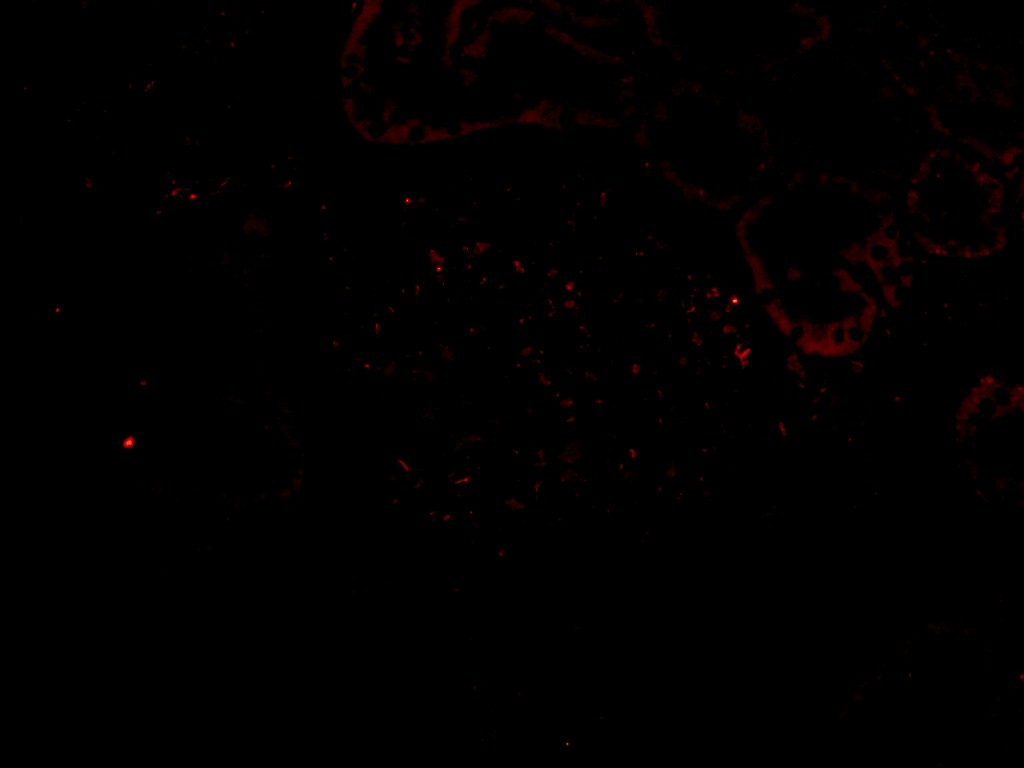

Supplement: Supplementary file 9 — Figure EV1 Source Data [file 44321_2025_315_MOESM9_ESM.zip › Figure EV1/EV1D/2-CD31-GLDC/LEE II/8 (1).jpg]

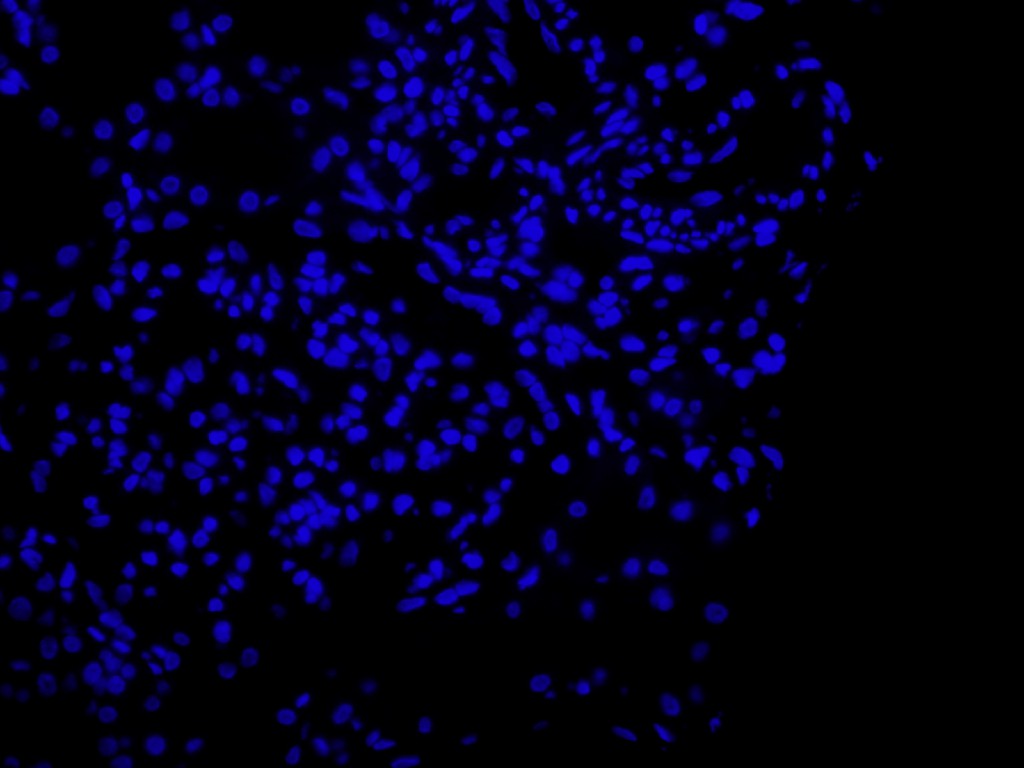

Supplement: Supplementary file 9 — Figure EV1 Source Data [file 44321_2025_315_MOESM9_ESM.zip › Figure EV1/EV1D/2-CD31-GLDC/LEE II/5 (3).jpg]

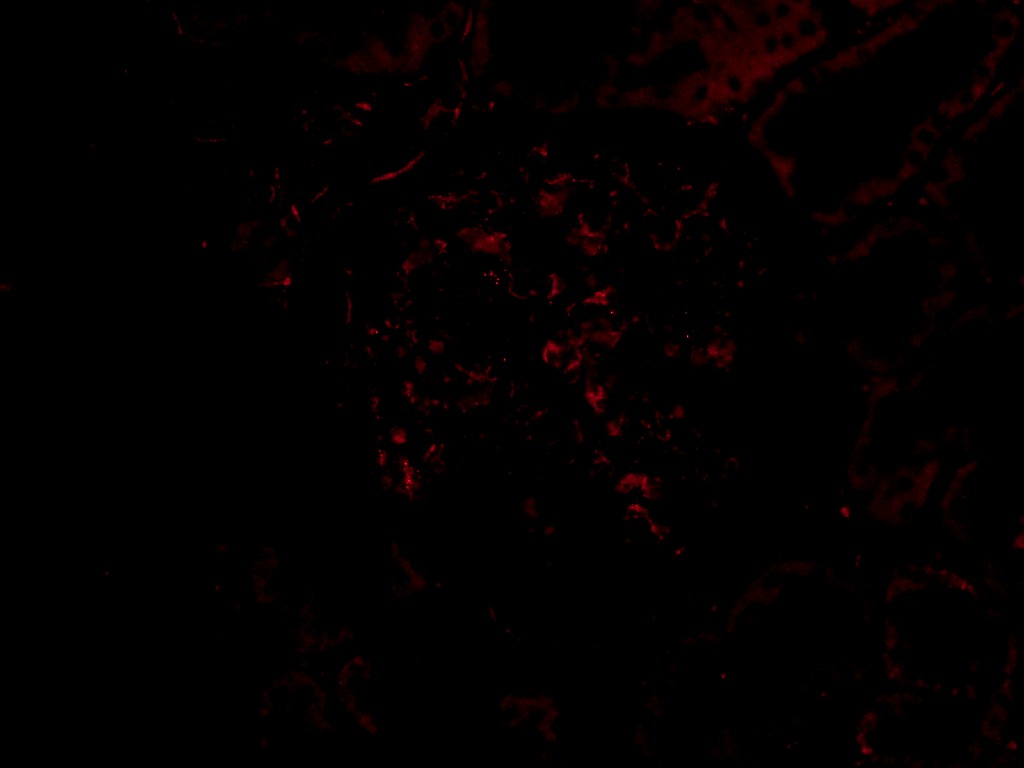

Supplement: Supplementary file 9 — Figure EV1 Source Data [file 44321_2025_315_MOESM9_ESM.zip › Figure EV1/EV1D/2-CD31-GLDC/LEE II/1 (1).jpg]

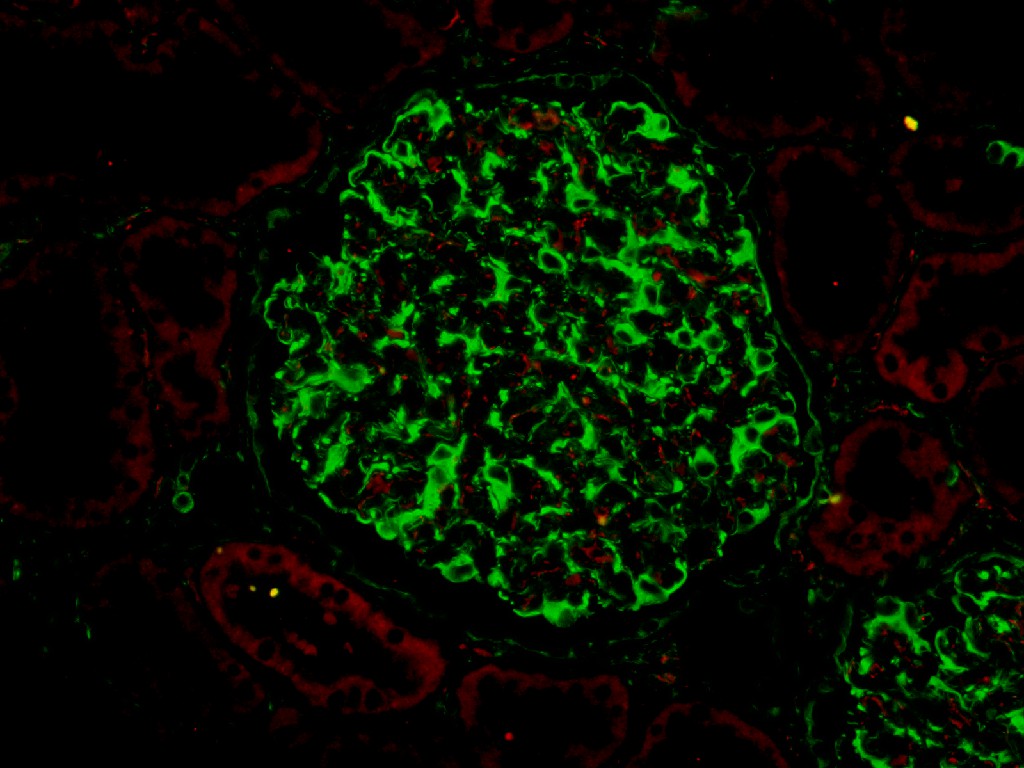

Supplement: Supplementary file 9 — Figure EV1 Source Data [file 44321_2025_315_MOESM9_ESM.zip › Figure EV1/EV1D/2-CD31-GLDC/LEE II/7 (4).jpg]

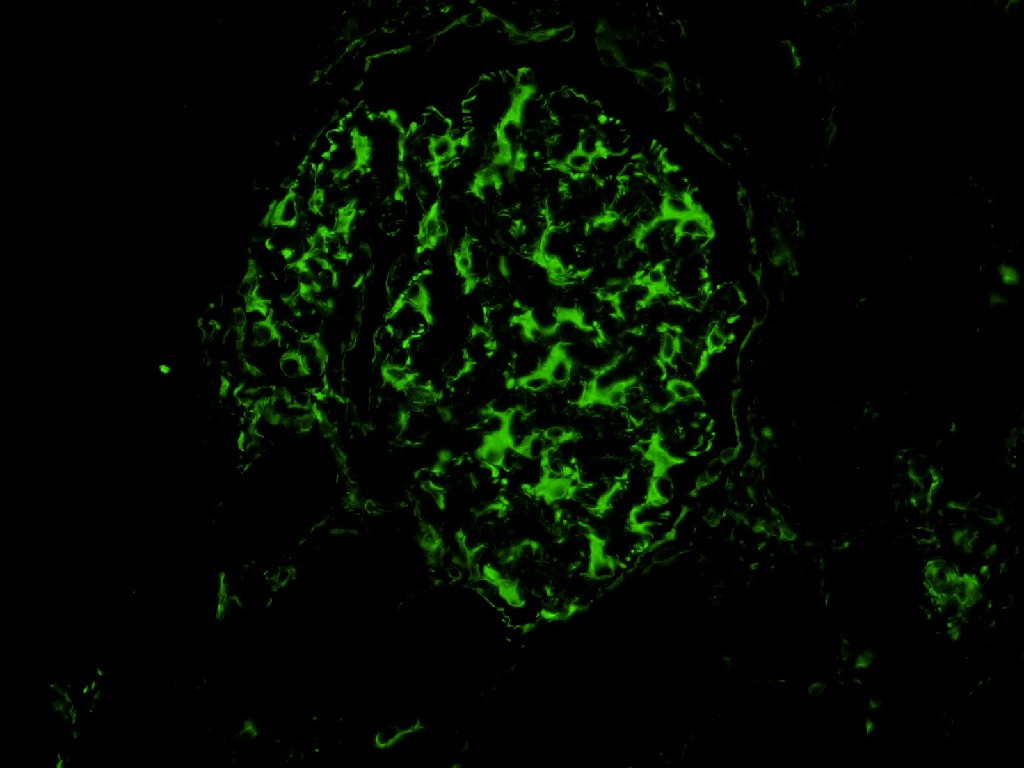

Supplement: Supplementary file 9 — Figure EV1 Source Data [file 44321_2025_315_MOESM9_ESM.zip › Figure EV1/EV1D/2-CD31-GLDC/LEE II/4 (2).jpg]

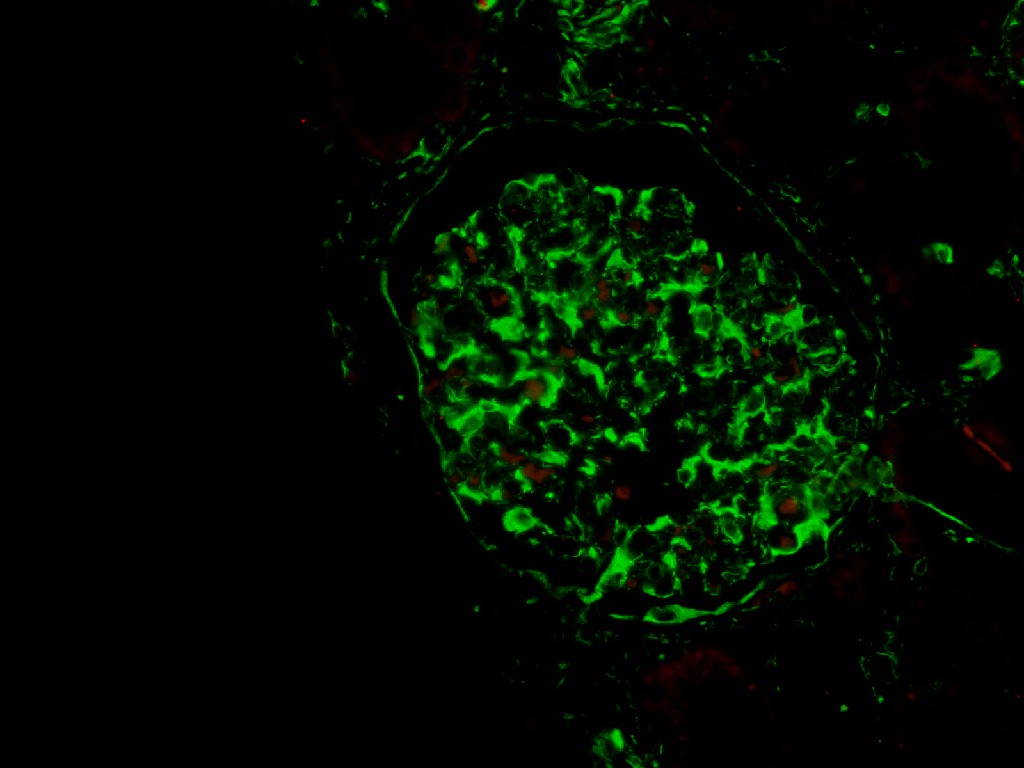

Supplement: Supplementary file 9 — Figure EV1 Source Data [file 44321_2025_315_MOESM9_ESM.zip › Figure EV1/EV1D/2-CD31-GLDC/LEE II/2 (4).jpg]

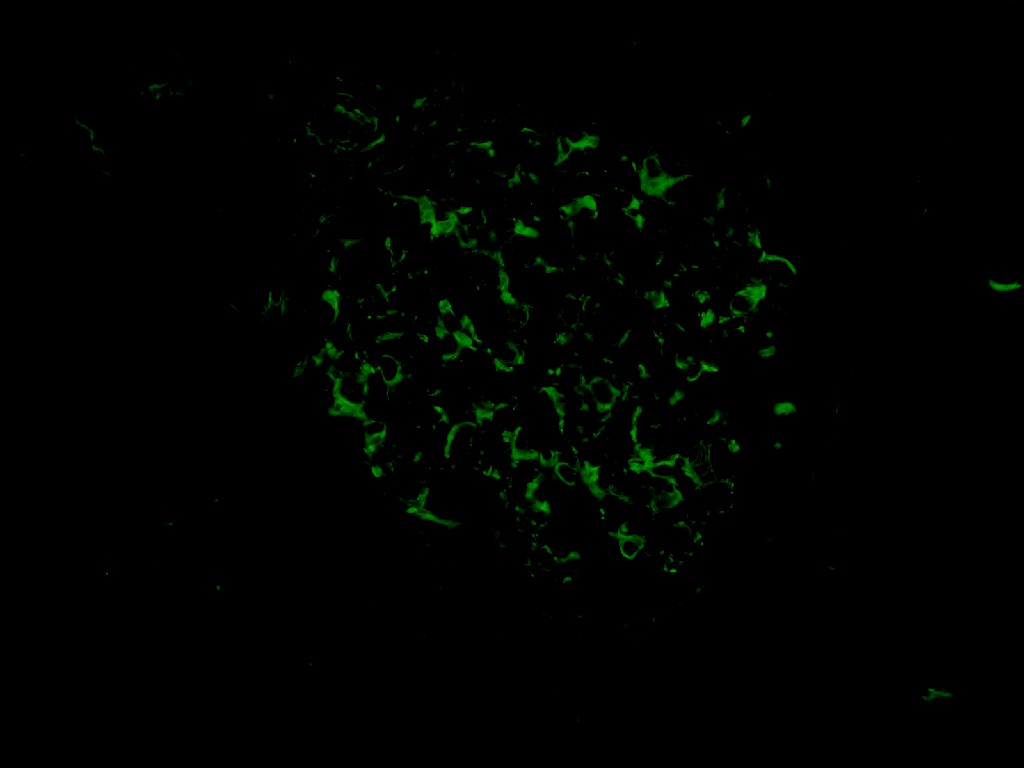

Supplement: Supplementary file 9 — Figure EV1 Source Data [file 44321_2025_315_MOESM9_ESM.zip › Figure EV1/EV1D/2-CD31-GLDC/LEE II/1 (2).jpg]

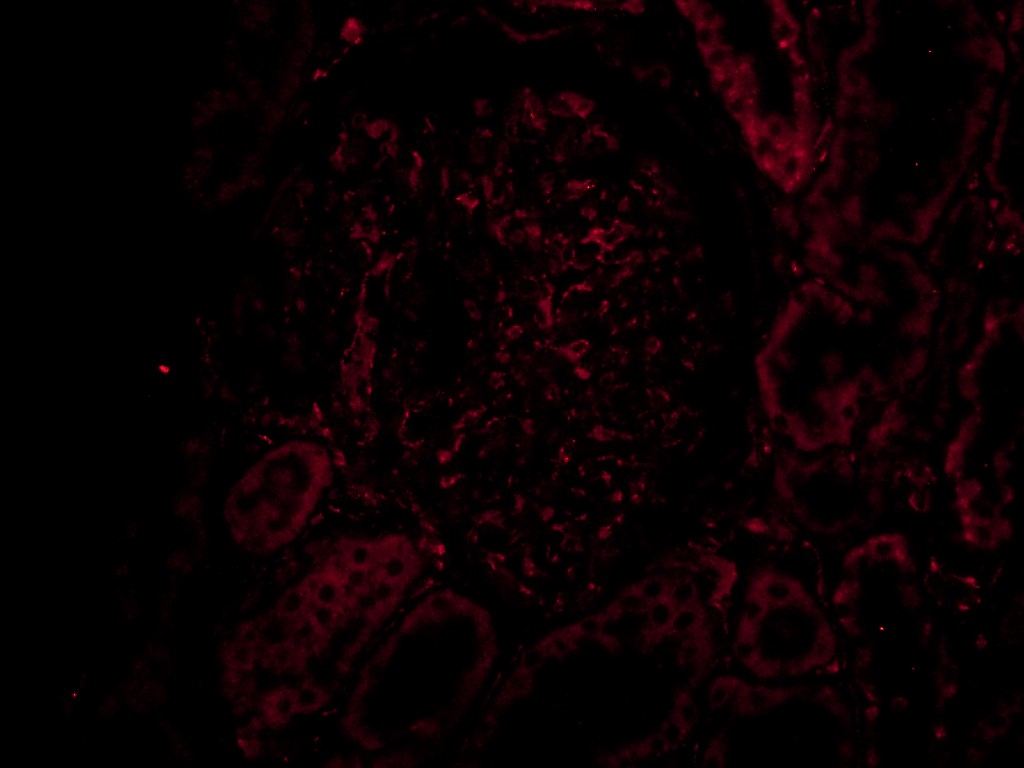

Supplement: Supplementary file 9 — Figure EV1 Source Data [file 44321_2025_315_MOESM9_ESM.zip › Figure EV1/EV1D/2-CD31-GLDC/LEE II/4 (1).jpg]

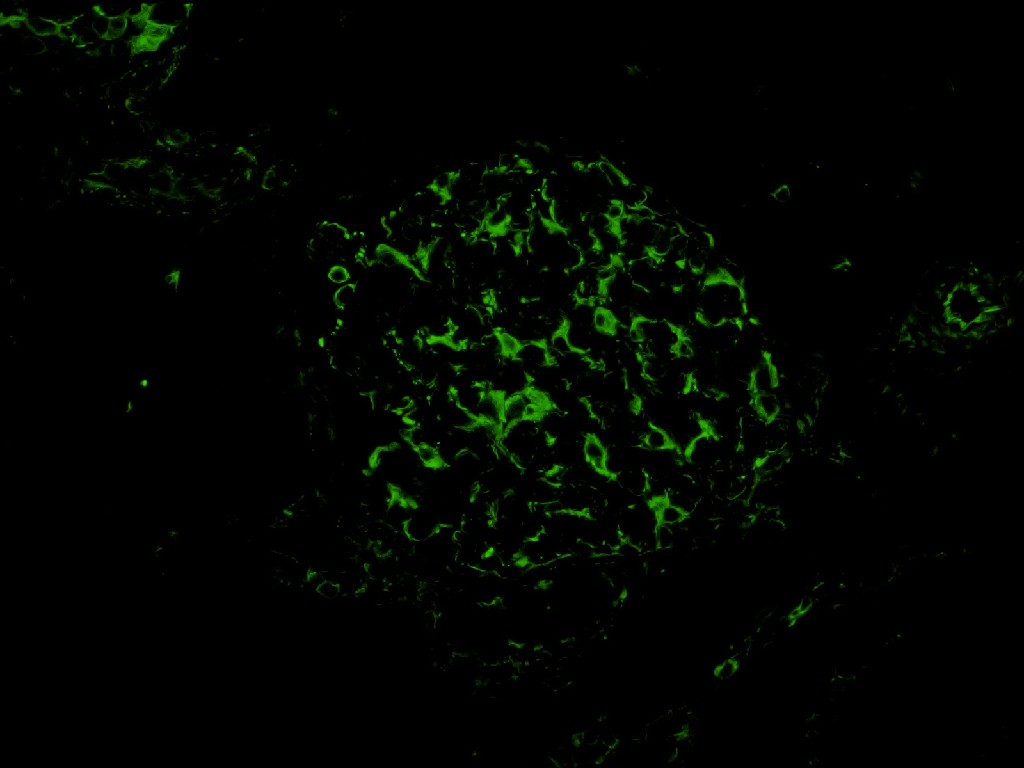

Supplement: Supplementary file 9 — Figure EV1 Source Data [file 44321_2025_315_MOESM9_ESM.zip › Figure EV1/EV1D/2-CD31-GLDC/LEE II/8 (2).jpg]

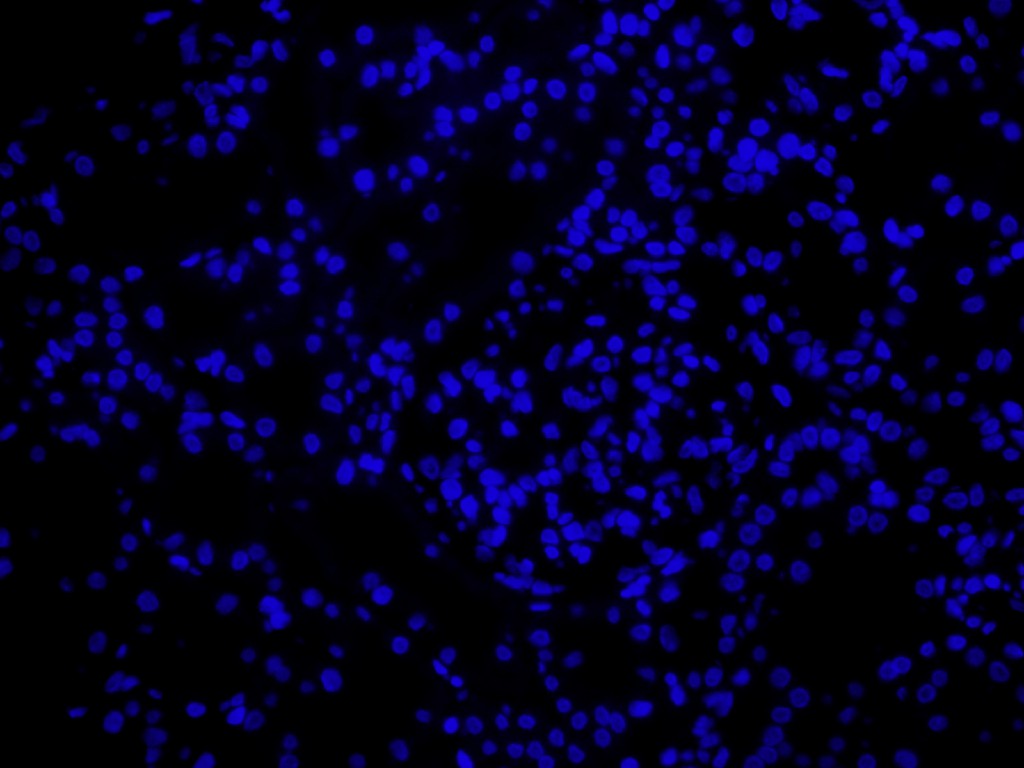

Supplement: Supplementary file 9 — Figure EV1 Source Data [file 44321_2025_315_MOESM9_ESM.zip › Figure EV1/EV1D/2-CD31-GLDC/LEE II/9 (3).jpg]

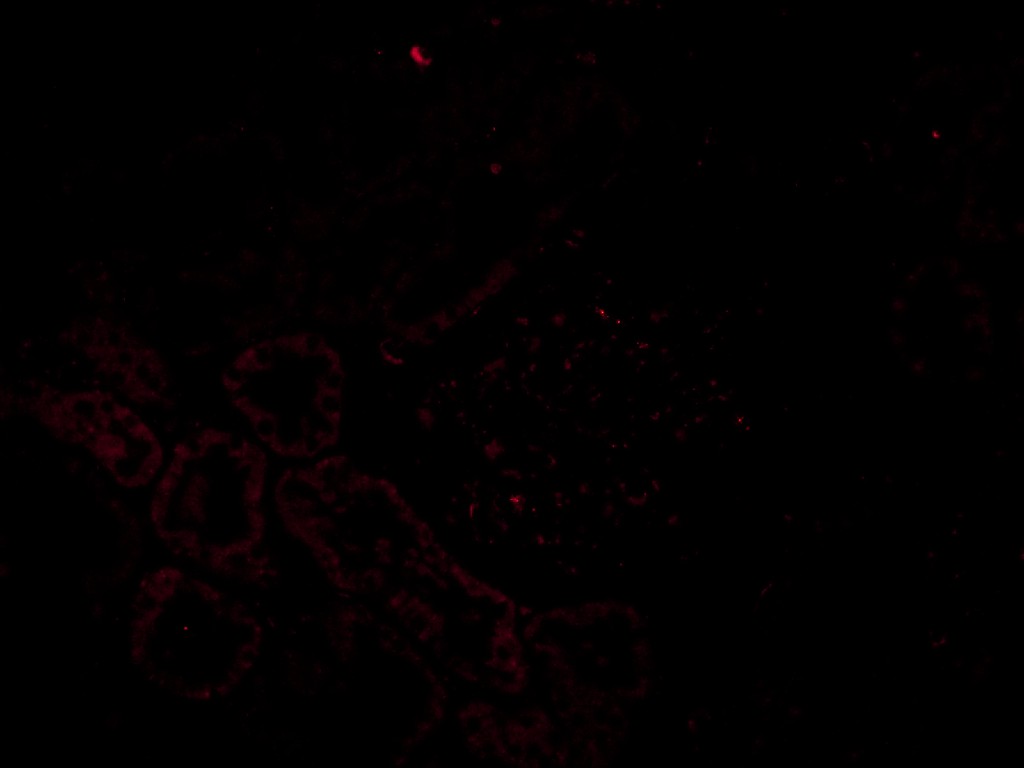

Supplement: Supplementary file 9 — Figure EV1 Source Data [file 44321_2025_315_MOESM9_ESM.zip › Figure EV1/EV1D/2-CD31-GLDC/LEE II/9 (2).jpg]

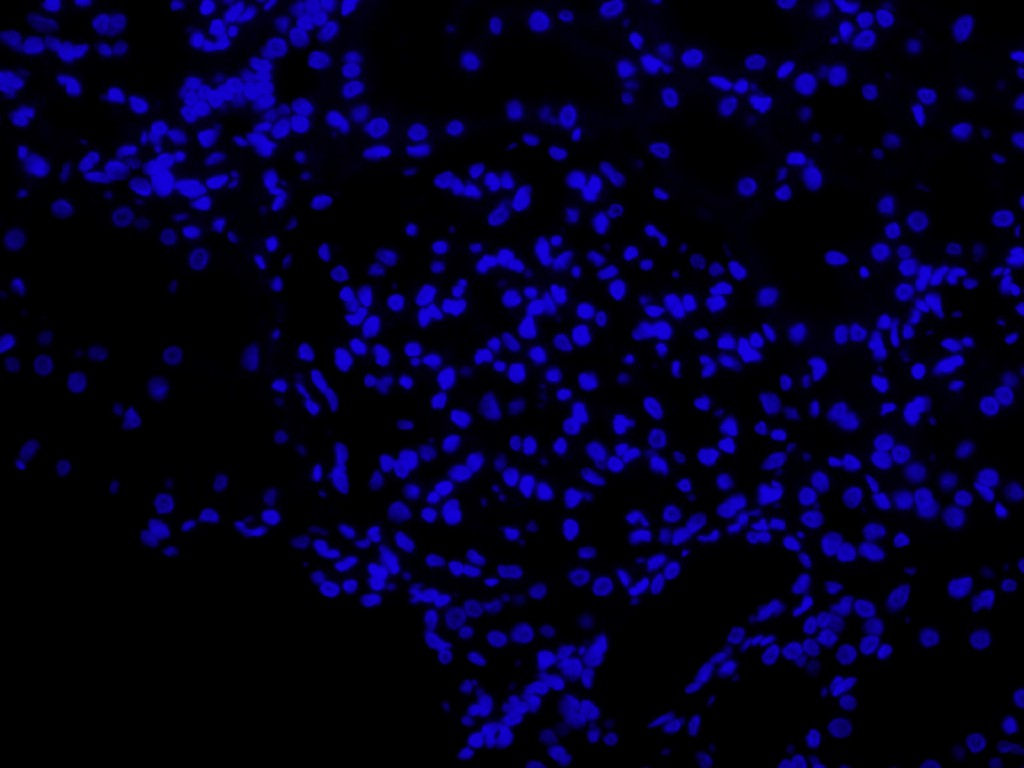

Supplement: Supplementary file 9 — Figure EV1 Source Data [file 44321_2025_315_MOESM9_ESM.zip › Figure EV1/EV1D/2-CD31-GLDC/LEE II/8 (3).jpg]

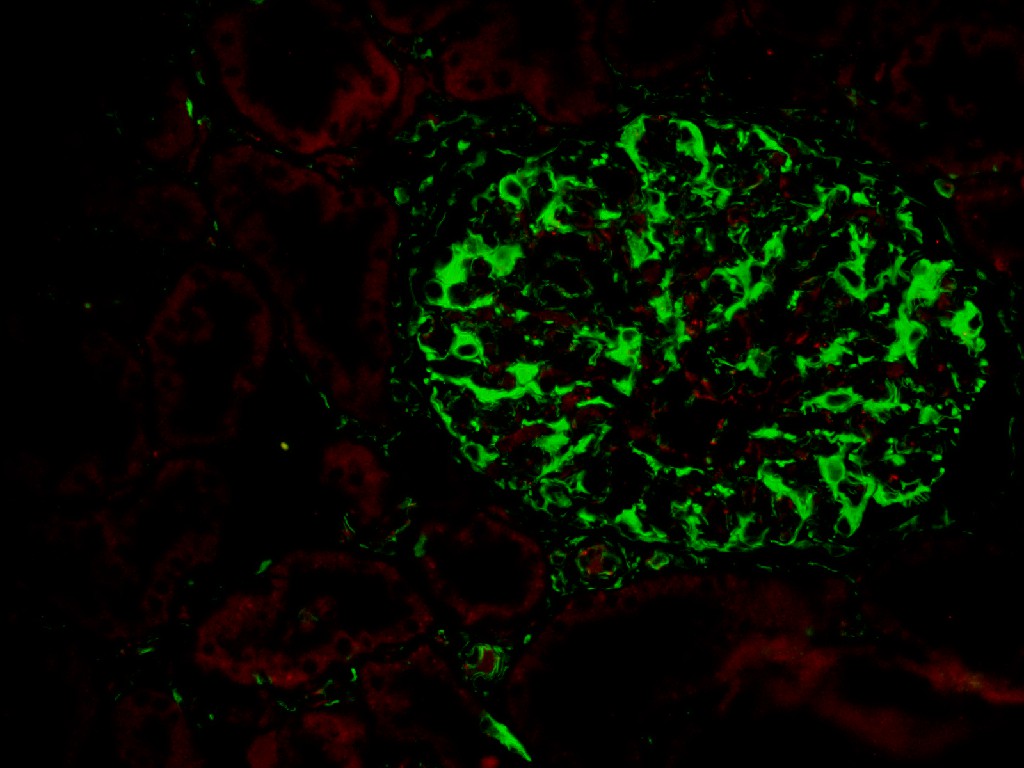

Supplement: Supplementary file 9 — Figure EV1 Source Data [file 44321_2025_315_MOESM9_ESM.zip › Figure EV1/EV1D/2-CD31-GLDC/LEE II/3 (4).jpg]

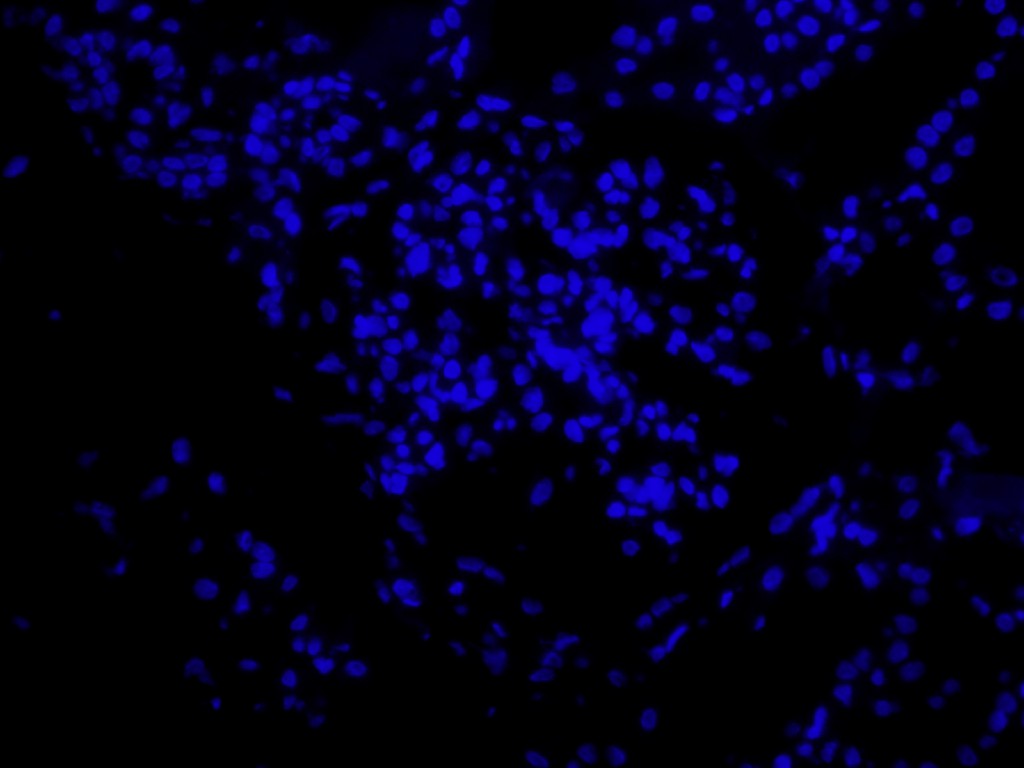

Supplement: Supplementary file 9 — Figure EV1 Source Data [file 44321_2025_315_MOESM9_ESM.zip › Figure EV1/EV1D/2-CD31-GLDC/LEE II/1 (3).jpg]

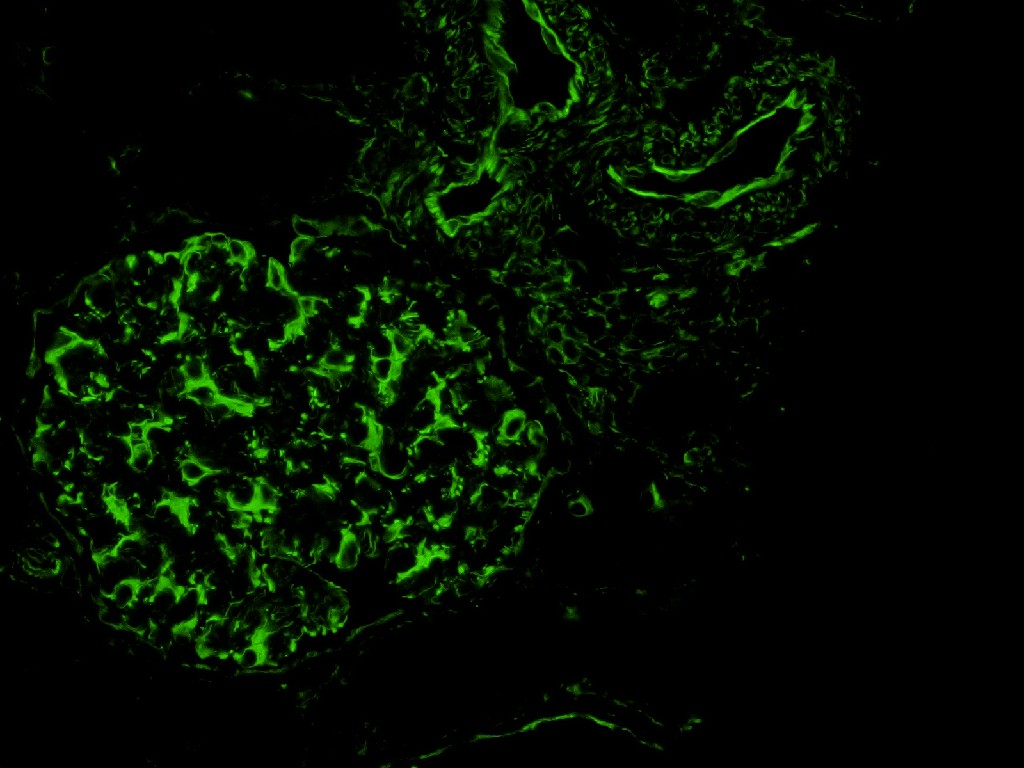

Supplement: Supplementary file 9 — Figure EV1 Source Data [file 44321_2025_315_MOESM9_ESM.zip › Figure EV1/EV1D/2-CD31-GLDC/LEE II/5 (1).jpg]

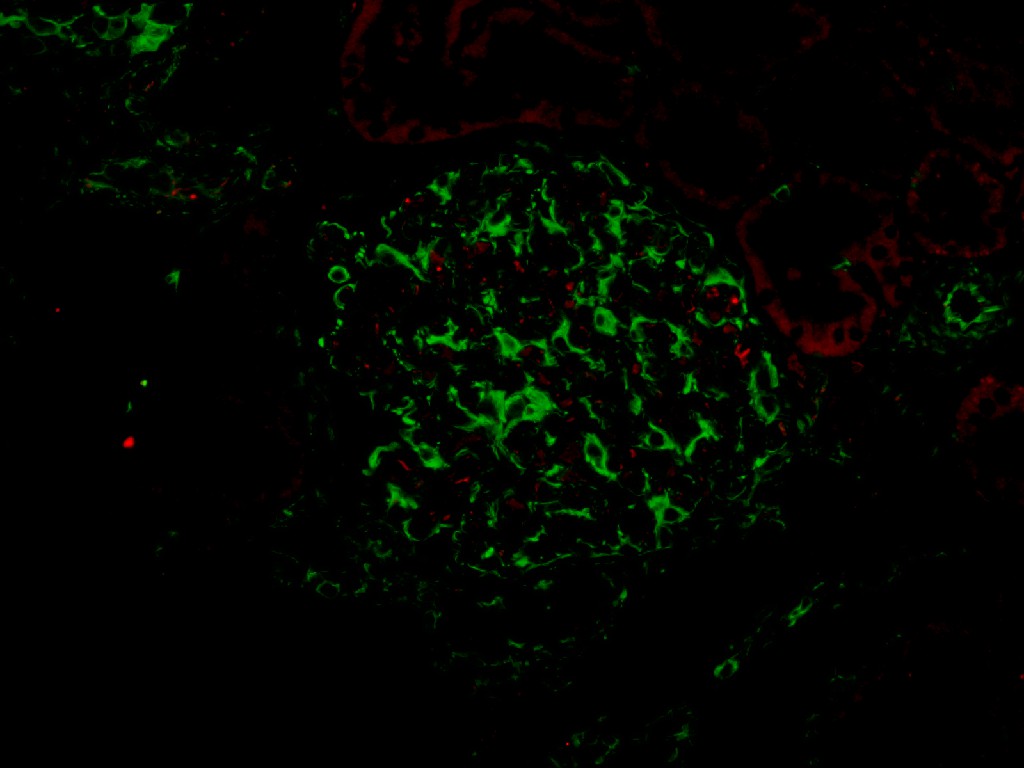

Supplement: Supplementary file 9 — Figure EV1 Source Data [file 44321_2025_315_MOESM9_ESM.zip › Figure EV1/EV1D/2-CD31-GLDC/LEE II/8 (4).jpg]
