# Supplementary material for: Glycine decarboxylase advances IgA nephropathy by boosting mesangial cell proliferation through the pyrimidine pathway
Source: EMBO Mol Med. 2025 Oct 13;17(11):3039–63. doi: 10.1038/s44321-025-00315-2 (PMC12603144; doi:10.1038/s44321-025-00315-2)
Supplement: Supplementary file 14 — Expanded View Figures [file 44321_2025_315_MOESM14_ESM.pdf]

Expanded View Figures

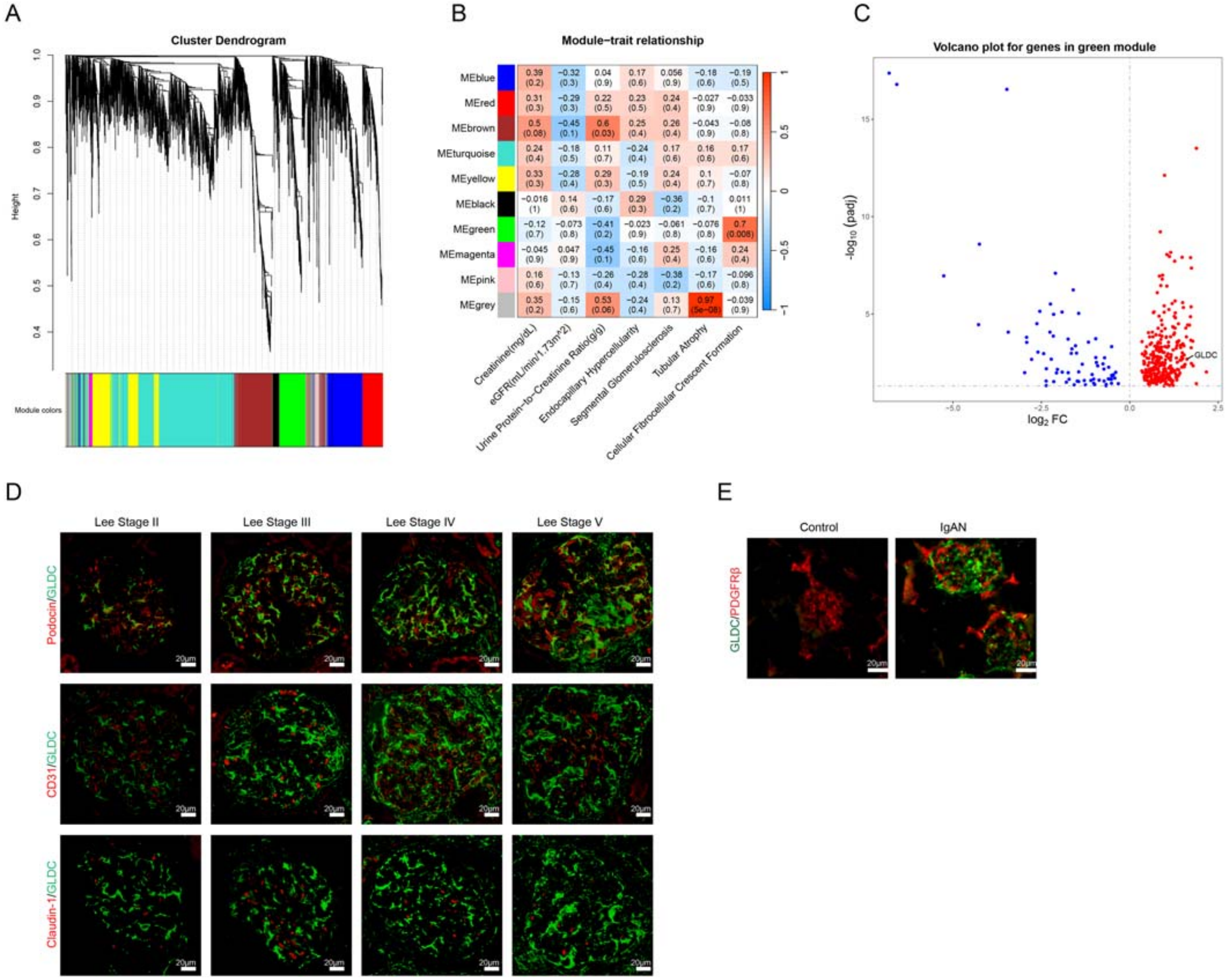

Figure EV1. Analysis of the correlation between GLDC-containing modules and crescent formation in the clinical IgAN dataset.

(**A**) The GSE141295 dataset was downloaded for differential expression analysis (IgAN vs Normal). (**B**) The correlation analysis between gene modules and clinical features. (**C**) KEGG pathway enrichment analysis. (**D**) Co-staining of GLDC with CD31 (an endothelial cell marker), podocin (a podocyte marker), and claudin-1 (a parietal epithelial cell marker and a component of crescents). (**E**) The expression of GLDC and PDGFRβ in normal and IgAN mice. Source data are available online for this figure.

 © The Author(s)

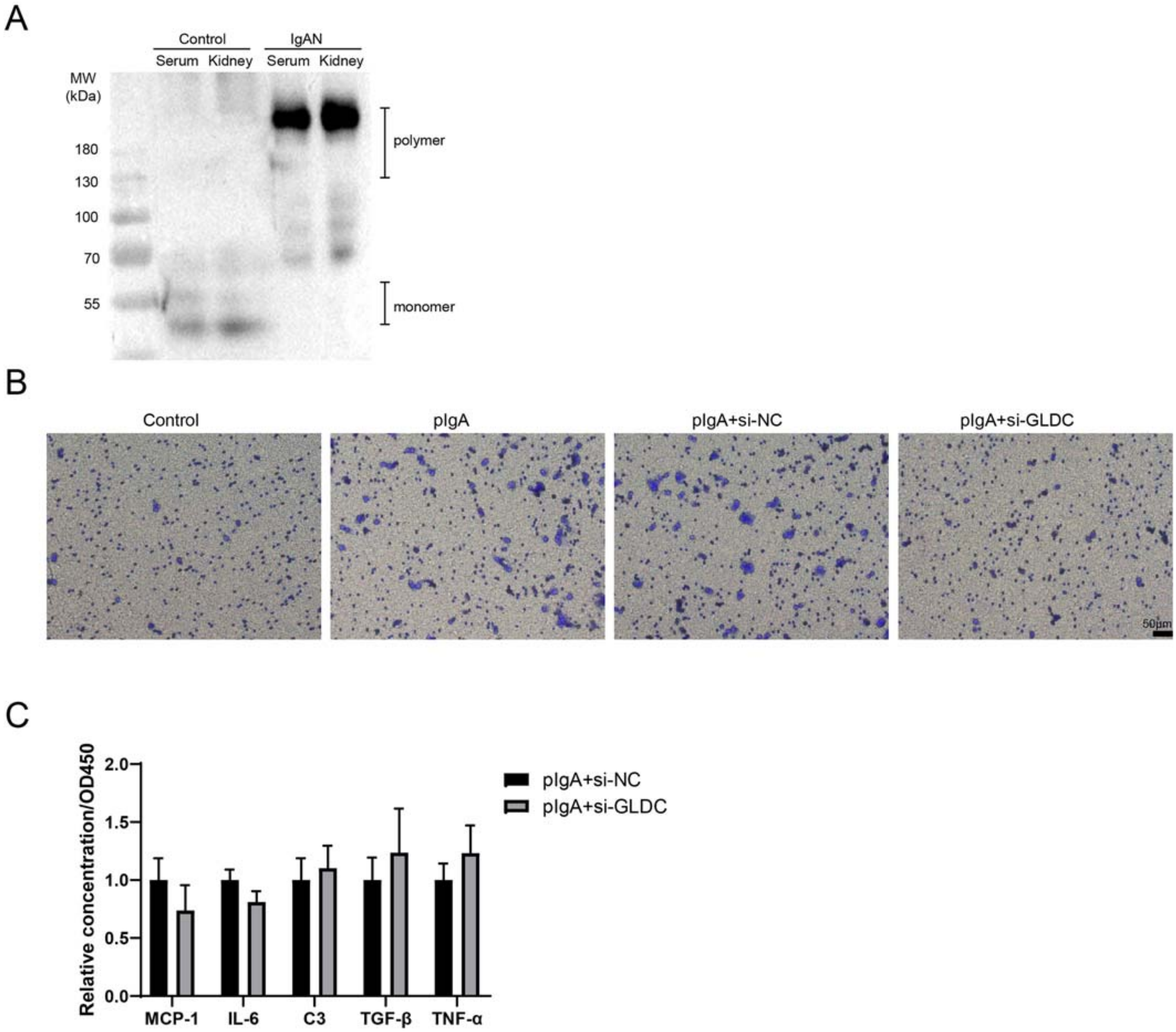

Figure EV2. The effect of GLDC on the chemotactic ability of glomerular mesangial cells.

(**A**) Expression of IgA in serum and kidney tissue of normal and IgAN model mice. (**B**) SV40-MES13 cells in the control, pIgA, pIgA + si-NC, pIgA + si-GLDC groups were co-cultured with Raw264.7 derived macrophage, and the chemotactic effects on macrophages were analyzed by Transwell assay. (**C**) ELISA was used to detect MCP-1, IL-6, C3, TGF-β1, and TNF-α content in the pIgA + si-NC and pIgA + si-GLDC groups. Source data are available online for this figure.

© The Author(s)

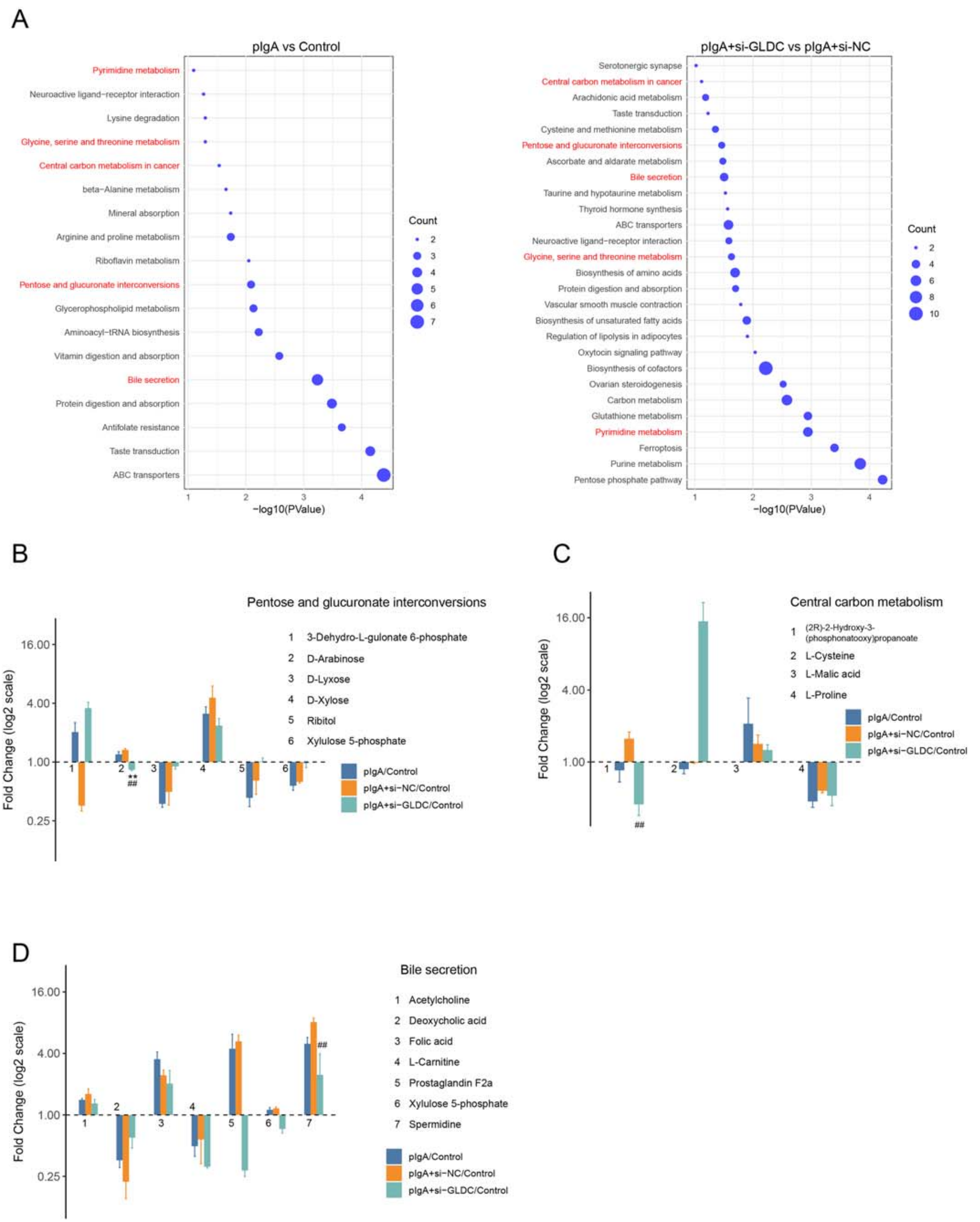
A
pIgA vs Control
Pyrimidine metabolism
Neuroactive ligand−receptor interaction
Lysine degradation
Glycine, serine and threonine metabolism
Central carbon metabolism in cancer
beta−Alanine metabolism
Mineral absorption
Arginine and proline metabolism
Riboflavin metabolism
Pentose and glucuronate interconversions
Glycerophospholipid metabolism
Aminoacyl−tRNA biosynthesis
Vitamin digestion and absorption
Bile secretion
Protein digestion and absorption
Antifolate resistance
Taste transduction
ABC transporters
−log10(PValue)
Count
pIgA+si-GLDC vs pIgA+si-NC
Serotonergic synapse
Central carbon metabolism in cancer
Arachidonic acid metabolism
Taste transduction
Cysteine and methionine metabolism
Pentose and glucuronate interconversions
Ascorbate and aldarate metabolism
Bile secretion
Taurine and hypotaurine metabolism
Thyroid hormone synthesis
ABC transporters
Neuroactive ligand−receptor interaction
Glycine, serine and threonine metabolism
Biosynthesis of amino acids
Protein digestion and absorption
Vascular smooth muscle contraction
Biosynthesis of unsaturated fatty acids
Regulation of lipolysis in adipocytes
Oxytocin signaling pathway
Biosynthesis of cofactors
Ovarian steroidogenesis
Carbon metabolism
Glutathione metabolism
Pyrimidine metabolism
Ferroptosis
Purine metabolism
Pentose phosphate pathway
−log10(PValue)
Count
B
Pentose and glucuronate interconversions
Fold Change (log2 scale)
1 3-Dehydro-L-gulonate 6-phosphate
2 D-Arabinose
3 D-Lyxose
4 D-Xylose
5 Ribitol
6 Xylulose 5-phosphate
pIgA/Control
pIgA+si−NC/Control
pIgA+si−GLDC/Control
C
Central carbon metabolism
Fold Change (log2 scale)
1 (2R)-2-Hydroxy-3-(phosphonatooxy)propanoate
2 L-Cysteine
3 L-Malic acid
4 L-Proline
pIgA/Control
pIgA+si−NC/Control
pIgA+si−GLDC/Control
D
Bile secretion
Fold Change (log2 scale)
1 Acetylcholine
2 Deoxycholic acid
3 Folic acid
4 L-Carnitine
5 Prostaglandin F2a
6 Xylulose 5-phosphate
7 Spermidine
pIgA/Control
pIgA+si−NC/Control
pIgA+si−GLDC/Control

© The Author(s)

Figure EV3. The key metabolic pathways that GLDC regulates glomerular mesangial cell proliferation.

(**A**) LC/MS non-target metabolomics analysis and KEGG pathway enrichment analysis were performed (control vs pIgA, pIgA + si-NC vs pIgA + si-GLDC). (**B–D**) The metabolites of pentose and glucuronate interconversions, central metabolism, bile secretion in the pIgA + si-NC and pIgA + si-GLDC groups were analyzed. $^{*}P < 0.05$. Source data are available online for this figure.

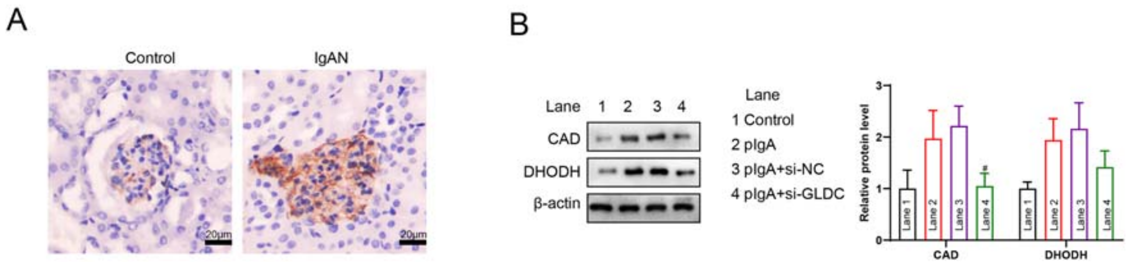

Figure EV4. The expressions of CAD and DHODH proteins.

(**A**) The positive expression of CAD in the glomeruli of control mice ($N = 6$) and IgAN model mice ($N = 6$) were detected by immunohistochemical staining assay. (**B**) Western blot assay was used to detect CAD and DHODH protein expressions in the control, pIgA, pIgA + si-NC, pIgA + si-GLDC group SV40-MES13 cells. $^{\#}P < 0.05$ vs pIgA + si-NC. Source data are available online for this figure.

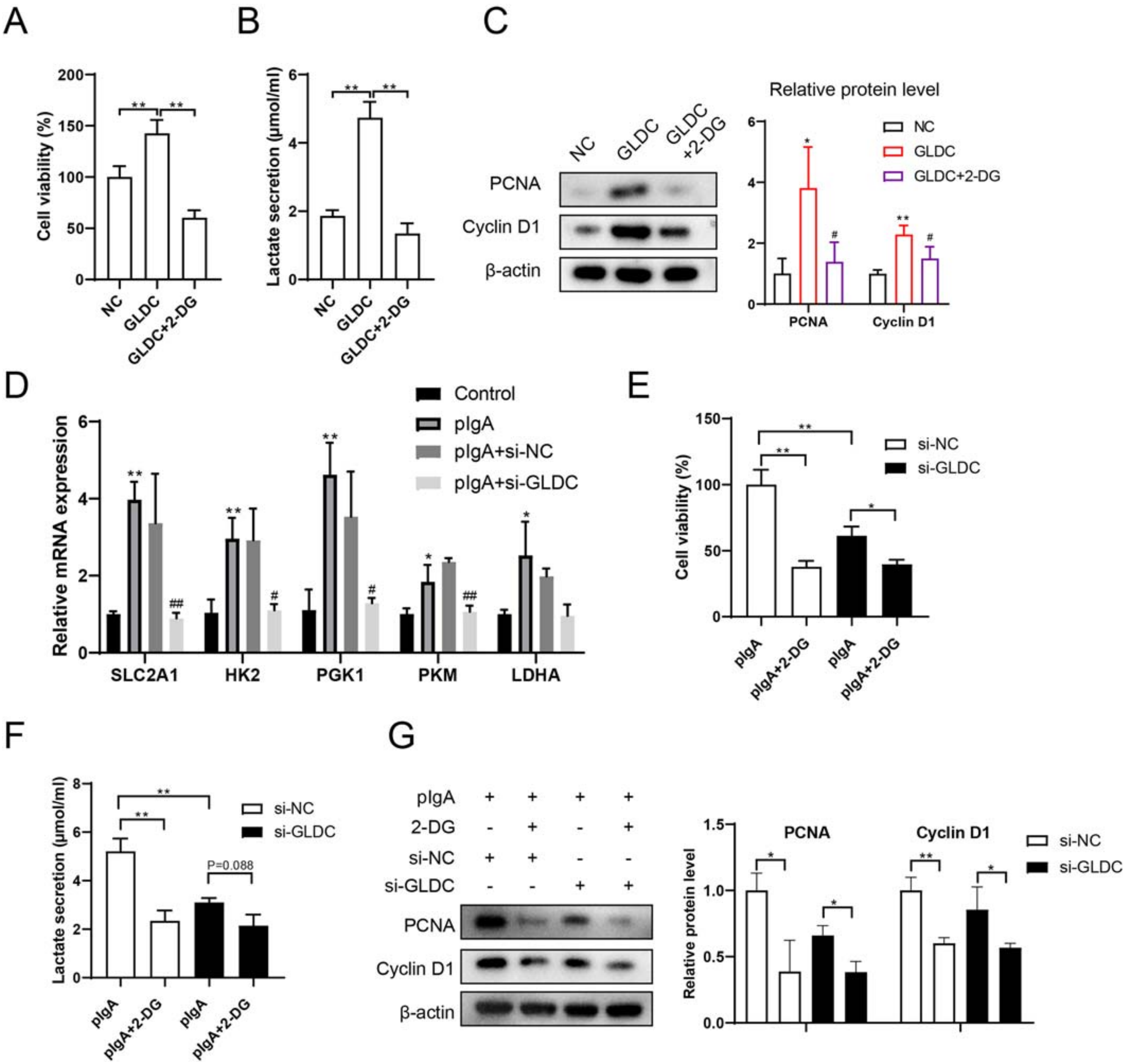

Figure EV5. The role of the glycolytic pathway in the regulation of mesangial cell growth by GLDC.

(**A–C**) SV40-MES13 cells were grouped into NC, GLDC, GLDC + 2-DG. (**A**) Cell viability assay was performed. $^{**}P < 0.01$. (**B**) Lactate secretion was detected. $^{**}P < 0.01$. (**C**) Western blot assay was used to detect Cyclin D1 and PCNA protein expressions. $^{**}P < 0.01$; $^{\#\#}P < 0.01$. (**D**) The mRNA levels of SLC2A1, HK2, PGK1, PKM, and LDHA were detected by qPCR in the control, pIgA, pIgA + si-NC, pIgA + si-GLDC group "-->SV40-MES13 cells. $^{*}P < 0.05$, $^{**}P < 0.01$; $^{\#}P < 0.05$, $^{\#\#}P < 0.01$. (**E–G**) SV40-MES13 cells were grouped into pIgA + si-NC, pIgA + si-GLDC, pIgA + si-NC + 2-DG, pIgA + si-GLDC + 2-DG. (**E**) Cell viability assay was performed. $^{*}P < 0.05$, $^{**}P < 0.01$. (**F**) Lactate secretion was detected. $^{**}P < 0.01$. (**G**) Western blot assay was used to detect Cyclin D1 and PCNA protein expressions. $^{**}P < 0.01$. Source data are available online for this figure.

© The Author(s)
